# Biogeographic venom variation in Russell’s viper (*Daboia russelii*) and the preclinical inefficacy of antivenom therapy in snakebite hotspots

**DOI:** 10.1371/journal.pntd.0009247

**Published:** 2021-03-25

**Authors:** R. R. Senji Laxme, Suyog Khochare, Saurabh Attarde, Vivek Suranse, Ashwin Iyer, Nicholas R. Casewell, Romulus Whitaker, Gerard Martin, Kartik Sunagar

**Affiliations:** 1 Evolutionary Venomics Lab. Centre for Ecological Sciences, Indian Institute of Science, Bangalore, Karnataka, India; 2 Centre for Snakebite Research & Interventions, Liverpool School of Tropical Medicine, Pembroke Place, Liverpool, United Kingdom; 3 Madras Crocodile Bank Trust/Centre for Herpetology. East Coast Road, Mamallapuram, Tamil Nadu, India; 4 The Liana Trust. Survey #1418/1419 Rathnapuri, Hunsur, Karnataka, India; University of Newcastle, AUSTRALIA

## Abstract

**Background:**

Snakebite in India results in over 58,000 fatalities and a vast number of morbidities annually. The majority of these clinically severe envenomings are attributed to Russell’s viper (*Daboia russelii*), which has a near pan-India distribution. Unfortunately, despite its medical significance, the influence of biogeography on the composition and potency of venom from disparate *D*. *russelii* populations, and the repercussions of venom variation on the neutralisation efficacy of marketed Indian antivenoms, remain elusive.

**Methods:**

Here, we employ an integrative approach comprising proteomic characterisation, biochemical analyses, pharmacological assessment, and venom toxicity profiling to elucidate the influence of varying ecology and environment on the pan-Indian populations of *D*. *russelii*. We then conducted *in vitro* venom recognition experiments and *in vivo* neutralisation assays to evaluate the efficacy of the commercial Indian antivenoms against the geographically disparate *D*. *russelii* populations.

**Findings:**

We reveal significant intraspecific variation in the composition, biochemical and pharmacological activities and potencies of *D*. *russelii* venoms sourced from five distinct biogeographic zones across India. Contrary to our understanding of the consequences of venom variation on the effectiveness of snakebite therapy, commercial antivenom exhibited surprisingly similar neutralisation potencies against the majority of the investigated populations, with the exception of low preclinical efficacy against the semi-arid population from northern India. However, the ability of Indian antivenoms to counter the severe morbid effects of *Daboia* envenoming remains to be evaluated.

**Conclusion:**

The concerning lack of antivenom efficacy against the north Indian population of *D*. *russelii*, as well as against two other ‘big four’ snake species in nearby locations, underscores the pressing need to develop pan-India effective antivenoms with improved efficacy in high snakebite burden locales.

## Author summary

The Russell’s viper (*Daboia russelii*), with a near-countrywide distribution, is arguably the deadliest snake species in India. Despite being responsible for the majority of snakebite deaths in the country, the influence of biogeography on its venom composition and potency, and the impact of this variation on snakebite therapy, is yet to be understood. Evaluation of the composition, biochemical activities, pharmacological implications and potencies of *D*. *russelii* venoms from five distinct biogeographic zones in India (>5,800 km) revealed remarkable intraspecific differences in venom profiles. Surprisingly, these observed differences did not seem to affect the marketed effectiveness of the commercial Indian antivenoms in countering the lethal effects of *D*. *russelii* venoms, with the exception of the North Indian semi-arid population. A similar lack of antivenom potency has also been documented in two other ‘big four’ snake species in these regions that suffer the brunt of snakebite. These alarming findings underscore the pressing need to develop pan-India efficacious antivenoms, especially for the treatment of snakebites in regions that are worst affected by this neglected tropical disease.

## Introduction

Globally, 5.4 million people suffer from snakebite, which results in over 137,000 annual deaths and nearly three times as many morbidities [1]. Asia accounts for over 70% of these cases, with India being its snakebite capital [1,2]. In India, the so-called ‘big four’ snakes, namely the spectacled cobra (*Naja naja*), common krait (*Bungarus caeruleus*), Russell’s viper (*Daboia russelii*) and saw-scaled viper (*Echis carinatus*), are considered to be the most medically important, with *D*. *russelii* seemingly being responsible for the majority of fatal envenomings and cases of long-term morbidity [2].

Variation in venom composition among and within snake species is seemingly driven by differing ecologies and environment and is a well-documented phenomenon [3–7]. These adaptive changes, particularly in a biodiverse region like the Indian subcontinent, can critically impact the clinical efficacy of antivenoms and, thus, poses an arduous challenge for countering geographical variations in snakebite pathologies. Considering its geological history and highly variable climatic and topological conditions, India can be divided into ten biogeographic zones: 1. Himalayas; 2. Trans-Himalayas; 3. Semi-arid regions; 4. Desert; 5. Western Ghats; 6. Deccan Plateau; 7. Gangetic Plain; 8. Coasts; 9. Northeast India; and 10. Islands [8]. Russell’s viper is arguably amongst the most widely distributed of the medically important Indian snakes and can be found in all biogeographical zones, with the exception of Trans-Himalayas, most of Northeast India and the Islands. The near-countrywide distribution across diverse habitats and presence even at elevations of 2,000 m and above in the Himalayan region is suggestive of the remarkable adaptability of this snake species. Despite this wide distribution, the influence of varying ecology and environment on *D*. *russelii* venom has not been comprehensively investigated to date. While some studies have assessed variability in venom proteomes and the influence of this variation on antivenoms via *in vitro* binding experiments [9–13], neutralisation potencies of commercial antivenoms are yet to be determined. Consequently, the absence of preclinical data has impeded the identification of the most medically important populations that may require more targeted therapy.

Here, we aim to bridge this knowledge gap by performing proteomic, biochemical and pharmacological characterisation of *D*. *russelii* venoms from five distinct biogeographic zones in India. We evaluate the influence of varying ecology and environment on the toxicity profiles of the pan-Indian populations of this species. Furthermore, using the World Health Organization (WHO)-recommended murine efficacy assays, we investigate the capability of conventional antivenoms to neutralise the venoms of geographically disparate *D*. *russelii* populations.

## Methods

### Ethics statement

WHO-recommended preclinical experiments were performed in the mouse model (CD-1 male mice; 18–22 g), following guidelines issued by the Committee for the Purpose of Control and Supervision of Experiments on Animals (CPCSEA), Government of India. All experiments were performed after obtaining the requisite approval from the Institutional Animal Ethics Committee (IAEC), Indian Institute of Science (IISc), Bangalore (CAF/Ethics/643/2018). A single best-binding antivenom was down-selected using *in vitro* assays (enzyme-linked immunosorbent assay) for testing the *in vivo* neutralisation potency of antivenom against *D*. *russelii* venoms. This down-selection strategy was crucial in greatly reducing the number of animals used in the *in vivo* experiments and minimising animal suffering. For investigating the coagulopathic effects of *D*. *russelii* venoms on human blood, ethical clearance was obtained from the Institute Human Ethical Committee (IHEC No: 5–24072019), and subsequently, blood was collected from healthy volunteers with informed consent.

### Sampling permits, snake venoms and antivenoms

Snake venoms were sourced from 48 individuals across a range of over 5800 km from various biogeographic zones of India, with appropriate permissions from the respective State Forest Departments: North- (Punjab: #3615;11/10/12), South- (Tamil Nadu), Southeast- (Andhra Pradesh:#13526/2017/WL-3), East- (West Bengal: 386/WL/4R-6/2017), Southwest- (Maharashtra: Desk-22(8)/Research/CR-80(16–17) /943/2017-18), and Central- (Madhya Pradesh: #/TK-1/48-II/606) India. The venoms were sampled individually or by pooling, flash-frozen with liquid nitrogen, and stored at -80° C after lyophilisation until further use. Sourced venom samples were subjected to preliminary reducing sodium dodecyl sulfate-polyacrylamide gel electrophoresis (SDS-PAGE) as a quality control measure and, thereafter, representative samples were down-selected for assessment. The details of *D*. *russelii* venom and commercial Indian antivenom samples analysed in this study are provided in Table 1 and S1 Table, respectively.

**Table 1:** Details of *D*. *russelii* venom samples.

Region	Biogeographical Zone	No. of individuals	Protein content (mg/ml)
North India Nawanshahr, Punjab (PB)	Semi-arid	2	0.430
South India Kancheepuram, Tamil Nadu (TN)	Coastal	*Multiple	0.680
Southeast India Visakhapatnam, Andhra Pradesh (AP)	Coastal	1	0.449
East India Kolkata, West Bengal (WB)	Gangetic Plain	1	0.488
West India Mahad, Maharashtra (MH)	Western Ghats	5	0.95
Central India Jabalpur, Madhya Pradesh (MP)	Deccan Plateau	3	0.293

Locations of *D*. *russelii* venoms sourced from various biogeographic zones in the country are listed in this table. State codes are indicated in parentheses, and the number of individual snakes from which these venoms were collected, and their protein concentrations have also been provided. The asterisk indicates the venom sample sourced from the Irula Snake Catchers Industrial Cooperative Society.

### Protein concentration

Lyophilised venom was reconstituted with molecular grade water, and the Bradford method was used for estimating protein concentration, with Bovine Serum Albumin (BSA) used as a standard [[14]; Table 1]. The total IgG content of antivenom was estimated by reconstituting the lyophilised contents of antivenom vials following the manufacturer’s guidelines and using the Bovine Gamma Globulin (BGG) standard curve (S1 Table).

### Gel electrophoresis

Reducing SDS-PAGE was performed to separate venom toxins. Venom samples, which were normalised for their protein content (12 μg), were run on a 12.5% gel in Tris-Glycine-SDS (TGS) buffer at 80 V [15], and the Precision Plus Protein Dual Color Standard (Bio-Rad) was used as a marker. The gel was stained with Coomassie Brilliant Blue R-250 (Sisco Research Laboratories Pvt. Ltd, India) and visualised in an iBright CL1000 gel documentation system (Thermo Fisher Scientific, USA).

### Reversed-phase high-performance liquid chromatography (RP-HPLC)

A slightly modified version of a previously published protocol [16] was used to fractionate the reconstituted venoms in a Shimadzu LC-20AD series HPLC system (Kyoto, Japan). A reverse phase C18 column with dimensions 4.6 x 250 mm, 5 μm particle size, and pore size of 300 Å (Shimadzu, Japan), was equilibrated with solution A [0.1% Trifluoroacetic acid (TFA) in water (v/v)] and loaded with 200 μg of each venom sample for fractionation. A constant flow rate of 1 ml/min was used for eluting fractions with the following concentration gradients of solution B [0.1% TFA in 100% acetonitrile (v/v)]: 5–15% for 10 mins, 15–45% over the next 60 mins, and finally 45–70% for 10 mins, and the absorbance was monitored at 215 nm.

### Liquid chromatography-tandem mass spectrometry (LC-MS/MS)

HPLC fractions (40 μg each) were subjected to electrospray ionisation tandem mass spectrometry (ESI-MS/MS) for the characterisation of proteomic profiles. Samples were reduced with dithiothreitol (DTT; 10 mM), alkylated using iodoacetamide (IAA; 30 mM), and subsequently digested with trypsin (0.2 μg/μl) overnight and desalted. A Thermo EASY nLC 1200 series system (Thermo Fisher Scientific, MA, USA) with a C18 nano-LC column (dimension 50 cm x 75 μm, 3 μm particle size and 100 Å pore size) was used for liquid chromatography of these processed samples. A sample volume of 2 μl was injected into the column and run with buffer A (0.1% formic acid in HPLC grade water) and buffer B (0.1% formic acid in 80% acetonitrile) solutions at a constant flow rate of 300 nl/min for 120 mins. The gradient of buffer B (10–45%) was used for the elution over the first 98 mins, followed by 45–95% over the next 4 mins and finally 95% for the last 18 mins. A Thermo Orbitrap Fusion Mass Spectrometer (Thermo Fisher Scientific, MA, USA) was used for mass spectrometric analyses of the samples. MS scans were performed using the following parameters: scan range (m/z) of 375–1700 with a resolution of 120000 and maximum injection time of 50 ms. Fragment scans (MS/MS) were performed using an ion trap detector with high collision energy fragmentation (30%), scan range (m/z) of 100–2000, and maximum injection time of 35 ms.

For identification of various toxin families in the proteomic profiles of venom fractions, the raw MS/MS spectra were searched against the SwissProt database (www.uniprot.org) using PEAKS Studio X Plus (Bioinformatics Solutions Inc., ON, Canada) with the following parameters: parent and fragment mass error tolerance limits of 10 ppm and 0.6 Da, respectively; ‘monoisotopic’ precursor ion search type; and ‘semispecific’ trypsin digestion. Carbamidomethylation and oxidation were specified as fixed and variable modifications, respectively. Error in peptide identification was minimised by fixing the False Discovery Rate (FDR) for peptide-spectrum matching at 0.1%, and the corresponding -10lgP cutoff value was automatically determined by PEAKS Studio. Hits with at least one unique matching peptide were considered for downstream analyses. Mass spectrometry data have been deposited to the ProteomeXchange Consortium via the PRIDE partner repository [17], with data identifier: PXD021060. The relative abundance of each toxin hit in a fraction was determined by estimating its area under the curve (AUC) for spectral intensities, obtained from PEAKS analysis [18], relative to the total AUC for all toxins in that fraction. These values were further normalised across fractions using the percentage of peak areas for the respective RP-HPLC fractions [19]. Thus, the relative abundance of a toxin hit (X) was calculated as follows:

$$Relative\;abundance\;of\;X(\%)=\sum_{n=1}^{N}\frac{AUC\;of\;X\;in\;Fraction\;F_{n\;}\times AUC\;of\;the\;chromatographic\;fraction\;F_{n}(\%)}{Total\;AUC\;of\;all\;toxin\;hits\;in\;fraction\;F_{n}}$$

Here, N indicates the number of fractions obtained from RP-HPLC.

### Venom biochemistry

Venom samples were assayed for various biochemical activities following previously described protocols [20] and are thus detailed in brief below.

### Phospholipase A_2_ (PLA_2_) assay

The PLA_2_ activity of the venom samples was assessed via turbidimetric assay. The substrate for the reaction was freshly prepared with chicken egg yolk dissolved in 0.9% NaCl solution, and its absorbance was made up to one at 740 nm [20,21]. The time-dependent kinetic assay was performed in triplicate with different concentrations of each venom sample (0.01 μg, 0.1 μg, 0.5 μg, and 1 μg) prepared in a 20 mM Tris-Cl buffer. The resulting absorbance was measured at 1-minute intervals for 60 mins at 740 nm using an Epoch 2 microplate spectrophotometer (BioTek Instruments, Inc., USA). Unit activity was calculated as the amount of crude venom required to reduce the absorbance of the substrate by 0.01 OD unit per min at the given wavelength [22].

### Snake venom protease assay

Proteolytic venom activity was assayed following a previously described protocol [23], wherein a pre-defined volume of azocasein substrate was incubated with a known concentration of crude venom at 37° C for 90 mins. The reaction was stopped with trichloroacetic acid post-incubation, and the resulting mixture was further subjected to centrifugation at 1000 x g for 5 mins, mixed with an equal volume of 0.5 M NaOH and finally, absorbance was measured at 440 nm. The relative protease activity of crude venoms was calculated in comparison with the purified protease from the bovine pancreas (Sigma-Aldrich, USA).

### L-amino acid oxidase (LAAO) assay

LAAO activity of snake venoms was evaluated with an endpoint assay that uses L-leucine as substrate, following a previously described protocol [20,24]. A mixture of crude venom and L-leucine (5 mM L-leucine, 50 nM Tris-HCl buffer, 5 IU/ml horseradish peroxidase, 2 mM o-phenylenediamine dihydrochloride) in 1:9 proportion was incubated at 37° C for an hour. The reaction was terminated with 2 M H_2_SO_4_, and an Epoch 2 microplate spectrophotometer was used to record the absorbance at 492 nm.

### DNase assay

The DNase activities of the venoms were assessed using a method described by Gercerker *et al*. [25] with slight modifications [20]. A predefined concentration of crude venom (0.05 μg/μl) was incubated with purified calf thymus DNA (Sigma-Aldrich, USA), reconstituted in phosphate buffer saline (PBS), followed by incubation at 37°C for 60 mins. Reaction mixtures were then subjected to electrophoresis on a 0.8% agarose gel and visualised on an iBright CL1000 gel documentation system.

### Fibrinogenolytic assay

The ability of *Daboia* venoms to degrade human fibrinogen was evaluated using an electrophoresis-based method, previously described by Ouyang and Teng [26]. Human fibrinogen (Sigma-Aldrich, USA) dissolved in PBS was incubated with a known concentration of venom (1.5 μg) at 37°C for 60 mins. Following the addition of an equal volume of loading dye (1 M Tris-HCl pH 6.8, 50% Glycerol, 0.5% Bromophenol blue, 10% SDS, 20% β-mercaptoethanol), the mixture was heated at 70° C for 10 mins. Samples were further subjected to 15% SDS-PAGE, and the gel was stained with Coomassie Brilliant Blue R-250 before visualisation in an iBright CL1000 (Thermo Fisher Scientific, USA) gel documentation system. The results were interpreted in comparison to a negative control consisting of human fibrinogen only, where all three bands (different chains of reduced fibrinogen) are observed intact.

### Blood coagulation assays

To assess the capability of *D*. *russelii* venom to perturb the coagulation cascade, specifically the extrinsic and intrinsic pathways, we quantified venom-induced alterations to the prothrombin time (PT) and partial thromboplastin time (aPTT), respectively. Blood samples collected from healthy male volunteers were centrifuged at 3000 x g for 10 mins at 4° C to separate platelet-poor plasma (PPP). Prewarmed calcium thromboplastin reagent (Uniplastin; Tulip diagnostics, Mumbai; 200 μl) and activated cephaloplastin reagent (Liquicelin-E; Tulip diagnostics, Mumbai; 100 μl) with 0.02 M Calcium chloride (CaCl_2;_ 100 μl) were mixed with 50 μl of PPP as per the manufacturer’s protocol, for measuring PT and aPTT, respectively. Following this, the mixture was treated with different concentrations of *D*. *russelii* venoms (0.5 or 5 μg in 50 μl), and the time taken for the appearance of the first fibrin clot was measured using a Hemostar XF 2.0 coagulometer. The results have been represented in the form of a heat-map generated using Graphpad Prism 8 (GraphPad Software, San Diego, California USA, www.graphpad.com).

### Turbidimetric coagulation assay

Procoagulant activities of *D*. *russelii* venoms were assessed by mixing various concentrations of venoms (15.6 to 250 ng/ml) with equal volumes of freshly collected PPP and 0.2 M CaCl_2_ (60 μl for 1 ml of PPP) at 37°C, following a previously described protocol [27]. The increase in turbidity of the mixture was recorded by measuring the optical density (OD) at 340 nm for every 60 seconds, over a period of 60 mins, in an Epoch 2 microplate spectrophotometer. A graph showing time versus absorbance was plotted, and the clotting time was defined as the time point where the OD increases by 0.02 units over the average OD units measured at the first two time points [27]. Subsequently, the neutralisation of procoagulant activities by the Indian polyvalent antivenom (Premium Serums) was assessed by incubating various concentrations of the venom (15.6 to 250 ng/ml) with 0.25 μg/μl (1:4) and 0.0625 μg/μl (1:16) of the antivenom at 37° C for 30 mins, followed by the addition of plasma and CaCl_2_ [28]. The assays were performed in triplicates.

### Haemolytic assay

The haemolytic activity of snake venom was determined by assessing the degradation of human red blood cells (RBC) using a previously described protocol [29]. RBCs isolated from whole blood were resuspended with 1X PBS and centrifuged at 3000 x g for 10 mins at 4° C. After centrifugation, the supernatant was discarded, and the RBC pellet was resuspended again in 1X PBS. The above procedure was repeated five times to remove undesirable blood factors and cellular debris. Thereafter, a 1% RBC suspension was prepared and mixed with various concentrations of the venoms (5, 10, 20 and 40 μg) in a 10:1 ratio and the reaction mixture incubated at 37° C for 24 hours. Following incubation, samples were centrifuged at 3000 x g for 10 mins, and the absorbance of the supernatant was measured at 540 nm using an Epoch 2 microplate spectrophotometer. The relative haemolytic activity of the venoms was calculated using 0.5% Triton X as the positive control and after accounting for background absorbance.

### Enzyme-linked immunosorbent assay (ELISA)

We investigated the *in vitro* venom recognition (i.e., binding) of commercial antivenoms using a previously described ELISA protocol [20,30]. Venom samples (100 ng) were diluted in a carbonate buffer (pH 9.6) and coated onto 96-well plates. Following overnight incubation at 4° C, the unbound venom was removed by washing with Tris-buffered saline with 1% Tween 20 (TBST). The venom-bound plate was then incubated with a blocking buffer (5% skimmed milk in TBST) for 3 hours at room temperature. The plate was then subjected to another round of TBST washing, which was followed by the addition of various dilutions of commercial equine Indian antivenoms (Premium Serums, VINS, Bharat, and Haffkine). Plate was then incubated overnight at 4°C, and the unbound antibodies removed by a TBST wash the next day. Horseradish peroxidase (HRP)-conjugated rabbit anti-horse secondary antibody (Sigma-Aldrich, USA) diluted in PBS (1:1000) was added, and the plate was incubated at room temperature for 2 hours. Post incubation, ABTS (2,2’-azino-bis(3-ethylbenzothiazoline-6-sulfonic acid)) substrate solution (Sigma-Aldrich, USA) was added and the absorbance measured at a wavelength of 405 nm for 40 mins in Epoch 2 microplate reader. IgG from a naive horse (Biorad) was used as the negative control to determine the cut-off for non-specific antibody binding, as described previously [20,30].

### Immunoblotting

Venom and antivenom immunoblotting experiments were performed following a previously described protocol [20,30]. Following the electrophoretic separation of crude venoms using SDS-PAGE (12.5%), a nitrocellulose membrane was used to electrotransfer the proteins as per the manufacturer’s instructions (BioRad, USA). To verify transfer efficacy, Ponceau S reversible stain was used, and the membrane was then incubated with a blocking buffer at 4° C overnight. After washing with TBST, the membrane was incubated with a known concentration of commercial antivenom at 4° C. On the following day, an HRP-conjugated rabbit anti-horse secondary antibody was added at a dilution of 1:2000, followed by six TBST washes to remove the unbound antivenom. Finally, following the manufacturer’s instructions (Thermo Fisher Scientific, USA), an enhanced chemiluminescence substrate was used to visualise the binding efficacy of commercial antivenoms to venom, and the membrane was imaged in an iBright CL1000 (Thermo Fisher Scientific, USA).

### *In vivo* venom toxicity and antivenom efficacy assays

Preclinical experiments were conducted using a murine model of envenoming to evaluate the toxicity of *D*. *russelii* venoms from various biogeographical zones and the efficacy of the marketed Indian antivenom in neutralising the lethal effects of these venoms.

### The intravenous median lethal dose (LD_50_)

The potencies of *Daboia* venoms from distinct biogeographic zones were determined by calculating the LD_50_ values (or the amount of venom required to kill 50% of the test population) by following WHO-recommended murine assay protocols [31]. Five concentrations of each crude venom were prepared in physiological saline (0.9% NaCl) and injected intravenously into the caudal vein of male CD-1 mice (500 μl/mouse). Subsequently, the number of dead and surviving mice in each venom dose group (n = 5) was recorded 24 hours later, and the LD_50_ and 95% confidence intervals were calculated using Probit statistics [32].

### The median effective dose (ED_50_) of antivenom

The preclinical efficacy of commercial antivenom in neutralising the venoms of pan-Indian populations of *D*. *russelii* was evaluated by calculating the ED_50_ value or the minimum amount of antivenom required to protect 50% of the test population injected with lethal doses of venom [31]. We selected the antivenom manufactured by Premium Serums for use in ED_50_ testing based on its superior *in vitro* venom recognition potential (determined by ELISA and western blotting experiments). This *in vitro* guided strategy significantly reduced the number of mice sacrificed in ED_50_ experiments. Briefly, various volumes of antivenoms were mixed with the challenge dose of venom (equivalent to 5X LD_50_ of each venom), followed by an incubation period of 30 mins at 37°C. Post incubation, each venom-antivenom mixture (n = 4 per venom) was intravenously injected into a group of five male CD-1 mice (18–22 g). The number of surviving and dead animals were monitored over 24 hours, and the resulting ED_50_ values and 95% confidence intervals were calculated using Probit statistics [32]. The volume of antivenom (μl) required to neutralise one milligram of venom was estimated following a recently proposed method [33]. The neutralisation potency of the commercial antivenom was calculated with the equation [20,34]. Here, n represents the number of LD_50_ used as the challenge dose.

$$Antivenom\;neutralisation\;potency(mg/ml)=\frac{\left(n-1){\times LD}_{50}\;of\;venom(mg/mouse\right)}{{ED}_{50}(ml)}$$

### Statistical analysis

Statistical comparisons of data generated in the various biochemical and ELISA assays were performed using One-way ANOVA and Two-way ANOVA with Tukey’s and Dunnett’s multiple comparison tests in GraphPad Prism (GraphPad Software 8.0, San Diego, California USA, www.graphpad.com).

## Results

### Venom proteomics

**Fig 1:**
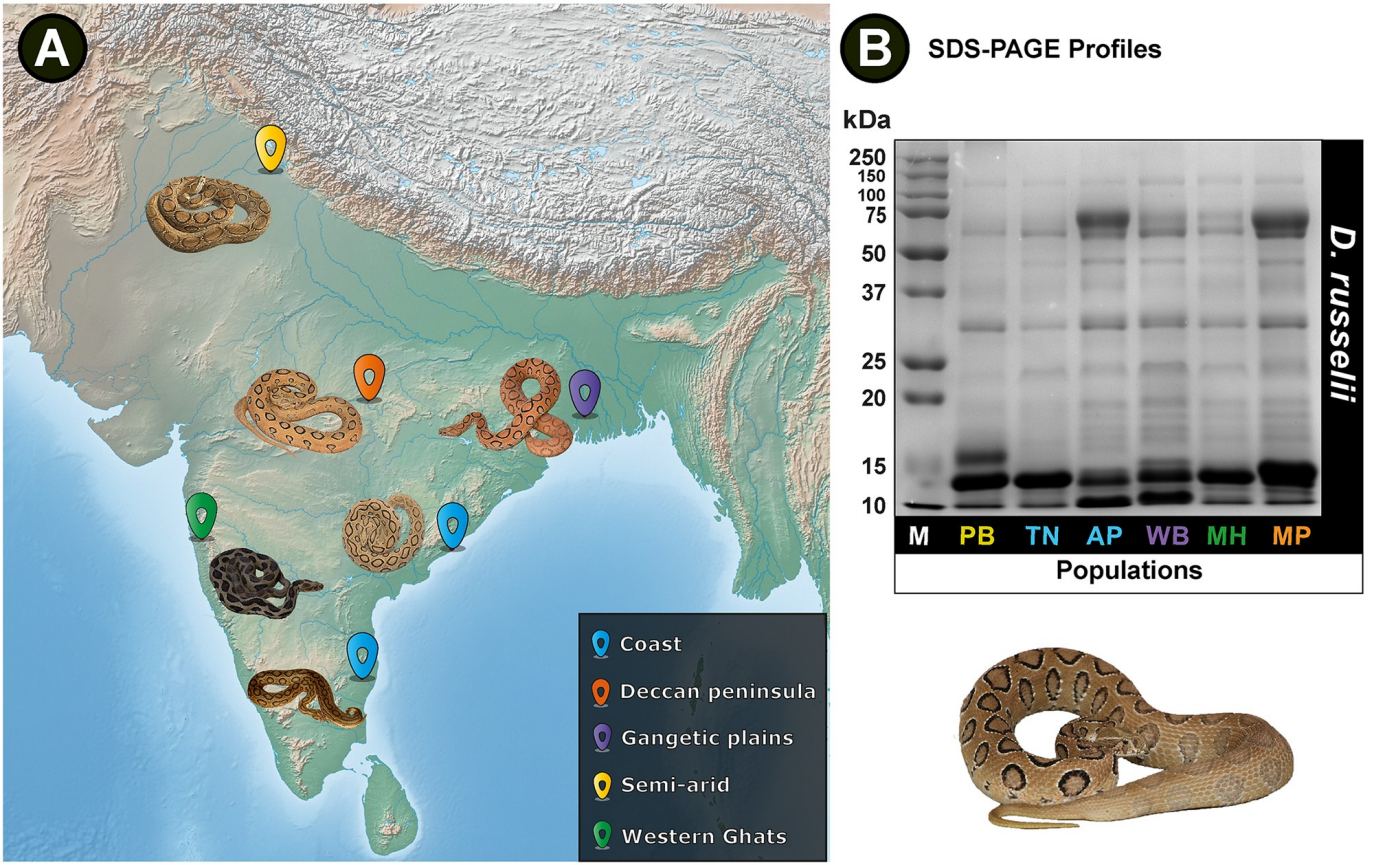
Map of India, prepared with QGIS 3.8.2 [35], depicts (A) sampling locations and (B) SDS-PAGE profiles of *D*. *russelii* venoms. **M:** Protein marker (units in kDa); **PB:** Punjab; **TN:** Tamil Nadu; **AP:** Andhra Pradesh; **WB:** West Bengal; **MH:** Maharashtra; **MP:** Madhya Pradesh.

Venoms sourced from *D*. *russelii* populations from five distinct biogeographical zones across India were separated using SDS-PAGE under reducing conditions (Table 1 and Fig 1). Analyses of SDS-PAGE profiles revealed considerable differences in band intensities and patterns, highlighting the significant interpopulation venom variation in this species (Fig 1).

**Fig 2:**
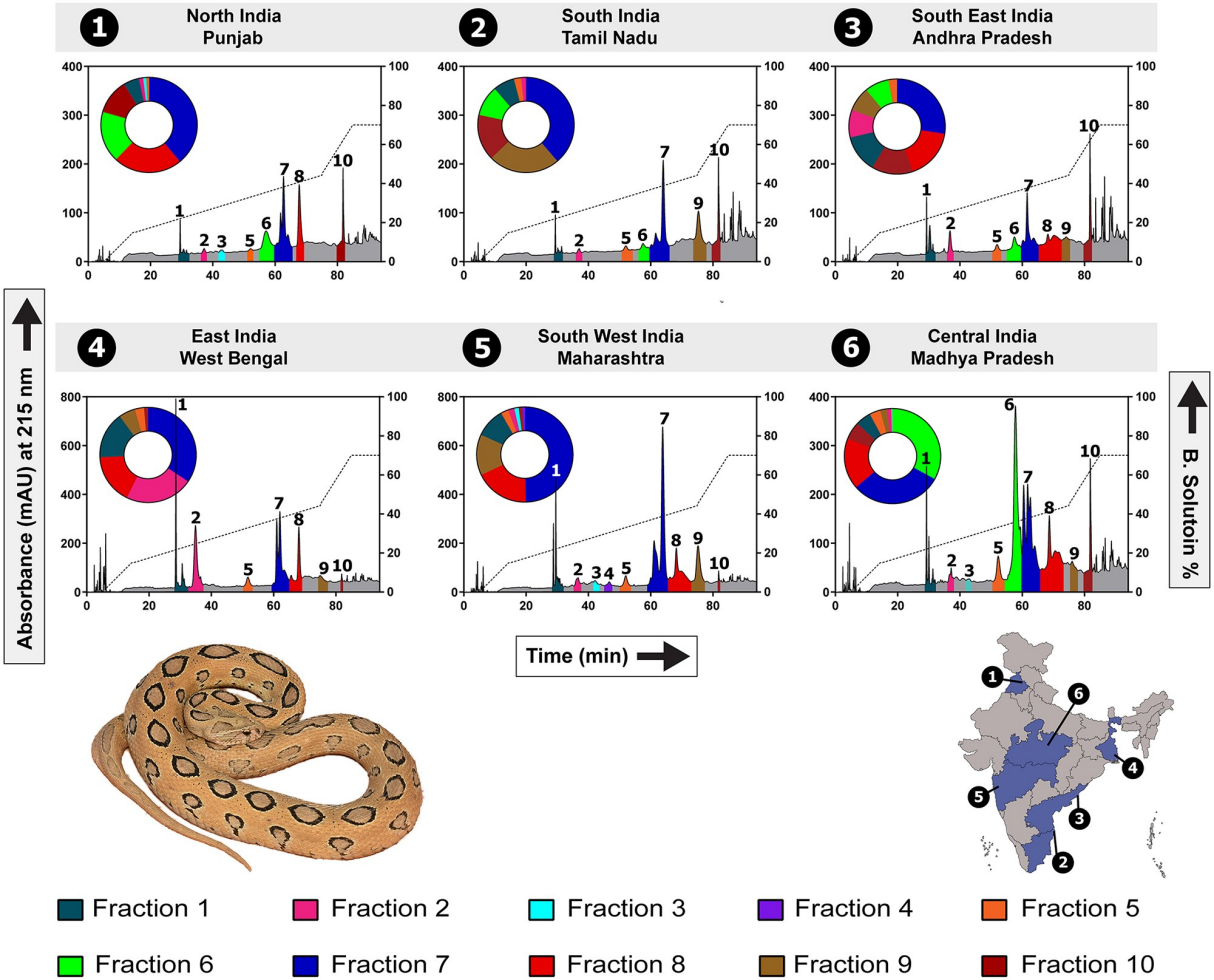
Venom variability in pan-Indian *D*. *russelii* populations. This figure depicts RP-HPLC profiles of *D*. *russelii* venoms sourced from distinct biogeographic zones. Venom profiles, shown here, highlight the considerable differences in the protein compositions of geographically distinct populations. The area under the curve for all labelled and uniquely colour coded fraction peaks are depicted in the corresponding doughnut charts.

To further resolve intraspecific variation, *D*. *russelii* venom samples were subjected to RP-HPLC, which unravelled finer differences in the venom compositions across the biogeographical zones (Fig 2). RP-HPLC profiles revealed ten major fractions/peaks, with notable differences observed in peaks #1, #6, #7 and #8 between samples. Interestingly, peak #6 was completely absent in the Western Ghats (MH) and Gangetic Plain (WB) populations while dominating the venom of the Deccan Plateau (MP) population. Similarly, peak #8 enriched the venoms of the semi-arid (PB) and Deccan Plateau (MP) populations while being absent from one of the coastal (TN) populations.

Populations from the Gangetic Plain (WB), Western Ghats (MH) and Deccan Plateau (MP) were selected for mass spectrometry analyses based on their unique HPLC and toxicity profiles (see below). Tandem mass spectrometry of venom fractions (n = 10) from these samples identified between 49 and 66 proteins from 13 toxin families, including phospholipase A_2_ (PLA_2_), Kunitz-type serine protease inhibitor (Kunitz), snake venom serine protease (SVSP), cysteine-rich secretory protein (CRISP), snaclec, snake venom metalloproteinase (SVMP), three-finger toxin [3FTx; neurotoxic-3FTx (N-3FTx) and cytotoxic-3FTx (C-3FTx)], L-amino-acid oxidase (LAAO), nerve growth factor (NGF), disintegrin, vascular endothelial growth factor (VEGF), 5’ Nucleotidase (5’-NTD) and C-type lectin (CTL) (S2–S4 Tables, S1 Data). The identification of 3FTx in *Daboia* venoms is particularly noteworthy as these toxins are rarely reported in the venom proteomes of viperid snakes [36,37].

**Fig 3:**
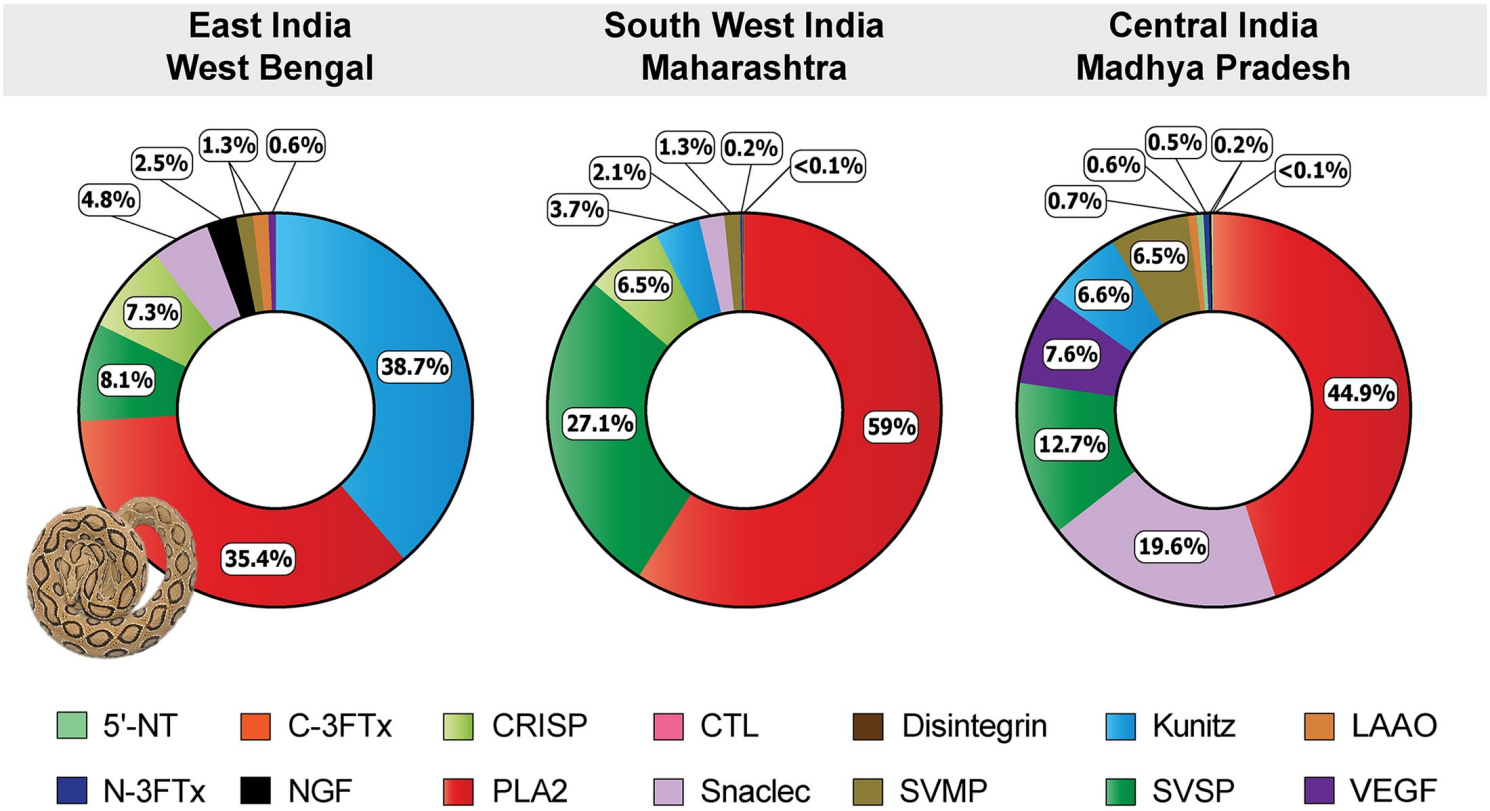
Comparative venom profiles of the biogeographically disparate *D*. *russelii* populations. This figure represents the relative composition of various toxins constituting *D*. *russelii* venoms, as estimated from the mass spectrometry and HPLC data. Each toxin type is uniquely colour coded, and its relative abundance is indicated.

Tandem mass spectrometry revealed significant differences in the venom compositions of the investigated *D*. *russelii* populations (Fig 3). Kunitz-type serine protease inhibitors were found to be more abundant in the venom of the Gangetic Plain (WB) population (~39%), in line with previous reports [38], whereas the Deccan Plateau (MP) and the Western Ghats (MH) populations contained very limited amounts of this toxin: 7% and 4%, respectively (Fig 3 and S2–S4 Tables). Considerable differences in the proportions of PLA_2_s were also observed. While 59% of the venom of the Western Ghats (MH) population consisted of PLA_2_, the Gangetic Plain (WB) and the Deccan Plateau (MP) populations respectively contained 35% and 45% of this toxin in their venom. In contrast to the previous report [9], SVSPs were found to be comparatively more abundant in the Western Ghats (MH) population (27%) than the Deccan Plateau (MP, 13%) and Gangetic Plain (WB, 8%) populations. Interestingly, snaclecs constituted nearly ~20% of the venom proteome of the Deccan Plateau (MP) population, whereas the Gangetic Plain (WB) and the Western Ghats (MH) populations contained only 5% and 2% of this toxin, respectively. While VEGF, a family of toxins responsible for inducing vascular permeability and lower blood pressure [39,40], was only identified in trace amounts in the other populations of *D*. *russelii* (<1%), it was found to constitute ~8% of the venom of the Deccan Plateau (MP) population (S2–S4 Tables).

### Venom biochemistry

The snake venom arsenal, which is composed of toxins with tremendous compositional and functional diversity, can inflict varied clinical manifestations in snakebite victims. To understand the differences in the biochemical and pharmacological effects of venoms from the pan-Indian populations of *D*. *russelii*, we subjected them to a battery of biochemical (PLA_2_, protease, LAAO, DNase, fibrinogenolytic) and pharmacological (haemolytic assay, plasma coagulation assay, prothrombin time and activated partial thromboplastin time) assessments.

### PLA_2_ assay

Snake venom PLA_2_s are known to exert a wide range of pharmacological effects in bite victims, including cytotoxicity, neurotoxicity, myotoxicity and perturbation of haemostasis [41]. Given their medical relevance, we assessed the abilities of *D*. *russelii* venoms in hydrolysing phospholipid substrates. The enzymatic PLA_2_ activity profiles of *D*. *russelii* differed between populations (p<0.05), with the Deccan Plateau (MP) population exhibiting the highest activity, while the coastal populations (TN and AP) showed intermediate activities that were indistinguishable from each other (S1A Fig). While *D*. *russelii* populations from the Western Ghats (MH) and Gangetic Plain (WB) were statistically similar to each other in their PLA_2_ activities, they were significantly lower than all other *D*. *russelii* venom samples under study (p<0.05). The semi-arid (PB) population exhibited the lowest PLA_2_ activity among all the tested populations. Low PLA_2_ activity, despite relatively high abundance in the proteome, is probably suggestive of the presence of non-catalytic PLA_2_s that enrich the venoms of many viperid snakes [42–44].

### Snake venom protease assay

Venoms of many snake species may contain a variety of proteolytic enzymes that cause serious clinical pathologies in bite victims [45,46]. Viperid venoms, in particular, are known to be dominated by SVSPs and SVMPs that disrupt haemostasis by targeting various factors involved in the blood coagulation cascade (e.g., thrombin, fibrinogen, plasminogen and platelets), often synergistically [47–49]. Examination of *D*. *russelii* venoms from distinct biogeographic locations revealed highly variable degrees of proteolysis. While the Deccan Plateau (MP) population and one of the coastal populations (AP) exhibited the highest proteolytic activities, followed by the population from the Western Ghats (MH), all others showed negligible effects (p<0.05; S1B Fig). Interestingly, the Western Ghats (MH) population that was rich in the overall protease content (SVSP and SVMP) exhibited relatively reduced proteolytic activity in comparison to the SVMP-rich Deccan Plateau (MP) population (p<0.05). Similarly, the low proportion of proteases in the Gangetic Plain (WB) population could be reflective of the negligible protease activity observed.

### LAAO assay

Snake venom LAAOs are theorised to contribute to the oxidative stress in bite victims as they catalyse the oxidative deamination of L-amino acids into ɑ-keto acids, releasing H_2_O_2_ as a byproduct [50]. Consequently, despite being secreted in relatively minimal amounts in many snake venoms, LAAO may contribute to a diversity of toxic effects, including cytotoxicity, induction of cell death, haemorrhage and inhibition of platelet aggregation [51–53]. When the crude venoms of *D*. *russelii* were tested for their ability to catalyse the oxidative deamination of L-amino acids, they exhibited substantial activity (S1C Fig). Though statistically significant differences in LAAO activities were observed between populations (p<0.05), further experiments are required to assess the clinical importance of this difference.

### DNase assay

Recently, it has been shown that the venoms of certain viperid snakes are capable of inducing a phenomenon called ETosis in bite victims, wherein cells extrude their nuclear DNA to form extracellular traps (ETs) [54,55]. As these traps restrict the diffusion of venom toxins, ETosis may significantly contribute to local tissue damage. However, the DNase activity of certain snake venoms can facilitate the diffusion of venom toxins by cleaving such extracellular traps [56,57]. Taking this into account, the DNase activities of the geographically distinct *D*. *russelii* venoms were assessed. With the exception of the semi-arid (PB) population, which showed atypically high activity, all other populations exhibited low to negligible DNase venom activities (S1D and S2 Figs). Additional experiments are necessary to understand the biological consequence of the unusually high DNase activity of the semi-arid (PB) *D*. *russelii* population.

### Fibrinogenolytic assay

Snake venoms are capable of perturbing the haemostasis of bite victims by cleaving the fibrinogen glycoprotein complex [58], which is composed of Aɑ, Bβ and γ subunits. When the fibrinogenolytic potential of *D*. *russelii* venoms was assessed, barring the semi-arid (PB) and Gangetic Plain (WB) populations, all *D*. *russelii* venoms either completely or partially cleaved the Aɑ subunit, while none of the venoms degraded the Bβ- and γ-chains (S3 Fig).

### Blood coagulation assays

**Fig 4:**
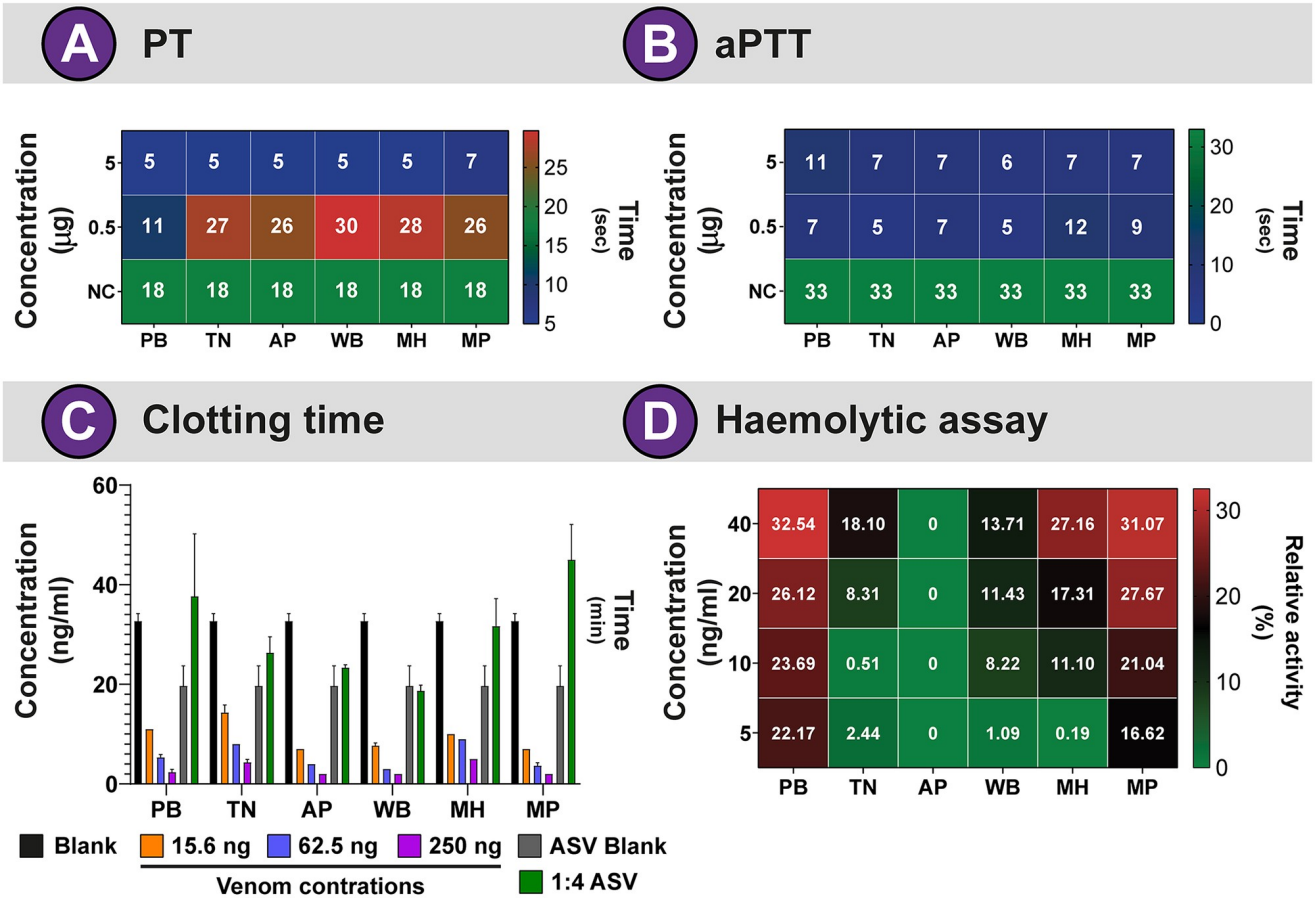
Coagulopathies induced by the biogeographically distinct populations of *D*. *russelii*. Heatmaps depict the influence of *D*. *russelii* venoms in impeding the **(A)** extrinsic and **(B)** intrinsic blood coagulation pathways, respectively. **(C)** The impact of varying concentrations of venoms (15.6 to 250 ng) on plasma clotting time (mins), and the ability of Premium Serums antivenom (1:4 dilution of 1 mg/ml or 0.25 μg/μl antivenom) in neutralising these effects are shown as bar graphs. The error bars represent the standard deviation among the replicates. **(D)** Haemolytic activities of *D*. *russelii* venoms have also been shown. Numbers inside each tile in A and B denote the plasma clotting time in seconds, and the numbers in D indicate the percentage of relative activity with respect to the positive control (0.5% Triton X). Blank: plasma control; **ASV Blank:** plasma + antivenom (1:4); **NC:** negative control; **PB:** Punjab; **TN:** Tamil Nadu; **AP:** Andhra Pradesh; **WB:** West Bengal; **MH:** Maharashtra; **MP**: Madhya Pradesh.

Viperid venoms are known to perturb hemostasis by inducing a range of clinical symptoms, such as local and systemic haemorrhage, venom-induced consumption coagulopathy (VICC), and alteration of blood pressure and vascular permeability [59]. Amongst the plethora of toxins in viper venoms, PLA_2_, SVSP and SVMP are known to exhibit potent procoagulant or anticoagulant effects by cleaving coagulation factors involved in the intrinsic or extrinsic coagulation cascade [60–62]. To evaluate the effects of *D*. *russelii* venoms on the intrinsic and extrinsic coagulation cascades, we estimated the prothrombin time (PT) and activated partial thromboplastin time (aPTT), respectively. The venoms of *D*. *russelii* from distinct biogeographies exhibited potent procoagulant activities, altering both the extrinsic and intrinsic pathways (Fig 4A and 4B). Notably, even at very low concentrations (5 μg), all *Daboia* venoms exhibited significant procoagulant effects within ~5 seconds (Fig 4A and 4B). In contrast, certain *D*. *russelii* populations have been preclinically [63] and clinically [64] documented to induce potent anticoagulant effects at higher venom concentrations. Anticoagulatory effects in bite victims are primarily caused by VICC, wherein the coagulation factors of the blood are depleted due to the activities of the procoagulant snake venom toxins, resulting in life-threatening haemorrhage [59]. As *D*. *russelii* can inject very large amounts of venom (an average of 200 mg of venom was extracted from 12 Russell’s vipers in this study), potent anticoagulant effects are likely seen in bite victims [65].

A turbidimetric plasma clotting time assay revealed similar procoagulatory effects of *Daboia* venoms. Consistent with the results of the PT and aPTT experiments, all populations were found to exhibit strong procoagulant effects (clotting time between 2 and 5 mins) (Fig 4C and S4 Fig). Moreover, when the ability of the best binding antivenom (Premium Serums; see the outcomes of binding experiments below) in neutralising these effects were tested under *in vitro* conditions, the clotting time of the plasma mixed with either the venom of the semi-arid (PB) population or the Deccan Plateau (MP) population was considerably prolonged (Fig 4D and S4 Fig). This is, perhaps, indicative of the neutralisation of procoagulant toxins, and not those responsible for anticoagulant effects. These experiments yielded identical results despite the repetition. In complete contrast, despite the treatment with the antivenom, considerable procoagulant effects were still seen in the Gangetic Plain (WB) and one of the coastal (AP) populations (Fig 4D and S4 Fig). Interestingly, the addition of antivenom to the venom of *D*. *russelii* from Western Ghats (MH) and the other coastal (TN) region resulted in clotting times that were comparable to the blank, suggesting the presence of procoagulant toxin-neutralising antibodies in the antivenom (Fig 4D and S4 Fig).

### Haemolytic assay

Intravascular haemolysis, which often results in adverse conditions, including renal failure, has been demonstrated as one of the major clinical pathologies of *D*. *russelii* envenomings [66]. The haemolytic effect of *D*. *russelii* venoms is predominantly attributed to secretory PLA_2_s that hydrolyse the phospholipid bilayer on the cellular membrane [67,68]. Since a significant effect of the venom was observed on egg yolk phospholipids in PLA_2_ assays, we assessed the direct haemolytic activity of *D*. *russelii* venoms on human erythrocytes. Here, the Gangetic Plain (WB) and coastal (AP and TN) populations of *D*. *russelii* exhibited negligible to no direct haemolytic activities (0 to 18%) at the highest venom concentration tested (40 μg), whereas the semi-arid (PB), Western Ghats (MH) and Deccan Plateau (MP) populations exhibited moderately increased haemolytic effects [26 to 31%; (Fig 4E)]. It is interesting to note that the semi-arid (PB) population showed increased haemolysis despite low PLA_2_ activity (S1A Fig), suggesting the potential pharmacological role of non-catalytic PLA_2_s or other toxin constituents [42,43].

### *In vitro* venom recognition potential of commercial Indian antivenoms

**Fig 5:**
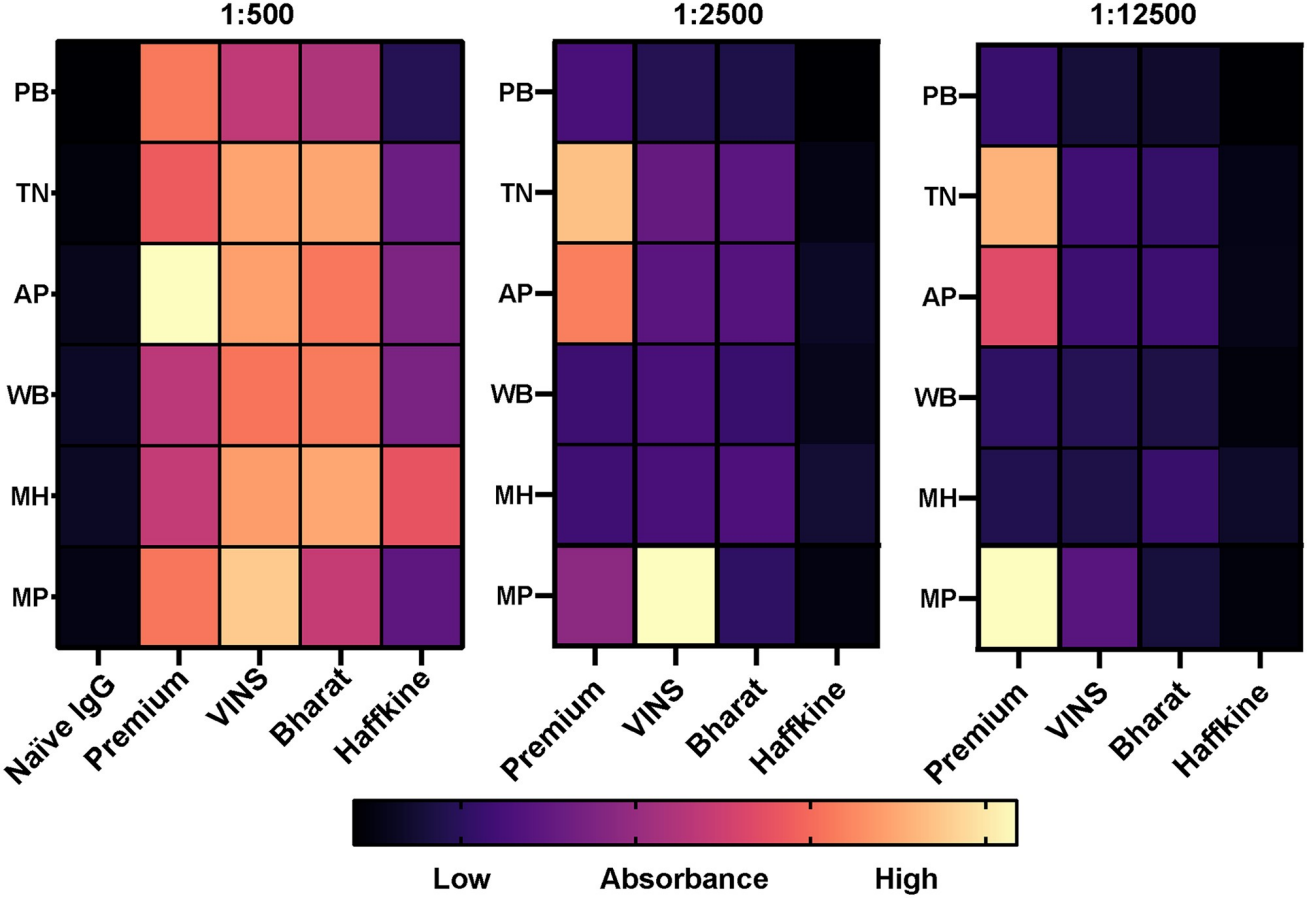
Immunological cross-reactivity of commercial Indian antivenoms against the pan-Indian populations of *D*. *russelii* venoms. Heatmaps, shown here, quantify the binding of commercial Indian antivenoms to the venoms of pan-Indian populations of *D*. *russelii*. Multiple dilutions of antivenoms (1:500, 1:2500 and 1:12500) were tested in indirect ELISA experiments. Non-specific binding of naive horse IgGs (1:4 dilution) to *Daboia* venoms is also shown in the first plate for reference. A gradient colour scale has been shown indicating the degree of binding from low (black) to high (cream). PB: Punjab; TN: Tamil Nadu; AP: Andhra Pradesh; WB: West Bengal; and MH: Maharashtra; MP: Madhya Pradesh.

The compositional differences in snake venoms from distinct populations are known to significantly affect the immunological cross-reactivity of antivenoms [5,10,63,69], thereby reducing their potential clinical effectiveness. We, therefore, assessed the venom recognition potential of the major Indian commercial polyvalent antivenoms against the venoms of the pan-Indian populations of *D*. *russelii*. In indirect ELISA experiments, the Premium Serums antivenom consistently recognised the venoms of the pan-Indian populations of *D*. *russelii* to a greater extent (end-point titres between 1:2500 to 1:12500) than that of its comparators, followed by the VINS antivenom (1:2500) (Fig 5 and S5 Fig). On the contrary, antivenoms manufactured by Bharat Serums (1:500 to 1:2500) and Haffkine (1:500) exhibited considerably poorer venom recognition capabilities. Surprisingly, despite sourcing venoms from the Maharashtra *Daboia* population as immunogens for antivenom production, the Haffkine antivenom failed to exhibit high binding titres against all populations, including this source population.

Furthermore, immunoblotting experiments, which were performed to identify venom toxins that are recognised by antivenoms, revealed that the Premium Serums antivenom recognised many low-, mid- and high-molecular-weight toxins found in the venom of the pan-Indian populations of *D*. *russelii* (S6A and S6B Fig). In contrast, the commercial products manufactured by VINS and Haffkine exhibited increased recognition only towards high- (> 50 kDa) and low- (<15 kDa) molecular weight toxins, while poor immunorecognition was observed in the case of Bharat Serum antivenoms (S6A and S6B Fig).

### Venom potency by murine median lethal dose (LD_50_) assay

**Fig 6:**
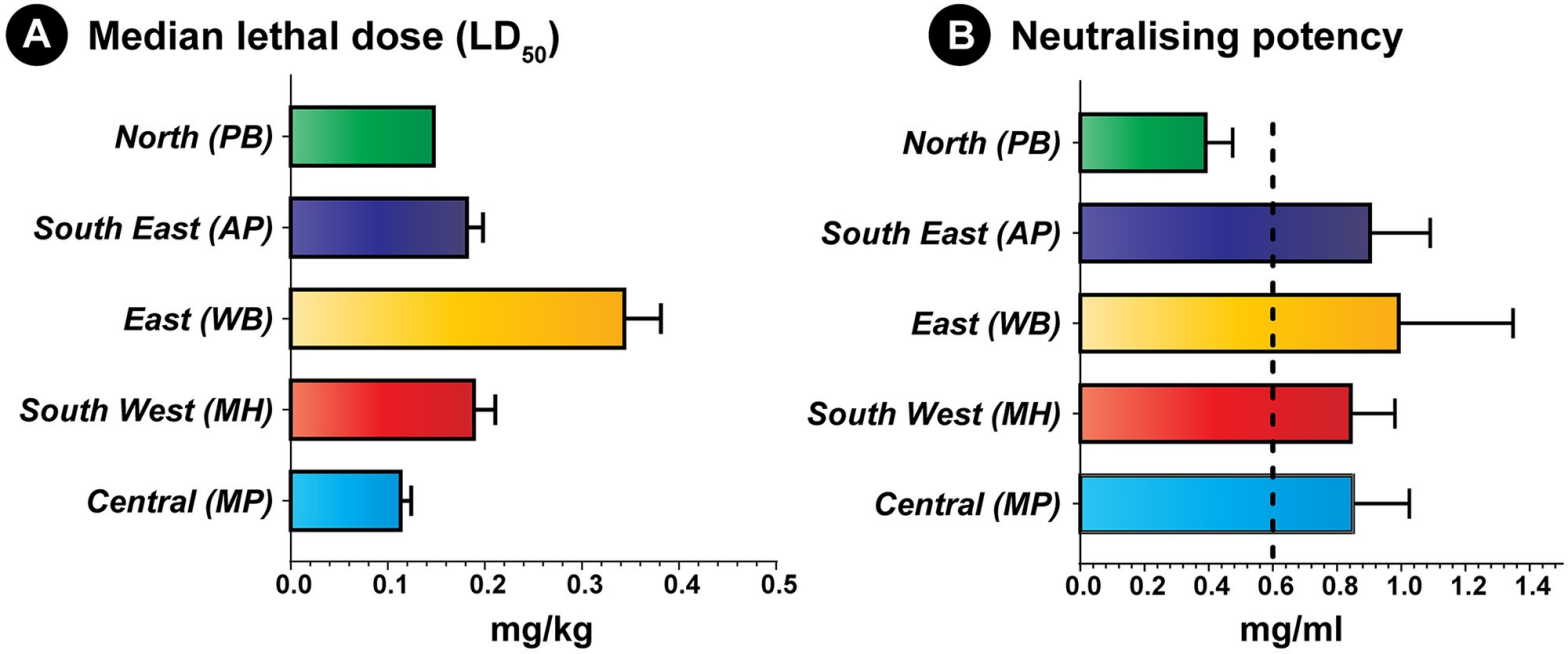
Venom potencies of the pan-Indian populations of *D*. *russelii* and the neutralisation potencies of the Premium Serums antivenom. This figure depicts **(A)** venom potencies (mg/kg) of various *D*. *russelii* populations and **(B)** the neutralising potencies (mg/ml) of the Premium Serums antivenom against them. The error bars show 95% confidence intervals, and the marketed neutralising potency (0.60 mg/ml) of the commercial product against *D*. *russelii* venom is shown as a vertical dotted line (panel B).

The proteomic composition and potency of venoms is significantly influenced by the ecology and environment of the snake species [4–7,70]. Evaluation of murine intravenous toxicity profiles revealed interesting differences between the pan-Indian populations of *D*. *russelii venoms*. While the Deccan Plateau (MP; 0.11 mg/kg) and the semi-arid (PB; 0.14 mg/kg) populations were highly potent, the Gangetic Plain (WB; 0.34 mg/kg) population was found to be considerably less potent, while the coastal (AP; 0.18 mg/kg) and Western Ghats (MH; 0.19 mg/kg) populations exhibited near equivalent, intermediary, potencies (Fig 6A and S5 Table).

### Antivenom efficacy via median effective dose (ED_50_) assay

Though the *in vitro* recognition and inhibition of biochemical/pharmacological activities by commercial antivenoms has been demonstrated against venoms of *D*. *russelii* from certain populations in India [9–13], their ability to neutralise the lethal effects in animal models is yet to be robustly evaluated. Considering its increased venom recognition capabilities, the Premium Serums antivenom was selected for use in the assessment of *in vivo* neutralisation against the pan-Indian venoms of *D*. *russelii*. Perhaps surprisingly, given the extent of venom compositional and functional variation observed in this study, the venom neutralising potencies for all populations of *D*. *russelii* (0.84–0.99 mg/ml) met the manufacturer’s marketed claim (0.6 mg/ml), with the exception of observations of poor neutralisation of the semi-arid (PB) population (0.39 mg/ml) (Fig 6B and S6 Table). These findings contrast with those recently observed with venom sourced from Indian *Naja* populations, where venom variation resulted in a dramatic lack of preclinical antivenom efficacy [71].

## Discussions

### Biogeographic variation in Russell’s viper venom

Biotic and abiotic factors are well-known to dictate snake venom compositions and potencies [5]. Unfortunately, the influence of diverse biogeographic conditions on the composition and potency of the medically most important Indian snakes, and the consequence of this variation on snakebite treatment, remains elusive. Here, evaluation of proteomic profiles of the pan-Indian populations of *D*. *russelii* from distinct biogeographic zones revealed considerable compositional differences in venom toxins, including PLA_2_, Kunitz, SVSP and snaclec. As variation in venom proteomes can significantly alter biochemical and pharmacological activities of snake venoms, we also subjected *D*. *russelii* venoms to a variety of biochemical assessments, including PLA_2_, LAAO, DNase, fibrinogenolytic, haemolytic and blood coagulation assays. The outcomes of these experiments highlighted significant differences in the activities of *D*. *russelii* venoms from distinct biogeographical zones. Moreover, they are suggestive of the differential abilities of these populations in inflicting cytotoxic, haemotoxic and procoagulant effects in human snakebite victims [65,72,73]. Such stark differences in composition and activities may be underpinned by a variety of factors across distinct agro-climatic conditions, including temperature, humidity, altitude, phylogenetic divergence and prey and predator abundance. Differences in toxin composition were also found to correlate with altered potencies of *Daboia* venoms, where the protease-rich Deccan Plateau (MP) and Kunitz-rich Gangetic Plain (WB) populations of *D*. *russelii* were the most and the least toxic populations, respectively (Figs 3 and 6A and S2–S4 Tables).

### Inadequacy of antivenom therapy in North Indian snakebite hotspots

**Fig 7:**
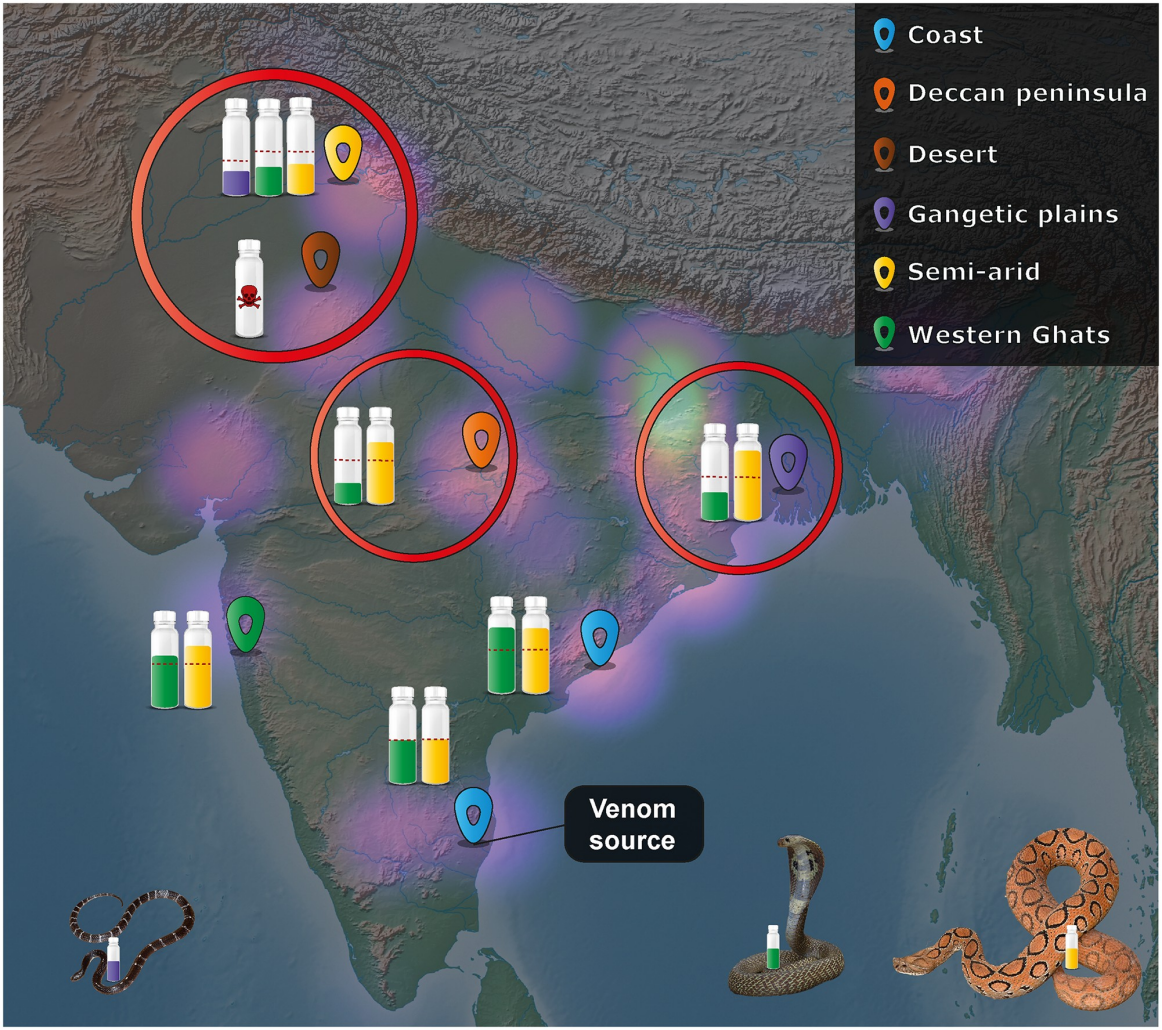
The preclinical inefficacy of Indian antivenom therapy in snakebite hotspots. This figure depicts the alarming preclinical ineffectiveness of commercial antivenoms in the major snakebite hotspots of India (highlighted in red circles). The relative differences in neutralisation potencies of antivenoms against the geographically distinct populations of *N*. *naja* (green vials) [71], *D*. *russelii* (yellow vials) and *B*. *caeruleus* (purple vial) [20] are shown in comparison to the venom source (for antivenom production) population in southern India. The red dotted lines on vials represent the marketed neutralising potency of the commercial products. Sampling locations have been indicated with uniquely coloured markers (top right box) on the biogeographical map of India that was prepared with QGIS 3.8.2 [35]. The intensity of purple clouds on the map is indicative of the estimated standardised snakebite death rates per million reported by Suraweera *et al*. 2020 [2], with the brighter regions representing the major hotspots.

Despite being the only curative therapeutic for snakebite in India, commercial polyvalent antivenoms that are marketed by several manufacturers across the country suffer from several critical limitations. Perhaps, their major potential limitation is a lack of effectiveness against geographically disparate snake populations, as antivenoms are customarily manufactured using venoms sourced from the ‘big four’ snakes in the southeastern part of the country [74]. Venom recognition experiments in this study revealed that the majority of marketed products lacked antibodies specific to several high-, mid- and low-molecular-weight toxins (S6A Fig), which was in line with previous findings [9,11,13,20]. Preclinical experiments conducted in a murine model of envenoming were undertaken to evaluate the neutralising potential of the Premium Serums antivenom, which exhibited superior *in vitro* venom recognition potential over its competitors. Though this antivenom met the marketed neutralising potency (0.60 mg/ml) for four out of the five *D*. *russelii* populations sampled across the various biogeographical zones in India (0.84 to 0.99 mg/ml), its potency against the semi-arid (PB) population was significantly lower (0.39 mg/ml). Similar concerning inefficacy of this antivenom has been previously reported against two of India’s other ‘big four’ snake species (*B*. *caeruleus* and *N*. *naja*) in this region [20,71], while a complete lack of neutralisation (challenge dose: 5X LD_50_) has also been recently reported against the Desert (Rajasthan) population of *N*. *naja* [71]. Improved neutralisation potential of the other commercial antivenoms (i.e., VINS, Bharat Serums and Haffkine) seems unlikely, as these products largely rely on the same venom source for immunisation and follow very similar immunisation protocols. This assertion is further supported by the outcomes of our *in vitro* binding experiments, which revealed comparably worse immunological binding of these antivenoms to venoms. Similarly, deficiencies in the neutralisation potency of Indian antivenoms (VINS) towards *D*. *russelii* from Pakistan has also been documented previously [75]. These disturbing findings, perhaps, explain the alarming rates of snakebite mortalities in the northern and (north)western regions of the country (Fig 7; [2]), further highlighting the pressing need to develop a pan-India effective antivenom therapy [20].

### Compositional variation in venoms cannot predict the underlying clinical outcomes

Intraspecific differences in venom composition, resulting from multiple biotic and abiotic factors, has been well-documented in snakes [63,70,76]. As a local adaptation to the changing ecology and environment, species that are characterised by large geographical distribution exhibit stark variations in the proteomic composition and toxicity. For instance, the venoms sourced from the pan-Indian populations of *N*. *naja*, yet another medically important Indian snake species with a near-countrywide distribution, has been shown to exhibit substantial differences in the abundances of lethal neurotoxins and cytotoxins [71,77–79]. These compositional differences led to reduced preclinical effectiveness of the marketed antivenoms in mitigating snakebite pathologies [71]. In complete contrast, despite the observed geographic variability in venom composition and toxicity profiles, we find that the lethal effects induced by venoms of the majority of investigated *D*. *russelii* populations are neutralised by the Premium Serums polyvalent antivenom, matching the marketed claim of effectiveness (S6 Table). Thus, the considerable variation observed in the venom proteomes of both *N*. *naja* and *D*. *russelii*, surprisingly, mostly translate into treatment challenges only for the former species. These contrasting findings highlight that intraspecific venom variation in itself cannot be a predictor for the (pre)clinical effectiveness of antivenoms. It should be noted, however, that the results presented here do not shed light on the effectiveness of antivenoms in mitigating the local, morbidity-causing effects associated with *D*. *russelii* envenomings, and this would be a valuable research line to pursue in the future.

In summary, comparative proteomics, biochemical and pharmacological assessments, and toxicity profiling experiments performed in this study reveal significant intraspecific variation in *D*. *russelii* venoms from five distinct biogeographic regions of India. The results of *in vitro* immunological assays identified the Premium Serums antivenom to be superior to its competitors (VINS, Bharat Serums and Haffkine) in terms of its venom recognition potential. Despite the considerable differences in venom proteomic profiles revealed, the Premium Serums antivenom exhibited surprisingly similar efficacy in countering the lethal effects of venom from four out of the five *D*. *russelii* populations in a mouse model of envenoming. However, this antivenom was found to be inefficacious in neutralising the lethal effects of the North Indian semi-arid (PB) population, a caveat previously also highlighted for two other ‘big four’ snakes [20,71]. These inadequacies of existing antivenom further highlight the compelling need to develop pan-India effective antivenoms to safeguard human lives in high snakebite burdened locales of India.

### Limitations of the study

Russell’s viper is amongst the most medically important Indian snakes, as it accounts for over 40% of snakebite fatalities in the country [2]. Surprisingly, though the polyvalent antivenom neutralised the lethality of *D*. *russelii* venoms from four out of five biogeographic zones investigated in this study, these conclusions were derived with respect to the neutralisation potency advertised by the antivenom manufacturers. However, since Indian antivenoms have not been evaluated through robust human clinical trials, it is essential to validate the accuracy of this cut off for effective treatment of *D*. *russelii* envenomings. Moreover, the neutralisation experiments in the mouse model employed here do not inform us of the ability of this commercial product in countering the morbid symptoms that incapacitate hundreds of thousands of Indians annually. Therefore, future work is warranted to evaluate the abilities of antivenoms to neutralise local pathologies (e.g., necrosis and haemorrhage) that result in immutable morbidities. Moreover, certain populations of *D*. *russelii* from Sri Lanka have been shown to secrete a form of basic PLA_2_ (U1-viperitoxin-Dr1a) that makes their venoms highly neurotoxic [73]. While their counterparts in South India have also been proposed to exhibit such neurotoxic effects [64,80], robust clinical evidence has been lacking. In our *in vivo* toxicity experiments, we did not observe any neurotoxic symptoms (e.g., ptosis, paralysis of the limbs, etc.) in mice injected with the venoms of the pan-Indian populations of *Daboia* snakes. Nonetheless, it will be important in the future to conduct neurotoxicity assays in mammalian model systems to delineate the abilities of the pan-Indian populations of *D*. *russelii* in inflicting neurotoxic symptoms. Furthermore, systematic documentation of epidemiological data and clinical evaluation of antivenom’s effectiveness is indispensable for assessing the shortcomings of the conventional antivenom therapy, particularly in regions that suffer the brunt of snakebite. Finally, assessing the avidity of venom-antivenom interactions and characterisation of unrecognised medically important toxins could further provide valuable information for improving the efficacy of conventional antivenoms.

## Supporting information

S1 Fig Biochemical venom variation in the pan-Indian populations of *D*. *russelii*. (DOCX)

S2 Fig DNase activities of *D*. *russelii* venoms demonstrated through agarose gel electrophoresis. (DOCX)

S3 Fig Fibrinogenolytic activities of *D*. *russelii* venoms from distinct biogeographical locations across India. (DOCX)

S4 Fig Plasma clotting times of *D*. *russelii* venoms and neutralisation by commercial antivenom. (DOCX)

S5 Fig IgG reactivity of commercial Indian antivenoms against *D*. *russelii* venoms determined by indirect ELISA. (DOCX)

S6 Fig Immunoblotting of commercial Indian antivenoms against *D*. *russelii* venoms. (DOCX)

S1 Table Details of commercial Indian antivenoms. (DOCX)

S2 Table The proteomic composition of the Gangetic Plain (WB) population. (DOCX)

S3 Table The proteomic composition of the Western Ghats (MH) population. (DOCX)

S4 Table The proteomic composition of the Deccan Plateau (MP) population. (DOCX)

S5 Table Toxicity profiles of the pan-Indian *D*. *russelii* populations. (DOCX)

S6 Table Median effective doses and neutralisation potencies of Premium Serums antivenom against the pan-Indian *D*. *russelii* venoms. (DOCX)

S1 Data Archive containing the results of proteomics analyses. (ZIP)
