# Supplementary material for: Biogeographic venom variation in Russell’s viper (Daboia russelii) and the preclinical inefficacy of antivenom therapy in snakebite hotspots
Source: PLoS Negl Trop Dis. 2021 Mar 25;15(3):e0009247. doi: 10.1371/journal.pntd.0009247 (PMC7993602; doi:10.1371/journal.pntd.0009247)
Supplement: S1 Fig — (DOCX) [file pntd.0009247.s001.docx]

**S1 Fig.** Biochemical venom variation in the pan-Indian populations of *D. russelii*.


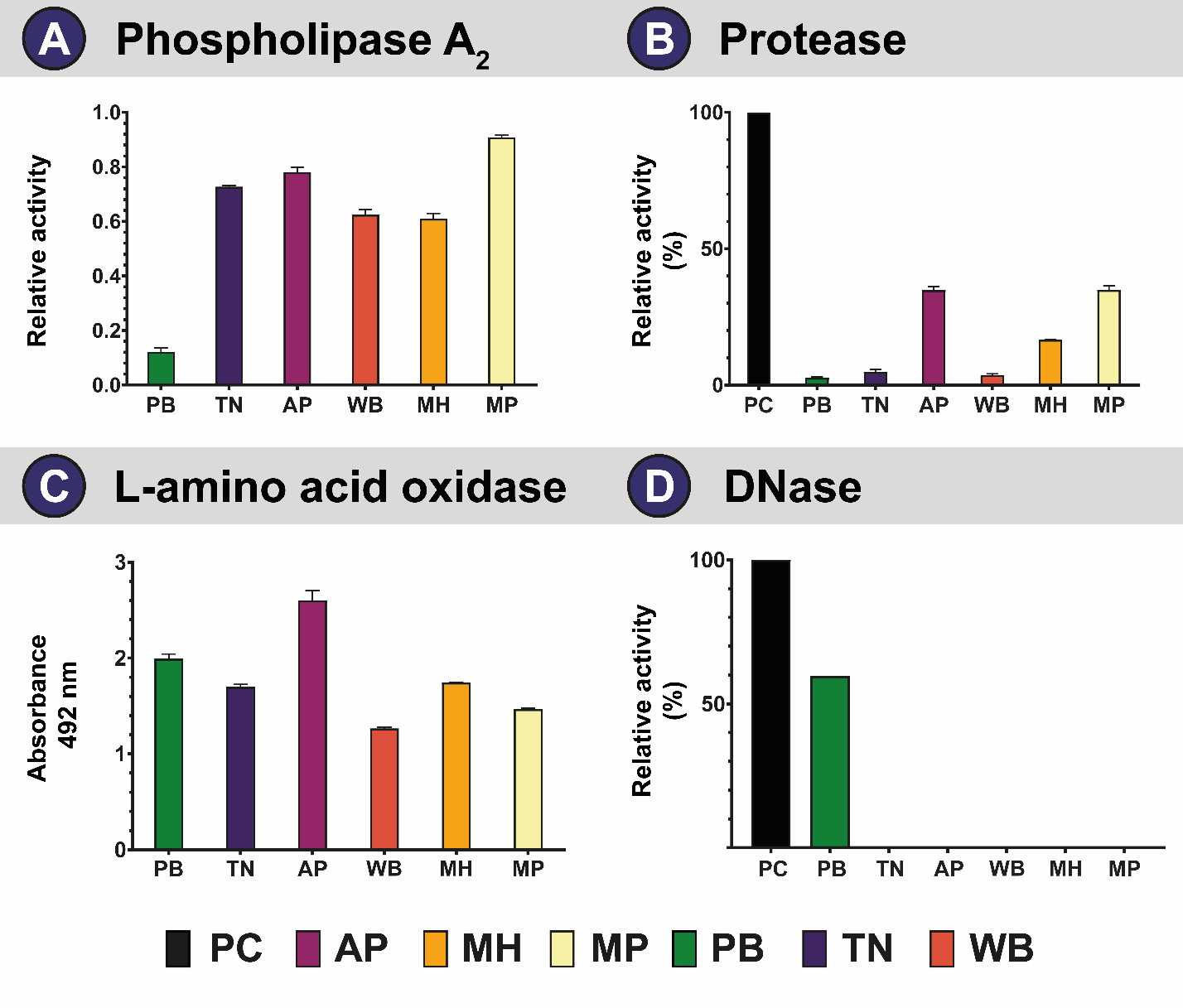


Venoms from the pan-Indian *D. russelii* populations were assayed for **(A)** phospholipase A_2_, **(B)** protease, **(C)** L-amino acid oxidase, and **(D)** DNase activities. All assays (except DNase) were conducted in triplicates and the error bars indicate the standard deviation. DNase activities were quantified by densitometric analyses using ImageJ software ([https://imagej.nih.gov/ij](https://imagej.nih.gov/ij/)). **PC:** Positive Control; **PB:** Punjab (semi-arid); **TN:** Tamil Nadu (coastal); **AP:** Andhra Pradesh (coastal); **WB**: West Bengal (Gangetic Plains); **MH:** Maharashtra (Western Ghats); **MP:** Madhya Pradesh (Deccan Plateau)
