# Supplementary material for: Biogeographic venom variation in Russell’s viper (Daboia russelii) and the preclinical inefficacy of antivenom therapy in snakebite hotspots
Source: PLoS Negl Trop Dis. 2021 Mar 25;15(3):e0009247. doi: 10.1371/journal.pntd.0009247 (PMC7993602; doi:10.1371/journal.pntd.0009247)
Supplement: S2 Fig — (DOCX) [file pntd.0009247.s002.docx]

**S2 Fig.** DNase activities of *D. russelii* venoms demonstrated through agarose gel electrophoresis.
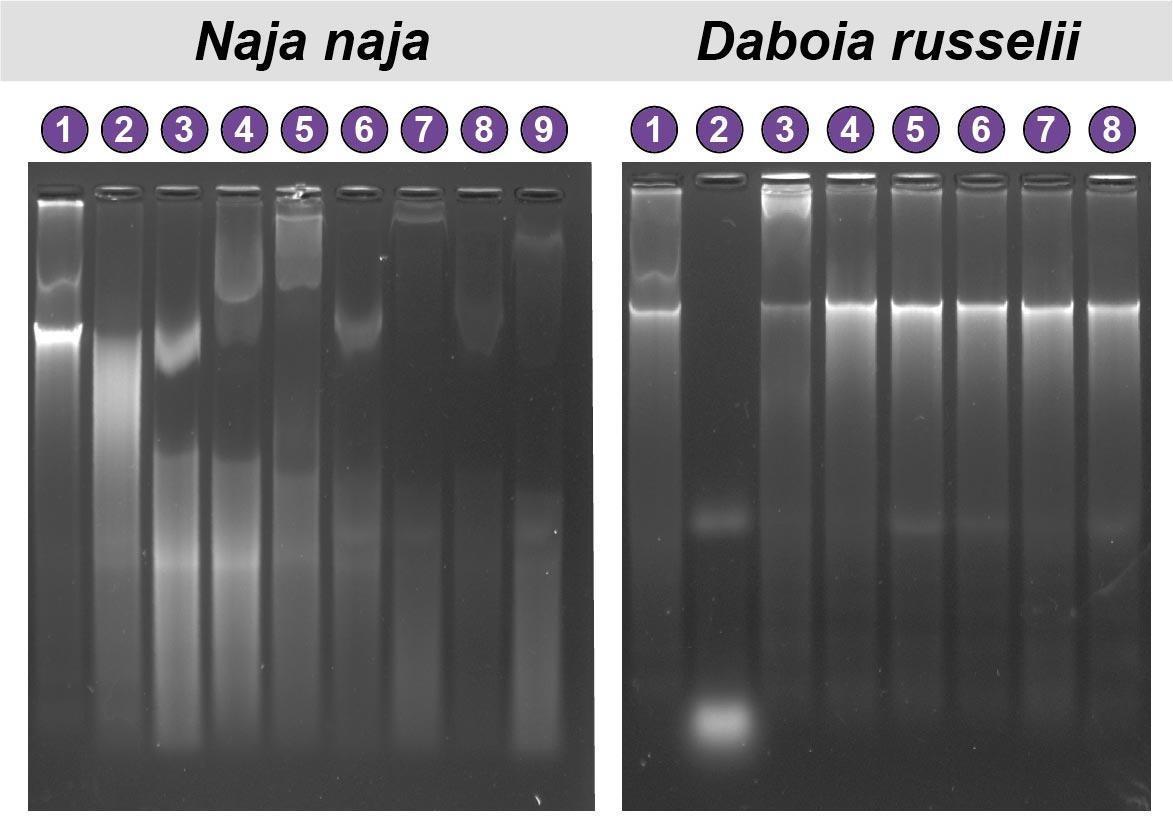


Agarose gel displaying DNase activities of geographically distinct *D. russelii* venoms. **1**: DNA only (negative control); **2**: DNA + 15 U DNase (positive control); **3**: Punjab (semi-arid); **4**: Tamil Nadu (coastal); **5**: Andhra Pradesh (coastal); **6**: West Bengal (Gangetic Plains); **7**: Maharashtra (Western Ghats); and **8**: Madhya Pradesh (Deccan Plateau).
