# Supplementary material for: Biogeographic venom variation in Russell’s viper (Daboia russelii) and the preclinical inefficacy of antivenom therapy in snakebite hotspots
Source: PLoS Negl Trop Dis. 2021 Mar 25;15(3):e0009247. doi: 10.1371/journal.pntd.0009247 (PMC7993602; doi:10.1371/journal.pntd.0009247)
Supplement: S3 Fig — (DOCX) [file pntd.0009247.s003.docx]

**S3 Fig.** Fibrinogenolytic activities of *D. russelii* venoms from distinct biogeographical locations across India.
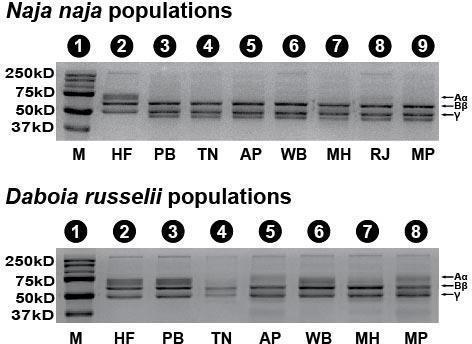


Fibrinogen degrading abilities of *D. russelii* venoms are depicted here. **M**: Pre-stained protein marker; **HF**: human fibrinogen; **PB**: Punjab (semi-arid); **TN**: Tamil Nadu (coastal); **AP**: Andhra Pradesh (coastal); **WB**: West Bengal (Gangetic Plains), **MH**: Maharashtra (Western Ghats); and **MP**: Madhya Pradesh (Deccan Plateau).
