# Supplementary material for: Biogeographic venom variation in Russell’s viper (Daboia russelii) and the preclinical inefficacy of antivenom therapy in snakebite hotspots
Source: PLoS Negl Trop Dis. 2021 Mar 25;15(3):e0009247. doi: 10.1371/journal.pntd.0009247 (PMC7993602; doi:10.1371/journal.pntd.0009247)
Supplement: S4 Fig — (DOCX) [file pntd.0009247.s004.docx]

**S4 Fig.** Plasma clotting times of *D. russelii* venoms and neutralisation by commercial antivenom.


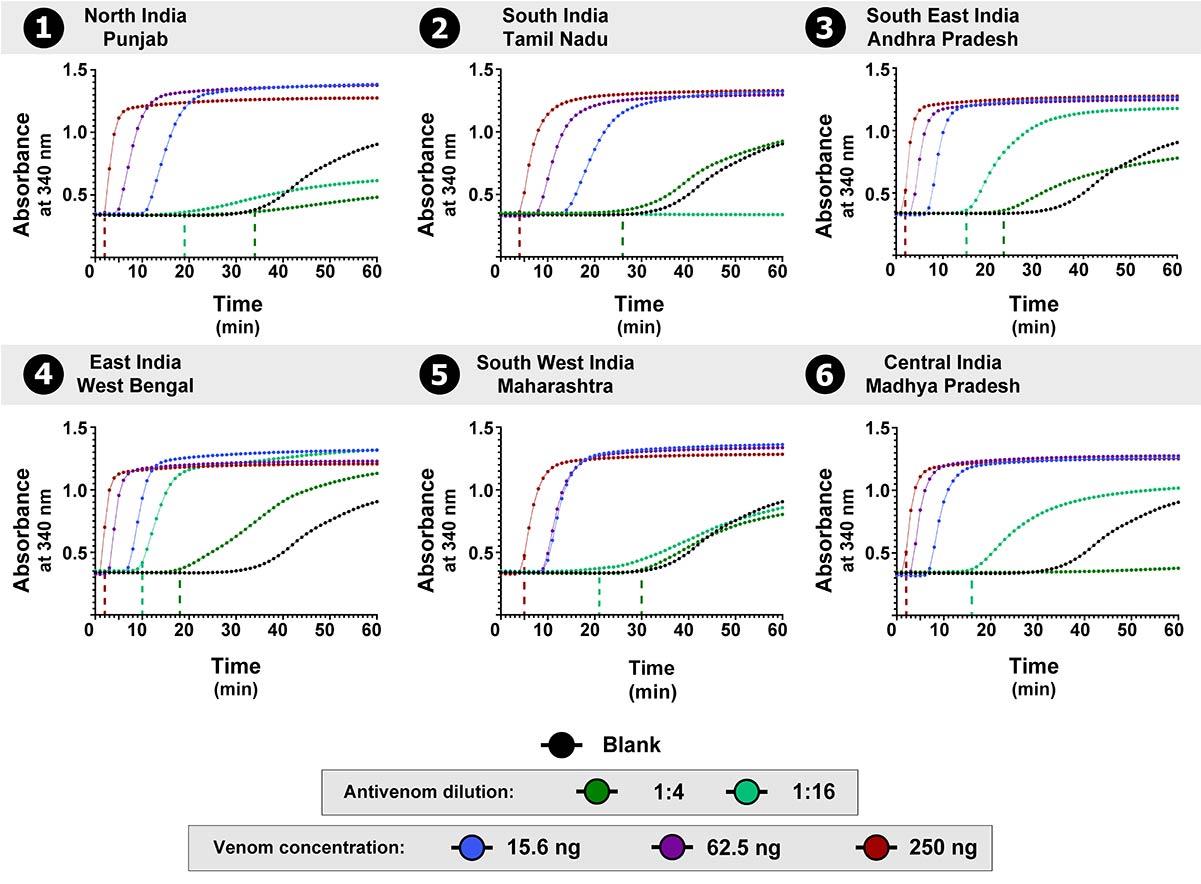


The plots depict the change in the turbidity of plasma over time (min), which indicates the formation of fibrin clots. Line graphs, representing the clotting activities of various venom concentrations (15.6 to 250 ng/ml) and the neutralising effect of Premium Serums antivenom (1:4 dilution of 1 mg/ml or 0.25 µg/µl and 1:16 of 1 mg/ml or 0.0625 µg/µl), are uniquely colour coded. The vertical dotted lines matching these colour codes indicate the clotting time and the reduction in clotting time after neutralisation.
