# Supplementary material for: Biogeographic venom variation in Russell’s viper (Daboia russelii) and the preclinical inefficacy of antivenom therapy in snakebite hotspots
Source: PLoS Negl Trop Dis. 2021 Mar 25;15(3):e0009247. doi: 10.1371/journal.pntd.0009247 (PMC7993602; doi:10.1371/journal.pntd.0009247)
Supplement: S5 Fig — (DOCX) [file pntd.0009247.s005.docx]

**S5 Fig.** IgG reactivity of commercial Indian antivenoms against *D. russelii* venoms determined by indirect ELISA.


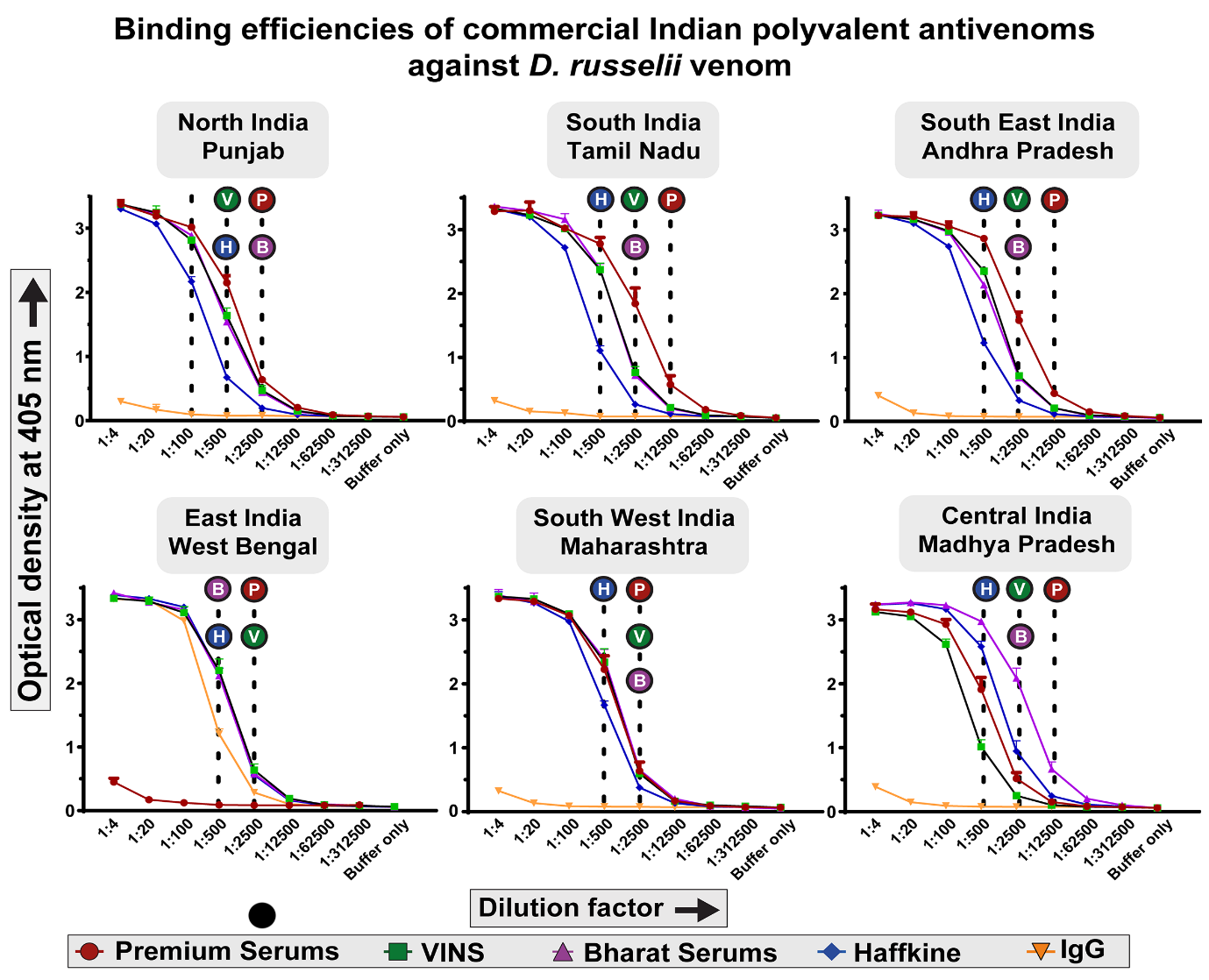


The line graphs represent the binding efficiencies of the commercial Indian antivenoms against the pan-Indian populations of *D. russelii*, estimated using indirect ELISA experiments for various dilutions of antivenoms (1:4 to 1:312,500). The absorbance values were recorded at 405nm to measure the venom recognition potential of antivenoms and plotted against the respective dilution of antivenom. Error bars denote the standard deviation for all assays performed in triplicates. The naive horse IgG (at 1:4 dilution) was used to determine the antivenom titres, which have been represented as dotted lines. The alphabets indicate the titres of antivenoms: **P**: Premium Serums & Vaccines Pvt. Ltd.; **V**: VINS Bioproducts Ltd.; **B**: Bharat Serums and Vaccines Ltd; and **H**: Haffkine BioPharmaceutical Corporation Ltd.
