# Supplementary material for: Biogeographic venom variation in Russell’s viper (Daboia russelii) and the preclinical inefficacy of antivenom therapy in snakebite hotspots
Source: PLoS Negl Trop Dis. 2021 Mar 25;15(3):e0009247. doi: 10.1371/journal.pntd.0009247 (PMC7993602; doi:10.1371/journal.pntd.0009247)
Supplement: S6 Fig — (DOCX) [file pntd.0009247.s006.docx]

**S6A Fig.** Immunoblotting of commercial Indian antivenoms against *D. russelii* venoms.

*
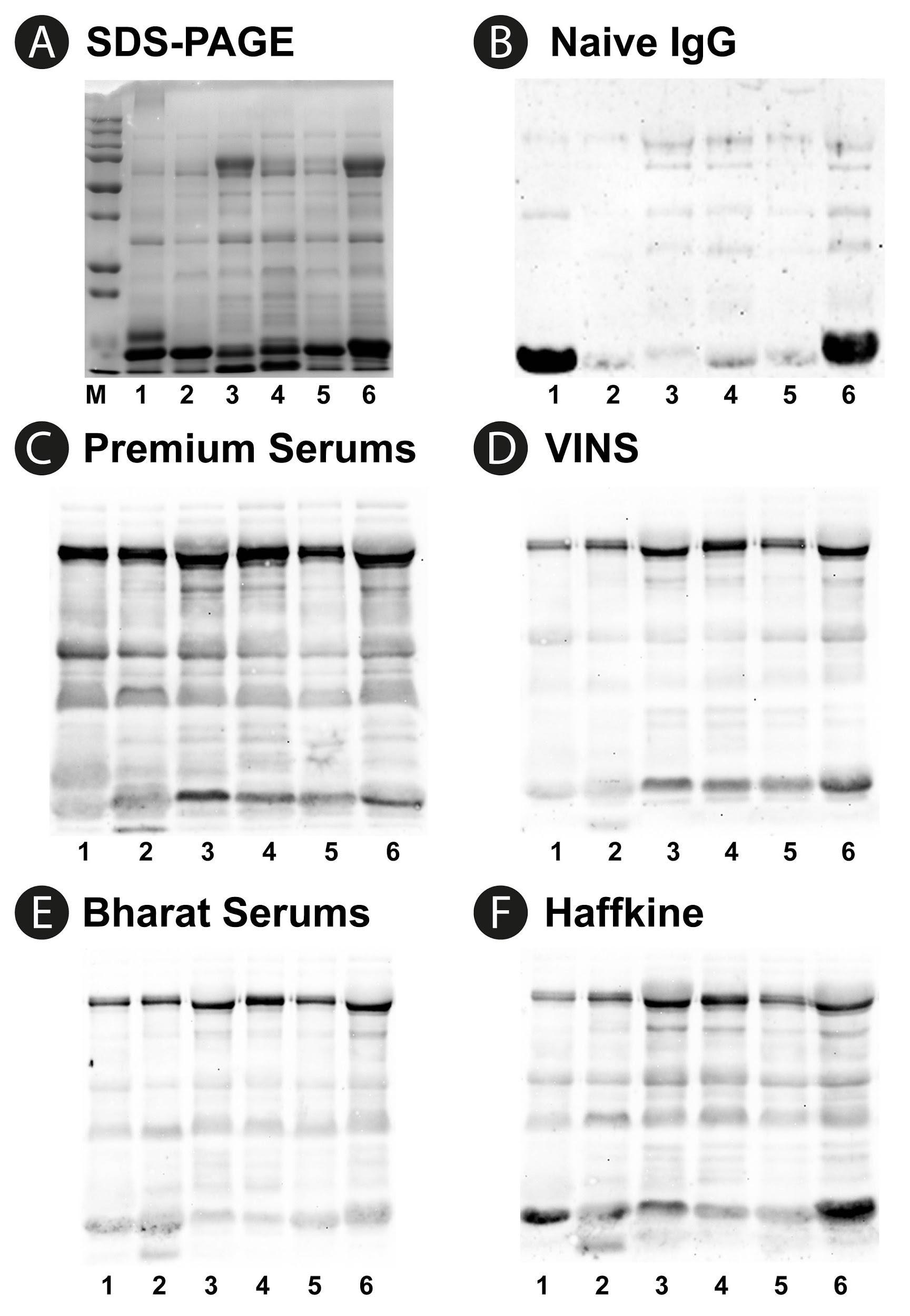
*

This figure highlights the immunological cross-reactivities of commercial Indian antivenoms against geographically disparate *D. russelii* venoms as western blots. **M**: Marker; **1**: Punjab (semi-arid); **2**: Tamil Nadu (coastal); **3**: Andhra Pradesh (coastal); **4**: West Bengal (Gangetic Plains); **5**: Maharashtra (Western Ghats); and **6**: Madhya Pradesh (Deccan Plateau).

**S6B Fig.** Heatmap depicting immunorecognition potential of commercial Indian antivenoms against *D. russelii*.

*
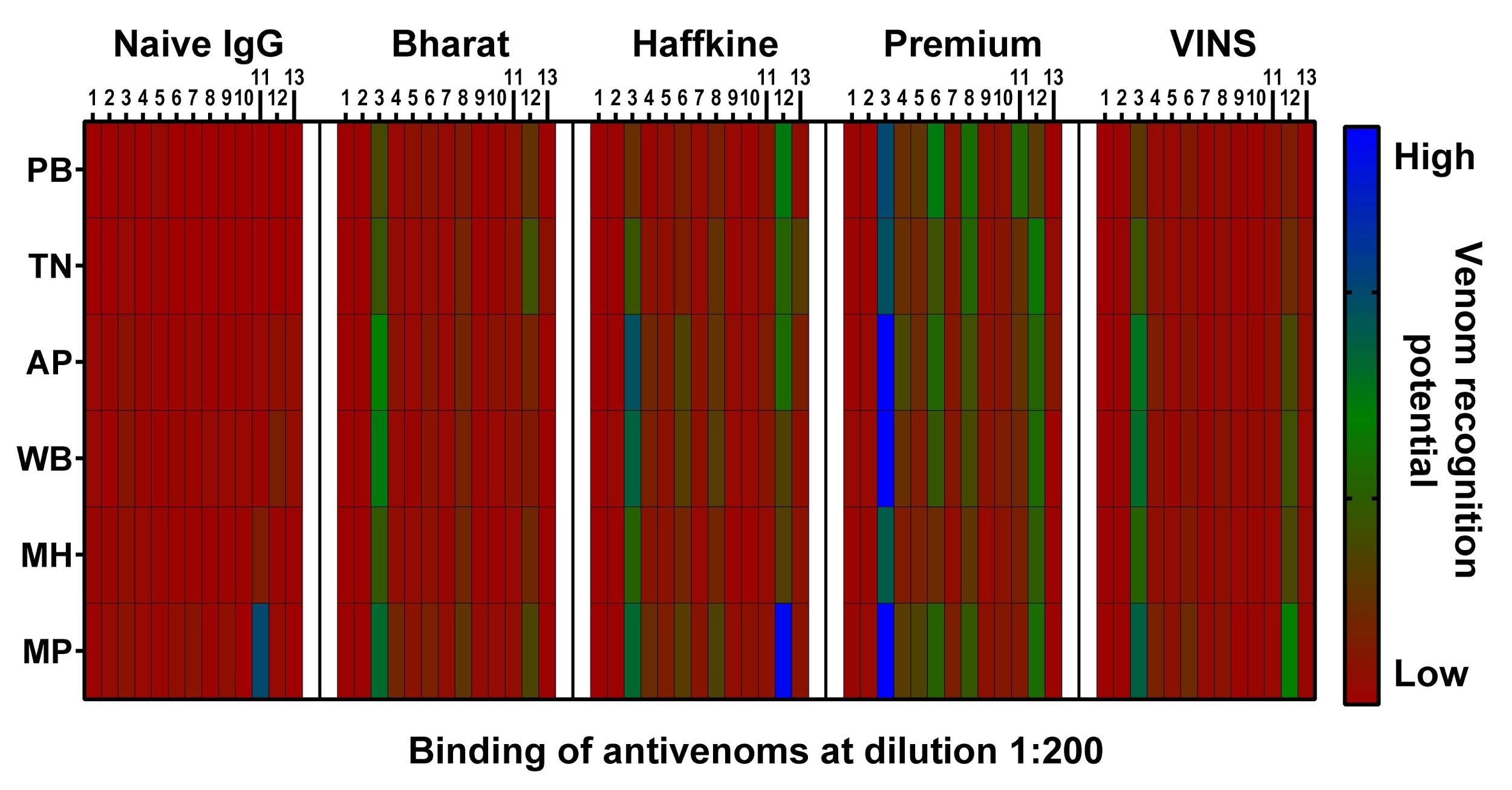
*

The cross-recognition capabilities of commercial Indian antivenoms and naive horse IgGs against pan-Indian populations of *D. russelii* venoms are depicted here. This heatmap, with a gradient of red (low binding) to blue (high binding), was generated using the values determined for individual immunoblot bands (1 to 13) by densitometric analysis using ImageJ software ([https://imagej.nih.gov/ij](https://imagej.nih.gov/ij/)). Populations: **PB:** Punjab (semi-arid); **TN:** Tamil Nadu (coastal); **AP:** Andhra Pradesh (coastal); **WB**: West Bengal (Gangetic Plains); **MH:** Maharashtra (Western Ghats); **MP:** Madhya Pradesh (Deccan Plateau).
