# Supplementary material for: Biogeographic venom variation in Russell’s viper (Daboia russelii) and the preclinical inefficacy of antivenom therapy in snakebite hotspots
Source: PLoS Negl Trop Dis. 2021 Mar 25;15(3):e0009247. doi: 10.1371/journal.pntd.0009247 (PMC7993602; doi:10.1371/journal.pntd.0009247)
Supplement: S1 Table — (DOCX) [file pntd.0009247.s007.docx]

**S1 Table.** Details of commercial Indian antivenoms.

| **Manufacturer** | **Batch** | **Manufacture**  **(M) and**  **expiry**  **(E) dates** | **Protein**  **content**  (mg/ml) | **Marketed neutralizing efficacy**  (mg/ml) |
| --- | --- | --- | --- | --- |
| **Bharat Serums and Vaccines Ltd.** | A05318087 | **M:** 10/2018  **E:** 09/2022 | 26.5 ± 0.77 | *N. naja*: 0.60  *B. caeruleus*: 0.45  *D. russelii*: 0.60  *E. carinatus*: 0.45 |
| **Haffkine BioPharmaceutical** **Corporation Ltd.** | AS180611 | **M:** 06/2018  **E:** 11/2022 | 24.7 ± 0.5 |  |
| **Premium Serums**  **& Vaccines Pvt.**  **Ltd.** | ASVS(I)-Lyo013 | **M:** 11/2018  **E:** 11/2022 | 26.2 ± 1.2 |  |
| **VINS Bioproducts** **Ltd.** | 01AS18067 | **M:** 11/2018  **E:** 10/2022 | 31.4 ± 0.54 |  |

The table outlines the commercial Indian antivenoms investigated in this study with corresponding batch numbers, manufacturing and expiration dates, protein concentrations of the vials as estimated by Bradford method, as well as the marketed neutralisation potencies against the ‘big four’ snakes.
