# Supplementary material for: Biogeographic venom variation in Russell’s viper (Daboia russelii) and the preclinical inefficacy of antivenom therapy in snakebite hotspots
Source: PLoS Negl Trop Dis. 2021 Mar 25;15(3):e0009247. doi: 10.1371/journal.pntd.0009247 (PMC7993602; doi:10.1371/journal.pntd.0009247)
Supplement: S2 Table — (DOCX) [file pntd.0009247.s008.docx]

**S2, S3, and S4 Tables:** Proteomic compositions of *D. russelii* venoms from various populations across India.

Peaks Studio X Plus was used to examine raw MS/MS spectra against Uniprot’s SwissProt database ([www.uniprot.com](http://www.uniprot.com)) for the identification of various toxin families present in the venom. The tables illustrates the key statistics of these searches, including the accession number, species name, -10lgP values, number of high confidence peptides, unique peptides, percent abundance of each toxin hit, average molecular mass (kDa), the toxin family of the matching Uniprot entry and the number of HPLC fraction in which the toxin was identified.

**S3 Table.** The proteomic composition of the Western Ghats (MH) population.

| **Sr.no.** | **Accession** | **Species** | **-10lgP** | **#Peptides** | **#Unique** | **Relative abundance of toxin hit (%)** | **Avg. Mass (kDa)** | **Toxin type** | **Fraction no.** |
| --- | --- | --- | --- | --- | --- | --- | --- | --- | --- |
| **Phospholipase A2 (PLA_2_): 59.05%** | | | | | | | | | |
| 1 | A8CG90 | *Daboia russelii* | 399.83 | 32 | 23 | 18.5634 | 15.46 | Basic PLA2 | 5,7A,7B,8 |
| 2 | A8CG86 | *Daboia russelii* | 366.02 | 28 | 12 | 12.3859 | 15.33 | Acidic PLA2 | 5,7A,7B,8,9 |
| 3 | P81458 | *Daboia russelii* | 355.02 | 22 | 13 | 11.4396 | 13.63 | Basic PLA2 | 5,7A,7B,8 |
| 4 | A8CG87 | *Daboia russelii* | 360.12 | 23 | 8 | 6.3695 | 15.59 | Acidic PLA2 | 1,7A,7B,8 |
| 5 | A8CG78 | *Daboia siamensis* | 360.12 | 23 | 8 | 6.3695 | 15.59 | Acidic PLA2 | 1,7A,7B,8 |
| 6 | A8CG89 | *Daboia russelii* | 338.21 | 16 | 1 | 2.7537 | 15.86 | Basic PLA2 | 1,7B,8 |
| 7 | P86368 | *Daboia russelii* | 309.36 | 20 | 6 | 0.7142 | 13.69 | Basic PLA2 | 1,2,3,4,8 |
| 8 | Q910A1 | *Vipera ammodytes* | 275.31 | 13 | 3 | 0.4356 | 15.44 | Acidic PLA2 | 7A,7B |
| 9 | C0HJL8 | *Bothriechis nigroviridis* | 72.02 | 1 | 1 | 0.0077 | 14.13 | PLA2 | 9 |
| 10 | Q02471 | *Daboia siamensis* | 257.78 | 7 | 1 | 0.0065 | 15.56 | Basic PLA2 | 7B,8 |
| 11 | O42191 | *Gloydius halys* | 141.74 | 1 | 1 | 0.0013 | 13.93 | Acidic PLA2 | 9 |
| **Snake venom serine protease (SVSP): 27.12%** | | | | | | | | | |
| 12 | P18964 | *Daboia siamensis* | 373.28 | 30 | 14 | 12.3614 | 26.18 | Factor V activator | 1,7B,8 |
| 13 | E5L0E4 | *Daboia siamensis* | 279.24 | 13 | 7 | 11.3034 | 28.04 | β-fibrinogenase | 1,7A,7B,8 |
| 14 | P18965 | *Daboia siamensis* | 367.57 | 25 | 9 | 1.5284 | 28.82 | Factor V activator | 7B,8 |
| 15 | E0Y419 | *Macrovipera lebetina* | 229.88 | 7 | 2 | 0.6183 | 28.30 | β-fibrinogenase | 1,5,7A,7B,8,9 |
| 16 | Q9PT40 | *Macrovipera lebetina* | 277.55 | 13 | 1 | 0.4552 | 28.89 | SVSP | 7B,8 |
| 17 | E0Y418 | *Macrovipera lebetina* | 199.43 | 5 | 3 | 0.2754 | 28.70 | SVSP | 9 |
| 18 | E0Y420 | *Macrovipera lebetina* | 199.18 | 5 | 1 | 0.2318 | 28.35 | SVSP | 7B,8 |
| 19 | E5L0E3 | *Daboia siamensis* | 183.46 | 6 | 4 | 0.1323 | 28.50 | α-fibrinogenase | 8,9 |
| 20 | P0DPS3 | *Vipera ammodytes* | 285.38 | 12 | 1 | 0.1182 | 22.64 | SVSP | 7A,7B |
| 21 | Q7T229 | *Bothrops jararacussu* | 190.72 | 5 | 2 | 0.0512 | 28.65 | SVSP | 8,9 |
| 22 | A0A1I9 | *Vipera ammodytes* | 289.14 | 13 | 1 | 0.0414 | 28.93 | SVSP | 8 |
| **Cysteine-rich secretory proteins (CRISP): 6.47%** | | | | | | | | | |
| 23 | B7FDI0 | *Vipera berus nikolskii* | 298.66 | 13 | 8 | 4.9331 | 24.61 | CRISP | 1,7A,7B |
| 24 | Q7ZZN9 | *Protobothrops jerdonii* | 235.44 | 7 | 1 | 1.5321 | 26.87 | CRISP | 2,7B,8 |
| **Kunitz-type serine protease inhibitor (Kunitz): 3.71%** | | | | | | | | | |
| 25 | A8Y7N6 | *Daboia siamensis* | 247.84 | 9 | 6 | 0.9388 | 9.44 | Kunitz | 2,3,4 |
| 26 | A8Y7N7 | *Daboia siamensis* | 247.84 | 9 | 6 | 0.9388 | 10.16 | Kunitz | 2,3,4 |
| 27 | Q2ES47 | *Daboia russelii* | 223.89 | 8 | 5 | 0.6676 | 9.15 | Kunitz | 2,3 |
| 28 | Q2ES49 | *Daboia russelii* | 116.91 | 2 | 2 | 0.5046 | 9.68 | Kunitz | 4 |
| 29 | A8Y7N8 | *Daboia siamensis* | 197.48 | 5 | 1 | 0.4274 | 10.01 | Kunitz | 1,10 |
| 30 | A8Y7P1 | *Daboia siamensis* | 198.31 | 5 | 2 | 0.1058 | 9.32 | Kunitz | 2,3 |
| 31 | A8Y7P5 | *Daboia siamensis* | 198.31 | 5 | 2 | 0.1058 | 9.90 | Kunitz | 2,3 |
| 32 | P24541 | *Eristicophis macmahoni* | 133.89 | 2 | 1 | 0.0005 | 6.77 | Kunitz | 3 |
| 33 | Q2ES50 | *Daboia russelii* | 171.66 | 4 | 1 | 0.0004 | 9.29 | Kunitz | 7A |
| 34 | A8Y7P0 | *Daboia siamensis* | 171.66 | 4 | 1 | 0.0004 | 9.29 | Kunitz | 7A |
| **Snaclec: 2.06%** | | | | | | | | | |
| 35 | Q38L02 | *Daboia siamensis* | 276.56 | 11 | 11 | 1.3814 | 17.51 | Snaclec | 1,7A,7B,8,9 |
| 36 | Q4PRC6 | *Daboia siamensis* | 199.9 | 5 | 4 | 0.4109 | 18.07 | Snaclec | 8,9 |
| 37 | Q696W1 | *Macrovipera lebetina* | 130.6 | 3 | 3 | 0.0905 | 18.09 | Snaclec | 3,8,9 |
| 38 | B4XSY8 | *Macrovipera lebetina* | 108.64 | 2 | 2 | 0.0575 | 15.31 | Snaclec | 8,9 |
| 39 | B4XSY7 | *Macrovipera lebetina* | 108.64 | 2 | 2 | 0.0575 | 17.72 | Snaclec | 8,9 |
| 40 | Q4PRD1 | *Daboia siamensis* | 116.79 | 1 | 1 | 0.0300 | 16.87 | Snaclec | 8 |
| 41 | Q56EB1 | *Bothrops jararaca* | 59.8 | 1 | 1 | 0.0108 | 17.58 | Snaclec | 7B,9 |
| 42 | B4XSY5 | *Macrovipera lebetina* | 91.08 | 1 | 1 | 0.0048 | 17.73 | Snaclec | 9 |
| 43 | B4XSY6 | *Macrovipera lebetina* | 91.08 | 1 | 1 | 0.0048 | 17.74 | Snaclec | 9 |
| 44 | W5XCJ6 | *Macrovipera lebetina* | 92.61 | 1 | 1 | 0.0040 | 17.55 | Snaclec | 9 |
| 45 | B4XSZ0 | *Macrovipera lebetina* | 92.61 | 1 | 1 | 0.0040 | 17.76 | Snaclec | 9 |
| 46 | B4XSY9 | *Macrovipera lebetina* | 92.61 | 1 | 1 | 0.0040 | 17.71 | Snaclec | 9 |
| **Snake venom metalloproteinase (SVMP): 1.29%** | | | | | | | | | |
| 47 | B8K1W0 | *Daboia russelii* | 356.05 | 32 | 26 | 1.2453 | 69.56 | SVMP | 3,10 |
| 48 | Q90495 | *Echis carinatus* | 141.05 | 3 | 1 | 0.0355 | 69.46 | SVMP | 2 |
| 49 | Q7LZ61 | *Daboia siamensis* | 249.25 | 8 | 2 | 0.0073 | 69.65 | Coagulation factor X-activating | 9 |
| 50 | Q4VM07 | *Macrovipera lebetina* | 210.67 | 5 | 1 | 0.0055 | 68.84 | SVMP | 10 |
| 51 | Q8JIR2 | *Protobothrops flavoviridis* | 119.48 | 3 | 1 | 0.0008 | 68.77 | SVMP | 10 |
| **Neurotoxic three-finger toxins (N-3FTx): 0.21%** | | | | | | | | | |
| 52 | P01391 | *Naja kaouthia* | 179.69 | 4 | 2 | 0.1740 | 7.83 | Type II (long)  α-neurotoxin | 2,3,4 |
| 53 | P25669 | *Naja naja* | 160.92 | 3 | 1 | 0.0189 | 7.82 | Type II (long)  α-neurotoxin | 2,3 |
| 54 | P25668 | *Naja naja* | 156.93 | 3 | 1 | 0.0129 | 7.85 | Type II (long)  α-neurotoxin | 3 |
| **L-amino-acid oxidase (LAAO): 0.04%** | | | | | | | | | |
| 55 | G8XQX1 | *Daboia russelii* | 227.31 | 9 | 9 | 0.0369 | 56.89 | LAAO | 8,9,10 |
| **Cytotoxic three-finger toxins (C-3FTx): 0.03%** | | | | | | | | | |
| 56 | P86382 | *Naja naja* | 73.18 | 1 | 1 | 0.0103 | 6.79 | Cytotoxin | 9 |
| 57 | P01445 | *Naja kaouthia* | 73.18 | 1 | 1 | 0.0103 | 6.75 | Cytotoxin | 9 |
| 58 | P86540 | *Naja naja* | 73.18 | 1 | 1 | 0.0103 | 6.79 | Cytotoxin | 9 |
| **Nerve growth factor (NGF): 0.02%** | | | | | | | | | |
| 59 | P25428 | *Macrovipera lebetina* | 124.88 | 3 | 2 | 0.0148 | 27.32 | NGF | 2,4 |
| 60 | P30894 | *Daboia russelii* | 110.35 | 2 | 1 | 0.0027 | 13.28 | NGF | 5,7B |
| **Disintegrin: 0.02%** | | | | | | | | | |
| 61 | Q7ZZM2 | *Protobothrops jerdonii* | 58.03 | 1 | 1 | 0.0110 | 11.85 | Disintegrin | 2 |
| 62 | Q3BK14 | *Macrovipera lebetina* | 58.03 | 1 | 1 | 0.0110 | 11.50 | Disintegrin | 2 |
| **Vascular endothelial growth factor (VEGF): 0.01%** | | | | | | | | | |
| 63 | P0DL42 | *Daboia siamensis* | 112.43 | 1 | 1 | 0.0033 | 12.55 | VEGF | 7B |
| 64 | P67861 | *Daboia russelii* | 112.43 | 1 | 1 | 0.0033 | 16.28 | VEGF | 7B |
| **5’-nucleotidase: 0.01%** | | | | | | | | | |
| 65 | B6EWW8 | *Gloydius brevicaudus* | 134.05 | 2 | 2 | 0.0032 | 64.43 | 5'-nucleotidase | 2,3 |
| 66 | F8S0Z7 | *Crotalus adamanteus* | 134.05 | 2 | 2 | 0.0032 | 64.68 | 5'-nucleotidase | 2,3 |
