# Supplementary material for: Biogeographic venom variation in Russell’s viper (Daboia russelii) and the preclinical inefficacy of antivenom therapy in snakebite hotspots
Source: PLoS Negl Trop Dis. 2021 Mar 25;15(3):e0009247. doi: 10.1371/journal.pntd.0009247 (PMC7993602; doi:10.1371/journal.pntd.0009247)
Supplement: S3 Table — (DOCX) [file pntd.0009247.s009.docx]

**S2, S3, and S4 Tables:** Proteomic compositions of *D. russelii* venoms from various populations across India.

Peaks Studio X Plus was used to examine raw MS/MS spectra against Uniprot’s SwissProt database ([www.uniprot.com](http://www.uniprot.com)) for the identification of various toxin families present in the venom. The tables illustrates the key statistics of these searches, including the accession number, species name, -10lgP values, number of high confidence peptides, unique peptides, percent abundance of each toxin hit, average molecular mass (kDa), the toxin family of the matching Uniprot entry and the number of HPLC fraction in which the toxin was identified.

**S4 Table.** The proteomic composition of the Deccan Plateau (MP) population.

| **Sr.no.** | **Accession** | **Species** | **-10lgP** | **#Peptides** | **#Unique** | **Relative abundance of toxin hit (%)** | **Avg. Mass (kDa)** | **Toxin type** | **Fraction no.** |
| --- | --- | --- | --- | --- | --- | --- | --- | --- | --- |
| **Phospholipase A2 (PLA_2_): 44.94%** | | | | | | | | | |
| 1 | A8CG87 | *Daboia russelii* | 293.14 | 18 | 5 | 13.1426 | 15.59 | Acidic PLA2 | 6 |
| 2 | A8CG78 | *Daboia siamensis* | 293.14 | 18 | 5 | 13.1426 | 15.59 | Acidic PLA2 | 6 |
| 3 | A8CG86 | *Daboia russelii* | 375.82 | 32 | 15 | 10.2376 | 15.33 | Acidic PLA2 | 7A,7B,8A,8B |
| 4 | A8CG90 | *Daboia russelii* | 252.73 | 10 | 5 | 6.2332 | 15.46 | Basic PLA2 | 5,6,7A,7B,8A |
| 5 | P31100 | *Daboia siamensis* | 320.83 | 20 | 5 | 1.0868 | 15.42 | Acidic PLA2 | 7B,9 |
| 6 | F8QN54 | *Vipera renardi* | 304.1 | 14 | 1 | 0.3371 | 15.64 | Basic PLA2 | 8A |
| 7 | P86368 | *Daboia russelii* | 411.84 | 40 | 1 | 0.3276 | 13.69 | Basic PLA2 | 7A |
| 8 | P84674 | *Daboia russelii* | 378.15 | 29 | 2 | 0.1507 | 13.59 | Basic PLA2 | 8A |
| 9 | P59071 | *Daboia russelii* | 405.81 | 40 | 1 | 0.1330 | 13.61 | Basic PLA2 | 7A |
| 10 | P81458 | *Daboia russelii* | 202.35 | 7 | 1 | 0.0613 | 13.63 | Basic PLA2 | 6 |
| 11 | P15445 | *Naja naja* | 197 | 4 | 4 | 0.0500 | 13.35 | Acidic PLA2 | 5,6 |
| 12 | A8CG82 | *Daboia siamensis* | 288.77 | 10 | 1 | 0.0340 | 15.84 | Basic PLA2 | 7A |
| **Snaclec: 19.57%** | | | | | | | | | |
| 13 | Q38L02 | *Daboia siamensis* | 295.45 | 14 | 14 | 5.5820 | 17.51 | Snaclec | 6,7A,7B,8A, 8B, 9,10 |
| 14 | B4XSZ0 | *Macrovipera lebetina* | 201.48 | 5 | 5 | 2.6763 | 17.76 | Snaclec | 7B,8A,8B,9,  10 |
| 15 | B4XSY9 | *Macrovipera lebetina* | 201.48 | 5 | 5 | 2.6763 | 17.71 | Snaclec | 7B,8A,8B,9,  10 |
| 16 | W5XCJ6 | *Macrovipera lebetina* | 201.48 | 5 | 5 | 2.6763 | 17.55 | Snaclec | 7B,8A,8B,9,  10 |
| 17 | Q4PRC6 | *Daboia siamensis* | 237.09 | 10 | 8 | 2.3537 | 18.07 | Snaclec | 7A,7B,8A,8B,  9,10 |
| 18 | Q4PRD1 | *Daboia siamensis* | 213.18 | 6 | 1 | 1.5072 | 16.87 | Snaclec | 7A,7B,8A,8B |
| 19 | Q696W1 | *Macrovipera lebetina* | 137.86 | 3 | 3 | 0.7329 | 18.09 | Snaclec | 7B,8A,8B,9 |
| 20 | B4XSY8 | *Macrovipera lebetina* | 148.2 | 3 | 3 | 0.4346 | 15.31 | Snaclec | 7B,8A,8B,9,  10 |
| 21 | B4XSY7 | *Macrovipera lebetina* | 148.2 | 3 | 3 | 0.4346 | 17.72 | Snaclec | 7B,8A,8B,9,  10 |
| 22 | Q56EB1 | *Bothrops jararaca* | 95.72 | 2 | 2 | 0.4323 | 17.58 | Snaclec | 6,7B,8B |
| 23 | Q4PRD2 | *Daboia siamensis* | 172.63 | 3 | 3 | 0.0427 | 18.34 | Snaclec | 7B,8B |
| 24 | B4XSY5 | *Macrovipera lebetina* | 104.73 | 2 | 2 | 0.0103 | 17.73 | Snaclec | 8B |
| 25 | B4XSY6 | *Macrovipera lebetina* | 104.73 | 2 | 2 | 0.0103 | 17.74 | Snaclec | 8B |
| 26 | P0DJL4 | *Daboia palaestinae* | 63.79 | 1 | 1 | 0.0033 | 12.13 | Snaclec | 7B |
| **Snake venom serine protease (SVSP): 12.73%** | | | | | | | | | |
| 27 | E0Y418 | *Macrovipera lebetina* | 207.81 | 9 | 8 | 7.3197 | 28.70 | SVSP | 7B,8A,8B |
| 28 | E5L0E4 | *Daboia siamensis* | 181.93 | 5 | 2 | 2.0602 | 28.04 | β-fibrinogenase | 6,7A,7B |
| 29 | E5L0E3 | *Daboia siamensis* | 262.8 | 10 | 9 | 1.7367 | 28.50 | α-fibrinogenase | 7B |
| 30 | P18964 | *Daboia siamensis* | 277.99 | 17 | 7 | 1.1965 | 26.18 | Factor V activator | 6,7A |
| 31 | P18965 | *Daboia siamensis* | 278.58 | 11 | 1 | 0.2568 | 28.82 | Factor V activator | 7A |
| 32 | P0DPS3 | *Vipera ammodytes* | 248.93 | 6 | 1 | 0.0496 | 22.64 | SVSP | 7A |
| 33 | E0Y419 | *Macrovipera lebetina* | 149.8 | 4 | 1 | 0.0397 | 28.30 | β-fibrinogenase | 6,7B |
| 34 | A0A1I9 | *Vipera ammodytes* | 249.61 | 6 | 1 | 0.0372 | 28.93 | SVSP | 7A |
| 35 | E0Y420 | *Macrovipera lebetina* | 117.23 | 3 | 1 | 0.0311 | 28.35 | SVSP | 6,7B |
| 36 | P86530 | *Daboia russelii* | 126.96 | 3 | 1 | 0.0032 | 1.73 | SVSP | 7B |
| **Vascular endothelial growth factor (VEGF): 7.58%** | | | | | | | | | |
| 37 | P0DL42 | *Daboia siamensis* | 262 | 14 | 13 | 3.7915 | 12.55 | VEGF | 5,6,7A |
| 38 | P67861 | *Daboia russelii* | 262 | 14 | 13 | 3.7915 | 16.28 | VEGF | 5,6,7A |
| **Kunitz-type serine protease inhibitor (Kunitz): 6.57%** | | | | | | | | | |
| 39 | Q2ES47 | *Daboia russelii* | 239.56 | 10 | 5 | 5.6130 | 9.15 | Kunitz | 1,2,3,5 |
| 40 | A8Y7N6 | *Daboia siamensis* | 120.63 | 3 | 1 | 0.2738 | 9.44 | Kunitz | 1,2,3 |
| 41 | A8Y7N7 | *Daboia siamensis* | 120.63 | 3 | 1 | 0.2738 | 10.16 | Kunitz | 1,2,3 |
| 42 | A8Y7N8 | *Daboia siamensis* | 172.91 | 4 | 1 | 0.1346 | 10.01 | Kunitz | 1 |
| 43 | A8Y7P1 | *Daboia siamensis* | 128.96 | 3 | 1 | 0.0600 | 9.32 | Kunitz | 1 |
| 44 | A8Y7P5 | *Daboia siamensis* | 128.96 | 3 | 1 | 0.0600 | 9.90 | Kunitz | 1 |
| 45 | H6VC06 | *Daboia russelii* | 177.41 | 5 | 1 | 0.0586 | 9.39 | Kunitz | 1 |
| 46 | A8Y7P4 | *Daboia siamensis* | 177.41 | 5 | 1 | 0.0586 | 9.37 | Kunitz | 1 |
| 47 | P24541 | *Eristicophis macmahoni* | 151.79 | 3 | 1 | 0.0276 | 6.77 | Kunitz | 1,2,3 |
| 48 | Q2ES50 | *Daboia russelii* | 201.08 | 6 | 1 | 0.0024 | 9.29 | Kunitz | 2,3,5 |
| 49 | A8Y7P0 | *Daboia siamensis* | 201.08 | 6 | 1 | 0.0024 | 9.29 | Kunitz | 2,3,5 |
| 50 | P00990 | *Daboia siamensis* | 201.08 | 6 | 1 | 0.0024 | 6.85 | Kunitz | 2,3,5 |
| 51 | A8Y7N9 | *Daboia siamensis* | 150.86 | 5 | 1 | 0.0004 | 10.35 | Kunitz | 2,3 |
| **Snake venom metalloproteinase (SVMP): 6.51%** | | | | | | | | | |
| 52 | B8K1W0 | *Daboia russelii* | 384.35 | 43 | 39 | 5.9421 | 69.56 | SVMP | 2,3,10 |
| 53 | Q7LZ61 | *Daboia siamensis* | 293.37 | 18 | 11 | 0.4653 | 69.65 | Coagulation factor X-activating | 8B,9 |
| 54 | Q4VM07 | *Macrovipera lebetina* | 169.04 | 3 | 1 | 0.0994 | 68.84 | SVMP | 10 |
| **L-amino-acid oxidase (LAAO): 0.72%** | | | | | | | | | |
| 55 | G8XQX1 | *Daboia russelii* | 364.11 | 31 | 6 | 0.7242 | 56.89 | LAAO | 7B,8A,8B,9,  10 |
| **5’-nucleotidase: 0.56%** | | | | | | | | | |
| 56 | B6EWW8 | *Gloydius brevicaudus* | 273.51 | 16 | 14 | 0.5588 | 64.43 | 5'-nucleotidase | 5,8A,9,10 |
| **Neurotoxic three-finger toxins (N-3FTx): 0.47%** | | | | | | | | | |
| 57 | P01391 | *Naja kaouthia* | 132.22 | 2 | 1 | 0.1971 | 7.83 | Type II (long)  α-neurotoxin | 1,2,3,5,6 |
| 58 | P25669 | *Naja naja* | 138.25 | 3 | 2 | 0.1791 | 7.82 | Type II (long)  α-neurotoxin | 1,2,3,5 |
| 59 | P59275 | *Naja kaouthia* | 55.4 | 1 | 1 | 0.0297 | 6.94 | Type I (short)  α-neurotoxin | 1 |
| 60 | P01427 | *Naja oxiana* | 55.4 | 1 | 1 | 0.0297 | 6.89 | Type I (short)  α-neurotoxin | 1 |
| 61 | P59276 | *Naja kaouthia* | 55.4 | 1 | 1 | 0.0297 | 6.86 | Type I (short)  α-neurotoxin | 1 |
| **Nerve growth factor (NGF): 0.18%** | | | | | | | | | |
| 62 | P30894 | *Daboia russelii* | 240.93 | 11 | 4 | 0.1393 | 13.28 | NGF | 5,6,7B |
| 63 | P25428 | *Macrovipera lebetina* | 205.95 | 9 | 2 | 0.0389 | 27.32 | NGF | 1 |
| **Cysteine-rich secretory proteins (CRISP): 0.16%** | | | | | | | | | |
| 64 | B7FDI0 | *Vipera nikolskii* | 186.5 | 5 | 2 | 0.0788 | 24.61 | CRISP | 6,7A |
| 65 | B7FDI1 | *Vipera berus* | 186.5 | 5 | 2 | 0.0788 | 26.51 | CRISP | 6,7A |
| **C-type lectin (CTL): 0.02%** | | | | | | | | | |
| 66 | Q6T7B7 | *Bitis gabonica* | 70.63 | 1 | 1 | 0.0186 | 18.63 | C-type lectin | 7B |
