# Supplementary material for: Biogeographic venom variation in Russell’s viper (Daboia russelii) and the preclinical inefficacy of antivenom therapy in snakebite hotspots
Source: PLoS Negl Trop Dis. 2021 Mar 25;15(3):e0009247. doi: 10.1371/journal.pntd.0009247 (PMC7993602; doi:10.1371/journal.pntd.0009247)
Supplement: S5 Table — (DOCX) [file pntd.0009247.s011.docx]

**S5 Table.** Toxicity profiles of the pan-Indian *D. russelii* populations.

| ***D. russelii* population** | **Venom Dose** (µg) | | | | | **Number of survivors** | | | | | **LD_50_** (µg/mouse) | **LD_50_**  (mg/kg) |
| --- | --- | --- | --- | --- | --- | --- | --- | --- | --- | --- | --- | --- |
| North India  Punjab | 3.27 | 4.08 | 5.12 | 6.4 | 8 | 2 | 1 | 0 | 0 | 0 | 2.97  2.46-3.58 | 0.14  0.12-0.17 |
| Southeast India  Andhra Pradesh | 3.27 | 4.08 | 5.12 | 6.4 | 8 | 4 | 1 | 0 | 0 | 0 | 3.65  3.37-3.95 | 0.18  0.16-0.19 |
| East India  West Bengal | 5.24 | 6.55 | 8.19 | 10.24 | 12.8 | 5 | 3 | 1 | 0 | 0 | 6.90  6.23-7.62 | 0.34  0.31-0.38 |
| Southwest India  Maharashtra | 3.2 | 4 | 5 | 6.25 | 7.81 | 4 | 2 | 0 | 0 | 0 | 3.80  3.42-4.21 | 0.19  0.17-0.21 |
| Central India  Madhya Pradesh | 2.04 | 2.56 | 3.2 | 4 | 5 | 4 | 1 | 0 | 0 | 0 | 2.29  2.11-2.48 | 0.11  0.10-0.12 |

This table presents the median lethal doses and the associated survivorship data to depict venom toxicity profiles of geographically disparate *D. russelii* populations.
