# Supplementary material for: Biogeographic venom variation in Russell’s viper (Daboia russelii) and the preclinical inefficacy of antivenom therapy in snakebite hotspots
Source: PLoS Negl Trop Dis. 2021 Mar 25;15(3):e0009247. doi: 10.1371/journal.pntd.0009247 (PMC7993602; doi:10.1371/journal.pntd.0009247)
Supplement: S6 Table — (DOCX) [file pntd.0009247.s012.docx]

**S6 Table.** Median effective doses and neutralisation potencies of Premium Serums antivenom against the pan-Indian *D. russelii* venoms*.*

| ***D. russelii* population** | **Amount of antivenom injected in venom-antivenom mixture** (µl) | | | | **ED_50_** (µl) | **ED_50_**  (µl antivenom/ mg venom) | **Potency of antivenom** (mg/ml) |
| --- | --- | --- | --- | --- | --- | --- | --- |
| North India  Punjab | 49.40 | 32.93 | 21.96 | 14.64 | 29.99  24.99-36.00 | 2019.53  1682.83-2424.24 | 0.396**^*^** |
| Southeast India  Andhra Pradesh | 32.94 | 21.97 | 14.64 | 9.76 | 16.07  13.39-19.30 | 880.55  733.70-1057.53 | 0.908 |
| East India  West Bengal | 73.96 | 49.40 | 32.93 | 21.97 | 27.66  20.47-37.37 | 801.74  593.33-1083.19 | 0.998 |
| Southwest India  Maharashtra | 32.94 | 21.97 | 14.64 | 9.76 | 17.93  15.52-20.71 | 943.68  816.84-1090.00 | 0.848 |
| Central India  Madhya Pradesh | 21.97 | 14.64 | 9.76 | 6.51 | 10.71  8.92-12.86 | 935.37  779.04-1123.14 | 0.855 |

The potencies of Premium Serums antivenom in neutralising the lethal effects of *D. russelii* venoms across pan-Indian populations are presented here. The estimated potency of the antivenom was found to meet the marketed potency (0.60 mg/ml), except for population marked with an ‘asterisk’ (*****), wherein the antivenom exhibited very poor neutralisation in the murine model.
