# Supplementary material for: Biogeographic venom variation in Russell’s viper (Daboia russelii) and the preclinical inefficacy of antivenom therapy in snakebite hotspots
Source: PLoS Negl Trop Dis. 2021 Mar 25;15(3):e0009247. doi: 10.1371/journal.pntd.0009247 (PMC7993602; doi:10.1371/journal.pntd.0009247)
Supplement: S1 Data — (ZIP) [file pntd.0009247.s013.zip › D. russelii_Maharashtra/D. russelii_Maharashtra.html]

proteins


Summary

  

# 1. Notes

# 2. Result Statistics

**Figure 1.**
False discovery rate (FDR) curve. X axis is the number of peptide-spectrum matches (PSM) being kept. Y axis is the corresponding FDR.

  


  

**Figure 2.**
PSM score distribution. **(a)**
Distribution of PEAKS peptide score; **(b)**
Scatterplot of PEAKS peptide score versus precursor mass error.

|  |  |  |  |
| --- | --- | --- | --- |
| **(a)**  |  | | **(b)**  |  | |

|  |  |  |  |  |  |  |  |  |  |  |  |  |  |  |  |  |  |  |  |  |  |  |  |  |  |  |  |  |  |  |  |  |  |  |  |  |  |  |  |  |  |  |  |  |  |  |  |  |  |  |  |  |  |  |  |  |  |  |  |  |  |  |  |  |  |  |  |  |  |  |  |  |  |  |  |  |  |  |  |  |  |  |  |  |  |  |  |  |  |  |  |  |  |  |  |  |  |  |  |  |  |  |  |  |  |  |  |  |  |  |  |  |  |  |  |  |  |  |  |  |  |  |  |  |  |  |  |  |  |  |  |  |  |  |  |  |  |  |  |  |  |  |  |  |  |  |  |  |  |  |  |  |
| --- | --- | --- | --- | --- | --- | --- | --- | --- | --- | --- | --- | --- | --- | --- | --- | --- | --- | --- | --- | --- | --- | --- | --- | --- | --- | --- | --- | --- | --- | --- | --- | --- | --- | --- | --- | --- | --- | --- | --- | --- | --- | --- | --- | --- | --- | --- | --- | --- | --- | --- | --- | --- | --- | --- | --- | --- | --- | --- | --- | --- | --- | --- | --- | --- | --- | --- | --- | --- | --- | --- | --- | --- | --- | --- | --- | --- | --- | --- | --- | --- | --- | --- | --- | --- | --- | --- | --- | --- | --- | --- | --- | --- | --- | --- | --- | --- | --- | --- | --- | --- | --- | --- | --- | --- | --- | --- | --- | --- | --- | --- | --- | --- | --- | --- | --- | --- | --- | --- | --- | --- | --- | --- | --- | --- | --- | --- | --- | --- | --- | --- | --- | --- | --- | --- | --- | --- | --- | --- | --- | --- | --- | --- | --- | --- | --- | --- | --- | --- | --- | --- | --- | --- |
| **Table 1.** Statistics of data.    |  |  |  |  |  |  |  |  |  |  |  |  | | --- | --- | --- | --- | --- | --- | --- | --- | --- | --- | --- | --- | |  | #Scans | | #Features | Identified | | | #Peptides | #Sequences | #Proteins\* | | | | MS1 | MS2 | #PSMs | #Scans | #Features | Groups | All | Top | | Total | 164380 | 134957 | 418651 | 1561 | 1552 | 18692 | 536 | 522 | 71 | 199 | 87 | | F1 | 16784 | 7977 | 27822 | 171 | 170 | 1587 | 144 | 141 | 37 | 78 | 42 | | F10 | 16805 | 8614 | 29111 | 127 | 127 | 1275 | 97 | 94 | 19 | 58 | 21 | | F2 | 16976 | 5207 | 19811 | 60 | 60 | 794 | 56 | 56 | 30 | 68 | 37 | | F3 | 16845 | 8165 | 22441 | 89 | 89 | 1355 | 79 | 79 | 23 | 56 | 28 | | F4 | 16823 | 7489 | 26452 | 106 | 106 | 1260 | 96 | 96 | 26 | 64 | 29 | | F5 | 16633 | 10053 | 26487 | 222 | 220 | 1380 | 83 | 81 | 18 | 43 | 19 | | F7A | 16266 | 16431 | 48762 | 168 | 164 | 2091 | 82 | 80 | 30 | 51 | 32 | | F7B | 15546 | 26186 | 70986 | 260 | 259 | 3289 | 173 | 166 | 37 | 70 | 39 | | F8 | 16381 | 15562 | 47712 | 150 | 150 | 1906 | 120 | 118 | 33 | 49 | 35 | | F9 | 15321 | 29273 | 99067 | 208 | 207 | 3755 | 150 | 148 | 40 | 100 | 46 |  \* proteins with significant peptides are used in counts. |

**Figure 3.**
Sample overlap for Proteins and Peptides (up to 8 samples). **(a)**
All Proteins; **(b)**
Top Proteins; **(c)**
Peptides;

|  |  |  |  |  |  |
| --- | --- | --- | --- | --- | --- |
| **(a)**  | **Do not support more than 8 samples** | | **(b)**  | **Do not support more than 8 samples** | | **(c)**  | **Do not support more than 8 samples** | |

**Figure 4.**
Distribution of peptide feature detection. **(a)**
Feature m/z distribution; **(b)**
Feature RT distribution.

|  |  |  |  |
| --- | --- | --- | --- |
| **(a)**  |  | | **(b)**  |  | |

**Figure 5.**
Distribution of identified peptide features. **(a)**
Feature abundance distribution; **(b)**
*De novo*
sequencing validation.

|  |  |  |  |
| --- | --- | --- | --- |
| **(a)**  |  | | **(b)**  |  | |

|  |  |  |  |  |  |  |  |  |  |  |  |  |  |  |  |  |  |  |  |  |  |  |  |  |  |  |  |  |  |  |  |  |  |  |  |  |  |  |  |  |
| --- | --- | --- | --- | --- | --- | --- | --- | --- | --- | --- | --- | --- | --- | --- | --- | --- | --- | --- | --- | --- | --- | --- | --- | --- | --- | --- | --- | --- | --- | --- | --- | --- | --- | --- | --- | --- | --- | --- | --- | --- |
| **Table 2.** Result filtration parameters.  | Peptide -10lgP | ≥53.3 | | PTM Ascore | ≥0 | | Protein -10lgP | ≥20 | | Proteins unique peptides | ≥1 | | De novo score(%) | ≥50% |    **Table 3.** Statistics of filtered result.  | FDR (Peptide-Spectrum Matches) | 0.1% | | FDR (Peptide Sequences) | 0.2% | | FDR (Protein Group) | 1.4% | | De Novo Only Spectra | 22768 | | **Table 4.** PTM profile.  | Name | ∆Mass | Position | #PSM | -10lgP | Abundance | AScore || Carbamidomethyl | 57.02 | C | 904 | 110.20 | 3.43E8 | 1000.00 | | Oxidation | 15.99 | M | 57 | 94.55 | 6.07E6 | 1000.00 | |

# 3. Experiment Control

**Figure 6.**
Precursor mass error of peptide-spectrum matches (PSM) in filtered result. **(a)**
Distribution of precursor mass error in ppm; **(b)**
Scatterplot of precursor m/z versus precursor mass error in ppm.

|  |  |  |  |
| --- | --- | --- | --- |
| **(a)**  |  | | **(b)**  |  | |

**Table 5.**
Number of identified peptides in each sample by the number of missed cleavages.

|  |  |  |  |  |  |  |  |  |  |  |  |  |  |  |  |  |  |  |  |  |  |  |  |  |  |  |  |  |  |  |  |  |  |  |  |  |  |  |  |  |  |  |  |  |  |  |  |  |  |  |  |  |  |  |  |  |  |  |  |  |  |  |  |  |  |  |
| --- | --- | --- | --- | --- | --- | --- | --- | --- | --- | --- | --- | --- | --- | --- | --- | --- | --- | --- | --- | --- | --- | --- | --- | --- | --- | --- | --- | --- | --- | --- | --- | --- | --- | --- | --- | --- | --- | --- | --- | --- | --- | --- | --- | --- | --- | --- | --- | --- | --- | --- | --- | --- | --- | --- | --- | --- | --- | --- | --- | --- | --- | --- | --- | --- | --- | --- |
| |  |  |  |  |  |  | | --- | --- | --- | --- | --- | --- | | Missed Cleavages | 0 | 1 | 2 | 3 | 4+ | | F1 | 138 | 5 | 1 | 0 | 0 | | F10 | 93 | 4 | 0 | 0 | 0 | | F2 | 55 | 1 | 0 | 0 | 0 | | F3 | 73 | 6 | 0 | 0 | 0 | | F4 | 91 | 4 | 1 | 0 | 0 | | F5 | 67 | 13 | 3 | 0 | 0 | | F7A | 67 | 13 | 2 | 0 | 0 | | F7B | 160 | 12 | 1 | 0 | 0 | | F8 | 116 | 4 | 0 | 0 | 0 | | F9 | 149 | 1 | 0 | 0 | 0 | |

# 4. Other Information

|  |  |  |  |
| --- | --- | --- | --- |
| **Table 6.** Search parameters.  | Search Engine Name: PEAKS Parent Mass Error Tolerance: 10.0 ppm Fragment Mass Error Tolerance: 0.6 Da Precursor Mass Search Type: monoisotopic Enzyme: Trypsin Max Missed Cleavages: 2 Digest Mode: Semispecific Fixed Modifications:    Carbamidomethylation: 57.02 Variable Modifications:    Oxidation (M): 15.99 Max Variable PTM Per Peptide: 3 Database: SwissProt Taxon: All Contaminant Database: cRAP\_contaminants Searched Entry: 560234 FDR Estimation: Enabled Merge Options: no merge Precursor Options: corrected Charge Options: no correction Filter Charge: 2 - 8 Process: true Associate chimera: yes | | **Table 7.** Instrument parameters.  | Fractions: DaRuMH\_F1.raw, DaRuMH\_F10.raw, DaRuMH\_F2.raw, DaRuMH \_F3.raw, DaRuMH\_F4.raw, DaRuMH\_F5.raw, DaRuMH\_F7A.raw, DaRuMH \_F7B.raw, DaRuMH\_F8.raw, DaRuMH\_F9.raw Ion Source: ESI(nano-spray) Fragmentation Mode: CID, CAD(y and b ions) MS Scan Mode: FT-ICR/Orbitrap MS/MS Scan Mode: FT-ICR/Orbitrap | |

  

Protein List

  

|  |
| --- |
| Protein Accession Contains: |
| Protein Description Contains: |
| Protein Sample Area >= |
| Protein PTM Contains: |

| Protein Group | Protein ID | Accession | -10lgP | Coverage (%) | Coverage (%) F1 | Coverage (%) F10 | Coverage (%) F2 | Coverage (%) F3 | Coverage (%) F4 | Coverage (%) F5 | Coverage (%) F7A | Coverage (%) F7B | Coverage (%) F8 | Coverage (%) F9 | Area F1 | Area F10 | Area F2 | Area F3 | Area F4 | Area F5 | Area F7A | Area F7B | Area F8 | Area F9 | #Peptides | #Unique | #Spec F1 | #Spec F10 | #Spec F2 | #Spec F3 | #Spec F4 | #Spec F5 | #Spec F7A | #Spec F7B | #Spec F8 | #Spec F9 | PTM | Avg. Mass | Description |
| --- | --- | --- | --- | --- | --- | --- | --- | --- | --- | --- | --- | --- | --- | --- | --- | --- | --- | --- | --- | --- | --- | --- | --- | --- | --- | --- | --- | --- | --- | --- | --- | --- | --- | --- | --- | --- | --- | --- | --- |
| 1 | 22 | A8CG90|PA2B2\_DABRR | 399.83 | 74 | 0 | 0 | 0 | 0 | 7 | 74 | 73 | 45 | 45 | 0 |  |  |  |  |  | 1.744E9 | 2.1535E9 | 3.9936E8 | 2.6607E6 |  | 32 | 23 | 0 | 0 | 0 | 0 | 1 | 143 | 87 | 16 | 6 | 0 | Y | 15461 | Basic phospholipase A2 Drk-b2 OS=Daboia russelii OX=8707 PE=1 SV=1 |
| 7 | 15 | P18964|VSPA\_DABSI | 373.28 | 74 | 60 | 0 | 0 | 0 | 0 | 0 | 0 | 72 | 68 | 19 | 4.5441E6 |  |  |  |  |  |  | 6.9595E7 | 1.6011E8 |  | 30 | 14 | 19 | 0 | 0 | 0 | 0 | 0 | 0 | 25 | 29 | 6 | Y | 26182 | Factor V activator RVV-V alpha OS=Daboia siamensis OX=343250 PE=1 SV=1 |
| 3 | 1 | P04264|K2C1\_HUMAN | 372.13 | 46 | 37 | 20 | 16 | 20 | 37 | 16 | 17 | 11 | 22 | 34 | 4.3073E7 | 2.4784E6 | 7.8838E5 | 2.4502E6 | 7.3777E6 | 2.8375E6 | 2.0502E6 | 1.1452E6 | 1.057E6 | 2.5566E7 | 27 | 19 | 28 | 11 | 9 | 11 | 21 | 15 | 9 | 7 | 11 | 25 | Y | 66039 | Keratin, type II cytoskeletal 1 OS=Homo sapiens OX=9606 GN=KRT1 PE=1 SV=6 |
| 11 | 33 | P18965|VSPG\_DABSI | 367.57 | 46 | 30 | 0 | 0 | 0 | 0 | 0 | 0 | 44 | 35 | 18 |  |  |  |  |  |  |  | 1.1866E7 | 2.1439E7 |  | 25 | 9 | 12 | 0 | 0 | 0 | 0 | 0 | 0 | 15 | 31 | 6 | Y | 28823 | Factor V activator RVV-V gamma OS=Daboia siamensis OX=343250 PE=1 SV=2 |
| 6 | 88 | A8CG86|PA2A1\_DABRR | 366.02 | 78 | 0 | 11 | 0 | 0 | 0 | 9 | 20 | 20 | 31 | 78 |  |  |  |  |  | 1.8285E7 | 1.3068E7 | 6.2845E6 | 2.7098E5 | 6.0408E8 | 28 | 12 | 0 | 1 | 0 | 0 | 0 | 11 | 6 | 4 | 4 | 60 | Y | 15329 | Acidic phospholipase A2 Drk-a1 OS=Daboia russelii OX=8707 PE=1 SV=1 |
| 4 | 81 | A8CG87|PA2A2\_DABRR | 360.12 | 71 | 21 | 0 | 9 | 18 | 28 | 7 | 58 | 71 | 9 | 0 | 7.2404E4 |  |  |  |  |  | 1.0705E7 | 3.2923E8 | 1.5164E5 |  | 23 | 8 | 2 | 0 | 1 | 3 | 5 | 2 | 12 | 49 | 1 | 0 | Y | 15586 | Acidic phospholipase A2 Drk-a2 OS=Daboia russelii OX=8707 PE=2 SV=1 |
| 4 | 82 | A8CG78|PA2A2\_DABSI | 360.12 | 71 | 21 | 0 | 9 | 18 | 28 | 7 | 58 | 71 | 9 | 0 | 7.2404E4 |  |  |  |  |  | 1.0705E7 | 3.2923E8 | 1.5164E5 |  | 23 | 8 | 2 | 0 | 1 | 3 | 5 | 2 | 12 | 49 | 1 | 0 | Y | 15586 | Acidic phospholipase A2 DsM-a2/DsM-a2' OS=Daboia siamensis OX=343250 PE=1 SV=1 |
| 15 | 7 | B8K1W0|VM3DK\_DABRR | 356.05 | 43 | 2 | 35 | 2 | 7 | 3 | 0 | 0 | 0 | 0 | 0 |  | 2.5307E8 |  | 1.1887E5 |  |  |  |  |  |  | 32 | 26 | 1 | 52 | 4 | 4 | 1 | 0 | 0 | 0 | 0 | 0 | Y | 69555 | Zinc metalloproteinase-disintegrin-like daborhagin-K OS=Daboia russelii OX=8707 PE=1 SV=1 |
| 2 | 69 | P81458|PA2B\_DABRR | 355.02 | 88 | 0 | 0 | 0 | 0 | 8 | 79 | 87 | 65 | 70 | 9 |  |  |  |  |  | 2.9345E8 | 1.5093E9 | 1.7234E8 | 2.8774E7 |  | 22 | 13 | 0 | 0 | 0 | 0 | 1 | 39 | 28 | 25 | 14 | 6 | Y | 13626 | Basic phospholipase A2 RVV-VD OS=Daboia russelii OX=8707 PE=1 SV=1 |
| 8 | 4 | P35527|K1C9\_HUMAN | 346.60 | 48 | 36 | 2 | 3 | 8 | 16 | 22 | 0 | 4 | 6 | 39 | 2.0143E7 | 2.7313E4 | 7.784E4 | 6.5421E5 | 1.5812E6 | 1.4371E6 |  | 6.0688E4 | 2.4259E5 | 1.6685E7 | 23 | 23 | 19 | 1 | 1 | 4 | 7 | 9 | 0 | 1 | 3 | 17 | Y | 62064 | Keratin, type I cytoskeletal 9 OS=Homo sapiens OX=9606 GN=KRT9 PE=1 SV=3 |
| 5 | 6 | P13645|K1C10\_HUMAN | 343.79 | 50 | 34 | 31 | 14 | 13 | 24 | 20 | 9 | 10 | 5 | 26 | 2.7361E7 | 4.4734E6 | 1.0949E6 | 1.2419E6 | 5.9377E6 | 1.2225E6 | 9.784E5 | 5.6664E5 | 1.8881E5 | 9.5841E6 | 25 | 21 | 20 | 14 | 8 | 7 | 13 | 9 | 6 | 4 | 2 | 15 | Y | 58827 | Keratin, type I cytoskeletal 10 OS=Homo sapiens OX=9606 GN=KRT10 PE=1 SV=6 |
| 12 | 60 | A8CG89|PA2B1\_DABRR | 338.21 | 57 | 50 | 0 | 0 | 0 | 0 | 0 | 18 | 57 | 50 | 0 | 9.1924E6 |  |  |  |  |  |  | 4.8483E6 | 1.4999E5 |  | 16 | 1 | 11 | 0 | 0 | 0 | 0 | 0 | 2 | 32 | 7 | 0 | Y | 15864 | Basic phospholipase A2 Drk-b1 OS=Daboia russelii OX=8707 PE=1 SV=1 |
| 14 | 84 | A8CG82|PA2B1\_DABSI | 334.18 | 51 | 43 | 0 | 0 | 0 | 0 | 0 | 18 | 45 | 38 | 0 | 0 |  |  |  |  |  |  |  |  |  | 15 | 1 | 10 | 0 | 0 | 0 | 0 | 0 | 2 | 29 | 5 | 0 | Y | 15843 | Basic phospholipase A2 DsM-b1/DsM-b1' OS=Daboia siamensis OX=343250 PE=1 SV=1 |
| 9 | 10 | P35908|K22E\_HUMAN | 329.47 | 40 | 30 | 20 | 11 | 8 | 22 | 7 | 7 | 6 | 5 | 16 | 7.3642E6 | 4.1806E5 | 4.2352E5 | 3.715E5 | 5.192E5 | 2.9462E5 | 6.2345E5 | 2.312E4 |  | 1.437E6 | 21 | 11 | 16 | 11 | 6 | 5 | 12 | 5 | 4 | 3 | 3 | 11 | Y | 65433 | Keratin, type II cytoskeletal 2 epidermal OS=Homo sapiens OX=9606 GN=KRT2 PE=1 SV=2 |
| 13 | 90 | P86368|PA2B3\_DABRR | 309.36 | 61 | 28 | 0 | 37 | 50 | 58 | 8 | 8 | 17 | 36 | 0 | 1.2505E5 |  | 1.0341E6 | 3.4535E7 | 1.7819E7 |  |  |  | 5.1561E4 |  | 20 | 6 | 4 | 0 | 10 | 17 | 16 | 2 | 1 | 3 | 3 | 0 | Y | 13687 | Basic phospholipase A2 3 OS=Daboia russelii OX=8707 PE=1 SV=1 |
| 43 | 154 | B7FDI0|CRVP\_VIPBN | 298.66 | 23 | 17 | 0 | 0 | 0 | 0 | 0 | 8 | 23 | 12 | 4 | 5.5704E6 |  |  |  |  |  | 2.0845E5 | 1.7387E8 |  |  | 13 | 8 | 4 | 0 | 0 | 0 | 0 | 0 | 1 | 16 | 3 | 1 | Y | 24612 | Cysteine-rich venom protein (Fragment) OS=Vipera berus nikolskii OX=1808362 PE=2 SV=1 |
| 20 | 43 | A0A1I9KNP0|VSPH1\_VIPAA | 289.14 | 39 | 8 | 0 | 0 | 0 | 0 | 0 | 22 | 34 | 34 | 15 |  |  |  |  |  |  |  |  | 6.8135E5 |  | 13 | 1 | 5 | 0 | 0 | 0 | 0 | 0 | 6 | 21 | 10 | 5 | Y | 28928 | Vaa serine proteinase homolog 1 OS=Vipera ammodytes ammodytes OX=8705 PE=1 SV=1 |
| 22 | 92 | P0DPS3|VASP1\_VIPAA | 285.38 | 34 | 14 | 0 | 0 | 0 | 0 | 0 | 18 | 34 | 28 | 24 |  |  |  |  |  |  | 7.5314E5 | 6.023E6 |  |  | 12 | 1 | 5 | 0 | 0 | 0 | 0 | 0 | 5 | 21 | 8 | 5 | Y | 22639 | Snake venom serine protease VaSP1 (Fragments) OS=Vipera ammodytes ammodytes OX=8705 PE=1 SV=1 |
| 23 | 34 | E5L0E4|VSPB\_DABSI | 279.24 | 41 | 14 | 0 | 0 | 0 | 0 | 3 | 14 | 35 | 24 | 10 | 1.232E7 |  |  |  |  |  | 1.2086E7 | 1.9899E8 | 6.4167E7 |  | 13 | 7 | 5 | 0 | 0 | 0 | 0 | 1 | 6 | 16 | 14 | 2 | Y | 28035 | Beta-fibrinogenase-like OS=Daboia siamensis OX=343250 PE=2 SV=1 |
| 21 | 57 | Q9PT40|VSP2\_MACLB | 277.55 | 40 | 13 | 0 | 0 | 0 | 0 | 0 | 14 | 40 | 34 | 15 |  |  |  |  |  |  |  | 1.4033E7 | 3.0772E6 |  | 13 | 1 | 6 | 0 | 0 | 0 | 0 | 0 | 5 | 21 | 10 | 5 | Y | 28894 | Venom serine proteinase-like protein 2 OS=Macrovipera lebetina OX=8709 PE=2 SV=1 |
| 34 | 149 | Q38L02|SLA\_DABSI | 276.56 | 42 | 13 | 0 | 0 | 0 | 0 | 0 | 8 | 28 | 42 | 39 | 4.5426E5 |  |  |  |  |  | 7.8149E4 | 6.4049E6 | 8.2332E6 | 3.1148E7 | 11 | 11 | 3 | 0 | 0 | 0 | 0 | 0 | 1 | 7 | 7 | 11 | Y | 17507 | Snaclec dabocetin subunit alpha OS=Daboia siamensis OX=343250 PE=1 SV=1 |
| 18 | 145 | Q910A1|PA2A1\_VIPAA | 275.31 | 47 | 12 | 0 | 9 | 18 | 25 | 7 | 33 | 45 | 0 | 0 |  |  |  |  |  |  | 1.9116E6 | 2.2378E7 |  |  | 13 | 3 | 1 | 0 | 1 | 3 | 4 | 2 | 6 | 19 | 0 | 0 | Y | 15435 | Acidic phospholipase A2 ammodytin I1 OS=Vipera ammodytes ammodytes OX=8705 PE=2 SV=1 |
| 41 | 208 | Q02471|PA2B4\_DABSI | 257.78 | 20 | 12 | 0 | 0 | 0 | 0 | 0 | 12 | 20 | 20 | 0 |  |  |  |  |  |  |  | 2.4555E5 | 2.9128E4 |  | 7 | 1 | 3 | 0 | 0 | 0 | 0 | 0 | 1 | 12 | 2 | 0 | Y | 15555 | Basic phospholipase A2 RV-4 OS=Daboia siamensis OX=343250 PE=1 SV=1 |
| 27 | 11 | P13647|K2C5\_HUMAN | 251.24 | 21 | 17 | 5 | 2 | 0 | 9 | 2 | 4 | 2 | 2 | 9 | 4.7707E5 |  |  |  | 4.2082E4 |  |  |  |  | 1.5833E5 | 12 | 2 | 10 | 3 | 1 | 0 | 6 | 2 | 2 | 1 | 1 | 7 | Y | 62378 | Keratin, type II cytoskeletal 5 OS=Homo sapiens OX=9606 GN=KRT5 PE=1 SV=3 |
| 76 | 47 | Q7LZ61|VM3CX\_DABSI | 249.25 | 8 | 2 | 0 | 2 | 0 | 1 | 0 | 0 | 2 | 0 | 6 |  |  |  |  |  |  |  |  |  | 3.6012E5 | 8 | 2 | 1 | 0 | 4 | 0 | 1 | 0 | 0 | 1 | 0 | 4 | Y | 69648 | Coagulation factor X-activating enzyme heavy chain OS=Daboia siamensis OX=343250 PE=1 SV=2 |
| 53 | 167 | A8Y7N6|VKTC3\_DABSI | 247.84 | 54 | 0 | 0 | 32 | 54 | 20 | 0 | 0 | 0 | 0 | 0 |  |  | 3.7347E6 | 3.1098E7 | 1.5243E5 |  |  |  |  |  | 9 | 6 | 0 | 0 | 4 | 10 | 1 | 0 | 0 | 0 | 0 | 0 | Y | 9443 | Kunitz-type serine protease inhibitor C3 OS=Daboia siamensis OX=343250 PE=2 SV=1 |
| 53 | 168 | A8Y7N7|VKTC4\_DABSI | 247.84 | 50 | 0 | 0 | 30 | 50 | 19 | 0 | 0 | 0 | 0 | 0 |  |  | 3.7347E6 | 3.1098E7 | 1.5243E5 |  |  |  |  |  | 9 | 6 | 0 | 0 | 4 | 10 | 1 | 0 | 0 | 0 | 0 | 0 | Y | 10162 | Kunitz-type serine protease inhibitor C4 OS=Daboia siamensis OX=343250 PE=2 SV=1 |
| 59 | 114 | Q7ZZN9|CRVP\_PROJR | 235.44 | 25 | 14 | 0 | 5 | 0 | 0 | 0 | 0 | 25 | 16 | 3 |  |  | 6.9905E5 |  |  |  |  | 6.2758E7 | 3.1943E6 |  | 7 | 1 | 5 | 0 | 1 | 0 | 0 | 0 | 0 | 10 | 4 | 1 | Y | 26865 | Cysteine-rich venom protein OS=Protobothrops jerdonii OX=242841 PE=1 SV=1 |
| 119 | 31 | O43790|KRT86\_HUMAN | 232.26 | 17 | 0 | 17 | 0 | 0 | 0 | 0 | 0 | 0 | 0 | 0 |  | 2.8442E5 |  |  |  |  |  |  |  |  | 6 | 2 | 0 | 8 | 0 | 0 | 0 | 0 | 0 | 0 | 0 | 0 | Y | 53501 | Keratin, type II cuticular Hb6 OS=Homo sapiens OX=9606 GN=KRT86 PE=1 SV=1 |
| 50 | 129 | E0Y419|VSPBF\_MACLB | 229.88 | 23 | 14 | 0 | 0 | 0 | 0 | 6 | 19 | 23 | 19 | 14 | 8.1646E5 |  |  |  |  | 3.7015E5 | 6.0638E6 | 6.8512E6 | 3.6491E6 | 2.7563E5 | 7 | 2 | 4 | 0 | 0 | 0 | 0 | 1 | 5 | 9 | 8 | 3 | Y | 28297 | Beta-fibrinogenase OS=Macrovipera lebetina OX=8709 PE=1 SV=1 |
| 58 | 14 | G8XQX1|OXLA\_DABRR | 227.31 | 19 | 0 | 19 | 0 | 0 | 0 | 0 | 0 | 0 | 6 | 2 |  | 2.206E6 |  |  |  |  |  |  | 2.8015E5 | 4.4799E5 | 9 | 9 | 0 | 10 | 0 | 0 | 0 | 0 | 0 | 0 | 2 | 1 | Y | 56888 | L-amino-acid oxidase OS=Daboia russelii OX=8707 PE=1 SV=1 |
| 96 | 307 | Q2ES47|VKT4\_DABRR | 223.89 | 48 | 0 | 0 | 32 | 48 | 0 | 0 | 0 | 0 | 0 | 0 |  |  | 7.8278E4 | 1.015E8 |  |  |  |  |  |  | 8 | 5 | 0 | 0 | 2 | 11 | 0 | 0 | 0 | 0 | 0 | 0 | Y | 9145 | Kunitz-type serine protease inhibitor 4 OS=Daboia russelii OX=8707 PE=2 SV=1 |
| 136 | 191 | Q4VM07|VM3VB\_MACLB | 210.67 | 6 | 2 | 4 | 2 | 0 | 0 | 0 | 0 | 0 | 0 | 0 |  | 1.1142E6 |  |  |  |  |  |  |  |  | 5 | 1 | 1 | 3 | 4 | 0 | 0 | 0 | 0 | 0 | 0 | 0 | N | 68843 | Zinc metalloproteinase-disintegrin-like VLAIP-B OS=Macrovipera lebetina OX=8709 PE=1 SV=1 |
| 88 | 16 | A5A6M5|K1H1\_PANTR | 206.84 | 24 | 2 | 23 | 2 | 2 | 0 | 0 | 0 | 0 | 0 | 0 |  | 2.6662E5 |  |  |  |  |  |  |  |  | 9 | 1 | 1 | 9 | 1 | 1 | 0 | 0 | 0 | 0 | 0 | 0 | Y | 47247 | Keratin, type I cuticular Ha1 OS=Pan troglodytes OX=9598 GN=KRT31 PE=2 SV=1 |
| 88 | 17 | Q15323|K1H1\_HUMAN | 206.84 | 24 | 2 | 23 | 2 | 2 | 0 | 0 | 0 | 0 | 0 | 0 |  | 2.6662E5 |  |  |  |  |  |  |  |  | 9 | 1 | 1 | 9 | 1 | 1 | 0 | 0 | 0 | 0 | 0 | 0 | Y | 47237 | Keratin, type I cuticular Ha1 OS=Homo sapiens OX=9606 GN=KRT31 PE=1 SV=3 |
| 46 | 35 | P02533|K1C14\_HUMAN | 201.77 | 18 | 12 | 2 | 1 | 3 | 2 | 0 | 0 | 2 | 0 | 9 |  | 1.7321E4 |  |  |  |  |  |  |  | 1.8198E5 | 8 | 2 | 6 | 1 | 1 | 2 | 1 | 0 | 0 | 1 | 0 | 3 | N | 51562 | Keratin, type I cytoskeletal 14 OS=Homo sapiens OX=9606 GN=KRT14 PE=1 SV=4 |
| 91 | 71 | Q4PRC6|SL7\_DABSI | 199.90 | 39 | 0 | 0 | 0 | 0 | 0 | 0 | 0 | 0 | 6 | 39 |  |  |  |  |  |  |  |  | 2.837E4 | 2.0305E7 | 5 | 4 | 0 | 0 | 0 | 0 | 0 | 0 | 0 | 0 | 1 | 8 | Y | 18067 | Snaclec 7 OS=Daboia siamensis OX=343250 PE=2 SV=1 |
| 75 | 127 | E0Y418|VSP1\_MACLB | 199.43 | 19 | 3 | 0 | 0 | 0 | 0 | 3 | 3 | 8 | 8 | 16 |  |  |  |  |  |  |  |  |  | 1.3666E7 | 5 | 3 | 1 | 0 | 0 | 0 | 0 | 1 | 1 | 2 | 2 | 6 | Y | 28702 | Serine protease VLSP-1 OS=Macrovipera lebetina OX=8709 PE=2 SV=1 |
| 77 | 148 | E0Y420|VSP3\_MACLB | 199.18 | 19 | 3 | 0 | 0 | 0 | 0 | 0 | 8 | 19 | 16 | 3 |  |  |  |  |  |  |  | 5.5452E6 | 2.0714E6 |  | 5 | 1 | 1 | 0 | 0 | 0 | 0 | 0 | 2 | 5 | 4 | 1 | Y | 28352 | Serine protease VLSP-3 OS=Macrovipera lebetina OX=8709 PE=2 SV=1 |
| 69 | 169 | A8Y7P1|VKTB1\_DABSI | 198.31 | 46 | 0 | 0 | 27 | 46 | 0 | 0 | 0 | 0 | 0 | 0 |  |  | 2.0182E5 | 1.0269E7 |  |  |  |  |  |  | 5 | 2 | 0 | 0 | 4 | 6 | 0 | 0 | 0 | 0 | 0 | 0 | Y | 9318 | Kunitz-type serine protease inhibitor B1 OS=Daboia siamensis OX=343250 PE=1 SV=1 |
| 69 | 170 | A8Y7P5|VKTB5\_DABSI | 198.31 | 43 | 0 | 0 | 26 | 43 | 0 | 0 | 0 | 0 | 0 | 0 |  |  | 2.0182E5 | 1.0269E7 |  |  |  |  |  |  | 5 | 2 | 0 | 0 | 4 | 6 | 0 | 0 | 0 | 0 | 0 | 0 | Y | 9901 | Kunitz-type serine protease inhibitor B5 OS=Daboia siamensis OX=343250 PE=2 SV=1 |
| 111 | 304 | A8Y7N8|VKTC5\_DABSI | 197.48 | 43 | 38 | 10 | 26 | 0 | 17 | 0 | 0 | 0 | 0 | 0 | 1.4811E6 | 3.1845E4 |  |  |  |  |  |  |  |  | 5 | 1 | 3 | 1 | 2 | 0 | 1 | 0 | 0 | 0 | 0 | 0 | Y | 10006 | Kunitz-type serine protease inhibitor C5 OS=Daboia siamensis OX=343250 PE=2 SV=1 |
| 118 | 28 | O76011|KRT34\_HUMAN | 191.62 | 20 | 0 | 20 | 0 | 0 | 0 | 0 | 0 | 0 | 0 | 0 |  | 9.9721E4 |  |  |  |  |  |  |  |  | 7 | 2 | 0 | 8 | 0 | 0 | 0 | 0 | 0 | 0 | 0 | 0 | Y | 49424 | Keratin, type I cuticular Ha4 OS=Homo sapiens OX=9606 GN=KRT34 PE=1 SV=2 |
| 94 | 123 | Q7T229|VSPH\_BOTJR | 190.72 | 20 | 3 | 0 | 0 | 0 | 0 | 0 | 8 | 12 | 17 | 8 |  |  |  |  |  |  |  |  | 8.2963E3 | 2.5156E6 | 5 | 2 | 1 | 0 | 0 | 0 | 0 | 0 | 2 | 3 | 4 | 2 | Y | 28654 | Snake venom serine protease homolog OS=Bothrops jararacussu OX=8726 PE=1 SV=1 |
| 62 | 41 | P08779|K1C16\_HUMAN | 185.71 | 12 | 10 | 0 | 1 | 3 | 2 | 0 | 0 | 2 | 0 | 5 |  |  |  |  |  |  |  |  |  | 9.5971E4 | 6 | 1 | 5 | 0 | 1 | 2 | 1 | 0 | 0 | 1 | 0 | 2 | Y | 51268 | Keratin, type I cytoskeletal 16 OS=Homo sapiens OX=9606 GN=KRT16 PE=1 SV=4 |
| 97 | 55 | E5L0E3|VSPAF\_DABSI | 183.46 | 19 | 3 | 0 | 0 | 0 | 0 | 0 | 3 | 6 | 16 | 12 |  |  |  |  |  |  |  |  | 1.2532E6 | 2.7914E6 | 6 | 4 | 1 | 0 | 0 | 0 | 0 | 0 | 1 | 2 | 5 | 4 | Y | 28496 | Alpha-fibrinogenase-like OS=Daboia siamensis OX=343250 PE=2 SV=1 |
| 108 | 240 | P01391|3L21\_NAJKA | 179.69 | 59 | 0 | 0 | 59 | 59 | 59 | 0 | 0 | 0 | 0 | 14 |  |  | 4.5612E5 | 9.1647E6 | 1.7959E6 |  |  |  |  |  | 4 | 2 | 0 | 0 | 3 | 5 | 4 | 0 | 0 | 0 | 0 | 1 | Y | 7831 | Alpha-cobratoxin OS=Naja kaouthia OX=8649 PE=1 SV=1 |
| 137 | 310 | Q2ES50|VKT1\_DABRR | 171.66 | 49 | 12 | 11 | 12 | 0 | 18 | 0 | 20 | 0 | 0 | 0 |  |  |  |  |  |  | 1.0169E5 |  |  |  | 4 | 1 | 1 | 1 | 1 | 0 | 1 | 0 | 1 | 0 | 0 | 0 | Y | 9287 | Kunitz-type serine protease inhibitor 1 OS=Daboia russelii OX=8707 PE=2 SV=1 |
| 137 | 311 | A8Y7P0|VKTB7\_DABSI | 171.66 | 49 | 12 | 11 | 12 | 0 | 18 | 0 | 20 | 0 | 0 | 0 |  |  |  |  |  |  | 1.0169E5 |  |  |  | 4 | 1 | 1 | 1 | 1 | 0 | 1 | 0 | 1 | 0 | 0 | 0 | Y | 9287 | Kunitz-type serine protease inhibitor 7 OS=Daboia siamensis OX=343250 PE=2 SV=1 |
| 89 | 96 | Q7Z794|K2C1B\_HUMAN | 161.31 | 4 | 4 | 2 | 2 | 2 | 2 | 2 | 2 | 2 | 2 | 2 | 5.2474E4 |  |  |  |  |  |  |  |  |  | 2 | 1 | 2 | 1 | 1 | 1 | 1 | 1 | 1 | 1 | 1 | 1 | N | 61901 | Keratin, type II cytoskeletal 1b OS=Homo sapiens OX=9606 GN=KRT77 PE=2 SV=3 |
| 128 | 217 | P25669|3L22\_NAJNA | 160.92 | 46 | 0 | 0 | 46 | 46 | 32 | 0 | 0 | 0 | 0 | 14 |  |  | 8.4091E4 | 3.5572E5 |  |  |  |  |  |  | 3 | 1 | 0 | 0 | 3 | 3 | 2 | 0 | 0 | 0 | 0 | 1 | Y | 7821 | Long neurotoxin 2 OS=Naja naja OX=35670 PE=1 SV=1 |
| 68 | 272 | Q6H3C5|PA2BB\_TRIST | 158.56 | 25 | 0 | 0 | 0 | 11 | 11 | 15 | 15 | 15 | 0 | 0 |  |  |  |  |  | 0 | 0 | 0 |  |  | 2 | 1 | 0 | 0 | 0 | 1 | 1 | 2 | 1 | 1 | 0 | 0 | Y | 13819 | Basic phospholipase A2 Ts-G6D49 OS=Trimeresurus stejnegeri OX=39682 PE=1 SV=2 |
| 126 | 220 | P25668|3L21\_NAJNA | 156.93 | 46 | 0 | 0 | 32 | 46 | 32 | 0 | 0 | 0 | 0 | 14 |  |  |  | 2.0135E6 |  |  |  |  |  |  | 3 | 1 | 0 | 0 | 2 | 3 | 2 | 0 | 0 | 0 | 0 | 1 | Y | 7847 | Long neurotoxin 1 OS=Naja naja OX=35670 PE=1 SV=1 |
| 82 | 212 | O42191|PA2A7\_GLOHA | 141.74 | 12 | 0 | 0 | 0 | 0 | 0 | 0 | 0 | 0 | 0 | 12 |  |  |  |  |  |  |  |  |  | 6.4196E4 | 1 | 1 | 0 | 0 | 0 | 0 | 0 | 0 | 0 | 0 | 0 | 1 | Y | 13927 | Acidic phospholipase A2 A OS=Gloydius halys OX=8714 PE=1 SV=2 |
| 168 | 507 | Q90495|VM3E\_ECHCA | 141.05 | 2 | 0 | 0 | 2 | 0 | 0 | 0 | 0 | 0 | 0 | 0 |  |  | 1.7976E5 |  |  |  |  |  |  |  | 3 | 1 | 0 | 0 | 3 | 0 | 0 | 0 | 0 | 0 | 0 | 0 | N | 69463 | Zinc metalloproteinase-disintegrin-like ecarin OS=Echis carinatus OX=40353 PE=1 SV=1 |
| 144 | 103 | B6EWW8|V5NTD\_GLOBR | 134.05 | 5 | 0 | 0 | 2 | 5 | 0 | 0 | 0 | 0 | 0 | 0 |  |  | 1.0261E4 | 1.8983E5 |  |  |  |  |  |  | 2 | 2 | 0 | 0 | 1 | 2 | 0 | 0 | 0 | 0 | 0 | 0 | Y | 64434 | Snake venom 5'-nucleotidase OS=Gloydius brevicaudus OX=259325 PE=2 SV=1 |
| 144 | 106 | F8S0Z7|V5NTD\_CROAD | 134.05 | 5 | 0 | 0 | 2 | 5 | 0 | 0 | 0 | 0 | 0 | 0 |  |  | 1.0261E4 | 1.8983E5 |  |  |  |  |  |  | 2 | 2 | 0 | 0 | 1 | 2 | 0 | 0 | 0 | 0 | 0 | 0 | Y | 64682 | Snake venom 5'-nucleotidase OS=Crotalus adamanteus OX=8729 PE=1 SV=2 |
| 164 | 979 | P24541|VKT\_ERIMA | 133.89 | 27 | 0 | 0 | 24 | 27 | 0 | 0 | 0 | 0 | 0 | 0 |  |  |  | 7.5031E4 |  |  |  |  |  |  | 2 | 1 | 0 | 0 | 1 | 2 | 0 | 0 | 0 | 0 | 0 | 0 | Y | 6772 | Kunitz-type serine protease inhibitor OS=Eristicophis macmahoni OX=110227 PE=1 SV=1 |
| 143 | 105 | P15924|DESP\_HUMAN | 132.32 | 1 | 1 | 0 | 0 | 0 | 0 | 0 | 0 | 0 | 0 | 0 | 2.8931E4 |  |  |  |  |  |  |  |  |  | 2 | 2 | 2 | 0 | 0 | 0 | 0 | 0 | 0 | 0 | 0 | 0 | Y | 331774 | Desmoplakin OS=Homo sapiens OX=9606 GN=DSP PE=1 SV=3 |
| 131 | 331 | Q696W1|SLLC2\_MACLB | 130.60 | 14 | 0 | 0 | 0 | 6 | 0 | 0 | 0 | 0 | 14 | 13 |  |  |  | 3.6952E5 |  |  |  |  | 7.3112E5 | 2.17E6 | 3 | 3 | 0 | 0 | 0 | 1 | 0 | 0 | 0 | 0 | 3 | 2 | Y | 18094 | Snaclec coagulation factor X-activating enzyme light chain 2 OS=Macrovipera lebetina OX=8709 GN=LC2 PE=1 SV=1 |
| 114 | 116 | Q8N1N4|K2C78\_HUMAN | 128.48 | 4 | 0 | 2 | 0 | 0 | 0 | 0 | 0 | 0 | 0 | 2 |  |  |  |  |  |  |  |  |  | 3.4036E4 | 2 | 1 | 0 | 1 | 0 | 0 | 0 | 0 | 0 | 0 | 0 | 1 | Y | 56866 | Keratin, type II cytoskeletal 78 OS=Homo sapiens OX=9606 GN=KRT78 PE=1 SV=2 |
| 129 | 210 | P25428|NGFV\_MACLB | 124.88 | 15 | 0 | 0 | 6 | 0 | 7 | 3 | 3 | 0 | 0 | 0 |  |  | 6.9372E4 |  | 8.3033E4 |  |  |  |  |  | 3 | 2 | 0 | 0 | 1 | 0 | 1 | 1 | 1 | 0 | 0 | 0 | Y | 27318 | Venom nerve growth factor OS=Macrovipera lebetina OX=8709 PE=1 SV=2 |
| 117 | 316 | P81605|DCD\_HUMAN | 123.31 | 41 | 10 | 10 | 10 | 21 | 20 | 0 | 10 | 10 | 0 | 10 | 5.9124E4 | 1.425E4 | 2.5225E4 | 9.4139E4 | 1.2771E5 |  | 2.8742E4 | 0 |  | 6.2008E4 | 3 | 3 | 1 | 1 | 1 | 1 | 2 | 0 | 1 | 1 | 0 | 1 | Y | 11284 | Dermcidin OS=Homo sapiens OX=9606 GN=DCD PE=1 SV=2 |
| 158 | 98 | Q8SPJ1|PLAK\_BOVIN | 120.39 | 1 | 1 | 0 | 0 | 0 | 0 | 0 | 0 | 0 | 0 | 0 | 2.2708E4 |  |  |  |  |  |  |  |  |  | 1 | 1 | 1 | 0 | 0 | 0 | 0 | 0 | 0 | 0 | 0 | 0 | N | 81821 | Junction plakoglobin OS=Bos taurus OX=9913 GN=JUP PE=2 SV=1 |
| 158 | 99 | Q02257|PLAK\_MOUSE | 120.39 | 1 | 1 | 0 | 0 | 0 | 0 | 0 | 0 | 0 | 0 | 0 | 2.2708E4 |  |  |  |  |  |  |  |  |  | 1 | 1 | 1 | 0 | 0 | 0 | 0 | 0 | 0 | 0 | 0 | 0 | N | 81801 | Junction plakoglobin OS=Mus musculus OX=10090 GN=Jup PE=1 SV=3 |
| 158 | 100 | P14923|PLAK\_HUMAN | 120.39 | 1 | 1 | 0 | 0 | 0 | 0 | 0 | 0 | 0 | 0 | 0 | 2.2708E4 |  |  |  |  |  |  |  |  |  | 1 | 1 | 1 | 0 | 0 | 0 | 0 | 0 | 0 | 0 | 0 | 0 | N | 81745 | Junction plakoglobin OS=Homo sapiens OX=9606 GN=JUP PE=1 SV=3 |
| 155 | 463 | Q8JIR2|VM3HA\_PROFL | 119.48 | 4 | 0 | 4 | 0 | 0 | 0 | 0 | 0 | 0 | 0 | 0 |  | 1.6367E5 |  |  |  |  |  |  |  |  | 3 | 1 | 0 | 3 | 0 | 0 | 0 | 0 | 0 | 0 | 0 | 0 | Y | 68766 | Zinc metalloproteinase/disintegrin-like HR1a OS=Protobothrops flavoviridis OX=88087 PE=1 SV=1 |
| 132 | 419 | Q2ES49|VKT2\_DABRR | 116.91 | 27 | 0 | 0 | 0 | 0 | 27 | 0 | 0 | 0 | 0 | 0 |  |  |  |  | 3.6105E7 |  |  |  |  |  | 2 | 2 | 0 | 0 | 0 | 0 | 4 | 0 | 0 | 0 | 0 | 0 | Y | 9683 | Kunitz-type serine protease inhibitor 2 OS=Daboia russelii OX=8707 PE=2 SV=1 |
| 145 | 248 | Q4PRD1|SLLC1\_DABSI | 116.79 | 12 | 0 | 0 | 0 | 0 | 0 | 0 | 0 | 0 | 12 | 0 |  |  |  |  |  |  |  |  | 4.9355E5 |  | 1 | 1 | 0 | 0 | 0 | 0 | 0 | 0 | 0 | 0 | 1 | 0 | Y | 16871 | Snaclec coagulation factor X-activating enzyme light chain 1 OS=Daboia siamensis OX=343250 GN=LC1 PE=1 SV=2 |
| 162 | 634 | P0DL42|TXVE\_DABSI | 112.43 | 17 | 0 | 0 | 0 | 0 | 0 | 0 | 0 | 17 | 0 | 0 |  |  |  |  |  |  |  | 1.723E5 |  |  | 1 | 1 | 0 | 0 | 0 | 0 | 0 | 0 | 0 | 1 | 0 | 0 | N | 12554 | Snake venom vascular endothelial growth factor toxin VR-1' OS=Daboia siamensis OX=343250 PE=1 SV=1 |
| 162 | 635 | P67861|TXVE\_DABRR | 112.43 | 13 | 0 | 0 | 0 | 0 | 0 | 0 | 0 | 13 | 0 | 0 |  |  |  |  |  |  |  | 1.723E5 |  |  | 1 | 1 | 0 | 0 | 0 | 0 | 0 | 0 | 0 | 1 | 0 | 0 | N | 16278 | Snake venom vascular endothelial growth factor toxin VR-1 OS=Daboia russelii OX=8707 PE=1 SV=2 |
| 141 | 275 | P30894|NGFV\_DABRR | 110.35 | 15 | 0 | 0 | 0 | 0 | 0 | 15 | 6 | 9 | 0 | 0 |  |  |  |  |  | 1.6231E5 |  | 1.3202E5 |  |  | 2 | 1 | 0 | 0 | 0 | 0 | 0 | 2 | 1 | 1 | 0 | 0 | Y | 13283 | Venom nerve growth factor OS=Daboia russelii OX=8707 PE=1 SV=1 |
| 159 | 549 | B4XSY8|SLAD\_MACLB | 108.64 | 17 | 0 | 0 | 0 | 0 | 0 | 0 | 0 | 0 | 8 | 17 |  |  |  |  |  |  |  |  | 2.1987E4 | 2.7845E6 | 2 | 2 | 0 | 0 | 0 | 0 | 0 | 0 | 0 | 0 | 1 | 2 | N | 15308 | Snaclec A13 OS=Macrovipera lebetina OX=8709 PE=2 SV=1 |
| 159 | 550 | B4XSY7|SLAC\_MACLB | 108.64 | 14 | 0 | 0 | 0 | 0 | 0 | 0 | 0 | 0 | 6 | 14 |  |  |  |  |  |  |  |  | 2.1987E4 | 2.7845E6 | 2 | 2 | 0 | 0 | 0 | 0 | 0 | 0 | 0 | 0 | 1 | 2 | N | 17717 | Snaclec A12 OS=Macrovipera lebetina OX=8709 PE=2 SV=1 |
| 150 | 919 | W5XCJ6|SLCIB\_MACLB | 92.61 | 8 | 0 | 0 | 0 | 0 | 0 | 0 | 0 | 0 | 0 | 8 |  |  |  |  |  |  |  |  |  | 1.9931E5 | 1 | 1 | 0 | 0 | 0 | 0 | 0 | 0 | 0 | 0 | 0 | 1 | Y | 17553 | Snaclec lebecin subunit beta OS=Macrovipera lebetina OX=8709 PE=1 SV=1 |
| 150 | 920 | B4XSZ0|SLAF\_MACLB | 92.61 | 8 | 0 | 0 | 0 | 0 | 0 | 0 | 0 | 0 | 0 | 8 |  |  |  |  |  |  |  |  |  | 1.9931E5 | 1 | 1 | 0 | 0 | 0 | 0 | 0 | 0 | 0 | 0 | 0 | 1 | Y | 17759 | Snaclec A15 OS=Macrovipera lebetina OX=8709 PE=2 SV=1 |
| 150 | 921 | B4XSY9|SLAE\_MACLB | 92.61 | 8 | 0 | 0 | 0 | 0 | 0 | 0 | 0 | 0 | 0 | 8 |  |  |  |  |  |  |  |  |  | 1.9931E5 | 1 | 1 | 0 | 0 | 0 | 0 | 0 | 0 | 0 | 0 | 0 | 1 | Y | 17711 | Snaclec A14 OS=Macrovipera lebetina OX=8709 PE=2 SV=1 |
| 181 | 1029 | B4XSY5|SLAA\_MACLB | 91.08 | 6 | 0 | 0 | 0 | 0 | 0 | 0 | 0 | 0 | 0 | 6 |  |  |  |  |  |  |  |  |  | 2.3905E5 | 1 | 1 | 0 | 0 | 0 | 0 | 0 | 0 | 0 | 0 | 0 | 1 | Y | 17731 | Snaclec A10 OS=Macrovipera lebetina OX=8709 PE=2 SV=1 |
| 181 | 1315 | B4XSY6|SLAB\_MACLB | 91.08 | 6 | 0 | 0 | 0 | 0 | 0 | 0 | 0 | 0 | 0 | 6 |  |  |  |  |  |  |  |  |  | 2.3905E5 | 1 | 1 | 0 | 0 | 0 | 0 | 0 | 0 | 0 | 0 | 0 | 1 | Y | 17738 | Snaclec A11 OS=Macrovipera lebetina OX=8709 PE=2 SV=1 |
| 163 | 605 | P86382|3SA7\_NAJNA | 73.18 | 13 | 0 | 0 | 0 | 0 | 0 | 0 | 0 | 0 | 0 | 13 |  |  |  |  |  |  |  |  |  | 5.1097E5 | 1 | 1 | 0 | 0 | 0 | 0 | 0 | 0 | 0 | 0 | 0 | 1 | Y | 6792 | Cytotoxin 7 OS=Naja naja OX=35670 PE=1 SV=1 |
| 163 | 697 | P01445|3SA7A\_NAJKA | 73.18 | 13 | 0 | 0 | 0 | 0 | 0 | 0 | 0 | 0 | 0 | 13 |  |  |  |  |  |  |  |  |  | 5.1097E5 | 1 | 1 | 0 | 0 | 0 | 0 | 0 | 0 | 0 | 0 | 0 | 1 | Y | 6745 | Cytotoxin 2 OS=Naja kaouthia OX=8649 PE=1 SV=1 |
| 163 | 698 | P86540|3SA8\_NAJNA | 73.18 | 13 | 0 | 0 | 0 | 0 | 0 | 0 | 0 | 0 | 0 | 13 |  |  |  |  |  |  |  |  |  | 5.1097E5 | 1 | 1 | 0 | 0 | 0 | 0 | 0 | 0 | 0 | 0 | 0 | 1 | Y | 6793 | Cytotoxin 8 OS=Naja naja OX=35670 PE=1 SV=2 |
| 171 | 458 | C0HJL8|PA2B\_BOTNI | 72.02 | 22 | 0 | 0 | 0 | 0 | 0 | 0 | 0 | 0 | 0 | 22 |  |  |  |  |  |  |  |  |  | 3.8023E5 | 1 | 1 | 0 | 0 | 0 | 0 | 0 | 0 | 0 | 0 | 0 | 1 | Y | 14126 | Phospholipase A2 nigroviriditoxin basic subunit B OS=Bothriechis nigroviridis OX=88079 PE=1 SV=1 |
| 156 | 1322 | Q56EB1|SLAA\_BOTJA | 59.80 | 10 | 0 | 0 | 0 | 0 | 0 | 0 | 0 | 10 | 0 | 10 |  |  |  |  |  |  |  | 2.2413E5 |  | 3.2302E5 | 1 | 1 | 0 | 0 | 0 | 0 | 0 | 0 | 0 | 1 | 0 | 1 | Y | 17577 | Snaclec bothrojaracin subunit alpha OS=Bothrops jararaca OX=8724 PE=1 SV=1 |
| 204 | 479 | Q7ZZM2|DIS\_PROJR | 58.03 | 12 | 0 | 0 | 12 | 0 | 0 | 0 | 0 | 0 | 0 | 0 |  |  | 5.5757E4 |  |  |  |  |  |  |  | 1 | 1 | 0 | 0 | 1 | 0 | 0 | 0 | 0 | 0 | 0 | 0 | N | 11849 | Disintegrin jerdostatin OS=Protobothrops jerdonii OX=242841 PE=1 SV=1 |
| 204 | 1320 | Q3BK14|DIS\_MACLB | 58.03 | 12 | 0 | 0 | 12 | 0 | 0 | 0 | 0 | 0 | 0 | 0 |  |  | 5.5757E4 |  |  |  |  |  |  |  | 1 | 1 | 0 | 0 | 1 | 0 | 0 | 0 | 0 | 0 | 0 | 0 | N | 11498 | Disintegrin lebestatin OS=Macrovipera lebetina OX=8709 PE=1 SV=1 |
| 185 | 398 | Q86YZ3|HORN\_HUMAN | 54.09 | 1 | 0 | 0 | 0 | 0 | 1 | 0 | 0 | 0 | 0 | 0 |  |  |  |  | 0 |  |  |  |  |  | 1 | 1 | 0 | 0 | 0 | 0 | 1 | 0 | 0 | 0 | 0 | 0 | N | 282389 | Hornerin OS=Homo sapiens OX=9606 GN=HRNR PE=1 SV=2 |
| total 87 proteins |
| --- |

  

A8CG90|PA2B2\_DABRR

back to list

  

| Protein Coverage
| Supporting Peptides
|

Protein Coverage:

Supporting Peptides:

| Peptide | Uniq | -10lgP | Mass | Length | ppm | m/z | z | RT | Fraction | Scan | Source File | Area F1 | Area F10 | Area F2 | Area F3 | Area F4 | Area F5 | Area F7A | Area F7B | Area F8 | Area F9 | #Feature | #Feature F1 | #Feature F10 | #Feature F2 | #Feature F3 | #Feature F4 | #Feature F5 | #Feature F7A | #Feature F7B | #Feature F8 | #Feature F9 | Start | End | PTM | AScore | Found By |
| --- | --- | --- | --- | --- | --- | --- | --- | --- | --- | --- | --- | --- | --- | --- | --- | --- | --- | --- | --- | --- | --- | --- | --- | --- | --- | --- | --- | --- | --- | --- | --- | --- | --- | --- | --- | --- | --- |
| K.NPLSSYSNYGC(+57.02)YC(+57.02)GWGGK.G | Y | 110.20 | 2068.8408 | 18 | -0.4 | 1035.4272 | 2 | 14.80 | 8 | F8:5483 | DaRuMH\_F7B.raw |  |  |  |  |  | 8.0497E8 | 1.5556E9 | 3.4711E8 |  |  | 8 | 0 | 0 | 0 | 0 | 0 | 3 | 2 | 3 | 0 | 0 | 32 | 49 | Carbamidomethylation | C11:Carbamidomethylation:1000.00;C13:Carbamidomethylation:1000.00 | PEAKS DB |
| K.TATYSYSFENGGIVC(+57.02)GDRDPC(+57.02)K.R | Y | 107.94 | 2496.0686 | 22 | -0.1 | 1249.0415 | 2 | 11.90 | 6 | F6:2670 | DaRuMH\_F5.raw |  |  |  |  |  | 4.0853E7 | 5.3005E7 |  |  |  | 4 | 0 | 0 | 0 | 0 | 0 | 2 | 2 | 0 | 0 | 0 | 77 | 98 | Carbamidomethylation | C15:Carbamidomethylation:1000.00;C21:Carbamidomethylation:1000.00 | PEAKS DB |
| R.AVC(+57.02)EC(+57.02)DRVAATC(+57.02)FR.D | N | 100.61 | 1713.7385 | 14 | 1.2 | 857.8776 | 2 | 11.27 | 6 | F6:2110 | DaRuMH\_F5.raw |  |  |  |  |  | 4.9245E6 | 1.9768E5 |  |  |  | 3 | 0 | 0 | 0 | 0 | 0 | 2 | 1 | 0 | 0 | 0 | 100 | 113 | Carbamidomethylation | C3:Carbamidomethylation:1000.00;C5:Carbamidomethylation:1000.00;C12:Carbamidomethylation:1000.00 | PEAKS DB |
| K.TATYSYSFENGGIVC(+57.02)GDR.D | Y | 100.41 | 1995.8633 | 18 | -1.1 | 998.9379 | 2 | 13.18 | 6 | F6:3704 | DaRuMH\_F5.raw |  |  |  |  |  | 5.2999E8 | 3.3723E8 | 2.5316E6 | 3.9579E5 |  | 20 | 0 | 0 | 0 | 0 | 0 | 7 | 11 | 1 | 1 | 0 | 77 | 94 | Carbamidomethylation | C15:Carbamidomethylation:1000.00 | PEAKS DB |
| K.NPLSSYSNYGC(+57.02)YC(+57.02)GWGGKG.K | Y | 88.88 | 2125.8623 | 19 | 0.0 | 1063.9385 | 2 | 14.63 | 8 | F8:5178 | DaRuMH\_F7B.raw |  |  |  |  |  | 4.0595E7 | 7.2817E7 | 1.2848E7 |  |  | 3 | 0 | 0 | 0 | 0 | 0 | 1 | 1 | 1 | 0 | 0 | 32 | 50 | Carbamidomethylation | C11:Carbamidomethylation:1000.00;C13:Carbamidomethylation:1000.00 | PEAKS DB |
| N.PLSSYSNYGC(+57.02)YC(+57.02)GWGGK.G | Y | 88.63 | 1954.7979 | 17 | 0.4 | 978.4066 | 2 | 12.48 | 6 | F6:3174 | DaRuMH\_F5.raw |  |  |  |  |  | 9.6974E5 | 2.0487E6 |  |  |  | 2 | 0 | 0 | 0 | 0 | 0 | 1 | 1 | 0 | 0 | 0 | 33 | 49 | Carbamidomethylation | C10:Carbamidomethylation:1000.00;C12:Carbamidomethylation:1000.00 | PEAKS DB |
| R.VAATC(+57.02)FRDNLNTYDKK.Y | N | 83.45 | 1914.9258 | 16 | 0.3 | 639.3160 | 3 | 11.26 | 6 | F6:2101 | DaRuMH\_F5.raw |  |  |  |  |  | 8.1557E6 | 1.2526E5 |  |  |  | 3 | 0 | 0 | 0 | 0 | 0 | 2 | 1 | 0 | 0 | 0 | 107 | 122 | Carbamidomethylation | C5:Carbamidomethylation:1000.00 | PEAKS DB |
| L.SSYSNYGC(+57.02)YC(+57.02)GWGGK.G | Y | 82.59 | 1744.6610 | 15 | -2.5 | 873.3356 | 2 | 11.84 | 6 | F6:2616 | DaRuMH\_F5.raw |  |  |  |  |  | 2.4996E7 | 2.8696E7 | 6.3648E6 |  |  | 3 | 0 | 0 | 0 | 0 | 0 | 1 | 1 | 1 | 0 | 0 | 35 | 49 | Carbamidomethylation | C8:Carbamidomethylation:1000.00;C10:Carbamidomethylation:1000.00 | PEAKS DB |
| Y.SFENGGIVC(+57.02)GDRDPC(+57.02)K.R | Y | 82.06 | 1809.7773 | 16 | 0.9 | 905.8967 | 2 | 11.29 | 6 | F6:2138 | DaRuMH\_F5.raw |  |  |  |  |  | 3.6444E6 | 3.4355E6 |  |  |  | 4 | 0 | 0 | 0 | 0 | 0 | 2 | 2 | 0 | 0 | 0 | 83 | 98 | Carbamidomethylation | C9:Carbamidomethylation:1000.00;C15:Carbamidomethylation:1000.00 | PEAKS DB |
| S.YSFENGGIVC(+57.02)GDR.D | Y | 81.51 | 1472.6354 | 13 | 1.0 | 737.3257 | 2 | 11.97 | 6 | F6:2731 | DaRuMH\_F5.raw |  |  |  |  |  | 1.2743E6 |  |  |  |  | 1 | 0 | 0 | 0 | 0 | 0 | 1 | 0 | 0 | 0 | 0 | 82 | 94 | Carbamidomethylation | C10:Carbamidomethylation:1000.00 | PEAKS DB |
| P.LSSYSNYGC(+57.02)YC(+57.02)GWGGK.G | Y | 81.41 | 1857.7450 | 16 | 0.6 | 929.8804 | 2 | 12.10 | 6 | F6:2846 | DaRuMH\_F5.raw |  |  |  |  |  | 1.0552E6 | 1.4962E6 |  |  |  | 2 | 0 | 0 | 0 | 0 | 0 | 1 | 1 | 0 | 0 | 0 | 34 | 49 | Carbamidomethylation | C9:Carbamidomethylation:1000.00;C11:Carbamidomethylation:1000.00 | PEAKS DB |
| R.MTAKNPLSSYSNYGC(+57.02)YC(+57.02)GWGGK.G | Y | 80.85 | 2500.0610 | 22 | 1.0 | 834.3618 | 3 | 12.42 | 6 | F6:3132 | DaRuMH\_F5.raw |  |  |  |  |  | 0 | 1.0811E6 |  |  |  | 1 | 0 | 0 | 0 | 0 | 0 | 0 | 1 | 0 | 0 | 0 | 28 | 49 | Carbamidomethylation | C15:Carbamidomethylation:1000.00;C17:Carbamidomethylation:1000.00 | PEAKS DB |
| R.C(+57.02)C(+57.02)FVHDC(+57.02)C(+57.02)YEKVNDC(+57.02)NPK.T | Y | 77.12 | 2403.9163 | 18 | -1.8 | 802.3113 | 3 | 11.08 | 6 | F6:1966 | DaRuMH\_F5.raw |  |  |  |  |  | 3.9574E6 | 2.7357E5 |  |  |  | 3 | 0 | 0 | 0 | 0 | 0 | 2 | 1 | 0 | 0 | 0 | 59 | 76 | Carbamidomethylation | C1:Carbamidomethylation:1000.00;C2:Carbamidomethylation:1000.00;C7:Carbamidomethylation:1000.00;C8:Carbamidomethylation:1000.00;C15:Carbamidomethylation:1000.00 | PEAKS DB |
| R.KYPPSQC(+57.02)TGTEQC(+57.02) | Y | 75.37 | 1554.6443 | 13 | 0.8 | 778.3301 | 2 | 10.64 | 8 | F8:1605 | DaRuMH\_F7B.raw |  |  |  |  |  | 8.0352E7 | 6.6078E7 | 3.0497E7 | 2.265E6 |  | 5 | 0 | 0 | 0 | 0 | 0 | 2 | 1 | 1 | 1 | 0 | 125 | 137 | Carbamidomethylation | C7:Carbamidomethylation:1000.00;C13:Carbamidomethylation:1000.00 | PEAKS DB |
| Y.SYSFENGGIVC(+57.02)GDR.D | Y | 74.59 | 1559.6675 | 14 | 0.4 | 780.8413 | 2 | 12.04 | 6 | F6:2773 | DaRuMH\_F5.raw |  |  |  |  |  | 6.7382E7 | 1.3409E7 |  |  |  | 2 | 0 | 0 | 0 | 0 | 0 | 1 | 1 | 0 | 0 | 0 | 81 | 94 | Carbamidomethylation | C11:Carbamidomethylation:1000.00 | PEAKS DB |
| K.YRKYPPSQC(+57.02)TGTEQC(+57.02) | Y | 74.44 | 1873.8087 | 15 | 0.4 | 937.9120 | 2 | 10.67 | 6 | F6:1612 | DaRuMH\_F5.raw |  |  |  |  |  | 1.6114E6 |  |  |  |  | 1 | 0 | 0 | 0 | 0 | 0 | 1 | 0 | 0 | 0 | 0 | 123 | 137 | Carbamidomethylation | C9:Carbamidomethylation:1000.00;C15:Carbamidomethylation:1000.00 | PEAKS DB |
| Y.SYSFENGGIVC(+57.02)GDRDPC(+57.02)K.R | Y | 74.10 | 2059.8728 | 18 | -0.5 | 1030.9431 | 2 | 11.64 | 6 | F6:2444 | DaRuMH\_F5.raw |  |  |  |  |  | 9.2293E5 | 4.1189E6 |  |  |  | 3 | 0 | 0 | 0 | 0 | 0 | 1 | 2 | 0 | 0 | 0 | 81 | 98 | Carbamidomethylation | C11:Carbamidomethylation:1000.00;C17:Carbamidomethylation:1000.00 | PEAKS DB |
| T.YSYSFENGGIVC(+57.02)GDR.D | Y | 73.23 | 1722.7307 | 15 | 0.7 | 862.3732 | 2 | 12.57 | 6 | F6:3243 | DaRuMH\_F5.raw |  |  |  |  |  | 7.1186E5 |  |  |  |  | 1 | 0 | 0 | 0 | 0 | 0 | 1 | 0 | 0 | 0 | 0 | 80 | 94 | Carbamidomethylation | C12:Carbamidomethylation:1000.00 | PEAKS DB |
| S.SYSNYGC(+57.02)YC(+57.02)GWGGK.G | N | 71.65 | 1657.6289 | 14 | -3.2 | 829.8191 | 2 | 11.84 | 6 | F6:2618 | DaRuMH\_F5.raw |  |  |  |  |  | 1.228E6 | 1.8099E5 |  |  |  | 2 | 0 | 0 | 0 | 0 | 0 | 1 | 1 | 0 | 0 | 0 | 36 | 49 | Carbamidomethylation | C7:Carbamidomethylation:1000.00;C9:Carbamidomethylation:1000.00 | PEAKS DB |
| S.YSNYGC(+57.02)YC(+57.02)GWGGK.G | N | 69.63 | 1570.5969 | 13 | 0.8 | 786.3064 | 2 | 11.77 | 6 | F6:2569 | DaRuMH\_F5.raw |  |  |  |  |  | 2.3062E6 | 1.8554E6 |  |  |  | 2 | 0 | 0 | 0 | 0 | 0 | 1 | 1 | 0 | 0 | 0 | 37 | 49 | Carbamidomethylation | C6:Carbamidomethylation:1000.00;C8:Carbamidomethylation:1000.00 | PEAKS DB |
| Y.SNYGC(+57.02)YC(+57.02)GWGGK.G | N | 67.11 | 1407.5336 | 12 | 0.0 | 704.7740 | 2 | 11.60 | 8 | F8:2516 | DaRuMH\_F7B.raw |  |  |  |  |  | 5.0464E7 | 7.1321E7 | 1.0326E7 | 4.1576E5 |  | 4 | 0 | 0 | 0 | 0 | 0 | 1 | 1 | 1 | 1 | 0 | 38 | 49 | Carbamidomethylation | C5:Carbamidomethylation:1000.00;C7:Carbamidomethylation:1000.00 | PEAKS DB |
| N.YGC(+57.02)YC(+57.02)GWGGK.G | N | 66.85 | 1206.4586 | 10 | -0.3 | 604.2364 | 2 | 11.56 | 8 | F8:2453 | DaRuMH\_F7B.raw |  |  |  |  | 2.4701E5 | 2.1367E7 | 1.6469E7 | 7.7105E6 |  |  | 4 | 0 | 0 | 0 | 0 | 1 | 1 | 1 | 1 | 0 | 0 | 40 | 49 | Carbamidomethylation | C3:Carbamidomethylation:1000.00;C5:Carbamidomethylation:1000.00 | PEAKS DB |
| Y.SFENGGIVC(+57.02)GDR.D | Y | 66.72 | 1309.5720 | 12 | 2.2 | 655.7932 | 2 | 11.54 | 7 | F7:2331 | DaRuMH\_F7A.raw |  |  |  |  |  | 3.2286E7 | 9.5706E6 |  |  |  | 2 | 0 | 0 | 0 | 0 | 0 | 1 | 1 | 0 | 0 | 0 | 83 | 94 | Carbamidomethylation | C9:Carbamidomethylation:1000.00 | PEAKS DB |
| A.KNPLSSYSNYGC(+57.02)YC(+57.02)GWGGK.G | Y | 64.97 | 2196.9358 | 19 | -2.3 | 733.3158 | 3 | 11.92 | 7 | F7:2673 | DaRuMH\_F7A.raw |  |  |  |  |  |  | 1.6674E6 |  |  |  | 1 | 0 | 0 | 0 | 0 | 0 | 0 | 1 | 0 | 0 | 0 | 31 | 49 | Carbamidomethylation | C12:Carbamidomethylation:1000.00;C14:Carbamidomethylation:1000.00 | PEAKS DB |
| S.FENGGIVC(+57.02)GDR.D | Y | 64.51 | 1222.5400 | 11 | -0.4 | 612.2770 | 2 | 11.38 | 6 | F6:2208 | DaRuMH\_F5.raw |  |  |  |  |  | 8.3891E6 |  |  |  |  | 1 | 0 | 0 | 0 | 0 | 0 | 1 | 0 | 0 | 0 | 0 | 84 | 94 | Carbamidomethylation | C8:Carbamidomethylation:1000.00 | PEAKS DB |
| K.TATYSYSFENGGIVC(+57.02)GDRD.P | Y | 62.81 | 2110.8901 | 19 | 3.5 | 1056.4536 | 2 | 13.81 | 7 | F7:4180 | DaRuMH\_F7A.raw |  |  |  |  |  | 3.4196E6 | 2.5402E6 |  |  |  | 2 | 0 | 0 | 0 | 0 | 0 | 1 | 1 | 0 | 0 | 0 | 77 | 95 | Carbamidomethylation | C15:Carbamidomethylation:1000.00 | PEAKS DB |
| Y.SFENGGIVC(+57.02)GDRDPC(+57.02)KR.A | Y | 59.67 | 1965.8785 | 17 | 0.0 | 492.4769 | 4 | 11.04 | 6 | F6:1923 | DaRuMH\_F5.raw |  |  |  |  |  | 4.2108E5 | 3.5504E5 |  |  |  | 2 | 0 | 0 | 0 | 0 | 0 | 1 | 1 | 0 | 0 | 0 | 83 | 99 | Carbamidomethylation | C9:Carbamidomethylation:1000.00;C15:Carbamidomethylation:1000.00 | PEAKS DB |
| R.C(+57.02)C(+57.02)FVHDC(+57.02)C(+57.02)YEK.V | N | 58.97 | 1576.5568 | 11 | -1.4 | 789.2845 | 2 | 11.02 | 8 | F8:1930 | DaRuMH\_F7B.raw |  |  |  |  |  |  | 8.6121E7 | 5.3269E7 | 3.4024E6 |  | 5 | 0 | 0 | 0 | 0 | 0 | 0 | 1 | 2 | 2 | 0 | 59 | 69 | Carbamidomethylation | C1:Carbamidomethylation:1000.00;C2:Carbamidomethylation:1000.00;C7:Carbamidomethylation:1000.00;C8:Carbamidomethylation:1000.00 | PEAKS DB |
| Y.GC(+57.02)YC(+57.02)GWGGK.G | N | 58.54 | 1043.3953 | 9 | -0.4 | 522.7047 | 2 | 11.29 | 6 | F6:2113 | DaRuMH\_F5.raw |  |  |  |  |  | 2.1095E6 |  | 4.3981E6 |  |  | 2 | 0 | 0 | 0 | 0 | 0 | 1 | 0 | 1 | 0 | 0 | 41 | 49 | Carbamidomethylation | C2:Carbamidomethylation:1000.00;C4:Carbamidomethylation:1000.00 | PEAKS DB |
| S.FENGGIVC(+57.02)GDRDPC(+57.02)K.R | Y | 57.22 | 1722.7454 | 15 | 0.4 | 575.2560 | 3 | 11.15 | 6 | F6:2008 | DaRuMH\_F5.raw |  |  |  |  |  | 4.4906E5 |  |  |  |  | 1 | 0 | 0 | 0 | 0 | 0 | 1 | 0 | 0 | 0 | 0 | 84 | 98 | Carbamidomethylation | C8:Carbamidomethylation:1000.00;C14:Carbamidomethylation:1000.00 | PEAKS DB |
| R.VAATC(+57.02)FR.D | N | 55.30 | 823.4011 | 7 | -0.5 | 412.7076 | 2 | 11.06 | 9 | F9:1884 | DaRuMH\_F8.raw |  |  |  |  |  |  |  |  | 3.3394E5 |  | 1 | 0 | 0 | 0 | 0 | 0 | 0 | 0 | 0 | 1 | 0 | 107 | 113 | Carbamidomethylation | C5:Carbamidomethylation:1000.00 | PEAKS DB |
| K.NPLSSYSNYGC(+57.02)Y.C | Y | 54.22 | 1423.5714 | 12 | 1.2 | 712.7938 | 2 | 16.25 | 6 | F6:6470 | DaRuMH\_F5.raw |  |  |  |  |  | 9.5746E7 |  |  |  |  | 1 | 0 | 0 | 0 | 0 | 0 | 1 | 0 | 0 | 0 | 0 | 32 | 43 | Carbamidomethylation | C11:Carbamidomethylation:1000.00 | PEAKS DB |
| total 32 peptides |
| --- |

P18964|VSPA\_DABSI

back to list

  

| Protein Coverage
| Supporting Peptides
|

Protein Coverage:

Supporting Peptides:

| Peptide | Uniq | -10lgP | Mass | Length | ppm | m/z | z | RT | Fraction | Scan | Source File | Area F1 | Area F10 | Area F2 | Area F3 | Area F4 | Area F5 | Area F7A | Area F7B | Area F8 | Area F9 | #Feature | #Feature F1 | #Feature F10 | #Feature F2 | #Feature F3 | #Feature F4 | #Feature F5 | #Feature F7A | #Feature F7B | #Feature F8 | #Feature F9 | Start | End | PTM | AScore | Found By |
| --- | --- | --- | --- | --- | --- | --- | --- | --- | --- | --- | --- | --- | --- | --- | --- | --- | --- | --- | --- | --- | --- | --- | --- | --- | --- | --- | --- | --- | --- | --- | --- | --- | --- | --- | --- | --- | --- |
| K.ISTTEDTYPDVPHC(+57.02)TNIFIVK.H | N | 103.59 | 2449.1836 | 21 | 0.1 | 817.4019 | 3 | 16.78 | 9 | F9:5911 | DaRuMH\_F8.raw | 5.7748E6 |  |  |  |  |  |  |  | 1.0928E8 | 1.2647E6 | 4 | 2 | 0 | 0 | 0 | 0 | 0 | 0 | 0 | 1 | 1 | 128 | 148 | Carbamidomethylation | C14:Carbamidomethylation:1000.00 | PEAKS DB |
| K.WC(+57.02)EPLYPWVPADSR.T | N | 94.10 | 1774.8137 | 14 | -0.1 | 888.4141 | 2 | 30.09 | 9 | F9:11338 | DaRuMH\_F8.raw | 1.0151E7 |  |  |  |  |  |  | 2.4833E7 | 6.9222E7 | 6.7024E6 | 6 | 2 | 0 | 0 | 0 | 0 | 0 | 0 | 1 | 2 | 1 | 151 | 164 | Carbamidomethylation | C2:Carbamidomethylation:1000.00 | PEAKS DB |
| K.ISTTEDTYPDVPHC(+57.02)TN.I | N | 87.28 | 1848.7836 | 16 | -0.5 | 925.3986 | 2 | 11.61 | 8 | F8:2480 | DaRuMH\_F7B.raw | 3.4511E6 |  |  |  |  |  |  | 5.0224E6 |  | 8.3947E5 | 3 | 1 | 0 | 0 | 0 | 0 | 0 | 0 | 1 | 0 | 1 | 128 | 143 | Carbamidomethylation | C14:Carbamidomethylation:1000.00 | PEAKS DB |
| R.DTC(+57.02)HGDSGGPLIC(+57.02)NGQIQGIVAGGSEPC(+57.02)GQHLKPAVYTK.V | Y | 86.65 | 4077.9094 | 39 | -2.5 | 1020.4821 | 4 | 12.13 | 8 | F8:2998 | DaRuMH\_F7B.raw |  |  |  |  |  |  |  | 1.5075E7 | 2.5556E7 |  | 3 | 0 | 0 | 0 | 0 | 0 | 0 | 0 | 2 | 1 | 0 | 176 | 214 | Carbamidomethylation | C3:Carbamidomethylation:1000.00;C13:Carbamidomethylation:1000.00;C28:Carbamidomethylation:1000.00 | PEAKS DB |
| Y.TSTSSTIHC(+57.02)GGALINR.E | Y | 86.32 | 1673.8156 | 16 | 0.3 | 837.9153 | 2 | 11.15 | 8 | F8:2073 | DaRuMH\_F7B.raw | 1.2526E6 |  |  |  |  |  |  | 1.1579E6 | 1.0808E5 |  | 5 | 2 | 0 | 0 | 0 | 0 | 0 | 0 | 2 | 1 | 0 | 20 | 35 | Carbamidomethylation | C9:Carbamidomethylation:1000.00 | PEAKS DB |
| R.RPVTYSTHIAPVSLPSR.S | N | 86.13 | 1880.0267 | 17 | -0.1 | 471.0139 | 4 | 11.57 | 9 | F9:2371 | DaRuMH\_F8.raw | 6.7237E4 |  |  |  |  |  |  | 1.5386E6 | 2.9671E6 |  | 5 | 1 | 0 | 0 | 0 | 0 | 0 | 0 | 2 | 2 | 0 | 96 | 112 |  |  | PEAKS DB |
| L.YTSTSSTIHC(+57.02)GGALINR.E | Y | 84.49 | 1836.8788 | 17 | 0.1 | 919.4468 | 2 | 11.30 | 8 | F8:2209 | DaRuMH\_F7B.raw | 5.0434E5 |  |  |  |  |  |  | 1.8852E6 |  |  | 4 | 2 | 0 | 0 | 0 | 0 | 0 | 0 | 2 | 0 | 0 | 19 | 35 | Carbamidomethylation | C10:Carbamidomethylation:1000.00 | PEAKS DB |
| R.EWVLTAAHC(+57.02)DR.R | N | 82.04 | 1356.6245 | 11 | -1.5 | 679.3185 | 2 | 11.66 | 9 | F9:2448 | DaRuMH\_F8.raw | 1.3767E6 |  |  |  |  |  |  | 7.987E6 | 4.3899E7 | 4.43E5 | 8 | 2 | 0 | 0 | 0 | 0 | 0 | 0 | 2 | 2 | 2 | 36 | 46 | Carbamidomethylation | C9:Carbamidomethylation:1000.00 | PEAKS DB |
| VVGGDEC(+57.02)NINEHPFLVALYTSTSSTIHC(+57.02)GGALINR.E | Y | 81.55 | 3800.8250 | 35 | 0.9 | 951.2144 | 4 | 31.82 | 8 | F8:17442 | DaRuMH\_F7B.raw |  |  |  |  |  |  |  | 3.3776E7 |  |  | 1 | 0 | 0 | 0 | 0 | 0 | 0 | 0 | 1 | 0 | 0 | 1 | 35 | Carbamidomethylation | C7:Carbamidomethylation:1000.00;C28:Carbamidomethylation:1000.00 | PEAKS DB |
| VVGGDEC(+57.02)NINEHPF.L | Y | 78.99 | 1585.6831 | 14 | 0.4 | 793.8492 | 2 | 11.88 | 9 | F9:2661 | DaRuMH\_F8.raw | 4.0075E5 |  |  |  |  |  |  |  | 2.4721E6 |  | 2 | 1 | 0 | 0 | 0 | 0 | 0 | 0 | 0 | 1 | 0 | 1 | 14 | Carbamidomethylation | C7:Carbamidomethylation:1000.00 | PEAKS DB |
| R.DTC(+57.02)HGDSGGPLIC(+57.02)NGQIQGIVAGGSEPC(+57.02)GQHLK.P | Y | 77.85 | 3418.5452 | 33 | 1.6 | 855.6450 | 4 | 12.20 | 8 | F8:3054 | DaRuMH\_F7B.raw | 2.3134E6 |  |  |  |  |  |  | 8.5753E6 | 3.4048E7 |  | 4 | 1 | 0 | 0 | 0 | 0 | 0 | 0 | 1 | 2 | 0 | 176 | 208 | Carbamidomethylation | C3:Carbamidomethylation:1000.00;C13:Carbamidomethylation:1000.00;C28:Carbamidomethylation:1000.00 | PEAKS DB |
| K.ISTTEDTYPDVPHC(+57.02)TNIF.I | N | 70.96 | 2108.9360 | 18 | 0.5 | 1055.4758 | 2 | 19.26 | 9 | F9:7487 | DaRuMH\_F8.raw |  |  |  |  |  |  |  |  | 2.0971E7 |  | 1 | 0 | 0 | 0 | 0 | 0 | 0 | 0 | 0 | 1 | 0 | 128 | 145 | Carbamidomethylation | C14:Carbamidomethylation:1000.00 | PEAKS DB |
| Y.STHIAPVSLPSR.S | N | 70.80 | 1263.6935 | 12 | 0.6 | 632.8544 | 2 | 11.43 | 8 | F8:2336 | DaRuMH\_F7B.raw | 2.7685E6 |  |  |  |  |  |  | 5.3843E6 | 8.8922E6 |  | 5 | 2 | 0 | 0 | 0 | 0 | 0 | 0 | 2 | 1 | 0 | 101 | 112 |  |  | PEAKS DB |
| K.ISTTEDTYPDVPH.C | N | 67.75 | 1473.6624 | 13 | 1.1 | 737.8392 | 2 | 11.75 | 9 | F9:2531 | DaRuMH\_F8.raw |  |  |  |  |  |  |  |  | 1.9511E6 |  | 1 | 0 | 0 | 0 | 0 | 0 | 0 | 0 | 0 | 1 | 0 | 128 | 140 |  |  | PEAKS DB |
| VVGGDEC(+57.02)NINEHPFLVALYTSTSSTIH.C | Y | 65.61 | 2959.4021 | 27 | 0.7 | 987.4753 | 3 | 30.39 | 8 | F8:16301 | DaRuMH\_F7B.raw |  |  |  |  |  |  |  | 8.3195E6 |  |  | 1 | 0 | 0 | 0 | 0 | 0 | 0 | 0 | 1 | 0 | 0 | 1 | 27 | Carbamidomethylation | C7:Carbamidomethylation:1000.00 | PEAKS DB |
| F.LVALYTSTSSTIHC(+57.02)GGALINR.E | Y | 65.12 | 2233.1526 | 21 | 1.5 | 745.3926 | 3 | 12.42 | 8 | F8:3289 | DaRuMH\_F7B.raw |  |  |  |  |  |  |  | 3.9831E5 |  |  | 1 | 0 | 0 | 0 | 0 | 0 | 0 | 0 | 1 | 0 | 0 | 15 | 35 | Carbamidomethylation | C14:Carbamidomethylation:1000.00 | PEAKS DB |
| VVGGDEC(+57.02)NINEHPFLVALY.T | Y | 64.39 | 2145.0200 | 19 | -0.2 | 1073.5171 | 2 | 34.44 | 9 | F9:13391 | DaRuMH\_F8.raw |  |  |  |  |  |  |  |  | 7.3848E7 |  | 1 | 0 | 0 | 0 | 0 | 0 | 0 | 0 | 0 | 1 | 0 | 1 | 19 | Carbamidomethylation | C7:Carbamidomethylation:1000.00 | PEAKS DB |
| S.STIHC(+57.02)GGALINR.E | Y | 63.01 | 1297.6561 | 12 | -0.6 | 433.5591 | 3 | 11.10 | 1 | F1:1976 | DaRuMH\_F1.raw | 7.3024E4 |  |  |  |  |  |  |  |  |  | 1 | 1 | 0 | 0 | 0 | 0 | 0 | 0 | 0 | 0 | 0 | 24 | 35 | Carbamidomethylation | C5:Carbamidomethylation:1000.00 | PEAKS DB |
| W.VLTAAHC(+57.02)DR.R | N | 62.64 | 1041.5026 | 9 | -1.2 | 521.7579 | 2 | 11.66 | 9 | F9:2450 | DaRuMH\_F8.raw |  |  |  |  |  |  |  |  | 1.2187E6 |  | 1 | 0 | 0 | 0 | 0 | 0 | 0 | 0 | 0 | 1 | 0 | 38 | 46 | Carbamidomethylation | C7:Carbamidomethylation:1000.00 | PEAKS DB |
| K.YFC(+57.02)LNTK.F | N | 62.64 | 944.4426 | 7 | -0.7 | 473.2282 | 2 | 11.72 | 1 | F1:2582 | DaRuMH\_F1.raw | 5.6715E5 |  |  |  |  |  |  | 8.6564E5 | 2.9998E6 |  | 3 | 1 | 0 | 0 | 0 | 0 | 0 | 0 | 1 | 1 | 0 | 74 | 80 | Carbamidomethylation | C3:Carbamidomethylation:1000.00 | PEAKS DB |
| VVGGDEC(+57.02)NINEHPFLVAL.Y | Y | 61.71 | 1981.9567 | 18 | 0.7 | 991.9863 | 2 | 30.46 | 9 | F9:11721 | DaRuMH\_F8.raw |  |  |  |  |  |  |  |  | 2.312E7 |  | 1 | 0 | 0 | 0 | 0 | 0 | 0 | 0 | 0 | 1 | 0 | 1 | 18 | Carbamidomethylation | C7:Carbamidomethylation:1000.00 | PEAKS DB |
| R.DTC(+57.02)HGDSGGPLIC(+57.02)NGQIQGIVAGGSEPC(+57.02)GQHLKPA.V | Y | 61.24 | 3586.6350 | 35 | 0.3 | 897.6663 | 4 | 12.33 | 9 | F9:3075 | DaRuMH\_F8.raw |  |  |  |  |  |  |  |  | 8.6092E5 |  | 1 | 0 | 0 | 0 | 0 | 0 | 0 | 0 | 0 | 1 | 0 | 176 | 210 | Carbamidomethylation | C3:Carbamidomethylation:1000.00;C13:Carbamidomethylation:1000.00;C28:Carbamidomethylation:1000.00 | PEAKS DB |
| V.TYSTHIAPVSLPSR.S | N | 61.16 | 1527.8046 | 14 | -2.7 | 510.2741 | 3 | 11.71 | 9 | F9:2491 | DaRuMH\_F8.raw |  |  |  |  |  |  |  |  | 0 |  | 0 | 0 | 0 | 0 | 0 | 0 | 0 | 0 | 0 | 0 | 0 | 99 | 112 |  |  | PEAKS DB |
| R.PVTYSTHIAPVSLPSR.S | N | 60.92 | 1723.9257 | 16 | -0.6 | 575.6488 | 3 | 11.88 | 9 | F9:2672 | DaRuMH\_F8.raw |  |  |  |  |  |  |  |  | 8.827E4 |  | 1 | 0 | 0 | 0 | 0 | 0 | 0 | 0 | 0 | 1 | 0 | 97 | 112 |  |  | PEAKS DB |
| K.VFDYNNWIQNIIAGNR.T | Y | 58.40 | 1935.9591 | 16 | -0.1 | 646.3269 | 3 | 49.10 | 9 | F9:17867 | DaRuMH\_F8.raw |  |  |  |  |  |  |  | 4.0747E5 | 9.5515E4 |  | 2 | 0 | 0 | 0 | 0 | 0 | 0 | 0 | 1 | 1 | 0 | 215 | 230 |  |  | PEAKS DB |
| K.FPNGLDK.D | N | 57.14 | 789.4021 | 7 | -1.4 | 395.7078 | 2 | 11.37 | 8 | F8:2253 | DaRuMH\_F7B.raw | 7.7704E5 |  |  |  |  |  |  | 1.3591E6 |  |  | 2 | 1 | 0 | 0 | 0 | 0 | 0 | 0 | 1 | 0 | 0 | 81 | 87 |  |  | PEAKS DB |
| H.C(+57.02)TNIFIVK.H | N | 56.67 | 993.5317 | 8 | 0.3 | 497.7733 | 2 | 12.59 | 9 | F9:3259 | DaRuMH\_F8.raw |  |  |  |  |  |  |  |  | 6.9308E5 |  | 1 | 0 | 0 | 0 | 0 | 0 | 0 | 0 | 0 | 1 | 0 | 141 | 148 | Carbamidomethylation | C1:Carbamidomethylation:1000.00 | PEAKS DB |
| S.TSSTIHC(+57.02)GGALINR.E | Y | 55.22 | 1485.7358 | 14 | 0.1 | 496.2526 | 3 | 11.06 | 8 | F8:1983 | DaRuMH\_F7B.raw |  |  |  |  |  |  |  | 0 |  |  | 0 | 0 | 0 | 0 | 0 | 0 | 0 | 0 | 0 | 0 | 0 | 22 | 35 | Carbamidomethylation | C7:Carbamidomethylation:1000.00 | PEAKS DB |
| R.TLC(+57.02)AGILK.G | N | 54.02 | 874.4946 | 8 | -0.7 | 438.2543 | 2 | 11.80 | 8 | F8:2668 | DaRuMH\_F7B.raw |  |  |  |  |  |  |  | 6.6304E6 |  |  | 1 | 0 | 0 | 0 | 0 | 0 | 0 | 0 | 1 | 0 | 0 | 165 | 172 | Carbamidomethylation | C3:Carbamidomethylation:1000.00 | PEAKS DB |
| R.EWVLTAAH.C | N | 53.60 | 925.4658 | 8 | 0.4 | 463.7404 | 2 | 13.15 | 8 | F8:3914 | DaRuMH\_F7B.raw |  |  |  |  |  |  |  | 3.0986E5 |  |  | 1 | 0 | 0 | 0 | 0 | 0 | 0 | 0 | 1 | 0 | 0 | 36 | 43 |  |  | PEAKS DB |
| total 30 peptides |
| --- |

P04264|K2C1\_HUMAN

back to list

  

| Protein Coverage
| Supporting Peptides
|

Protein Coverage:

Supporting Peptides:

| Peptide | Uniq | -10lgP | Mass | Length | ppm | m/z | z | RT | Fraction | Scan | Source File | Area F1 | Area F10 | Area F2 | Area F3 | Area F4 | Area F5 | Area F7A | Area F7B | Area F8 | Area F9 | #Feature | #Feature F1 | #Feature F10 | #Feature F2 | #Feature F3 | #Feature F4 | #Feature F5 | #Feature F7A | #Feature F7B | #Feature F8 | #Feature F9 | Start | End | PTM | AScore | Found By |
| --- | --- | --- | --- | --- | --- | --- | --- | --- | --- | --- | --- | --- | --- | --- | --- | --- | --- | --- | --- | --- | --- | --- | --- | --- | --- | --- | --- | --- | --- | --- | --- | --- | --- | --- | --- | --- | --- |
| R.FSSC(+57.02)GGGGGSFGAGGGFGSR.S | Y | 106.29 | 1764.7274 | 20 | -0.5 | 883.3705 | 2 | 11.74 | 1 | F1:2602 | DaRuMH\_F1.raw | 3.1365E6 | 9.3824E4 |  | 9.6602E4 | 4.3821E5 |  |  |  | 1.0089E5 | 8.8013E5 | 6 | 1 | 1 | 0 | 1 | 1 | 0 | 0 | 0 | 1 | 1 | 46 | 65 | Carbamidomethylation | C4:Carbamidomethylation:1000.00 | PEAKS DB |
| K.QISNLQQSISDAEQR.G | Y | 105.71 | 1715.8438 | 15 | 0.9 | 858.9299 | 2 | 11.88 | 1 | F1:2747 | DaRuMH\_F1.raw | 1.4974E6 | 1.9122E5 |  | 1.7318E5 | 1.6638E5 |  | 1.1501E5 |  |  | 6.7879E5 | 8 | 2 | 1 | 0 | 1 | 1 | 0 | 1 | 0 | 0 | 2 | 418 | 432 |  |  | PEAKS DB |
| R.GGGGGGYGSGGSSYGSGGGSYGSGGGGGGGR.G | Y | 100.94 | 2382.9446 | 31 | 0.3 | 1192.4800 | 2 | 11.03 | 1 | F1:1907 | DaRuMH\_F1.raw | 5.3034E5 |  |  |  | 4.1713E5 |  |  |  | 7.7456E4 | 3.5961E5 | 4 | 1 | 0 | 0 | 0 | 1 | 0 | 0 | 0 | 1 | 1 | 519 | 549 |  |  | PEAKS DB |
| R.SGGGFSSGSAGIINYQR.R | Y | 95.52 | 1656.7855 | 17 | 1.3 | 829.4011 | 2 | 11.91 | 1 | F1:2775 | DaRuMH\_F1.raw | 2.0916E5 |  |  |  | 1.1975E5 |  |  |  |  |  | 2 | 1 | 0 | 0 | 0 | 1 | 0 | 0 | 0 | 0 | 0 | 13 | 29 |  |  | PEAKS DB |
| R.THNLEPYFESFINNLR.R | Y | 94.73 | 1992.9694 | 16 | 1.5 | 665.3314 | 3 | 34.38 | 10 | F10:17198 | DaRuMH\_F9.raw |  | 6.7835E5 |  |  | 6.0678E5 | 1.0291E6 | 7.8871E5 |  | 3.2194E5 | 4.3986E6 | 8 | 0 | 1 | 0 | 0 | 1 | 2 | 1 | 0 | 1 | 2 | 224 | 239 |  |  | PEAKS DB |
| R.FLEQQNQVLQTK.W | N | 86.18 | 1474.7780 | 12 | -1.5 | 738.3951 | 2 | 11.69 | 10 | F10:2507 | DaRuMH\_F9.raw | 1.2606E7 | 4.3269E5 | 3.8807E5 | 1.3371E6 | 1.6646E6 | 5.0153E5 | 4.6914E5 | 3.9811E5 | 4.8981E5 | 3.5935E6 | 10 | 1 | 1 | 1 | 1 | 1 | 1 | 1 | 1 | 1 | 1 | 200 | 211 |  |  | PEAKS DB |
| K.WELLQQVDTSTR.T | Y | 83.93 | 1474.7416 | 12 | 0.6 | 738.3785 | 2 | 17.53 | 10 | F10:7474 | DaRuMH\_F9.raw | 6.4781E6 |  | 1.0178E5 | 3.5697E5 | 1.1847E6 |  |  | 1.9304E5 |  | 4.8539E6 | 6 | 1 | 0 | 1 | 1 | 1 | 0 | 0 | 1 | 0 | 1 | 212 | 223 |  |  | PEAKS DB |
| R.TNAENEFVTIK.K | Y | 80.23 | 1264.6299 | 11 | -2.8 | 633.3204 | 2 | 11.84 | 10 | F10:2656 | DaRuMH\_F9.raw | 7.1834E6 | 2.0251E5 | 1.9451E5 | 5.3068E5 | 4.8282E5 | 3.1956E5 | 1.5344E5 |  | 1.3329E5 | 1.5592E6 | 9 | 1 | 1 | 1 | 1 | 1 | 1 | 1 | 0 | 1 | 1 | 278 | 288 |  |  | PEAKS DB |
| K.LNDLEDALQQAK.E | Y | 79.39 | 1356.6885 | 12 | 0.5 | 679.3519 | 2 | 12.87 | 3 | F3:3199 | DaRuMH\_F2.raw | 1.2645E7 | 6.0554E5 | 2.8858E5 | 9.0288E5 | 1.418E6 | 8.9167E5 | 4.4644E5 | 5.3288E5 |  | 4.9621E6 | 9 | 1 | 1 | 1 | 1 | 1 | 1 | 1 | 1 | 0 | 1 | 444 | 455 |  |  | PEAKS DB |
| K.SKAEAESLYQSK.Y | Y | 75.82 | 1339.6619 | 12 | 0.6 | 670.8386 | 2 | 10.77 | 1 | F1:1662 | DaRuMH\_F1.raw | 1.1536E6 |  |  |  |  |  |  |  |  |  | 2 | 2 | 0 | 0 | 0 | 0 | 0 | 0 | 0 | 0 | 0 | 365 | 376 |  |  | PEAKS DB |
| K.LALDLEIATYR.T | N | 75.63 | 1276.7026 | 11 | 0.1 | 639.3586 | 2 | 24.59 | 6 | F6:9907 | DaRuMH\_F5.raw |  | 4.7723E5 |  |  | 4.4526E5 | 7.6125E5 | 5.2926E5 | 4.3012E5 | 2.5261E5 | 3.3926E6 | 7 | 0 | 1 | 0 | 0 | 1 | 1 | 1 | 1 | 1 | 1 | 473 | 483 |  |  | PEAKS DB |
| K.AQYEDIAQK.S | N | 73.35 | 1064.5138 | 9 | 0.7 | 533.2645 | 2 | 11.08 | 10 | F10:1930 | DaRuMH\_F9.raw | 1.0569E6 | 5.332E4 | 1.8455E4 | 2.2136E5 | 5.8087E5 |  |  |  | 2.5639E4 | 6.1011E5 | 7 | 1 | 1 | 1 | 1 | 1 | 0 | 0 | 0 | 1 | 1 | 356 | 364 |  |  | PEAKS DB |
| K.AEAESLYQSK.Y | Y | 73.21 | 1124.5349 | 10 | -0.6 | 563.2744 | 2 | 11.16 | 1 | F1:2033 | DaRuMH\_F1.raw | 9.4459E5 |  |  |  | 3.8714E5 |  |  |  |  | 6.4398E5 | 3 | 1 | 0 | 0 | 0 | 1 | 0 | 0 | 0 | 0 | 1 | 367 | 376 |  |  | PEAKS DB |
| N.QSLLQPLNVEIDPEIQK.V | Y | 73.16 | 1963.0625 | 17 | 0.8 | 655.3619 | 3 | 29.11 | 1 | F1:8328 | DaRuMH\_F1.raw | 2.5374E5 |  |  |  |  |  |  |  |  |  | 1 | 1 | 0 | 0 | 0 | 0 | 0 | 0 | 0 | 0 | 0 | 159 | 175 |  |  | PEAKS DB |
| K.SLNNQFASFIDK.V | Y | 72.46 | 1382.6830 | 12 | 0.7 | 692.3492 | 2 | 17.51 | 1 | F1:5544 | DaRuMH\_F1.raw | 8.1352E6 | 6.1795E5 | 2.0351E5 |  | 1.1413E6 | 5.9716E5 | 5.4661E5 | 4.1925E5 | 3.3097E5 | 3.8291E6 | 9 | 1 | 1 | 1 | 0 | 1 | 1 | 1 | 1 | 1 | 1 | 186 | 197 |  |  | PEAKS DB |
| N.VSVSVSTSHTTISGGGSR.G | Y | 67.62 | 1717.8595 | 18 | -0.1 | 573.6271 | 3 | 11.15 | 10 | F10:1996 | DaRuMH\_F9.raw |  |  |  |  |  |  |  |  |  | 1.2715E5 | 1 | 0 | 0 | 0 | 0 | 0 | 0 | 0 | 0 | 0 | 1 | 501 | 518 |  |  | PEAKS DB |
| R.SLDLDSIIAEVK.A | N | 66.46 | 1301.7078 | 12 | 0.3 | 651.8613 | 2 | 36.20 | 1 | F1:10077 | DaRuMH\_F1.raw | 6.9315E6 |  | 1.0092E5 | 4.0144E5 | 3.262E6 | 1.3149E6 | 1.0222E6 | 9.6817E5 | 9.9203E5 | 8.0758E6 | 11 | 2 | 0 | 1 | 1 | 1 | 1 | 1 | 1 | 1 | 2 | 344 | 355 |  |  | PEAKS DB |
| L.NVEIDPEIQK.V | N | 65.48 | 1183.6084 | 10 | -1.3 | 592.8107 | 2 | 11.69 | 10 | F10:2508 | DaRuMH\_F9.raw |  |  | 4.5616E4 |  | 3.0057E5 | 1.0887E5 |  |  |  | 8.985E5 | 4 | 0 | 0 | 1 | 0 | 1 | 1 | 0 | 0 | 0 | 1 | 166 | 175 |  |  | PEAKS DB |
| K.YEELQITAGR.H | N | 64.61 | 1178.5931 | 10 | -4.0 | 590.3015 | 2 | 11.79 | 9 | F9:2579 | DaRuMH\_F8.raw | 0 |  | 9.1945E4 | 5.3318E5 | 1.5834E6 | 2.2943E5 | 9.1824E4 |  | 2.0072E5 |  | 6 | 0 | 0 | 1 | 1 | 1 | 1 | 1 | 0 | 1 | 0 | 377 | 386 |  |  | PEAKS DB |
| R.DYQELMNTK.L | N | 64.24 | 1140.5121 | 9 | -0.5 | 571.2631 | 2 | 11.82 | 1 | F1:2685 | DaRuMH\_F1.raw | 6.8408E5 |  |  |  |  |  |  |  |  |  | 1 | 1 | 0 | 0 | 0 | 0 | 0 | 0 | 0 | 0 | 0 | 464 | 472 |  |  | PEAKS DB |
| R.TNAENEFVTIKK.D | Y | 64.19 | 1392.7249 | 12 | -0.8 | 465.2485 | 3 | 11.32 | 5 | F5:2134 | DaRuMH\_F4.raw |  |  |  |  | 1.0926E5 |  |  |  |  |  | 1 | 0 | 0 | 0 | 0 | 1 | 0 | 0 | 0 | 0 | 0 | 278 | 289 |  |  | PEAKS DB |
| R.TLLEGEESR.M | Y | 60.04 | 1032.5087 | 9 | 0.1 | 517.2617 | 2 | 11.32 | 1 | F1:2180 | DaRuMH\_F1.raw | 7.0108E5 | 8.9009E4 |  | 2.9581E5 | 3.8599E5 |  |  |  |  | 5.0837E5 | 5 | 1 | 1 | 0 | 1 | 1 | 0 | 0 | 0 | 0 | 1 | 484 | 492 |  |  | PEAKS DB |
| K.NMQDM(+15.99)VEDYR.N | Y | 58.82 | 1315.5173 | 10 | 0.7 | 658.7664 | 2 | 11.47 | 1 | F1:2320 | DaRuMH\_F1.raw | 2.057E5 |  |  |  |  |  |  |  |  |  | 1 | 1 | 0 | 0 | 0 | 0 | 0 | 0 | 0 | 0 | 0 | 258 | 267 | Oxidation (M) | M5:Oxidation (M):91.37 | PEAKS DB |
| K.SKAEAESLYQSKYEELQITAGR.H | Y | 58.22 | 2500.2444 | 22 | 0.7 | 626.0688 | 4 | 11.77 | 5 | F5:2579 | DaRuMH\_F4.raw |  |  |  |  | 1.3292E5 |  |  |  |  |  | 1 | 0 | 0 | 0 | 0 | 1 | 0 | 0 | 0 | 0 | 0 | 365 | 386 |  |  | PEAKS DB |
| R.THNLEPYFESF.I | Y | 57.44 | 1382.6143 | 11 | 0.2 | 692.3145 | 2 | 20.48 | 9 | F9:8065 | DaRuMH\_F8.raw |  |  |  |  |  |  |  |  | 9.2428E4 | 2.7653E6 | 2 | 0 | 0 | 0 | 0 | 0 | 0 | 0 | 0 | 1 | 1 | 224 | 234 |  |  | PEAKS DB |
| R.SLVNLGGSK.S | Y | 56.88 | 873.4919 | 9 | -0.7 | 437.7529 | 2 | 11.58 | 5 | F5:2406 | DaRuMH\_F4.raw |  |  |  |  | 3.8737E5 |  |  |  |  |  | 1 | 0 | 0 | 0 | 0 | 1 | 0 | 0 | 0 | 0 | 0 | 66 | 74 |  |  | PEAKS DB |
| R.NKYEDEINKR.T | N | 56.06 | 1307.6470 | 10 | -2.2 | 436.8886 | 3 | 10.74 | 1 | F1:1618 | DaRuMH\_F1.raw | 5.4056E6 |  |  |  |  |  |  |  |  |  | 1 | 1 | 0 | 0 | 0 | 0 | 0 | 0 | 0 | 0 | 0 | 268 | 277 |  |  | PEAKS DB |
| K.NM(+15.99)QDM(+15.99)VEDYR.N | Y | 54.86 | 1331.5122 | 10 | 6.2 | 666.7637 | 2 | 11.03 | 4 | F4:1878 | DaRuMH\_F3.raw |  |  |  | 9.4073E4 |  |  |  |  |  |  | 1 | 0 | 0 | 0 | 1 | 0 | 0 | 0 | 0 | 0 | 0 | 258 | 267 | Oxidation (M) | M2:Oxidation (M):1000.00;M5:Oxidation (M):1000.00 | PEAKS DB |
| total 28 peptides |
| --- |

P18965|VSPG\_DABSI

back to list

  

| Protein Coverage
| Supporting Peptides
|

Protein Coverage:

Supporting Peptides:

| Peptide | Uniq | -10lgP | Mass | Length | ppm | m/z | z | RT | Fraction | Scan | Source File | Area F1 | Area F10 | Area F2 | Area F3 | Area F4 | Area F5 | Area F7A | Area F7B | Area F8 | Area F9 | #Feature | #Feature F1 | #Feature F10 | #Feature F2 | #Feature F3 | #Feature F4 | #Feature F5 | #Feature F7A | #Feature F7B | #Feature F8 | #Feature F9 | Start | End | PTM | AScore | Found By |
| --- | --- | --- | --- | --- | --- | --- | --- | --- | --- | --- | --- | --- | --- | --- | --- | --- | --- | --- | --- | --- | --- | --- | --- | --- | --- | --- | --- | --- | --- | --- | --- | --- | --- | --- | --- | --- | --- |
| L.YTSASSTIHC(+57.02)AGALINR.E | Y | 105.75 | 1820.8839 | 17 | 0.9 | 911.4500 | 2 | 11.64 | 9 | F9:2429 | DaRuMH\_F8.raw |  |  |  |  |  |  |  |  | 5.0803E6 |  | 2 | 0 | 0 | 0 | 0 | 0 | 0 | 0 | 0 | 2 | 0 | 43 | 59 | Carbamidomethylation | C10:Carbamidomethylation:1000.00 | PEAKS DB |
| K.ISTTEDTYPDVPHC(+57.02)TNIFIVK.H | N | 103.59 | 2449.1836 | 21 | 0.1 | 817.4019 | 3 | 16.78 | 9 | F9:5911 | DaRuMH\_F8.raw | 5.7748E6 |  |  |  |  |  |  |  | 1.0928E8 | 1.2647E6 | 4 | 2 | 0 | 0 | 0 | 0 | 0 | 0 | 0 | 1 | 1 | 152 | 172 | Carbamidomethylation | C14:Carbamidomethylation:1000.00 | PEAKS DB |
| Y.TSASSTIHC(+57.02)AGALINR.E | Y | 100.14 | 1657.8206 | 16 | 0.8 | 829.9182 | 2 | 11.51 | 9 | F9:2302 | DaRuMH\_F8.raw |  |  |  |  |  |  |  | 4.8607E5 | 1.4679E7 |  | 3 | 0 | 0 | 0 | 0 | 0 | 0 | 0 | 1 | 2 | 0 | 44 | 59 | Carbamidomethylation | C9:Carbamidomethylation:1000.00 | PEAKS DB |
| K.WC(+57.02)EPLYPWVPADSR.T | N | 94.10 | 1774.8137 | 14 | -0.1 | 888.4141 | 2 | 30.09 | 9 | F9:11338 | DaRuMH\_F8.raw | 1.0151E7 |  |  |  |  |  |  | 2.4833E7 | 6.9222E7 | 6.7024E6 | 6 | 2 | 0 | 0 | 0 | 0 | 0 | 0 | 1 | 2 | 1 | 175 | 188 | Carbamidomethylation | C2:Carbamidomethylation:1000.00 | PEAKS DB |
| K.ISTTEDTYPDVPHC(+57.02)TN.I | N | 87.28 | 1848.7836 | 16 | -0.5 | 925.3986 | 2 | 11.61 | 8 | F8:2480 | DaRuMH\_F7B.raw | 3.4511E6 |  |  |  |  |  |  | 5.0224E6 |  | 8.3947E5 | 3 | 1 | 0 | 0 | 0 | 0 | 0 | 0 | 1 | 0 | 1 | 152 | 167 | Carbamidomethylation | C14:Carbamidomethylation:1000.00 | PEAKS DB |
| R.RPVTYSTHIAPVSLPSR.S | N | 86.13 | 1880.0267 | 17 | -0.1 | 471.0139 | 4 | 11.57 | 9 | F9:2371 | DaRuMH\_F8.raw | 6.7237E4 |  |  |  |  |  |  | 1.5386E6 | 2.9671E6 |  | 5 | 1 | 0 | 0 | 0 | 0 | 0 | 0 | 2 | 2 | 0 | 120 | 136 |  |  | PEAKS DB |
| T.SASSTIHC(+57.02)AGALINR.E | Y | 86.01 | 1556.7729 | 15 | 0.9 | 779.3945 | 2 | 11.44 | 9 | F9:2244 | DaRuMH\_F8.raw |  |  |  |  |  |  |  |  | 8.1495E5 |  | 2 | 0 | 0 | 0 | 0 | 0 | 0 | 0 | 0 | 2 | 0 | 45 | 59 | Carbamidomethylation | C8:Carbamidomethylation:1000.00 | PEAKS DB |
| R.EWVLTAAHC(+57.02)DR.R | N | 82.04 | 1356.6245 | 11 | -1.5 | 679.3185 | 2 | 11.66 | 9 | F9:2448 | DaRuMH\_F8.raw | 1.3767E6 |  |  |  |  |  |  | 7.987E6 | 4.3899E7 | 4.43E5 | 8 | 2 | 0 | 0 | 0 | 0 | 0 | 0 | 2 | 2 | 2 | 60 | 70 | Carbamidomethylation | C9:Carbamidomethylation:1000.00 | PEAKS DB |
| K.ISTTEDTYPDVPHC(+57.02)TNIF.I | N | 70.96 | 2108.9360 | 18 | 0.5 | 1055.4758 | 2 | 19.26 | 9 | F9:7487 | DaRuMH\_F8.raw |  |  |  |  |  |  |  |  | 2.0971E7 |  | 1 | 0 | 0 | 0 | 0 | 0 | 0 | 0 | 0 | 1 | 0 | 152 | 169 | Carbamidomethylation | C14:Carbamidomethylation:1000.00 | PEAKS DB |
| Y.STHIAPVSLPSR.S | N | 70.80 | 1263.6935 | 12 | 0.6 | 632.8544 | 2 | 11.43 | 8 | F8:2336 | DaRuMH\_F7B.raw | 2.7685E6 |  |  |  |  |  |  | 5.3843E6 | 8.8922E6 |  | 5 | 2 | 0 | 0 | 0 | 0 | 0 | 0 | 2 | 1 | 0 | 125 | 136 |  |  | PEAKS DB |
| L.VVGGDEC(+57.02)NINEHPFLVALYTSASSTIHC(+57.02)AGALINR.E | Y | 70.26 | 3784.8301 | 35 | 0.4 | 947.2152 | 4 | 45.27 | 8 | F8:23652 | DaRuMH\_F7B.raw |  |  |  |  |  |  |  | 1.138E7 |  |  | 1 | 0 | 0 | 0 | 0 | 0 | 0 | 0 | 1 | 0 | 0 | 25 | 59 | Carbamidomethylation | C7:Carbamidomethylation:1000.00;C28:Carbamidomethylation:1000.00 | PEAKS DB |
| S.ASSTIHC(+57.02)AGALINR.E | Y | 69.54 | 1469.7408 | 14 | 0.8 | 490.9213 | 3 | 11.26 | 9 | F9:2090 | DaRuMH\_F8.raw |  |  |  |  |  |  |  |  | 4.0635E5 |  | 1 | 0 | 0 | 0 | 0 | 0 | 0 | 0 | 0 | 1 | 0 | 46 | 59 | Carbamidomethylation | C7:Carbamidomethylation:1000.00 | PEAKS DB |
| K.ISTTEDTYPDVPH.C | N | 67.75 | 1473.6624 | 13 | 1.1 | 737.8392 | 2 | 11.75 | 9 | F9:2531 | DaRuMH\_F8.raw |  |  |  |  |  |  |  |  | 1.9511E6 |  | 1 | 0 | 0 | 0 | 0 | 0 | 0 | 0 | 0 | 1 | 0 | 152 | 164 |  |  | PEAKS DB |
| L.VALYTSASSTIHC(+57.02)AGALINR.E | Y | 67.49 | 2104.0735 | 20 | 0.9 | 702.3657 | 3 | 13.53 | 9 | F9:3938 | DaRuMH\_F8.raw |  |  |  |  |  |  |  |  | 2.1605E5 |  | 1 | 0 | 0 | 0 | 0 | 0 | 0 | 0 | 0 | 1 | 0 | 40 | 59 | Carbamidomethylation | C13:Carbamidomethylation:1000.00 | PEAKS DB |
| A.SSTIHC(+57.02)AGALINR.E | Y | 66.93 | 1398.7037 | 13 | 0.5 | 467.2421 | 3 | 11.24 | 9 | F9:2049 | DaRuMH\_F8.raw |  |  |  |  |  |  |  |  | 1.1239E5 |  | 1 | 0 | 0 | 0 | 0 | 0 | 0 | 0 | 0 | 1 | 0 | 47 | 59 | Carbamidomethylation | C6:Carbamidomethylation:1000.00 | PEAKS DB |
| S.STIHC(+57.02)AGALINR.E | Y | 66.44 | 1311.6718 | 12 | 0.7 | 656.8436 | 2 | 11.19 | 9 | F9:2013 | DaRuMH\_F8.raw |  |  |  |  |  |  |  |  | 5.365E4 |  | 1 | 0 | 0 | 0 | 0 | 0 | 0 | 0 | 0 | 1 | 0 | 48 | 59 | Carbamidomethylation | C5:Carbamidomethylation:1000.00 | PEAKS DB |
| W.VLTAAHC(+57.02)DR.R | N | 62.64 | 1041.5026 | 9 | -1.2 | 521.7579 | 2 | 11.66 | 9 | F9:2450 | DaRuMH\_F8.raw |  |  |  |  |  |  |  |  | 1.2187E6 |  | 1 | 0 | 0 | 0 | 0 | 0 | 0 | 0 | 0 | 1 | 0 | 62 | 70 | Carbamidomethylation | C7:Carbamidomethylation:1000.00 | PEAKS DB |
| K.YFC(+57.02)LNTK.F | N | 62.64 | 944.4426 | 7 | -0.7 | 473.2282 | 2 | 11.72 | 1 | F1:2582 | DaRuMH\_F1.raw | 5.6715E5 |  |  |  |  |  |  | 8.6564E5 | 2.9998E6 |  | 3 | 1 | 0 | 0 | 0 | 0 | 0 | 0 | 1 | 1 | 0 | 98 | 104 | Carbamidomethylation | C3:Carbamidomethylation:1000.00 | PEAKS DB |
| V.TYSTHIAPVSLPSR.S | N | 61.16 | 1527.8046 | 14 | -2.7 | 510.2741 | 3 | 11.71 | 9 | F9:2491 | DaRuMH\_F8.raw |  |  |  |  |  |  |  |  | 0 |  | 0 | 0 | 0 | 0 | 0 | 0 | 0 | 0 | 0 | 0 | 0 | 123 | 136 |  |  | PEAKS DB |
| R.PVTYSTHIAPVSLPSR.S | N | 60.92 | 1723.9257 | 16 | -0.6 | 575.6488 | 3 | 11.88 | 9 | F9:2672 | DaRuMH\_F8.raw |  |  |  |  |  |  |  |  | 8.827E4 |  | 1 | 0 | 0 | 0 | 0 | 0 | 0 | 0 | 0 | 1 | 0 | 121 | 136 |  |  | PEAKS DB |
| K.FPNGLDK.D | N | 57.14 | 789.4021 | 7 | -1.4 | 395.7078 | 2 | 11.37 | 8 | F8:2253 | DaRuMH\_F7B.raw | 7.7704E5 |  |  |  |  |  |  | 1.3591E6 |  |  | 2 | 1 | 0 | 0 | 0 | 0 | 0 | 0 | 1 | 0 | 0 | 105 | 111 |  |  | PEAKS DB |
| H.C(+57.02)TNIFIVK.H | N | 56.67 | 993.5317 | 8 | 0.3 | 497.7733 | 2 | 12.59 | 9 | F9:3259 | DaRuMH\_F8.raw |  |  |  |  |  |  |  |  | 6.9308E5 |  | 1 | 0 | 0 | 0 | 0 | 0 | 0 | 0 | 0 | 1 | 0 | 165 | 172 | Carbamidomethylation | C1:Carbamidomethylation:1000.00 | PEAKS DB |
| R.TLC(+57.02)AGILK.G | N | 54.02 | 874.4946 | 8 | -0.7 | 438.2543 | 2 | 11.80 | 8 | F8:2668 | DaRuMH\_F7B.raw |  |  |  |  |  |  |  | 6.6304E6 |  |  | 1 | 0 | 0 | 0 | 0 | 0 | 0 | 0 | 1 | 0 | 0 | 189 | 196 | Carbamidomethylation | C3:Carbamidomethylation:1000.00 | PEAKS DB |
| R.EWVLTAAH.C | N | 53.60 | 925.4658 | 8 | 0.4 | 463.7404 | 2 | 13.15 | 8 | F8:3914 | DaRuMH\_F7B.raw |  |  |  |  |  |  |  | 3.0986E5 |  |  | 1 | 0 | 0 | 0 | 0 | 0 | 0 | 0 | 1 | 0 | 0 | 60 | 67 |  |  | PEAKS DB |
| S.TIHC(+57.02)AGALINR.E | Y | 53.31 | 1224.6398 | 11 | 0.0 | 409.2206 | 3 | 11.13 | 9 | F9:1950 | DaRuMH\_F8.raw |  |  |  |  |  |  |  |  | 7.6174E4 |  | 1 | 0 | 0 | 0 | 0 | 0 | 0 | 0 | 0 | 1 | 0 | 49 | 59 | Carbamidomethylation | C4:Carbamidomethylation:1000.00 | PEAKS DB |
| total 25 peptides |
| --- |

A8CG86|PA2A1\_DABRR

back to list

  

| Protein Coverage
| Supporting Peptides
|

Protein Coverage:

Supporting Peptides:

| Peptide | Uniq | -10lgP | Mass | Length | ppm | m/z | z | RT | Fraction | Scan | Source File | Area F1 | Area F10 | Area F2 | Area F3 | Area F4 | Area F5 | Area F7A | Area F7B | Area F8 | Area F9 | #Feature | #Feature F1 | #Feature F10 | #Feature F2 | #Feature F3 | #Feature F4 | #Feature F5 | #Feature F7A | #Feature F7B | #Feature F8 | #Feature F9 | Start | End | PTM | AScore | Found By |
| --- | --- | --- | --- | --- | --- | --- | --- | --- | --- | --- | --- | --- | --- | --- | --- | --- | --- | --- | --- | --- | --- | --- | --- | --- | --- | --- | --- | --- | --- | --- | --- | --- | --- | --- | --- | --- | --- |
| R.AAAIC(+57.02)LGQNVNTYDK.N | N | 103.57 | 1636.7878 | 15 | -1.6 | 819.3999 | 2 | 11.81 | 10 | F10:2632 | DaRuMH\_F9.raw |  | 6.5958E4 |  |  |  |  | 0 | 1.6075E5 | 4.0522E5 | 4.0826E8 | 7 | 0 | 1 | 0 | 0 | 0 | 0 | 0 | 1 | 1 | 4 | 107 | 121 | Carbamidomethylation | C5:Carbamidomethylation:1000.00 | PEAKS DB |
| Y.AIYGC(+57.02)YC(+57.02)GWGGQGKPQDATDR.C | Y | 99.20 | 2359.0110 | 21 | -0.5 | 787.3439 | 3 | 11.66 | 10 | F10:2488 | DaRuMH\_F9.raw |  |  |  |  |  |  |  |  |  | 2.4914E6 | 1 | 0 | 0 | 0 | 0 | 0 | 0 | 0 | 0 | 0 | 1 | 38 | 58 | Carbamidomethylation | C5:Carbamidomethylation:1000.00;C7:Carbamidomethylation:1000.00 | PEAKS DB |
| R.C(+57.02)C(+57.02)FVHDC(+57.02)C(+57.02)YGTVNDC(+57.02)NPK.M | N | 96.20 | 2304.8479 | 18 | -3.2 | 1153.4275 | 2 | 11.34 | 10 | F10:2180 | DaRuMH\_F9.raw |  |  |  |  |  |  |  |  |  | 3.8577E7 | 2 | 0 | 0 | 0 | 0 | 0 | 0 | 0 | 0 | 0 | 2 | 59 | 76 | Carbamidomethylation | C1:Carbamidomethylation:1000.00;C2:Carbamidomethylation:1000.00;C7:Carbamidomethylation:1000.00;C8:Carbamidomethylation:1000.00;C15:Carbamidomethylation:1000.00 | PEAKS DB |
| Y.SYSFENGDIVC(+57.02)GDNNLC(+57.02)LK.T | Y | 94.50 | 2203.9514 | 19 | -0.4 | 1102.9825 | 2 | 17.31 | 10 | F10:7227 | DaRuMH\_F9.raw |  |  |  |  |  |  |  |  |  | 3.0962E8 | 2 | 0 | 0 | 0 | 0 | 0 | 0 | 0 | 0 | 0 | 2 | 81 | 99 | Carbamidomethylation | C11:Carbamidomethylation:1000.00;C17:Carbamidomethylation:1000.00 | PEAKS DB |
| K.EAVHSYAIYGC(+57.02)YC(+57.02)GWGGQGKPQDATDR.C | Y | 91.99 | 3045.3132 | 27 | 0.5 | 762.3360 | 4 | 11.79 | 10 | F10:2616 | DaRuMH\_F9.raw |  |  |  |  |  |  |  |  |  | 7.9569E5 | 1 | 0 | 0 | 0 | 0 | 0 | 0 | 0 | 0 | 0 | 1 | 32 | 58 | Carbamidomethylation | C11:Carbamidomethylation:1000.00;C13:Carbamidomethylation:1000.00 | PEAKS DB |
| C.FVHDC(+57.02)C(+57.02)YGTVNDC(+57.02)NPK.M | N | 85.60 | 1984.7866 | 16 | 1.1 | 993.4017 | 2 | 11.10 | 10 | F10:1955 | DaRuMH\_F9.raw |  |  |  |  |  |  |  |  |  | 2.0549E6 | 2 | 0 | 0 | 0 | 0 | 0 | 0 | 0 | 0 | 0 | 2 | 61 | 76 | Carbamidomethylation | C5:Carbamidomethylation:1000.00;C6:Carbamidomethylation:1000.00;C13:Carbamidomethylation:1000.00 | PEAKS DB |
| Y.SFENGDIVC(+57.02)GDNNLC(+57.02)LK.T | Y | 85.31 | 1953.8561 | 17 | -1.0 | 977.9343 | 2 | 14.32 | 10 | F10:4985 | DaRuMH\_F9.raw |  |  |  |  |  |  |  |  |  | 1.2241E8 | 1 | 0 | 0 | 0 | 0 | 0 | 0 | 0 | 0 | 0 | 1 | 83 | 99 | Carbamidomethylation | C9:Carbamidomethylation:1000.00;C15:Carbamidomethylation:1000.00 | PEAKS DB |
| A.IC(+57.02)LGQNVNTYDK.N | N | 81.43 | 1423.6765 | 12 | 0.8 | 712.8461 | 2 | 11.62 | 10 | F10:2443 | DaRuMH\_F9.raw |  |  |  |  |  |  |  |  |  | 1.9767E6 | 1 | 0 | 0 | 0 | 0 | 0 | 0 | 0 | 0 | 0 | 1 | 110 | 121 | Carbamidomethylation | C2:Carbamidomethylation:1000.00 | PEAKS DB |
| Y.C(+57.02)GWGGQGKPQDATDR.C | N | 77.69 | 1631.7111 | 15 | -1.2 | 816.8618 | 2 | 10.80 | 10 | F10:1670 | DaRuMH\_F9.raw |  |  |  |  |  |  |  |  |  | 4.9083E6 | 2 | 0 | 0 | 0 | 0 | 0 | 0 | 0 | 0 | 0 | 2 | 44 | 58 | Carbamidomethylation | C1:Carbamidomethylation:1000.00 | PEAKS DB |
| F.ENGDIVC(+57.02)GDNNLC(+57.02)LK.T | Y | 76.39 | 1719.7556 | 15 | -0.7 | 860.8845 | 2 | 11.94 | 10 | F10:2758 | DaRuMH\_F9.raw |  |  |  |  |  |  |  |  |  | 7.5661E5 | 1 | 0 | 0 | 0 | 0 | 0 | 0 | 0 | 0 | 0 | 1 | 85 | 99 | Carbamidomethylation | C7:Carbamidomethylation:1000.00;C13:Carbamidomethylation:1000.00 | PEAKS DB |
| K.NYENYAISHC(+57.02)TEESEQC(+57.02) | Y | 76.12 | 2132.8052 | 17 | -4.2 | 1067.4054 | 2 | 11.51 | 10 | F10:2313 | DaRuMH\_F9.raw |  |  |  |  |  |  |  |  | 2.7098E5 | 9.5464E7 | 2 | 0 | 0 | 0 | 0 | 0 | 0 | 0 | 0 | 1 | 1 | 122 | 138 | Carbamidomethylation | C10:Carbamidomethylation:1000.00;C17:Carbamidomethylation:1000.00 | PEAKS DB |
| C.LGQNVNTYDK.N | N | 76.12 | 1150.5619 | 10 | -1.5 | 576.2874 | 2 | 10.98 | 10 | F10:1838 | DaRuMH\_F9.raw |  |  |  |  |  |  |  |  |  | 6.256E6 | 1 | 0 | 0 | 0 | 0 | 0 | 0 | 0 | 0 | 0 | 1 | 112 | 121 |  |  | PEAKS DB |
| F.VHDC(+57.02)C(+57.02)YGTVNDC(+57.02)NPK.M | N | 74.69 | 1837.7181 | 15 | 0.4 | 919.8667 | 2 | 10.75 | 10 | F10:1631 | DaRuMH\_F9.raw |  |  |  |  |  |  |  |  |  | 1.3411E6 | 2 | 0 | 0 | 0 | 0 | 0 | 0 | 0 | 0 | 0 | 2 | 62 | 76 | Carbamidomethylation | C4:Carbamidomethylation:1000.00;C5:Carbamidomethylation:1000.00;C12:Carbamidomethylation:1000.00 | PEAKS DB |
| G.NLFQFAEMIVK.M | N | 74.29 | 1338.7006 | 11 | 0.8 | 670.3581 | 2 | 47.10 | 8 | F8:24564 | DaRuMH\_F7B.raw |  |  |  |  |  | 6.0127E7 | 6.7566E7 | 2.1668E7 | 6.6474E6 | 1.2473E8 | 5 | 0 | 0 | 0 | 0 | 0 | 1 | 1 | 1 | 1 | 1 | 17 | 27 |  |  | PEAKS DB |
| C.YC(+57.02)GWGGQGKPQDATDR.C | N | 73.84 | 1794.7743 | 16 | 0.6 | 599.2657 | 3 | 11.08 | 10 | F10:1931 | DaRuMH\_F9.raw |  |  |  |  |  |  |  |  |  | 1.5717E6 | 2 | 0 | 0 | 0 | 0 | 0 | 0 | 0 | 0 | 0 | 2 | 43 | 58 | Carbamidomethylation | C2:Carbamidomethylation:1000.00 | PEAKS DB |
| G.NLFQFAEM(+15.99)IVK.M | N | 73.14 | 1354.6954 | 11 | 0.1 | 678.3550 | 2 | 40.13 | 10 | F10:19775 | DaRuMH\_F9.raw |  |  |  |  |  | 1.265E7 | 7.6352E6 | 1.1953E6 | 1.7887E6 | 5.3762E7 | 11 | 0 | 0 | 0 | 0 | 0 | 4 | 2 | 1 | 1 | 3 | 17 | 27 | Oxidation (M) | M8:Oxidation (M):1000.00 | PEAKS DB |
| C.C(+57.02)YGTVNDC(+57.02)NPK.M | N | 72.33 | 1326.5333 | 11 | -0.8 | 664.2734 | 2 | 10.80 | 10 | F10:1671 | DaRuMH\_F9.raw |  |  |  |  |  |  |  |  |  | 1.3019E5 | 1 | 0 | 0 | 0 | 0 | 0 | 0 | 0 | 0 | 0 | 1 | 66 | 76 | Carbamidomethylation | C1:Carbamidomethylation:1000.00;C8:Carbamidomethylation:1000.00 | PEAKS DB |
| E.GNLFQFAEMIVK.M | Y | 69.53 | 1395.7220 | 12 | 0.3 | 698.8685 | 2 | 56.80 | 10 | F10:26610 | DaRuMH\_F9.raw |  |  |  |  |  | 1.6652E7 | 1.3068E7 | 6.2845E6 |  | 5.3831E7 | 4 | 0 | 0 | 0 | 0 | 0 | 1 | 1 | 1 | 0 | 1 | 16 | 27 |  |  | PEAKS DB |
| D.IVC(+57.02)GDNNLC(+57.02)LK.T | Y | 69.32 | 1304.6217 | 11 | 0.0 | 653.3181 | 2 | 11.60 | 10 | F10:2422 | DaRuMH\_F9.raw |  |  |  |  |  |  |  |  |  | 4.9772E5 | 1 | 0 | 0 | 0 | 0 | 0 | 0 | 0 | 0 | 0 | 1 | 89 | 99 | Carbamidomethylation | C3:Carbamidomethylation:1000.00;C9:Carbamidomethylation:1000.00 | PEAKS DB |
| K.EAVHSYAIYGC(+57.02)YC(+57.02)GWGGQGK.P | Y | 68.08 | 2261.9622 | 20 | 1.6 | 1131.9901 | 2 | 12.01 | 10 | F10:2827 | DaRuMH\_F9.raw |  |  |  |  |  |  |  |  |  | 1.1481E6 | 2 | 0 | 0 | 0 | 0 | 0 | 0 | 0 | 0 | 0 | 2 | 32 | 51 | Carbamidomethylation | C11:Carbamidomethylation:1000.00;C13:Carbamidomethylation:1000.00 | PEAKS DB |
| H.DC(+57.02)C(+57.02)YGTVNDC(+57.02)NPK.M | N | 65.93 | 1601.5908 | 13 | 0.7 | 801.8032 | 2 | 11.08 | 10 | F10:1951 | DaRuMH\_F9.raw |  |  |  |  |  |  |  |  |  | 1.226E6 | 1 | 0 | 0 | 0 | 0 | 0 | 0 | 0 | 0 | 0 | 1 | 64 | 76 | Carbamidomethylation | C2:Carbamidomethylation:1000.00;C3:Carbamidomethylation:1000.00;C10:Carbamidomethylation:1000.00 | PEAKS DB |
| E.GNLFQFAEM(+15.99)IVK.M | Y | 65.50 | 1411.7169 | 12 | 0.5 | 706.8661 | 2 | 48.95 | 10 | F10:23691 | DaRuMH\_F9.raw |  |  |  |  |  | 1.6329E6 |  |  |  | 1.2222E7 | 2 | 0 | 0 | 0 | 0 | 0 | 1 | 0 | 0 | 0 | 1 | 16 | 27 | Oxidation (M) | M9:Oxidation (M):1000.00 | PEAKS DB |
| D.C(+57.02)C(+57.02)YGTVNDC(+57.02)NPK.M | N | 65.49 | 1486.5640 | 12 | 0.7 | 744.2898 | 2 | 10.93 | 10 | F10:1805 | DaRuMH\_F9.raw |  |  |  |  |  |  |  |  |  | 1.4308E5 | 1 | 0 | 0 | 0 | 0 | 0 | 0 | 0 | 0 | 0 | 1 | 65 | 76 | Carbamidomethylation | C1:Carbamidomethylation:1000.00;C2:Carbamidomethylation:1000.00;C9:Carbamidomethylation:1000.00 | PEAKS DB |
| K.EAVHSYAIYGC(+57.02).Y | N | 64.73 | 1268.5496 | 11 | -0.5 | 635.2817 | 2 | 11.79 | 10 | F10:2614 | DaRuMH\_F9.raw |  |  |  |  |  |  |  |  |  | 5.1272E5 | 1 | 0 | 0 | 0 | 0 | 0 | 0 | 0 | 0 | 0 | 1 | 32 | 42 | Carbamidomethylation | C11:Carbamidomethylation:1000.00 | PEAKS DB |
| C.YGTVNDC(+57.02)NPK.M | N | 62.86 | 1166.5026 | 10 | -2.6 | 584.2570 | 2 | 10.71 | 10 | F10:1615 | DaRuMH\_F9.raw |  |  |  |  |  |  |  |  |  | 1.6889E6 | 1 | 0 | 0 | 0 | 0 | 0 | 0 | 0 | 0 | 0 | 1 | 67 | 76 | Carbamidomethylation | C7:Carbamidomethylation:1000.00 | PEAKS DB |
| K.EAVHSYAIYGC(+57.02)Y.C | N | 60.47 | 1431.6129 | 12 | 1.7 | 716.8149 | 2 | 12.28 | 10 | F10:3221 | DaRuMH\_F9.raw |  |  |  |  |  |  |  |  |  | 1.4675E7 | 1 | 0 | 0 | 0 | 0 | 0 | 0 | 0 | 0 | 0 | 1 | 32 | 43 | Carbamidomethylation | C11:Carbamidomethylation:1000.00 | PEAKS DB |
| A.AAIC(+57.02)LGQNVNTYDK.N | N | 56.43 | 1565.7507 | 14 | -0.2 | 783.8824 | 2 | 11.79 | 10 | F10:2618 | DaRuMH\_F9.raw |  |  |  |  |  |  |  |  |  | 9.0238E4 | 1 | 0 | 0 | 0 | 0 | 0 | 0 | 0 | 0 | 0 | 1 | 108 | 121 | Carbamidomethylation | C4:Carbamidomethylation:1000.00 | PEAKS DB |
| N.GDIVC(+57.02)GDNNLC(+57.02)LK.T | Y | 54.68 | 1476.6700 | 13 | 2.1 | 739.3439 | 2 | 11.90 | 10 | F10:2711 | DaRuMH\_F9.raw |  |  |  |  |  |  |  |  |  | 3.7323E5 | 1 | 0 | 0 | 0 | 0 | 0 | 0 | 0 | 0 | 0 | 1 | 87 | 99 | Carbamidomethylation | C5:Carbamidomethylation:1000.00;C11:Carbamidomethylation:1000.00 | PEAKS DB |
| N.YAISHC(+57.02)TEESEQC(+57.02) | Y | 54.63 | 1612.6134 | 13 | 1.5 | 807.3152 | 2 | 11.04 | 10 | F10:1888 | DaRuMH\_F9.raw |  |  |  |  |  |  |  |  |  | 3.7362E6 | 1 | 0 | 0 | 0 | 0 | 0 | 0 | 0 | 0 | 0 | 1 | 126 | 138 | Carbamidomethylation | C6:Carbamidomethylation:1000.00;C13:Carbamidomethylation:1000.00 | PEAKS DB |
| N.YENYAISHC(+57.02)TEESEQC(+57.02) | Y | 54.51 | 2018.7622 | 16 | -0.1 | 1010.3882 | 2 | 11.37 | 10 | F10:2225 | DaRuMH\_F9.raw |  |  |  |  |  |  |  |  |  | 7.4111E5 | 1 | 0 | 0 | 0 | 0 | 0 | 0 | 0 | 0 | 0 | 1 | 123 | 138 | Carbamidomethylation | C9:Carbamidomethylation:1000.00;C16:Carbamidomethylation:1000.00 | PEAKS DB |
| total 30 peptides |
| --- |

A8CG87|PA2A2\_DABRR

back to list

  

| Protein Coverage
| Supporting Peptides
|

Protein Coverage:

Supporting Peptides:

| Peptide | Uniq | -10lgP | Mass | Length | ppm | m/z | z | RT | Fraction | Scan | Source File | Area F1 | Area F10 | Area F2 | Area F3 | Area F4 | Area F5 | Area F7A | Area F7B | Area F8 | Area F9 | #Feature | #Feature F1 | #Feature F10 | #Feature F2 | #Feature F3 | #Feature F4 | #Feature F5 | #Feature F7A | #Feature F7B | #Feature F8 | #Feature F9 | Start | End | PTM | AScore | Found By |
| --- | --- | --- | --- | --- | --- | --- | --- | --- | --- | --- | --- | --- | --- | --- | --- | --- | --- | --- | --- | --- | --- | --- | --- | --- | --- | --- | --- | --- | --- | --- | --- | --- | --- | --- | --- | --- | --- |
| K.TGNFGLLSYVYYGC(+57.02)YC(+57.02)GWGGK.G | Y | 104.51 | 2421.0559 | 21 | 0.3 | 1211.5356 | 2 | 46.26 | 8 | F8:24174 | DaRuMH\_F7B.raw |  |  |  |  |  |  |  | 4.4703E7 |  |  | 3 | 0 | 0 | 0 | 0 | 0 | 0 | 0 | 3 | 0 | 0 | 29 | 49 | Carbamidomethylation | C14:Carbamidomethylation:1000.00;C16:Carbamidomethylation:1000.00 | PEAKS DB |
| K.TATYSYSFENGDIVC(+57.02)GGDDPC(+57.02)LR.A | Y | 97.25 | 2596.0845 | 23 | 1.2 | 1299.0510 | 2 | 18.07 | 8 | F8:8050 | DaRuMH\_F7B.raw |  |  |  |  |  |  |  | 4.4528E7 |  |  | 3 | 0 | 0 | 0 | 0 | 0 | 0 | 0 | 3 | 0 | 0 | 77 | 99 | Carbamidomethylation | C15:Carbamidomethylation:1000.00;C21:Carbamidomethylation:1000.00 | PEAKS DB |
| Y.SFENGDIVC(+57.02)GGDDPC(+57.02)LR.A | N | 92.97 | 1909.7935 | 17 | -0.2 | 955.9038 | 2 | 12.54 | 8 | F8:3310 | DaRuMH\_F7B.raw | 2.7917E5 |  |  |  | 3.143E5 |  | 3.2525E6 | 8.3842E7 |  |  | 5 | 1 | 0 | 0 | 0 | 1 | 0 | 1 | 2 | 0 | 0 | 83 | 99 | Carbamidomethylation | C9:Carbamidomethylation:1000.00;C15:Carbamidomethylation:1000.00 | PEAKS DB |
| Y.VYYGC(+57.02)YC(+57.02)GWGGK.G | Y | 87.18 | 1468.5903 | 12 | -0.4 | 735.3021 | 2 | 12.01 | 8 | F8:2874 | DaRuMH\_F7B.raw | 7.2404E4 |  |  |  |  |  | 1.3584E6 | 4.6684E7 |  |  | 3 | 1 | 0 | 0 | 0 | 0 | 0 | 1 | 1 | 0 | 0 | 38 | 49 | Carbamidomethylation | C5:Carbamidomethylation:1000.00;C7:Carbamidomethylation:1000.00 | PEAKS DB |
| D.IVC(+57.02)GGDDPC(+57.02)LR.A | N | 86.87 | 1260.5591 | 11 | -0.7 | 631.2864 | 2 | 11.30 | 8 | F8:2189 | DaRuMH\_F7B.raw |  |  |  |  |  |  |  | 1.7117E6 |  |  | 1 | 0 | 0 | 0 | 0 | 0 | 0 | 0 | 1 | 0 | 0 | 89 | 99 | Carbamidomethylation | C3:Carbamidomethylation:1000.00;C9:Carbamidomethylation:1000.00 | PEAKS DB |
| R.AVC(+57.02)EC(+57.02)DRVAAIC(+57.02)FR.E | N | 83.05 | 1725.7749 | 14 | 0.1 | 576.2656 | 3 | 11.72 | 8 | F8:2607 | DaRuMH\_F7B.raw |  |  |  |  |  |  | 5.3532E5 | 1.584E6 |  |  | 3 | 0 | 0 | 0 | 0 | 0 | 0 | 1 | 2 | 0 | 0 | 100 | 113 | Carbamidomethylation | C3:Carbamidomethylation:1000.00;C5:Carbamidomethylation:1000.00;C12:Carbamidomethylation:1000.00 | PEAKS DB |
| Y.SYSFENGDIVC(+57.02)GGDDPC(+57.02)LR.A | N | 82.84 | 2159.8887 | 19 | -1.4 | 1080.9476 | 2 | 14.88 | 7 | F7:5003 | DaRuMH\_F7A.raw |  |  |  |  |  |  | 5.5744E6 | 7.2374E7 |  |  | 2 | 0 | 0 | 0 | 0 | 0 | 0 | 1 | 1 | 0 | 0 | 81 | 99 | Carbamidomethylation | C11:Carbamidomethylation:1000.00;C17:Carbamidomethylation:1000.00 | PEAKS DB |
| K.YM(+15.99)LYSIFDC(+57.02)K.E | N | 81.85 | 1354.5938 | 10 | 0.1 | 678.3042 | 2 | 21.36 | 8 | F8:10863 | DaRuMH\_F7B.raw |  |  |  |  |  |  | 1.83E6 | 6.2816E6 |  |  | 5 | 0 | 0 | 0 | 0 | 0 | 0 | 2 | 3 | 0 | 0 | 123 | 132 | Oxidation (M); Carbamidomethylation | M2:Oxidation (M):1000.00;C9:Carbamidomethylation:1000.00 | PEAKS DB |
| K.YMLYSIFDC(+57.02)K.E | N | 80.90 | 1338.5988 | 10 | 1.1 | 670.3074 | 2 | 27.75 | 8 | F8:14512 | DaRuMH\_F7B.raw |  |  |  |  |  |  |  | 9.2178E6 |  |  | 1 | 0 | 0 | 0 | 0 | 0 | 0 | 0 | 1 | 0 | 0 | 123 | 132 | Carbamidomethylation | C9:Carbamidomethylation:1000.00 | PEAKS DB |
| S.FENGDIVC(+57.02)GGDDPC(+57.02)LR.A | N | 79.08 | 1822.7614 | 16 | 0.0 | 912.3879 | 2 | 12.08 | 8 | F8:2936 | DaRuMH\_F7B.raw |  |  |  |  |  |  |  | 6.2569E6 |  |  | 1 | 0 | 0 | 0 | 0 | 0 | 0 | 0 | 1 | 0 | 0 | 84 | 99 | Carbamidomethylation | C8:Carbamidomethylation:1000.00;C14:Carbamidomethylation:1000.00 | PEAKS DB |
| G.NLYQFGEMINQK.T | Y | 78.16 | 1483.7129 | 12 | 0.1 | 742.8638 | 2 | 17.00 | 8 | F8:7465 | DaRuMH\_F7B.raw |  |  |  |  |  |  | 7.1113E6 | 1.2488E8 | 1.5164E5 |  | 4 | 0 | 0 | 0 | 0 | 0 | 0 | 1 | 2 | 1 | 0 | 17 | 28 |  |  | PEAKS DB |
| F.ENGDIVC(+57.02)GGDDPC(+57.02)LR.A | N | 72.96 | 1675.6930 | 15 | 0.0 | 838.8538 | 2 | 11.69 | 8 | F8:2579 | DaRuMH\_F7B.raw |  |  |  |  |  |  |  | 9.3479E5 |  |  | 1 | 0 | 0 | 0 | 0 | 0 | 0 | 0 | 1 | 0 | 0 | 85 | 99 | Carbamidomethylation | C7:Carbamidomethylation:1000.00;C13:Carbamidomethylation:1000.00 | PEAKS DB |
| R.C(+57.02)C(+57.02)FVHDC(+57.02)C(+57.02)YGR.V | N | 71.07 | 1532.5417 | 11 | 2.9 | 767.2786 | 2 | 11.11 | 7 | F7:1942 | DaRuMH\_F7A.raw |  |  |  | 7.7102E5 | 7.7933E5 |  | 9.6866E5 | 7.6051E7 |  |  | 6 | 0 | 0 | 0 | 1 | 2 | 0 | 1 | 2 | 0 | 0 | 59 | 69 | Carbamidomethylation | C1:Carbamidomethylation:1000.00;C2:Carbamidomethylation:1000.00;C7:Carbamidomethylation:1000.00;C8:Carbamidomethylation:1000.00 | PEAKS DB |
| V.YYGC(+57.02)YC(+57.02)GWGGK.G | N | 70.74 | 1369.5220 | 11 | 0.3 | 685.7685 | 2 | 11.78 | 8 | F8:2687 | DaRuMH\_F7B.raw |  |  |  |  |  |  |  | 7.8895E5 |  |  | 1 | 0 | 0 | 0 | 0 | 0 | 0 | 0 | 1 | 0 | 0 | 39 | 49 | Carbamidomethylation | C4:Carbamidomethylation:1000.00;C6:Carbamidomethylation:1000.00 | PEAKS DB |
| Y.SIFDC(+57.02)KEESDQC(+57.02) | N | 68.40 | 1516.5809 | 12 | 0.1 | 759.2979 | 2 | 11.60 | 8 | F8:2495 | DaRuMH\_F7B.raw |  |  |  |  |  |  |  | 2.5725E7 |  |  | 1 | 0 | 0 | 0 | 0 | 0 | 0 | 0 | 1 | 0 | 0 | 127 | 138 | Carbamidomethylation | C5:Carbamidomethylation:1000.00;C12:Carbamidomethylation:1000.00 | PEAKS DB |
| L.SYVYYGC(+57.02)YC(+57.02)GWGGK.G | Y | 67.68 | 1718.6858 | 14 | 1.3 | 860.3493 | 2 | 13.96 | 7 | F7:4299 | DaRuMH\_F7A.raw |  |  |  |  |  |  | 6.9683E5 | 6.7437E6 |  |  | 2 | 0 | 0 | 0 | 0 | 0 | 0 | 1 | 1 | 0 | 0 | 36 | 49 | Carbamidomethylation | C7:Carbamidomethylation:1000.00;C9:Carbamidomethylation:1000.00 | PEAKS DB |
| G.NLYQFGEM(+15.99)INQK.T | Y | 67.03 | 1499.7079 | 12 | 0.1 | 750.8613 | 2 | 12.35 | 8 | F8:3167 | DaRuMH\_F7B.raw |  |  |  |  |  |  | 1.5381E6 | 1.3829E7 |  |  | 3 | 0 | 0 | 0 | 0 | 0 | 0 | 1 | 2 | 0 | 0 | 17 | 28 | Oxidation (M) | M8:Oxidation (M):1000.00 | PEAKS DB |
| Y.YGC(+57.02)YC(+57.02)GWGGK.G | N | 66.85 | 1206.4586 | 10 | -0.3 | 604.2364 | 2 | 11.56 | 8 | F8:2453 | DaRuMH\_F7B.raw |  |  |  |  | 2.4701E5 | 2.1367E7 | 1.6469E7 | 7.7105E6 |  |  | 4 | 0 | 0 | 0 | 0 | 1 | 1 | 1 | 1 | 0 | 0 | 40 | 49 | Carbamidomethylation | C3:Carbamidomethylation:1000.00;C5:Carbamidomethylation:1000.00 | PEAKS DB |
| E.NGDIVC(+57.02)GGDDPC(+57.02)LR.A | N | 66.25 | 1546.6504 | 14 | -0.7 | 774.3319 | 2 | 11.60 | 8 | F8:2517 | DaRuMH\_F7B.raw |  |  |  | 2.5237E5 |  |  |  | 9.3572E5 |  |  | 2 | 0 | 0 | 0 | 1 | 0 | 0 | 0 | 1 | 0 | 0 | 86 | 99 | Carbamidomethylation | C6:Carbamidomethylation:1000.00;C12:Carbamidomethylation:1000.00 | PEAKS DB |
| S.YVYYGC(+57.02)YC(+57.02)GWGGK.G | Y | 65.92 | 1631.6537 | 13 | 1.4 | 816.8353 | 2 | 13.24 | 8 | F8:3950 | DaRuMH\_F7B.raw |  |  |  |  |  |  |  | 1.3791E6 |  |  | 1 | 0 | 0 | 0 | 0 | 0 | 0 | 0 | 1 | 0 | 0 | 37 | 49 | Carbamidomethylation | C6:Carbamidomethylation:1000.00;C8:Carbamidomethylation:1000.00 | PEAKS DB |
| N.GDIVC(+57.02)GGDDPC(+57.02)LR.A | N | 65.50 | 1432.6074 | 13 | 0.0 | 717.3110 | 2 | 11.60 | 5 | F5:2413 | DaRuMH\_F4.raw |  |  | 1.0971E5 | 1.1137E6 | 1.1053E6 |  |  | 1.6452E6 |  |  | 4 | 0 | 0 | 1 | 1 | 1 | 0 | 0 | 1 | 0 | 0 | 87 | 99 | Carbamidomethylation | C5:Carbamidomethylation:1000.00;C11:Carbamidomethylation:1000.00 | PEAKS DB |
| E.GNLYQFGEMINQK.T | Y | 65.34 | 1540.7344 | 13 | 0.5 | 771.3749 | 2 | 20.75 | 8 | F8:10398 | DaRuMH\_F7B.raw |  |  |  |  |  |  |  | 2.886E7 |  |  | 1 | 0 | 0 | 0 | 0 | 0 | 0 | 0 | 1 | 0 | 0 | 16 | 28 |  |  | PEAKS DB |
| K.TGNFGLLSYVYYGC(+57.02)Y.C | Y | 62.93 | 1775.7865 | 15 | 0.8 | 888.9012 | 2 | 57.48 | 8 | F8:28096 | DaRuMH\_F7B.raw |  |  |  |  |  |  |  | 1.763E7 |  |  | 1 | 0 | 0 | 0 | 0 | 0 | 0 | 0 | 1 | 0 | 0 | 29 | 43 | Carbamidomethylation | C14:Carbamidomethylation:1000.00 | PEAKS DB |
| Y.GC(+57.02)YC(+57.02)GWGGK.G | N | 58.54 | 1043.3953 | 9 | -0.4 | 522.7047 | 2 | 11.29 | 6 | F6:2113 | DaRuMH\_F5.raw |  |  |  |  |  | 2.1095E6 |  | 4.3981E6 |  |  | 2 | 0 | 0 | 0 | 0 | 0 | 1 | 0 | 1 | 0 | 0 | 41 | 49 | Carbamidomethylation | C2:Carbamidomethylation:1000.00;C4:Carbamidomethylation:1000.00 | PEAKS DB |
| C.FVHDC(+57.02)C(+57.02)YGR.V | N | 56.61 | 1212.4805 | 9 | -0.3 | 405.1673 | 3 | 10.65 | 8 | F8:1634 | DaRuMH\_F7B.raw |  |  |  |  |  |  |  | 1.579E5 |  |  | 1 | 0 | 0 | 0 | 0 | 0 | 0 | 0 | 1 | 0 | 0 | 61 | 69 | Carbamidomethylation | C5:Carbamidomethylation:1000.00;C6:Carbamidomethylation:1000.00 | PEAKS DB |
| total 25 peptides |
| --- |

A8CG78|PA2A2\_DABSI

back to list

  

| Protein Coverage
| Supporting Peptides
|

Protein Coverage:

Supporting Peptides:

| Peptide | Uniq | -10lgP | Mass | Length | ppm | m/z | z | RT | Fraction | Scan | Source File | Area F1 | Area F10 | Area F2 | Area F3 | Area F4 | Area F5 | Area F7A | Area F7B | Area F8 | Area F9 | #Feature | #Feature F1 | #Feature F10 | #Feature F2 | #Feature F3 | #Feature F4 | #Feature F5 | #Feature F7A | #Feature F7B | #Feature F8 | #Feature F9 | Start | End | PTM | AScore | Found By |
| --- | --- | --- | --- | --- | --- | --- | --- | --- | --- | --- | --- | --- | --- | --- | --- | --- | --- | --- | --- | --- | --- | --- | --- | --- | --- | --- | --- | --- | --- | --- | --- | --- | --- | --- | --- | --- | --- |
| K.TGNFGLLSYVYYGC(+57.02)YC(+57.02)GWGGK.G | Y | 104.51 | 2421.0559 | 21 | 0.3 | 1211.5356 | 2 | 46.26 | 8 | F8:24174 | DaRuMH\_F7B.raw |  |  |  |  |  |  |  | 4.4703E7 |  |  | 3 | 0 | 0 | 0 | 0 | 0 | 0 | 0 | 3 | 0 | 0 | 29 | 49 | Carbamidomethylation | C14:Carbamidomethylation:1000.00;C16:Carbamidomethylation:1000.00 | PEAKS DB |
| K.TATYSYSFENGDIVC(+57.02)GGDDPC(+57.02)LR.A | Y | 97.25 | 2596.0845 | 23 | 1.2 | 1299.0510 | 2 | 18.07 | 8 | F8:8050 | DaRuMH\_F7B.raw |  |  |  |  |  |  |  | 4.4528E7 |  |  | 3 | 0 | 0 | 0 | 0 | 0 | 0 | 0 | 3 | 0 | 0 | 77 | 99 | Carbamidomethylation | C15:Carbamidomethylation:1000.00;C21:Carbamidomethylation:1000.00 | PEAKS DB |
| Y.SFENGDIVC(+57.02)GGDDPC(+57.02)LR.A | N | 92.97 | 1909.7935 | 17 | -0.2 | 955.9038 | 2 | 12.54 | 8 | F8:3310 | DaRuMH\_F7B.raw | 2.7917E5 |  |  |  | 3.143E5 |  | 3.2525E6 | 8.3842E7 |  |  | 5 | 1 | 0 | 0 | 0 | 1 | 0 | 1 | 2 | 0 | 0 | 83 | 99 | Carbamidomethylation | C9:Carbamidomethylation:1000.00;C15:Carbamidomethylation:1000.00 | PEAKS DB |
| Y.VYYGC(+57.02)YC(+57.02)GWGGK.G | Y | 87.18 | 1468.5903 | 12 | -0.4 | 735.3021 | 2 | 12.01 | 8 | F8:2874 | DaRuMH\_F7B.raw | 7.2404E4 |  |  |  |  |  | 1.3584E6 | 4.6684E7 |  |  | 3 | 1 | 0 | 0 | 0 | 0 | 0 | 1 | 1 | 0 | 0 | 38 | 49 | Carbamidomethylation | C5:Carbamidomethylation:1000.00;C7:Carbamidomethylation:1000.00 | PEAKS DB |
| D.IVC(+57.02)GGDDPC(+57.02)LR.A | N | 86.87 | 1260.5591 | 11 | -0.7 | 631.2864 | 2 | 11.30 | 8 | F8:2189 | DaRuMH\_F7B.raw |  |  |  |  |  |  |  | 1.7117E6 |  |  | 1 | 0 | 0 | 0 | 0 | 0 | 0 | 0 | 1 | 0 | 0 | 89 | 99 | Carbamidomethylation | C3:Carbamidomethylation:1000.00;C9:Carbamidomethylation:1000.00 | PEAKS DB |
| R.AVC(+57.02)EC(+57.02)DRVAAIC(+57.02)FR.E | N | 83.05 | 1725.7749 | 14 | 0.1 | 576.2656 | 3 | 11.72 | 8 | F8:2607 | DaRuMH\_F7B.raw |  |  |  |  |  |  | 5.3532E5 | 1.584E6 |  |  | 3 | 0 | 0 | 0 | 0 | 0 | 0 | 1 | 2 | 0 | 0 | 100 | 113 | Carbamidomethylation | C3:Carbamidomethylation:1000.00;C5:Carbamidomethylation:1000.00;C12:Carbamidomethylation:1000.00 | PEAKS DB |
| Y.SYSFENGDIVC(+57.02)GGDDPC(+57.02)LR.A | N | 82.84 | 2159.8887 | 19 | -1.4 | 1080.9476 | 2 | 14.88 | 7 | F7:5003 | DaRuMH\_F7A.raw |  |  |  |  |  |  | 5.5744E6 | 7.2374E7 |  |  | 2 | 0 | 0 | 0 | 0 | 0 | 0 | 1 | 1 | 0 | 0 | 81 | 99 | Carbamidomethylation | C11:Carbamidomethylation:1000.00;C17:Carbamidomethylation:1000.00 | PEAKS DB |
| K.YM(+15.99)LYSIFDC(+57.02)K.E | N | 81.85 | 1354.5938 | 10 | 0.1 | 678.3042 | 2 | 21.36 | 8 | F8:10863 | DaRuMH\_F7B.raw |  |  |  |  |  |  | 1.83E6 | 6.2816E6 |  |  | 5 | 0 | 0 | 0 | 0 | 0 | 0 | 2 | 3 | 0 | 0 | 123 | 132 | Oxidation (M); Carbamidomethylation | M2:Oxidation (M):1000.00;C9:Carbamidomethylation:1000.00 | PEAKS DB |
| K.YMLYSIFDC(+57.02)K.E | N | 80.90 | 1338.5988 | 10 | 1.1 | 670.3074 | 2 | 27.75 | 8 | F8:14512 | DaRuMH\_F7B.raw |  |  |  |  |  |  |  | 9.2178E6 |  |  | 1 | 0 | 0 | 0 | 0 | 0 | 0 | 0 | 1 | 0 | 0 | 123 | 132 | Carbamidomethylation | C9:Carbamidomethylation:1000.00 | PEAKS DB |
| S.FENGDIVC(+57.02)GGDDPC(+57.02)LR.A | N | 79.08 | 1822.7614 | 16 | 0.0 | 912.3879 | 2 | 12.08 | 8 | F8:2936 | DaRuMH\_F7B.raw |  |  |  |  |  |  |  | 6.2569E6 |  |  | 1 | 0 | 0 | 0 | 0 | 0 | 0 | 0 | 1 | 0 | 0 | 84 | 99 | Carbamidomethylation | C8:Carbamidomethylation:1000.00;C14:Carbamidomethylation:1000.00 | PEAKS DB |
| G.NLYQFGEMINQK.T | Y | 78.16 | 1483.7129 | 12 | 0.1 | 742.8638 | 2 | 17.00 | 8 | F8:7465 | DaRuMH\_F7B.raw |  |  |  |  |  |  | 7.1113E6 | 1.2488E8 | 1.5164E5 |  | 4 | 0 | 0 | 0 | 0 | 0 | 0 | 1 | 2 | 1 | 0 | 17 | 28 |  |  | PEAKS DB |
| F.ENGDIVC(+57.02)GGDDPC(+57.02)LR.A | N | 72.96 | 1675.6930 | 15 | 0.0 | 838.8538 | 2 | 11.69 | 8 | F8:2579 | DaRuMH\_F7B.raw |  |  |  |  |  |  |  | 9.3479E5 |  |  | 1 | 0 | 0 | 0 | 0 | 0 | 0 | 0 | 1 | 0 | 0 | 85 | 99 | Carbamidomethylation | C7:Carbamidomethylation:1000.00;C13:Carbamidomethylation:1000.00 | PEAKS DB |
| R.C(+57.02)C(+57.02)FVHDC(+57.02)C(+57.02)YGR.V | N | 71.07 | 1532.5417 | 11 | 2.9 | 767.2786 | 2 | 11.11 | 7 | F7:1942 | DaRuMH\_F7A.raw |  |  |  | 7.7102E5 | 7.7933E5 |  | 9.6866E5 | 7.6051E7 |  |  | 6 | 0 | 0 | 0 | 1 | 2 | 0 | 1 | 2 | 0 | 0 | 59 | 69 | Carbamidomethylation | C1:Carbamidomethylation:1000.00;C2:Carbamidomethylation:1000.00;C7:Carbamidomethylation:1000.00;C8:Carbamidomethylation:1000.00 | PEAKS DB |
| V.YYGC(+57.02)YC(+57.02)GWGGK.G | N | 70.74 | 1369.5220 | 11 | 0.3 | 685.7685 | 2 | 11.78 | 8 | F8:2687 | DaRuMH\_F7B.raw |  |  |  |  |  |  |  | 7.8895E5 |  |  | 1 | 0 | 0 | 0 | 0 | 0 | 0 | 0 | 1 | 0 | 0 | 39 | 49 | Carbamidomethylation | C4:Carbamidomethylation:1000.00;C6:Carbamidomethylation:1000.00 | PEAKS DB |
| Y.SIFDC(+57.02)KEESDQC(+57.02) | N | 68.40 | 1516.5809 | 12 | 0.1 | 759.2979 | 2 | 11.60 | 8 | F8:2495 | DaRuMH\_F7B.raw |  |  |  |  |  |  |  | 2.5725E7 |  |  | 1 | 0 | 0 | 0 | 0 | 0 | 0 | 0 | 1 | 0 | 0 | 127 | 138 | Carbamidomethylation | C5:Carbamidomethylation:1000.00;C12:Carbamidomethylation:1000.00 | PEAKS DB |
| L.SYVYYGC(+57.02)YC(+57.02)GWGGK.G | Y | 67.68 | 1718.6858 | 14 | 1.3 | 860.3493 | 2 | 13.96 | 7 | F7:4299 | DaRuMH\_F7A.raw |  |  |  |  |  |  | 6.9683E5 | 6.7437E6 |  |  | 2 | 0 | 0 | 0 | 0 | 0 | 0 | 1 | 1 | 0 | 0 | 36 | 49 | Carbamidomethylation | C7:Carbamidomethylation:1000.00;C9:Carbamidomethylation:1000.00 | PEAKS DB |
| G.NLYQFGEM(+15.99)INQK.T | Y | 67.03 | 1499.7079 | 12 | 0.1 | 750.8613 | 2 | 12.35 | 8 | F8:3167 | DaRuMH\_F7B.raw |  |  |  |  |  |  | 1.5381E6 | 1.3829E7 |  |  | 3 | 0 | 0 | 0 | 0 | 0 | 0 | 1 | 2 | 0 | 0 | 17 | 28 | Oxidation (M) | M8:Oxidation (M):1000.00 | PEAKS DB |
| Y.YGC(+57.02)YC(+57.02)GWGGK.G | N | 66.85 | 1206.4586 | 10 | -0.3 | 604.2364 | 2 | 11.56 | 8 | F8:2453 | DaRuMH\_F7B.raw |  |  |  |  | 2.4701E5 | 2.1367E7 | 1.6469E7 | 7.7105E6 |  |  | 4 | 0 | 0 | 0 | 0 | 1 | 1 | 1 | 1 | 0 | 0 | 40 | 49 | Carbamidomethylation | C3:Carbamidomethylation:1000.00;C5:Carbamidomethylation:1000.00 | PEAKS DB |
| E.NGDIVC(+57.02)GGDDPC(+57.02)LR.A | N | 66.25 | 1546.6504 | 14 | -0.7 | 774.3319 | 2 | 11.60 | 8 | F8:2517 | DaRuMH\_F7B.raw |  |  |  | 2.5237E5 |  |  |  | 9.3572E5 |  |  | 2 | 0 | 0 | 0 | 1 | 0 | 0 | 0 | 1 | 0 | 0 | 86 | 99 | Carbamidomethylation | C6:Carbamidomethylation:1000.00;C12:Carbamidomethylation:1000.00 | PEAKS DB |
| S.YVYYGC(+57.02)YC(+57.02)GWGGK.G | Y | 65.92 | 1631.6537 | 13 | 1.4 | 816.8353 | 2 | 13.24 | 8 | F8:3950 | DaRuMH\_F7B.raw |  |  |  |  |  |  |  | 1.3791E6 |  |  | 1 | 0 | 0 | 0 | 0 | 0 | 0 | 0 | 1 | 0 | 0 | 37 | 49 | Carbamidomethylation | C6:Carbamidomethylation:1000.00;C8:Carbamidomethylation:1000.00 | PEAKS DB |
| N.GDIVC(+57.02)GGDDPC(+57.02)LR.A | N | 65.50 | 1432.6074 | 13 | 0.0 | 717.3110 | 2 | 11.60 | 5 | F5:2413 | DaRuMH\_F4.raw |  |  | 1.0971E5 | 1.1137E6 | 1.1053E6 |  |  | 1.6452E6 |  |  | 4 | 0 | 0 | 1 | 1 | 1 | 0 | 0 | 1 | 0 | 0 | 87 | 99 | Carbamidomethylation | C5:Carbamidomethylation:1000.00;C11:Carbamidomethylation:1000.00 | PEAKS DB |
| E.GNLYQFGEMINQK.T | Y | 65.34 | 1540.7344 | 13 | 0.5 | 771.3749 | 2 | 20.75 | 8 | F8:10398 | DaRuMH\_F7B.raw |  |  |  |  |  |  |  | 2.886E7 |  |  | 1 | 0 | 0 | 0 | 0 | 0 | 0 | 0 | 1 | 0 | 0 | 16 | 28 |  |  | PEAKS DB |
| K.TGNFGLLSYVYYGC(+57.02)Y.C | Y | 62.93 | 1775.7865 | 15 | 0.8 | 888.9012 | 2 | 57.48 | 8 | F8:28096 | DaRuMH\_F7B.raw |  |  |  |  |  |  |  | 1.763E7 |  |  | 1 | 0 | 0 | 0 | 0 | 0 | 0 | 0 | 1 | 0 | 0 | 29 | 43 | Carbamidomethylation | C14:Carbamidomethylation:1000.00 | PEAKS DB |
| Y.GC(+57.02)YC(+57.02)GWGGK.G | N | 58.54 | 1043.3953 | 9 | -0.4 | 522.7047 | 2 | 11.29 | 6 | F6:2113 | DaRuMH\_F5.raw |  |  |  |  |  | 2.1095E6 |  | 4.3981E6 |  |  | 2 | 0 | 0 | 0 | 0 | 0 | 1 | 0 | 1 | 0 | 0 | 41 | 49 | Carbamidomethylation | C2:Carbamidomethylation:1000.00;C4:Carbamidomethylation:1000.00 | PEAKS DB |
| C.FVHDC(+57.02)C(+57.02)YGR.V | N | 56.61 | 1212.4805 | 9 | -0.3 | 405.1673 | 3 | 10.65 | 8 | F8:1634 | DaRuMH\_F7B.raw |  |  |  |  |  |  |  | 1.579E5 |  |  | 1 | 0 | 0 | 0 | 0 | 0 | 0 | 0 | 1 | 0 | 0 | 61 | 69 | Carbamidomethylation | C5:Carbamidomethylation:1000.00;C6:Carbamidomethylation:1000.00 | PEAKS DB |
| total 25 peptides |
| --- |

B8K1W0|VM3DK\_DABRR

back to list

  

| Protein Coverage
| Supporting Peptides
|

Protein Coverage:

Supporting Peptides:

| Peptide | Uniq | -10lgP | Mass | Length | ppm | m/z | z | RT | Fraction | Scan | Source File | Area F1 | Area F10 | Area F2 | Area F3 | Area F4 | Area F5 | Area F7A | Area F7B | Area F8 | Area F9 | #Feature | #Feature F1 | #Feature F10 | #Feature F2 | #Feature F3 | #Feature F4 | #Feature F5 | #Feature F7A | #Feature F7B | #Feature F8 | #Feature F9 | Start | End | PTM | AScore | Found By |
| --- | --- | --- | --- | --- | --- | --- | --- | --- | --- | --- | --- | --- | --- | --- | --- | --- | --- | --- | --- | --- | --- | --- | --- | --- | --- | --- | --- | --- | --- | --- | --- | --- | --- | --- | --- | --- | --- |
| L.ESGNVNDYEVVYPQK.V | N | 99.26 | 1739.8002 | 15 | 0.4 | 870.9077 | 2 | 11.79 | 3 | F3:2512 | DaRuMH\_F2.raw | 1.5166E5 |  | 1.2778E6 |  |  |  |  |  |  |  | 2 | 1 | 0 | 1 | 0 | 0 | 0 | 0 | 0 | 0 | 0 | 24 | 38 |  |  | PEAKS DB |
| R.FLTEHNPEC(+57.02)IINPPLR.T | Y | 88.54 | 1948.9829 | 16 | -0.1 | 650.6682 | 3 | 12.93 | 2 | F2:3417 | DaRuMH\_F10.raw |  | 6.0396E7 |  |  |  |  |  |  |  |  | 2 | 0 | 2 | 0 | 0 | 0 | 0 | 0 | 0 | 0 | 0 | 386 | 401 | Carbamidomethylation | C9:Carbamidomethylation:1000.00 | PEAKS DB |
| R.TWVFELVNTINEIFK.Y | Y | 85.82 | 1851.9771 | 15 | 0.2 | 926.9960 | 2 | 80.27 | 2 | F2:18694 | DaRuMH\_F10.raw |  | 9.412E6 |  |  |  |  |  |  |  |  | 2 | 0 | 2 | 0 | 0 | 0 | 0 | 0 | 0 | 0 | 0 | 228 | 242 |  |  | PEAKS DB |
| R.YFNPYSYVELIITVDHSMVTK.Y | Y | 85.35 | 2518.2454 | 21 | 0.1 | 840.4225 | 3 | 59.02 | 2 | F2:15114 | DaRuMH\_F10.raw |  | 1.5949E6 |  |  |  |  |  |  |  |  | 1 | 0 | 1 | 0 | 0 | 0 | 0 | 0 | 0 | 0 | 0 | 198 | 218 |  |  | PEAKS DB |
| R.TDIVSPPAC(+57.02)GNELLER.G | Y | 84.82 | 1769.8618 | 16 | 1.0 | 590.9618 | 3 | 13.61 | 2 | F2:3692 | DaRuMH\_F10.raw |  | 6.6778E7 |  |  |  |  |  |  |  |  | 2 | 0 | 2 | 0 | 0 | 0 | 0 | 0 | 0 | 0 | 0 | 402 | 417 | Carbamidomethylation | C9:Carbamidomethylation:1000.00 | PEAKS DB |
| R.YFNPYSYVELIITVDHSM(+15.99)VTK.Y | Y | 84.67 | 2534.2402 | 21 | 0.4 | 845.7543 | 3 | 50.13 | 2 | F2:13232 | DaRuMH\_F10.raw |  | 1.7123E6 |  |  |  |  |  |  |  |  | 1 | 0 | 1 | 0 | 0 | 0 | 0 | 0 | 0 | 0 | 0 | 198 | 218 | Oxidation (M) | M18:Oxidation (M):1000.00 | PEAKS DB |
| K.DLFSEDYSETHYSPDGR.E | N | 83.02 | 2016.8336 | 17 | 4.3 | 1009.4227 | 2 | 12.44 | 4 | F4:3205 | DaRuMH\_F3.raw |  |  |  | 2.316E6 | 5.9298E4 |  |  |  |  |  | 3 | 0 | 0 | 0 | 2 | 1 | 0 | 0 | 0 | 0 | 0 | 78 | 94 |  |  | PEAKS DB |
| K.YSVGVVQDHSK.I | Y | 82.85 | 1217.6040 | 11 | 1.3 | 609.8101 | 2 | 10.96 | 2 | F2:1819 | DaRuMH\_F10.raw |  | 1.9579E6 |  |  |  |  |  |  |  |  | 2 | 0 | 2 | 0 | 0 | 0 | 0 | 0 | 0 | 0 | 0 | 318 | 328 |  |  | PEAKS DB |
| K.LHSWVEC(+57.02)ESGK.C | Y | 80.88 | 1330.5975 | 11 | 0.2 | 666.3062 | 2 | 11.13 | 2 | F2:1973 | DaRuMH\_F10.raw |  | 3.6602E6 |  | 1.0231E5 |  |  |  |  |  |  | 3 | 0 | 2 | 0 | 1 | 0 | 0 | 0 | 0 | 0 | 0 | 441 | 451 | Carbamidomethylation | C7:Carbamidomethylation:1000.00 | PEAKS DB |
| R.SANC(+57.02)PVDEFHENGR.P | Y | 80.53 | 1630.6794 | 14 | -0.1 | 816.3469 | 2 | 11.11 | 2 | F2:1974 | DaRuMH\_F10.raw |  | 3.2355E5 |  |  |  |  |  |  |  |  | 1 | 0 | 1 | 0 | 0 | 0 | 0 | 0 | 0 | 0 | 0 | 482 | 495 | Carbamidomethylation | C4:Carbamidomethylation:1000.00 | PEAKS DB |
| E.SGNVNDYEVVYPQK.V | N | 80.53 | 1610.7576 | 14 | 1.1 | 806.3870 | 2 | 11.73 | 3 | F3:2451 | DaRuMH\_F2.raw |  |  | 3.1229E5 |  |  |  |  |  |  |  | 1 | 0 | 0 | 1 | 0 | 0 | 0 | 0 | 0 | 0 | 0 | 25 | 38 |  |  | PEAKS DB |
| S.GNVNDYEVVYPQK.V | N | 78.53 | 1523.7256 | 13 | 0.7 | 762.8706 | 2 | 11.75 | 3 | F3:2472 | DaRuMH\_F2.raw |  |  | 1.2901E5 |  |  |  |  |  |  |  | 1 | 0 | 0 | 1 | 0 | 0 | 0 | 0 | 0 | 0 | 0 | 26 | 38 |  |  | PEAKS DB |
| Y.SC(+57.02)IMSAVLGDQPSK.Y | Y | 77.03 | 1491.7062 | 14 | 0.3 | 746.8606 | 2 | 14.02 | 2 | F2:3935 | DaRuMH\_F10.raw |  | 5.6488E5 |  |  |  |  |  |  |  |  | 1 | 0 | 1 | 0 | 0 | 0 | 0 | 0 | 0 | 0 | 0 | 360 | 373 | Carbamidomethylation | C2:Carbamidomethylation:1000.00 | PEAKS DB |
| R.GEEC(+57.02)DC(+57.02)GSPENC(+57.02)R.D | Y | 72.06 | 1568.5289 | 13 | -0.4 | 785.2714 | 2 | 10.65 | 2 | F2:1579 | DaRuMH\_F10.raw |  | 4.8085E6 |  |  |  |  |  |  |  |  | 1 | 0 | 1 | 0 | 0 | 0 | 0 | 0 | 0 | 0 | 0 | 418 | 430 | Carbamidomethylation | C4:Carbamidomethylation:1000.00;C6:Carbamidomethylation:1000.00;C12:Carbamidomethylation:1000.00 | PEAKS DB |
| Y.SC(+57.02)IM(+15.99)SAVLGDQPSK.Y | Y | 68.09 | 1507.7010 | 14 | 1.1 | 754.8586 | 2 | 11.58 | 2 | F2:2415 | DaRuMH\_F10.raw |  | 2.2927E5 |  |  |  |  |  |  |  |  | 1 | 0 | 1 | 0 | 0 | 0 | 0 | 0 | 0 | 0 | 0 | 360 | 373 | Carbamidomethylation; Oxidation (M) | C2:Carbamidomethylation:1000.00;M4:Oxidation (M):1000.00 | PEAKS DB |
| R.VPLVGLEIWK.N | Y | 67.75 | 1152.6907 | 10 | 0.4 | 577.3528 | 2 | 32.05 | 2 | F2:8853 | DaRuMH\_F10.raw |  | 7.6235E7 |  |  |  |  |  |  |  |  | 1 | 0 | 1 | 0 | 0 | 0 | 0 | 0 | 0 | 0 | 0 | 248 | 257 |  |  | PEAKS DB |
| R.GEEC(+57.02)DC(+57.02)GSPENC(+57.02)RDPC(+57.02)C(+57.02)DAASC(+57.02)K.L | Y | 67.55 | 2732.9287 | 23 | 0.4 | 911.9839 | 3 | 10.76 | 2 | F2:1657 | DaRuMH\_F10.raw |  | 1.4656E6 |  |  |  |  |  |  |  |  | 1 | 0 | 1 | 0 | 0 | 0 | 0 | 0 | 0 | 0 | 0 | 418 | 440 | Carbamidomethylation | C4:Carbamidomethylation:1000.00;C6:Carbamidomethylation:1000.00;C12:Carbamidomethylation:1000.00;C16:Carbamidomethylation:1000.00;C17:Carbamidomethylation:1000.00;C22:Carbamidomethylation:1000.00 | PEAKS DB |
| Y.SYVELIITVDHSMVTK.Y | Y | 67.30 | 1833.9546 | 16 | 0.9 | 612.3260 | 3 | 27.18 | 2 | F2:7658 | DaRuMH\_F10.raw |  | 2.4247E6 |  |  |  |  |  |  |  |  | 2 | 0 | 2 | 0 | 0 | 0 | 0 | 0 | 0 | 0 | 0 | 203 | 218 |  |  | PEAKS DB |
| C.IMSAVLGDQPSK.Y | Y | 62.78 | 1244.6434 | 12 | 0.2 | 623.3291 | 2 | 11.69 | 2 | F2:2540 | DaRuMH\_F10.raw |  | 3.4513E5 |  |  |  |  |  |  |  |  | 1 | 0 | 1 | 0 | 0 | 0 | 0 | 0 | 0 | 0 | 0 | 362 | 373 |  |  | PEAKS DB |
| V.TQTNWESDEPIK.K | N | 62.34 | 1446.6627 | 12 | 0.2 | 724.3387 | 2 | 11.62 | 2 | F2:2463 | DaRuMH\_F10.raw |  | 8.3861E4 |  |  |  |  |  |  |  |  | 1 | 0 | 1 | 0 | 0 | 0 | 0 | 0 | 0 | 0 | 0 | 173 | 184 |  |  | PEAKS DB |
| R.SANC(+57.02)PVDEFHENGRPC(+57.02).L | Y | 60.86 | 1887.7628 | 16 | 0.2 | 944.8889 | 2 | 11.26 | 2 | F2:2122 | DaRuMH\_F10.raw |  | 6.1615E4 |  |  |  |  |  |  |  |  | 1 | 0 | 1 | 0 | 0 | 0 | 0 | 0 | 0 | 0 | 0 | 482 | 497 | Carbamidomethylation | C4:Carbamidomethylation:1000.00;C16:Carbamidomethylation:1000.00 | PEAKS DB |
| R.SANC(+57.02)PVDEFHEN.G | Y | 60.40 | 1417.5568 | 12 | 0.7 | 709.7861 | 2 | 11.44 | 2 | F2:2291 | DaRuMH\_F10.raw |  | 3.8885E5 |  |  |  |  |  |  |  |  | 1 | 0 | 1 | 0 | 0 | 0 | 0 | 0 | 0 | 0 | 0 | 482 | 493 | Carbamidomethylation | C4:Carbamidomethylation:1000.00 | PEAKS DB |
| R.LGVYYAYC(+57.02)R.K | Y | 60.18 | 1163.5433 | 9 | 0.8 | 582.7794 | 2 | 11.90 | 2 | F2:2732 | DaRuMH\_F10.raw |  | 9.1321E4 |  |  |  |  |  |  |  |  | 1 | 0 | 1 | 0 | 0 | 0 | 0 | 0 | 0 | 0 | 0 | 537 | 545 | Carbamidomethylation | C8:Carbamidomethylation:1000.00 | PEAKS DB |
| K.YSVGVVQDH.S | Y | 59.92 | 1002.4771 | 9 | -0.3 | 502.2456 | 2 | 11.44 | 2 | F2:2309 | DaRuMH\_F10.raw |  | 2.5871E5 |  |  |  |  |  |  |  |  | 1 | 0 | 1 | 0 | 0 | 0 | 0 | 0 | 0 | 0 | 0 | 318 | 326 |  |  | PEAKS DB |
| R.TDIVSPPAC(+57.02)GN.E | Y | 59.10 | 1129.5073 | 11 | 1.2 | 565.7616 | 2 | 11.85 | 2 | F2:2629 | DaRuMH\_F10.raw |  | 1.5179E6 |  |  |  |  |  |  |  |  | 1 | 0 | 1 | 0 | 0 | 0 | 0 | 0 | 0 | 0 | 0 | 402 | 412 | Carbamidomethylation | C9:Carbamidomethylation:1000.00 | PEAKS DB |
| K.ASLLVATSER.N | Y | 58.81 | 1045.5768 | 10 | 0.3 | 523.7958 | 2 | 11.69 | 2 | F2:2520 | DaRuMH\_F10.raw |  | 1.5224E6 |  |  |  |  |  |  |  |  | 1 | 0 | 1 | 0 | 0 | 0 | 0 | 0 | 0 | 0 | 0 | 186 | 195 |  |  | PEAKS DB |
| T.C(+57.02)GGYSC(+57.02)IMSAVLGDQPSK.Y | Y | 57.54 | 1928.8430 | 18 | 1.3 | 965.4301 | 2 | 24.52 | 2 | F2:7085 | DaRuMH\_F10.raw |  | 1.1633E4 |  |  |  |  |  |  |  |  | 1 | 0 | 1 | 0 | 0 | 0 | 0 | 0 | 0 | 0 | 0 | 356 | 373 | Carbamidomethylation | C1:Carbamidomethylation:1000.00;C6:Carbamidomethylation:1000.00 | PEAKS DB |
| R.SANC(+57.02)PVDEFHENGRPC(+57.02)LHNFGY.C | Y | 57.31 | 2619.1018 | 22 | 0.6 | 874.0417 | 3 | 11.83 | 2 | F2:2647 | DaRuMH\_F10.raw |  | 3.8481E5 |  |  |  |  |  |  |  |  | 1 | 0 | 1 | 0 | 0 | 0 | 0 | 0 | 0 | 0 | 0 | 482 | 503 | Carbamidomethylation | C4:Carbamidomethylation:1000.00;C16:Carbamidomethylation:1000.00 | PEAKS DB |
| K.YKNDLTAIR.T | Y | 55.88 | 1092.5928 | 9 | -0.4 | 547.3035 | 2 | 11.14 | 2 | F2:2001 | DaRuMH\_F10.raw |  | 2.4313E5 |  |  |  |  |  |  |  |  | 1 | 0 | 1 | 0 | 0 | 0 | 0 | 0 | 0 | 0 | 0 | 219 | 227 |  |  | PEAKS DB |
| Y.SYVELIITVDHSM(+15.99)VTK.Y | Y | 55.46 | 1849.9496 | 16 | 0.0 | 617.6572 | 3 | 17.55 | 2 | F2:5189 | DaRuMH\_F10.raw |  | 1.5947E6 |  |  |  |  |  |  |  |  | 1 | 0 | 1 | 0 | 0 | 0 | 0 | 0 | 0 | 0 | 0 | 203 | 218 | Oxidation (M) | M13:Oxidation (M):1000.00 | PEAKS DB |
| K.VC(+57.02)SNGQC(+57.02)VDLNIAY | Y | 55.08 | 1611.7021 | 14 | 1.3 | 806.8594 | 2 | 19.99 | 2 | F2:5815 | DaRuMH\_F10.raw |  | 1.2231E7 |  |  |  |  |  |  |  |  | 1 | 0 | 1 | 0 | 0 | 0 | 0 | 0 | 0 | 0 | 0 | 602 | 615 | Carbamidomethylation | C2:Carbamidomethylation:1000.00;C7:Carbamidomethylation:1000.00 | PEAKS DB |
| R.SANC(+57.02)PVDEFHENGRPC(+57.02)LH.N | Y | 54.95 | 2137.9058 | 18 | -0.4 | 535.4835 | 4 | 11.31 | 2 | F2:2162 | DaRuMH\_F10.raw |  | 3.8745E5 |  |  |  |  |  |  |  |  | 1 | 0 | 1 | 0 | 0 | 0 | 0 | 0 | 0 | 0 | 0 | 482 | 499 | Carbamidomethylation | C4:Carbamidomethylation:1000.00;C16:Carbamidomethylation:1000.00 | PEAKS DB |
| V.TQTNWESDEPIKK.A | N | 54.81 | 1574.7576 | 13 | 0.6 | 525.9268 | 3 | 11.17 | 2 | F2:2030 | DaRuMH\_F10.raw |  | 1.6419E5 |  |  |  |  |  |  |  |  | 1 | 0 | 1 | 0 | 0 | 0 | 0 | 0 | 0 | 0 | 0 | 173 | 185 |  |  | PEAKS DB |
| R.DEC(+57.02)DKAEQC(+57.02)TGR.S | Y | 54.45 | 1467.5718 | 12 | 5.9 | 490.1979 | 3 | 10.70 | 4 | F4:1581 | DaRuMH\_F3.raw |  |  |  | 1.6558E4 |  |  |  |  |  |  | 1 | 0 | 0 | 0 | 1 | 0 | 0 | 0 | 0 | 0 | 0 | 470 | 481 | Carbamidomethylation | C3:Carbamidomethylation:1000.00;C9:Carbamidomethylation:1000.00 | PEAKS DB |
| F.ELVNTINEIFK.Y | Y | 53.90 | 1318.7133 | 11 | 0.2 | 660.3640 | 2 | 26.32 | 2 | F2:7455 | DaRuMH\_F10.raw |  | 2.4728E6 |  |  |  |  |  |  |  |  | 1 | 0 | 1 | 0 | 0 | 0 | 0 | 0 | 0 | 0 | 0 | 232 | 242 |  |  | PEAKS DB |
| total 35 peptides |
| --- |

P81458|PA2B\_DABRR

back to list

  

| Protein Coverage
| Supporting Peptides
|

Protein Coverage:

Supporting Peptides:

| Peptide | Uniq | -10lgP | Mass | Length | ppm | m/z | z | RT | Fraction | Scan | Source File | Area F1 | Area F10 | Area F2 | Area F3 | Area F4 | Area F5 | Area F7A | Area F7B | Area F8 | Area F9 | #Feature | #Feature F1 | #Feature F10 | #Feature F2 | #Feature F3 | #Feature F4 | #Feature F5 | #Feature F7A | #Feature F7B | #Feature F8 | #Feature F9 | Start | End | PTM | AScore | Found By |
| --- | --- | --- | --- | --- | --- | --- | --- | --- | --- | --- | --- | --- | --- | --- | --- | --- | --- | --- | --- | --- | --- | --- | --- | --- | --- | --- | --- | --- | --- | --- | --- | --- | --- | --- | --- | --- | --- |
| R.AVC(+57.02)EC(+57.02)DRVAATC(+57.02)FR.D | N | 100.61 | 1713.7385 | 14 | 1.2 | 857.8776 | 2 | 11.27 | 6 | F6:2110 | DaRuMH\_F5.raw |  |  |  |  |  | 4.9245E6 | 1.9768E5 |  |  |  | 3 | 0 | 0 | 0 | 0 | 0 | 2 | 1 | 0 | 0 | 0 | 84 | 97 | Carbamidomethylation | C3:Carbamidomethylation:1000.00;C5:Carbamidomethylation:1000.00;C12:Carbamidomethylation:1000.00 | PEAKS DB |
| K.NPLSSYSDYGC(+57.02)YC(+57.02)GWGGK.G | Y | 100.37 | 2069.8247 | 18 | 5.1 | 1035.9249 | 2 | 15.61 | 8 | F8:6011 | DaRuMH\_F7B.raw |  |  |  |  |  | 1.926E8 | 1.2526E9 | 4.9279E7 | 1.8125E7 |  | 8 | 0 | 0 | 0 | 0 | 0 | 3 | 2 | 2 | 1 | 0 | 16 | 33 | Carbamidomethylation | C11:Carbamidomethylation:1000.00;C13:Carbamidomethylation:1000.00 | PEAKS DB |
| K.LSLYSYSFQNGGIVC(+57.02)GDNHSC(+57.02)K.R | Y | 91.40 | 2505.1052 | 22 | 2.8 | 836.0427 | 3 | 15.50 | 7 | F7:6013 | DaRuMH\_F7A.raw |  |  |  |  |  | 1.7918E7 | 1.8326E8 | 7.436E7 | 2.5474E6 |  | 7 | 0 | 0 | 0 | 0 | 0 | 1 | 3 | 2 | 1 | 0 | 61 | 82 | Carbamidomethylation | C15:Carbamidomethylation:1000.00;C21:Carbamidomethylation:1000.00 | PEAKS DB |
| Y.SFQNGGIVC(+57.02)GDNHSC(+57.02)K.R | Y | 89.79 | 1778.7465 | 16 | -2.3 | 890.3784 | 2 | 10.97 | 8 | F8:1900 | DaRuMH\_F7B.raw |  |  |  |  |  | 5.1106E6 | 1.8601E7 | 1.8083E7 | 1.0219E6 |  | 8 | 0 | 0 | 0 | 0 | 0 | 2 | 2 | 2 | 2 | 0 | 67 | 82 | Carbamidomethylation | C9:Carbamidomethylation:1000.00;C15:Carbamidomethylation:1000.00 | PEAKS DB |
| K.LSLYSYSFQNGGIVC(+57.02)GDNHSC(+57.02)KR.A | Y | 88.24 | 2661.2063 | 23 | 2.2 | 666.3103 | 4 | 12.19 | 6 | F6:2928 | DaRuMH\_F5.raw |  |  |  |  |  | 3.9279E6 | 5.3708E6 | 7.046E6 | 2.4668E5 |  | 7 | 0 | 0 | 0 | 0 | 0 | 2 | 2 | 2 | 1 | 0 | 61 | 83 | Carbamidomethylation | C15:Carbamidomethylation:1000.00;C21:Carbamidomethylation:1000.00 | PEAKS DB |
| Y.SYSFQNGGIVC(+57.02)GDNHSC(+57.02)K.R | Y | 87.13 | 2028.8418 | 18 | 0.6 | 1015.4288 | 2 | 11.43 | 8 | F8:2333 | DaRuMH\_F7B.raw |  |  |  |  |  | 9.2566E6 | 2.8762E7 | 8.3297E6 | 1.249E6 |  | 8 | 0 | 0 | 0 | 0 | 0 | 2 | 2 | 2 | 2 | 0 | 65 | 82 | Carbamidomethylation | C11:Carbamidomethylation:1000.00;C17:Carbamidomethylation:1000.00 | PEAKS DB |
| L.SSYSDYGC(+57.02)YC(+57.02)GWGGK.G | Y | 84.79 | 1745.6450 | 15 | 0.9 | 873.8306 | 2 | 11.97 | 6 | F6:2734 | DaRuMH\_F5.raw |  |  |  |  |  | 4.567E6 | 1.2671E6 | 1.5233E6 |  |  | 3 | 0 | 0 | 0 | 0 | 0 | 1 | 1 | 1 | 0 | 0 | 19 | 33 | Carbamidomethylation | C8:Carbamidomethylation:1000.00;C10:Carbamidomethylation:1000.00 | PEAKS DB |
| R.VAATC(+57.02)FRDNLNTYDKK.Y | N | 83.45 | 1914.9258 | 16 | 0.3 | 639.3160 | 3 | 11.26 | 6 | F6:2101 | DaRuMH\_F5.raw |  |  |  |  |  | 8.1557E6 | 1.2526E5 |  |  |  | 3 | 0 | 0 | 0 | 0 | 0 | 2 | 1 | 0 | 0 | 0 | 91 | 106 | Carbamidomethylation | C5:Carbamidomethylation:1000.00 | PEAKS DB |
| K.YHNYPPSQC(+57.02)TGTEQC(+57.02) | Y | 79.21 | 1840.7145 | 15 | -1.3 | 921.3633 | 2 | 10.87 | 6 | F6:1730 | DaRuMH\_F5.raw |  |  |  |  |  | 4.26E7 | 1.9271E7 | 8.8939E6 | 5.584E6 |  | 4 | 0 | 0 | 0 | 0 | 0 | 1 | 1 | 1 | 1 | 0 | 107 | 121 | Carbamidomethylation | C9:Carbamidomethylation:1000.00;C15:Carbamidomethylation:1000.00 | PEAKS DB |
| NLFQFAEMIVK.M | N | 74.29 | 1338.7006 | 11 | 0.8 | 670.3581 | 2 | 47.10 | 8 | F8:24564 | DaRuMH\_F7B.raw |  |  |  |  |  | 6.0127E7 | 6.7566E7 | 2.1668E7 | 6.6474E6 | 1.2473E8 | 5 | 0 | 0 | 0 | 0 | 0 | 1 | 1 | 1 | 1 | 1 | 1 | 11 |  |  | PEAKS DB |
| K.NPLSSYSDYGC(+57.02)YC(+57.02)GWGGKG.K | Y | 73.29 | 2126.8462 | 19 | -0.4 | 1064.4299 | 2 | 15.09 | 8 | F8:5991 | DaRuMH\_F7B.raw |  |  |  |  |  | 1.6278E7 |  | 4.1252E6 |  |  | 4 | 0 | 0 | 0 | 0 | 0 | 2 | 0 | 2 | 0 | 0 | 16 | 34 | Carbamidomethylation | C11:Carbamidomethylation:1000.00;C13:Carbamidomethylation:1000.00 | PEAKS DB |
| NLFQFAEM(+15.99)IVK.M | N | 73.14 | 1354.6954 | 11 | 0.1 | 678.3550 | 2 | 40.13 | 10 | F10:19775 | DaRuMH\_F9.raw |  |  |  |  |  | 1.265E7 | 7.6352E6 | 1.1953E6 | 1.7887E6 | 5.3762E7 | 11 | 0 | 0 | 0 | 0 | 0 | 4 | 2 | 1 | 1 | 3 | 1 | 11 | Oxidation (M) | M8:Oxidation (M):1000.00 | PEAKS DB |
| K.MTGKNPLSSYSDYGC(+57.02)YC(+57.02)GWGGK.G | Y | 71.30 | 2487.0293 | 22 | 0.7 | 830.0176 | 3 | 12.55 | 6 | F6:3198 | DaRuMH\_F5.raw |  |  |  |  |  | 5.599E5 | 1.0352E5 |  |  |  | 2 | 0 | 0 | 0 | 0 | 0 | 1 | 1 | 0 | 0 | 0 | 12 | 33 | Carbamidomethylation | C15:Carbamidomethylation:1000.00;C17:Carbamidomethylation:1000.00 | PEAKS DB |
| S.YSDYGC(+57.02)YC(+57.02)GWGGK.G | N | 70.24 | 1571.5809 | 13 | 0.5 | 786.7981 | 2 | 11.87 | 6 | F6:2651 | DaRuMH\_F5.raw |  |  |  |  |  | 2.4169E5 |  |  |  |  | 1 | 0 | 0 | 0 | 0 | 0 | 1 | 0 | 0 | 0 | 0 | 21 | 33 | Carbamidomethylation | C6:Carbamidomethylation:1000.00;C8:Carbamidomethylation:1000.00 | PEAKS DB |
| D.YGC(+57.02)YC(+57.02)GWGGK.G | N | 66.85 | 1206.4586 | 10 | -0.3 | 604.2364 | 2 | 11.56 | 8 | F8:2453 | DaRuMH\_F7B.raw |  |  |  |  | 2.4701E5 | 2.1367E7 | 1.6469E7 | 7.7105E6 |  |  | 4 | 0 | 0 | 0 | 0 | 1 | 1 | 1 | 1 | 0 | 0 | 24 | 33 | Carbamidomethylation | C3:Carbamidomethylation:1000.00;C5:Carbamidomethylation:1000.00 | PEAKS DB |
| N.PLSSYSDYGC(+57.02)YC(+57.02)GWGGK.G | Y | 65.30 | 1955.7819 | 17 | 1.5 | 978.8997 | 2 | 13.32 | 6 | F6:3782 | DaRuMH\_F5.raw |  |  |  |  |  | 2.2719E5 |  |  |  |  | 1 | 0 | 0 | 0 | 0 | 0 | 1 | 0 | 0 | 0 | 0 | 17 | 33 | Carbamidomethylation | C10:Carbamidomethylation:1000.00;C12:Carbamidomethylation:1000.00 | PEAKS DB |
| L.YSYSFQNGGIVC(+57.02)GDNHSC(+57.02)K.R | Y | 63.86 | 2191.9050 | 19 | 0.6 | 731.6427 | 3 | 11.62 | 8 | F8:2541 | DaRuMH\_F7B.raw |  |  |  |  |  |  |  | 4.906E5 |  |  | 1 | 0 | 0 | 0 | 0 | 0 | 0 | 0 | 1 | 0 | 0 | 64 | 82 | Carbamidomethylation | C12:Carbamidomethylation:1000.00;C18:Carbamidomethylation:1000.00 | PEAKS DB |
| P.LSSYSDYGC(+57.02)YC(+57.02)GWGGK.G | Y | 62.39 | 1858.7290 | 16 | 0.4 | 930.3722 | 2 | 12.28 | 6 | F6:3016 | DaRuMH\_F5.raw |  |  |  |  |  | 2.8345E5 |  |  |  |  | 1 | 0 | 0 | 0 | 0 | 0 | 1 | 0 | 0 | 0 | 0 | 18 | 33 | Carbamidomethylation | C9:Carbamidomethylation:1000.00;C11:Carbamidomethylation:1000.00 | PEAKS DB |
| Y.SDYGC(+57.02)YC(+57.02)GWGGK.G | N | 62.29 | 1408.5176 | 12 | -0.6 | 705.2656 | 2 | 11.73 | 6 | F6:2540 | DaRuMH\_F5.raw |  |  |  |  |  | 1.1062E6 | 5.2638E5 |  | 1.2785E5 |  | 4 | 0 | 0 | 0 | 0 | 0 | 2 | 1 | 0 | 1 | 0 | 22 | 33 | Carbamidomethylation | C5:Carbamidomethylation:1000.00;C7:Carbamidomethylation:1000.00 | PEAKS DB |
| R.C(+57.02)C(+57.02)FVHDC(+57.02)C(+57.02)YEK.V | N | 58.97 | 1576.5568 | 11 | -1.4 | 789.2845 | 2 | 11.02 | 8 | F8:1930 | DaRuMH\_F7B.raw |  |  |  |  |  |  | 8.6121E7 | 5.3269E7 | 3.4024E6 |  | 5 | 0 | 0 | 0 | 0 | 0 | 0 | 1 | 2 | 2 | 0 | 43 | 53 | Carbamidomethylation | C1:Carbamidomethylation:1000.00;C2:Carbamidomethylation:1000.00;C7:Carbamidomethylation:1000.00;C8:Carbamidomethylation:1000.00 | PEAKS DB |
| Y.GC(+57.02)YC(+57.02)GWGGK.G | N | 58.54 | 1043.3953 | 9 | -0.4 | 522.7047 | 2 | 11.29 | 6 | F6:2113 | DaRuMH\_F5.raw |  |  |  |  |  | 2.1095E6 |  | 4.3981E6 |  |  | 2 | 0 | 0 | 0 | 0 | 0 | 1 | 0 | 1 | 0 | 0 | 25 | 33 | Carbamidomethylation | C2:Carbamidomethylation:1000.00;C4:Carbamidomethylation:1000.00 | PEAKS DB |
| S.FQNGGIVC(+57.02)GDNHSC(+57.02)K.R | Y | 57.56 | 1691.7144 | 15 | 0.8 | 564.9125 | 3 | 10.81 | 8 | F8:1767 | DaRuMH\_F7B.raw |  |  |  |  |  | 1.192E5 |  | 2.0899E5 |  |  | 2 | 0 | 0 | 0 | 0 | 0 | 1 | 0 | 1 | 0 | 0 | 68 | 82 | Carbamidomethylation | C8:Carbamidomethylation:1000.00;C14:Carbamidomethylation:1000.00 | PEAKS DB |
| R.VAATC(+57.02)FR.D | N | 55.30 | 823.4011 | 7 | -0.5 | 412.7076 | 2 | 11.06 | 9 | F9:1884 | DaRuMH\_F8.raw |  |  |  |  |  |  |  |  | 3.3394E5 |  | 1 | 0 | 0 | 0 | 0 | 0 | 0 | 0 | 0 | 1 | 0 | 91 | 97 | Carbamidomethylation | C5:Carbamidomethylation:1000.00 | PEAKS DB |
| total 23 peptides |
| --- |

P35527|K1C9\_HUMAN

back to list

  

| Protein Coverage
| Supporting Peptides
|

Protein Coverage:

Supporting Peptides:

| Peptide | Uniq | -10lgP | Mass | Length | ppm | m/z | z | RT | Fraction | Scan | Source File | Area F1 | Area F10 | Area F2 | Area F3 | Area F4 | Area F5 | Area F7A | Area F7B | Area F8 | Area F9 | #Feature | #Feature F1 | #Feature F10 | #Feature F2 | #Feature F3 | #Feature F4 | #Feature F5 | #Feature F7A | #Feature F7B | #Feature F8 | #Feature F9 | Start | End | PTM | AScore | Found By |
| --- | --- | --- | --- | --- | --- | --- | --- | --- | --- | --- | --- | --- | --- | --- | --- | --- | --- | --- | --- | --- | --- | --- | --- | --- | --- | --- | --- | --- | --- | --- | --- | --- | --- | --- | --- | --- | --- |
| R.GGSGGSYGGGGSGGGYGGGSGSR.G | Y | 102.54 | 1790.7203 | 23 | -1.4 | 896.3662 | 2 | 10.74 | 1 | F1:1625 | DaRuMH\_F1.raw | 3.1532E6 |  |  |  | 2.3772E5 |  |  |  |  | 6.0978E5 | 3 | 1 | 0 | 0 | 0 | 1 | 0 | 0 | 0 | 0 | 1 | 491 | 513 |  |  | PEAKS DB |
| R.SGGGGGGGLGSGGSIR.S | Y | 96.73 | 1231.5905 | 16 | 0.0 | 616.8025 | 2 | 11.01 | 1 | F1:1890 | DaRuMH\_F1.raw | 3.4911E5 |  | 7.784E4 | 2.3247E5 | 1.6241E5 |  |  |  | 1.3865E4 | 1.8478E5 | 6 | 1 | 0 | 1 | 1 | 1 | 0 | 0 | 0 | 1 | 1 | 14 | 29 |  |  | PEAKS DB |
| K.VQALEEANNDLENK.I | Y | 94.31 | 1585.7583 | 14 | 1.4 | 793.8875 | 2 | 11.55 | 1 | F1:2411 | DaRuMH\_F1.raw | 1.6405E6 |  |  |  | 2.6523E5 | 1.33E5 |  |  |  | 1.0185E6 | 5 | 2 | 0 | 0 | 0 | 1 | 1 | 0 | 0 | 0 | 1 | 171 | 184 |  |  | PEAKS DB |
| F.GGFGGGAGGGDGGILTANEK.S | Y | 83.88 | 1690.7910 | 20 | 0.0 | 846.4028 | 2 | 11.95 | 1 | F1:2727 | DaRuMH\_F1.raw | 5.6604E5 |  |  |  |  |  |  |  |  |  | 1 | 1 | 0 | 0 | 0 | 0 | 0 | 0 | 0 | 0 | 0 | 135 | 154 |  |  | PEAKS DB |
| R.QGVDADINGLR.Q | Y | 83.58 | 1156.5836 | 11 | -1.7 | 579.2981 | 2 | 11.71 | 10 | F10:2527 | DaRuMH\_F9.raw | 1.4479E6 |  |  | 1.8378E5 | 3.3854E5 | 1.1176E5 |  |  | 1.2685E5 | 8.2457E5 | 6 | 1 | 0 | 0 | 1 | 1 | 1 | 0 | 0 | 1 | 1 | 251 | 261 |  |  | PEAKS DB |
| Y.SYGGGSGGGFSASSLGGGFGGGSR.G | Y | 79.12 | 2021.8827 | 24 | 1.0 | 1011.9496 | 2 | 12.52 | 6 | F6:3214 | DaRuMH\_F5.raw | 1.2258E6 |  |  |  |  | 1.2873E5 |  |  |  | 1.2633E6 | 3 | 1 | 0 | 0 | 0 | 0 | 1 | 0 | 0 | 0 | 1 | 72 | 95 |  |  | PEAKS DB |
| F.SASSLGGGFGGGSR.G | Y | 74.59 | 1195.5581 | 14 | -3.2 | 598.7844 | 2 | 11.34 | 10 | F10:2193 | DaRuMH\_F9.raw |  |  |  |  |  |  |  |  |  | 1.4705E5 | 1 | 0 | 0 | 0 | 0 | 0 | 0 | 0 | 0 | 0 | 1 | 82 | 95 |  |  | PEAKS DB |
| K.DQIVDLTVGNNK.T | Y | 72.45 | 1314.6780 | 12 | -0.7 | 658.3458 | 2 | 12.46 | 10 | F10:3206 | DaRuMH\_F9.raw | 7.1038E5 | 2.7313E4 |  | 5.1187E4 | 1.5865E5 | 7.1205E4 |  |  |  | 9.4154E5 | 6 | 1 | 1 | 0 | 1 | 1 | 1 | 0 | 0 | 0 | 1 | 213 | 224 |  |  | PEAKS DB |
| Q.ISNLEAQITDVR.Q | Y | 70.65 | 1357.7201 | 12 | 0.5 | 679.8677 | 2 | 12.21 | 10 | F10:3002 | DaRuMH\_F9.raw | 2.5809E5 |  |  |  | 4.7899E4 |  |  |  |  | 4.5958E5 | 3 | 1 | 0 | 0 | 0 | 1 | 0 | 0 | 0 | 0 | 1 | 416 | 427 |  |  | PEAKS DB |
| R.QEYEQLIAK.N | Y | 68.77 | 1120.5764 | 9 | -0.7 | 561.2951 | 2 | 11.64 | 10 | F10:2474 | DaRuMH\_F9.raw | 1.6877E6 |  |  | 1.8677E5 | 3.7072E5 | 9.3099E4 |  |  | 1.0188E5 | 6.3727E5 | 6 | 1 | 0 | 0 | 1 | 1 | 1 | 0 | 0 | 1 | 1 | 328 | 336 |  |  | PEAKS DB |
| R.QVLDNLTMEK.S | Y | 67.53 | 1189.6013 | 10 | -0.4 | 595.8077 | 2 | 11.95 | 1 | F1:2825 | DaRuMH\_F1.raw | 1.9312E5 |  |  |  |  |  |  |  |  |  | 1 | 1 | 0 | 0 | 0 | 0 | 0 | 0 | 0 | 0 | 0 | 262 | 271 |  |  | PEAKS DB |
| K.EIETYHNLLEGGQEDFESSGAGK.I | Y | 66.03 | 2509.1245 | 23 | 1.3 | 837.3832 | 3 | 12.93 | 10 | F10:3713 | DaRuMH\_F9.raw | 1.7592E6 |  |  |  |  | 3.1765E5 |  |  |  | 1.9344E6 | 3 | 1 | 0 | 0 | 0 | 0 | 1 | 0 | 0 | 0 | 1 | 450 | 472 |  |  | PEAKS DB |
| R.FSSSSGYGGGSSR.V | Y | 62.40 | 1234.5214 | 13 | -1.4 | 618.2671 | 2 | 10.74 | 1 | F1:1641 | DaRuMH\_F1.raw | 1.6901E6 |  |  |  |  |  |  |  |  |  | 1 | 1 | 0 | 0 | 0 | 0 | 0 | 0 | 0 | 0 | 0 | 47 | 59 |  |  | PEAKS DB |
| K.SDLEM(+15.99)QYETLQEELM(+15.99)ALK.K | Y | 61.95 | 2202.0071 | 18 | 0.4 | 1102.0112 | 2 | 43.40 | 10 | F10:21386 | DaRuMH\_F9.raw |  |  |  |  |  |  |  |  |  | 7.8544E5 | 1 | 0 | 0 | 0 | 0 | 0 | 0 | 0 | 0 | 0 | 1 | 272 | 289 | Oxidation (M) | M5:Oxidation (M):1000.00;M15:Oxidation (M):1000.00 | PEAKS DB |
| M.IQEQISNLEAQITDVR.Q | Y | 61.25 | 1855.9639 | 16 | 1.2 | 619.6627 | 3 | 19.60 | 6 | F6:7947 | DaRuMH\_F5.raw |  |  |  |  |  | 1.3171E4 |  |  |  |  | 1 | 0 | 0 | 0 | 0 | 0 | 1 | 0 | 0 | 0 | 0 | 412 | 427 |  |  | PEAKS DB |
| R.QVLDNLTM(+15.99)EK.S | Y | 61.09 | 1205.5962 | 10 | -1.1 | 603.8047 | 2 | 11.65 | 1 | F1:2538 | DaRuMH\_F1.raw | 4.2918E5 |  |  |  |  |  |  |  |  | 1.6505E5 | 2 | 1 | 0 | 0 | 0 | 0 | 0 | 0 | 0 | 0 | 1 | 262 | 271 | Oxidation (M) | M8:Oxidation (M):1000.00 | PEAKS DB |
| K.IQDWYDK.K | Y | 59.79 | 966.4447 | 7 | -0.3 | 484.2295 | 2 | 11.61 | 1 | F1:2478 | DaRuMH\_F1.raw | 5.4744E5 |  |  |  |  |  |  |  |  | 2.5497E5 | 2 | 1 | 0 | 0 | 0 | 0 | 0 | 0 | 0 | 0 | 1 | 185 | 191 |  |  | PEAKS DB |
| K.NYSPYYNTIDDLK.D | Y | 59.74 | 1604.7358 | 13 | 0.4 | 803.3755 | 2 | 13.51 | 1 | F1:3781 | DaRuMH\_F1.raw | 8.8666E5 |  |  |  |  |  |  |  |  | 6.9787E5 | 2 | 1 | 0 | 0 | 0 | 0 | 0 | 0 | 0 | 0 | 1 | 200 | 212 |  |  | PEAKS DB |
| K.TLLDIDNTR.M | Y | 59.18 | 1059.5560 | 9 | -0.3 | 530.7852 | 2 | 12.23 | 10 | F10:3023 | DaRuMH\_F9.raw | 3.2653E6 |  |  |  |  | 3.2668E5 |  |  |  | 1.9902E6 | 3 | 1 | 0 | 0 | 0 | 0 | 1 | 0 | 0 | 0 | 1 | 225 | 233 |  |  | PEAKS DB |
| R.HGVQELEIELQSQLSK.K | Y | 58.86 | 1836.9581 | 16 | -0.4 | 613.3264 | 3 | 18.11 | 6 | F6:7180 | DaRuMH\_F5.raw |  |  |  |  |  | 2.4178E5 |  |  |  |  | 1 | 0 | 0 | 0 | 0 | 0 | 1 | 0 | 0 | 0 | 0 | 375 | 390 |  |  | PEAKS DB |
| R.YC(+57.02)GQLQM(+15.99)IQEQISNLEAQITDVR.Q | Y | 56.71 | 2752.3159 | 23 | 0.5 | 918.4464 | 3 | 55.41 | 10 | F10:26052 | DaRuMH\_F9.raw |  |  |  |  |  |  |  | 6.0688E4 |  | 5.0374E5 | 2 | 0 | 0 | 0 | 0 | 0 | 0 | 0 | 1 | 0 | 1 | 405 | 427 | Carbamidomethylation; Oxidation (M) | C2:Carbamidomethylation:1000.00;M7:Oxidation (M):1000.00 | PEAKS DB |
| K.DIENQYETQITQIEHEVSSSGQEVQSSAK.E | Y | 56.61 | 3263.5066 | 29 | 0.1 | 1088.8429 | 3 | 30.25 | 10 | F10:15191 | DaRuMH\_F9.raw |  |  |  |  |  |  |  |  |  | 4.2671E6 | 1 | 0 | 0 | 0 | 0 | 0 | 0 | 0 | 0 | 0 | 1 | 340 | 368 |  |  | PEAKS DB |
| R.QFSSSYLSR.S | Y | 56.04 | 1073.5142 | 9 | 0.5 | 537.7646 | 2 | 11.57 | 1 | F1:2453 | DaRuMH\_F1.raw | 1.755E5 |  |  |  |  |  |  |  |  |  | 1 | 1 | 0 | 0 | 0 | 0 | 0 | 0 | 0 | 0 | 0 | 5 | 13 |  |  | PEAKS DB |
| Y.YNTIDDLK.D | Y | 53.47 | 980.4814 | 8 | 0.6 | 491.2483 | 2 | 11.53 | 1 | F1:2414 | DaRuMH\_F1.raw | 1.5727E5 |  |  |  |  |  |  |  |  |  | 1 | 1 | 0 | 0 | 0 | 0 | 0 | 0 | 0 | 0 | 0 | 205 | 212 |  |  | PEAKS DB |
| total 24 peptides |
| --- |

P13645|K1C10\_HUMAN

back to list

  

| Protein Coverage
| Supporting Peptides
|

Protein Coverage:

Supporting Peptides:

| Peptide | Uniq | -10lgP | Mass | Length | ppm | m/z | z | RT | Fraction | Scan | Source File | Area F1 | Area F10 | Area F2 | Area F3 | Area F4 | Area F5 | Area F7A | Area F7B | Area F8 | Area F9 | #Feature | #Feature F1 | #Feature F10 | #Feature F2 | #Feature F3 | #Feature F4 | #Feature F5 | #Feature F7A | #Feature F7B | #Feature F8 | #Feature F9 | Start | End | PTM | AScore | Found By |
| --- | --- | --- | --- | --- | --- | --- | --- | --- | --- | --- | --- | --- | --- | --- | --- | --- | --- | --- | --- | --- | --- | --- | --- | --- | --- | --- | --- | --- | --- | --- | --- | --- | --- | --- | --- | --- | --- |
| K.ELTTEIDNNIEQISSYK.S | Y | 100.03 | 1995.9636 | 17 | 0.4 | 998.9895 | 2 | 20.14 | 1 | F1:6274 | DaRuMH\_F1.raw | 7.3625E6 | 7.4953E5 |  |  | 9.2955E5 |  |  |  | 1.6804E5 |  | 5 | 2 | 1 | 0 | 0 | 1 | 0 | 0 | 0 | 1 | 0 | 346 | 362 |  |  | PEAKS DB |
| R.ALEESNYELEGK.I | Y | 91.44 | 1380.6409 | 12 | 0.1 | 691.3278 | 2 | 11.53 | 1 | F1:2390 | DaRuMH\_F1.raw | 4.805E6 | 2.0128E5 | 2.4401E5 | 5.0787E5 | 7.5928E5 | 1.3136E5 |  |  |  | 5.5381E5 | 7 | 1 | 1 | 1 | 1 | 1 | 1 | 0 | 0 | 0 | 1 | 166 | 177 |  |  | PEAKS DB |
| R.SQYEQLAEQNR.K | Y | 89.33 | 1364.6321 | 11 | 0.3 | 683.3235 | 2 | 11.22 | 10 | F10:2058 | DaRuMH\_F9.raw | 2.09E6 | 1.1555E5 |  |  | 7.7981E5 | 6.2693E4 |  |  | 2.0764E4 | 8.073E5 | 6 | 1 | 1 | 0 | 0 | 1 | 1 | 0 | 0 | 1 | 1 | 323 | 333 |  |  | PEAKS DB |
| R.SGGGGGGGGC(+57.02)GGGGGVSSLR.I | Y | 86.05 | 1548.6699 | 20 | 0.7 | 775.3428 | 2 | 10.97 | 1 | F1:1845 | DaRuMH\_F1.raw | 1.6406E5 |  |  |  |  |  |  |  |  |  | 1 | 1 | 0 | 0 | 0 | 0 | 0 | 0 | 0 | 0 | 0 | 16 | 35 | Carbamidomethylation | C10:Carbamidomethylation:1000.00 | PEAKS DB |
| K.QSLEASLAETEGR.Y | Y | 82.36 | 1389.6736 | 13 | 0.5 | 695.8444 | 2 | 11.87 | 6 | F6:2655 | DaRuMH\_F5.raw |  | 3.0016E5 | 1.1376E5 | 1.663E5 | 1.4498E5 | 1.2677E5 | 1.1627E5 |  |  | 6.1339E5 | 7 | 0 | 1 | 1 | 1 | 1 | 1 | 1 | 0 | 0 | 1 | 387 | 399 |  |  | PEAKS DB |
| R.NVQALEIELQSQLALK.Q | Y | 81.80 | 1796.0043 | 16 | 0.3 | 899.0096 | 2 | 34.14 | 2 | F2:9456 | DaRuMH\_F10.raw |  | 3.3829E5 |  |  | 1.1839E5 | 1.8559E5 | 3.2803E5 |  |  | 1.7956E6 | 7 | 0 | 1 | 0 | 0 | 1 | 1 | 2 | 0 | 0 | 2 | 371 | 386 |  |  | PEAKS DB |
| K.NQILNLTTDNANILLQIDNAR.L | Y | 80.39 | 2366.2554 | 21 | -0.1 | 1184.1349 | 2 | 39.70 | 10 | F10:19356 | DaRuMH\_F9.raw |  | 1.7397E6 |  |  |  | 2.8304E5 |  | 3.3532E5 |  | 2.2366E6 | 5 | 0 | 2 | 0 | 0 | 0 | 1 | 0 | 1 | 0 | 1 | 208 | 228 |  |  | PEAKS DB |
| R.AETEC(+57.02)QNTEYQQLLDIK.I | Y | 76.18 | 2081.9575 | 17 | 0.5 | 1041.9866 | 2 | 16.60 | 1 | F1:5224 | DaRuMH\_F1.raw | 6.6492E6 |  |  |  |  |  | 5.341E5 | 0 |  |  | 2 | 1 | 0 | 0 | 0 | 0 | 0 | 1 | 0 | 0 | 0 | 423 | 439 | Carbamidomethylation | C5:Carbamidomethylation:1000.00 | PEAKS DB |
| R.SLLEGEGSSGGGGR.G | Y | 75.41 | 1261.5898 | 14 | 0.7 | 631.8026 | 2 | 11.27 | 1 | F1:2140 | DaRuMH\_F1.raw | 5.6226E5 | 3.5239E4 |  | 0 | 3.8951E5 |  |  |  |  | 1.2712E5 | 4 | 1 | 1 | 0 | 0 | 1 | 0 | 0 | 0 | 0 | 1 | 451 | 464 |  |  | PEAKS DB |
| R.QSVEADINGLR.R | N | 73.49 | 1200.6099 | 11 | -0.3 | 601.3120 | 2 | 11.79 | 3 | F3:2514 | DaRuMH\_F2.raw | 3.1844E6 | 1.422E5 | 2.3819E5 | 2.2901E5 | 2.5573E5 | 2.0361E5 |  |  |  | 2.8329E5 | 7 | 1 | 1 | 1 | 1 | 1 | 1 | 0 | 0 | 0 | 1 | 246 | 256 |  |  | PEAKS DB |
| R.SQYEQLAEQNRK.D | Y | 73.44 | 1492.7269 | 12 | 0.6 | 747.3712 | 2 | 10.92 | 1 | F1:1803 | DaRuMH\_F1.raw | 2.3845E5 |  |  | 9.9057E4 | 1.5572E5 |  |  |  |  |  | 4 | 1 | 0 | 0 | 1 | 2 | 0 | 0 | 0 | 0 | 0 | 323 | 334 |  |  | PEAKS DB |
| R.LENEIQTYR.S | Y | 69.70 | 1164.5775 | 9 | 0.7 | 583.2964 | 2 | 11.37 | 3 | F3:2124 | DaRuMH\_F2.raw | 3.15E6 | 1.84E5 | 1.9818E5 | 4.6868E5 | 4.3147E5 |  |  |  |  | 4.3631E5 | 6 | 1 | 1 | 1 | 1 | 1 | 0 | 0 | 0 | 0 | 1 | 442 | 450 |  |  | PEAKS DB |
| L.TTDNANILLQIDNAR.L | Y | 68.91 | 1670.8588 | 15 | 0.9 | 836.4374 | 2 | 17.62 | 1 | F1:5464 | DaRuMH\_F1.raw | 1.0662E5 |  |  |  |  |  |  |  |  |  | 1 | 1 | 0 | 0 | 0 | 0 | 0 | 0 | 0 | 0 | 0 | 214 | 228 |  |  | PEAKS DB |
| R.YC(+57.02)VQLSQIQAQISALEEQLQQIR.A | Y | 68.36 | 2745.4119 | 23 | 0.8 | 916.1453 | 3 | 64.28 | 2 | F2:16071 | DaRuMH\_F10.raw |  | 5.6217E5 |  |  |  |  |  |  |  |  | 1 | 0 | 1 | 0 | 0 | 0 | 0 | 0 | 0 | 0 | 0 | 400 | 422 | Carbamidomethylation | C2:Carbamidomethylation:1000.00 | PEAKS DB |
| K.ADLEM(+15.99)QIESLTEELAYLK.K | Y | 66.37 | 2111.0344 | 18 | 0.7 | 1056.5253 | 2 | 65.64 | 10 | F10:29241 | DaRuMH\_F9.raw |  |  |  |  |  |  |  |  |  | 5.3693E5 | 1 | 0 | 0 | 0 | 0 | 0 | 0 | 0 | 0 | 0 | 1 | 267 | 284 | Oxidation (M) | M5:Oxidation (M):1000.00 | PEAKS DB |
| K.YENEVALR.Q | Y | 65.35 | 992.4927 | 8 | 0.7 | 497.2540 | 2 | 11.33 | 3 | F3:2064 | DaRuMH\_F2.raw | 1.3411E6 | 8.9287E4 | 8.1226E4 |  | 1.5586E5 |  |  |  |  | 3.5166E5 | 5 | 1 | 1 | 1 | 0 | 1 | 0 | 0 | 0 | 0 | 1 | 238 | 245 |  |  | PEAKS DB |
| K.VTMQNLNDR.L | N | 62.03 | 1089.5237 | 9 | 0.4 | 545.7693 | 2 | 11.23 | 1 | F1:2101 | DaRuMH\_F1.raw | 3.6337E5 |  |  |  |  |  |  |  |  |  | 1 | 1 | 0 | 0 | 0 | 0 | 0 | 0 | 0 | 0 | 0 | 148 | 156 |  |  | PEAKS DB |
| R.VLDELTLTK.A | N | 61.69 | 1030.5911 | 9 | 0.0 | 516.3028 | 2 | 12.52 | 10 | F10:3255 | DaRuMH\_F9.raw | 5.2659E6 |  | 2.6354E5 |  |  | 2.8109E5 | 2.238E5 |  |  | 1.1045E6 | 5 | 1 | 0 | 1 | 0 | 0 | 1 | 1 | 0 | 0 | 1 | 258 | 266 |  |  | PEAKS DB |
| Q.ISALEEQLQQIR.A | Y | 60.94 | 1426.7780 | 12 | 0.7 | 714.3967 | 2 | 15.01 | 10 | F10:5297 | DaRuMH\_F9.raw |  |  | 4.5776E5 |  |  | 2.5293E5 |  | 1.339E5 |  | 2.1253E6 | 4 | 0 | 0 | 1 | 0 | 0 | 1 | 0 | 1 | 0 | 1 | 411 | 422 |  |  | PEAKS DB |
| K.GSLGGGFSSGGFSGGSFSR.G | Y | 59.21 | 1706.7648 | 19 | 0.6 | 854.3901 | 2 | 13.16 | 5 | F5:3535 | DaRuMH\_F4.raw | 5.8211E5 |  |  |  | 1.1622E6 |  |  |  |  |  | 2 | 1 | 0 | 0 | 0 | 1 | 0 | 0 | 0 | 0 | 0 | 41 | 59 |  |  | PEAKS DB |
| R.LKYENEVALR.Q | Y | 58.91 | 1233.6716 | 10 | 1.0 | 412.2316 | 3 | 11.36 | 1 | F1:2224 | DaRuMH\_F1.raw | 2.5704E5 |  |  |  |  |  |  |  |  |  | 2 | 2 | 0 | 0 | 0 | 0 | 0 | 0 | 0 | 0 | 0 | 236 | 245 |  |  | PEAKS DB |
| S.SGGYGGLGGFGGGSFR.G | Y | 56.43 | 1431.6531 | 16 | 0.7 | 716.8343 | 2 | 13.66 | 1 | F1:3884 | DaRuMH\_F1.raw | 5.2995E4 |  |  |  |  |  |  |  |  |  | 1 | 1 | 0 | 0 | 0 | 0 | 0 | 0 | 0 | 0 | 0 | 71 | 86 |  |  | PEAKS DB |
| K.DAEAWFNEK.S | Y | 55.88 | 1108.4825 | 9 | -0.5 | 555.2483 | 2 | 13.47 | 8 | F8:4154 | DaRuMH\_F7B.raw |  |  |  |  | 9.1095E5 | 1.8013E5 |  | 9.7424E4 |  |  | 3 | 0 | 0 | 0 | 0 | 1 | 1 | 0 | 1 | 0 | 0 | 335 | 343 |  |  | PEAKS DB |
| R.LAADDFR.L | N | 55.79 | 806.3922 | 7 | 5.8 | 404.2034 | 2 | 11.46 | 4 | F4:2305 | DaRuMH\_F3.raw | 3.0917E5 |  | 1.9305E4 | 0 |  |  |  |  |  |  | 2 | 1 | 0 | 1 | 0 | 0 | 0 | 0 | 0 | 0 | 0 | 229 | 235 |  |  | PEAKS DB |
| R.GSSGGGC(+57.02)FGGSSGGYGGLGGFGGGSFR.G | Y | 55.19 | 2341.9771 | 27 | 0.4 | 1171.9962 | 2 | 16.94 | 2 | F2:4777 | DaRuMH\_F10.raw |  | 1.582E5 |  |  |  |  |  |  |  |  | 1 | 0 | 1 | 0 | 0 | 0 | 0 | 0 | 0 | 0 | 0 | 60 | 86 | Carbamidomethylation | C7:Carbamidomethylation:1000.00 | PEAKS DB |
| total 25 peptides |
| --- |

A8CG89|PA2B1\_DABRR

back to list

  

| Protein Coverage
| Supporting Peptides
|

Protein Coverage:

Supporting Peptides:

| Peptide | Uniq | -10lgP | Mass | Length | ppm | m/z | z | RT | Fraction | Scan | Source File | Area F1 | Area F10 | Area F2 | Area F3 | Area F4 | Area F5 | Area F7A | Area F7B | Area F8 | Area F9 | #Feature | #Feature F1 | #Feature F10 | #Feature F2 | #Feature F3 | #Feature F4 | #Feature F5 | #Feature F7A | #Feature F7B | #Feature F8 | #Feature F9 | Start | End | PTM | AScore | Found By |
| --- | --- | --- | --- | --- | --- | --- | --- | --- | --- | --- | --- | --- | --- | --- | --- | --- | --- | --- | --- | --- | --- | --- | --- | --- | --- | --- | --- | --- | --- | --- | --- | --- | --- | --- | --- | --- | --- |
| R.VAAIC(+57.02)LGQNVNTYNK.G | N | 105.49 | 1663.8352 | 15 | 1.4 | 832.9260 | 2 | 11.91 | 1 | F1:2762 | DaRuMH\_F1.raw | 6.3444E6 |  |  |  |  |  |  | 1.6535E8 | 2.2851E6 |  | 5 | 1 | 0 | 0 | 0 | 0 | 0 | 0 | 3 | 1 | 0 | 107 | 121 | Carbamidomethylation | C5:Carbamidomethylation:1000.00 | PEAKS DB |
| K.YISYGC(+57.02)YC(+57.02)GWGGQGTPK.D | N | 96.29 | 1952.8185 | 17 | 2.1 | 977.4185 | 2 | 12.66 | 8 | F8:3720 | DaRuMH\_F7B.raw | 2.7838E6 |  |  |  |  |  | 5.7551E5 | 1.1722E8 | 1.2076E6 |  | 6 | 1 | 0 | 0 | 0 | 0 | 0 | 1 | 3 | 1 | 0 | 37 | 53 | Carbamidomethylation | C6:Carbamidomethylation:1000.00;C8:Carbamidomethylation:1000.00 | PEAKS DB |
| Y.ISYGC(+57.02)YC(+57.02)GWGGQGTPK.D | N | 94.64 | 1789.7552 | 16 | 0.8 | 895.8856 | 2 | 11.97 | 8 | F8:2858 | DaRuMH\_F7B.raw |  |  |  |  |  |  |  | 1.2387E6 |  |  | 1 | 0 | 0 | 0 | 0 | 0 | 0 | 0 | 1 | 0 | 0 | 38 | 53 | Carbamidomethylation | C5:Carbamidomethylation:1000.00;C7:Carbamidomethylation:1000.00 | PEAKS DB |
| R.VAAIC(+57.02)LGQNVNTYNKG.Y | N | 89.87 | 1720.8567 | 16 | -0.7 | 861.4351 | 2 | 12.04 | 8 | F8:2775 | DaRuMH\_F7B.raw | 4.0306E6 |  |  |  |  |  |  | 8.8783E7 | 0 |  | 2 | 1 | 0 | 0 | 0 | 0 | 0 | 0 | 1 | 0 | 0 | 107 | 122 | Carbamidomethylation | C5:Carbamidomethylation:1000.00 | PEAKS DB |
| K.GYMFLSSYYC(+57.02)R.Q | N | 86.62 | 1445.6107 | 11 | 1.2 | 723.8135 | 2 | 20.19 | 8 | F8:9722 | DaRuMH\_F7B.raw |  |  |  |  |  |  |  | 6.9621E6 |  |  | 1 | 0 | 0 | 0 | 0 | 0 | 0 | 0 | 1 | 0 | 0 | 122 | 132 | Carbamidomethylation | C10:Carbamidomethylation:1000.00 | PEAKS DB |
| A.IC(+57.02)LGQNVNTYNK.G | N | 86.20 | 1422.6925 | 12 | 0.5 | 712.3539 | 2 | 11.52 | 8 | F8:2412 | DaRuMH\_F7B.raw |  |  |  |  |  |  |  | 2.4755E6 |  |  | 1 | 0 | 0 | 0 | 0 | 0 | 0 | 0 | 1 | 0 | 0 | 110 | 121 | Carbamidomethylation | C2:Carbamidomethylation:1000.00 | PEAKS DB |
| R.C(+57.02)C(+57.02)FVHDC(+57.02)C(+57.02)YAR.V | N | 82.94 | 1546.5574 | 11 | 0.5 | 774.2864 | 2 | 11.10 | 8 | F8:2015 | DaRuMH\_F7B.raw | 1.3534E6 |  |  |  |  |  |  | 3.8568E7 | 2.4142E5 |  | 5 | 2 | 0 | 0 | 0 | 0 | 0 | 0 | 2 | 1 | 0 | 59 | 69 | Carbamidomethylation | C1:Carbamidomethylation:1000.00;C2:Carbamidomethylation:1000.00;C7:Carbamidomethylation:1000.00;C8:Carbamidomethylation:1000.00 | PEAKS DB |
| Y.GC(+57.02)YC(+57.02)GWGGQGTPK.D | N | 80.61 | 1426.5758 | 13 | 0.8 | 714.2958 | 2 | 11.39 | 8 | F8:2290 | DaRuMH\_F7B.raw | 1.8108E5 |  |  |  |  |  |  | 4.0555E6 |  |  | 2 | 1 | 0 | 0 | 0 | 0 | 0 | 0 | 1 | 0 | 0 | 41 | 53 | Carbamidomethylation | C2:Carbamidomethylation:1000.00;C4:Carbamidomethylation:1000.00 | PEAKS DB |
| S.YGC(+57.02)YC(+57.02)GWGGQGTPK.D | N | 78.48 | 1589.6392 | 14 | 0.8 | 795.8275 | 2 | 11.62 | 8 | F8:2519 | DaRuMH\_F7B.raw |  |  |  |  |  |  |  | 5.925E5 |  |  | 1 | 0 | 0 | 0 | 0 | 0 | 0 | 0 | 1 | 0 | 0 | 40 | 53 | Carbamidomethylation | C3:Carbamidomethylation:1000.00;C5:Carbamidomethylation:1000.00 | PEAKS DB |
| K.LVEYSYSYR.T | N | 74.21 | 1178.5608 | 9 | -4.5 | 590.2850 | 2 | 11.67 | 8 | F8:2558 | DaRuMH\_F7B.raw | 1.5083E6 |  |  |  |  |  |  | 5.6544E7 | 5.2054E5 |  | 3 | 1 | 0 | 0 | 0 | 0 | 0 | 0 | 1 | 1 | 0 | 77 | 85 |  |  | PEAKS DB |
| C.LGQNVNTYNK.G | N | 73.67 | 1149.5779 | 10 | -0.6 | 575.7959 | 2 | 10.81 | 8 | F8:1754 | DaRuMH\_F7B.raw | 4.6E5 |  |  |  |  |  |  | 1.7442E5 |  |  | 2 | 1 | 0 | 0 | 0 | 0 | 0 | 0 | 1 | 0 | 0 | 112 | 121 |  |  | PEAKS DB |
| K.IVC(+57.02)ETYNR.C | Y | 69.92 | 1053.4913 | 8 | 0.1 | 527.7530 | 2 | 10.83 | 8 | F8:1774 | DaRuMH\_F7B.raw | 9.1924E6 |  |  |  |  |  |  | 4.8483E6 | 1.4999E5 |  | 3 | 1 | 0 | 0 | 0 | 0 | 0 | 0 | 1 | 1 | 0 | 89 | 96 | Carbamidomethylation | C3:Carbamidomethylation:1000.00 | PEAKS DB |
| Y.C(+57.02)GWGGQGTPK.D | N | 68.84 | 1046.4603 | 10 | -2.1 | 524.2363 | 2 | 10.97 | 8 | F8:1880 | DaRuMH\_F7B.raw | 4.3739E5 |  |  |  |  |  |  | 2.4018E6 |  |  | 2 | 1 | 0 | 0 | 0 | 0 | 0 | 0 | 1 | 0 | 0 | 44 | 53 | Carbamidomethylation | C1:Carbamidomethylation:1000.00 | PEAKS DB |
| C.YC(+57.02)GWGGQGTPK.D | N | 68.68 | 1209.5237 | 11 | -0.3 | 605.7689 | 2 | 11.30 | 8 | F8:2229 | DaRuMH\_F7B.raw |  |  |  |  |  |  |  | 6.5322E5 |  |  | 1 | 0 | 0 | 0 | 0 | 0 | 0 | 0 | 1 | 0 | 0 | 43 | 53 | Carbamidomethylation | C2:Carbamidomethylation:1000.00 | PEAKS DB |
| K.GYM(+15.99)FLSSYYC(+57.02)R.Q | N | 64.61 | 1461.6057 | 11 | 0.1 | 731.8102 | 2 | 20.18 | 8 | F8:9724 | DaRuMH\_F7B.raw |  |  |  |  |  |  |  | 3.09E6 |  |  | 2 | 0 | 0 | 0 | 0 | 0 | 0 | 0 | 2 | 0 | 0 | 122 | 132 | Oxidation (M); Carbamidomethylation | M3:Oxidation (M):1000.00;C10:Carbamidomethylation:1000.00 | PEAKS DB |
| K.QEAFSFFK.Y | N | 60.54 | 1002.4810 | 8 | 0.2 | 502.2479 | 2 | 17.29 | 8 | F8:7738 | DaRuMH\_F7B.raw | 2.371E6 |  |  |  |  |  | 2.8721E5 | 5.8246E7 | 5.8746E5 |  | 4 | 1 | 0 | 0 | 0 | 0 | 0 | 1 | 1 | 1 | 0 | 29 | 36 |  |  | PEAKS DB |
| R.VAAIC(+57.02)LGQNVNTY.N | N | 56.98 | 1421.6973 | 13 | 0.5 | 711.8563 | 2 | 18.61 | 8 | F8:8585 | DaRuMH\_F7B.raw |  |  |  |  |  |  |  | 3.2825E7 |  |  | 1 | 0 | 0 | 0 | 0 | 0 | 0 | 0 | 1 | 0 | 0 | 107 | 119 | Carbamidomethylation | C5:Carbamidomethylation:1000.00 | PEAKS DB |
| total 17 peptides |
| --- |

A8CG82|PA2B1\_DABSI

back to list

  

| Protein Coverage
| Supporting Peptides
|

Protein Coverage:

Supporting Peptides:

| Peptide | Uniq | -10lgP | Mass | Length | ppm | m/z | z | RT | Fraction | Scan | Source File | Area F1 | Area F10 | Area F2 | Area F3 | Area F4 | Area F5 | Area F7A | Area F7B | Area F8 | Area F9 | #Feature | #Feature F1 | #Feature F10 | #Feature F2 | #Feature F3 | #Feature F4 | #Feature F5 | #Feature F7A | #Feature F7B | #Feature F8 | #Feature F9 | Start | End | PTM | AScore | Found By |
| --- | --- | --- | --- | --- | --- | --- | --- | --- | --- | --- | --- | --- | --- | --- | --- | --- | --- | --- | --- | --- | --- | --- | --- | --- | --- | --- | --- | --- | --- | --- | --- | --- | --- | --- | --- | --- | --- |
| R.VAAIC(+57.02)LGQNVNTYNK.G | N | 105.49 | 1663.8352 | 15 | 1.4 | 832.9260 | 2 | 11.91 | 1 | F1:2762 | DaRuMH\_F1.raw | 6.3444E6 |  |  |  |  |  |  | 1.6535E8 | 2.2851E6 |  | 5 | 1 | 0 | 0 | 0 | 0 | 0 | 0 | 3 | 1 | 0 | 107 | 121 | Carbamidomethylation | C5:Carbamidomethylation:1000.00 | PEAKS DB |
| K.YISYGC(+57.02)YC(+57.02)GWGGQGTPK.D | N | 96.29 | 1952.8185 | 17 | 2.1 | 977.4185 | 2 | 12.66 | 8 | F8:3720 | DaRuMH\_F7B.raw | 2.7838E6 |  |  |  |  |  | 5.7551E5 | 1.1722E8 | 1.2076E6 |  | 6 | 1 | 0 | 0 | 0 | 0 | 0 | 1 | 3 | 1 | 0 | 37 | 53 | Carbamidomethylation | C6:Carbamidomethylation:1000.00;C8:Carbamidomethylation:1000.00 | PEAKS DB |
| Y.ISYGC(+57.02)YC(+57.02)GWGGQGTPK.D | N | 94.64 | 1789.7552 | 16 | 0.8 | 895.8856 | 2 | 11.97 | 8 | F8:2858 | DaRuMH\_F7B.raw |  |  |  |  |  |  |  | 1.2387E6 |  |  | 1 | 0 | 0 | 0 | 0 | 0 | 0 | 0 | 1 | 0 | 0 | 38 | 53 | Carbamidomethylation | C5:Carbamidomethylation:1000.00;C7:Carbamidomethylation:1000.00 | PEAKS DB |
| R.VAAIC(+57.02)LGQNVNTYNKG.Y | N | 89.87 | 1720.8567 | 16 | -0.7 | 861.4351 | 2 | 12.04 | 8 | F8:2775 | DaRuMH\_F7B.raw | 4.0306E6 |  |  |  |  |  |  | 8.8783E7 | 0 |  | 2 | 1 | 0 | 0 | 0 | 0 | 0 | 0 | 1 | 0 | 0 | 107 | 122 | Carbamidomethylation | C5:Carbamidomethylation:1000.00 | PEAKS DB |
| K.GYMFLSSYYC(+57.02)R.Q | N | 86.62 | 1445.6107 | 11 | 1.2 | 723.8135 | 2 | 20.19 | 8 | F8:9722 | DaRuMH\_F7B.raw |  |  |  |  |  |  |  | 6.9621E6 |  |  | 1 | 0 | 0 | 0 | 0 | 0 | 0 | 0 | 1 | 0 | 0 | 122 | 132 | Carbamidomethylation | C10:Carbamidomethylation:1000.00 | PEAKS DB |
| A.IC(+57.02)LGQNVNTYNK.G | N | 86.20 | 1422.6925 | 12 | 0.5 | 712.3539 | 2 | 11.52 | 8 | F8:2412 | DaRuMH\_F7B.raw |  |  |  |  |  |  |  | 2.4755E6 |  |  | 1 | 0 | 0 | 0 | 0 | 0 | 0 | 0 | 1 | 0 | 0 | 110 | 121 | Carbamidomethylation | C2:Carbamidomethylation:1000.00 | PEAKS DB |
| R.C(+57.02)C(+57.02)FVHDC(+57.02)C(+57.02)YAR.V | N | 82.94 | 1546.5574 | 11 | 0.5 | 774.2864 | 2 | 11.10 | 8 | F8:2015 | DaRuMH\_F7B.raw | 1.3534E6 |  |  |  |  |  |  | 3.8568E7 | 2.4142E5 |  | 5 | 2 | 0 | 0 | 0 | 0 | 0 | 0 | 2 | 1 | 0 | 59 | 69 | Carbamidomethylation | C1:Carbamidomethylation:1000.00;C2:Carbamidomethylation:1000.00;C7:Carbamidomethylation:1000.00;C8:Carbamidomethylation:1000.00 | PEAKS DB |
| Y.GC(+57.02)YC(+57.02)GWGGQGTPK.D | N | 80.61 | 1426.5758 | 13 | 0.8 | 714.2958 | 2 | 11.39 | 8 | F8:2290 | DaRuMH\_F7B.raw | 1.8108E5 |  |  |  |  |  |  | 4.0555E6 |  |  | 2 | 1 | 0 | 0 | 0 | 0 | 0 | 0 | 1 | 0 | 0 | 41 | 53 | Carbamidomethylation | C2:Carbamidomethylation:1000.00;C4:Carbamidomethylation:1000.00 | PEAKS DB |
| S.YGC(+57.02)YC(+57.02)GWGGQGTPK.D | N | 78.48 | 1589.6392 | 14 | 0.8 | 795.8275 | 2 | 11.62 | 8 | F8:2519 | DaRuMH\_F7B.raw |  |  |  |  |  |  |  | 5.925E5 |  |  | 1 | 0 | 0 | 0 | 0 | 0 | 0 | 0 | 1 | 0 | 0 | 40 | 53 | Carbamidomethylation | C3:Carbamidomethylation:1000.00;C5:Carbamidomethylation:1000.00 | PEAKS DB |
| C.LGQNVNTYNK.G | N | 73.67 | 1149.5779 | 10 | -0.6 | 575.7959 | 2 | 10.81 | 8 | F8:1754 | DaRuMH\_F7B.raw | 4.6E5 |  |  |  |  |  |  | 1.7442E5 |  |  | 2 | 1 | 0 | 0 | 0 | 0 | 0 | 0 | 1 | 0 | 0 | 112 | 121 |  |  | PEAKS DB |
| Y.C(+57.02)GWGGQGTPK.D | N | 68.84 | 1046.4603 | 10 | -2.1 | 524.2363 | 2 | 10.97 | 8 | F8:1880 | DaRuMH\_F7B.raw | 4.3739E5 |  |  |  |  |  |  | 2.4018E6 |  |  | 2 | 1 | 0 | 0 | 0 | 0 | 0 | 0 | 1 | 0 | 0 | 44 | 53 | Carbamidomethylation | C1:Carbamidomethylation:1000.00 | PEAKS DB |
| C.YC(+57.02)GWGGQGTPK.D | N | 68.68 | 1209.5237 | 11 | -0.3 | 605.7689 | 2 | 11.30 | 8 | F8:2229 | DaRuMH\_F7B.raw |  |  |  |  |  |  |  | 6.5322E5 |  |  | 1 | 0 | 0 | 0 | 0 | 0 | 0 | 0 | 1 | 0 | 0 | 43 | 53 | Carbamidomethylation | C2:Carbamidomethylation:1000.00 | PEAKS DB |
| K.GYM(+15.99)FLSSYYC(+57.02)R.Q | N | 64.61 | 1461.6057 | 11 | 0.1 | 731.8102 | 2 | 20.18 | 8 | F8:9724 | DaRuMH\_F7B.raw |  |  |  |  |  |  |  | 3.09E6 |  |  | 2 | 0 | 0 | 0 | 0 | 0 | 0 | 0 | 2 | 0 | 0 | 122 | 132 | Oxidation (M); Carbamidomethylation | M3:Oxidation (M):1000.00;C10:Carbamidomethylation:1000.00 | PEAKS DB |
| K.IVC(+57.02)ENYNR.C | Y | 62.48 | 1066.4866 | 8 | 0.2 | 534.2507 | 2 | 10.84 | 1 | F1:1719 | DaRuMH\_F1.raw | 0 |  |  |  |  |  |  |  |  |  | 0 | 0 | 0 | 0 | 0 | 0 | 0 | 0 | 0 | 0 | 0 | 89 | 96 | Carbamidomethylation | C3:Carbamidomethylation:1000.00 | PEAKS DB |
| K.QEAFSFFK.Y | N | 60.54 | 1002.4810 | 8 | 0.2 | 502.2479 | 2 | 17.29 | 8 | F8:7738 | DaRuMH\_F7B.raw | 2.371E6 |  |  |  |  |  | 2.8721E5 | 5.8246E7 | 5.8746E5 |  | 4 | 1 | 0 | 0 | 0 | 0 | 0 | 1 | 1 | 1 | 0 | 29 | 36 |  |  | PEAKS DB |
| R.VAAIC(+57.02)LGQNVNTY.N | N | 56.98 | 1421.6973 | 13 | 0.5 | 711.8563 | 2 | 18.61 | 8 | F8:8585 | DaRuMH\_F7B.raw |  |  |  |  |  |  |  | 3.2825E7 |  |  | 1 | 0 | 0 | 0 | 0 | 0 | 0 | 0 | 1 | 0 | 0 | 107 | 119 | Carbamidomethylation | C5:Carbamidomethylation:1000.00 | PEAKS DB |
| total 16 peptides |
| --- |

P35908|K22E\_HUMAN

back to list

  

| Protein Coverage
| Supporting Peptides
|

Protein Coverage:

Supporting Peptides:

| Peptide | Uniq | -10lgP | Mass | Length | ppm | m/z | z | RT | Fraction | Scan | Source File | Area F1 | Area F10 | Area F2 | Area F3 | Area F4 | Area F5 | Area F7A | Area F7B | Area F8 | Area F9 | #Feature | #Feature F1 | #Feature F10 | #Feature F2 | #Feature F3 | #Feature F4 | #Feature F5 | #Feature F7A | #Feature F7B | #Feature F8 | #Feature F9 | Start | End | PTM | AScore | Found By |
| --- | --- | --- | --- | --- | --- | --- | --- | --- | --- | --- | --- | --- | --- | --- | --- | --- | --- | --- | --- | --- | --- | --- | --- | --- | --- | --- | --- | --- | --- | --- | --- | --- | --- | --- | --- | --- | --- |
| R.GGSGGGGSISGGGYGSGGGSGGR.Y | Y | 102.41 | 1740.7411 | 23 | 1.0 | 871.3787 | 2 | 10.77 | 1 | F1:1657 | DaRuMH\_F1.raw | 3.043E5 |  |  |  |  |  |  |  |  |  | 1 | 1 | 0 | 0 | 0 | 0 | 0 | 0 | 0 | 0 | 0 | 550 | 572 |  |  | PEAKS DB |
| K.NVQDAIADAEQR.G | Y | 89.44 | 1328.6321 | 12 | -0.4 | 665.3231 | 2 | 11.67 | 1 | F1:2556 | DaRuMH\_F1.raw | 3.1467E5 | 8.4416E4 | 1.1455E4 |  | 2.7641E4 |  |  |  |  | 1.444E5 | 5 | 1 | 1 | 1 | 0 | 1 | 0 | 0 | 0 | 0 | 1 | 419 | 430 |  |  | PEAKS DB |
| R.FLEQQNQVLQTK.W | N | 86.18 | 1474.7780 | 12 | -1.5 | 738.3951 | 2 | 11.69 | 10 | F10:2507 | DaRuMH\_F9.raw | 1.2606E7 | 4.3269E5 | 3.8807E5 | 1.3371E6 | 1.6646E6 | 5.0153E5 | 4.6914E5 | 3.9811E5 | 4.8981E5 | 3.5935E6 | 10 | 1 | 1 | 1 | 1 | 1 | 1 | 1 | 1 | 1 | 1 | 198 | 209 |  |  | PEAKS DB |
| R.GFSSGSAVVSGGSR.R | N | 84.62 | 1253.6000 | 14 | 0.9 | 627.8078 | 2 | 11.21 | 10 | F10:2079 | DaRuMH\_F9.raw | 1.9202E5 | 4.2177E4 |  |  | 1.3535E5 |  |  |  |  | 1.1091E5 | 4 | 1 | 1 | 0 | 0 | 1 | 0 | 0 | 0 | 0 | 1 | 21 | 34 |  |  | PEAKS DB |
| R.NLDLDSIIAEVK.A | N | 84.25 | 1328.7188 | 12 | 0.3 | 665.3668 | 2 | 36.47 | 3 | F3:8177 | DaRuMH\_F2.raw | 1.633E6 | 5.015E5 | 9.1776E4 |  | 8.3506E5 |  | 5.5657E5 | 4.6037E5 | 2.1133E5 | 2.4217E6 | 8 | 1 | 1 | 1 | 0 | 1 | 0 | 1 | 1 | 1 | 1 | 342 | 353 |  |  | PEAKS DB |
| K.YEELQVTVGR.H | Y | 80.25 | 1192.6088 | 10 | 0.9 | 597.3122 | 2 | 11.84 | 1 | F1:2706 | DaRuMH\_F1.raw | 3.2732E5 | 7.3003E4 | 9.5717E4 | 9.3665E4 | 1.1515E5 |  |  |  |  |  | 5 | 1 | 1 | 1 | 1 | 1 | 0 | 0 | 0 | 0 | 0 | 375 | 384 |  |  | PEAKS DB |
| R.TAAENDFVTLK.K | N | 79.01 | 1207.6084 | 11 | 0.1 | 604.8115 | 2 | 11.94 | 3 | F3:2649 | DaRuMH\_F2.raw | 1.0157E6 | 1.1887E5 | 1.5464E5 | 1.7081E5 | 1.512E5 | 9.8135E4 |  |  | 1.7377E4 | 2.3596E5 | 8 | 1 | 1 | 1 | 1 | 1 | 1 | 0 | 0 | 1 | 1 | 276 | 286 |  |  | PEAKS DB |
| K.LALDVEIATYR.K | N | 76.33 | 1262.6870 | 11 | 1.0 | 632.3514 | 2 | 19.40 | 10 | F10:8852 | DaRuMH\_F9.raw |  | 2.1656E5 |  |  | 5.9819E4 | 2.2865E5 | 6.9929E4 |  |  | 7.4922E5 | 5 | 0 | 1 | 0 | 0 | 1 | 1 | 1 | 0 | 0 | 1 | 471 | 481 |  |  | PEAKS DB |
| K.LNDLEEALQQAK.E | Y | 76.27 | 1370.7041 | 12 | 0.2 | 686.3595 | 2 | 13.78 | 1 | F1:3898 | DaRuMH\_F1.raw | 2.4883E6 | 2.5849E5 | 3.1635E5 | 2.7783E5 | 3.3852E5 | 2.9462E5 |  |  |  | 1.1091E6 | 7 | 1 | 1 | 1 | 1 | 1 | 1 | 0 | 0 | 0 | 1 | 442 | 453 |  |  | PEAKS DB |
| K.VDLLNQEIEFLK.V | Y | 75.99 | 1459.7922 | 12 | 2.5 | 730.9036 | 2 | 34.24 | 7 | F7:14165 | DaRuMH\_F7A.raw | 1.559E6 |  |  |  |  |  | 6.2345E5 |  |  |  | 2 | 1 | 0 | 0 | 0 | 0 | 0 | 1 | 0 | 0 | 0 | 303 | 314 |  |  | PEAKS DB |
| R.SKEEAEALYHSK.Y | Y | 72.94 | 1390.6729 | 12 | -2.1 | 464.5639 | 3 | 10.74 | 1 | F1:1622 | DaRuMH\_F1.raw | 2.1271E6 |  |  |  |  |  |  |  |  |  | 1 | 1 | 0 | 0 | 0 | 0 | 0 | 0 | 0 | 0 | 0 | 363 | 374 |  |  | PEAKS DB |
| R.HGGGGGGFGGGGFGSR.S | Y | 71.20 | 1319.5754 | 16 | 0.3 | 440.8659 | 3 | 11.08 | 1 | F1:1974 | DaRuMH\_F1.raw | 5.8043E4 | 2.1465E3 |  |  | 1.9654E4 |  |  |  |  |  | 3 | 1 | 1 | 0 | 0 | 1 | 0 | 0 | 0 | 0 | 0 | 46 | 61 |  |  | PEAKS DB |
| R.STSSFSC(+57.02)LSR.H | Y | 68.88 | 1130.5026 | 10 | -0.2 | 566.2584 | 2 | 11.43 | 1 | F1:2287 | DaRuMH\_F1.raw | 1.8551E5 |  |  |  |  |  |  |  |  |  | 1 | 1 | 0 | 0 | 0 | 0 | 0 | 0 | 0 | 0 | 0 | 36 | 45 | Carbamidomethylation | C7:Carbamidomethylation:1000.00 | PEAKS DB |
| K.AQYEEIAQR.S | N | 64.85 | 1106.5356 | 9 | -0.1 | 554.2750 | 2 | 11.12 | 5 | F5:1951 | DaRuMH\_F4.raw | 2.965E5 | 4.6505E4 |  | 1.2689E5 | 1.6019E5 |  |  |  |  | 2.0437E5 | 5 | 1 | 1 | 0 | 1 | 1 | 0 | 0 | 0 | 0 | 1 | 354 | 362 |  |  | PEAKS DB |
| R.DYQELMNVK.L | N | 64.78 | 1138.5328 | 9 | 0.6 | 570.2740 | 2 | 12.35 | 1 | F1:3168 | DaRuMH\_F1.raw | 1.3811E5 |  |  |  |  |  |  |  |  |  | 1 | 1 | 0 | 0 | 0 | 0 | 0 | 0 | 0 | 0 | 0 | 462 | 470 |  |  | PEAKS DB |
| R.PINLEPIFQGYIDSLK.R | Y | 61.52 | 1845.9875 | 16 | 0.3 | 616.3367 | 3 | 52.11 | 8 | F8:26304 | DaRuMH\_F7B.raw |  |  |  |  |  |  |  | 2.312E4 |  |  | 1 | 0 | 0 | 0 | 0 | 0 | 0 | 0 | 1 | 0 | 0 | 222 | 237 |  |  | PEAKS DB |
| R.LQGEIAHVK.K | N | 60.82 | 993.5607 | 9 | 0.8 | 497.7880 | 2 | 10.76 | 1 | F1:1660 | DaRuMH\_F1.raw | 1.9392E5 |  |  |  |  |  |  |  |  |  | 1 | 1 | 0 | 0 | 0 | 0 | 0 | 0 | 0 | 0 | 0 | 406 | 414 |  |  | PEAKS DB |
| R.YLDGLTAER.T | Y | 59.51 | 1036.5189 | 9 | -1.0 | 519.2662 | 2 | 11.79 | 10 | F10:2620 | DaRuMH\_F9.raw |  |  |  |  |  |  |  |  |  | 1.8355E5 | 1 | 0 | 0 | 0 | 0 | 0 | 0 | 0 | 0 | 0 | 1 | 239 | 247 |  |  | PEAKS DB |
| K.VDPEIQNVK.A | N | 59.49 | 1040.5502 | 9 | 0.6 | 521.2827 | 2 | 11.25 | 1 | F1:2139 | DaRuMH\_F1.raw | 2.8994E5 |  |  |  |  |  |  |  |  |  | 1 | 1 | 0 | 0 | 0 | 0 | 0 | 0 | 0 | 0 | 0 | 167 | 175 |  |  | PEAKS DB |
| R.GSSSGGGYSSGSSSYGSGGR.Q | Y | 58.41 | 1739.6982 | 20 | 0.5 | 870.8568 | 2 | 10.69 | 5 | F5:1612 | DaRuMH\_F4.raw |  |  |  |  | 1.8231E4 |  |  |  |  |  | 1 | 0 | 0 | 0 | 0 | 1 | 0 | 0 | 0 | 0 | 0 | 525 | 544 |  |  | PEAKS DB |
| K.LLEGEEC(+57.02)R.M | N | 57.87 | 1004.4597 | 8 | 1.1 | 503.2376 | 2 | 10.99 | 2 | F2:1866 | DaRuMH\_F10.raw |  | 1.3429E4 |  |  |  |  |  |  |  |  | 1 | 0 | 1 | 0 | 0 | 0 | 0 | 0 | 0 | 0 | 0 | 483 | 490 | Carbamidomethylation | C7:Carbamidomethylation:1000.00 | PEAKS DB |
| total 21 peptides |
| --- |

P86368|PA2B3\_DABRR

back to list

  

| Protein Coverage
| Supporting Peptides
|

Protein Coverage:

Supporting Peptides:

| Peptide | Uniq | -10lgP | Mass | Length | ppm | m/z | z | RT | Fraction | Scan | Source File | Area F1 | Area F10 | Area F2 | Area F3 | Area F4 | Area F5 | Area F7A | Area F7B | Area F8 | Area F9 | #Feature | #Feature F1 | #Feature F10 | #Feature F2 | #Feature F3 | #Feature F4 | #Feature F5 | #Feature F7A | #Feature F7B | #Feature F8 | #Feature F9 | Start | End | PTM | AScore | Found By |
| --- | --- | --- | --- | --- | --- | --- | --- | --- | --- | --- | --- | --- | --- | --- | --- | --- | --- | --- | --- | --- | --- | --- | --- | --- | --- | --- | --- | --- | --- | --- | --- | --- | --- | --- | --- | --- | --- |
| R.VNGAIVC(+57.02)EQGTSC(+57.02)ENR.I | Y | 94.84 | 1792.7832 | 16 | 0.3 | 897.3991 | 2 | 11.17 | 3 | F3:1923 | DaRuMH\_F2.raw | 3.5907E4 |  | 1.0987E5 | 2.1304E7 | 1.1721E6 |  |  |  | 5.1561E4 |  | 7 | 1 | 0 | 1 | 3 | 1 | 0 | 0 | 0 | 1 | 0 | 69 | 84 | Carbamidomethylation | C7:Carbamidomethylation:1000.00;C13:Carbamidomethylation:1000.00 | PEAKS DB |
| R.C(+57.02)C(+57.02)FVHDC(+57.02)C(+57.02)YGNLPDC(+57.02)NPK.S | N | 87.19 | 2314.8687 | 18 | 0.3 | 1158.4419 | 2 | 11.55 | 5 | F5:2369 | DaRuMH\_F4.raw | 3.1129E5 |  | 3.3212E6 | 1.4676E8 | 1.5328E7 |  |  |  | 3.5682E5 |  | 8 | 1 | 0 | 2 | 2 | 2 | 0 | 0 | 0 | 1 | 0 | 43 | 60 | Carbamidomethylation | C1:Carbamidomethylation:1000.00;C2:Carbamidomethylation:1000.00;C7:Carbamidomethylation:1000.00;C8:Carbamidomethylation:1000.00;C15:Carbamidomethylation:1000.00 | PEAKS DB |
| G.AIVC(+57.02)EQGTSC(+57.02)ENR.I | Y | 79.61 | 1522.6504 | 13 | 0.5 | 762.3329 | 2 | 10.91 | 3 | F3:1692 | DaRuMH\_F2.raw | 8.9138E4 |  | 1.9271E5 | 5.4585E6 | 2.3711E6 |  |  |  |  |  | 4 | 1 | 0 | 1 | 1 | 1 | 0 | 0 | 0 | 0 | 0 | 72 | 84 | Carbamidomethylation | C4:Carbamidomethylation:1000.00;C10:Carbamidomethylation:1000.00 | PEAKS DB |
| V.NGAIVC(+57.02)EQGTSC(+57.02)ENR.I | Y | 76.76 | 1693.7148 | 15 | 0.5 | 847.8651 | 2 | 11.53 | 5 | F5:2343 | DaRuMH\_F4.raw |  |  | 1.8868E5 | 6.827E6 | 5.4192E6 |  |  |  |  |  | 4 | 0 | 0 | 1 | 1 | 2 | 0 | 0 | 0 | 0 | 0 | 70 | 84 | Carbamidomethylation | C6:Carbamidomethylation:1000.00;C12:Carbamidomethylation:1000.00 | PEAKS DB |
| N.GAIVC(+57.02)EQGTSC(+57.02)ENR.I | Y | 75.83 | 1579.6719 | 14 | 1.3 | 790.8442 | 2 | 10.95 | 5 | F5:1747 | DaRuMH\_F4.raw | 0 |  | 5.4287E5 |  | 8.7569E6 |  |  |  |  |  | 2 | 0 | 0 | 1 | 0 | 1 | 0 | 0 | 0 | 0 | 0 | 71 | 84 | Carbamidomethylation | C5:Carbamidomethylation:1000.00;C11:Carbamidomethylation:1000.00 | PEAKS DB |
| R.IC(+57.02)EC(+57.02)DKAAAIC(+57.02)FR.R | N | 73.96 | 1612.7159 | 13 | 6.1 | 538.5795 | 3 | 11.48 | 4 | F4:2342 | DaRuMH\_F3.raw |  |  |  | 5.6073E5 | 3.0117E4 |  |  |  |  |  | 2 | 0 | 0 | 0 | 1 | 1 | 0 | 0 | 0 | 0 | 0 | 85 | 97 | Carbamidomethylation | C2:Carbamidomethylation:1000.00;C4:Carbamidomethylation:1000.00;C11:Carbamidomethylation:1000.00 | PEAKS DB |
| C.YGNLPDC(+57.02)NPK.S | N | 70.66 | 1176.5233 | 10 | -0.1 | 589.2689 | 2 | 11.16 | 5 | F5:1987 | DaRuMH\_F4.raw |  |  |  | 8.5094E5 | 1.5865E5 |  |  |  |  |  | 2 | 0 | 0 | 0 | 1 | 1 | 0 | 0 | 0 | 0 | 0 | 51 | 60 | Carbamidomethylation | C7:Carbamidomethylation:1000.00 | PEAKS DB |
| C.C(+57.02)YGNLPDC(+57.02)NPK.S | N | 67.80 | 1336.5540 | 11 | 6.2 | 669.2845 | 2 | 11.27 | 4 | F4:2144 | DaRuMH\_F3.raw |  |  |  | 6.368E5 | 1.2947E5 |  |  |  |  |  | 2 | 0 | 0 | 0 | 1 | 1 | 0 | 0 | 0 | 0 | 0 | 50 | 60 | Carbamidomethylation | C1:Carbamidomethylation:1000.00;C8:Carbamidomethylation:1000.00 | PEAKS DB |
| S.YGC(+57.02)YC(+57.02)GWGGK.A | N | 66.85 | 1206.4586 | 10 | -0.3 | 604.2364 | 2 | 11.56 | 8 | F8:2453 | DaRuMH\_F7B.raw |  |  |  |  | 2.4701E5 | 2.1367E7 | 1.6469E7 | 7.7105E6 |  |  | 4 | 0 | 0 | 0 | 0 | 1 | 1 | 1 | 1 | 0 | 0 | 24 | 33 | Carbamidomethylation | C3:Carbamidomethylation:1000.00;C5:Carbamidomethylation:1000.00 | PEAKS DB |
| S.SYGC(+57.02)YC(+57.02)GWGGK.A | N | 66.77 | 1293.4907 | 11 | 0.8 | 647.7532 | 2 | 11.60 | 5 | F5:2407 | DaRuMH\_F4.raw |  |  | 1.9452E5 | 5.4914E6 | 2.175E6 |  |  |  |  |  | 3 | 0 | 0 | 1 | 1 | 1 | 0 | 0 | 0 | 0 | 0 | 23 | 33 | Carbamidomethylation | C4:Carbamidomethylation:1000.00;C6:Carbamidomethylation:1000.00 | PEAKS DB |
| A.IVC(+57.02)EQGTSC(+57.02)ENR.I | Y | 64.99 | 1451.6133 | 12 | 6.6 | 726.8146 | 2 | 10.70 | 4 | F4:1590 | DaRuMH\_F3.raw |  |  |  | 2.5255E5 | 9.9936E4 |  |  |  |  |  | 2 | 0 | 0 | 0 | 1 | 1 | 0 | 0 | 0 | 0 | 0 | 73 | 84 | Carbamidomethylation | C3:Carbamidomethylation:1000.00;C9:Carbamidomethylation:1000.00 | PEAKS DB |
| Y.SSYGC(+57.02)YC(+57.02)GWGGK.A | N | 64.86 | 1380.5227 | 12 | -0.5 | 691.2683 | 2 | 11.62 | 5 | F5:2415 | DaRuMH\_F4.raw |  |  |  | 1.124E7 | 2.4271E6 |  |  |  |  |  | 2 | 0 | 0 | 0 | 1 | 1 | 0 | 0 | 0 | 0 | 0 | 22 | 33 | Carbamidomethylation | C5:Carbamidomethylation:1000.00;C7:Carbamidomethylation:1000.00 | PEAKS DB |
| D.C(+57.02)C(+57.02)YGNLPDC(+57.02)NPK.S | N | 64.16 | 1496.5846 | 12 | 0.6 | 749.3000 | 2 | 11.36 | 5 | F5:2178 | DaRuMH\_F4.raw |  |  | 1.7672E4 | 4.9522E5 | 1.2789E5 |  |  |  |  |  | 3 | 0 | 0 | 1 | 1 | 1 | 0 | 0 | 0 | 0 | 0 | 49 | 60 | Carbamidomethylation | C1:Carbamidomethylation:1000.00;C2:Carbamidomethylation:1000.00;C9:Carbamidomethylation:1000.00 | PEAKS DB |
| H.DC(+57.02)C(+57.02)YGNLPDC(+57.02)NPK.S | N | 64.10 | 1611.6116 | 13 | 6.8 | 806.8139 | 2 | 11.50 | 4 | F4:2345 | DaRuMH\_F3.raw |  |  |  | 6.3212E5 |  |  |  |  |  |  | 1 | 0 | 0 | 0 | 1 | 0 | 0 | 0 | 0 | 0 | 0 | 48 | 60 | Carbamidomethylation | C2:Carbamidomethylation:1000.00;C3:Carbamidomethylation:1000.00;C10:Carbamidomethylation:1000.00 | PEAKS DB |
| F.VHDC(+57.02)C(+57.02)YGNLPDC(+57.02)NPK.S | N | 61.66 | 1847.7389 | 15 | 5.4 | 616.9200 | 3 | 11.16 | 4 | F4:2016 | DaRuMH\_F3.raw |  |  | 1.6032E5 | 9.3266E6 |  |  |  |  |  |  | 2 | 0 | 0 | 1 | 1 | 0 | 0 | 0 | 0 | 0 | 0 | 46 | 60 | Carbamidomethylation | C4:Carbamidomethylation:1000.00;C5:Carbamidomethylation:1000.00;C12:Carbamidomethylation:1000.00 | PEAKS DB |
| Y.GC(+57.02)YC(+57.02)GWGGK.A | N | 58.54 | 1043.3953 | 9 | -0.4 | 522.7047 | 2 | 11.29 | 6 | F6:2113 | DaRuMH\_F5.raw |  |  |  |  |  | 2.1095E6 |  | 4.3981E6 |  |  | 2 | 0 | 0 | 0 | 0 | 0 | 1 | 0 | 1 | 0 | 0 | 25 | 33 | Carbamidomethylation | C2:Carbamidomethylation:1000.00;C4:Carbamidomethylation:1000.00 | PEAKS DB |
| K.RVNGAIVC(+57.02)EQGTSC(+57.02)ENR.I | Y | 58.13 | 1948.8843 | 17 | 6.0 | 650.6356 | 3 | 10.85 | 4 | F4:1715 | DaRuMH\_F3.raw |  |  |  | 6.9239E5 |  |  |  |  |  |  | 1 | 0 | 0 | 0 | 1 | 0 | 0 | 0 | 0 | 0 | 0 | 68 | 84 | Carbamidomethylation | C8:Carbamidomethylation:1000.00;C14:Carbamidomethylation:1000.00 | PEAKS DB |
| Y.GNLPDC(+57.02)NPK.S | N | 56.42 | 1013.4600 | 9 | 0.5 | 507.7375 | 2 | 10.79 | 3 | F3:1629 | DaRuMH\_F2.raw |  |  | 9.4181E4 |  |  |  |  |  |  |  | 1 | 0 | 0 | 1 | 0 | 0 | 0 | 0 | 0 | 0 | 0 | 52 | 60 | Carbamidomethylation | C6:Carbamidomethylation:1000.00 | PEAKS DB |
| I.YM(+15.99)LYPDFLC(+57.02)K.G | N | 55.67 | 1364.6145 | 10 | 0.4 | 683.3148 | 2 | 23.28 | 9 | F9:9040 | DaRuMH\_F8.raw |  |  |  |  |  |  |  | 4.1319E4 | 8.7654E4 |  | 2 | 0 | 0 | 0 | 0 | 0 | 0 | 0 | 1 | 1 | 0 | 107 | 116 | Oxidation (M); Carbamidomethylation | M2:Oxidation (M):1000.00;C9:Carbamidomethylation:1000.00 | PEAKS DB |
| L.YPDFLC(+57.02)KGELK.C | N | 53.47 | 1368.6747 | 11 | 0.8 | 457.2325 | 3 | 11.92 | 5 | F5:2722 | DaRuMH\_F4.raw |  |  |  |  | 1.6062E5 |  |  |  |  |  | 1 | 0 | 0 | 0 | 0 | 1 | 0 | 0 | 0 | 0 | 0 | 110 | 120 | Carbamidomethylation | C6:Carbamidomethylation:1000.00 | PEAKS DB |
| total 20 peptides |
| --- |

B7FDI0|CRVP\_VIPBN

back to list

  

| Protein Coverage
| Supporting Peptides
|

Protein Coverage:

Supporting Peptides:

| Peptide | Uniq | -10lgP | Mass | Length | ppm | m/z | z | RT | Fraction | Scan | Source File | Area F1 | Area F10 | Area F2 | Area F3 | Area F4 | Area F5 | Area F7A | Area F7B | Area F8 | Area F9 | #Feature | #Feature F1 | #Feature F10 | #Feature F2 | #Feature F3 | #Feature F4 | #Feature F5 | #Feature F7A | #Feature F7B | #Feature F8 | #Feature F9 | Start | End | PTM | AScore | Found By |
| --- | --- | --- | --- | --- | --- | --- | --- | --- | --- | --- | --- | --- | --- | --- | --- | --- | --- | --- | --- | --- | --- | --- | --- | --- | --- | --- | --- | --- | --- | --- | --- | --- | --- | --- | --- | --- | --- |
| K.DFVYGQGASPANAVVGHYTQIVWYK.S | Y | 106.44 | 2769.3550 | 25 | 1.1 | 924.1266 | 3 | 31.14 | 8 | F8:16688 | DaRuMH\_F7B.raw |  |  |  |  |  |  |  | 1.2388E8 |  |  | 1 | 0 | 0 | 0 | 0 | 0 | 0 | 0 | 1 | 0 | 0 | 100 | 124 |  |  | PEAKS DB |
| K.M(+15.99)EWYPEAAANAER.W | N | 94.55 | 1552.6616 | 13 | -3.9 | 777.3351 | 2 | 11.86 | 8 | F8:2751 | DaRuMH\_F7B.raw | 5.2819E5 |  |  |  |  |  |  | 6.0748E6 | 2.2981E5 |  | 3 | 1 | 0 | 0 | 0 | 0 | 0 | 0 | 1 | 1 | 0 | 40 | 52 | Oxidation (M) | M1:Oxidation (M):1000.00 | PEAKS DB |
| K.MEWYPEAAANAER.W | N | 93.30 | 1536.6667 | 13 | 2.1 | 769.3423 | 2 | 12.88 | 8 | F8:3614 | DaRuMH\_F7B.raw |  |  |  |  |  |  |  | 1.0703E7 | 2.998E5 |  | 2 | 0 | 0 | 0 | 0 | 0 | 0 | 0 | 1 | 1 | 0 | 40 | 52 |  |  | PEAKS DB |
| K.DFVYGQGASPANAVVGH.Y | Y | 85.38 | 1687.7954 | 17 | -0.1 | 844.9049 | 2 | 14.31 | 8 | F8:5218 | DaRuMH\_F7B.raw | 5.5704E6 |  |  |  |  |  | 2.0845E5 | 3.4957E7 |  |  | 3 | 1 | 0 | 0 | 0 | 0 | 0 | 1 | 1 | 0 | 0 | 100 | 116 |  |  | PEAKS DB |
| N.AVVGHYTQIVWYK.S | N | 80.53 | 1562.8245 | 13 | -1.5 | 521.9480 | 3 | 12.13 | 8 | F8:3006 | DaRuMH\_F7B.raw |  |  |  |  |  |  |  | 1.9751E6 | 4.4197E4 |  | 2 | 0 | 0 | 0 | 0 | 0 | 0 | 0 | 1 | 1 | 0 | 112 | 124 |  |  | PEAKS DB |
| A.NAVVGHYTQIVWYK.S | Y | 78.24 | 1676.8674 | 14 | 0.3 | 839.4413 | 2 | 12.29 | 8 | F8:3165 | DaRuMH\_F7B.raw |  |  |  |  |  |  |  | 4.492E5 |  |  | 1 | 0 | 0 | 0 | 0 | 0 | 0 | 0 | 1 | 0 | 0 | 111 | 124 |  |  | PEAKS DB |
| H.YTQIVWYK.S | N | 77.79 | 1099.5702 | 8 | 0.9 | 550.7928 | 2 | 12.97 | 8 | F8:3679 | DaRuMH\_F7B.raw | 2.3904E6 |  |  |  |  |  |  | 1.823E7 |  | 5.9692E4 | 3 | 1 | 0 | 0 | 0 | 0 | 0 | 0 | 1 | 0 | 1 | 117 | 124 |  |  | PEAKS DB |
| K.DFVYGQGASPANAVVGHYTQ.I | Y | 74.01 | 2079.9651 | 20 | 0.2 | 1040.9900 | 2 | 17.01 | 8 | F8:7119 | DaRuMH\_F7B.raw |  |  |  |  |  |  |  | 5.4281E6 |  |  | 1 | 0 | 0 | 0 | 0 | 0 | 0 | 0 | 1 | 0 | 0 | 100 | 119 |  |  | PEAKS DB |
| K.DFVYGQGASPAN.A | Y | 73.20 | 1224.5410 | 12 | 0.1 | 613.2778 | 2 | 13.66 | 8 | F8:4318 | DaRuMH\_F7B.raw |  |  |  |  |  |  |  | 8.3701E5 |  |  | 1 | 0 | 0 | 0 | 0 | 0 | 0 | 0 | 1 | 0 | 0 | 100 | 111 |  |  | PEAKS DB |
| K.DFVYGQGASPANAVVGHYT.Q | Y | 70.00 | 1951.9064 | 19 | -1.1 | 976.9594 | 2 | 17.93 | 8 | F8:7887 | DaRuMH\_F7B.raw |  |  |  |  |  |  |  | 1.4185E6 |  |  | 1 | 0 | 0 | 0 | 0 | 0 | 0 | 0 | 1 | 0 | 0 | 100 | 118 |  |  | PEAKS DB |
| M.EWYPEAAANAER.W | N | 68.66 | 1405.6262 | 12 | 0.1 | 703.8204 | 2 | 11.82 | 8 | F8:2734 | DaRuMH\_F7B.raw | 5.4508E4 |  |  |  |  |  |  | 4.7194E5 |  |  | 2 | 1 | 0 | 0 | 0 | 0 | 0 | 0 | 1 | 0 | 0 | 41 | 52 |  |  | PEAKS DB |
| K.DFVYGQGASPANAVVGHYTQIVWYKSYR.S | Y | 59.30 | 3175.5515 | 28 | 0.9 | 794.8959 | 4 | 25.73 | 8 | F8:13333 | DaRuMH\_F7B.raw |  |  |  |  |  |  |  | 1.4298E6 |  |  | 1 | 0 | 0 | 0 | 0 | 0 | 0 | 0 | 1 | 0 | 0 | 100 | 127 |  |  | PEAKS DB |
| N.VDFDSESPR.K | N | 56.99 | 1050.4618 | 9 | -0.7 | 526.2378 | 2 | 11.30 | 8 | F8:2216 | DaRuMH\_F7B.raw |  |  |  |  |  |  |  | 1.059E5 |  |  | 1 | 0 | 0 | 0 | 0 | 0 | 0 | 0 | 1 | 0 | 0 | 3 | 11 |  |  | PEAKS DB |
| E.KDFVYGQGASPANAVVGHYTQIVWYK.S | Y | 55.89 | 2897.4500 | 26 | 0.5 | 725.3701 | 4 | 19.73 | 8 | F8:9286 | DaRuMH\_F7B.raw |  |  |  |  |  |  |  | 5.4726E6 |  |  | 1 | 0 | 0 | 0 | 0 | 0 | 0 | 0 | 1 | 0 | 0 | 99 | 124 |  |  | PEAKS DB |
| total 14 peptides |
| --- |

A0A1I9KNP0|VSPH1\_VIPAA

back to list

  

| Protein Coverage
| Supporting Peptides
|

Protein Coverage:

Supporting Peptides:

| Peptide | Uniq | -10lgP | Mass | Length | ppm | m/z | z | RT | Fraction | Scan | Source File | Area F1 | Area F10 | Area F2 | Area F3 | Area F4 | Area F5 | Area F7A | Area F7B | Area F8 | Area F9 | #Feature | #Feature F1 | #Feature F10 | #Feature F2 | #Feature F3 | #Feature F4 | #Feature F5 | #Feature F7A | #Feature F7B | #Feature F8 | #Feature F9 | Start | End | PTM | AScore | Found By |
| --- | --- | --- | --- | --- | --- | --- | --- | --- | --- | --- | --- | --- | --- | --- | --- | --- | --- | --- | --- | --- | --- | --- | --- | --- | --- | --- | --- | --- | --- | --- | --- | --- | --- | --- | --- | --- | --- |
| L.VIGGDEC(+57.02)NINEHPFLVALHTAR.S | N | 100.05 | 2461.2173 | 22 | -1.0 | 821.4122 | 3 | 12.77 | 8 | F8:3513 | DaRuMH\_F7B.raw |  |  |  |  |  |  | 4.4366E6 | 4.7169E7 | 0 |  | 3 | 0 | 0 | 0 | 0 | 0 | 0 | 1 | 2 | 0 | 0 | 25 | 46 | Carbamidomethylation | C7:Carbamidomethylation:1000.00 | PEAKS DB |
| R.TLC(+57.02)AGILQGGIDSC(+57.02)K.V | N | 92.56 | 1591.7698 | 15 | -0.2 | 796.8920 | 2 | 13.38 | 8 | F8:4045 | DaRuMH\_F7B.raw | 1.1044E7 |  |  |  |  |  | 9.9073E6 | 8.8801E7 | 2.2455E7 | 1.2588E6 | 10 | 2 | 0 | 0 | 0 | 0 | 0 | 2 | 2 | 2 | 2 | 189 | 203 | Carbamidomethylation | C3:Carbamidomethylation:1000.00;C14:Carbamidomethylation:1000.00 | PEAKS DB |
| R.FYC(+57.02)AGTLINQEWVLTAAR.C | N | 84.88 | 2112.0461 | 18 | 0.6 | 1057.0310 | 2 | 35.63 | 8 | F8:19679 | DaRuMH\_F7B.raw |  |  |  |  |  |  |  | 1.8188E7 | 1.6959E6 | 3.2784E5 | 5 | 0 | 0 | 0 | 0 | 0 | 0 | 0 | 2 | 1 | 2 | 50 | 67 | Carbamidomethylation | C3:Carbamidomethylation:1000.00 | PEAKS DB |
| L.SLPSSPPSVGSVC(+57.02)R.I | N | 81.50 | 1428.7031 | 14 | 0.2 | 715.3590 | 2 | 11.65 | 8 | F8:2499 | DaRuMH\_F7B.raw |  |  |  |  |  |  | 2.3471E6 | 1.3457E7 | 5.5106E6 |  | 3 | 0 | 0 | 0 | 0 | 0 | 0 | 1 | 1 | 1 | 0 | 132 | 145 | Carbamidomethylation | C13:Carbamidomethylation:1000.00 | PEAKS DB |
| K.FFC(+57.02)LSSK.T | N | 67.85 | 887.4211 | 7 | -0.6 | 444.7176 | 2 | 12.08 | 8 | F8:2955 | DaRuMH\_F7B.raw | 2.546E6 |  |  |  |  |  | 6.2481E5 | 9.2089E6 | 3.0567E6 | 5.9046E5 | 5 | 1 | 0 | 0 | 0 | 0 | 0 | 1 | 1 | 1 | 1 | 98 | 104 | Carbamidomethylation | C3:Carbamidomethylation:1000.00 | PEAKS DB |
| K.RPVNDSTHIAPLSLPSSPPSVGSVC(+57.02)R.I | Y | 65.04 | 2729.3918 | 26 | 0.7 | 683.3557 | 4 | 12.10 | 9 | F9:2817 | DaRuMH\_F8.raw |  |  |  |  |  |  |  |  | 6.8135E5 |  | 1 | 0 | 0 | 0 | 0 | 0 | 0 | 0 | 0 | 1 | 0 | 120 | 145 | Carbamidomethylation | C25:Carbamidomethylation:1000.00 | PEAKS DB |
| L.INQEWVLTAAR.C | N | 63.82 | 1299.6935 | 11 | -1.1 | 650.8533 | 2 | 14.08 | 8 | F8:4680 | DaRuMH\_F7B.raw |  |  |  |  |  |  |  | 7.9259E5 |  |  | 1 | 0 | 0 | 0 | 0 | 0 | 0 | 0 | 1 | 0 | 0 | 57 | 67 |  |  | PEAKS DB |
| T.LINQEWVLTAAR.C | N | 62.20 | 1412.7776 | 12 | 0.8 | 707.3966 | 2 | 18.28 | 8 | F8:8111 | DaRuMH\_F7B.raw |  |  |  |  |  |  |  | 6.3653E5 |  |  | 1 | 0 | 0 | 0 | 0 | 0 | 0 | 0 | 1 | 0 | 0 | 56 | 67 |  |  | PEAKS DB |
| G.GDEC(+57.02)NINEHPFLVALHTAR.S | N | 61.35 | 2192.0432 | 19 | -0.4 | 549.0179 | 4 | 12.08 | 8 | F8:2965 | DaRuMH\_F7B.raw |  |  |  |  |  |  |  | 9.861E4 |  |  | 1 | 0 | 0 | 0 | 0 | 0 | 0 | 0 | 1 | 0 | 0 | 28 | 46 | Carbamidomethylation | C4:Carbamidomethylation:1000.00 | PEAKS DB |
| C.AGTLINQEWVLTAAR.C | N | 60.47 | 1641.8838 | 15 | 0.6 | 821.9496 | 2 | 23.66 | 8 | F8:12367 | DaRuMH\_F7B.raw |  |  |  |  |  |  |  | 3.7209E6 |  |  | 1 | 0 | 0 | 0 | 0 | 0 | 0 | 0 | 1 | 0 | 0 | 53 | 67 |  |  | PEAKS DB |
| Y.C(+57.02)AGTLINQEWVLTAAR.C | N | 57.21 | 1801.9144 | 16 | 0.5 | 901.9649 | 2 | 24.60 | 8 | F8:12913 | DaRuMH\_F7B.raw |  |  |  |  |  |  |  | 1.5416E6 |  |  | 1 | 0 | 0 | 0 | 0 | 0 | 0 | 0 | 1 | 0 | 0 | 52 | 67 | Carbamidomethylation | C1:Carbamidomethylation:1000.00 | PEAKS DB |
| C.AGILQGGIDSC(+57.02)K.V | N | 56.15 | 1217.6074 | 12 | -1.7 | 609.8099 | 2 | 11.74 | 8 | F8:2646 | DaRuMH\_F7B.raw |  |  |  |  |  |  |  | 3.398E5 |  |  | 1 | 0 | 0 | 0 | 0 | 0 | 0 | 0 | 1 | 0 | 0 | 192 | 203 | Carbamidomethylation | C11:Carbamidomethylation:1000.00 | PEAKS DB |
| K.TYTRWDKDIMLIR.L | N | 54.25 | 1709.8923 | 13 | -0.6 | 428.4801 | 4 | 12.61 | 8 | F8:3435 | DaRuMH\_F7B.raw |  |  |  |  |  |  |  | 5.1875E4 |  |  | 1 | 0 | 0 | 0 | 0 | 0 | 0 | 0 | 1 | 0 | 0 | 105 | 117 |  |  | PEAKS DB |
| total 13 peptides |
| --- |

P0DPS3|VASP1\_VIPAA

back to list

  

| Protein Coverage
| Supporting Peptides
|

Protein Coverage:

Supporting Peptides:

| Peptide | Uniq | -10lgP | Mass | Length | ppm | m/z | z | RT | Fraction | Scan | Source File | Area F1 | Area F10 | Area F2 | Area F3 | Area F4 | Area F5 | Area F7A | Area F7B | Area F8 | Area F9 | #Feature | #Feature F1 | #Feature F10 | #Feature F2 | #Feature F3 | #Feature F4 | #Feature F5 | #Feature F7A | #Feature F7B | #Feature F8 | #Feature F9 | Start | End | PTM | AScore | Found By |
| --- | --- | --- | --- | --- | --- | --- | --- | --- | --- | --- | --- | --- | --- | --- | --- | --- | --- | --- | --- | --- | --- | --- | --- | --- | --- | --- | --- | --- | --- | --- | --- | --- | --- | --- | --- | --- | --- |
| VIGGDEC(+57.02)NINEHPFLVALHTAR.X | N | 100.05 | 2461.2173 | 22 | -1.0 | 821.4122 | 3 | 12.77 | 8 | F8:3513 | DaRuMH\_F7B.raw |  |  |  |  |  |  | 4.4366E6 | 4.7169E7 | 0 |  | 3 | 0 | 0 | 0 | 0 | 0 | 0 | 1 | 2 | 0 | 0 | 1 | 22 | Carbamidomethylation | C7:Carbamidomethylation:1000.00 | PEAKS DB |
| R.TLC(+57.02)AGILQGGIDSC(+57.02)K.G | N | 92.56 | 1591.7698 | 15 | -0.2 | 796.8920 | 2 | 13.38 | 8 | F8:4045 | DaRuMH\_F7B.raw | 1.1044E7 |  |  |  |  |  | 9.9073E6 | 8.8801E7 | 2.2455E7 | 1.2588E6 | 10 | 2 | 0 | 0 | 0 | 0 | 0 | 2 | 2 | 2 | 2 | 165 | 179 | Carbamidomethylation | C3:Carbamidomethylation:1000.00;C14:Carbamidomethylation:1000.00 | PEAKS DB |
| R.FYC(+57.02)AGTLINQEWVLTAAR.C | N | 84.88 | 2112.0461 | 18 | 0.6 | 1057.0310 | 2 | 35.63 | 8 | F8:19679 | DaRuMH\_F7B.raw |  |  |  |  |  |  |  | 1.8188E7 | 1.6959E6 | 3.2784E5 | 5 | 0 | 0 | 0 | 0 | 0 | 0 | 0 | 2 | 1 | 2 | 26 | 43 | Carbamidomethylation | C3:Carbamidomethylation:1000.00 | PEAKS DB |
| VIGGDEC(+57.02)NINEHPF.L | N | 83.37 | 1599.6987 | 14 | -0.4 | 800.8563 | 2 | 12.06 | 10 | F10:2839 | DaRuMH\_F9.raw | 2.1782E5 |  |  |  |  |  |  | 1.1833E6 | 2.9737E5 | 1.7159E6 | 4 | 1 | 0 | 0 | 0 | 0 | 0 | 0 | 1 | 1 | 1 | 1 | 14 | Carbamidomethylation | C7:Carbamidomethylation:1000.00 | PEAKS DB |
| VIGGDEC(+57.02)NINEHPFLVALH.T | Y | 64.14 | 2133.0312 | 19 | 3.5 | 712.0186 | 3 | 17.46 | 7 | F7:6979 | DaRuMH\_F7A.raw |  |  |  |  |  |  | 7.5314E5 | 6.023E6 |  |  | 2 | 0 | 0 | 0 | 0 | 0 | 0 | 1 | 1 | 0 | 0 | 1 | 19 | Carbamidomethylation | C7:Carbamidomethylation:1000.00 | PEAKS DB |
| L.INQEWVLTAAR.C | N | 63.82 | 1299.6935 | 11 | -1.1 | 650.8533 | 2 | 14.08 | 8 | F8:4680 | DaRuMH\_F7B.raw |  |  |  |  |  |  |  | 7.9259E5 |  |  | 1 | 0 | 0 | 0 | 0 | 0 | 0 | 0 | 1 | 0 | 0 | 33 | 43 |  |  | PEAKS DB |
| T.LINQEWVLTAAR.C | N | 62.20 | 1412.7776 | 12 | 0.8 | 707.3966 | 2 | 18.28 | 8 | F8:8111 | DaRuMH\_F7B.raw |  |  |  |  |  |  |  | 6.3653E5 |  |  | 1 | 0 | 0 | 0 | 0 | 0 | 0 | 0 | 1 | 0 | 0 | 32 | 43 |  |  | PEAKS DB |
| G.GDEC(+57.02)NINEHPFLVALHTAR.X | N | 61.35 | 2192.0432 | 19 | -0.4 | 549.0179 | 4 | 12.08 | 8 | F8:2965 | DaRuMH\_F7B.raw |  |  |  |  |  |  |  | 9.861E4 |  |  | 1 | 0 | 0 | 0 | 0 | 0 | 0 | 0 | 1 | 0 | 0 | 4 | 22 | Carbamidomethylation | C4:Carbamidomethylation:1000.00 | PEAKS DB |
| C.AGTLINQEWVLTAAR.C | N | 60.47 | 1641.8838 | 15 | 0.6 | 821.9496 | 2 | 23.66 | 8 | F8:12367 | DaRuMH\_F7B.raw |  |  |  |  |  |  |  | 3.7209E6 |  |  | 1 | 0 | 0 | 0 | 0 | 0 | 0 | 0 | 1 | 0 | 0 | 29 | 43 |  |  | PEAKS DB |
| Y.C(+57.02)AGTLINQEWVLTAAR.C | N | 57.21 | 1801.9144 | 16 | 0.5 | 901.9649 | 2 | 24.60 | 8 | F8:12913 | DaRuMH\_F7B.raw |  |  |  |  |  |  |  | 1.5416E6 |  |  | 1 | 0 | 0 | 0 | 0 | 0 | 0 | 0 | 1 | 0 | 0 | 28 | 43 | Carbamidomethylation | C1:Carbamidomethylation:1000.00 | PEAKS DB |
| C.AGILQGGIDSC(+57.02)K.G | N | 56.15 | 1217.6074 | 12 | -1.7 | 609.8099 | 2 | 11.74 | 8 | F8:2646 | DaRuMH\_F7B.raw |  |  |  |  |  |  |  | 3.398E5 |  |  | 1 | 0 | 0 | 0 | 0 | 0 | 0 | 0 | 1 | 0 | 0 | 168 | 179 | Carbamidomethylation | C11:Carbamidomethylation:1000.00 | PEAKS DB |
| X.TYTRWDKDIMLIR.L | N | 54.25 | 1709.8923 | 13 | -0.6 | 428.4801 | 4 | 12.61 | 8 | F8:3435 | DaRuMH\_F7B.raw |  |  |  |  |  |  |  | 5.1875E4 |  |  | 1 | 0 | 0 | 0 | 0 | 0 | 0 | 0 | 1 | 0 | 0 | 81 | 93 |  |  | PEAKS DB |
| total 12 peptides |
| --- |

E5L0E4|VSPB\_DABSI

back to list

  

| Protein Coverage
| Supporting Peptides
|

Protein Coverage:

Supporting Peptides:

| Peptide | Uniq | -10lgP | Mass | Length | ppm | m/z | z | RT | Fraction | Scan | Source File | Area F1 | Area F10 | Area F2 | Area F3 | Area F4 | Area F5 | Area F7A | Area F7B | Area F8 | Area F9 | #Feature | #Feature F1 | #Feature F10 | #Feature F2 | #Feature F3 | #Feature F4 | #Feature F5 | #Feature F7A | #Feature F7B | #Feature F8 | #Feature F9 | Start | End | PTM | AScore | Found By |
| --- | --- | --- | --- | --- | --- | --- | --- | --- | --- | --- | --- | --- | --- | --- | --- | --- | --- | --- | --- | --- | --- | --- | --- | --- | --- | --- | --- | --- | --- | --- | --- | --- | --- | --- | --- | --- | --- |
| L.VVGGDEC(+57.02)NINEHR.S | N | 96.07 | 1497.6630 | 13 | -1.0 | 749.8380 | 2 | 10.70 | 9 | F9:1591 | DaRuMH\_F8.raw | 1.9072E7 |  |  |  |  |  | 3.6253E5 | 1.0498E7 | 2.9349E7 | 8.3285E5 | 8 | 2 | 0 | 0 | 0 | 0 | 0 | 1 | 2 | 2 | 1 | 25 | 37 | Carbamidomethylation | C7:Carbamidomethylation:1000.00 | PEAKS DB |
| K.TSTYIAPLSLPSSPPR.V | Y | 95.75 | 1685.8988 | 16 | 0.6 | 843.9572 | 2 | 18.23 | 9 | F9:6909 | DaRuMH\_F8.raw | 1.232E7 |  |  |  |  |  | 1.2086E7 | 9.646E7 | 6.3099E7 |  | 7 | 1 | 0 | 0 | 0 | 0 | 0 | 2 | 2 | 2 | 0 | 120 | 135 |  |  | PEAKS DB |
| N.SFGC(+57.02)SGTLINQQWVLSAVHC(+57.02)DM(+15.99)ENVR.I | Y | 74.89 | 3023.3689 | 26 | 0.9 | 1008.7979 | 3 | 26.85 | 9 | F9:10285 | DaRuMH\_F8.raw |  |  |  |  |  |  |  |  | 5.2695E5 |  | 1 | 0 | 0 | 0 | 0 | 0 | 0 | 0 | 0 | 1 | 0 | 46 | 71 | Carbamidomethylation; Oxidation (M) | C4:Carbamidomethylation:1000.00;C20:Carbamidomethylation:1000.00;M22:Oxidation (M):1000.00 | PEAKS DB |
| K.VYDYTDWIQSIIAGN.T | Y | 73.53 | 1756.8308 | 15 | 0.5 | 879.4232 | 2 | 71.48 | 8 | F8:31661 | DaRuMH\_F7B.raw |  |  |  |  |  |  |  | 1.1491E7 |  |  | 1 | 0 | 0 | 0 | 0 | 0 | 0 | 0 | 1 | 0 | 0 | 235 | 249 |  |  | PEAKS DB |
| L.SAVHC(+57.02)DMENVR.I | Y | 72.75 | 1316.5602 | 11 | 0.3 | 439.8608 | 3 | 10.69 | 8 | F8:1656 | DaRuMH\_F7B.raw |  |  |  |  |  |  |  | 2.6552E4 | 8.0201E3 |  | 2 | 0 | 0 | 0 | 0 | 0 | 0 | 0 | 1 | 1 | 0 | 61 | 71 | Carbamidomethylation | C5:Carbamidomethylation:1000.00 | PEAKS DB |
| C.SGTLINQQWVLSAVHC(+57.02)DMENVR.I | Y | 70.29 | 2556.2214 | 22 | 0.5 | 853.0815 | 3 | 29.36 | 9 | F9:11094 | DaRuMH\_F8.raw |  |  |  |  |  |  |  |  | 5.3341E5 |  | 1 | 0 | 0 | 0 | 0 | 0 | 0 | 0 | 0 | 1 | 0 | 50 | 71 | Carbamidomethylation | C16:Carbamidomethylation:1000.00 | PEAKS DB |
| V.GGDEC(+57.02)NINEHR.S | N | 67.81 | 1299.5262 | 11 | 0.2 | 650.7705 | 2 | 10.70 | 9 | F9:1597 | DaRuMH\_F8.raw |  |  |  |  |  |  |  | 2.6409E5 | 7.79E5 |  | 2 | 0 | 0 | 0 | 0 | 0 | 0 | 0 | 1 | 1 | 0 | 27 | 37 | Carbamidomethylation | C5:Carbamidomethylation:1000.00 | PEAKS DB |
| Y.IAPLSLPSSPPR.V | N | 63.55 | 1233.7080 | 12 | 0.4 | 617.8615 | 2 | 13.19 | 8 | F8:3892 | DaRuMH\_F7B.raw |  |  |  |  |  |  | 7.6058E5 | 3.9765E6 | 3.6374E6 |  | 3 | 0 | 0 | 0 | 0 | 0 | 0 | 1 | 1 | 1 | 0 | 124 | 135 |  |  | PEAKS DB |
| Q.SIIAGNTAATC(+57.02)PP | N | 63.48 | 1271.6179 | 13 | 0.0 | 636.8162 | 2 | 12.17 | 10 | F10:2939 | DaRuMH\_F9.raw |  |  |  |  |  |  |  | 5.7393E5 |  | 1.5935E6 | 2 | 0 | 0 | 0 | 0 | 0 | 0 | 0 | 1 | 0 | 1 | 244 | 256 | Carbamidomethylation | C11:Carbamidomethylation:1000.00 | PEAKS DB |
| K.VYDYTDWIQSIIAGNTAATC(+57.02)PP | Y | 63.11 | 2455.1365 | 22 | 0.3 | 1228.5759 | 2 | 72.21 | 8 | F8:31942 | DaRuMH\_F7B.raw |  |  |  |  |  |  |  | 9.0821E7 |  |  | 1 | 0 | 0 | 0 | 0 | 0 | 0 | 0 | 1 | 0 | 0 | 235 | 256 | Carbamidomethylation | C20:Carbamidomethylation:1000.00 | PEAKS DB |
| R.FFC(+57.02)LSNK.N | N | 60.97 | 914.4320 | 7 | 0.1 | 458.2233 | 2 | 11.99 | 8 | F8:2873 | DaRuMH\_F7B.raw | 1.4908E6 |  |  |  |  | 7.7161E4 | 1.1075E6 | 1.618E7 | 9.6782E6 |  | 5 | 1 | 0 | 0 | 0 | 0 | 1 | 1 | 1 | 1 | 0 | 94 | 100 | Carbamidomethylation | C3:Carbamidomethylation:1000.00 | PEAKS DB |
| R.NNAEIRLPEER.F | Y | 60.36 | 1339.6843 | 11 | 1.2 | 447.5692 | 3 | 11.35 | 8 | F8:2260 | DaRuMH\_F7B.raw |  |  |  |  |  |  |  | 1.9417E5 |  |  | 1 | 0 | 0 | 0 | 0 | 0 | 0 | 0 | 1 | 0 | 0 | 83 | 93 |  |  | PEAKS DB |
| K.WDKDIMLIK.L | N | 53.69 | 1160.6263 | 9 | 0.0 | 581.3204 | 2 | 12.61 | 8 | F8:3439 | DaRuMH\_F7B.raw |  |  |  |  |  |  |  | 3.5006E4 |  |  | 1 | 0 | 0 | 0 | 0 | 0 | 0 | 0 | 1 | 0 | 0 | 105 | 113 |  |  | PEAKS DB |
| total 13 peptides |
| --- |

Q9PT40|VSP2\_MACLB

back to list

  

| Protein Coverage
| Supporting Peptides
|

Protein Coverage:

Supporting Peptides:

| Peptide | Uniq | -10lgP | Mass | Length | ppm | m/z | z | RT | Fraction | Scan | Source File | Area F1 | Area F10 | Area F2 | Area F3 | Area F4 | Area F5 | Area F7A | Area F7B | Area F8 | Area F9 | #Feature | #Feature F1 | #Feature F10 | #Feature F2 | #Feature F3 | #Feature F4 | #Feature F5 | #Feature F7A | #Feature F7B | #Feature F8 | #Feature F9 | Start | End | PTM | AScore | Found By |
| --- | --- | --- | --- | --- | --- | --- | --- | --- | --- | --- | --- | --- | --- | --- | --- | --- | --- | --- | --- | --- | --- | --- | --- | --- | --- | --- | --- | --- | --- | --- | --- | --- | --- | --- | --- | --- | --- |
| R.TLC(+57.02)AGILQGGIDSC(+57.02)K.V | N | 92.56 | 1591.7698 | 15 | -0.2 | 796.8920 | 2 | 13.38 | 8 | F8:4045 | DaRuMH\_F7B.raw | 1.1044E7 |  |  |  |  |  | 9.9073E6 | 8.8801E7 | 2.2455E7 | 1.2588E6 | 10 | 2 | 0 | 0 | 0 | 0 | 0 | 2 | 2 | 2 | 2 | 189 | 203 | Carbamidomethylation | C3:Carbamidomethylation:1000.00;C14:Carbamidomethylation:1000.00 | PEAKS DB |
| K.VDNGGPLIC(+57.02)NGQIQGIVSWGGHPC(+57.02).A | Y | 89.97 | 2534.1794 | 24 | 0.4 | 1268.0975 | 2 | 30.25 | 9 | F9:11522 | DaRuMH\_F8.raw |  |  |  |  |  |  |  | 1.4033E7 | 3.0772E6 |  | 2 | 0 | 0 | 0 | 0 | 0 | 0 | 0 | 1 | 1 | 0 | 204 | 227 | Carbamidomethylation | C9:Carbamidomethylation:1000.00;C24:Carbamidomethylation:1000.00 | PEAKS DB |
| R.FYC(+57.02)AGTLINQEWVLTAAR.C | N | 84.88 | 2112.0461 | 18 | 0.6 | 1057.0310 | 2 | 35.63 | 8 | F8:19679 | DaRuMH\_F7B.raw |  |  |  |  |  |  |  | 1.8188E7 | 1.6959E6 | 3.2784E5 | 5 | 0 | 0 | 0 | 0 | 0 | 0 | 0 | 2 | 1 | 2 | 50 | 67 | Carbamidomethylation | C3:Carbamidomethylation:1000.00 | PEAKS DB |
| L.SLPSSPPSVGSVC(+57.02)R.I | N | 81.50 | 1428.7031 | 14 | 0.2 | 715.3590 | 2 | 11.65 | 8 | F8:2499 | DaRuMH\_F7B.raw |  |  |  |  |  |  | 2.3471E6 | 1.3457E7 | 5.5106E6 |  | 3 | 0 | 0 | 0 | 0 | 0 | 0 | 1 | 1 | 1 | 0 | 132 | 145 | Carbamidomethylation | C13:Carbamidomethylation:1000.00 | PEAKS DB |
| K.FFC(+57.02)LSSK.T | N | 67.85 | 887.4211 | 7 | -0.6 | 444.7176 | 2 | 12.08 | 8 | F8:2955 | DaRuMH\_F7B.raw | 2.546E6 |  |  |  |  |  | 6.2481E5 | 9.2089E6 | 3.0567E6 | 5.9046E5 | 5 | 1 | 0 | 0 | 0 | 0 | 0 | 1 | 1 | 1 | 1 | 98 | 104 | Carbamidomethylation | C3:Carbamidomethylation:1000.00 | PEAKS DB |
| L.INQEWVLTAAR.C | N | 63.82 | 1299.6935 | 11 | -1.1 | 650.8533 | 2 | 14.08 | 8 | F8:4680 | DaRuMH\_F7B.raw |  |  |  |  |  |  |  | 7.9259E5 |  |  | 1 | 0 | 0 | 0 | 0 | 0 | 0 | 0 | 1 | 0 | 0 | 57 | 67 |  |  | PEAKS DB |
| K.VTYPDVPHC(+57.02)AN.I | N | 62.92 | 1271.5604 | 11 | 0.2 | 636.7876 | 2 | 11.48 | 8 | F8:2367 | DaRuMH\_F7B.raw | 9.73E5 |  |  |  |  |  |  | 4.1766E6 | 1.4461E6 |  | 3 | 1 | 0 | 0 | 0 | 0 | 0 | 0 | 1 | 1 | 0 | 157 | 167 | Carbamidomethylation | C9:Carbamidomethylation:1000.00 | PEAKS DB |
| T.LINQEWVLTAAR.C | N | 62.20 | 1412.7776 | 12 | 0.8 | 707.3966 | 2 | 18.28 | 8 | F8:8111 | DaRuMH\_F7B.raw |  |  |  |  |  |  |  | 6.3653E5 |  |  | 1 | 0 | 0 | 0 | 0 | 0 | 0 | 0 | 1 | 0 | 0 | 56 | 67 |  |  | PEAKS DB |
| C.AGTLINQEWVLTAAR.C | N | 60.47 | 1641.8838 | 15 | 0.6 | 821.9496 | 2 | 23.66 | 8 | F8:12367 | DaRuMH\_F7B.raw |  |  |  |  |  |  |  | 3.7209E6 |  |  | 1 | 0 | 0 | 0 | 0 | 0 | 0 | 0 | 1 | 0 | 0 | 53 | 67 |  |  | PEAKS DB |
| Y.C(+57.02)AGTLINQEWVLTAAR.C | N | 57.21 | 1801.9144 | 16 | 0.5 | 901.9649 | 2 | 24.60 | 8 | F8:12913 | DaRuMH\_F7B.raw |  |  |  |  |  |  |  | 1.5416E6 |  |  | 1 | 0 | 0 | 0 | 0 | 0 | 0 | 0 | 1 | 0 | 0 | 52 | 67 | Carbamidomethylation | C1:Carbamidomethylation:1000.00 | PEAKS DB |
| C.AGILQGGIDSC(+57.02)K.V | N | 56.15 | 1217.6074 | 12 | -1.7 | 609.8099 | 2 | 11.74 | 8 | F8:2646 | DaRuMH\_F7B.raw |  |  |  |  |  |  |  | 3.398E5 |  |  | 1 | 0 | 0 | 0 | 0 | 0 | 0 | 0 | 1 | 0 | 0 | 192 | 203 | Carbamidomethylation | C11:Carbamidomethylation:1000.00 | PEAKS DB |
| K.VTYPDVPHC(+57.02)ANIN.M | N | 55.92 | 1498.6875 | 13 | 0.2 | 750.3512 | 2 | 11.80 | 8 | F8:2694 | DaRuMH\_F7B.raw |  |  |  |  |  |  |  | 3.137E5 |  |  | 1 | 0 | 0 | 0 | 0 | 0 | 0 | 0 | 1 | 0 | 0 | 157 | 169 | Carbamidomethylation | C9:Carbamidomethylation:1000.00 | PEAKS DB |
| K.TYTRWDKDIMLIR.L | N | 54.25 | 1709.8923 | 13 | -0.6 | 428.4801 | 4 | 12.61 | 8 | F8:3435 | DaRuMH\_F7B.raw |  |  |  |  |  |  |  | 5.1875E4 |  |  | 1 | 0 | 0 | 0 | 0 | 0 | 0 | 0 | 1 | 0 | 0 | 105 | 117 |  |  | PEAKS DB |
| total 13 peptides |
| --- |

Q38L02|SLA\_DABSI

back to list

  

| Protein Coverage
| Supporting Peptides
|

Protein Coverage:

Supporting Peptides:

| Peptide | Uniq | -10lgP | Mass | Length | ppm | m/z | z | RT | Fraction | Scan | Source File | Area F1 | Area F10 | Area F2 | Area F3 | Area F4 | Area F5 | Area F7A | Area F7B | Area F8 | Area F9 | #Feature | #Feature F1 | #Feature F10 | #Feature F2 | #Feature F3 | #Feature F4 | #Feature F5 | #Feature F7A | #Feature F7B | #Feature F8 | #Feature F9 | Start | End | PTM | AScore | Found By |
| --- | --- | --- | --- | --- | --- | --- | --- | --- | --- | --- | --- | --- | --- | --- | --- | --- | --- | --- | --- | --- | --- | --- | --- | --- | --- | --- | --- | --- | --- | --- | --- | --- | --- | --- | --- | --- | --- |
| A.SIESVEEANFVAQLASETLTK.S | Y | 91.79 | 2265.1375 | 21 | 0.0 | 1133.5760 | 2 | 47.27 | 10 | F10:22892 | DaRuMH\_F9.raw |  |  |  |  |  |  |  |  |  | 3.3074E6 | 2 | 0 | 0 | 0 | 0 | 0 | 0 | 0 | 0 | 0 | 2 | 64 | 84 |  |  | PEAKS DB |
| K.YHEWITLPC(+57.02)GDK.N | Y | 88.18 | 1517.6973 | 12 | 0.9 | 759.8566 | 2 | 12.12 | 10 | F10:2919 | DaRuMH\_F9.raw | 3.7522E5 |  |  |  |  |  | 7.8149E4 | 3.0807E6 | 5.3959E6 | 7.7251E6 | 10 | 2 | 0 | 0 | 0 | 0 | 0 | 1 | 2 | 2 | 3 | 132 | 143 | Carbamidomethylation | C9:Carbamidomethylation:1000.00 | PEAKS DB |
| C.SSHWTDGSAVSYETVTK.Y | Y | 87.54 | 1853.8431 | 17 | -4.9 | 927.9243 | 2 | 11.53 | 10 | F10:2376 | DaRuMH\_F9.raw |  |  |  |  |  |  |  |  |  | 6.6792E5 | 2 | 0 | 0 | 0 | 0 | 0 | 0 | 0 | 0 | 0 | 2 | 103 | 119 |  |  | PEAKS DB |
| R.QQC(+57.02)SSHWTDGSAVSYETVTK.Y | Y | 78.83 | 2269.9910 | 20 | 1.1 | 757.6718 | 3 | 11.55 | 9 | F9:2348 | DaRuMH\_F8.raw |  |  |  |  |  |  |  |  | 7.0791E5 |  | 1 | 0 | 0 | 0 | 0 | 0 | 0 | 0 | 0 | 1 | 0 | 100 | 119 | Carbamidomethylation | C3:Carbamidomethylation:1000.00 | PEAKS DB |
| H.LASIESVEEANFVAQLASETLTK.S | Y | 74.72 | 2449.2588 | 23 | 0.6 | 817.4274 | 3 | 52.76 | 8 | F8:26581 | DaRuMH\_F7B.raw |  |  |  |  |  |  |  | 2.4864E6 |  | 1.7538E7 | 3 | 0 | 0 | 0 | 0 | 0 | 0 | 0 | 1 | 0 | 2 | 62 | 84 |  |  | PEAKS DB |
| H.WTDGSAVSYETVTK.Y | Y | 73.28 | 1542.7202 | 14 | 1.2 | 772.3683 | 2 | 11.77 | 10 | F10:2613 | DaRuMH\_F9.raw |  |  |  |  |  |  |  |  |  | 7.2737E5 | 1 | 0 | 0 | 0 | 0 | 0 | 0 | 0 | 0 | 0 | 1 | 106 | 119 |  |  | PEAKS DB |
| E.SVEEANFVAQLASETLTK.S | Y | 69.79 | 1935.9789 | 18 | 0.0 | 968.9967 | 2 | 37.64 | 8 | F8:20396 | DaRuMH\_F7B.raw |  |  |  |  |  |  |  | 1.1109E5 |  |  | 1 | 0 | 0 | 0 | 0 | 0 | 0 | 0 | 1 | 0 | 0 | 67 | 84 |  |  | PEAKS DB |
| K.YHAWIGLR.D | Y | 69.12 | 1014.5399 | 8 | -0.5 | 508.2770 | 2 | 11.75 | 10 | F10:2569 | DaRuMH\_F9.raw | 7.9034E4 |  |  |  |  |  |  | 6.139E5 | 9.3808E5 | 1.1824E6 | 4 | 1 | 0 | 0 | 0 | 0 | 0 | 0 | 1 | 1 | 1 | 87 | 94 |  |  | PEAKS DB |
| F.VAQLASETLTK.S | Y | 57.04 | 1159.6448 | 11 | -0.3 | 580.8295 | 2 | 11.51 | 9 | F9:2308 | DaRuMH\_F8.raw |  |  |  |  |  |  |  |  | 4.7609E4 |  | 1 | 0 | 0 | 0 | 0 | 0 | 0 | 0 | 0 | 1 | 0 | 74 | 84 |  |  | PEAKS DB |
| W.HLASIESVEEANFVAQLASETLTK.S | Y | 55.24 | 2586.3176 | 24 | 0.1 | 1294.1663 | 2 | 42.45 | 9 | F9:15963 | DaRuMH\_F8.raw |  |  |  |  |  |  |  |  | 1.1437E6 |  | 2 | 0 | 0 | 0 | 0 | 0 | 0 | 0 | 0 | 2 | 0 | 61 | 84 |  |  | PEAKS DB |
| L.ASIESVEEANFVAQLASETLTK.S | Y | 54.24 | 2336.1746 | 22 | 0.7 | 1169.0953 | 2 | 47.76 | 8 | F8:24674 | DaRuMH\_F7B.raw |  |  |  |  |  |  |  | 1.1278E5 |  |  | 1 | 0 | 0 | 0 | 0 | 0 | 0 | 0 | 1 | 0 | 0 | 63 | 84 |  |  | PEAKS DB |
| total 11 peptides |
| --- |

Q910A1|PA2A1\_VIPAA

back to list

  

| Protein Coverage
| Supporting Peptides
|

Protein Coverage:

Supporting Peptides:

| Peptide | Uniq | -10lgP | Mass | Length | ppm | m/z | z | RT | Fraction | Scan | Source File | Area F1 | Area F10 | Area F2 | Area F3 | Area F4 | Area F5 | Area F7A | Area F7B | Area F8 | Area F9 | #Feature | #Feature F1 | #Feature F10 | #Feature F2 | #Feature F3 | #Feature F4 | #Feature F5 | #Feature F7A | #Feature F7B | #Feature F8 | #Feature F9 | Start | End | PTM | AScore | Found By |
| --- | --- | --- | --- | --- | --- | --- | --- | --- | --- | --- | --- | --- | --- | --- | --- | --- | --- | --- | --- | --- | --- | --- | --- | --- | --- | --- | --- | --- | --- | --- | --- | --- | --- | --- | --- | --- | --- |
| R.VAAIC(+57.02)FGENM(+15.99)NTYDK.K | Y | 90.97 | 1747.7545 | 15 | 0.5 | 874.8850 | 2 | 11.99 | 8 | F8:2856 | DaRuMH\_F7B.raw |  |  |  |  |  |  |  | 2.405E6 |  |  | 1 | 0 | 0 | 0 | 0 | 0 | 0 | 0 | 1 | 0 | 0 | 107 | 121 | Carbamidomethylation; Oxidation (M) | C5:Carbamidomethylation:1000.00;M10:Oxidation (M):1000.00 | PEAKS DB |
| R.VAAIC(+57.02)FGENMNTYDK.K | Y | 87.16 | 1731.7596 | 15 | 0.7 | 866.8877 | 2 | 13.59 | 8 | F8:4463 | DaRuMH\_F7B.raw |  |  |  |  |  |  | 1.9116E6 | 1.9089E7 |  |  | 2 | 0 | 0 | 0 | 0 | 0 | 0 | 1 | 1 | 0 | 0 | 107 | 121 | Carbamidomethylation | C5:Carbamidomethylation:1000.00 | PEAKS DB |
| D.IVC(+57.02)GGDDPC(+57.02)LR.A | N | 86.87 | 1260.5591 | 11 | -0.7 | 631.2864 | 2 | 11.30 | 8 | F8:2189 | DaRuMH\_F7B.raw |  |  |  |  |  |  |  | 1.7117E6 |  |  | 1 | 0 | 0 | 0 | 0 | 0 | 0 | 0 | 1 | 0 | 0 | 89 | 99 | Carbamidomethylation | C3:Carbamidomethylation:1000.00;C9:Carbamidomethylation:1000.00 | PEAKS DB |
| K.YM(+15.99)LYSLFDC(+57.02)K.E | N | 81.85 | 1354.5938 | 10 | 0.1 | 678.3042 | 2 | 21.36 | 8 | F8:10863 | DaRuMH\_F7B.raw |  |  |  |  |  |  | 1.83E6 | 6.2816E6 |  |  | 5 | 0 | 0 | 0 | 0 | 0 | 0 | 2 | 3 | 0 | 0 | 123 | 132 | Oxidation (M); Carbamidomethylation | M2:Oxidation (M):1000.00;C9:Carbamidomethylation:1000.00 | PEAKS DB |
| K.YMLYSLFDC(+57.02)K.E | N | 80.90 | 1338.5988 | 10 | 1.1 | 670.3074 | 2 | 27.75 | 8 | F8:14512 | DaRuMH\_F7B.raw |  |  |  |  |  |  |  | 9.2178E6 |  |  | 1 | 0 | 0 | 0 | 0 | 0 | 0 | 0 | 1 | 0 | 0 | 123 | 132 | Carbamidomethylation | C9:Carbamidomethylation:1000.00 | PEAKS DB |
| R.C(+57.02)C(+57.02)FVHDC(+57.02)C(+57.02)YGR.V | N | 71.07 | 1532.5417 | 11 | 2.9 | 767.2786 | 2 | 11.11 | 7 | F7:1942 | DaRuMH\_F7A.raw |  |  |  | 7.7102E5 | 7.7933E5 |  | 9.6866E5 | 7.6051E7 |  |  | 6 | 0 | 0 | 0 | 1 | 2 | 0 | 1 | 2 | 0 | 0 | 59 | 69 | Carbamidomethylation | C1:Carbamidomethylation:1000.00;C2:Carbamidomethylation:1000.00;C7:Carbamidomethylation:1000.00;C8:Carbamidomethylation:1000.00 | PEAKS DB |
| I.YYGC(+57.02)YC(+57.02)GWGGK.G | N | 70.74 | 1369.5220 | 11 | 0.3 | 685.7685 | 2 | 11.78 | 8 | F8:2687 | DaRuMH\_F7B.raw |  |  |  |  |  |  |  | 7.8895E5 |  |  | 1 | 0 | 0 | 0 | 0 | 0 | 0 | 0 | 1 | 0 | 0 | 39 | 49 | Carbamidomethylation | C4:Carbamidomethylation:1000.00;C6:Carbamidomethylation:1000.00 | PEAKS DB |
| Y.YGC(+57.02)YC(+57.02)GWGGK.G | N | 66.85 | 1206.4586 | 10 | -0.3 | 604.2364 | 2 | 11.56 | 8 | F8:2453 | DaRuMH\_F7B.raw |  |  |  |  | 2.4701E5 | 2.1367E7 | 1.6469E7 | 7.7105E6 |  |  | 4 | 0 | 0 | 0 | 0 | 1 | 1 | 1 | 1 | 0 | 0 | 40 | 49 | Carbamidomethylation | C3:Carbamidomethylation:1000.00;C5:Carbamidomethylation:1000.00 | PEAKS DB |
| Q.NGDIVC(+57.02)GGDDPC(+57.02)LR.A | N | 66.25 | 1546.6504 | 14 | -0.7 | 774.3319 | 2 | 11.60 | 8 | F8:2517 | DaRuMH\_F7B.raw |  |  |  | 2.5237E5 |  |  |  | 9.3572E5 |  |  | 2 | 0 | 0 | 0 | 1 | 0 | 0 | 0 | 1 | 0 | 0 | 86 | 99 | Carbamidomethylation | C6:Carbamidomethylation:1000.00;C12:Carbamidomethylation:1000.00 | PEAKS DB |
| A.IC(+57.02)FGENMNTYDK.K | Y | 66.10 | 1490.6169 | 12 | -3.9 | 746.3129 | 2 | 11.95 | 8 | F8:2854 | DaRuMH\_F7B.raw |  |  |  |  |  |  |  | 3.4539E5 |  |  | 1 | 0 | 0 | 0 | 0 | 0 | 0 | 0 | 1 | 0 | 0 | 110 | 121 | Carbamidomethylation | C2:Carbamidomethylation:1000.00 | PEAKS DB |
| N.GDIVC(+57.02)GGDDPC(+57.02)LR.A | N | 65.50 | 1432.6074 | 13 | 0.0 | 717.3110 | 2 | 11.60 | 5 | F5:2413 | DaRuMH\_F4.raw |  |  | 1.0971E5 | 1.1137E6 | 1.1053E6 |  |  | 1.6452E6 |  |  | 4 | 0 | 0 | 1 | 1 | 1 | 0 | 0 | 1 | 0 | 0 | 87 | 99 | Carbamidomethylation | C5:Carbamidomethylation:1000.00;C11:Carbamidomethylation:1000.00 | PEAKS DB |
| Y.GC(+57.02)YC(+57.02)GWGGK.G | N | 58.54 | 1043.3953 | 9 | -0.4 | 522.7047 | 2 | 11.29 | 6 | F6:2113 | DaRuMH\_F5.raw |  |  |  |  |  | 2.1095E6 |  | 4.3981E6 |  |  | 2 | 0 | 0 | 0 | 0 | 0 | 1 | 0 | 1 | 0 | 0 | 41 | 49 | Carbamidomethylation | C2:Carbamidomethylation:1000.00;C4:Carbamidomethylation:1000.00 | PEAKS DB |
| C.FVHDC(+57.02)C(+57.02)YGR.V | N | 56.61 | 1212.4805 | 9 | -0.3 | 405.1673 | 3 | 10.65 | 8 | F8:1634 | DaRuMH\_F7B.raw |  |  |  |  |  |  |  | 1.579E5 |  |  | 1 | 0 | 0 | 0 | 0 | 0 | 0 | 0 | 1 | 0 | 0 | 61 | 69 | Carbamidomethylation | C5:Carbamidomethylation:1000.00;C6:Carbamidomethylation:1000.00 | PEAKS DB |
| Y.SFQNGDIVC(+57.02)GGDDPC(+57.02)LR.A | N | 53.81 | 1908.8094 | 17 | 0.6 | 955.4126 | 2 | 12.06 | 1 | F1:2918 | DaRuMH\_F1.raw | 1.2126E5 |  |  |  |  |  |  |  |  |  | 1 | 1 | 0 | 0 | 0 | 0 | 0 | 0 | 0 | 0 | 0 | 83 | 99 | Carbamidomethylation | C9:Carbamidomethylation:1000.00;C15:Carbamidomethylation:1000.00 | PEAKS DB |
| R.VAAIC(+57.02)FGENMNTYDKK.Y | Y | 53.70 | 1859.8546 | 16 | -0.2 | 620.9587 | 3 | 11.82 | 8 | F8:2735 | DaRuMH\_F7B.raw |  |  |  |  |  |  |  | 5.3891E5 |  |  | 1 | 0 | 0 | 0 | 0 | 0 | 0 | 0 | 1 | 0 | 0 | 107 | 122 | Carbamidomethylation | C5:Carbamidomethylation:1000.00 | PEAKS DB |
| total 15 peptides |
| --- |

Q02471|PA2B4\_DABSI

back to list

  

| Protein Coverage
| Supporting Peptides
|

Protein Coverage:

Supporting Peptides:

| Peptide | Uniq | -10lgP | Mass | Length | ppm | m/z | z | RT | Fraction | Scan | Source File | Area F1 | Area F10 | Area F2 | Area F3 | Area F4 | Area F5 | Area F7A | Area F7B | Area F8 | Area F9 | #Feature | #Feature F1 | #Feature F10 | #Feature F2 | #Feature F3 | #Feature F4 | #Feature F5 | #Feature F7A | #Feature F7B | #Feature F8 | #Feature F9 | Start | End | PTM | AScore | Found By |
| --- | --- | --- | --- | --- | --- | --- | --- | --- | --- | --- | --- | --- | --- | --- | --- | --- | --- | --- | --- | --- | --- | --- | --- | --- | --- | --- | --- | --- | --- | --- | --- | --- | --- | --- | --- | --- | --- |
| N.YISYGC(+57.02)YC(+57.02)GWGGQGTPK.D | N | 96.29 | 1952.8185 | 17 | 2.1 | 977.4185 | 2 | 12.66 | 8 | F8:3720 | DaRuMH\_F7B.raw | 2.7838E6 |  |  |  |  |  | 5.7551E5 | 1.1722E8 | 1.2076E6 |  | 6 | 1 | 0 | 0 | 0 | 0 | 0 | 1 | 3 | 1 | 0 | 37 | 53 | Carbamidomethylation | C6:Carbamidomethylation:1000.00;C8:Carbamidomethylation:1000.00 | PEAKS DB |
| Y.ISYGC(+57.02)YC(+57.02)GWGGQGTPK.D | N | 94.64 | 1789.7552 | 16 | 0.8 | 895.8856 | 2 | 11.97 | 8 | F8:2858 | DaRuMH\_F7B.raw |  |  |  |  |  |  |  | 1.2387E6 |  |  | 1 | 0 | 0 | 0 | 0 | 0 | 0 | 0 | 1 | 0 | 0 | 38 | 53 | Carbamidomethylation | C5:Carbamidomethylation:1000.00;C7:Carbamidomethylation:1000.00 | PEAKS DB |
| Y.GC(+57.02)YC(+57.02)GWGGQGTPK.D | N | 80.61 | 1426.5758 | 13 | 0.8 | 714.2958 | 2 | 11.39 | 8 | F8:2290 | DaRuMH\_F7B.raw | 1.8108E5 |  |  |  |  |  |  | 4.0555E6 |  |  | 2 | 1 | 0 | 0 | 0 | 0 | 0 | 0 | 1 | 0 | 0 | 41 | 53 | Carbamidomethylation | C2:Carbamidomethylation:1000.00;C4:Carbamidomethylation:1000.00 | PEAKS DB |
| S.YGC(+57.02)YC(+57.02)GWGGQGTPK.D | N | 78.48 | 1589.6392 | 14 | 0.8 | 795.8275 | 2 | 11.62 | 8 | F8:2519 | DaRuMH\_F7B.raw |  |  |  |  |  |  |  | 5.925E5 |  |  | 1 | 0 | 0 | 0 | 0 | 0 | 0 | 0 | 1 | 0 | 0 | 40 | 53 | Carbamidomethylation | C3:Carbamidomethylation:1000.00;C5:Carbamidomethylation:1000.00 | PEAKS DB |
| Y.C(+57.02)GWGGQGTPK.D | N | 68.84 | 1046.4603 | 10 | -2.1 | 524.2363 | 2 | 10.97 | 8 | F8:1880 | DaRuMH\_F7B.raw | 4.3739E5 |  |  |  |  |  |  | 2.4018E6 |  |  | 2 | 1 | 0 | 0 | 0 | 0 | 0 | 0 | 1 | 0 | 0 | 44 | 53 | Carbamidomethylation | C1:Carbamidomethylation:1000.00 | PEAKS DB |
| C.YC(+57.02)GWGGQGTPK.D | N | 68.68 | 1209.5237 | 11 | -0.3 | 605.7689 | 2 | 11.30 | 8 | F8:2229 | DaRuMH\_F7B.raw |  |  |  |  |  |  |  | 6.5322E5 |  |  | 1 | 0 | 0 | 0 | 0 | 0 | 0 | 0 | 1 | 0 | 0 | 43 | 53 | Carbamidomethylation | C2:Carbamidomethylation:1000.00 | PEAKS DB |
| K.LAIYSYSFQR.G | Y | 62.92 | 1246.6345 | 10 | 0.7 | 624.3250 | 2 | 14.67 | 8 | F8:5175 | DaRuMH\_F7B.raw |  |  |  |  |  |  |  | 2.4555E5 | 2.9128E4 |  | 2 | 0 | 0 | 0 | 0 | 0 | 0 | 0 | 1 | 1 | 0 | 77 | 86 |  |  | PEAKS DB |
| total 7 peptides |
| --- |

P13647|K2C5\_HUMAN

back to list

  

| Protein Coverage
| Supporting Peptides
|

Protein Coverage:

Supporting Peptides:

| Peptide | Uniq | -10lgP | Mass | Length | ppm | m/z | z | RT | Fraction | Scan | Source File | Area F1 | Area F10 | Area F2 | Area F3 | Area F4 | Area F5 | Area F7A | Area F7B | Area F8 | Area F9 | #Feature | #Feature F1 | #Feature F10 | #Feature F2 | #Feature F3 | #Feature F4 | #Feature F5 | #Feature F7A | #Feature F7B | #Feature F8 | #Feature F9 | Start | End | PTM | AScore | Found By |
| --- | --- | --- | --- | --- | --- | --- | --- | --- | --- | --- | --- | --- | --- | --- | --- | --- | --- | --- | --- | --- | --- | --- | --- | --- | --- | --- | --- | --- | --- | --- | --- | --- | --- | --- | --- | --- | --- |
| R.NLDLDSIIAEVK.A | N | 84.25 | 1328.7188 | 12 | 0.3 | 665.3668 | 2 | 36.47 | 3 | F3:8177 | DaRuMH\_F2.raw | 1.633E6 | 5.015E5 | 9.1776E4 |  | 8.3506E5 |  | 5.5657E5 | 4.6037E5 | 2.1133E5 | 2.4217E6 | 8 | 1 | 1 | 1 | 0 | 1 | 0 | 1 | 1 | 1 | 1 | 332 | 343 |  |  | PEAKS DB |
| R.VSLAGAC(+57.02)GVGGYGSR.S | Y | 77.58 | 1409.6721 | 15 | 0.6 | 705.8438 | 2 | 11.67 | 1 | F1:2561 | DaRuMH\_F1.raw | 1.5553E5 |  |  |  |  |  |  |  |  |  | 1 | 1 | 0 | 0 | 0 | 0 | 0 | 0 | 0 | 0 | 0 | 49 | 63 | Carbamidomethylation | C7:Carbamidomethylation:1000.00 | PEAKS DB |
| K.LALDVEIATYR.K | N | 76.33 | 1262.6870 | 11 | 1.0 | 632.3514 | 2 | 19.40 | 10 | F10:8852 | DaRuMH\_F9.raw |  | 2.1656E5 |  |  | 5.9819E4 | 2.2865E5 | 6.9929E4 |  |  | 7.4922E5 | 5 | 0 | 1 | 0 | 0 | 1 | 1 | 1 | 0 | 0 | 1 | 461 | 471 |  |  | PEAKS DB |
| K.LAELEEALQK.A | Y | 70.43 | 1142.6183 | 10 | -0.1 | 572.3163 | 2 | 12.10 | 10 | F10:2892 | DaRuMH\_F9.raw | 3.2155E5 |  |  |  | 4.2082E4 |  |  |  |  | 1.5833E5 | 3 | 1 | 0 | 0 | 0 | 1 | 0 | 0 | 0 | 0 | 1 | 432 | 441 |  |  | PEAKS DB |
| K.YEELQQTAGR.H | N | 70.29 | 1193.5676 | 10 | 0.5 | 597.7914 | 2 | 11.10 | 1 | F1:1973 | DaRuMH\_F1.raw | 6.4797E4 |  |  |  | 4.9821E4 |  |  |  |  | 4.5584E4 | 3 | 1 | 0 | 0 | 0 | 1 | 0 | 0 | 0 | 0 | 1 | 365 | 374 |  |  | PEAKS DB |
| R.TEAESWYQTK.Y | N | 68.58 | 1241.5564 | 10 | 0.8 | 621.7859 | 2 | 11.51 | 1 | F1:2370 | DaRuMH\_F1.raw | 1.0499E5 |  |  |  |  |  |  |  |  |  | 1 | 1 | 0 | 0 | 0 | 0 | 0 | 0 | 0 | 0 | 0 | 355 | 364 |  |  | PEAKS DB |
| K.WTLLQEQGTK.T | N | 62.45 | 1202.6295 | 10 | -0.4 | 602.3218 | 2 | 12.37 | 1 | F1:3185 | DaRuMH\_F1.raw | 1.8633E5 |  |  |  |  |  |  |  |  | 2.9166E5 | 2 | 1 | 0 | 0 | 0 | 0 | 0 | 0 | 0 | 0 | 1 | 200 | 209 |  |  | PEAKS DB |
| K.AQYEEIANR.S | N | 59.21 | 1092.5199 | 9 | 0.8 | 547.2676 | 2 | 11.10 | 5 | F5:1935 | DaRuMH\_F4.raw |  |  |  |  | 5.3406E4 |  |  |  |  |  | 1 | 0 | 0 | 0 | 0 | 1 | 0 | 0 | 0 | 0 | 0 | 344 | 352 |  |  | PEAKS DB |
| R.NMQDLVEDFK.N | N | 57.91 | 1237.5648 | 10 | 0.4 | 619.7899 | 2 | 18.11 | 1 | F1:5617 | DaRuMH\_F1.raw | 9.935E4 |  |  |  |  |  |  |  |  |  | 1 | 1 | 0 | 0 | 0 | 0 | 0 | 0 | 0 | 0 | 0 | 246 | 255 |  |  | PEAKS DB |
| K.LLEGEEC(+57.02)R.L | N | 57.87 | 1004.4597 | 8 | 1.1 | 503.2376 | 2 | 10.99 | 2 | F2:1866 | DaRuMH\_F10.raw |  | 1.3429E4 |  |  |  |  |  |  |  |  | 1 | 0 | 1 | 0 | 0 | 0 | 0 | 0 | 0 | 0 | 0 | 473 | 480 | Carbamidomethylation | C7:Carbamidomethylation:1000.00 | PEAKS DB |
| K.NKYEDEINKR.T | N | 56.06 | 1307.6470 | 10 | -2.2 | 436.8886 | 3 | 10.74 | 1 | F1:1618 | DaRuMH\_F1.raw | 5.4056E6 |  |  |  |  |  |  |  |  |  | 1 | 1 | 0 | 0 | 0 | 0 | 0 | 0 | 0 | 0 | 0 | 256 | 265 |  |  | PEAKS DB |
| R.TTAENEFVMLK.K | N | 55.39 | 1281.6274 | 11 | 0.3 | 641.8212 | 2 | 13.30 | 1 | F1:3664 | DaRuMH\_F1.raw | 6.3078E4 |  |  |  |  |  |  |  |  |  | 1 | 1 | 0 | 0 | 0 | 0 | 0 | 0 | 0 | 0 | 0 | 266 | 276 |  |  | PEAKS DB |
| R.TTAENEFVM(+15.99)LK.K | N | 54.96 | 1297.6224 | 11 | -0.2 | 649.8184 | 2 | 11.95 | 1 | F1:2818 | DaRuMH\_F1.raw | 6.5214E4 |  |  |  |  |  |  |  |  |  | 1 | 1 | 0 | 0 | 0 | 0 | 0 | 0 | 0 | 0 | 0 | 266 | 276 | Oxidation (M) | M9:Oxidation (M):1000.00 | PEAKS DB |
| total 13 peptides |
| --- |

Q7LZ61|VM3CX\_DABSI

back to list

  

| Protein Coverage
| Supporting Peptides
|

Protein Coverage:

Supporting Peptides:

| Peptide | Uniq | -10lgP | Mass | Length | ppm | m/z | z | RT | Fraction | Scan | Source File | Area F1 | Area F10 | Area F2 | Area F3 | Area F4 | Area F5 | Area F7A | Area F7B | Area F8 | Area F9 | #Feature | #Feature F1 | #Feature F10 | #Feature F2 | #Feature F3 | #Feature F4 | #Feature F5 | #Feature F7A | #Feature F7B | #Feature F8 | #Feature F9 | Start | End | PTM | AScore | Found By |
| --- | --- | --- | --- | --- | --- | --- | --- | --- | --- | --- | --- | --- | --- | --- | --- | --- | --- | --- | --- | --- | --- | --- | --- | --- | --- | --- | --- | --- | --- | --- | --- | --- | --- | --- | --- | --- | --- |
| L.ESGNVNDYEVVYPQK.V | N | 99.26 | 1739.8002 | 15 | 0.4 | 870.9077 | 2 | 11.79 | 3 | F3:2512 | DaRuMH\_F2.raw | 1.5166E5 |  | 1.2778E6 |  |  |  |  |  |  |  | 2 | 1 | 0 | 1 | 0 | 0 | 0 | 0 | 0 | 0 | 0 | 24 | 38 |  |  | PEAKS DB |
| E.SGNVNDYEVVYPQK.V | N | 80.53 | 1610.7576 | 14 | 1.1 | 806.3870 | 2 | 11.73 | 3 | F3:2451 | DaRuMH\_F2.raw |  |  | 3.1229E5 |  |  |  |  |  |  |  | 1 | 0 | 0 | 1 | 0 | 0 | 0 | 0 | 0 | 0 | 0 | 25 | 38 |  |  | PEAKS DB |
| S.GNVNDYEVVYPQK.V | N | 78.53 | 1523.7256 | 13 | 0.7 | 762.8706 | 2 | 11.75 | 3 | F3:2472 | DaRuMH\_F2.raw |  |  | 1.2901E5 |  |  |  |  |  |  |  | 1 | 0 | 0 | 1 | 0 | 0 | 0 | 0 | 0 | 0 | 0 | 26 | 38 |  |  | PEAKS DB |
| K.DSC(+57.02)FQENLK.G | Y | 75.26 | 1139.4917 | 9 | -5.5 | 570.7500 | 2 | 11.48 | 10 | F10:2312 | DaRuMH\_F9.raw |  |  |  |  |  |  |  |  |  | 1.5374E5 | 1 | 0 | 0 | 0 | 0 | 0 | 0 | 0 | 0 | 0 | 1 | 523 | 531 | Carbamidomethylation | C3:Carbamidomethylation:1000.00 | PEAKS DB |
| R.NQC(+57.02)ISLFGSR.A | N | 63.26 | 1180.5659 | 10 | -0.3 | 591.2900 | 2 | 12.54 | 10 | F10:3256 | DaRuMH\_F9.raw |  |  |  |  |  |  |  | 1.4055E5 |  | 2.1574E6 | 2 | 0 | 0 | 0 | 0 | 0 | 0 | 0 | 1 | 0 | 1 | 508 | 517 | Carbamidomethylation | C3:Carbamidomethylation:1000.00 | PEAKS DB |
| R.LFC(+57.02)LNNSPR.N | Y | 60.10 | 1119.5494 | 9 | 1.7 | 560.7830 | 2 | 11.90 | 10 | F10:2722 | DaRuMH\_F9.raw |  |  |  |  |  |  |  |  |  | 2.0638E5 | 1 | 0 | 0 | 0 | 0 | 0 | 0 | 0 | 0 | 0 | 1 | 558 | 566 | Carbamidomethylation | C3:Carbamidomethylation:1000.00 | PEAKS DB |
| K.IPC(+57.02)APQDVK.C | N | 59.57 | 1026.5168 | 9 | 0.3 | 514.2659 | 2 | 11.18 | 10 | F10:2024 | DaRuMH\_F9.raw |  |  |  |  |  |  |  |  |  | 0 | 0 | 0 | 0 | 0 | 0 | 0 | 0 | 0 | 0 | 0 | 0 | 546 | 554 | Carbamidomethylation | C3:Carbamidomethylation:1000.00 | PEAKS DB |
| N.QC(+57.02)ISLFGSR.A | N | 59.17 | 1066.5229 | 9 | -0.1 | 534.2687 | 2 | 12.75 | 5 | F5:3379 | DaRuMH\_F4.raw |  |  |  |  | 2.597E5 |  |  |  |  |  | 1 | 0 | 0 | 0 | 0 | 1 | 0 | 0 | 0 | 0 | 0 | 509 | 517 | Carbamidomethylation | C2:Carbamidomethylation:1000.00 | PEAKS DB |
| total 8 peptides |
| --- |

A8Y7N6|VKTC3\_DABSI

back to list

  

| Protein Coverage
| Supporting Peptides
|

Protein Coverage:

Supporting Peptides:

| Peptide | Uniq | -10lgP | Mass | Length | ppm | m/z | z | RT | Fraction | Scan | Source File | Area F1 | Area F10 | Area F2 | Area F3 | Area F4 | Area F5 | Area F7A | Area F7B | Area F8 | Area F9 | #Feature | #Feature F1 | #Feature F10 | #Feature F2 | #Feature F3 | #Feature F4 | #Feature F5 | #Feature F7A | #Feature F7B | #Feature F8 | #Feature F9 | Start | End | PTM | AScore | Found By |
| --- | --- | --- | --- | --- | --- | --- | --- | --- | --- | --- | --- | --- | --- | --- | --- | --- | --- | --- | --- | --- | --- | --- | --- | --- | --- | --- | --- | --- | --- | --- | --- | --- | --- | --- | --- | --- | --- |
| K.EFIYGGC(+57.02)HGNANNFPTR.D | Y | 91.58 | 1952.8588 | 17 | 0.1 | 977.4368 | 2 | 11.62 | 3 | F3:2334 | DaRuMH\_F2.raw |  |  | 3.7347E6 | 1.0916E7 | 1.5243E5 |  |  |  |  |  | 4 | 0 | 0 | 2 | 1 | 1 | 0 | 0 | 0 | 0 | 0 | 58 | 74 | Carbamidomethylation | C7:Carbamidomethylation:1000.00 | PEAKS DB |
| F.IYGGC(+57.02)HGNANNFPTR.D | Y | 77.02 | 1676.7478 | 15 | 5.7 | 559.9232 | 3 | 11.03 | 4 | F4:1891 | DaRuMH\_F3.raw |  |  |  | 1.5705E6 |  |  |  |  |  |  | 1 | 0 | 0 | 0 | 1 | 0 | 0 | 0 | 0 | 0 | 0 | 60 | 74 | Carbamidomethylation | C5:Carbamidomethylation:1000.00 | PEAKS DB |
| K.C(+57.02)KEFIYGGC(+57.02)HGNANNFPTR.D | Y | 67.93 | 2240.9844 | 19 | 5.5 | 561.2532 | 4 | 11.34 | 4 | F4:2184 | DaRuMH\_F3.raw |  |  |  | 3.138E6 |  |  |  |  |  |  | 2 | 0 | 0 | 0 | 2 | 0 | 0 | 0 | 0 | 0 | 0 | 56 | 74 | Carbamidomethylation | C1:Carbamidomethylation:1000.00;C9:Carbamidomethylation:1000.00 | PEAKS DB |
| R.SFYYDSESK.K | N | 67.86 | 1124.4662 | 9 | 1.1 | 563.2410 | 2 | 11.46 | 3 | F3:2160 | DaRuMH\_F2.raw |  |  | 2.8891E6 | 1.2846E8 |  |  |  |  |  |  | 2 | 0 | 0 | 1 | 1 | 0 | 0 | 0 | 0 | 0 | 0 | 46 | 54 |  |  | PEAKS DB |
| K.EFIYGGC(+57.02)HGNAN.N | N | 64.05 | 1337.5459 | 12 | 6.3 | 669.7806 | 2 | 11.46 | 4 | F4:2300 | DaRuMH\_F3.raw |  |  |  | 1.3232E7 |  |  |  |  |  |  | 1 | 0 | 0 | 0 | 1 | 0 | 0 | 0 | 0 | 0 | 0 | 58 | 69 | Carbamidomethylation | C7:Carbamidomethylation:1000.00 | PEAKS DB |
| R.SFYYDSESKK.C | N | 61.36 | 1252.5612 | 10 | 7.0 | 418.5282 | 3 | 10.99 | 4 | F4:1828 | DaRuMH\_F3.raw |  |  | 4.7424E4 | 2.6207E6 |  |  |  |  |  |  | 2 | 0 | 0 | 1 | 1 | 0 | 0 | 0 | 0 | 0 | 0 | 46 | 55 |  |  | PEAKS DB |
| D.PGEC(+57.02)M(+15.99)AYIR.S | Y | 60.62 | 1111.4790 | 9 | 5.4 | 556.7466 | 2 | 11.14 | 4 | F4:2001 | DaRuMH\_F3.raw |  |  |  | 3.0781E5 |  |  |  |  |  |  | 1 | 0 | 0 | 0 | 1 | 0 | 0 | 0 | 0 | 0 | 0 | 37 | 45 | Carbamidomethylation; Oxidation (M) | C4:Carbamidomethylation:1000.00;M5:Oxidation (M):1000.00 | PEAKS DB |
| K.FC(+57.02)YLPADPGEC(+57.02)M(+15.99)AYIR.S | Y | 60.41 | 1977.8423 | 16 | 6.0 | 989.9287 | 2 | 17.84 | 4 | F4:5763 | DaRuMH\_F3.raw |  |  |  | 1.491E7 |  |  |  |  |  |  | 1 | 0 | 0 | 0 | 1 | 0 | 0 | 0 | 0 | 0 | 0 | 30 | 45 | Carbamidomethylation; Oxidation (M) | C2:Carbamidomethylation:1000.00;C11:Carbamidomethylation:1000.00;M12:Oxidation (M):1000.00 | PEAKS DB |
| C.YLPADPGEC(+57.02)M(+15.99)AYIR.S | Y | 53.81 | 1670.7432 | 14 | 6.5 | 836.3795 | 2 | 11.99 | 4 | F4:2808 | DaRuMH\_F3.raw |  |  |  | 2.5535E5 |  |  |  |  |  |  | 1 | 0 | 0 | 0 | 1 | 0 | 0 | 0 | 0 | 0 | 0 | 32 | 45 | Carbamidomethylation; Oxidation (M) | C9:Carbamidomethylation:1000.00;M10:Oxidation (M):1000.00 | PEAKS DB |
| total 9 peptides |
| --- |

A8Y7N7|VKTC4\_DABSI

back to list

  

| Protein Coverage
| Supporting Peptides
|

Protein Coverage:

Supporting Peptides:

| Peptide | Uniq | -10lgP | Mass | Length | ppm | m/z | z | RT | Fraction | Scan | Source File | Area F1 | Area F10 | Area F2 | Area F3 | Area F4 | Area F5 | Area F7A | Area F7B | Area F8 | Area F9 | #Feature | #Feature F1 | #Feature F10 | #Feature F2 | #Feature F3 | #Feature F4 | #Feature F5 | #Feature F7A | #Feature F7B | #Feature F8 | #Feature F9 | Start | End | PTM | AScore | Found By |
| --- | --- | --- | --- | --- | --- | --- | --- | --- | --- | --- | --- | --- | --- | --- | --- | --- | --- | --- | --- | --- | --- | --- | --- | --- | --- | --- | --- | --- | --- | --- | --- | --- | --- | --- | --- | --- | --- |
| K.EFIYGGC(+57.02)HGNANNFPTR.D | Y | 91.58 | 1952.8588 | 17 | 0.1 | 977.4368 | 2 | 11.62 | 3 | F3:2334 | DaRuMH\_F2.raw |  |  | 3.7347E6 | 1.0916E7 | 1.5243E5 |  |  |  |  |  | 4 | 0 | 0 | 2 | 1 | 1 | 0 | 0 | 0 | 0 | 0 | 58 | 74 | Carbamidomethylation | C7:Carbamidomethylation:1000.00 | PEAKS DB |
| F.IYGGC(+57.02)HGNANNFPTR.D | Y | 77.02 | 1676.7478 | 15 | 5.7 | 559.9232 | 3 | 11.03 | 4 | F4:1891 | DaRuMH\_F3.raw |  |  |  | 1.5705E6 |  |  |  |  |  |  | 1 | 0 | 0 | 0 | 1 | 0 | 0 | 0 | 0 | 0 | 0 | 60 | 74 | Carbamidomethylation | C5:Carbamidomethylation:1000.00 | PEAKS DB |
| K.C(+57.02)KEFIYGGC(+57.02)HGNANNFPTR.D | Y | 67.93 | 2240.9844 | 19 | 5.5 | 561.2532 | 4 | 11.34 | 4 | F4:2184 | DaRuMH\_F3.raw |  |  |  | 3.138E6 |  |  |  |  |  |  | 2 | 0 | 0 | 0 | 2 | 0 | 0 | 0 | 0 | 0 | 0 | 56 | 74 | Carbamidomethylation | C1:Carbamidomethylation:1000.00;C9:Carbamidomethylation:1000.00 | PEAKS DB |
| R.SFYYDSESK.K | N | 67.86 | 1124.4662 | 9 | 1.1 | 563.2410 | 2 | 11.46 | 3 | F3:2160 | DaRuMH\_F2.raw |  |  | 2.8891E6 | 1.2846E8 |  |  |  |  |  |  | 2 | 0 | 0 | 1 | 1 | 0 | 0 | 0 | 0 | 0 | 0 | 46 | 54 |  |  | PEAKS DB |
| K.EFIYGGC(+57.02)HGNAN.N | N | 64.05 | 1337.5459 | 12 | 6.3 | 669.7806 | 2 | 11.46 | 4 | F4:2300 | DaRuMH\_F3.raw |  |  |  | 1.3232E7 |  |  |  |  |  |  | 1 | 0 | 0 | 0 | 1 | 0 | 0 | 0 | 0 | 0 | 0 | 58 | 69 | Carbamidomethylation | C7:Carbamidomethylation:1000.00 | PEAKS DB |
| R.SFYYDSESKK.C | N | 61.36 | 1252.5612 | 10 | 7.0 | 418.5282 | 3 | 10.99 | 4 | F4:1828 | DaRuMH\_F3.raw |  |  | 4.7424E4 | 2.6207E6 |  |  |  |  |  |  | 2 | 0 | 0 | 1 | 1 | 0 | 0 | 0 | 0 | 0 | 0 | 46 | 55 |  |  | PEAKS DB |
| D.PGEC(+57.02)M(+15.99)AYIR.S | Y | 60.62 | 1111.4790 | 9 | 5.4 | 556.7466 | 2 | 11.14 | 4 | F4:2001 | DaRuMH\_F3.raw |  |  |  | 3.0781E5 |  |  |  |  |  |  | 1 | 0 | 0 | 0 | 1 | 0 | 0 | 0 | 0 | 0 | 0 | 37 | 45 | Carbamidomethylation; Oxidation (M) | C4:Carbamidomethylation:1000.00;M5:Oxidation (M):1000.00 | PEAKS DB |
| K.FC(+57.02)YLPADPGEC(+57.02)M(+15.99)AYIR.S | Y | 60.41 | 1977.8423 | 16 | 6.0 | 989.9287 | 2 | 17.84 | 4 | F4:5763 | DaRuMH\_F3.raw |  |  |  | 1.491E7 |  |  |  |  |  |  | 1 | 0 | 0 | 0 | 1 | 0 | 0 | 0 | 0 | 0 | 0 | 30 | 45 | Carbamidomethylation; Oxidation (M) | C2:Carbamidomethylation:1000.00;C11:Carbamidomethylation:1000.00;M12:Oxidation (M):1000.00 | PEAKS DB |
| C.YLPADPGEC(+57.02)M(+15.99)AYIR.S | Y | 53.81 | 1670.7432 | 14 | 6.5 | 836.3795 | 2 | 11.99 | 4 | F4:2808 | DaRuMH\_F3.raw |  |  |  | 2.5535E5 |  |  |  |  |  |  | 1 | 0 | 0 | 0 | 1 | 0 | 0 | 0 | 0 | 0 | 0 | 32 | 45 | Carbamidomethylation; Oxidation (M) | C9:Carbamidomethylation:1000.00;M10:Oxidation (M):1000.00 | PEAKS DB |
| total 9 peptides |
| --- |

Q7ZZN9|CRVP\_PROJR

back to list

  

| Protein Coverage
| Supporting Peptides
|

Protein Coverage:

Supporting Peptides:

| Peptide | Uniq | -10lgP | Mass | Length | ppm | m/z | z | RT | Fraction | Scan | Source File | Area F1 | Area F10 | Area F2 | Area F3 | Area F4 | Area F5 | Area F7A | Area F7B | Area F8 | Area F9 | #Feature | #Feature F1 | #Feature F10 | #Feature F2 | #Feature F3 | #Feature F4 | #Feature F5 | #Feature F7A | #Feature F7B | #Feature F8 | #Feature F9 | Start | End | PTM | AScore | Found By |
| --- | --- | --- | --- | --- | --- | --- | --- | --- | --- | --- | --- | --- | --- | --- | --- | --- | --- | --- | --- | --- | --- | --- | --- | --- | --- | --- | --- | --- | --- | --- | --- | --- | --- | --- | --- | --- | --- |
| K.M(+15.99)EWYPEAAANAER.W | N | 94.55 | 1552.6616 | 13 | -3.9 | 777.3351 | 2 | 11.86 | 8 | F8:2751 | DaRuMH\_F7B.raw | 5.2819E5 |  |  |  |  |  |  | 6.0748E6 | 2.2981E5 |  | 3 | 1 | 0 | 0 | 0 | 0 | 0 | 0 | 1 | 1 | 0 | 58 | 70 | Oxidation (M) | M1:Oxidation (M):1000.00 | PEAKS DB |
| K.MEWYPEAAANAER.W | N | 93.30 | 1536.6667 | 13 | 2.1 | 769.3423 | 2 | 12.88 | 8 | F8:3614 | DaRuMH\_F7B.raw |  |  |  |  |  |  |  | 1.0703E7 | 2.998E5 |  | 2 | 0 | 0 | 0 | 0 | 0 | 0 | 0 | 1 | 1 | 0 | 58 | 70 |  |  | PEAKS DB |
| K.C(+57.02)GENIYMSPYPMK.W | N | 87.92 | 1588.6724 | 13 | 0.2 | 795.3436 | 2 | 13.13 | 8 | F8:3881 | DaRuMH\_F7B.raw | 1.4844E5 |  |  |  |  |  |  | 6.5312E5 |  |  | 2 | 1 | 0 | 0 | 0 | 0 | 0 | 0 | 1 | 0 | 0 | 92 | 104 | Carbamidomethylation | C1:Carbamidomethylation:1000.00 | PEAKS DB |
| D.AVVGHYTQIVWYK.S | N | 80.53 | 1562.8245 | 13 | -1.5 | 521.9480 | 3 | 12.13 | 8 | F8:3006 | DaRuMH\_F7B.raw |  |  |  |  |  |  |  | 1.9751E6 | 4.4197E4 |  | 2 | 0 | 0 | 0 | 0 | 0 | 0 | 0 | 1 | 1 | 0 | 130 | 142 |  |  | PEAKS DB |
| H.YTQIVWYK.S | N | 77.79 | 1099.5702 | 8 | 0.9 | 550.7928 | 2 | 12.97 | 8 | F8:3679 | DaRuMH\_F7B.raw | 2.3904E6 |  |  |  |  |  |  | 1.823E7 |  | 5.9692E4 | 3 | 1 | 0 | 0 | 0 | 0 | 0 | 0 | 1 | 0 | 1 | 135 | 142 |  |  | PEAKS DB |
| K.C(+57.02)GENIYMSPYPM(+15.99)K.W | N | 76.47 | 1604.6674 | 13 | 0.0 | 803.3409 | 2 | 11.88 | 1 | F1:2761 | DaRuMH\_F1.raw | 1.0285E5 |  |  |  |  |  |  | 2.5226E5 |  |  | 2 | 1 | 0 | 0 | 0 | 0 | 0 | 0 | 1 | 0 | 0 | 92 | 104 | Carbamidomethylation; Oxidation (M) | C1:Carbamidomethylation:1000.00;M12:Oxidation (M):42.97 | PEAKS DB |
| M.EWYPEAAANAER.W | N | 68.66 | 1405.6262 | 12 | 0.1 | 703.8204 | 2 | 11.82 | 8 | F8:2734 | DaRuMH\_F7B.raw | 5.4508E4 |  |  |  |  |  |  | 4.7194E5 |  |  | 2 | 1 | 0 | 0 | 0 | 0 | 0 | 0 | 1 | 0 | 0 | 59 | 70 |  |  | PEAKS DB |
| N.C(+57.02)PASC(+57.02)FC(+57.02)HNEII | Y | 58.63 | 1506.6053 | 12 | -1.6 | 754.3088 | 2 | 12.13 | 8 | F8:2976 | DaRuMH\_F7B.raw |  |  | 6.9905E5 |  |  |  |  | 6.2758E7 | 3.1943E6 |  | 3 | 0 | 0 | 1 | 0 | 0 | 0 | 0 | 1 | 1 | 0 | 229 | 240 | Carbamidomethylation | C1:Carbamidomethylation:1000.00;C5:Carbamidomethylation:1000.00;C7:Carbamidomethylation:1000.00 | PEAKS DB |
| N.VDFDSESPR.K | N | 56.99 | 1050.4618 | 9 | -0.7 | 526.2378 | 2 | 11.30 | 8 | F8:2216 | DaRuMH\_F7B.raw |  |  |  |  |  |  |  | 1.059E5 |  |  | 1 | 0 | 0 | 0 | 0 | 0 | 0 | 0 | 1 | 0 | 0 | 21 | 29 |  |  | PEAKS DB |
| total 9 peptides |
| --- |

O43790|KRT86\_HUMAN

back to list

  

| Protein Coverage
| Supporting Peptides
|

Protein Coverage:

Supporting Peptides:

| Peptide | Uniq | -10lgP | Mass | Length | ppm | m/z | z | RT | Fraction | Scan | Source File | Area F1 | Area F10 | Area F2 | Area F3 | Area F4 | Area F5 | Area F7A | Area F7B | Area F8 | Area F9 | #Feature | #Feature F1 | #Feature F10 | #Feature F2 | #Feature F3 | #Feature F4 | #Feature F5 | #Feature F7A | #Feature F7B | #Feature F8 | #Feature F9 | Start | End | PTM | AScore | Found By |
| --- | --- | --- | --- | --- | --- | --- | --- | --- | --- | --- | --- | --- | --- | --- | --- | --- | --- | --- | --- | --- | --- | --- | --- | --- | --- | --- | --- | --- | --- | --- | --- | --- | --- | --- | --- | --- | --- |
| K.LEAAVAQSEQQGEAALSDAR.C | Y | 109.49 | 2042.9868 | 20 | 1.2 | 1022.5020 | 2 | 11.87 | 2 | F2:2688 | DaRuMH\_F10.raw |  | 2.252E5 |  |  |  |  |  |  |  |  | 1 | 0 | 1 | 0 | 0 | 0 | 0 | 0 | 0 | 0 | 0 | 346 | 365 |  |  | PEAKS DB |
| R.GGVVC(+57.02)GDLC(+57.02)ASTTAPVVSTR.V | Y | 74.76 | 2005.9561 | 20 | 0.4 | 1003.9857 | 2 | 12.07 | 2 | F2:2853 | DaRuMH\_F10.raw |  | 5.9222E4 |  |  |  |  |  |  |  |  | 1 | 0 | 1 | 0 | 0 | 0 | 0 | 0 | 0 | 0 | 0 | 433 | 452 | Carbamidomethylation | C5:Carbamidomethylation:1000.00;C9:Carbamidomethylation:1000.00 | PEAKS DB |
| R.LASELNHVQEVLEGYK.K | N | 66.68 | 1827.9366 | 16 | -0.1 | 610.3195 | 3 | 16.09 | 2 | F2:4582 | DaRuMH\_F10.raw |  | 1.1736E5 |  |  |  |  |  |  |  |  | 1 | 0 | 1 | 0 | 0 | 0 | 0 | 0 | 0 | 0 | 0 | 176 | 191 |  |  | PEAKS DB |
| K.LGLDIEIATYR.R | N | 59.27 | 1262.6870 | 11 | 0.3 | 632.3510 | 2 | 23.70 | 2 | F2:6972 | DaRuMH\_F10.raw |  | 3.4553E5 |  |  |  |  |  |  |  |  | 1 | 0 | 1 | 0 | 0 | 0 | 0 | 0 | 0 | 0 | 0 | 397 | 407 |  |  | PEAKS DB |
| K.LAELEGALQK.A | N | 55.09 | 1070.5972 | 10 | 0.8 | 536.3063 | 2 | 11.78 | 2 | F2:2604 | DaRuMH\_F10.raw |  | 8.7297E4 |  |  |  |  |  |  |  |  | 1 | 0 | 1 | 0 | 0 | 0 | 0 | 0 | 0 | 0 | 0 | 368 | 377 |  |  | PEAKS DB |
| R.FAAFIDK.V | N | 53.55 | 810.4276 | 7 | 0.6 | 406.2213 | 2 | 12.03 | 2 | F2:2816 | DaRuMH\_F10.raw |  | 4.7783E4 |  |  |  |  |  |  |  |  | 1 | 0 | 1 | 0 | 0 | 0 | 0 | 0 | 0 | 0 | 0 | 117 | 123 |  |  | PEAKS DB |
| total 6 peptides |
| --- |

E0Y419|VSPBF\_MACLB

back to list

  

| Protein Coverage
| Supporting Peptides
|

Protein Coverage:

Supporting Peptides:

| Peptide | Uniq | -10lgP | Mass | Length | ppm | m/z | z | RT | Fraction | Scan | Source File | Area F1 | Area F10 | Area F2 | Area F3 | Area F4 | Area F5 | Area F7A | Area F7B | Area F8 | Area F9 | #Feature | #Feature F1 | #Feature F10 | #Feature F2 | #Feature F3 | #Feature F4 | #Feature F5 | #Feature F7A | #Feature F7B | #Feature F8 | #Feature F9 | Start | End | PTM | AScore | Found By |
| --- | --- | --- | --- | --- | --- | --- | --- | --- | --- | --- | --- | --- | --- | --- | --- | --- | --- | --- | --- | --- | --- | --- | --- | --- | --- | --- | --- | --- | --- | --- | --- | --- | --- | --- | --- | --- | --- |
| L.VVGGDEC(+57.02)NINEHR.S | N | 96.07 | 1497.6630 | 13 | -1.0 | 749.8380 | 2 | 10.70 | 9 | F9:1591 | DaRuMH\_F8.raw | 1.9072E7 |  |  |  |  |  | 3.6253E5 | 1.0498E7 | 2.9349E7 | 8.3285E5 | 8 | 2 | 0 | 0 | 0 | 0 | 0 | 1 | 2 | 2 | 1 | 25 | 37 | Carbamidomethylation | C7:Carbamidomethylation:1000.00 | PEAKS DB |
| R.TLC(+57.02)AGILQGGIDTC(+57.02)K.G | Y | 86.59 | 1605.7854 | 15 | 1.1 | 803.9009 | 2 | 13.79 | 8 | F8:4392 | DaRuMH\_F7B.raw | 8.1646E5 |  |  |  |  | 3.7015E5 | 6.0638E6 | 6.8512E6 | 3.576E6 | 2.7563E5 | 8 | 1 | 0 | 0 | 0 | 0 | 1 | 1 | 2 | 2 | 1 | 186 | 200 | Carbamidomethylation | C3:Carbamidomethylation:1000.00;C14:Carbamidomethylation:1000.00 | PEAKS DB |
| L.SLPSSPPSVGSVC(+57.02)R.I | N | 81.50 | 1428.7031 | 14 | 0.2 | 715.3590 | 2 | 11.65 | 8 | F8:2499 | DaRuMH\_F7B.raw |  |  |  |  |  |  | 2.3471E6 | 1.3457E7 | 5.5106E6 |  | 3 | 0 | 0 | 0 | 0 | 0 | 0 | 1 | 1 | 1 | 0 | 129 | 142 | Carbamidomethylation | C13:Carbamidomethylation:1000.00 | PEAKS DB |
| R.TLC(+57.02)AGILQGGIDTC(+57.02)KG.D | Y | 68.23 | 1662.8069 | 16 | 0.4 | 832.4111 | 2 | 13.59 | 9 | F9:3988 | DaRuMH\_F8.raw |  |  |  |  |  |  |  |  | 7.3083E4 |  | 1 | 0 | 0 | 0 | 0 | 0 | 0 | 0 | 0 | 1 | 0 | 186 | 201 | Carbamidomethylation | C3:Carbamidomethylation:1000.00;C14:Carbamidomethylation:1000.00 | PEAKS DB |
| K.FFC(+57.02)LSSK.N | N | 67.85 | 887.4211 | 7 | -0.6 | 444.7176 | 2 | 12.08 | 8 | F8:2955 | DaRuMH\_F7B.raw | 2.546E6 |  |  |  |  |  | 6.2481E5 | 9.2089E6 | 3.0567E6 | 5.9046E5 | 5 | 1 | 0 | 0 | 0 | 0 | 0 | 1 | 1 | 1 | 1 | 95 | 101 | Carbamidomethylation | C3:Carbamidomethylation:1000.00 | PEAKS DB |
| V.GGDEC(+57.02)NINEHR.S | N | 67.81 | 1299.5262 | 11 | 0.2 | 650.7705 | 2 | 10.70 | 9 | F9:1597 | DaRuMH\_F8.raw |  |  |  |  |  |  |  | 2.6409E5 | 7.79E5 |  | 2 | 0 | 0 | 0 | 0 | 0 | 0 | 0 | 1 | 1 | 0 | 27 | 37 | Carbamidomethylation | C5:Carbamidomethylation:1000.00 | PEAKS DB |
| E.WDKDIMLIK.M | N | 53.69 | 1160.6263 | 9 | 0.0 | 581.3204 | 2 | 12.61 | 8 | F8:3439 | DaRuMH\_F7B.raw |  |  |  |  |  |  |  | 3.5006E4 |  |  | 1 | 0 | 0 | 0 | 0 | 0 | 0 | 0 | 1 | 0 | 0 | 106 | 114 |  |  | PEAKS DB |
| total 7 peptides |
| --- |

G8XQX1|OXLA\_DABRR

back to list

  

| Protein Coverage
| Supporting Peptides
|

Protein Coverage:

Supporting Peptides:

| Peptide | Uniq | -10lgP | Mass | Length | ppm | m/z | z | RT | Fraction | Scan | Source File | Area F1 | Area F10 | Area F2 | Area F3 | Area F4 | Area F5 | Area F7A | Area F7B | Area F8 | Area F9 | #Feature | #Feature F1 | #Feature F10 | #Feature F2 | #Feature F3 | #Feature F4 | #Feature F5 | #Feature F7A | #Feature F7B | #Feature F8 | #Feature F9 | Start | End | PTM | AScore | Found By |
| --- | --- | --- | --- | --- | --- | --- | --- | --- | --- | --- | --- | --- | --- | --- | --- | --- | --- | --- | --- | --- | --- | --- | --- | --- | --- | --- | --- | --- | --- | --- | --- | --- | --- | --- | --- | --- | --- |
| R.EDDYEEFLEIAK.N | Y | 79.00 | 1499.6667 | 12 | 0.4 | 750.8409 | 2 | 28.33 | 10 | F10:14250 | DaRuMH\_F9.raw |  | 6.1666E5 |  |  |  |  |  |  | 2.2898E5 | 4.4799E5 | 3 | 0 | 1 | 0 | 0 | 0 | 0 | 0 | 0 | 1 | 1 | 31 | 42 |  |  | PEAKS DB |
| C.ADDKNPLEEC(+57.02)FR.E | Y | 76.71 | 1492.6616 | 12 | -0.7 | 498.5608 | 3 | 11.69 | 2 | F2:2537 | DaRuMH\_F10.raw |  | 8.1048E4 |  |  |  |  |  |  |  |  | 1 | 0 | 1 | 0 | 0 | 0 | 0 | 0 | 0 | 0 | 0 | 19 | 30 | Carbamidomethylation | C10:Carbamidomethylation:1000.00 | PEAKS DB |
| K.FWEDDGIQGGK.S | Y | 67.49 | 1250.5568 | 11 | 1.0 | 626.2863 | 2 | 11.87 | 2 | F2:2694 | DaRuMH\_F10.raw |  | 1.6983E5 |  |  |  |  |  |  |  |  | 1 | 0 | 1 | 0 | 0 | 0 | 0 | 0 | 0 | 0 | 0 | 353 | 363 |  |  | PEAKS DB |
| K.HDDIFAYEK.R | Y | 58.79 | 1136.5138 | 9 | 1.3 | 569.2649 | 2 | 11.51 | 2 | F2:2372 | DaRuMH\_F10.raw |  | 1.3796E5 |  |  |  |  |  |  |  |  | 1 | 0 | 1 | 0 | 0 | 0 | 0 | 0 | 0 | 0 | 0 | 241 | 249 |  |  | PEAKS DB |
| K.NLLLETADYVIVC(+57.02)TTSR.A | Y | 57.48 | 1967.0034 | 17 | 0.8 | 984.5098 | 2 | 47.12 | 2 | F2:12593 | DaRuMH\_F10.raw |  | 9.0641E5 |  |  |  |  |  |  | 5.1175E4 |  | 3 | 0 | 2 | 0 | 0 | 0 | 0 | 0 | 0 | 1 | 0 | 299 | 315 | Carbamidomethylation | C13:Carbamidomethylation:1000.00 | PEAKS DB |
| Y.QFQHFSEALTAPVGR.I | Y | 56.73 | 1686.8478 | 15 | -0.2 | 563.2897 | 3 | 11.85 | 2 | F2:2671 | DaRuMH\_F10.raw |  | 1.2926E5 |  |  |  |  |  |  |  |  | 1 | 0 | 1 | 0 | 0 | 0 | 0 | 0 | 0 | 0 | 0 | 455 | 469 |  |  | PEAKS DB |
| R.ITFKPPLPPK.K | Y | 56.17 | 1136.6957 | 10 | -1.2 | 379.9054 | 3 | 11.76 | 2 | F2:2594 | DaRuMH\_F10.raw |  | 4.7179E4 |  |  |  |  |  |  |  |  | 1 | 0 | 1 | 0 | 0 | 0 | 0 | 0 | 0 | 0 | 0 | 320 | 329 |  |  | PEAKS DB |
| F.TPYQFQHFSEALTAPVGR.I | Y | 55.72 | 2048.0115 | 18 | 0.0 | 683.6777 | 3 | 15.12 | 2 | F2:4316 | DaRuMH\_F10.raw |  | 5.7205E4 |  |  |  |  |  |  |  |  | 1 | 0 | 1 | 0 | 0 | 0 | 0 | 0 | 0 | 0 | 0 | 452 | 469 |  |  | PEAKS DB |
| F.C(+57.02)YPSIIQK.W | Y | 53.78 | 1007.5110 | 8 | -0.6 | 504.7625 | 2 | 11.58 | 2 | F2:2430 | DaRuMH\_F10.raw |  | 6.048E4 |  |  |  |  |  |  |  |  | 1 | 0 | 1 | 0 | 0 | 0 | 0 | 0 | 0 | 0 | 0 | 430 | 437 | Carbamidomethylation | C1:Carbamidomethylation:1000.00 | PEAKS DB |
| total 9 peptides |
| --- |

Q2ES47|VKT4\_DABRR

back to list

  

| Protein Coverage
| Supporting Peptides
|

Protein Coverage:

Supporting Peptides:

| Peptide | Uniq | -10lgP | Mass | Length | ppm | m/z | z | RT | Fraction | Scan | Source File | Area F1 | Area F10 | Area F2 | Area F3 | Area F4 | Area F5 | Area F7A | Area F7B | Area F8 | Area F9 | #Feature | #Feature F1 | #Feature F10 | #Feature F2 | #Feature F3 | #Feature F4 | #Feature F5 | #Feature F7A | #Feature F7B | #Feature F8 | #Feature F9 | Start | End | PTM | AScore | Found By |
| --- | --- | --- | --- | --- | --- | --- | --- | --- | --- | --- | --- | --- | --- | --- | --- | --- | --- | --- | --- | --- | --- | --- | --- | --- | --- | --- | --- | --- | --- | --- | --- | --- | --- | --- | --- | --- | --- |
| F.IYGGC(+57.02)GGNANNFETR.D | N | 88.83 | 1628.7001 | 15 | 1.5 | 815.3585 | 2 | 11.35 | 3 | F3:2069 | DaRuMH\_F2.raw |  |  | 7.886E5 | 1.9848E7 |  |  |  |  |  |  | 2 | 0 | 0 | 1 | 1 | 0 | 0 | 0 | 0 | 0 | 0 | 60 | 74 | Carbamidomethylation | C5:Carbamidomethylation:1000.00 | PEAKS DB |
| A.SNQC(+57.02)QGFIYGGC(+57.02)GGNANNFETR.D | Y | 76.52 | 2450.0127 | 22 | 6.3 | 1226.0143 | 2 | 11.97 | 4 | F4:2786 | DaRuMH\_F3.raw |  |  |  | 1.4216E6 |  |  |  |  |  |  | 2 | 0 | 0 | 0 | 2 | 0 | 0 | 0 | 0 | 0 | 0 | 53 | 74 | Carbamidomethylation | C4:Carbamidomethylation:1000.00;C12:Carbamidomethylation:1000.00 | PEAKS DB |
| N.QC(+57.02)QGFIYGGC(+57.02)GGNANNFETR.D | Y | 74.16 | 2248.9377 | 20 | 6.1 | 1125.4766 | 2 | 12.02 | 4 | F4:2823 | DaRuMH\_F3.raw |  |  |  | 6.7606E5 |  |  |  |  |  |  | 2 | 0 | 0 | 0 | 2 | 0 | 0 | 0 | 0 | 0 | 0 | 55 | 74 | Carbamidomethylation | C2:Carbamidomethylation:1000.00;C10:Carbamidomethylation:1000.00 | PEAKS DB |
| K.FC(+57.02)HLPVDSGIC(+57.02)R.A | Y | 72.63 | 1459.6700 | 12 | -0.5 | 487.5637 | 3 | 11.62 | 3 | F3:2350 | DaRuMH\_F2.raw |  |  | 7.8278E4 | 9.083E4 |  |  |  |  |  |  | 2 | 0 | 0 | 1 | 1 | 0 | 0 | 0 | 0 | 0 | 0 | 30 | 41 | Carbamidomethylation | C2:Carbamidomethylation:1000.00;C11:Carbamidomethylation:1000.00 | PEAKS DB |
| C.QGFIYGGC(+57.02)GGNANNFETR.D | Y | 69.64 | 1960.8486 | 18 | 6.6 | 981.4325 | 2 | 12.04 | 4 | F4:2847 | DaRuMH\_F3.raw |  |  |  | 4.6474E5 |  |  |  |  |  |  | 1 | 0 | 0 | 0 | 1 | 0 | 0 | 0 | 0 | 0 | 0 | 57 | 74 | Carbamidomethylation | C8:Carbamidomethylation:1000.00 | PEAKS DB |
| R.FYYNPASNQC(+57.02)QGF.I | N | 62.91 | 1594.6510 | 13 | 6.3 | 798.3333 | 2 | 19.03 | 4 | F4:6084 | DaRuMH\_F3.raw |  |  |  | 1.2889E7 |  |  |  |  |  |  | 1 | 0 | 0 | 0 | 1 | 0 | 0 | 0 | 0 | 0 | 0 | 47 | 59 | Carbamidomethylation | C10:Carbamidomethylation:1000.00 | PEAKS DB |
| K.FC(+57.02)HLPVDSGIC(+57.02).R | Y | 58.39 | 1303.5690 | 11 | 6.1 | 652.7920 | 2 | 12.40 | 4 | F4:3152 | DaRuMH\_F3.raw |  |  |  | 9.8849E7 |  |  |  |  |  |  | 1 | 0 | 0 | 0 | 1 | 0 | 0 | 0 | 0 | 0 | 0 | 30 | 40 | Carbamidomethylation | C2:Carbamidomethylation:1000.00;C11:Carbamidomethylation:1000.00 | PEAKS DB |
| R.FYYNPASNQC(+57.02)Q.G | N | 54.05 | 1390.5612 | 11 | 6.7 | 696.2885 | 2 | 12.20 | 4 | F4:3017 | DaRuMH\_F3.raw |  |  |  | 1.2E5 |  |  |  |  |  |  | 1 | 0 | 0 | 0 | 1 | 0 | 0 | 0 | 0 | 0 | 0 | 47 | 57 | Carbamidomethylation | C10:Carbamidomethylation:1000.00 | PEAKS DB |
| total 8 peptides |
| --- |

Q4VM07|VM3VB\_MACLB

back to list

  

| Protein Coverage
| Supporting Peptides
|

Protein Coverage:

Supporting Peptides:

| Peptide | Uniq | -10lgP | Mass | Length | ppm | m/z | z | RT | Fraction | Scan | Source File | Area F1 | Area F10 | Area F2 | Area F3 | Area F4 | Area F5 | Area F7A | Area F7B | Area F8 | Area F9 | #Feature | #Feature F1 | #Feature F10 | #Feature F2 | #Feature F3 | #Feature F4 | #Feature F5 | #Feature F7A | #Feature F7B | #Feature F8 | #Feature F9 | Start | End | PTM | AScore | Found By |
| --- | --- | --- | --- | --- | --- | --- | --- | --- | --- | --- | --- | --- | --- | --- | --- | --- | --- | --- | --- | --- | --- | --- | --- | --- | --- | --- | --- | --- | --- | --- | --- | --- | --- | --- | --- | --- | --- |
| L.ESGNVNDYEVVYPQK.I | N | 99.26 | 1739.8002 | 15 | 0.4 | 870.9077 | 2 | 11.79 | 3 | F3:2512 | DaRuMH\_F2.raw | 1.5166E5 |  | 1.2778E6 |  |  |  |  |  |  |  | 2 | 1 | 0 | 1 | 0 | 0 | 0 | 0 | 0 | 0 | 0 | 24 | 38 |  |  | PEAKS DB |
| E.SGNVNDYEVVYPQK.I | N | 80.53 | 1610.7576 | 14 | 1.1 | 806.3870 | 2 | 11.73 | 3 | F3:2451 | DaRuMH\_F2.raw |  |  | 3.1229E5 |  |  |  |  |  |  |  | 1 | 0 | 0 | 1 | 0 | 0 | 0 | 0 | 0 | 0 | 0 | 25 | 38 |  |  | PEAKS DB |
| S.GNVNDYEVVYPQK.I | N | 78.53 | 1523.7256 | 13 | 0.7 | 762.8706 | 2 | 11.75 | 3 | F3:2472 | DaRuMH\_F2.raw |  |  | 1.2901E5 |  |  |  |  |  |  |  | 1 | 0 | 0 | 1 | 0 | 0 | 0 | 0 | 0 | 0 | 0 | 26 | 38 |  |  | PEAKS DB |
| K.YSVGIVQDHSK.I | Y | 76.11 | 1231.6196 | 11 | -0.3 | 616.8169 | 2 | 11.20 | 2 | F2:2042 | DaRuMH\_F10.raw |  | 1.1142E6 |  |  |  |  |  |  |  |  | 2 | 0 | 2 | 0 | 0 | 0 | 0 | 0 | 0 | 0 | 0 | 317 | 327 |  |  | PEAKS DB |
| V.TQTNWESDEPIK.A | N | 62.34 | 1446.6627 | 12 | 0.2 | 724.3387 | 2 | 11.62 | 2 | F2:2463 | DaRuMH\_F10.raw |  | 8.3861E4 |  |  |  |  |  |  |  |  | 1 | 0 | 1 | 0 | 0 | 0 | 0 | 0 | 0 | 0 | 0 | 173 | 184 |  |  | PEAKS DB |
| total 5 peptides |
| --- |

A5A6M5|K1H1\_PANTR

back to list

  

| Protein Coverage
| Supporting Peptides
|

Protein Coverage:

Supporting Peptides:

| Peptide | Uniq | -10lgP | Mass | Length | ppm | m/z | z | RT | Fraction | Scan | Source File | Area F1 | Area F10 | Area F2 | Area F3 | Area F4 | Area F5 | Area F7A | Area F7B | Area F8 | Area F9 | #Feature | #Feature F1 | #Feature F10 | #Feature F2 | #Feature F3 | #Feature F4 | #Feature F5 | #Feature F7A | #Feature F7B | #Feature F8 | #Feature F9 | Start | End | PTM | AScore | Found By |
| --- | --- | --- | --- | --- | --- | --- | --- | --- | --- | --- | --- | --- | --- | --- | --- | --- | --- | --- | --- | --- | --- | --- | --- | --- | --- | --- | --- | --- | --- | --- | --- | --- | --- | --- | --- | --- | --- |
| R.DSLENTLTESEAR.Y | N | 71.36 | 1463.6740 | 13 | 0.3 | 732.8445 | 2 | 12.07 | 2 | F2:2843 | DaRuMH\_F10.raw |  | 1.1372E5 |  |  |  |  |  |  |  |  | 1 | 0 | 1 | 0 | 0 | 0 | 0 | 0 | 0 | 0 | 0 | 294 | 306 |  |  | PEAKS DB |
| R.LEC(+57.02)EINTYR.S | N | 64.62 | 1196.5496 | 9 | 0.1 | 599.2821 | 2 | 11.39 | 2 | F2:2246 | DaRuMH\_F10.raw |  | 1.0317E5 |  |  |  |  |  |  |  |  | 1 | 0 | 1 | 0 | 0 | 0 | 0 | 0 | 0 | 0 | 0 | 349 | 357 | Carbamidomethylation | C3:Carbamidomethylation:1000.00 | PEAKS DB |
| R.LNVEVDAAPTVDLNR.V | N | 64.53 | 1624.8420 | 15 | 1.0 | 813.4291 | 2 | 14.34 | 2 | F2:3996 | DaRuMH\_F10.raw |  | 1.8344E5 |  |  |  |  |  |  |  |  | 1 | 0 | 1 | 0 | 0 | 0 | 0 | 0 | 0 | 0 | 0 | 209 | 223 |  |  | PEAKS DB |
| R.QNQEYQVLLDVR.A | N | 63.26 | 1503.7681 | 12 | 0.1 | 752.8914 | 2 | 16.02 | 2 | F2:4538 | DaRuMH\_F10.raw |  | 5.597E5 |  |  |  |  |  |  |  |  | 1 | 0 | 1 | 0 | 0 | 0 | 0 | 0 | 0 | 0 | 0 | 335 | 346 |  |  | PEAKS DB |
| R.ILDELTLC(+57.02)K.S | N | 62.63 | 1103.5896 | 9 | 1.2 | 552.8027 | 2 | 13.99 | 2 | F2:3889 | DaRuMH\_F10.raw |  | 7.8371E4 |  |  |  |  |  |  |  |  | 1 | 0 | 1 | 0 | 0 | 0 | 0 | 0 | 0 | 0 | 0 | 165 | 173 | Carbamidomethylation | C8:Carbamidomethylation:1000.00 | PEAKS DB |
| R.SQYEALVETNR.R | N | 61.76 | 1308.6310 | 11 | 0.6 | 655.3232 | 2 | 11.56 | 2 | F2:2420 | DaRuMH\_F10.raw |  | 6.4459E4 |  |  |  |  |  |  |  |  | 1 | 0 | 1 | 0 | 0 | 0 | 0 | 0 | 0 | 0 | 0 | 230 | 240 |  |  | PEAKS DB |
| R.EVEQWFTTQTEELNK.Q | Y | 59.74 | 1880.8792 | 15 | 0.2 | 941.4470 | 2 | 17.78 | 2 | F2:4977 | DaRuMH\_F10.raw |  | 2.6662E5 |  |  |  |  |  |  |  |  | 1 | 0 | 1 | 0 | 0 | 0 | 0 | 0 | 0 | 0 | 0 | 242 | 256 |  |  | PEAKS DB |
| R.DNAELENLIR.E | N | 56.29 | 1185.5989 | 10 | 1.1 | 593.8074 | 2 | 16.86 | 2 | F2:4755 | DaRuMH\_F10.raw |  | 2.442E5 |  |  |  |  |  |  |  |  | 1 | 0 | 1 | 0 | 0 | 0 | 0 | 0 | 0 | 0 | 0 | 80 | 89 |  |  | PEAKS DB |
| K.LAADDFR.T | N | 55.79 | 806.3922 | 7 | 5.8 | 404.2034 | 2 | 11.46 | 4 | F4:2305 | DaRuMH\_F3.raw | 3.0917E5 |  | 1.9305E4 | 0 |  |  |  |  |  |  | 2 | 1 | 0 | 1 | 0 | 0 | 0 | 0 | 0 | 0 | 0 | 136 | 142 |  |  | PEAKS DB |
| total 9 peptides |
| --- |

Q15323|K1H1\_HUMAN

back to list

  

| Protein Coverage
| Supporting Peptides
|

Protein Coverage:

Supporting Peptides:

| Peptide | Uniq | -10lgP | Mass | Length | ppm | m/z | z | RT | Fraction | Scan | Source File | Area F1 | Area F10 | Area F2 | Area F3 | Area F4 | Area F5 | Area F7A | Area F7B | Area F8 | Area F9 | #Feature | #Feature F1 | #Feature F10 | #Feature F2 | #Feature F3 | #Feature F4 | #Feature F5 | #Feature F7A | #Feature F7B | #Feature F8 | #Feature F9 | Start | End | PTM | AScore | Found By |
| --- | --- | --- | --- | --- | --- | --- | --- | --- | --- | --- | --- | --- | --- | --- | --- | --- | --- | --- | --- | --- | --- | --- | --- | --- | --- | --- | --- | --- | --- | --- | --- | --- | --- | --- | --- | --- | --- |
| R.DSLENTLTESEAR.Y | N | 71.36 | 1463.6740 | 13 | 0.3 | 732.8445 | 2 | 12.07 | 2 | F2:2843 | DaRuMH\_F10.raw |  | 1.1372E5 |  |  |  |  |  |  |  |  | 1 | 0 | 1 | 0 | 0 | 0 | 0 | 0 | 0 | 0 | 0 | 294 | 306 |  |  | PEAKS DB |
| R.LEC(+57.02)EINTYR.S | N | 64.62 | 1196.5496 | 9 | 0.1 | 599.2821 | 2 | 11.39 | 2 | F2:2246 | DaRuMH\_F10.raw |  | 1.0317E5 |  |  |  |  |  |  |  |  | 1 | 0 | 1 | 0 | 0 | 0 | 0 | 0 | 0 | 0 | 0 | 349 | 357 | Carbamidomethylation | C3:Carbamidomethylation:1000.00 | PEAKS DB |
| R.LNVEVDAAPTVDLNR.V | N | 64.53 | 1624.8420 | 15 | 1.0 | 813.4291 | 2 | 14.34 | 2 | F2:3996 | DaRuMH\_F10.raw |  | 1.8344E5 |  |  |  |  |  |  |  |  | 1 | 0 | 1 | 0 | 0 | 0 | 0 | 0 | 0 | 0 | 0 | 209 | 223 |  |  | PEAKS DB |
| R.QNQEYQVLLDVR.A | N | 63.26 | 1503.7681 | 12 | 0.1 | 752.8914 | 2 | 16.02 | 2 | F2:4538 | DaRuMH\_F10.raw |  | 5.597E5 |  |  |  |  |  |  |  |  | 1 | 0 | 1 | 0 | 0 | 0 | 0 | 0 | 0 | 0 | 0 | 335 | 346 |  |  | PEAKS DB |
| R.ILDELTLC(+57.02)K.S | N | 62.63 | 1103.5896 | 9 | 1.2 | 552.8027 | 2 | 13.99 | 2 | F2:3889 | DaRuMH\_F10.raw |  | 7.8371E4 |  |  |  |  |  |  |  |  | 1 | 0 | 1 | 0 | 0 | 0 | 0 | 0 | 0 | 0 | 0 | 165 | 173 | Carbamidomethylation | C8:Carbamidomethylation:1000.00 | PEAKS DB |
| R.SQYEALVETNR.R | N | 61.76 | 1308.6310 | 11 | 0.6 | 655.3232 | 2 | 11.56 | 2 | F2:2420 | DaRuMH\_F10.raw |  | 6.4459E4 |  |  |  |  |  |  |  |  | 1 | 0 | 1 | 0 | 0 | 0 | 0 | 0 | 0 | 0 | 0 | 230 | 240 |  |  | PEAKS DB |
| R.EVEQWFTTQTEELNK.Q | Y | 59.74 | 1880.8792 | 15 | 0.2 | 941.4470 | 2 | 17.78 | 2 | F2:4977 | DaRuMH\_F10.raw |  | 2.6662E5 |  |  |  |  |  |  |  |  | 1 | 0 | 1 | 0 | 0 | 0 | 0 | 0 | 0 | 0 | 0 | 242 | 256 |  |  | PEAKS DB |
| R.DNAELENLIR.E | N | 56.29 | 1185.5989 | 10 | 1.1 | 593.8074 | 2 | 16.86 | 2 | F2:4755 | DaRuMH\_F10.raw |  | 2.442E5 |  |  |  |  |  |  |  |  | 1 | 0 | 1 | 0 | 0 | 0 | 0 | 0 | 0 | 0 | 0 | 80 | 89 |  |  | PEAKS DB |
| K.LAADDFR.T | N | 55.79 | 806.3922 | 7 | 5.8 | 404.2034 | 2 | 11.46 | 4 | F4:2305 | DaRuMH\_F3.raw | 3.0917E5 |  | 1.9305E4 | 0 |  |  |  |  |  |  | 2 | 1 | 0 | 1 | 0 | 0 | 0 | 0 | 0 | 0 | 0 | 136 | 142 |  |  | PEAKS DB |
| total 9 peptides |
| --- |

P02533|K1C14\_HUMAN

back to list

  

| Protein Coverage
| Supporting Peptides
|

Protein Coverage:

Supporting Peptides:

| Peptide | Uniq | -10lgP | Mass | Length | ppm | m/z | z | RT | Fraction | Scan | Source File | Area F1 | Area F10 | Area F2 | Area F3 | Area F4 | Area F5 | Area F7A | Area F7B | Area F8 | Area F9 | #Feature | #Feature F1 | #Feature F10 | #Feature F2 | #Feature F3 | #Feature F4 | #Feature F5 | #Feature F7A | #Feature F7B | #Feature F8 | #Feature F9 | Start | End | PTM | AScore | Found By |
| --- | --- | --- | --- | --- | --- | --- | --- | --- | --- | --- | --- | --- | --- | --- | --- | --- | --- | --- | --- | --- | --- | --- | --- | --- | --- | --- | --- | --- | --- | --- | --- | --- | --- | --- | --- | --- | --- |
| R.ALEEANADLEVK.I | N | 73.35 | 1300.6510 | 12 | -0.3 | 651.3326 | 2 | 11.62 | 10 | F10:2472 | DaRuMH\_F9.raw | 2.2113E5 |  |  |  |  |  |  |  |  | 1.2264E5 | 2 | 1 | 0 | 0 | 0 | 0 | 0 | 0 | 0 | 0 | 1 | 135 | 146 |  |  | PEAKS DB |
| R.EVATNSELVQSGK.S | N | 72.96 | 1360.6833 | 13 | 0.6 | 681.3494 | 2 | 11.17 | 10 | F10:2037 | DaRuMH\_F9.raw | 6.2613E4 |  |  |  |  |  |  |  |  | 9.2342E4 | 2 | 1 | 0 | 0 | 0 | 0 | 0 | 0 | 0 | 0 | 1 | 316 | 328 |  |  | PEAKS DB |
| K.VTMQNLNDR.L | N | 62.03 | 1089.5237 | 9 | 0.4 | 545.7693 | 2 | 11.23 | 1 | F1:2101 | DaRuMH\_F1.raw | 3.6337E5 |  |  |  |  |  |  |  |  |  | 1 | 1 | 0 | 0 | 0 | 0 | 0 | 0 | 0 | 0 | 0 | 117 | 125 |  |  | PEAKS DB |
| R.VLDELTLAR.A | N | 59.08 | 1028.5865 | 9 | 5.5 | 515.3004 | 2 | 13.27 | 4 | F4:3756 | DaRuMH\_F3.raw | 3.7324E5 |  |  | 7.5584E4 |  |  |  | 3.1071E4 |  |  | 3 | 1 | 0 | 0 | 1 | 0 | 0 | 0 | 1 | 0 | 0 | 224 | 232 |  |  | PEAKS DB |
| R.LEQEIATYR.R | N | 58.00 | 1121.5717 | 9 | -0.7 | 561.7927 | 2 | 11.45 | 1 | F1:2307 | DaRuMH\_F1.raw | 1.6181E5 |  |  |  | 3.2981E4 |  |  |  |  |  | 2 | 1 | 0 | 0 | 0 | 1 | 0 | 0 | 0 | 0 | 0 | 408 | 416 |  |  | PEAKS DB |
| R.LAADDFR.T | N | 55.79 | 806.3922 | 7 | 5.8 | 404.2034 | 2 | 11.46 | 4 | F4:2305 | DaRuMH\_F3.raw | 3.0917E5 |  | 1.9305E4 | 0 |  |  |  |  |  |  | 2 | 1 | 0 | 1 | 0 | 0 | 0 | 0 | 0 | 0 | 0 | 195 | 201 |  |  | PEAKS DB |
| K.ILTATVDNANVLLQIDNAR.L | Y | 55.04 | 2053.1167 | 19 | 0.2 | 685.3796 | 3 | 28.08 | 10 | F10:14134 | DaRuMH\_F9.raw |  |  |  |  |  |  |  |  |  | 1.8198E5 | 1 | 0 | 0 | 0 | 0 | 0 | 0 | 0 | 0 | 0 | 1 | 176 | 194 |  |  | PEAKS DB |
| K.DAEEWFFTK.T | Y | 54.42 | 1171.5186 | 9 | 0.4 | 586.7668 | 2 | 24.65 | 2 | F2:7098 | DaRuMH\_F10.raw |  | 1.7321E4 |  |  |  |  |  |  |  |  | 1 | 0 | 1 | 0 | 0 | 0 | 0 | 0 | 0 | 0 | 0 | 301 | 309 |  |  | PEAKS DB |
| total 8 peptides |
| --- |

Q4PRC6|SL7\_DABSI

back to list

  

| Protein Coverage
| Supporting Peptides
|

Protein Coverage:

Supporting Peptides:

| Peptide | Uniq | -10lgP | Mass | Length | ppm | m/z | z | RT | Fraction | Scan | Source File | Area F1 | Area F10 | Area F2 | Area F3 | Area F4 | Area F5 | Area F7A | Area F7B | Area F8 | Area F9 | #Feature | #Feature F1 | #Feature F10 | #Feature F2 | #Feature F3 | #Feature F4 | #Feature F5 | #Feature F7A | #Feature F7B | #Feature F8 | #Feature F9 | Start | End | PTM | AScore | Found By |
| --- | --- | --- | --- | --- | --- | --- | --- | --- | --- | --- | --- | --- | --- | --- | --- | --- | --- | --- | --- | --- | --- | --- | --- | --- | --- | --- | --- | --- | --- | --- | --- | --- | --- | --- | --- | --- | --- |
| K.QDC(+57.02)LSDWSFYEGYC(+57.02)YK.V | N | 83.90 | 2119.8291 | 16 | 1.2 | 1060.9231 | 2 | 27.52 | 10 | F10:13868 | DaRuMH\_F9.raw |  |  |  |  |  |  |  |  |  | 9.4015E6 | 1 | 0 | 0 | 0 | 0 | 0 | 0 | 0 | 0 | 0 | 1 | 25 | 40 | Carbamidomethylation | C3:Carbamidomethylation:1000.00;C14:Carbamidomethylation:1000.00 | PEAKS DB |
| R.WSDGVNLDYK.A | Y | 81.86 | 1195.5509 | 10 | 1.4 | 598.7836 | 2 | 12.08 | 10 | F10:2858 | DaRuMH\_F9.raw |  |  |  |  |  |  |  |  |  | 1.0773E7 | 1 | 0 | 0 | 0 | 0 | 0 | 0 | 0 | 0 | 0 | 1 | 104 | 113 |  |  | PEAKS DB |
| R.FDFFWIGLR.D | Y | 67.36 | 1199.6127 | 9 | 0.7 | 600.8140 | 2 | 52.90 | 9 | F9:19031 | DaRuMH\_F8.raw |  |  |  |  |  |  |  |  | 2.837E4 | 7.0316E6 | 3 | 0 | 0 | 0 | 0 | 0 | 0 | 0 | 0 | 1 | 2 | 86 | 94 |  |  | PEAKS DB |
| K.TTDNQWLR.W | Y | 58.23 | 1032.4989 | 8 | 0.0 | 517.2567 | 2 | 11.64 | 10 | F10:2530 | DaRuMH\_F9.raw |  |  |  |  |  |  |  |  |  | 1.3338E6 | 1 | 0 | 0 | 0 | 0 | 0 | 0 | 0 | 0 | 0 | 1 | 126 | 133 |  |  | PEAKS DB |
| K.FC(+57.02)NEQVNGGYLVSFR.S | Y | 55.31 | 1788.8253 | 15 | -0.2 | 895.4198 | 2 | 16.79 | 10 | F10:6708 | DaRuMH\_F9.raw |  |  |  |  |  |  |  |  |  | 1.1665E6 | 1 | 0 | 0 | 0 | 0 | 0 | 0 | 0 | 0 | 0 | 1 | 54 | 68 | Carbamidomethylation | C2:Carbamidomethylation:1000.00 | PEAKS DB |
| total 5 peptides |
| --- |

E0Y418|VSP1\_MACLB

back to list

  

| Protein Coverage
| Supporting Peptides
|

Protein Coverage:

Supporting Peptides:

| Peptide | Uniq | -10lgP | Mass | Length | ppm | m/z | z | RT | Fraction | Scan | Source File | Area F1 | Area F10 | Area F2 | Area F3 | Area F4 | Area F5 | Area F7A | Area F7B | Area F8 | Area F9 | #Feature | #Feature F1 | #Feature F10 | #Feature F2 | #Feature F3 | #Feature F4 | #Feature F5 | #Feature F7A | #Feature F7B | #Feature F8 | #Feature F9 | Start | End | PTM | AScore | Found By |
| --- | --- | --- | --- | --- | --- | --- | --- | --- | --- | --- | --- | --- | --- | --- | --- | --- | --- | --- | --- | --- | --- | --- | --- | --- | --- | --- | --- | --- | --- | --- | --- | --- | --- | --- | --- | --- | --- |
| Q.GGIDTC(+57.02)LADSGGPLIC(+57.02)NGQFQGIVAWGR.H | Y | 79.19 | 2918.3804 | 28 | 0.6 | 973.8013 | 3 | 42.29 | 10 | F10:20657 | DaRuMH\_F9.raw |  |  |  |  |  |  |  |  |  | 1.1242E7 | 1 | 0 | 0 | 0 | 0 | 0 | 0 | 0 | 0 | 0 | 1 | 197 | 224 | Carbamidomethylation | C6:Carbamidomethylation:1000.00;C16:Carbamidomethylation:1000.00 | PEAKS DB |
| F.SLPSSPPTVGSVC(+57.02)R.I | N | 74.53 | 1442.7188 | 14 | -0.3 | 722.3665 | 2 | 11.68 | 9 | F9:2441 | DaRuMH\_F8.raw |  |  |  |  |  |  |  | 3.7249E6 | 3.8511E6 | 5.9759E7 | 3 | 0 | 0 | 0 | 0 | 0 | 0 | 0 | 1 | 1 | 1 | 132 | 145 | Carbamidomethylation | C13:Carbamidomethylation:1000.00 | PEAKS DB |
| C.NGQFQGIVAWGR.H | Y | 66.68 | 1331.6735 | 12 | 0.9 | 666.8446 | 2 | 16.27 | 10 | F10:6345 | DaRuMH\_F9.raw |  |  |  |  |  |  |  |  |  | 1.3967E6 | 2 | 0 | 0 | 0 | 0 | 0 | 0 | 0 | 0 | 0 | 2 | 213 | 224 |  |  | PEAKS DB |
| M.FFC(+57.02)LSNK.S | N | 60.97 | 914.4320 | 7 | 0.1 | 458.2233 | 2 | 11.99 | 8 | F8:2873 | DaRuMH\_F7B.raw | 1.4908E6 |  |  |  |  | 7.7161E4 | 1.1075E6 | 1.618E7 | 9.6782E6 |  | 5 | 1 | 0 | 0 | 0 | 0 | 1 | 1 | 1 | 1 | 0 | 98 | 104 | Carbamidomethylation | C3:Carbamidomethylation:1000.00 | PEAKS DB |
| N.GQFQGIVAWGR.H | Y | 58.60 | 1217.6305 | 11 | 0.8 | 609.8230 | 2 | 16.34 | 10 | F10:6378 | DaRuMH\_F9.raw |  |  |  |  |  |  |  |  |  | 1.0267E6 | 1 | 0 | 0 | 0 | 0 | 0 | 0 | 0 | 0 | 0 | 1 | 214 | 224 |  |  | PEAKS DB |
| total 5 peptides |
| --- |

E0Y420|VSP3\_MACLB

back to list

  

| Protein Coverage
| Supporting Peptides
|

Protein Coverage:

Supporting Peptides:

| Peptide | Uniq | -10lgP | Mass | Length | ppm | m/z | z | RT | Fraction | Scan | Source File | Area F1 | Area F10 | Area F2 | Area F3 | Area F4 | Area F5 | Area F7A | Area F7B | Area F8 | Area F9 | #Feature | #Feature F1 | #Feature F10 | #Feature F2 | #Feature F3 | #Feature F4 | #Feature F5 | #Feature F7A | #Feature F7B | #Feature F8 | #Feature F9 | Start | End | PTM | AScore | Found By |
| --- | --- | --- | --- | --- | --- | --- | --- | --- | --- | --- | --- | --- | --- | --- | --- | --- | --- | --- | --- | --- | --- | --- | --- | --- | --- | --- | --- | --- | --- | --- | --- | --- | --- | --- | --- | --- | --- |
| K.TSTHIAPLSLPSSPPSVGSVC(+57.02)R.I | Y | 90.92 | 2249.1475 | 22 | 0.3 | 750.7233 | 3 | 13.02 | 8 | F8:3721 | DaRuMH\_F7B.raw |  |  |  |  |  |  |  | 5.5452E6 | 2.0714E6 |  | 2 | 0 | 0 | 0 | 0 | 0 | 0 | 0 | 1 | 1 | 0 | 122 | 143 | Carbamidomethylation | C21:Carbamidomethylation:1000.00 | PEAKS DB |
| L.SLPSSPPSVGSVC(+57.02)R.I | N | 81.50 | 1428.7031 | 14 | 0.2 | 715.3590 | 2 | 11.65 | 8 | F8:2499 | DaRuMH\_F7B.raw |  |  |  |  |  |  | 2.3471E6 | 1.3457E7 | 5.5106E6 |  | 3 | 0 | 0 | 0 | 0 | 0 | 0 | 1 | 1 | 1 | 0 | 130 | 143 | Carbamidomethylation | C13:Carbamidomethylation:1000.00 | PEAKS DB |
| K.FFC(+57.02)LSSK.N | N | 67.85 | 887.4211 | 7 | -0.6 | 444.7176 | 2 | 12.08 | 8 | F8:2955 | DaRuMH\_F7B.raw | 2.546E6 |  |  |  |  |  | 6.2481E5 | 9.2089E6 | 3.0567E6 | 5.9046E5 | 5 | 1 | 0 | 0 | 0 | 0 | 0 | 1 | 1 | 1 | 1 | 96 | 102 | Carbamidomethylation | C3:Carbamidomethylation:1000.00 | PEAKS DB |
| I.GGDEC(+57.02)NINEHR.S | N | 67.81 | 1299.5262 | 11 | 0.2 | 650.7705 | 2 | 10.70 | 9 | F9:1597 | DaRuMH\_F8.raw |  |  |  |  |  |  |  | 2.6409E5 | 7.79E5 |  | 2 | 0 | 0 | 0 | 0 | 0 | 0 | 0 | 1 | 1 | 0 | 27 | 37 | Carbamidomethylation | C5:Carbamidomethylation:1000.00 | PEAKS DB |
| K.WDKDIMLIK.L | N | 53.69 | 1160.6263 | 9 | 0.0 | 581.3204 | 2 | 12.61 | 8 | F8:3439 | DaRuMH\_F7B.raw |  |  |  |  |  |  |  | 3.5006E4 |  |  | 1 | 0 | 0 | 0 | 0 | 0 | 0 | 0 | 1 | 0 | 0 | 107 | 115 |  |  | PEAKS DB |
| total 5 peptides |
| --- |

A8Y7P1|VKTB1\_DABSI

back to list

  

| Protein Coverage
| Supporting Peptides
|

Protein Coverage:

Supporting Peptides:

| Peptide | Uniq | -10lgP | Mass | Length | ppm | m/z | z | RT | Fraction | Scan | Source File | Area F1 | Area F10 | Area F2 | Area F3 | Area F4 | Area F5 | Area F7A | Area F7B | Area F8 | Area F9 | #Feature | #Feature F1 | #Feature F10 | #Feature F2 | #Feature F3 | #Feature F4 | #Feature F5 | #Feature F7A | #Feature F7B | #Feature F8 | #Feature F9 | Start | End | PTM | AScore | Found By |
| --- | --- | --- | --- | --- | --- | --- | --- | --- | --- | --- | --- | --- | --- | --- | --- | --- | --- | --- | --- | --- | --- | --- | --- | --- | --- | --- | --- | --- | --- | --- | --- | --- | --- | --- | --- | --- | --- |
| K.FC(+57.02)YLPADPGEC(+57.02)LAHM(+15.99)R.S | Y | 71.56 | 1951.8379 | 16 | 6.4 | 651.6204 | 3 | 12.29 | 4 | F4:3037 | DaRuMH\_F3.raw |  |  |  | 5.2498E6 |  |  |  |  |  |  | 1 | 0 | 0 | 0 | 1 | 0 | 0 | 0 | 0 | 0 | 0 | 30 | 45 | Carbamidomethylation; Oxidation (M) | C2:Carbamidomethylation:1000.00;C11:Carbamidomethylation:1000.00;M15:Oxidation (M):1000.00 | PEAKS DB |
| K.EFIYGGC(+57.02)HGNANK.F | Y | 69.36 | 1465.6409 | 13 | 6.5 | 733.8282 | 2 | 11.03 | 4 | F4:1890 | DaRuMH\_F3.raw |  |  | 2.0182E5 | 5.0194E6 |  |  |  |  |  |  | 4 | 0 | 0 | 2 | 2 | 0 | 0 | 0 | 0 | 0 | 0 | 58 | 70 | Carbamidomethylation | C7:Carbamidomethylation:1000.00 | PEAKS DB |
| R.SFYYDSESK.K | N | 67.86 | 1124.4662 | 9 | 1.1 | 563.2410 | 2 | 11.46 | 3 | F3:2160 | DaRuMH\_F2.raw |  |  | 2.8891E6 | 1.2846E8 |  |  |  |  |  |  | 2 | 0 | 0 | 1 | 1 | 0 | 0 | 0 | 0 | 0 | 0 | 46 | 54 |  |  | PEAKS DB |
| K.EFIYGGC(+57.02)HGNAN.K | N | 64.05 | 1337.5459 | 12 | 6.3 | 669.7806 | 2 | 11.46 | 4 | F4:2300 | DaRuMH\_F3.raw |  |  |  | 1.3232E7 |  |  |  |  |  |  | 1 | 0 | 0 | 0 | 1 | 0 | 0 | 0 | 0 | 0 | 0 | 58 | 69 | Carbamidomethylation | C7:Carbamidomethylation:1000.00 | PEAKS DB |
| R.SFYYDSESKK.C | N | 61.36 | 1252.5612 | 10 | 7.0 | 418.5282 | 3 | 10.99 | 4 | F4:1828 | DaRuMH\_F3.raw |  |  | 4.7424E4 | 2.6207E6 |  |  |  |  |  |  | 2 | 0 | 0 | 1 | 1 | 0 | 0 | 0 | 0 | 0 | 0 | 46 | 55 |  |  | PEAKS DB |
| total 5 peptides |
| --- |

A8Y7P5|VKTB5\_DABSI

back to list

  

| Protein Coverage
| Supporting Peptides
|

Protein Coverage:

Supporting Peptides:

| Peptide | Uniq | -10lgP | Mass | Length | ppm | m/z | z | RT | Fraction | Scan | Source File | Area F1 | Area F10 | Area F2 | Area F3 | Area F4 | Area F5 | Area F7A | Area F7B | Area F8 | Area F9 | #Feature | #Feature F1 | #Feature F10 | #Feature F2 | #Feature F3 | #Feature F4 | #Feature F5 | #Feature F7A | #Feature F7B | #Feature F8 | #Feature F9 | Start | End | PTM | AScore | Found By |
| --- | --- | --- | --- | --- | --- | --- | --- | --- | --- | --- | --- | --- | --- | --- | --- | --- | --- | --- | --- | --- | --- | --- | --- | --- | --- | --- | --- | --- | --- | --- | --- | --- | --- | --- | --- | --- | --- |
| K.FC(+57.02)YLPADPGEC(+57.02)LAHM(+15.99)R.S | Y | 71.56 | 1951.8379 | 16 | 6.4 | 651.6204 | 3 | 12.29 | 4 | F4:3037 | DaRuMH\_F3.raw |  |  |  | 5.2498E6 |  |  |  |  |  |  | 1 | 0 | 0 | 0 | 1 | 0 | 0 | 0 | 0 | 0 | 0 | 30 | 45 | Carbamidomethylation; Oxidation (M) | C2:Carbamidomethylation:1000.00;C11:Carbamidomethylation:1000.00;M15:Oxidation (M):1000.00 | PEAKS DB |
| K.EFIYGGC(+57.02)HGNANK.F | Y | 69.36 | 1465.6409 | 13 | 6.5 | 733.8282 | 2 | 11.03 | 4 | F4:1890 | DaRuMH\_F3.raw |  |  | 2.0182E5 | 5.0194E6 |  |  |  |  |  |  | 4 | 0 | 0 | 2 | 2 | 0 | 0 | 0 | 0 | 0 | 0 | 58 | 70 | Carbamidomethylation | C7:Carbamidomethylation:1000.00 | PEAKS DB |
| R.SFYYDSESK.K | N | 67.86 | 1124.4662 | 9 | 1.1 | 563.2410 | 2 | 11.46 | 3 | F3:2160 | DaRuMH\_F2.raw |  |  | 2.8891E6 | 1.2846E8 |  |  |  |  |  |  | 2 | 0 | 0 | 1 | 1 | 0 | 0 | 0 | 0 | 0 | 0 | 46 | 54 |  |  | PEAKS DB |
| K.EFIYGGC(+57.02)HGNAN.K | N | 64.05 | 1337.5459 | 12 | 6.3 | 669.7806 | 2 | 11.46 | 4 | F4:2300 | DaRuMH\_F3.raw |  |  |  | 1.3232E7 |  |  |  |  |  |  | 1 | 0 | 0 | 0 | 1 | 0 | 0 | 0 | 0 | 0 | 0 | 58 | 69 | Carbamidomethylation | C7:Carbamidomethylation:1000.00 | PEAKS DB |
| R.SFYYDSESKK.C | N | 61.36 | 1252.5612 | 10 | 7.0 | 418.5282 | 3 | 10.99 | 4 | F4:1828 | DaRuMH\_F3.raw |  |  | 4.7424E4 | 2.6207E6 |  |  |  |  |  |  | 2 | 0 | 0 | 1 | 1 | 0 | 0 | 0 | 0 | 0 | 0 | 46 | 55 |  |  | PEAKS DB |
| total 5 peptides |
| --- |

A8Y7N8|VKTC5\_DABSI

back to list

  

| Protein Coverage
| Supporting Peptides
|

Protein Coverage:

Supporting Peptides:

| Peptide | Uniq | -10lgP | Mass | Length | ppm | m/z | z | RT | Fraction | Scan | Source File | Area F1 | Area F10 | Area F2 | Area F3 | Area F4 | Area F5 | Area F7A | Area F7B | Area F8 | Area F9 | #Feature | #Feature F1 | #Feature F10 | #Feature F2 | #Feature F3 | #Feature F4 | #Feature F5 | #Feature F7A | #Feature F7B | #Feature F8 | #Feature F9 | Start | End | PTM | AScore | Found By |
| --- | --- | --- | --- | --- | --- | --- | --- | --- | --- | --- | --- | --- | --- | --- | --- | --- | --- | --- | --- | --- | --- | --- | --- | --- | --- | --- | --- | --- | --- | --- | --- | --- | --- | --- | --- | --- | --- |
| F.FYGGC(+57.02)GGNDNNFETR.K | N | 80.84 | 1706.6743 | 15 | 0.8 | 854.3452 | 2 | 11.51 | 1 | F1:2367 | DaRuMH\_F1.raw | 7.0187E5 |  |  |  |  |  |  |  |  |  | 1 | 1 | 0 | 0 | 0 | 0 | 0 | 0 | 0 | 0 | 0 | 60 | 74 | Carbamidomethylation | C5:Carbamidomethylation:1000.00 | PEAKS DB |
| T.FC(+57.02)NLAPESGR.C | N | 78.41 | 1149.5237 | 10 | 0.3 | 575.7693 | 2 | 11.37 | 3 | F3:2103 | DaRuMH\_F2.raw | 1.8346E6 |  | 2.7999E5 |  |  |  |  |  |  |  | 2 | 1 | 0 | 1 | 0 | 0 | 0 | 0 | 0 | 0 | 0 | 30 | 39 | Carbamidomethylation | C2:Carbamidomethylation:1000.00 | PEAKS DB |
| G.HDRPTFC(+57.02)NLAPESGR.C | N | 77.03 | 1755.8110 | 15 | 0.4 | 586.2778 | 3 | 11.25 | 5 | F5:2071 | DaRuMH\_F4.raw |  |  |  |  | 2.8813E5 |  |  |  |  |  | 1 | 0 | 0 | 0 | 0 | 1 | 0 | 0 | 0 | 0 | 0 | 25 | 39 | Carbamidomethylation | C7:Carbamidomethylation:1000.00 | PEAKS DB |
| R.IYYNPDSNK.C | Y | 63.73 | 1112.5138 | 9 | -0.4 | 557.2640 | 2 | 11.19 | 1 | F1:2054 | DaRuMH\_F1.raw | 1.4811E6 | 3.1845E4 |  |  |  |  |  |  |  |  | 2 | 1 | 1 | 0 | 0 | 0 | 0 | 0 | 0 | 0 | 0 | 47 | 55 |  |  | PEAKS DB |
| Y.GGC(+57.02)GGNDNNFETR.K | N | 59.36 | 1396.5426 | 13 | 0.5 | 699.2789 | 2 | 10.77 | 3 | F3:1627 | DaRuMH\_F2.raw |  |  | 4.2905E4 |  |  |  |  |  |  |  | 1 | 0 | 0 | 1 | 0 | 0 | 0 | 0 | 0 | 0 | 0 | 62 | 74 | Carbamidomethylation | C3:Carbamidomethylation:1000.00 | PEAKS DB |
| total 5 peptides |
| --- |

O76011|KRT34\_HUMAN

back to list

  

| Protein Coverage
| Supporting Peptides
|

Protein Coverage:

Supporting Peptides:

| Peptide | Uniq | -10lgP | Mass | Length | ppm | m/z | z | RT | Fraction | Scan | Source File | Area F1 | Area F10 | Area F2 | Area F3 | Area F4 | Area F5 | Area F7A | Area F7B | Area F8 | Area F9 | #Feature | #Feature F1 | #Feature F10 | #Feature F2 | #Feature F3 | #Feature F4 | #Feature F5 | #Feature F7A | #Feature F7B | #Feature F8 | #Feature F9 | Start | End | PTM | AScore | Found By |
| --- | --- | --- | --- | --- | --- | --- | --- | --- | --- | --- | --- | --- | --- | --- | --- | --- | --- | --- | --- | --- | --- | --- | --- | --- | --- | --- | --- | --- | --- | --- | --- | --- | --- | --- | --- | --- | --- |
| R.SQYEALVEINR.R | Y | 70.42 | 1320.6674 | 11 | 0.6 | 661.3414 | 2 | 12.25 | 2 | F2:3009 | DaRuMH\_F10.raw |  | 6.0593E4 |  |  |  |  |  |  |  |  | 1 | 0 | 1 | 0 | 0 | 0 | 0 | 0 | 0 | 0 | 0 | 272 | 282 |  |  | PEAKS DB |
| R.LEC(+57.02)EINTYR.S | N | 64.62 | 1196.5496 | 9 | 0.1 | 599.2821 | 2 | 11.39 | 2 | F2:2246 | DaRuMH\_F10.raw |  | 1.0317E5 |  |  |  |  |  |  |  |  | 1 | 0 | 1 | 0 | 0 | 0 | 0 | 0 | 0 | 0 | 0 | 391 | 399 | Carbamidomethylation | C3:Carbamidomethylation:1000.00 | PEAKS DB |
| R.EVEQWFATQTEELNK.Q | N | 64.08 | 1850.8687 | 15 | 0.7 | 926.4423 | 2 | 18.38 | 2 | F2:5187 | DaRuMH\_F10.raw |  | 3.0771E5 |  |  |  |  |  |  |  |  | 1 | 0 | 1 | 0 | 0 | 0 | 0 | 0 | 0 | 0 | 0 | 284 | 298 |  |  | PEAKS DB |
| R.QNQEYQVLLDVR.A | N | 63.26 | 1503.7681 | 12 | 0.1 | 752.8914 | 2 | 16.02 | 2 | F2:4538 | DaRuMH\_F10.raw |  | 5.597E5 |  |  |  |  |  |  |  |  | 1 | 0 | 1 | 0 | 0 | 0 | 0 | 0 | 0 | 0 | 0 | 377 | 388 |  |  | PEAKS DB |
| R.ILDELTLC(+57.02)K.S | N | 62.63 | 1103.5896 | 9 | 1.2 | 552.8027 | 2 | 13.99 | 2 | F2:3889 | DaRuMH\_F10.raw |  | 7.8371E4 |  |  |  |  |  |  |  |  | 1 | 0 | 1 | 0 | 0 | 0 | 0 | 0 | 0 | 0 | 0 | 207 | 215 | Carbamidomethylation | C8:Carbamidomethylation:1000.00 | PEAKS DB |
| K.SDLESQVESLR.E | Y | 56.66 | 1261.6150 | 11 | 0.5 | 631.8151 | 2 | 12.20 | 2 | F2:2975 | DaRuMH\_F10.raw |  | 3.9128E4 |  |  |  |  |  |  |  |  | 1 | 0 | 1 | 0 | 0 | 0 | 0 | 0 | 0 | 0 | 0 | 216 | 226 |  |  | PEAKS DB |
| K.QVVSSSEQLQSC(+57.02)QAEIIELR.R | N | 55.18 | 2303.1426 | 20 | 1.2 | 768.7224 | 3 | 15.97 | 2 | F2:4542 | DaRuMH\_F10.raw |  | 4.1268E4 |  |  |  |  |  |  |  |  | 1 | 0 | 1 | 0 | 0 | 0 | 0 | 0 | 0 | 0 | 0 | 299 | 318 | Carbamidomethylation | C12:Carbamidomethylation:1000.00 | PEAKS DB |
| total 7 peptides |
| --- |

Q7T229|VSPH\_BOTJR

back to list

  

| Protein Coverage
| Supporting Peptides
|

Protein Coverage:

Supporting Peptides:

| Peptide | Uniq | -10lgP | Mass | Length | ppm | m/z | z | RT | Fraction | Scan | Source File | Area F1 | Area F10 | Area F2 | Area F3 | Area F4 | Area F5 | Area F7A | Area F7B | Area F8 | Area F9 | #Feature | #Feature F1 | #Feature F10 | #Feature F2 | #Feature F3 | #Feature F4 | #Feature F5 | #Feature F7A | #Feature F7B | #Feature F8 | #Feature F9 | Start | End | PTM | AScore | Found By |
| --- | --- | --- | --- | --- | --- | --- | --- | --- | --- | --- | --- | --- | --- | --- | --- | --- | --- | --- | --- | --- | --- | --- | --- | --- | --- | --- | --- | --- | --- | --- | --- | --- | --- | --- | --- | --- | --- |
| I.SLPSSPPSVGSVC(+57.02)R.I | N | 81.50 | 1428.7031 | 14 | 0.2 | 715.3590 | 2 | 11.65 | 8 | F8:2499 | DaRuMH\_F7B.raw |  |  |  |  |  |  | 2.3471E6 | 1.3457E7 | 5.5106E6 |  | 3 | 0 | 0 | 0 | 0 | 0 | 0 | 1 | 1 | 1 | 0 | 132 | 145 | Carbamidomethylation | C13:Carbamidomethylation:1000.00 | PEAKS DB |
| K.FFC(+57.02)LSSK.T | N | 67.85 | 887.4211 | 7 | -0.6 | 444.7176 | 2 | 12.08 | 8 | F8:2955 | DaRuMH\_F7B.raw | 2.546E6 |  |  |  |  |  | 6.2481E5 | 9.2089E6 | 3.0567E6 | 5.9046E5 | 5 | 1 | 0 | 0 | 0 | 0 | 0 | 1 | 1 | 1 | 1 | 98 | 104 | Carbamidomethylation | C3:Carbamidomethylation:1000.00 | PEAKS DB |
| I.GGDEC(+57.02)NINEHR.F | N | 67.81 | 1299.5262 | 11 | 0.2 | 650.7705 | 2 | 10.70 | 9 | F9:1597 | DaRuMH\_F8.raw |  |  |  |  |  |  |  | 2.6409E5 | 7.79E5 |  | 2 | 0 | 0 | 0 | 0 | 0 | 0 | 0 | 1 | 1 | 0 | 27 | 37 | Carbamidomethylation | C5:Carbamidomethylation:1000.00 | PEAKS DB |
| R.FHC(+57.02)SGTLINQEWVLT.A | Y | 56.47 | 1803.8615 | 15 | 0.9 | 902.9388 | 2 | 27.69 | 10 | F10:14004 | DaRuMH\_F9.raw |  |  |  |  |  |  |  |  |  | 2.5156E6 | 1 | 0 | 0 | 0 | 0 | 0 | 0 | 0 | 0 | 0 | 1 | 50 | 64 | Carbamidomethylation | C3:Carbamidomethylation:1000.00 | PEAKS DB |
| Q.EWVLTAANC(+57.02)DR.K | Y | 55.72 | 1333.6085 | 11 | 0.3 | 667.8118 | 2 | 12.63 | 9 | F9:3330 | DaRuMH\_F8.raw |  |  |  |  |  |  |  |  | 8.2963E3 |  | 1 | 0 | 0 | 0 | 0 | 0 | 0 | 0 | 0 | 1 | 0 | 60 | 70 | Carbamidomethylation | C9:Carbamidomethylation:1000.00 | PEAKS DB |
| total 5 peptides |
| --- |

P08779|K1C16\_HUMAN

back to list

  

| Protein Coverage
| Supporting Peptides
|

Protein Coverage:

Supporting Peptides:

| Peptide | Uniq | -10lgP | Mass | Length | ppm | m/z | z | RT | Fraction | Scan | Source File | Area F1 | Area F10 | Area F2 | Area F3 | Area F4 | Area F5 | Area F7A | Area F7B | Area F8 | Area F9 | #Feature | #Feature F1 | #Feature F10 | #Feature F2 | #Feature F3 | #Feature F4 | #Feature F5 | #Feature F7A | #Feature F7B | #Feature F8 | #Feature F9 | Start | End | PTM | AScore | Found By |
| --- | --- | --- | --- | --- | --- | --- | --- | --- | --- | --- | --- | --- | --- | --- | --- | --- | --- | --- | --- | --- | --- | --- | --- | --- | --- | --- | --- | --- | --- | --- | --- | --- | --- | --- | --- | --- | --- |
| R.ALEEANADLEVK.I | N | 73.35 | 1300.6510 | 12 | -0.3 | 651.3326 | 2 | 11.62 | 10 | F10:2472 | DaRuMH\_F9.raw | 2.2113E5 |  |  |  |  |  |  |  |  | 1.2264E5 | 2 | 1 | 0 | 0 | 0 | 0 | 0 | 0 | 0 | 0 | 1 | 137 | 148 |  |  | PEAKS DB |
| K.VTMQNLNDR.L | N | 62.03 | 1089.5237 | 9 | 0.4 | 545.7693 | 2 | 11.23 | 1 | F1:2101 | DaRuMH\_F1.raw | 3.6337E5 |  |  |  |  |  |  |  |  |  | 1 | 1 | 0 | 0 | 0 | 0 | 0 | 0 | 0 | 0 | 0 | 119 | 127 |  |  | PEAKS DB |
| R.VLDELTLAR.T | N | 59.08 | 1028.5865 | 9 | 5.5 | 515.3004 | 2 | 13.27 | 4 | F4:3756 | DaRuMH\_F3.raw | 3.7324E5 |  |  | 7.5584E4 |  |  |  | 3.1071E4 |  |  | 3 | 1 | 0 | 0 | 1 | 0 | 0 | 0 | 1 | 0 | 0 | 226 | 234 |  |  | PEAKS DB |
| R.LEQEIATYR.R | N | 58.00 | 1121.5717 | 9 | -0.7 | 561.7927 | 2 | 11.45 | 1 | F1:2307 | DaRuMH\_F1.raw | 1.6181E5 |  |  |  | 3.2981E4 |  |  |  |  |  | 2 | 1 | 0 | 0 | 0 | 1 | 0 | 0 | 0 | 0 | 0 | 410 | 418 |  |  | PEAKS DB |
| R.LAADDFR.T | N | 55.79 | 806.3922 | 7 | 5.8 | 404.2034 | 2 | 11.46 | 4 | F4:2305 | DaRuMH\_F3.raw | 3.0917E5 |  | 1.9305E4 | 0 |  |  |  |  |  |  | 2 | 1 | 0 | 1 | 0 | 0 | 0 | 0 | 0 | 0 | 0 | 197 | 203 |  |  | PEAKS DB |
| R.TDLEM(+15.99)QIEGLK.E | Y | 54.37 | 1291.6329 | 11 | -0.2 | 646.8236 | 2 | 13.66 | 10 | F10:4226 | DaRuMH\_F9.raw |  |  |  |  |  |  |  |  |  | 9.5971E4 | 1 | 0 | 0 | 0 | 0 | 0 | 0 | 0 | 0 | 0 | 1 | 235 | 245 | Oxidation (M) | M5:Oxidation (M):1000.00 | PEAKS DB |
| total 6 peptides |
| --- |

E5L0E3|VSPAF\_DABSI

back to list

  

| Protein Coverage
| Supporting Peptides
|

Protein Coverage:

Supporting Peptides:

| Peptide | Uniq | -10lgP | Mass | Length | ppm | m/z | z | RT | Fraction | Scan | Source File | Area F1 | Area F10 | Area F2 | Area F3 | Area F4 | Area F5 | Area F7A | Area F7B | Area F8 | Area F9 | #Feature | #Feature F1 | #Feature F10 | #Feature F2 | #Feature F3 | #Feature F4 | #Feature F5 | #Feature F7A | #Feature F7B | #Feature F8 | #Feature F9 | Start | End | PTM | AScore | Found By |
| --- | --- | --- | --- | --- | --- | --- | --- | --- | --- | --- | --- | --- | --- | --- | --- | --- | --- | --- | --- | --- | --- | --- | --- | --- | --- | --- | --- | --- | --- | --- | --- | --- | --- | --- | --- | --- | --- |
| K.FFC(+57.02)LSSK.S | N | 67.85 | 887.4211 | 7 | -0.6 | 444.7176 | 2 | 12.08 | 8 | F8:2955 | DaRuMH\_F7B.raw | 2.546E6 |  |  |  |  |  | 6.2481E5 | 9.2089E6 | 3.0567E6 | 5.9046E5 | 5 | 1 | 0 | 0 | 0 | 0 | 0 | 1 | 1 | 1 | 1 | 96 | 102 | Carbamidomethylation | C3:Carbamidomethylation:1000.00 | PEAKS DB |
| Y.STHIASLSLPSNPPR.V | Y | 67.83 | 1575.8369 | 15 | -2.0 | 526.2852 | 3 | 11.69 | 10 | F10:2514 | DaRuMH\_F9.raw |  |  |  |  |  |  |  |  | 6.5085E5 | 1.4536E6 | 2 | 0 | 0 | 0 | 0 | 0 | 0 | 0 | 0 | 1 | 1 | 123 | 137 |  |  | PEAKS DB |
| R.TLC(+57.02)AGVSGR.R | Y | 67.29 | 919.4545 | 9 | 0.3 | 460.7347 | 2 | 10.92 | 9 | F9:1744 | DaRuMH\_F8.raw |  |  |  |  |  |  |  |  | 2.7232E4 | 6.6916E4 | 2 | 0 | 0 | 0 | 0 | 0 | 0 | 0 | 0 | 1 | 1 | 187 | 195 | Carbamidomethylation | C3:Carbamidomethylation:1000.00 | PEAKS DB |
| H.IASLSLPSNPPR.V | Y | 55.22 | 1250.6982 | 12 | 0.1 | 626.3564 | 2 | 12.08 | 10 | F10:2880 | DaRuMH\_F9.raw |  |  |  |  |  |  |  |  | 4.9683E5 | 1.2708E6 | 2 | 0 | 0 | 0 | 0 | 0 | 0 | 0 | 0 | 1 | 1 | 126 | 137 |  |  | PEAKS DB |
| K.ILPFVPHC(+57.02)AN.I | Y | 55.07 | 1166.5906 | 10 | 0.4 | 584.3028 | 2 | 14.41 | 9 | F9:4494 | DaRuMH\_F8.raw |  |  |  |  |  |  |  |  | 7.826E4 |  | 1 | 0 | 0 | 0 | 0 | 0 | 0 | 0 | 0 | 1 | 0 | 156 | 165 | Carbamidomethylation | C8:Carbamidomethylation:1000.00 | PEAKS DB |
| K.WDKDIMLIK.L | N | 53.69 | 1160.6263 | 9 | 0.0 | 581.3204 | 2 | 12.61 | 8 | F8:3439 | DaRuMH\_F7B.raw |  |  |  |  |  |  |  | 3.5006E4 |  |  | 1 | 0 | 0 | 0 | 0 | 0 | 0 | 0 | 1 | 0 | 0 | 107 | 115 |  |  | PEAKS DB |
| total 6 peptides |
| --- |

P01391|3L21\_NAJKA

back to list

  

| Protein Coverage
| Supporting Peptides
|

Protein Coverage:

Supporting Peptides:

| Peptide | Uniq | -10lgP | Mass | Length | ppm | m/z | z | RT | Fraction | Scan | Source File | Area F1 | Area F10 | Area F2 | Area F3 | Area F4 | Area F5 | Area F7A | Area F7B | Area F8 | Area F9 | #Feature | #Feature F1 | #Feature F10 | #Feature F2 | #Feature F3 | #Feature F4 | #Feature F5 | #Feature F7A | #Feature F7B | #Feature F8 | #Feature F9 | Start | End | PTM | AScore | Found By |
| --- | --- | --- | --- | --- | --- | --- | --- | --- | --- | --- | --- | --- | --- | --- | --- | --- | --- | --- | --- | --- | --- | --- | --- | --- | --- | --- | --- | --- | --- | --- | --- | --- | --- | --- | --- | --- | --- |
| K.TGVDIQC(+57.02)C(+57.02)STDNC(+57.02)NPFPTR.K | Y | 94.19 | 2240.9248 | 19 | 0.5 | 1121.4702 | 2 | 12.00 | 3 | F3:2693 | DaRuMH\_F2.raw |  |  | 4.5612E5 | 8.2245E6 | 1.6373E6 |  |  |  |  |  | 4 | 0 | 0 | 1 | 2 | 1 | 0 | 0 | 0 | 0 | 0 | 50 | 68 | Carbamidomethylation | C7:Carbamidomethylation:1000.00;C8:Carbamidomethylation:1000.00;C13:Carbamidomethylation:1000.00 | PEAKS DB |
| R.VDLGC(+57.02)AATC(+57.02)PTVK.T | N | 79.07 | 1390.6584 | 13 | 0.2 | 696.3367 | 2 | 11.58 | 3 | F3:2315 | DaRuMH\_F2.raw |  |  | 3.5822E4 | 3.9358E6 | 5.0757E5 |  |  |  |  |  | 3 | 0 | 0 | 1 | 1 | 1 | 0 | 0 | 0 | 0 | 0 | 37 | 49 | Carbamidomethylation | C5:Carbamidomethylation:1000.00;C9:Carbamidomethylation:1000.00 | PEAKS DB |
| R.C(+57.02)FITPDITSK.D | N | 70.72 | 1180.5798 | 10 | 6.3 | 591.2975 | 2 | 12.40 | 4 | F4:3137 | DaRuMH\_F3.raw |  |  | 2.9192E5 | 7.4152E6 | 1.306E6 |  |  |  |  | 4.1739E4 | 4 | 0 | 0 | 1 | 1 | 1 | 0 | 0 | 0 | 0 | 1 | 3 | 12 | Carbamidomethylation | C1:Carbamidomethylation:1000.00 | PEAKS DB |
| C.STDNC(+57.02)NPFPTR.K | Y | 60.59 | 1307.5564 | 11 | 5.8 | 654.7855 | 2 | 11.41 | 4 | F4:2248 | DaRuMH\_F3.raw |  |  |  | 9.4024E5 | 1.5857E5 |  |  |  |  |  | 2 | 0 | 0 | 0 | 1 | 1 | 0 | 0 | 0 | 0 | 0 | 58 | 68 | Carbamidomethylation | C5:Carbamidomethylation:1000.00 | PEAKS DB |
| total 4 peptides |
| --- |

Q2ES50|VKT1\_DABRR

back to list

  

| Protein Coverage
| Supporting Peptides
|

Protein Coverage:

Supporting Peptides:

| Peptide | Uniq | -10lgP | Mass | Length | ppm | m/z | z | RT | Fraction | Scan | Source File | Area F1 | Area F10 | Area F2 | Area F3 | Area F4 | Area F5 | Area F7A | Area F7B | Area F8 | Area F9 | #Feature | #Feature F1 | #Feature F10 | #Feature F2 | #Feature F3 | #Feature F4 | #Feature F5 | #Feature F7A | #Feature F7B | #Feature F8 | #Feature F9 | Start | End | PTM | AScore | Found By |
| --- | --- | --- | --- | --- | --- | --- | --- | --- | --- | --- | --- | --- | --- | --- | --- | --- | --- | --- | --- | --- | --- | --- | --- | --- | --- | --- | --- | --- | --- | --- | --- | --- | --- | --- | --- | --- | --- |
| T.FC(+57.02)NLAPESGR.C | N | 78.41 | 1149.5237 | 10 | 0.3 | 575.7693 | 2 | 11.37 | 3 | F3:2103 | DaRuMH\_F2.raw | 1.8346E6 |  | 2.7999E5 |  |  |  |  |  |  |  | 2 | 1 | 0 | 1 | 0 | 0 | 0 | 0 | 0 | 0 | 0 | 30 | 39 | Carbamidomethylation | C2:Carbamidomethylation:1000.00 | PEAKS DB |
| G.HDRPTFC(+57.02)NLAPESGR.C | N | 77.03 | 1755.8110 | 15 | 0.4 | 586.2778 | 3 | 11.25 | 5 | F5:2071 | DaRuMH\_F4.raw |  |  |  |  | 2.8813E5 |  |  |  |  |  | 1 | 0 | 0 | 0 | 0 | 1 | 0 | 0 | 0 | 0 | 0 | 25 | 39 | Carbamidomethylation | C7:Carbamidomethylation:1000.00 | PEAKS DB |
| K.VFFYGGC(+57.02)GGNANNFETR.D | Y | 68.97 | 1908.8213 | 17 | 3.5 | 955.4191 | 2 | 13.88 | 7 | F7:4246 | DaRuMH\_F7A.raw |  |  |  |  |  |  | 1.0169E5 |  |  |  | 1 | 0 | 0 | 0 | 0 | 0 | 0 | 1 | 0 | 0 | 0 | 58 | 74 | Carbamidomethylation | C7:Carbamidomethylation:1000.00 | PEAKS DB |
| R.IYYNLESNK.C | N | 56.41 | 1142.5608 | 9 | -0.1 | 572.2876 | 2 | 11.56 | 2 | F2:2417 | DaRuMH\_F10.raw |  | 6.9474E4 |  |  |  |  |  |  |  |  | 1 | 0 | 1 | 0 | 0 | 0 | 0 | 0 | 0 | 0 | 0 | 47 | 55 |  |  | PEAKS DB |
| total 4 peptides |
| --- |

A8Y7P0|VKTB7\_DABSI

back to list

  

| Protein Coverage
| Supporting Peptides
|

Protein Coverage:

Supporting Peptides:

| Peptide | Uniq | -10lgP | Mass | Length | ppm | m/z | z | RT | Fraction | Scan | Source File | Area F1 | Area F10 | Area F2 | Area F3 | Area F4 | Area F5 | Area F7A | Area F7B | Area F8 | Area F9 | #Feature | #Feature F1 | #Feature F10 | #Feature F2 | #Feature F3 | #Feature F4 | #Feature F5 | #Feature F7A | #Feature F7B | #Feature F8 | #Feature F9 | Start | End | PTM | AScore | Found By |
| --- | --- | --- | --- | --- | --- | --- | --- | --- | --- | --- | --- | --- | --- | --- | --- | --- | --- | --- | --- | --- | --- | --- | --- | --- | --- | --- | --- | --- | --- | --- | --- | --- | --- | --- | --- | --- | --- |
| T.FC(+57.02)NLAPESGR.C | N | 78.41 | 1149.5237 | 10 | 0.3 | 575.7693 | 2 | 11.37 | 3 | F3:2103 | DaRuMH\_F2.raw | 1.8346E6 |  | 2.7999E5 |  |  |  |  |  |  |  | 2 | 1 | 0 | 1 | 0 | 0 | 0 | 0 | 0 | 0 | 0 | 30 | 39 | Carbamidomethylation | C2:Carbamidomethylation:1000.00 | PEAKS DB |
| G.HDRPTFC(+57.02)NLAPESGR.C | N | 77.03 | 1755.8110 | 15 | 0.4 | 586.2778 | 3 | 11.25 | 5 | F5:2071 | DaRuMH\_F4.raw |  |  |  |  | 2.8813E5 |  |  |  |  |  | 1 | 0 | 0 | 0 | 0 | 1 | 0 | 0 | 0 | 0 | 0 | 25 | 39 | Carbamidomethylation | C7:Carbamidomethylation:1000.00 | PEAKS DB |
| K.VFFYGGC(+57.02)GGNANNFETR.D | Y | 68.97 | 1908.8213 | 17 | 3.5 | 955.4191 | 2 | 13.88 | 7 | F7:4246 | DaRuMH\_F7A.raw |  |  |  |  |  |  | 1.0169E5 |  |  |  | 1 | 0 | 0 | 0 | 0 | 0 | 0 | 1 | 0 | 0 | 0 | 58 | 74 | Carbamidomethylation | C7:Carbamidomethylation:1000.00 | PEAKS DB |
| R.IYYNLESNK.C | N | 56.41 | 1142.5608 | 9 | -0.1 | 572.2876 | 2 | 11.56 | 2 | F2:2417 | DaRuMH\_F10.raw |  | 6.9474E4 |  |  |  |  |  |  |  |  | 1 | 0 | 1 | 0 | 0 | 0 | 0 | 0 | 0 | 0 | 0 | 47 | 55 |  |  | PEAKS DB |
| total 4 peptides |
| --- |

Q7Z794|K2C1B\_HUMAN

back to list

  

| Protein Coverage
| Supporting Peptides
|

Protein Coverage:

Supporting Peptides:

| Peptide | Uniq | -10lgP | Mass | Length | ppm | m/z | z | RT | Fraction | Scan | Source File | Area F1 | Area F10 | Area F2 | Area F3 | Area F4 | Area F5 | Area F7A | Area F7B | Area F8 | Area F9 | #Feature | #Feature F1 | #Feature F10 | #Feature F2 | #Feature F3 | #Feature F4 | #Feature F5 | #Feature F7A | #Feature F7B | #Feature F8 | #Feature F9 | Start | End | PTM | AScore | Found By |
| --- | --- | --- | --- | --- | --- | --- | --- | --- | --- | --- | --- | --- | --- | --- | --- | --- | --- | --- | --- | --- | --- | --- | --- | --- | --- | --- | --- | --- | --- | --- | --- | --- | --- | --- | --- | --- | --- |
| R.FLEQQNQVLQTK.W | N | 86.18 | 1474.7780 | 12 | -1.5 | 738.3951 | 2 | 11.69 | 10 | F10:2507 | DaRuMH\_F9.raw | 1.2606E7 | 4.3269E5 | 3.8807E5 | 1.3371E6 | 1.6646E6 | 5.0153E5 | 4.6914E5 | 3.9811E5 | 4.8981E5 | 3.5935E6 | 10 | 1 | 1 | 1 | 1 | 1 | 1 | 1 | 1 | 1 | 1 | 184 | 195 |  |  | PEAKS DB |
| K.LQDLEEALQQSK.E | Y | 57.77 | 1400.7147 | 12 | 0.2 | 701.3648 | 2 | 12.31 | 1 | F1:3153 | DaRuMH\_F1.raw | 5.2474E4 |  |  |  |  |  |  |  |  |  | 1 | 1 | 0 | 0 | 0 | 0 | 0 | 0 | 0 | 0 | 0 | 428 | 439 |  |  | PEAKS DB |
| total 2 peptides |
| --- |

P25669|3L22\_NAJNA

back to list

  

| Protein Coverage
| Supporting Peptides
|

Protein Coverage:

Supporting Peptides:

| Peptide | Uniq | -10lgP | Mass | Length | ppm | m/z | z | RT | Fraction | Scan | Source File | Area F1 | Area F10 | Area F2 | Area F3 | Area F4 | Area F5 | Area F7A | Area F7B | Area F8 | Area F9 | #Feature | #Feature F1 | #Feature F10 | #Feature F2 | #Feature F3 | #Feature F4 | #Feature F5 | #Feature F7A | #Feature F7B | #Feature F8 | #Feature F9 | Start | End | PTM | AScore | Found By |
| --- | --- | --- | --- | --- | --- | --- | --- | --- | --- | --- | --- | --- | --- | --- | --- | --- | --- | --- | --- | --- | --- | --- | --- | --- | --- | --- | --- | --- | --- | --- | --- | --- | --- | --- | --- | --- | --- |
| R.VDLGC(+57.02)AATC(+57.02)PTVR.T | N | 94.05 | 1418.6646 | 13 | 0.2 | 710.3397 | 2 | 11.62 | 3 | F3:2358 | DaRuMH\_F2.raw |  |  | 2.6643E5 | 2.248E6 | 3.9104E5 |  |  |  |  |  | 3 | 0 | 0 | 1 | 1 | 1 | 0 | 0 | 0 | 0 | 0 | 37 | 49 | Carbamidomethylation | C5:Carbamidomethylation:1000.00;C9:Carbamidomethylation:1000.00 | PEAKS DB |
| R.C(+57.02)FITPDITSK.D | N | 70.72 | 1180.5798 | 10 | 6.3 | 591.2975 | 2 | 12.40 | 4 | F4:3137 | DaRuMH\_F3.raw |  |  | 2.9192E5 | 7.4152E6 | 1.306E6 |  |  |  |  | 4.1739E4 | 4 | 0 | 0 | 1 | 1 | 1 | 0 | 0 | 0 | 0 | 1 | 3 | 12 | Carbamidomethylation | C1:Carbamidomethylation:1000.00 | PEAKS DB |
| K.TWC(+57.02)DGFC(+57.02)SSR.G | Y | 67.36 | 1274.4808 | 10 | 0.7 | 638.2482 | 2 | 11.77 | 3 | F3:2493 | DaRuMH\_F2.raw |  |  | 8.4091E4 | 3.5572E5 |  |  |  |  |  |  | 2 | 0 | 0 | 1 | 1 | 0 | 0 | 0 | 0 | 0 | 0 | 24 | 33 | Carbamidomethylation | C3:Carbamidomethylation:1000.00;C7:Carbamidomethylation:1000.00 | PEAKS DB |
| total 3 peptides |
| --- |

Q6H3C5|PA2BB\_TRIST

back to list

  

| Protein Coverage
| Supporting Peptides
|

Protein Coverage:

Supporting Peptides:

| Peptide | Uniq | -10lgP | Mass | Length | ppm | m/z | z | RT | Fraction | Scan | Source File | Area F1 | Area F10 | Area F2 | Area F3 | Area F4 | Area F5 | Area F7A | Area F7B | Area F8 | Area F9 | #Feature | #Feature F1 | #Feature F10 | #Feature F2 | #Feature F3 | #Feature F4 | #Feature F5 | #Feature F7A | #Feature F7B | #Feature F8 | #Feature F9 | Start | End | PTM | AScore | Found By |
| --- | --- | --- | --- | --- | --- | --- | --- | --- | --- | --- | --- | --- | --- | --- | --- | --- | --- | --- | --- | --- | --- | --- | --- | --- | --- | --- | --- | --- | --- | --- | --- | --- | --- | --- | --- | --- | --- |
| Q.IC(+57.02)EC(+57.02)DKAAAIC(+57.02)FR.E | N | 73.96 | 1612.7159 | 13 | 6.1 | 538.5795 | 3 | 11.48 | 4 | F4:2342 | DaRuMH\_F3.raw |  |  |  | 5.6073E5 | 3.0117E4 |  |  |  |  |  | 2 | 0 | 0 | 0 | 1 | 1 | 0 | 0 | 0 | 0 | 0 | 85 | 97 | Carbamidomethylation | C2:Carbamidomethylation:1000.00;C4:Carbamidomethylation:1000.00;C11:Carbamidomethylation:1000.00 | PEAKS DB |
| K.NPLSSYISYGC(+57.02)YC(+57.02)GWGGQ.G | Y | 65.47 | 2067.8455 | 18 | -8.9 | 1034.9208 | 2 | 14.39 | 6 | F6:4602 | DaRuMH\_F5.raw |  |  |  |  |  | 0 | 0 | 0 |  |  | 0 | 0 | 0 | 0 | 0 | 0 | 0 | 0 | 0 | 0 | 0 | 16 | 33 | Carbamidomethylation | C11:Carbamidomethylation:1000.00;C13:Carbamidomethylation:1000.00 | PEAKS DB |
| total 2 peptides |
| --- |

P25668|3L21\_NAJNA

back to list

  

| Protein Coverage
| Supporting Peptides
|

Protein Coverage:

Supporting Peptides:

| Peptide | Uniq | -10lgP | Mass | Length | ppm | m/z | z | RT | Fraction | Scan | Source File | Area F1 | Area F10 | Area F2 | Area F3 | Area F4 | Area F5 | Area F7A | Area F7B | Area F8 | Area F9 | #Feature | #Feature F1 | #Feature F10 | #Feature F2 | #Feature F3 | #Feature F4 | #Feature F5 | #Feature F7A | #Feature F7B | #Feature F8 | #Feature F9 | Start | End | PTM | AScore | Found By |
| --- | --- | --- | --- | --- | --- | --- | --- | --- | --- | --- | --- | --- | --- | --- | --- | --- | --- | --- | --- | --- | --- | --- | --- | --- | --- | --- | --- | --- | --- | --- | --- | --- | --- | --- | --- | --- | --- |
| R.VDLGC(+57.02)AATC(+57.02)PTVR.T | N | 94.05 | 1418.6646 | 13 | 0.2 | 710.3397 | 2 | 11.62 | 3 | F3:2358 | DaRuMH\_F2.raw |  |  | 2.6643E5 | 2.248E6 | 3.9104E5 |  |  |  |  |  | 3 | 0 | 0 | 1 | 1 | 1 | 0 | 0 | 0 | 0 | 0 | 37 | 49 | Carbamidomethylation | C5:Carbamidomethylation:1000.00;C9:Carbamidomethylation:1000.00 | PEAKS DB |
| R.C(+57.02)FITPDITSK.D | N | 70.72 | 1180.5798 | 10 | 6.3 | 591.2975 | 2 | 12.40 | 4 | F4:3137 | DaRuMH\_F3.raw |  |  | 2.9192E5 | 7.4152E6 | 1.306E6 |  |  |  |  | 4.1739E4 | 4 | 0 | 0 | 1 | 1 | 1 | 0 | 0 | 0 | 0 | 1 | 3 | 12 | Carbamidomethylation | C1:Carbamidomethylation:1000.00 | PEAKS DB |
| K.TWC(+57.02)DGFC(+57.02)SIR.G | Y | 55.37 | 1300.5328 | 10 | 6.5 | 651.2742 | 2 | 13.22 | 4 | F4:3684 | DaRuMH\_F3.raw |  |  |  | 2.0135E6 |  |  |  |  |  |  | 1 | 0 | 0 | 0 | 1 | 0 | 0 | 0 | 0 | 0 | 0 | 24 | 33 | Carbamidomethylation | C3:Carbamidomethylation:1000.00;C7:Carbamidomethylation:1000.00 | PEAKS DB |
| total 3 peptides |
| --- |

O42191|PA2A7\_GLOHA

back to list

  

| Protein Coverage
| Supporting Peptides
|

Protein Coverage:

Supporting Peptides:

| Peptide | Uniq | -10lgP | Mass | Length | ppm | m/z | z | RT | Fraction | Scan | Source File | Area F1 | Area F10 | Area F2 | Area F3 | Area F4 | Area F5 | Area F7A | Area F7B | Area F8 | Area F9 | #Feature | #Feature F1 | #Feature F10 | #Feature F2 | #Feature F3 | #Feature F4 | #Feature F5 | #Feature F7A | #Feature F7B | #Feature F8 | #Feature F9 | Start | End | PTM | AScore | Found By |
| --- | --- | --- | --- | --- | --- | --- | --- | --- | --- | --- | --- | --- | --- | --- | --- | --- | --- | --- | --- | --- | --- | --- | --- | --- | --- | --- | --- | --- | --- | --- | --- | --- | --- | --- | --- | --- | --- |
| Y.C(+57.02)GWGGQGRPQDATDR.C | Y | 66.82 | 1659.7172 | 15 | -6.5 | 830.8605 | 2 | 11.32 | 10 | F10:2158 | DaRuMH\_F9.raw |  |  |  |  |  |  |  |  |  | 6.4196E4 | 1 | 0 | 0 | 0 | 0 | 0 | 0 | 0 | 0 | 0 | 1 | 28 | 42 | Carbamidomethylation | C1:Carbamidomethylation:1000.00 | PEAKS DB |
| total 1 peptides |
| --- |

Q90495|VM3E\_ECHCA

back to list

  

| Protein Coverage
| Supporting Peptides
|

Protein Coverage:

Supporting Peptides:

| Peptide | Uniq | -10lgP | Mass | Length | ppm | m/z | z | RT | Fraction | Scan | Source File | Area F1 | Area F10 | Area F2 | Area F3 | Area F4 | Area F5 | Area F7A | Area F7B | Area F8 | Area F9 | #Feature | #Feature F1 | #Feature F10 | #Feature F2 | #Feature F3 | #Feature F4 | #Feature F5 | #Feature F7A | #Feature F7B | #Feature F8 | #Feature F9 | Start | End | PTM | AScore | Found By |
| --- | --- | --- | --- | --- | --- | --- | --- | --- | --- | --- | --- | --- | --- | --- | --- | --- | --- | --- | --- | --- | --- | --- | --- | --- | --- | --- | --- | --- | --- | --- | --- | --- | --- | --- | --- | --- | --- |
| G.SGNVNDYEVVYPQK.V | N | 80.53 | 1610.7576 | 14 | 1.1 | 806.3870 | 2 | 11.73 | 3 | F3:2451 | DaRuMH\_F2.raw |  |  | 3.1229E5 |  |  |  |  |  |  |  | 1 | 0 | 0 | 1 | 0 | 0 | 0 | 0 | 0 | 0 | 0 | 25 | 38 |  |  | PEAKS DB |
| S.GNVNDYEVVYPQK.V | N | 78.53 | 1523.7256 | 13 | 0.7 | 762.8706 | 2 | 11.75 | 3 | F3:2472 | DaRuMH\_F2.raw |  |  | 1.2901E5 |  |  |  |  |  |  |  | 1 | 0 | 0 | 1 | 0 | 0 | 0 | 0 | 0 | 0 | 0 | 26 | 38 |  |  | PEAKS DB |
| L.GSGNVNDYEVVYPQK.V | Y | 63.76 | 1667.7791 | 15 | 1.2 | 834.8978 | 2 | 11.75 | 3 | F3:2458 | DaRuMH\_F2.raw |  |  | 1.7976E5 |  |  |  |  |  |  |  | 1 | 0 | 0 | 1 | 0 | 0 | 0 | 0 | 0 | 0 | 0 | 24 | 38 |  |  | PEAKS DB |
| total 3 peptides |
| --- |

B6EWW8|V5NTD\_GLOBR

back to list

  

| Protein Coverage
| Supporting Peptides
|

Protein Coverage:

Supporting Peptides:

| Peptide | Uniq | -10lgP | Mass | Length | ppm | m/z | z | RT | Fraction | Scan | Source File | Area F1 | Area F10 | Area F2 | Area F3 | Area F4 | Area F5 | Area F7A | Area F7B | Area F8 | Area F9 | #Feature | #Feature F1 | #Feature F10 | #Feature F2 | #Feature F3 | #Feature F4 | #Feature F5 | #Feature F7A | #Feature F7B | #Feature F8 | #Feature F9 | Start | End | PTM | AScore | Found By |
| --- | --- | --- | --- | --- | --- | --- | --- | --- | --- | --- | --- | --- | --- | --- | --- | --- | --- | --- | --- | --- | --- | --- | --- | --- | --- | --- | --- | --- | --- | --- | --- | --- | --- | --- | --- | --- | --- |
| K.C(+57.02)TGQDC(+57.02)YGGVAR.R | Y | 63.25 | 1342.5394 | 12 | 6.4 | 672.2775 | 2 | 10.91 | 4 | F4:1777 | DaRuMH\_F3.raw |  |  | 1.0261E4 | 1.3715E5 |  |  |  |  |  |  | 2 | 0 | 0 | 1 | 1 | 0 | 0 | 0 | 0 | 0 | 0 | 66 | 77 | Carbamidomethylation | C1:Carbamidomethylation:1000.00;C6:Carbamidomethylation:1000.00 | PEAKS DB |
| G.SFELTILHTNDVHAR.V | Y | 55.42 | 1751.8955 | 15 | 5.5 | 584.9723 | 3 | 12.02 | 4 | F4:2834 | DaRuMH\_F3.raw |  |  |  | 5.2685E4 |  |  |  |  |  |  | 1 | 0 | 0 | 0 | 1 | 0 | 0 | 0 | 0 | 0 | 0 | 41 | 55 |  |  | PEAKS DB |
| total 2 peptides |
| --- |

F8S0Z7|V5NTD\_CROAD

back to list

  

| Protein Coverage
| Supporting Peptides
|

Protein Coverage:

Supporting Peptides:

| Peptide | Uniq | -10lgP | Mass | Length | ppm | m/z | z | RT | Fraction | Scan | Source File | Area F1 | Area F10 | Area F2 | Area F3 | Area F4 | Area F5 | Area F7A | Area F7B | Area F8 | Area F9 | #Feature | #Feature F1 | #Feature F10 | #Feature F2 | #Feature F3 | #Feature F4 | #Feature F5 | #Feature F7A | #Feature F7B | #Feature F8 | #Feature F9 | Start | End | PTM | AScore | Found By |
| --- | --- | --- | --- | --- | --- | --- | --- | --- | --- | --- | --- | --- | --- | --- | --- | --- | --- | --- | --- | --- | --- | --- | --- | --- | --- | --- | --- | --- | --- | --- | --- | --- | --- | --- | --- | --- | --- |
| K.C(+57.02)TGQDC(+57.02)YGGVAR.R | Y | 63.25 | 1342.5394 | 12 | 6.4 | 672.2775 | 2 | 10.91 | 4 | F4:1777 | DaRuMH\_F3.raw |  |  | 1.0261E4 | 1.3715E5 |  |  |  |  |  |  | 2 | 0 | 0 | 1 | 1 | 0 | 0 | 0 | 0 | 0 | 0 | 66 | 77 | Carbamidomethylation | C1:Carbamidomethylation:1000.00;C6:Carbamidomethylation:1000.00 | PEAKS DB |
| G.SFELTILHTNDVHAR.V | Y | 55.42 | 1751.8955 | 15 | 5.5 | 584.9723 | 3 | 12.02 | 4 | F4:2834 | DaRuMH\_F3.raw |  |  |  | 5.2685E4 |  |  |  |  |  |  | 1 | 0 | 0 | 0 | 1 | 0 | 0 | 0 | 0 | 0 | 0 | 41 | 55 |  |  | PEAKS DB |
| total 2 peptides |
| --- |

P24541|VKT\_ERIMA

back to list

  

| Protein Coverage
| Supporting Peptides
|

Protein Coverage:

Supporting Peptides:

| Peptide | Uniq | -10lgP | Mass | Length | ppm | m/z | z | RT | Fraction | Scan | Source File | Area F1 | Area F10 | Area F2 | Area F3 | Area F4 | Area F5 | Area F7A | Area F7B | Area F8 | Area F9 | #Feature | #Feature F1 | #Feature F10 | #Feature F2 | #Feature F3 | #Feature F4 | #Feature F5 | #Feature F7A | #Feature F7B | #Feature F8 | #Feature F9 | Start | End | PTM | AScore | Found By |
| --- | --- | --- | --- | --- | --- | --- | --- | --- | --- | --- | --- | --- | --- | --- | --- | --- | --- | --- | --- | --- | --- | --- | --- | --- | --- | --- | --- | --- | --- | --- | --- | --- | --- | --- | --- | --- | --- |
| F.IYGGC(+57.02)GGNANNFETR.A | N | 88.83 | 1628.7001 | 15 | 1.5 | 815.3585 | 2 | 11.35 | 3 | F3:2069 | DaRuMH\_F2.raw |  |  | 7.886E5 | 1.9848E7 |  |  |  |  |  |  | 2 | 0 | 0 | 1 | 1 | 0 | 0 | 0 | 0 | 0 | 0 | 31 | 45 | Carbamidomethylation | C5:Carbamidomethylation:1000.00 | PEAKS DB |
| K.NFIYGGC(+57.02)GGNANNFETR.A | Y | 56.98 | 1889.8115 | 17 | 7.1 | 945.9144 | 2 | 12.06 | 4 | F4:2864 | DaRuMH\_F3.raw |  |  |  | 7.5031E4 |  |  |  |  |  |  | 1 | 0 | 0 | 0 | 1 | 0 | 0 | 0 | 0 | 0 | 0 | 29 | 45 | Carbamidomethylation | C7:Carbamidomethylation:1000.00 | PEAKS DB |
| total 2 peptides |
| --- |

P15924|DESP\_HUMAN

back to list

  

| Protein Coverage
| Supporting Peptides
|

Protein Coverage:

Supporting Peptides:

| Peptide | Uniq | -10lgP | Mass | Length | ppm | m/z | z | RT | Fraction | Scan | Source File | Area F1 | Area F10 | Area F2 | Area F3 | Area F4 | Area F5 | Area F7A | Area F7B | Area F8 | Area F9 | #Feature | #Feature F1 | #Feature F10 | #Feature F2 | #Feature F3 | #Feature F4 | #Feature F5 | #Feature F7A | #Feature F7B | #Feature F8 | #Feature F9 | Start | End | PTM | AScore | Found By |
| --- | --- | --- | --- | --- | --- | --- | --- | --- | --- | --- | --- | --- | --- | --- | --- | --- | --- | --- | --- | --- | --- | --- | --- | --- | --- | --- | --- | --- | --- | --- | --- | --- | --- | --- | --- | --- | --- |
| R.TM(+15.99)IQSPSGVILQEAADVHAR.Y | Y | 56.02 | 2138.0789 | 20 | 0.1 | 713.7003 | 3 | 13.35 | 1 | F1:3740 | DaRuMH\_F1.raw | 2.2826E4 |  |  |  |  |  |  |  |  |  | 1 | 1 | 0 | 0 | 0 | 0 | 0 | 0 | 0 | 0 | 0 | 983 | 1002 | Oxidation (M) | M2:Oxidation (M):1000.00 | PEAKS DB |
| R.LNDSILQATEQR.R | Y | 54.34 | 1386.7103 | 12 | 0.5 | 694.3628 | 2 | 11.97 | 1 | F1:2821 | DaRuMH\_F1.raw | 6.105E3 |  |  |  |  |  |  |  |  |  | 1 | 1 | 0 | 0 | 0 | 0 | 0 | 0 | 0 | 0 | 0 | 1256 | 1267 |  |  | PEAKS DB |
| total 2 peptides |
| --- |

Q696W1|SLLC2\_MACLB

back to list

  

| Protein Coverage
| Supporting Peptides
|

Protein Coverage:

Supporting Peptides:

| Peptide | Uniq | -10lgP | Mass | Length | ppm | m/z | z | RT | Fraction | Scan | Source File | Area F1 | Area F10 | Area F2 | Area F3 | Area F4 | Area F5 | Area F7A | Area F7B | Area F8 | Area F9 | #Feature | #Feature F1 | #Feature F10 | #Feature F2 | #Feature F3 | #Feature F4 | #Feature F5 | #Feature F7A | #Feature F7B | #Feature F8 | #Feature F9 | Start | End | PTM | AScore | Found By |
| --- | --- | --- | --- | --- | --- | --- | --- | --- | --- | --- | --- | --- | --- | --- | --- | --- | --- | --- | --- | --- | --- | --- | --- | --- | --- | --- | --- | --- | --- | --- | --- | --- | --- | --- | --- | --- | --- |
| G.LDC(+57.02)PPDSSPYR.Y | Y | 69.88 | 1305.5659 | 11 | 0.2 | 653.7903 | 2 | 11.28 | 10 | F10:2119 | DaRuMH\_F9.raw |  |  |  |  |  |  |  |  | 1.6347E5 | 9.6341E5 | 2 | 0 | 0 | 0 | 0 | 0 | 0 | 0 | 0 | 1 | 1 | 25 | 35 | Carbamidomethylation | C3:Carbamidomethylation:1000.00 | PEAKS DB |
| N.C(+57.02)EEPYPFVC(+57.02)K.V | Y | 63.48 | 1327.5576 | 10 | 0.4 | 664.7864 | 2 | 12.01 | 10 | F10:2816 | DaRuMH\_F9.raw |  |  |  | 3.6952E5 |  |  |  |  | 3.5954E5 | 1.2066E6 | 3 | 0 | 0 | 0 | 1 | 0 | 0 | 0 | 0 | 1 | 1 | 144 | 153 | Carbamidomethylation | C1:Carbamidomethylation:1000.00;C9:Carbamidomethylation:1000.00 | PEAKS DB |
| A.GLDC(+57.02)PPDSSPYR.Y | Y | 56.27 | 1362.5874 | 12 | 0.1 | 682.3010 | 2 | 11.42 | 9 | F9:2141 | DaRuMH\_F8.raw |  |  |  |  |  |  |  |  | 2.081E5 |  | 1 | 0 | 0 | 0 | 0 | 0 | 0 | 0 | 0 | 1 | 0 | 24 | 35 | Carbamidomethylation | C4:Carbamidomethylation:1000.00 | PEAKS DB |
| total 3 peptides |
| --- |

Q8N1N4|K2C78\_HUMAN

back to list

  

| Protein Coverage
| Supporting Peptides
|

Protein Coverage:

Supporting Peptides:

| Peptide | Uniq | -10lgP | Mass | Length | ppm | m/z | z | RT | Fraction | Scan | Source File | Area F1 | Area F10 | Area F2 | Area F3 | Area F4 | Area F5 | Area F7A | Area F7B | Area F8 | Area F9 | #Feature | #Feature F1 | #Feature F10 | #Feature F2 | #Feature F3 | #Feature F4 | #Feature F5 | #Feature F7A | #Feature F7B | #Feature F8 | #Feature F9 | Start | End | PTM | AScore | Found By |
| --- | --- | --- | --- | --- | --- | --- | --- | --- | --- | --- | --- | --- | --- | --- | --- | --- | --- | --- | --- | --- | --- | --- | --- | --- | --- | --- | --- | --- | --- | --- | --- | --- | --- | --- | --- | --- | --- |
| R.LLEGEEC(+57.02)R.M | N | 57.87 | 1004.4597 | 8 | 1.1 | 503.2376 | 2 | 10.99 | 2 | F2:1866 | DaRuMH\_F10.raw |  | 1.3429E4 |  |  |  |  |  |  |  |  | 1 | 0 | 1 | 0 | 0 | 0 | 0 | 0 | 0 | 0 | 0 | 416 | 423 | Carbamidomethylation | C7:Carbamidomethylation:1000.00 | PEAKS DB |
| R.YLDFSSIITEVR.A | Y | 55.78 | 1441.7452 | 12 | -0.7 | 721.8794 | 2 | 38.80 | 10 | F10:18963 | DaRuMH\_F9.raw |  |  |  |  |  |  |  |  |  | 3.4036E4 | 1 | 0 | 0 | 0 | 0 | 0 | 0 | 0 | 0 | 0 | 1 | 275 | 286 |  |  | PEAKS DB |
| total 2 peptides |
| --- |

P25428|NGFV\_MACLB

back to list

  

| Protein Coverage
| Supporting Peptides
|

Protein Coverage:

Supporting Peptides:

| Peptide | Uniq | -10lgP | Mass | Length | ppm | m/z | z | RT | Fraction | Scan | Source File | Area F1 | Area F10 | Area F2 | Area F3 | Area F4 | Area F5 | Area F7A | Area F7B | Area F8 | Area F9 | #Feature | #Feature F1 | #Feature F10 | #Feature F2 | #Feature F3 | #Feature F4 | #Feature F5 | #Feature F7A | #Feature F7B | #Feature F8 | #Feature F9 | Start | End | PTM | AScore | Found By |
| --- | --- | --- | --- | --- | --- | --- | --- | --- | --- | --- | --- | --- | --- | --- | --- | --- | --- | --- | --- | --- | --- | --- | --- | --- | --- | --- | --- | --- | --- | --- | --- | --- | --- | --- | --- | --- | --- |
| R.QYFFETK.C | N | 58.15 | 961.4545 | 7 | 0.1 | 481.7346 | 2 | 12.14 | 6 | F6:2899 | DaRuMH\_F5.raw |  |  |  |  |  | 2.9469E5 | 2.6433E5 |  |  |  | 2 | 0 | 0 | 0 | 0 | 0 | 1 | 1 | 0 | 0 | 0 | 175 | 181 |  |  | PEAKS DB |
| R.DEQSVEFLDNADSLNR.N | Y | 55.92 | 1850.8282 | 16 | 0.4 | 926.4218 | 2 | 17.34 | 5 | F5:5058 | DaRuMH\_F4.raw |  |  |  |  | 8.3033E4 |  |  |  |  |  | 1 | 0 | 0 | 0 | 0 | 1 | 0 | 0 | 0 | 0 | 0 | 104 | 119 |  |  | PEAKS DB |
| G.SPATPDLSDTSC(+57.02)AK.T | Y | 54.97 | 1448.6453 | 14 | 0.7 | 725.3304 | 2 | 11.26 | 3 | F3:2019 | DaRuMH\_F2.raw |  |  | 6.9372E4 |  |  |  |  |  |  |  | 1 | 0 | 0 | 1 | 0 | 0 | 0 | 0 | 0 | 0 | 0 | 30 | 43 | Carbamidomethylation | C12:Carbamidomethylation:1000.00 | PEAKS DB |
| total 3 peptides |
| --- |

P81605|DCD\_HUMAN

back to list

  

| Protein Coverage
| Supporting Peptides
|

Protein Coverage:

Supporting Peptides:

| Peptide | Uniq | -10lgP | Mass | Length | ppm | m/z | z | RT | Fraction | Scan | Source File | Area F1 | Area F10 | Area F2 | Area F3 | Area F4 | Area F5 | Area F7A | Area F7B | Area F8 | Area F9 | #Feature | #Feature F1 | #Feature F10 | #Feature F2 | #Feature F3 | #Feature F4 | #Feature F5 | #Feature F7A | #Feature F7B | #Feature F8 | #Feature F9 | Start | End | PTM | AScore | Found By |
| --- | --- | --- | --- | --- | --- | --- | --- | --- | --- | --- | --- | --- | --- | --- | --- | --- | --- | --- | --- | --- | --- | --- | --- | --- | --- | --- | --- | --- | --- | --- | --- | --- | --- | --- | --- | --- | --- |
| K.DAVEDLESVGK.G | Y | 65.76 | 1160.5560 | 11 | 2.8 | 581.2856 | 2 | 12.41 | 7 | F7:3085 | DaRuMH\_F7A.raw | 5.9124E4 |  |  |  | 6.8641E4 |  | 2.8742E4 | 0 |  | 6.2008E4 | 4 | 1 | 0 | 0 | 0 | 1 | 0 | 1 | 0 | 0 | 1 | 86 | 96 |  |  | PEAKS DB |
| K.ENAGEDPGLAR.Q | Y | 58.25 | 1127.5206 | 11 | 1.0 | 564.7681 | 2 | 10.98 | 3 | F3:1738 | DaRuMH\_F2.raw |  | 1.425E4 | 2.5225E4 |  | 5.9069E4 |  |  |  |  |  | 3 | 0 | 1 | 1 | 0 | 1 | 0 | 0 | 0 | 0 | 0 | 43 | 53 |  |  | PEAKS DB |
| A.YDPEAASAPGSGNPC(+57.02)HEASAAQK.E | Y | 54.72 | 2313.9919 | 23 | 6.3 | 772.3384 | 3 | 10.97 | 4 | F4:1849 | DaRuMH\_F3.raw |  |  |  | 9.4139E4 |  |  |  |  |  |  | 1 | 0 | 0 | 0 | 1 | 0 | 0 | 0 | 0 | 0 | 0 | 20 | 42 | Carbamidomethylation | C15:Carbamidomethylation:1000.00 | PEAKS DB |
| total 3 peptides |
| --- |

Q8SPJ1|PLAK\_BOVIN

back to list

  

| Protein Coverage
| Supporting Peptides
|

Protein Coverage:

Supporting Peptides:

| Peptide | Uniq | -10lgP | Mass | Length | ppm | m/z | z | RT | Fraction | Scan | Source File | Area F1 | Area F10 | Area F2 | Area F3 | Area F4 | Area F5 | Area F7A | Area F7B | Area F8 | Area F9 | #Feature | #Feature F1 | #Feature F10 | #Feature F2 | #Feature F3 | #Feature F4 | #Feature F5 | #Feature F7A | #Feature F7B | #Feature F8 | #Feature F9 | Start | End | PTM | AScore | Found By |
| --- | --- | --- | --- | --- | --- | --- | --- | --- | --- | --- | --- | --- | --- | --- | --- | --- | --- | --- | --- | --- | --- | --- | --- | --- | --- | --- | --- | --- | --- | --- | --- | --- | --- | --- | --- | --- | --- |
| R.VSVELTNSLFK.H | Y | 60.97 | 1235.6761 | 11 | 0.5 | 618.8456 | 2 | 17.80 | 1 | F1:5530 | DaRuMH\_F1.raw | 2.2708E4 |  |  |  |  |  |  |  |  |  | 1 | 1 | 0 | 0 | 0 | 0 | 0 | 0 | 0 | 0 | 0 | 664 | 674 |  |  | PEAKS DB |
| total 1 peptides |
| --- |

Q02257|PLAK\_MOUSE

back to list

  

| Protein Coverage
| Supporting Peptides
|

Protein Coverage:

Supporting Peptides:

| Peptide | Uniq | -10lgP | Mass | Length | ppm | m/z | z | RT | Fraction | Scan | Source File | Area F1 | Area F10 | Area F2 | Area F3 | Area F4 | Area F5 | Area F7A | Area F7B | Area F8 | Area F9 | #Feature | #Feature F1 | #Feature F10 | #Feature F2 | #Feature F3 | #Feature F4 | #Feature F5 | #Feature F7A | #Feature F7B | #Feature F8 | #Feature F9 | Start | End | PTM | AScore | Found By |
| --- | --- | --- | --- | --- | --- | --- | --- | --- | --- | --- | --- | --- | --- | --- | --- | --- | --- | --- | --- | --- | --- | --- | --- | --- | --- | --- | --- | --- | --- | --- | --- | --- | --- | --- | --- | --- | --- |
| R.VSVELTNSLFK.H | Y | 60.97 | 1235.6761 | 11 | 0.5 | 618.8456 | 2 | 17.80 | 1 | F1:5530 | DaRuMH\_F1.raw | 2.2708E4 |  |  |  |  |  |  |  |  |  | 1 | 1 | 0 | 0 | 0 | 0 | 0 | 0 | 0 | 0 | 0 | 664 | 674 |  |  | PEAKS DB |
| total 1 peptides |
| --- |

P14923|PLAK\_HUMAN

back to list

  

| Protein Coverage
| Supporting Peptides
|

Protein Coverage:

Supporting Peptides:

| Peptide | Uniq | -10lgP | Mass | Length | ppm | m/z | z | RT | Fraction | Scan | Source File | Area F1 | Area F10 | Area F2 | Area F3 | Area F4 | Area F5 | Area F7A | Area F7B | Area F8 | Area F9 | #Feature | #Feature F1 | #Feature F10 | #Feature F2 | #Feature F3 | #Feature F4 | #Feature F5 | #Feature F7A | #Feature F7B | #Feature F8 | #Feature F9 | Start | End | PTM | AScore | Found By |
| --- | --- | --- | --- | --- | --- | --- | --- | --- | --- | --- | --- | --- | --- | --- | --- | --- | --- | --- | --- | --- | --- | --- | --- | --- | --- | --- | --- | --- | --- | --- | --- | --- | --- | --- | --- | --- | --- |
| R.VSVELTNSLFK.H | Y | 60.97 | 1235.6761 | 11 | 0.5 | 618.8456 | 2 | 17.80 | 1 | F1:5530 | DaRuMH\_F1.raw | 2.2708E4 |  |  |  |  |  |  |  |  |  | 1 | 1 | 0 | 0 | 0 | 0 | 0 | 0 | 0 | 0 | 0 | 664 | 674 |  |  | PEAKS DB |
| total 1 peptides |
| --- |

Q8JIR2|VM3HA\_PROFL

back to list

  

| Protein Coverage
| Supporting Peptides
|

Protein Coverage:

Supporting Peptides:

| Peptide | Uniq | -10lgP | Mass | Length | ppm | m/z | z | RT | Fraction | Scan | Source File | Area F1 | Area F10 | Area F2 | Area F3 | Area F4 | Area F5 | Area F7A | Area F7B | Area F8 | Area F9 | #Feature | #Feature F1 | #Feature F10 | #Feature F2 | #Feature F3 | #Feature F4 | #Feature F5 | #Feature F7A | #Feature F7B | #Feature F8 | #Feature F9 | Start | End | PTM | AScore | Found By |
| --- | --- | --- | --- | --- | --- | --- | --- | --- | --- | --- | --- | --- | --- | --- | --- | --- | --- | --- | --- | --- | --- | --- | --- | --- | --- | --- | --- | --- | --- | --- | --- | --- | --- | --- | --- | --- | --- |
| V.TQTNWESDEPIK.K | N | 62.34 | 1446.6627 | 12 | 0.2 | 724.3387 | 2 | 11.62 | 2 | F2:2463 | DaRuMH\_F10.raw |  | 8.3861E4 |  |  |  |  |  |  |  |  | 1 | 0 | 1 | 0 | 0 | 0 | 0 | 0 | 0 | 0 | 0 | 170 | 181 |  |  | PEAKS DB |
| N.SC(+57.02)IMSAVISDQPSK.Y | Y | 56.11 | 1521.7167 | 14 | 0.5 | 761.8660 | 2 | 13.20 | 2 | F2:3481 | DaRuMH\_F10.raw |  | 1.6367E5 |  |  |  |  |  |  |  |  | 1 | 0 | 1 | 0 | 0 | 0 | 0 | 0 | 0 | 0 | 0 | 357 | 370 | Carbamidomethylation | C2:Carbamidomethylation:1000.00 | PEAKS DB |
| V.TQTNWESDEPIKK.A | N | 54.81 | 1574.7576 | 13 | 0.6 | 525.9268 | 3 | 11.17 | 2 | F2:2030 | DaRuMH\_F10.raw |  | 1.6419E5 |  |  |  |  |  |  |  |  | 1 | 0 | 1 | 0 | 0 | 0 | 0 | 0 | 0 | 0 | 0 | 170 | 182 |  |  | PEAKS DB |
| total 3 peptides |
| --- |

Q2ES49|VKT2\_DABRR

back to list

  

| Protein Coverage
| Supporting Peptides
|

Protein Coverage:

Supporting Peptides:

| Peptide | Uniq | -10lgP | Mass | Length | ppm | m/z | z | RT | Fraction | Scan | Source File | Area F1 | Area F10 | Area F2 | Area F3 | Area F4 | Area F5 | Area F7A | Area F7B | Area F8 | Area F9 | #Feature | #Feature F1 | #Feature F10 | #Feature F2 | #Feature F3 | #Feature F4 | #Feature F5 | #Feature F7A | #Feature F7B | #Feature F8 | #Feature F9 | Start | End | PTM | AScore | Found By |
| --- | --- | --- | --- | --- | --- | --- | --- | --- | --- | --- | --- | --- | --- | --- | --- | --- | --- | --- | --- | --- | --- | --- | --- | --- | --- | --- | --- | --- | --- | --- | --- | --- | --- | --- | --- | --- | --- |
| R.FYYNPATNQC(+57.02)QGF.L | Y | 60.72 | 1608.6667 | 13 | 0.7 | 805.3412 | 2 | 19.81 | 5 | F5:5857 | DaRuMH\_F4.raw |  |  |  |  | 2.0448E7 |  |  |  |  |  | 1 | 0 | 0 | 0 | 0 | 1 | 0 | 0 | 0 | 0 | 0 | 47 | 59 | Carbamidomethylation | C10:Carbamidomethylation:1000.00 | PEAKS DB |
| K.FC(+57.02)FLRPDFGR.Y | Y | 59.18 | 1313.6339 | 10 | -0.1 | 438.8852 | 3 | 16.70 | 5 | F5:4867 | DaRuMH\_F4.raw |  |  |  |  | 1.5656E7 |  |  |  |  |  | 1 | 0 | 0 | 0 | 0 | 1 | 0 | 0 | 0 | 0 | 0 | 30 | 39 | Carbamidomethylation | C2:Carbamidomethylation:1000.00 | PEAKS DB |
| total 2 peptides |
| --- |

Q4PRD1|SLLC1\_DABSI

back to list

  

| Protein Coverage
| Supporting Peptides
|

Protein Coverage:

Supporting Peptides:

| Peptide | Uniq | -10lgP | Mass | Length | ppm | m/z | z | RT | Fraction | Scan | Source File | Area F1 | Area F10 | Area F2 | Area F3 | Area F4 | Area F5 | Area F7A | Area F7B | Area F8 | Area F9 | #Feature | #Feature F1 | #Feature F10 | #Feature F2 | #Feature F3 | #Feature F4 | #Feature F5 | #Feature F7A | #Feature F7B | #Feature F8 | #Feature F9 | Start | End | PTM | AScore | Found By |
| --- | --- | --- | --- | --- | --- | --- | --- | --- | --- | --- | --- | --- | --- | --- | --- | --- | --- | --- | --- | --- | --- | --- | --- | --- | --- | --- | --- | --- | --- | --- | --- | --- | --- | --- | --- | --- | --- |
| A.VLDC(+57.02)PSGWLSYEQHC(+57.02)YK.G | Y | 70.90 | 2140.9346 | 17 | 0.3 | 714.6523 | 3 | 13.42 | 9 | F9:3859 | DaRuMH\_F8.raw |  |  |  |  |  |  |  |  | 4.9355E5 |  | 1 | 0 | 0 | 0 | 0 | 0 | 0 | 0 | 0 | 1 | 0 | 24 | 40 | Carbamidomethylation | C4:Carbamidomethylation:1000.00;C15:Carbamidomethylation:1000.00 | PEAKS DB |
| total 1 peptides |
| --- |

P0DL42|TXVE\_DABSI

back to list

  

| Protein Coverage
| Supporting Peptides
|

Protein Coverage:

Supporting Peptides:

| Peptide | Uniq | -10lgP | Mass | Length | ppm | m/z | z | RT | Fraction | Scan | Source File | Area F1 | Area F10 | Area F2 | Area F3 | Area F4 | Area F5 | Area F7A | Area F7B | Area F8 | Area F9 | #Feature | #Feature F1 | #Feature F10 | #Feature F2 | #Feature F3 | #Feature F4 | #Feature F5 | #Feature F7A | #Feature F7B | #Feature F8 | #Feature F9 | Start | End | PTM | AScore | Found By |
| --- | --- | --- | --- | --- | --- | --- | --- | --- | --- | --- | --- | --- | --- | --- | --- | --- | --- | --- | --- | --- | --- | --- | --- | --- | --- | --- | --- | --- | --- | --- | --- | --- | --- | --- | --- | --- | --- |
| R.ETLVSILQEHPDEISDIFR.P | Y | 76.93 | 2240.1323 | 19 | 0.6 | 747.7185 | 3 | 40.99 | 8 | F8:21852 | DaRuMH\_F7B.raw |  |  |  |  |  |  |  | 1.723E5 |  |  | 1 | 0 | 0 | 0 | 0 | 0 | 0 | 0 | 1 | 0 | 0 | 18 | 36 |  |  | PEAKS DB |
| total 1 peptides |
| --- |

P67861|TXVE\_DABRR

back to list

  

| Protein Coverage
| Supporting Peptides
|

Protein Coverage:

Supporting Peptides:

| Peptide | Uniq | -10lgP | Mass | Length | ppm | m/z | z | RT | Fraction | Scan | Source File | Area F1 | Area F10 | Area F2 | Area F3 | Area F4 | Area F5 | Area F7A | Area F7B | Area F8 | Area F9 | #Feature | #Feature F1 | #Feature F10 | #Feature F2 | #Feature F3 | #Feature F4 | #Feature F5 | #Feature F7A | #Feature F7B | #Feature F8 | #Feature F9 | Start | End | PTM | AScore | Found By |
| --- | --- | --- | --- | --- | --- | --- | --- | --- | --- | --- | --- | --- | --- | --- | --- | --- | --- | --- | --- | --- | --- | --- | --- | --- | --- | --- | --- | --- | --- | --- | --- | --- | --- | --- | --- | --- | --- |
| R.ETLVSILQEHPDEISDIFR.P | Y | 76.93 | 2240.1323 | 19 | 0.6 | 747.7185 | 3 | 40.99 | 8 | F8:21852 | DaRuMH\_F7B.raw |  |  |  |  |  |  |  | 1.723E5 |  |  | 1 | 0 | 0 | 0 | 0 | 0 | 0 | 0 | 1 | 0 | 0 | 42 | 60 |  |  | PEAKS DB |
| total 1 peptides |
| --- |

P30894|NGFV\_DABRR

back to list

  

| Protein Coverage
| Supporting Peptides
|

Protein Coverage:

Supporting Peptides:

| Peptide | Uniq | -10lgP | Mass | Length | ppm | m/z | z | RT | Fraction | Scan | Source File | Area F1 | Area F10 | Area F2 | Area F3 | Area F4 | Area F5 | Area F7A | Area F7B | Area F8 | Area F9 | #Feature | #Feature F1 | #Feature F10 | #Feature F2 | #Feature F3 | #Feature F4 | #Feature F5 | #Feature F7A | #Feature F7B | #Feature F8 | #Feature F9 | Start | End | PTM | AScore | Found By |
| --- | --- | --- | --- | --- | --- | --- | --- | --- | --- | --- | --- | --- | --- | --- | --- | --- | --- | --- | --- | --- | --- | --- | --- | --- | --- | --- | --- | --- | --- | --- | --- | --- | --- | --- | --- | --- | --- |
| R.INTAC(+57.02)VC(+57.02)VISR.K | Y | 65.84 | 1291.6377 | 11 | -0.3 | 646.8259 | 2 | 11.69 | 8 | F8:2582 | DaRuMH\_F7B.raw |  |  |  |  |  | 1.6231E5 |  | 1.3202E5 |  |  | 2 | 0 | 0 | 0 | 0 | 0 | 1 | 0 | 1 | 0 | 0 | 101 | 111 | Carbamidomethylation | C5:Carbamidomethylation:1000.00;C7:Carbamidomethylation:1000.00 | PEAKS DB |
| K.QYFFETK.C | N | 58.15 | 961.4545 | 7 | 0.1 | 481.7346 | 2 | 12.14 | 6 | F6:2899 | DaRuMH\_F5.raw |  |  |  |  |  | 2.9469E5 | 2.6433E5 |  |  |  | 2 | 0 | 0 | 0 | 0 | 0 | 1 | 1 | 0 | 0 | 0 | 48 | 54 |  |  | PEAKS DB |
| total 2 peptides |
| --- |

B4XSY8|SLAD\_MACLB

back to list

  

| Protein Coverage
| Supporting Peptides
|

Protein Coverage:

Supporting Peptides:

| Peptide | Uniq | -10lgP | Mass | Length | ppm | m/z | z | RT | Fraction | Scan | Source File | Area F1 | Area F10 | Area F2 | Area F3 | Area F4 | Area F5 | Area F7A | Area F7B | Area F8 | Area F9 | #Feature | #Feature F1 | #Feature F10 | #Feature F2 | #Feature F3 | #Feature F4 | #Feature F5 | #Feature F7A | #Feature F7B | #Feature F8 | #Feature F9 | Start | End | PTM | AScore | Found By |
| --- | --- | --- | --- | --- | --- | --- | --- | --- | --- | --- | --- | --- | --- | --- | --- | --- | --- | --- | --- | --- | --- | --- | --- | --- | --- | --- | --- | --- | --- | --- | --- | --- | --- | --- | --- | --- | --- |
| K.HLATIEWLGK.A | Y | 79.71 | 1166.6448 | 10 | 0.2 | 584.3298 | 2 | 12.26 | 10 | F10:3024 | DaRuMH\_F9.raw |  |  |  |  |  |  |  |  | 2.1987E4 | 2.4998E6 | 2 | 0 | 0 | 0 | 0 | 0 | 0 | 0 | 0 | 1 | 1 | 40 | 49 |  |  | PEAKS DB |
| K.ANFVAELVTLMK.L | Y | 57.86 | 1334.7268 | 12 | 0.3 | 668.3708 | 2 | 51.14 | 10 | F10:24338 | DaRuMH\_F9.raw |  |  |  |  |  |  |  |  |  | 2.8472E5 | 1 | 0 | 0 | 0 | 0 | 0 | 0 | 0 | 0 | 0 | 1 | 50 | 61 |  |  | PEAKS DB |
| total 2 peptides |
| --- |

B4XSY7|SLAC\_MACLB

back to list

  

| Protein Coverage
| Supporting Peptides
|

Protein Coverage:

Supporting Peptides:

| Peptide | Uniq | -10lgP | Mass | Length | ppm | m/z | z | RT | Fraction | Scan | Source File | Area F1 | Area F10 | Area F2 | Area F3 | Area F4 | Area F5 | Area F7A | Area F7B | Area F8 | Area F9 | #Feature | #Feature F1 | #Feature F10 | #Feature F2 | #Feature F3 | #Feature F4 | #Feature F5 | #Feature F7A | #Feature F7B | #Feature F8 | #Feature F9 | Start | End | PTM | AScore | Found By |
| --- | --- | --- | --- | --- | --- | --- | --- | --- | --- | --- | --- | --- | --- | --- | --- | --- | --- | --- | --- | --- | --- | --- | --- | --- | --- | --- | --- | --- | --- | --- | --- | --- | --- | --- | --- | --- | --- |
| K.HLATIEWLGK.A | Y | 79.71 | 1166.6448 | 10 | 0.2 | 584.3298 | 2 | 12.26 | 10 | F10:3024 | DaRuMH\_F9.raw |  |  |  |  |  |  |  |  | 2.1987E4 | 2.4998E6 | 2 | 0 | 0 | 0 | 0 | 0 | 0 | 0 | 0 | 1 | 1 | 63 | 72 |  |  | PEAKS DB |
| K.ANFVAELVTLMK.L | Y | 57.86 | 1334.7268 | 12 | 0.3 | 668.3708 | 2 | 51.14 | 10 | F10:24338 | DaRuMH\_F9.raw |  |  |  |  |  |  |  |  |  | 2.8472E5 | 1 | 0 | 0 | 0 | 0 | 0 | 0 | 0 | 0 | 0 | 1 | 73 | 84 |  |  | PEAKS DB |
| total 2 peptides |
| --- |

W5XCJ6|SLCIB\_MACLB

back to list

  

| Protein Coverage
| Supporting Peptides
|

Protein Coverage:

Supporting Peptides:

| Peptide | Uniq | -10lgP | Mass | Length | ppm | m/z | z | RT | Fraction | Scan | Source File | Area F1 | Area F10 | Area F2 | Area F3 | Area F4 | Area F5 | Area F7A | Area F7B | Area F8 | Area F9 | #Feature | #Feature F1 | #Feature F10 | #Feature F2 | #Feature F3 | #Feature F4 | #Feature F5 | #Feature F7A | #Feature F7B | #Feature F8 | #Feature F9 | Start | End | PTM | AScore | Found By |
| --- | --- | --- | --- | --- | --- | --- | --- | --- | --- | --- | --- | --- | --- | --- | --- | --- | --- | --- | --- | --- | --- | --- | --- | --- | --- | --- | --- | --- | --- | --- | --- | --- | --- | --- | --- | --- | --- |
| F.NLSC(+57.02)GDDYPFVC(+57.02)K.S | Y | 53.79 | 1573.6541 | 13 | 1.9 | 787.8358 | 2 | 12.33 | 10 | F10:3108 | DaRuMH\_F9.raw |  |  |  |  |  |  |  |  |  | 1.9931E5 | 1 | 0 | 0 | 0 | 0 | 0 | 0 | 0 | 0 | 0 | 1 | 139 | 151 | Carbamidomethylation | C4:Carbamidomethylation:1000.00;C12:Carbamidomethylation:1000.00 | PEAKS DB |
| total 1 peptides |
| --- |

B4XSZ0|SLAF\_MACLB

back to list

  

| Protein Coverage
| Supporting Peptides
|

Protein Coverage:

Supporting Peptides:

| Peptide | Uniq | -10lgP | Mass | Length | ppm | m/z | z | RT | Fraction | Scan | Source File | Area F1 | Area F10 | Area F2 | Area F3 | Area F4 | Area F5 | Area F7A | Area F7B | Area F8 | Area F9 | #Feature | #Feature F1 | #Feature F10 | #Feature F2 | #Feature F3 | #Feature F4 | #Feature F5 | #Feature F7A | #Feature F7B | #Feature F8 | #Feature F9 | Start | End | PTM | AScore | Found By |
| --- | --- | --- | --- | --- | --- | --- | --- | --- | --- | --- | --- | --- | --- | --- | --- | --- | --- | --- | --- | --- | --- | --- | --- | --- | --- | --- | --- | --- | --- | --- | --- | --- | --- | --- | --- | --- | --- |
| F.NLSC(+57.02)GDDYPFVC(+57.02)K.S | Y | 53.79 | 1573.6541 | 13 | 1.9 | 787.8358 | 2 | 12.33 | 10 | F10:3108 | DaRuMH\_F9.raw |  |  |  |  |  |  |  |  |  | 1.9931E5 | 1 | 0 | 0 | 0 | 0 | 0 | 0 | 0 | 0 | 0 | 1 | 141 | 153 | Carbamidomethylation | C4:Carbamidomethylation:1000.00;C12:Carbamidomethylation:1000.00 | PEAKS DB |
| total 1 peptides |
| --- |

B4XSY9|SLAE\_MACLB

back to list

  

| Protein Coverage
| Supporting Peptides
|

Protein Coverage:

Supporting Peptides:

| Peptide | Uniq | -10lgP | Mass | Length | ppm | m/z | z | RT | Fraction | Scan | Source File | Area F1 | Area F10 | Area F2 | Area F3 | Area F4 | Area F5 | Area F7A | Area F7B | Area F8 | Area F9 | #Feature | #Feature F1 | #Feature F10 | #Feature F2 | #Feature F3 | #Feature F4 | #Feature F5 | #Feature F7A | #Feature F7B | #Feature F8 | #Feature F9 | Start | End | PTM | AScore | Found By |
| --- | --- | --- | --- | --- | --- | --- | --- | --- | --- | --- | --- | --- | --- | --- | --- | --- | --- | --- | --- | --- | --- | --- | --- | --- | --- | --- | --- | --- | --- | --- | --- | --- | --- | --- | --- | --- | --- |
| F.NLSC(+57.02)GDDYPFVC(+57.02)K.S | Y | 53.79 | 1573.6541 | 13 | 1.9 | 787.8358 | 2 | 12.33 | 10 | F10:3108 | DaRuMH\_F9.raw |  |  |  |  |  |  |  |  |  | 1.9931E5 | 1 | 0 | 0 | 0 | 0 | 0 | 0 | 0 | 0 | 0 | 1 | 141 | 153 | Carbamidomethylation | C4:Carbamidomethylation:1000.00;C12:Carbamidomethylation:1000.00 | PEAKS DB |
| total 1 peptides |
| --- |

B4XSY5|SLAA\_MACLB

back to list

  

| Protein Coverage
| Supporting Peptides
|

Protein Coverage:

Supporting Peptides:

| Peptide | Uniq | -10lgP | Mass | Length | ppm | m/z | z | RT | Fraction | Scan | Source File | Area F1 | Area F10 | Area F2 | Area F3 | Area F4 | Area F5 | Area F7A | Area F7B | Area F8 | Area F9 | #Feature | #Feature F1 | #Feature F10 | #Feature F2 | #Feature F3 | #Feature F4 | #Feature F5 | #Feature F7A | #Feature F7B | #Feature F8 | #Feature F9 | Start | End | PTM | AScore | Found By |
| --- | --- | --- | --- | --- | --- | --- | --- | --- | --- | --- | --- | --- | --- | --- | --- | --- | --- | --- | --- | --- | --- | --- | --- | --- | --- | --- | --- | --- | --- | --- | --- | --- | --- | --- | --- | --- | --- |
| H.C(+57.02)YQAVDEPK.S | Y | 71.95 | 1108.4858 | 9 | 1.2 | 555.2509 | 2 | 10.95 | 10 | F10:1804 | DaRuMH\_F9.raw |  |  |  |  |  |  |  |  |  | 2.3905E5 | 1 | 0 | 0 | 0 | 0 | 0 | 0 | 0 | 0 | 0 | 1 | 38 | 46 | Carbamidomethylation | C1:Carbamidomethylation:1000.00 | PEAKS DB |
| total 1 peptides |
| --- |

B4XSY6|SLAB\_MACLB

back to list

  

| Protein Coverage
| Supporting Peptides
|

Protein Coverage:

Supporting Peptides:

| Peptide | Uniq | -10lgP | Mass | Length | ppm | m/z | z | RT | Fraction | Scan | Source File | Area F1 | Area F10 | Area F2 | Area F3 | Area F4 | Area F5 | Area F7A | Area F7B | Area F8 | Area F9 | #Feature | #Feature F1 | #Feature F10 | #Feature F2 | #Feature F3 | #Feature F4 | #Feature F5 | #Feature F7A | #Feature F7B | #Feature F8 | #Feature F9 | Start | End | PTM | AScore | Found By |
| --- | --- | --- | --- | --- | --- | --- | --- | --- | --- | --- | --- | --- | --- | --- | --- | --- | --- | --- | --- | --- | --- | --- | --- | --- | --- | --- | --- | --- | --- | --- | --- | --- | --- | --- | --- | --- | --- |
| H.C(+57.02)YQAVDEPK.S | Y | 71.95 | 1108.4858 | 9 | 1.2 | 555.2509 | 2 | 10.95 | 10 | F10:1804 | DaRuMH\_F9.raw |  |  |  |  |  |  |  |  |  | 2.3905E5 | 1 | 0 | 0 | 0 | 0 | 0 | 0 | 0 | 0 | 0 | 1 | 38 | 46 | Carbamidomethylation | C1:Carbamidomethylation:1000.00 | PEAKS DB |
| total 1 peptides |
| --- |

P86382|3SA7\_NAJNA

back to list

  

| Protein Coverage
| Supporting Peptides
|

Protein Coverage:

Supporting Peptides:

| Peptide | Uniq | -10lgP | Mass | Length | ppm | m/z | z | RT | Fraction | Scan | Source File | Area F1 | Area F10 | Area F2 | Area F3 | Area F4 | Area F5 | Area F7A | Area F7B | Area F8 | Area F9 | #Feature | #Feature F1 | #Feature F10 | #Feature F2 | #Feature F3 | #Feature F4 | #Feature F5 | #Feature F7A | #Feature F7B | #Feature F8 | #Feature F9 | Start | End | PTM | AScore | Found By |
| --- | --- | --- | --- | --- | --- | --- | --- | --- | --- | --- | --- | --- | --- | --- | --- | --- | --- | --- | --- | --- | --- | --- | --- | --- | --- | --- | --- | --- | --- | --- | --- | --- | --- | --- | --- | --- | --- |
| R.GC(+57.02)IDVC(+57.02)PK.N | Y | 54.49 | 947.4205 | 8 | 0.8 | 474.7179 | 2 | 11.17 | 10 | F10:2015 | DaRuMH\_F9.raw |  |  |  |  |  |  |  |  |  | 5.1097E5 | 1 | 0 | 0 | 0 | 0 | 0 | 0 | 0 | 0 | 0 | 1 | 37 | 44 | Carbamidomethylation | C2:Carbamidomethylation:1000.00;C6:Carbamidomethylation:1000.00 | PEAKS DB |
| total 1 peptides |
| --- |

P01445|3SA7A\_NAJKA

back to list

  

| Protein Coverage
| Supporting Peptides
|

Protein Coverage:

Supporting Peptides:

| Peptide | Uniq | -10lgP | Mass | Length | ppm | m/z | z | RT | Fraction | Scan | Source File | Area F1 | Area F10 | Area F2 | Area F3 | Area F4 | Area F5 | Area F7A | Area F7B | Area F8 | Area F9 | #Feature | #Feature F1 | #Feature F10 | #Feature F2 | #Feature F3 | #Feature F4 | #Feature F5 | #Feature F7A | #Feature F7B | #Feature F8 | #Feature F9 | Start | End | PTM | AScore | Found By |
| --- | --- | --- | --- | --- | --- | --- | --- | --- | --- | --- | --- | --- | --- | --- | --- | --- | --- | --- | --- | --- | --- | --- | --- | --- | --- | --- | --- | --- | --- | --- | --- | --- | --- | --- | --- | --- | --- |
| R.GC(+57.02)IDVC(+57.02)PK.N | Y | 54.49 | 947.4205 | 8 | 0.8 | 474.7179 | 2 | 11.17 | 10 | F10:2015 | DaRuMH\_F9.raw |  |  |  |  |  |  |  |  |  | 5.1097E5 | 1 | 0 | 0 | 0 | 0 | 0 | 0 | 0 | 0 | 0 | 1 | 37 | 44 | Carbamidomethylation | C2:Carbamidomethylation:1000.00;C6:Carbamidomethylation:1000.00 | PEAKS DB |
| total 1 peptides |
| --- |

P86540|3SA8\_NAJNA

back to list

  

| Protein Coverage
| Supporting Peptides
|

Protein Coverage:

Supporting Peptides:

| Peptide | Uniq | -10lgP | Mass | Length | ppm | m/z | z | RT | Fraction | Scan | Source File | Area F1 | Area F10 | Area F2 | Area F3 | Area F4 | Area F5 | Area F7A | Area F7B | Area F8 | Area F9 | #Feature | #Feature F1 | #Feature F10 | #Feature F2 | #Feature F3 | #Feature F4 | #Feature F5 | #Feature F7A | #Feature F7B | #Feature F8 | #Feature F9 | Start | End | PTM | AScore | Found By |
| --- | --- | --- | --- | --- | --- | --- | --- | --- | --- | --- | --- | --- | --- | --- | --- | --- | --- | --- | --- | --- | --- | --- | --- | --- | --- | --- | --- | --- | --- | --- | --- | --- | --- | --- | --- | --- | --- |
| R.GC(+57.02)IDVC(+57.02)PK.N | Y | 54.49 | 947.4205 | 8 | 0.8 | 474.7179 | 2 | 11.17 | 10 | F10:2015 | DaRuMH\_F9.raw |  |  |  |  |  |  |  |  |  | 5.1097E5 | 1 | 0 | 0 | 0 | 0 | 0 | 0 | 0 | 0 | 0 | 1 | 37 | 44 | Carbamidomethylation | C2:Carbamidomethylation:1000.00;C6:Carbamidomethylation:1000.00 | PEAKS DB |
| total 1 peptides |
| --- |

C0HJL8|PA2B\_BOTNI

back to list

  

| Protein Coverage
| Supporting Peptides
|

Protein Coverage:

Supporting Peptides:

| Peptide | Uniq | -10lgP | Mass | Length | ppm | m/z | z | RT | Fraction | Scan | Source File | Area F1 | Area F10 | Area F2 | Area F3 | Area F4 | Area F5 | Area F7A | Area F7B | Area F8 | Area F9 | #Feature | #Feature F1 | #Feature F10 | #Feature F2 | #Feature F3 | #Feature F4 | #Feature F5 | #Feature F7A | #Feature F7B | #Feature F8 | #Feature F9 | Start | End | PTM | AScore | Found By |
| --- | --- | --- | --- | --- | --- | --- | --- | --- | --- | --- | --- | --- | --- | --- | --- | --- | --- | --- | --- | --- | --- | --- | --- | --- | --- | --- | --- | --- | --- | --- | --- | --- | --- | --- | --- | --- | --- |
| K.NAVPFYAFYGC(+57.02)YC(+57.02)GWGGQGQPKDATDR.C | Y | 54.50 | 3084.3281 | 27 | -0.2 | 1029.1165 | 3 | 12.30 | 10 | F10:3086 | DaRuMH\_F9.raw |  |  |  |  |  |  |  |  |  | 3.8023E5 | 1 | 0 | 0 | 0 | 0 | 0 | 0 | 0 | 0 | 0 | 1 | 16 | 42 | Carbamidomethylation | C11:Carbamidomethylation:1000.00;C13:Carbamidomethylation:1000.00 | PEAKS DB |
| total 1 peptides |
| --- |

Q56EB1|SLAA\_BOTJA

back to list

  

| Protein Coverage
| Supporting Peptides
|

Protein Coverage:

Supporting Peptides:

| Peptide | Uniq | -10lgP | Mass | Length | ppm | m/z | z | RT | Fraction | Scan | Source File | Area F1 | Area F10 | Area F2 | Area F3 | Area F4 | Area F5 | Area F7A | Area F7B | Area F8 | Area F9 | #Feature | #Feature F1 | #Feature F10 | #Feature F2 | #Feature F3 | #Feature F4 | #Feature F5 | #Feature F7A | #Feature F7B | #Feature F8 | #Feature F9 | Start | End | PTM | AScore | Found By |
| --- | --- | --- | --- | --- | --- | --- | --- | --- | --- | --- | --- | --- | --- | --- | --- | --- | --- | --- | --- | --- | --- | --- | --- | --- | --- | --- | --- | --- | --- | --- | --- | --- | --- | --- | --- | --- | --- |
| A.DC(+57.02)PSDWSSHEGHC(+57.02)YK.F | Y | 59.80 | 1863.6941 | 15 | -0.6 | 466.9305 | 4 | 10.83 | 8 | F8:1780 | DaRuMH\_F7B.raw |  |  |  |  |  |  |  | 2.2413E5 |  | 3.2302E5 | 2 | 0 | 0 | 0 | 0 | 0 | 0 | 0 | 1 | 0 | 1 | 24 | 38 | Carbamidomethylation | C2:Carbamidomethylation:1000.00;C13:Carbamidomethylation:1000.00 | PEAKS DB |
| total 1 peptides |
| --- |

Q7ZZM2|DIS\_PROJR

back to list

  

| Protein Coverage
| Supporting Peptides
|

Protein Coverage:

Supporting Peptides:

| Peptide | Uniq | -10lgP | Mass | Length | ppm | m/z | z | RT | Fraction | Scan | Source File | Area F1 | Area F10 | Area F2 | Area F3 | Area F4 | Area F5 | Area F7A | Area F7B | Area F8 | Area F9 | #Feature | #Feature F1 | #Feature F10 | #Feature F2 | #Feature F3 | #Feature F4 | #Feature F5 | #Feature F7A | #Feature F7B | #Feature F8 | #Feature F9 | Start | End | PTM | AScore | Found By |
| --- | --- | --- | --- | --- | --- | --- | --- | --- | --- | --- | --- | --- | --- | --- | --- | --- | --- | --- | --- | --- | --- | --- | --- | --- | --- | --- | --- | --- | --- | --- | --- | --- | --- | --- | --- | --- | --- |
| Y.EVVNPGTVTGLPK.G | Y | 58.03 | 1309.7241 | 13 | 0.6 | 655.8698 | 2 | 11.91 | 3 | F3:2630 | DaRuMH\_F2.raw |  |  | 5.5757E4 |  |  |  |  |  |  |  | 1 | 0 | 0 | 1 | 0 | 0 | 0 | 0 | 0 | 0 | 0 | 32 | 44 |  |  | PEAKS DB |
| total 1 peptides |
| --- |

Q3BK14|DIS\_MACLB

back to list

  

| Protein Coverage
| Supporting Peptides
|

Protein Coverage:

Supporting Peptides:

| Peptide | Uniq | -10lgP | Mass | Length | ppm | m/z | z | RT | Fraction | Scan | Source File | Area F1 | Area F10 | Area F2 | Area F3 | Area F4 | Area F5 | Area F7A | Area F7B | Area F8 | Area F9 | #Feature | #Feature F1 | #Feature F10 | #Feature F2 | #Feature F3 | #Feature F4 | #Feature F5 | #Feature F7A | #Feature F7B | #Feature F8 | #Feature F9 | Start | End | PTM | AScore | Found By |
| --- | --- | --- | --- | --- | --- | --- | --- | --- | --- | --- | --- | --- | --- | --- | --- | --- | --- | --- | --- | --- | --- | --- | --- | --- | --- | --- | --- | --- | --- | --- | --- | --- | --- | --- | --- | --- | --- |
| Y.EVVNPGTVTGLPK.G | Y | 58.03 | 1309.7241 | 13 | 0.6 | 655.8698 | 2 | 11.91 | 3 | F3:2630 | DaRuMH\_F2.raw |  |  | 5.5757E4 |  |  |  |  |  |  |  | 1 | 0 | 0 | 1 | 0 | 0 | 0 | 0 | 0 | 0 | 0 | 32 | 44 |  |  | PEAKS DB |
| total 1 peptides |
| --- |

Q86YZ3|HORN\_HUMAN

back to list

  

| Protein Coverage
| Supporting Peptides
|

Protein Coverage:

Supporting Peptides:

| Peptide | Uniq | -10lgP | Mass | Length | ppm | m/z | z | RT | Fraction | Scan | Source File | Area F1 | Area F10 | Area F2 | Area F3 | Area F4 | Area F5 | Area F7A | Area F7B | Area F8 | Area F9 | #Feature | #Feature F1 | #Feature F10 | #Feature F2 | #Feature F3 | #Feature F4 | #Feature F5 | #Feature F7A | #Feature F7B | #Feature F8 | #Feature F9 | Start | End | PTM | AScore | Found By |
| --- | --- | --- | --- | --- | --- | --- | --- | --- | --- | --- | --- | --- | --- | --- | --- | --- | --- | --- | --- | --- | --- | --- | --- | --- | --- | --- | --- | --- | --- | --- | --- | --- | --- | --- | --- | --- | --- |
| R.GPYESGSGHSSGLGHR.E | Y | 54.09 | 1583.7076 | 16 | -0.1 | 528.9098 | 3 | 10.66 | 5 | F5:1587 | DaRuMH\_F4.raw |  |  |  |  | 0 |  |  |  |  |  | 0 | 0 | 0 | 0 | 0 | 0 | 0 | 0 | 0 | 0 | 0 | 1038 | 1053 |  |  | PEAKS DB |
| total 1 peptides |
| --- |

Peptide List

  
  

---

Prepared with PEAKS ™ (bioinfor.com)
