# Supplementary material for: Biogeographic venom variation in Russell’s viper (Daboia russelii) and the preclinical inefficacy of antivenom therapy in snakebite hotspots
Source: PLoS Negl Trop Dis. 2021 Mar 25;15(3):e0009247. doi: 10.1371/journal.pntd.0009247 (PMC7993602; doi:10.1371/journal.pntd.0009247)
Supplement: S1 Data — (ZIP) [file pntd.0009247.s013.zip › D. russelii_West Bengal/D. russelii_West Bengal.html]

proteins


Summary

  

# 1. Notes

# 2. Result Statistics

**Figure 1.**
False discovery rate (FDR) curve. X axis is the number of peptide-spectrum matches (PSM) being kept. Y axis is the corresponding FDR.

  


  

**Figure 2.**
PSM score distribution. **(a)**
Distribution of PEAKS peptide score; **(b)**
Scatterplot of PEAKS peptide score versus precursor mass error.

|  |  |  |  |
| --- | --- | --- | --- |
| **(a)**  |  | | **(b)**  |  | |

|  |  |  |  |  |  |  |  |  |  |  |  |  |  |  |  |  |  |  |  |  |  |  |  |  |  |  |  |  |  |  |  |  |  |  |  |  |  |  |  |  |  |  |  |  |  |  |  |  |  |  |  |  |  |  |  |  |  |  |  |  |  |  |  |  |  |  |  |  |  |  |  |  |  |  |  |  |  |  |  |  |  |  |  |  |  |  |  |  |  |  |  |  |  |  |  |  |  |  |  |  |  |  |  |  |  |  |  |  |  |  |  |  |  |  |  |  |  |  |  |  |  |  |  |  |  |  |  |  |  |  |  |  |  |  |  |  |  |  |  |  |
| --- | --- | --- | --- | --- | --- | --- | --- | --- | --- | --- | --- | --- | --- | --- | --- | --- | --- | --- | --- | --- | --- | --- | --- | --- | --- | --- | --- | --- | --- | --- | --- | --- | --- | --- | --- | --- | --- | --- | --- | --- | --- | --- | --- | --- | --- | --- | --- | --- | --- | --- | --- | --- | --- | --- | --- | --- | --- | --- | --- | --- | --- | --- | --- | --- | --- | --- | --- | --- | --- | --- | --- | --- | --- | --- | --- | --- | --- | --- | --- | --- | --- | --- | --- | --- | --- | --- | --- | --- | --- | --- | --- | --- | --- | --- | --- | --- | --- | --- | --- | --- | --- | --- | --- | --- | --- | --- | --- | --- | --- | --- | --- | --- | --- | --- | --- | --- | --- | --- | --- | --- | --- | --- | --- | --- | --- | --- | --- | --- | --- | --- | --- | --- | --- | --- | --- | --- | --- | --- | --- | --- |
| **Table 1.** Statistics of data.    |  |  |  |  |  |  |  |  |  |  |  |  | | --- | --- | --- | --- | --- | --- | --- | --- | --- | --- | --- | --- | |  | #Scans | | #Features | Identified | | | #Peptides | #Sequences | #Proteins\* | | | | MS1 | MS2 | #PSMs | #Scans | #Features | Groups | All | Top | | Total | 138667 | 284674 | 581440 | 298 | 291 | 19087 | 122 | 119 | 42 | 155 | 76 | | F1 | 16449 | 17876 | 53662 | 21 | 20 | 1608 | 16 | 16 | 9 | 54 | 19 | | F10 | 16746 | 11293 | 36942 | 15 | 15 | 784 | 14 | 14 | 7 | 21 | 10 | | F2 | 15885 | 33512 | 69791 | 34 | 34 | 2480 | 11 | 11 | 4 | 16 | 8 | | F5 | 16726 | 11489 | 41976 | 9 | 9 | 955 | 9 | 9 | 5 | 18 | 8 | | F7A | 13873 | 51642 | 96096 | 33 | 32 | 3462 | 29 | 29 | 16 | 51 | 22 | | F7B | 12943 | 59220 | 90091 | 87 | 86 | 4114 | 43 | 42 | 19 | 41 | 27 | | F8A | 15634 | 38723 | 72032 | 41 | 41 | 2650 | 30 | 29 | 18 | 46 | 32 | | F8B | 13623 | 51536 | 87204 | 46 | 42 | 2483 | 23 | 23 | 12 | 33 | 23 | | F9 | 16788 | 9383 | 33646 | 12 | 12 | 551 | 11 | 11 | 9 | 20 | 13 |  \* proteins with significant peptides are used in counts. |

**Figure 3.**
Sample overlap for Proteins and Peptides (up to 8 samples). **(a)**
All Proteins; **(b)**
Top Proteins; **(c)**
Peptides;

|  |  |  |  |  |  |
| --- | --- | --- | --- | --- | --- |
| **(a)**  | **Do not support more than 8 samples** | | **(b)**  | **Do not support more than 8 samples** | | **(c)**  | **Do not support more than 8 samples** | |

**Figure 4.**
Distribution of peptide feature detection. **(a)**
Feature m/z distribution; **(b)**
Feature RT distribution.

|  |  |  |  |
| --- | --- | --- | --- |
| **(a)**  |  | | **(b)**  |  | |

**Figure 5.**
Distribution of identified peptide features. **(a)**
Feature abundance distribution; **(b)**
*De novo*
sequencing validation.

|  |  |  |  |
| --- | --- | --- | --- |
| **(a)**  |  | | **(b)**  |  | |

|  |  |  |  |  |  |  |  |  |  |  |  |  |  |  |  |  |  |  |  |  |  |  |  |  |  |  |  |  |  |  |  |  |  |  |  |  |  |  |  |  |
| --- | --- | --- | --- | --- | --- | --- | --- | --- | --- | --- | --- | --- | --- | --- | --- | --- | --- | --- | --- | --- | --- | --- | --- | --- | --- | --- | --- | --- | --- | --- | --- | --- | --- | --- | --- | --- | --- | --- | --- | --- |
| **Table 2.** Result filtration parameters.  | Peptide -10lgP | ≥38.6 | | PTM Ascore | ≥0 | | Protein -10lgP | ≥20 | | Proteins unique peptides | ≥1 | | De novo score(%) | ≥50% |    **Table 3.** Statistics of filtered result.  | FDR (Peptide-Spectrum Matches) | 0.0% | | FDR (Peptide Sequences) | 0.0% | | FDR (Protein Group) | 0.0% | | De Novo Only Spectra | 26020 | | **Table 4.** PTM profile.  | Name | ∆Mass | Position | #PSM | -10lgP | Abundance | AScore || Carbamidomethyl | 57.02 | C | 175 | 78.75 | 4.11E7 | 1000.00 | | Oxidation | 15.99 | M | 9 | 56.52 | 5.59E7 | 1000.00 | |

# 3. Experiment Control

**Figure 6.**
Precursor mass error of peptide-spectrum matches (PSM) in filtered result. **(a)**
Distribution of precursor mass error in ppm; **(b)**
Scatterplot of precursor m/z versus precursor mass error in ppm.

|  |  |  |  |
| --- | --- | --- | --- |
| **(a)**  |  | | **(b)**  |  | |

**Table 5.**
Number of identified peptides in each sample by the number of missed cleavages.

|  |  |  |  |  |  |  |  |  |  |  |  |  |  |  |  |  |  |  |  |  |  |  |  |  |  |  |  |  |  |  |  |  |  |  |  |  |  |  |  |  |  |  |  |  |  |  |  |  |  |  |  |  |  |  |  |  |  |  |  |  |
| --- | --- | --- | --- | --- | --- | --- | --- | --- | --- | --- | --- | --- | --- | --- | --- | --- | --- | --- | --- | --- | --- | --- | --- | --- | --- | --- | --- | --- | --- | --- | --- | --- | --- | --- | --- | --- | --- | --- | --- | --- | --- | --- | --- | --- | --- | --- | --- | --- | --- | --- | --- | --- | --- | --- | --- | --- | --- | --- | --- | --- |
| |  |  |  |  |  |  | | --- | --- | --- | --- | --- | --- | | Missed Cleavages | 0 | 1 | 2 | 3 | 4+ | | F1 | 16 | 0 | 0 | 0 | 0 | | F10 | 13 | 1 | 0 | 0 | 0 | | F2 | 11 | 0 | 0 | 0 | 0 | | F5 | 8 | 1 | 0 | 0 | 0 | | F7A | 28 | 1 | 0 | 0 | 0 | | F7B | 40 | 3 | 0 | 0 | 0 | | F8A | 28 | 2 | 0 | 0 | 0 | | F8B | 23 | 0 | 0 | 0 | 0 | | F9 | 11 | 0 | 0 | 0 | 0 | |

# 4. Other Information

|  |  |  |  |
| --- | --- | --- | --- |
| **Table 6.** Search parameters.  | Search Engine Name: PEAKS Parent Mass Error Tolerance: 10.0 ppm Fragment Mass Error Tolerance: 0.6 Da Precursor Mass Search Type: monoisotopic Enzyme: Trypsin Max Missed Cleavages: 2 Digest Mode: Semispecific Fixed Modifications:    Carbamidomethylation: 57.02 Variable Modifications:    Oxidation (M): 15.99 Max Variable PTM Per Peptide: 3 Database: SwissProt Taxon: All Contaminant Database: cRAP\_contaminants Searched Entry: 560234 FDR Estimation: Enabled Merge Options: no merge Precursor Options: corrected Charge Options: no correction Filter Charge: 2 - 8 Process: true Associate chimera: yes | | **Table 7.** Instrument parameters.  | Fractions: DaRuWB\_F1.raw, DaRuWB\_F10.raw, DaRuWB\_F2.raw, DaRuWB \_F5.raw, DaRuWB\_F7A.raw, DaRuWB\_F7B.raw, DaRuWB\_F8A.raw, DaRu WB\_F8B.raw, DaRuWB\_F9.raw Ion Source: ESI(nano-spray) Fragmentation Mode: CID, CAD(y and b ions) MS Scan Mode: FT-ICR/Orbitrap MS/MS Scan Mode: FT-ICR/Orbitrap | |

  

Protein List

  

|  |
| --- |
| Protein Accession Contains: |
| Protein Description Contains: |
| Protein Sample Area >= |
| Protein PTM Contains: |

| Protein Group | Protein ID | Accession | -10lgP | Coverage (%) | Coverage (%) F1 | Coverage (%) F10 | Coverage (%) F2 | Coverage (%) F5 | Coverage (%) F7A | Coverage (%) F7B | Coverage (%) F8A | Coverage (%) F8B | Coverage (%) F9 | Area F1 | Area F10 | Area F2 | Area F5 | Area F7A | Area F7B | Area F8A | Area F8B | Area F9 | #Peptides | #Unique | #Spec F1 | #Spec F10 | #Spec F2 | #Spec F5 | #Spec F7A | #Spec F7B | #Spec F8A | #Spec F8B | #Spec F9 | PTM | Avg. Mass | Description |
| --- | --- | --- | --- | --- | --- | --- | --- | --- | --- | --- | --- | --- | --- | --- | --- | --- | --- | --- | --- | --- | --- | --- | --- | --- | --- | --- | --- | --- | --- | --- | --- | --- | --- | --- | --- | --- |
| 3 | 7 | A8CG86|PA2A1\_DABRR | 179.85 | 52 | 0 | 0 | 0 | 0 | 20 | 11 | 41 | 51 | 11 |  |  |  |  | 1.0171E6 |  | 7.1359E6 | 1.7344E8 |  | 10 | 4 | 0 | 0 | 0 | 0 | 2 | 1 | 5 | 27 | 1 | Y | 15329 | Acidic phospholipase A2 Drk-a1 OS=Daboia russelii OX=8707 PE=1 SV=1 |
| 6 | 2 | P04264|K2C1\_HUMAN | 169.93 | 17 | 2 | 11 | 5 | 4 | 2 | 3 | 2 | 6 | 7 |  | 1.7726E7 | 1.2444E7 | 5.6537E6 | 2.7796E7 |  |  | 5.0285E5 | 4.0757E6 | 9 | 6 | 1 | 7 | 4 | 2 | 2 | 2 | 2 | 3 | 4 | N | 66039 | Keratin, type II cytoskeletal 1 OS=Homo sapiens OX=9606 GN=KRT1 PE=1 SV=6 |
| 6 | 3 | A5A6M6|K2C1\_PANTR | 169.93 | 17 | 2 | 11 | 5 | 4 | 2 | 3 | 2 | 6 | 7 |  | 1.7726E7 | 1.2444E7 | 5.6537E6 | 2.7796E7 |  |  | 5.0285E5 | 4.0757E6 | 9 | 6 | 1 | 7 | 4 | 2 | 2 | 2 | 2 | 3 | 4 | N | 65489 | Keratin, type II cytoskeletal 1 OS=Pan troglodytes OX=9598 GN=KRT1 PE=2 SV=1 |
| 1 | 6 | A8CG89|PA2B1\_DABRR | 167.51 | 43 | 0 | 0 | 0 | 0 | 17 | 43 | 11 | 0 | 0 |  |  |  |  | 4.111E7 | 7.8883E9 | 1.1103E7 |  |  | 7 | 2 | 0 | 0 | 0 | 0 | 2 | 41 | 2 | 0 | 0 | Y | 15864 | Basic phospholipase A2 Drk-b1 OS=Daboia russelii OX=8707 PE=1 SV=1 |
| 2 | 81 | Q2ES50|VKT1\_DABRR | 161.89 | 37 | 19 | 0 | 37 | 12 | 0 | 0 | 0 | 0 | 0 | 4.7137E7 |  | 6.915E9 |  |  |  |  |  |  | 7 | 1 | 8 | 0 | 28 | 1 | 0 | 0 | 0 | 0 | 0 | Y | 9287 | Kunitz-type serine protease inhibitor 1 OS=Daboia russelii OX=8707 PE=2 SV=1 |
| 2 | 82 | A8Y7P0|VKTB7\_DABSI | 161.89 | 37 | 19 | 0 | 37 | 12 | 0 | 0 | 0 | 0 | 0 | 4.7137E7 |  | 6.915E9 |  |  |  |  |  |  | 7 | 1 | 8 | 0 | 28 | 1 | 0 | 0 | 0 | 0 | 0 | Y | 9287 | Kunitz-type serine protease inhibitor 7 OS=Daboia siamensis OX=343250 PE=2 SV=1 |
| 5 | 4 | P35527|K1C9\_HUMAN | 159.95 | 18 | 2 | 2 | 2 | 6 | 6 | 6 | 9 | 4 | 2 | 2.3583E5 | 1.2366E6 | 7.9395E5 | 4.2079E6 | 1.215E7 | 9.727E6 | 7.5795E6 | 2.2982E6 | 7.8101E5 | 8 | 8 | 1 | 1 | 1 | 3 | 3 | 3 | 5 | 2 | 2 | N | 62064 | Keratin, type I cytoskeletal 9 OS=Homo sapiens OX=9606 GN=KRT9 PE=1 SV=3 |
| 12 | 12 | P0DPS3|VASP1\_VIPAA | 155.25 | 32 | 0 | 0 | 0 | 0 | 20 | 32 | 11 | 0 | 0 |  |  |  |  | 6.1274E7 | 2.1415E8 | 8.1222E6 |  |  | 5 | 2 | 0 | 0 | 0 | 0 | 4 | 9 | 2 | 0 | 0 | Y | 22639 | Snake venom serine protease VaSP1 (Fragments) OS=Vipera ammodytes ammodytes OX=8705 PE=1 SV=1 |
| 7 | 15 | P31100|PA2A7\_DABSI | 149.28 | 47 | 0 | 0 | 0 | 0 | 11 | 11 | 33 | 47 | 11 |  |  |  |  |  |  | 1.6264E6 | 5.0159E7 |  | 6 | 2 | 0 | 0 | 0 | 0 | 1 | 1 | 3 | 20 | 1 | Y | 15421 | Acidic phospholipase A2 RV-7 OS=Daboia siamensis OX=343250 PE=1 SV=1 |
| 8 | 8 | P18965|VSPG\_DABSI | 144.25 | 42 | 0 | 0 | 0 | 0 | 0 | 38 | 18 | 0 | 0 |  |  |  |  |  | 1.0813E9 | 3.632E8 |  |  | 8 | 7 | 0 | 0 | 0 | 0 | 0 | 8 | 8 | 0 | 0 | Y | 28823 | Factor V activator RVV-V gamma OS=Daboia siamensis OX=343250 PE=1 SV=2 |
| 16 | 19 | Q5XQN5|K2C5\_BOVIN | 125.77 | 5 | 0 | 2 | 0 | 0 | 3 | 2 | 2 | 0 | 4 |  |  |  |  | 4.1311E5 |  | 4.7333E5 |  |  | 3 | 1 | 0 | 1 | 0 | 0 | 2 | 1 | 1 | 0 | 2 | N | 62937 | Keratin, type II cytoskeletal 5 OS=Bos taurus OX=9913 GN=KRT5 PE=1 SV=1 |
| 16 | 20 | Q6P6Q2|K2C5\_RAT | 125.77 | 6 | 0 | 2 | 0 | 0 | 4 | 2 | 2 | 0 | 4 |  |  |  |  | 4.1311E5 |  | 4.7333E5 |  |  | 3 | 1 | 0 | 1 | 0 | 0 | 2 | 1 | 1 | 0 | 2 | N | 61826 | Keratin, type II cytoskeletal 5 OS=Rattus norvegicus OX=10116 GN=Krt5 PE=1 SV=1 |
| 16 | 21 | Q922U2|K2C5\_MOUSE | 125.77 | 6 | 0 | 2 | 0 | 0 | 4 | 2 | 2 | 0 | 4 |  |  |  |  | 4.1311E5 |  | 4.7333E5 |  |  | 3 | 1 | 0 | 1 | 0 | 0 | 2 | 1 | 1 | 0 | 2 | N | 61767 | Keratin, type II cytoskeletal 5 OS=Mus musculus OX=10090 GN=Krt5 PE=1 SV=1 |
| 15 | 16 | P35908|K22E\_HUMAN | 124.63 | 7 | 0 | 4 | 0 | 0 | 2 | 0 | 0 | 2 | 3 |  | 2.8299E6 |  |  |  |  |  |  | 6.4318E5 | 4 | 2 | 0 | 2 | 0 | 0 | 1 | 0 | 0 | 1 | 2 | N | 65433 | Keratin, type II cytoskeletal 2 epidermal OS=Homo sapiens OX=9606 GN=KRT2 PE=1 SV=2 |
| 40 | 31 | Q2MY38|PAT13\_SOLTU | 118.32 | 10 | 10 | 0 | 0 | 0 | 0 | 0 | 0 | 0 | 0 | 4.1315E6 |  |  |  |  |  |  |  |  | 3 | 2 | 3 | 0 | 0 | 0 | 0 | 0 | 0 | 0 | 0 | N | 42424 | Patatin-13 OS=Solanum tuberosum OX=4113 PE=2 SV=1 |
| 13 | 34 | Q7ZT99|CRVP\_CROAT | 116.43 | 15 | 0 | 0 | 0 | 0 | 15 | 15 | 5 | 0 | 0 |  |  |  |  | 5.9493E7 | 8.676E8 | 5.9622E5 |  |  | 4 | 3 | 0 | 0 | 0 | 0 | 4 | 7 | 1 | 0 | 0 | Y | 26647 | Cysteine-rich venom protein catrin OS=Crotalus atrox OX=8730 PE=1 SV=1 |
| 13 | 35 | Q7ZTA0|CRVP\_AGKPI | 116.43 | 15 | 0 | 0 | 0 | 0 | 15 | 15 | 5 | 0 | 0 |  |  |  |  | 5.9493E7 | 8.676E8 | 5.9622E5 |  |  | 4 | 3 | 0 | 0 | 0 | 0 | 4 | 7 | 1 | 0 | 0 | Y | 26681 | Cysteine-rich venom protein piscivorin OS=Agkistrodon piscivorus piscivorus OX=8716 PE=1 SV=1 |
| 20 | 32 | A8CG87|PA2A2\_DABRR | 115.38 | 23 | 0 | 0 | 0 | 0 | 23 | 7 | 0 | 0 | 0 |  |  |  |  | 7.3865E7 |  |  |  |  | 3 | 1 | 0 | 0 | 0 | 0 | 4 | 1 | 0 | 0 | 0 | Y | 15586 | Acidic phospholipase A2 Drk-a2 OS=Daboia russelii OX=8707 PE=2 SV=1 |
| 20 | 33 | A8CG78|PA2A2\_DABSI | 115.38 | 23 | 0 | 0 | 0 | 0 | 23 | 7 | 0 | 0 | 0 |  |  |  |  | 7.3865E7 |  |  |  |  | 3 | 1 | 0 | 0 | 0 | 0 | 4 | 1 | 0 | 0 | 0 | Y | 15586 | Acidic phospholipase A2 DsM-a2/DsM-a2' OS=Daboia siamensis OX=343250 PE=1 SV=1 |
| 23 | 25 | E5L0E3|VSPAF\_DABSI | 114.16 | 18 | 0 | 0 | 0 | 0 | 0 | 0 | 18 | 3 | 0 |  |  |  |  |  |  | 3.8312E8 | 2.6212E6 |  | 4 | 4 | 0 | 0 | 0 | 0 | 0 | 0 | 7 | 1 | 0 | Y | 28496 | Alpha-fibrinogenase-like OS=Daboia siamensis OX=343250 PE=2 SV=1 |
| 28 | 44 | E5L0E4|VSPB\_DABSI | 111.41 | 9 | 0 | 0 | 0 | 0 | 5 | 9 | 5 | 0 | 0 |  |  |  |  |  | 1.1862E6 |  |  |  | 2 | 1 | 0 | 0 | 0 | 0 | 1 | 2 | 1 | 0 | 0 | Y | 28035 | Beta-fibrinogenase-like OS=Daboia siamensis OX=343250 PE=2 SV=1 |
| 14 | 108 | J3SDX0|VSP4\_CROAD | 106.48 | 16 | 0 | 0 | 0 | 0 | 16 | 13 | 13 | 0 | 0 |  |  |  |  | 7.6428E5 |  |  |  |  | 3 | 1 | 0 | 0 | 0 | 0 | 3 | 4 | 4 | 0 | 0 | Y | 28923 | Snake venom serine proteinase 4a OS=Crotalus adamanteus OX=8729 PE=1 SV=1 |
| 17 | 28 | P02533|K1C14\_HUMAN | 101.84 | 8 | 0 | 3 | 0 | 0 | 2 | 3 | 0 | 2 | 0 |  | 4.1201E5 |  |  | 1.0386E6 | 2.7111E5 |  | 2.1496E5 |  | 3 | 3 | 0 | 1 | 0 | 0 | 1 | 1 | 0 | 1 | 0 | N | 51562 | Keratin, type I cytoskeletal 14 OS=Homo sapiens OX=9606 GN=KRT14 PE=1 SV=4 |
| 21 | 23 | P13645|K1C10\_HUMAN | 100.76 | 8 | 0 | 6 | 0 | 0 | 5 | 0 | 0 | 0 | 0 |  | 1.029E6 |  |  | 1.7818E7 |  |  |  |  | 3 | 3 | 0 | 2 | 0 | 0 | 2 | 0 | 0 | 0 | 0 | Y | 58827 | Keratin, type I cytoskeletal 10 OS=Homo sapiens OX=9606 GN=KRT10 PE=1 SV=6 |
| 31 | 330 | C0HLB2|VKT\_PSEPC | 98.05 | 22 | 0 | 0 | 22 | 0 | 0 | 0 | 0 | 0 | 0 |  |  | 0 |  |  |  |  |  |  | 3 | 1 | 0 | 0 | 4 | 0 | 0 | 0 | 0 | 0 | 0 | Y | 7668 | Kunitz-type serine protease inhibitor PPTI OS=Pseudocerastes persicus OX=47769 PE=1 SV=1 |
| 31 | 333 | P24541|VKT\_ERIMA | 98.05 | 24 | 0 | 0 | 24 | 0 | 0 | 0 | 0 | 0 | 0 |  |  | 0 |  |  |  |  |  |  | 3 | 1 | 0 | 0 | 4 | 0 | 0 | 0 | 0 | 0 | 0 | Y | 6772 | Kunitz-type serine protease inhibitor OS=Eristicophis macmahoni OX=110227 PE=1 SV=1 |
| 31 | 341 | Q2ES47|VKT4\_DABRR | 98.05 | 18 | 0 | 0 | 18 | 0 | 0 | 0 | 0 | 0 | 0 |  |  | 0 |  |  |  |  |  |  | 3 | 1 | 0 | 0 | 4 | 0 | 0 | 0 | 0 | 0 | 0 | Y | 9145 | Kunitz-type serine protease inhibitor 4 OS=Daboia russelii OX=8707 PE=2 SV=1 |
| 34 | 69 | P30894|NGFV\_DABRR | 98.04 | 19 | 0 | 0 | 0 | 19 | 0 | 0 | 0 | 0 | 0 |  |  |  | 1.2024E8 |  |  |  |  |  | 2 | 2 | 0 | 0 | 0 | 2 | 0 | 0 | 0 | 0 | 0 | Y | 13283 | Venom nerve growth factor OS=Daboia russelii OX=8707 PE=1 SV=1 |
| 38 | 61 | G8XQX1|OXLA\_DABRR | 97.96 | 5 | 0 | 0 | 0 | 0 | 0 | 0 | 3 | 0 | 2 |  |  |  |  |  |  | 2.8108E5 |  | 3.1694E6 | 2 | 2 | 0 | 0 | 0 | 0 | 0 | 0 | 1 | 0 | 1 | Y | 56888 | L-amino-acid oxidase OS=Daboia russelii OX=8707 PE=1 SV=1 |
| 33 | 48 | O22507|LOX17\_SOLTU | 97.48 | 3 | 3 | 0 | 0 | 0 | 0 | 0 | 0 | 0 | 0 | 4.0499E5 |  |  |  |  |  |  |  |  | 2 | 2 | 2 | 0 | 0 | 0 | 0 | 0 | 0 | 0 | 0 | N | 97065 | Probable linoleate 9S-lipoxygenase 7 OS=Solanum tuberosum OX=4113 GN=LOX1.7 PE=2 SV=1 |
| 33 | 50 | O22508|LOX18\_SOLTU | 97.48 | 3 | 3 | 0 | 0 | 0 | 0 | 0 | 0 | 0 | 0 | 4.0499E5 |  |  |  |  |  |  |  |  | 2 | 2 | 2 | 0 | 0 | 0 | 0 | 0 | 0 | 0 | 0 | N | 97020 | Probable linoleate 9S-lipoxygenase 8 OS=Solanum tuberosum OX=4113 GN=LOX1.8 PE=2 SV=1 |
| 45 | 237 | Q6WP39|OXLA\_TRIST | 94.26 | 4 | 0 | 0 | 0 | 0 | 0 | 0 | 0 | 4 | 0 |  |  |  |  |  |  |  | 5.2576E6 |  | 1 | 1 | 0 | 0 | 0 | 0 | 0 | 0 | 0 | 1 | 0 | N | 58601 | L-amino-acid oxidase OS=Trimeresurus stejnegeri OX=39682 PE=1 SV=1 |
| 26 | 113 | B7FDI1|CRVP\_VIPBE | 92.44 | 16 | 0 | 0 | 0 | 0 | 16 | 5 | 0 | 0 | 0 |  |  |  |  | 4.3914E6 |  |  |  |  | 2 | 1 | 0 | 0 | 0 | 0 | 2 | 4 | 0 | 0 | 0 | Y | 26509 | Cysteine-rich venom protein OS=Vipera berus OX=31155 PE=1 SV=1 |
| 26 | 114 | B7FDI0|CRVP\_VIPBN | 92.44 | 18 | 0 | 0 | 0 | 0 | 18 | 6 | 0 | 0 | 0 |  |  |  |  | 4.3914E6 |  |  |  |  | 2 | 1 | 0 | 0 | 0 | 0 | 2 | 4 | 0 | 0 | 0 | Y | 24612 | Cysteine-rich venom protein (Fragment) OS=Vipera berus nikolskii OX=1808362 PE=2 SV=1 |
| 49 | 68 | Q02471|PA2B4\_DABSI | 91.93 | 19 | 0 | 0 | 0 | 0 | 0 | 19 | 0 | 0 | 0 |  |  |  |  |  | 1.0574E7 |  |  |  | 2 | 1 | 0 | 0 | 0 | 0 | 0 | 2 | 0 | 0 | 0 | Y | 15555 | Basic phospholipase A2 RV-4 OS=Daboia siamensis OX=343250 PE=1 SV=1 |
| 46 | 88 | P20347|CPI1\_SOLTU | 89.92 | 9 | 9 | 0 | 0 | 0 | 0 | 0 | 0 | 0 | 0 | 4.8192E6 |  |  |  |  |  |  |  |  | 2 | 1 | 3 | 0 | 0 | 0 | 0 | 0 | 0 | 0 | 0 | N | 24684 | Cysteine protease inhibitor 1 OS=Solanum tuberosum OX=4113 PE=1 SV=3 |
| 46 | 109 | O24387|CPI5\_SOLTU | 89.92 | 14 | 14 | 0 | 0 | 0 | 0 | 0 | 0 | 0 | 0 | 4.8192E6 |  |  |  |  |  |  |  |  | 2 | 1 | 3 | 0 | 0 | 0 | 0 | 0 | 0 | 0 | 0 | N | 16869 | Cysteine protease inhibitor 5 (Fragment) OS=Solanum tuberosum OX=4113 PE=2 SV=1 |
| 46 | 130 | O24388|CPI3\_SOLTU | 89.92 | 14 | 14 | 0 | 0 | 0 | 0 | 0 | 0 | 0 | 0 | 4.8192E6 |  |  |  |  |  |  |  |  | 2 | 1 | 3 | 0 | 0 | 0 | 0 | 0 | 0 | 0 | 0 | N | 16378 | Cysteine protease inhibitor 3 (Fragment) OS=Solanum tuberosum OX=4113 PE=2 SV=1 |
| 32 | 118 | Q8JFG1|PA2H\_VIPAP | 88.16 | 20 | 0 | 0 | 0 | 0 | 0 | 0 | 9 | 11 | 0 |  |  |  |  |  |  |  | 2.5614E7 |  | 2 | 1 | 0 | 0 | 0 | 0 | 0 | 0 | 1 | 5 | 0 | Y | 15411 | Acidic phospholipase A2 inhibitor vaspin A chain OS=Vipera aspis aspis OX=194601 PE=2 SV=1 |
| 32 | 119 | Q10754|PA2H\_VIPAZ | 88.16 | 20 | 0 | 0 | 0 | 0 | 0 | 0 | 9 | 11 | 0 |  |  |  |  |  |  |  | 2.5614E7 |  | 2 | 1 | 0 | 0 | 0 | 0 | 0 | 0 | 1 | 5 | 0 | Y | 15411 | Acidic phospholipase A2 inhibitor vaspin A chain OS=Vipera aspis zinnikeri OX=55427 PE=1 SV=2 |
| 32 | 127 | A4VBF0|PA2H\_VIPBN | 88.16 | 20 | 0 | 0 | 0 | 0 | 0 | 0 | 9 | 11 | 0 |  |  |  |  |  |  |  | 2.5614E7 |  | 2 | 1 | 0 | 0 | 0 | 0 | 0 | 0 | 1 | 5 | 0 | Y | 15381 | Acidic phospholipase A2 inhibitor chain HPD-1I OS=Vipera berus nikolskii OX=1808362 PE=1 SV=1 |
| 32 | 128 | P04084|PA2A\_VIPAE | 88.16 | 22 | 0 | 0 | 0 | 0 | 0 | 0 | 10 | 12 | 0 |  |  |  |  |  |  |  | 2.5614E7 |  | 2 | 1 | 0 | 0 | 0 | 0 | 0 | 0 | 1 | 5 | 0 | Y | 13639 | Acidic phospholipase A2 homolog vipoxin A chain OS=Vipera ammodytes meridionalis OX=73841 PE=1 SV=3 |
| 43 | 66 | Q38L02|SLA\_DABSI | 85.90 | 23 | 0 | 0 | 0 | 0 | 0 | 15 | 8 | 0 | 0 |  |  |  |  |  | 2.2022E7 | 6.6672E6 |  |  | 2 | 2 | 0 | 0 | 0 | 0 | 0 | 1 | 1 | 0 | 0 | Y | 17507 | Snaclec dabocetin subunit alpha OS=Daboia siamensis OX=343250 PE=1 SV=1 |
| 42 | 46 | B8K1W0|VM3DK\_DABRR | 81.07 | 5 | 0 | 5 | 0 | 0 | 0 | 0 | 0 | 0 | 0 |  | 2.3247E6 |  |  |  |  |  |  |  | 2 | 2 | 0 | 2 | 0 | 0 | 0 | 0 | 0 | 0 | 0 | Y | 69555 | Zinc metalloproteinase-disintegrin-like daborhagin-K OS=Daboia russelii OX=8707 PE=1 SV=1 |
| 47 | 137 | Q3YJS9|PT3K1\_SOLTU | 78.48 | 7 | 7 | 0 | 0 | 0 | 0 | 0 | 0 | 0 | 0 | 1.0466E6 |  |  |  |  |  |  |  |  | 2 | 1 | 2 | 0 | 0 | 0 | 0 | 0 | 0 | 0 | 0 | N | 41193 | Probable inactive patatin-3-Kuras 1 OS=Solanum tuberosum OX=4113 GN=pat3-k1 PE=1 SV=1 |
| 44 | 67 | Q4PRC6|SL7\_DABSI | 77.48 | 10 | 0 | 0 | 0 | 0 | 0 | 0 | 0 | 0 | 10 |  |  |  |  |  |  |  |  | 1.4055E5 | 1 | 1 | 0 | 0 | 0 | 0 | 0 | 0 | 0 | 0 | 1 | Y | 18067 | Snaclec 7 OS=Daboia siamensis OX=343250 PE=2 SV=1 |
| 55 | 120 | Q00652|CPI9\_SOLTU | 70.21 | 11 | 11 | 0 | 0 | 0 | 0 | 0 | 0 | 0 | 0 | 2.9655E5 |  |  |  |  |  |  |  |  | 2 | 1 | 2 | 0 | 0 | 0 | 0 | 0 | 0 | 0 | 0 | N | 24739 | Cysteine protease inhibitor 9 OS=Solanum tuberosum OX=4113 PE=2 SV=1 |
| 53 | 294 | D1MGU0|SLA\_PROJR | 66.27 | 9 | 0 | 0 | 0 | 0 | 0 | 9 | 0 | 9 | 0 |  |  |  |  |  | 9.0764E5 |  | 4.0188E6 |  | 1 | 1 | 0 | 0 | 0 | 0 | 0 | 1 | 0 | 1 | 0 | N | 18146 | Snaclec jerdonibitin subunit alpha OS=Protobothrops jerdonii OX=242841 PE=1 SV=1 |
| 35 | 233 | Q4PRD1|SLLC1\_DABSI | 65.64 | 20 | 0 | 0 | 0 | 0 | 0 | 11 | 9 | 11 | 0 |  |  |  |  |  | 9.605E5 | 3.934E5 | 8.9402E5 |  | 2 | 2 | 0 | 0 | 0 | 0 | 0 | 1 | 1 | 1 | 0 | Y | 16871 | Snaclec coagulation factor X-activating enzyme light chain 1 OS=Daboia siamensis OX=343250 GN=LC1 PE=1 SV=2 |
| 35 | 234 | Q4PRC7|SL6\_DABSI | 65.64 | 20 | 0 | 0 | 0 | 0 | 0 | 11 | 9 | 11 | 0 |  |  |  |  |  | 9.605E5 | 3.934E5 | 8.9402E5 |  | 2 | 2 | 0 | 0 | 0 | 0 | 0 | 1 | 1 | 1 | 0 | Y | 16584 | Snaclec 6 OS=Daboia siamensis OX=343250 PE=2 SV=1 |
| 35 | 235 | Q4PRC9|SL4\_DABSI | 65.64 | 20 | 0 | 0 | 0 | 0 | 0 | 11 | 9 | 11 | 0 |  |  |  |  |  | 9.605E5 | 3.934E5 | 8.9402E5 |  | 2 | 2 | 0 | 0 | 0 | 0 | 0 | 1 | 1 | 1 | 0 | Y | 16811 | Snaclec 4 OS=Daboia siamensis OX=343250 PE=2 SV=1 |
| 63 | 214 | P58515|SPI2\_SOLTU | 47.48 | 6 | 6 | 0 | 0 | 0 | 0 | 0 | 0 | 0 | 0 | 1.3526E6 |  |  |  |  |  |  |  |  | 1 | 1 | 1 | 0 | 0 | 0 | 0 | 0 | 0 | 0 | 0 | N | 20116 | Serine protease inhibitor 2 OS=Solanum tuberosum OX=4113 PE=1 SV=1 |
| 63 | 215 | Q41433|SPI6\_SOLTU | 47.48 | 5 | 5 | 0 | 0 | 0 | 0 | 0 | 0 | 0 | 0 | 1.3526E6 |  |  |  |  |  |  |  |  | 1 | 1 | 1 | 0 | 0 | 0 | 0 | 0 | 0 | 0 | 0 | N | 24097 | Probable serine protease inhibitor 6 OS=Solanum tuberosum OX=4113 PE=2 SV=1 |
| 63 | 230 | P58514|SPI1\_SOLTU | 47.48 | 5 | 5 | 0 | 0 | 0 | 0 | 0 | 0 | 0 | 0 | 1.3526E6 |  |  |  |  |  |  |  |  | 1 | 1 | 1 | 0 | 0 | 0 | 0 | 0 | 0 | 0 | 0 | N | 24009 | Serine protease inhibitor 1 OS=Solanum tuberosum OX=4113 PE=1 SV=2 |
| 63 | 236 | Q41480|API1\_SOLTU | 47.48 | 5 | 5 | 0 | 0 | 0 | 0 | 0 | 0 | 0 | 0 | 1.3526E6 |  |  |  |  |  |  |  |  | 1 | 1 | 1 | 0 | 0 | 0 | 0 | 0 | 0 | 0 | 0 | N | 24546 | Aspartic protease inhibitor 1 OS=Solanum tuberosum OX=4113 PE=1 SV=2 |
| 63 | 249 | P30941|SPI7\_SOLTU | 47.48 | 5 | 5 | 0 | 0 | 0 | 0 | 0 | 0 | 0 | 0 | 1.3526E6 |  |  |  |  |  |  |  |  | 1 | 1 | 1 | 0 | 0 | 0 | 0 | 0 | 0 | 0 | 0 | N | 24028 | Serine protease inhibitor 7 OS=Solanum tuberosum OX=4113 PE=1 SV=2 |
| 63 | 336 | Q41448|API7\_SOLTU | 47.48 | 5 | 5 | 0 | 0 | 0 | 0 | 0 | 0 | 0 | 0 | 1.3526E6 |  |  |  |  |  |  |  |  | 1 | 1 | 1 | 0 | 0 | 0 | 0 | 0 | 0 | 0 | 0 | N | 24485 | Aspartic protease inhibitor 7 OS=Solanum tuberosum OX=4113 PE=2 SV=1 |
| 75 | 370 | P85487|UP03\_PINHA | 44.15 | 100 | 0 | 0 | 0 | 0 | 100 | 0 | 0 | 0 | 0 |  |  |  |  | 0 |  |  |  |  | 1 | 1 | 0 | 0 | 0 | 0 | 1 | 0 | 0 | 0 | 0 | N | 1158 | Unknown protein 3 (Fragment) OS=Pinus halepensis OX=71633 PE=1 SV=1 |
| 51 | 270 | P15445|PA2A2\_NAJNA | 41.21 | 12 | 0 | 0 | 0 | 0 | 0 | 0 | 12 | 0 | 0 |  |  |  |  |  |  | 4.2829E6 |  |  | 1 | 1 | 0 | 0 | 0 | 0 | 0 | 0 | 1 | 0 | 0 | Y | 13346 | Acidic phospholipase A2 2 OS=Naja naja OX=35670 PE=1 SV=1 |
| 51 | 315 | P60045|PA2A3\_NAJSG | 41.21 | 11 | 0 | 0 | 0 | 0 | 0 | 0 | 11 | 0 | 0 |  |  |  |  |  |  | 4.2829E6 |  |  | 1 | 1 | 0 | 0 | 0 | 0 | 0 | 0 | 1 | 0 | 0 | Y | 13969 | Acidic phospholipase A2 3 (Fragment) OS=Naja sagittifera OX=195058 PE=1 SV=1 |
| 65 | 261 | Q4PRC8|SL5\_DABSI | 41.12 | 8 | 0 | 0 | 0 | 0 | 0 | 0 | 0 | 0 | 8 |  |  |  |  |  |  |  |  | 6.465E6 | 1 | 1 | 0 | 0 | 0 | 0 | 0 | 0 | 0 | 0 | 1 | Y | 17131 | Snaclec 5 OS=Daboia siamensis OX=343250 PE=2 SV=1 |
| 65 | 272 | Q4PRD0|SL3\_DABSI | 41.12 | 8 | 0 | 0 | 0 | 0 | 0 | 0 | 0 | 0 | 8 |  |  |  |  |  |  |  |  | 6.465E6 | 1 | 1 | 0 | 0 | 0 | 0 | 0 | 0 | 0 | 0 | 1 | Y | 16910 | Snaclec 3 OS=Daboia siamensis OX=343250 PE=2 SV=1 |
| 67 | 266 | P0CG03|VSPBH\_BOTAL | 40.53 | 4 | 0 | 0 | 0 | 0 | 0 | 4 | 0 | 0 | 0 |  |  |  |  |  | 1.9742E5 |  |  |  | 1 | 1 | 0 | 0 | 0 | 0 | 0 | 1 | 0 | 0 | 0 | N | 28045 | Thrombin-like enzyme bhalternin OS=Bothrops alternatus OX=64174 PE=1 SV=1 |
| 57 | 357 | P0DJL3|SLB\_TRIPP | 40.28 | 8 | 0 | 0 | 0 | 0 | 0 | 0 | 8 | 0 | 0 |  |  |  |  |  |  | 5.4749E5 |  |  | 1 | 1 | 0 | 0 | 0 | 0 | 0 | 0 | 1 | 0 | 0 | N | 14498 | Snaclec purpureotin subunit beta OS=Trimeresurus purpureomaculatus OX=101163 PE=1 SV=1 |
| 57 | 358 | P81116|SLBB\_TRIAB | 40.28 | 7 | 0 | 0 | 0 | 0 | 0 | 0 | 7 | 0 | 0 |  |  |  |  |  |  | 5.4749E5 |  |  | 1 | 1 | 0 | 0 | 0 | 0 | 0 | 0 | 1 | 0 | 0 | N | 16871 | Snaclec alboaggregin-B subunit beta OS=Trimeresurus albolabris OX=8765 PE=1 SV=2 |
| 57 | 369 | D1MGU1|SLB\_PROJR | 40.28 | 7 | 0 | 0 | 0 | 0 | 0 | 0 | 7 | 0 | 0 |  |  |  |  |  |  | 5.4749E5 |  |  | 1 | 1 | 0 | 0 | 0 | 0 | 0 | 0 | 1 | 0 | 0 | N | 16834 | Snaclec jerdonibitin subunit beta OS=Protobothrops jerdonii OX=242841 PE=1 SV=1 |
| 57 | 380 | Q71RQ9|SLBB1\_TRIST | 40.28 | 7 | 0 | 0 | 0 | 0 | 0 | 0 | 7 | 0 | 0 |  |  |  |  |  |  | 5.4749E5 |  |  | 1 | 1 | 0 | 0 | 0 | 0 | 0 | 0 | 1 | 0 | 0 | N | 16806 | Snaclec stejaggregin-B subunit beta-1 OS=Trimeresurus stejnegeri OX=39682 PE=2 SV=1 |
| 57 | 381 | Q71RQ8|SLBB2\_TRIST | 40.28 | 7 | 0 | 0 | 0 | 0 | 0 | 0 | 7 | 0 | 0 |  |  |  |  |  |  | 5.4749E5 |  |  | 1 | 1 | 0 | 0 | 0 | 0 | 0 | 0 | 1 | 0 | 0 | N | 16820 | Snaclec stejaggregin-B subunit beta-2 OS=Trimeresurus stejnegeri OX=39682 PE=2 SV=1 |
| 61 | 287 | A8E2V8|PA2A\_TRIGS | 39.48 | 11 | 0 | 0 | 0 | 0 | 0 | 0 | 0 | 11 | 0 |  |  |  |  |  |  |  | 3.5116E5 |  | 1 | 1 | 0 | 0 | 0 | 0 | 0 | 0 | 0 | 1 | 0 | Y | 15689 | Acidic phospholipase A2 Tgc-E6 OS=Trimeresurus gracilis OX=109781 PE=1 SV=1 |
| 61 | 293 | Q800C2|PA2AG\_CROVV | 39.48 | 11 | 0 | 0 | 0 | 0 | 0 | 0 | 0 | 11 | 0 |  |  |  |  |  |  |  | 3.5116E5 |  | 1 | 1 | 0 | 0 | 0 | 0 | 0 | 0 | 0 | 1 | 0 | Y | 15556 | Acidic phospholipase A2 Cvv-E6g OS=Crotalus viridis viridis OX=8742 PE=2 SV=1 |
| 61 | 308 | Q800C3|PA2AF\_CROVV | 39.48 | 11 | 0 | 0 | 0 | 0 | 0 | 0 | 0 | 11 | 0 |  |  |  |  |  |  |  | 3.5116E5 |  | 1 | 1 | 0 | 0 | 0 | 0 | 0 | 0 | 0 | 1 | 0 | Y | 15642 | Acidic phospholipase A2 Cvv-E6f OS=Crotalus viridis viridis OX=8742 PE=1 SV=1 |
| 61 | 318 | Q7LZQ4|PA2A\_GLOUS | 39.48 | 12 | 0 | 0 | 0 | 0 | 0 | 0 | 0 | 12 | 0 |  |  |  |  |  |  |  | 3.5116E5 |  | 1 | 1 | 0 | 0 | 0 | 0 | 0 | 0 | 0 | 1 | 0 | Y | 13975 | Acidic phospholipase A2 OS=Gloydius ussuriensis OX=35671 PE=1 SV=1 |
| 61 | 319 | P14418|PA2A\_GLOHA | 39.48 | 12 | 0 | 0 | 0 | 0 | 0 | 0 | 0 | 12 | 0 |  |  |  |  |  |  |  | 3.5116E5 |  | 1 | 1 | 0 | 0 | 0 | 0 | 0 | 0 | 0 | 1 | 0 | Y | 13974 | Acidic phospholipase A2 OS=Gloydius halys OX=8714 PE=1 SV=1 |
| 61 | 320 | O42191|PA2A7\_GLOHA | 39.48 | 12 | 0 | 0 | 0 | 0 | 0 | 0 | 0 | 12 | 0 |  |  |  |  |  |  |  | 3.5116E5 |  | 1 | 1 | 0 | 0 | 0 | 0 | 0 | 0 | 0 | 1 | 0 | Y | 13927 | Acidic phospholipase A2 A OS=Gloydius halys OX=8714 PE=1 SV=2 |
| 68 | 325 | P0DL42|TXVE\_DABSI | 39.27 | 10 | 0 | 0 | 0 | 10 | 0 | 0 | 0 | 0 | 0 |  |  |  | 1.4239E7 |  |  |  |  |  | 1 | 1 | 0 | 0 | 0 | 1 | 0 | 0 | 0 | 0 | 0 | N | 12554 | Snake venom vascular endothelial growth factor toxin VR-1' OS=Daboia siamensis OX=343250 PE=1 SV=1 |
| 68 | 326 | P67861|TXVE\_DABRR | 39.27 | 8 | 0 | 0 | 0 | 8 | 0 | 0 | 0 | 0 | 0 |  |  |  | 1.4239E7 |  |  |  |  |  | 1 | 1 | 0 | 0 | 0 | 1 | 0 | 0 | 0 | 0 | 0 | N | 16278 | Snake venom vascular endothelial growth factor toxin VR-1 OS=Daboia russelii OX=8707 PE=1 SV=2 |
| total 76 proteins |
| --- |

  

A8CG86|PA2A1\_DABRR

back to list

  

| Protein Coverage
| Supporting Peptides
|

Protein Coverage:

Supporting Peptides:

| Peptide | Uniq | -10lgP | Mass | Length | ppm | m/z | z | RT | Fraction | Scan | Source File | Area F1 | Area F10 | Area F2 | Area F5 | Area F7A | Area F7B | Area F8A | Area F8B | Area F9 | #Feature | #Feature F1 | #Feature F10 | #Feature F2 | #Feature F5 | #Feature F7A | #Feature F7B | #Feature F8A | #Feature F8B | #Feature F9 | Start | End | PTM | AScore | Found By |
| --- | --- | --- | --- | --- | --- | --- | --- | --- | --- | --- | --- | --- | --- | --- | --- | --- | --- | --- | --- | --- | --- | --- | --- | --- | --- | --- | --- | --- | --- | --- | --- | --- | --- | --- | --- |
| R.AAAIC(+57.02)LGQNVNTYDK.N | N | 71.83 | 1636.7878 | 15 | 7.7 | 819.4014 | 2 | 38.92 | 8 | F8:17315 | DaRuWB\_F8B.raw |  |  |  |  | 1.6069E6 | 5.9E6 | 4.9743E7 | 4.0309E9 | 1.1367E6 | 10 | 0 | 0 | 0 | 0 | 1 | 1 | 1 | 6 | 1 | 107 | 121 | Carbamidomethylation | C5:Carbamidomethylation:1000.00 | PEAKS DB |
| K.EAVHSYAIYGC(+57.02)YC(+57.02)GWGGQGK.P | Y | 59.45 | 2261.9622 | 20 | 8.9 | 754.9957 | 3 | 45.84 | 8 | F8:22610 | DaRuWB\_F8B.raw |  |  |  |  |  |  |  | 3.7362E7 |  | 1 | 0 | 0 | 0 | 0 | 0 | 0 | 0 | 1 | 0 | 32 | 51 | Carbamidomethylation | C11:Carbamidomethylation:1000.00;C13:Carbamidomethylation:1000.00 | PEAKS DB |
| K.EAVHSYAIYGC(+57.02)YC(+57.02)GWGGQGKPQDATDR.C | Y | 56.23 | 3045.3132 | 27 | 8.0 | 762.3359 | 4 | 41.66 | 8 | F8:19276 | DaRuWB\_F8B.raw |  |  |  |  |  |  |  | 1.0442E8 |  | 2 | 0 | 0 | 0 | 0 | 0 | 0 | 0 | 2 | 0 | 32 | 58 | Carbamidomethylation | C11:Carbamidomethylation:1000.00;C13:Carbamidomethylation:1000.00 | PEAKS DB |
| C.LGQNVNTYDK.N | N | 53.56 | 1150.5619 | 10 | 7.6 | 576.2883 | 2 | 17.65 | 8 | F8:3600 | DaRuWB\_F8B.raw |  |  |  |  |  |  |  | 5.0031E7 |  | 1 | 0 | 0 | 0 | 0 | 0 | 0 | 0 | 1 | 0 | 112 | 121 |  |  | PEAKS DB |
| G.NLFQFAEM(+15.99)IVK.M | Y | 52.13 | 1354.6954 | 11 | 2.5 | 678.3552 | 2 | 78.51 | 7 | F7:41725 | DaRuWB\_F8A.raw |  |  |  |  |  |  | 3.472E6 | 3.1659E7 |  | 2 | 0 | 0 | 0 | 0 | 0 | 0 | 1 | 1 | 0 | 17 | 27 | Oxidation (M) | M8:Oxidation (M):1000.00 | PEAKS DB |
| R.C(+57.02)C(+57.02)FVHDC(+57.02)C(+57.02)YGTVNDC(+57.02)NPK.M | N | 49.12 | 2304.8479 | 18 | 3.5 | 769.2909 | 3 | 28.10 | 7 | F7:8813 | DaRuWB\_F8A.raw |  |  |  |  |  |  | 1.6135E7 | 9.4236E6 |  | 2 | 0 | 0 | 0 | 0 | 0 | 0 | 1 | 1 | 0 | 59 | 76 | Carbamidomethylation | C1:Carbamidomethylation:1000.00;C2:Carbamidomethylation:1000.00;C7:Carbamidomethylation:1000.00;C8:Carbamidomethylation:1000.00;C15:Carbamidomethylation:1000.00 | PEAKS DB |
| Y.C(+57.02)GWGGQGKPQDATDR.C | N | 48.43 | 1631.7111 | 15 | 7.5 | 816.8628 | 2 | 13.27 | 8 | F8:2695 | DaRuWB\_F8B.raw |  |  |  |  |  |  |  | 3.6964E7 |  | 2 | 0 | 0 | 0 | 0 | 0 | 0 | 0 | 2 | 0 | 44 | 58 | Carbamidomethylation | C1:Carbamidomethylation:1000.00 | PEAKS DB |
| H.DC(+57.02)C(+57.02)YGTVNDC(+57.02)NPK.M | N | 46.84 | 1601.5908 | 13 | 8.3 | 801.8033 | 2 | 20.71 | 8 | F8:5108 | DaRuWB\_F8B.raw |  |  |  |  |  |  |  | 2.14E6 |  | 1 | 0 | 0 | 0 | 0 | 0 | 0 | 0 | 1 | 0 | 64 | 76 | Carbamidomethylation | C2:Carbamidomethylation:1000.00;C3:Carbamidomethylation:1000.00;C10:Carbamidomethylation:1000.00 | PEAKS DB |
| K.EAVHSYAIYGC(+57.02)Y.C | N | 40.08 | 1431.6129 | 12 | 2.4 | 716.8139 | 2 | 45.05 | 7 | F7:19798 | DaRuWB\_F8A.raw |  |  |  |  |  |  | 4.5632E6 |  |  | 1 | 0 | 0 | 0 | 0 | 0 | 0 | 1 | 0 | 0 | 32 | 43 | Carbamidomethylation | C11:Carbamidomethylation:1000.00 | PEAKS DB |
| E.GNLFQFAEMIVK.M | Y | 39.13 | 1395.7220 | 12 | 6.5 | 698.8690 | 2 | 92.94 | 5 | F5:57076 | DaRuWB\_F7A.raw |  |  |  |  | 1.0171E6 |  |  |  |  | 1 | 0 | 0 | 0 | 0 | 1 | 0 | 0 | 0 | 0 | 16 | 27 |  |  | PEAKS DB |
| G.NLFQFAEMIVK.M | Y | 39.01 | 1338.7006 | 11 | 2.9 | 670.3580 | 2 | 85.92 | 7 | F7:44793 | DaRuWB\_F8A.raw |  |  |  |  |  |  | 3.6639E6 |  |  | 1 | 0 | 0 | 0 | 0 | 0 | 0 | 1 | 0 | 0 | 17 | 27 |  |  | PEAKS DB |
| total 11 peptides |
| --- |

P04264|K2C1\_HUMAN

back to list

  

| Protein Coverage
| Supporting Peptides
|

Protein Coverage:

Supporting Peptides:

| Peptide | Uniq | -10lgP | Mass | Length | ppm | m/z | z | RT | Fraction | Scan | Source File | Area F1 | Area F10 | Area F2 | Area F5 | Area F7A | Area F7B | Area F8A | Area F8B | Area F9 | #Feature | #Feature F1 | #Feature F10 | #Feature F2 | #Feature F5 | #Feature F7A | #Feature F7B | #Feature F8A | #Feature F8B | #Feature F9 | Start | End | PTM | AScore | Found By |
| --- | --- | --- | --- | --- | --- | --- | --- | --- | --- | --- | --- | --- | --- | --- | --- | --- | --- | --- | --- | --- | --- | --- | --- | --- | --- | --- | --- | --- | --- | --- | --- | --- | --- | --- | --- |
| K.LNDLEDALQQAK.E | Y | 63.00 | 1356.6885 | 12 | 0.1 | 679.3516 | 2 | 47.17 | 2 | F2:10204 | DaRuWB\_F10.raw |  | 6.9891E6 |  |  |  |  |  |  |  | 1 | 0 | 1 | 0 | 0 | 0 | 0 | 0 | 0 | 0 | 444 | 455 |  |  | PEAKS DB |
| R.SLDLDSIIAEVK.A | N | 61.51 | 1301.7078 | 12 | 0.8 | 651.8617 | 2 | 75.59 | 2 | F2:17300 | DaRuWB\_F10.raw |  | 7.8623E6 |  |  |  | 7.5254E4 |  |  | 3.1084E6 | 3 | 0 | 1 | 0 | 0 | 0 | 1 | 0 | 0 | 1 | 344 | 355 |  |  | PEAKS DB |
| R.SGGGFSSGSAGIINYQR.R | Y | 59.31 | 1656.7855 | 17 | 8.4 | 829.4008 | 2 | 40.67 | 8 | F8:18600 | DaRuWB\_F8B.raw |  |  |  |  |  |  |  | 5.0285E5 |  | 1 | 0 | 0 | 0 | 0 | 0 | 0 | 0 | 1 | 0 | 13 | 29 |  |  | PEAKS DB |
| R.TNAENEFVTIK.K | Y | 56.52 | 1264.6299 | 11 | 0.3 | 633.3224 | 2 | 37.18 | 4 | F4:9090 | DaRuWB\_F5.raw |  |  |  | 4.883E6 |  |  |  |  |  | 1 | 0 | 0 | 0 | 1 | 0 | 0 | 0 | 0 | 0 | 278 | 288 |  |  | PEAKS DB |
| K.WELLQQVDTSTR.T | Y | 55.24 | 1474.7416 | 12 | 0.7 | 738.3786 | 2 | 55.81 | 9 | F9:11455 | DaRuWB\_F9.raw |  | 4.0778E6 | 5.7923E6 | 7.7076E5 | 2.7796E7 |  |  |  | 2.03E6 | 6 | 0 | 1 | 1 | 1 | 2 | 0 | 0 | 0 | 1 | 212 | 223 |  |  | PEAKS DB |
| K.SLNNQFASFIDK.V | Y | 54.76 | 1382.6830 | 12 | 0.5 | 692.3491 | 2 | 56.43 | 9 | F9:11645 | DaRuWB\_F9.raw |  | 4.6419E6 | 6.6522E6 |  |  |  |  |  | 2.0456E6 | 3 | 0 | 1 | 1 | 0 | 0 | 0 | 0 | 0 | 1 | 186 | 197 |  |  | PEAKS DB |
| K.AEAESLYQSK.Y | Y | 52.40 | 1124.5349 | 10 | 0.2 | 563.2748 | 2 | 19.83 | 2 | F2:3584 | DaRuWB\_F10.raw |  | 2.0175E6 |  |  |  |  |  |  |  | 1 | 0 | 1 | 0 | 0 | 0 | 0 | 0 | 0 | 0 | 367 | 376 |  |  | PEAKS DB |
| K.YEELQITAGR.H | N | 51.54 | 1178.5931 | 10 | 2.0 | 590.3037 | 2 | 36.07 | 7 | F7:14101 | DaRuWB\_F8A.raw | 1.4837E6 | 4.9035E6 | 6.4817E6 |  |  | 1.0712E7 | 1.4221E7 | 2.9188E6 | 1.7883E6 | 7 | 1 | 1 | 1 | 0 | 0 | 1 | 1 | 1 | 1 | 377 | 386 |  |  | PEAKS DB |
| R.FLEQQNQVLQTK.W | N | 41.80 | 1474.7780 | 12 | 9.0 | 738.3973 | 2 | 35.43 | 8 | F8:14439 | DaRuWB\_F8B.raw |  |  |  |  |  |  |  | 4.5765E6 |  | 1 | 0 | 0 | 0 | 0 | 0 | 0 | 0 | 1 | 0 | 200 | 211 |  |  | PEAKS DB |
| total 9 peptides |
| --- |

A5A6M6|K2C1\_PANTR

back to list

  

| Protein Coverage
| Supporting Peptides
|

Protein Coverage:

Supporting Peptides:

| Peptide | Uniq | -10lgP | Mass | Length | ppm | m/z | z | RT | Fraction | Scan | Source File | Area F1 | Area F10 | Area F2 | Area F5 | Area F7A | Area F7B | Area F8A | Area F8B | Area F9 | #Feature | #Feature F1 | #Feature F10 | #Feature F2 | #Feature F5 | #Feature F7A | #Feature F7B | #Feature F8A | #Feature F8B | #Feature F9 | Start | End | PTM | AScore | Found By |
| --- | --- | --- | --- | --- | --- | --- | --- | --- | --- | --- | --- | --- | --- | --- | --- | --- | --- | --- | --- | --- | --- | --- | --- | --- | --- | --- | --- | --- | --- | --- | --- | --- | --- | --- | --- |
| K.LNDLEDALQQAK.E | Y | 63.00 | 1356.6885 | 12 | 0.1 | 679.3516 | 2 | 47.17 | 2 | F2:10204 | DaRuWB\_F10.raw |  | 6.9891E6 |  |  |  |  |  |  |  | 1 | 0 | 1 | 0 | 0 | 0 | 0 | 0 | 0 | 0 | 439 | 450 |  |  | PEAKS DB |
| R.SLDLDSIIAEVK.A | N | 61.51 | 1301.7078 | 12 | 0.8 | 651.8617 | 2 | 75.59 | 2 | F2:17300 | DaRuWB\_F10.raw |  | 7.8623E6 |  |  |  | 7.5254E4 |  |  | 3.1084E6 | 3 | 0 | 1 | 0 | 0 | 0 | 1 | 0 | 0 | 1 | 339 | 350 |  |  | PEAKS DB |
| R.SGGGFSSGSAGIINYQR.R | Y | 59.31 | 1656.7855 | 17 | 8.4 | 829.4008 | 2 | 40.67 | 8 | F8:18600 | DaRuWB\_F8B.raw |  |  |  |  |  |  |  | 5.0285E5 |  | 1 | 0 | 0 | 0 | 0 | 0 | 0 | 0 | 1 | 0 | 13 | 29 |  |  | PEAKS DB |
| R.TNAENEFVTIK.K | Y | 56.52 | 1264.6299 | 11 | 0.3 | 633.3224 | 2 | 37.18 | 4 | F4:9090 | DaRuWB\_F5.raw |  |  |  | 4.883E6 |  |  |  |  |  | 1 | 0 | 0 | 0 | 1 | 0 | 0 | 0 | 0 | 0 | 273 | 283 |  |  | PEAKS DB |
| K.WELLQQVDTSTR.T | Y | 55.24 | 1474.7416 | 12 | 0.7 | 738.3786 | 2 | 55.81 | 9 | F9:11455 | DaRuWB\_F9.raw |  | 4.0778E6 | 5.7923E6 | 7.7076E5 | 2.7796E7 |  |  |  | 2.03E6 | 6 | 0 | 1 | 1 | 1 | 2 | 0 | 0 | 0 | 1 | 207 | 218 |  |  | PEAKS DB |
| K.SLNNQFASFIDK.V | Y | 54.76 | 1382.6830 | 12 | 0.5 | 692.3491 | 2 | 56.43 | 9 | F9:11645 | DaRuWB\_F9.raw |  | 4.6419E6 | 6.6522E6 |  |  |  |  |  | 2.0456E6 | 3 | 0 | 1 | 1 | 0 | 0 | 0 | 0 | 0 | 1 | 181 | 192 |  |  | PEAKS DB |
| K.AEAESLYQSK.Y | Y | 52.40 | 1124.5349 | 10 | 0.2 | 563.2748 | 2 | 19.83 | 2 | F2:3584 | DaRuWB\_F10.raw |  | 2.0175E6 |  |  |  |  |  |  |  | 1 | 0 | 1 | 0 | 0 | 0 | 0 | 0 | 0 | 0 | 362 | 371 |  |  | PEAKS DB |
| K.YEELQITAGR.H | N | 51.54 | 1178.5931 | 10 | 2.0 | 590.3037 | 2 | 36.07 | 7 | F7:14101 | DaRuWB\_F8A.raw | 1.4837E6 | 4.9035E6 | 6.4817E6 |  |  | 1.0712E7 | 1.4221E7 | 2.9188E6 | 1.7883E6 | 7 | 1 | 1 | 1 | 0 | 0 | 1 | 1 | 1 | 1 | 372 | 381 |  |  | PEAKS DB |
| R.FLEQQNQVLQTK.W | N | 41.80 | 1474.7780 | 12 | 9.0 | 738.3973 | 2 | 35.43 | 8 | F8:14439 | DaRuWB\_F8B.raw |  |  |  |  |  |  |  | 4.5765E6 |  | 1 | 0 | 0 | 0 | 0 | 0 | 0 | 0 | 1 | 0 | 195 | 206 |  |  | PEAKS DB |
| total 9 peptides |
| --- |

A8CG89|PA2B1\_DABRR

back to list

  

| Protein Coverage
| Supporting Peptides
|

Protein Coverage:

Supporting Peptides:

| Peptide | Uniq | -10lgP | Mass | Length | ppm | m/z | z | RT | Fraction | Scan | Source File | Area F1 | Area F10 | Area F2 | Area F5 | Area F7A | Area F7B | Area F8A | Area F8B | Area F9 | #Feature | #Feature F1 | #Feature F10 | #Feature F2 | #Feature F5 | #Feature F7A | #Feature F7B | #Feature F8A | #Feature F8B | #Feature F9 | Start | End | PTM | AScore | Found By |
| --- | --- | --- | --- | --- | --- | --- | --- | --- | --- | --- | --- | --- | --- | --- | --- | --- | --- | --- | --- | --- | --- | --- | --- | --- | --- | --- | --- | --- | --- | --- | --- | --- | --- | --- | --- |
| R.VAAIC(+57.02)LGQNVNTYNK.G | Y | 78.75 | 1663.8352 | 15 | 6.8 | 832.9260 | 2 | 40.50 | 5 | F5:19394 | DaRuWB\_F7A.raw |  |  |  |  | 4.111E7 | 7.5452E9 | 1.1103E7 |  |  | 11 | 0 | 0 | 0 | 0 | 1 | 9 | 1 | 0 | 0 | 107 | 121 | Carbamidomethylation | C5:Carbamidomethylation:1000.00 | PEAKS DB |
| Y.GC(+57.02)YC(+57.02)GWGGQGTPK.D | N | 61.85 | 1426.5758 | 13 | 2.6 | 714.2950 | 2 | 27.48 | 6 | F6:10372 | DaRuWB\_F7B.raw |  |  |  |  |  | 1.3458E8 |  |  |  | 1 | 0 | 0 | 0 | 0 | 0 | 1 | 0 | 0 | 0 | 41 | 53 | Carbamidomethylation | C2:Carbamidomethylation:1000.00;C4:Carbamidomethylation:1000.00 | PEAKS DB |
| K.GYMFLSSYYC(+57.02)R.Q | Y | 61.23 | 1445.6107 | 11 | 3.3 | 723.8130 | 2 | 59.07 | 6 | F6:35464 | DaRuWB\_F7B.raw |  |  |  |  |  | 3.4313E8 |  |  |  | 1 | 0 | 0 | 0 | 0 | 0 | 1 | 0 | 0 | 0 | 122 | 132 | Carbamidomethylation | C10:Carbamidomethylation:1000.00 | PEAKS DB |
| C.LGQNVNTYNK.G | N | 56.02 | 1149.5779 | 10 | 2.9 | 575.7963 | 2 | 14.40 | 6 | F6:2612 | DaRuWB\_F7B.raw |  |  |  |  |  | 1.4787E7 |  |  |  | 1 | 0 | 0 | 0 | 0 | 0 | 1 | 0 | 0 | 0 | 112 | 121 |  |  | PEAKS DB |
| K.LVEYSYSYR.T | N | 49.02 | 1178.5608 | 9 | 3.2 | 590.2879 | 2 | 34.18 | 6 | F6:15740 | DaRuWB\_F7B.raw |  |  |  |  | 2.9391E7 | 3.5022E9 |  |  |  | 2 | 0 | 0 | 0 | 0 | 1 | 1 | 0 | 0 | 0 | 77 | 85 |  |  | PEAKS DB |
| R.C(+57.02)C(+57.02)FVHDC(+57.02)C(+57.02)YAR.V | N | 45.18 | 1546.5574 | 11 | 2.4 | 516.5262 | 3 | 21.08 | 6 | F6:6004 | DaRuWB\_F7B.raw |  |  |  |  |  | 2.5948E9 |  |  |  | 1 | 0 | 0 | 0 | 0 | 0 | 1 | 0 | 0 | 0 | 59 | 69 | Carbamidomethylation | C1:Carbamidomethylation:1000.00;C2:Carbamidomethylation:1000.00;C7:Carbamidomethylation:1000.00;C8:Carbamidomethylation:1000.00 | PEAKS DB |
| C.FVHDC(+57.02)C(+57.02)YAR.V | N | 42.53 | 1226.4961 | 9 | 3.3 | 614.2557 | 2 | 12.76 | 6 | F6:1984 | DaRuWB\_F7B.raw |  |  |  |  |  | 3.9205E5 |  |  |  | 1 | 0 | 0 | 0 | 0 | 0 | 1 | 0 | 0 | 0 | 61 | 69 | Carbamidomethylation | C5:Carbamidomethylation:1000.00;C6:Carbamidomethylation:1000.00 | PEAKS DB |
| total 7 peptides |
| --- |

Q2ES50|VKT1\_DABRR

back to list

  

| Protein Coverage
| Supporting Peptides
|

Protein Coverage:

Supporting Peptides:

| Peptide | Uniq | -10lgP | Mass | Length | ppm | m/z | z | RT | Fraction | Scan | Source File | Area F1 | Area F10 | Area F2 | Area F5 | Area F7A | Area F7B | Area F8A | Area F8B | Area F9 | #Feature | #Feature F1 | #Feature F10 | #Feature F2 | #Feature F5 | #Feature F7A | #Feature F7B | #Feature F8A | #Feature F8B | #Feature F9 | Start | End | PTM | AScore | Found By |
| --- | --- | --- | --- | --- | --- | --- | --- | --- | --- | --- | --- | --- | --- | --- | --- | --- | --- | --- | --- | --- | --- | --- | --- | --- | --- | --- | --- | --- | --- | --- | --- | --- | --- | --- | --- |
| S.GHDRPTFC(+57.02)NLAPESGR.C | Y | 67.49 | 1812.8325 | 16 | 8.7 | 605.2853 | 3 | 26.04 | 3 | F3:8720 | DaRuWB\_F2.raw | 4.7137E7 |  | 6.915E9 |  |  |  |  |  |  | 6 | 2 | 0 | 4 | 0 | 0 | 0 | 0 | 0 | 0 | 24 | 39 | Carbamidomethylation | C8:Carbamidomethylation:1000.00 | PEAKS DB |
| G.HDRPTFC(+57.02)NLAPESGR.C | N | 63.39 | 1755.8110 | 15 | 8.2 | 586.2778 | 3 | 27.80 | 3 | F3:9849 | DaRuWB\_F2.raw | 9.4426E7 |  | 1.1198E9 |  |  |  |  |  |  | 6 | 2 | 0 | 4 | 0 | 0 | 0 | 0 | 0 | 0 | 25 | 39 | Carbamidomethylation | C7:Carbamidomethylation:1000.00 | PEAKS DB |
| T.FC(+57.02)NLAPESGR.C | N | 58.26 | 1149.5237 | 10 | 9.2 | 575.7699 | 2 | 25.76 | 3 | F3:8637 | DaRuWB\_F2.raw | 2.8445E6 |  | 1.5101E7 | 1.2778E5 |  |  |  |  |  | 3 | 1 | 0 | 1 | 1 | 0 | 0 | 0 | 0 | 0 | 30 | 39 | Carbamidomethylation | C2:Carbamidomethylation:1000.00 | PEAKS DB |
| F.FYGGC(+57.02)GGNANNFETR.D | N | 55.20 | 1662.6844 | 15 | 8.2 | 832.3499 | 2 | 30.77 | 3 | F3:11661 | DaRuWB\_F2.raw |  |  | 2.2104E8 |  |  |  |  |  |  | 1 | 0 | 0 | 1 | 0 | 0 | 0 | 0 | 0 | 0 | 60 | 74 | Carbamidomethylation | C5:Carbamidomethylation:1000.00 | PEAKS DB |
| F.YGGC(+57.02)GGNANNFETR.D | N | 51.77 | 1515.6161 | 14 | 8.1 | 758.8156 | 2 | 20.19 | 3 | F3:5193 | DaRuWB\_F2.raw |  |  | 3.2933E8 |  |  |  |  |  |  | 1 | 0 | 0 | 1 | 0 | 0 | 0 | 0 | 0 | 0 | 61 | 74 | Carbamidomethylation | C4:Carbamidomethylation:1000.00 | PEAKS DB |
| P.TFC(+57.02)NLAPESGR.C | N | 49.39 | 1250.5713 | 11 | 8.1 | 626.2933 | 2 | 30.31 | 1 | F1:11995 | DaRuWB\_F1.raw | 3.2129E6 |  |  |  |  |  |  |  |  | 1 | 1 | 0 | 0 | 0 | 0 | 0 | 0 | 0 | 0 | 29 | 39 | Carbamidomethylation | C3:Carbamidomethylation:1000.00 | PEAKS DB |
| G.C(+57.02)GGNANNFETR.D | N | 43.34 | 1238.5098 | 11 | 8.3 | 620.2625 | 2 | 12.95 | 3 | F3:2045 | DaRuWB\_F2.raw |  |  | 8.3524E5 |  |  |  |  |  |  | 1 | 0 | 0 | 1 | 0 | 0 | 0 | 0 | 0 | 0 | 64 | 74 | Carbamidomethylation | C1:Carbamidomethylation:1000.00 | PEAKS DB |
| total 7 peptides |
| --- |

A8Y7P0|VKTB7\_DABSI

back to list

  

| Protein Coverage
| Supporting Peptides
|

Protein Coverage:

Supporting Peptides:

| Peptide | Uniq | -10lgP | Mass | Length | ppm | m/z | z | RT | Fraction | Scan | Source File | Area F1 | Area F10 | Area F2 | Area F5 | Area F7A | Area F7B | Area F8A | Area F8B | Area F9 | #Feature | #Feature F1 | #Feature F10 | #Feature F2 | #Feature F5 | #Feature F7A | #Feature F7B | #Feature F8A | #Feature F8B | #Feature F9 | Start | End | PTM | AScore | Found By |
| --- | --- | --- | --- | --- | --- | --- | --- | --- | --- | --- | --- | --- | --- | --- | --- | --- | --- | --- | --- | --- | --- | --- | --- | --- | --- | --- | --- | --- | --- | --- | --- | --- | --- | --- | --- |
| S.GHDRPTFC(+57.02)NLAPESGR.C | Y | 67.49 | 1812.8325 | 16 | 8.7 | 605.2853 | 3 | 26.04 | 3 | F3:8720 | DaRuWB\_F2.raw | 4.7137E7 |  | 6.915E9 |  |  |  |  |  |  | 6 | 2 | 0 | 4 | 0 | 0 | 0 | 0 | 0 | 0 | 24 | 39 | Carbamidomethylation | C8:Carbamidomethylation:1000.00 | PEAKS DB |
| G.HDRPTFC(+57.02)NLAPESGR.C | N | 63.39 | 1755.8110 | 15 | 8.2 | 586.2778 | 3 | 27.80 | 3 | F3:9849 | DaRuWB\_F2.raw | 9.4426E7 |  | 1.1198E9 |  |  |  |  |  |  | 6 | 2 | 0 | 4 | 0 | 0 | 0 | 0 | 0 | 0 | 25 | 39 | Carbamidomethylation | C7:Carbamidomethylation:1000.00 | PEAKS DB |
| T.FC(+57.02)NLAPESGR.C | N | 58.26 | 1149.5237 | 10 | 9.2 | 575.7699 | 2 | 25.76 | 3 | F3:8637 | DaRuWB\_F2.raw | 2.8445E6 |  | 1.5101E7 | 1.2778E5 |  |  |  |  |  | 3 | 1 | 0 | 1 | 1 | 0 | 0 | 0 | 0 | 0 | 30 | 39 | Carbamidomethylation | C2:Carbamidomethylation:1000.00 | PEAKS DB |
| F.FYGGC(+57.02)GGNANNFETR.D | N | 55.20 | 1662.6844 | 15 | 8.2 | 832.3499 | 2 | 30.77 | 3 | F3:11661 | DaRuWB\_F2.raw |  |  | 2.2104E8 |  |  |  |  |  |  | 1 | 0 | 0 | 1 | 0 | 0 | 0 | 0 | 0 | 0 | 60 | 74 | Carbamidomethylation | C5:Carbamidomethylation:1000.00 | PEAKS DB |
| F.YGGC(+57.02)GGNANNFETR.D | N | 51.77 | 1515.6161 | 14 | 8.1 | 758.8156 | 2 | 20.19 | 3 | F3:5193 | DaRuWB\_F2.raw |  |  | 3.2933E8 |  |  |  |  |  |  | 1 | 0 | 0 | 1 | 0 | 0 | 0 | 0 | 0 | 0 | 61 | 74 | Carbamidomethylation | C4:Carbamidomethylation:1000.00 | PEAKS DB |
| P.TFC(+57.02)NLAPESGR.C | N | 49.39 | 1250.5713 | 11 | 8.1 | 626.2933 | 2 | 30.31 | 1 | F1:11995 | DaRuWB\_F1.raw | 3.2129E6 |  |  |  |  |  |  |  |  | 1 | 1 | 0 | 0 | 0 | 0 | 0 | 0 | 0 | 0 | 29 | 39 | Carbamidomethylation | C3:Carbamidomethylation:1000.00 | PEAKS DB |
| G.C(+57.02)GGNANNFETR.D | N | 43.34 | 1238.5098 | 11 | 8.3 | 620.2625 | 2 | 12.95 | 3 | F3:2045 | DaRuWB\_F2.raw |  |  | 8.3524E5 |  |  |  |  |  |  | 1 | 0 | 0 | 1 | 0 | 0 | 0 | 0 | 0 | 0 | 64 | 74 | Carbamidomethylation | C1:Carbamidomethylation:1000.00 | PEAKS DB |
| total 7 peptides |
| --- |

P35527|K1C9\_HUMAN

back to list

  

| Protein Coverage
| Supporting Peptides
|

Protein Coverage:

Supporting Peptides:

| Peptide | Uniq | -10lgP | Mass | Length | ppm | m/z | z | RT | Fraction | Scan | Source File | Area F1 | Area F10 | Area F2 | Area F5 | Area F7A | Area F7B | Area F8A | Area F8B | Area F9 | #Feature | #Feature F1 | #Feature F10 | #Feature F2 | #Feature F5 | #Feature F7A | #Feature F7B | #Feature F8A | #Feature F8B | #Feature F9 | Start | End | PTM | AScore | Found By |
| --- | --- | --- | --- | --- | --- | --- | --- | --- | --- | --- | --- | --- | --- | --- | --- | --- | --- | --- | --- | --- | --- | --- | --- | --- | --- | --- | --- | --- | --- | --- | --- | --- | --- | --- | --- |
| R.FSSSSGYGGGSSR.V | Y | 58.22 | 1234.5214 | 13 | 1.0 | 618.2686 | 2 | 12.72 | 4 | F4:1966 | DaRuWB\_F5.raw | 2.3583E5 | 1.2366E6 | 7.9395E5 | 3.151E5 | 4.0541E5 | 4.9385E5 | 7.6551E5 | 4.263E5 |  | 9 | 1 | 1 | 1 | 1 | 1 | 1 | 2 | 1 | 0 | 47 | 59 |  |  | PEAKS DB |
| R.QGVDADINGLR.Q | Y | 56.14 | 1156.5836 | 11 | 5.7 | 579.2992 | 2 | 34.71 | 5 | F5:15260 | DaRuWB\_F7A.raw |  |  |  | 3.3679E6 | 8.9693E6 | 8.6289E6 | 3.991E6 |  | 7.8101E5 | 5 | 0 | 0 | 0 | 1 | 1 | 1 | 1 | 0 | 1 | 251 | 261 |  |  | PEAKS DB |
| K.NYSPYYNTIDDLK.D | Y | 56.04 | 1604.7358 | 13 | 5.9 | 803.3755 | 2 | 49.71 | 5 | F5:26747 | DaRuWB\_F7A.raw |  |  |  |  | 2.7757E6 |  |  |  |  | 1 | 0 | 0 | 0 | 0 | 1 | 0 | 0 | 0 | 0 | 200 | 212 |  |  | PEAKS DB |
| F.SASSLGGGFGGGSR.G | Y | 48.16 | 1195.5581 | 14 | -0.7 | 598.7859 | 2 | 24.24 | 4 | F4:5185 | DaRuWB\_F5.raw |  |  |  | 5.2487E5 |  |  |  |  |  | 1 | 0 | 0 | 0 | 1 | 0 | 0 | 0 | 0 | 0 | 82 | 95 |  |  | PEAKS DB |
| K.TLNDMRQEYEQLIAK.N | Y | 48.11 | 1850.9197 | 15 | 2.9 | 617.9810 | 3 | 54.18 | 7 | F7:26137 | DaRuWB\_F8A.raw |  |  |  |  |  |  | 2.5916E6 |  |  | 1 | 0 | 0 | 0 | 0 | 0 | 0 | 1 | 0 | 0 | 322 | 336 |  |  | PEAKS DB |
| K.VQALEEANNDLENK.I | Y | 47.48 | 1585.7583 | 14 | 8.2 | 793.8870 | 2 | 31.76 | 8 | F8:11545 | DaRuWB\_F8B.raw |  |  |  |  |  |  |  | 1.8719E6 |  | 1 | 0 | 0 | 0 | 0 | 0 | 0 | 0 | 1 | 0 | 171 | 184 |  |  | PEAKS DB |
| T.QIEHEVSSSGQEVQSSAK.E | Y | 43.25 | 1928.9075 | 18 | 3.2 | 643.9771 | 3 | 14.73 | 7 | F7:2551 | DaRuWB\_F8A.raw |  |  |  |  |  |  | 2.3132E5 |  |  | 1 | 0 | 0 | 0 | 0 | 0 | 0 | 1 | 0 | 0 | 351 | 368 |  |  | PEAKS DB |
| M.IQEQISNLEAQITDVR.Q | Y | 41.79 | 1855.9639 | 16 | 3.8 | 619.6625 | 3 | 59.03 | 6 | F6:35447 | DaRuWB\_F7B.raw |  |  |  |  |  | 6.0425E5 |  |  |  | 1 | 0 | 0 | 0 | 0 | 0 | 1 | 0 | 0 | 0 | 412 | 427 |  |  | PEAKS DB |
| total 8 peptides |
| --- |

P0DPS3|VASP1\_VIPAA

back to list

  

| Protein Coverage
| Supporting Peptides
|

Protein Coverage:

Supporting Peptides:

| Peptide | Uniq | -10lgP | Mass | Length | ppm | m/z | z | RT | Fraction | Scan | Source File | Area F1 | Area F10 | Area F2 | Area F5 | Area F7A | Area F7B | Area F8A | Area F8B | Area F9 | #Feature | #Feature F1 | #Feature F10 | #Feature F2 | #Feature F5 | #Feature F7A | #Feature F7B | #Feature F8A | #Feature F8B | #Feature F9 | Start | End | PTM | AScore | Found By |
| --- | --- | --- | --- | --- | --- | --- | --- | --- | --- | --- | --- | --- | --- | --- | --- | --- | --- | --- | --- | --- | --- | --- | --- | --- | --- | --- | --- | --- | --- | --- | --- | --- | --- | --- | --- |
| VIGGDEC(+57.02)NINEHPFLVALHTAR.X | Y | 78.46 | 2461.2173 | 22 | 2.6 | 616.3115 | 4 | 48.75 | 6 | F6:27122 | DaRuWB\_F7B.raw |  |  |  |  | 6.1274E7 | 2.1002E8 | 8.1222E6 |  |  | 4 | 0 | 0 | 0 | 0 | 1 | 2 | 1 | 0 | 0 | 1 | 22 | Carbamidomethylation | C7:Carbamidomethylation:1000.00 | PEAKS DB |
| R.TLC(+57.02)AGILQGGIDSC(+57.02)KG.I | Y | 59.22 | 1648.7913 | 16 | 3.6 | 825.4036 | 2 | 47.74 | 6 | F6:26385 | DaRuWB\_F7B.raw |  |  |  |  |  | 4.1315E6 |  |  |  | 1 | 0 | 0 | 0 | 0 | 0 | 1 | 0 | 0 | 0 | 165 | 180 | Carbamidomethylation | C3:Carbamidomethylation:1000.00;C14:Carbamidomethylation:1000.00 | PEAKS DB |
| C.AGTLINQEWVLTAAR.C | N | 52.27 | 1641.8838 | 15 | 6.3 | 821.9498 | 2 | 63.26 | 5 | F5:37816 | DaRuWB\_F7A.raw |  |  |  |  | 9.6221E5 | 3.8518E6 |  |  |  | 2 | 0 | 0 | 0 | 0 | 1 | 1 | 0 | 0 | 0 | 29 | 43 |  |  | PEAKS DB |
| R.FYC(+57.02)AGTLINQEWVLTAAR.C | N | 42.81 | 2112.0461 | 18 | 3.5 | 705.0232 | 3 | 111.52 | 6 | F6:70106 | DaRuWB\_F7B.raw |  |  |  |  | 2.7992E6 | 7.9821E5 |  |  |  | 2 | 0 | 0 | 0 | 0 | 1 | 1 | 0 | 0 | 0 | 26 | 43 | Carbamidomethylation | C3:Carbamidomethylation:1000.00 | PEAKS DB |
| K.VTYPDVPH.C | N | 39.34 | 926.4498 | 8 | 2.3 | 464.2319 | 2 | 32.18 | 6 | F6:14040 | DaRuWB\_F7B.raw |  |  |  |  |  | 1.8876E7 |  |  |  | 1 | 0 | 0 | 0 | 0 | 0 | 1 | 0 | 0 | 0 | 133 | 140 |  |  | PEAKS DB |
| total 5 peptides |
| --- |

P31100|PA2A7\_DABSI

back to list

  

| Protein Coverage
| Supporting Peptides
|

Protein Coverage:

Supporting Peptides:

| Peptide | Uniq | -10lgP | Mass | Length | ppm | m/z | z | RT | Fraction | Scan | Source File | Area F1 | Area F10 | Area F2 | Area F5 | Area F7A | Area F7B | Area F8A | Area F8B | Area F9 | #Feature | #Feature F1 | #Feature F10 | #Feature F2 | #Feature F5 | #Feature F7A | #Feature F7B | #Feature F8A | #Feature F8B | #Feature F9 | Start | End | PTM | AScore | Found By |
| --- | --- | --- | --- | --- | --- | --- | --- | --- | --- | --- | --- | --- | --- | --- | --- | --- | --- | --- | --- | --- | --- | --- | --- | --- | --- | --- | --- | --- | --- | --- | --- | --- | --- | --- | --- |
| R.AAAIC(+57.02)LGQNVNTYDK.N | N | 71.83 | 1636.7878 | 15 | 7.7 | 819.4014 | 2 | 38.92 | 8 | F8:17315 | DaRuWB\_F8B.raw |  |  |  |  | 1.6069E6 | 5.9E6 | 4.9743E7 | 4.0309E9 | 1.1367E6 | 10 | 0 | 0 | 0 | 0 | 1 | 1 | 1 | 6 | 1 | 107 | 121 | Carbamidomethylation | C5:Carbamidomethylation:1000.00 | PEAKS DB |
| K.EVVHSYAIYGC(+57.02)YC(+57.02)GWGGQGR.A | Y | 56.94 | 2317.9998 | 20 | 8.6 | 773.6747 | 3 | 48.35 | 8 | F8:24968 | DaRuWB\_F8B.raw |  |  |  |  |  |  |  | 3.7914E6 |  | 1 | 0 | 0 | 0 | 0 | 0 | 0 | 0 | 1 | 0 | 32 | 51 | Carbamidomethylation | C11:Carbamidomethylation:1000.00;C13:Carbamidomethylation:1000.00 | PEAKS DB |
| G.NLFQFGEMILEK.T | Y | 53.88 | 1467.7432 | 12 | 8.3 | 734.8795 | 2 | 82.46 | 8 | F8:50783 | DaRuWB\_F8B.raw |  |  |  |  |  |  |  | 4.6367E7 |  | 1 | 0 | 0 | 0 | 0 | 0 | 0 | 0 | 1 | 0 | 17 | 28 |  |  | PEAKS DB |
| C.LGQNVNTYDK.N | N | 53.56 | 1150.5619 | 10 | 7.6 | 576.2883 | 2 | 17.65 | 8 | F8:3600 | DaRuWB\_F8B.raw |  |  |  |  |  |  |  | 5.0031E7 |  | 1 | 0 | 0 | 0 | 0 | 0 | 0 | 0 | 1 | 0 | 112 | 121 |  |  | PEAKS DB |
| R.C(+57.02)C(+57.02)FVHDC(+57.02)C(+57.02)YGTVNDC(+57.02)NPK.T | N | 49.12 | 2304.8479 | 18 | 3.5 | 769.2909 | 3 | 28.10 | 7 | F7:8813 | DaRuWB\_F8A.raw |  |  |  |  |  |  | 1.6135E7 | 9.4236E6 |  | 2 | 0 | 0 | 0 | 0 | 0 | 0 | 1 | 1 | 0 | 59 | 76 | Carbamidomethylation | C1:Carbamidomethylation:1000.00;C2:Carbamidomethylation:1000.00;C7:Carbamidomethylation:1000.00;C8:Carbamidomethylation:1000.00;C15:Carbamidomethylation:1000.00 | PEAKS DB |
| H.DC(+57.02)C(+57.02)YGTVNDC(+57.02)NPK.T | N | 46.84 | 1601.5908 | 13 | 8.3 | 801.8033 | 2 | 20.71 | 8 | F8:5108 | DaRuWB\_F8B.raw |  |  |  |  |  |  |  | 2.14E6 |  | 1 | 0 | 0 | 0 | 0 | 0 | 0 | 0 | 1 | 0 | 64 | 76 | Carbamidomethylation | C2:Carbamidomethylation:1000.00;C3:Carbamidomethylation:1000.00;C10:Carbamidomethylation:1000.00 | PEAKS DB |
| G.NLFQFGEM(+15.99)ILEK.T | Y | 42.33 | 1483.7380 | 12 | 2.7 | 742.8766 | 2 | 77.08 | 7 | F7:40920 | DaRuWB\_F8A.raw |  |  |  |  |  |  | 1.6264E6 |  |  | 1 | 0 | 0 | 0 | 0 | 0 | 0 | 1 | 0 | 0 | 17 | 28 | Oxidation (M) | M8:Oxidation (M):1000.00 | PEAKS DB |
| total 7 peptides |
| --- |

P18965|VSPG\_DABSI

back to list

  

| Protein Coverage
| Supporting Peptides
|

Protein Coverage:

Supporting Peptides:

| Peptide | Uniq | -10lgP | Mass | Length | ppm | m/z | z | RT | Fraction | Scan | Source File | Area F1 | Area F10 | Area F2 | Area F5 | Area F7A | Area F7B | Area F8A | Area F8B | Area F9 | #Feature | #Feature F1 | #Feature F10 | #Feature F2 | #Feature F5 | #Feature F7A | #Feature F7B | #Feature F8A | #Feature F8B | #Feature F9 | Start | End | PTM | AScore | Found By |
| --- | --- | --- | --- | --- | --- | --- | --- | --- | --- | --- | --- | --- | --- | --- | --- | --- | --- | --- | --- | --- | --- | --- | --- | --- | --- | --- | --- | --- | --- | --- | --- | --- | --- | --- | --- |
| R.EWVLTAAHC(+57.02)DR.R | N | 60.88 | 1356.6245 | 11 | 1.7 | 679.3192 | 2 | 33.03 | 7 | F7:11853 | DaRuWB\_F8A.raw |  |  |  |  |  | 3.6725E8 | 9.1266E7 |  |  | 3 | 0 | 0 | 0 | 0 | 0 | 1 | 2 | 0 | 0 | 60 | 70 | Carbamidomethylation | C9:Carbamidomethylation:1000.00 | PEAKS DB |
| K.ISTTEDTYPDVPHC(+57.02)TNIFIVK.H | Y | 50.49 | 2449.1836 | 21 | 3.2 | 817.4026 | 3 | 55.74 | 7 | F7:27238 | DaRuWB\_F8A.raw |  |  |  |  |  | 8.1099E8 | 2.2223E8 |  |  | 2 | 0 | 0 | 0 | 0 | 0 | 1 | 1 | 0 | 0 | 152 | 172 | Carbamidomethylation | C14:Carbamidomethylation:1000.00 | PEAKS DB |
| K.HKWC(+57.02)EPLYPWVPADSR.T | Y | 49.91 | 2039.9675 | 16 | 2.4 | 680.9962 | 3 | 49.10 | 6 | F6:27608 | DaRuWB\_F7B.raw |  |  |  |  |  | 3.7207E7 |  |  |  | 1 | 0 | 0 | 0 | 0 | 0 | 1 | 0 | 0 | 0 | 173 | 188 | Carbamidomethylation | C4:Carbamidomethylation:1000.00 | PEAKS DB |
| F.LVALYTSASSTIHC(+57.02)AGALINR.E | Y | 47.04 | 2217.1575 | 21 | 4.2 | 740.0609 | 3 | 57.93 | 6 | F6:34544 | DaRuWB\_F7B.raw |  |  |  |  |  | 8.4169E6 |  |  |  | 1 | 0 | 0 | 0 | 0 | 0 | 1 | 0 | 0 | 0 | 39 | 59 | Carbamidomethylation | C14:Carbamidomethylation:1000.00 | PEAKS DB |
| R.RPVTYSTHIAPVSLPSR.S | Y | 43.75 | 1880.0267 | 17 | 2.8 | 627.6829 | 3 | 33.40 | 6 | F6:15464 | DaRuWB\_F7B.raw |  |  |  |  |  | 1.2133E8 |  |  |  | 1 | 0 | 0 | 0 | 0 | 0 | 1 | 0 | 0 | 0 | 120 | 136 |  |  | PEAKS DB |
| K.YFC(+57.02)LNTK.F | Y | 39.92 | 944.4426 | 7 | 2.3 | 473.2283 | 2 | 31.97 | 6 | F6:13969 | DaRuWB\_F7B.raw |  |  |  |  |  | 4.632E7 |  |  |  | 1 | 0 | 0 | 0 | 0 | 0 | 1 | 0 | 0 | 0 | 98 | 104 | Carbamidomethylation | C3:Carbamidomethylation:1000.00 | PEAKS DB |
| K.FPNGLDK.D | Y | 39.56 | 789.4021 | 7 | 2.5 | 395.7084 | 2 | 24.84 | 7 | F7:6904 | DaRuWB\_F8A.raw |  |  |  |  |  | 5.6992E7 | 1.9252E7 |  |  | 2 | 0 | 0 | 0 | 0 | 0 | 1 | 1 | 0 | 0 | 105 | 111 |  |  | PEAKS DB |
| R.TLC(+57.02)AGILK.G | Y | 38.86 | 874.4946 | 8 | 2.3 | 438.2546 | 2 | 34.11 | 7 | F7:12716 | DaRuWB\_F8A.raw |  |  |  |  |  |  | 1.2172E8 |  |  | 1 | 0 | 0 | 0 | 0 | 0 | 0 | 1 | 0 | 0 | 189 | 196 | Carbamidomethylation | C3:Carbamidomethylation:1000.00 | PEAKS DB |
| total 8 peptides |
| --- |

Q5XQN5|K2C5\_BOVIN

back to list

  

| Protein Coverage
| Supporting Peptides
|

Protein Coverage:

Supporting Peptides:

| Peptide | Uniq | -10lgP | Mass | Length | ppm | m/z | z | RT | Fraction | Scan | Source File | Area F1 | Area F10 | Area F2 | Area F5 | Area F7A | Area F7B | Area F8A | Area F8B | Area F9 | #Feature | #Feature F1 | #Feature F10 | #Feature F2 | #Feature F5 | #Feature F7A | #Feature F7B | #Feature F8A | #Feature F8B | #Feature F9 | Start | End | PTM | AScore | Found By |
| --- | --- | --- | --- | --- | --- | --- | --- | --- | --- | --- | --- | --- | --- | --- | --- | --- | --- | --- | --- | --- | --- | --- | --- | --- | --- | --- | --- | --- | --- | --- | --- | --- | --- | --- | --- |
| R.SLDLDSIIAEVK.A | N | 61.51 | 1301.7078 | 12 | 0.8 | 651.8617 | 2 | 75.59 | 2 | F2:17300 | DaRuWB\_F10.raw |  | 7.8623E6 |  |  |  | 7.5254E4 |  |  | 3.1084E6 | 3 | 0 | 1 | 0 | 0 | 0 | 1 | 0 | 0 | 1 | 333 | 344 |  |  | PEAKS DB |
| K.LALDVEIATYR.K | N | 54.90 | 1262.6870 | 11 | 6.0 | 632.3511 | 2 | 58.17 | 5 | F5:33519 | DaRuWB\_F7A.raw |  |  |  |  | 2.0956E6 |  |  |  | 1.0959E5 | 2 | 0 | 0 | 0 | 0 | 1 | 0 | 0 | 0 | 1 | 462 | 472 |  |  | PEAKS DB |
| K.YEELQQTAGR.H | Y | 44.53 | 1193.5676 | 10 | 2.8 | 597.7914 | 2 | 18.92 | 7 | F7:4114 | DaRuWB\_F8A.raw |  |  |  |  | 4.1311E5 |  | 4.7333E5 |  |  | 2 | 0 | 0 | 0 | 0 | 1 | 0 | 1 | 0 | 0 | 366 | 375 |  |  | PEAKS DB |
| total 3 peptides |
| --- |

Q6P6Q2|K2C5\_RAT

back to list

  

| Protein Coverage
| Supporting Peptides
|

Protein Coverage:

Supporting Peptides:

| Peptide | Uniq | -10lgP | Mass | Length | ppm | m/z | z | RT | Fraction | Scan | Source File | Area F1 | Area F10 | Area F2 | Area F5 | Area F7A | Area F7B | Area F8A | Area F8B | Area F9 | #Feature | #Feature F1 | #Feature F10 | #Feature F2 | #Feature F5 | #Feature F7A | #Feature F7B | #Feature F8A | #Feature F8B | #Feature F9 | Start | End | PTM | AScore | Found By |
| --- | --- | --- | --- | --- | --- | --- | --- | --- | --- | --- | --- | --- | --- | --- | --- | --- | --- | --- | --- | --- | --- | --- | --- | --- | --- | --- | --- | --- | --- | --- | --- | --- | --- | --- | --- |
| R.SLDLDSIIAEVK.A | N | 61.51 | 1301.7078 | 12 | 0.8 | 651.8617 | 2 | 75.59 | 2 | F2:17300 | DaRuWB\_F10.raw |  | 7.8623E6 |  |  |  | 7.5254E4 |  |  | 3.1084E6 | 3 | 0 | 1 | 0 | 0 | 0 | 1 | 0 | 0 | 1 | 328 | 339 |  |  | PEAKS DB |
| K.LALDVEIATYR.K | N | 54.90 | 1262.6870 | 11 | 6.0 | 632.3511 | 2 | 58.17 | 5 | F5:33519 | DaRuWB\_F7A.raw |  |  |  |  | 2.0956E6 |  |  |  | 1.0959E5 | 2 | 0 | 0 | 0 | 0 | 1 | 0 | 0 | 0 | 1 | 457 | 467 |  |  | PEAKS DB |
| K.YEELQQTAGR.H | Y | 44.53 | 1193.5676 | 10 | 2.8 | 597.7914 | 2 | 18.92 | 7 | F7:4114 | DaRuWB\_F8A.raw |  |  |  |  | 4.1311E5 |  | 4.7333E5 |  |  | 2 | 0 | 0 | 0 | 0 | 1 | 0 | 1 | 0 | 0 | 361 | 370 |  |  | PEAKS DB |
| total 3 peptides |
| --- |

Q922U2|K2C5\_MOUSE

back to list

  

| Protein Coverage
| Supporting Peptides
|

Protein Coverage:

Supporting Peptides:

| Peptide | Uniq | -10lgP | Mass | Length | ppm | m/z | z | RT | Fraction | Scan | Source File | Area F1 | Area F10 | Area F2 | Area F5 | Area F7A | Area F7B | Area F8A | Area F8B | Area F9 | #Feature | #Feature F1 | #Feature F10 | #Feature F2 | #Feature F5 | #Feature F7A | #Feature F7B | #Feature F8A | #Feature F8B | #Feature F9 | Start | End | PTM | AScore | Found By |
| --- | --- | --- | --- | --- | --- | --- | --- | --- | --- | --- | --- | --- | --- | --- | --- | --- | --- | --- | --- | --- | --- | --- | --- | --- | --- | --- | --- | --- | --- | --- | --- | --- | --- | --- | --- |
| R.SLDLDSIIAEVK.A | N | 61.51 | 1301.7078 | 12 | 0.8 | 651.8617 | 2 | 75.59 | 2 | F2:17300 | DaRuWB\_F10.raw |  | 7.8623E6 |  |  |  | 7.5254E4 |  |  | 3.1084E6 | 3 | 0 | 1 | 0 | 0 | 0 | 1 | 0 | 0 | 1 | 326 | 337 |  |  | PEAKS DB |
| K.LALDVEIATYR.K | N | 54.90 | 1262.6870 | 11 | 6.0 | 632.3511 | 2 | 58.17 | 5 | F5:33519 | DaRuWB\_F7A.raw |  |  |  |  | 2.0956E6 |  |  |  | 1.0959E5 | 2 | 0 | 0 | 0 | 0 | 1 | 0 | 0 | 0 | 1 | 455 | 465 |  |  | PEAKS DB |
| K.YEELQQTAGR.H | Y | 44.53 | 1193.5676 | 10 | 2.8 | 597.7914 | 2 | 18.92 | 7 | F7:4114 | DaRuWB\_F8A.raw |  |  |  |  | 4.1311E5 |  | 4.7333E5 |  |  | 2 | 0 | 0 | 0 | 0 | 1 | 0 | 1 | 0 | 0 | 359 | 368 |  |  | PEAKS DB |
| total 3 peptides |
| --- |

P35908|K22E\_HUMAN

back to list

  

| Protein Coverage
| Supporting Peptides
|

Protein Coverage:

Supporting Peptides:

| Peptide | Uniq | -10lgP | Mass | Length | ppm | m/z | z | RT | Fraction | Scan | Source File | Area F1 | Area F10 | Area F2 | Area F5 | Area F7A | Area F7B | Area F8A | Area F8B | Area F9 | #Feature | #Feature F1 | #Feature F10 | #Feature F2 | #Feature F5 | #Feature F7A | #Feature F7B | #Feature F8A | #Feature F8B | #Feature F9 | Start | End | PTM | AScore | Found By |
| --- | --- | --- | --- | --- | --- | --- | --- | --- | --- | --- | --- | --- | --- | --- | --- | --- | --- | --- | --- | --- | --- | --- | --- | --- | --- | --- | --- | --- | --- | --- | --- | --- | --- | --- | --- |
| K.LALDVEIATYR.K | N | 54.90 | 1262.6870 | 11 | 6.0 | 632.3511 | 2 | 58.17 | 5 | F5:33519 | DaRuWB\_F7A.raw |  |  |  |  | 2.0956E6 |  |  |  | 1.0959E5 | 2 | 0 | 0 | 0 | 0 | 1 | 0 | 0 | 0 | 1 | 471 | 481 |  |  | PEAKS DB |
| R.TAAENDFVTLK.K | Y | 52.95 | 1207.6084 | 11 | 0.3 | 604.8116 | 2 | 38.71 | 2 | F2:8153 | DaRuWB\_F10.raw |  | 2.566E6 |  |  |  |  |  |  | 6.4318E5 | 2 | 0 | 1 | 0 | 0 | 0 | 0 | 0 | 0 | 1 | 276 | 286 |  |  | PEAKS DB |
| R.SKEEAEALYHSK.Y | Y | 43.41 | 1390.6729 | 12 | 0.5 | 464.5651 | 3 | 12.70 | 2 | F2:1945 | DaRuWB\_F10.raw |  | 2.6385E5 |  |  |  |  |  |  |  | 1 | 0 | 1 | 0 | 0 | 0 | 0 | 0 | 0 | 0 | 363 | 374 |  |  | PEAKS DB |
| R.FLEQQNQVLQTK.W | N | 41.80 | 1474.7780 | 12 | 9.0 | 738.3973 | 2 | 35.43 | 8 | F8:14439 | DaRuWB\_F8B.raw |  |  |  |  |  |  |  | 4.5765E6 |  | 1 | 0 | 0 | 0 | 0 | 0 | 0 | 0 | 1 | 0 | 198 | 209 |  |  | PEAKS DB |
| total 4 peptides |
| --- |

Q2MY38|PAT13\_SOLTU

back to list

  

| Protein Coverage
| Supporting Peptides
|

Protein Coverage:

Supporting Peptides:

| Peptide | Uniq | -10lgP | Mass | Length | ppm | m/z | z | RT | Fraction | Scan | Source File | Area F1 | Area F10 | Area F2 | Area F5 | Area F7A | Area F7B | Area F8A | Area F8B | Area F9 | #Feature | #Feature F1 | #Feature F10 | #Feature F2 | #Feature F5 | #Feature F7A | #Feature F7B | #Feature F8A | #Feature F8B | #Feature F9 | Start | End | PTM | AScore | Found By |
| --- | --- | --- | --- | --- | --- | --- | --- | --- | --- | --- | --- | --- | --- | --- | --- | --- | --- | --- | --- | --- | --- | --- | --- | --- | --- | --- | --- | --- | --- | --- | --- | --- | --- | --- | --- |
| R.AEEDPAFASIR.S | Y | 64.28 | 1204.5724 | 11 | 8.2 | 603.2939 | 2 | 37.81 | 1 | F1:15183 | DaRuWB\_F1.raw | 1.8178E6 |  |  |  |  |  |  |  |  | 1 | 1 | 0 | 0 | 0 | 0 | 0 | 0 | 0 | 0 | 237 | 247 |  |  | PEAKS DB |
| K.DSPETYEEALK.R | Y | 59.66 | 1280.5771 | 11 | 7.8 | 641.2961 | 2 | 37.40 | 1 | F1:15090 | DaRuWB\_F1.raw | 2.3136E6 |  |  |  |  |  |  |  |  | 1 | 1 | 0 | 0 | 0 | 0 | 0 | 0 | 0 | 0 | 358 | 368 |  |  | PEAKS DB |
| R.VHQALTEVAISSFDIK.T | N | 43.81 | 1756.9359 | 16 | 8.1 | 586.6530 | 3 | 55.50 | 1 | F1:21166 | DaRuWB\_F1.raw | 7.1562E5 |  |  |  |  |  |  |  |  | 1 | 1 | 0 | 0 | 0 | 0 | 0 | 0 | 0 | 0 | 144 | 159 |  |  | PEAKS DB |
| total 3 peptides |
| --- |

Q7ZT99|CRVP\_CROAT

back to list

  

| Protein Coverage
| Supporting Peptides
|

Protein Coverage:

Supporting Peptides:

| Peptide | Uniq | -10lgP | Mass | Length | ppm | m/z | z | RT | Fraction | Scan | Source File | Area F1 | Area F10 | Area F2 | Area F5 | Area F7A | Area F7B | Area F8A | Area F8B | Area F9 | #Feature | #Feature F1 | #Feature F10 | #Feature F2 | #Feature F5 | #Feature F7A | #Feature F7B | #Feature F8A | #Feature F8B | #Feature F9 | Start | End | PTM | AScore | Found By |
| --- | --- | --- | --- | --- | --- | --- | --- | --- | --- | --- | --- | --- | --- | --- | --- | --- | --- | --- | --- | --- | --- | --- | --- | --- | --- | --- | --- | --- | --- | --- | --- | --- | --- | --- | --- |
| F.YVC(+57.02)QYC(+57.02)PAGNIIGK.T | Y | 59.87 | 1641.7643 | 14 | 5.5 | 821.8894 | 2 | 42.45 | 5 | F5:20872 | DaRuWB\_F7A.raw |  |  |  |  | 4.4196E6 | 6.6103E7 |  |  |  | 2 | 0 | 0 | 0 | 0 | 1 | 1 | 0 | 0 | 0 | 162 | 175 | Carbamidomethylation | C3:Carbamidomethylation:1000.00;C6:Carbamidomethylation:1000.00 | PEAKS DB |
| K.MEWYPEAAANAER.W | N | 57.52 | 1536.6667 | 13 | 3.1 | 769.3409 | 2 | 46.26 | 6 | F6:25146 | DaRuWB\_F7B.raw |  |  |  |  |  | 1.9608E8 |  |  |  | 1 | 0 | 0 | 0 | 0 | 0 | 1 | 0 | 0 | 0 | 58 | 70 |  |  | PEAKS DB |
| K.M(+15.99)EWYPEAAANAER.W | N | 56.52 | 1552.6616 | 13 | 3.5 | 777.3386 | 2 | 39.32 | 6 | F6:20095 | DaRuWB\_F7B.raw |  |  |  |  | 4.7631E6 | 8.8636E7 |  |  |  | 3 | 0 | 0 | 0 | 0 | 1 | 2 | 0 | 0 | 0 | 58 | 70 | Oxidation (M) | M1:Oxidation (M):1000.00 | PEAKS DB |
| C.QYC(+57.02)PAGNIIGK.T | Y | 50.65 | 1219.6019 | 11 | 6.3 | 610.8087 | 2 | 32.54 | 5 | F5:13578 | DaRuWB\_F7A.raw |  |  |  |  | 3.0791E6 | 4.1988E7 | 5.9622E5 |  |  | 3 | 0 | 0 | 0 | 0 | 1 | 1 | 1 | 0 | 0 | 165 | 175 | Carbamidomethylation | C3:Carbamidomethylation:1000.00 | PEAKS DB |
| G.SVDFDSESPR.K | Y | 43.64 | 1137.4938 | 10 | 3.4 | 569.7545 | 2 | 25.11 | 6 | F6:8652 | DaRuWB\_F7B.raw |  |  |  |  | 5.1994E7 | 7.595E8 |  |  |  | 2 | 0 | 0 | 0 | 0 | 1 | 1 | 0 | 0 | 0 | 20 | 29 |  |  | PEAKS DB |
| total 5 peptides |
| --- |

Q7ZTA0|CRVP\_AGKPI

back to list

  

| Protein Coverage
| Supporting Peptides
|

Protein Coverage:

Supporting Peptides:

| Peptide | Uniq | -10lgP | Mass | Length | ppm | m/z | z | RT | Fraction | Scan | Source File | Area F1 | Area F10 | Area F2 | Area F5 | Area F7A | Area F7B | Area F8A | Area F8B | Area F9 | #Feature | #Feature F1 | #Feature F10 | #Feature F2 | #Feature F5 | #Feature F7A | #Feature F7B | #Feature F8A | #Feature F8B | #Feature F9 | Start | End | PTM | AScore | Found By |
| --- | --- | --- | --- | --- | --- | --- | --- | --- | --- | --- | --- | --- | --- | --- | --- | --- | --- | --- | --- | --- | --- | --- | --- | --- | --- | --- | --- | --- | --- | --- | --- | --- | --- | --- | --- |
| F.YVC(+57.02)QYC(+57.02)PAGNIIGK.I | Y | 59.87 | 1641.7643 | 14 | 5.5 | 821.8894 | 2 | 42.45 | 5 | F5:20872 | DaRuWB\_F7A.raw |  |  |  |  | 4.4196E6 | 6.6103E7 |  |  |  | 2 | 0 | 0 | 0 | 0 | 1 | 1 | 0 | 0 | 0 | 162 | 175 | Carbamidomethylation | C3:Carbamidomethylation:1000.00;C6:Carbamidomethylation:1000.00 | PEAKS DB |
| K.MEWYPEAAANAER.W | N | 57.52 | 1536.6667 | 13 | 3.1 | 769.3409 | 2 | 46.26 | 6 | F6:25146 | DaRuWB\_F7B.raw |  |  |  |  |  | 1.9608E8 |  |  |  | 1 | 0 | 0 | 0 | 0 | 0 | 1 | 0 | 0 | 0 | 58 | 70 |  |  | PEAKS DB |
| K.M(+15.99)EWYPEAAANAER.W | N | 56.52 | 1552.6616 | 13 | 3.5 | 777.3386 | 2 | 39.32 | 6 | F6:20095 | DaRuWB\_F7B.raw |  |  |  |  | 4.7631E6 | 8.8636E7 |  |  |  | 3 | 0 | 0 | 0 | 0 | 1 | 2 | 0 | 0 | 0 | 58 | 70 | Oxidation (M) | M1:Oxidation (M):1000.00 | PEAKS DB |
| C.QYC(+57.02)PAGNIIGK.I | Y | 50.65 | 1219.6019 | 11 | 6.3 | 610.8087 | 2 | 32.54 | 5 | F5:13578 | DaRuWB\_F7A.raw |  |  |  |  | 3.0791E6 | 4.1988E7 | 5.9622E5 |  |  | 3 | 0 | 0 | 0 | 0 | 1 | 1 | 1 | 0 | 0 | 165 | 175 | Carbamidomethylation | C3:Carbamidomethylation:1000.00 | PEAKS DB |
| G.SVDFDSESPR.K | Y | 43.64 | 1137.4938 | 10 | 3.4 | 569.7545 | 2 | 25.11 | 6 | F6:8652 | DaRuWB\_F7B.raw |  |  |  |  | 5.1994E7 | 7.595E8 |  |  |  | 2 | 0 | 0 | 0 | 0 | 1 | 1 | 0 | 0 | 0 | 20 | 29 |  |  | PEAKS DB |
| total 5 peptides |
| --- |

A8CG87|PA2A2\_DABRR

back to list

  

| Protein Coverage
| Supporting Peptides
|

Protein Coverage:

Supporting Peptides:

| Peptide | Uniq | -10lgP | Mass | Length | ppm | m/z | z | RT | Fraction | Scan | Source File | Area F1 | Area F10 | Area F2 | Area F5 | Area F7A | Area F7B | Area F8A | Area F8B | Area F9 | #Feature | #Feature F1 | #Feature F10 | #Feature F2 | #Feature F5 | #Feature F7A | #Feature F7B | #Feature F8A | #Feature F8B | #Feature F9 | Start | End | PTM | AScore | Found By |
| --- | --- | --- | --- | --- | --- | --- | --- | --- | --- | --- | --- | --- | --- | --- | --- | --- | --- | --- | --- | --- | --- | --- | --- | --- | --- | --- | --- | --- | --- | --- | --- | --- | --- | --- | --- |
| K.YMLYSIFDC(+57.02)K.E | N | 51.02 | 1338.5988 | 10 | 6.6 | 670.3074 | 2 | 66.79 | 5 | F5:41033 | DaRuWB\_F7A.raw |  |  |  |  | 1.0278E8 | 6.8865E6 |  |  |  | 3 | 0 | 0 | 0 | 0 | 2 | 1 | 0 | 0 | 0 | 123 | 132 | Carbamidomethylation | C9:Carbamidomethylation:1000.00 | PEAKS DB |
| E.GNLYQFGEM(+15.99)INQK.T | Y | 45.00 | 1556.7292 | 13 | 6.5 | 779.3727 | 2 | 48.93 | 5 | F5:25972 | DaRuWB\_F7A.raw |  |  |  |  | 7.3865E7 |  |  |  |  | 1 | 0 | 0 | 0 | 0 | 1 | 0 | 0 | 0 | 0 | 16 | 28 | Oxidation (M) | M9:Oxidation (M):1000.00 | PEAKS DB |
| C.FVHDC(+57.02)C(+57.02)YGR.V | N | 44.06 | 1212.4805 | 9 | 5.0 | 405.1672 | 3 | 12.76 | 5 | F5:1997 | DaRuWB\_F7A.raw |  |  |  |  | 0 |  |  |  |  | 0 | 0 | 0 | 0 | 0 | 0 | 0 | 0 | 0 | 0 | 61 | 69 | Carbamidomethylation | C5:Carbamidomethylation:1000.00;C6:Carbamidomethylation:1000.00 | PEAKS DB |
| total 3 peptides |
| --- |

A8CG78|PA2A2\_DABSI

back to list

  

| Protein Coverage
| Supporting Peptides
|

Protein Coverage:

Supporting Peptides:

| Peptide | Uniq | -10lgP | Mass | Length | ppm | m/z | z | RT | Fraction | Scan | Source File | Area F1 | Area F10 | Area F2 | Area F5 | Area F7A | Area F7B | Area F8A | Area F8B | Area F9 | #Feature | #Feature F1 | #Feature F10 | #Feature F2 | #Feature F5 | #Feature F7A | #Feature F7B | #Feature F8A | #Feature F8B | #Feature F9 | Start | End | PTM | AScore | Found By |
| --- | --- | --- | --- | --- | --- | --- | --- | --- | --- | --- | --- | --- | --- | --- | --- | --- | --- | --- | --- | --- | --- | --- | --- | --- | --- | --- | --- | --- | --- | --- | --- | --- | --- | --- | --- |
| K.YMLYSIFDC(+57.02)K.E | N | 51.02 | 1338.5988 | 10 | 6.6 | 670.3074 | 2 | 66.79 | 5 | F5:41033 | DaRuWB\_F7A.raw |  |  |  |  | 1.0278E8 | 6.8865E6 |  |  |  | 3 | 0 | 0 | 0 | 0 | 2 | 1 | 0 | 0 | 0 | 123 | 132 | Carbamidomethylation | C9:Carbamidomethylation:1000.00 | PEAKS DB |
| E.GNLYQFGEM(+15.99)INQK.T | Y | 45.00 | 1556.7292 | 13 | 6.5 | 779.3727 | 2 | 48.93 | 5 | F5:25972 | DaRuWB\_F7A.raw |  |  |  |  | 7.3865E7 |  |  |  |  | 1 | 0 | 0 | 0 | 0 | 1 | 0 | 0 | 0 | 0 | 16 | 28 | Oxidation (M) | M9:Oxidation (M):1000.00 | PEAKS DB |
| C.FVHDC(+57.02)C(+57.02)YGR.V | N | 44.06 | 1212.4805 | 9 | 5.0 | 405.1672 | 3 | 12.76 | 5 | F5:1997 | DaRuWB\_F7A.raw |  |  |  |  | 0 |  |  |  |  | 0 | 0 | 0 | 0 | 0 | 0 | 0 | 0 | 0 | 0 | 61 | 69 | Carbamidomethylation | C5:Carbamidomethylation:1000.00;C6:Carbamidomethylation:1000.00 | PEAKS DB |
| total 3 peptides |
| --- |

E5L0E3|VSPAF\_DABSI

back to list

  

| Protein Coverage
| Supporting Peptides
|

Protein Coverage:

Supporting Peptides:

| Peptide | Uniq | -10lgP | Mass | Length | ppm | m/z | z | RT | Fraction | Scan | Source File | Area F1 | Area F10 | Area F2 | Area F5 | Area F7A | Area F7B | Area F8A | Area F8B | Area F9 | #Feature | #Feature F1 | #Feature F10 | #Feature F2 | #Feature F5 | #Feature F7A | #Feature F7B | #Feature F8A | #Feature F8B | #Feature F9 | Start | End | PTM | AScore | Found By |
| --- | --- | --- | --- | --- | --- | --- | --- | --- | --- | --- | --- | --- | --- | --- | --- | --- | --- | --- | --- | --- | --- | --- | --- | --- | --- | --- | --- | --- | --- | --- | --- | --- | --- | --- | --- |
| R.TLC(+57.02)AGVSGR.R | Y | 56.56 | 919.4545 | 9 | 1.7 | 460.7343 | 2 | 15.36 | 7 | F7:2805 | DaRuWB\_F8A.raw |  |  |  |  |  |  | 2.7941E7 | 2.6212E6 |  | 2 | 0 | 0 | 0 | 0 | 0 | 0 | 1 | 1 | 0 | 187 | 195 | Carbamidomethylation | C3:Carbamidomethylation:1000.00 | PEAKS DB |
| Y.STHIASLSLPSNPPR.V | Y | 47.95 | 1575.8369 | 15 | 1.3 | 394.9662 | 4 | 36.76 | 7 | F7:14337 | DaRuWB\_F8A.raw |  |  |  |  |  |  | 2.3304E5 |  |  | 1 | 0 | 0 | 0 | 0 | 0 | 0 | 1 | 0 | 0 | 123 | 137 |  |  | PEAKS DB |
| R.IMGWGSITSPK.K | Y | 46.02 | 1175.6008 | 11 | 2.2 | 588.8077 | 2 | 50.62 | 7 | F7:23553 | DaRuWB\_F8A.raw |  |  |  |  |  |  | 2.4201E7 |  |  | 1 | 0 | 0 | 0 | 0 | 0 | 0 | 1 | 0 | 0 | 144 | 154 |  |  | PEAKS DB |
| R.VIYRPLPEQSR.T | Y | 44.84 | 1356.7513 | 11 | 1.9 | 453.2576 | 3 | 23.93 | 7 | F7:6684 | DaRuWB\_F8A.raw |  |  |  |  |  |  | 3.3074E8 |  |  | 1 | 0 | 0 | 0 | 0 | 0 | 0 | 1 | 0 | 0 | 176 | 186 |  |  | PEAKS DB |
| total 4 peptides |
| --- |

E5L0E4|VSPB\_DABSI

back to list

  

| Protein Coverage
| Supporting Peptides
|

Protein Coverage:

Supporting Peptides:

| Peptide | Uniq | -10lgP | Mass | Length | ppm | m/z | z | RT | Fraction | Scan | Source File | Area F1 | Area F10 | Area F2 | Area F5 | Area F7A | Area F7B | Area F8A | Area F8B | Area F9 | #Feature | #Feature F1 | #Feature F10 | #Feature F2 | #Feature F5 | #Feature F7A | #Feature F7B | #Feature F8A | #Feature F8B | #Feature F9 | Start | End | PTM | AScore | Found By |
| --- | --- | --- | --- | --- | --- | --- | --- | --- | --- | --- | --- | --- | --- | --- | --- | --- | --- | --- | --- | --- | --- | --- | --- | --- | --- | --- | --- | --- | --- | --- | --- | --- | --- | --- | --- |
| L.VVGGDEC(+57.02)NINEHR.S | N | 70.48 | 1497.6630 | 13 | 2.3 | 749.8384 | 2 | 12.72 | 6 | F6:1959 | DaRuWB\_F7B.raw |  |  |  |  | 1.9131E6 | 4.0371E6 | 1.0934E6 |  |  | 3 | 0 | 0 | 0 | 0 | 1 | 1 | 1 | 0 | 0 | 25 | 37 | Carbamidomethylation | C7:Carbamidomethylation:1000.00 | PEAKS DB |
| R.NNAEIRLPEER.F | Y | 40.07 | 1339.6843 | 11 | 2.5 | 447.5686 | 3 | 26.08 | 6 | F6:9388 | DaRuWB\_F7B.raw |  |  |  |  |  | 1.1862E6 |  |  |  | 1 | 0 | 0 | 0 | 0 | 0 | 1 | 0 | 0 | 0 | 83 | 93 |  |  | PEAKS DB |
| total 2 peptides |
| --- |

J3SDX0|VSP4\_CROAD

back to list

  

| Protein Coverage
| Supporting Peptides
|

Protein Coverage:

Supporting Peptides:

| Peptide | Uniq | -10lgP | Mass | Length | ppm | m/z | z | RT | Fraction | Scan | Source File | Area F1 | Area F10 | Area F2 | Area F5 | Area F7A | Area F7B | Area F8A | Area F8B | Area F9 | #Feature | #Feature F1 | #Feature F10 | #Feature F2 | #Feature F5 | #Feature F7A | #Feature F7B | #Feature F8A | #Feature F8B | #Feature F9 | Start | End | PTM | AScore | Found By |
| --- | --- | --- | --- | --- | --- | --- | --- | --- | --- | --- | --- | --- | --- | --- | --- | --- | --- | --- | --- | --- | --- | --- | --- | --- | --- | --- | --- | --- | --- | --- | --- | --- | --- | --- | --- |
| E.EWVLTAAHC(+57.02)DR.R | N | 60.88 | 1356.6245 | 11 | 1.7 | 679.3192 | 2 | 33.03 | 7 | F7:11853 | DaRuWB\_F8A.raw |  |  |  |  |  | 3.6725E8 | 9.1266E7 |  |  | 3 | 0 | 0 | 0 | 0 | 0 | 1 | 2 | 0 | 0 | 58 | 68 | Carbamidomethylation | C9:Carbamidomethylation:1000.00 | PEAKS DB |
| S.NSEHIAPLSLPSSPPSVGSVC(+57.02)R.I | N | 57.48 | 2290.1375 | 22 | 3.5 | 764.3870 | 3 | 49.42 | 6 | F6:27883 | DaRuWB\_F7B.raw |  |  |  |  | 7.3886E7 | 1.4476E8 | 5.0003E6 |  |  | 3 | 0 | 0 | 0 | 0 | 1 | 1 | 1 | 0 | 0 | 122 | 143 | Carbamidomethylation | C21:Carbamidomethylation:1000.00 | PEAKS DB |
| L.C(+57.02)GGTLLNEEWVLTAAHC(+57.02)DR.R | Y | 50.58 | 2200.9993 | 19 | 5.9 | 734.6740 | 3 | 57.74 | 5 | F5:33285 | DaRuWB\_F7A.raw |  |  |  |  | 7.6428E5 |  |  |  |  | 1 | 0 | 0 | 0 | 0 | 1 | 0 | 0 | 0 | 0 | 50 | 68 | Carbamidomethylation | C1:Carbamidomethylation:1000.00;C17:Carbamidomethylation:1000.00 | PEAKS DB |
| total 3 peptides |
| --- |

P02533|K1C14\_HUMAN

back to list

  

| Protein Coverage
| Supporting Peptides
|

Protein Coverage:

Supporting Peptides:

| Peptide | Uniq | -10lgP | Mass | Length | ppm | m/z | z | RT | Fraction | Scan | Source File | Area F1 | Area F10 | Area F2 | Area F5 | Area F7A | Area F7B | Area F8A | Area F8B | Area F9 | #Feature | #Feature F1 | #Feature F10 | #Feature F2 | #Feature F5 | #Feature F7A | #Feature F7B | #Feature F8A | #Feature F8B | #Feature F9 | Start | End | PTM | AScore | Found By |
| --- | --- | --- | --- | --- | --- | --- | --- | --- | --- | --- | --- | --- | --- | --- | --- | --- | --- | --- | --- | --- | --- | --- | --- | --- | --- | --- | --- | --- | --- | --- | --- | --- | --- | --- | --- |
| K.DAEEWFFTK.T | Y | 46.49 | 1171.5186 | 9 | 8.2 | 586.7670 | 2 | 63.77 | 8 | F8:37153 | DaRuWB\_F8B.raw |  |  |  |  | 1.0386E6 |  |  | 2.1496E5 |  | 2 | 0 | 0 | 0 | 0 | 1 | 0 | 0 | 1 | 0 | 301 | 309 |  |  | PEAKS DB |
| R.APSTYGGGLSVSSSR.F | Y | 39.99 | 1424.6896 | 15 | 3.3 | 713.3524 | 2 | 27.82 | 6 | F6:10695 | DaRuWB\_F7B.raw |  |  |  |  |  | 2.7111E5 |  |  |  | 1 | 0 | 0 | 0 | 0 | 0 | 1 | 0 | 0 | 0 | 42 | 56 |  |  | PEAKS DB |
| R.EVATNSELVQSGK.S | Y | 39.77 | 1360.6833 | 13 | 0.4 | 681.3492 | 2 | 21.39 | 2 | F2:3931 | DaRuWB\_F10.raw |  | 4.1201E5 |  |  |  |  |  |  |  | 1 | 0 | 1 | 0 | 0 | 0 | 0 | 0 | 0 | 0 | 316 | 328 |  |  | PEAKS DB |
| total 3 peptides |
| --- |

P13645|K1C10\_HUMAN

back to list

  

| Protein Coverage
| Supporting Peptides
|

Protein Coverage:

Supporting Peptides:

| Peptide | Uniq | -10lgP | Mass | Length | ppm | m/z | z | RT | Fraction | Scan | Source File | Area F1 | Area F10 | Area F2 | Area F5 | Area F7A | Area F7B | Area F8A | Area F8B | Area F9 | #Feature | #Feature F1 | #Feature F10 | #Feature F2 | #Feature F5 | #Feature F7A | #Feature F7B | #Feature F8A | #Feature F8B | #Feature F9 | Start | End | PTM | AScore | Found By |
| --- | --- | --- | --- | --- | --- | --- | --- | --- | --- | --- | --- | --- | --- | --- | --- | --- | --- | --- | --- | --- | --- | --- | --- | --- | --- | --- | --- | --- | --- | --- | --- | --- | --- | --- | --- |
| R.QSVEADINGLR.R | Y | 51.35 | 1200.6099 | 11 | 5.8 | 601.3124 | 2 | 36.98 | 5 | F5:16757 | DaRuWB\_F7A.raw |  |  |  |  | 1.5752E7 |  |  |  |  | 1 | 0 | 0 | 0 | 0 | 1 | 0 | 0 | 0 | 0 | 246 | 256 |  |  | PEAKS DB |
| K.ADLEM(+15.99)QIESLTEELAYLK.K | Y | 50.78 | 2111.0344 | 18 | 0.9 | 704.6860 | 3 | 98.03 | 2 | F2:22511 | DaRuWB\_F10.raw |  | 2.8338E5 |  |  |  |  |  |  |  | 1 | 0 | 1 | 0 | 0 | 0 | 0 | 0 | 0 | 0 | 267 | 284 | Oxidation (M) | M5:Oxidation (M):1000.00 | PEAKS DB |
| R.NVQALEIELQSQLALK.Q | Y | 44.72 | 1796.0043 | 16 | 5.6 | 599.6754 | 3 | 73.00 | 5 | F5:46148 | DaRuWB\_F7A.raw |  | 7.4565E5 |  |  | 2.0661E6 |  |  |  |  | 2 | 0 | 1 | 0 | 0 | 1 | 0 | 0 | 0 | 0 | 371 | 386 |  |  | PEAKS DB |
| total 3 peptides |
| --- |

C0HLB2|VKT\_PSEPC

back to list

  

| Protein Coverage
| Supporting Peptides
|

Protein Coverage:

Supporting Peptides:

| Peptide | Uniq | -10lgP | Mass | Length | ppm | m/z | z | RT | Fraction | Scan | Source File | Area F1 | Area F10 | Area F2 | Area F5 | Area F7A | Area F7B | Area F8A | Area F8B | Area F9 | #Feature | #Feature F1 | #Feature F10 | #Feature F2 | #Feature F5 | #Feature F7A | #Feature F7B | #Feature F8A | #Feature F8B | #Feature F9 | Start | End | PTM | AScore | Found By |
| --- | --- | --- | --- | --- | --- | --- | --- | --- | --- | --- | --- | --- | --- | --- | --- | --- | --- | --- | --- | --- | --- | --- | --- | --- | --- | --- | --- | --- | --- | --- | --- | --- | --- | --- | --- |
| I.YGGC(+57.02)GGNANNFETR.A | N | 51.77 | 1515.6161 | 14 | 8.1 | 758.8156 | 2 | 20.19 | 3 | F3:5193 | DaRuWB\_F2.raw |  |  | 3.2933E8 |  |  |  |  |  |  | 1 | 0 | 0 | 1 | 0 | 0 | 0 | 0 | 0 | 0 | 37 | 50 | Carbamidomethylation | C4:Carbamidomethylation:1000.00 | PEAKS DB |
| F.IYGGC(+57.02)GGNANNFETR.A | Y | 44.85 | 1628.7001 | 15 | 8.3 | 815.3577 | 2 | 28.33 | 3 | F3:10186 | DaRuWB\_F2.raw |  |  | 0 |  |  |  |  |  |  | 0 | 0 | 0 | 0 | 0 | 0 | 0 | 0 | 0 | 0 | 36 | 50 | Carbamidomethylation | C5:Carbamidomethylation:1000.00 | PEAKS DB |
| G.C(+57.02)GGNANNFETR.A | N | 43.34 | 1238.5098 | 11 | 8.3 | 620.2625 | 2 | 12.95 | 3 | F3:2045 | DaRuWB\_F2.raw |  |  | 8.3524E5 |  |  |  |  |  |  | 1 | 0 | 0 | 1 | 0 | 0 | 0 | 0 | 0 | 0 | 40 | 50 | Carbamidomethylation | C1:Carbamidomethylation:1000.00 | PEAKS DB |
| total 3 peptides |
| --- |

P24541|VKT\_ERIMA

back to list

  

| Protein Coverage
| Supporting Peptides
|

Protein Coverage:

Supporting Peptides:

| Peptide | Uniq | -10lgP | Mass | Length | ppm | m/z | z | RT | Fraction | Scan | Source File | Area F1 | Area F10 | Area F2 | Area F5 | Area F7A | Area F7B | Area F8A | Area F8B | Area F9 | #Feature | #Feature F1 | #Feature F10 | #Feature F2 | #Feature F5 | #Feature F7A | #Feature F7B | #Feature F8A | #Feature F8B | #Feature F9 | Start | End | PTM | AScore | Found By |
| --- | --- | --- | --- | --- | --- | --- | --- | --- | --- | --- | --- | --- | --- | --- | --- | --- | --- | --- | --- | --- | --- | --- | --- | --- | --- | --- | --- | --- | --- | --- | --- | --- | --- | --- | --- |
| I.YGGC(+57.02)GGNANNFETR.A | N | 51.77 | 1515.6161 | 14 | 8.1 | 758.8156 | 2 | 20.19 | 3 | F3:5193 | DaRuWB\_F2.raw |  |  | 3.2933E8 |  |  |  |  |  |  | 1 | 0 | 0 | 1 | 0 | 0 | 0 | 0 | 0 | 0 | 32 | 45 | Carbamidomethylation | C4:Carbamidomethylation:1000.00 | PEAKS DB |
| F.IYGGC(+57.02)GGNANNFETR.A | Y | 44.85 | 1628.7001 | 15 | 8.3 | 815.3577 | 2 | 28.33 | 3 | F3:10186 | DaRuWB\_F2.raw |  |  | 0 |  |  |  |  |  |  | 0 | 0 | 0 | 0 | 0 | 0 | 0 | 0 | 0 | 0 | 31 | 45 | Carbamidomethylation | C5:Carbamidomethylation:1000.00 | PEAKS DB |
| G.C(+57.02)GGNANNFETR.A | N | 43.34 | 1238.5098 | 11 | 8.3 | 620.2625 | 2 | 12.95 | 3 | F3:2045 | DaRuWB\_F2.raw |  |  | 8.3524E5 |  |  |  |  |  |  | 1 | 0 | 0 | 1 | 0 | 0 | 0 | 0 | 0 | 0 | 35 | 45 | Carbamidomethylation | C1:Carbamidomethylation:1000.00 | PEAKS DB |
| total 3 peptides |
| --- |

Q2ES47|VKT4\_DABRR

back to list

  

| Protein Coverage
| Supporting Peptides
|

Protein Coverage:

Supporting Peptides:

| Peptide | Uniq | -10lgP | Mass | Length | ppm | m/z | z | RT | Fraction | Scan | Source File | Area F1 | Area F10 | Area F2 | Area F5 | Area F7A | Area F7B | Area F8A | Area F8B | Area F9 | #Feature | #Feature F1 | #Feature F10 | #Feature F2 | #Feature F5 | #Feature F7A | #Feature F7B | #Feature F8A | #Feature F8B | #Feature F9 | Start | End | PTM | AScore | Found By |
| --- | --- | --- | --- | --- | --- | --- | --- | --- | --- | --- | --- | --- | --- | --- | --- | --- | --- | --- | --- | --- | --- | --- | --- | --- | --- | --- | --- | --- | --- | --- | --- | --- | --- | --- | --- |
| I.YGGC(+57.02)GGNANNFETR.D | N | 51.77 | 1515.6161 | 14 | 8.1 | 758.8156 | 2 | 20.19 | 3 | F3:5193 | DaRuWB\_F2.raw |  |  | 3.2933E8 |  |  |  |  |  |  | 1 | 0 | 0 | 1 | 0 | 0 | 0 | 0 | 0 | 0 | 61 | 74 | Carbamidomethylation | C4:Carbamidomethylation:1000.00 | PEAKS DB |
| F.IYGGC(+57.02)GGNANNFETR.D | Y | 44.85 | 1628.7001 | 15 | 8.3 | 815.3577 | 2 | 28.33 | 3 | F3:10186 | DaRuWB\_F2.raw |  |  | 0 |  |  |  |  |  |  | 0 | 0 | 0 | 0 | 0 | 0 | 0 | 0 | 0 | 0 | 60 | 74 | Carbamidomethylation | C5:Carbamidomethylation:1000.00 | PEAKS DB |
| G.C(+57.02)GGNANNFETR.D | N | 43.34 | 1238.5098 | 11 | 8.3 | 620.2625 | 2 | 12.95 | 3 | F3:2045 | DaRuWB\_F2.raw |  |  | 8.3524E5 |  |  |  |  |  |  | 1 | 0 | 0 | 1 | 0 | 0 | 0 | 0 | 0 | 0 | 64 | 74 | Carbamidomethylation | C1:Carbamidomethylation:1000.00 | PEAKS DB |
| total 3 peptides |
| --- |

P30894|NGFV\_DABRR

back to list

  

| Protein Coverage
| Supporting Peptides
|

Protein Coverage:

Supporting Peptides:

| Peptide | Uniq | -10lgP | Mass | Length | ppm | m/z | z | RT | Fraction | Scan | Source File | Area F1 | Area F10 | Area F2 | Area F5 | Area F7A | Area F7B | Area F8A | Area F8B | Area F9 | #Feature | #Feature F1 | #Feature F10 | #Feature F2 | #Feature F5 | #Feature F7A | #Feature F7B | #Feature F8A | #Feature F8B | #Feature F9 | Start | End | PTM | AScore | Found By |
| --- | --- | --- | --- | --- | --- | --- | --- | --- | --- | --- | --- | --- | --- | --- | --- | --- | --- | --- | --- | --- | --- | --- | --- | --- | --- | --- | --- | --- | --- | --- | --- | --- | --- | --- | --- |
| R.INTAC(+57.02)VC(+57.02)VISR.K | Y | 59.57 | 1291.6377 | 11 | 0.8 | 646.8267 | 2 | 34.70 | 4 | F4:8328 | DaRuWB\_F5.raw |  |  |  | 1.0152E8 |  |  |  |  |  | 1 | 0 | 0 | 0 | 1 | 0 | 0 | 0 | 0 | 0 | 101 | 111 | Carbamidomethylation | C5:Carbamidomethylation:1000.00;C7:Carbamidomethylation:1000.00 | PEAKS DB |
| N.SYC(+57.02)TTTDTFVR.A | Y | 51.47 | 1349.5922 | 11 | 0.6 | 675.8038 | 2 | 32.44 | 4 | F4:7665 | DaRuWB\_F5.raw |  |  |  | 1.8722E7 |  |  |  |  |  | 1 | 0 | 0 | 0 | 1 | 0 | 0 | 0 | 0 | 0 | 75 | 85 | Carbamidomethylation | C3:Carbamidomethylation:1000.00 | PEAKS DB |
| total 2 peptides |
| --- |

G8XQX1|OXLA\_DABRR

back to list

  

| Protein Coverage
| Supporting Peptides
|

Protein Coverage:

Supporting Peptides:

| Peptide | Uniq | -10lgP | Mass | Length | ppm | m/z | z | RT | Fraction | Scan | Source File | Area F1 | Area F10 | Area F2 | Area F5 | Area F7A | Area F7B | Area F8A | Area F8B | Area F9 | #Feature | #Feature F1 | #Feature F10 | #Feature F2 | #Feature F5 | #Feature F7A | #Feature F7B | #Feature F8A | #Feature F8B | #Feature F9 | Start | End | PTM | AScore | Found By |
| --- | --- | --- | --- | --- | --- | --- | --- | --- | --- | --- | --- | --- | --- | --- | --- | --- | --- | --- | --- | --- | --- | --- | --- | --- | --- | --- | --- | --- | --- | --- | --- | --- | --- | --- | --- |
| K.SAGQLYQESLGK.A | Y | 58.44 | 1279.6407 | 12 | 0.6 | 640.8281 | 2 | 31.19 | 9 | F9:5600 | DaRuWB\_F9.raw |  |  |  |  |  |  |  |  | 3.1694E6 | 1 | 0 | 0 | 0 | 0 | 0 | 0 | 0 | 0 | 1 | 170 | 181 |  |  | PEAKS DB |
| K.KDLQTFC(+57.02)YPSIIQK.W | Y | 39.31 | 1739.8916 | 14 | 3.0 | 580.9716 | 3 | 47.37 | 7 | F7:21413 | DaRuWB\_F8A.raw |  |  |  |  |  |  | 2.8108E5 |  |  | 1 | 0 | 0 | 0 | 0 | 0 | 0 | 1 | 0 | 0 | 424 | 437 | Carbamidomethylation | C7:Carbamidomethylation:1000.00 | PEAKS DB |
| total 2 peptides |
| --- |

O22507|LOX17\_SOLTU

back to list

  

| Protein Coverage
| Supporting Peptides
|

Protein Coverage:

Supporting Peptides:

| Peptide | Uniq | -10lgP | Mass | Length | ppm | m/z | z | RT | Fraction | Scan | Source File | Area F1 | Area F10 | Area F2 | Area F5 | Area F7A | Area F7B | Area F8A | Area F8B | Area F9 | #Feature | #Feature F1 | #Feature F10 | #Feature F2 | #Feature F5 | #Feature F7A | #Feature F7B | #Feature F8A | #Feature F8B | #Feature F9 | Start | End | PTM | AScore | Found By |
| --- | --- | --- | --- | --- | --- | --- | --- | --- | --- | --- | --- | --- | --- | --- | --- | --- | --- | --- | --- | --- | --- | --- | --- | --- | --- | --- | --- | --- | --- | --- | --- | --- | --- | --- | --- |
| R.GVAVEDSSSPHGVR.L | Y | 49.14 | 1395.6742 | 14 | 8.1 | 466.2323 | 3 | 13.11 | 1 | F1:2178 | DaRuWB\_F1.raw | 2.4042E5 |  |  |  |  |  |  |  |  | 1 | 1 | 0 | 0 | 0 | 0 | 0 | 0 | 0 | 0 | 612 | 625 |  |  | PEAKS DB |
| F.YYGSDEEILK.D | Y | 38.95 | 1215.5659 | 10 | 7.7 | 608.7904 | 2 | 36.72 | 1 | F1:14770 | DaRuWB\_F1.raw | 1.6457E5 |  |  |  |  |  |  |  |  | 1 | 1 | 0 | 0 | 0 | 0 | 0 | 0 | 0 | 0 | 655 | 664 |  |  | PEAKS DB |
| total 2 peptides |
| --- |

O22508|LOX18\_SOLTU

back to list

  

| Protein Coverage
| Supporting Peptides
|

Protein Coverage:

Supporting Peptides:

| Peptide | Uniq | -10lgP | Mass | Length | ppm | m/z | z | RT | Fraction | Scan | Source File | Area F1 | Area F10 | Area F2 | Area F5 | Area F7A | Area F7B | Area F8A | Area F8B | Area F9 | #Feature | #Feature F1 | #Feature F10 | #Feature F2 | #Feature F5 | #Feature F7A | #Feature F7B | #Feature F8A | #Feature F8B | #Feature F9 | Start | End | PTM | AScore | Found By |
| --- | --- | --- | --- | --- | --- | --- | --- | --- | --- | --- | --- | --- | --- | --- | --- | --- | --- | --- | --- | --- | --- | --- | --- | --- | --- | --- | --- | --- | --- | --- | --- | --- | --- | --- | --- |
| R.GVAVEDSSSPHGVR.L | Y | 49.14 | 1395.6742 | 14 | 8.1 | 466.2323 | 3 | 13.11 | 1 | F1:2178 | DaRuWB\_F1.raw | 2.4042E5 |  |  |  |  |  |  |  |  | 1 | 1 | 0 | 0 | 0 | 0 | 0 | 0 | 0 | 0 | 612 | 625 |  |  | PEAKS DB |
| F.YYGSDEEILK.D | Y | 38.95 | 1215.5659 | 10 | 7.7 | 608.7904 | 2 | 36.72 | 1 | F1:14770 | DaRuWB\_F1.raw | 1.6457E5 |  |  |  |  |  |  |  |  | 1 | 1 | 0 | 0 | 0 | 0 | 0 | 0 | 0 | 0 | 655 | 664 |  |  | PEAKS DB |
| total 2 peptides |
| --- |

Q6WP39|OXLA\_TRIST

back to list

  

| Protein Coverage
| Supporting Peptides
|

Protein Coverage:

Supporting Peptides:

| Peptide | Uniq | -10lgP | Mass | Length | ppm | m/z | z | RT | Fraction | Scan | Source File | Area F1 | Area F10 | Area F2 | Area F5 | Area F7A | Area F7B | Area F8A | Area F8B | Area F9 | #Feature | #Feature F1 | #Feature F10 | #Feature F2 | #Feature F5 | #Feature F7A | #Feature F7B | #Feature F8A | #Feature F8B | #Feature F9 | Start | End | PTM | AScore | Found By |
| --- | --- | --- | --- | --- | --- | --- | --- | --- | --- | --- | --- | --- | --- | --- | --- | --- | --- | --- | --- | --- | --- | --- | --- | --- | --- | --- | --- | --- | --- | --- | --- | --- | --- | --- | --- |
| K.FNLQLNEFSQENDNAWHFVK.N | Y | 77.22 | 2479.1555 | 20 | 8.4 | 827.3932 | 3 | 70.45 | 8 | F8:42267 | DaRuWB\_F8B.raw |  |  |  |  |  |  |  | 5.2576E6 |  | 1 | 0 | 0 | 0 | 0 | 0 | 0 | 0 | 1 | 0 | 123 | 142 |  |  | PEAKS DB |
| total 1 peptides |
| --- |

B7FDI1|CRVP\_VIPBE

back to list

  

| Protein Coverage
| Supporting Peptides
|

Protein Coverage:

Supporting Peptides:

| Peptide | Uniq | -10lgP | Mass | Length | ppm | m/z | z | RT | Fraction | Scan | Source File | Area F1 | Area F10 | Area F2 | Area F5 | Area F7A | Area F7B | Area F8A | Area F8B | Area F9 | #Feature | #Feature F1 | #Feature F10 | #Feature F2 | #Feature F5 | #Feature F7A | #Feature F7B | #Feature F8A | #Feature F8B | #Feature F9 | Start | End | PTM | AScore | Found By |
| --- | --- | --- | --- | --- | --- | --- | --- | --- | --- | --- | --- | --- | --- | --- | --- | --- | --- | --- | --- | --- | --- | --- | --- | --- | --- | --- | --- | --- | --- | --- | --- | --- | --- | --- | --- |
| K.MEWYPEAAANAER.W | N | 57.52 | 1536.6667 | 13 | 3.1 | 769.3409 | 2 | 46.26 | 6 | F6:25146 | DaRuWB\_F7B.raw |  |  |  |  |  | 1.9608E8 |  |  |  | 1 | 0 | 0 | 0 | 0 | 0 | 1 | 0 | 0 | 0 | 58 | 70 |  |  | PEAKS DB |
| K.M(+15.99)EWYPEAAANAER.W | N | 56.52 | 1552.6616 | 13 | 3.5 | 777.3386 | 2 | 39.32 | 6 | F6:20095 | DaRuWB\_F7B.raw |  |  |  |  | 4.7631E6 | 8.8636E7 |  |  |  | 3 | 0 | 0 | 0 | 0 | 1 | 2 | 0 | 0 | 0 | 58 | 70 | Oxidation (M) | M1:Oxidation (M):1000.00 | PEAKS DB |
| E.KDFVYGQGASPANAVVGHYTQIVWYK.S | Y | 44.34 | 2897.4500 | 26 | 6.3 | 725.3704 | 4 | 59.16 | 5 | F5:34377 | DaRuWB\_F7A.raw |  |  |  |  | 4.3914E6 |  |  |  |  | 1 | 0 | 0 | 0 | 0 | 1 | 0 | 0 | 0 | 0 | 117 | 142 |  |  | PEAKS DB |
| total 3 peptides |
| --- |

B7FDI0|CRVP\_VIPBN

back to list

  

| Protein Coverage
| Supporting Peptides
|

Protein Coverage:

Supporting Peptides:

| Peptide | Uniq | -10lgP | Mass | Length | ppm | m/z | z | RT | Fraction | Scan | Source File | Area F1 | Area F10 | Area F2 | Area F5 | Area F7A | Area F7B | Area F8A | Area F8B | Area F9 | #Feature | #Feature F1 | #Feature F10 | #Feature F2 | #Feature F5 | #Feature F7A | #Feature F7B | #Feature F8A | #Feature F8B | #Feature F9 | Start | End | PTM | AScore | Found By |
| --- | --- | --- | --- | --- | --- | --- | --- | --- | --- | --- | --- | --- | --- | --- | --- | --- | --- | --- | --- | --- | --- | --- | --- | --- | --- | --- | --- | --- | --- | --- | --- | --- | --- | --- | --- |
| K.MEWYPEAAANAER.W | N | 57.52 | 1536.6667 | 13 | 3.1 | 769.3409 | 2 | 46.26 | 6 | F6:25146 | DaRuWB\_F7B.raw |  |  |  |  |  | 1.9608E8 |  |  |  | 1 | 0 | 0 | 0 | 0 | 0 | 1 | 0 | 0 | 0 | 40 | 52 |  |  | PEAKS DB |
| K.M(+15.99)EWYPEAAANAER.W | N | 56.52 | 1552.6616 | 13 | 3.5 | 777.3386 | 2 | 39.32 | 6 | F6:20095 | DaRuWB\_F7B.raw |  |  |  |  | 4.7631E6 | 8.8636E7 |  |  |  | 3 | 0 | 0 | 0 | 0 | 1 | 2 | 0 | 0 | 0 | 40 | 52 | Oxidation (M) | M1:Oxidation (M):1000.00 | PEAKS DB |
| E.KDFVYGQGASPANAVVGHYTQIVWYK.S | Y | 44.34 | 2897.4500 | 26 | 6.3 | 725.3704 | 4 | 59.16 | 5 | F5:34377 | DaRuWB\_F7A.raw |  |  |  |  | 4.3914E6 |  |  |  |  | 1 | 0 | 0 | 0 | 0 | 1 | 0 | 0 | 0 | 0 | 99 | 124 |  |  | PEAKS DB |
| total 3 peptides |
| --- |

Q02471|PA2B4\_DABSI

back to list

  

| Protein Coverage
| Supporting Peptides
|

Protein Coverage:

Supporting Peptides:

| Peptide | Uniq | -10lgP | Mass | Length | ppm | m/z | z | RT | Fraction | Scan | Source File | Area F1 | Area F10 | Area F2 | Area F5 | Area F7A | Area F7B | Area F8A | Area F8B | Area F9 | #Feature | #Feature F1 | #Feature F10 | #Feature F2 | #Feature F5 | #Feature F7A | #Feature F7B | #Feature F8A | #Feature F8B | #Feature F9 | Start | End | PTM | AScore | Found By |
| --- | --- | --- | --- | --- | --- | --- | --- | --- | --- | --- | --- | --- | --- | --- | --- | --- | --- | --- | --- | --- | --- | --- | --- | --- | --- | --- | --- | --- | --- | --- | --- | --- | --- | --- | --- |
| Y.GC(+57.02)YC(+57.02)GWGGQGTPK.D | N | 61.85 | 1426.5758 | 13 | 2.6 | 714.2950 | 2 | 27.48 | 6 | F6:10372 | DaRuWB\_F7B.raw |  |  |  |  |  | 1.3458E8 |  |  |  | 1 | 0 | 0 | 0 | 0 | 0 | 1 | 0 | 0 | 0 | 41 | 53 | Carbamidomethylation | C2:Carbamidomethylation:1000.00;C4:Carbamidomethylation:1000.00 | PEAKS DB |
| R.C(+57.02)C(+57.02)FVHDC(+57.02)C(+57.02)YGGVK.G | Y | 60.16 | 1660.6255 | 13 | 3.6 | 831.3207 | 2 | 24.74 | 6 | F6:8552 | DaRuWB\_F7B.raw |  |  |  |  |  | 1.0574E7 |  |  |  | 1 | 0 | 0 | 0 | 0 | 0 | 1 | 0 | 0 | 0 | 59 | 71 | Carbamidomethylation | C1:Carbamidomethylation:1000.00;C2:Carbamidomethylation:1000.00;C7:Carbamidomethylation:1000.00;C8:Carbamidomethylation:1000.00 | PEAKS DB |
| total 2 peptides |
| --- |

P20347|CPI1\_SOLTU

back to list

  

| Protein Coverage
| Supporting Peptides
|

Protein Coverage:

Supporting Peptides:

| Peptide | Uniq | -10lgP | Mass | Length | ppm | m/z | z | RT | Fraction | Scan | Source File | Area F1 | Area F10 | Area F2 | Area F5 | Area F7A | Area F7B | Area F8A | Area F8B | Area F9 | #Feature | #Feature F1 | #Feature F10 | #Feature F2 | #Feature F5 | #Feature F7A | #Feature F7B | #Feature F8A | #Feature F8B | #Feature F9 | Start | End | PTM | AScore | Found By |
| --- | --- | --- | --- | --- | --- | --- | --- | --- | --- | --- | --- | --- | --- | --- | --- | --- | --- | --- | --- | --- | --- | --- | --- | --- | --- | --- | --- | --- | --- | --- | --- | --- | --- | --- | --- |
| K.VNDEQLVVTGGK.V | Y | 67.80 | 1257.6565 | 12 | 7.5 | 629.8356 | 2 | 28.73 | 1 | F1:11226 | DaRuWB\_F1.raw | 4.8192E6 |  |  |  |  |  |  |  |  | 2 | 2 | 0 | 0 | 0 | 0 | 0 | 0 | 0 | 0 | 144 | 155 |  |  | PEAKS DB |
| K.VGNENDIFK.I | N | 44.23 | 1034.5033 | 9 | 6.7 | 518.2585 | 2 | 30.00 | 1 | F1:11774 | DaRuWB\_F1.raw | 1.8699E6 |  |  |  |  |  |  |  |  | 1 | 1 | 0 | 0 | 0 | 0 | 0 | 0 | 0 | 0 | 156 | 164 |  |  | PEAKS DB |
| total 2 peptides |
| --- |

O24387|CPI5\_SOLTU

back to list

  

| Protein Coverage
| Supporting Peptides
|

Protein Coverage:

Supporting Peptides:

| Peptide | Uniq | -10lgP | Mass | Length | ppm | m/z | z | RT | Fraction | Scan | Source File | Area F1 | Area F10 | Area F2 | Area F5 | Area F7A | Area F7B | Area F8A | Area F8B | Area F9 | #Feature | #Feature F1 | #Feature F10 | #Feature F2 | #Feature F5 | #Feature F7A | #Feature F7B | #Feature F8A | #Feature F8B | #Feature F9 | Start | End | PTM | AScore | Found By |
| --- | --- | --- | --- | --- | --- | --- | --- | --- | --- | --- | --- | --- | --- | --- | --- | --- | --- | --- | --- | --- | --- | --- | --- | --- | --- | --- | --- | --- | --- | --- | --- | --- | --- | --- | --- |
| K.VNDEQLVVTGGK.V | Y | 67.80 | 1257.6565 | 12 | 7.5 | 629.8356 | 2 | 28.73 | 1 | F1:11226 | DaRuWB\_F1.raw | 4.8192E6 |  |  |  |  |  |  |  |  | 2 | 2 | 0 | 0 | 0 | 0 | 0 | 0 | 0 | 0 | 73 | 84 |  |  | PEAKS DB |
| K.VGNENDIFK.I | N | 44.23 | 1034.5033 | 9 | 6.7 | 518.2585 | 2 | 30.00 | 1 | F1:11774 | DaRuWB\_F1.raw | 1.8699E6 |  |  |  |  |  |  |  |  | 1 | 1 | 0 | 0 | 0 | 0 | 0 | 0 | 0 | 0 | 85 | 93 |  |  | PEAKS DB |
| total 2 peptides |
| --- |

O24388|CPI3\_SOLTU

back to list

  

| Protein Coverage
| Supporting Peptides
|

Protein Coverage:

Supporting Peptides:

| Peptide | Uniq | -10lgP | Mass | Length | ppm | m/z | z | RT | Fraction | Scan | Source File | Area F1 | Area F10 | Area F2 | Area F5 | Area F7A | Area F7B | Area F8A | Area F8B | Area F9 | #Feature | #Feature F1 | #Feature F10 | #Feature F2 | #Feature F5 | #Feature F7A | #Feature F7B | #Feature F8A | #Feature F8B | #Feature F9 | Start | End | PTM | AScore | Found By |
| --- | --- | --- | --- | --- | --- | --- | --- | --- | --- | --- | --- | --- | --- | --- | --- | --- | --- | --- | --- | --- | --- | --- | --- | --- | --- | --- | --- | --- | --- | --- | --- | --- | --- | --- | --- |
| K.VNDEQLVVTGGK.V | Y | 67.80 | 1257.6565 | 12 | 7.5 | 629.8356 | 2 | 28.73 | 1 | F1:11226 | DaRuWB\_F1.raw | 4.8192E6 |  |  |  |  |  |  |  |  | 2 | 2 | 0 | 0 | 0 | 0 | 0 | 0 | 0 | 0 | 68 | 79 |  |  | PEAKS DB |
| K.VGNENDIFK.I | N | 44.23 | 1034.5033 | 9 | 6.7 | 518.2585 | 2 | 30.00 | 1 | F1:11774 | DaRuWB\_F1.raw | 1.8699E6 |  |  |  |  |  |  |  |  | 1 | 1 | 0 | 0 | 0 | 0 | 0 | 0 | 0 | 0 | 80 | 88 |  |  | PEAKS DB |
| total 2 peptides |
| --- |

Q8JFG1|PA2H\_VIPAP

back to list

  

| Protein Coverage
| Supporting Peptides
|

Protein Coverage:

Supporting Peptides:

| Peptide | Uniq | -10lgP | Mass | Length | ppm | m/z | z | RT | Fraction | Scan | Source File | Area F1 | Area F10 | Area F2 | Area F5 | Area F7A | Area F7B | Area F8A | Area F8B | Area F9 | #Feature | #Feature F1 | #Feature F10 | #Feature F2 | #Feature F5 | #Feature F7A | #Feature F7B | #Feature F8A | #Feature F8B | #Feature F9 | Start | End | PTM | AScore | Found By |
| --- | --- | --- | --- | --- | --- | --- | --- | --- | --- | --- | --- | --- | --- | --- | --- | --- | --- | --- | --- | --- | --- | --- | --- | --- | --- | --- | --- | --- | --- | --- | --- | --- | --- | --- | --- |
| R.AAAIC(+57.02)LGENVNTYDK.N | Y | 68.12 | 1637.7719 | 15 | 8.7 | 819.8942 | 2 | 40.75 | 8 | F8:18687 | DaRuWB\_F8B.raw |  |  |  |  |  |  |  | 2.5614E7 |  | 5 | 0 | 0 | 0 | 0 | 0 | 0 | 0 | 5 | 0 | 107 | 121 | Carbamidomethylation | C5:Carbamidomethylation:1000.00 | PEAKS DB |
| K.EAVHSYAIYGC(+57.02)Y.C | N | 40.08 | 1431.6129 | 12 | 2.4 | 716.8139 | 2 | 45.05 | 7 | F7:19798 | DaRuWB\_F8A.raw |  |  |  |  |  |  | 4.5632E6 |  |  | 1 | 0 | 0 | 0 | 0 | 0 | 0 | 1 | 0 | 0 | 32 | 43 | Carbamidomethylation | C11:Carbamidomethylation:1000.00 | PEAKS DB |
| total 2 peptides |
| --- |

Q10754|PA2H\_VIPAZ

back to list

  

| Protein Coverage
| Supporting Peptides
|

Protein Coverage:

Supporting Peptides:

| Peptide | Uniq | -10lgP | Mass | Length | ppm | m/z | z | RT | Fraction | Scan | Source File | Area F1 | Area F10 | Area F2 | Area F5 | Area F7A | Area F7B | Area F8A | Area F8B | Area F9 | #Feature | #Feature F1 | #Feature F10 | #Feature F2 | #Feature F5 | #Feature F7A | #Feature F7B | #Feature F8A | #Feature F8B | #Feature F9 | Start | End | PTM | AScore | Found By |
| --- | --- | --- | --- | --- | --- | --- | --- | --- | --- | --- | --- | --- | --- | --- | --- | --- | --- | --- | --- | --- | --- | --- | --- | --- | --- | --- | --- | --- | --- | --- | --- | --- | --- | --- | --- |
| R.AAAIC(+57.02)LGENVNTYDK.N | Y | 68.12 | 1637.7719 | 15 | 8.7 | 819.8942 | 2 | 40.75 | 8 | F8:18687 | DaRuWB\_F8B.raw |  |  |  |  |  |  |  | 2.5614E7 |  | 5 | 0 | 0 | 0 | 0 | 0 | 0 | 0 | 5 | 0 | 107 | 121 | Carbamidomethylation | C5:Carbamidomethylation:1000.00 | PEAKS DB |
| K.EAVHSYAIYGC(+57.02)Y.C | N | 40.08 | 1431.6129 | 12 | 2.4 | 716.8139 | 2 | 45.05 | 7 | F7:19798 | DaRuWB\_F8A.raw |  |  |  |  |  |  | 4.5632E6 |  |  | 1 | 0 | 0 | 0 | 0 | 0 | 0 | 1 | 0 | 0 | 32 | 43 | Carbamidomethylation | C11:Carbamidomethylation:1000.00 | PEAKS DB |
| total 2 peptides |
| --- |

A4VBF0|PA2H\_VIPBN

back to list

  

| Protein Coverage
| Supporting Peptides
|

Protein Coverage:

Supporting Peptides:

| Peptide | Uniq | -10lgP | Mass | Length | ppm | m/z | z | RT | Fraction | Scan | Source File | Area F1 | Area F10 | Area F2 | Area F5 | Area F7A | Area F7B | Area F8A | Area F8B | Area F9 | #Feature | #Feature F1 | #Feature F10 | #Feature F2 | #Feature F5 | #Feature F7A | #Feature F7B | #Feature F8A | #Feature F8B | #Feature F9 | Start | End | PTM | AScore | Found By |
| --- | --- | --- | --- | --- | --- | --- | --- | --- | --- | --- | --- | --- | --- | --- | --- | --- | --- | --- | --- | --- | --- | --- | --- | --- | --- | --- | --- | --- | --- | --- | --- | --- | --- | --- | --- |
| R.AAAIC(+57.02)LGENVNTYDK.N | Y | 68.12 | 1637.7719 | 15 | 8.7 | 819.8942 | 2 | 40.75 | 8 | F8:18687 | DaRuWB\_F8B.raw |  |  |  |  |  |  |  | 2.5614E7 |  | 5 | 0 | 0 | 0 | 0 | 0 | 0 | 0 | 5 | 0 | 107 | 121 | Carbamidomethylation | C5:Carbamidomethylation:1000.00 | PEAKS DB |
| K.EAVHSYAIYGC(+57.02)Y.C | N | 40.08 | 1431.6129 | 12 | 2.4 | 716.8139 | 2 | 45.05 | 7 | F7:19798 | DaRuWB\_F8A.raw |  |  |  |  |  |  | 4.5632E6 |  |  | 1 | 0 | 0 | 0 | 0 | 0 | 0 | 1 | 0 | 0 | 32 | 43 | Carbamidomethylation | C11:Carbamidomethylation:1000.00 | PEAKS DB |
| total 2 peptides |
| --- |

P04084|PA2A\_VIPAE

back to list

  

| Protein Coverage
| Supporting Peptides
|

Protein Coverage:

Supporting Peptides:

| Peptide | Uniq | -10lgP | Mass | Length | ppm | m/z | z | RT | Fraction | Scan | Source File | Area F1 | Area F10 | Area F2 | Area F5 | Area F7A | Area F7B | Area F8A | Area F8B | Area F9 | #Feature | #Feature F1 | #Feature F10 | #Feature F2 | #Feature F5 | #Feature F7A | #Feature F7B | #Feature F8A | #Feature F8B | #Feature F9 | Start | End | PTM | AScore | Found By |
| --- | --- | --- | --- | --- | --- | --- | --- | --- | --- | --- | --- | --- | --- | --- | --- | --- | --- | --- | --- | --- | --- | --- | --- | --- | --- | --- | --- | --- | --- | --- | --- | --- | --- | --- | --- |
| R.AAAIC(+57.02)LGENVNTYDK.N | Y | 68.12 | 1637.7719 | 15 | 8.7 | 819.8942 | 2 | 40.75 | 8 | F8:18687 | DaRuWB\_F8B.raw |  |  |  |  |  |  |  | 2.5614E7 |  | 5 | 0 | 0 | 0 | 0 | 0 | 0 | 0 | 5 | 0 | 91 | 105 | Carbamidomethylation | C5:Carbamidomethylation:1000.00 | PEAKS DB |
| K.EAVHSYAIYGC(+57.02)Y.C | N | 40.08 | 1431.6129 | 12 | 2.4 | 716.8139 | 2 | 45.05 | 7 | F7:19798 | DaRuWB\_F8A.raw |  |  |  |  |  |  | 4.5632E6 |  |  | 1 | 0 | 0 | 0 | 0 | 0 | 0 | 1 | 0 | 0 | 16 | 27 | Carbamidomethylation | C11:Carbamidomethylation:1000.00 | PEAKS DB |
| total 2 peptides |
| --- |

Q38L02|SLA\_DABSI

back to list

  

| Protein Coverage
| Supporting Peptides
|

Protein Coverage:

Supporting Peptides:

| Peptide | Uniq | -10lgP | Mass | Length | ppm | m/z | z | RT | Fraction | Scan | Source File | Area F1 | Area F10 | Area F2 | Area F5 | Area F7A | Area F7B | Area F8A | Area F8B | Area F9 | #Feature | #Feature F1 | #Feature F10 | #Feature F2 | #Feature F5 | #Feature F7A | #Feature F7B | #Feature F8A | #Feature F8B | #Feature F9 | Start | End | PTM | AScore | Found By |
| --- | --- | --- | --- | --- | --- | --- | --- | --- | --- | --- | --- | --- | --- | --- | --- | --- | --- | --- | --- | --- | --- | --- | --- | --- | --- | --- | --- | --- | --- | --- | --- | --- | --- | --- | --- |
| H.LASIESVEEANFVAQLASETLTK.S | Y | 54.04 | 2449.2588 | 23 | 4.0 | 817.4279 | 3 | 88.48 | 6 | F6:59887 | DaRuWB\_F7B.raw |  |  |  |  |  | 2.2022E7 |  |  |  | 1 | 0 | 0 | 0 | 0 | 0 | 1 | 0 | 0 | 0 | 62 | 84 |  |  | PEAKS DB |
| K.YHEWITLPC(+57.02)GDK.N | Y | 39.92 | 1517.6973 | 12 | 3.1 | 759.8566 | 2 | 44.91 | 7 | F7:19990 | DaRuWB\_F8A.raw |  |  |  |  |  |  | 6.6672E6 |  |  | 1 | 0 | 0 | 0 | 0 | 0 | 0 | 1 | 0 | 0 | 132 | 143 | Carbamidomethylation | C9:Carbamidomethylation:1000.00 | PEAKS DB |
| total 2 peptides |
| --- |

B8K1W0|VM3DK\_DABRR

back to list

  

| Protein Coverage
| Supporting Peptides
|

Protein Coverage:

Supporting Peptides:

| Peptide | Uniq | -10lgP | Mass | Length | ppm | m/z | z | RT | Fraction | Scan | Source File | Area F1 | Area F10 | Area F2 | Area F5 | Area F7A | Area F7B | Area F8A | Area F8B | Area F9 | #Feature | #Feature F1 | #Feature F10 | #Feature F2 | #Feature F5 | #Feature F7A | #Feature F7B | #Feature F8A | #Feature F8B | #Feature F9 | Start | End | PTM | AScore | Found By |
| --- | --- | --- | --- | --- | --- | --- | --- | --- | --- | --- | --- | --- | --- | --- | --- | --- | --- | --- | --- | --- | --- | --- | --- | --- | --- | --- | --- | --- | --- | --- | --- | --- | --- | --- | --- |
| R.SANC(+57.02)PVDEFHENGR.P | Y | 47.30 | 1630.6794 | 14 | 0.8 | 816.3477 | 2 | 21.69 | 2 | F2:4026 | DaRuWB\_F10.raw |  | 1.5967E5 |  |  |  |  |  |  |  | 1 | 0 | 1 | 0 | 0 | 0 | 0 | 0 | 0 | 0 | 482 | 495 | Carbamidomethylation | C4:Carbamidomethylation:1000.00 | PEAKS DB |
| K.VC(+57.02)SNGQC(+57.02)VDLNIAY | Y | 43.01 | 1611.7021 | 14 | 0.8 | 806.8590 | 2 | 59.07 | 2 | F2:13136 | DaRuWB\_F10.raw |  | 2.165E6 |  |  |  |  |  |  |  | 1 | 0 | 1 | 0 | 0 | 0 | 0 | 0 | 0 | 0 | 602 | 615 | Carbamidomethylation | C2:Carbamidomethylation:1000.00;C7:Carbamidomethylation:1000.00 | PEAKS DB |
| total 2 peptides |
| --- |

Q3YJS9|PT3K1\_SOLTU

back to list

  

| Protein Coverage
| Supporting Peptides
|

Protein Coverage:

Supporting Peptides:

| Peptide | Uniq | -10lgP | Mass | Length | ppm | m/z | z | RT | Fraction | Scan | Source File | Area F1 | Area F10 | Area F2 | Area F5 | Area F7A | Area F7B | Area F8A | Area F8B | Area F9 | #Feature | #Feature F1 | #Feature F10 | #Feature F2 | #Feature F5 | #Feature F7A | #Feature F7B | #Feature F8A | #Feature F8B | #Feature F9 | Start | End | PTM | AScore | Found By |
| --- | --- | --- | --- | --- | --- | --- | --- | --- | --- | --- | --- | --- | --- | --- | --- | --- | --- | --- | --- | --- | --- | --- | --- | --- | --- | --- | --- | --- | --- | --- | --- | --- | --- | --- | --- |
| R.VHQALTEVAISSFDIK.T | N | 43.81 | 1756.9359 | 16 | 8.1 | 586.6530 | 3 | 55.50 | 1 | F1:21166 | DaRuWB\_F1.raw | 7.1562E5 |  |  |  |  |  |  |  |  | 1 | 1 | 0 | 0 | 0 | 0 | 0 | 0 | 0 | 0 | 132 | 147 |  |  | PEAKS DB |
| R.LAQEDPAFASIK.S | Y | 43.73 | 1288.6663 | 12 | 7.5 | 645.3405 | 2 | 39.85 | 1 | F1:15915 | DaRuWB\_F1.raw | 1.0466E6 |  |  |  |  |  |  |  |  | 1 | 1 | 0 | 0 | 0 | 0 | 0 | 0 | 0 | 0 | 223 | 234 |  |  | PEAKS DB |
| total 2 peptides |
| --- |

Q4PRC6|SL7\_DABSI

back to list

  

| Protein Coverage
| Supporting Peptides
|

Protein Coverage:

Supporting Peptides:

| Peptide | Uniq | -10lgP | Mass | Length | ppm | m/z | z | RT | Fraction | Scan | Source File | Area F1 | Area F10 | Area F2 | Area F5 | Area F7A | Area F7B | Area F8A | Area F8B | Area F9 | #Feature | #Feature F1 | #Feature F10 | #Feature F2 | #Feature F5 | #Feature F7A | #Feature F7B | #Feature F8A | #Feature F8B | #Feature F9 | Start | End | PTM | AScore | Found By |
| --- | --- | --- | --- | --- | --- | --- | --- | --- | --- | --- | --- | --- | --- | --- | --- | --- | --- | --- | --- | --- | --- | --- | --- | --- | --- | --- | --- | --- | --- | --- | --- | --- | --- | --- | --- |
| K.FC(+57.02)NEQVNGGYLVSFR.S | Y | 47.45 | 1788.8253 | 15 | 0.6 | 597.2827 | 3 | 55.34 | 9 | F9:11348 | DaRuWB\_F9.raw |  |  |  |  |  |  |  |  | 1.4055E5 | 1 | 0 | 0 | 0 | 0 | 0 | 0 | 0 | 0 | 1 | 54 | 68 | Carbamidomethylation | C2:Carbamidomethylation:1000.00 | PEAKS DB |
| total 1 peptides |
| --- |

Q00652|CPI9\_SOLTU

back to list

  

| Protein Coverage
| Supporting Peptides
|

Protein Coverage:

Supporting Peptides:

| Peptide | Uniq | -10lgP | Mass | Length | ppm | m/z | z | RT | Fraction | Scan | Source File | Area F1 | Area F10 | Area F2 | Area F5 | Area F7A | Area F7B | Area F8A | Area F8B | Area F9 | #Feature | #Feature F1 | #Feature F10 | #Feature F2 | #Feature F5 | #Feature F7A | #Feature F7B | #Feature F8A | #Feature F8B | #Feature F9 | Start | End | PTM | AScore | Found By |
| --- | --- | --- | --- | --- | --- | --- | --- | --- | --- | --- | --- | --- | --- | --- | --- | --- | --- | --- | --- | --- | --- | --- | --- | --- | --- | --- | --- | --- | --- | --- | --- | --- | --- | --- | --- |
| N.LVLPEVYDQDGHPLR.I | Y | 48.09 | 1749.9049 | 15 | 7.6 | 584.3090 | 3 | 50.09 | 1 | F1:19307 | DaRuWB\_F1.raw | 2.9655E5 |  |  |  |  |  |  |  |  | 1 | 1 | 0 | 0 | 0 | 0 | 0 | 0 | 0 | 0 | 43 | 57 |  |  | PEAKS DB |
| Q.VGNENDIFK.I | N | 44.23 | 1034.5033 | 9 | 6.7 | 518.2585 | 2 | 30.00 | 1 | F1:11774 | DaRuWB\_F1.raw | 1.8699E6 |  |  |  |  |  |  |  |  | 1 | 1 | 0 | 0 | 0 | 0 | 0 | 0 | 0 | 0 | 156 | 164 |  |  | PEAKS DB |
| total 2 peptides |
| --- |

D1MGU0|SLA\_PROJR

back to list

  

| Protein Coverage
| Supporting Peptides
|

Protein Coverage:

Supporting Peptides:

| Peptide | Uniq | -10lgP | Mass | Length | ppm | m/z | z | RT | Fraction | Scan | Source File | Area F1 | Area F10 | Area F2 | Area F5 | Area F7A | Area F7B | Area F8A | Area F8B | Area F9 | #Feature | #Feature F1 | #Feature F10 | #Feature F2 | #Feature F5 | #Feature F7A | #Feature F7B | #Feature F8A | #Feature F8B | #Feature F9 | Start | End | PTM | AScore | Found By |
| --- | --- | --- | --- | --- | --- | --- | --- | --- | --- | --- | --- | --- | --- | --- | --- | --- | --- | --- | --- | --- | --- | --- | --- | --- | --- | --- | --- | --- | --- | --- | --- | --- | --- | --- | --- |
| R.EAVFVAQLLSENIK.A | Y | 66.27 | 1559.8558 | 14 | 7.9 | 780.9355 | 2 | 74.33 | 8 | F8:45015 | DaRuWB\_F8B.raw |  |  |  |  |  | 9.0764E5 |  | 4.0188E6 |  | 2 | 0 | 0 | 0 | 0 | 0 | 1 | 0 | 1 | 0 | 70 | 83 |  |  | PEAKS DB |
| total 1 peptides |
| --- |

Q4PRD1|SLLC1\_DABSI

back to list

  

| Protein Coverage
| Supporting Peptides
|

Protein Coverage:

Supporting Peptides:

| Peptide | Uniq | -10lgP | Mass | Length | ppm | m/z | z | RT | Fraction | Scan | Source File | Area F1 | Area F10 | Area F2 | Area F5 | Area F7A | Area F7B | Area F8A | Area F8B | Area F9 | #Feature | #Feature F1 | #Feature F10 | #Feature F2 | #Feature F5 | #Feature F7A | #Feature F7B | #Feature F8A | #Feature F8B | #Feature F9 | Start | End | PTM | AScore | Found By |
| --- | --- | --- | --- | --- | --- | --- | --- | --- | --- | --- | --- | --- | --- | --- | --- | --- | --- | --- | --- | --- | --- | --- | --- | --- | --- | --- | --- | --- | --- | --- | --- | --- | --- | --- | --- |
| K.SMTC(+57.02)NFIAPVVC(+57.02)K.F | Y | 45.63 | 1525.7091 | 13 | 3.3 | 763.8627 | 2 | 51.23 | 7 | F7:23992 | DaRuWB\_F8A.raw |  |  |  |  |  |  | 3.934E5 |  |  | 1 | 0 | 0 | 0 | 0 | 0 | 0 | 1 | 0 | 0 | 133 | 145 | Carbamidomethylation | C4:Carbamidomethylation:1000.00;C12:Carbamidomethylation:1000.00 | PEAKS DB |
| K.ALAEESYC(+57.02)LIMITHEK.E | Y | 40.00 | 1906.9169 | 16 | 3.0 | 636.6464 | 3 | 54.86 | 6 | F6:32154 | DaRuWB\_F7B.raw |  |  |  |  |  | 9.605E5 |  | 8.9402E5 |  | 2 | 0 | 0 | 0 | 0 | 0 | 1 | 0 | 1 | 0 | 114 | 129 | Carbamidomethylation | C8:Carbamidomethylation:1000.00 | PEAKS DB |
| total 2 peptides |
| --- |

Q4PRC7|SL6\_DABSI

back to list

  

| Protein Coverage
| Supporting Peptides
|

Protein Coverage:

Supporting Peptides:

| Peptide | Uniq | -10lgP | Mass | Length | ppm | m/z | z | RT | Fraction | Scan | Source File | Area F1 | Area F10 | Area F2 | Area F5 | Area F7A | Area F7B | Area F8A | Area F8B | Area F9 | #Feature | #Feature F1 | #Feature F10 | #Feature F2 | #Feature F5 | #Feature F7A | #Feature F7B | #Feature F8A | #Feature F8B | #Feature F9 | Start | End | PTM | AScore | Found By |
| --- | --- | --- | --- | --- | --- | --- | --- | --- | --- | --- | --- | --- | --- | --- | --- | --- | --- | --- | --- | --- | --- | --- | --- | --- | --- | --- | --- | --- | --- | --- | --- | --- | --- | --- | --- |
| K.SMTC(+57.02)NFIAPVVC(+57.02)K.F | Y | 45.63 | 1525.7091 | 13 | 3.3 | 763.8627 | 2 | 51.23 | 7 | F7:23992 | DaRuWB\_F8A.raw |  |  |  |  |  |  | 3.934E5 |  |  | 1 | 0 | 0 | 0 | 0 | 0 | 0 | 1 | 0 | 0 | 131 | 143 | Carbamidomethylation | C4:Carbamidomethylation:1000.00;C12:Carbamidomethylation:1000.00 | PEAKS DB |
| K.ALAEESYC(+57.02)LIMITHEK.V | Y | 40.00 | 1906.9169 | 16 | 3.0 | 636.6464 | 3 | 54.86 | 6 | F6:32154 | DaRuWB\_F7B.raw |  |  |  |  |  | 9.605E5 |  | 8.9402E5 |  | 2 | 0 | 0 | 0 | 0 | 0 | 1 | 0 | 1 | 0 | 112 | 127 | Carbamidomethylation | C8:Carbamidomethylation:1000.00 | PEAKS DB |
| total 2 peptides |
| --- |

Q4PRC9|SL4\_DABSI

back to list

  

| Protein Coverage
| Supporting Peptides
|

Protein Coverage:

Supporting Peptides:

| Peptide | Uniq | -10lgP | Mass | Length | ppm | m/z | z | RT | Fraction | Scan | Source File | Area F1 | Area F10 | Area F2 | Area F5 | Area F7A | Area F7B | Area F8A | Area F8B | Area F9 | #Feature | #Feature F1 | #Feature F10 | #Feature F2 | #Feature F5 | #Feature F7A | #Feature F7B | #Feature F8A | #Feature F8B | #Feature F9 | Start | End | PTM | AScore | Found By |
| --- | --- | --- | --- | --- | --- | --- | --- | --- | --- | --- | --- | --- | --- | --- | --- | --- | --- | --- | --- | --- | --- | --- | --- | --- | --- | --- | --- | --- | --- | --- | --- | --- | --- | --- | --- |
| K.SMTC(+57.02)NFIAPVVC(+57.02)K.F | Y | 45.63 | 1525.7091 | 13 | 3.3 | 763.8627 | 2 | 51.23 | 7 | F7:23992 | DaRuWB\_F8A.raw |  |  |  |  |  |  | 3.934E5 |  |  | 1 | 0 | 0 | 0 | 0 | 0 | 0 | 1 | 0 | 0 | 133 | 145 | Carbamidomethylation | C4:Carbamidomethylation:1000.00;C12:Carbamidomethylation:1000.00 | PEAKS DB |
| K.ALAEESYC(+57.02)LIMITHEK.V | Y | 40.00 | 1906.9169 | 16 | 3.0 | 636.6464 | 3 | 54.86 | 6 | F6:32154 | DaRuWB\_F7B.raw |  |  |  |  |  | 9.605E5 |  | 8.9402E5 |  | 2 | 0 | 0 | 0 | 0 | 0 | 1 | 0 | 1 | 0 | 114 | 129 | Carbamidomethylation | C8:Carbamidomethylation:1000.00 | PEAKS DB |
| total 2 peptides |
| --- |

P58515|SPI2\_SOLTU

back to list

  

| Protein Coverage
| Supporting Peptides
|

Protein Coverage:

Supporting Peptides:

| Peptide | Uniq | -10lgP | Mass | Length | ppm | m/z | z | RT | Fraction | Scan | Source File | Area F1 | Area F10 | Area F2 | Area F5 | Area F7A | Area F7B | Area F8A | Area F8B | Area F9 | #Feature | #Feature F1 | #Feature F10 | #Feature F2 | #Feature F5 | #Feature F7A | #Feature F7B | #Feature F8A | #Feature F8B | #Feature F9 | Start | End | PTM | AScore | Found By |
| --- | --- | --- | --- | --- | --- | --- | --- | --- | --- | --- | --- | --- | --- | --- | --- | --- | --- | --- | --- | --- | --- | --- | --- | --- | --- | --- | --- | --- | --- | --- | --- | --- | --- | --- | --- |
| F.WGALGGDVYLGK.S | Y | 47.48 | 1234.6345 | 12 | 8.5 | 618.3252 | 2 | 54.68 | 1 | F1:20872 | DaRuWB\_F1.raw | 1.3526E6 |  |  |  |  |  |  |  |  | 1 | 1 | 0 | 0 | 0 | 0 | 0 | 0 | 0 | 0 | 29 | 40 |  |  | PEAKS DB |
| total 1 peptides |
| --- |

Q41433|SPI6\_SOLTU

back to list

  

| Protein Coverage
| Supporting Peptides
|

Protein Coverage:

Supporting Peptides:

| Peptide | Uniq | -10lgP | Mass | Length | ppm | m/z | z | RT | Fraction | Scan | Source File | Area F1 | Area F10 | Area F2 | Area F5 | Area F7A | Area F7B | Area F8A | Area F8B | Area F9 | #Feature | #Feature F1 | #Feature F10 | #Feature F2 | #Feature F5 | #Feature F7A | #Feature F7B | #Feature F8A | #Feature F8B | #Feature F9 | Start | End | PTM | AScore | Found By |
| --- | --- | --- | --- | --- | --- | --- | --- | --- | --- | --- | --- | --- | --- | --- | --- | --- | --- | --- | --- | --- | --- | --- | --- | --- | --- | --- | --- | --- | --- | --- | --- | --- | --- | --- | --- |
| F.WGALGGDVYLGK.S | Y | 47.48 | 1234.6345 | 12 | 8.5 | 618.3252 | 2 | 54.68 | 1 | F1:20872 | DaRuWB\_F1.raw | 1.3526E6 |  |  |  |  |  |  |  |  | 1 | 1 | 0 | 0 | 0 | 0 | 0 | 0 | 0 | 0 | 57 | 68 |  |  | PEAKS DB |
| total 1 peptides |
| --- |

P58514|SPI1\_SOLTU

back to list

  

| Protein Coverage
| Supporting Peptides
|

Protein Coverage:

Supporting Peptides:

| Peptide | Uniq | -10lgP | Mass | Length | ppm | m/z | z | RT | Fraction | Scan | Source File | Area F1 | Area F10 | Area F2 | Area F5 | Area F7A | Area F7B | Area F8A | Area F8B | Area F9 | #Feature | #Feature F1 | #Feature F10 | #Feature F2 | #Feature F5 | #Feature F7A | #Feature F7B | #Feature F8A | #Feature F8B | #Feature F9 | Start | End | PTM | AScore | Found By |
| --- | --- | --- | --- | --- | --- | --- | --- | --- | --- | --- | --- | --- | --- | --- | --- | --- | --- | --- | --- | --- | --- | --- | --- | --- | --- | --- | --- | --- | --- | --- | --- | --- | --- | --- | --- |
| F.WGALGGDVYLGK.S | Y | 47.48 | 1234.6345 | 12 | 8.5 | 618.3252 | 2 | 54.68 | 1 | F1:20872 | DaRuWB\_F1.raw | 1.3526E6 |  |  |  |  |  |  |  |  | 1 | 1 | 0 | 0 | 0 | 0 | 0 | 0 | 0 | 0 | 57 | 68 |  |  | PEAKS DB |
| total 1 peptides |
| --- |

Q41480|API1\_SOLTU

back to list

  

| Protein Coverage
| Supporting Peptides
|

Protein Coverage:

Supporting Peptides:

| Peptide | Uniq | -10lgP | Mass | Length | ppm | m/z | z | RT | Fraction | Scan | Source File | Area F1 | Area F10 | Area F2 | Area F5 | Area F7A | Area F7B | Area F8A | Area F8B | Area F9 | #Feature | #Feature F1 | #Feature F10 | #Feature F2 | #Feature F5 | #Feature F7A | #Feature F7B | #Feature F8A | #Feature F8B | #Feature F9 | Start | End | PTM | AScore | Found By |
| --- | --- | --- | --- | --- | --- | --- | --- | --- | --- | --- | --- | --- | --- | --- | --- | --- | --- | --- | --- | --- | --- | --- | --- | --- | --- | --- | --- | --- | --- | --- | --- | --- | --- | --- | --- |
| F.WGALGGDVYLGK.S | Y | 47.48 | 1234.6345 | 12 | 8.5 | 618.3252 | 2 | 54.68 | 1 | F1:20872 | DaRuWB\_F1.raw | 1.3526E6 |  |  |  |  |  |  |  |  | 1 | 1 | 0 | 0 | 0 | 0 | 0 | 0 | 0 | 0 | 61 | 72 |  |  | PEAKS DB |
| total 1 peptides |
| --- |

P30941|SPI7\_SOLTU

back to list

  

| Protein Coverage
| Supporting Peptides
|

Protein Coverage:

Supporting Peptides:

| Peptide | Uniq | -10lgP | Mass | Length | ppm | m/z | z | RT | Fraction | Scan | Source File | Area F1 | Area F10 | Area F2 | Area F5 | Area F7A | Area F7B | Area F8A | Area F8B | Area F9 | #Feature | #Feature F1 | #Feature F10 | #Feature F2 | #Feature F5 | #Feature F7A | #Feature F7B | #Feature F8A | #Feature F8B | #Feature F9 | Start | End | PTM | AScore | Found By |
| --- | --- | --- | --- | --- | --- | --- | --- | --- | --- | --- | --- | --- | --- | --- | --- | --- | --- | --- | --- | --- | --- | --- | --- | --- | --- | --- | --- | --- | --- | --- | --- | --- | --- | --- | --- |
| F.WGALGGDVYLGK.S | Y | 47.48 | 1234.6345 | 12 | 8.5 | 618.3252 | 2 | 54.68 | 1 | F1:20872 | DaRuWB\_F1.raw | 1.3526E6 |  |  |  |  |  |  |  |  | 1 | 1 | 0 | 0 | 0 | 0 | 0 | 0 | 0 | 0 | 57 | 68 |  |  | PEAKS DB |
| total 1 peptides |
| --- |

Q41448|API7\_SOLTU

back to list

  

| Protein Coverage
| Supporting Peptides
|

Protein Coverage:

Supporting Peptides:

| Peptide | Uniq | -10lgP | Mass | Length | ppm | m/z | z | RT | Fraction | Scan | Source File | Area F1 | Area F10 | Area F2 | Area F5 | Area F7A | Area F7B | Area F8A | Area F8B | Area F9 | #Feature | #Feature F1 | #Feature F10 | #Feature F2 | #Feature F5 | #Feature F7A | #Feature F7B | #Feature F8A | #Feature F8B | #Feature F9 | Start | End | PTM | AScore | Found By |
| --- | --- | --- | --- | --- | --- | --- | --- | --- | --- | --- | --- | --- | --- | --- | --- | --- | --- | --- | --- | --- | --- | --- | --- | --- | --- | --- | --- | --- | --- | --- | --- | --- | --- | --- | --- |
| F.WGALGGDVYLGK.S | Y | 47.48 | 1234.6345 | 12 | 8.5 | 618.3252 | 2 | 54.68 | 1 | F1:20872 | DaRuWB\_F1.raw | 1.3526E6 |  |  |  |  |  |  |  |  | 1 | 1 | 0 | 0 | 0 | 0 | 0 | 0 | 0 | 0 | 61 | 72 |  |  | PEAKS DB |
| total 1 peptides |
| --- |

P85487|UP03\_PINHA

back to list

  

| Protein Coverage
| Supporting Peptides
|

Protein Coverage:

Supporting Peptides:

| Peptide | Uniq | -10lgP | Mass | Length | ppm | m/z | z | RT | Fraction | Scan | Source File | Area F1 | Area F10 | Area F2 | Area F5 | Area F7A | Area F7B | Area F8A | Area F8B | Area F9 | #Feature | #Feature F1 | #Feature F10 | #Feature F2 | #Feature F5 | #Feature F7A | #Feature F7B | #Feature F8A | #Feature F8B | #Feature F9 | Start | End | PTM | AScore | Found By |
| --- | --- | --- | --- | --- | --- | --- | --- | --- | --- | --- | --- | --- | --- | --- | --- | --- | --- | --- | --- | --- | --- | --- | --- | --- | --- | --- | --- | --- | --- | --- | --- | --- | --- | --- | --- |
| WVDADLNGLR | Y | 44.15 | 1157.5829 | 10 | 9.1 | 579.8008 | 2 | 34.79 | 5 | F5:15260 | DaRuWB\_F7A.raw |  |  |  |  | 0 |  |  |  |  | 0 | 0 | 0 | 0 | 0 | 0 | 0 | 0 | 0 | 0 | 1 | 10 |  |  | PEAKS DB |
| total 1 peptides |
| --- |

P15445|PA2A2\_NAJNA

back to list

  

| Protein Coverage
| Supporting Peptides
|

Protein Coverage:

Supporting Peptides:

| Peptide | Uniq | -10lgP | Mass | Length | ppm | m/z | z | RT | Fraction | Scan | Source File | Area F1 | Area F10 | Area F2 | Area F5 | Area F7A | Area F7B | Area F8A | Area F8B | Area F9 | #Feature | #Feature F1 | #Feature F10 | #Feature F2 | #Feature F5 | #Feature F7A | #Feature F7B | #Feature F8A | #Feature F8B | #Feature F9 | Start | End | PTM | AScore | Found By |
| --- | --- | --- | --- | --- | --- | --- | --- | --- | --- | --- | --- | --- | --- | --- | --- | --- | --- | --- | --- | --- | --- | --- | --- | --- | --- | --- | --- | --- | --- | --- | --- | --- | --- | --- | --- |
| G.DNNAC(+57.02)AASVC(+57.02)DC(+57.02)DR.L | Y | 41.21 | 1626.5820 | 14 | 3.6 | 814.2994 | 2 | 16.16 | 7 | F7:3110 | DaRuWB\_F8A.raw |  |  |  |  |  |  | 4.2829E6 |  |  | 1 | 0 | 0 | 0 | 0 | 0 | 0 | 1 | 0 | 0 | 81 | 94 | Carbamidomethylation | C5:Carbamidomethylation:1000.00;C10:Carbamidomethylation:1000.00;C12:Carbamidomethylation:1000.00 | PEAKS DB |
| total 1 peptides |
| --- |

P60045|PA2A3\_NAJSG

back to list

  

| Protein Coverage
| Supporting Peptides
|

Protein Coverage:

Supporting Peptides:

| Peptide | Uniq | -10lgP | Mass | Length | ppm | m/z | z | RT | Fraction | Scan | Source File | Area F1 | Area F10 | Area F2 | Area F5 | Area F7A | Area F7B | Area F8A | Area F8B | Area F9 | #Feature | #Feature F1 | #Feature F10 | #Feature F2 | #Feature F5 | #Feature F7A | #Feature F7B | #Feature F8A | #Feature F8B | #Feature F9 | Start | End | PTM | AScore | Found By |
| --- | --- | --- | --- | --- | --- | --- | --- | --- | --- | --- | --- | --- | --- | --- | --- | --- | --- | --- | --- | --- | --- | --- | --- | --- | --- | --- | --- | --- | --- | --- | --- | --- | --- | --- | --- |
| G.DNNAC(+57.02)AASVC(+57.02)DC(+57.02)DR.L | Y | 41.21 | 1626.5820 | 14 | 3.6 | 814.2994 | 2 | 16.16 | 7 | F7:3110 | DaRuWB\_F8A.raw |  |  |  |  |  |  | 4.2829E6 |  |  | 1 | 0 | 0 | 0 | 0 | 0 | 0 | 1 | 0 | 0 | 88 | 101 | Carbamidomethylation | C5:Carbamidomethylation:1000.00;C10:Carbamidomethylation:1000.00;C12:Carbamidomethylation:1000.00 | PEAKS DB |
| total 1 peptides |
| --- |

Q4PRC8|SL5\_DABSI

back to list

  

| Protein Coverage
| Supporting Peptides
|

Protein Coverage:

Supporting Peptides:

| Peptide | Uniq | -10lgP | Mass | Length | ppm | m/z | z | RT | Fraction | Scan | Source File | Area F1 | Area F10 | Area F2 | Area F5 | Area F7A | Area F7B | Area F8A | Area F8B | Area F9 | #Feature | #Feature F1 | #Feature F10 | #Feature F2 | #Feature F5 | #Feature F7A | #Feature F7B | #Feature F8A | #Feature F8B | #Feature F9 | Start | End | PTM | AScore | Found By |
| --- | --- | --- | --- | --- | --- | --- | --- | --- | --- | --- | --- | --- | --- | --- | --- | --- | --- | --- | --- | --- | --- | --- | --- | --- | --- | --- | --- | --- | --- | --- | --- | --- | --- | --- | --- |
| K.AWNEGTNC(+57.02)FVFK.I | Y | 41.12 | 1471.6554 | 12 | 1.2 | 736.8359 | 2 | 52.53 | 9 | F9:10755 | DaRuWB\_F9.raw |  |  |  |  |  |  |  |  | 6.465E6 | 1 | 0 | 0 | 0 | 0 | 0 | 0 | 0 | 0 | 1 | 114 | 125 | Carbamidomethylation | C8:Carbamidomethylation:1000.00 | PEAKS DB |
| total 1 peptides |
| --- |

Q4PRD0|SL3\_DABSI

back to list

  

| Protein Coverage
| Supporting Peptides
|

Protein Coverage:

Supporting Peptides:

| Peptide | Uniq | -10lgP | Mass | Length | ppm | m/z | z | RT | Fraction | Scan | Source File | Area F1 | Area F10 | Area F2 | Area F5 | Area F7A | Area F7B | Area F8A | Area F8B | Area F9 | #Feature | #Feature F1 | #Feature F10 | #Feature F2 | #Feature F5 | #Feature F7A | #Feature F7B | #Feature F8A | #Feature F8B | #Feature F9 | Start | End | PTM | AScore | Found By |
| --- | --- | --- | --- | --- | --- | --- | --- | --- | --- | --- | --- | --- | --- | --- | --- | --- | --- | --- | --- | --- | --- | --- | --- | --- | --- | --- | --- | --- | --- | --- | --- | --- | --- | --- | --- |
| K.AWNEGTNC(+57.02)FVFK.I | Y | 41.12 | 1471.6554 | 12 | 1.2 | 736.8359 | 2 | 52.53 | 9 | F9:10755 | DaRuWB\_F9.raw |  |  |  |  |  |  |  |  | 6.465E6 | 1 | 0 | 0 | 0 | 0 | 0 | 0 | 0 | 0 | 1 | 114 | 125 | Carbamidomethylation | C8:Carbamidomethylation:1000.00 | PEAKS DB |
| total 1 peptides |
| --- |

P0CG03|VSPBH\_BOTAL

back to list

  

| Protein Coverage
| Supporting Peptides
|

Protein Coverage:

Supporting Peptides:

| Peptide | Uniq | -10lgP | Mass | Length | ppm | m/z | z | RT | Fraction | Scan | Source File | Area F1 | Area F10 | Area F2 | Area F5 | Area F7A | Area F7B | Area F8A | Area F8B | Area F9 | #Feature | #Feature F1 | #Feature F10 | #Feature F2 | #Feature F5 | #Feature F7A | #Feature F7B | #Feature F8A | #Feature F8B | #Feature F9 | Start | End | PTM | AScore | Found By |
| --- | --- | --- | --- | --- | --- | --- | --- | --- | --- | --- | --- | --- | --- | --- | --- | --- | --- | --- | --- | --- | --- | --- | --- | --- | --- | --- | --- | --- | --- | --- | --- | --- | --- | --- | --- |
| R.VMGWGTISPTK.V | Y | 40.53 | 1175.6008 | 11 | 3.0 | 588.8078 | 2 | 45.56 | 6 | F6:24627 | DaRuWB\_F7B.raw |  |  |  |  |  | 1.9742E5 |  |  |  | 1 | 0 | 0 | 0 | 0 | 0 | 1 | 0 | 0 | 0 | 146 | 156 |  |  | PEAKS DB |
| total 1 peptides |
| --- |

P0DJL3|SLB\_TRIPP

back to list

  

| Protein Coverage
| Supporting Peptides
|

Protein Coverage:

Supporting Peptides:

| Peptide | Uniq | -10lgP | Mass | Length | ppm | m/z | z | RT | Fraction | Scan | Source File | Area F1 | Area F10 | Area F2 | Area F5 | Area F7A | Area F7B | Area F8A | Area F8B | Area F9 | #Feature | #Feature F1 | #Feature F10 | #Feature F2 | #Feature F5 | #Feature F7A | #Feature F7B | #Feature F8A | #Feature F8B | #Feature F9 | Start | End | PTM | AScore | Found By |
| --- | --- | --- | --- | --- | --- | --- | --- | --- | --- | --- | --- | --- | --- | --- | --- | --- | --- | --- | --- | --- | --- | --- | --- | --- | --- | --- | --- | --- | --- | --- | --- | --- | --- | --- | --- |
| R.LQWSDGTELK.Y | Y | 40.28 | 1175.5823 | 10 | 2.9 | 588.7988 | 2 | 40.12 | 7 | F7:16567 | DaRuWB\_F8A.raw |  |  |  |  |  |  | 5.4749E5 |  |  | 1 | 0 | 0 | 0 | 0 | 0 | 0 | 1 | 0 | 0 | 77 | 86 |  |  | PEAKS DB |
| total 1 peptides |
| --- |

P81116|SLBB\_TRIAB

back to list

  

| Protein Coverage
| Supporting Peptides
|

Protein Coverage:

Supporting Peptides:

| Peptide | Uniq | -10lgP | Mass | Length | ppm | m/z | z | RT | Fraction | Scan | Source File | Area F1 | Area F10 | Area F2 | Area F5 | Area F7A | Area F7B | Area F8A | Area F8B | Area F9 | #Feature | #Feature F1 | #Feature F10 | #Feature F2 | #Feature F5 | #Feature F7A | #Feature F7B | #Feature F8A | #Feature F8B | #Feature F9 | Start | End | PTM | AScore | Found By |
| --- | --- | --- | --- | --- | --- | --- | --- | --- | --- | --- | --- | --- | --- | --- | --- | --- | --- | --- | --- | --- | --- | --- | --- | --- | --- | --- | --- | --- | --- | --- | --- | --- | --- | --- | --- |
| R.LQWSDGTELK.Y | Y | 40.28 | 1175.5823 | 10 | 2.9 | 588.7988 | 2 | 40.12 | 7 | F7:16567 | DaRuWB\_F8A.raw |  |  |  |  |  |  | 5.4749E5 |  |  | 1 | 0 | 0 | 0 | 0 | 0 | 0 | 1 | 0 | 0 | 100 | 109 |  |  | PEAKS DB |
| total 1 peptides |
| --- |

D1MGU1|SLB\_PROJR

back to list

  

| Protein Coverage
| Supporting Peptides
|

Protein Coverage:

Supporting Peptides:

| Peptide | Uniq | -10lgP | Mass | Length | ppm | m/z | z | RT | Fraction | Scan | Source File | Area F1 | Area F10 | Area F2 | Area F5 | Area F7A | Area F7B | Area F8A | Area F8B | Area F9 | #Feature | #Feature F1 | #Feature F10 | #Feature F2 | #Feature F5 | #Feature F7A | #Feature F7B | #Feature F8A | #Feature F8B | #Feature F9 | Start | End | PTM | AScore | Found By |
| --- | --- | --- | --- | --- | --- | --- | --- | --- | --- | --- | --- | --- | --- | --- | --- | --- | --- | --- | --- | --- | --- | --- | --- | --- | --- | --- | --- | --- | --- | --- | --- | --- | --- | --- | --- |
| R.LQWSDGTELK.Y | Y | 40.28 | 1175.5823 | 10 | 2.9 | 588.7988 | 2 | 40.12 | 7 | F7:16567 | DaRuWB\_F8A.raw |  |  |  |  |  |  | 5.4749E5 |  |  | 1 | 0 | 0 | 0 | 0 | 0 | 0 | 1 | 0 | 0 | 100 | 109 |  |  | PEAKS DB |
| total 1 peptides |
| --- |

Q71RQ9|SLBB1\_TRIST

back to list

  

| Protein Coverage
| Supporting Peptides
|

Protein Coverage:

Supporting Peptides:

| Peptide | Uniq | -10lgP | Mass | Length | ppm | m/z | z | RT | Fraction | Scan | Source File | Area F1 | Area F10 | Area F2 | Area F5 | Area F7A | Area F7B | Area F8A | Area F8B | Area F9 | #Feature | #Feature F1 | #Feature F10 | #Feature F2 | #Feature F5 | #Feature F7A | #Feature F7B | #Feature F8A | #Feature F8B | #Feature F9 | Start | End | PTM | AScore | Found By |
| --- | --- | --- | --- | --- | --- | --- | --- | --- | --- | --- | --- | --- | --- | --- | --- | --- | --- | --- | --- | --- | --- | --- | --- | --- | --- | --- | --- | --- | --- | --- | --- | --- | --- | --- | --- |
| K.LQWSDGTELK.Y | Y | 40.28 | 1175.5823 | 10 | 2.9 | 588.7988 | 2 | 40.12 | 7 | F7:16567 | DaRuWB\_F8A.raw |  |  |  |  |  |  | 5.4749E5 |  |  | 1 | 0 | 0 | 0 | 0 | 0 | 0 | 1 | 0 | 0 | 100 | 109 |  |  | PEAKS DB |
| total 1 peptides |
| --- |

Q71RQ8|SLBB2\_TRIST

back to list

  

| Protein Coverage
| Supporting Peptides
|

Protein Coverage:

Supporting Peptides:

| Peptide | Uniq | -10lgP | Mass | Length | ppm | m/z | z | RT | Fraction | Scan | Source File | Area F1 | Area F10 | Area F2 | Area F5 | Area F7A | Area F7B | Area F8A | Area F8B | Area F9 | #Feature | #Feature F1 | #Feature F10 | #Feature F2 | #Feature F5 | #Feature F7A | #Feature F7B | #Feature F8A | #Feature F8B | #Feature F9 | Start | End | PTM | AScore | Found By |
| --- | --- | --- | --- | --- | --- | --- | --- | --- | --- | --- | --- | --- | --- | --- | --- | --- | --- | --- | --- | --- | --- | --- | --- | --- | --- | --- | --- | --- | --- | --- | --- | --- | --- | --- | --- |
| K.LQWSDGTELK.Y | Y | 40.28 | 1175.5823 | 10 | 2.9 | 588.7988 | 2 | 40.12 | 7 | F7:16567 | DaRuWB\_F8A.raw |  |  |  |  |  |  | 5.4749E5 |  |  | 1 | 0 | 0 | 0 | 0 | 0 | 0 | 1 | 0 | 0 | 100 | 109 |  |  | PEAKS DB |
| total 1 peptides |
| --- |

A8E2V8|PA2A\_TRIGS

back to list

  

| Protein Coverage
| Supporting Peptides
|

Protein Coverage:

Supporting Peptides:

| Peptide | Uniq | -10lgP | Mass | Length | ppm | m/z | z | RT | Fraction | Scan | Source File | Area F1 | Area F10 | Area F2 | Area F5 | Area F7A | Area F7B | Area F8A | Area F8B | Area F9 | #Feature | #Feature F1 | #Feature F10 | #Feature F2 | #Feature F5 | #Feature F7A | #Feature F7B | #Feature F8A | #Feature F8B | #Feature F9 | Start | End | PTM | AScore | Found By |
| --- | --- | --- | --- | --- | --- | --- | --- | --- | --- | --- | --- | --- | --- | --- | --- | --- | --- | --- | --- | --- | --- | --- | --- | --- | --- | --- | --- | --- | --- | --- | --- | --- | --- | --- | --- |
| Y.C(+57.02)GWGGQGRPQDATDR.C | Y | 39.48 | 1659.7172 | 15 | 1.4 | 830.8608 | 2 | 26.80 | 8 | F8:8064 | DaRuWB\_F8B.raw |  |  |  |  |  |  |  | 3.5116E5 |  | 1 | 0 | 0 | 0 | 0 | 0 | 0 | 0 | 1 | 0 | 44 | 58 | Carbamidomethylation | C1:Carbamidomethylation:1000.00 | PEAKS DB |
| total 1 peptides |
| --- |

Q800C2|PA2AG\_CROVV

back to list

  

| Protein Coverage
| Supporting Peptides
|

Protein Coverage:

Supporting Peptides:

| Peptide | Uniq | -10lgP | Mass | Length | ppm | m/z | z | RT | Fraction | Scan | Source File | Area F1 | Area F10 | Area F2 | Area F5 | Area F7A | Area F7B | Area F8A | Area F8B | Area F9 | #Feature | #Feature F1 | #Feature F10 | #Feature F2 | #Feature F5 | #Feature F7A | #Feature F7B | #Feature F8A | #Feature F8B | #Feature F9 | Start | End | PTM | AScore | Found By |
| --- | --- | --- | --- | --- | --- | --- | --- | --- | --- | --- | --- | --- | --- | --- | --- | --- | --- | --- | --- | --- | --- | --- | --- | --- | --- | --- | --- | --- | --- | --- | --- | --- | --- | --- | --- |
| Y.C(+57.02)GWGGQGRPQDATDR.C | Y | 39.48 | 1659.7172 | 15 | 1.4 | 830.8608 | 2 | 26.80 | 8 | F8:8064 | DaRuWB\_F8B.raw |  |  |  |  |  |  |  | 3.5116E5 |  | 1 | 0 | 0 | 0 | 0 | 0 | 0 | 0 | 1 | 0 | 44 | 58 | Carbamidomethylation | C1:Carbamidomethylation:1000.00 | PEAKS DB |
| total 1 peptides |
| --- |

Q800C3|PA2AF\_CROVV

back to list

  

| Protein Coverage
| Supporting Peptides
|

Protein Coverage:

Supporting Peptides:

| Peptide | Uniq | -10lgP | Mass | Length | ppm | m/z | z | RT | Fraction | Scan | Source File | Area F1 | Area F10 | Area F2 | Area F5 | Area F7A | Area F7B | Area F8A | Area F8B | Area F9 | #Feature | #Feature F1 | #Feature F10 | #Feature F2 | #Feature F5 | #Feature F7A | #Feature F7B | #Feature F8A | #Feature F8B | #Feature F9 | Start | End | PTM | AScore | Found By |
| --- | --- | --- | --- | --- | --- | --- | --- | --- | --- | --- | --- | --- | --- | --- | --- | --- | --- | --- | --- | --- | --- | --- | --- | --- | --- | --- | --- | --- | --- | --- | --- | --- | --- | --- | --- |
| Y.C(+57.02)GWGGQGRPQDATDR.C | Y | 39.48 | 1659.7172 | 15 | 1.4 | 830.8608 | 2 | 26.80 | 8 | F8:8064 | DaRuWB\_F8B.raw |  |  |  |  |  |  |  | 3.5116E5 |  | 1 | 0 | 0 | 0 | 0 | 0 | 0 | 0 | 1 | 0 | 44 | 58 | Carbamidomethylation | C1:Carbamidomethylation:1000.00 | PEAKS DB |
| total 1 peptides |
| --- |

Q7LZQ4|PA2A\_GLOUS

back to list

  

| Protein Coverage
| Supporting Peptides
|

Protein Coverage:

Supporting Peptides:

| Peptide | Uniq | -10lgP | Mass | Length | ppm | m/z | z | RT | Fraction | Scan | Source File | Area F1 | Area F10 | Area F2 | Area F5 | Area F7A | Area F7B | Area F8A | Area F8B | Area F9 | #Feature | #Feature F1 | #Feature F10 | #Feature F2 | #Feature F5 | #Feature F7A | #Feature F7B | #Feature F8A | #Feature F8B | #Feature F9 | Start | End | PTM | AScore | Found By |
| --- | --- | --- | --- | --- | --- | --- | --- | --- | --- | --- | --- | --- | --- | --- | --- | --- | --- | --- | --- | --- | --- | --- | --- | --- | --- | --- | --- | --- | --- | --- | --- | --- | --- | --- | --- |
| Y.C(+57.02)GWGGQGRPQDATDR.C | Y | 39.48 | 1659.7172 | 15 | 1.4 | 830.8608 | 2 | 26.80 | 8 | F8:8064 | DaRuWB\_F8B.raw |  |  |  |  |  |  |  | 3.5116E5 |  | 1 | 0 | 0 | 0 | 0 | 0 | 0 | 0 | 1 | 0 | 28 | 42 | Carbamidomethylation | C1:Carbamidomethylation:1000.00 | PEAKS DB |
| total 1 peptides |
| --- |

P14418|PA2A\_GLOHA

back to list

  

| Protein Coverage
| Supporting Peptides
|

Protein Coverage:

Supporting Peptides:

| Peptide | Uniq | -10lgP | Mass | Length | ppm | m/z | z | RT | Fraction | Scan | Source File | Area F1 | Area F10 | Area F2 | Area F5 | Area F7A | Area F7B | Area F8A | Area F8B | Area F9 | #Feature | #Feature F1 | #Feature F10 | #Feature F2 | #Feature F5 | #Feature F7A | #Feature F7B | #Feature F8A | #Feature F8B | #Feature F9 | Start | End | PTM | AScore | Found By |
| --- | --- | --- | --- | --- | --- | --- | --- | --- | --- | --- | --- | --- | --- | --- | --- | --- | --- | --- | --- | --- | --- | --- | --- | --- | --- | --- | --- | --- | --- | --- | --- | --- | --- | --- | --- |
| Y.C(+57.02)GWGGQGRPQDATDR.C | Y | 39.48 | 1659.7172 | 15 | 1.4 | 830.8608 | 2 | 26.80 | 8 | F8:8064 | DaRuWB\_F8B.raw |  |  |  |  |  |  |  | 3.5116E5 |  | 1 | 0 | 0 | 0 | 0 | 0 | 0 | 0 | 1 | 0 | 28 | 42 | Carbamidomethylation | C1:Carbamidomethylation:1000.00 | PEAKS DB |
| total 1 peptides |
| --- |

O42191|PA2A7\_GLOHA

back to list

  

| Protein Coverage
| Supporting Peptides
|

Protein Coverage:

Supporting Peptides:

| Peptide | Uniq | -10lgP | Mass | Length | ppm | m/z | z | RT | Fraction | Scan | Source File | Area F1 | Area F10 | Area F2 | Area F5 | Area F7A | Area F7B | Area F8A | Area F8B | Area F9 | #Feature | #Feature F1 | #Feature F10 | #Feature F2 | #Feature F5 | #Feature F7A | #Feature F7B | #Feature F8A | #Feature F8B | #Feature F9 | Start | End | PTM | AScore | Found By |
| --- | --- | --- | --- | --- | --- | --- | --- | --- | --- | --- | --- | --- | --- | --- | --- | --- | --- | --- | --- | --- | --- | --- | --- | --- | --- | --- | --- | --- | --- | --- | --- | --- | --- | --- | --- |
| Y.C(+57.02)GWGGQGRPQDATDR.C | Y | 39.48 | 1659.7172 | 15 | 1.4 | 830.8608 | 2 | 26.80 | 8 | F8:8064 | DaRuWB\_F8B.raw |  |  |  |  |  |  |  | 3.5116E5 |  | 1 | 0 | 0 | 0 | 0 | 0 | 0 | 0 | 1 | 0 | 28 | 42 | Carbamidomethylation | C1:Carbamidomethylation:1000.00 | PEAKS DB |
| total 1 peptides |
| --- |

P0DL42|TXVE\_DABSI

back to list

  

| Protein Coverage
| Supporting Peptides
|

Protein Coverage:

Supporting Peptides:

| Peptide | Uniq | -10lgP | Mass | Length | ppm | m/z | z | RT | Fraction | Scan | Source File | Area F1 | Area F10 | Area F2 | Area F5 | Area F7A | Area F7B | Area F8A | Area F8B | Area F9 | #Feature | #Feature F1 | #Feature F10 | #Feature F2 | #Feature F5 | #Feature F7A | #Feature F7B | #Feature F8A | #Feature F8B | #Feature F9 | Start | End | PTM | AScore | Found By |
| --- | --- | --- | --- | --- | --- | --- | --- | --- | --- | --- | --- | --- | --- | --- | --- | --- | --- | --- | --- | --- | --- | --- | --- | --- | --- | --- | --- | --- | --- | --- | --- | --- | --- | --- | --- |
| K.QGEPEGPKEPR | Y | 39.27 | 1222.5941 | 11 | -0.9 | 408.5383 | 3 | 12.69 | 4 | F4:1918 | DaRuWB\_F5.raw |  |  |  | 1.4239E7 |  |  |  |  |  | 1 | 0 | 0 | 0 | 1 | 0 | 0 | 0 | 0 | 0 | 99 | 109 |  |  | PEAKS DB |
| total 1 peptides |
| --- |

P67861|TXVE\_DABRR

back to list

  

| Protein Coverage
| Supporting Peptides
|

Protein Coverage:

Supporting Peptides:

| Peptide | Uniq | -10lgP | Mass | Length | ppm | m/z | z | RT | Fraction | Scan | Source File | Area F1 | Area F10 | Area F2 | Area F5 | Area F7A | Area F7B | Area F8A | Area F8B | Area F9 | #Feature | #Feature F1 | #Feature F10 | #Feature F2 | #Feature F5 | #Feature F7A | #Feature F7B | #Feature F8A | #Feature F8B | #Feature F9 | Start | End | PTM | AScore | Found By |
| --- | --- | --- | --- | --- | --- | --- | --- | --- | --- | --- | --- | --- | --- | --- | --- | --- | --- | --- | --- | --- | --- | --- | --- | --- | --- | --- | --- | --- | --- | --- | --- | --- | --- | --- | --- |
| K.QGEPEGPKEPR.R | Y | 39.27 | 1222.5941 | 11 | -0.9 | 408.5383 | 3 | 12.69 | 4 | F4:1918 | DaRuWB\_F5.raw |  |  |  | 1.4239E7 |  |  |  |  |  | 1 | 0 | 0 | 0 | 1 | 0 | 0 | 0 | 0 | 0 | 123 | 133 |  |  | PEAKS DB |
| total 1 peptides |
| --- |

Peptide List

  
  

---

Prepared with PEAKS ™ (bioinfor.com)
