# Supplementary material for: Biogeographic venom variation in Russell’s viper (Daboia russelii) and the preclinical inefficacy of antivenom therapy in snakebite hotspots
Source: PLoS Negl Trop Dis. 2021 Mar 25;15(3):e0009247. doi: 10.1371/journal.pntd.0009247 (PMC7993602; doi:10.1371/journal.pntd.0009247)
Supplement: S1 Data — (ZIP) [file pntd.0009247.s013.zip › D. russelii_Madhya Pradesh/D. russelii_Madhya Pradesh.html]

proteins


Summary

  

# 1. Notes

# 2. Result Statistics

**Figure 1.**
False discovery rate (FDR) curve. X axis is the number of peptide-spectrum matches (PSM) being kept. Y axis is the corresponding FDR.

  


  

**Figure 2.**
PSM score distribution. **(a)**
Distribution of PEAKS peptide score; **(b)**
Scatterplot of PEAKS peptide score versus precursor mass error.

|  |  |  |  |
| --- | --- | --- | --- |
| **(a)**  |  | | **(b)**  |  | |

|  |  |  |  |  |  |  |  |  |  |  |  |  |  |  |  |  |  |  |  |  |  |  |  |  |  |  |  |  |  |  |  |  |  |  |  |  |  |  |  |  |  |  |  |  |  |  |  |  |  |  |  |  |  |  |  |  |  |  |  |  |  |  |  |  |  |  |  |  |  |  |  |  |  |  |  |  |  |  |  |  |  |  |  |  |  |  |  |  |  |  |  |  |  |  |  |  |  |  |  |  |  |  |  |  |  |  |  |  |  |  |  |  |  |  |  |  |  |  |  |  |  |  |  |  |  |  |  |  |  |  |  |  |  |  |  |  |  |  |  |  |  |  |  |  |  |  |  |  |  |  |  |  |
| --- | --- | --- | --- | --- | --- | --- | --- | --- | --- | --- | --- | --- | --- | --- | --- | --- | --- | --- | --- | --- | --- | --- | --- | --- | --- | --- | --- | --- | --- | --- | --- | --- | --- | --- | --- | --- | --- | --- | --- | --- | --- | --- | --- | --- | --- | --- | --- | --- | --- | --- | --- | --- | --- | --- | --- | --- | --- | --- | --- | --- | --- | --- | --- | --- | --- | --- | --- | --- | --- | --- | --- | --- | --- | --- | --- | --- | --- | --- | --- | --- | --- | --- | --- | --- | --- | --- | --- | --- | --- | --- | --- | --- | --- | --- | --- | --- | --- | --- | --- | --- | --- | --- | --- | --- | --- | --- | --- | --- | --- | --- | --- | --- | --- | --- | --- | --- | --- | --- | --- | --- | --- | --- | --- | --- | --- | --- | --- | --- | --- | --- | --- | --- | --- | --- | --- | --- | --- | --- | --- | --- | --- | --- | --- | --- | --- | --- | --- | --- | --- | --- | --- | --- |
| **Table 1.** Statistics of data.    |  |  |  |  |  |  |  |  |  |  |  |  | | --- | --- | --- | --- | --- | --- | --- | --- | --- | --- | --- | --- | |  | #Scans | | #Features | Identified | | | #Peptides | #Sequences | #Proteins\* | | | | MS1 | MS2 | #PSMs | #Scans | #Features | Groups | All | Top | | Total | 162539 | 154581 | 461993 | 1769 | 1748 | 20191 | 613 | 582 | 77 | 275 | 135 | | F1 | 16944 | 5007 | 18585 | 78 | 78 | 905 | 74 | 73 | 25 | 60 | 32 | | F10 | 16715 | 8998 | 29667 | 135 | 135 | 1198 | 94 | 92 | 17 | 51 | 23 | | F2\_3 | 16958 | 4992 | 17537 | 78 | 78 | 840 | 72 | 72 | 30 | 67 | 35 | | F5 | 16530 | 13927 | 43520 | 143 | 143 | 1691 | 95 | 94 | 24 | 116 | 28 | | F6 | 16303 | 16006 | 44309 | 216 | 212 | 2442 | 157 | 152 | 37 | 114 | 40 | | F7A | 15719 | 23213 | 70649 | 193 | 190 | 2742 | 97 | 92 | 27 | 35 | 30 | | F7B | 15204 | 29445 | 81101 | 311 | 309 | 3802 | 218 | 210 | 48 | 84 | 58 | | F8A | 16091 | 21747 | 56568 | 325 | 315 | 2936 | 148 | 137 | 28 | 67 | 31 | | F8B | 15443 | 21489 | 66085 | 175 | 174 | 2558 | 134 | 133 | 41 | 120 | 80 | | F9 | 16632 | 9757 | 33972 | 115 | 114 | 1077 | 100 | 96 | 31 | 80 | 38 |  \* proteins with significant peptides are used in counts. |

**Figure 3.**
Sample overlap for Proteins and Peptides (up to 8 samples). **(a)**
All Proteins; **(b)**
Top Proteins; **(c)**
Peptides;

|  |  |  |  |  |  |
| --- | --- | --- | --- | --- | --- |
| **(a)**  | **Do not support more than 8 samples** | | **(b)**  | **Do not support more than 8 samples** | | **(c)**  | **Do not support more than 8 samples** | |

**Figure 4.**
Distribution of peptide feature detection. **(a)**
Feature m/z distribution; **(b)**
Feature RT distribution.

|  |  |  |  |
| --- | --- | --- | --- |
| **(a)**  |  | | **(b)**  |  | |

**Figure 5.**
Distribution of identified peptide features. **(a)**
Feature abundance distribution; **(b)**
*De novo*
sequencing validation.

|  |  |  |  |
| --- | --- | --- | --- |
| **(a)**  |  | | **(b)**  |  | |

|  |  |  |  |  |  |  |  |  |  |  |  |  |  |  |  |  |  |  |  |  |  |  |  |  |  |  |  |  |  |  |  |  |  |  |  |  |  |  |  |  |
| --- | --- | --- | --- | --- | --- | --- | --- | --- | --- | --- | --- | --- | --- | --- | --- | --- | --- | --- | --- | --- | --- | --- | --- | --- | --- | --- | --- | --- | --- | --- | --- | --- | --- | --- | --- | --- | --- | --- | --- | --- |
| **Table 2.** Result filtration parameters.  | Peptide -10lgP | ≥52 | | PTM Ascore | ≥0 | | Protein -10lgP | ≥20 | | Proteins unique peptides | ≥1 | | De novo score(%) | ≥50% |    **Table 3.** Statistics of filtered result.  | FDR (Peptide-Spectrum Matches) | 0.1% | | FDR (Peptide Sequences) | 0.2% | | FDR (Protein Group) | 1.3% | | De Novo Only Spectra | 24584 | | **Table 4.** PTM profile.  | Name | ∆Mass | Position | #PSM | -10lgP | Abundance | AScore || Carbamidomethyl | 57.02 | C | 910 | 113.76 | 1.7E6 | 1000.00 | | Oxidation | 15.99 | M | 150 | 96.55 | 1.34E7 | 26.02 | |

# 3. Experiment Control

**Figure 6.**
Precursor mass error of peptide-spectrum matches (PSM) in filtered result. **(a)**
Distribution of precursor mass error in ppm; **(b)**
Scatterplot of precursor m/z versus precursor mass error in ppm.

|  |  |  |  |
| --- | --- | --- | --- |
| **(a)**  |  | | **(b)**  |  | |

**Table 5.**
Number of identified peptides in each sample by the number of missed cleavages.

|  |  |  |  |  |  |  |  |  |  |  |  |  |  |  |  |  |  |  |  |  |  |  |  |  |  |  |  |  |  |  |  |  |  |  |  |  |  |  |  |  |  |  |  |  |  |  |  |  |  |  |  |  |  |  |  |  |  |  |  |  |  |  |  |  |  |  |
| --- | --- | --- | --- | --- | --- | --- | --- | --- | --- | --- | --- | --- | --- | --- | --- | --- | --- | --- | --- | --- | --- | --- | --- | --- | --- | --- | --- | --- | --- | --- | --- | --- | --- | --- | --- | --- | --- | --- | --- | --- | --- | --- | --- | --- | --- | --- | --- | --- | --- | --- | --- | --- | --- | --- | --- | --- | --- | --- | --- | --- | --- | --- | --- | --- | --- | --- |
| |  |  |  |  |  |  | | --- | --- | --- | --- | --- | --- | | Missed Cleavages | 0 | 1 | 2 | 3 | 4+ | | F1 | 70 | 4 | 0 | 0 | 0 | | F10 | 85 | 9 | 0 | 0 | 0 | | F2\_3 | 69 | 3 | 0 | 0 | 0 | | F5 | 88 | 6 | 1 | 0 | 0 | | F6 | 153 | 4 | 0 | 0 | 0 | | F7A | 69 | 21 | 7 | 0 | 0 | | F7B | 208 | 10 | 0 | 0 | 0 | | F8A | 131 | 15 | 2 | 0 | 0 | | F8B | 129 | 4 | 1 | 0 | 0 | | F9 | 95 | 4 | 1 | 0 | 0 | |

# 4. Other Information

|  |  |  |  |
| --- | --- | --- | --- |
| **Table 6.** Search parameters.  | Search Engine Name: PEAKS Parent Mass Error Tolerance: 10.0 ppm Fragment Mass Error Tolerance: 0.6 Da Precursor Mass Search Type: monoisotopic Enzyme: Trypsin Max Missed Cleavages: 2 Digest Mode: Semispecific Fixed Modifications:    Carbamidomethylation: 57.02 Variable Modifications:    Oxidation (M): 15.99 Max Variable PTM Per Peptide: 3 Database: SwissProt Taxon: All Contaminant Database: cRAP\_contaminants Searched Entry: 560234 FDR Estimation: Enabled Merge Options: no merge Precursor Options: corrected Charge Options: no correction Filter Charge: 2 - 8 Process: true Associate chimera: yes | | **Table 7.** Instrument parameters.  | Fractions: DaRuMP\_F1.raw, DaRuMP\_F10.raw, DaRuMP\_F2\_F3.raw, DaR uMP\_F5.raw, DaRuMP\_F6.raw, DaRuMP\_F7A.raw, DaRuMP\_F7B.raw, Da RuMP\_F8A.raw, DaRuMP\_F8B.raw, DaRuMP\_F9.raw Ion Source: ESI(nano-spray) Fragmentation Mode: CID, CAD(y and b ions) MS Scan Mode: FT-ICR/Orbitrap MS/MS Scan Mode: FT-ICR/Orbitrap | |

  

Protein List

  

|  |
| --- |
| Protein Accession Contains: |
| Protein Description Contains: |
| Protein Sample Area >= |
| Protein PTM Contains: |

| Protein Group | Protein ID | Accession | -10lgP | Coverage (%) | Coverage (%) F1 | Coverage (%) F10 | Coverage (%) F2\_3 | Coverage (%) F5 | Coverage (%) F6 | Coverage (%) F7A | Coverage (%) F7B | Coverage (%) F8A | Coverage (%) F8B | Coverage (%) F9 | Area F1 | Area F10 | Area F2\_3 | Area F5 | Area F6 | Area F7A | Area F7B | Area F8A | Area F8B | Area F9 | #Peptides | #Unique | #Spec F1 | #Spec F10 | #Spec F2\_3 | #Spec F5 | #Spec F6 | #Spec F7A | #Spec F7B | #Spec F8A | #Spec F8B | #Spec F9 | PTM | Avg. Mass | Description |
| --- | --- | --- | --- | --- | --- | --- | --- | --- | --- | --- | --- | --- | --- | --- | --- | --- | --- | --- | --- | --- | --- | --- | --- | --- | --- | --- | --- | --- | --- | --- | --- | --- | --- | --- | --- | --- | --- | --- | --- |
| 1 | 50 | P86368|PA2B3\_DABRR | 411.84 | 87 | 36 | 0 | 7 | 0 | 45 | 69 | 45 | 76 | 13 | 61 |  |  |  |  |  | 3.8436E6 |  |  |  |  | 40 | 1 | 4 | 0 | 1 | 0 | 7 | 55 | 9 | 140 | 1 | 18 | Y | 13687 | Basic phospholipase A2 3 OS=Daboia russelii OX=8707 PE=1 SV=1 |
| 3 | 48 | P59071|PA2B8\_DABRR | 405.81 | 96 | 24 | 0 | 7 | 0 | 46 | 96 | 46 | 47 | 0 | 33 |  |  |  |  |  | 1.5602E6 |  |  |  |  | 40 | 1 | 2 | 0 | 1 | 0 | 7 | 114 | 11 | 15 | 0 | 6 | Y | 13611 | Basic phospholipase A2 VRV-PL-VIIIa OS=Daboia russelii OX=8707 PE=1 SV=1 |
| 11 | 1 | B8K1W0|VM3DK\_DABRR | 384.35 | 53 | 2 | 48 | 6 | 0 | 0 | 0 | 0 | 0 | 0 | 0 |  | 4.8924E8 | 2.9045E5 |  |  |  |  |  |  |  | 43 | 39 | 1 | 75 | 5 | 0 | 0 | 0 | 0 | 0 | 0 | 0 | Y | 69555 | Zinc metalloproteinase-disintegrin-like daborhagin-K OS=Daboia russelii OX=8707 PE=1 SV=1 |
| 2 | 76 | P84674|PA2B5\_DABRR | 378.15 | 63 | 21 | 0 | 7 | 0 | 30 | 42 | 30 | 59 | 13 | 46 |  |  |  |  |  |  |  | 9.2593E6 |  |  | 29 | 2 | 3 | 0 | 1 | 0 | 6 | 36 | 7 | 135 | 1 | 15 | Y | 13587 | Basic phospholipase A2 VRV-PL-V OS=Daboia russelii OX=8707 PE=1 SV=1 |
| 6 | 92 | A8CG86|PA2A1\_DABRR | 375.82 | 81 | 0 | 0 | 0 | 11 | 8 | 9 | 81 | 23 | 59 | 11 |  |  |  |  |  | 7.5329E6 | 6.5479E8 | 7.6226E4 | 4.2724E6 |  | 32 | 15 | 0 | 0 | 0 | 1 | 2 | 5 | 88 | 2 | 10 | 1 | Y | 15329 | Acidic phospholipase A2 Drk-a1 OS=Daboia russelii OX=8707 PE=1 SV=1 |
| 12 | 2 | G8XQX1|OXLA\_DABRR | 364.11 | 52 | 0 | 12 | 2 | 10 | 7 | 0 | 6 | 46 | 6 | 35 |  | 2.4366E5 |  |  |  |  | 5.6955E4 | 2.256E7 | 1.6233E5 | 1.3237E6 | 31 | 6 | 0 | 6 | 1 | 6 | 3 | 0 | 3 | 48 | 2 | 21 | Y | 56888 | L-amino-acid oxidase OS=Daboia russelii OX=8707 PE=1 SV=1 |
| 5 | 3 | P04264|K2C1\_HUMAN | 342.97 | 39 | 20 | 24 | 17 | 30 | 32 | 2 | 36 | 18 | 31 | 22 | 4.8406E6 | 2.8271E6 | 1.0989E6 | 1.1875E7 | 1.0959E7 | 1.3906E5 | 2.8475E7 | 8.1473E6 | 5.86E6 | 1.54E6 | 23 | 16 | 10 | 13 | 8 | 20 | 18 | 1 | 23 | 12 | 20 | 13 | Y | 66039 | Keratin, type II cytoskeletal 1 OS=Homo sapiens OX=9606 GN=KRT1 PE=1 SV=6 |
| 14 | 112 | P31100|PA2A7\_DABSI | 320.83 | 77 | 0 | 0 | 0 | 11 | 0 | 0 | 77 | 11 | 24 | 20 |  |  |  |  |  |  | 7.4865E7 |  |  | 4.5122E4 | 20 | 5 | 0 | 0 | 0 | 1 | 0 | 0 | 54 | 1 | 4 | 2 | Y | 15421 | Acidic phospholipase A2 RV-7 OS=Daboia siamensis OX=343250 PE=1 SV=1 |
| 8 | 6 | P35527|K1C9\_HUMAN | 319.78 | 60 | 17 | 12 | 9 | 26 | 31 | 0 | 45 | 22 | 34 | 26 | 3.2027E6 | 5.6878E5 | 8.6792E5 | 6.3275E6 | 8.3397E6 |  | 2.5074E7 | 1.9062E6 | 5.823E6 | 7.4124E5 | 27 | 27 | 9 | 6 | 5 | 15 | 12 | 0 | 21 | 9 | 13 | 10 | Y | 62064 | Keratin, type I cytoskeletal 9 OS=Homo sapiens OX=9606 GN=KRT9 PE=1 SV=3 |
| 9 | 175 | F8QN54|PA2B\_VIPRE | 304.10 | 38 | 13 | 0 | 0 | 0 | 13 | 17 | 19 | 34 | 0 | 24 |  |  |  |  |  |  |  | 2.0711E7 |  |  | 14 | 1 | 1 | 0 | 0 | 0 | 1 | 12 | 3 | 56 | 0 | 7 | Y | 15636 | Basic phospholipase A2 vurtoxin OS=Vipera renardi OX=927686 PE=1 SV=1 |
| 7 | 8 | P13645|K1C10\_HUMAN | 300.29 | 39 | 16 | 18 | 15 | 23 | 21 | 3 | 31 | 18 | 13 | 16 | 2.0549E6 | 1.4904E6 | 5.8475E5 | 5.746E6 | 4.1487E6 | 4.981E4 | 1.2583E7 | 4.4776E6 | 1.131E6 | 1.104E6 | 19 | 15 | 8 | 9 | 9 | 12 | 10 | 1 | 16 | 9 | 7 | 9 | Y | 58827 | Keratin, type I cytoskeletal 10 OS=Homo sapiens OX=9606 GN=KRT10 PE=1 SV=6 |
| 24 | 116 | Q38L02|SLA\_DABSI | 295.45 | 51 | 0 | 13 | 0 | 0 | 13 | 34 | 51 | 29 | 49 | 8 |  | 2.4866E5 |  |  | 4.7464E6 | 8.3424E6 | 6.2894E7 | 1.0838E7 | 8.1309E7 | 9.9643E4 | 14 | 14 | 0 | 3 | 0 | 0 | 3 | 4 | 11 | 6 | 20 | 2 | Y | 17507 | Snaclec dabocetin subunit alpha OS=Daboia siamensis OX=343250 PE=1 SV=1 |
| 45 | 12 | Q7LZ61|VM3CX\_DABSI | 293.37 | 28 | 0 | 5 | 2 | 0 | 0 | 0 | 2 | 2 | 26 | 5 |  |  |  |  |  |  |  |  | 1.0038E7 | 5.3482E4 | 18 | 11 | 0 | 4 | 2 | 0 | 0 | 0 | 1 | 1 | 18 | 3 | Y | 69648 | Coagulation factor X-activating enzyme heavy chain OS=Daboia siamensis OX=343250 PE=1 SV=2 |
| 10 | 77 | A8CG87|PA2A2\_DABRR | 293.14 | 57 | 0 | 0 | 7 | 8 | 57 | 12 | 7 | 0 | 0 | 7 |  |  |  |  | 5.8601E8 |  |  |  |  |  | 18 | 5 | 0 | 0 | 1 | 1 | 52 | 2 | 1 | 0 | 0 | 1 | Y | 15586 | Acidic phospholipase A2 Drk-a2 OS=Daboia russelii OX=8707 PE=2 SV=1 |
| 10 | 78 | A8CG78|PA2A2\_DABSI | 293.14 | 57 | 0 | 0 | 7 | 8 | 57 | 12 | 7 | 0 | 0 | 7 |  |  |  |  | 5.8601E8 |  |  |  |  |  | 18 | 5 | 0 | 0 | 1 | 1 | 52 | 2 | 1 | 0 | 0 | 1 | Y | 15586 | Acidic phospholipase A2 DsM-a2/DsM-a2' OS=Daboia siamensis OX=343250 PE=1 SV=1 |
| 42 | 90 | A8CG82|PA2B1\_DABSI | 288.77 | 51 | 0 | 0 | 0 | 0 | 23 | 51 | 31 | 0 | 11 | 0 |  |  |  |  |  | 3.9936E5 |  |  |  |  | 10 | 1 | 0 | 0 | 0 | 0 | 2 | 14 | 3 | 0 | 1 | 0 | Y | 15843 | Basic phospholipase A2 DsM-b1/DsM-b1' OS=Daboia siamensis OX=343250 PE=1 SV=1 |
| 33 | 61 | P18965|VSPG\_DABSI | 278.58 | 47 | 0 | 0 | 0 | 0 | 31 | 44 | 25 | 3 | 13 | 0 |  |  |  |  |  | 3.0131E6 |  |  |  |  | 11 | 1 | 0 | 0 | 0 | 0 | 11 | 12 | 7 | 1 | 4 | 0 | Y | 28823 | Factor V activator RVV-V gamma OS=Daboia siamensis OX=343250 PE=1 SV=2 |
| 27 | 22 | P18964|VSPA\_DABSI | 277.99 | 75 | 0 | 0 | 0 | 0 | 59 | 72 | 27 | 3 | 31 | 0 |  |  |  |  | 1.0793E7 | 1.1198E7 |  |  | 0 |  | 17 | 7 | 0 | 0 | 0 | 0 | 16 | 16 | 7 | 1 | 5 | 0 | Y | 26182 | Factor V activator RVV-V alpha OS=Daboia siamensis OX=343250 PE=1 SV=1 |
| 47 | 14 | B6EWW8|V5NTD\_GLOBR | 273.51 | 23 | 0 | 5 | 0 | 2 | 0 | 0 | 0 | 21 | 0 | 5 |  | 8.3028E4 |  | 9.019E4 |  |  |  | 2.8233E7 |  | 3.7534E5 | 16 | 14 | 0 | 2 | 0 | 1 | 0 | 0 | 0 | 16 | 0 | 2 | Y | 64434 | Snake venom 5'-nucleotidase OS=Gloydius brevicaudus OX=259325 PE=2 SV=1 |
| 13 | 11 | P35908|K22E\_HUMAN | 271.78 | 32 | 18 | 13 | 12 | 10 | 19 | 2 | 18 | 13 | 18 | 20 | 3.9451E5 | 3.2736E5 | 7.4708E4 | 1.1643E4 | 1.7792E6 |  | 4.0526E6 | 1.4551E6 | 1.5159E6 | 7.0884E5 | 16 | 9 | 10 | 7 | 6 | 6 | 15 | 1 | 11 | 6 | 9 | 12 | Y | 65433 | Keratin, type II cytoskeletal 2 epidermal OS=Homo sapiens OX=9606 GN=KRT2 PE=1 SV=2 |
| 74 | 39 | E5L0E3|VSPAF\_DABSI | 262.80 | 40 | 0 | 0 | 0 | 0 | 3 | 3 | 40 | 0 | 3 | 0 |  |  |  |  |  |  | 1.2095E8 |  |  |  | 10 | 9 | 0 | 0 | 0 | 0 | 1 | 1 | 13 | 0 | 1 | 0 | Y | 28496 | Alpha-fibrinogenase-like OS=Daboia siamensis OX=343250 PE=2 SV=1 |
| 15 | 145 | P0DL42|TXVE\_DABSI | 262.00 | 60 | 0 | 0 | 0 | 48 | 49 | 17 | 0 | 0 | 0 | 0 |  |  |  | 1.2314E9 | 8.7473E7 | 1.0005E5 |  |  |  |  | 14 | 13 | 0 | 0 | 0 | 45 | 8 | 1 | 0 | 0 | 0 | 0 | Y | 12554 | Snake venom vascular endothelial growth factor toxin VR-1' OS=Daboia siamensis OX=343250 PE=1 SV=1 |
| 15 | 146 | P67861|TXVE\_DABRR | 262.00 | 45 | 0 | 0 | 0 | 36 | 37 | 13 | 0 | 0 | 0 | 0 |  |  |  | 1.2314E9 | 8.7473E7 | 1.0005E5 |  |  |  |  | 14 | 13 | 0 | 0 | 0 | 45 | 8 | 1 | 0 | 0 | 0 | 0 | Y | 16278 | Snake venom vascular endothelial growth factor toxin VR-1 OS=Daboia russelii OX=8707 PE=1 SV=2 |
| 30 | 128 | A8CG90|PA2B2\_DABRR | 252.73 | 42 | 0 | 0 | 7 | 23 | 31 | 21 | 30 | 13 | 0 | 7 |  |  |  | 9.9018E5 | 9.6837E7 | 4.6393E7 | 7.1449E6 | 1.8353E5 |  |  | 10 | 5 | 0 | 0 | 1 | 2 | 11 | 5 | 6 | 1 | 0 | 1 | Y | 15461 | Basic phospholipase A2 Drk-b2 OS=Daboia russelii OX=8707 PE=1 SV=1 |
| 57 | 79 | A0A1I9KNP0|VSPH1\_VIPAA | 249.61 | 31 | 0 | 0 | 0 | 0 | 27 | 27 | 22 | 0 | 14 | 0 |  |  |  |  |  | 4.3597E5 |  |  |  |  | 6 | 1 | 0 | 0 | 0 | 0 | 7 | 8 | 6 | 0 | 3 | 0 | Y | 28928 | Vaa serine proteinase homolog 1 OS=Vipera ammodytes ammodytes OX=8705 PE=1 SV=1 |
| 66 | 121 | P0DPS3|VASP1\_VIPAA | 248.93 | 24 | 0 | 0 | 0 | 0 | 24 | 19 | 18 | 0 | 8 | 0 |  |  |  |  |  | 5.823E5 |  |  |  |  | 6 | 1 | 0 | 0 | 0 | 0 | 5 | 7 | 6 | 0 | 1 | 0 | Y | 22639 | Snake venom serine protease VaSP1 (Fragments) OS=Vipera ammodytes ammodytes OX=8705 PE=1 SV=1 |
| 36 | 100 | P30894|NGFV\_DABRR | 240.93 | 46 | 0 | 0 | 0 | 46 | 36 | 0 | 9 | 0 | 0 | 0 |  |  |  | 1.4058E7 | 5.1605E6 |  | 1.9163E5 |  |  |  | 11 | 4 | 0 | 0 | 0 | 19 | 6 | 0 | 1 | 0 | 0 | 0 | Y | 13283 | Venom nerve growth factor OS=Daboia russelii OX=8707 PE=1 SV=1 |
| 58 | 315 | Q2ES47|VKT4\_DABRR | 239.56 | 48 | 48 | 0 | 48 | 32 | 0 | 0 | 0 | 0 | 0 | 0 | 2.6051E7 |  | 5.6005E7 | 2.1399E6 |  |  |  |  |  |  | 10 | 5 | 9 | 0 | 14 | 2 | 0 | 0 | 0 | 0 | 0 | 0 | Y | 9145 | Kunitz-type serine protease inhibitor 4 OS=Daboia russelii OX=8707 PE=2 SV=1 |
| 16 | 56 | Q4PRC6|SL7\_DABSI | 237.09 | 56 | 0 | 11 | 0 | 0 | 0 | 6 | 41 | 29 | 46 | 11 |  | 1.82E5 |  |  |  | 1.1977E5 | 2.5538E7 | 9.0796E6 | 3.9929E7 | 1.2039E5 | 10 | 8 | 0 | 2 | 0 | 0 | 0 | 1 | 13 | 8 | 23 | 2 | Y | 18067 | Snaclec 7 OS=Daboia siamensis OX=343250 PE=2 SV=1 |
| 102 | 24 | O43790|KRT86\_HUMAN | 234.39 | 18 | 0 | 0 | 16 | 0 | 0 | 0 | 0 | 0 | 2 | 0 |  |  | 3.3761E5 |  |  |  |  |  |  |  | 8 | 4 | 0 | 0 | 7 | 0 | 0 | 0 | 0 | 0 | 1 | 0 | Y | 53501 | Keratin, type II cuticular Hb6 OS=Homo sapiens OX=9606 GN=KRT86 PE=1 SV=1 |
| 35 | 17 | P08779|K1C16\_HUMAN | 221.59 | 30 | 8 | 0 | 5 | 4 | 12 | 0 | 4 | 0 | 2 | 18 | 4.1435E4 |  | 5.9728E4 |  | 1.7923E5 |  |  |  |  | 3.2701E4 | 12 | 5 | 4 | 0 | 2 | 2 | 4 | 0 | 2 | 0 | 1 | 7 | Y | 51268 | Keratin, type I cytoskeletal 16 OS=Homo sapiens OX=9606 GN=KRT16 PE=1 SV=4 |
| 29 | 19 | P02533|K1C14\_HUMAN | 218.04 | 33 | 11 | 0 | 2 | 12 | 4 | 0 | 4 | 0 | 8 | 15 | 0 |  |  | 1.1503E5 | 1.1637E5 |  |  |  | 1.5217E5 |  | 12 | 4 | 4 | 0 | 1 | 5 | 2 | 0 | 2 | 0 | 3 | 6 | Y | 51562 | Keratin, type I cytoskeletal 14 OS=Homo sapiens OX=9606 GN=KRT14 PE=1 SV=4 |
| 70 | 117 | Q4PRD1|SLLC1\_DABSI | 213.18 | 50 | 0 | 0 | 0 | 0 | 0 | 12 | 50 | 23 | 32 | 0 |  |  |  |  |  | 3.242E5 | 7.5372E7 | 2.5215E6 | 7.9221E6 |  | 6 | 1 | 0 | 0 | 0 | 0 | 0 | 1 | 12 | 3 | 4 | 0 | Y | 16871 | Snaclec coagulation factor X-activating enzyme light chain 1 OS=Daboia siamensis OX=343250 GN=LC1 PE=1 SV=2 |
| 60 | 104 | E0Y418|VSP1\_MACLB | 207.81 | 23 | 0 | 0 | 0 | 0 | 3 | 3 | 23 | 16 | 17 | 0 |  |  |  |  |  |  | 4.0476E8 | 1.5465E6 | 3.2973E7 |  | 9 | 8 | 0 | 0 | 0 | 0 | 1 | 1 | 11 | 2 | 7 | 0 | Y | 28702 | Serine protease VLSP-1 OS=Macrovipera lebetina OX=8709 PE=2 SV=1 |
| 83 | 197 | P25428|NGFV\_MACLB | 205.95 | 18 | 6 | 0 | 0 | 12 | 9 | 0 | 0 | 0 | 0 | 0 | 2.682E5 |  |  |  |  |  |  |  |  |  | 9 | 2 | 2 | 0 | 0 | 9 | 3 | 0 | 0 | 0 | 0 | 0 | Y | 27318 | Venom nerve growth factor OS=Macrovipera lebetina OX=8709 PE=1 SV=2 |
| 17 | 157 | P81458|PA2B\_DABRR | 202.35 | 43 | 0 | 0 | 7 | 0 | 33 | 26 | 36 | 11 | 9 | 7 |  |  |  |  | 2.7352E6 |  |  |  |  |  | 7 | 1 | 0 | 0 | 1 | 0 | 7 | 7 | 19 | 1 | 2 | 1 | Y | 13626 | Basic phospholipase A2 RVV-VD OS=Daboia russelii OX=8707 PE=1 SV=1 |
| 81 | 164 | B4XSZ0|SLAF\_MACLB | 201.48 | 24 | 0 | 10 | 0 | 0 | 0 | 0 | 10 | 24 | 10 | 10 |  | 1.2983E6 |  |  |  |  | 1.2734E6 | 1.2533E8 | 9.8789E5 | 2.1342E6 | 5 | 5 | 0 | 1 | 0 | 0 | 0 | 0 | 1 | 10 | 1 | 2 | Y | 17759 | Snaclec A15 OS=Macrovipera lebetina OX=8709 PE=2 SV=1 |
| 81 | 165 | B4XSY9|SLAE\_MACLB | 201.48 | 24 | 0 | 10 | 0 | 0 | 0 | 0 | 10 | 24 | 10 | 10 |  | 1.2983E6 |  |  |  |  | 1.2734E6 | 1.2533E8 | 9.8789E5 | 2.1342E6 | 5 | 5 | 0 | 1 | 0 | 0 | 0 | 0 | 1 | 10 | 1 | 2 | Y | 17711 | Snaclec A14 OS=Macrovipera lebetina OX=8709 PE=2 SV=1 |
| 81 | 213 | W5XCJ6|SLCIB\_MACLB | 201.48 | 25 | 0 | 10 | 0 | 0 | 0 | 0 | 10 | 25 | 10 | 10 |  | 1.2983E6 |  |  |  |  | 1.2734E6 | 1.2533E8 | 9.8789E5 | 2.1342E6 | 5 | 5 | 0 | 1 | 0 | 0 | 0 | 0 | 1 | 10 | 1 | 2 | Y | 17553 | Snaclec lebecin subunit beta OS=Macrovipera lebetina OX=8709 PE=1 SV=1 |
| 107 | 303 | Q2ES50|VKT1\_DABRR | 201.08 | 50 | 30 | 0 | 20 | 20 | 0 | 0 | 0 | 0 | 0 | 23 |  |  | 6.0872E4 | 2.9768E5 |  |  |  |  |  |  | 6 | 1 | 4 | 0 | 2 | 1 | 0 | 0 | 0 | 0 | 0 | 2 | Y | 9287 | Kunitz-type serine protease inhibitor 1 OS=Daboia russelii OX=8707 PE=2 SV=1 |
| 107 | 304 | A8Y7P0|VKTB7\_DABSI | 201.08 | 50 | 30 | 0 | 20 | 20 | 0 | 0 | 0 | 0 | 0 | 23 |  |  | 6.0872E4 | 2.9768E5 |  |  |  |  |  |  | 6 | 1 | 4 | 0 | 2 | 1 | 0 | 0 | 0 | 0 | 0 | 2 | Y | 9287 | Kunitz-type serine protease inhibitor 7 OS=Daboia siamensis OX=343250 PE=2 SV=1 |
| 107 | 310 | P00990|VKT2\_DABSI | 201.08 | 70 | 42 | 0 | 28 | 28 | 0 | 0 | 0 | 0 | 0 | 32 |  |  | 6.0872E4 | 2.9768E5 |  |  |  |  |  |  | 6 | 1 | 4 | 0 | 2 | 1 | 0 | 0 | 0 | 0 | 0 | 2 | Y | 6850 | Kunitz-type serine protease inhibitor 2 OS=Daboia siamensis OX=343250 PE=1 SV=1 |
| 28 | 23 | P13647|K2C5\_HUMAN | 198.89 | 19 | 2 | 5 | 0 | 5 | 6 | 2 | 11 | 2 | 8 | 7 | 1.0191E5 |  |  | 8.911E4 | 9.2326E4 |  | 4.8722E4 |  |  |  | 10 | 3 | 1 | 2 | 0 | 4 | 6 | 1 | 7 | 1 | 5 | 4 | Y | 62378 | Keratin, type II cytoskeletal 5 OS=Homo sapiens OX=9606 GN=KRT5 PE=1 SV=3 |
| 105 | 114 | P15445|PA2A2\_NAJNA | 197.00 | 47 | 0 | 0 | 0 | 34 | 47 | 0 | 0 | 0 | 0 | 0 |  |  |  | 1.2123E6 | 2.1498E6 |  |  |  |  |  | 4 | 4 | 0 | 0 | 0 | 3 | 4 | 0 | 0 | 0 | 0 | 0 | Y | 13346 | Acidic phospholipase A2 2 OS=Naja naja OX=35670 PE=1 SV=1 |
| 103 | 41 | A5A6M5|K1H1\_PANTR | 191.91 | 15 | 0 | 0 | 15 | 0 | 0 | 0 | 0 | 0 | 0 | 0 |  |  | 8.3082E3 |  |  |  |  |  |  |  | 6 | 1 | 0 | 0 | 6 | 0 | 0 | 0 | 0 | 0 | 0 | 0 | Y | 47247 | Keratin, type I cuticular Ha1 OS=Pan troglodytes OX=9598 GN=KRT31 PE=2 SV=1 |
| 103 | 42 | Q15323|K1H1\_HUMAN | 191.91 | 15 | 0 | 0 | 15 | 0 | 0 | 0 | 0 | 0 | 0 | 0 |  |  | 8.3082E3 |  |  |  |  |  |  |  | 6 | 1 | 0 | 0 | 6 | 0 | 0 | 0 | 0 | 0 | 0 | 0 | Y | 47237 | Keratin, type I cuticular Ha1 OS=Homo sapiens OX=9606 GN=KRT31 PE=1 SV=3 |
| 114 | 118 | Q8JFG1|PA2H\_VIPAP | 190.64 | 50 | 0 | 0 | 0 | 0 | 0 | 0 | 50 | 0 | 0 | 0 |  |  |  |  |  |  | 0 |  |  |  | 7 | 1 | 0 | 0 | 0 | 0 | 0 | 0 | 8 | 0 | 0 | 0 | Y | 15411 | Acidic phospholipase A2 inhibitor vaspin A chain OS=Vipera aspis aspis OX=194601 PE=2 SV=1 |
| 114 | 119 | Q10754|PA2H\_VIPAZ | 190.64 | 50 | 0 | 0 | 0 | 0 | 0 | 0 | 50 | 0 | 0 | 0 |  |  |  |  |  |  | 0 |  |  |  | 7 | 1 | 0 | 0 | 0 | 0 | 0 | 0 | 8 | 0 | 0 | 0 | Y | 15411 | Acidic phospholipase A2 inhibitor vaspin A chain OS=Vipera aspis zinnikeri OX=55427 PE=1 SV=2 |
| 114 | 120 | A4VBF0|PA2H\_VIPBN | 190.64 | 50 | 0 | 0 | 0 | 0 | 0 | 0 | 50 | 0 | 0 | 0 |  |  |  |  |  |  | 0 |  |  |  | 7 | 1 | 0 | 0 | 0 | 0 | 0 | 0 | 8 | 0 | 0 | 0 | Y | 15381 | Acidic phospholipase A2 inhibitor chain HPD-1I OS=Vipera berus nikolskii OX=1808362 PE=1 SV=1 |
| 114 | 139 | P04084|PA2A\_VIPAE | 190.64 | 57 | 0 | 0 | 0 | 0 | 0 | 0 | 57 | 0 | 0 | 0 |  |  |  |  |  |  | 0 |  |  |  | 7 | 1 | 0 | 0 | 0 | 0 | 0 | 0 | 8 | 0 | 0 | 0 | Y | 13639 | Acidic phospholipase A2 homolog vipoxin A chain OS=Vipera ammodytes meridionalis OX=73841 PE=1 SV=3 |
| 93 | 34 | Q14525|KT33B\_HUMAN | 187.55 | 19 | 0 | 0 | 14 | 0 | 0 | 0 | 0 | 0 | 5 | 0 |  |  | 4.2338E4 |  |  |  |  |  |  |  | 6 | 1 | 0 | 0 | 5 | 0 | 0 | 0 | 0 | 0 | 1 | 0 | Y | 46214 | Keratin, type I cuticular Ha3-II OS=Homo sapiens OX=9606 GN=KRT33B PE=1 SV=3 |
| 92 | 195 | B7FDI0|CRVP\_VIPBN | 186.50 | 17 | 0 | 0 | 0 | 0 | 12 | 17 | 4 | 0 | 0 | 0 |  |  |  |  | 2.4806E5 | 8.5913E5 |  |  |  |  | 5 | 2 | 0 | 0 | 0 | 0 | 6 | 3 | 1 | 0 | 0 | 0 | Y | 24612 | Cysteine-rich venom protein (Fragment) OS=Vipera berus nikolskii OX=1808362 PE=2 SV=1 |
| 92 | 208 | B7FDI1|CRVP\_VIPBE | 186.50 | 16 | 0 | 0 | 0 | 0 | 11 | 16 | 3 | 0 | 0 | 0 |  |  |  |  | 2.4806E5 | 8.5913E5 |  |  |  |  | 5 | 2 | 0 | 0 | 0 | 0 | 6 | 3 | 1 | 0 | 0 | 0 | Y | 26509 | Cysteine-rich venom protein OS=Vipera berus OX=31155 PE=1 SV=1 |
| 109 | 30 | P78386|KRT85\_HUMAN | 183.31 | 15 | 0 | 0 | 13 | 0 | 0 | 0 | 0 | 0 | 2 | 0 |  |  | 4.6219E4 |  |  |  |  |  |  |  | 7 | 2 | 0 | 0 | 6 | 0 | 0 | 0 | 0 | 0 | 1 | 0 | Y | 55802 | Keratin, type II cuticular Hb5 OS=Homo sapiens OX=9606 GN=KRT85 PE=1 SV=1 |
| 73 | 96 | E5L0E4|VSPB\_DABSI | 181.93 | 19 | 0 | 0 | 0 | 0 | 12 | 14 | 19 | 0 | 10 | 0 |  |  |  |  | 4.684E5 | 2.1116E7 | 1.7399E7 |  |  |  | 5 | 2 | 0 | 0 | 0 | 0 | 4 | 5 | 6 | 0 | 3 | 0 | Y | 28035 | Beta-fibrinogenase-like OS=Daboia siamensis OX=343250 PE=2 SV=1 |
| 121 | 316 | H6VC06|VKTK2\_DABRR | 177.41 | 50 | 50 | 0 | 0 | 0 | 0 | 0 | 0 | 0 | 0 | 23 | 4.0457E5 |  |  |  |  |  |  |  |  |  | 5 | 1 | 5 | 0 | 0 | 0 | 0 | 0 | 0 | 0 | 0 | 2 | Y | 9385 | Kunitz-type serine protease inhibitor DrKIn-II OS=Daboia russelii OX=8707 PE=1 SV=1 |
| 121 | 317 | A8Y7P4|VKTB4\_DABSI | 177.41 | 50 | 50 | 0 | 0 | 0 | 0 | 0 | 0 | 0 | 0 | 23 | 4.0457E5 |  |  |  |  |  |  |  |  |  | 5 | 1 | 5 | 0 | 0 | 0 | 0 | 0 | 0 | 0 | 0 | 2 | Y | 9371 | Kunitz-type serine protease inhibitor B4 OS=Daboia siamensis OX=343250 PE=2 SV=1 |
| 120 | 318 | A8Y7N8|VKTC5\_DABSI | 172.91 | 43 | 43 | 0 | 0 | 0 | 0 | 0 | 0 | 0 | 0 | 11 | 9.291E5 |  |  |  |  |  |  |  |  |  | 4 | 1 | 4 | 0 | 0 | 0 | 0 | 0 | 0 | 0 | 0 | 1 | Y | 10006 | Kunitz-type serine protease inhibitor C5 OS=Daboia siamensis OX=343250 PE=2 SV=1 |
| 135 | 179 | Q4PRD2|SLLC2\_DABSI | 172.63 | 20 | 0 | 0 | 0 | 0 | 0 | 0 | 11 | 0 | 20 | 0 |  |  |  |  |  |  | 5.6986E5 |  | 7.6767E5 |  | 3 | 3 | 0 | 0 | 0 | 0 | 0 | 0 | 1 | 0 | 3 | 0 | Y | 18337 | Snaclec coagulation factor X-activating enzyme light chain 2 OS=Daboia siamensis OX=343250 GN=LC2 PE=1 SV=1 |
| 142 | 256 | Q4VM07|VM3VB\_MACLB | 169.04 | 4 | 0 | 2 | 2 | 0 | 0 | 0 | 0 | 0 | 0 | 0 |  | 8.1974E6 |  |  |  |  |  |  |  |  | 3 | 1 | 0 | 2 | 2 | 0 | 0 | 0 | 0 | 0 | 0 | 0 | N | 68843 | Zinc metalloproteinase-disintegrin-like VLAIP-B OS=Macrovipera lebetina OX=8709 PE=1 SV=1 |
| 136 | 63 | O76011|KRT34\_HUMAN | 158.96 | 11 | 0 | 0 | 11 | 0 | 0 | 0 | 0 | 0 | 0 | 0 |  |  | 5.7452E4 |  |  |  |  |  |  |  | 4 | 1 | 0 | 0 | 4 | 0 | 0 | 0 | 0 | 0 | 0 | 0 | Y | 49424 | Keratin, type I cuticular Ha4 OS=Homo sapiens OX=9606 GN=KRT34 PE=1 SV=2 |
| 139 | 832 | P24541|VKT\_ERIMA | 151.79 | 27 | 27 | 0 | 27 | 24 | 0 | 0 | 0 | 0 | 0 | 0 | 1.1365E5 |  | 3.3823E5 |  |  |  |  |  |  |  | 3 | 1 | 2 | 0 | 4 | 1 | 0 | 0 | 0 | 0 | 0 | 0 | Y | 6772 | Kunitz-type serine protease inhibitor OS=Eristicophis macmahoni OX=110227 PE=1 SV=1 |
| 115 | 723 | A8Y7N9|VKTC6\_DABSI | 150.86 | 29 | 14 | 0 | 29 | 0 | 0 | 0 | 0 | 0 | 0 | 0 |  |  | 1.2178E4 |  |  |  |  |  |  |  | 5 | 1 | 3 | 0 | 4 | 0 | 0 | 0 | 0 | 0 | 0 | 0 | Y | 10349 | Kunitz-type serine protease inhibitor C6 OS=Daboia siamensis OX=343250 PE=2 SV=1 |
| 95 | 206 | E0Y419|VSPBF\_MACLB | 149.80 | 19 | 0 | 0 | 0 | 0 | 19 | 8 | 19 | 0 | 13 | 0 |  |  |  |  | 1.3902E6 |  | 5.9583E5 |  |  |  | 4 | 1 | 0 | 0 | 0 | 0 | 5 | 2 | 4 | 0 | 4 | 0 | Y | 28297 | Beta-fibrinogenase OS=Macrovipera lebetina OX=8709 PE=1 SV=1 |
| 123 | 159 | B4XSY8|SLAD\_MACLB | 148.20 | 26 | 0 | 17 | 0 | 0 | 0 | 0 | 17 | 17 | 26 | 8 |  | 1.765E5 |  |  |  |  | 6.3527E6 | 2.8558E6 | 6.4297E6 | 2.1545E4 | 3 | 3 | 0 | 2 | 0 | 0 | 0 | 0 | 2 | 2 | 3 | 1 | Y | 15308 | Snaclec A13 OS=Macrovipera lebetina OX=8709 PE=2 SV=1 |
| 123 | 160 | B4XSY7|SLAC\_MACLB | 148.20 | 22 | 0 | 14 | 0 | 0 | 0 | 0 | 14 | 14 | 22 | 6 |  | 1.765E5 |  |  |  |  | 6.3527E6 | 2.8558E6 | 6.4297E6 | 2.1545E4 | 3 | 3 | 0 | 2 | 0 | 0 | 0 | 0 | 2 | 2 | 3 | 1 | Y | 17717 | Snaclec A12 OS=Macrovipera lebetina OX=8709 PE=2 SV=1 |
| 91 | 185 | Q6IFZ6|K2C1B\_MOUSE | 141.19 | 6 | 2 | 2 | 2 | 2 | 2 | 0 | 2 | 4 | 4 | 4 |  |  |  |  |  |  |  | 6.0324E3 |  |  | 3 | 1 | 1 | 1 | 1 | 1 | 1 | 0 | 1 | 2 | 2 | 2 | N | 61359 | Keratin, type II cytoskeletal 1b OS=Mus musculus OX=10090 GN=Krt77 PE=1 SV=1 |
| 118 | 314 | P25669|3L22\_NAJNA | 138.25 | 46 | 46 | 0 | 32 | 32 | 14 | 0 | 14 | 14 | 0 | 14 | 1.1971E6 |  | 1.7044E5 | 4.235E4 |  |  |  |  |  |  | 3 | 2 | 3 | 0 | 2 | 2 | 1 | 0 | 1 | 1 | 0 | 1 | Y | 7821 | Long neurotoxin 2 OS=Naja naja OX=35670 PE=1 SV=1 |
| 116 | 276 | Q696W1|SLLC2\_MACLB | 137.86 | 20 | 0 | 0 | 0 | 0 | 0 | 0 | 20 | 6 | 20 | 6 |  |  |  |  |  |  | 2.6213E7 | 1.0407E6 | 7.1436E6 | 7.033E4 | 3 | 3 | 0 | 0 | 0 | 0 | 0 | 0 | 3 | 1 | 4 | 1 | Y | 18094 | Snaclec coagulation factor X-activating enzyme light chain 2 OS=Macrovipera lebetina OX=8709 GN=LC2 PE=1 SV=1 |
| 130 | 83 | P02845|VIT2\_CHICK | 133.96 | 1 | 0 | 0 | 0 | 0 | 1 | 0 | 0 | 0 | 0 | 0 |  |  |  |  | 7.0545E4 |  |  |  |  |  | 2 | 2 | 0 | 0 | 0 | 0 | 2 | 0 | 0 | 0 | 0 | 0 | Y | 204807 | Vitellogenin-2 OS=Gallus gallus OX=9031 GN=VTG2 PE=1 SV=1 |
| 110 | 322 | P01391|3L21\_NAJKA | 132.22 | 41 | 41 | 0 | 27 | 41 | 41 | 0 | 14 | 14 | 0 | 14 | 1.3021E6 |  | 1.8401E5 | 1.2306E5 | 1.0165E5 |  |  |  |  |  | 2 | 1 | 2 | 0 | 1 | 2 | 2 | 0 | 1 | 1 | 0 | 1 | Y | 7831 | Alpha-cobratoxin OS=Naja kaouthia OX=8649 PE=1 SV=1 |
| 140 | 297 | A8Y7P1|VKTB1\_DABSI | 128.96 | 31 | 31 | 0 | 0 | 0 | 0 | 0 | 0 | 0 | 0 | 0 | 4.1392E5 |  |  |  |  |  |  |  |  |  | 3 | 1 | 3 | 0 | 0 | 0 | 0 | 0 | 0 | 0 | 0 | 0 | Y | 9318 | Kunitz-type serine protease inhibitor B1 OS=Daboia siamensis OX=343250 PE=1 SV=1 |
| 140 | 298 | A8Y7P5|VKTB5\_DABSI | 128.96 | 29 | 29 | 0 | 0 | 0 | 0 | 0 | 0 | 0 | 0 | 0 | 4.1392E5 |  |  |  |  |  |  |  |  |  | 3 | 1 | 3 | 0 | 0 | 0 | 0 | 0 | 0 | 0 | 0 | 0 | Y | 9901 | Kunitz-type serine protease inhibitor B5 OS=Daboia siamensis OX=343250 PE=2 SV=1 |
| 159 | 1561 | P86530|VSP1\_DABRR | 126.96 | 100 | 0 | 0 | 0 | 0 | 0 | 0 | 100 | 0 | 0 | 0 |  |  |  |  |  |  | 2.2404E5 |  |  |  | 3 | 1 | 0 | 0 | 0 | 0 | 0 | 0 | 3 | 0 | 0 | 0 | Y | 1728 | Vipera russelli proteinase RVV-V homolog 1 (Fragment) OS=Daboia russelii OX=8707 PE=1 SV=1 |
| 141 | 333 | A8Y7N6|VKTC3\_DABSI | 120.63 | 32 | 32 | 0 | 20 | 0 | 0 | 0 | 0 | 0 | 0 | 0 | 1.8749E6 |  | 6.7275E4 |  |  |  |  |  |  |  | 3 | 1 | 4 | 0 | 1 | 0 | 0 | 0 | 0 | 0 | 0 | 0 | Y | 9443 | Kunitz-type serine protease inhibitor C3 OS=Daboia siamensis OX=343250 PE=2 SV=1 |
| 141 | 334 | A8Y7N7|VKTC4\_DABSI | 120.63 | 30 | 30 | 0 | 19 | 0 | 0 | 0 | 0 | 0 | 0 | 0 | 1.8749E6 |  | 6.7275E4 |  |  |  |  |  |  |  | 3 | 1 | 4 | 0 | 1 | 0 | 0 | 0 | 0 | 0 | 0 | 0 | Y | 10162 | Kunitz-type serine protease inhibitor C4 OS=Daboia siamensis OX=343250 PE=2 SV=1 |
| 89 | 143 | P00761|TRYP\_PIG | 119.65 | 4 | 0 | 0 | 0 | 0 | 0 | 0 | 0 | 0 | 4 | 4 |  |  |  |  |  |  |  |  | 1.0145E5 | 1.5066E5 | 1 | 1 | 0 | 0 | 0 | 0 | 0 | 0 | 0 | 0 | 1 | 1 | N | 24409 | Trypsin OS=Sus scrofa OX=9823 PE=1 SV=1 |
| 133 | 397 | E0Y420|VSP3\_MACLB | 117.23 | 11 | 0 | 0 | 0 | 0 | 11 | 3 | 11 | 0 | 8 | 0 |  |  |  |  | 1.0232E6 |  | 5.6989E5 |  |  |  | 3 | 1 | 0 | 0 | 0 | 0 | 3 | 1 | 3 | 0 | 2 | 0 | Y | 28352 | Serine protease VLSP-3 OS=Macrovipera lebetina OX=8709 PE=2 SV=1 |
| 144 | 202 | A7ISW1|QPCT\_BOIIR | 109.65 | 4 | 0 | 4 | 0 | 0 | 0 | 0 | 0 | 0 | 0 | 0 |  | 2.9141E5 |  |  |  |  |  |  |  |  | 1 | 1 | 0 | 2 | 0 | 0 | 0 | 0 | 0 | 0 | 0 | 0 | N | 41990 | Glutaminyl-peptide cyclotransferase OS=Boiga irregularis OX=92519 GN=QPCT PE=2 SV=1 |
| 144 | 203 | Q90YA8|QPCT\_GLOBL | 109.65 | 4 | 0 | 4 | 0 | 0 | 0 | 0 | 0 | 0 | 0 | 0 |  | 2.9141E5 |  |  |  |  |  |  |  |  | 1 | 1 | 0 | 2 | 0 | 0 | 0 | 0 | 0 | 0 | 0 | 0 | N | 42267 | Glutaminyl-peptide cyclotransferase OS=Gloydius blomhoffii OX=242054 GN=QPCT PE=2 SV=1 |
| 144 | 204 | A7ISW2|QPCT\_BOIDE | 109.65 | 4 | 0 | 4 | 0 | 0 | 0 | 0 | 0 | 0 | 0 | 0 |  | 2.9141E5 |  |  |  |  |  |  |  |  | 1 | 1 | 0 | 2 | 0 | 0 | 0 | 0 | 0 | 0 | 0 | 0 | N | 41990 | Glutaminyl-peptide cyclotransferase OS=Boiga dendrophila OX=46286 GN=QPCT PE=2 SV=1 |
| 144 | 212 | Q9YIB5|QPCT\_BOTJA | 109.65 | 4 | 0 | 4 | 0 | 0 | 0 | 0 | 0 | 0 | 0 | 0 |  | 2.9141E5 |  |  |  |  |  |  |  |  | 1 | 1 | 0 | 2 | 0 | 0 | 0 | 0 | 0 | 0 | 0 | 0 | N | 42204 | Glutaminyl-peptide cyclotransferase OS=Bothrops jararaca OX=8724 GN=QPCT PE=2 SV=1 |
| 149 | 587 | B4XSY5|SLAA\_MACLB | 104.73 | 10 | 0 | 0 | 0 | 0 | 0 | 0 | 0 | 0 | 10 | 0 |  |  |  |  |  |  |  |  | 2.2975E5 |  | 2 | 2 | 0 | 0 | 0 | 0 | 0 | 0 | 0 | 0 | 2 | 0 | Y | 17731 | Snaclec A10 OS=Macrovipera lebetina OX=8709 PE=2 SV=1 |
| 149 | 806 | B4XSY6|SLAB\_MACLB | 104.73 | 10 | 0 | 0 | 0 | 0 | 0 | 0 | 0 | 0 | 10 | 0 |  |  |  |  |  |  |  |  | 2.2975E5 |  | 2 | 2 | 0 | 0 | 0 | 0 | 0 | 0 | 0 | 0 | 2 | 0 | Y | 17738 | Snaclec A11 OS=Macrovipera lebetina OX=8709 PE=2 SV=1 |
| 113 | 326 | P81605|DCD\_HUMAN | 100.27 | 10 | 0 | 10 | 10 | 0 | 0 | 0 | 0 | 0 | 0 | 0 |  | 1.0373E4 | 2.099E4 |  |  |  |  |  |  |  | 1 | 1 | 0 | 1 | 1 | 0 | 0 | 0 | 0 | 0 | 0 | 0 | N | 11284 | Dermcidin OS=Homo sapiens OX=9606 GN=DCD PE=1 SV=2 |
| 155 | 228 | P10981|ACT5\_DROME | 99.51 | 8 | 0 | 0 | 0 | 0 | 0 | 0 | 0 | 0 | 8 | 0 |  |  |  |  |  |  |  |  | 2.4657E5 |  | 2 | 2 | 0 | 0 | 0 | 0 | 0 | 0 | 0 | 0 | 2 | 0 | N | 41802 | Actin-87E OS=Drosophila melanogaster OX=7227 GN=Act87E PE=1 SV=1 |
| 155 | 229 | P45886|ACT3\_BACDO | 99.51 | 8 | 0 | 0 | 0 | 0 | 0 | 0 | 0 | 0 | 8 | 0 |  |  |  |  |  |  |  |  | 2.4657E5 |  | 2 | 2 | 0 | 0 | 0 | 0 | 0 | 0 | 0 | 0 | 2 | 0 | N | 41816 | Actin-3, muscle-specific OS=Bactrocera dorsalis OX=27457 PE=2 SV=1 |
| 155 | 230 | P45885|ACT2\_BACDO | 99.51 | 8 | 0 | 0 | 0 | 0 | 0 | 0 | 0 | 0 | 8 | 0 |  |  |  |  |  |  |  |  | 2.4657E5 |  | 2 | 2 | 0 | 0 | 0 | 0 | 0 | 0 | 0 | 0 | 2 | 0 | N | 41803 | Actin-2, muscle-specific OS=Bactrocera dorsalis OX=27457 PE=2 SV=1 |
| 155 | 231 | P07836|ACT1\_BOMMO | 99.51 | 8 | 0 | 0 | 0 | 0 | 0 | 0 | 0 | 0 | 8 | 0 |  |  |  |  |  |  |  |  | 2.4657E5 |  | 2 | 2 | 0 | 0 | 0 | 0 | 0 | 0 | 0 | 0 | 2 | 0 | N | 41876 | Actin, muscle-type A1 OS=Bombyx mori OX=7091 PE=3 SV=1 |
| 155 | 232 | P02574|ACT4\_DROME | 99.51 | 8 | 0 | 0 | 0 | 0 | 0 | 0 | 0 | 0 | 8 | 0 |  |  |  |  |  |  |  |  | 2.4657E5 |  | 2 | 2 | 0 | 0 | 0 | 0 | 0 | 0 | 0 | 0 | 2 | 0 | N | 41787 | Actin, larval muscle OS=Drosophila melanogaster OX=7227 GN=Act79B PE=1 SV=2 |
| 155 | 233 | P45887|ACT5\_BACDO | 99.51 | 8 | 0 | 0 | 0 | 0 | 0 | 0 | 0 | 0 | 8 | 0 |  |  |  |  |  |  |  |  | 2.4657E5 |  | 2 | 2 | 0 | 0 | 0 | 0 | 0 | 0 | 0 | 0 | 2 | 0 | N | 41771 | Actin-5, muscle-specific OS=Bactrocera dorsalis OX=27457 PE=2 SV=1 |
| 155 | 237 | P49871|ACT\_MANSE | 99.51 | 8 | 0 | 0 | 0 | 0 | 0 | 0 | 0 | 0 | 8 | 0 |  |  |  |  |  |  |  |  | 2.4657E5 |  | 2 | 2 | 0 | 0 | 0 | 0 | 0 | 0 | 0 | 0 | 2 | 0 | N | 41777 | Actin, muscle OS=Manduca sexta OX=7130 PE=2 SV=1 |
| 127 | 897 | Q56EB1|SLAA\_BOTJA | 95.72 | 10 | 0 | 0 | 0 | 0 | 10 | 0 | 10 | 0 | 10 | 0 |  |  |  |  | 3.2841E5 |  | 6.7439E6 |  | 7.2964E6 |  | 2 | 2 | 0 | 0 | 0 | 0 | 1 | 0 | 4 | 0 | 4 | 0 | Y | 17577 | Snaclec bothrojaracin subunit alpha OS=Bothrops jararaca OX=8724 PE=1 SV=1 |
| 197 | 1298 | Q6T7B7|LEC1\_BITGA | 70.63 | 8 | 0 | 0 | 0 | 0 | 0 | 0 | 8 | 0 | 0 | 0 |  |  |  |  |  |  | 1.2984E6 |  |  |  | 1 | 1 | 0 | 0 | 0 | 0 | 0 | 0 | 1 | 0 | 0 | 0 | Y | 18626 | C-type lectin 1 OS=Bitis gabonica OX=8694 PE=2 SV=1 |
| 204 | 516 | Q6LAF3|H4\_FLATR | 66.51 | 10 | 0 | 0 | 0 | 0 | 0 | 0 | 0 | 0 | 10 | 0 |  |  |  |  |  |  |  |  | 6.5839E4 |  | 1 | 1 | 0 | 0 | 0 | 0 | 0 | 0 | 0 | 0 | 1 | 0 | N | 11409 | Histone H4 OS=Flaveria trinervia OX=4227 GN=hh4 PE=3 SV=3 |
| 204 | 517 | P62887|H4\_LOLTE | 66.51 | 10 | 0 | 0 | 0 | 0 | 0 | 0 | 0 | 0 | 10 | 0 |  |  |  |  |  |  |  |  | 6.5839E4 |  | 1 | 1 | 0 | 0 | 0 | 0 | 0 | 0 | 0 | 0 | 1 | 0 | N | 11409 | Histone H4 OS=Lolium temulentum OX=34176 PE=3 SV=2 |
| 204 | 518 | P59259|H4\_ARATH | 66.51 | 10 | 0 | 0 | 0 | 0 | 0 | 0 | 0 | 0 | 10 | 0 |  |  |  |  |  |  |  |  | 6.5839E4 |  | 1 | 1 | 0 | 0 | 0 | 0 | 0 | 0 | 0 | 0 | 1 | 0 | N | 11409 | Histone H4 OS=Arabidopsis thaliana OX=3702 GN=At1g07660 PE=1 SV=2 |
| 204 | 519 | Q6PMI5|H4\_CHEMJ | 66.51 | 10 | 0 | 0 | 0 | 0 | 0 | 0 | 0 | 0 | 10 | 0 |  |  |  |  |  |  |  |  | 6.5839E4 |  | 1 | 1 | 0 | 0 | 0 | 0 | 0 | 0 | 0 | 0 | 1 | 0 | N | 11409 | Histone H4 OS=Chelidonium majus OX=71251 PE=3 SV=3 |
| 204 | 520 | Q43083|H4\_PYRSA | 66.51 | 10 | 0 | 0 | 0 | 0 | 0 | 0 | 0 | 0 | 10 | 0 |  |  |  |  |  |  |  |  | 6.5839E4 |  | 1 | 1 | 0 | 0 | 0 | 0 | 0 | 0 | 0 | 0 | 1 | 0 | N | 11425 | Histone H4 OS=Pyrenomonas salina OX=3034 PE=3 SV=3 |
| 204 | 521 | Q6WZ83|H4\_EUCGL | 66.51 | 10 | 0 | 0 | 0 | 0 | 0 | 0 | 0 | 0 | 10 | 0 |  |  |  |  |  |  |  |  | 6.5839E4 |  | 1 | 1 | 0 | 0 | 0 | 0 | 0 | 0 | 0 | 0 | 1 | 0 | N | 11409 | Histone H4 OS=Eucalyptus globulus OX=34317 PE=3 SV=3 |
| 204 | 999 | P70081|H48\_CHICK | 66.51 | 10 | 0 | 0 | 0 | 0 | 0 | 0 | 0 | 0 | 10 | 0 |  |  |  |  |  |  |  |  | 6.5839E4 |  | 1 | 1 | 0 | 0 | 0 | 0 | 0 | 0 | 0 | 0 | 1 | 0 | N | 11439 | Histone H4 type VIII OS=Gallus gallus OX=9031 GN=H4-VIII PE=3 SV=3 |
| 204 | 1000 | P84049|H4\_MYRRU | 66.51 | 10 | 0 | 0 | 0 | 0 | 0 | 0 | 0 | 0 | 10 | 0 |  |  |  |  |  |  |  |  | 6.5839E4 |  | 1 | 1 | 0 | 0 | 0 | 0 | 0 | 0 | 0 | 0 | 1 | 0 | N | 11381 | Histone H4 OS=Myrmica ruginodis OX=34708 GN=His4 PE=3 SV=2 |
| 204 | 1001 | Q76FF1|H4\_DROOR | 66.51 | 10 | 0 | 0 | 0 | 0 | 0 | 0 | 0 | 0 | 10 | 0 |  |  |  |  |  |  |  |  | 6.5839E4 |  | 1 | 1 | 0 | 0 | 0 | 0 | 0 | 0 | 0 | 0 | 1 | 0 | N | 11381 | Histone H4 OS=Drosophila orena OX=7233 GN=His4 PE=3 SV=3 |
| 204 | 1002 | P62800|H4\_CAIMO | 66.51 | 10 | 0 | 0 | 0 | 0 | 0 | 0 | 0 | 0 | 10 | 0 |  |  |  |  |  |  |  |  | 6.5839E4 |  | 1 | 1 | 0 | 0 | 0 | 0 | 0 | 0 | 0 | 0 | 1 | 0 | N | 11367 | Histone H4 OS=Cairina moschata OX=8855 PE=3 SV=2 |
| 204 | 1003 | Q6WV73|H4\_MYTCA | 66.51 | 10 | 0 | 0 | 0 | 0 | 0 | 0 | 0 | 0 | 10 | 0 |  |  |  |  |  |  |  |  | 6.5839E4 |  | 1 | 1 | 0 | 0 | 0 | 0 | 0 | 0 | 0 | 0 | 1 | 0 | N | 11367 | Histone H4 OS=Mytilus californianus OX=6549 PE=3 SV=3 |
| 204 | 1004 | Q6WV72|H4\_MYTTR | 66.51 | 10 | 0 | 0 | 0 | 0 | 0 | 0 | 0 | 0 | 10 | 0 |  |  |  |  |  |  |  |  | 6.5839E4 |  | 1 | 1 | 0 | 0 | 0 | 0 | 0 | 0 | 0 | 0 | 1 | 0 | N | 11367 | Histone H4 OS=Mytilus trossulus OX=6551 PE=3 SV=3 |
| 204 | 1005 | P62779|H4\_PYCHE | 66.51 | 10 | 0 | 0 | 0 | 0 | 0 | 0 | 0 | 0 | 10 | 0 |  |  |  |  |  |  |  |  | 6.5839E4 |  | 1 | 1 | 0 | 0 | 0 | 0 | 0 | 0 | 0 | 0 | 1 | 0 | N | 11369 | Histone H4 OS=Pycnopodia helianthoides OX=7614 PE=3 SV=2 |
| 204 | 1006 | Q28DR4|H4\_XENTR | 66.51 | 10 | 0 | 0 | 0 | 0 | 0 | 0 | 0 | 0 | 10 | 0 |  |  |  |  |  |  |  |  | 6.5839E4 |  | 1 | 1 | 0 | 0 | 0 | 0 | 0 | 0 | 0 | 0 | 1 | 0 | N | 11367 | Histone H4 OS=Xenopus tropicalis OX=8364 GN=TGas006m08.1 PE=3 SV=1 |
| 204 | 1007 | P91882|H4\_DIAPU | 66.51 | 10 | 0 | 0 | 0 | 0 | 0 | 0 | 0 | 0 | 10 | 0 |  |  |  |  |  |  |  |  | 6.5839E4 |  | 1 | 1 | 0 | 0 | 0 | 0 | 0 | 0 | 0 | 0 | 1 | 0 | N | 11453 | Histone H4 OS=Diadromus pulchellus OX=7420 PE=3 SV=3 |
| 204 | 1008 | Q6WV74|H4\_MYTCH | 66.51 | 10 | 0 | 0 | 0 | 0 | 0 | 0 | 0 | 0 | 10 | 0 |  |  |  |  |  |  |  |  | 6.5839E4 |  | 1 | 1 | 0 | 0 | 0 | 0 | 0 | 0 | 0 | 0 | 1 | 0 | N | 11395 | Histone H4 OS=Mytilus chilensis OX=173667 PE=3 SV=3 |
| 204 | 1009 | Q6LAF1|H4\_DENKL | 66.51 | 10 | 0 | 0 | 0 | 0 | 0 | 0 | 0 | 0 | 10 | 0 |  |  |  |  |  |  |  |  | 6.5839E4 |  | 1 | 1 | 0 | 0 | 0 | 0 | 0 | 0 | 0 | 0 | 1 | 0 | N | 11381 | Histone H4 OS=Dendronephthya klunzingeri OX=84964 GN=H4DEKL PE=3 SV=3 |
| 204 | 1010 | P62784|H4\_CAEEL | 66.51 | 10 | 0 | 0 | 0 | 0 | 0 | 0 | 0 | 0 | 10 | 0 |  |  |  |  |  |  |  |  | 6.5839E4 |  | 1 | 1 | 0 | 0 | 0 | 0 | 0 | 0 | 0 | 0 | 1 | 0 | N | 11369 | Histone H4 OS=Caenorhabditis elegans OX=6239 GN=his-1 PE=1 SV=2 |
| 204 | 1011 | Q6WV90|H4\_MYTGA | 66.51 | 10 | 0 | 0 | 0 | 0 | 0 | 0 | 0 | 0 | 10 | 0 |  |  |  |  |  |  |  |  | 6.5839E4 |  | 1 | 1 | 0 | 0 | 0 | 0 | 0 | 0 | 0 | 0 | 1 | 0 | N | 11367 | Histone H4 OS=Mytilus galloprovincialis OX=29158 PE=3 SV=3 |
| 204 | 1012 | P62782|H4\_LYTPI | 66.51 | 10 | 0 | 0 | 0 | 0 | 0 | 0 | 0 | 0 | 10 | 0 |  |  |  |  |  |  |  |  | 6.5839E4 |  | 1 | 1 | 0 | 0 | 0 | 0 | 0 | 0 | 0 | 0 | 1 | 0 | N | 11369 | Histone H4 OS=Lytechinus pictus OX=7653 PE=3 SV=2 |
| 204 | 1013 | P84046|H4\_CHITH | 66.51 | 10 | 0 | 0 | 0 | 0 | 0 | 0 | 0 | 0 | 10 | 0 |  |  |  |  |  |  |  |  | 6.5839E4 |  | 1 | 1 | 0 | 0 | 0 | 0 | 0 | 0 | 0 | 0 | 1 | 0 | N | 11381 | Histone H4 OS=Chironomus thummi thummi OX=7155 GN=His4 PE=3 SV=2 |
| 204 | 1014 | P62803|H4\_BOVIN | 66.51 | 10 | 0 | 0 | 0 | 0 | 0 | 0 | 0 | 0 | 10 | 0 |  |  |  |  |  |  |  |  | 6.5839E4 |  | 1 | 1 | 0 | 0 | 0 | 0 | 0 | 0 | 0 | 0 | 1 | 0 | N | 11367 | Histone H4 OS=Bos taurus OX=9913 PE=1 SV=2 |
| 204 | 1015 | P62776|H4\_HOLTU | 66.51 | 10 | 0 | 0 | 0 | 0 | 0 | 0 | 0 | 0 | 10 | 0 |  |  |  |  |  |  |  |  | 6.5839E4 |  | 1 | 1 | 0 | 0 | 0 | 0 | 0 | 0 | 0 | 0 | 1 | 0 | N | 11369 | Histone H4 OS=Holothuria tubulosa OX=7685 PE=3 SV=2 |
| 204 | 1016 | Q71V09|H4\_CAPAN | 66.51 | 10 | 0 | 0 | 0 | 0 | 0 | 0 | 0 | 0 | 10 | 0 |  |  |  |  |  |  |  |  | 6.5839E4 |  | 1 | 1 | 0 | 0 | 0 | 0 | 0 | 0 | 0 | 0 | 1 | 0 | N | 11425 | Histone H4 OS=Capsicum annuum OX=4072 PE=3 SV=3 |
| 204 | 1017 | Q7KQD1|H4\_CHAVR | 66.51 | 10 | 0 | 0 | 0 | 0 | 0 | 0 | 0 | 0 | 10 | 0 |  |  |  |  |  |  |  |  | 6.5839E4 |  | 1 | 1 | 0 | 0 | 0 | 0 | 0 | 0 | 0 | 0 | 1 | 0 | N | 11367 | Histone H4 OS=Chaetopterus variopedatus OX=34590 PE=3 SV=3 |
| 204 | 1018 | P50566|H4\_CHLRE | 66.51 | 10 | 0 | 0 | 0 | 0 | 0 | 0 | 0 | 0 | 10 | 0 |  |  |  |  |  |  |  |  | 6.5839E4 |  | 1 | 1 | 0 | 0 | 0 | 0 | 0 | 0 | 0 | 0 | 1 | 0 | N | 11457 | Histone H4 OS=Chlamydomonas reinhardtii OX=3055 GN=H4-I PE=3 SV=2 |
| 204 | 1019 | Q76FD9|H4\_DROSE | 66.51 | 10 | 0 | 0 | 0 | 0 | 0 | 0 | 0 | 0 | 10 | 0 |  |  |  |  |  |  |  |  | 6.5839E4 |  | 1 | 1 | 0 | 0 | 0 | 0 | 0 | 0 | 0 | 0 | 1 | 0 | N | 11381 | Histone H4 OS=Drosophila sechellia OX=7238 GN=His4 PE=3 SV=3 |
| 204 | 1020 | P62805|H4\_HUMAN | 66.51 | 10 | 0 | 0 | 0 | 0 | 0 | 0 | 0 | 0 | 10 | 0 |  |  |  |  |  |  |  |  | 6.5839E4 |  | 1 | 1 | 0 | 0 | 0 | 0 | 0 | 0 | 0 | 0 | 1 | 0 | N | 11367 | Histone H4 OS=Homo sapiens OX=9606 GN=HIST1H4A PE=1 SV=2 |
| 204 | 1021 | P84044|H4\_DROYA | 66.51 | 10 | 0 | 0 | 0 | 0 | 0 | 0 | 0 | 0 | 10 | 0 |  |  |  |  |  |  |  |  | 6.5839E4 |  | 1 | 1 | 0 | 0 | 0 | 0 | 0 | 0 | 0 | 0 | 1 | 0 | N | 11381 | Histone H4 OS=Drosophila yakuba OX=7245 GN=His4 PE=3 SV=2 |
| 204 | 1022 | Q7K8C0|H4\_MYTED | 66.51 | 10 | 0 | 0 | 0 | 0 | 0 | 0 | 0 | 0 | 10 | 0 |  |  |  |  |  |  |  |  | 6.5839E4 |  | 1 | 1 | 0 | 0 | 0 | 0 | 0 | 0 | 0 | 0 | 1 | 0 | N | 11367 | Histone H4 OS=Mytilus edulis OX=6550 PE=3 SV=3 |
| 167 | 1348 | P0DJL4|SLA\_DABPA | 63.79 | 11 | 0 | 0 | 0 | 0 | 0 | 0 | 11 | 0 | 0 | 0 |  |  |  |  |  |  | 2.3103E5 |  |  |  | 1 | 1 | 0 | 0 | 0 | 0 | 0 | 0 | 1 | 0 | 0 | 0 | Y | 12125 | Snaclec VP12 subunit A (Fragments) OS=Daboia palaestinae OX=1170828 PE=1 SV=1 |
| 186 | 331 | P82993|AMYB\_HORVS | 62.40 | 2 | 0 | 0 | 0 | 0 | 0 | 0 | 2 | 0 | 0 | 0 |  |  |  |  |  |  | 1.7334E5 |  |  |  | 1 | 1 | 0 | 0 | 0 | 0 | 0 | 0 | 1 | 0 | 0 | 0 | N | 59639 | Beta-amylase OS=Hordeum vulgare subsp. spontaneum OX=77009 GN=BMY1 PE=1 SV=1 |
| 186 | 337 | P16098|AMYB\_HORVU | 62.40 | 2 | 0 | 0 | 0 | 0 | 0 | 0 | 2 | 0 | 0 | 0 |  |  |  |  |  |  | 1.7334E5 |  |  |  | 1 | 1 | 0 | 0 | 0 | 0 | 0 | 0 | 1 | 0 | 0 | 0 | N | 59647 | Beta-amylase OS=Hordeum vulgare OX=4513 GN=BMY1 PE=1 SV=1 |
| 186 | 789 | P30271|AMYB\_SECCE | 62.40 | 5 | 0 | 0 | 0 | 0 | 0 | 0 | 5 | 0 | 0 | 0 |  |  |  |  |  |  | 1.7334E5 |  |  |  | 1 | 1 | 0 | 0 | 0 | 0 | 0 | 0 | 1 | 0 | 0 | 0 | N | 24349 | Beta-amylase (Fragment) OS=Secale cereale OX=4550 GN=BMY1 PE=2 SV=1 |
| 214 | 837 | P17314|IAAC3\_WHEAT | 62.37 | 10 | 0 | 0 | 0 | 0 | 0 | 0 | 10 | 0 | 0 | 0 |  |  |  |  |  |  | 1.8949E5 |  |  |  | 1 | 1 | 0 | 0 | 0 | 0 | 0 | 0 | 1 | 0 | 0 | 0 | Y | 18221 | Alpha-amylase/trypsin inhibitor CM3 OS=Triticum aestivum OX=4565 PE=1 SV=1 |
| 171 | 381 | P05661|MYSA\_DROME | 60.97 | 1 | 0 | 0 | 0 | 0 | 0 | 0 | 0 | 0 | 1 | 0 |  |  |  |  |  |  |  |  | 4.0995E4 |  | 1 | 1 | 0 | 0 | 0 | 0 | 0 | 0 | 0 | 0 | 1 | 0 | N | 224463 | Myosin heavy chain, muscle OS=Drosophila melanogaster OX=7227 GN=Mhc PE=1 SV=4 |
| 210 | 1054 | P05109|S10A8\_HUMAN | 59.56 | 12 | 0 | 0 | 0 | 0 | 0 | 0 | 12 | 0 | 0 | 0 |  |  |  |  |  |  | 1.2697E5 |  |  |  | 1 | 1 | 0 | 0 | 0 | 0 | 0 | 0 | 1 | 0 | 0 | 0 | Y | 10835 | Protein S100-A8 OS=Homo sapiens OX=9606 GN=S100A8 PE=1 SV=1 |
| 169 | 1589 | P59276|3S1C\_NAJKA | 55.40 | 18 | 18 | 0 | 0 | 0 | 0 | 0 | 0 | 0 | 0 | 0 | 2.0483E5 |  |  |  |  |  |  |  |  |  | 1 | 1 | 1 | 0 | 0 | 0 | 0 | 0 | 0 | 0 | 0 | 0 | Y | 6859 | Cobrotoxin-c OS=Naja kaouthia OX=8649 PE=1 SV=1 |
| 169 | 1590 | P59275|3S1B\_NAJKA | 55.40 | 18 | 18 | 0 | 0 | 0 | 0 | 0 | 0 | 0 | 0 | 0 | 2.0483E5 |  |  |  |  |  |  |  |  |  | 1 | 1 | 1 | 0 | 0 | 0 | 0 | 0 | 0 | 0 | 0 | 0 | Y | 6944 | Cobrotoxin-b OS=Naja kaouthia OX=8649 PE=1 SV=1 |
| 169 | 1591 | P01427|3S11\_NAJOX | 55.40 | 18 | 18 | 0 | 0 | 0 | 0 | 0 | 0 | 0 | 0 | 0 | 2.0483E5 |  |  |  |  |  |  |  |  |  | 1 | 1 | 1 | 0 | 0 | 0 | 0 | 0 | 0 | 0 | 0 | 0 | Y | 6885 | Short neurotoxin 1 OS=Naja oxiana OX=8657 PE=1 SV=1 |
| 203 | 1292 | Q9NQ30|ESM1\_HUMAN | 53.96 | 5 | 0 | 0 | 0 | 5 | 0 | 0 | 0 | 0 | 0 | 0 |  |  |  | 3.5692E4 |  |  |  |  |  |  | 1 | 1 | 0 | 0 | 0 | 1 | 0 | 0 | 0 | 0 | 0 | 0 | Y | 20095 | Endothelial cell-specific molecule 1 OS=Homo sapiens OX=9606 GN=ESM1 PE=1 SV=2 |
| total 135 proteins |
| --- |

  

P86368|PA2B3\_DABRR

back to list

  

| Protein Coverage
| Supporting Peptides
|

Protein Coverage:

Supporting Peptides:

| Peptide | Uniq | -10lgP | Mass | Length | ppm | m/z | z | RT | Fraction | Scan | Source File | Area F1 | Area F10 | Area F2\_3 | Area F5 | Area F6 | Area F7A | Area F7B | Area F8A | Area F8B | Area F9 | #Feature | #Feature F1 | #Feature F10 | #Feature F2\_3 | #Feature F5 | #Feature F6 | #Feature F7A | #Feature F7B | #Feature F8A | #Feature F8B | #Feature F9 | Start | End | PTM | AScore | Found By |
| --- | --- | --- | --- | --- | --- | --- | --- | --- | --- | --- | --- | --- | --- | --- | --- | --- | --- | --- | --- | --- | --- | --- | --- | --- | --- | --- | --- | --- | --- | --- | --- | --- | --- | --- | --- | --- | --- |
| R.VNGAIVC(+57.02)EQGTSC(+57.02)ENR.I | N | 112.07 | 1792.7832 | 16 | -9.1 | 897.3907 | 2 | 15.49 | 8 | F8:2314 | DaRuMP\_F8A.raw |  |  |  |  | 6.4795E5 | 2.662E7 | 1.3092E6 | 4.1633E8 | 2.4228E4 | 1.7554E6 | 14 | 0 | 0 | 0 | 0 | 1 | 3 | 1 | 6 | 1 | 2 | 69 | 84 | Carbamidomethylation | C7:Carbamidomethylation:1000.00;C13:Carbamidomethylation:1000.00 | PEAKS DB |
| K.LAVPFYSSYGC(+57.02)YC(+57.02)GWGGK.A | N | 103.36 | 2070.8967 | 18 | -1.2 | 1036.4543 | 2 | 30.18 | 8 | F8:12713 | DaRuMP\_F8A.raw |  |  |  |  |  |  |  | 4.2764E8 |  |  | 3 | 0 | 0 | 0 | 0 | 0 | 0 | 0 | 3 | 0 | 0 | 16 | 33 | Carbamidomethylation | C11:Carbamidomethylation:1000.00;C13:Carbamidomethylation:1000.00 | PEAKS DB |
| R.C(+57.02)C(+57.02)FVHDC(+57.02)C(+57.02)YGNLPDC(+57.02)NPK.S | N | 103.24 | 2314.8687 | 18 | 1.4 | 1158.4404 | 2 | 11.58 | 6 | F6:2373 | DaRuMP\_F7A.raw | 2.8891E5 |  |  |  | 1.2509E6 | 4.8203E8 | 1.5976E7 | 2.1062E8 |  | 1.2674E5 | 9 | 1 | 0 | 0 | 0 | 1 | 2 | 2 | 2 | 0 | 1 | 43 | 60 | Carbamidomethylation | C1:Carbamidomethylation:1000.00;C2:Carbamidomethylation:1000.00;C7:Carbamidomethylation:1000.00;C8:Carbamidomethylation:1000.00;C15:Carbamidomethylation:1000.00 | PEAKS DB |
| R.IC(+57.02)EC(+57.02)DKAAAIC(+57.02)FR.R | N | 98.35 | 1612.7159 | 13 | 1.9 | 538.5790 | 3 | 11.50 | 6 | F6:2309 | DaRuMP\_F7A.raw |  |  |  |  |  | 7.839E6 |  |  |  |  | 2 | 0 | 0 | 0 | 0 | 0 | 2 | 0 | 0 | 0 | 0 | 85 | 97 | Carbamidomethylation | C2:Carbamidomethylation:1000.00;C4:Carbamidomethylation:1000.00;C11:Carbamidomethylation:1000.00 | PEAKS DB |
| SLLEFGM(+15.99)MILEETGK.L | N | 96.55 | 1712.8364 | 15 | 0.6 | 857.4260 | 2 | 53.14 | 8 | F8:23593 | DaRuMP\_F8A.raw |  |  |  |  |  |  |  | 2.8084E7 |  | 3.2977E5 | 5 | 0 | 0 | 0 | 0 | 0 | 0 | 0 | 4 | 0 | 1 | 1 | 15 | Oxidation (M) | M7:Oxidation (M):26.02 | PEAKS DB |
| Q.GTSC(+57.02)ENRIC(+57.02)EC(+57.02)DKAAAIC(+57.02)FR.R | N | 93.89 | 2417.0344 | 20 | -1.5 | 806.6823 | 3 | 11.44 | 6 | F6:2255 | DaRuMP\_F7A.raw |  |  |  |  |  | 5.1971E6 |  |  |  |  | 2 | 0 | 0 | 0 | 0 | 0 | 2 | 0 | 0 | 0 | 0 | 78 | 97 | Carbamidomethylation | C4:Carbamidomethylation:1000.00;C9:Carbamidomethylation:1000.00;C11:Carbamidomethylation:1000.00;C18:Carbamidomethylation:1000.00 | PEAKS DB |
| C.FVHDC(+57.02)C(+57.02)YGNLPDC(+57.02)NPK.S | N | 93.60 | 1994.8074 | 16 | 2.2 | 998.4108 | 2 | 11.39 | 6 | F6:2215 | DaRuMP\_F7A.raw |  |  |  |  |  | 2.6011E6 |  |  |  |  | 2 | 0 | 0 | 0 | 0 | 0 | 2 | 0 | 0 | 0 | 0 | 45 | 60 | Carbamidomethylation | C5:Carbamidomethylation:1000.00;C6:Carbamidomethylation:1000.00;C13:Carbamidomethylation:1000.00 | PEAKS DB |
| SLLEFGMM(+15.99)ILEETGK.L | N | 91.71 | 1712.8364 | 15 | 0.2 | 857.4257 | 2 | 60.23 | 8 | F8:26218 | DaRuMP\_F8A.raw |  |  |  |  |  |  |  | 1.5322E7 |  | 3.2977E5 | 4 | 0 | 0 | 0 | 0 | 0 | 0 | 0 | 3 | 0 | 1 | 1 | 15 | Oxidation (M) | M8:Oxidation (M):40.00 | PEAKS DB |
| K.RVNGAIVC(+57.02)EQGTSC(+57.02)ENR.I | N | 91.41 | 1948.8843 | 17 | -8.1 | 975.4415 | 2 | 15.49 | 8 | F8:2300 | DaRuMP\_F8A.raw |  |  |  |  |  | 5.318E5 |  | 1.1178E7 |  | 2.4029E5 | 4 | 0 | 0 | 0 | 0 | 0 | 2 | 0 | 1 | 0 | 1 | 68 | 84 | Carbamidomethylation | C8:Carbamidomethylation:1000.00;C14:Carbamidomethylation:1000.00 | PEAKS DB |
| SLLEFGMMILEETGK.L | N | 87.31 | 1696.8416 | 15 | 0.5 | 849.4285 | 2 | 66.01 | 8 | F8:27922 | DaRuMP\_F8A.raw |  |  |  |  |  |  |  | 2.2161E7 |  |  | 2 | 0 | 0 | 0 | 0 | 0 | 0 | 0 | 2 | 0 | 0 | 1 | 15 |  |  | PEAKS DB |
| SLLEFGM(+15.99)M(+15.99)ILEETGK.L | N | 86.23 | 1728.8314 | 15 | 0.1 | 865.4231 | 2 | 45.65 | 8 | F8:20926 | DaRuMP\_F8A.raw |  |  |  |  |  |  |  | 1.0085E7 |  | 7.4736E5 | 8 | 0 | 0 | 0 | 0 | 0 | 0 | 0 | 6 | 0 | 2 | 1 | 15 | Oxidation (M) | M7:Oxidation (M):1000.00;M8:Oxidation (M):1000.00 | PEAKS DB |
| R.VNGAIVC(+57.02)EQGTSC(+57.02)ENRIC(+57.02)EC(+57.02)DKAAAIC(+57.02)FR.R | Y | 85.96 | 3387.4885 | 29 | 0.0 | 1130.1674 | 3 | 11.77 | 6 | F6:2567 | DaRuMP\_F7A.raw |  |  |  |  |  | 3.8436E6 |  |  |  |  | 2 | 0 | 0 | 0 | 0 | 0 | 2 | 0 | 0 | 0 | 0 | 69 | 97 | Carbamidomethylation | C7:Carbamidomethylation:1000.00;C13:Carbamidomethylation:1000.00;C18:Carbamidomethylation:1000.00;C20:Carbamidomethylation:1000.00;C27:Carbamidomethylation:1000.00 | PEAKS DB |
| K.IYMLYPDFLC(+57.02)K.G | N | 81.94 | 1461.7036 | 11 | 0.9 | 731.8597 | 2 | 35.78 | 8 | F8:15918 | DaRuMP\_F8A.raw |  |  |  |  |  |  |  | 6.9733E8 |  | 1.5491E6 | 10 | 0 | 0 | 0 | 0 | 0 | 0 | 0 | 9 | 0 | 1 | 106 | 116 | Carbamidomethylation | C10:Carbamidomethylation:1000.00 | PEAKS DB |
| R.VNGAIVC(+57.02)EQGTSC(+57.02)ENRIC(+57.02)EC(+57.02)DK.A | N | 80.69 | 2598.0930 | 22 | 2.6 | 867.0385 | 3 | 11.30 | 6 | F6:2134 | DaRuMP\_F7A.raw |  |  |  |  |  | 2.3203E5 |  |  |  |  | 1 | 0 | 0 | 0 | 0 | 0 | 1 | 0 | 0 | 0 | 0 | 69 | 90 | Carbamidomethylation | C7:Carbamidomethylation:1000.00;C13:Carbamidomethylation:1000.00;C18:Carbamidomethylation:1000.00;C20:Carbamidomethylation:1000.00 | PEAKS DB |
| K.IYM(+15.99)LYPDFLC(+57.02)K.G | N | 79.86 | 1477.6985 | 11 | 0.6 | 739.8569 | 2 | 35.27 | 8 | F8:17625 | DaRuMP\_F8A.raw |  |  |  |  |  |  |  | 1.6878E8 |  | 1.8039E6 | 6 | 0 | 0 | 0 | 0 | 0 | 0 | 0 | 4 | 0 | 2 | 106 | 116 | Oxidation (M); Carbamidomethylation | M3:Oxidation (M):1000.00;C10:Carbamidomethylation:1000.00 | PEAKS DB |
| N.GAIVC(+57.02)EQGTSC(+57.02)ENR.I | N | 79.00 | 1579.6719 | 14 | -8.2 | 790.8367 | 2 | 15.49 | 8 | F8:2301 | DaRuMP\_F8A.raw |  |  |  |  | 1.7032E5 |  | 3.5504E5 | 1.6512E6 |  | 5.094E4 | 4 | 0 | 0 | 0 | 0 | 1 | 0 | 1 | 1 | 0 | 1 | 71 | 84 | Carbamidomethylation | C5:Carbamidomethylation:1000.00;C11:Carbamidomethylation:1000.00 | PEAKS DB |
| K.DATDRC(+57.02)C(+57.02)FVHDC(+57.02)C(+57.02)YGNLPDC(+57.02)NPK.S | N | 78.23 | 2873.1084 | 23 | 1.9 | 958.7096 | 3 | 11.58 | 6 | F6:2387 | DaRuMP\_F7A.raw |  |  |  |  |  | 1.375E6 |  |  |  |  | 1 | 0 | 0 | 0 | 0 | 0 | 1 | 0 | 0 | 0 | 0 | 38 | 60 | Carbamidomethylation | C6:Carbamidomethylation:1000.00;C7:Carbamidomethylation:1000.00;C12:Carbamidomethylation:1000.00;C13:Carbamidomethylation:1000.00;C20:Carbamidomethylation:1000.00 | PEAKS DB |
| F.VHDC(+57.02)C(+57.02)YGNLPDC(+57.02)NPK.S | N | 77.58 | 1847.7389 | 15 | 1.3 | 924.8757 | 2 | 11.13 | 6 | F6:1990 | DaRuMP\_F7A.raw |  |  |  |  |  | 4.0829E5 |  |  |  |  | 1 | 0 | 0 | 0 | 0 | 0 | 1 | 0 | 0 | 0 | 0 | 46 | 60 | Carbamidomethylation | C4:Carbamidomethylation:1000.00;C5:Carbamidomethylation:1000.00;C12:Carbamidomethylation:1000.00 | PEAKS DB |
| D.C(+57.02)C(+57.02)YGNLPDC(+57.02)NPK.S | N | 77.58 | 1496.5846 | 12 | 3.4 | 749.3003 | 2 | 11.37 | 6 | F6:2187 | DaRuMP\_F7A.raw |  |  |  |  |  | 1.8907E6 |  | 0 |  | 5.5068E4 | 2 | 0 | 0 | 0 | 0 | 0 | 1 | 0 | 0 | 0 | 1 | 49 | 60 | Carbamidomethylation | C1:Carbamidomethylation:1000.00;C2:Carbamidomethylation:1000.00;C9:Carbamidomethylation:1000.00 | PEAKS DB |
| I.YMLYPDFLC(+57.02)KGELK.C | N | 77.16 | 1775.8625 | 14 | 2.8 | 592.9617 | 3 | 21.57 | 6 | F6:10344 | DaRuMP\_F7A.raw |  |  |  |  |  | 3.328E6 |  |  |  |  | 2 | 0 | 0 | 0 | 0 | 0 | 2 | 0 | 0 | 0 | 0 | 107 | 120 | Carbamidomethylation | C9:Carbamidomethylation:1000.00 | PEAKS DB |
| K.IYMLYPDFLC(+57.02)KGELK.C | N | 76.28 | 1888.9467 | 15 | -0.4 | 630.6559 | 3 | 29.27 | 8 | F8:11875 | DaRuMP\_F8A.raw |  |  |  |  |  |  |  | 6.0575E6 |  |  | 1 | 0 | 0 | 0 | 0 | 0 | 0 | 0 | 1 | 0 | 0 | 106 | 120 | Carbamidomethylation | C10:Carbamidomethylation:1000.00 | PEAKS DB |
| R.VNGAIVC(+57.02)EQGTSC(+57.02)ENRI.C | N | 75.51 | 1905.8673 | 17 | -5.2 | 953.9360 | 2 | 15.56 | 8 | F8:2361 | DaRuMP\_F8A.raw |  |  |  |  |  |  |  | 1.5204E5 |  |  | 1 | 0 | 0 | 0 | 0 | 0 | 0 | 0 | 1 | 0 | 0 | 69 | 85 | Carbamidomethylation | C7:Carbamidomethylation:1000.00;C13:Carbamidomethylation:1000.00 | PEAKS DB |
| C.C(+57.02)YGNLPDC(+57.02)NPK.S | N | 75.32 | 1336.5540 | 11 | 3.2 | 669.2848 | 2 | 11.29 | 6 | F6:2118 | DaRuMP\_F7A.raw |  |  |  |  |  | 3.3405E5 |  |  |  |  | 1 | 0 | 0 | 0 | 0 | 0 | 1 | 0 | 0 | 0 | 0 | 50 | 60 | Carbamidomethylation | C1:Carbamidomethylation:1000.00;C8:Carbamidomethylation:1000.00 | PEAKS DB |
| I.YM(+15.99)LYPDFLC(+57.02)K.G | N | 73.83 | 1364.6145 | 10 | 2.7 | 683.3147 | 2 | 24.65 | 6 | F6:13883 | DaRuMP\_F7A.raw |  |  |  |  | 5.5321E5 | 5.3341E7 |  |  |  |  | 5 | 0 | 0 | 0 | 0 | 1 | 4 | 0 | 0 | 0 | 0 | 107 | 116 | Oxidation (M); Carbamidomethylation | M2:Oxidation (M):1000.00;C9:Carbamidomethylation:1000.00 | PEAKS DB |
| G.AIVC(+57.02)EQGTSC(+57.02)ENR.I | N | 73.19 | 1522.6504 | 13 | 0.4 | 762.3328 | 2 | 10.89 | 1 | F1:1784 | DaRuMP\_F1.raw | 1.1385E6 |  |  |  | 4.2735E4 |  | 6.204E4 |  |  |  | 3 | 1 | 0 | 0 | 0 | 1 | 0 | 1 | 0 | 0 | 0 | 72 | 84 | Carbamidomethylation | C4:Carbamidomethylation:1000.00;C10:Carbamidomethylation:1000.00 | PEAKS DB |
| Y.SSYGC(+57.02)YC(+57.02)GWGGK.A | N | 71.73 | 1380.5227 | 12 | 2.9 | 691.2690 | 2 | 11.64 | 6 | F6:2416 | DaRuMP\_F7A.raw |  |  |  |  |  | 7.7516E6 | 7.2278E5 | 2.165E6 |  |  | 3 | 0 | 0 | 0 | 0 | 0 | 1 | 1 | 1 | 0 | 0 | 22 | 33 | Carbamidomethylation | C5:Carbamidomethylation:1000.00;C7:Carbamidomethylation:1000.00 | PEAKS DB |
| C.YGNLPDC(+57.02)NPK.S | N | 71.12 | 1176.5233 | 10 | 2.2 | 589.2688 | 2 | 11.15 | 6 | F6:1993 | DaRuMP\_F7A.raw |  |  |  |  |  | 3.1081E5 |  |  |  |  | 1 | 0 | 0 | 0 | 0 | 0 | 1 | 0 | 0 | 0 | 0 | 51 | 60 | Carbamidomethylation | C7:Carbamidomethylation:1000.00 | PEAKS DB |
| S.SYGC(+57.02)YC(+57.02)GWGGK.A | N | 71.11 | 1293.4907 | 11 | 2.9 | 647.7529 | 2 | 11.61 | 6 | F6:2413 | DaRuMP\_F7A.raw | 3.6812E5 |  |  |  |  | 1.7045E6 | 5.493E5 | 4.5706E5 |  |  | 4 | 1 | 0 | 0 | 0 | 0 | 1 | 1 | 1 | 0 | 0 | 23 | 33 | Carbamidomethylation | C4:Carbamidomethylation:1000.00;C6:Carbamidomethylation:1000.00 | PEAKS DB |
| I.YMLYPDFLC(+57.02)K.G | N | 69.49 | 1348.6195 | 10 | 2.9 | 675.3173 | 2 | 29.67 | 6 | F6:15617 | DaRuMP\_F7A.raw |  |  |  |  |  | 3.1666E8 |  | 4.8942E5 |  |  | 5 | 0 | 0 | 0 | 0 | 0 | 4 | 0 | 1 | 0 | 0 | 107 | 116 | Carbamidomethylation | C9:Carbamidomethylation:1000.00 | PEAKS DB |
| I.YMLYPDFLC(+57.02)KGELKC(+57.02) | N | 66.36 | 1935.8933 | 15 | 3.3 | 646.3056 | 3 | 22.67 | 6 | F6:11114 | DaRuMP\_F7A.raw |  |  |  |  |  | 2.893E6 |  |  |  |  | 1 | 0 | 0 | 0 | 0 | 0 | 1 | 0 | 0 | 0 | 0 | 107 | 121 | Carbamidomethylation | C9:Carbamidomethylation:1000.00;C15:Carbamidomethylation:1000.00 | PEAKS DB |
| V.NGAIVC(+57.02)EQGTSC(+57.02)ENR.I | N | 66.07 | 1693.7148 | 15 | 0.7 | 847.8653 | 2 | 11.59 | 1 | F1:2441 | DaRuMP\_F1.raw | 1.0878E5 |  |  |  |  |  |  |  |  |  | 1 | 1 | 0 | 0 | 0 | 0 | 0 | 0 | 0 | 0 | 0 | 70 | 84 | Carbamidomethylation | C6:Carbamidomethylation:1000.00;C12:Carbamidomethylation:1000.00 | PEAKS DB |
| F.YSSYGC(+57.02)YC(+57.02)GWGGK.A | N | 65.06 | 1543.5861 | 13 | -2.2 | 772.7986 | 2 | 15.78 | 8 | F8:2544 | DaRuMP\_F8A.raw |  |  |  |  |  | 1.7203E7 |  | 4.8744E8 |  | 1.8379E6 | 3 | 0 | 0 | 0 | 0 | 0 | 1 | 0 | 1 | 0 | 1 | 21 | 33 | Carbamidomethylation | C6:Carbamidomethylation:1000.00;C8:Carbamidomethylation:1000.00 | PEAKS DB |
| R.C(+57.02)C(+57.02)FVHDC(+57.02)C(+57.02)YGNLPDC(+57.02)NPKSDR.Y | N | 64.41 | 2673.0288 | 21 | -1.4 | 669.2619 | 4 | 11.44 | 6 | F6:2235 | DaRuMP\_F7A.raw |  |  |  |  |  | 6.5158E5 |  |  |  |  | 1 | 0 | 0 | 0 | 0 | 0 | 1 | 0 | 0 | 0 | 0 | 43 | 63 | Carbamidomethylation | C1:Carbamidomethylation:1000.00;C2:Carbamidomethylation:1000.00;C7:Carbamidomethylation:1000.00;C8:Carbamidomethylation:1000.00;C15:Carbamidomethylation:1000.00 | PEAKS DB |
| Y.GC(+57.02)YC(+57.02)GWGGK.A | N | 64.20 | 1043.3953 | 9 | 3.2 | 522.7053 | 2 | 11.29 | 6 | F6:2116 | DaRuMP\_F7A.raw |  |  | 6.3696E4 |  | 1.7244E6 | 2.9315E6 | 3.3122E5 |  |  | 4.6531E4 | 5 | 0 | 0 | 1 | 0 | 1 | 1 | 1 | 0 | 0 | 1 | 25 | 33 | Carbamidomethylation | C2:Carbamidomethylation:1000.00;C4:Carbamidomethylation:1000.00 | PEAKS DB |
| K.IYMLYPDFLC(+57.02)KG.E | N | 64.08 | 1518.7251 | 12 | 0.3 | 760.3701 | 2 | 36.91 | 8 | F8:16676 | DaRuMP\_F8A.raw |  |  |  |  |  |  |  | 2.2208E7 |  |  | 1 | 0 | 0 | 0 | 0 | 0 | 0 | 0 | 1 | 0 | 0 | 106 | 117 | Carbamidomethylation | C10:Carbamidomethylation:1000.00 | PEAKS DB |
| G.KLAVPFYSSYGC(+57.02)YC(+57.02)GWGGK.A | N | 63.92 | 2198.9917 | 19 | 0.8 | 734.0051 | 3 | 20.69 | 8 | F8:6459 | DaRuMP\_F8A.raw |  |  |  |  |  |  |  | 1.4071E5 |  |  | 1 | 0 | 0 | 0 | 0 | 0 | 0 | 0 | 1 | 0 | 0 | 15 | 33 | Carbamidomethylation | C12:Carbamidomethylation:1000.00;C14:Carbamidomethylation:1000.00 | PEAKS DB |
| H.DC(+57.02)C(+57.02)YGNLPDC(+57.02)NPK.S | N | 60.75 | 1611.6116 | 13 | 2.6 | 806.8132 | 2 | 11.50 | 6 | F6:2310 | DaRuMP\_F7A.raw |  |  |  |  |  | 4.1823E5 |  |  |  |  | 1 | 0 | 0 | 0 | 0 | 0 | 1 | 0 | 0 | 0 | 0 | 48 | 60 | Carbamidomethylation | C2:Carbamidomethylation:1000.00;C3:Carbamidomethylation:1000.00;C10:Carbamidomethylation:1000.00 | PEAKS DB |
| P.FYSSYGC(+57.02)YC(+57.02)GWGGK.A | N | 60.24 | 1690.6544 | 14 | 1.0 | 846.3353 | 2 | 16.53 | 8 | F8:3231 | DaRuMP\_F8A.raw |  |  |  |  |  |  |  | 7.1685E4 |  |  | 1 | 0 | 0 | 0 | 0 | 0 | 0 | 0 | 1 | 0 | 0 | 20 | 33 | Carbamidomethylation | C7:Carbamidomethylation:1000.00;C9:Carbamidomethylation:1000.00 | PEAKS DB |
| K.IYM(+15.99)LYPDFLC(+57.02)KG.E | N | 59.35 | 1534.7200 | 12 | 0.8 | 768.3679 | 2 | 28.04 | 8 | F8:11781 | DaRuMP\_F8A.raw |  |  |  |  |  |  |  | 5.8595E6 |  |  | 1 | 0 | 0 | 0 | 0 | 0 | 0 | 0 | 1 | 0 | 0 | 106 | 117 | Oxidation (M); Carbamidomethylation | M3:Oxidation (M):1000.00;C10:Carbamidomethylation:1000.00 | PEAKS DB |
| S.YGC(+57.02)YC(+57.02)GWGGK.A | N | 58.24 | 1206.4586 | 10 | 0.0 | 604.2366 | 2 | 11.59 | 5 | F5:2425 | DaRuMP\_F6.raw |  |  |  |  | 1.3442E7 |  |  |  |  |  | 1 | 0 | 0 | 0 | 0 | 1 | 0 | 0 | 0 | 0 | 0 | 24 | 33 | Carbamidomethylation | C3:Carbamidomethylation:1000.00;C5:Carbamidomethylation:1000.00 | PEAKS DB |
| F.GMMILEETGK.L | N | 55.74 | 1107.5304 | 10 | 0.0 | 554.7725 | 2 | 16.49 | 8 | F8:3169 | DaRuMP\_F8A.raw |  |  |  |  |  |  |  | 1.5241E6 |  |  | 1 | 0 | 0 | 0 | 0 | 0 | 0 | 0 | 1 | 0 | 0 | 6 | 15 |  |  | PEAKS DB |
| Y.MLYPDFLC(+57.02)K.G | N | 54.73 | 1185.5563 | 9 | 0.4 | 593.7856 | 2 | 24.23 | 8 | F8:8823 | DaRuMP\_F8A.raw |  |  |  |  |  |  |  | 5.9068E6 |  |  | 1 | 0 | 0 | 0 | 0 | 0 | 0 | 0 | 1 | 0 | 0 | 108 | 116 | Carbamidomethylation | C8:Carbamidomethylation:1000.00 | PEAKS DB |
| K.AAAIC(+57.02)FR.R | N | 54.51 | 807.4061 | 7 | -5.0 | 404.7083 | 2 | 17.94 | 8 | F8:4539 | DaRuMP\_F8A.raw |  |  |  |  |  |  |  | 2.6963E7 |  |  | 2 | 0 | 0 | 0 | 0 | 0 | 0 | 0 | 2 | 0 | 0 | 91 | 97 | Carbamidomethylation | C5:Carbamidomethylation:1000.00 | PEAKS DB |
| M.MILEETGK.L | N | 54.26 | 919.4684 | 8 | -0.1 | 460.7415 | 2 | 11.49 | 7 | F7:2297 | DaRuMP\_F7B.raw |  |  |  |  |  |  | 4.4177E5 | 1.1565E6 |  |  | 2 | 0 | 0 | 0 | 0 | 0 | 0 | 1 | 1 | 0 | 0 | 8 | 15 |  |  | PEAKS DB |
| A.IVC(+57.02)EQGTSC(+57.02)ENR.I | N | 52.07 | 1451.6133 | 12 | 1.7 | 726.8152 | 2 | 15.50 | 8 | F8:2299 | DaRuMP\_F8A.raw |  |  |  |  |  |  |  | 0 |  |  | 0 | 0 | 0 | 0 | 0 | 0 | 0 | 0 | 0 | 0 | 0 | 73 | 84 | Carbamidomethylation | C3:Carbamidomethylation:1000.00;C9:Carbamidomethylation:1000.00 | PEAKS DB |
| K.IYMLYPDFLC(+57.02)KGELKC(+57.02) | N | 52.05 | 2048.9773 | 16 | 0.4 | 684.0000 | 3 | 30.31 | 8 | F8:12508 | DaRuMP\_F8A.raw |  |  |  |  |  |  |  | 2.9166E6 |  |  | 1 | 0 | 0 | 0 | 0 | 0 | 0 | 0 | 1 | 0 | 0 | 106 | 121 | Carbamidomethylation | C10:Carbamidomethylation:1000.00;C16:Carbamidomethylation:1000.00 | PEAKS DB |
| total 46 peptides |
| --- |

P59071|PA2B8\_DABRR

back to list

  

| Protein Coverage
| Supporting Peptides
|

Protein Coverage:

Supporting Peptides:

| Peptide | Uniq | -10lgP | Mass | Length | ppm | m/z | z | RT | Fraction | Scan | Source File | Area F1 | Area F10 | Area F2\_3 | Area F5 | Area F6 | Area F7A | Area F7B | Area F8A | Area F8B | Area F9 | #Feature | #Feature F1 | #Feature F10 | #Feature F2\_3 | #Feature F5 | #Feature F6 | #Feature F7A | #Feature F7B | #Feature F8A | #Feature F8B | #Feature F9 | Start | End | PTM | AScore | Found By |
| --- | --- | --- | --- | --- | --- | --- | --- | --- | --- | --- | --- | --- | --- | --- | --- | --- | --- | --- | --- | --- | --- | --- | --- | --- | --- | --- | --- | --- | --- | --- | --- | --- | --- | --- | --- | --- | --- |
| R.C(+57.02)C(+57.02)FVHDC(+57.02)C(+57.02)YGNLPDC(+57.02)NPK.S | N | 103.24 | 2314.8687 | 18 | 1.4 | 1158.4404 | 2 | 11.58 | 6 | F6:2373 | DaRuMP\_F7A.raw | 2.8891E5 |  |  |  | 1.2509E6 | 4.8203E8 | 1.5976E7 | 2.1062E8 |  | 1.2674E5 | 9 | 1 | 0 | 0 | 0 | 1 | 2 | 2 | 2 | 0 | 1 | 43 | 60 | Carbamidomethylation | C1:Carbamidomethylation:1000.00;C2:Carbamidomethylation:1000.00;C7:Carbamidomethylation:1000.00;C8:Carbamidomethylation:1000.00;C15:Carbamidomethylation:1000.00 | PEAKS DB |
| R.IC(+57.02)EC(+57.02)DKAAAIC(+57.02)FRQNLNTYSK.K | N | 102.88 | 2561.1824 | 21 | 3.5 | 641.3035 | 4 | 11.64 | 6 | F6:2438 | DaRuMP\_F7A.raw |  |  |  |  |  | 1.1977E6 |  |  |  |  | 1 | 0 | 0 | 0 | 0 | 0 | 1 | 0 | 0 | 0 | 0 | 85 | 105 | Carbamidomethylation | C2:Carbamidomethylation:1000.00;C4:Carbamidomethylation:1000.00;C11:Carbamidomethylation:1000.00 | PEAKS DB |
| K.LAIPSYSSYGC(+57.02)YC(+57.02)GWGGK.G | N | 101.59 | 2024.8760 | 18 | 2.6 | 1013.4454 | 2 | 20.00 | 6 | F6:9229 | DaRuMP\_F7A.raw |  |  |  |  | 5.0664E6 | 1.6353E9 | 2.2166E7 |  |  |  | 9 | 0 | 0 | 0 | 0 | 1 | 7 | 1 | 0 | 0 | 0 | 16 | 33 | Carbamidomethylation | C11:Carbamidomethylation:1000.00;C13:Carbamidomethylation:1000.00 | PEAKS DB |
| R.IC(+57.02)EC(+57.02)DKAAAIC(+57.02)FR.Q | N | 98.35 | 1612.7159 | 13 | 1.9 | 538.5790 | 3 | 11.50 | 6 | F6:2309 | DaRuMP\_F7A.raw |  |  |  |  |  | 7.839E6 |  |  |  |  | 2 | 0 | 0 | 0 | 0 | 0 | 2 | 0 | 0 | 0 | 0 | 85 | 97 | Carbamidomethylation | C2:Carbamidomethylation:1000.00;C4:Carbamidomethylation:1000.00;C11:Carbamidomethylation:1000.00 | PEAKS DB |
| K.LAIPSYSSYGC(+57.02)YC(+57.02)GWGGKG.T | N | 94.80 | 2081.8975 | 19 | 2.7 | 1041.9563 | 2 | 19.60 | 6 | F6:9397 | DaRuMP\_F7A.raw |  |  |  |  |  | 3.7058E8 | 2.9762E6 |  |  |  | 2 | 0 | 0 | 0 | 0 | 0 | 1 | 1 | 0 | 0 | 0 | 16 | 34 | Carbamidomethylation | C11:Carbamidomethylation:1000.00;C13:Carbamidomethylation:1000.00 | PEAKS DB |
| K.LAIPSYSSYGC(+57.02)YC(+57.02)GWGGKGTPK.D | N | 94.72 | 2408.0928 | 22 | 2.8 | 803.7051 | 3 | 13.48 | 6 | F6:3933 | DaRuMP\_F7A.raw |  |  |  |  |  | 1.2619E7 |  |  |  |  | 1 | 0 | 0 | 0 | 0 | 0 | 1 | 0 | 0 | 0 | 0 | 16 | 37 | Carbamidomethylation | C11:Carbamidomethylation:1000.00;C13:Carbamidomethylation:1000.00 | PEAKS DB |
| K.GTSC(+57.02)ENRIC(+57.02)EC(+57.02)DKAAAIC(+57.02)FR.Q | N | 93.89 | 2417.0344 | 20 | -1.5 | 806.6823 | 3 | 11.44 | 6 | F6:2255 | DaRuMP\_F7A.raw |  |  |  |  |  | 5.1971E6 |  |  |  |  | 2 | 0 | 0 | 0 | 0 | 0 | 2 | 0 | 0 | 0 | 0 | 78 | 97 | Carbamidomethylation | C4:Carbamidomethylation:1000.00;C9:Carbamidomethylation:1000.00;C11:Carbamidomethylation:1000.00;C18:Carbamidomethylation:1000.00 | PEAKS DB |
| C.FVHDC(+57.02)C(+57.02)YGNLPDC(+57.02)NPK.S | N | 93.60 | 1994.8074 | 16 | 2.2 | 998.4108 | 2 | 11.39 | 6 | F6:2215 | DaRuMP\_F7A.raw |  |  |  |  |  | 2.6011E6 |  |  |  |  | 2 | 0 | 0 | 0 | 0 | 0 | 2 | 0 | 0 | 0 | 0 | 45 | 60 | Carbamidomethylation | C5:Carbamidomethylation:1000.00;C6:Carbamidomethylation:1000.00;C13:Carbamidomethylation:1000.00 | PEAKS DB |
| K.AAAIC(+57.02)FRQNLNTYSK.K | N | 90.83 | 1755.8726 | 15 | 4.0 | 586.2991 | 3 | 11.60 | 6 | F6:2389 | DaRuMP\_F7A.raw |  |  |  |  |  | 1.4564E6 |  |  |  |  | 2 | 0 | 0 | 0 | 0 | 0 | 2 | 0 | 0 | 0 | 0 | 91 | 105 | Carbamidomethylation | C5:Carbamidomethylation:1000.00 | PEAKS DB |
| R.VNGAIVC(+57.02)EKGTSC(+57.02)ENR.I | N | 82.66 | 1792.8196 | 16 | 3.3 | 897.4178 | 2 | 10.74 | 6 | F6:1635 | DaRuMP\_F7A.raw |  |  |  |  |  | 1.2457E5 |  |  |  |  | 2 | 0 | 0 | 0 | 0 | 0 | 2 | 0 | 0 | 0 | 0 | 69 | 84 | Carbamidomethylation | C7:Carbamidomethylation:1000.00;C13:Carbamidomethylation:1000.00 | PEAKS DB |
| K.MILEETGKLAIPSYSSYGC(+57.02)YC(+57.02)GWGGK.G | N | 82.54 | 2926.3340 | 26 | 3.0 | 976.4525 | 3 | 25.54 | 6 | F6:13433 | DaRuMP\_F7A.raw |  |  |  |  |  | 1.8064E7 |  |  |  |  | 2 | 0 | 0 | 0 | 0 | 0 | 2 | 0 | 0 | 0 | 0 | 8 | 33 | Carbamidomethylation | C19:Carbamidomethylation:1000.00;C21:Carbamidomethylation:1000.00 | PEAKS DB |
| K.KYMLYPDFLC(+57.02)KGELK.C | Y | 80.23 | 1903.9575 | 15 | 2.8 | 635.6600 | 3 | 13.88 | 6 | F6:4296 | DaRuMP\_F7A.raw |  |  |  |  |  | 1.3266E6 |  |  |  |  | 1 | 0 | 0 | 0 | 0 | 0 | 1 | 0 | 0 | 0 | 0 | 106 | 120 | Carbamidomethylation | C10:Carbamidomethylation:1000.00 | PEAKS DB |
| K.KYM(+15.99)LYPDFLC(+57.02)K.G | N | 79.91 | 1492.7095 | 11 | -2.7 | 747.3582 | 2 | 14.15 | 6 | F6:4416 | DaRuMP\_F7A.raw |  |  |  |  |  | 2.4358E7 | 6.9046E5 |  |  |  | 5 | 0 | 0 | 0 | 0 | 0 | 4 | 1 | 0 | 0 | 0 | 106 | 116 | Oxidation (M); Carbamidomethylation | M3:Oxidation (M):1000.00;C10:Carbamidomethylation:1000.00 | PEAKS DB |
| L.AIPSYSSYGC(+57.02)YC(+57.02)GWGGK.G | N | 78.39 | 1911.7920 | 17 | 2.5 | 956.9033 | 2 | 13.74 | 6 | F6:4134 | DaRuMP\_F7A.raw |  |  |  |  |  | 6.7076E6 |  |  |  |  | 1 | 0 | 0 | 0 | 0 | 0 | 1 | 0 | 0 | 0 | 0 | 17 | 33 | Carbamidomethylation | C10:Carbamidomethylation:1000.00;C12:Carbamidomethylation:1000.00 | PEAKS DB |
| K.DATDRC(+57.02)C(+57.02)FVHDC(+57.02)C(+57.02)YGNLPDC(+57.02)NPK.S | N | 78.23 | 2873.1084 | 23 | 1.9 | 958.7096 | 3 | 11.58 | 6 | F6:2387 | DaRuMP\_F7A.raw |  |  |  |  |  | 1.375E6 |  |  |  |  | 1 | 0 | 0 | 0 | 0 | 0 | 1 | 0 | 0 | 0 | 0 | 38 | 60 | Carbamidomethylation | C6:Carbamidomethylation:1000.00;C7:Carbamidomethylation:1000.00;C12:Carbamidomethylation:1000.00;C13:Carbamidomethylation:1000.00;C20:Carbamidomethylation:1000.00 | PEAKS DB |
| F.VHDC(+57.02)C(+57.02)YGNLPDC(+57.02)NPK.S | N | 77.58 | 1847.7389 | 15 | 1.3 | 924.8757 | 2 | 11.13 | 6 | F6:1990 | DaRuMP\_F7A.raw |  |  |  |  |  | 4.0829E5 |  |  |  |  | 1 | 0 | 0 | 0 | 0 | 0 | 1 | 0 | 0 | 0 | 0 | 46 | 60 | Carbamidomethylation | C4:Carbamidomethylation:1000.00;C5:Carbamidomethylation:1000.00;C12:Carbamidomethylation:1000.00 | PEAKS DB |
| D.C(+57.02)C(+57.02)YGNLPDC(+57.02)NPK.S | N | 77.58 | 1496.5846 | 12 | 3.4 | 749.3003 | 2 | 11.37 | 6 | F6:2187 | DaRuMP\_F7A.raw |  |  |  |  |  | 1.8907E6 |  | 0 |  | 5.5068E4 | 2 | 0 | 0 | 0 | 0 | 0 | 1 | 0 | 0 | 0 | 1 | 49 | 60 | Carbamidomethylation | C1:Carbamidomethylation:1000.00;C2:Carbamidomethylation:1000.00;C9:Carbamidomethylation:1000.00 | PEAKS DB |
| K.YMLYPDFLC(+57.02)KGELK.C | N | 77.16 | 1775.8625 | 14 | 2.8 | 592.9617 | 3 | 21.57 | 6 | F6:10344 | DaRuMP\_F7A.raw |  |  |  |  |  | 3.328E6 |  |  |  |  | 2 | 0 | 0 | 0 | 0 | 0 | 2 | 0 | 0 | 0 | 0 | 107 | 120 | Carbamidomethylation | C9:Carbamidomethylation:1000.00 | PEAKS DB |
| K.KYMLYPDFLC(+57.02)K.G | N | 75.39 | 1476.7145 | 11 | 0.6 | 739.3649 | 2 | 16.97 | 7 | F7:7076 | DaRuMP\_F7B.raw |  |  |  |  | 0 | 1.759E7 | 4.3463E5 | 4.6011E4 |  |  | 4 | 0 | 0 | 0 | 0 | 0 | 1 | 2 | 1 | 0 | 0 | 106 | 116 | Carbamidomethylation | C10:Carbamidomethylation:1000.00 | PEAKS DB |
| C.C(+57.02)YGNLPDC(+57.02)NPK.S | N | 75.32 | 1336.5540 | 11 | 3.2 | 669.2848 | 2 | 11.29 | 6 | F6:2118 | DaRuMP\_F7A.raw |  |  |  |  |  | 3.3405E5 |  |  |  |  | 1 | 0 | 0 | 0 | 0 | 0 | 1 | 0 | 0 | 0 | 0 | 50 | 60 | Carbamidomethylation | C1:Carbamidomethylation:1000.00;C8:Carbamidomethylation:1000.00 | PEAKS DB |
| K.M(+15.99)ILEETGKLAIPSYSSYGC(+57.02)YC(+57.02)GWGGK.G | N | 75.14 | 2942.3289 | 26 | 2.9 | 981.7841 | 3 | 21.53 | 6 | F6:10261 | DaRuMP\_F7A.raw |  |  |  |  |  | 3.3346E6 |  |  |  |  | 1 | 0 | 0 | 0 | 0 | 0 | 1 | 0 | 0 | 0 | 0 | 8 | 33 | Oxidation (M); Carbamidomethylation | M1:Oxidation (M):1000.00;C19:Carbamidomethylation:1000.00;C21:Carbamidomethylation:1000.00 | PEAKS DB |
| K.YM(+15.99)LYPDFLC(+57.02)K.G | N | 73.83 | 1364.6145 | 10 | 2.7 | 683.3147 | 2 | 24.65 | 6 | F6:13883 | DaRuMP\_F7A.raw |  |  |  |  | 5.5321E5 | 5.3341E7 |  |  |  |  | 5 | 0 | 0 | 0 | 0 | 1 | 4 | 0 | 0 | 0 | 0 | 107 | 116 | Oxidation (M); Carbamidomethylation | M2:Oxidation (M):1000.00;C9:Carbamidomethylation:1000.00 | PEAKS DB |
| Y.SSYGC(+57.02)YC(+57.02)GWGGK.G | N | 71.73 | 1380.5227 | 12 | 2.9 | 691.2690 | 2 | 11.64 | 6 | F6:2416 | DaRuMP\_F7A.raw |  |  |  |  |  | 7.7516E6 | 7.2278E5 | 2.165E6 |  |  | 3 | 0 | 0 | 0 | 0 | 0 | 1 | 1 | 1 | 0 | 0 | 22 | 33 | Carbamidomethylation | C5:Carbamidomethylation:1000.00;C7:Carbamidomethylation:1000.00 | PEAKS DB |
| C.YGNLPDC(+57.02)NPK.S | N | 71.12 | 1176.5233 | 10 | 2.2 | 589.2688 | 2 | 11.15 | 6 | F6:1993 | DaRuMP\_F7A.raw |  |  |  |  |  | 3.1081E5 |  |  |  |  | 1 | 0 | 0 | 0 | 0 | 0 | 1 | 0 | 0 | 0 | 0 | 51 | 60 | Carbamidomethylation | C7:Carbamidomethylation:1000.00 | PEAKS DB |
| S.SYGC(+57.02)YC(+57.02)GWGGK.G | N | 71.11 | 1293.4907 | 11 | 2.9 | 647.7529 | 2 | 11.61 | 6 | F6:2413 | DaRuMP\_F7A.raw | 3.6812E5 |  |  |  |  | 1.7045E6 | 5.493E5 | 4.5706E5 |  |  | 4 | 1 | 0 | 0 | 0 | 0 | 1 | 1 | 1 | 0 | 0 | 23 | 33 | Carbamidomethylation | C4:Carbamidomethylation:1000.00;C6:Carbamidomethylation:1000.00 | PEAKS DB |
| K.YMLYPDFLC(+57.02)K.G | N | 69.49 | 1348.6195 | 10 | 2.9 | 675.3173 | 2 | 29.67 | 6 | F6:15617 | DaRuMP\_F7A.raw |  |  |  |  |  | 3.1666E8 |  | 4.8942E5 |  |  | 5 | 0 | 0 | 0 | 0 | 0 | 4 | 0 | 1 | 0 | 0 | 107 | 116 | Carbamidomethylation | C9:Carbamidomethylation:1000.00 | PEAKS DB |
| R.VNGAIVC(+57.02)EK.G | N | 68.51 | 988.5012 | 9 | 1.6 | 495.2574 | 2 | 10.95 | 6 | F6:1793 | DaRuMP\_F7A.raw |  |  |  |  | 1.1512E5 | 2.4623E5 |  |  |  | 2.0223E4 | 3 | 0 | 0 | 0 | 0 | 1 | 1 | 0 | 0 | 0 | 1 | 69 | 77 | Carbamidomethylation | C7:Carbamidomethylation:1000.00 | PEAKS DB |
| SLLEFGKMILEETGKLAIPSYSSYGC(+57.02)YC(+57.02)GWGGK.G | N | 66.87 | 3700.7615 | 33 | 3.4 | 926.1985 | 4 | 57.67 | 6 | F6:27647 | DaRuMP\_F7A.raw |  |  |  |  |  | 9.1491E6 |  |  |  |  | 1 | 0 | 0 | 0 | 0 | 0 | 1 | 0 | 0 | 0 | 0 | 1 | 33 | Carbamidomethylation | C26:Carbamidomethylation:1000.00;C28:Carbamidomethylation:1000.00 | PEAKS DB |
| K.YMLYPDFLC(+57.02)KGELKC(+57.02) | N | 66.36 | 1935.8933 | 15 | 3.3 | 646.3056 | 3 | 22.67 | 6 | F6:11114 | DaRuMP\_F7A.raw |  |  |  |  |  | 2.893E6 |  |  |  |  | 1 | 0 | 0 | 0 | 0 | 0 | 1 | 0 | 0 | 0 | 0 | 107 | 121 | Carbamidomethylation | C9:Carbamidomethylation:1000.00;C15:Carbamidomethylation:1000.00 | PEAKS DB |
| S.YSSYGC(+57.02)YC(+57.02)GWGGK.G | N | 65.06 | 1543.5861 | 13 | -2.2 | 772.7986 | 2 | 15.78 | 8 | F8:2544 | DaRuMP\_F8A.raw |  |  |  |  |  | 1.7203E7 |  | 4.8744E8 |  | 1.8379E6 | 3 | 0 | 0 | 0 | 0 | 0 | 1 | 0 | 1 | 0 | 1 | 21 | 33 | Carbamidomethylation | C6:Carbamidomethylation:1000.00;C8:Carbamidomethylation:1000.00 | PEAKS DB |
| SLLEFGKMILEETGK.L | N | 64.69 | 1693.8960 | 15 | 3.3 | 565.6398 | 3 | 36.64 | 6 | F6:19168 | DaRuMP\_F7A.raw |  |  |  |  |  | 6.88E5 |  |  |  |  | 2 | 0 | 0 | 0 | 0 | 0 | 2 | 0 | 0 | 0 | 0 | 1 | 15 |  |  | PEAKS DB |
| R.C(+57.02)C(+57.02)FVHDC(+57.02)C(+57.02)YGNLPDC(+57.02)NPKSDR.Y | N | 64.41 | 2673.0288 | 21 | -1.4 | 669.2619 | 4 | 11.44 | 6 | F6:2235 | DaRuMP\_F7A.raw |  |  |  |  |  | 6.5158E5 |  |  |  |  | 1 | 0 | 0 | 0 | 0 | 0 | 1 | 0 | 0 | 0 | 0 | 43 | 63 | Carbamidomethylation | C1:Carbamidomethylation:1000.00;C2:Carbamidomethylation:1000.00;C7:Carbamidomethylation:1000.00;C8:Carbamidomethylation:1000.00;C15:Carbamidomethylation:1000.00 | PEAKS DB |
| Y.GC(+57.02)YC(+57.02)GWGGK.G | N | 64.20 | 1043.3953 | 9 | 3.2 | 522.7053 | 2 | 11.29 | 6 | F6:2116 | DaRuMP\_F7A.raw |  |  | 6.3696E4 |  | 1.7244E6 | 2.9315E6 | 3.3122E5 |  |  | 4.6531E4 | 5 | 0 | 0 | 1 | 0 | 1 | 1 | 1 | 0 | 0 | 1 | 25 | 33 | Carbamidomethylation | C2:Carbamidomethylation:1000.00;C4:Carbamidomethylation:1000.00 | PEAKS DB |
| K.KYM(+15.99)LYPDFLC(+57.02)KGELK.C | Y | 63.23 | 1919.9525 | 15 | 2.5 | 640.9915 | 3 | 12.44 | 6 | F6:3142 | DaRuMP\_F7A.raw |  |  |  |  |  | 2.336E5 |  |  |  |  | 2 | 0 | 0 | 0 | 0 | 0 | 2 | 0 | 0 | 0 | 0 | 106 | 120 | Oxidation (M); Carbamidomethylation | M3:Oxidation (M):1000.00;C10:Carbamidomethylation:1000.00 | PEAKS DB |
| K.LAIPSYSSYGC(+57.02)YC(+57.02)GW.G | N | 62.39 | 1782.7382 | 15 | 3.5 | 892.3773 | 2 | 38.70 | 6 | F6:20107 | DaRuMP\_F7A.raw |  |  |  |  |  | 8.0533E6 |  |  |  |  | 1 | 0 | 0 | 0 | 0 | 0 | 1 | 0 | 0 | 0 | 0 | 16 | 30 | Carbamidomethylation | C11:Carbamidomethylation:1000.00;C13:Carbamidomethylation:1000.00 | PEAKS DB |
| M.ILEETGKLAIPSYSSYGC(+57.02)YC(+57.02)GWGGK.G | N | 61.35 | 2795.2935 | 25 | 1.5 | 932.7709 | 3 | 20.00 | 6 | F6:9260 | DaRuMP\_F7A.raw |  |  |  |  |  | 1.6934E6 |  |  |  |  | 1 | 0 | 0 | 0 | 0 | 0 | 1 | 0 | 0 | 0 | 0 | 9 | 33 | Carbamidomethylation | C18:Carbamidomethylation:1000.00;C20:Carbamidomethylation:1000.00 | PEAKS DB |
| H.DC(+57.02)C(+57.02)YGNLPDC(+57.02)NPK.S | N | 60.75 | 1611.6116 | 13 | 2.6 | 806.8132 | 2 | 11.50 | 6 | F6:2310 | DaRuMP\_F7A.raw |  |  |  |  |  | 4.1823E5 |  |  |  |  | 1 | 0 | 0 | 0 | 0 | 0 | 1 | 0 | 0 | 0 | 0 | 48 | 60 | Carbamidomethylation | C2:Carbamidomethylation:1000.00;C3:Carbamidomethylation:1000.00;C10:Carbamidomethylation:1000.00 | PEAKS DB |
| K.LAIPSYSSYGC(+57.02)Y.C | N | 59.81 | 1379.6067 | 12 | 2.6 | 690.8107 | 2 | 24.63 | 6 | F6:12734 | DaRuMP\_F7A.raw |  |  |  |  |  | 1.1741E8 |  |  |  |  | 1 | 0 | 0 | 0 | 0 | 0 | 1 | 0 | 0 | 0 | 0 | 16 | 27 | Carbamidomethylation | C11:Carbamidomethylation:1000.00 | PEAKS DB |
| S.YGC(+57.02)YC(+57.02)GWGGK.G | N | 58.24 | 1206.4586 | 10 | 0.0 | 604.2366 | 2 | 11.59 | 5 | F5:2425 | DaRuMP\_F6.raw |  |  |  |  | 1.3442E7 |  |  |  |  |  | 1 | 0 | 0 | 0 | 0 | 1 | 0 | 0 | 0 | 0 | 0 | 24 | 33 | Carbamidomethylation | C3:Carbamidomethylation:1000.00;C5:Carbamidomethylation:1000.00 | PEAKS DB |
| E.ETGKLAIPSYSSYGC(+57.02)YC(+57.02)GWGGK.G | N | 57.51 | 2440.0828 | 22 | -8.1 | 814.3596 | 3 | 16.08 | 6 | F6:6009 | DaRuMP\_F7A.raw |  |  |  |  |  | 7.6476E5 |  |  |  |  | 1 | 0 | 0 | 0 | 0 | 0 | 1 | 0 | 0 | 0 | 0 | 12 | 33 | Carbamidomethylation | C15:Carbamidomethylation:1000.00;C17:Carbamidomethylation:1000.00 | PEAKS DB |
| R.VNGAIVC(+57.02)EKG.T | N | 56.49 | 1045.5226 | 10 | 1.9 | 523.7683 | 2 | 11.01 | 6 | F6:1789 | DaRuMP\_F7A.raw |  |  |  |  |  | 4.0766E6 |  |  |  |  | 1 | 0 | 0 | 0 | 0 | 0 | 1 | 0 | 0 | 0 | 0 | 69 | 78 | Carbamidomethylation | C7:Carbamidomethylation:1000.00 | PEAKS DB |
| Y.MLYPDFLC(+57.02)K.G | N | 54.73 | 1185.5563 | 9 | 0.4 | 593.7856 | 2 | 24.23 | 8 | F8:8823 | DaRuMP\_F8A.raw |  |  |  |  |  |  |  | 5.9068E6 |  |  | 1 | 0 | 0 | 0 | 0 | 0 | 0 | 0 | 1 | 0 | 0 | 108 | 116 | Carbamidomethylation | C8:Carbamidomethylation:1000.00 | PEAKS DB |
| K.AAAIC(+57.02)FR.Q | N | 54.51 | 807.4061 | 7 | -5.0 | 404.7083 | 2 | 17.94 | 8 | F8:4539 | DaRuMP\_F8A.raw |  |  |  |  |  |  |  | 2.6963E7 |  |  | 2 | 0 | 0 | 0 | 0 | 0 | 0 | 0 | 2 | 0 | 0 | 91 | 97 | Carbamidomethylation | C5:Carbamidomethylation:1000.00 | PEAKS DB |
| K.MILEETGK.L | N | 54.26 | 919.4684 | 8 | -0.1 | 460.7415 | 2 | 11.49 | 7 | F7:2297 | DaRuMP\_F7B.raw |  |  |  |  |  |  | 4.4177E5 | 1.1565E6 |  |  | 2 | 0 | 0 | 0 | 0 | 0 | 0 | 1 | 1 | 0 | 0 | 8 | 15 |  |  | PEAKS DB |
| total 44 peptides |
| --- |

B8K1W0|VM3DK\_DABRR

back to list

  

| Protein Coverage
| Supporting Peptides
|

Protein Coverage:

Supporting Peptides:

| Peptide | Uniq | -10lgP | Mass | Length | ppm | m/z | z | RT | Fraction | Scan | Source File | Area F1 | Area F10 | Area F2\_3 | Area F5 | Area F6 | Area F7A | Area F7B | Area F8A | Area F8B | Area F9 | #Feature | #Feature F1 | #Feature F10 | #Feature F2\_3 | #Feature F5 | #Feature F6 | #Feature F7A | #Feature F7B | #Feature F8A | #Feature F8B | #Feature F9 | Start | End | PTM | AScore | Found By |
| --- | --- | --- | --- | --- | --- | --- | --- | --- | --- | --- | --- | --- | --- | --- | --- | --- | --- | --- | --- | --- | --- | --- | --- | --- | --- | --- | --- | --- | --- | --- | --- | --- | --- | --- | --- | --- | --- |
| R.YFNPYSYVELIITVDHSMVTK.Y | Y | 96.90 | 2518.2454 | 21 | 0.0 | 840.4224 | 3 | 59.95 | 2 | F2:15989 | DaRuMP\_F10.raw |  | 2.7926E6 |  |  |  |  |  |  |  |  | 1 | 0 | 1 | 0 | 0 | 0 | 0 | 0 | 0 | 0 | 0 | 198 | 218 |  |  | PEAKS DB |
| R.FLTEHNPEC(+57.02)IINPPLR.T | Y | 96.35 | 1948.9829 | 16 | 0.2 | 975.4990 | 2 | 12.88 | 2 | F2:3486 | DaRuMP\_F10.raw |  | 1.4086E8 |  |  |  |  |  |  |  |  | 3 | 0 | 3 | 0 | 0 | 0 | 0 | 0 | 0 | 0 | 0 | 386 | 401 | Carbamidomethylation | C9:Carbamidomethylation:1000.00 | PEAKS DB |
| K.YSVGVVQDHSK.I | Y | 91.43 | 1217.6040 | 11 | 0.4 | 609.8095 | 2 | 11.01 | 2 | F2:1863 | DaRuMP\_F10.raw |  | 7.8975E6 |  |  |  |  |  |  |  |  | 2 | 0 | 2 | 0 | 0 | 0 | 0 | 0 | 0 | 0 | 0 | 318 | 328 |  |  | PEAKS DB |
| R.TDIVSPPAC(+57.02)GNELLER.G | Y | 89.96 | 1769.8618 | 16 | -0.1 | 885.9381 | 2 | 13.95 | 2 | F2:3751 | DaRuMP\_F10.raw |  | 1.4132E8 |  |  |  |  |  |  |  |  | 2 | 0 | 2 | 0 | 0 | 0 | 0 | 0 | 0 | 0 | 0 | 402 | 417 | Carbamidomethylation | C9:Carbamidomethylation:1000.00 | PEAKS DB |
| K.LHSWVEC(+57.02)ESGK.C | Y | 85.44 | 1330.5975 | 11 | -1.3 | 666.3052 | 2 | 11.16 | 2 | F2:2010 | DaRuMP\_F10.raw |  | 1.3987E7 | 2.884E4 |  |  |  |  |  |  |  | 3 | 0 | 2 | 1 | 0 | 0 | 0 | 0 | 0 | 0 | 0 | 441 | 451 | Carbamidomethylation | C7:Carbamidomethylation:1000.00 | PEAKS DB |
| L.ESGNVNDYEVVYPQK.V | N | 83.54 | 1739.8002 | 15 | 0.4 | 870.9077 | 2 | 11.80 | 3 | F3:2602 | DaRuMP\_F2\_F3.raw |  |  | 1.005E6 |  |  |  |  |  |  |  | 1 | 0 | 0 | 1 | 0 | 0 | 0 | 0 | 0 | 0 | 0 | 24 | 38 |  |  | PEAKS DB |
| R.TWVFELVNTINEIFK.Y | Y | 81.99 | 1851.9771 | 15 | 0.3 | 926.9961 | 2 | 81.53 | 2 | F2:19458 | DaRuMP\_F10.raw |  | 2.1679E6 |  |  |  |  |  |  |  |  | 1 | 0 | 1 | 0 | 0 | 0 | 0 | 0 | 0 | 0 | 0 | 228 | 242 |  |  | PEAKS DB |
| R.RFLTEHNPEC(+57.02)IINPPLR.T | Y | 80.86 | 2105.0840 | 17 | 0.5 | 702.7023 | 3 | 11.85 | 2 | F2:2666 | DaRuMP\_F10.raw |  | 4.5794E5 |  |  |  |  |  |  |  |  | 1 | 0 | 1 | 0 | 0 | 0 | 0 | 0 | 0 | 0 | 0 | 385 | 401 | Carbamidomethylation | C10:Carbamidomethylation:1000.00 | PEAKS DB |
| R.YFNPYSYVELIITVDHSM(+15.99)VTK.Y | Y | 80.58 | 2534.2402 | 21 | 0.7 | 845.7546 | 3 | 50.94 | 2 | F2:13988 | DaRuMP\_F10.raw |  | 1.4157E6 |  |  |  |  |  |  |  |  | 1 | 0 | 1 | 0 | 0 | 0 | 0 | 0 | 0 | 0 | 0 | 198 | 218 | Oxidation (M) | M18:Oxidation (M):1000.00 | PEAKS DB |
| K.SPGNQIPC(+57.02)LPYYIPSDENK.G | Y | 75.80 | 2191.0256 | 19 | 0.2 | 1096.5203 | 2 | 22.29 | 2 | F2:6801 | DaRuMP\_F10.raw |  | 7.9359E6 |  |  |  |  |  |  |  |  | 1 | 0 | 1 | 0 | 0 | 0 | 0 | 0 | 0 | 0 | 0 | 570 | 588 | Carbamidomethylation | C8:Carbamidomethylation:1000.00 | PEAKS DB |
| Y.SC(+57.02)IM(+15.99)SAVLGDQPSK.Y | Y | 74.47 | 1507.7010 | 14 | 0.5 | 754.8582 | 2 | 11.60 | 2 | F2:2431 | DaRuMP\_F10.raw |  | 1.7608E5 |  |  |  |  |  |  |  |  | 1 | 0 | 1 | 0 | 0 | 0 | 0 | 0 | 0 | 0 | 0 | 360 | 373 | Carbamidomethylation; Oxidation (M) | C2:Carbamidomethylation:1000.00;M4:Oxidation (M):1000.00 | PEAKS DB |
| R.PC(+57.02)LHNFGYC(+57.02)YNGK.C | Y | 74.28 | 1628.6864 | 13 | 0.1 | 543.9028 | 3 | 11.48 | 2 | F2:2347 | DaRuMP\_F10.raw |  | 3.3084E5 |  |  |  |  |  |  |  |  | 1 | 0 | 1 | 0 | 0 | 0 | 0 | 0 | 0 | 0 | 0 | 496 | 508 | Carbamidomethylation | C2:Carbamidomethylation:1000.00;C9:Carbamidomethylation:1000.00 | PEAKS DB |
| E.SGNVNDYEVVYPQK.V | N | 72.78 | 1610.7576 | 14 | 0.7 | 806.3866 | 2 | 11.74 | 3 | F3:2548 | DaRuMP\_F2\_F3.raw |  |  | 4.3606E4 |  |  |  |  |  |  |  | 1 | 0 | 0 | 1 | 0 | 0 | 0 | 0 | 0 | 0 | 0 | 25 | 38 |  |  | PEAKS DB |
| K.YKNDLTAIR.T | Y | 72.38 | 1092.5928 | 9 | 0.1 | 547.3037 | 2 | 11.18 | 2 | F2:2042 | DaRuMP\_F10.raw |  | 1.3707E6 |  |  |  |  |  |  |  |  | 1 | 0 | 1 | 0 | 0 | 0 | 0 | 0 | 0 | 0 | 0 | 219 | 227 |  |  | PEAKS DB |
| R.SANC(+57.02)PVDEFHENGR.P | Y | 72.22 | 1630.6794 | 14 | -1.3 | 816.3459 | 2 | 11.16 | 2 | F2:2022 | DaRuMP\_F10.raw |  | 2.536E6 |  |  |  |  |  |  |  |  | 2 | 0 | 2 | 0 | 0 | 0 | 0 | 0 | 0 | 0 | 0 | 482 | 495 | Carbamidomethylation | C4:Carbamidomethylation:1000.00 | PEAKS DB |
| R.LGVYYAYC(+57.02)R.K | Y | 69.61 | 1163.5433 | 9 | 0.8 | 582.7794 | 2 | 11.92 | 2 | F2:2746 | DaRuMP\_F10.raw |  | 9.8603E5 |  |  |  |  |  |  |  |  | 1 | 0 | 1 | 0 | 0 | 0 | 0 | 0 | 0 | 0 | 0 | 537 | 545 | Carbamidomethylation | C8:Carbamidomethylation:1000.00 | PEAKS DB |
| R.DEC(+57.02)DKAEQC(+57.02)TGR.S | Y | 69.32 | 1467.5718 | 12 | -5.0 | 734.7895 | 2 | 10.68 | 2 | F2:1616 | DaRuMP\_F10.raw |  | 1.0602E7 | 2.5015E5 |  |  |  |  |  |  |  | 3 | 0 | 2 | 1 | 0 | 0 | 0 | 0 | 0 | 0 | 0 | 470 | 481 | Carbamidomethylation | C3:Carbamidomethylation:1000.00;C9:Carbamidomethylation:1000.00 | PEAKS DB |
| R.VPLVGLEIWK.N | Y | 68.51 | 1152.6907 | 10 | 0.2 | 577.3527 | 2 | 32.65 | 2 | F2:9673 | DaRuMP\_F10.raw |  | 1.2424E8 |  |  |  |  |  |  |  |  | 1 | 0 | 1 | 0 | 0 | 0 | 0 | 0 | 0 | 0 | 0 | 248 | 257 |  |  | PEAKS DB |
| Y.SYVELIITVDHSMVTK.Y | Y | 67.53 | 1833.9546 | 16 | 0.3 | 917.9849 | 2 | 27.55 | 2 | F2:8877 | DaRuMP\_F10.raw |  | 1.3957E6 |  |  |  |  |  |  |  |  | 1 | 0 | 1 | 0 | 0 | 0 | 0 | 0 | 0 | 0 | 0 | 203 | 218 |  |  | PEAKS DB |
| K.YFSNC(+57.02)SYNQYR.R | Y | 67.46 | 1500.6093 | 11 | 0.1 | 751.3120 | 2 | 11.48 | 2 | F2:2326 | DaRuMP\_F10.raw |  | 5.8794E5 |  |  |  |  |  |  |  |  | 1 | 0 | 1 | 0 | 0 | 0 | 0 | 0 | 0 | 0 | 0 | 374 | 384 | Carbamidomethylation | C5:Carbamidomethylation:1000.00 | PEAKS DB |
| R.TDIVSPPAC(+57.02)GN.E | Y | 67.18 | 1129.5073 | 11 | -0.1 | 565.7609 | 2 | 11.87 | 2 | F2:2633 | DaRuMP\_F10.raw |  | 1.2131E6 |  |  |  |  |  |  |  |  | 1 | 0 | 1 | 0 | 0 | 0 | 0 | 0 | 0 | 0 | 0 | 402 | 412 | Carbamidomethylation | C9:Carbamidomethylation:1000.00 | PEAKS DB |
| Y.SC(+57.02)IMSAVLGDQPSK.Y | Y | 66.92 | 1491.7062 | 14 | 0.6 | 746.8608 | 2 | 14.26 | 2 | F2:4000 | DaRuMP\_F10.raw |  | 3.1209E6 |  |  |  |  |  |  |  |  | 1 | 0 | 1 | 0 | 0 | 0 | 0 | 0 | 0 | 0 | 0 | 360 | 373 | Carbamidomethylation | C2:Carbamidomethylation:1000.00 | PEAKS DB |
| R.SANC(+57.02)PVDEFHENGRPC(+57.02).L | Y | 66.13 | 1887.7628 | 16 | 0.5 | 944.8892 | 2 | 11.28 | 2 | F2:2142 | DaRuMP\_F10.raw |  | 8.2776E4 |  |  |  |  |  |  |  |  | 1 | 0 | 1 | 0 | 0 | 0 | 0 | 0 | 0 | 0 | 0 | 482 | 497 | Carbamidomethylation | C4:Carbamidomethylation:1000.00;C16:Carbamidomethylation:1000.00 | PEAKS DB |
| C.LHNFGYC(+57.02)YNGK.C | Y | 65.77 | 1371.6030 | 11 | 1.0 | 686.8094 | 2 | 11.28 | 2 | F2:2139 | DaRuMP\_F10.raw |  | 3.1944E4 |  |  |  |  |  |  |  |  | 1 | 0 | 1 | 0 | 0 | 0 | 0 | 0 | 0 | 0 | 0 | 498 | 508 | Carbamidomethylation | C7:Carbamidomethylation:1000.00 | PEAKS DB |
| R.SANC(+57.02)PVDEFHENGRPC(+57.02)L.H | Y | 65.58 | 2000.8469 | 17 | -0.1 | 667.9562 | 3 | 11.71 | 2 | F2:2542 | DaRuMP\_F10.raw |  | 1.0519E5 |  |  |  |  |  |  |  |  | 1 | 0 | 1 | 0 | 0 | 0 | 0 | 0 | 0 | 0 | 0 | 482 | 498 | Carbamidomethylation | C4:Carbamidomethylation:1000.00;C16:Carbamidomethylation:1000.00 | PEAKS DB |
| K.ASLLVATSER.N | Y | 64.99 | 1045.5768 | 10 | 0.1 | 523.7957 | 2 | 11.71 | 2 | F2:2538 | DaRuMP\_F10.raw |  | 7.7502E5 |  |  |  |  |  |  |  |  | 1 | 0 | 1 | 0 | 0 | 0 | 0 | 0 | 0 | 0 | 0 | 186 | 195 |  |  | PEAKS DB |
| V.TQTNWESDEPIKK.A | N | 63.45 | 1574.7576 | 13 | -0.3 | 525.9263 | 3 | 11.22 | 2 | F2:2067 | DaRuMP\_F10.raw |  | 1.0323E5 |  |  |  |  |  |  |  |  | 1 | 0 | 1 | 0 | 0 | 0 | 0 | 0 | 0 | 0 | 0 | 173 | 185 |  |  | PEAKS DB |
| R.VPLVGLEIWKNR.D | Y | 62.55 | 1422.8347 | 12 | 0.2 | 475.2856 | 3 | 18.94 | 2 | F2:5625 | DaRuMP\_F10.raw |  | 5.2304E4 |  |  |  |  |  |  |  |  | 2 | 0 | 2 | 0 | 0 | 0 | 0 | 0 | 0 | 0 | 0 | 248 | 259 |  |  | PEAKS DB |
| R.GEEC(+57.02)DC(+57.02)GSPENC(+57.02)RDPC(+57.02)C(+57.02)DAASC(+57.02)K.L | Y | 62.01 | 2732.9287 | 23 | -0.5 | 911.9830 | 3 | 10.80 | 2 | F2:1687 | DaRuMP\_F10.raw |  | 3.7366E6 |  |  |  |  |  |  |  |  | 1 | 0 | 1 | 0 | 0 | 0 | 0 | 0 | 0 | 0 | 0 | 418 | 440 | Carbamidomethylation | C4:Carbamidomethylation:1000.00;C6:Carbamidomethylation:1000.00;C12:Carbamidomethylation:1000.00;C16:Carbamidomethylation:1000.00;C17:Carbamidomethylation:1000.00;C22:Carbamidomethylation:1000.00 | PEAKS DB |
| C.IMSAVLGDQPSK.Y | Y | 61.79 | 1244.6434 | 12 | 0.0 | 623.3290 | 2 | 11.74 | 2 | F2:2558 | DaRuMP\_F10.raw |  | 1.6778E6 |  |  |  |  |  |  |  |  | 1 | 0 | 1 | 0 | 0 | 0 | 0 | 0 | 0 | 0 | 0 | 362 | 373 |  |  | PEAKS DB |
| R.GEEC(+57.02)DC(+57.02)GSPENC(+57.02)R.D | Y | 61.14 | 1568.5289 | 13 | -5.3 | 785.2676 | 2 | 10.68 | 2 | F2:1619 | DaRuMP\_F10.raw |  | 4.1513E6 |  |  |  |  |  |  |  |  | 1 | 0 | 1 | 0 | 0 | 0 | 0 | 0 | 0 | 0 | 0 | 418 | 430 | Carbamidomethylation | C4:Carbamidomethylation:1000.00;C6:Carbamidomethylation:1000.00;C12:Carbamidomethylation:1000.00 | PEAKS DB |
| R.SANC(+57.02)PVDEFHENGRPC(+57.02)LHNFGY.C | Y | 60.90 | 2619.1018 | 22 | 0.5 | 874.0416 | 3 | 11.85 | 2 | F2:2673 | DaRuMP\_F10.raw |  | 5.7948E5 |  |  |  |  |  |  |  |  | 1 | 0 | 1 | 0 | 0 | 0 | 0 | 0 | 0 | 0 | 0 | 482 | 503 | Carbamidomethylation | C4:Carbamidomethylation:1000.00;C16:Carbamidomethylation:1000.00 | PEAKS DB |
| D.IVSPPAC(+57.02)GNELLER.G | Y | 60.19 | 1553.7871 | 14 | 0.8 | 777.9015 | 2 | 12.09 | 2 | F2:2854 | DaRuMP\_F10.raw |  | 2.584E5 |  |  |  |  |  |  |  |  | 1 | 0 | 1 | 0 | 0 | 0 | 0 | 0 | 0 | 0 | 0 | 404 | 417 | Carbamidomethylation | C7:Carbamidomethylation:1000.00 | PEAKS DB |
| F.SEDYSETHYSPDGR.E | N | 58.50 | 1641.6543 | 14 | -0.2 | 548.2253 | 3 | 10.98 | 1 | F1:1869 | DaRuMP\_F1.raw | 8.8827E4 |  |  |  |  |  |  |  |  |  | 1 | 1 | 0 | 0 | 0 | 0 | 0 | 0 | 0 | 0 | 0 | 81 | 94 |  |  | PEAKS DB |
| R.VAVTMAHEIGHNLGLTHDGVY.C | Y | 57.99 | 2233.0950 | 21 | 0.5 | 559.2813 | 4 | 12.18 | 2 | F2:2939 | DaRuMP\_F10.raw |  | 5.4469E5 |  |  |  |  |  |  |  |  | 1 | 0 | 1 | 0 | 0 | 0 | 0 | 0 | 0 | 0 | 0 | 333 | 353 |  |  | PEAKS DB |
| K.LHSWVEC(+57.02)ESGKC(+57.02)C(+57.02)NQC(+57.02)R.F | Y | 57.80 | 2208.8921 | 17 | 0.1 | 553.2303 | 4 | 10.94 | 2 | F2:1806 | DaRuMP\_F10.raw |  | 8.3735E4 |  |  |  |  |  |  |  |  | 1 | 0 | 1 | 0 | 0 | 0 | 0 | 0 | 0 | 0 | 0 | 441 | 457 | Carbamidomethylation | C7:Carbamidomethylation:1000.00;C12:Carbamidomethylation:1000.00;C13:Carbamidomethylation:1000.00;C16:Carbamidomethylation:1000.00 | PEAKS DB |
| K.VC(+57.02)SNGQC(+57.02)VDLNIAY | Y | 57.73 | 1611.7021 | 14 | 0.4 | 806.8586 | 2 | 20.37 | 2 | F2:6176 | DaRuMP\_F10.raw |  | 1.0186E7 |  |  |  |  |  |  |  |  | 1 | 0 | 1 | 0 | 0 | 0 | 0 | 0 | 0 | 0 | 0 | 602 | 615 | Carbamidomethylation | C2:Carbamidomethylation:1000.00;C7:Carbamidomethylation:1000.00 | PEAKS DB |
| R.VAVTMAHEIGHNLGL.T | Y | 56.09 | 1560.8082 | 15 | 0.1 | 521.2767 | 3 | 13.07 | 2 | F2:3472 | DaRuMP\_F10.raw |  | 1.5932E5 |  |  |  |  |  |  |  |  | 1 | 0 | 1 | 0 | 0 | 0 | 0 | 0 | 0 | 0 | 0 | 333 | 347 |  |  | PEAKS DB |
| R.VAVTMAHEIGHNLGLTHDGVYC(+57.02)TC(+57.02).G | Y | 55.53 | 2654.2039 | 24 | 0.6 | 885.7424 | 3 | 12.11 | 2 | F2:2894 | DaRuMP\_F10.raw |  | 1.8236E5 |  |  |  |  |  |  |  |  | 1 | 0 | 1 | 0 | 0 | 0 | 0 | 0 | 0 | 0 | 0 | 333 | 356 | Carbamidomethylation | C22:Carbamidomethylation:1000.00;C24:Carbamidomethylation:1000.00 | PEAKS DB |
| R.SANC(+57.02)PVDEFHEN.G | Y | 55.05 | 1417.5568 | 12 | 0.7 | 709.7861 | 2 | 11.46 | 2 | F2:2311 | DaRuMP\_F10.raw |  | 2.4239E5 |  |  |  |  |  |  |  |  | 1 | 0 | 1 | 0 | 0 | 0 | 0 | 0 | 0 | 0 | 0 | 482 | 493 | Carbamidomethylation | C4:Carbamidomethylation:1000.00 | PEAKS DB |
| C.LPYYIPSDENK.G | Y | 54.83 | 1337.6503 | 11 | 0.3 | 669.8326 | 2 | 12.14 | 2 | F2:2913 | DaRuMP\_F10.raw |  | 6.012E5 |  |  |  |  |  |  |  |  | 1 | 0 | 1 | 0 | 0 | 0 | 0 | 0 | 0 | 0 | 0 | 578 | 588 |  |  | PEAKS DB |
| L.HSWVEC(+57.02)ESGK.C | Y | 54.47 | 1217.5135 | 10 | 0.0 | 609.7640 | 2 | 10.81 | 3 | F3:1682 | DaRuMP\_F2\_F3.raw |  |  | 1.1462E4 |  |  |  |  |  |  |  | 1 | 0 | 0 | 1 | 0 | 0 | 0 | 0 | 0 | 0 | 0 | 442 | 451 | Carbamidomethylation | C6:Carbamidomethylation:1000.00 | PEAKS DB |
| K.YSVGVVQDH.S | Y | 54.30 | 1002.4771 | 9 | -0.3 | 502.2457 | 2 | 11.46 | 2 | F2:2327 | DaRuMP\_F10.raw |  | 1.5168E5 |  |  |  |  |  |  |  |  | 1 | 0 | 1 | 0 | 0 | 0 | 0 | 0 | 0 | 0 | 0 | 318 | 326 |  |  | PEAKS DB |
| N.PEC(+57.02)IINPPLR.T | Y | 53.80 | 1207.6383 | 10 | -0.4 | 604.8262 | 2 | 12.18 | 2 | F2:2943 | DaRuMP\_F10.raw |  | 6.4328E4 |  |  |  |  |  |  |  |  | 1 | 0 | 1 | 0 | 0 | 0 | 0 | 0 | 0 | 0 | 0 | 392 | 401 | Carbamidomethylation | C3:Carbamidomethylation:1000.00 | PEAKS DB |
| R.SANC(+57.02)PVDEFHENGRPC(+57.02)LH.N | Y | 52.61 | 2137.9058 | 18 | 0.7 | 535.4841 | 4 | 11.32 | 2 | F2:2182 | DaRuMP\_F10.raw |  | 1.7825E5 |  |  |  |  |  |  |  |  | 1 | 0 | 1 | 0 | 0 | 0 | 0 | 0 | 0 | 0 | 0 | 482 | 499 | Carbamidomethylation | C4:Carbamidomethylation:1000.00;C16:Carbamidomethylation:1000.00 | PEAKS DB |
| total 45 peptides |
| --- |

P84674|PA2B5\_DABRR

back to list

  

| Protein Coverage
| Supporting Peptides
|

Protein Coverage:

Supporting Peptides:

| Peptide | Uniq | -10lgP | Mass | Length | ppm | m/z | z | RT | Fraction | Scan | Source File | Area F1 | Area F10 | Area F2\_3 | Area F5 | Area F6 | Area F7A | Area F7B | Area F8A | Area F8B | Area F9 | #Feature | #Feature F1 | #Feature F10 | #Feature F2\_3 | #Feature F5 | #Feature F6 | #Feature F7A | #Feature F7B | #Feature F8A | #Feature F8B | #Feature F9 | Start | End | PTM | AScore | Found By |
| --- | --- | --- | --- | --- | --- | --- | --- | --- | --- | --- | --- | --- | --- | --- | --- | --- | --- | --- | --- | --- | --- | --- | --- | --- | --- | --- | --- | --- | --- | --- | --- | --- | --- | --- | --- | --- | --- |
| R.VNGAIVC(+57.02)EQGTSC(+57.02)ENR.I | N | 112.07 | 1792.7832 | 16 | -9.1 | 897.3907 | 2 | 15.49 | 8 | F8:2314 | DaRuMP\_F8A.raw |  |  |  |  | 6.4795E5 | 2.662E7 | 1.3092E6 | 4.1633E8 | 2.4228E4 | 1.7554E6 | 14 | 0 | 0 | 0 | 0 | 1 | 3 | 1 | 6 | 1 | 2 | 69 | 84 | Carbamidomethylation | C7:Carbamidomethylation:1000.00;C13:Carbamidomethylation:1000.00 | PEAKS DB |
| K.LAVPFYSSYGC(+57.02)YC(+57.02)GWGGK.G | N | 103.36 | 2070.8967 | 18 | -1.2 | 1036.4543 | 2 | 30.18 | 8 | F8:12713 | DaRuMP\_F8A.raw |  |  |  |  |  |  |  | 4.2764E8 |  |  | 3 | 0 | 0 | 0 | 0 | 0 | 0 | 0 | 3 | 0 | 0 | 16 | 33 | Carbamidomethylation | C11:Carbamidomethylation:1000.00;C13:Carbamidomethylation:1000.00 | PEAKS DB |
| K.LAVPFYSSYGC(+57.02)YC(+57.02)GWGGKGTPK.D | Y | 97.10 | 2454.1135 | 22 | 0.8 | 819.0458 | 3 | 20.80 | 8 | F8:6539 | DaRuMP\_F8A.raw |  |  |  |  |  |  |  | 2.3768E6 |  |  | 1 | 0 | 0 | 0 | 0 | 0 | 0 | 0 | 1 | 0 | 0 | 16 | 37 | Carbamidomethylation | C11:Carbamidomethylation:1000.00;C13:Carbamidomethylation:1000.00 | PEAKS DB |
| SLLEFGM(+15.99)MILEETGK.L | N | 96.55 | 1712.8364 | 15 | 0.6 | 857.4260 | 2 | 53.14 | 8 | F8:23593 | DaRuMP\_F8A.raw |  |  |  |  |  |  |  | 2.8084E7 |  | 3.2977E5 | 5 | 0 | 0 | 0 | 0 | 0 | 0 | 0 | 4 | 0 | 1 | 1 | 15 | Oxidation (M) | M7:Oxidation (M):26.02 | PEAKS DB |
| SLLEFGMM(+15.99)ILEETGK.L | N | 91.71 | 1712.8364 | 15 | 0.2 | 857.4257 | 2 | 60.23 | 8 | F8:26218 | DaRuMP\_F8A.raw |  |  |  |  |  |  |  | 1.5322E7 |  | 3.2977E5 | 4 | 0 | 0 | 0 | 0 | 0 | 0 | 0 | 3 | 0 | 1 | 1 | 15 | Oxidation (M) | M8:Oxidation (M):40.00 | PEAKS DB |
| K.RVNGAIVC(+57.02)EQGTSC(+57.02)ENR.I | N | 91.41 | 1948.8843 | 17 | -8.1 | 975.4415 | 2 | 15.49 | 8 | F8:2300 | DaRuMP\_F8A.raw |  |  |  |  |  | 5.318E5 |  | 1.1178E7 |  | 2.4029E5 | 4 | 0 | 0 | 0 | 0 | 0 | 2 | 0 | 1 | 0 | 1 | 68 | 84 | Carbamidomethylation | C8:Carbamidomethylation:1000.00;C14:Carbamidomethylation:1000.00 | PEAKS DB |
| SLLEFGMMILEETGK.L | N | 87.31 | 1696.8416 | 15 | 0.5 | 849.4285 | 2 | 66.01 | 8 | F8:27922 | DaRuMP\_F8A.raw |  |  |  |  |  |  |  | 2.2161E7 |  |  | 2 | 0 | 0 | 0 | 0 | 0 | 0 | 0 | 2 | 0 | 0 | 1 | 15 |  |  | PEAKS DB |
| SLLEFGM(+15.99)M(+15.99)ILEETGK.L | N | 86.23 | 1728.8314 | 15 | 0.1 | 865.4231 | 2 | 45.65 | 8 | F8:20926 | DaRuMP\_F8A.raw |  |  |  |  |  |  |  | 1.0085E7 |  | 7.4736E5 | 8 | 0 | 0 | 0 | 0 | 0 | 0 | 0 | 6 | 0 | 2 | 1 | 15 | Oxidation (M) | M7:Oxidation (M):1000.00;M8:Oxidation (M):1000.00 | PEAKS DB |
| K.IYMLYPDFLC(+57.02)K.G | N | 81.94 | 1461.7036 | 11 | 0.9 | 731.8597 | 2 | 35.78 | 8 | F8:15918 | DaRuMP\_F8A.raw |  |  |  |  |  |  |  | 6.9733E8 |  | 1.5491E6 | 10 | 0 | 0 | 0 | 0 | 0 | 0 | 0 | 9 | 0 | 1 | 106 | 116 | Carbamidomethylation | C10:Carbamidomethylation:1000.00 | PEAKS DB |
| R.VNGAIVC(+57.02)EQGTSC(+57.02)ENRIC(+57.02)EC(+57.02)DK.A | N | 80.69 | 2598.0930 | 22 | 2.6 | 867.0385 | 3 | 11.30 | 6 | F6:2134 | DaRuMP\_F7A.raw |  |  |  |  |  | 2.3203E5 |  |  |  |  | 1 | 0 | 0 | 0 | 0 | 0 | 1 | 0 | 0 | 0 | 0 | 69 | 90 | Carbamidomethylation | C7:Carbamidomethylation:1000.00;C13:Carbamidomethylation:1000.00;C18:Carbamidomethylation:1000.00;C20:Carbamidomethylation:1000.00 | PEAKS DB |
| K.IYM(+15.99)LYPDFLC(+57.02)K.G | N | 79.86 | 1477.6985 | 11 | 0.6 | 739.8569 | 2 | 35.27 | 8 | F8:17625 | DaRuMP\_F8A.raw |  |  |  |  |  |  |  | 1.6878E8 |  | 1.8039E6 | 6 | 0 | 0 | 0 | 0 | 0 | 0 | 0 | 4 | 0 | 2 | 106 | 116 | Oxidation (M); Carbamidomethylation | M3:Oxidation (M):1000.00;C10:Carbamidomethylation:1000.00 | PEAKS DB |
| N.GAIVC(+57.02)EQGTSC(+57.02)ENR.I | N | 79.00 | 1579.6719 | 14 | -8.2 | 790.8367 | 2 | 15.49 | 8 | F8:2301 | DaRuMP\_F8A.raw |  |  |  |  | 1.7032E5 |  | 3.5504E5 | 1.6512E6 |  | 5.094E4 | 4 | 0 | 0 | 0 | 0 | 1 | 0 | 1 | 1 | 0 | 1 | 71 | 84 | Carbamidomethylation | C5:Carbamidomethylation:1000.00;C11:Carbamidomethylation:1000.00 | PEAKS DB |
| I.YMLYPDFLC(+57.02)KGELK.C | N | 77.16 | 1775.8625 | 14 | 2.8 | 592.9617 | 3 | 21.57 | 6 | F6:10344 | DaRuMP\_F7A.raw |  |  |  |  |  | 3.328E6 |  |  |  |  | 2 | 0 | 0 | 0 | 0 | 0 | 2 | 0 | 0 | 0 | 0 | 107 | 120 | Carbamidomethylation | C9:Carbamidomethylation:1000.00 | PEAKS DB |
| K.IYMLYPDFLC(+57.02)KGELK.C | N | 76.28 | 1888.9467 | 15 | -0.4 | 630.6559 | 3 | 29.27 | 8 | F8:11875 | DaRuMP\_F8A.raw |  |  |  |  |  |  |  | 6.0575E6 |  |  | 1 | 0 | 0 | 0 | 0 | 0 | 0 | 0 | 1 | 0 | 0 | 106 | 120 | Carbamidomethylation | C10:Carbamidomethylation:1000.00 | PEAKS DB |
| R.VNGAIVC(+57.02)EQGTSC(+57.02)ENRI.C | N | 75.51 | 1905.8673 | 17 | -5.2 | 953.9360 | 2 | 15.56 | 8 | F8:2361 | DaRuMP\_F8A.raw |  |  |  |  |  |  |  | 1.5204E5 |  |  | 1 | 0 | 0 | 0 | 0 | 0 | 0 | 0 | 1 | 0 | 0 | 69 | 85 | Carbamidomethylation | C7:Carbamidomethylation:1000.00;C13:Carbamidomethylation:1000.00 | PEAKS DB |
| I.YM(+15.99)LYPDFLC(+57.02)K.G | N | 73.83 | 1364.6145 | 10 | 2.7 | 683.3147 | 2 | 24.65 | 6 | F6:13883 | DaRuMP\_F7A.raw |  |  |  |  | 5.5321E5 | 5.3341E7 |  |  |  |  | 5 | 0 | 0 | 0 | 0 | 1 | 4 | 0 | 0 | 0 | 0 | 107 | 116 | Oxidation (M); Carbamidomethylation | M2:Oxidation (M):1000.00;C9:Carbamidomethylation:1000.00 | PEAKS DB |
| G.AIVC(+57.02)EQGTSC(+57.02)ENR.I | N | 73.19 | 1522.6504 | 13 | 0.4 | 762.3328 | 2 | 10.89 | 1 | F1:1784 | DaRuMP\_F1.raw | 1.1385E6 |  |  |  | 4.2735E4 |  | 6.204E4 |  |  |  | 3 | 1 | 0 | 0 | 0 | 1 | 0 | 1 | 0 | 0 | 0 | 72 | 84 | Carbamidomethylation | C4:Carbamidomethylation:1000.00;C10:Carbamidomethylation:1000.00 | PEAKS DB |
| Y.SSYGC(+57.02)YC(+57.02)GWGGK.G | N | 71.73 | 1380.5227 | 12 | 2.9 | 691.2690 | 2 | 11.64 | 6 | F6:2416 | DaRuMP\_F7A.raw |  |  |  |  |  | 7.7516E6 | 7.2278E5 | 2.165E6 |  |  | 3 | 0 | 0 | 0 | 0 | 0 | 1 | 1 | 1 | 0 | 0 | 22 | 33 | Carbamidomethylation | C5:Carbamidomethylation:1000.00;C7:Carbamidomethylation:1000.00 | PEAKS DB |
| S.SYGC(+57.02)YC(+57.02)GWGGK.G | N | 71.11 | 1293.4907 | 11 | 2.9 | 647.7529 | 2 | 11.61 | 6 | F6:2413 | DaRuMP\_F7A.raw | 3.6812E5 |  |  |  |  | 1.7045E6 | 5.493E5 | 4.5706E5 |  |  | 4 | 1 | 0 | 0 | 0 | 0 | 1 | 1 | 1 | 0 | 0 | 23 | 33 | Carbamidomethylation | C4:Carbamidomethylation:1000.00;C6:Carbamidomethylation:1000.00 | PEAKS DB |
| I.YMLYPDFLC(+57.02)K.G | N | 69.49 | 1348.6195 | 10 | 2.9 | 675.3173 | 2 | 29.67 | 6 | F6:15617 | DaRuMP\_F7A.raw |  |  |  |  |  | 3.1666E8 |  | 4.8942E5 |  |  | 5 | 0 | 0 | 0 | 0 | 0 | 4 | 0 | 1 | 0 | 0 | 107 | 116 | Carbamidomethylation | C9:Carbamidomethylation:1000.00 | PEAKS DB |
| I.YMLYPDFLC(+57.02)KGELKC(+57.02) | N | 66.36 | 1935.8933 | 15 | 3.3 | 646.3056 | 3 | 22.67 | 6 | F6:11114 | DaRuMP\_F7A.raw |  |  |  |  |  | 2.893E6 |  |  |  |  | 1 | 0 | 0 | 0 | 0 | 0 | 1 | 0 | 0 | 0 | 0 | 107 | 121 | Carbamidomethylation | C9:Carbamidomethylation:1000.00;C15:Carbamidomethylation:1000.00 | PEAKS DB |
| V.NGAIVC(+57.02)EQGTSC(+57.02)ENR.I | N | 66.07 | 1693.7148 | 15 | 0.7 | 847.8653 | 2 | 11.59 | 1 | F1:2441 | DaRuMP\_F1.raw | 1.0878E5 |  |  |  |  |  |  |  |  |  | 1 | 1 | 0 | 0 | 0 | 0 | 0 | 0 | 0 | 0 | 0 | 70 | 84 | Carbamidomethylation | C6:Carbamidomethylation:1000.00;C12:Carbamidomethylation:1000.00 | PEAKS DB |
| F.YSSYGC(+57.02)YC(+57.02)GWGGK.G | N | 65.06 | 1543.5861 | 13 | -2.2 | 772.7986 | 2 | 15.78 | 8 | F8:2544 | DaRuMP\_F8A.raw |  |  |  |  |  | 1.7203E7 |  | 4.8744E8 |  | 1.8379E6 | 3 | 0 | 0 | 0 | 0 | 0 | 1 | 0 | 1 | 0 | 1 | 21 | 33 | Carbamidomethylation | C6:Carbamidomethylation:1000.00;C8:Carbamidomethylation:1000.00 | PEAKS DB |
| Y.GC(+57.02)YC(+57.02)GWGGK.G | N | 64.20 | 1043.3953 | 9 | 3.2 | 522.7053 | 2 | 11.29 | 6 | F6:2116 | DaRuMP\_F7A.raw |  |  | 6.3696E4 |  | 1.7244E6 | 2.9315E6 | 3.3122E5 |  |  | 4.6531E4 | 5 | 0 | 0 | 1 | 0 | 1 | 1 | 1 | 0 | 0 | 1 | 25 | 33 | Carbamidomethylation | C2:Carbamidomethylation:1000.00;C4:Carbamidomethylation:1000.00 | PEAKS DB |
| K.IYMLYPDFLC(+57.02)KG.E | N | 64.08 | 1518.7251 | 12 | 0.3 | 760.3701 | 2 | 36.91 | 8 | F8:16676 | DaRuMP\_F8A.raw |  |  |  |  |  |  |  | 2.2208E7 |  |  | 1 | 0 | 0 | 0 | 0 | 0 | 0 | 0 | 1 | 0 | 0 | 106 | 117 | Carbamidomethylation | C10:Carbamidomethylation:1000.00 | PEAKS DB |
| G.KLAVPFYSSYGC(+57.02)YC(+57.02)GWGGK.G | N | 63.92 | 2198.9917 | 19 | 0.8 | 734.0051 | 3 | 20.69 | 8 | F8:6459 | DaRuMP\_F8A.raw |  |  |  |  |  |  |  | 1.4071E5 |  |  | 1 | 0 | 0 | 0 | 0 | 0 | 0 | 0 | 1 | 0 | 0 | 15 | 33 | Carbamidomethylation | C12:Carbamidomethylation:1000.00;C14:Carbamidomethylation:1000.00 | PEAKS DB |
| K.LAVPFYSSYGC(+57.02)YC(+57.02)GWGGKG.T | Y | 63.06 | 2127.9182 | 19 | 0.0 | 1064.9664 | 2 | 29.87 | 8 | F8:12225 | DaRuMP\_F8A.raw |  |  |  |  |  |  |  | 6.8825E6 |  |  | 1 | 0 | 0 | 0 | 0 | 0 | 0 | 0 | 1 | 0 | 0 | 16 | 34 | Carbamidomethylation | C11:Carbamidomethylation:1000.00;C13:Carbamidomethylation:1000.00 | PEAKS DB |
| P.FYSSYGC(+57.02)YC(+57.02)GWGGK.G | N | 60.24 | 1690.6544 | 14 | 1.0 | 846.3353 | 2 | 16.53 | 8 | F8:3231 | DaRuMP\_F8A.raw |  |  |  |  |  |  |  | 7.1685E4 |  |  | 1 | 0 | 0 | 0 | 0 | 0 | 0 | 0 | 1 | 0 | 0 | 20 | 33 | Carbamidomethylation | C7:Carbamidomethylation:1000.00;C9:Carbamidomethylation:1000.00 | PEAKS DB |
| K.IYM(+15.99)LYPDFLC(+57.02)KG.E | N | 59.35 | 1534.7200 | 12 | 0.8 | 768.3679 | 2 | 28.04 | 8 | F8:11781 | DaRuMP\_F8A.raw |  |  |  |  |  |  |  | 5.8595E6 |  |  | 1 | 0 | 0 | 0 | 0 | 0 | 0 | 0 | 1 | 0 | 0 | 106 | 117 | Oxidation (M); Carbamidomethylation | M3:Oxidation (M):1000.00;C10:Carbamidomethylation:1000.00 | PEAKS DB |
| S.YGC(+57.02)YC(+57.02)GWGGK.G | N | 58.24 | 1206.4586 | 10 | 0.0 | 604.2366 | 2 | 11.59 | 5 | F5:2425 | DaRuMP\_F6.raw |  |  |  |  | 1.3442E7 |  |  |  |  |  | 1 | 0 | 0 | 0 | 0 | 1 | 0 | 0 | 0 | 0 | 0 | 24 | 33 | Carbamidomethylation | C3:Carbamidomethylation:1000.00;C5:Carbamidomethylation:1000.00 | PEAKS DB |
| F.GMMILEETGK.L | N | 55.74 | 1107.5304 | 10 | 0.0 | 554.7725 | 2 | 16.49 | 8 | F8:3169 | DaRuMP\_F8A.raw |  |  |  |  |  |  |  | 1.5241E6 |  |  | 1 | 0 | 0 | 0 | 0 | 0 | 0 | 0 | 1 | 0 | 0 | 6 | 15 |  |  | PEAKS DB |
| Y.MLYPDFLC(+57.02)K.G | N | 54.73 | 1185.5563 | 9 | 0.4 | 593.7856 | 2 | 24.23 | 8 | F8:8823 | DaRuMP\_F8A.raw |  |  |  |  |  |  |  | 5.9068E6 |  |  | 1 | 0 | 0 | 0 | 0 | 0 | 0 | 0 | 1 | 0 | 0 | 108 | 116 | Carbamidomethylation | C8:Carbamidomethylation:1000.00 | PEAKS DB |
| M.MILEETGK.L | N | 54.26 | 919.4684 | 8 | -0.1 | 460.7415 | 2 | 11.49 | 7 | F7:2297 | DaRuMP\_F7B.raw |  |  |  |  |  |  | 4.4177E5 | 1.1565E6 |  |  | 2 | 0 | 0 | 0 | 0 | 0 | 0 | 1 | 1 | 0 | 0 | 8 | 15 |  |  | PEAKS DB |
| A.IVC(+57.02)EQGTSC(+57.02)ENR.I | N | 52.07 | 1451.6133 | 12 | 1.7 | 726.8152 | 2 | 15.50 | 8 | F8:2299 | DaRuMP\_F8A.raw |  |  |  |  |  |  |  | 0 |  |  | 0 | 0 | 0 | 0 | 0 | 0 | 0 | 0 | 0 | 0 | 0 | 73 | 84 | Carbamidomethylation | C3:Carbamidomethylation:1000.00;C9:Carbamidomethylation:1000.00 | PEAKS DB |
| K.IYMLYPDFLC(+57.02)KGELKC(+57.02) | N | 52.05 | 2048.9773 | 16 | 0.4 | 684.0000 | 3 | 30.31 | 8 | F8:12508 | DaRuMP\_F8A.raw |  |  |  |  |  |  |  | 2.9166E6 |  |  | 1 | 0 | 0 | 0 | 0 | 0 | 0 | 0 | 1 | 0 | 0 | 106 | 121 | Carbamidomethylation | C10:Carbamidomethylation:1000.00;C16:Carbamidomethylation:1000.00 | PEAKS DB |
| total 35 peptides |
| --- |

A8CG86|PA2A1\_DABRR

back to list

  

| Protein Coverage
| Supporting Peptides
|

Protein Coverage:

Supporting Peptides:

| Peptide | Uniq | -10lgP | Mass | Length | ppm | m/z | z | RT | Fraction | Scan | Source File | Area F1 | Area F10 | Area F2\_3 | Area F5 | Area F6 | Area F7A | Area F7B | Area F8A | Area F8B | Area F9 | #Feature | #Feature F1 | #Feature F10 | #Feature F2\_3 | #Feature F5 | #Feature F6 | #Feature F7A | #Feature F7B | #Feature F8A | #Feature F8B | #Feature F9 | Start | End | PTM | AScore | Found By |
| --- | --- | --- | --- | --- | --- | --- | --- | --- | --- | --- | --- | --- | --- | --- | --- | --- | --- | --- | --- | --- | --- | --- | --- | --- | --- | --- | --- | --- | --- | --- | --- | --- | --- | --- | --- | --- | --- |
| Y.AIYGC(+57.02)YC(+57.02)GWGGQGKPQDATDR.C | Y | 109.13 | 2359.0110 | 21 | -1.7 | 1180.5107 | 2 | 11.73 | 7 | F7:2538 | DaRuMP\_F7B.raw |  |  |  |  |  |  | 6.2869E7 |  | 4.2611E5 |  | 3 | 0 | 0 | 0 | 0 | 0 | 0 | 2 | 0 | 1 | 0 | 38 | 58 | Carbamidomethylation | C5:Carbamidomethylation:1000.00;C7:Carbamidomethylation:1000.00 | PEAKS DB |
| R.AAAIC(+57.02)LGQNVNTYDK.N | N | 107.30 | 1636.7878 | 15 | 0.5 | 819.4016 | 2 | 15.81 | 8 | F8:2590 | DaRuMP\_F8A.raw |  |  |  | 3.1809E4 |  |  | 4.2432E6 | 1.2492E6 | 7.5459E6 | 5.5577E4 | 11 | 0 | 0 | 0 | 1 | 0 | 0 | 7 | 1 | 1 | 1 | 107 | 121 | Carbamidomethylation | C5:Carbamidomethylation:1000.00 | PEAKS DB |
| K.EAVHSYAIYGC(+57.02)YC(+57.02)GWGGQGKPQDATDR.C | Y | 101.37 | 3045.3132 | 27 | -2.6 | 1016.1090 | 3 | 11.86 | 7 | F7:2667 | DaRuMP\_F7B.raw |  |  |  |  |  |  | 1.0888E7 |  |  |  | 2 | 0 | 0 | 0 | 0 | 0 | 0 | 2 | 0 | 0 | 0 | 32 | 58 | Carbamidomethylation | C11:Carbamidomethylation:1000.00;C13:Carbamidomethylation:1000.00 | PEAKS DB |
| K.EAVHSYAIYGC(+57.02)YC(+57.02)GWGGQGK.P | Y | 92.10 | 2261.9622 | 20 | 1.6 | 754.9958 | 3 | 12.17 | 7 | F7:2971 | DaRuMP\_F7B.raw |  |  |  |  |  |  | 5.8126E6 |  |  |  | 2 | 0 | 0 | 0 | 0 | 0 | 0 | 2 | 0 | 0 | 0 | 32 | 51 | Carbamidomethylation | C11:Carbamidomethylation:1000.00;C13:Carbamidomethylation:1000.00 | PEAKS DB |
| Y.SYSFENGDIVC(+57.02)GDNNLC(+57.02)LK.T | Y | 86.98 | 2203.9514 | 19 | 0.4 | 1102.9834 | 2 | 17.45 | 7 | F7:7483 | DaRuMP\_F7B.raw |  |  |  |  |  |  | 1.6691E8 |  |  |  | 2 | 0 | 0 | 0 | 0 | 0 | 0 | 2 | 0 | 0 | 0 | 81 | 99 | Carbamidomethylation | C11:Carbamidomethylation:1000.00;C17:Carbamidomethylation:1000.00 | PEAKS DB |
| R.C(+57.02)C(+57.02)FVHDC(+57.02)C(+57.02)YGTVNDC(+57.02)NPK.M | N | 85.50 | 2304.8479 | 18 | -4.5 | 1153.4260 | 2 | 11.35 | 7 | F7:2154 | DaRuMP\_F7B.raw |  |  |  |  |  |  | 2.4107E8 |  | 2.0528E6 |  | 4 | 0 | 0 | 0 | 0 | 0 | 0 | 2 | 0 | 2 | 0 | 59 | 76 | Carbamidomethylation | C1:Carbamidomethylation:1000.00;C2:Carbamidomethylation:1000.00;C7:Carbamidomethylation:1000.00;C8:Carbamidomethylation:1000.00;C15:Carbamidomethylation:1000.00 | PEAKS DB |
| A.IC(+57.02)LGQNVNTYDK.N | N | 75.69 | 1423.6765 | 12 | 1.1 | 712.8463 | 2 | 11.67 | 7 | F7:2480 | DaRuMP\_F7B.raw |  |  |  |  |  |  | 2.3217E6 |  |  |  | 1 | 0 | 0 | 0 | 0 | 0 | 0 | 1 | 0 | 0 | 0 | 110 | 121 | Carbamidomethylation | C2:Carbamidomethylation:1000.00 | PEAKS DB |
| F.ENGDIVC(+57.02)GDNNLC(+57.02)LK.T | Y | 74.73 | 1719.7556 | 15 | 0.2 | 860.8853 | 2 | 12.08 | 7 | F7:2874 | DaRuMP\_F7B.raw |  |  |  |  |  |  | 3.2611E5 |  |  |  | 1 | 0 | 0 | 0 | 0 | 0 | 0 | 1 | 0 | 0 | 0 | 85 | 99 | Carbamidomethylation | C7:Carbamidomethylation:1000.00;C13:Carbamidomethylation:1000.00 | PEAKS DB |
| Y.GC(+57.02)YC(+57.02)GWGGQGKPQDATDR.C | N | 74.59 | 2011.8264 | 18 | 0.4 | 671.6163 | 3 | 11.15 | 7 | F7:1976 | DaRuMP\_F7B.raw |  |  |  |  |  |  | 1.2594E6 |  |  |  | 2 | 0 | 0 | 0 | 0 | 0 | 0 | 2 | 0 | 0 | 0 | 41 | 58 | Carbamidomethylation | C2:Carbamidomethylation:1000.00;C4:Carbamidomethylation:1000.00 | PEAKS DB |
| F.VHDC(+57.02)C(+57.02)YGTVNDC(+57.02)NPK.M | N | 74.37 | 1837.7181 | 15 | 0.5 | 919.8668 | 2 | 10.77 | 7 | F7:1624 | DaRuMP\_F7B.raw |  |  |  |  |  |  | 3.0234E5 |  |  |  | 1 | 0 | 0 | 0 | 0 | 0 | 0 | 1 | 0 | 0 | 0 | 62 | 76 | Carbamidomethylation | C4:Carbamidomethylation:1000.00;C5:Carbamidomethylation:1000.00;C12:Carbamidomethylation:1000.00 | PEAKS DB |
| C.YC(+57.02)GWGGQGKPQDATDR.C | N | 74.18 | 1794.7743 | 16 | 0.8 | 898.3951 | 2 | 11.06 | 7 | F7:1911 | DaRuMP\_F7B.raw |  |  |  |  |  |  | 4.1947E6 |  |  |  | 2 | 0 | 0 | 0 | 0 | 0 | 0 | 2 | 0 | 0 | 0 | 43 | 58 | Carbamidomethylation | C2:Carbamidomethylation:1000.00 | PEAKS DB |
| C.LGQNVNTYDK.N | N | 72.81 | 1150.5619 | 10 | 0.1 | 576.2883 | 2 | 10.97 | 7 | F7:1808 | DaRuMP\_F7B.raw |  |  |  |  |  |  | 2.3797E6 |  | 2.684E4 |  | 2 | 0 | 0 | 0 | 0 | 0 | 0 | 1 | 0 | 1 | 0 | 112 | 121 |  |  | PEAKS DB |
| K.NYENYAISHC(+57.02)TEESEQC(+57.02) | Y | 71.83 | 2132.8052 | 17 | -0.1 | 1067.4098 | 2 | 11.54 | 9 | F9:2385 | DaRuMP\_F8B.raw |  |  |  |  |  |  | 2.6296E8 |  | 3.8463E6 |  | 2 | 0 | 0 | 0 | 0 | 0 | 0 | 1 | 0 | 1 | 0 | 122 | 138 | Carbamidomethylation | C10:Carbamidomethylation:1000.00;C17:Carbamidomethylation:1000.00 | PEAKS DB |
| Y.C(+57.02)GWGGQGKPQDATDR.C | N | 71.26 | 1631.7111 | 15 | -1.7 | 816.8615 | 2 | 10.83 | 7 | F7:1653 | DaRuMP\_F7B.raw |  |  |  |  |  |  | 1.4108E7 |  | 3.3325E5 |  | 4 | 0 | 0 | 0 | 0 | 0 | 0 | 2 | 0 | 2 | 0 | 44 | 58 | Carbamidomethylation | C1:Carbamidomethylation:1000.00 | PEAKS DB |
| Y.SFENGDIVC(+57.02)GDNNLC(+57.02)LK.T | Y | 69.79 | 1953.8561 | 17 | 0.7 | 977.9360 | 2 | 14.48 | 7 | F7:4890 | DaRuMP\_F7B.raw |  |  |  |  |  |  | 7.0221E7 | 7.6226E4 |  |  | 2 | 0 | 0 | 0 | 0 | 0 | 0 | 1 | 1 | 0 | 0 | 83 | 99 | Carbamidomethylation | C9:Carbamidomethylation:1000.00;C15:Carbamidomethylation:1000.00 | PEAKS DB |
| E.GNLFQFAEMIVK.M | Y | 68.44 | 1395.7220 | 12 | 0.0 | 698.8683 | 2 | 57.20 | 7 | F7:27016 | DaRuMP\_F7B.raw |  |  |  |  |  | 7.5329E6 | 4.9502E7 |  |  |  | 2 | 0 | 0 | 0 | 0 | 0 | 1 | 1 | 0 | 0 | 0 | 16 | 27 |  |  | PEAKS DB |
| G.NLFQFAEM(+15.99)IVK.M | N | 68.34 | 1354.6954 | 11 | 0.7 | 678.3555 | 2 | 39.86 | 7 | F7:20408 | DaRuMP\_F7B.raw |  |  |  |  | 5.4462E5 | 9.9642E5 | 4.936E7 |  | 6.8164E5 |  | 7 | 0 | 0 | 0 | 0 | 1 | 2 | 3 | 0 | 1 | 0 | 17 | 27 | Oxidation (M) | M8:Oxidation (M):1000.00 | PEAKS DB |
| G.NLFQFAEMIVK.M | N | 68.31 | 1338.7006 | 11 | 0.3 | 670.3577 | 2 | 48.04 | 9 | F9:16002 | DaRuMP\_F8B.raw |  |  |  |  | 2.0517E7 | 1.6603E7 | 2.2778E8 |  | 4.1784E6 |  | 6 | 0 | 0 | 0 | 0 | 1 | 1 | 3 | 0 | 1 | 0 | 17 | 27 |  |  | PEAKS DB |
| D.C(+57.02)C(+57.02)YGTVNDC(+57.02)NPK.M | N | 67.90 | 1486.5640 | 12 | 0.1 | 744.2893 | 2 | 10.93 | 7 | F7:1780 | DaRuMP\_F7B.raw |  |  |  |  |  |  | 2.6177E5 |  |  |  | 1 | 0 | 0 | 0 | 0 | 0 | 0 | 1 | 0 | 0 | 0 | 65 | 76 | Carbamidomethylation | C1:Carbamidomethylation:1000.00;C2:Carbamidomethylation:1000.00;C9:Carbamidomethylation:1000.00 | PEAKS DB |
| A.IYGC(+57.02)YC(+57.02)GWGGQGKPQDATDR.C | Y | 65.69 | 2287.9739 | 20 | -0.7 | 763.6647 | 3 | 11.61 | 7 | F7:2420 | DaRuMP\_F7B.raw |  |  |  |  |  |  | 3.4151E5 |  |  |  | 1 | 0 | 0 | 0 | 0 | 0 | 0 | 1 | 0 | 0 | 0 | 39 | 58 | Carbamidomethylation | C4:Carbamidomethylation:1000.00;C6:Carbamidomethylation:1000.00 | PEAKS DB |
| C.FVHDC(+57.02)C(+57.02)YGTVNDC(+57.02)NPK.M | N | 64.69 | 1984.7866 | 16 | 0.4 | 662.6031 | 3 | 11.09 | 7 | F7:1920 | DaRuMP\_F7B.raw |  |  |  |  |  |  | 8.4449E5 |  |  |  | 2 | 0 | 0 | 0 | 0 | 0 | 0 | 2 | 0 | 0 | 0 | 61 | 76 | Carbamidomethylation | C5:Carbamidomethylation:1000.00;C6:Carbamidomethylation:1000.00;C13:Carbamidomethylation:1000.00 | PEAKS DB |
| K.M(+15.99)ATYSYSFENGDIVC(+57.02)GDNNLC(+57.02)LK.T | Y | 64.44 | 2686.1350 | 23 | 0.1 | 1344.0750 | 2 | 20.70 | 7 | F7:10097 | DaRuMP\_F7B.raw |  |  |  |  |  |  | 9.4327E6 |  |  |  | 1 | 0 | 0 | 0 | 0 | 0 | 0 | 1 | 0 | 0 | 0 | 77 | 99 | Oxidation (M); Carbamidomethylation | M1:Oxidation (M):1000.00;C15:Carbamidomethylation:1000.00;C21:Carbamidomethylation:1000.00 | PEAKS DB |
| C.C(+57.02)YGTVNDC(+57.02)NPK.M | N | 61.83 | 1326.5333 | 11 | -2.4 | 664.2723 | 2 | 10.80 | 7 | F7:1661 | DaRuMP\_F7B.raw |  |  |  |  |  |  | 5.2926E4 |  |  |  | 1 | 0 | 0 | 0 | 0 | 0 | 0 | 1 | 0 | 0 | 0 | 66 | 76 | Carbamidomethylation | C1:Carbamidomethylation:1000.00;C8:Carbamidomethylation:1000.00 | PEAKS DB |
| D.IVC(+57.02)GDNNLC(+57.02)LK.T | Y | 61.69 | 1304.6217 | 11 | 1.1 | 653.3188 | 2 | 11.65 | 7 | F7:2457 | DaRuMP\_F7B.raw |  |  |  |  |  |  | 5.8764E5 |  |  |  | 1 | 0 | 0 | 0 | 0 | 0 | 0 | 1 | 0 | 0 | 0 | 89 | 99 | Carbamidomethylation | C3:Carbamidomethylation:1000.00;C9:Carbamidomethylation:1000.00 | PEAKS DB |
| E.GNLFQFAEM(+15.99)IVK.M | Y | 60.39 | 1411.7169 | 12 | 0.2 | 706.8658 | 2 | 49.29 | 7 | F7:24397 | DaRuMP\_F7B.raw |  |  |  |  |  |  | 6.6472E6 |  |  |  | 2 | 0 | 0 | 0 | 0 | 0 | 0 | 2 | 0 | 0 | 0 | 16 | 27 | Oxidation (M) | M9:Oxidation (M):1000.00 | PEAKS DB |
| K.EAVHSYAIYGC(+57.02)Y.C | N | 59.86 | 1431.6129 | 12 | -1.6 | 716.8126 | 2 | 12.47 | 7 | F7:3181 | DaRuMP\_F7B.raw |  |  |  |  |  |  | 9.7658E7 |  |  |  | 1 | 0 | 0 | 0 | 0 | 0 | 0 | 1 | 0 | 0 | 0 | 32 | 43 | Carbamidomethylation | C11:Carbamidomethylation:1000.00 | PEAKS DB |
| H.DC(+57.02)C(+57.02)YGTVNDC(+57.02)NPK.M | N | 56.84 | 1601.5908 | 13 | 0.9 | 801.8034 | 2 | 11.09 | 7 | F7:1923 | DaRuMP\_F7B.raw |  |  |  |  |  |  | 1.757E5 |  |  |  | 1 | 0 | 0 | 0 | 0 | 0 | 0 | 1 | 0 | 0 | 0 | 64 | 76 | Carbamidomethylation | C2:Carbamidomethylation:1000.00;C3:Carbamidomethylation:1000.00;C10:Carbamidomethylation:1000.00 | PEAKS DB |
| R.AAAIC(+57.02)LGQNVN.T | N | 56.67 | 1129.5549 | 11 | 0.7 | 565.7852 | 2 | 12.72 | 7 | F7:3394 | DaRuMP\_F7B.raw |  |  |  |  |  |  | 1.2993E7 |  |  |  | 1 | 0 | 0 | 0 | 0 | 0 | 0 | 1 | 0 | 0 | 0 | 107 | 117 | Carbamidomethylation | C5:Carbamidomethylation:1000.00 | PEAKS DB |
| K.EAVHSYAIYGC(+57.02).Y | N | 54.88 | 1268.5496 | 11 | -1.4 | 635.2812 | 2 | 11.86 | 7 | F7:2668 | DaRuMP\_F7B.raw |  |  |  |  |  |  | 2.17E6 |  |  |  | 1 | 0 | 0 | 0 | 0 | 0 | 0 | 1 | 0 | 0 | 0 | 32 | 42 | Carbamidomethylation | C11:Carbamidomethylation:1000.00 | PEAKS DB |
| N.YENYAISHC(+57.02)TEESEQC(+57.02) | Y | 53.62 | 2018.7622 | 16 | 0.4 | 1010.3888 | 2 | 11.40 | 7 | F7:2203 | DaRuMP\_F7B.raw |  |  |  |  |  |  | 1.4419E6 |  |  |  | 1 | 0 | 0 | 0 | 0 | 0 | 0 | 1 | 0 | 0 | 0 | 123 | 138 | Carbamidomethylation | C9:Carbamidomethylation:1000.00;C16:Carbamidomethylation:1000.00 | PEAKS DB |
| E.NGDIVC(+57.02)GDNNLC(+57.02)LK.T | Y | 53.35 | 1590.7130 | 14 | 1.8 | 796.3652 | 2 | 16.20 | 7 | F7:6451 | DaRuMP\_F7B.raw |  |  |  |  |  |  | 7.8054E4 |  |  |  | 1 | 0 | 0 | 0 | 0 | 0 | 0 | 1 | 0 | 0 | 0 | 86 | 99 | Carbamidomethylation | C6:Carbamidomethylation:1000.00;C12:Carbamidomethylation:1000.00 | PEAKS DB |
| N.GDIVC(+57.02)GDNNLC(+57.02)LK.T | Y | 52.51 | 1476.6700 | 13 | -0.2 | 739.3422 | 2 | 11.99 | 7 | F7:2806 | DaRuMP\_F7B.raw |  |  |  |  |  |  | 8.2472E5 |  |  |  | 1 | 0 | 0 | 0 | 0 | 0 | 0 | 1 | 0 | 0 | 0 | 87 | 99 | Carbamidomethylation | C5:Carbamidomethylation:1000.00;C11:Carbamidomethylation:1000.00 | PEAKS DB |
| K.NYENYAISH.C | Y | 52.40 | 1109.4778 | 9 | 1.0 | 555.7467 | 2 | 11.46 | 7 | F7:2268 | DaRuMP\_F7B.raw |  |  |  |  |  |  | 5.9414E6 |  |  |  | 1 | 0 | 0 | 0 | 0 | 0 | 0 | 1 | 0 | 0 | 0 | 122 | 130 |  |  | PEAKS DB |
| R.AAAIC(+57.02)LGQNVNTYDKN.Y | N | 52.23 | 1750.8308 | 16 | -4.3 | 876.4189 | 2 | 11.91 | 7 | F7:2705 | DaRuMP\_F7B.raw |  |  |  |  |  |  | 3.5566E7 |  |  |  | 1 | 0 | 0 | 0 | 0 | 0 | 0 | 1 | 0 | 0 | 0 | 107 | 122 | Carbamidomethylation | C5:Carbamidomethylation:1000.00 | PEAKS DB |
| total 34 peptides |
| --- |

G8XQX1|OXLA\_DABRR

back to list

  

| Protein Coverage
| Supporting Peptides
|

Protein Coverage:

Supporting Peptides:

| Peptide | Uniq | -10lgP | Mass | Length | ppm | m/z | z | RT | Fraction | Scan | Source File | Area F1 | Area F10 | Area F2\_3 | Area F5 | Area F6 | Area F7A | Area F7B | Area F8A | Area F8B | Area F9 | #Feature | #Feature F1 | #Feature F10 | #Feature F2\_3 | #Feature F5 | #Feature F6 | #Feature F7A | #Feature F7B | #Feature F8A | #Feature F8B | #Feature F9 | Start | End | PTM | AScore | Found By |
| --- | --- | --- | --- | --- | --- | --- | --- | --- | --- | --- | --- | --- | --- | --- | --- | --- | --- | --- | --- | --- | --- | --- | --- | --- | --- | --- | --- | --- | --- | --- | --- | --- | --- | --- | --- | --- | --- |
| K.NLLLETADYVIVC(+57.02)TTSR.A | Y | 101.39 | 1967.0034 | 17 | 0.0 | 984.5090 | 2 | 48.34 | 8 | F8:21853 | DaRuMP\_F8A.raw |  | 2.4366E5 |  |  |  |  | 5.6955E4 | 1.3841E7 | 1.6233E5 | 1.1409E6 | 9 | 0 | 1 | 0 | 0 | 0 | 0 | 2 | 3 | 1 | 2 | 299 | 315 | Carbamidomethylation | C13:Carbamidomethylation:1000.00 | PEAKS DB |
| K.HIVIVGAGMSGLSAAYVLAGAGHK.V | Y | 98.34 | 2278.2256 | 24 | 0.7 | 760.4163 | 3 | 22.57 | 8 | F8:7791 | DaRuMP\_F8A.raw |  |  |  |  |  |  |  | 5.9179E6 |  |  | 2 | 0 | 0 | 0 | 0 | 0 | 0 | 0 | 2 | 0 | 0 | 53 | 76 |  |  | PEAKS DB |
| K.LNEFVQETENGWYFIK.N | N | 92.59 | 2015.9629 | 16 | 0.4 | 1008.9891 | 2 | 35.58 | 8 | F8:15823 | DaRuMP\_F8A.raw |  |  |  |  |  |  |  | 1.7621E7 |  | 1.5632E6 | 2 | 0 | 0 | 0 | 0 | 0 | 0 | 0 | 1 | 0 | 1 | 127 | 142 |  |  | PEAKS DB |
| F.TPYQFQHFSEALTAPVGR.I | N | 92.07 | 2048.0115 | 18 | 0.7 | 683.6782 | 3 | 17.61 | 8 | F8:4206 | DaRuMP\_F8A.raw |  |  |  |  |  |  |  | 2.4882E6 |  | 1.5444E5 | 2 | 0 | 0 | 0 | 0 | 0 | 0 | 0 | 1 | 0 | 1 | 452 | 469 |  |  | PEAKS DB |
| Y.QFQHFSEALTAPVGR.I | N | 91.13 | 1686.8478 | 15 | -0.2 | 844.4310 | 2 | 15.86 | 8 | F8:2636 | DaRuMP\_F8A.raw |  |  |  |  |  |  |  | 7.6172E6 |  | 2.1969E5 | 3 | 0 | 0 | 0 | 0 | 0 | 0 | 0 | 2 | 0 | 1 | 455 | 469 |  |  | PEAKS DB |
| C.ADDKNPLEEC(+57.02)FR.E | N | 89.94 | 1492.6616 | 12 | -1.6 | 498.5604 | 3 | 15.69 | 8 | F8:2491 | DaRuMP\_F8A.raw |  |  | 1.1227E5 | 8.0172E5 |  |  |  | 1.2218E6 |  | 1.4778E5 | 6 | 0 | 0 | 1 | 2 | 0 | 0 | 0 | 2 | 0 | 1 | 19 | 30 | Carbamidomethylation | C10:Carbamidomethylation:1000.00 | PEAKS DB |
| K.HIVIVGAGM(+15.99)SGLSAAYVLAGAGHK.V | Y | 87.74 | 2294.2205 | 24 | 0.0 | 574.5624 | 4 | 17.12 | 8 | F8:3722 | DaRuMP\_F8A.raw |  |  |  |  |  |  |  | 2.4166E5 |  |  | 1 | 0 | 0 | 0 | 0 | 0 | 0 | 0 | 1 | 0 | 0 | 53 | 76 | Oxidation (M) | M9:Oxidation (M):1000.00 | PEAKS DB |
| K.KDLQTFC(+57.02)YPSIIQK.W | N | 86.78 | 1739.8916 | 14 | 1.5 | 870.9543 | 2 | 16.41 | 8 | F8:3113 | DaRuMP\_F8A.raw |  | 6.9924E4 |  |  |  |  |  | 2.8253E6 |  |  | 3 | 0 | 1 | 0 | 0 | 0 | 0 | 0 | 2 | 0 | 0 | 424 | 437 | Carbamidomethylation | C7:Carbamidomethylation:1000.00 | PEAKS DB |
| K.SAGQLYQESLGK.A | N | 85.18 | 1279.6407 | 12 | -5.1 | 640.8244 | 2 | 15.54 | 8 | F8:2339 | DaRuMP\_F8A.raw |  | 1.6374E5 |  | 1.0755E6 | 2.4507E5 |  |  | 2.009E6 | 5.1718E4 | 6.2426E5 | 6 | 0 | 1 | 0 | 1 | 1 | 0 | 0 | 1 | 1 | 1 | 170 | 181 |  |  | PEAKS DB |
| K.DLQTFC(+57.02)YPSIIQK.W | N | 84.36 | 1611.7966 | 13 | 0.8 | 806.9062 | 2 | 25.51 | 8 | F8:9569 | DaRuMP\_F8A.raw |  |  |  |  | 6.4484E4 |  |  | 6.2384E6 |  | 5.9524E5 | 3 | 0 | 0 | 0 | 0 | 1 | 0 | 0 | 1 | 0 | 1 | 425 | 437 | Carbamidomethylation | C6:Carbamidomethylation:1000.00 | PEAKS DB |
| K.HDDIFAYEK.R | N | 84.29 | 1136.5138 | 9 | -6.0 | 569.2607 | 2 | 15.56 | 8 | F8:2357 | DaRuMP\_F8A.raw |  | 6.5548E4 |  |  |  |  |  | 1.0794E6 |  | 4.6555E5 | 3 | 0 | 1 | 0 | 0 | 0 | 0 | 0 | 1 | 0 | 1 | 241 | 249 |  |  | PEAKS DB |
| R.EDDYEEFLEIAK.N | N | 80.40 | 1499.6667 | 12 | -0.8 | 750.8400 | 2 | 30.08 | 8 | F8:12407 | DaRuMP\_F8A.raw |  |  |  | 1.7264E6 | 6.1612E5 |  | 1.5316E5 | 9.9291E6 |  |  | 4 | 0 | 0 | 0 | 1 | 1 | 0 | 1 | 1 | 0 | 0 | 31 | 42 |  |  | PEAKS DB |
| K.NPLEEC(+57.02)FR.E | N | 79.95 | 1063.4757 | 8 | -1.1 | 532.7446 | 2 | 15.77 | 8 | F8:2554 | DaRuMP\_F8A.raw |  |  |  | 1.4943E5 |  |  |  | 0 |  | 3.5066E4 | 2 | 0 | 0 | 0 | 1 | 0 | 0 | 0 | 0 | 0 | 1 | 23 | 30 | Carbamidomethylation | C6:Carbamidomethylation:1000.00 | PEAKS DB |
| K.FWEDDGIQGGK.S | N | 76.18 | 1250.5568 | 11 | 0.6 | 626.2860 | 2 | 15.90 | 8 | F8:2659 | DaRuMP\_F8A.raw |  |  |  |  |  |  |  | 3.0992E6 |  | 4.0109E5 | 2 | 0 | 0 | 0 | 0 | 0 | 0 | 0 | 1 | 0 | 1 | 353 | 363 |  |  | PEAKS DB |
| K.EGWYANLGPMR.V | N | 75.54 | 1292.5972 | 11 | -1.2 | 647.3051 | 2 | 19.59 | 8 | F8:5648 | DaRuMP\_F8A.raw |  |  |  |  |  |  |  | 1.6289E6 |  |  | 1 | 0 | 0 | 0 | 0 | 0 | 0 | 0 | 1 | 0 | 0 | 98 | 108 |  |  | PEAKS DB |
| R.IFFAGEYTANAHGWIDSTIK.S | N | 72.97 | 2240.0901 | 20 | 1.1 | 747.7048 | 3 | 25.51 | 8 | F8:9752 | DaRuMP\_F8A.raw |  |  |  |  |  |  |  | 3.4932E6 |  |  | 1 | 0 | 0 | 0 | 0 | 0 | 0 | 0 | 1 | 0 | 0 | 470 | 489 |  |  | PEAKS DB |
| Y.TANAHGWIDSTIK.S | N | 71.18 | 1412.7048 | 13 | 0.1 | 707.3597 | 2 | 11.57 | 4 | F4:2431 | DaRuMP\_F5.raw |  |  |  | 2.144E5 |  |  |  |  |  |  | 1 | 0 | 0 | 0 | 1 | 0 | 0 | 0 | 0 | 0 | 0 | 477 | 489 |  |  | PEAKS DB |
| K.VTVTYQTTQK.N | N | 68.81 | 1167.6135 | 10 | -0.6 | 584.8137 | 2 | 11.05 | 2 | F2:1924 | DaRuMP\_F10.raw |  | 4.3249E4 |  |  |  |  |  |  |  | 3.5672E5 | 2 | 0 | 1 | 0 | 0 | 0 | 0 | 0 | 0 | 0 | 1 | 289 | 298 |  |  | PEAKS DB |
| H.FSEALTAPVGR.I | N | 66.80 | 1146.6033 | 11 | -0.4 | 574.3087 | 2 | 15.81 | 8 | F8:2617 | DaRuMP\_F8A.raw |  |  |  |  |  |  |  | 5.5732E5 |  | 3.961E4 | 2 | 0 | 0 | 0 | 0 | 0 | 0 | 0 | 1 | 0 | 1 | 459 | 469 |  |  | PEAKS DB |
| L.SAAYVLAGAGHK.V | Y | 65.92 | 1143.6036 | 12 | 0.9 | 572.8096 | 2 | 11.14 | 10 | F10:1903 | DaRuMP\_F9.raw |  |  |  |  |  |  |  |  |  | 2.6575E4 | 2 | 0 | 0 | 0 | 0 | 0 | 0 | 0 | 0 | 0 | 2 | 65 | 76 |  |  | PEAKS DB |
| R.ITFKPPLPPK.K | N | 65.51 | 1136.6957 | 10 | 0.2 | 569.3552 | 2 | 15.71 | 8 | F8:2528 | DaRuMP\_F8A.raw |  |  |  |  |  |  |  | 1.439E6 |  |  | 2 | 0 | 0 | 0 | 0 | 0 | 0 | 0 | 2 | 0 | 0 | 320 | 329 |  |  | PEAKS DB |
| K.EGWYANLGPM(+15.99)R.V | N | 64.37 | 1308.5920 | 11 | 1.0 | 655.3040 | 2 | 16.55 | 8 | F8:3244 | DaRuMP\_F8A.raw |  |  |  |  |  |  |  | 2.9764E5 |  |  | 1 | 0 | 0 | 0 | 0 | 0 | 0 | 0 | 1 | 0 | 0 | 98 | 108 | Oxidation (M) | M10:Oxidation (M):1000.00 | PEAKS DB |
| L.NLNEC(+57.02)ADIVFNDLSSIHQLPK.K | N | 63.85 | 2426.1899 | 21 | -0.5 | 809.7369 | 3 | 32.47 | 8 | F8:14251 | DaRuMP\_F8A.raw |  |  |  |  |  |  |  | 7.8479E6 |  |  | 1 | 0 | 0 | 0 | 0 | 0 | 0 | 0 | 1 | 0 | 0 | 403 | 423 | Carbamidomethylation | C5:Carbamidomethylation:1000.00 | PEAKS DB |
| N.AHGWIDSTIK.S | N | 61.24 | 1126.5771 | 10 | 1.0 | 564.2964 | 2 | 11.55 | 10 | F10:2294 | DaRuMP\_F9.raw |  |  |  |  |  |  |  |  |  | 3.545E4 | 1 | 0 | 0 | 0 | 0 | 0 | 0 | 0 | 0 | 0 | 1 | 480 | 489 |  |  | PEAKS DB |
| R.FDEIVGGMDQLPTSMYR.A | N | 60.90 | 1957.8914 | 17 | 0.2 | 979.9531 | 2 | 28.07 | 10 | F10:6312 | DaRuMP\_F9.raw |  |  |  |  |  |  |  |  |  | 5.604E4 | 1 | 0 | 0 | 0 | 0 | 0 | 0 | 0 | 0 | 0 | 1 | 251 | 267 |  |  | PEAKS DB |
| C.ADDKNPLEEC(+57.02)FREDDYEEFLEIAK.N | N | 60.77 | 2974.3176 | 24 | 1.1 | 744.5875 | 4 | 32.16 | 8 | F8:13665 | DaRuMP\_F8A.raw |  |  |  |  |  |  |  | 1.0108E7 |  |  | 1 | 0 | 0 | 0 | 0 | 0 | 0 | 0 | 1 | 0 | 0 | 19 | 42 | Carbamidomethylation | C10:Carbamidomethylation:1000.00 | PEAKS DB |
| K.RFDEIVGGMDQLPTSMYR.A | N | 59.57 | 2113.9924 | 18 | 1.3 | 705.6724 | 3 | 23.09 | 8 | F8:8155 | DaRuMP\_F8A.raw |  |  |  |  |  |  |  | 7.0864E4 |  |  | 1 | 0 | 0 | 0 | 0 | 0 | 0 | 0 | 1 | 0 | 0 | 250 | 267 |  |  | PEAKS DB |
| K.YPVKPSEAGK.S | N | 59.51 | 1074.5709 | 10 | 0.0 | 538.2927 | 2 | 10.71 | 10 | F10:1602 | DaRuMP\_F9.raw |  |  |  |  |  |  |  |  |  | 2.7264E5 | 1 | 0 | 0 | 0 | 0 | 0 | 0 | 0 | 0 | 0 | 1 | 160 | 169 |  |  | PEAKS DB |
| R.FDEIVGGM(+15.99)DQLPTSMYR.A | N | 58.98 | 1973.8862 | 17 | -0.4 | 987.9500 | 2 | 21.41 | 8 | F8:6914 | DaRuMP\_F8A.raw |  |  |  |  |  |  |  | 1.0566E5 |  |  | 1 | 0 | 0 | 0 | 0 | 0 | 0 | 0 | 1 | 0 | 0 | 251 | 267 | Oxidation (M) | M8:Oxidation (M):29.93 | PEAKS DB |
| F.C(+57.02)YPSIIQK.W | N | 58.48 | 1007.5110 | 8 | -7.2 | 504.7592 | 2 | 15.60 | 8 | F8:2390 | DaRuMP\_F8A.raw |  |  |  |  |  |  |  | 2.9364E5 |  | 1.3613E5 | 2 | 0 | 0 | 0 | 0 | 0 | 0 | 0 | 1 | 0 | 1 | 430 | 437 | Carbamidomethylation | C1:Carbamidomethylation:1000.00 | PEAKS DB |
| K.VTVLEASER.P | Y | 58.11 | 1002.5345 | 9 | 0.6 | 502.2748 | 2 | 11.41 | 10 | F10:2162 | DaRuMP\_F9.raw |  |  |  |  |  |  |  |  |  | 1.562E5 | 1 | 0 | 0 | 0 | 0 | 0 | 0 | 0 | 0 | 0 | 1 | 77 | 85 |  |  | PEAKS DB |
| M.SGLSAAYVLAGAGHK.V | Y | 54.61 | 1400.7412 | 15 | -0.1 | 467.9210 | 3 | 15.88 | 8 | F8:2682 | DaRuMP\_F8A.raw |  |  |  |  |  |  |  | 9.7734E4 |  |  | 1 | 0 | 0 | 0 | 0 | 0 | 0 | 0 | 1 | 0 | 0 | 62 | 76 |  |  | PEAKS DB |
| R.AIEESVHFK.A | Y | 54.21 | 1058.5397 | 9 | -8.3 | 530.2727 | 2 | 15.49 | 8 | F8:2324 | DaRuMP\_F8A.raw |  |  |  |  |  |  |  | 2.462E6 |  |  | 1 | 0 | 0 | 0 | 0 | 0 | 0 | 0 | 1 | 0 | 0 | 268 | 276 |  |  | PEAKS DB |
| R.IFFAGEYTANAH.G | N | 52.38 | 1339.6196 | 12 | 0.3 | 670.8173 | 2 | 17.47 | 8 | F8:3958 | DaRuMP\_F8A.raw |  |  |  |  |  |  |  | 1.2445E6 |  |  | 1 | 0 | 0 | 0 | 0 | 0 | 0 | 0 | 1 | 0 | 0 | 470 | 481 |  |  | PEAKS DB |
| total 34 peptides |
| --- |

P04264|K2C1\_HUMAN

back to list

  

| Protein Coverage
| Supporting Peptides
|

Protein Coverage:

Supporting Peptides:

| Peptide | Uniq | -10lgP | Mass | Length | ppm | m/z | z | RT | Fraction | Scan | Source File | Area F1 | Area F10 | Area F2\_3 | Area F5 | Area F6 | Area F7A | Area F7B | Area F8A | Area F8B | Area F9 | #Feature | #Feature F1 | #Feature F10 | #Feature F2\_3 | #Feature F5 | #Feature F6 | #Feature F7A | #Feature F7B | #Feature F8A | #Feature F8B | #Feature F9 | Start | End | PTM | AScore | Found By |
| --- | --- | --- | --- | --- | --- | --- | --- | --- | --- | --- | --- | --- | --- | --- | --- | --- | --- | --- | --- | --- | --- | --- | --- | --- | --- | --- | --- | --- | --- | --- | --- | --- | --- | --- | --- | --- | --- |
| R.FSSC(+57.02)GGGGGSFGAGGGFGSR.S | Y | 104.48 | 1764.7274 | 20 | 1.1 | 883.3719 | 2 | 11.76 | 9 | F9:2607 | DaRuMP\_F8B.raw | 1.2524E5 | 2.6265E5 |  | 1.0085E6 | 9.9634E5 |  | 2.5247E6 | 5.0633E5 | 6.8056E5 | 7.9853E4 | 8 | 1 | 1 | 0 | 1 | 1 | 0 | 1 | 1 | 1 | 1 | 46 | 65 | Carbamidomethylation | C4:Carbamidomethylation:1000.00 | PEAKS DB |
| R.THNLEPYFESFINNLR.R | Y | 94.92 | 1992.9694 | 16 | 0.6 | 665.3308 | 3 | 34.24 | 7 | F7:17826 | DaRuMP\_F7B.raw |  | 1.0547E6 |  | 2.1623E6 | 1.9947E6 | 1.3906E5 | 6.7864E6 | 2.5985E6 | 1.3055E6 | 4.1382E5 | 10 | 0 | 1 | 0 | 1 | 1 | 1 | 2 | 2 | 1 | 1 | 224 | 239 |  |  | PEAKS DB |
| R.GGGGGGYGSGGSSYGSGGGSYGSGGGGGGGR.G | Y | 92.19 | 2382.9446 | 31 | 0.1 | 1192.4797 | 2 | 11.00 | 7 | F7:1827 | DaRuMP\_F7B.raw | 1.059E5 | 6.023E4 | 4.3622E4 | 2.9796E5 | 1.5875E5 |  | 4.6702E5 |  |  |  | 6 | 1 | 1 | 1 | 1 | 1 | 0 | 1 | 0 | 0 | 0 | 519 | 549 |  |  | PEAKS DB |
| K.QISNLQQSISDAEQR.G | Y | 85.27 | 1715.8438 | 15 | -1.1 | 858.9282 | 2 | 11.92 | 5 | F5:2767 | DaRuMP\_F6.raw | 1.9619E5 | 1.7937E5 |  | 6.7235E5 | 5.7628E5 |  | 1.0861E6 |  | 1.2037E5 |  | 6 | 1 | 1 | 0 | 1 | 1 | 0 | 1 | 0 | 1 | 0 | 418 | 432 |  |  | PEAKS DB |
| K.LNDLEDALQQAK.E | Y | 81.52 | 1356.6885 | 12 | -2.7 | 679.3497 | 2 | 16.83 | 8 | F8:3459 | DaRuMP\_F8A.raw | 2.6415E6 | 8.4795E5 | 5.2109E5 | 1.9116E6 | 2.7262E6 |  | 6.8018E6 | 2.0426E6 | 1.7792E6 | 7.1255E5 | 9 | 1 | 1 | 1 | 1 | 1 | 0 | 1 | 1 | 1 | 1 | 444 | 455 |  |  | PEAKS DB |
| R.FLEQQNQVLQTK.W | N | 81.01 | 1474.7780 | 12 | 0.8 | 738.3969 | 2 | 11.70 | 3 | F3:2504 | DaRuMP\_F2\_F3.raw | 2.5845E6 | 6.3219E5 | 7.403E5 | 2.7387E6 | 2.4581E6 |  | 6.9952E6 | 1.1298E6 | 1.6497E6 | 4.0389E5 | 9 | 1 | 1 | 1 | 1 | 1 | 0 | 1 | 1 | 1 | 1 | 200 | 211 |  |  | PEAKS DB |
| R.TNAENEFVTIK.K | Y | 78.52 | 1264.6299 | 11 | 0.0 | 633.3222 | 2 | 15.81 | 8 | F8:2616 | DaRuMP\_F8A.raw | 1.64E6 | 2.8553E5 | 2.3723E5 | 1.8554E6 | 1.3015E6 |  | 3.1822E6 | 7.0873E5 |  | 2.0119E5 | 8 | 1 | 1 | 1 | 1 | 1 | 0 | 1 | 1 | 0 | 1 | 278 | 288 |  |  | PEAKS DB |
| K.SLNNQFASFIDK.V | Y | 78.51 | 1382.6830 | 12 | 0.3 | 692.3490 | 2 | 20.63 | 8 | F8:6361 | DaRuMP\_F8A.raw |  |  | 2.2646E5 | 2.1242E6 | 2.4771E6 |  | 6.0942E6 | 2.2912E6 | 1.4201E6 |  | 6 | 0 | 0 | 1 | 1 | 1 | 0 | 1 | 1 | 1 | 0 | 186 | 197 |  |  | PEAKS DB |
| R.SGGGFSSGSAGIINYQR.R | Y | 76.56 | 1656.7855 | 17 | -2.4 | 829.3981 | 2 | 11.97 | 5 | F5:2814 | DaRuMP\_F6.raw |  |  |  | 1.0897E5 | 1.807E5 |  | 1.7368E5 |  | 9.033E4 | 6.1752E4 | 5 | 0 | 0 | 0 | 1 | 1 | 0 | 1 | 0 | 1 | 1 | 13 | 29 |  |  | PEAKS DB |
| K.LALDLEIATYR.T | N | 74.71 | 1276.7026 | 11 | 1.0 | 639.3593 | 2 | 24.69 | 7 | F7:13531 | DaRuMP\_F7B.raw |  |  |  | 7.5779E5 | 8.0796E5 |  | 1.3667E6 | 9.5474E5 | 1.0533E6 | 4.3021E5 | 6 | 0 | 0 | 0 | 1 | 1 | 0 | 1 | 1 | 1 | 1 | 473 | 483 |  |  | PEAKS DB |
| K.YEELQITAGR.H | N | 70.13 | 1178.5931 | 10 | 0.7 | 590.3043 | 2 | 11.78 | 3 | F3:2585 | DaRuMP\_F2\_F3.raw | 1.0838E6 | 1.0203E5 | 3.5577E5 | 1.1108E6 | 9.1708E5 |  | 2.1181E6 | 9.1018E5 | 0 |  | 7 | 1 | 1 | 1 | 1 | 1 | 0 | 1 | 1 | 0 | 0 | 377 | 386 |  |  | PEAKS DB |
| K.AQYEDIAQK.S | N | 68.82 | 1064.5138 | 9 | 0.7 | 533.2645 | 2 | 11.06 | 9 | F9:1926 | DaRuMP\_F8B.raw | 2.8739E5 | 7.1096E4 |  |  | 2.5101E5 |  | 3.2729E5 |  | 1.4028E5 | 1.0019E5 | 6 | 1 | 1 | 0 | 0 | 1 | 0 | 1 | 0 | 1 | 1 | 356 | 364 |  |  | PEAKS DB |
| K.WELLQQVDTSTR.T | Y | 67.72 | 1474.7416 | 12 | 0.1 | 738.3782 | 2 | 17.63 | 3 | F3:4746 | DaRuMP\_F2\_F3.raw |  |  | 1.6161E4 |  |  |  | 7.2292E5 |  | 2.0193E5 |  | 3 | 0 | 0 | 1 | 0 | 0 | 0 | 1 | 0 | 1 | 0 | 212 | 223 |  |  | PEAKS DB |
| K.AEAESLYQSK.Y | Y | 67.04 | 1124.5349 | 10 | 0.0 | 563.2747 | 2 | 11.11 | 7 | F7:1954 | DaRuMP\_F7B.raw | 1.1083E5 |  | 5.433E4 | 1.9018E5 | 1.7405E5 |  | 1.9384E5 |  | 7.5619E4 | 7.0837E4 | 7 | 1 | 0 | 1 | 1 | 1 | 0 | 1 | 0 | 1 | 1 | 367 | 376 |  |  | PEAKS DB |
| K.SKAEAESLYQSK.Y | Y | 64.86 | 1339.6619 | 12 | 0.4 | 670.8385 | 2 | 10.79 | 7 | F7:1659 | DaRuMP\_F7B.raw | 2.0951E4 |  |  |  |  |  | 2.2013E4 |  |  |  | 2 | 1 | 0 | 0 | 0 | 0 | 0 | 1 | 0 | 0 | 0 | 365 | 376 |  |  | PEAKS DB |
| R.SLDLDSIIAEVK.A | N | 62.01 | 1301.7078 | 12 | 0.6 | 651.8616 | 2 | 37.21 | 7 | F7:19223 | DaRuMP\_F7B.raw |  | 1.3779E6 |  | 3.8084E6 | 3.5548E6 |  | 7.363E6 | 3.8684E6 | 2.9241E6 | 1.025E6 | 7 | 0 | 1 | 0 | 1 | 1 | 0 | 1 | 1 | 1 | 1 | 344 | 355 |  |  | PEAKS DB |
| K.SKAEAESLYQSKYEELQITAGR.H | Y | 61.91 | 2500.2444 | 22 | 0.5 | 626.0687 | 4 | 11.80 | 4 | F4:2669 | DaRuMP\_F5.raw |  |  |  | 1.0311E5 |  |  |  |  |  |  | 1 | 0 | 0 | 0 | 1 | 0 | 0 | 0 | 0 | 0 | 0 | 365 | 386 |  |  | PEAKS DB |
| R.TNAENEFVTIKK.D | Y | 60.91 | 1392.7249 | 12 | 0.6 | 697.3701 | 2 | 11.32 | 4 | F4:2201 | DaRuMP\_F5.raw |  |  |  | 3.8359E5 |  |  |  |  |  |  | 2 | 0 | 0 | 0 | 2 | 0 | 0 | 0 | 0 | 0 | 0 | 278 | 289 |  |  | PEAKS DB |
| R.TLLEGEESR.M | Y | 59.75 | 1032.5087 | 9 | 0.4 | 517.2618 | 2 | 11.30 | 7 | F7:2126 | DaRuMP\_F7B.raw |  | 1.3666E5 |  |  | 3.7387E5 |  | 4.1979E5 |  | 1.4597E5 |  | 4 | 0 | 1 | 0 | 0 | 1 | 0 | 1 | 0 | 1 | 0 | 484 | 492 |  |  | PEAKS DB |
| R.NKYEDEINKR.T | N | 56.45 | 1307.6470 | 10 | 0.1 | 436.8896 | 3 | 10.71 | 10 | F10:1603 | DaRuMP\_F9.raw |  |  |  |  |  |  |  |  | 2.9837E5 | 1.8812E5 | 2 | 0 | 0 | 0 | 0 | 0 | 0 | 0 | 0 | 1 | 1 | 268 | 277 |  |  | PEAKS DB |
| K.SLNNQFASFIDKVR.F | Y | 55.66 | 1637.8525 | 14 | -0.1 | 546.9581 | 3 | 18.39 | 4 | F4:6482 | DaRuMP\_F5.raw |  |  |  | 1.0565E6 |  |  |  |  |  |  | 1 | 0 | 0 | 0 | 1 | 0 | 0 | 0 | 0 | 0 | 0 | 186 | 199 |  |  | PEAKS DB |
| L.NVEIDPEIQK.V | N | 53.62 | 1183.6084 | 10 | -1.5 | 592.8106 | 2 | 11.76 | 7 | F7:2561 | DaRuMP\_F7B.raw |  |  |  |  |  |  | 0 |  |  |  | 0 | 0 | 0 | 0 | 0 | 0 | 0 | 0 | 0 | 0 | 0 | 166 | 175 |  |  | PEAKS DB |
| K.NM(+15.99)QDM(+15.99)VEDYR.N | Y | 52.60 | 1331.5122 | 10 | 0.1 | 666.7634 | 2 | 11.04 | 9 | F9:1904 | DaRuMP\_F8B.raw |  |  |  |  |  |  |  |  | 4.041E4 |  | 1 | 0 | 0 | 0 | 0 | 0 | 0 | 0 | 0 | 1 | 0 | 258 | 267 | Oxidation (M) | M2:Oxidation (M):1000.00;M5:Oxidation (M):1000.00 | PEAKS DB |
| total 23 peptides |
| --- |

P31100|PA2A7\_DABSI

back to list

  

| Protein Coverage
| Supporting Peptides
|

Protein Coverage:

Supporting Peptides:

| Peptide | Uniq | -10lgP | Mass | Length | ppm | m/z | z | RT | Fraction | Scan | Source File | Area F1 | Area F10 | Area F2\_3 | Area F5 | Area F6 | Area F7A | Area F7B | Area F8A | Area F8B | Area F9 | #Feature | #Feature F1 | #Feature F10 | #Feature F2\_3 | #Feature F5 | #Feature F6 | #Feature F7A | #Feature F7B | #Feature F8A | #Feature F8B | #Feature F9 | Start | End | PTM | AScore | Found By |
| --- | --- | --- | --- | --- | --- | --- | --- | --- | --- | --- | --- | --- | --- | --- | --- | --- | --- | --- | --- | --- | --- | --- | --- | --- | --- | --- | --- | --- | --- | --- | --- | --- | --- | --- | --- | --- | --- |
| R.AAAIC(+57.02)LGQNVNTYDK.N | N | 107.30 | 1636.7878 | 15 | 0.5 | 819.4016 | 2 | 15.81 | 8 | F8:2590 | DaRuMP\_F8A.raw |  |  |  | 3.1809E4 |  |  | 4.2432E6 | 1.2492E6 | 7.5459E6 | 5.5577E4 | 11 | 0 | 0 | 0 | 1 | 0 | 0 | 7 | 1 | 1 | 1 | 107 | 121 | Carbamidomethylation | C5:Carbamidomethylation:1000.00 | PEAKS DB |
| K.EVVHSYAIYGC(+57.02)YC(+57.02)GWGGQGR.A | Y | 96.51 | 2317.9998 | 20 | 1.0 | 773.6746 | 3 | 12.54 | 7 | F7:3292 | DaRuMP\_F7B.raw |  |  |  |  |  |  | 1.7081E6 |  |  |  | 2 | 0 | 0 | 0 | 0 | 0 | 0 | 2 | 0 | 0 | 0 | 32 | 51 | Carbamidomethylation | C11:Carbamidomethylation:1000.00;C13:Carbamidomethylation:1000.00 | PEAKS DB |
| Y.AIYGC(+57.02)YC(+57.02)GWGGQGR.A | N | 86.30 | 1603.6660 | 14 | 1.3 | 802.8413 | 2 | 12.17 | 7 | F7:2932 | DaRuMP\_F7B.raw |  |  |  |  |  |  | 4.3334E6 |  |  |  | 1 | 0 | 0 | 0 | 0 | 0 | 0 | 1 | 0 | 0 | 0 | 38 | 51 | Carbamidomethylation | C5:Carbamidomethylation:1000.00;C7:Carbamidomethylation:1000.00 | PEAKS DB |
| R.C(+57.02)C(+57.02)FVHDC(+57.02)C(+57.02)YGTVNDC(+57.02)NPK.T | N | 85.50 | 2304.8479 | 18 | -4.5 | 1153.4260 | 2 | 11.35 | 7 | F7:2154 | DaRuMP\_F7B.raw |  |  |  |  |  |  | 2.4107E8 |  | 2.0528E6 |  | 4 | 0 | 0 | 0 | 0 | 0 | 0 | 2 | 0 | 2 | 0 | 59 | 76 | Carbamidomethylation | C1:Carbamidomethylation:1000.00;C2:Carbamidomethylation:1000.00;C7:Carbamidomethylation:1000.00;C8:Carbamidomethylation:1000.00;C15:Carbamidomethylation:1000.00 | PEAKS DB |
| A.IC(+57.02)LGQNVNTYDK.N | N | 75.69 | 1423.6765 | 12 | 1.1 | 712.8463 | 2 | 11.67 | 7 | F7:2480 | DaRuMP\_F7B.raw |  |  |  |  |  |  | 2.3217E6 |  |  |  | 1 | 0 | 0 | 0 | 0 | 0 | 0 | 1 | 0 | 0 | 0 | 110 | 121 | Carbamidomethylation | C2:Carbamidomethylation:1000.00 | PEAKS DB |
| F.VHDC(+57.02)C(+57.02)YGTVNDC(+57.02)NPK.T | N | 74.37 | 1837.7181 | 15 | 0.5 | 919.8668 | 2 | 10.77 | 7 | F7:1624 | DaRuMP\_F7B.raw |  |  |  |  |  |  | 3.0234E5 |  |  |  | 1 | 0 | 0 | 0 | 0 | 0 | 0 | 1 | 0 | 0 | 0 | 62 | 76 | Carbamidomethylation | C4:Carbamidomethylation:1000.00;C5:Carbamidomethylation:1000.00;C12:Carbamidomethylation:1000.00 | PEAKS DB |
| C.LGQNVNTYDK.N | N | 72.81 | 1150.5619 | 10 | 0.1 | 576.2883 | 2 | 10.97 | 7 | F7:1808 | DaRuMP\_F7B.raw |  |  |  |  |  |  | 2.3797E6 |  | 2.684E4 |  | 2 | 0 | 0 | 0 | 0 | 0 | 0 | 1 | 0 | 1 | 0 | 112 | 121 |  |  | PEAKS DB |
| G.NLFQFGEMILEK.T | Y | 70.56 | 1467.7432 | 12 | 0.8 | 734.8795 | 2 | 45.20 | 7 | F7:22864 | DaRuMP\_F7B.raw |  |  |  |  |  |  | 2.2986E7 |  |  | 4.5122E4 | 2 | 0 | 0 | 0 | 0 | 0 | 0 | 1 | 0 | 0 | 1 | 17 | 28 |  |  | PEAKS DB |
| D.C(+57.02)C(+57.02)YGTVNDC(+57.02)NPK.T | N | 67.90 | 1486.5640 | 12 | 0.1 | 744.2893 | 2 | 10.93 | 7 | F7:1780 | DaRuMP\_F7B.raw |  |  |  |  |  |  | 2.6177E5 |  |  |  | 1 | 0 | 0 | 0 | 0 | 0 | 0 | 1 | 0 | 0 | 0 | 65 | 76 | Carbamidomethylation | C1:Carbamidomethylation:1000.00;C2:Carbamidomethylation:1000.00;C9:Carbamidomethylation:1000.00 | PEAKS DB |
| C.FVHDC(+57.02)C(+57.02)YGTVNDC(+57.02)NPK.T | N | 64.69 | 1984.7866 | 16 | 0.4 | 662.6031 | 3 | 11.09 | 7 | F7:1920 | DaRuMP\_F7B.raw |  |  |  |  |  |  | 8.4449E5 |  |  |  | 2 | 0 | 0 | 0 | 0 | 0 | 0 | 2 | 0 | 0 | 0 | 61 | 76 | Carbamidomethylation | C5:Carbamidomethylation:1000.00;C6:Carbamidomethylation:1000.00;C13:Carbamidomethylation:1000.00 | PEAKS DB |
| C.YC(+57.02)GWGGQGR.A | N | 64.40 | 1039.4294 | 9 | -0.3 | 520.7219 | 2 | 11.24 | 7 | F7:2062 | DaRuMP\_F7B.raw |  |  |  |  |  |  | 2.1146E5 |  |  |  | 1 | 0 | 0 | 0 | 0 | 0 | 0 | 1 | 0 | 0 | 0 | 43 | 51 | Carbamidomethylation | C2:Carbamidomethylation:1000.00 | PEAKS DB |
| G.NLFQFGEM(+15.99)ILEK.T | Y | 63.73 | 1483.7380 | 12 | 0.1 | 742.8764 | 2 | 38.82 | 7 | F7:19876 | DaRuMP\_F7B.raw |  |  |  |  |  |  | 4.3491E6 |  |  |  | 2 | 0 | 0 | 0 | 0 | 0 | 0 | 2 | 0 | 0 | 0 | 17 | 28 | Oxidation (M) | M8:Oxidation (M):1000.00 | PEAKS DB |
| C.C(+57.02)YGTVNDC(+57.02)NPK.T | N | 61.83 | 1326.5333 | 11 | -2.4 | 664.2723 | 2 | 10.80 | 7 | F7:1661 | DaRuMP\_F7B.raw |  |  |  |  |  |  | 5.2926E4 |  |  |  | 1 | 0 | 0 | 0 | 0 | 0 | 0 | 1 | 0 | 0 | 0 | 66 | 76 | Carbamidomethylation | C1:Carbamidomethylation:1000.00;C8:Carbamidomethylation:1000.00 | PEAKS DB |
| K.TATYSYSFENGDIVC(+57.02)GDNDLC(+57.02)LR.T | Y | 61.67 | 2669.1375 | 23 | 0.1 | 1335.5762 | 2 | 23.63 | 7 | F7:12366 | DaRuMP\_F7B.raw |  |  |  |  |  |  | 3.6205E7 |  |  |  | 2 | 0 | 0 | 0 | 0 | 0 | 0 | 2 | 0 | 0 | 0 | 77 | 99 | Carbamidomethylation | C15:Carbamidomethylation:1000.00;C21:Carbamidomethylation:1000.00 | PEAKS DB |
| K.NYEYYSISHC(+57.02)TEESEQC(+57.02) | N | 60.58 | 2197.8206 | 17 | 0.4 | 1099.9180 | 2 | 11.80 | 7 | F7:2602 | DaRuMP\_F7B.raw |  |  |  |  |  |  | 9.133E6 |  |  |  | 1 | 0 | 0 | 0 | 0 | 0 | 0 | 1 | 0 | 0 | 0 | 122 | 138 | Carbamidomethylation | C10:Carbamidomethylation:1000.00;C17:Carbamidomethylation:1000.00 | PEAKS DB |
| H.DC(+57.02)C(+57.02)YGTVNDC(+57.02)NPK.T | N | 56.84 | 1601.5908 | 13 | 0.9 | 801.8034 | 2 | 11.09 | 7 | F7:1923 | DaRuMP\_F7B.raw |  |  |  |  |  |  | 1.757E5 |  |  |  | 1 | 0 | 0 | 0 | 0 | 0 | 0 | 1 | 0 | 0 | 0 | 64 | 76 | Carbamidomethylation | C2:Carbamidomethylation:1000.00;C3:Carbamidomethylation:1000.00;C10:Carbamidomethylation:1000.00 | PEAKS DB |
| R.AAAIC(+57.02)LGQNVN.T | N | 56.67 | 1129.5549 | 11 | 0.7 | 565.7852 | 2 | 12.72 | 7 | F7:3394 | DaRuMP\_F7B.raw |  |  |  |  |  |  | 1.2993E7 |  |  |  | 1 | 0 | 0 | 0 | 0 | 0 | 0 | 1 | 0 | 0 | 0 | 107 | 117 | Carbamidomethylation | C5:Carbamidomethylation:1000.00 | PEAKS DB |
| E.GNLFQFGEM(+15.99)ILEK.T | Y | 54.53 | 1540.7595 | 13 | 0.1 | 771.3871 | 2 | 46.60 | 7 | F7:23400 | DaRuMP\_F7B.raw |  |  |  |  |  |  | 8.144E5 |  |  |  | 1 | 0 | 0 | 0 | 0 | 0 | 0 | 1 | 0 | 0 | 0 | 16 | 28 | Oxidation (M) | M9:Oxidation (M):1000.00 | PEAKS DB |
| K.EVVHSYAIYGC(+57.02)Y.C | Y | 53.20 | 1459.6442 | 12 | 0.9 | 730.8300 | 2 | 13.22 | 7 | F7:3831 | DaRuMP\_F7B.raw |  |  |  |  |  |  | 8.8019E6 |  |  |  | 1 | 0 | 0 | 0 | 0 | 0 | 0 | 1 | 0 | 0 | 0 | 32 | 43 | Carbamidomethylation | C11:Carbamidomethylation:1000.00 | PEAKS DB |
| Y.SFENGDIVC(+57.02)GDNDLC(+57.02)LR.T | N | 52.93 | 1982.8462 | 17 | 0.7 | 992.4310 | 2 | 16.34 | 7 | F7:6484 | DaRuMP\_F7B.raw |  |  |  |  |  |  | 6.9494E6 |  |  |  | 1 | 0 | 0 | 0 | 0 | 0 | 0 | 1 | 0 | 0 | 0 | 83 | 99 | Carbamidomethylation | C9:Carbamidomethylation:1000.00;C15:Carbamidomethylation:1000.00 | PEAKS DB |
| R.AAAIC(+57.02)LGQNVNTYDKN.Y | N | 52.23 | 1750.8308 | 16 | -4.3 | 876.4189 | 2 | 11.91 | 7 | F7:2705 | DaRuMP\_F7B.raw |  |  |  |  |  |  | 3.5566E7 |  |  |  | 1 | 0 | 0 | 0 | 0 | 0 | 0 | 1 | 0 | 0 | 0 | 107 | 122 | Carbamidomethylation | C5:Carbamidomethylation:1000.00 | PEAKS DB |
| total 21 peptides |
| --- |

P35527|K1C9\_HUMAN

back to list

  

| Protein Coverage
| Supporting Peptides
|

Protein Coverage:

Supporting Peptides:

| Peptide | Uniq | -10lgP | Mass | Length | ppm | m/z | z | RT | Fraction | Scan | Source File | Area F1 | Area F10 | Area F2\_3 | Area F5 | Area F6 | Area F7A | Area F7B | Area F8A | Area F8B | Area F9 | #Feature | #Feature F1 | #Feature F10 | #Feature F2\_3 | #Feature F5 | #Feature F6 | #Feature F7A | #Feature F7B | #Feature F8A | #Feature F8B | #Feature F9 | Start | End | PTM | AScore | Found By |
| --- | --- | --- | --- | --- | --- | --- | --- | --- | --- | --- | --- | --- | --- | --- | --- | --- | --- | --- | --- | --- | --- | --- | --- | --- | --- | --- | --- | --- | --- | --- | --- | --- | --- | --- | --- | --- | --- |
| R.GGSGGSYGGGGSGGGYGGGSGSR.G | Y | 94.97 | 1790.7203 | 23 | 1.3 | 896.3687 | 2 | 10.70 | 9 | F9:1615 | DaRuMP\_F8B.raw | 9.8072E5 |  |  |  |  |  | 8.2654E5 |  | 4.0432E5 | 1.4157E5 | 4 | 1 | 0 | 0 | 0 | 0 | 0 | 1 | 0 | 1 | 1 | 491 | 513 |  |  | PEAKS DB |
| K.VQALEEANNDLENK.I | Y | 90.91 | 1585.7583 | 14 | 0.8 | 793.8871 | 2 | 11.56 | 2 | F2:2391 | DaRuMP\_F10.raw | 5.7317E5 | 1.0932E5 | 1.6146E5 | 6.4964E5 | 6.1318E5 |  | 1.6597E6 |  | 4.3305E5 | 1.1873E5 | 9 | 1 | 1 | 1 | 2 | 1 | 0 | 1 | 0 | 1 | 1 | 171 | 184 |  |  | PEAKS DB |
| R.SGGGGGGGLGSGGSIR.S | Y | 81.91 | 1231.5905 | 16 | 0.4 | 616.8027 | 2 | 10.95 | 5 | F5:1825 | DaRuMP\_F6.raw | 3.2584E5 | 1.2995E5 | 9.3335E4 | 1.622E5 | 1.0096E5 |  | 1.5962E5 |  | 5.5012E4 | 1.5222E5 | 8 | 1 | 1 | 1 | 1 | 1 | 0 | 1 | 0 | 1 | 1 | 14 | 29 |  |  | PEAKS DB |
| K.EIETYHNLLEGGQEDFESSGAGK.I | Y | 80.00 | 2509.1245 | 23 | 0.9 | 837.3829 | 3 | 16.62 | 8 | F8:3305 | DaRuMP\_F8A.raw |  |  |  |  | 1.1428E6 |  | 3.3869E6 | 7.4085E5 |  |  | 3 | 0 | 0 | 0 | 0 | 1 | 0 | 1 | 1 | 0 | 0 | 450 | 472 |  |  | PEAKS DB |
| R.QGVDADINGLR.Q | Y | 77.24 | 1156.5836 | 11 | 0.4 | 579.2993 | 2 | 11.72 | 3 | F3:2527 | DaRuMP\_F2\_F3.raw | 5.3153E5 | 3.6793E4 | 1.5028E5 | 9.7114E5 | 3.2744E5 |  | 9.1409E5 | 1.2254E5 | 2.9766E5 | 6.6045E4 | 9 | 1 | 1 | 1 | 1 | 1 | 0 | 1 | 1 | 1 | 1 | 251 | 261 |  |  | PEAKS DB |
| Y.SYGGGSGGGFSASSLGGGFGGGSR.G | Y | 76.53 | 2021.8827 | 24 | 0.2 | 1011.9488 | 2 | 12.60 | 5 | F5:3380 | DaRuMP\_F6.raw |  |  |  |  | 4.0384E5 |  | 1.1682E6 | 2.4826E5 |  |  | 3 | 0 | 0 | 0 | 0 | 1 | 0 | 1 | 1 | 0 | 0 | 72 | 95 |  |  | PEAKS DB |
| R.HGVQELEIELQSQLSK.K | Y | 72.36 | 1836.9581 | 16 | 0.4 | 919.4867 | 2 | 17.83 | 4 | F4:6324 | DaRuMP\_F5.raw |  |  |  | 1.0656E6 | 7.063E5 |  | 1.4171E6 | 1.8489E5 |  | 6.5026E4 | 6 | 0 | 0 | 0 | 2 | 1 | 0 | 1 | 1 | 0 | 1 | 375 | 390 |  |  | PEAKS DB |
| K.DQIVDLTVGNNK.T | Y | 71.60 | 1314.6780 | 12 | 0.8 | 658.3468 | 2 | 16.55 | 8 | F8:3233 | DaRuMP\_F8A.raw | 6.6299E4 |  |  | 2.108E5 |  |  | 4.1522E5 | 6.8461E4 |  |  | 4 | 1 | 0 | 0 | 1 | 0 | 0 | 1 | 1 | 0 | 0 | 213 | 224 |  |  | PEAKS DB |
| R.QEYEQLIAK.N | Y | 70.23 | 1120.5764 | 9 | -0.5 | 561.2952 | 2 | 11.65 | 3 | F3:2457 | DaRuMP\_F2\_F3.raw | 4.482E5 |  | 1.7987E5 | 4.2296E5 | 4.7924E5 |  | 1.4085E6 | 0 | 3.3651E5 | 1.2114E5 | 7 | 1 | 0 | 1 | 1 | 1 | 0 | 1 | 0 | 1 | 1 | 328 | 336 |  |  | PEAKS DB |
| R.QEIEC(+57.02)QNQEYSLLLSIK.M | Y | 67.47 | 2094.0303 | 17 | 0.7 | 1048.0232 | 2 | 26.29 | 7 | F7:14062 | DaRuMP\_F7B.raw |  |  |  | 1.0382E6 | 9.9535E5 |  | 1.6736E6 | 0 | 1.5185E6 |  | 4 | 0 | 0 | 0 | 1 | 1 | 0 | 1 | 0 | 1 | 0 | 428 | 444 | Carbamidomethylation | C5:Carbamidomethylation:1000.00 | PEAKS DB |
| K.DIENQYETQITQIEHEVSSSGQEVQSSAK.E | Y | 65.61 | 3263.5066 | 29 | 0.6 | 1088.8435 | 3 | 30.54 | 5 | F5:13231 | DaRuMP\_F6.raw |  |  |  |  | 1.5862E6 |  | 3.2033E6 |  | 1.5059E6 |  | 3 | 0 | 0 | 0 | 0 | 1 | 0 | 1 | 0 | 1 | 0 | 340 | 368 |  |  | PEAKS DB |
| K.SDLEM(+15.99)QYETLQEELM(+15.99)ALK.K | Y | 64.01 | 2202.0071 | 18 | 0.4 | 1102.0112 | 2 | 44.05 | 5 | F5:17850 | DaRuMP\_F6.raw |  |  |  | 2.5797E5 | 6.1808E5 |  | 7.5839E5 |  |  |  | 3 | 0 | 0 | 0 | 1 | 1 | 0 | 1 | 0 | 0 | 0 | 272 | 289 | Oxidation (M) | M5:Oxidation (M):1000.00;M15:Oxidation (M):1000.00 | PEAKS DB |
| K.NYSPYYNTIDDLKDQIVDLTVGNNK.T | Y | 63.99 | 2901.4031 | 25 | 1.0 | 968.1426 | 3 | 43.11 | 7 | F7:21898 | DaRuMP\_F7B.raw |  |  |  |  |  |  | 3.2612E6 |  |  |  | 1 | 0 | 0 | 0 | 0 | 0 | 0 | 1 | 0 | 0 | 0 | 200 | 224 |  |  | PEAKS DB |
| R.YC(+57.02)GQLQM(+15.99)IQEQISNLEAQITDVR.Q | Y | 62.94 | 2752.3159 | 23 | 0.7 | 918.4465 | 3 | 55.80 | 7 | F7:26636 | DaRuMP\_F7B.raw |  |  |  |  |  |  | 6.737E5 |  |  |  | 1 | 0 | 0 | 0 | 0 | 0 | 0 | 1 | 0 | 0 | 0 | 405 | 427 | Carbamidomethylation; Oxidation (M) | C2:Carbamidomethylation:1000.00;M7:Oxidation (M):1000.00 | PEAKS DB |
| F.GGFGGGAGGGDGGILTANEK.S | Y | 62.81 | 1690.7910 | 20 | 1.1 | 846.4037 | 2 | 11.99 | 10 | F10:2730 | DaRuMP\_F9.raw |  |  |  |  |  |  |  |  |  | 1.123E4 | 1 | 0 | 0 | 0 | 0 | 0 | 0 | 0 | 0 | 0 | 1 | 135 | 154 |  |  | PEAKS DB |
| Q.ISNLEAQITDVR.Q | Y | 62.10 | 1357.7201 | 12 | 0.2 | 679.8674 | 2 | 12.35 | 7 | F7:3144 | DaRuMP\_F7B.raw | 8.6713E4 |  |  | 7.101E4 |  |  | 1.5443E5 |  | 4.0523E4 | 1.918E4 | 5 | 1 | 0 | 0 | 1 | 0 | 0 | 1 | 0 | 1 | 1 | 416 | 427 |  |  | PEAKS DB |
| R.FSSSSGYGGGSSR.V | Y | 61.19 | 1234.5214 | 13 | 0.6 | 618.2683 | 2 | 10.71 | 9 | F9:1639 | DaRuMP\_F8B.raw |  | 0 |  |  |  |  |  |  | 1.1562E5 |  | 1 | 0 | 0 | 0 | 0 | 0 | 0 | 0 | 0 | 1 | 0 | 47 | 59 |  |  | PEAKS DB |
| R.QVLDNLTM(+15.99)EK.S | Y | 60.98 | 1205.5962 | 10 | -3.6 | 603.8032 | 2 | 11.66 | 4 | F4:2530 | DaRuMP\_F5.raw | 1.4367E5 |  |  | 2.2252E5 | 2.3675E5 |  |  |  |  |  | 3 | 1 | 0 | 0 | 1 | 1 | 0 | 0 | 0 | 0 | 0 | 262 | 271 | Oxidation (M) | M8:Oxidation (M):1000.00 | PEAKS DB |
| R.GGSGGSHGGGSGFGGESGGSYGGGEEASGSGGGYGGGSGK.S | Y | 60.90 | 3222.2742 | 40 | 0.2 | 1075.0989 | 3 | 11.08 | 10 | F10:1880 | DaRuMP\_F9.raw |  |  |  |  |  |  |  |  | 1.233E5 | 3.6861E4 | 2 | 0 | 0 | 0 | 0 | 0 | 0 | 0 | 0 | 1 | 1 | 580 | 619 |  |  | PEAKS DB |
| K.TLLDIDNTR.M | Y | 60.76 | 1059.5560 | 9 | 0.6 | 530.7856 | 2 | 16.31 | 8 | F8:3034 | DaRuMP\_F8A.raw |  | 2.5986E5 | 2.8296E5 | 1.2073E6 | 1.1295E6 |  | 3.1585E6 | 4.5901E5 | 7.43E5 |  | 7 | 0 | 1 | 1 | 1 | 1 | 0 | 1 | 1 | 1 | 0 | 225 | 233 |  |  | PEAKS DB |
| K.STMQELNSR.L | Y | 59.67 | 1064.4921 | 9 | -0.4 | 533.2531 | 2 | 11.01 | 7 | F7:1851 | DaRuMP\_F7B.raw |  |  |  |  |  |  | 8.1649E3 |  |  |  | 1 | 0 | 0 | 0 | 0 | 0 | 0 | 1 | 0 | 0 | 0 | 155 | 163 |  |  | PEAKS DB |
| M.IQEQISNLEAQITDVR.Q | Y | 59.61 | 1855.9639 | 16 | 1.0 | 928.9902 | 2 | 22.25 | 8 | F8:7580 | DaRuMP\_F8A.raw |  |  |  |  |  |  | 6.0627E4 | 8.2139E4 | 9.5374E4 |  | 3 | 0 | 0 | 0 | 0 | 0 | 0 | 1 | 1 | 1 | 0 | 412 | 427 |  |  | PEAKS DB |
| R.QVLDNLTMEK.S | Y | 58.35 | 1189.6013 | 10 | 0.1 | 595.8080 | 2 | 11.99 | 1 | F1:2847 | DaRuMP\_F1.raw | 4.6556E4 |  |  |  |  |  |  |  |  |  | 1 | 1 | 0 | 0 | 0 | 0 | 0 | 0 | 0 | 0 | 0 | 262 | 271 |  |  | PEAKS DB |
| K.NYSPYYNTIDDLK.D | Y | 56.63 | 1604.7358 | 13 | 1.0 | 803.3760 | 2 | 13.78 | 7 | F7:4333 | DaRuMP\_F7B.raw |  | 3.2862E4 |  |  |  |  | 5.1578E5 |  | 1.5425E5 |  | 3 | 0 | 1 | 0 | 0 | 0 | 0 | 1 | 0 | 1 | 0 | 200 | 212 |  |  | PEAKS DB |
| K.IQDWYDK.K | Y | 56.07 | 966.4447 | 7 | -0.1 | 484.2296 | 2 | 11.67 | 7 | F7:2477 | DaRuMP\_F7B.raw |  |  |  |  |  |  | 1.4241E5 |  |  |  | 1 | 0 | 0 | 0 | 0 | 0 | 0 | 1 | 0 | 0 | 0 | 185 | 191 |  |  | PEAKS DB |
| R.MTLDDFR.I | Y | 54.77 | 896.4062 | 7 | 1.0 | 449.2108 | 2 | 12.53 | 4 | F4:3274 | DaRuMP\_F5.raw |  |  |  | 4.815E4 |  |  | 1.0848E5 |  |  |  | 2 | 0 | 0 | 0 | 1 | 0 | 0 | 1 | 0 | 0 | 0 | 234 | 240 |  |  | PEAKS DB |
| S.GGGGGGGLGSGGSIR.S | Y | 53.82 | 1144.5585 | 15 | 0.5 | 573.2868 | 2 | 10.99 | 10 | F10:1800 | DaRuMP\_F9.raw |  |  |  |  |  |  |  |  |  | 9.2335E3 | 1 | 0 | 0 | 0 | 0 | 0 | 0 | 0 | 0 | 0 | 1 | 15 | 29 |  |  | PEAKS DB |
| R.QFSSSYLSR.S | Y | 52.66 | 1073.5142 | 9 | 0.9 | 537.7648 | 2 | 11.57 | 4 | F4:2434 | DaRuMP\_F5.raw |  |  |  | 0 |  |  |  |  |  |  | 0 | 0 | 0 | 0 | 0 | 0 | 0 | 0 | 0 | 0 | 0 | 5 | 13 |  |  | PEAKS DB |
| total 28 peptides |
| --- |

F8QN54|PA2B\_VIPRE

back to list

  

| Protein Coverage
| Supporting Peptides
|

Protein Coverage:

Supporting Peptides:

| Peptide | Uniq | -10lgP | Mass | Length | ppm | m/z | z | RT | Fraction | Scan | Source File | Area F1 | Area F10 | Area F2\_3 | Area F5 | Area F6 | Area F7A | Area F7B | Area F8A | Area F8B | Area F9 | #Feature | #Feature F1 | #Feature F10 | #Feature F2\_3 | #Feature F5 | #Feature F6 | #Feature F7A | #Feature F7B | #Feature F8A | #Feature F8B | #Feature F9 | Start | End | PTM | AScore | Found By |
| --- | --- | --- | --- | --- | --- | --- | --- | --- | --- | --- | --- | --- | --- | --- | --- | --- | --- | --- | --- | --- | --- | --- | --- | --- | --- | --- | --- | --- | --- | --- | --- | --- | --- | --- | --- | --- | --- |
| R.C(+57.02)C(+57.02)FVHDC(+57.02)C(+57.02)YGNLPDC(+57.02)NPK.I | N | 103.24 | 2314.8687 | 18 | 1.4 | 1158.4404 | 2 | 11.58 | 6 | F6:2373 | DaRuMP\_F7A.raw | 2.8891E5 |  |  |  | 1.2509E6 | 4.8203E8 | 1.5976E7 | 2.1062E8 |  | 1.2674E5 | 9 | 1 | 0 | 0 | 0 | 1 | 2 | 2 | 2 | 0 | 1 | 59 | 76 | Carbamidomethylation | C1:Carbamidomethylation:1000.00;C2:Carbamidomethylation:1000.00;C7:Carbamidomethylation:1000.00;C8:Carbamidomethylation:1000.00;C15:Carbamidomethylation:1000.00 | PEAKS DB |
| G.SLLEFGM(+15.99)MILEETGK.N | N | 96.55 | 1712.8364 | 15 | 0.6 | 857.4260 | 2 | 53.14 | 8 | F8:23593 | DaRuMP\_F8A.raw |  |  |  |  |  |  |  | 2.8084E7 |  | 3.2977E5 | 5 | 0 | 0 | 0 | 0 | 0 | 0 | 0 | 4 | 0 | 1 | 17 | 31 | Oxidation (M) | M7:Oxidation (M):26.02 | PEAKS DB |
| C.FVHDC(+57.02)C(+57.02)YGNLPDC(+57.02)NPK.I | N | 93.60 | 1994.8074 | 16 | 2.2 | 998.4108 | 2 | 11.39 | 6 | F6:2215 | DaRuMP\_F7A.raw |  |  |  |  |  | 2.6011E6 |  |  |  |  | 2 | 0 | 0 | 0 | 0 | 0 | 2 | 0 | 0 | 0 | 0 | 61 | 76 | Carbamidomethylation | C5:Carbamidomethylation:1000.00;C6:Carbamidomethylation:1000.00;C13:Carbamidomethylation:1000.00 | PEAKS DB |
| G.SLLEFGMM(+15.99)ILEETGK.N | N | 91.71 | 1712.8364 | 15 | 0.2 | 857.4257 | 2 | 60.23 | 8 | F8:26218 | DaRuMP\_F8A.raw |  |  |  |  |  |  |  | 1.5322E7 |  | 3.2977E5 | 4 | 0 | 0 | 0 | 0 | 0 | 0 | 0 | 3 | 0 | 1 | 17 | 31 | Oxidation (M) | M8:Oxidation (M):40.00 | PEAKS DB |
| G.SLLEFGMMILEETGK.N | N | 87.31 | 1696.8416 | 15 | 0.5 | 849.4285 | 2 | 66.01 | 8 | F8:27922 | DaRuMP\_F8A.raw |  |  |  |  |  |  |  | 2.2161E7 |  |  | 2 | 0 | 0 | 0 | 0 | 0 | 0 | 0 | 2 | 0 | 0 | 17 | 31 |  |  | PEAKS DB |
| G.SLLEFGM(+15.99)M(+15.99)ILEETGK.N | N | 86.23 | 1728.8314 | 15 | 0.1 | 865.4231 | 2 | 45.65 | 8 | F8:20926 | DaRuMP\_F8A.raw |  |  |  |  |  |  |  | 1.0085E7 |  | 7.4736E5 | 8 | 0 | 0 | 0 | 0 | 0 | 0 | 0 | 6 | 0 | 2 | 17 | 31 | Oxidation (M) | M7:Oxidation (M):1000.00;M8:Oxidation (M):1000.00 | PEAKS DB |
| E.GSLLEFGM(+15.99)MILEETGK.N | Y | 84.15 | 1769.8579 | 16 | 1.0 | 885.9371 | 2 | 58.89 | 8 | F8:26196 | DaRuMP\_F8A.raw |  |  |  |  |  |  |  | 1.3134E7 |  |  | 3 | 0 | 0 | 0 | 0 | 0 | 0 | 0 | 3 | 0 | 0 | 16 | 31 | Oxidation (M) | M8:Oxidation (M):40.00 | PEAKS DB |
| E.GSLLEFGMM(+15.99)ILEETGK.N | Y | 81.76 | 1769.8579 | 16 | 0.2 | 885.9364 | 2 | 68.19 | 8 | F8:28361 | DaRuMP\_F8A.raw |  |  |  |  |  |  |  | 3.1905E6 |  |  | 2 | 0 | 0 | 0 | 0 | 0 | 0 | 0 | 2 | 0 | 0 | 16 | 31 | Oxidation (M) | M9:Oxidation (M):30.46 | PEAKS DB |
| K.DATDRC(+57.02)C(+57.02)FVHDC(+57.02)C(+57.02)YGNLPDC(+57.02)NPK.I | N | 78.23 | 2873.1084 | 23 | 1.9 | 958.7096 | 3 | 11.58 | 6 | F6:2387 | DaRuMP\_F7A.raw |  |  |  |  |  | 1.375E6 |  |  |  |  | 1 | 0 | 0 | 0 | 0 | 0 | 1 | 0 | 0 | 0 | 0 | 54 | 76 | Carbamidomethylation | C6:Carbamidomethylation:1000.00;C7:Carbamidomethylation:1000.00;C12:Carbamidomethylation:1000.00;C13:Carbamidomethylation:1000.00;C20:Carbamidomethylation:1000.00 | PEAKS DB |
| E.GSLLEFGMMILEETGK.N | Y | 77.74 | 1753.8630 | 16 | 0.3 | 877.9391 | 2 | 74.74 | 8 | F8:29667 | DaRuMP\_F8A.raw |  |  |  |  |  |  |  | 4.3861E6 |  |  | 1 | 0 | 0 | 0 | 0 | 0 | 0 | 0 | 1 | 0 | 0 | 16 | 31 |  |  | PEAKS DB |
| F.VHDC(+57.02)C(+57.02)YGNLPDC(+57.02)NPK.I | N | 77.58 | 1847.7389 | 15 | 1.3 | 924.8757 | 2 | 11.13 | 6 | F6:1990 | DaRuMP\_F7A.raw |  |  |  |  |  | 4.0829E5 |  |  |  |  | 1 | 0 | 0 | 0 | 0 | 0 | 1 | 0 | 0 | 0 | 0 | 62 | 76 | Carbamidomethylation | C4:Carbamidomethylation:1000.00;C5:Carbamidomethylation:1000.00;C12:Carbamidomethylation:1000.00 | PEAKS DB |
| D.C(+57.02)C(+57.02)YGNLPDC(+57.02)NPK.I | N | 77.58 | 1496.5846 | 12 | 3.4 | 749.3003 | 2 | 11.37 | 6 | F6:2187 | DaRuMP\_F7A.raw |  |  |  |  |  | 1.8907E6 |  | 0 |  | 5.5068E4 | 2 | 0 | 0 | 0 | 0 | 0 | 1 | 0 | 0 | 0 | 1 | 65 | 76 | Carbamidomethylation | C1:Carbamidomethylation:1000.00;C2:Carbamidomethylation:1000.00;C9:Carbamidomethylation:1000.00 | PEAKS DB |
| C.C(+57.02)YGNLPDC(+57.02)NPK.I | N | 75.32 | 1336.5540 | 11 | 3.2 | 669.2848 | 2 | 11.29 | 6 | F6:2118 | DaRuMP\_F7A.raw |  |  |  |  |  | 3.3405E5 |  |  |  |  | 1 | 0 | 0 | 0 | 0 | 0 | 1 | 0 | 0 | 0 | 0 | 66 | 76 | Carbamidomethylation | C1:Carbamidomethylation:1000.00;C8:Carbamidomethylation:1000.00 | PEAKS DB |
| E.GSLLEFGM(+15.99)M(+15.99)ILEETGK.N | Y | 72.42 | 1785.8528 | 16 | 0.8 | 893.9344 | 2 | 52.22 | 8 | F8:23250 | DaRuMP\_F8A.raw |  |  |  |  |  |  |  | 0 |  |  | 0 | 0 | 0 | 0 | 0 | 0 | 0 | 0 | 0 | 0 | 0 | 16 | 31 | Oxidation (M) | M8:Oxidation (M):1000.00;M9:Oxidation (M):1000.00 | PEAKS DB |
| C.YGNLPDC(+57.02)NPK.I | N | 71.12 | 1176.5233 | 10 | 2.2 | 589.2688 | 2 | 11.15 | 6 | F6:1993 | DaRuMP\_F7A.raw |  |  |  |  |  | 3.1081E5 |  |  |  |  | 1 | 0 | 0 | 0 | 0 | 0 | 1 | 0 | 0 | 0 | 0 | 67 | 76 | Carbamidomethylation | C7:Carbamidomethylation:1000.00 | PEAKS DB |
| R.IC(+57.02)EC(+57.02)DRAAAIC(+57.02)FR.K | N | 71.06 | 1640.7222 | 13 | -5.5 | 821.3638 | 2 | 16.53 | 8 | F8:3246 | DaRuMP\_F8A.raw |  |  |  |  |  |  |  | 9.4518E4 |  |  | 1 | 0 | 0 | 0 | 0 | 0 | 0 | 0 | 1 | 0 | 0 | 101 | 113 | Carbamidomethylation | C2:Carbamidomethylation:1000.00;C4:Carbamidomethylation:1000.00;C11:Carbamidomethylation:1000.00 | PEAKS DB |
| H.DC(+57.02)C(+57.02)YGNLPDC(+57.02)NPK.I | N | 60.75 | 1611.6116 | 13 | 2.6 | 806.8132 | 2 | 11.50 | 6 | F6:2310 | DaRuMP\_F7A.raw |  |  |  |  |  | 4.1823E5 |  |  |  |  | 1 | 0 | 0 | 0 | 0 | 0 | 1 | 0 | 0 | 0 | 0 | 64 | 76 | Carbamidomethylation | C2:Carbamidomethylation:1000.00;C3:Carbamidomethylation:1000.00;C10:Carbamidomethylation:1000.00 | PEAKS DB |
| F.GMMILEETGK.N | N | 55.74 | 1107.5304 | 10 | 0.0 | 554.7725 | 2 | 16.49 | 8 | F8:3169 | DaRuMP\_F8A.raw |  |  |  |  |  |  |  | 1.5241E6 |  |  | 1 | 0 | 0 | 0 | 0 | 0 | 0 | 0 | 1 | 0 | 0 | 22 | 31 |  |  | PEAKS DB |
| R.AAAIC(+57.02)FR.K | N | 54.51 | 807.4061 | 7 | -5.0 | 404.7083 | 2 | 17.94 | 8 | F8:4539 | DaRuMP\_F8A.raw |  |  |  |  |  |  |  | 2.6963E7 |  |  | 2 | 0 | 0 | 0 | 0 | 0 | 0 | 0 | 2 | 0 | 0 | 107 | 113 | Carbamidomethylation | C5:Carbamidomethylation:1000.00 | PEAKS DB |
| M.MILEETGK.N | N | 54.26 | 919.4684 | 8 | -0.1 | 460.7415 | 2 | 11.49 | 7 | F7:2297 | DaRuMP\_F7B.raw |  |  |  |  |  |  | 4.4177E5 | 1.1565E6 |  |  | 2 | 0 | 0 | 0 | 0 | 0 | 0 | 1 | 1 | 0 | 0 | 24 | 31 |  |  | PEAKS DB |
| total 20 peptides |
| --- |

P13645|K1C10\_HUMAN

back to list

  

| Protein Coverage
| Supporting Peptides
|

Protein Coverage:

Supporting Peptides:

| Peptide | Uniq | -10lgP | Mass | Length | ppm | m/z | z | RT | Fraction | Scan | Source File | Area F1 | Area F10 | Area F2\_3 | Area F5 | Area F6 | Area F7A | Area F7B | Area F8A | Area F8B | Area F9 | #Feature | #Feature F1 | #Feature F10 | #Feature F2\_3 | #Feature F5 | #Feature F6 | #Feature F7A | #Feature F7B | #Feature F8A | #Feature F8B | #Feature F9 | Start | End | PTM | AScore | Found By |
| --- | --- | --- | --- | --- | --- | --- | --- | --- | --- | --- | --- | --- | --- | --- | --- | --- | --- | --- | --- | --- | --- | --- | --- | --- | --- | --- | --- | --- | --- | --- | --- | --- | --- | --- | --- | --- | --- |
| R.ALEESNYELEGK.I | Y | 82.12 | 1380.6409 | 12 | 0.8 | 691.3282 | 2 | 11.52 | 3 | F3:2330 | DaRuMP\_F2\_F3.raw | 4.7541E5 | 1.9666E5 | 2.0094E5 | 7.684E5 | 6.3534E5 |  | 1.5277E6 |  | 2.7689E5 | 1.0978E5 | 8 | 1 | 1 | 1 | 1 | 1 | 0 | 1 | 0 | 1 | 1 | 166 | 177 |  |  | PEAKS DB |
| R.SQYEQLAEQNR.K | Y | 82.08 | 1364.6321 | 11 | -1.9 | 683.3220 | 2 | 11.20 | 7 | F7:2037 | DaRuMP\_F7B.raw |  | 1.9063E5 | 1.0258E5 | 2.5526E5 | 2.7789E5 |  | 1.0378E6 |  | 1.424E5 | 1.3271E5 | 7 | 0 | 1 | 1 | 1 | 1 | 0 | 1 | 0 | 1 | 1 | 323 | 333 |  |  | PEAKS DB |
| R.SGGGGGGGGC(+57.02)GGGGGVSSLR.I | Y | 80.02 | 1548.6699 | 20 | 0.3 | 775.3425 | 2 | 10.93 | 7 | F7:1775 | DaRuMP\_F7B.raw |  |  |  |  | 8.9041E3 |  | 1.5864E4 |  |  |  | 2 | 0 | 0 | 0 | 0 | 1 | 0 | 1 | 0 | 0 | 0 | 16 | 35 | Carbamidomethylation | C10:Carbamidomethylation:1000.00 | PEAKS DB |
| K.ELTTEIDNNIEQISSYK.S | Y | 79.66 | 1995.9636 | 17 | 0.7 | 998.9898 | 2 | 20.70 | 7 | F7:10159 | DaRuMP\_F7B.raw |  | 3.9219E4 |  | 1.0343E6 | 1.5917E6 |  | 1.9378E6 |  |  |  | 4 | 0 | 1 | 0 | 1 | 1 | 0 | 1 | 0 | 0 | 0 | 346 | 362 |  |  | PEAKS DB |
| K.QSLEASLAETEGR.Y | Y | 77.15 | 1389.6736 | 13 | 0.2 | 695.8442 | 2 | 11.86 | 3 | F3:2665 | DaRuMP\_F2\_F3.raw | 2.2908E5 |  | 6.7771E4 | 4.4066E5 | 3.6498E5 |  | 1.0165E6 | 3.2487E5 | 6.8461E4 | 3.3985E4 | 8 | 1 | 0 | 1 | 1 | 1 | 0 | 1 | 1 | 1 | 1 | 387 | 399 |  |  | PEAKS DB |
| R.SLLEGEGSSGGGGR.G | Y | 77.08 | 1261.5898 | 14 | 0.7 | 631.8026 | 2 | 11.26 | 7 | F7:2080 | DaRuMP\_F7B.raw | 2.6183E5 |  | 5.7073E4 |  |  |  | 1.9296E5 |  |  | 9.1004E4 | 4 | 1 | 0 | 1 | 0 | 0 | 0 | 1 | 0 | 0 | 1 | 451 | 464 |  |  | PEAKS DB |
| K.NQILNLTTDNANILLQIDNAR.L | Y | 72.82 | 2366.2554 | 21 | 0.0 | 789.7590 | 3 | 40.93 | 8 | F8:18658 | DaRuMP\_F8A.raw |  | 4.7668E5 |  |  |  |  |  | 1.4404E6 |  |  | 3 | 0 | 2 | 0 | 0 | 0 | 0 | 0 | 1 | 0 | 0 | 208 | 228 |  |  | PEAKS DB |
| R.NVQALEIELQSQLALK.Q | Y | 72.52 | 1796.0043 | 16 | 0.6 | 899.0099 | 2 | 34.49 | 7 | F7:18036 | DaRuMP\_F7B.raw |  | 2.1155E5 |  |  |  |  | 1.1781E6 | 7.816E5 |  | 1.3221E5 | 6 | 0 | 1 | 0 | 0 | 0 | 0 | 2 | 2 | 0 | 1 | 371 | 386 |  |  | PEAKS DB |
| K.GSLGGGFSSGGFSGGSFSR.G | Y | 70.00 | 1706.7648 | 19 | 1.0 | 854.3905 | 2 | 13.40 | 7 | F7:4024 | DaRuMP\_F7B.raw |  |  |  | 5.625E5 |  | 4.981E4 | 9.8346E5 | 3.0941E5 |  | 2.551E5 | 5 | 0 | 0 | 0 | 1 | 0 | 1 | 1 | 1 | 0 | 1 | 41 | 59 |  |  | PEAKS DB |
| R.LENEIQTYR.S | Y | 68.18 | 1164.5775 | 9 | 0.6 | 583.2964 | 2 | 11.39 | 3 | F3:2187 | DaRuMP\_F2\_F3.raw | 5.9885E5 | 3.1346E5 | 1.5639E5 | 4.7082E5 | 4.4801E5 |  | 1.4174E6 |  | 9.9576E4 | 2.2538E5 | 8 | 1 | 1 | 1 | 1 | 1 | 0 | 1 | 0 | 1 | 1 | 442 | 450 |  |  | PEAKS DB |
| R.QSVEADINGLR.R | N | 64.15 | 1200.6099 | 11 | 0.8 | 601.3127 | 2 | 11.83 | 9 | F9:2680 | DaRuMP\_F8B.raw |  |  | 1.0108E5 | 3.4591E5 | 3.3814E5 |  | 7.3089E5 | 0 | 2.2041E5 |  | 5 | 0 | 0 | 1 | 1 | 1 | 0 | 1 | 0 | 1 | 0 | 246 | 256 |  |  | PEAKS DB |
| R.SQYEQLAEQNRK.D | Y | 63.56 | 1492.7269 | 12 | 0.1 | 498.5829 | 3 | 10.90 | 10 | F10:1706 | DaRuMP\_F9.raw | 1.8476E5 |  |  |  |  |  |  |  |  | 1.2377E5 | 3 | 1 | 0 | 0 | 0 | 0 | 0 | 0 | 0 | 0 | 2 | 323 | 334 |  |  | PEAKS DB |
| K.DAEAWFNEK.S | N | 63.08 | 1108.4825 | 9 | 0.4 | 555.2488 | 2 | 17.10 | 8 | F8:3709 | DaRuMP\_F8A.raw |  |  | 1.7688E5 |  |  |  | 1.1325E6 | 8.46E5 |  |  | 3 | 0 | 0 | 1 | 0 | 0 | 0 | 1 | 1 | 0 | 0 | 335 | 343 |  |  | PEAKS DB |
| K.YENEVALR.Q | Y | 63.06 | 992.4927 | 8 | -4.7 | 497.2513 | 2 | 11.35 | 7 | F7:2163 | DaRuMP\_F7B.raw | 1.9372E5 | 6.2185E4 |  | 2.698E5 | 9.5596E4 |  | 8.9001E5 |  | 1.4894E5 |  | 6 | 1 | 1 | 0 | 1 | 1 | 0 | 1 | 0 | 1 | 0 | 238 | 245 |  |  | PEAKS DB |
| R.AETEC(+57.02)QNTEYQQLLDIK.I | Y | 62.16 | 2081.9575 | 17 | 0.2 | 694.9933 | 3 | 16.92 | 1 | F1:4591 | DaRuMP\_F1.raw | 1.1121E5 |  |  | 1.8811E6 |  |  |  | 1.6213E6 |  |  | 3 | 1 | 0 | 0 | 1 | 0 | 0 | 0 | 1 | 0 | 0 | 423 | 439 | Carbamidomethylation | C5:Carbamidomethylation:1000.00 | PEAKS DB |
| R.VLDELTLTK.A | N | 60.94 | 1030.5911 | 9 | -0.6 | 516.3025 | 2 | 12.64 | 5 | F5:3408 | DaRuMP\_F6.raw | 5.1116E5 | 2.653E5 | 1.9756E5 | 7.0728E5 | 9.2005E5 |  | 1.9504E6 |  |  |  | 6 | 1 | 1 | 1 | 1 | 1 | 0 | 1 | 0 | 0 | 0 | 258 | 266 |  |  | PEAKS DB |
| Q.ISALEEQLQQIR.A | Y | 59.39 | 1426.7780 | 12 | 0.5 | 714.3966 | 2 | 15.27 | 9 | F9:5068 | DaRuMP\_F8B.raw |  |  |  |  | 7.2629E5 |  | 2.3854E6 |  | 3.9477E5 |  | 3 | 0 | 0 | 0 | 0 | 1 | 0 | 1 | 0 | 1 | 0 | 411 | 422 |  |  | PEAKS DB |
| R.LKYENEVALR.Q | Y | 53.91 | 1233.6716 | 10 | 0.8 | 412.2315 | 3 | 11.34 | 4 | F4:2219 | DaRuMP\_F5.raw |  |  |  | 6.3132E4 |  |  |  |  |  |  | 1 | 0 | 0 | 0 | 1 | 0 | 0 | 0 | 0 | 0 | 0 | 236 | 245 |  |  | PEAKS DB |
| K.VTMQNLNDR.L | N | 53.34 | 1089.5237 | 9 | 0.3 | 545.7693 | 2 | 11.22 | 4 | F4:2061 | DaRuMP\_F5.raw |  |  |  | 3.3198E4 |  |  |  |  |  |  | 1 | 0 | 0 | 0 | 1 | 0 | 0 | 0 | 0 | 0 | 0 | 148 | 156 |  |  | PEAKS DB |
| total 19 peptides |
| --- |

Q38L02|SLA\_DABSI

back to list

  

| Protein Coverage
| Supporting Peptides
|

Protein Coverage:

Supporting Peptides:

| Peptide | Uniq | -10lgP | Mass | Length | ppm | m/z | z | RT | Fraction | Scan | Source File | Area F1 | Area F10 | Area F2\_3 | Area F5 | Area F6 | Area F7A | Area F7B | Area F8A | Area F8B | Area F9 | #Feature | #Feature F1 | #Feature F10 | #Feature F2\_3 | #Feature F5 | #Feature F6 | #Feature F7A | #Feature F7B | #Feature F8A | #Feature F8B | #Feature F9 | Start | End | PTM | AScore | Found By |
| --- | --- | --- | --- | --- | --- | --- | --- | --- | --- | --- | --- | --- | --- | --- | --- | --- | --- | --- | --- | --- | --- | --- | --- | --- | --- | --- | --- | --- | --- | --- | --- | --- | --- | --- | --- | --- | --- |
| R.QQC(+57.02)SSHWTDGSAVSYETVTK.Y | Y | 112.51 | 2269.9910 | 20 | 0.0 | 1136.0028 | 2 | 11.57 | 9 | F9:2408 | DaRuMP\_F8B.raw |  |  |  |  |  |  | 3.3425E6 |  | 1.965E7 |  | 4 | 0 | 0 | 0 | 0 | 0 | 0 | 2 | 0 | 2 | 0 | 100 | 119 | Carbamidomethylation | C3:Carbamidomethylation:1000.00 | PEAKS DB |
| K.YHEWITLPC(+57.02)GDK.N | Y | 86.46 | 1517.6973 | 12 | 0.5 | 759.8563 | 2 | 12.26 | 9 | F9:3026 | DaRuMP\_F8B.raw |  | 1.8432E5 |  |  | 3.4628E6 | 7.0971E5 | 3.6608E7 | 2.0117E6 | 3.2731E7 | 9.9643E4 | 15 | 0 | 2 | 0 | 0 | 2 | 2 | 3 | 2 | 2 | 2 | 132 | 143 | Carbamidomethylation | C9:Carbamidomethylation:1000.00 | PEAKS DB |
| A.SIESVEEANFVAQLASETLTK.S | Y | 81.01 | 2265.1375 | 21 | -0.1 | 1133.5759 | 2 | 48.30 | 9 | F9:16086 | DaRuMP\_F8B.raw |  |  |  |  |  |  |  |  | 4.4493E6 |  | 1 | 0 | 0 | 0 | 0 | 0 | 0 | 0 | 0 | 1 | 0 | 64 | 84 |  |  | PEAKS DB |
| C.SSHWTDGSAVSYETVTK.Y | Y | 78.25 | 1853.8431 | 17 | 0.4 | 927.9292 | 2 | 11.57 | 9 | F9:2417 | DaRuMP\_F8B.raw |  |  |  |  |  |  | 3.7632E5 |  | 1.5782E6 |  | 3 | 0 | 0 | 0 | 0 | 0 | 0 | 1 | 0 | 2 | 0 | 103 | 119 |  |  | PEAKS DB |
| K.FC(+57.02)TQQANGWHLASIESVEEANFVAQLASETLTK.S | Y | 75.81 | 3678.7625 | 33 | 0.2 | 1227.2617 | 3 | 54.93 | 7 | F7:26346 | DaRuMP\_F7B.raw |  |  |  |  |  | 6.9047E6 | 7.3224E6 | 6.9051E6 | 6.5826E6 |  | 4 | 0 | 0 | 0 | 0 | 0 | 1 | 1 | 1 | 1 | 0 | 52 | 84 | Carbamidomethylation | C2:Carbamidomethylation:1000.00 | PEAKS DB |
| W.HLASIESVEEANFVAQLASETLTK.S | Y | 70.41 | 2586.3176 | 24 | 1.4 | 863.1144 | 3 | 43.33 | 8 | F8:19779 | DaRuMP\_F8A.raw |  |  |  |  |  |  |  | 6.4887E5 | 4.4161E6 |  | 4 | 0 | 0 | 0 | 0 | 0 | 0 | 0 | 2 | 2 | 0 | 61 | 84 |  |  | PEAKS DB |
| K.YHAWIGLR.D | Y | 66.22 | 1014.5399 | 8 | 1.1 | 508.2766 | 2 | 11.75 | 6 | F6:2552 | DaRuMP\_F7A.raw |  | 6.4341E4 |  |  | 1.2835E6 | 7.2795E5 | 1.2783E7 |  | 7.811E6 |  | 5 | 0 | 1 | 0 | 0 | 1 | 1 | 1 | 0 | 1 | 0 | 87 | 94 |  |  | PEAKS DB |
| K.YHEWITLPC(+57.02)GDKNPFIC(+57.02)K.S | Y | 65.35 | 2277.0710 | 18 | -0.1 | 570.2750 | 4 | 15.98 | 7 | F7:6217 | DaRuMP\_F7B.raw |  |  |  |  |  |  | 3.1551E5 |  |  |  | 1 | 0 | 0 | 0 | 0 | 0 | 0 | 1 | 0 | 0 | 0 | 132 | 149 | Carbamidomethylation | C9:Carbamidomethylation:1000.00;C17:Carbamidomethylation:1000.00 | PEAKS DB |
| K.YHEWITLPC(+57.02)GDKNPF.I | Y | 65.19 | 1875.8613 | 15 | -0.1 | 938.9379 | 2 | 20.04 | 9 | F9:7488 | DaRuMP\_F8B.raw |  |  |  |  |  |  | 2.1457E6 |  | 3.5912E6 |  | 2 | 0 | 0 | 0 | 0 | 0 | 0 | 1 | 0 | 1 | 0 | 132 | 146 | Carbamidomethylation | C9:Carbamidomethylation:1000.00 | PEAKS DB |
| D.GSAVSYETVTK.Y | Y | 59.48 | 1140.5663 | 11 | 0.5 | 571.2907 | 2 | 11.24 | 9 | F9:2112 | DaRuMP\_F8B.raw |  |  |  |  |  |  |  |  | 9.2596E4 |  | 1 | 0 | 0 | 0 | 0 | 0 | 0 | 0 | 0 | 1 | 0 | 109 | 119 |  |  | PEAKS DB |
| N.FVAQLASETLTK.S | Y | 57.28 | 1306.7133 | 12 | 0.6 | 654.3643 | 2 | 12.65 | 7 | F7:3384 | DaRuMP\_F7B.raw |  |  |  |  |  |  | 0 |  |  |  | 0 | 0 | 0 | 0 | 0 | 0 | 0 | 0 | 0 | 0 | 0 | 73 | 84 |  |  | PEAKS DB |
| F.VAQLASETLTK.S | Y | 56.21 | 1159.6448 | 11 | -0.3 | 580.8295 | 2 | 11.52 | 9 | F9:2387 | DaRuMP\_F8B.raw |  |  |  |  |  |  |  |  | 4.0729E5 |  | 1 | 0 | 0 | 0 | 0 | 0 | 0 | 0 | 0 | 1 | 0 | 74 | 84 |  |  | PEAKS DB |
| N.GWHLASIESVEEANFVAQLASETLTK.S | Y | 55.18 | 2829.4185 | 26 | 1.1 | 944.1478 | 3 | 52.79 | 8 | F8:23373 | DaRuMP\_F8A.raw |  |  |  |  |  |  |  | 1.272E6 |  |  | 1 | 0 | 0 | 0 | 0 | 0 | 0 | 0 | 1 | 0 | 0 | 59 | 84 |  |  | PEAKS DB |
| H.LASIESVEEANFVAQLASETLTK.S | Y | 52.55 | 2449.2588 | 23 | 0.7 | 1225.6376 | 2 | 54.62 | 9 | F9:17782 | DaRuMP\_F8B.raw |  |  |  |  |  |  |  |  | 0 |  | 0 | 0 | 0 | 0 | 0 | 0 | 0 | 0 | 0 | 0 | 0 | 62 | 84 |  |  | PEAKS DB |
| total 14 peptides |
| --- |

Q7LZ61|VM3CX\_DABSI

back to list

  

| Protein Coverage
| Supporting Peptides
|

Protein Coverage:

Supporting Peptides:

| Peptide | Uniq | -10lgP | Mass | Length | ppm | m/z | z | RT | Fraction | Scan | Source File | Area F1 | Area F10 | Area F2\_3 | Area F5 | Area F6 | Area F7A | Area F7B | Area F8A | Area F8B | Area F9 | #Feature | #Feature F1 | #Feature F10 | #Feature F2\_3 | #Feature F5 | #Feature F6 | #Feature F7A | #Feature F7B | #Feature F8A | #Feature F8B | #Feature F9 | Start | End | PTM | AScore | Found By |
| --- | --- | --- | --- | --- | --- | --- | --- | --- | --- | --- | --- | --- | --- | --- | --- | --- | --- | --- | --- | --- | --- | --- | --- | --- | --- | --- | --- | --- | --- | --- | --- | --- | --- | --- | --- | --- | --- |
| K.SHDNALLFTDMR.F | Y | 93.59 | 1418.6613 | 12 | 0.7 | 710.3384 | 2 | 12.35 | 9 | F9:3136 | DaRuMP\_F8B.raw |  |  |  |  |  |  |  |  | 3.9605E5 |  | 2 | 0 | 0 | 0 | 0 | 0 | 0 | 0 | 0 | 2 | 0 | 282 | 293 |  |  | PEAKS DB |
| L.ESGNVNDYEVVYPQK.V | N | 83.54 | 1739.8002 | 15 | 0.4 | 870.9077 | 2 | 11.80 | 3 | F3:2602 | DaRuMP\_F2\_F3.raw |  |  | 1.005E6 |  |  |  |  |  |  |  | 1 | 0 | 0 | 1 | 0 | 0 | 0 | 0 | 0 | 0 | 0 | 24 | 38 |  |  | PEAKS DB |
| K.LKPGAEC(+57.02)GNGLC(+57.02)C(+57.02)YQC(+57.02)K.I | Y | 80.83 | 2013.8529 | 17 | 1.3 | 672.2925 | 3 | 11.14 | 9 | F9:2005 | DaRuMP\_F8B.raw |  |  |  |  |  |  |  |  | 1.1977E6 |  | 2 | 0 | 0 | 0 | 0 | 0 | 0 | 0 | 0 | 2 | 0 | 435 | 451 | Carbamidomethylation | C7:Carbamidomethylation:1000.00;C12:Carbamidomethylation:1000.00;C13:Carbamidomethylation:1000.00;C16:Carbamidomethylation:1000.00 | PEAKS DB |
| R.FDLNTLGITFLAGMC(+57.02)QAYR.S | Y | 77.48 | 2190.0601 | 19 | 0.4 | 1096.0377 | 2 | 65.98 | 9 | F9:20545 | DaRuMP\_F8B.raw |  |  |  |  |  |  |  |  | 3.3808E5 |  | 1 | 0 | 0 | 0 | 0 | 0 | 0 | 0 | 0 | 1 | 0 | 294 | 312 | Carbamidomethylation | C15:Carbamidomethylation:1000.00 | PEAKS DB |
| R.GYC(+57.02)YNGDC(+57.02)PIMR.N | Y | 75.60 | 1504.5897 | 12 | 0.5 | 753.3025 | 2 | 11.76 | 9 | F9:2615 | DaRuMP\_F8B.raw |  |  |  |  |  |  |  |  | 4.0043E5 |  | 1 | 0 | 0 | 0 | 0 | 0 | 0 | 0 | 0 | 1 | 0 | 496 | 507 | Carbamidomethylation | C3:Carbamidomethylation:1000.00;C8:Carbamidomethylation:1000.00 | PEAKS DB |
| E.SGNVNDYEVVYPQK.V | N | 72.78 | 1610.7576 | 14 | 0.7 | 806.3866 | 2 | 11.74 | 3 | F3:2548 | DaRuMP\_F2\_F3.raw |  |  | 4.3606E4 |  |  |  |  |  |  |  | 1 | 0 | 0 | 1 | 0 | 0 | 0 | 0 | 0 | 0 | 0 | 25 | 38 |  |  | PEAKS DB |
| A.SQLVSTSAQFNK.I | Y | 72.35 | 1308.6674 | 12 | 0.4 | 655.3412 | 2 | 11.48 | 9 | F9:2333 | DaRuMP\_F8B.raw |  |  |  |  |  |  |  |  | 8.7552E5 |  | 1 | 0 | 0 | 0 | 0 | 0 | 0 | 0 | 0 | 1 | 0 | 187 | 198 |  |  | PEAKS DB |
| K.DSC(+57.02)FQENLK.G | Y | 72.03 | 1139.4917 | 9 | 1.0 | 570.7537 | 2 | 11.50 | 9 | F9:2343 | DaRuMP\_F8B.raw |  |  |  |  |  |  |  |  | 1.8605E6 | 5.3482E4 | 2 | 0 | 0 | 0 | 0 | 0 | 0 | 0 | 0 | 1 | 1 | 523 | 531 | Carbamidomethylation | C3:Carbamidomethylation:1000.00 | PEAKS DB |
| R.NQC(+57.02)ISLFGSR.A | N | 71.43 | 1180.5659 | 10 | 0.7 | 591.2906 | 2 | 16.62 | 8 | F8:3306 | DaRuMP\_F8A.raw |  | 1.4017E5 |  |  |  |  | 4.3071E5 | 2.9086E5 | 1.0773E7 | 2.2935E5 | 5 | 0 | 1 | 0 | 0 | 0 | 0 | 1 | 1 | 1 | 1 | 508 | 517 | Carbamidomethylation | C3:Carbamidomethylation:1000.00 | PEAKS DB |
| T.LIGVEFWC(+57.02)DR.D | Y | 70.87 | 1293.6176 | 10 | 0.9 | 647.8167 | 2 | 26.80 | 9 | F9:9950 | DaRuMP\_F8B.raw |  |  |  |  |  |  |  |  | 1.6962E6 |  | 1 | 0 | 0 | 0 | 0 | 0 | 0 | 0 | 0 | 1 | 0 | 244 | 253 | Carbamidomethylation | C8:Carbamidomethylation:1000.00 | PEAKS DB |
| K.GSYYGYC(+57.02)R.K | N | 66.12 | 1024.4072 | 8 | -0.3 | 513.2108 | 2 | 11.17 | 9 | F9:2047 | DaRuMP\_F8B.raw |  | 5.5048E3 |  |  |  |  |  |  | 3.3568E5 |  | 2 | 0 | 1 | 0 | 0 | 0 | 0 | 0 | 0 | 1 | 0 | 532 | 539 | Carbamidomethylation | C7:Carbamidomethylation:1000.00 | PEAKS DB |
| H.VTLIGVEFWC(+57.02)DR.D | Y | 66.06 | 1493.7336 | 12 | 0.6 | 747.8745 | 2 | 36.07 | 9 | F9:12740 | DaRuMP\_F8B.raw |  |  |  |  |  |  |  |  | 5.1358E5 |  | 1 | 0 | 0 | 0 | 0 | 0 | 0 | 0 | 0 | 1 | 0 | 242 | 253 | Carbamidomethylation | C10:Carbamidomethylation:1000.00 | PEAKS DB |
| K.IPC(+57.02)APQDVK.C | N | 63.97 | 1026.5168 | 9 | -0.1 | 514.2656 | 2 | 11.17 | 9 | F9:2029 | DaRuMP\_F8B.raw |  |  |  |  |  |  |  |  | 2.893E5 |  | 1 | 0 | 0 | 0 | 0 | 0 | 0 | 0 | 0 | 1 | 0 | 546 | 554 | Carbamidomethylation | C3:Carbamidomethylation:1000.00 | PEAKS DB |
| R.KIPC(+57.02)APQDVK.C | N | 63.12 | 1154.6117 | 10 | -0.4 | 578.3129 | 2 | 10.92 | 9 | F9:1781 | DaRuMP\_F8B.raw |  | 1.8307E4 |  |  |  |  |  |  | 7.6002E5 | 9.1773E4 | 4 | 0 | 1 | 0 | 0 | 0 | 0 | 0 | 0 | 2 | 1 | 545 | 554 | Carbamidomethylation | C4:Carbamidomethylation:1000.00 | PEAKS DB |
| R.DEC(+57.02)DVPEHC(+57.02)TGQSAEC(+57.02)PR.D | Y | 61.07 | 2145.8149 | 18 | 0.9 | 716.2795 | 3 | 10.99 | 9 | F9:1864 | DaRuMP\_F8B.raw |  |  |  |  |  |  |  |  | 3.1025E4 |  | 1 | 0 | 0 | 0 | 0 | 0 | 0 | 0 | 0 | 1 | 0 | 464 | 481 | Carbamidomethylation | C3:Carbamidomethylation:1000.00;C9:Carbamidomethylation:1000.00;C16:Carbamidomethylation:1000.00 | PEAKS DB |
| R.LFC(+57.02)LNNSPR.N | Y | 60.43 | 1119.5494 | 9 | 0.8 | 560.7825 | 2 | 11.96 | 9 | F9:2782 | DaRuMP\_F8B.raw |  |  |  |  |  |  |  |  | 2.642E6 |  | 1 | 0 | 0 | 0 | 0 | 0 | 0 | 0 | 0 | 1 | 0 | 558 | 566 | Carbamidomethylation | C3:Carbamidomethylation:1000.00 | PEAKS DB |
| N.QC(+57.02)ISLFGSR.A | N | 59.98 | 1066.5229 | 9 | 0.5 | 534.2690 | 2 | 12.78 | 2 | F2:3320 | DaRuMP\_F10.raw |  | 4.5112E4 |  |  |  |  |  |  |  |  | 1 | 0 | 1 | 0 | 0 | 0 | 0 | 0 | 0 | 0 | 0 | 509 | 517 | Carbamidomethylation | C2:Carbamidomethylation:1000.00 | PEAKS DB |
| K.TAVIMAHELSH.N | Y | 57.24 | 1207.6019 | 11 | 0.4 | 604.8085 | 2 | 11.44 | 9 | F9:2301 | DaRuMP\_F8B.raw |  |  |  |  |  |  |  |  | 8.7084E4 |  | 1 | 0 | 0 | 0 | 0 | 0 | 0 | 0 | 0 | 1 | 0 | 327 | 337 |  |  | PEAKS DB |
| total 18 peptides |
| --- |

A8CG87|PA2A2\_DABRR

back to list

  

| Protein Coverage
| Supporting Peptides
|

Protein Coverage:

Supporting Peptides:

| Peptide | Uniq | -10lgP | Mass | Length | ppm | m/z | z | RT | Fraction | Scan | Source File | Area F1 | Area F10 | Area F2\_3 | Area F5 | Area F6 | Area F7A | Area F7B | Area F8A | Area F8B | Area F9 | #Feature | #Feature F1 | #Feature F10 | #Feature F2\_3 | #Feature F5 | #Feature F6 | #Feature F7A | #Feature F7B | #Feature F8A | #Feature F8B | #Feature F9 | Start | End | PTM | AScore | Found By |
| --- | --- | --- | --- | --- | --- | --- | --- | --- | --- | --- | --- | --- | --- | --- | --- | --- | --- | --- | --- | --- | --- | --- | --- | --- | --- | --- | --- | --- | --- | --- | --- | --- | --- | --- | --- | --- | --- |
| K.TATYSYSFENGDIVC(+57.02)GGDDPC(+57.02)LR.A | Y | 90.28 | 2596.0845 | 23 | -0.3 | 1299.0491 | 2 | 18.19 | 5 | F5:7942 | DaRuMP\_F6.raw |  |  |  |  | 3.9602E8 |  |  |  |  |  | 3 | 0 | 0 | 0 | 0 | 3 | 0 | 0 | 0 | 0 | 0 | 77 | 99 | Carbamidomethylation | C15:Carbamidomethylation:1000.00;C21:Carbamidomethylation:1000.00 | PEAKS DB |
| Y.SFENGDIVC(+57.02)GGDDPC(+57.02)LR.A | N | 76.10 | 1909.7935 | 17 | -0.8 | 955.9033 | 2 | 12.50 | 5 | F5:3255 | DaRuMP\_F6.raw |  |  |  |  | 8.2046E7 |  |  |  |  |  | 3 | 0 | 0 | 0 | 0 | 3 | 0 | 0 | 0 | 0 | 0 | 83 | 99 | Carbamidomethylation | C9:Carbamidomethylation:1000.00;C15:Carbamidomethylation:1000.00 | PEAKS DB |
| Y.VYYGC(+57.02)YC(+57.02)GWGGK.G | Y | 74.47 | 1468.5903 | 12 | 1.2 | 735.3033 | 2 | 12.07 | 5 | F5:2870 | DaRuMP\_F6.raw |  |  |  |  | 6.7447E7 |  |  |  |  |  | 1 | 0 | 0 | 0 | 0 | 1 | 0 | 0 | 0 | 0 | 0 | 38 | 49 | Carbamidomethylation | C5:Carbamidomethylation:1000.00;C7:Carbamidomethylation:1000.00 | PEAKS DB |
| D.IVC(+57.02)GGDDPC(+57.02)LR.A | N | 72.92 | 1260.5591 | 11 | -0.7 | 631.2864 | 2 | 11.33 | 5 | F5:2185 | DaRuMP\_F6.raw |  |  |  |  | 1.5095E6 |  |  |  |  |  | 1 | 0 | 0 | 0 | 0 | 1 | 0 | 0 | 0 | 0 | 0 | 89 | 99 | Carbamidomethylation | C3:Carbamidomethylation:1000.00;C9:Carbamidomethylation:1000.00 | PEAKS DB |
| K.YM(+15.99)LYSIFDC(+57.02)K.E | N | 72.09 | 1354.5938 | 10 | 0.3 | 678.3044 | 2 | 22.06 | 5 | F5:10148 | DaRuMP\_F6.raw |  |  |  |  | 1.3733E7 |  |  |  |  |  | 3 | 0 | 0 | 0 | 0 | 3 | 0 | 0 | 0 | 0 | 0 | 123 | 132 | Oxidation (M); Carbamidomethylation | M2:Oxidation (M):1000.00;C9:Carbamidomethylation:1000.00 | PEAKS DB |
| Y.SYSFENGDIVC(+57.02)GGDDPC(+57.02)LR.A | N | 70.84 | 2159.8887 | 19 | 0.7 | 1080.9524 | 2 | 15.53 | 5 | F5:5583 | DaRuMP\_F6.raw |  |  |  |  | 3.9345E7 |  |  |  |  |  | 2 | 0 | 0 | 0 | 0 | 2 | 0 | 0 | 0 | 0 | 0 | 81 | 99 | Carbamidomethylation | C11:Carbamidomethylation:1000.00;C17:Carbamidomethylation:1000.00 | PEAKS DB |
| K.YMLYSIFDC(+57.02)K.E | N | 68.36 | 1338.5988 | 10 | 0.5 | 670.3070 | 2 | 28.05 | 5 | F5:12336 | DaRuMP\_F6.raw |  |  |  |  | 1.8104E7 |  |  |  |  |  | 3 | 0 | 0 | 0 | 0 | 3 | 0 | 0 | 0 | 0 | 0 | 123 | 132 | Carbamidomethylation | C9:Carbamidomethylation:1000.00 | PEAKS DB |
| F.ENGDIVC(+57.02)GGDDPC(+57.02)LR.A | N | 67.14 | 1675.6930 | 15 | -0.5 | 838.8534 | 2 | 11.72 | 5 | F5:2585 | DaRuMP\_F6.raw |  |  |  |  | 2.9876E5 |  |  |  |  |  | 1 | 0 | 0 | 0 | 0 | 1 | 0 | 0 | 0 | 0 | 0 | 85 | 99 | Carbamidomethylation | C7:Carbamidomethylation:1000.00;C13:Carbamidomethylation:1000.00 | PEAKS DB |
| Y.GC(+57.02)YC(+57.02)GWGGK.G | N | 64.20 | 1043.3953 | 9 | 3.2 | 522.7053 | 2 | 11.29 | 6 | F6:2116 | DaRuMP\_F7A.raw |  |  | 6.3696E4 |  | 1.7244E6 | 2.9315E6 | 3.3122E5 |  |  | 4.6531E4 | 5 | 0 | 0 | 1 | 0 | 1 | 1 | 1 | 0 | 0 | 1 | 41 | 49 | Carbamidomethylation | C2:Carbamidomethylation:1000.00;C4:Carbamidomethylation:1000.00 | PEAKS DB |
| G.NLYQFGEMINQK.T | Y | 63.22 | 1483.7129 | 12 | 0.6 | 742.8641 | 2 | 17.07 | 5 | F5:6874 | DaRuMP\_F6.raw |  |  |  |  | 7.0654E7 |  |  |  |  |  | 1 | 0 | 0 | 0 | 0 | 1 | 0 | 0 | 0 | 0 | 0 | 17 | 28 |  |  | PEAKS DB |
| L.SYVYYGC(+57.02)YC(+57.02)GWGGK.G | Y | 62.77 | 1718.6858 | 14 | 1.1 | 860.3511 | 2 | 14.05 | 5 | F5:4860 | DaRuMP\_F6.raw |  |  |  |  | 3.1276E7 |  |  |  |  |  | 1 | 0 | 0 | 0 | 0 | 1 | 0 | 0 | 0 | 0 | 0 | 36 | 49 | Carbamidomethylation | C7:Carbamidomethylation:1000.00;C9:Carbamidomethylation:1000.00 | PEAKS DB |
| V.YYGC(+57.02)YC(+57.02)GWGGK.G | N | 61.96 | 1369.5220 | 11 | 0.4 | 685.7686 | 2 | 11.84 | 5 | F5:2680 | DaRuMP\_F6.raw |  |  |  |  | 1.0137E6 |  |  |  |  |  | 1 | 0 | 0 | 0 | 0 | 1 | 0 | 0 | 0 | 0 | 0 | 39 | 49 | Carbamidomethylation | C4:Carbamidomethylation:1000.00;C6:Carbamidomethylation:1000.00 | PEAKS DB |
| S.YSFENGDIVC(+57.02)GGDDPC(+57.02)LR.A | N | 61.91 | 2072.8567 | 18 | 0.8 | 1037.4364 | 2 | 15.49 | 5 | F5:5628 | DaRuMP\_F6.raw |  |  |  |  | 2.9315E6 |  |  |  |  |  | 1 | 0 | 0 | 0 | 0 | 1 | 0 | 0 | 0 | 0 | 0 | 82 | 99 | Carbamidomethylation | C10:Carbamidomethylation:1000.00;C16:Carbamidomethylation:1000.00 | PEAKS DB |
| G.NLYQFGEM(+15.99)INQK.T | Y | 61.51 | 1499.7079 | 12 | 1.1 | 750.8621 | 2 | 12.38 | 5 | F5:3168 | DaRuMP\_F6.raw |  |  |  |  | 8.9437E6 |  |  |  |  |  | 2 | 0 | 0 | 0 | 0 | 2 | 0 | 0 | 0 | 0 | 0 | 17 | 28 | Oxidation (M) | M8:Oxidation (M):1000.00 | PEAKS DB |
| R.C(+57.02)C(+57.02)FVHDC(+57.02)C(+57.02)YGR.V | N | 60.45 | 1532.5417 | 11 | 0.1 | 767.2782 | 2 | 11.12 | 5 | F5:1975 | DaRuMP\_F6.raw |  |  |  | 2.7083E5 | 1.1953E8 |  |  |  |  |  | 3 | 0 | 0 | 0 | 1 | 2 | 0 | 0 | 0 | 0 | 0 | 59 | 69 | Carbamidomethylation | C1:Carbamidomethylation:1000.00;C2:Carbamidomethylation:1000.00;C7:Carbamidomethylation:1000.00;C8:Carbamidomethylation:1000.00 | PEAKS DB |
| S.FENGDIVC(+57.02)GGDDPC(+57.02)LR.A | N | 60.39 | 1822.7614 | 16 | 0.0 | 912.3879 | 2 | 12.13 | 5 | F5:2950 | DaRuMP\_F6.raw |  |  |  |  | 6.8459E5 |  |  |  |  |  | 1 | 0 | 0 | 0 | 0 | 1 | 0 | 0 | 0 | 0 | 0 | 84 | 99 | Carbamidomethylation | C8:Carbamidomethylation:1000.00;C14:Carbamidomethylation:1000.00 | PEAKS DB |
| N.GDIVC(+57.02)GGDDPC(+57.02)LR.A | N | 59.01 | 1432.6074 | 13 | 0.4 | 717.3113 | 2 | 11.65 | 5 | F5:2512 | DaRuMP\_F6.raw |  |  |  |  | 1.6133E6 |  |  |  |  |  | 1 | 0 | 0 | 0 | 0 | 1 | 0 | 0 | 0 | 0 | 0 | 87 | 99 | Carbamidomethylation | C5:Carbamidomethylation:1000.00;C11:Carbamidomethylation:1000.00 | PEAKS DB |
| Y.YGC(+57.02)YC(+57.02)GWGGK.G | N | 58.24 | 1206.4586 | 10 | 0.0 | 604.2366 | 2 | 11.59 | 5 | F5:2425 | DaRuMP\_F6.raw |  |  |  |  | 1.3442E7 |  |  |  |  |  | 1 | 0 | 0 | 0 | 0 | 1 | 0 | 0 | 0 | 0 | 0 | 40 | 49 | Carbamidomethylation | C3:Carbamidomethylation:1000.00;C5:Carbamidomethylation:1000.00 | PEAKS DB |
| E.GNLYQFGEMINQK.T | Y | 55.11 | 1540.7344 | 13 | 0.9 | 771.3751 | 2 | 20.94 | 5 | F5:9545 | DaRuMP\_F6.raw |  |  |  |  | 1.1672E7 |  |  |  |  |  | 1 | 0 | 0 | 0 | 0 | 1 | 0 | 0 | 0 | 0 | 0 | 16 | 28 |  |  | PEAKS DB |
| R.VAAIC(+57.02)FR.E | N | 54.87 | 835.4374 | 7 | 2.9 | 418.7262 | 2 | 11.77 | 6 | F6:2551 | DaRuMP\_F7A.raw |  |  |  |  | 0 | 3.5471E6 |  |  |  |  | 1 | 0 | 0 | 0 | 0 | 0 | 1 | 0 | 0 | 0 | 0 | 107 | 113 | Carbamidomethylation | C5:Carbamidomethylation:1000.00 | PEAKS DB |
| total 20 peptides |
| --- |

A8CG78|PA2A2\_DABSI

back to list

  

| Protein Coverage
| Supporting Peptides
|

Protein Coverage:

Supporting Peptides:

| Peptide | Uniq | -10lgP | Mass | Length | ppm | m/z | z | RT | Fraction | Scan | Source File | Area F1 | Area F10 | Area F2\_3 | Area F5 | Area F6 | Area F7A | Area F7B | Area F8A | Area F8B | Area F9 | #Feature | #Feature F1 | #Feature F10 | #Feature F2\_3 | #Feature F5 | #Feature F6 | #Feature F7A | #Feature F7B | #Feature F8A | #Feature F8B | #Feature F9 | Start | End | PTM | AScore | Found By |
| --- | --- | --- | --- | --- | --- | --- | --- | --- | --- | --- | --- | --- | --- | --- | --- | --- | --- | --- | --- | --- | --- | --- | --- | --- | --- | --- | --- | --- | --- | --- | --- | --- | --- | --- | --- | --- | --- |
| K.TATYSYSFENGDIVC(+57.02)GGDDPC(+57.02)LR.A | Y | 90.28 | 2596.0845 | 23 | -0.3 | 1299.0491 | 2 | 18.19 | 5 | F5:7942 | DaRuMP\_F6.raw |  |  |  |  | 3.9602E8 |  |  |  |  |  | 3 | 0 | 0 | 0 | 0 | 3 | 0 | 0 | 0 | 0 | 0 | 77 | 99 | Carbamidomethylation | C15:Carbamidomethylation:1000.00;C21:Carbamidomethylation:1000.00 | PEAKS DB |
| Y.SFENGDIVC(+57.02)GGDDPC(+57.02)LR.A | N | 76.10 | 1909.7935 | 17 | -0.8 | 955.9033 | 2 | 12.50 | 5 | F5:3255 | DaRuMP\_F6.raw |  |  |  |  | 8.2046E7 |  |  |  |  |  | 3 | 0 | 0 | 0 | 0 | 3 | 0 | 0 | 0 | 0 | 0 | 83 | 99 | Carbamidomethylation | C9:Carbamidomethylation:1000.00;C15:Carbamidomethylation:1000.00 | PEAKS DB |
| Y.VYYGC(+57.02)YC(+57.02)GWGGK.G | Y | 74.47 | 1468.5903 | 12 | 1.2 | 735.3033 | 2 | 12.07 | 5 | F5:2870 | DaRuMP\_F6.raw |  |  |  |  | 6.7447E7 |  |  |  |  |  | 1 | 0 | 0 | 0 | 0 | 1 | 0 | 0 | 0 | 0 | 0 | 38 | 49 | Carbamidomethylation | C5:Carbamidomethylation:1000.00;C7:Carbamidomethylation:1000.00 | PEAKS DB |
| D.IVC(+57.02)GGDDPC(+57.02)LR.A | N | 72.92 | 1260.5591 | 11 | -0.7 | 631.2864 | 2 | 11.33 | 5 | F5:2185 | DaRuMP\_F6.raw |  |  |  |  | 1.5095E6 |  |  |  |  |  | 1 | 0 | 0 | 0 | 0 | 1 | 0 | 0 | 0 | 0 | 0 | 89 | 99 | Carbamidomethylation | C3:Carbamidomethylation:1000.00;C9:Carbamidomethylation:1000.00 | PEAKS DB |
| K.YM(+15.99)LYSIFDC(+57.02)K.E | N | 72.09 | 1354.5938 | 10 | 0.3 | 678.3044 | 2 | 22.06 | 5 | F5:10148 | DaRuMP\_F6.raw |  |  |  |  | 1.3733E7 |  |  |  |  |  | 3 | 0 | 0 | 0 | 0 | 3 | 0 | 0 | 0 | 0 | 0 | 123 | 132 | Oxidation (M); Carbamidomethylation | M2:Oxidation (M):1000.00;C9:Carbamidomethylation:1000.00 | PEAKS DB |
| Y.SYSFENGDIVC(+57.02)GGDDPC(+57.02)LR.A | N | 70.84 | 2159.8887 | 19 | 0.7 | 1080.9524 | 2 | 15.53 | 5 | F5:5583 | DaRuMP\_F6.raw |  |  |  |  | 3.9345E7 |  |  |  |  |  | 2 | 0 | 0 | 0 | 0 | 2 | 0 | 0 | 0 | 0 | 0 | 81 | 99 | Carbamidomethylation | C11:Carbamidomethylation:1000.00;C17:Carbamidomethylation:1000.00 | PEAKS DB |
| K.YMLYSIFDC(+57.02)K.E | N | 68.36 | 1338.5988 | 10 | 0.5 | 670.3070 | 2 | 28.05 | 5 | F5:12336 | DaRuMP\_F6.raw |  |  |  |  | 1.8104E7 |  |  |  |  |  | 3 | 0 | 0 | 0 | 0 | 3 | 0 | 0 | 0 | 0 | 0 | 123 | 132 | Carbamidomethylation | C9:Carbamidomethylation:1000.00 | PEAKS DB |
| F.ENGDIVC(+57.02)GGDDPC(+57.02)LR.A | N | 67.14 | 1675.6930 | 15 | -0.5 | 838.8534 | 2 | 11.72 | 5 | F5:2585 | DaRuMP\_F6.raw |  |  |  |  | 2.9876E5 |  |  |  |  |  | 1 | 0 | 0 | 0 | 0 | 1 | 0 | 0 | 0 | 0 | 0 | 85 | 99 | Carbamidomethylation | C7:Carbamidomethylation:1000.00;C13:Carbamidomethylation:1000.00 | PEAKS DB |
| Y.GC(+57.02)YC(+57.02)GWGGK.G | N | 64.20 | 1043.3953 | 9 | 3.2 | 522.7053 | 2 | 11.29 | 6 | F6:2116 | DaRuMP\_F7A.raw |  |  | 6.3696E4 |  | 1.7244E6 | 2.9315E6 | 3.3122E5 |  |  | 4.6531E4 | 5 | 0 | 0 | 1 | 0 | 1 | 1 | 1 | 0 | 0 | 1 | 41 | 49 | Carbamidomethylation | C2:Carbamidomethylation:1000.00;C4:Carbamidomethylation:1000.00 | PEAKS DB |
| G.NLYQFGEMINQK.T | Y | 63.22 | 1483.7129 | 12 | 0.6 | 742.8641 | 2 | 17.07 | 5 | F5:6874 | DaRuMP\_F6.raw |  |  |  |  | 7.0654E7 |  |  |  |  |  | 1 | 0 | 0 | 0 | 0 | 1 | 0 | 0 | 0 | 0 | 0 | 17 | 28 |  |  | PEAKS DB |
| L.SYVYYGC(+57.02)YC(+57.02)GWGGK.G | Y | 62.77 | 1718.6858 | 14 | 1.1 | 860.3511 | 2 | 14.05 | 5 | F5:4860 | DaRuMP\_F6.raw |  |  |  |  | 3.1276E7 |  |  |  |  |  | 1 | 0 | 0 | 0 | 0 | 1 | 0 | 0 | 0 | 0 | 0 | 36 | 49 | Carbamidomethylation | C7:Carbamidomethylation:1000.00;C9:Carbamidomethylation:1000.00 | PEAKS DB |
| V.YYGC(+57.02)YC(+57.02)GWGGK.G | N | 61.96 | 1369.5220 | 11 | 0.4 | 685.7686 | 2 | 11.84 | 5 | F5:2680 | DaRuMP\_F6.raw |  |  |  |  | 1.0137E6 |  |  |  |  |  | 1 | 0 | 0 | 0 | 0 | 1 | 0 | 0 | 0 | 0 | 0 | 39 | 49 | Carbamidomethylation | C4:Carbamidomethylation:1000.00;C6:Carbamidomethylation:1000.00 | PEAKS DB |
| S.YSFENGDIVC(+57.02)GGDDPC(+57.02)LR.A | N | 61.91 | 2072.8567 | 18 | 0.8 | 1037.4364 | 2 | 15.49 | 5 | F5:5628 | DaRuMP\_F6.raw |  |  |  |  | 2.9315E6 |  |  |  |  |  | 1 | 0 | 0 | 0 | 0 | 1 | 0 | 0 | 0 | 0 | 0 | 82 | 99 | Carbamidomethylation | C10:Carbamidomethylation:1000.00;C16:Carbamidomethylation:1000.00 | PEAKS DB |
| G.NLYQFGEM(+15.99)INQK.T | Y | 61.51 | 1499.7079 | 12 | 1.1 | 750.8621 | 2 | 12.38 | 5 | F5:3168 | DaRuMP\_F6.raw |  |  |  |  | 8.9437E6 |  |  |  |  |  | 2 | 0 | 0 | 0 | 0 | 2 | 0 | 0 | 0 | 0 | 0 | 17 | 28 | Oxidation (M) | M8:Oxidation (M):1000.00 | PEAKS DB |
| R.C(+57.02)C(+57.02)FVHDC(+57.02)C(+57.02)YGR.V | N | 60.45 | 1532.5417 | 11 | 0.1 | 767.2782 | 2 | 11.12 | 5 | F5:1975 | DaRuMP\_F6.raw |  |  |  | 2.7083E5 | 1.1953E8 |  |  |  |  |  | 3 | 0 | 0 | 0 | 1 | 2 | 0 | 0 | 0 | 0 | 0 | 59 | 69 | Carbamidomethylation | C1:Carbamidomethylation:1000.00;C2:Carbamidomethylation:1000.00;C7:Carbamidomethylation:1000.00;C8:Carbamidomethylation:1000.00 | PEAKS DB |
| S.FENGDIVC(+57.02)GGDDPC(+57.02)LR.A | N | 60.39 | 1822.7614 | 16 | 0.0 | 912.3879 | 2 | 12.13 | 5 | F5:2950 | DaRuMP\_F6.raw |  |  |  |  | 6.8459E5 |  |  |  |  |  | 1 | 0 | 0 | 0 | 0 | 1 | 0 | 0 | 0 | 0 | 0 | 84 | 99 | Carbamidomethylation | C8:Carbamidomethylation:1000.00;C14:Carbamidomethylation:1000.00 | PEAKS DB |
| N.GDIVC(+57.02)GGDDPC(+57.02)LR.A | N | 59.01 | 1432.6074 | 13 | 0.4 | 717.3113 | 2 | 11.65 | 5 | F5:2512 | DaRuMP\_F6.raw |  |  |  |  | 1.6133E6 |  |  |  |  |  | 1 | 0 | 0 | 0 | 0 | 1 | 0 | 0 | 0 | 0 | 0 | 87 | 99 | Carbamidomethylation | C5:Carbamidomethylation:1000.00;C11:Carbamidomethylation:1000.00 | PEAKS DB |
| Y.YGC(+57.02)YC(+57.02)GWGGK.G | N | 58.24 | 1206.4586 | 10 | 0.0 | 604.2366 | 2 | 11.59 | 5 | F5:2425 | DaRuMP\_F6.raw |  |  |  |  | 1.3442E7 |  |  |  |  |  | 1 | 0 | 0 | 0 | 0 | 1 | 0 | 0 | 0 | 0 | 0 | 40 | 49 | Carbamidomethylation | C3:Carbamidomethylation:1000.00;C5:Carbamidomethylation:1000.00 | PEAKS DB |
| E.GNLYQFGEMINQK.T | Y | 55.11 | 1540.7344 | 13 | 0.9 | 771.3751 | 2 | 20.94 | 5 | F5:9545 | DaRuMP\_F6.raw |  |  |  |  | 1.1672E7 |  |  |  |  |  | 1 | 0 | 0 | 0 | 0 | 1 | 0 | 0 | 0 | 0 | 0 | 16 | 28 |  |  | PEAKS DB |
| R.VAAIC(+57.02)FR.E | N | 54.87 | 835.4374 | 7 | 2.9 | 418.7262 | 2 | 11.77 | 6 | F6:2551 | DaRuMP\_F7A.raw |  |  |  |  | 0 | 3.5471E6 |  |  |  |  | 1 | 0 | 0 | 0 | 0 | 0 | 1 | 0 | 0 | 0 | 0 | 107 | 113 | Carbamidomethylation | C5:Carbamidomethylation:1000.00 | PEAKS DB |
| total 20 peptides |
| --- |

A8CG82|PA2B1\_DABSI

back to list

  

| Protein Coverage
| Supporting Peptides
|

Protein Coverage:

Supporting Peptides:

| Peptide | Uniq | -10lgP | Mass | Length | ppm | m/z | z | RT | Fraction | Scan | Source File | Area F1 | Area F10 | Area F2\_3 | Area F5 | Area F6 | Area F7A | Area F7B | Area F8A | Area F8B | Area F9 | #Feature | #Feature F1 | #Feature F10 | #Feature F2\_3 | #Feature F5 | #Feature F6 | #Feature F7A | #Feature F7B | #Feature F8A | #Feature F8B | #Feature F9 | Start | End | PTM | AScore | Found By |
| --- | --- | --- | --- | --- | --- | --- | --- | --- | --- | --- | --- | --- | --- | --- | --- | --- | --- | --- | --- | --- | --- | --- | --- | --- | --- | --- | --- | --- | --- | --- | --- | --- | --- | --- | --- | --- | --- |
| K.YISYGC(+57.02)YC(+57.02)GWGGQGTPK.D | N | 103.43 | 1952.8185 | 17 | 2.3 | 977.4164 | 2 | 12.58 | 6 | F6:3227 | DaRuMP\_F7A.raw |  |  |  |  | 2.2395E5 | 4.299E7 | 9.7922E5 |  |  |  | 4 | 0 | 0 | 0 | 0 | 1 | 2 | 1 | 0 | 0 | 0 | 37 | 53 | Carbamidomethylation | C6:Carbamidomethylation:1000.00;C8:Carbamidomethylation:1000.00 | PEAKS DB |
| R.VAAIC(+57.02)LGQNVNTYNK.G | N | 100.11 | 1663.8352 | 15 | 1.0 | 832.9257 | 2 | 11.96 | 9 | F9:2785 | DaRuMP\_F8B.raw |  |  |  |  | 4.7724E5 | 4.6856E7 | 1.6056E6 |  | 4.2087E5 |  | 4 | 0 | 0 | 0 | 0 | 1 | 1 | 1 | 0 | 1 | 0 | 107 | 121 | Carbamidomethylation | C5:Carbamidomethylation:1000.00 | PEAKS DB |
| R.VAAIC(+57.02)LGQNVNTYNKG.Y | N | 89.72 | 1720.8567 | 16 | 3.4 | 861.4365 | 2 | 12.08 | 6 | F6:2676 | DaRuMP\_F7A.raw |  |  |  |  |  | 1.1354E7 |  |  |  |  | 1 | 0 | 0 | 0 | 0 | 0 | 1 | 0 | 0 | 0 | 0 | 107 | 122 | Carbamidomethylation | C5:Carbamidomethylation:1000.00 | PEAKS DB |
| Y.GC(+57.02)YC(+57.02)GWGGQGTPK.D | N | 76.61 | 1426.5758 | 13 | 2.9 | 714.2955 | 2 | 11.41 | 6 | F6:2230 | DaRuMP\_F7A.raw |  |  |  |  |  | 1.1168E5 |  |  |  |  | 1 | 0 | 0 | 0 | 0 | 0 | 1 | 0 | 0 | 0 | 0 | 41 | 53 | Carbamidomethylation | C2:Carbamidomethylation:1000.00;C4:Carbamidomethylation:1000.00 | PEAKS DB |
| Y.ISYGC(+57.02)YC(+57.02)GWGGQGTPK.D | N | 76.20 | 1789.7552 | 16 | 3.6 | 895.8859 | 2 | 11.98 | 6 | F6:2768 | DaRuMP\_F7A.raw |  |  |  |  |  | 4.0004E5 |  |  |  |  | 1 | 0 | 0 | 0 | 0 | 0 | 1 | 0 | 0 | 0 | 0 | 38 | 53 | Carbamidomethylation | C5:Carbamidomethylation:1000.00;C7:Carbamidomethylation:1000.00 | PEAKS DB |
| R.C(+57.02)C(+57.02)FVHDC(+57.02)C(+57.02)YAR.V | N | 75.28 | 1546.5574 | 11 | 1.6 | 774.2853 | 2 | 11.10 | 6 | F6:1930 | DaRuMP\_F7A.raw |  |  |  |  |  | 2.7639E6 | 1.6247E5 |  |  |  | 3 | 0 | 0 | 0 | 0 | 0 | 2 | 1 | 0 | 0 | 0 | 59 | 69 | Carbamidomethylation | C1:Carbamidomethylation:1000.00;C2:Carbamidomethylation:1000.00;C7:Carbamidomethylation:1000.00;C8:Carbamidomethylation:1000.00 | PEAKS DB |
| K.GYMFLSSYYC(+57.02)R.Q | N | 73.97 | 1445.6107 | 11 | 2.8 | 723.8129 | 2 | 20.17 | 6 | F6:9319 | DaRuMP\_F7A.raw |  |  |  |  |  | 1.0534E6 |  |  |  |  | 1 | 0 | 0 | 0 | 0 | 0 | 1 | 0 | 0 | 0 | 0 | 122 | 132 | Carbamidomethylation | C10:Carbamidomethylation:1000.00 | PEAKS DB |
| K.IVC(+57.02)ENYNR.C | Y | 70.52 | 1066.4866 | 8 | 1.7 | 534.2502 | 2 | 10.76 | 6 | F6:1638 | DaRuMP\_F7A.raw |  |  |  |  |  | 3.9936E5 |  |  |  |  | 1 | 0 | 0 | 0 | 0 | 0 | 1 | 0 | 0 | 0 | 0 | 89 | 96 | Carbamidomethylation | C3:Carbamidomethylation:1000.00 | PEAKS DB |
| K.QEAFSFFK.Y | N | 61.68 | 1002.4810 | 8 | 3.1 | 502.2481 | 2 | 17.34 | 6 | F6:7044 | DaRuMP\_F7A.raw |  |  |  |  |  | 3.1285E5 |  |  |  |  | 1 | 0 | 0 | 0 | 0 | 0 | 1 | 0 | 0 | 0 | 0 | 29 | 36 |  |  | PEAKS DB |
| R.VAAIC(+57.02)LGQNVNTY.N | N | 55.83 | 1421.6973 | 13 | 4.2 | 711.8572 | 2 | 18.78 | 6 | F6:8560 | DaRuMP\_F7A.raw |  |  |  |  |  | 8.6437E6 |  |  |  |  | 1 | 0 | 0 | 0 | 0 | 0 | 1 | 0 | 0 | 0 | 0 | 107 | 119 | Carbamidomethylation | C5:Carbamidomethylation:1000.00 | PEAKS DB |
| total 10 peptides |
| --- |

P18965|VSPG\_DABSI

back to list

  

| Protein Coverage
| Supporting Peptides
|

Protein Coverage:

Supporting Peptides:

| Peptide | Uniq | -10lgP | Mass | Length | ppm | m/z | z | RT | Fraction | Scan | Source File | Area F1 | Area F10 | Area F2\_3 | Area F5 | Area F6 | Area F7A | Area F7B | Area F8A | Area F8B | Area F9 | #Feature | #Feature F1 | #Feature F10 | #Feature F2\_3 | #Feature F5 | #Feature F6 | #Feature F7A | #Feature F7B | #Feature F8A | #Feature F8B | #Feature F9 | Start | End | PTM | AScore | Found By |
| --- | --- | --- | --- | --- | --- | --- | --- | --- | --- | --- | --- | --- | --- | --- | --- | --- | --- | --- | --- | --- | --- | --- | --- | --- | --- | --- | --- | --- | --- | --- | --- | --- | --- | --- | --- | --- | --- |
| L.VVGGDEC(+57.02)NINEHPFLVALYTSASSTIHC(+57.02)AGALINR.E | Y | 112.64 | 3784.8301 | 35 | 2.5 | 947.2148 | 4 | 45.32 | 6 | F6:23143 | DaRuMP\_F7A.raw |  |  |  |  |  | 3.0131E6 |  |  |  |  | 1 | 0 | 0 | 0 | 0 | 0 | 1 | 0 | 0 | 0 | 0 | 25 | 59 | Carbamidomethylation | C7:Carbamidomethylation:1000.00;C28:Carbamidomethylation:1000.00 | PEAKS DB |
| K.ISTTEDTYPDVPHC(+57.02)TNIFIVK.H | N | 82.13 | 2449.1836 | 21 | 3.7 | 817.4028 | 3 | 16.70 | 6 | F6:6525 | DaRuMP\_F7A.raw |  |  |  |  |  | 7.9072E6 | 2.9622E6 |  |  |  | 2 | 0 | 0 | 0 | 0 | 0 | 1 | 1 | 0 | 0 | 0 | 152 | 172 | Carbamidomethylation | C14:Carbamidomethylation:1000.00 | PEAKS DB |
| R.EWVLTAAHC(+57.02)DR.R | N | 78.50 | 1356.6245 | 11 | 3.6 | 679.3203 | 2 | 11.66 | 6 | F6:2454 | DaRuMP\_F7A.raw |  |  |  |  | 7.9894E6 | 8.3434E5 | 8.335E5 |  | 1.6664E5 |  | 7 | 0 | 0 | 0 | 0 | 2 | 2 | 2 | 0 | 1 | 0 | 60 | 70 | Carbamidomethylation | C9:Carbamidomethylation:1000.00 | PEAKS DB |
| K.ISTTEDTYPDVPHC(+57.02)TN.I | N | 75.52 | 1848.7836 | 16 | -0.4 | 925.3987 | 2 | 11.61 | 5 | F5:2486 | DaRuMP\_F6.raw |  |  |  |  | 2.5361E6 | 1.7964E5 |  |  | 8.2217E4 |  | 3 | 0 | 0 | 0 | 0 | 1 | 1 | 0 | 0 | 1 | 0 | 152 | 167 | Carbamidomethylation | C14:Carbamidomethylation:1000.00 | PEAKS DB |
| R.RPVTYSTHIAPVSLPSR.S | N | 75.09 | 1880.0267 | 17 | 2.9 | 627.6832 | 3 | 11.52 | 6 | F6:2353 | DaRuMP\_F7A.raw |  |  |  |  | 3.1226E6 | 4.0742E5 | 2.5159E5 |  |  |  | 4 | 0 | 0 | 0 | 0 | 2 | 1 | 1 | 0 | 0 | 0 | 120 | 136 |  |  | PEAKS DB |
| Y.STHIAPVSLPSR.S | N | 70.68 | 1263.6935 | 12 | 0.5 | 632.8543 | 2 | 11.47 | 5 | F5:2315 | DaRuMP\_F6.raw |  |  |  |  | 4.3808E6 | 5.7195E5 | 2.2102E5 |  |  |  | 5 | 0 | 0 | 0 | 0 | 2 | 2 | 1 | 0 | 0 | 0 | 125 | 136 |  |  | PEAKS DB |
| K.WC(+57.02)EPLYPWVPADSR.T | N | 59.36 | 1774.8137 | 14 | 2.9 | 888.4146 | 2 | 30.30 | 6 | F6:15902 | DaRuMP\_F7A.raw |  |  |  |  | 1.9987E6 | 1.1273E6 |  |  |  |  | 2 | 0 | 0 | 0 | 0 | 1 | 1 | 0 | 0 | 0 | 0 | 175 | 188 | Carbamidomethylation | C2:Carbamidomethylation:1000.00 | PEAKS DB |
| K.HKWC(+57.02)EPLYPWVPADSR.T | N | 57.24 | 2039.9675 | 16 | 0.7 | 680.9952 | 3 | 13.13 | 6 | F6:3711 | DaRuMP\_F7A.raw |  |  |  |  |  | 3.3023E5 |  |  |  |  | 1 | 0 | 0 | 0 | 0 | 0 | 1 | 0 | 0 | 0 | 0 | 173 | 188 | Carbamidomethylation | C4:Carbamidomethylation:1000.00 | PEAKS DB |
| R.TLC(+57.02)AGILK.G | N | 56.73 | 874.4946 | 8 | 0.1 | 438.2546 | 2 | 11.83 | 9 | F9:2657 | DaRuMP\_F8B.raw |  |  |  |  | 7.6058E6 | 1.0726E6 | 4.7944E7 | 5.4914E5 | 5.8276E6 |  | 5 | 0 | 0 | 0 | 0 | 1 | 1 | 1 | 1 | 1 | 0 | 189 | 196 | Carbamidomethylation | C3:Carbamidomethylation:1000.00 | PEAKS DB |
| K.YFC(+57.02)LNTK.F | N | 54.53 | 944.4426 | 7 | 0.6 | 473.2289 | 2 | 11.74 | 5 | F5:2586 | DaRuMP\_F6.raw |  |  |  |  | 1.7778E6 |  | 1.2376E5 |  |  |  | 2 | 0 | 0 | 0 | 0 | 1 | 0 | 1 | 0 | 0 | 0 | 98 | 104 | Carbamidomethylation | C3:Carbamidomethylation:1000.00 | PEAKS DB |
| K.FPNGLDK.D | N | 54.24 | 789.4021 | 7 | 1.3 | 395.7079 | 2 | 11.37 | 6 | F6:2200 | DaRuMP\_F7A.raw |  |  |  |  | 1.1226E6 | 6.5621E4 |  |  |  |  | 2 | 0 | 0 | 0 | 0 | 1 | 1 | 0 | 0 | 0 | 0 | 105 | 111 |  |  | PEAKS DB |
| total 11 peptides |
| --- |

P18964|VSPA\_DABSI

back to list

  

| Protein Coverage
| Supporting Peptides
|

Protein Coverage:

Supporting Peptides:

| Peptide | Uniq | -10lgP | Mass | Length | ppm | m/z | z | RT | Fraction | Scan | Source File | Area F1 | Area F10 | Area F2\_3 | Area F5 | Area F6 | Area F7A | Area F7B | Area F8A | Area F8B | Area F9 | #Feature | #Feature F1 | #Feature F10 | #Feature F2\_3 | #Feature F5 | #Feature F6 | #Feature F7A | #Feature F7B | #Feature F8A | #Feature F8B | #Feature F9 | Start | End | PTM | AScore | Found By |
| --- | --- | --- | --- | --- | --- | --- | --- | --- | --- | --- | --- | --- | --- | --- | --- | --- | --- | --- | --- | --- | --- | --- | --- | --- | --- | --- | --- | --- | --- | --- | --- | --- | --- | --- | --- | --- | --- |
| K.ISTTEDTYPDVPHC(+57.02)TNIFIVK.H | N | 82.13 | 2449.1836 | 21 | 3.7 | 817.4028 | 3 | 16.70 | 6 | F6:6525 | DaRuMP\_F7A.raw |  |  |  |  |  | 7.9072E6 | 2.9622E6 |  |  |  | 2 | 0 | 0 | 0 | 0 | 0 | 1 | 1 | 0 | 0 | 0 | 128 | 148 | Carbamidomethylation | C14:Carbamidomethylation:1000.00 | PEAKS DB |
| Y.TSTSSTIHC(+57.02)GGALINR.E | Y | 79.14 | 1673.8156 | 16 | -0.5 | 837.9146 | 2 | 11.19 | 5 | F5:2060 | DaRuMP\_F6.raw |  |  |  |  | 8.2036E5 |  |  |  |  |  | 2 | 0 | 0 | 0 | 0 | 2 | 0 | 0 | 0 | 0 | 0 | 20 | 35 | Carbamidomethylation | C9:Carbamidomethylation:1000.00 | PEAKS DB |
| R.EWVLTAAHC(+57.02)DR.R | N | 78.50 | 1356.6245 | 11 | 3.6 | 679.3203 | 2 | 11.66 | 6 | F6:2454 | DaRuMP\_F7A.raw |  |  |  |  | 7.9894E6 | 8.3434E5 | 8.335E5 |  | 1.6664E5 |  | 7 | 0 | 0 | 0 | 0 | 2 | 2 | 2 | 0 | 1 | 0 | 36 | 46 | Carbamidomethylation | C9:Carbamidomethylation:1000.00 | PEAKS DB |
| VVGGDEC(+57.02)NINEHPFLVALYTSTSSTIHC(+57.02)GGALINR.E | Y | 76.98 | 3800.8250 | 35 | 3.4 | 951.2145 | 4 | 31.91 | 6 | F6:16980 | DaRuMP\_F7A.raw |  |  |  |  |  | 6.9042E6 |  |  |  |  | 1 | 0 | 0 | 0 | 0 | 0 | 1 | 0 | 0 | 0 | 0 | 1 | 35 | Carbamidomethylation | C7:Carbamidomethylation:1000.00;C28:Carbamidomethylation:1000.00 | PEAKS DB |
| R.DTC(+57.02)HGDSGGPLIC(+57.02)NGQIQGIVAGGSEPC(+57.02)GQHLK.P | Y | 76.39 | 3418.5452 | 33 | 0.7 | 855.6442 | 4 | 12.26 | 5 | F5:3080 | DaRuMP\_F6.raw |  |  |  |  | 6.4722E6 | 5.8411E5 |  |  |  |  | 2 | 0 | 0 | 0 | 0 | 1 | 1 | 0 | 0 | 0 | 0 | 176 | 208 | Carbamidomethylation | C3:Carbamidomethylation:1000.00;C13:Carbamidomethylation:1000.00;C28:Carbamidomethylation:1000.00 | PEAKS DB |
| K.ISTTEDTYPDVPHC(+57.02)TN.I | N | 75.52 | 1848.7836 | 16 | -0.4 | 925.3987 | 2 | 11.61 | 5 | F5:2486 | DaRuMP\_F6.raw |  |  |  |  | 2.5361E6 | 1.7964E5 |  |  | 8.2217E4 |  | 3 | 0 | 0 | 0 | 0 | 1 | 1 | 0 | 0 | 1 | 0 | 128 | 143 | Carbamidomethylation | C14:Carbamidomethylation:1000.00 | PEAKS DB |
| R.RPVTYSTHIAPVSLPSR.S | N | 75.09 | 1880.0267 | 17 | 2.9 | 627.6832 | 3 | 11.52 | 6 | F6:2353 | DaRuMP\_F7A.raw |  |  |  |  | 3.1226E6 | 4.0742E5 | 2.5159E5 |  |  |  | 4 | 0 | 0 | 0 | 0 | 2 | 1 | 1 | 0 | 0 | 0 | 96 | 112 |  |  | PEAKS DB |
| F.LVALYTSTSSTIHC(+57.02)GGALINR.E | Y | 73.29 | 2233.1526 | 21 | 1.2 | 745.3923 | 3 | 12.41 | 5 | F5:3215 | DaRuMP\_F6.raw |  |  |  |  | 2.633E5 |  |  |  |  |  | 1 | 0 | 0 | 0 | 0 | 1 | 0 | 0 | 0 | 0 | 0 | 15 | 35 | Carbamidomethylation | C14:Carbamidomethylation:1000.00 | PEAKS DB |
| Y.STHIAPVSLPSR.S | N | 70.68 | 1263.6935 | 12 | 0.5 | 632.8543 | 2 | 11.47 | 5 | F5:2315 | DaRuMP\_F6.raw |  |  |  |  | 4.3808E6 | 5.7195E5 | 2.2102E5 |  |  |  | 5 | 0 | 0 | 0 | 0 | 2 | 2 | 1 | 0 | 0 | 0 | 101 | 112 |  |  | PEAKS DB |
| R.DTC(+57.02)HGDSGGPLIC(+57.02)NGQIQGIVAGGSEPC(+57.02)GQHLKPAVYTK.V | Y | 61.88 | 4077.9094 | 39 | -0.9 | 1020.4838 | 4 | 12.18 | 5 | F5:3006 | DaRuMP\_F6.raw |  |  |  |  | 3.2375E6 | 2.9852E6 |  |  |  |  | 3 | 0 | 0 | 0 | 0 | 1 | 2 | 0 | 0 | 0 | 0 | 176 | 214 | Carbamidomethylation | C3:Carbamidomethylation:1000.00;C13:Carbamidomethylation:1000.00;C28:Carbamidomethylation:1000.00 | PEAKS DB |
| K.VFDYNNWIQNIIAGNR.T | Y | 61.34 | 1935.9591 | 16 | 3.0 | 968.9874 | 2 | 49.36 | 6 | F6:24589 | DaRuMP\_F7A.raw |  |  |  |  |  | 7.2425E5 |  |  |  |  | 1 | 0 | 0 | 0 | 0 | 0 | 1 | 0 | 0 | 0 | 0 | 215 | 230 |  |  | PEAKS DB |
| K.WC(+57.02)EPLYPWVPADSR.T | N | 59.36 | 1774.8137 | 14 | 2.9 | 888.4146 | 2 | 30.30 | 6 | F6:15902 | DaRuMP\_F7A.raw |  |  |  |  | 1.9987E6 | 1.1273E6 |  |  |  |  | 2 | 0 | 0 | 0 | 0 | 1 | 1 | 0 | 0 | 0 | 0 | 151 | 164 | Carbamidomethylation | C2:Carbamidomethylation:1000.00 | PEAKS DB |
| K.HKWC(+57.02)EPLYPWVPADSR.T | N | 57.24 | 2039.9675 | 16 | 0.7 | 680.9952 | 3 | 13.13 | 6 | F6:3711 | DaRuMP\_F7A.raw |  |  |  |  |  | 3.3023E5 |  |  |  |  | 1 | 0 | 0 | 0 | 0 | 0 | 1 | 0 | 0 | 0 | 0 | 149 | 164 | Carbamidomethylation | C4:Carbamidomethylation:1000.00 | PEAKS DB |
| R.TLC(+57.02)AGILK.G | N | 56.73 | 874.4946 | 8 | 0.1 | 438.2546 | 2 | 11.83 | 9 | F9:2657 | DaRuMP\_F8B.raw |  |  |  |  | 7.6058E6 | 1.0726E6 | 4.7944E7 | 5.4914E5 | 5.8276E6 |  | 5 | 0 | 0 | 0 | 0 | 1 | 1 | 1 | 1 | 1 | 0 | 165 | 172 | Carbamidomethylation | C3:Carbamidomethylation:1000.00 | PEAKS DB |
| K.YFC(+57.02)LNTK.F | N | 54.53 | 944.4426 | 7 | 0.6 | 473.2289 | 2 | 11.74 | 5 | F5:2586 | DaRuMP\_F6.raw |  |  |  |  | 1.7778E6 |  | 1.2376E5 |  |  |  | 2 | 0 | 0 | 0 | 0 | 1 | 0 | 1 | 0 | 0 | 0 | 74 | 80 | Carbamidomethylation | C3:Carbamidomethylation:1000.00 | PEAKS DB |
| K.FPNGLDK.D | N | 54.24 | 789.4021 | 7 | 1.3 | 395.7079 | 2 | 11.37 | 6 | F6:2200 | DaRuMP\_F7A.raw |  |  |  |  | 1.1226E6 | 6.5621E4 |  |  |  |  | 2 | 0 | 0 | 0 | 0 | 1 | 1 | 0 | 0 | 0 | 0 | 81 | 87 |  |  | PEAKS DB |
| R.DTC(+57.02)HGDSGGPLIC(+57.02)NGQIQGIVAGGSEPC(+57.02)GQHLKPAVY.T | Y | 53.06 | 3848.7668 | 37 | -5.8 | 963.1934 | 4 | 16.82 | 9 | F9:5918 | DaRuMP\_F8B.raw |  |  |  |  |  |  |  |  | 0 |  | 0 | 0 | 0 | 0 | 0 | 0 | 0 | 0 | 0 | 0 | 0 | 176 | 212 | Carbamidomethylation | C3:Carbamidomethylation:1000.00;C13:Carbamidomethylation:1000.00;C28:Carbamidomethylation:1000.00 | PEAKS DB |
| total 17 peptides |
| --- |

B6EWW8|V5NTD\_GLOBR

back to list

  

| Protein Coverage
| Supporting Peptides
|

Protein Coverage:

Supporting Peptides:

| Peptide | Uniq | -10lgP | Mass | Length | ppm | m/z | z | RT | Fraction | Scan | Source File | Area F1 | Area F10 | Area F2\_3 | Area F5 | Area F6 | Area F7A | Area F7B | Area F8A | Area F8B | Area F9 | #Feature | #Feature F1 | #Feature F10 | #Feature F2\_3 | #Feature F5 | #Feature F6 | #Feature F7A | #Feature F7B | #Feature F8A | #Feature F8B | #Feature F9 | Start | End | PTM | AScore | Found By |
| --- | --- | --- | --- | --- | --- | --- | --- | --- | --- | --- | --- | --- | --- | --- | --- | --- | --- | --- | --- | --- | --- | --- | --- | --- | --- | --- | --- | --- | --- | --- | --- | --- | --- | --- | --- | --- | --- |
| K.IIALGHSGFFEDQR.I | Y | 88.72 | 1588.7997 | 14 | -0.2 | 530.6071 | 3 | 16.62 | 8 | F8:3270 | DaRuMP\_F8A.raw |  | 1.821E4 |  |  |  |  |  | 3.175E6 |  |  | 3 | 0 | 1 | 0 | 0 | 0 | 0 | 0 | 2 | 0 | 0 | 229 | 242 |  |  | PEAKS DB |
| K.YLGYLNVIFDDK.G | Y | 81.80 | 1458.7394 | 12 | 0.7 | 730.3774 | 2 | 39.15 | 8 | F8:17751 | DaRuMP\_F8A.raw |  |  |  |  |  |  |  | 6.4879E6 |  |  | 1 | 0 | 0 | 0 | 0 | 0 | 0 | 0 | 1 | 0 | 0 | 300 | 311 |  |  | PEAKS DB |
| K.ETPVLSNPGPYLEFR.D | Y | 76.39 | 1717.8674 | 15 | 0.2 | 859.9412 | 2 | 25.14 | 8 | F8:9588 | DaRuMP\_F8A.raw |  | 6.4818E4 |  |  |  |  |  | 1.1097E7 |  | 2.673E5 | 3 | 0 | 1 | 0 | 0 | 0 | 0 | 0 | 1 | 0 | 1 | 194 | 208 |  |  | PEAKS DB |
| R.QVPVVQAYAFGK.Y | Y | 75.32 | 1305.7081 | 12 | 0.7 | 653.8618 | 2 | 17.13 | 8 | F8:3707 | DaRuMP\_F8A.raw |  |  |  |  |  |  |  | 1.9684E6 |  |  | 1 | 0 | 0 | 0 | 0 | 0 | 0 | 0 | 1 | 0 | 0 | 288 | 299 |  |  | PEAKS DB |
| A.GSFELTILHTNDVHAR.V | Y | 73.34 | 1808.9169 | 16 | 0.2 | 603.9797 | 3 | 16.13 | 8 | F8:2893 | DaRuMP\_F8A.raw |  |  |  |  |  |  |  | 2.9814E5 |  |  | 1 | 0 | 0 | 0 | 0 | 0 | 0 | 0 | 1 | 0 | 0 | 40 | 55 |  |  | PEAKS DB |
| G.SFELTILHTNDVHAR.V | N | 72.96 | 1751.8955 | 15 | -0.3 | 438.9810 | 4 | 16.00 | 8 | F8:2784 | DaRuMP\_F8A.raw |  |  |  |  |  |  |  | 2.2772E5 |  |  | 1 | 0 | 0 | 0 | 0 | 0 | 0 | 0 | 1 | 0 | 0 | 41 | 55 |  |  | PEAKS DB |
| L.GYDAMALGNHEFDNGLAGLLDPLLK.H | Y | 71.07 | 2643.3003 | 25 | 0.9 | 882.1082 | 3 | 56.35 | 8 | F8:24706 | DaRuMP\_F8A.raw |  |  |  |  |  |  |  | 6.3587E5 |  |  | 1 | 0 | 0 | 0 | 0 | 0 | 0 | 0 | 1 | 0 | 0 | 123 | 147 |  |  | PEAKS DB |
| K.HANFPILSANIRPK.G | Y | 69.75 | 1576.8838 | 14 | -1.2 | 526.6346 | 3 | 15.69 | 8 | F8:2480 | DaRuMP\_F8A.raw |  |  |  |  |  |  |  | 5.3338E5 |  |  | 1 | 0 | 0 | 0 | 0 | 0 | 0 | 0 | 1 | 0 | 0 | 148 | 161 |  |  | PEAKS DB |
| S.FELTILHTNDVHAR.V | N | 68.11 | 1664.8634 | 14 | 0.7 | 555.9622 | 3 | 15.95 | 8 | F8:2724 | DaRuMP\_F8A.raw |  |  |  |  |  |  |  | 1.1005E5 |  |  | 1 | 0 | 0 | 0 | 0 | 0 | 0 | 0 | 1 | 0 | 0 | 42 | 55 |  |  | PEAKS DB |
| K.HANFPILSANIR.P | Y | 63.47 | 1351.7361 | 12 | 0.3 | 676.8755 | 2 | 16.21 | 8 | F8:2997 | DaRuMP\_F8A.raw |  |  |  |  |  |  |  | 6.6519E4 |  |  | 1 | 0 | 0 | 0 | 0 | 0 | 0 | 0 | 1 | 0 | 0 | 148 | 159 |  |  | PEAKS DB |
| A.AGSFELTILHTNDVHAR.V | Y | 60.36 | 1879.9540 | 17 | -0.1 | 627.6586 | 3 | 16.07 | 8 | F8:2809 | DaRuMP\_F8A.raw |  |  |  |  |  |  |  | 1.0255E6 |  |  | 1 | 0 | 0 | 0 | 0 | 0 | 0 | 0 | 1 | 0 | 0 | 39 | 55 |  |  | PEAKS DB |
| L.GYLNVIFDDK.G | Y | 59.33 | 1182.5920 | 10 | 0.5 | 592.3036 | 2 | 22.48 | 4 | F4:8147 | DaRuMP\_F5.raw |  |  |  | 9.019E4 |  |  |  |  |  |  | 1 | 0 | 0 | 0 | 1 | 0 | 0 | 0 | 0 | 0 | 0 | 302 | 311 |  |  | PEAKS DB |
| K.HANFPILSAN.I | Y | 58.52 | 1082.5509 | 10 | 0.3 | 542.2829 | 2 | 17.17 | 8 | F8:3727 | DaRuMP\_F8A.raw |  |  |  |  |  |  |  | 2.3552E6 |  |  | 1 | 0 | 0 | 0 | 0 | 0 | 0 | 0 | 1 | 0 | 0 | 148 | 157 |  |  | PEAKS DB |
| K.C(+57.02)TGQDC(+57.02)YGGVAR.R | Y | 58.29 | 1342.5394 | 12 | 0.2 | 672.2771 | 2 | 10.97 | 10 | F10:1757 | DaRuMP\_F9.raw |  |  |  |  |  |  |  |  |  | 1.0803E5 | 1 | 0 | 0 | 0 | 0 | 0 | 0 | 0 | 0 | 0 | 1 | 66 | 77 | Carbamidomethylation | C1:Carbamidomethylation:1000.00;C6:Carbamidomethylation:1000.00 | PEAKS DB |
| K.VGIIGYTTK.E | Y | 56.15 | 950.5436 | 9 | -2.4 | 476.2780 | 2 | 15.79 | 8 | F8:2578 | DaRuMP\_F8A.raw |  |  |  |  |  |  |  | 5.8973E5 |  |  | 1 | 0 | 0 | 0 | 0 | 0 | 0 | 0 | 1 | 0 | 0 | 185 | 193 |  |  | PEAKS DB |
| K.VFPAVEGR.V | Y | 53.40 | 873.4708 | 8 | -5.8 | 437.7401 | 2 | 15.58 | 8 | F8:2379 | DaRuMP\_F8A.raw |  |  |  |  |  |  |  | 0 |  |  | 0 | 0 | 0 | 0 | 0 | 0 | 0 | 0 | 0 | 0 | 0 | 553 | 560 |  |  | PEAKS DB |
| total 16 peptides |
| --- |

P35908|K22E\_HUMAN

back to list

  

| Protein Coverage
| Supporting Peptides
|

Protein Coverage:

Supporting Peptides:

| Peptide | Uniq | -10lgP | Mass | Length | ppm | m/z | z | RT | Fraction | Scan | Source File | Area F1 | Area F10 | Area F2\_3 | Area F5 | Area F6 | Area F7A | Area F7B | Area F8A | Area F8B | Area F9 | #Feature | #Feature F1 | #Feature F10 | #Feature F2\_3 | #Feature F5 | #Feature F6 | #Feature F7A | #Feature F7B | #Feature F8A | #Feature F8B | #Feature F9 | Start | End | PTM | AScore | Found By |
| --- | --- | --- | --- | --- | --- | --- | --- | --- | --- | --- | --- | --- | --- | --- | --- | --- | --- | --- | --- | --- | --- | --- | --- | --- | --- | --- | --- | --- | --- | --- | --- | --- | --- | --- | --- | --- | --- |
| R.FLEQQNQVLQTK.W | N | 81.01 | 1474.7780 | 12 | 0.8 | 738.3969 | 2 | 11.70 | 3 | F3:2504 | DaRuMP\_F2\_F3.raw | 2.5845E6 | 6.3219E5 | 7.403E5 | 2.7387E6 | 2.4581E6 |  | 6.9952E6 | 1.1298E6 | 1.6497E6 | 4.0389E5 | 9 | 1 | 1 | 1 | 1 | 1 | 0 | 1 | 1 | 1 | 1 | 198 | 209 |  |  | PEAKS DB |
| R.GFSSGSAVVSGGSR.R | N | 78.82 | 1253.6000 | 14 | -2.0 | 627.8060 | 2 | 11.22 | 7 | F7:2044 | DaRuMP\_F7B.raw | 5.709E4 | 1.2426E5 | 2.7983E4 | 8.7515E4 | 6.6192E4 |  | 1.7227E5 |  | 3.398E4 | 6.6249E4 | 8 | 1 | 1 | 1 | 1 | 1 | 0 | 1 | 0 | 1 | 1 | 21 | 34 |  |  | PEAKS DB |
| K.NVQDAIADAEQR.G | Y | 78.40 | 1328.6321 | 12 | 0.6 | 665.3237 | 2 | 11.69 | 2 | F2:2519 | DaRuMP\_F10.raw | 4.6536E4 | 7.8451E4 | 1.6831E4 |  | 1.3604E5 |  |  |  |  |  | 4 | 1 | 1 | 1 | 0 | 1 | 0 | 0 | 0 | 0 | 0 | 419 | 430 |  |  | PEAKS DB |
| R.HGGGGGGFGGGGFGSR.S | Y | 76.80 | 1319.5754 | 16 | 0.3 | 440.8659 | 3 | 11.04 | 7 | F7:1876 | DaRuMP\_F7B.raw | 1.6104E4 |  | 3.9539E3 | 1.1643E4 |  |  | 3.6496E4 |  |  | 2.0642E4 | 7 | 2 | 0 | 1 | 1 | 0 | 0 | 1 | 0 | 0 | 2 | 46 | 61 |  |  | PEAKS DB |
| K.LNDLEEALQQAK.E | Y | 76.10 | 1370.7041 | 12 | 0.6 | 686.3597 | 2 | 17.20 | 8 | F8:3831 | DaRuMP\_F8A.raw |  | 2.4891E5 |  |  | 1.0214E6 |  | 2.0248E6 | 5.0955E5 | 4.911E5 | 2.5031E5 | 6 | 0 | 1 | 0 | 0 | 1 | 0 | 1 | 1 | 1 | 1 | 442 | 453 |  |  | PEAKS DB |
| K.YEELQVTVGR.H | Y | 74.92 | 1192.6088 | 10 | -0.6 | 597.3113 | 2 | 11.88 | 5 | F5:2747 | DaRuMP\_F6.raw | 1.0692E5 |  | 5.3923E4 |  | 3.4414E5 |  |  | 9.012E4 | 1.0651E5 | 5.8325E4 | 6 | 1 | 0 | 1 | 0 | 1 | 0 | 0 | 1 | 1 | 1 | 375 | 384 |  |  | PEAKS DB |
| R.GGSGGGGSISGGGYGSGGGSGGR.Y | Y | 69.67 | 1740.7411 | 23 | 0.1 | 871.3779 | 2 | 10.73 | 1 | F1:1645 | DaRuMP\_F1.raw | 2.3552E4 |  |  |  |  |  |  |  | 4.9982E4 |  | 2 | 1 | 0 | 0 | 0 | 0 | 0 | 0 | 0 | 1 | 0 | 550 | 572 |  |  | PEAKS DB |
| R.TAAENDFVTLK.K | N | 69.34 | 1207.6084 | 11 | -0.5 | 604.8112 | 2 | 12.01 | 5 | F5:2848 | DaRuMP\_F6.raw | 2.6278E5 | 1.1452E5 | 8.2358E4 | 3.4179E5 | 4.1886E5 |  |  |  |  | 1.1415E5 | 6 | 1 | 1 | 1 | 1 | 1 | 0 | 0 | 0 | 0 | 1 | 276 | 286 |  |  | PEAKS DB |
| R.YLDGLTAER.T | Y | 65.58 | 1036.5189 | 9 | -0.7 | 519.2664 | 2 | 11.86 | 7 | F7:2690 | DaRuMP\_F7B.raw | 2.014E5 |  |  |  | 2.0847E5 |  | 3.0297E5 |  |  |  | 3 | 1 | 0 | 0 | 0 | 1 | 0 | 1 | 0 | 0 | 0 | 239 | 247 |  |  | PEAKS DB |
| R.NLDLDSIIAEVK.A | N | 65.38 | 1328.7188 | 12 | 3.0 | 665.3670 | 2 | 37.00 | 6 | F6:19466 | DaRuMP\_F7A.raw |  | 5.6789E5 |  |  | 1.9663E6 | 5.3878E4 | 2.6962E6 | 1.693E6 | 9.4573E5 | 7.5449E5 | 7 | 0 | 1 | 0 | 0 | 1 | 1 | 1 | 1 | 1 | 1 | 342 | 353 |  |  | PEAKS DB |
| K.AQYEEIAQR.S | N | 63.85 | 1106.5356 | 9 | -0.4 | 554.2749 | 2 | 11.15 | 7 | F7:1975 | DaRuMP\_F7B.raw | 8.2668E4 |  |  |  | 1.7455E5 |  | 1.5377E5 |  | 4.796E4 | 1.2654E5 | 5 | 1 | 0 | 0 | 0 | 1 | 0 | 1 | 0 | 1 | 1 | 354 | 362 |  |  | PEAKS DB |
| K.LALDVEIATYR.K | N | 62.47 | 1262.6870 | 11 | 0.1 | 632.3508 | 2 | 19.53 | 5 | F5:9106 | DaRuMP\_F6.raw |  |  |  | 1.0143E5 | 2.8248E5 |  | 3.5938E5 |  | 1.8812E5 | 2.4586E5 | 5 | 0 | 0 | 0 | 1 | 1 | 0 | 1 | 0 | 1 | 1 | 471 | 481 |  |  | PEAKS DB |
| K.SISISVAGGGGGFGAAGGFGGR.G | Y | 62.32 | 1837.9070 | 22 | 0.4 | 919.9611 | 2 | 21.78 | 8 | F8:7766 | DaRuMP\_F8A.raw |  |  |  |  |  |  |  | 1.6462E5 |  |  | 1 | 0 | 0 | 0 | 0 | 0 | 0 | 0 | 1 | 0 | 0 | 71 | 92 |  |  | PEAKS DB |
| R.DYQELMNVK.L | N | 59.72 | 1138.5328 | 9 | -0.6 | 570.2734 | 2 | 12.61 | 7 | F7:3342 | DaRuMP\_F7B.raw |  | 3.3693E4 |  |  |  |  | 9.8528E4 |  |  |  | 2 | 0 | 1 | 0 | 0 | 0 | 0 | 1 | 0 | 0 | 0 | 462 | 470 |  |  | PEAKS DB |
| K.VDLLNQEIEFLK.V | Y | 58.85 | 1459.7922 | 12 | 0.2 | 730.9035 | 2 | 35.58 | 8 | F8:15744 | DaRuMP\_F8A.raw |  |  |  |  |  |  | 1.6883E6 | 6.9075E5 | 8.6834E5 | 3.5058E5 | 4 | 0 | 0 | 0 | 0 | 0 | 0 | 1 | 1 | 1 | 1 | 303 | 314 |  |  | PEAKS DB |
| R.STSSFSC(+57.02)LSR.H | Y | 56.17 | 1130.5026 | 10 | 0.4 | 566.2588 | 2 | 11.45 | 5 | F5:2294 | DaRuMP\_F6.raw |  |  |  |  | 6.9109E4 |  |  |  |  | 2.8985E4 | 2 | 0 | 0 | 0 | 0 | 1 | 0 | 0 | 0 | 0 | 1 | 36 | 45 | Carbamidomethylation | C7:Carbamidomethylation:1000.00 | PEAKS DB |
| total 16 peptides |
| --- |

E5L0E3|VSPAF\_DABSI

back to list

  

| Protein Coverage
| Supporting Peptides
|

Protein Coverage:

Supporting Peptides:

| Peptide | Uniq | -10lgP | Mass | Length | ppm | m/z | z | RT | Fraction | Scan | Source File | Area F1 | Area F10 | Area F2\_3 | Area F5 | Area F6 | Area F7A | Area F7B | Area F8A | Area F8B | Area F9 | #Feature | #Feature F1 | #Feature F10 | #Feature F2\_3 | #Feature F5 | #Feature F6 | #Feature F7A | #Feature F7B | #Feature F8A | #Feature F8B | #Feature F9 | Start | End | PTM | AScore | Found By |
| --- | --- | --- | --- | --- | --- | --- | --- | --- | --- | --- | --- | --- | --- | --- | --- | --- | --- | --- | --- | --- | --- | --- | --- | --- | --- | --- | --- | --- | --- | --- | --- | --- | --- | --- | --- | --- | --- |
| K.LNKPVTYSTHIASLSLPSNPPR.V | Y | 101.34 | 2391.2910 | 22 | -1.1 | 798.1034 | 3 | 11.88 | 7 | F7:2692 | DaRuMP\_F7B.raw |  |  |  |  |  |  | 7.3383E6 |  |  |  | 2 | 0 | 0 | 0 | 0 | 0 | 0 | 2 | 0 | 0 | 0 | 116 | 137 |  |  | PEAKS DB |
| K.VFDYTDWIHSIIAGN.T | Y | 78.38 | 1749.8362 | 15 | 0.6 | 875.9259 | 2 | 60.51 | 7 | F7:28278 | DaRuMP\_F7B.raw |  |  |  |  |  |  | 5.2655E6 |  |  |  | 1 | 0 | 0 | 0 | 0 | 0 | 0 | 1 | 0 | 0 | 0 | 237 | 251 |  |  | PEAKS DB |
| K.VFDYTDWIHSIIAGNTAATC(+57.02)PS | Y | 70.35 | 2438.1213 | 22 | 0.3 | 1220.0684 | 2 | 59.33 | 7 | F7:27980 | DaRuMP\_F7B.raw |  |  |  |  |  |  | 3.7928E7 |  |  |  | 1 | 0 | 0 | 0 | 0 | 0 | 0 | 1 | 0 | 0 | 0 | 237 | 258 | Carbamidomethylation | C20:Carbamidomethylation:1000.00 | PEAKS DB |
| R.IGSC(+57.02)LGDSGGPLIC(+57.02)NGQIQGIVSWGSDPC(+57.02)VNR.G | Y | 68.40 | 3372.5649 | 32 | 0.3 | 1125.1959 | 3 | 36.81 | 7 | F7:18945 | DaRuMP\_F7B.raw |  |  |  |  |  |  | 1.067E7 |  |  |  | 1 | 0 | 0 | 0 | 0 | 0 | 0 | 1 | 0 | 0 | 0 | 197 | 228 | Carbamidomethylation | C4:Carbamidomethylation:1000.00;C14:Carbamidomethylation:1000.00;C29:Carbamidomethylation:1000.00 | PEAKS DB |
| K.FFC(+57.02)LSSK.S | N | 65.32 | 887.4211 | 7 | 0.1 | 444.7168 | 2 | 12.11 | 6 | F6:2844 | DaRuMP\_F7A.raw |  |  |  |  | 2.8418E6 | 8.5101E5 | 1.0094E7 |  | 1.4913E5 |  | 4 | 0 | 0 | 0 | 0 | 1 | 1 | 1 | 0 | 1 | 0 | 96 | 102 | Carbamidomethylation | C3:Carbamidomethylation:1000.00 | PEAKS DB |
| R.IM(+15.99)GWGSITSPK.K | Y | 64.29 | 1191.5958 | 11 | 1.6 | 596.8062 | 2 | 12.15 | 7 | F7:2935 | DaRuMP\_F7B.raw |  |  |  |  |  |  | 1.1614E6 |  |  |  | 1 | 0 | 0 | 0 | 0 | 0 | 0 | 1 | 0 | 0 | 0 | 144 | 154 | Oxidation (M) | M2:Oxidation (M):1000.00 | PEAKS DB |
| Y.STHIASLSLPSNPPR.V | Y | 63.04 | 1575.8369 | 15 | -0.4 | 526.2860 | 3 | 11.76 | 7 | F7:2535 | DaRuMP\_F7B.raw |  |  |  |  |  |  | 3.0293E7 |  |  |  | 2 | 0 | 0 | 0 | 0 | 0 | 0 | 2 | 0 | 0 | 0 | 123 | 137 |  |  | PEAKS DB |
| R.IMGWGSITSPK.K | Y | 60.52 | 1175.6008 | 11 | -0.5 | 588.8074 | 2 | 14.88 | 7 | F7:5220 | DaRuMP\_F7B.raw |  |  |  |  |  |  | 7.5759E6 |  |  |  | 1 | 0 | 0 | 0 | 0 | 0 | 0 | 1 | 0 | 0 | 0 | 144 | 154 |  |  | PEAKS DB |
| H.IASLSLPSNPPR.V | Y | 59.69 | 1250.6982 | 12 | 2.0 | 626.3577 | 2 | 12.22 | 7 | F7:2974 | DaRuMP\_F7B.raw |  |  |  |  |  |  | 1.3283E7 |  |  |  | 1 | 0 | 0 | 0 | 0 | 0 | 0 | 1 | 0 | 0 | 0 | 126 | 137 |  |  | PEAKS DB |
| Q.GIVSWGSDPC(+57.02)VNR.G | Y | 54.75 | 1445.6721 | 13 | 0.7 | 723.8439 | 2 | 12.38 | 7 | F7:3145 | DaRuMP\_F7B.raw |  |  |  |  |  |  | 3.2795E5 |  |  |  | 1 | 0 | 0 | 0 | 0 | 0 | 0 | 1 | 0 | 0 | 0 | 216 | 228 | Carbamidomethylation | C10:Carbamidomethylation:1000.00 | PEAKS DB |
| K.ILPFVPHC(+57.02)AN.I | Y | 52.04 | 1166.5906 | 10 | 0.7 | 584.3030 | 2 | 14.55 | 7 | F7:4956 | DaRuMP\_F7B.raw |  |  |  |  |  |  | 7.104E6 |  |  |  | 1 | 0 | 0 | 0 | 0 | 0 | 0 | 1 | 0 | 0 | 0 | 156 | 165 | Carbamidomethylation | C8:Carbamidomethylation:1000.00 | PEAKS DB |
| total 11 peptides |
| --- |

P0DL42|TXVE\_DABSI

back to list

  

| Protein Coverage
| Supporting Peptides
|

Protein Coverage:

Supporting Peptides:

| Peptide | Uniq | -10lgP | Mass | Length | ppm | m/z | z | RT | Fraction | Scan | Source File | Area F1 | Area F10 | Area F2\_3 | Area F5 | Area F6 | Area F7A | Area F7B | Area F8A | Area F8B | Area F9 | #Feature | #Feature F1 | #Feature F10 | #Feature F2\_3 | #Feature F5 | #Feature F6 | #Feature F7A | #Feature F7B | #Feature F8A | #Feature F8B | #Feature F9 | Start | End | PTM | AScore | Found By |
| --- | --- | --- | --- | --- | --- | --- | --- | --- | --- | --- | --- | --- | --- | --- | --- | --- | --- | --- | --- | --- | --- | --- | --- | --- | --- | --- | --- | --- | --- | --- | --- | --- | --- | --- | --- | --- | --- |
| R.ETLVSILQEHPDEISDIFR.P | Y | 93.37 | 2240.1323 | 19 | -0.2 | 1121.0732 | 2 | 41.06 | 4 | F4:13968 | DaRuMP\_F5.raw |  |  |  | 6.8593E8 | 5.5618E7 | 1.0005E5 |  |  |  |  | 9 | 0 | 0 | 0 | 6 | 2 | 1 | 0 | 0 | 0 | 0 | 18 | 36 |  |  | PEAKS DB |
| R.ETLVSILQEHPDEISDIFRPSC(+57.02)VAVLR.C | Y | 76.51 | 3122.6069 | 27 | -0.7 | 1041.8755 | 3 | 41.60 | 4 | F4:14403 | DaRuMP\_F5.raw |  |  |  | 2.2769E8 |  |  |  |  |  |  | 2 | 0 | 0 | 0 | 2 | 0 | 0 | 0 | 0 | 0 | 0 | 18 | 44 | Carbamidomethylation | C22:Carbamidomethylation:1000.00 | PEAKS DB |
| R.ETLVSILQEHPDEISDIFRPS.C | Y | 71.18 | 2424.2173 | 21 | 0.5 | 809.0801 | 3 | 42.10 | 4 | F4:14537 | DaRuMP\_F5.raw |  |  |  | 1.9182E8 | 1.8067E7 |  |  |  |  |  | 4 | 0 | 0 | 0 | 3 | 1 | 0 | 0 | 0 | 0 | 0 | 18 | 38 |  |  | PEAKS DB |
| K.HTADIQIMR.M | Y | 67.46 | 1083.5496 | 9 | 0.7 | 542.7824 | 2 | 11.28 | 5 | F5:2136 | DaRuMP\_F6.raw |  |  |  | 5.9711E7 | 3.7607E6 |  |  |  |  |  | 2 | 0 | 0 | 0 | 1 | 1 | 0 | 0 | 0 | 0 | 0 | 62 | 70 |  |  | PEAKS DB |
| D.IFRPSC(+57.02)VAVLR.C | N | 66.00 | 1316.7386 | 11 | 0.0 | 439.9202 | 3 | 11.78 | 4 | F4:2647 | DaRuMP\_F5.raw |  |  |  | 9.4465E5 |  |  |  |  |  |  | 1 | 0 | 0 | 0 | 1 | 0 | 0 | 0 | 0 | 0 | 0 | 34 | 44 | Carbamidomethylation | C6:Carbamidomethylation:1000.00 | PEAKS DB |
| R.ETLVSILQEHPDEISDIFRPSC(+57.02).V | Y | 65.86 | 2584.2478 | 22 | 0.2 | 862.4233 | 3 | 42.53 | 4 | F4:14803 | DaRuMP\_F5.raw |  |  |  | 2.9031E7 |  |  |  |  |  |  | 1 | 0 | 0 | 0 | 1 | 0 | 0 | 0 | 0 | 0 | 0 | 18 | 39 | Carbamidomethylation | C22:Carbamidomethylation:1000.00 | PEAKS DB |
| S.ILQEHPDEISDIFRPSC(+57.02)VAVLR.C | Y | 64.13 | 2593.3323 | 22 | 0.5 | 649.3406 | 4 | 20.53 | 4 | F4:7492 | DaRuMP\_F5.raw |  |  |  | 1.739E6 |  |  |  |  |  |  | 1 | 0 | 0 | 0 | 1 | 0 | 0 | 0 | 0 | 0 | 0 | 23 | 44 | Carbamidomethylation | C17:Carbamidomethylation:1000.00 | PEAKS DB |
| R.ETLVSILQEHPDEISD.I | Y | 63.64 | 1823.8789 | 16 | 0.2 | 912.9469 | 2 | 26.65 | 4 | F4:9509 | DaRuMP\_F5.raw |  |  |  | 7.4938E6 |  |  |  |  |  |  | 1 | 0 | 0 | 0 | 1 | 0 | 0 | 0 | 0 | 0 | 0 | 18 | 33 |  |  | PEAKS DB |
| K.HTADIQIM(+15.99)R.M | Y | 63.64 | 1099.5444 | 9 | -0.6 | 550.7792 | 2 | 10.84 | 4 | F4:1716 | DaRuMP\_F5.raw |  |  |  | 6.5574E6 | 8.7927E5 |  |  |  |  |  | 2 | 0 | 0 | 0 | 1 | 1 | 0 | 0 | 0 | 0 | 0 | 62 | 70 | Oxidation (M) | M8:Oxidation (M):1000.00 | PEAKS DB |
| R.ETLVSILQEHPDEISDIF.R | Y | 63.25 | 2084.0312 | 18 | 1.7 | 1043.0247 | 2 | 52.48 | 4 | F4:17753 | DaRuMP\_F5.raw |  |  |  | 1.136E6 |  |  |  |  |  |  | 1 | 0 | 0 | 0 | 1 | 0 | 0 | 0 | 0 | 0 | 0 | 18 | 35 |  |  | PEAKS DB |
| R.SAC(+57.02)QTRETLVSILQEHPDEISDIFRPSC(+57.02)VAVLR.C | Y | 61.54 | 3825.9141 | 33 | 1.9 | 766.1915 | 5 | 34.51 | 4 | F4:11631 | DaRuMP\_F5.raw |  |  |  | 7.5094E6 |  |  |  |  |  |  | 1 | 0 | 0 | 0 | 1 | 0 | 0 | 0 | 0 | 0 | 0 | 12 | 44 | Carbamidomethylation | C3:Carbamidomethylation:1000.00;C28:Carbamidomethylation:1000.00 | PEAKS DB |
| K.QGEPEGPKEPR | Y | 61.15 | 1222.5941 | 11 | -2.2 | 408.5378 | 3 | 10.69 | 5 | F5:1607 | DaRuMP\_F6.raw |  |  |  |  | 2.2643E6 |  |  |  |  |  | 1 | 0 | 0 | 0 | 0 | 1 | 0 | 0 | 0 | 0 | 0 | 99 | 109 |  |  | PEAKS DB |
| K.FMEHTAC(+57.02)EC(+57.02)RPR.W | Y | 59.32 | 1592.6647 | 12 | -1.7 | 531.8946 | 3 | 10.69 | 5 | F5:1598 | DaRuMP\_F6.raw |  |  |  |  | 6.8832E6 |  |  |  |  |  | 1 | 0 | 0 | 0 | 0 | 1 | 0 | 0 | 0 | 0 | 0 | 85 | 96 | Carbamidomethylation | C7:Carbamidomethylation:1000.00;C9:Carbamidomethylation:1000.00 | PEAKS DB |
| R.ETLVSILQEHPD.E | Y | 56.77 | 1379.6932 | 12 | 0.4 | 690.8542 | 2 | 21.36 | 4 | F4:7873 | DaRuMP\_F5.raw |  |  |  | 5.1704E6 |  |  |  |  |  |  | 1 | 0 | 0 | 0 | 1 | 0 | 0 | 0 | 0 | 0 | 0 | 18 | 29 |  |  | PEAKS DB |
| K.FMEHTAC(+57.02)EC(+57.02)R.P | Y | 53.20 | 1339.5107 | 10 | -0.8 | 670.7621 | 2 | 10.67 | 4 | F4:1603 | DaRuMP\_F5.raw |  |  |  | 7.6512E6 |  |  |  |  |  |  | 1 | 0 | 0 | 0 | 1 | 0 | 0 | 0 | 0 | 0 | 0 | 85 | 94 | Carbamidomethylation | C7:Carbamidomethylation:1000.00;C9:Carbamidomethylation:1000.00 | PEAKS DB |
| total 15 peptides |
| --- |

P67861|TXVE\_DABRR

back to list

  

| Protein Coverage
| Supporting Peptides
|

Protein Coverage:

Supporting Peptides:

| Peptide | Uniq | -10lgP | Mass | Length | ppm | m/z | z | RT | Fraction | Scan | Source File | Area F1 | Area F10 | Area F2\_3 | Area F5 | Area F6 | Area F7A | Area F7B | Area F8A | Area F8B | Area F9 | #Feature | #Feature F1 | #Feature F10 | #Feature F2\_3 | #Feature F5 | #Feature F6 | #Feature F7A | #Feature F7B | #Feature F8A | #Feature F8B | #Feature F9 | Start | End | PTM | AScore | Found By |
| --- | --- | --- | --- | --- | --- | --- | --- | --- | --- | --- | --- | --- | --- | --- | --- | --- | --- | --- | --- | --- | --- | --- | --- | --- | --- | --- | --- | --- | --- | --- | --- | --- | --- | --- | --- | --- | --- |
| R.ETLVSILQEHPDEISDIFR.P | Y | 93.37 | 2240.1323 | 19 | -0.2 | 1121.0732 | 2 | 41.06 | 4 | F4:13968 | DaRuMP\_F5.raw |  |  |  | 6.8593E8 | 5.5618E7 | 1.0005E5 |  |  |  |  | 9 | 0 | 0 | 0 | 6 | 2 | 1 | 0 | 0 | 0 | 0 | 42 | 60 |  |  | PEAKS DB |
| R.ETLVSILQEHPDEISDIFRPSC(+57.02)VAVLR.C | Y | 76.51 | 3122.6069 | 27 | -0.7 | 1041.8755 | 3 | 41.60 | 4 | F4:14403 | DaRuMP\_F5.raw |  |  |  | 2.2769E8 |  |  |  |  |  |  | 2 | 0 | 0 | 0 | 2 | 0 | 0 | 0 | 0 | 0 | 0 | 42 | 68 | Carbamidomethylation | C22:Carbamidomethylation:1000.00 | PEAKS DB |
| R.ETLVSILQEHPDEISDIFRPS.C | Y | 71.18 | 2424.2173 | 21 | 0.5 | 809.0801 | 3 | 42.10 | 4 | F4:14537 | DaRuMP\_F5.raw |  |  |  | 1.9182E8 | 1.8067E7 |  |  |  |  |  | 4 | 0 | 0 | 0 | 3 | 1 | 0 | 0 | 0 | 0 | 0 | 42 | 62 |  |  | PEAKS DB |
| K.HTADIQIMR.M | Y | 67.46 | 1083.5496 | 9 | 0.7 | 542.7824 | 2 | 11.28 | 5 | F5:2136 | DaRuMP\_F6.raw |  |  |  | 5.9711E7 | 3.7607E6 |  |  |  |  |  | 2 | 0 | 0 | 0 | 1 | 1 | 0 | 0 | 0 | 0 | 0 | 86 | 94 |  |  | PEAKS DB |
| D.IFRPSC(+57.02)VAVLR.C | N | 66.00 | 1316.7386 | 11 | 0.0 | 439.9202 | 3 | 11.78 | 4 | F4:2647 | DaRuMP\_F5.raw |  |  |  | 9.4465E5 |  |  |  |  |  |  | 1 | 0 | 0 | 0 | 1 | 0 | 0 | 0 | 0 | 0 | 0 | 58 | 68 | Carbamidomethylation | C6:Carbamidomethylation:1000.00 | PEAKS DB |
| R.ETLVSILQEHPDEISDIFRPSC(+57.02).V | Y | 65.86 | 2584.2478 | 22 | 0.2 | 862.4233 | 3 | 42.53 | 4 | F4:14803 | DaRuMP\_F5.raw |  |  |  | 2.9031E7 |  |  |  |  |  |  | 1 | 0 | 0 | 0 | 1 | 0 | 0 | 0 | 0 | 0 | 0 | 42 | 63 | Carbamidomethylation | C22:Carbamidomethylation:1000.00 | PEAKS DB |
| S.ILQEHPDEISDIFRPSC(+57.02)VAVLR.C | Y | 64.13 | 2593.3323 | 22 | 0.5 | 649.3406 | 4 | 20.53 | 4 | F4:7492 | DaRuMP\_F5.raw |  |  |  | 1.739E6 |  |  |  |  |  |  | 1 | 0 | 0 | 0 | 1 | 0 | 0 | 0 | 0 | 0 | 0 | 47 | 68 | Carbamidomethylation | C17:Carbamidomethylation:1000.00 | PEAKS DB |
| R.ETLVSILQEHPDEISD.I | Y | 63.64 | 1823.8789 | 16 | 0.2 | 912.9469 | 2 | 26.65 | 4 | F4:9509 | DaRuMP\_F5.raw |  |  |  | 7.4938E6 |  |  |  |  |  |  | 1 | 0 | 0 | 0 | 1 | 0 | 0 | 0 | 0 | 0 | 0 | 42 | 57 |  |  | PEAKS DB |
| K.HTADIQIM(+15.99)R.M | Y | 63.64 | 1099.5444 | 9 | -0.6 | 550.7792 | 2 | 10.84 | 4 | F4:1716 | DaRuMP\_F5.raw |  |  |  | 6.5574E6 | 8.7927E5 |  |  |  |  |  | 2 | 0 | 0 | 0 | 1 | 1 | 0 | 0 | 0 | 0 | 0 | 86 | 94 | Oxidation (M) | M8:Oxidation (M):1000.00 | PEAKS DB |
| R.ETLVSILQEHPDEISDIF.R | Y | 63.25 | 2084.0312 | 18 | 1.7 | 1043.0247 | 2 | 52.48 | 4 | F4:17753 | DaRuMP\_F5.raw |  |  |  | 1.136E6 |  |  |  |  |  |  | 1 | 0 | 0 | 0 | 1 | 0 | 0 | 0 | 0 | 0 | 0 | 42 | 59 |  |  | PEAKS DB |
| R.SAC(+57.02)QTRETLVSILQEHPDEISDIFRPSC(+57.02)VAVLR.C | Y | 61.54 | 3825.9141 | 33 | 1.9 | 766.1915 | 5 | 34.51 | 4 | F4:11631 | DaRuMP\_F5.raw |  |  |  | 7.5094E6 |  |  |  |  |  |  | 1 | 0 | 0 | 0 | 1 | 0 | 0 | 0 | 0 | 0 | 0 | 36 | 68 | Carbamidomethylation | C3:Carbamidomethylation:1000.00;C28:Carbamidomethylation:1000.00 | PEAKS DB |
| K.QGEPEGPKEPR.R | Y | 61.15 | 1222.5941 | 11 | -2.2 | 408.5378 | 3 | 10.69 | 5 | F5:1607 | DaRuMP\_F6.raw |  |  |  |  | 2.2643E6 |  |  |  |  |  | 1 | 0 | 0 | 0 | 0 | 1 | 0 | 0 | 0 | 0 | 0 | 123 | 133 |  |  | PEAKS DB |
| K.FMEHTAC(+57.02)EC(+57.02)RPR.W | Y | 59.32 | 1592.6647 | 12 | -1.7 | 531.8946 | 3 | 10.69 | 5 | F5:1598 | DaRuMP\_F6.raw |  |  |  |  | 6.8832E6 |  |  |  |  |  | 1 | 0 | 0 | 0 | 0 | 1 | 0 | 0 | 0 | 0 | 0 | 109 | 120 | Carbamidomethylation | C7:Carbamidomethylation:1000.00;C9:Carbamidomethylation:1000.00 | PEAKS DB |
| R.ETLVSILQEHPD.E | Y | 56.77 | 1379.6932 | 12 | 0.4 | 690.8542 | 2 | 21.36 | 4 | F4:7873 | DaRuMP\_F5.raw |  |  |  | 5.1704E6 |  |  |  |  |  |  | 1 | 0 | 0 | 0 | 1 | 0 | 0 | 0 | 0 | 0 | 0 | 42 | 53 |  |  | PEAKS DB |
| K.FMEHTAC(+57.02)EC(+57.02)R.P | Y | 53.20 | 1339.5107 | 10 | -0.8 | 670.7621 | 2 | 10.67 | 4 | F4:1603 | DaRuMP\_F5.raw |  |  |  | 7.6512E6 |  |  |  |  |  |  | 1 | 0 | 0 | 0 | 1 | 0 | 0 | 0 | 0 | 0 | 0 | 109 | 118 | Carbamidomethylation | C7:Carbamidomethylation:1000.00;C9:Carbamidomethylation:1000.00 | PEAKS DB |
| total 15 peptides |
| --- |

A8CG90|PA2B2\_DABRR

back to list

  

| Protein Coverage
| Supporting Peptides
|

Protein Coverage:

Supporting Peptides:

| Peptide | Uniq | -10lgP | Mass | Length | ppm | m/z | z | RT | Fraction | Scan | Source File | Area F1 | Area F10 | Area F2\_3 | Area F5 | Area F6 | Area F7A | Area F7B | Area F8A | Area F8B | Area F9 | #Feature | #Feature F1 | #Feature F10 | #Feature F2\_3 | #Feature F5 | #Feature F6 | #Feature F7A | #Feature F7B | #Feature F8A | #Feature F8B | #Feature F9 | Start | End | PTM | AScore | Found By |
| --- | --- | --- | --- | --- | --- | --- | --- | --- | --- | --- | --- | --- | --- | --- | --- | --- | --- | --- | --- | --- | --- | --- | --- | --- | --- | --- | --- | --- | --- | --- | --- | --- | --- | --- | --- | --- | --- |
| K.NPLSSYSNYGC(+57.02)YC(+57.02)GWGGK.G | Y | 97.40 | 2068.8408 | 18 | 3.0 | 1035.4282 | 2 | 14.84 | 6 | F6:5012 | DaRuMP\_F7A.raw |  |  |  | 9.4622E5 | 8.1997E7 | 4.6393E7 | 7.1449E6 | 1.8353E5 |  |  | 5 | 0 | 0 | 0 | 1 | 1 | 1 | 1 | 1 | 0 | 0 | 32 | 49 | Carbamidomethylation | C11:Carbamidomethylation:1000.00;C13:Carbamidomethylation:1000.00 | PEAKS DB |
| K.NPLSSYSNYGC(+57.02)YC(+57.02)GWGGKG.K | Y | 76.37 | 2125.8623 | 19 | -0.2 | 1063.9382 | 2 | 14.65 | 5 | F5:5114 | DaRuMP\_F6.raw |  |  |  |  | 7.6372E6 |  |  |  |  |  | 1 | 0 | 0 | 0 | 0 | 1 | 0 | 0 | 0 | 0 | 0 | 32 | 50 | Carbamidomethylation | C11:Carbamidomethylation:1000.00;C13:Carbamidomethylation:1000.00 | PEAKS DB |
| Y.GC(+57.02)YC(+57.02)GWGGK.G | N | 64.20 | 1043.3953 | 9 | 3.2 | 522.7053 | 2 | 11.29 | 6 | F6:2116 | DaRuMP\_F7A.raw |  |  | 6.3696E4 |  | 1.7244E6 | 2.9315E6 | 3.3122E5 |  |  | 4.6531E4 | 5 | 0 | 0 | 1 | 0 | 1 | 1 | 1 | 0 | 0 | 1 | 41 | 49 | Carbamidomethylation | C2:Carbamidomethylation:1000.00;C4:Carbamidomethylation:1000.00 | PEAKS DB |
| R.KYPPSQC(+57.02)TGTEQC(+57.02) | Y | 62.54 | 1554.6443 | 13 | -1.3 | 778.3284 | 2 | 10.69 | 5 | F5:1600 | DaRuMP\_F6.raw |  |  |  |  | 5.0931E6 |  |  |  |  |  | 1 | 0 | 0 | 0 | 0 | 1 | 0 | 0 | 0 | 0 | 0 | 125 | 137 | Carbamidomethylation | C7:Carbamidomethylation:1000.00;C13:Carbamidomethylation:1000.00 | PEAKS DB |
| L.SSYSNYGC(+57.02)YC(+57.02)GWGGK.G | Y | 60.83 | 1744.6610 | 15 | -1.0 | 873.3369 | 2 | 11.88 | 5 | F5:2743 | DaRuMP\_F6.raw |  |  |  |  | 2.1105E6 |  |  |  |  |  | 1 | 0 | 0 | 0 | 0 | 1 | 0 | 0 | 0 | 0 | 0 | 35 | 49 | Carbamidomethylation | C8:Carbamidomethylation:1000.00;C10:Carbamidomethylation:1000.00 | PEAKS DB |
| K.YPPSQC(+57.02)TGTEQC(+57.02) | N | 60.57 | 1426.5493 | 12 | 0.7 | 714.2824 | 2 | 11.09 | 7 | F7:1878 | DaRuMP\_F7B.raw |  |  |  |  |  |  | 2.5518E5 |  |  |  | 1 | 0 | 0 | 0 | 0 | 0 | 0 | 1 | 0 | 0 | 0 | 126 | 137 | Carbamidomethylation | C6:Carbamidomethylation:1000.00;C12:Carbamidomethylation:1000.00 | PEAKS DB |
| Y.SNYGC(+57.02)YC(+57.02)GWGGK.G | N | 59.54 | 1407.5336 | 12 | -0.4 | 704.7738 | 2 | 11.63 | 5 | F5:2501 | DaRuMP\_F6.raw |  |  |  |  | 2.6878E6 |  | 2.2947E5 |  |  |  | 2 | 0 | 0 | 0 | 0 | 1 | 0 | 1 | 0 | 0 | 0 | 38 | 49 | Carbamidomethylation | C5:Carbamidomethylation:1000.00;C7:Carbamidomethylation:1000.00 | PEAKS DB |
| R.C(+57.02)C(+57.02)FVHDC(+57.02)C(+57.02)YEK.V | N | 58.26 | 1576.5568 | 11 | 2.8 | 789.2859 | 2 | 11.03 | 6 | F6:1881 | DaRuMP\_F7A.raw |  |  |  |  | 1.9651E7 | 6.2122E5 | 9.491E5 |  |  |  | 6 | 0 | 0 | 0 | 0 | 2 | 2 | 2 | 0 | 0 | 0 | 59 | 69 | Carbamidomethylation | C1:Carbamidomethylation:1000.00;C2:Carbamidomethylation:1000.00;C7:Carbamidomethylation:1000.00;C8:Carbamidomethylation:1000.00 | PEAKS DB |
| N.YGC(+57.02)YC(+57.02)GWGGK.G | N | 58.24 | 1206.4586 | 10 | 0.0 | 604.2366 | 2 | 11.59 | 5 | F5:2425 | DaRuMP\_F6.raw |  |  |  |  | 1.3442E7 |  |  |  |  |  | 1 | 0 | 0 | 0 | 0 | 1 | 0 | 0 | 0 | 0 | 0 | 40 | 49 | Carbamidomethylation | C3:Carbamidomethylation:1000.00;C5:Carbamidomethylation:1000.00 | PEAKS DB |
| Y.SYSFENGGIVC(+57.02)GDR.D | Y | 55.10 | 1559.6675 | 14 | 1.0 | 780.8418 | 2 | 11.99 | 4 | F4:2851 | DaRuMP\_F5.raw |  |  |  | 4.3969E4 |  |  |  |  |  |  | 1 | 0 | 0 | 0 | 1 | 0 | 0 | 0 | 0 | 0 | 0 | 81 | 94 | Carbamidomethylation | C11:Carbamidomethylation:1000.00 | PEAKS DB |
| total 10 peptides |
| --- |

A0A1I9KNP0|VSPH1\_VIPAA

back to list

  

| Protein Coverage
| Supporting Peptides
|

Protein Coverage:

Supporting Peptides:

| Peptide | Uniq | -10lgP | Mass | Length | ppm | m/z | z | RT | Fraction | Scan | Source File | Area F1 | Area F10 | Area F2\_3 | Area F5 | Area F6 | Area F7A | Area F7B | Area F8A | Area F8B | Area F9 | #Feature | #Feature F1 | #Feature F10 | #Feature F2\_3 | #Feature F5 | #Feature F6 | #Feature F7A | #Feature F7B | #Feature F8A | #Feature F8B | #Feature F9 | Start | End | PTM | AScore | Found By |
| --- | --- | --- | --- | --- | --- | --- | --- | --- | --- | --- | --- | --- | --- | --- | --- | --- | --- | --- | --- | --- | --- | --- | --- | --- | --- | --- | --- | --- | --- | --- | --- | --- | --- | --- | --- | --- | --- |
| L.VIGGDEC(+57.02)NINEHPFLVALHTAR.S | N | 113.76 | 2461.2173 | 22 | 3.4 | 821.4138 | 3 | 12.61 | 6 | F6:3275 | DaRuMP\_F7A.raw |  |  |  |  | 1.6884E7 | 1.4716E7 | 3.9734E6 |  |  |  | 5 | 0 | 0 | 0 | 0 | 2 | 2 | 1 | 0 | 0 | 0 | 25 | 46 | Carbamidomethylation | C7:Carbamidomethylation:1000.00 | PEAKS DB |
| R.TLC(+57.02)AGILQGGIDSC(+57.02)K.V | N | 92.53 | 1591.7698 | 15 | 3.3 | 796.8928 | 2 | 13.45 | 6 | F6:3934 | DaRuMP\_F7A.raw |  |  |  |  | 1.8743E7 | 1.4122E7 | 7.3243E6 |  | 5.5508E5 |  | 7 | 0 | 0 | 0 | 0 | 2 | 2 | 2 | 0 | 1 | 0 | 189 | 203 | Carbamidomethylation | C3:Carbamidomethylation:1000.00;C14:Carbamidomethylation:1000.00 | PEAKS DB |
| K.RPVNDSTHIAPLSLPSSPPSVGSVC(+57.02)R.I | Y | 80.81 | 2729.3918 | 26 | 3.1 | 683.3557 | 4 | 12.14 | 6 | F6:2880 | DaRuMP\_F7A.raw |  |  |  |  |  | 4.3597E5 |  |  |  |  | 1 | 0 | 0 | 0 | 0 | 0 | 1 | 0 | 0 | 0 | 0 | 120 | 145 | Carbamidomethylation | C25:Carbamidomethylation:1000.00 | PEAKS DB |
| K.FFC(+57.02)LSSK.T | N | 65.32 | 887.4211 | 7 | 0.1 | 444.7168 | 2 | 12.11 | 6 | F6:2844 | DaRuMP\_F7A.raw |  |  |  |  | 2.8418E6 | 8.5101E5 | 1.0094E7 |  | 1.4913E5 |  | 4 | 0 | 0 | 0 | 0 | 1 | 1 | 1 | 0 | 1 | 0 | 98 | 104 | Carbamidomethylation | C3:Carbamidomethylation:1000.00 | PEAKS DB |
| L.SLPSSPPSVGSVC(+57.02)R.I | N | 62.46 | 1428.7031 | 14 | -0.8 | 715.3583 | 2 | 11.69 | 7 | F7:2521 | DaRuMP\_F7B.raw |  |  |  |  | 1.8242E6 |  | 1.0733E6 |  | 1.7309E5 |  | 3 | 0 | 0 | 0 | 0 | 1 | 0 | 1 | 0 | 1 | 0 | 132 | 145 | Carbamidomethylation | C13:Carbamidomethylation:1000.00 | PEAKS DB |
| L.INQEWVLTAAR.C | N | 52.15 | 1299.6935 | 11 | 0.2 | 650.8541 | 2 | 14.19 | 5 | F5:4651 | DaRuMP\_F6.raw |  |  |  |  | 2.7645E5 |  |  |  |  |  | 1 | 0 | 0 | 0 | 0 | 1 | 0 | 0 | 0 | 0 | 0 | 57 | 67 |  |  | PEAKS DB |
| total 6 peptides |
| --- |

P0DPS3|VASP1\_VIPAA

back to list

  

| Protein Coverage
| Supporting Peptides
|

Protein Coverage:

Supporting Peptides:

| Peptide | Uniq | -10lgP | Mass | Length | ppm | m/z | z | RT | Fraction | Scan | Source File | Area F1 | Area F10 | Area F2\_3 | Area F5 | Area F6 | Area F7A | Area F7B | Area F8A | Area F8B | Area F9 | #Feature | #Feature F1 | #Feature F10 | #Feature F2\_3 | #Feature F5 | #Feature F6 | #Feature F7A | #Feature F7B | #Feature F8A | #Feature F8B | #Feature F9 | Start | End | PTM | AScore | Found By |
| --- | --- | --- | --- | --- | --- | --- | --- | --- | --- | --- | --- | --- | --- | --- | --- | --- | --- | --- | --- | --- | --- | --- | --- | --- | --- | --- | --- | --- | --- | --- | --- | --- | --- | --- | --- | --- | --- |
| VIGGDEC(+57.02)NINEHPFLVALHTAR.X | N | 113.76 | 2461.2173 | 22 | 3.4 | 821.4138 | 3 | 12.61 | 6 | F6:3275 | DaRuMP\_F7A.raw |  |  |  |  | 1.6884E7 | 1.4716E7 | 3.9734E6 |  |  |  | 5 | 0 | 0 | 0 | 0 | 2 | 2 | 1 | 0 | 0 | 0 | 1 | 22 | Carbamidomethylation | C7:Carbamidomethylation:1000.00 | PEAKS DB |
| R.TLC(+57.02)AGILQGGIDSC(+57.02)K.G | N | 92.53 | 1591.7698 | 15 | 3.3 | 796.8928 | 2 | 13.45 | 6 | F6:3934 | DaRuMP\_F7A.raw |  |  |  |  | 1.8743E7 | 1.4122E7 | 7.3243E6 |  | 5.5508E5 |  | 7 | 0 | 0 | 0 | 0 | 2 | 2 | 2 | 0 | 1 | 0 | 165 | 179 | Carbamidomethylation | C3:Carbamidomethylation:1000.00;C14:Carbamidomethylation:1000.00 | PEAKS DB |
| VIGGDEC(+57.02)NINEHPF.L | N | 78.07 | 1599.6987 | 14 | -1.0 | 800.8558 | 2 | 12.19 | 7 | F7:2994 | DaRuMP\_F7B.raw |  |  |  |  |  |  | 1.6317E6 |  |  |  | 1 | 0 | 0 | 0 | 0 | 0 | 0 | 1 | 0 | 0 | 0 | 1 | 14 | Carbamidomethylation | C7:Carbamidomethylation:1000.00 | PEAKS DB |
| R.TLC(+57.02)AGILQGGIDSC(+57.02)KG.I | Y | 68.64 | 1648.7913 | 16 | 3.3 | 825.4036 | 2 | 13.32 | 6 | F6:3870 | DaRuMP\_F7A.raw |  |  |  |  |  | 5.823E5 |  |  |  |  | 1 | 0 | 0 | 0 | 0 | 0 | 1 | 0 | 0 | 0 | 0 | 165 | 180 | Carbamidomethylation | C3:Carbamidomethylation:1000.00;C14:Carbamidomethylation:1000.00 | PEAKS DB |
| VIGGDEC(+57.02)NINEHPFL.V | N | 59.49 | 1712.7828 | 15 | 0.3 | 857.3989 | 2 | 16.34 | 7 | F7:6526 | DaRuMP\_F7B.raw |  |  |  |  |  |  | 6.2556E5 |  |  |  | 1 | 0 | 0 | 0 | 0 | 0 | 0 | 1 | 0 | 0 | 0 | 1 | 15 | Carbamidomethylation | C7:Carbamidomethylation:1000.00 | PEAKS DB |
| L.INQEWVLTAAR.C | N | 52.15 | 1299.6935 | 11 | 0.2 | 650.8541 | 2 | 14.19 | 5 | F5:4651 | DaRuMP\_F6.raw |  |  |  |  | 2.7645E5 |  |  |  |  |  | 1 | 0 | 0 | 0 | 0 | 1 | 0 | 0 | 0 | 0 | 0 | 33 | 43 |  |  | PEAKS DB |
| total 6 peptides |
| --- |

P30894|NGFV\_DABRR

back to list

  

| Protein Coverage
| Supporting Peptides
|

Protein Coverage:

Supporting Peptides:

| Peptide | Uniq | -10lgP | Mass | Length | ppm | m/z | z | RT | Fraction | Scan | Source File | Area F1 | Area F10 | Area F2\_3 | Area F5 | Area F6 | Area F7A | Area F7B | Area F8A | Area F8B | Area F9 | #Feature | #Feature F1 | #Feature F10 | #Feature F2\_3 | #Feature F5 | #Feature F6 | #Feature F7A | #Feature F7B | #Feature F8A | #Feature F8B | #Feature F9 | Start | End | PTM | AScore | Found By |
| --- | --- | --- | --- | --- | --- | --- | --- | --- | --- | --- | --- | --- | --- | --- | --- | --- | --- | --- | --- | --- | --- | --- | --- | --- | --- | --- | --- | --- | --- | --- | --- | --- | --- | --- | --- | --- | --- |
| K.HWNSYC(+57.02)TTTDTFVR.A | N | 86.15 | 1786.7733 | 14 | -3.6 | 894.3907 | 2 | 11.66 | 4 | F4:2511 | DaRuMP\_F5.raw |  |  |  | 6.4449E7 | 9.8334E5 |  |  |  |  |  | 3 | 0 | 0 | 0 | 2 | 1 | 0 | 0 | 0 | 0 | 0 | 72 | 85 | Carbamidomethylation | C6:Carbamidomethylation:1000.00 | PEAKS DB |
| K.NPNPVPSGC(+57.02)R.G | Y | 73.59 | 1096.5083 | 10 | -0.5 | 549.2612 | 2 | 10.74 | 5 | F5:1640 | DaRuMP\_F6.raw |  |  |  | 1.0845E7 | 5.2428E5 |  |  |  |  |  | 2 | 0 | 0 | 0 | 1 | 1 | 0 | 0 | 0 | 0 | 0 | 57 | 66 | Carbamidomethylation | C9:Carbamidomethylation:1000.00 | PEAKS DB |
| R.INTAC(+57.02)VC(+57.02)VISR.K | Y | 71.71 | 1291.6377 | 11 | -0.1 | 646.8260 | 2 | 11.72 | 5 | F5:2564 | DaRuMP\_F6.raw |  |  |  | 1.7738E6 | 4.6362E6 |  | 1.9163E5 |  |  |  | 4 | 0 | 0 | 0 | 2 | 1 | 0 | 1 | 0 | 0 | 0 | 101 | 111 | Carbamidomethylation | C5:Carbamidomethylation:1000.00;C7:Carbamidomethylation:1000.00 | PEAKS DB |
| R.FIRINTAC(+57.02)VC(+57.02)VISR.K | Y | 66.36 | 1707.8912 | 14 | -0.9 | 570.3038 | 3 | 12.25 | 4 | F4:3043 | DaRuMP\_F5.raw |  |  |  | 2.9174E5 |  |  |  |  |  |  | 1 | 0 | 0 | 0 | 1 | 0 | 0 | 0 | 0 | 0 | 0 | 98 | 111 | Carbamidomethylation | C8:Carbamidomethylation:1000.00;C10:Carbamidomethylation:1000.00 | PEAKS DB |
| W.NSYC(+57.02)TTTDTFVR.A | N | 64.53 | 1463.6351 | 12 | 0.8 | 732.8254 | 2 | 11.62 | 4 | F4:2468 | DaRuMP\_F5.raw |  |  |  | 8.0646E5 |  |  |  |  |  |  | 1 | 0 | 0 | 0 | 1 | 0 | 0 | 0 | 0 | 0 | 0 | 74 | 85 | Carbamidomethylation | C4:Carbamidomethylation:1000.00 | PEAKS DB |
| K.QYFFETK.C | N | 60.12 | 961.4545 | 7 | -1.3 | 481.7339 | 2 | 12.18 | 5 | F5:2995 | DaRuMP\_F6.raw |  |  |  | 8.2477E7 | 8.2976E6 |  |  |  |  |  | 2 | 0 | 0 | 0 | 1 | 1 | 0 | 0 | 0 | 0 | 0 | 48 | 54 |  |  | PEAKS DB |
| N.SYC(+57.02)TTTDTFVR.A | N | 58.35 | 1349.5922 | 11 | 0.0 | 675.8033 | 2 | 11.60 | 4 | F4:2409 | DaRuMP\_F5.raw |  |  |  | 1.7586E7 | 5.092E5 |  |  |  |  |  | 2 | 0 | 0 | 0 | 1 | 1 | 0 | 0 | 0 | 0 | 0 | 75 | 85 | Carbamidomethylation | C3:Carbamidomethylation:1000.00 | PEAKS DB |
| Y.C(+57.02)TTTDTFVR.A | N | 56.05 | 1099.4968 | 9 | 0.6 | 550.7560 | 2 | 11.30 | 4 | F4:2168 | DaRuMP\_F5.raw |  |  |  | 9.521E6 |  |  |  |  |  |  | 1 | 0 | 0 | 0 | 1 | 0 | 0 | 0 | 0 | 0 | 0 | 77 | 85 | Carbamidomethylation | C1:Carbamidomethylation:1000.00 | PEAKS DB |
| N.TAC(+57.02)VC(+57.02)VISR.K | N | 55.28 | 1064.5107 | 9 | 0.3 | 533.2628 | 2 | 11.34 | 4 | F4:2206 | DaRuMP\_F5.raw |  |  |  | 3.4147E5 |  |  |  |  |  |  | 1 | 0 | 0 | 0 | 1 | 0 | 0 | 0 | 0 | 0 | 0 | 103 | 111 | Carbamidomethylation | C3:Carbamidomethylation:1000.00;C5:Carbamidomethylation:1000.00 | PEAKS DB |
| S.YC(+57.02)TTTDTFVR.A | N | 53.44 | 1262.5602 | 10 | 0.9 | 632.2879 | 2 | 11.56 | 4 | F4:2432 | DaRuMP\_F5.raw |  |  |  | 1.2365E5 |  |  |  |  |  |  | 1 | 0 | 0 | 0 | 1 | 0 | 0 | 0 | 0 | 0 | 0 | 76 | 85 | Carbamidomethylation | C2:Carbamidomethylation:1000.00 | PEAKS DB |
| D.VNLNNNVYK.Q | Y | 52.46 | 1076.5614 | 9 | 1.5 | 539.2888 | 2 | 11.27 | 4 | F4:2139 | DaRuMP\_F5.raw |  |  |  | 1.1468E6 |  |  |  |  |  |  | 1 | 0 | 0 | 0 | 1 | 0 | 0 | 0 | 0 | 0 | 0 | 39 | 47 |  |  | PEAKS DB |
| total 11 peptides |
| --- |

Q2ES47|VKT4\_DABRR

back to list

  

| Protein Coverage
| Supporting Peptides
|

Protein Coverage:

Supporting Peptides:

| Peptide | Uniq | -10lgP | Mass | Length | ppm | m/z | z | RT | Fraction | Scan | Source File | Area F1 | Area F10 | Area F2\_3 | Area F5 | Area F6 | Area F7A | Area F7B | Area F8A | Area F8B | Area F9 | #Feature | #Feature F1 | #Feature F10 | #Feature F2\_3 | #Feature F5 | #Feature F6 | #Feature F7A | #Feature F7B | #Feature F8A | #Feature F8B | #Feature F9 | Start | End | PTM | AScore | Found By |
| --- | --- | --- | --- | --- | --- | --- | --- | --- | --- | --- | --- | --- | --- | --- | --- | --- | --- | --- | --- | --- | --- | --- | --- | --- | --- | --- | --- | --- | --- | --- | --- | --- | --- | --- | --- | --- | --- |
| Q.GFIYGGC(+57.02)GGNANNFETR.D | Y | 86.62 | 1832.7900 | 17 | 0.3 | 917.4026 | 2 | 12.06 | 3 | F3:2837 | DaRuMP\_F2\_F3.raw | 2.3931E5 |  | 5.5296E5 |  |  |  |  |  |  |  | 2 | 1 | 0 | 1 | 0 | 0 | 0 | 0 | 0 | 0 | 0 | 58 | 74 | Carbamidomethylation | C7:Carbamidomethylation:1000.00 | PEAKS DB |
| F.IYGGC(+57.02)GGNANNFETR.D | N | 84.62 | 1628.7001 | 15 | -2.6 | 815.3552 | 2 | 11.33 | 3 | F3:2146 | DaRuMP\_F2\_F3.raw | 9.5628E6 |  | 1.7411E7 | 9.596E5 |  |  |  |  |  |  | 3 | 1 | 0 | 1 | 1 | 0 | 0 | 0 | 0 | 0 | 0 | 60 | 74 | Carbamidomethylation | C5:Carbamidomethylation:1000.00 | PEAKS DB |
| K.FC(+57.02)HLPVDSGIC(+57.02)R.A | Y | 82.74 | 1459.6700 | 12 | -3.0 | 487.5625 | 3 | 11.60 | 3 | F3:2389 | DaRuMP\_F2\_F3.raw | 2.3284E7 |  | 5.0752E7 | 2.1399E6 |  |  |  |  |  |  | 4 | 1 | 0 | 2 | 1 | 0 | 0 | 0 | 0 | 0 | 0 | 30 | 41 | Carbamidomethylation | C2:Carbamidomethylation:1000.00;C11:Carbamidomethylation:1000.00 | PEAKS DB |
| A.SNQC(+57.02)QGFIYGGC(+57.02)GGNANNFETR.D | Y | 80.26 | 2450.0127 | 22 | 1.6 | 817.6795 | 3 | 12.00 | 3 | F3:2776 | DaRuMP\_F2\_F3.raw | 1.9473E6 |  | 3.1496E6 |  |  |  |  |  |  |  | 4 | 2 | 0 | 2 | 0 | 0 | 0 | 0 | 0 | 0 | 0 | 53 | 74 | Carbamidomethylation | C4:Carbamidomethylation:1000.00;C12:Carbamidomethylation:1000.00 | PEAKS DB |
| C.QGFIYGGC(+57.02)GGNANNFETR.D | Y | 71.76 | 1960.8486 | 18 | 1.1 | 981.4327 | 2 | 12.07 | 3 | F3:2869 | DaRuMP\_F2\_F3.raw |  |  | 6.4527E5 |  |  |  |  |  |  |  | 1 | 0 | 0 | 1 | 0 | 0 | 0 | 0 | 0 | 0 | 0 | 57 | 74 | Carbamidomethylation | C8:Carbamidomethylation:1000.00 | PEAKS DB |
| R.FYYNPASNQC(+57.02)QGF.I | N | 66.89 | 1594.6510 | 13 | 1.1 | 798.3337 | 2 | 19.30 | 3 | F3:5295 | DaRuMP\_F2\_F3.raw | 1.2086E7 |  | 1.8478E7 |  |  |  |  |  |  |  | 2 | 1 | 0 | 1 | 0 | 0 | 0 | 0 | 0 | 0 | 0 | 47 | 59 | Carbamidomethylation | C10:Carbamidomethylation:1000.00 | PEAKS DB |
| N.QC(+57.02)QGFIYGGC(+57.02)GGNANNFETR.D | Y | 65.27 | 2248.9377 | 20 | 0.6 | 1125.4768 | 2 | 12.04 | 3 | F3:2820 | DaRuMP\_F2\_F3.raw | 5.7944E5 |  | 9.0456E5 |  |  |  |  |  |  |  | 3 | 1 | 0 | 2 | 0 | 0 | 0 | 0 | 0 | 0 | 0 | 55 | 74 | Carbamidomethylation | C2:Carbamidomethylation:1000.00;C10:Carbamidomethylation:1000.00 | PEAKS DB |
| Y.GGC(+57.02)GGNANNFETR.D | N | 55.54 | 1352.5527 | 13 | 0.7 | 677.2841 | 2 | 10.77 | 3 | F3:1663 | DaRuMP\_F2\_F3.raw |  |  | 3.6976E4 |  |  |  |  |  |  |  | 1 | 0 | 0 | 1 | 0 | 0 | 0 | 0 | 0 | 0 | 0 | 62 | 74 | Carbamidomethylation | C3:Carbamidomethylation:1000.00 | PEAKS DB |
| R.FYYNPASNQC(+57.02).Q | N | 53.29 | 1262.5026 | 10 | 0.2 | 632.2587 | 2 | 12.80 | 3 | F3:3327 | DaRuMP\_F2\_F3.raw |  |  | 3.4592E5 |  |  |  |  |  |  |  | 1 | 0 | 0 | 1 | 0 | 0 | 0 | 0 | 0 | 0 | 0 | 47 | 56 | Carbamidomethylation | C10:Carbamidomethylation:1000.00 | PEAKS DB |
| R.FYYNPASNQC(+57.02)Q.G | N | 52.69 | 1390.5612 | 11 | 0.7 | 696.2883 | 2 | 12.27 | 1 | F1:3065 | DaRuMP\_F1.raw | 1.5225E5 |  |  |  |  |  |  |  |  |  | 1 | 1 | 0 | 0 | 0 | 0 | 0 | 0 | 0 | 0 | 0 | 47 | 57 | Carbamidomethylation | C10:Carbamidomethylation:1000.00 | PEAKS DB |
| total 10 peptides |
| --- |

Q4PRC6|SL7\_DABSI

back to list

  

| Protein Coverage
| Supporting Peptides
|

Protein Coverage:

Supporting Peptides:

| Peptide | Uniq | -10lgP | Mass | Length | ppm | m/z | z | RT | Fraction | Scan | Source File | Area F1 | Area F10 | Area F2\_3 | Area F5 | Area F6 | Area F7A | Area F7B | Area F8A | Area F8B | Area F9 | #Feature | #Feature F1 | #Feature F10 | #Feature F2\_3 | #Feature F5 | #Feature F6 | #Feature F7A | #Feature F7B | #Feature F8A | #Feature F8B | #Feature F9 | Start | End | PTM | AScore | Found By |
| --- | --- | --- | --- | --- | --- | --- | --- | --- | --- | --- | --- | --- | --- | --- | --- | --- | --- | --- | --- | --- | --- | --- | --- | --- | --- | --- | --- | --- | --- | --- | --- | --- | --- | --- | --- | --- | --- |
| R.WSDGVNLDYK.A | Y | 82.04 | 1195.5509 | 10 | 0.3 | 598.7829 | 2 | 12.19 | 9 | F9:2993 | DaRuMP\_F8B.raw |  |  |  |  |  |  | 3.0104E6 |  | 8.2446E5 |  | 2 | 0 | 0 | 0 | 0 | 0 | 0 | 1 | 0 | 1 | 0 | 104 | 113 |  |  | PEAKS DB |
| K.QDC(+57.02)LSDWSFYEGYC(+57.02)YK.V | N | 81.28 | 2119.8291 | 16 | 0.3 | 1060.9221 | 2 | 28.22 | 9 | F9:10430 | DaRuMP\_F8B.raw |  |  |  |  |  |  | 8.9231E6 | 3.1833E6 | 3.7588E7 |  | 4 | 0 | 0 | 0 | 0 | 0 | 0 | 1 | 1 | 2 | 0 | 25 | 40 | Carbamidomethylation | C3:Carbamidomethylation:1000.00;C14:Carbamidomethylation:1000.00 | PEAKS DB |
| K.FC(+57.02)NEQVNGGYLVSFR.S | Y | 79.06 | 1788.8253 | 15 | 0.6 | 895.4205 | 2 | 17.18 | 9 | F9:6232 | DaRuMP\_F8B.raw |  |  |  |  |  |  |  |  | 5.2244E6 |  | 1 | 0 | 0 | 0 | 0 | 0 | 0 | 0 | 0 | 1 | 0 | 54 | 68 | Carbamidomethylation | C2:Carbamidomethylation:1000.00 | PEAKS DB |
| R.FDFFWIGLR.D | Y | 76.06 | 1199.6127 | 9 | 0.4 | 600.8138 | 2 | 53.80 | 9 | F9:17642 | DaRuMP\_F8B.raw |  |  |  |  |  | 1.1977E5 | 1.046E7 | 7.9285E6 | 1.7319E7 | 1.0207E5 | 9 | 0 | 0 | 0 | 0 | 0 | 1 | 2 | 2 | 3 | 1 | 86 | 94 |  |  | PEAKS DB |
| A.KQDC(+57.02)LSDWSFYEGYC(+57.02)YK.V | N | 68.02 | 2247.9241 | 17 | -0.9 | 1124.9683 | 2 | 17.56 | 7 | F7:7737 | DaRuMP\_F7B.raw |  |  |  |  |  |  | 1.5535E7 | 1.9403E6 | 2.4047E7 |  | 5 | 0 | 0 | 0 | 0 | 0 | 0 | 2 | 1 | 2 | 0 | 24 | 40 | Carbamidomethylation | C4:Carbamidomethylation:1000.00;C15:Carbamidomethylation:1000.00 | PEAKS DB |
| R.SSEEMDFVIR.M | Y | 66.78 | 1211.5492 | 10 | -0.1 | 606.7818 | 2 | 17.31 | 8 | F8:3875 | DaRuMP\_F8A.raw |  | 9.6293E4 |  |  |  |  | 8.0695E6 | 1.0214E6 | 1.2077E7 |  | 4 | 0 | 1 | 0 | 0 | 0 | 0 | 1 | 1 | 1 | 0 | 69 | 78 |  |  | PEAKS DB |
| K.TTDNQWLR.W | Y | 62.55 | 1032.4989 | 8 | -1.8 | 517.2558 | 2 | 11.71 | 7 | F7:2518 | DaRuMP\_F7B.raw |  |  |  |  |  |  | 3.0248E6 | 1.2967E5 | 4.0474E6 |  | 3 | 0 | 0 | 0 | 0 | 0 | 0 | 1 | 1 | 1 | 0 | 126 | 133 |  |  | PEAKS DB |
| W.SDGVNLDYK.A | Y | 60.01 | 1009.4716 | 9 | 0.6 | 505.7434 | 2 | 11.50 | 9 | F9:2351 | DaRuMP\_F8B.raw |  |  |  |  |  |  | 5.4295E5 |  | 4.3639E5 |  | 2 | 0 | 0 | 0 | 0 | 0 | 0 | 1 | 0 | 1 | 0 | 105 | 113 |  |  | PEAKS DB |
| R.EPNC(+57.02)FVSK.T | Y | 53.54 | 979.4433 | 8 | -0.3 | 490.7288 | 2 | 11.12 | 10 | F10:1886 | DaRuMP\_F9.raw |  |  |  |  |  |  |  |  |  | 1.8329E4 | 1 | 0 | 0 | 0 | 0 | 0 | 0 | 0 | 0 | 0 | 1 | 118 | 125 | Carbamidomethylation | C4:Carbamidomethylation:1000.00 | PEAKS DB |
| R.MTFPIFR.F | Y | 52.32 | 910.4734 | 7 | 0.1 | 456.2440 | 2 | 21.52 | 7 | F7:10743 | DaRuMP\_F7B.raw |  |  |  |  |  |  | 4.3061E5 |  |  |  | 1 | 0 | 0 | 0 | 0 | 0 | 0 | 1 | 0 | 0 | 0 | 79 | 85 |  |  | PEAKS DB |
| R.M(+15.99)TFPIFR.F | Y | 52.30 | 926.4684 | 7 | 0.5 | 464.2417 | 2 | 15.23 | 2 | F2:4447 | DaRuMP\_F10.raw |  | 8.5708E4 |  |  |  |  |  |  |  |  | 1 | 0 | 1 | 0 | 0 | 0 | 0 | 0 | 0 | 0 | 0 | 79 | 85 | Oxidation (M) | M1:Oxidation (M):1000.00 | PEAKS DB |
| total 11 peptides |
| --- |

O43790|KRT86\_HUMAN

back to list

  

| Protein Coverage
| Supporting Peptides
|

Protein Coverage:

Supporting Peptides:

| Peptide | Uniq | -10lgP | Mass | Length | ppm | m/z | z | RT | Fraction | Scan | Source File | Area F1 | Area F10 | Area F2\_3 | Area F5 | Area F6 | Area F7A | Area F7B | Area F8A | Area F8B | Area F9 | #Feature | #Feature F1 | #Feature F10 | #Feature F2\_3 | #Feature F5 | #Feature F6 | #Feature F7A | #Feature F7B | #Feature F8A | #Feature F8B | #Feature F9 | Start | End | PTM | AScore | Found By |
| --- | --- | --- | --- | --- | --- | --- | --- | --- | --- | --- | --- | --- | --- | --- | --- | --- | --- | --- | --- | --- | --- | --- | --- | --- | --- | --- | --- | --- | --- | --- | --- | --- | --- | --- | --- | --- | --- |
| K.LEAAVAQSEQQGEAALSDAR.C | Y | 98.39 | 2042.9868 | 20 | 0.8 | 1022.5015 | 2 | 11.89 | 3 | F3:2697 | DaRuMP\_F2\_F3.raw |  |  | 1.1444E5 |  |  |  |  |  |  |  | 1 | 0 | 0 | 1 | 0 | 0 | 0 | 0 | 0 | 0 | 0 | 346 | 365 |  |  | PEAKS DB |
| K.AQYDDIVTR.S | Y | 70.55 | 1079.5247 | 9 | -0.4 | 540.7694 | 2 | 11.49 | 3 | F3:2304 | DaRuMP\_F2\_F3.raw |  |  | 1.0993E5 |  |  |  |  |  |  |  | 1 | 0 | 0 | 1 | 0 | 0 | 0 | 0 | 0 | 0 | 0 | 280 | 288 |  |  | PEAKS DB |
| R.ATAENEFVALK.K | Y | 65.02 | 1191.6135 | 11 | -0.3 | 596.8138 | 2 | 11.99 | 3 | F3:2775 | DaRuMP\_F2\_F3.raw |  |  | 8.1668E4 |  |  |  |  |  |  |  | 1 | 0 | 0 | 1 | 0 | 0 | 0 | 0 | 0 | 0 | 0 | 202 | 212 |  |  | PEAKS DB |
| K.LAELEGALQK.A | N | 60.61 | 1070.5972 | 10 | 0.8 | 536.3063 | 2 | 11.80 | 3 | F3:2606 | DaRuMP\_F2\_F3.raw |  |  | 2.276E5 |  |  |  |  |  |  |  | 1 | 0 | 0 | 1 | 0 | 0 | 0 | 0 | 0 | 0 | 0 | 368 | 377 |  |  | PEAKS DB |
| K.LGLDIEIATYR.R | N | 58.31 | 1262.6870 | 11 | 0.9 | 632.3513 | 2 | 24.34 | 9 | F9:9182 | DaRuMP\_F8B.raw |  |  |  |  |  |  |  |  | 3.0158E4 |  | 1 | 0 | 0 | 0 | 0 | 0 | 0 | 0 | 0 | 1 | 0 | 397 | 407 |  |  | PEAKS DB |
| R.C(+57.02)C(+57.02)ITAAPYR.G | Y | 56.82 | 1110.4950 | 9 | -3.8 | 556.2527 | 2 | 11.33 | 3 | F3:2148 | DaRuMP\_F2\_F3.raw |  |  | 3.1563E4 |  |  |  |  |  |  |  | 1 | 0 | 0 | 1 | 0 | 0 | 0 | 0 | 0 | 0 | 0 | 25 | 33 | Carbamidomethylation | C1:Carbamidomethylation:1000.00;C2:Carbamidomethylation:1000.00 | PEAKS DB |
| R.TKEEINELNR.M | N | 53.74 | 1244.6360 | 10 | 0.0 | 415.8860 | 3 | 10.81 | 3 | F3:1680 | DaRuMP\_F2\_F3.raw |  |  | 1.0146E4 |  |  |  |  |  |  |  | 1 | 0 | 0 | 1 | 0 | 0 | 0 | 0 | 0 | 0 | 0 | 318 | 327 |  |  | PEAKS DB |
| R.AEAESWYR.S | N | 52.72 | 1010.4457 | 8 | 0.5 | 506.2304 | 2 | 11.49 | 3 | F3:2305 | DaRuMP\_F2\_F3.raw |  |  | 5.3586E4 |  |  |  |  |  |  |  | 1 | 0 | 0 | 1 | 0 | 0 | 0 | 0 | 0 | 0 | 0 | 291 | 298 |  |  | PEAKS DB |
| total 8 peptides |
| --- |

P08779|K1C16\_HUMAN

back to list

  

| Protein Coverage
| Supporting Peptides
|

Protein Coverage:

Supporting Peptides:

| Peptide | Uniq | -10lgP | Mass | Length | ppm | m/z | z | RT | Fraction | Scan | Source File | Area F1 | Area F10 | Area F2\_3 | Area F5 | Area F6 | Area F7A | Area F7B | Area F8A | Area F8B | Area F9 | #Feature | #Feature F1 | #Feature F10 | #Feature F2\_3 | #Feature F5 | #Feature F6 | #Feature F7A | #Feature F7B | #Feature F8A | #Feature F8B | #Feature F9 | Start | End | PTM | AScore | Found By |
| --- | --- | --- | --- | --- | --- | --- | --- | --- | --- | --- | --- | --- | --- | --- | --- | --- | --- | --- | --- | --- | --- | --- | --- | --- | --- | --- | --- | --- | --- | --- | --- | --- | --- | --- | --- | --- | --- |
| R.APSTYGGGLSVSSR.F | Y | 73.75 | 1337.6575 | 14 | 0.3 | 669.8362 | 2 | 11.43 | 3 | F3:2254 | DaRuMP\_F2\_F3.raw |  |  | 5.9728E4 |  |  |  |  |  |  | 0 | 1 | 0 | 0 | 1 | 0 | 0 | 0 | 0 | 0 | 0 | 0 | 42 | 55 |  |  | PEAKS DB |
| R.ALEEANADLEVK.I | N | 68.92 | 1300.6510 | 12 | -0.3 | 651.3326 | 2 | 11.67 | 10 | F10:2424 | DaRuMP\_F9.raw |  |  |  | 0 | 1.4338E5 |  |  |  |  | 9.0463E4 | 2 | 0 | 0 | 0 | 0 | 1 | 0 | 0 | 0 | 0 | 1 | 137 | 148 |  |  | PEAKS DB |
| K.EVASNSELVQSSR.S | Y | 64.85 | 1404.6844 | 13 | 0.2 | 703.3496 | 2 | 11.15 | 5 | F5:2029 | DaRuMP\_F6.raw |  |  |  |  | 3.4564E4 |  |  |  |  | 3.2701E4 | 2 | 0 | 0 | 0 | 0 | 1 | 0 | 0 | 0 | 0 | 1 | 318 | 330 |  |  | PEAKS DB |
| R.ISSVLAGGSC(+57.02)R.A | N | 59.84 | 1105.5549 | 11 | 0.6 | 553.7851 | 2 | 11.36 | 10 | F10:2130 | DaRuMP\_F9.raw | 3.8905E4 |  |  |  |  |  |  |  |  | 1.3619E4 | 2 | 1 | 0 | 0 | 0 | 0 | 0 | 0 | 0 | 0 | 1 | 31 | 41 | Carbamidomethylation | C10:Carbamidomethylation:1000.00 | PEAKS DB |
| R.VLDELTLAR.T | N | 58.45 | 1028.5865 | 9 | 0.6 | 515.3008 | 2 | 13.41 | 3 | F3:3664 | DaRuMP\_F2\_F3.raw |  |  | 4.3847E4 |  |  |  | 5.0014E5 |  | 7.5689E4 |  | 3 | 0 | 0 | 1 | 0 | 0 | 0 | 1 | 0 | 1 | 0 | 226 | 234 |  |  | PEAKS DB |
| K.GSC(+57.02)GIGGGIGGGSSR.I | N | 57.68 | 1277.5782 | 15 | 0.6 | 639.7968 | 2 | 11.16 | 10 | F10:1922 | DaRuMP\_F9.raw |  |  |  |  |  |  |  |  |  | 1.7567E4 | 1 | 0 | 0 | 0 | 0 | 0 | 0 | 0 | 0 | 0 | 1 | 16 | 30 | Carbamidomethylation | C3:Carbamidomethylation:1000.00 | PEAKS DB |
| K.IIAATIENAQPILQIDNAR.L | Y | 55.31 | 2063.1375 | 19 | 1.0 | 688.7205 | 3 | 24.05 | 5 | F5:10830 | DaRuMP\_F6.raw |  |  |  |  | 1.3482E5 |  |  |  |  |  | 1 | 0 | 0 | 0 | 0 | 1 | 0 | 0 | 0 | 0 | 0 | 178 | 196 |  |  | PEAKS DB |
| R.LEQEIATYR.R | N | 55.07 | 1121.5717 | 9 | 0.7 | 561.7935 | 2 | 11.47 | 7 | F7:2292 | DaRuMP\_F7B.raw | 1.1095E5 |  |  |  |  |  | 1.2503E5 |  |  | 9.8436E4 | 3 | 1 | 0 | 0 | 0 | 0 | 0 | 1 | 0 | 0 | 1 | 410 | 418 |  |  | PEAKS DB |
| K.ASLENSLEETK.G | N | 54.81 | 1219.5931 | 11 | 0.0 | 610.8038 | 2 | 11.52 | 1 | F1:2399 | DaRuMP\_F1.raw | 6.5853E4 |  |  |  |  |  |  |  |  | 7.657E4 | 2 | 1 | 0 | 0 | 0 | 0 | 0 | 0 | 0 | 0 | 1 | 355 | 365 |  |  | PEAKS DB |
| K.VTMQNLNDR.L | N | 53.34 | 1089.5237 | 9 | 0.3 | 545.7693 | 2 | 11.22 | 4 | F4:2061 | DaRuMP\_F5.raw |  |  |  | 3.3198E4 |  |  |  |  |  |  | 1 | 0 | 0 | 0 | 1 | 0 | 0 | 0 | 0 | 0 | 0 | 119 | 127 |  |  | PEAKS DB |
| R.TDLEMQIEGLK.E | Y | 52.84 | 1275.6381 | 11 | 0.7 | 638.8267 | 2 | 16.03 | 5 | F5:6082 | DaRuMP\_F6.raw |  |  |  |  | 9.8473E3 |  |  |  |  |  | 1 | 0 | 0 | 0 | 0 | 1 | 0 | 0 | 0 | 0 | 0 | 235 | 245 |  |  | PEAKS DB |
| R.DAETWFLSK.T | Y | 52.33 | 1095.5237 | 9 | 0.3 | 548.7693 | 2 | 20.87 | 1 | F1:5510 | DaRuMP\_F1.raw | 4.1435E4 |  |  |  |  |  |  |  |  |  | 1 | 1 | 0 | 0 | 0 | 0 | 0 | 0 | 0 | 0 | 0 | 303 | 311 |  |  | PEAKS DB |
| total 12 peptides |
| --- |

P02533|K1C14\_HUMAN

back to list

  

| Protein Coverage
| Supporting Peptides
|

Protein Coverage:

Supporting Peptides:

| Peptide | Uniq | -10lgP | Mass | Length | ppm | m/z | z | RT | Fraction | Scan | Source File | Area F1 | Area F10 | Area F2\_3 | Area F5 | Area F6 | Area F7A | Area F7B | Area F8A | Area F8B | Area F9 | #Feature | #Feature F1 | #Feature F10 | #Feature F2\_3 | #Feature F5 | #Feature F6 | #Feature F7A | #Feature F7B | #Feature F8A | #Feature F8B | #Feature F9 | Start | End | PTM | AScore | Found By |
| --- | --- | --- | --- | --- | --- | --- | --- | --- | --- | --- | --- | --- | --- | --- | --- | --- | --- | --- | --- | --- | --- | --- | --- | --- | --- | --- | --- | --- | --- | --- | --- | --- | --- | --- | --- | --- | --- |
| R.EVATNSELVQSGK.S | N | 72.11 | 1360.6833 | 13 | 0.5 | 681.3493 | 2 | 11.20 | 10 | F10:1968 | DaRuMP\_F9.raw |  |  |  | 1.3917E4 |  |  |  |  |  | 2.4107E4 | 2 | 0 | 0 | 0 | 1 | 0 | 0 | 0 | 0 | 0 | 1 | 316 | 328 |  |  | PEAKS DB |
| R.ALEEANADLEVK.I | N | 68.92 | 1300.6510 | 12 | -0.3 | 651.3326 | 2 | 11.67 | 10 | F10:2424 | DaRuMP\_F9.raw |  |  |  | 0 | 1.4338E5 |  |  |  |  | 9.0463E4 | 2 | 0 | 0 | 0 | 0 | 1 | 0 | 0 | 0 | 0 | 1 | 135 | 146 |  |  | PEAKS DB |
| K.ILTATVDNANVLLQIDNAR.L | Y | 65.07 | 2053.1167 | 19 | -0.1 | 1027.5656 | 2 | 28.80 | 9 | F9:10715 | DaRuMP\_F8B.raw |  |  |  |  |  |  |  |  | 9.1354E4 |  | 1 | 0 | 0 | 0 | 0 | 0 | 0 | 0 | 0 | 1 | 0 | 176 | 194 |  |  | PEAKS DB |
| R.APSTYGGGLSVSSSR.F | Y | 61.82 | 1424.6896 | 15 | -0.6 | 713.3516 | 2 | 11.43 | 4 | F4:2296 | DaRuMP\_F5.raw |  |  |  | 6.4481E4 |  |  |  |  |  |  | 1 | 0 | 0 | 0 | 1 | 0 | 0 | 0 | 0 | 0 | 0 | 42 | 56 |  |  | PEAKS DB |
| R.LLEGEDAHLSSSQFSSGSQSSR.D | Y | 60.80 | 2308.0566 | 22 | 1.2 | 770.3604 | 3 | 11.59 | 1 | F1:2444 | DaRuMP\_F1.raw | 0 |  |  |  |  |  |  |  |  |  | 0 | 0 | 0 | 0 | 0 | 0 | 0 | 0 | 0 | 0 | 0 | 418 | 439 |  |  | PEAKS DB |
| R.ISSVLAGGSC(+57.02)R.A | N | 59.84 | 1105.5549 | 11 | 0.6 | 553.7851 | 2 | 11.36 | 10 | F10:2130 | DaRuMP\_F9.raw | 3.8905E4 |  |  |  |  |  |  |  |  | 1.3619E4 | 2 | 1 | 0 | 0 | 0 | 0 | 0 | 0 | 0 | 0 | 1 | 31 | 41 | Carbamidomethylation | C10:Carbamidomethylation:1000.00 | PEAKS DB |
| R.VLDELTLAR.A | N | 58.45 | 1028.5865 | 9 | 0.6 | 515.3008 | 2 | 13.41 | 3 | F3:3664 | DaRuMP\_F2\_F3.raw |  |  | 4.3847E4 |  |  |  | 5.0014E5 |  | 7.5689E4 |  | 3 | 0 | 0 | 1 | 0 | 0 | 0 | 1 | 0 | 1 | 0 | 224 | 232 |  |  | PEAKS DB |
| K.GSC(+57.02)GIGGGIGGGSSR.I | N | 57.68 | 1277.5782 | 15 | 0.6 | 639.7968 | 2 | 11.16 | 10 | F10:1922 | DaRuMP\_F9.raw |  |  |  |  |  |  |  |  |  | 1.7567E4 | 1 | 0 | 0 | 0 | 0 | 0 | 0 | 0 | 0 | 0 | 1 | 16 | 30 | Carbamidomethylation | C3:Carbamidomethylation:1000.00 | PEAKS DB |
| K.DAEEWFFTK.T | Y | 57.38 | 1171.5186 | 9 | -0.3 | 586.7664 | 2 | 25.19 | 9 | F9:9480 | DaRuMP\_F8B.raw |  |  |  | 5.0549E4 | 1.1637E5 |  |  |  | 6.0812E4 |  | 3 | 0 | 0 | 0 | 1 | 1 | 0 | 0 | 0 | 1 | 0 | 301 | 309 |  |  | PEAKS DB |
| R.LEQEIATYR.R | N | 55.07 | 1121.5717 | 9 | 0.7 | 561.7935 | 2 | 11.47 | 7 | F7:2292 | DaRuMP\_F7B.raw | 1.1095E5 |  |  |  |  |  | 1.2503E5 |  |  | 9.8436E4 | 3 | 1 | 0 | 0 | 0 | 0 | 0 | 1 | 0 | 0 | 1 | 408 | 416 |  |  | PEAKS DB |
| K.ASLENSLEETK.G | N | 54.81 | 1219.5931 | 11 | 0.0 | 610.8038 | 2 | 11.52 | 1 | F1:2399 | DaRuMP\_F1.raw | 6.5853E4 |  |  |  |  |  |  |  |  | 7.657E4 | 2 | 1 | 0 | 0 | 0 | 0 | 0 | 0 | 0 | 0 | 1 | 353 | 363 |  |  | PEAKS DB |
| K.VTMQNLNDR.L | N | 53.34 | 1089.5237 | 9 | 0.3 | 545.7693 | 2 | 11.22 | 4 | F4:2061 | DaRuMP\_F5.raw |  |  |  | 3.3198E4 |  |  |  |  |  |  | 1 | 0 | 0 | 0 | 1 | 0 | 0 | 0 | 0 | 0 | 0 | 117 | 125 |  |  | PEAKS DB |
| total 12 peptides |
| --- |

Q4PRD1|SLLC1\_DABSI

back to list

  

| Protein Coverage
| Supporting Peptides
|

Protein Coverage:

Supporting Peptides:

| Peptide | Uniq | -10lgP | Mass | Length | ppm | m/z | z | RT | Fraction | Scan | Source File | Area F1 | Area F10 | Area F2\_3 | Area F5 | Area F6 | Area F7A | Area F7B | Area F8A | Area F8B | Area F9 | #Feature | #Feature F1 | #Feature F10 | #Feature F2\_3 | #Feature F5 | #Feature F6 | #Feature F7A | #Feature F7B | #Feature F8A | #Feature F8B | #Feature F9 | Start | End | PTM | AScore | Found By |
| --- | --- | --- | --- | --- | --- | --- | --- | --- | --- | --- | --- | --- | --- | --- | --- | --- | --- | --- | --- | --- | --- | --- | --- | --- | --- | --- | --- | --- | --- | --- | --- | --- | --- | --- | --- | --- | --- |
| A.VLDC(+57.02)PSGWLSYEQHC(+57.02)YK.G | Y | 86.59 | 2140.9346 | 17 | -5.3 | 1071.4689 | 2 | 16.88 | 8 | F8:3504 | DaRuMP\_F8A.raw |  |  |  |  |  | 3.242E5 | 7.5372E7 | 2.5215E6 | 7.9221E6 |  | 6 | 0 | 0 | 0 | 0 | 0 | 1 | 2 | 2 | 1 | 0 | 24 | 40 | Carbamidomethylation | C4:Carbamidomethylation:1000.00;C15:Carbamidomethylation:1000.00 | PEAKS DB |
| K.SMTC(+57.02)NFIAPVVC(+57.02)K.F | N | 79.38 | 1525.7091 | 13 | 0.1 | 763.8619 | 2 | 14.92 | 7 | F7:5322 | DaRuMP\_F7B.raw |  |  |  |  |  |  | 2.8509E6 |  | 5.5532E5 |  | 2 | 0 | 0 | 0 | 0 | 0 | 0 | 1 | 0 | 1 | 0 | 133 | 145 | Carbamidomethylation | C4:Carbamidomethylation:1000.00;C12:Carbamidomethylation:1000.00 | PEAKS DB |
| K.SM(+15.99)TC(+57.02)NFIAPVVC(+57.02)K.F | N | 79.24 | 1541.7041 | 13 | -1.0 | 771.8585 | 2 | 12.47 | 7 | F7:3244 | DaRuMP\_F7B.raw |  |  |  |  |  |  | 1.8621E6 |  |  |  | 1 | 0 | 0 | 0 | 0 | 0 | 0 | 1 | 0 | 0 | 0 | 133 | 145 | Oxidation (M); Carbamidomethylation | M2:Oxidation (M):1000.00;C4:Carbamidomethylation:1000.00;C12:Carbamidomethylation:1000.00 | PEAKS DB |
| K.ALAEESYC(+57.02)LIM(+15.99)ITHEK.E | N | 78.53 | 1922.9117 | 16 | 0.3 | 641.9780 | 3 | 13.47 | 7 | F7:4084 | DaRuMP\_F7B.raw |  |  |  |  |  |  | 7.9361E5 |  |  |  | 1 | 0 | 0 | 0 | 0 | 0 | 0 | 1 | 0 | 0 | 0 | 114 | 129 | Carbamidomethylation; Oxidation (M) | C8:Carbamidomethylation:1000.00;M11:Oxidation (M):1000.00 | PEAKS DB |
| E.NLEYPATWIGLGNMWK.D | N | 75.43 | 1891.9291 | 16 | 0.8 | 946.9725 | 2 | 47.25 | 7 | F7:23629 | DaRuMP\_F7B.raw |  |  |  |  |  |  | 6.6221E6 |  |  |  | 1 | 0 | 0 | 0 | 0 | 0 | 0 | 1 | 0 | 0 | 0 | 83 | 98 |  |  | PEAKS DB |
| K.ALAEESYC(+57.02)LIMITHEK.E | N | 75.09 | 1906.9169 | 16 | 0.6 | 636.6466 | 3 | 19.30 | 8 | F8:5458 | DaRuMP\_F8A.raw |  |  |  |  |  |  | 3.6414E6 | 3.0576E5 | 2.7651E6 |  | 3 | 0 | 0 | 0 | 0 | 0 | 0 | 1 | 1 | 1 | 0 | 114 | 129 | Carbamidomethylation | C8:Carbamidomethylation:1000.00 | PEAKS DB |
| E.NLEYPATWIGLGNM(+15.99)WK.D | N | 61.74 | 1907.9240 | 16 | 0.6 | 954.9698 | 2 | 37.60 | 7 | F7:19194 | DaRuMP\_F7B.raw |  |  |  |  |  |  | 2.2926E6 |  |  |  | 1 | 0 | 0 | 0 | 0 | 0 | 0 | 1 | 0 | 0 | 0 | 83 | 98 | Oxidation (M) | M14:Oxidation (M):1000.00 | PEAKS DB |
| K.GSHLVSLHSR.E | N | 57.25 | 1091.5836 | 10 | 0.8 | 546.7995 | 2 | 10.73 | 7 | F7:1599 | DaRuMP\_F7B.raw |  |  |  |  |  |  | 5.6617E5 |  |  |  | 1 | 0 | 0 | 0 | 0 | 0 | 0 | 1 | 0 | 0 | 0 | 61 | 70 |  |  | PEAKS DB |
| K.KGSHLVSLHSR.E | N | 52.34 | 1219.6785 | 11 | 1.0 | 407.5672 | 3 | 10.73 | 7 | F7:1593 | DaRuMP\_F7B.raw |  |  |  |  |  |  | 5.6236E5 |  |  |  | 1 | 0 | 0 | 0 | 0 | 0 | 0 | 1 | 0 | 0 | 0 | 60 | 70 |  |  | PEAKS DB |
| total 9 peptides |
| --- |

E0Y418|VSP1\_MACLB

back to list

  

| Protein Coverage
| Supporting Peptides
|

Protein Coverage:

Supporting Peptides:

| Peptide | Uniq | -10lgP | Mass | Length | ppm | m/z | z | RT | Fraction | Scan | Source File | Area F1 | Area F10 | Area F2\_3 | Area F5 | Area F6 | Area F7A | Area F7B | Area F8A | Area F8B | Area F9 | #Feature | #Feature F1 | #Feature F10 | #Feature F2\_3 | #Feature F5 | #Feature F6 | #Feature F7A | #Feature F7B | #Feature F8A | #Feature F8B | #Feature F9 | Start | End | PTM | AScore | Found By |
| --- | --- | --- | --- | --- | --- | --- | --- | --- | --- | --- | --- | --- | --- | --- | --- | --- | --- | --- | --- | --- | --- | --- | --- | --- | --- | --- | --- | --- | --- | --- | --- | --- | --- | --- | --- | --- | --- |
| Q.GGIDTC(+57.02)LADSGGPLIC(+57.02)NGQFQGIVAWGR.H | Y | 75.34 | 2918.3804 | 28 | 1.3 | 973.8020 | 3 | 42.80 | 7 | F7:21656 | DaRuMP\_F7B.raw |  |  |  |  |  |  | 1.1307E7 | 1.5465E6 |  |  | 3 | 0 | 0 | 0 | 0 | 0 | 0 | 2 | 1 | 0 | 0 | 197 | 224 | Carbamidomethylation | C6:Carbamidomethylation:1000.00;C16:Carbamidomethylation:1000.00 | PEAKS DB |
| F.SLPSSPPTVGSVC(+57.02)R.I | Y | 74.68 | 1442.7188 | 14 | -1.8 | 722.3654 | 2 | 11.73 | 7 | F7:2474 | DaRuMP\_F7B.raw |  |  |  |  |  |  | 2.8954E8 | 0 | 2.1337E7 |  | 2 | 0 | 0 | 0 | 0 | 0 | 0 | 1 | 0 | 1 | 0 | 132 | 145 | Carbamidomethylation | C13:Carbamidomethylation:1000.00 | PEAKS DB |
| C.NGQFQGIVAWGR.H | Y | 69.49 | 1331.6735 | 12 | 0.1 | 666.8441 | 2 | 16.38 | 7 | F7:6598 | DaRuMP\_F7B.raw |  |  |  |  |  |  | 7.116E5 |  |  |  | 1 | 0 | 0 | 0 | 0 | 0 | 0 | 1 | 0 | 0 | 0 | 213 | 224 |  |  | PEAKS DB |
| L.IC(+57.02)NGQFQGIVAWGR.H | Y | 69.11 | 1604.7882 | 14 | 0.1 | 803.4015 | 2 | 18.79 | 7 | F7:8567 | DaRuMP\_F7B.raw |  |  |  |  |  |  | 1.3102E6 |  | 2.3661E5 |  | 2 | 0 | 0 | 0 | 0 | 0 | 0 | 1 | 0 | 1 | 0 | 211 | 224 | Carbamidomethylation | C2:Carbamidomethylation:1000.00 | PEAKS DB |
| H.IAPFSLPSSPPTVGSVC(+57.02)R.I | Y | 60.38 | 1870.9611 | 18 | 0.9 | 936.4886 | 2 | 22.39 | 7 | F7:11419 | DaRuMP\_F7B.raw |  |  |  |  |  |  | 1.7428E7 |  | 5.701E6 |  | 3 | 0 | 0 | 0 | 0 | 0 | 0 | 1 | 0 | 2 | 0 | 128 | 145 | Carbamidomethylation | C17:Carbamidomethylation:1000.00 | PEAKS DB |
| D.SGGPLIC(+57.02)NGQFQGIVAWGR.H | Y | 58.07 | 2015.9999 | 19 | 0.4 | 1009.0076 | 2 | 30.45 | 9 | F9:11154 | DaRuMP\_F8B.raw |  |  |  |  |  |  |  |  | 1.2983E5 |  | 1 | 0 | 0 | 0 | 0 | 0 | 0 | 0 | 0 | 1 | 0 | 206 | 224 | Carbamidomethylation | C7:Carbamidomethylation:1000.00 | PEAKS DB |
| N.GQFQGIVAWGR.H | Y | 58.05 | 1217.6305 | 11 | 0.4 | 609.8228 | 2 | 16.65 | 9 | F9:5803 | DaRuMP\_F8B.raw |  |  |  |  |  |  | 9.849E5 |  | 2.5788E5 |  | 2 | 0 | 0 | 0 | 0 | 0 | 0 | 1 | 0 | 1 | 0 | 214 | 224 |  |  | PEAKS DB |
| E.IYDYSVC(+57.02)R.K | Y | 57.09 | 1074.4805 | 8 | 1.5 | 538.2483 | 2 | 11.50 | 9 | F9:2341 | DaRuMP\_F8B.raw |  |  |  |  |  |  | 8.3472E7 |  | 5.3112E6 |  | 2 | 0 | 0 | 0 | 0 | 0 | 0 | 1 | 0 | 1 | 0 | 170 | 177 | Carbamidomethylation | C7:Carbamidomethylation:1000.00 | PEAKS DB |
| M.FFC(+57.02)LSNK.S | N | 56.09 | 914.4320 | 7 | 2.5 | 458.2233 | 2 | 12.01 | 6 | F6:2752 | DaRuMP\_F7A.raw |  |  |  |  | 2.015E6 | 1.6117E6 | 2.3973E6 |  |  |  | 3 | 0 | 0 | 0 | 0 | 1 | 1 | 1 | 0 | 0 | 0 | 98 | 104 | Carbamidomethylation | C3:Carbamidomethylation:1000.00 | PEAKS DB |
| total 9 peptides |
| --- |

P25428|NGFV\_MACLB

back to list

  

| Protein Coverage
| Supporting Peptides
|

Protein Coverage:

Supporting Peptides:

| Peptide | Uniq | -10lgP | Mass | Length | ppm | m/z | z | RT | Fraction | Scan | Source File | Area F1 | Area F10 | Area F2\_3 | Area F5 | Area F6 | Area F7A | Area F7B | Area F8A | Area F8B | Area F9 | #Feature | #Feature F1 | #Feature F10 | #Feature F2\_3 | #Feature F5 | #Feature F6 | #Feature F7A | #Feature F7B | #Feature F8A | #Feature F8B | #Feature F9 | Start | End | PTM | AScore | Found By |
| --- | --- | --- | --- | --- | --- | --- | --- | --- | --- | --- | --- | --- | --- | --- | --- | --- | --- | --- | --- | --- | --- | --- | --- | --- | --- | --- | --- | --- | --- | --- | --- | --- | --- | --- | --- | --- | --- |
| K.HWNSYC(+57.02)TTTDTFVR.A | N | 86.15 | 1786.7733 | 14 | -3.6 | 894.3907 | 2 | 11.66 | 4 | F4:2511 | DaRuMP\_F5.raw |  |  |  | 6.4449E7 | 9.8334E5 |  |  |  |  |  | 3 | 0 | 0 | 0 | 2 | 1 | 0 | 0 | 0 | 0 | 0 | 199 | 212 | Carbamidomethylation | C6:Carbamidomethylation:1000.00 | PEAKS DB |
| W.NSYC(+57.02)TTTDTFVR.A | N | 64.53 | 1463.6351 | 12 | 0.8 | 732.8254 | 2 | 11.62 | 4 | F4:2468 | DaRuMP\_F5.raw |  |  |  | 8.0646E5 |  |  |  |  |  |  | 1 | 0 | 0 | 0 | 1 | 0 | 0 | 0 | 0 | 0 | 0 | 201 | 212 | Carbamidomethylation | C4:Carbamidomethylation:1000.00 | PEAKS DB |
| R.QYFFETK.C | N | 60.12 | 961.4545 | 7 | -1.3 | 481.7339 | 2 | 12.18 | 5 | F5:2995 | DaRuMP\_F6.raw |  |  |  | 8.2477E7 | 8.2976E6 |  |  |  |  |  | 2 | 0 | 0 | 0 | 1 | 1 | 0 | 0 | 0 | 0 | 0 | 175 | 181 |  |  | PEAKS DB |
| N.SYC(+57.02)TTTDTFVR.A | N | 58.35 | 1349.5922 | 11 | 0.0 | 675.8033 | 2 | 11.60 | 4 | F4:2409 | DaRuMP\_F5.raw |  |  |  | 1.7586E7 | 5.092E5 |  |  |  |  |  | 2 | 0 | 0 | 0 | 1 | 1 | 0 | 0 | 0 | 0 | 0 | 202 | 212 | Carbamidomethylation | C3:Carbamidomethylation:1000.00 | PEAKS DB |
| L.GSPATPDLSDTSC(+57.02)AK.T | Y | 56.24 | 1505.6667 | 15 | -0.2 | 753.8405 | 2 | 11.30 | 1 | F1:2177 | DaRuMP\_F1.raw | 1.1256E5 |  |  |  |  |  |  |  |  |  | 1 | 1 | 0 | 0 | 0 | 0 | 0 | 0 | 0 | 0 | 0 | 29 | 43 | Carbamidomethylation | C13:Carbamidomethylation:1000.00 | PEAKS DB |
| Y.C(+57.02)TTTDTFVR.A | N | 56.05 | 1099.4968 | 9 | 0.6 | 550.7560 | 2 | 11.30 | 4 | F4:2168 | DaRuMP\_F5.raw |  |  |  | 9.521E6 |  |  |  |  |  |  | 1 | 0 | 0 | 0 | 1 | 0 | 0 | 0 | 0 | 0 | 0 | 204 | 212 | Carbamidomethylation | C1:Carbamidomethylation:1000.00 | PEAKS DB |
| D.TAC(+57.02)VC(+57.02)VISR.K | N | 55.28 | 1064.5107 | 9 | 0.3 | 533.2628 | 2 | 11.34 | 4 | F4:2206 | DaRuMP\_F5.raw |  |  |  | 3.4147E5 |  |  |  |  |  |  | 1 | 0 | 0 | 0 | 1 | 0 | 0 | 0 | 0 | 0 | 0 | 230 | 238 | Carbamidomethylation | C3:Carbamidomethylation:1000.00;C5:Carbamidomethylation:1000.00 | PEAKS DB |
| S.YC(+57.02)TTTDTFVR.A | N | 53.44 | 1262.5602 | 10 | 0.9 | 632.2879 | 2 | 11.56 | 4 | F4:2432 | DaRuMP\_F5.raw |  |  |  | 1.2365E5 |  |  |  |  |  |  | 1 | 0 | 0 | 0 | 1 | 0 | 0 | 0 | 0 | 0 | 0 | 203 | 212 | Carbamidomethylation | C2:Carbamidomethylation:1000.00 | PEAKS DB |
| G.SPATPDLSDTSC(+57.02)AK.T | Y | 53.32 | 1448.6453 | 14 | -0.4 | 725.3296 | 2 | 11.30 | 1 | F1:2156 | DaRuMP\_F1.raw | 1.5564E5 |  |  |  |  |  |  |  |  |  | 1 | 1 | 0 | 0 | 0 | 0 | 0 | 0 | 0 | 0 | 0 | 30 | 43 | Carbamidomethylation | C12:Carbamidomethylation:1000.00 | PEAKS DB |
| total 9 peptides |
| --- |

P81458|PA2B\_DABRR

back to list

  

| Protein Coverage
| Supporting Peptides
|

Protein Coverage:

Supporting Peptides:

| Peptide | Uniq | -10lgP | Mass | Length | ppm | m/z | z | RT | Fraction | Scan | Source File | Area F1 | Area F10 | Area F2\_3 | Area F5 | Area F6 | Area F7A | Area F7B | Area F8A | Area F8B | Area F9 | #Feature | #Feature F1 | #Feature F10 | #Feature F2\_3 | #Feature F5 | #Feature F6 | #Feature F7A | #Feature F7B | #Feature F8A | #Feature F8B | #Feature F9 | Start | End | PTM | AScore | Found By |
| --- | --- | --- | --- | --- | --- | --- | --- | --- | --- | --- | --- | --- | --- | --- | --- | --- | --- | --- | --- | --- | --- | --- | --- | --- | --- | --- | --- | --- | --- | --- | --- | --- | --- | --- | --- | --- | --- |
| K.NPLSSYSDYGC(+57.02)YC(+57.02)GWGGK.G | Y | 72.94 | 2069.8247 | 18 | 0.8 | 1035.9204 | 2 | 15.95 | 5 | F5:5920 | DaRuMP\_F6.raw |  |  |  |  | 2.7352E6 |  |  |  |  |  | 1 | 0 | 0 | 0 | 0 | 1 | 0 | 0 | 0 | 0 | 0 | 16 | 33 | Carbamidomethylation | C11:Carbamidomethylation:1000.00;C13:Carbamidomethylation:1000.00 | PEAKS DB |
| NLFQFAEM(+15.99)IVK.M | N | 68.34 | 1354.6954 | 11 | 0.7 | 678.3555 | 2 | 39.86 | 7 | F7:20408 | DaRuMP\_F7B.raw |  |  |  |  | 5.4462E5 | 9.9642E5 | 4.936E7 |  | 6.8164E5 |  | 7 | 0 | 0 | 0 | 0 | 1 | 2 | 3 | 0 | 1 | 0 | 1 | 11 | Oxidation (M) | M8:Oxidation (M):1000.00 | PEAKS DB |
| NLFQFAEMIVK.M | N | 68.31 | 1338.7006 | 11 | 0.3 | 670.3577 | 2 | 48.04 | 9 | F9:16002 | DaRuMP\_F8B.raw |  |  |  |  | 2.0517E7 | 1.6603E7 | 2.2778E8 |  | 4.1784E6 |  | 6 | 0 | 0 | 0 | 0 | 1 | 1 | 3 | 0 | 1 | 0 | 1 | 11 |  |  | PEAKS DB |
| Y.GC(+57.02)YC(+57.02)GWGGK.G | N | 64.20 | 1043.3953 | 9 | 3.2 | 522.7053 | 2 | 11.29 | 6 | F6:2116 | DaRuMP\_F7A.raw |  |  | 6.3696E4 |  | 1.7244E6 | 2.9315E6 | 3.3122E5 |  |  | 4.6531E4 | 5 | 0 | 0 | 1 | 0 | 1 | 1 | 1 | 0 | 0 | 1 | 25 | 33 | Carbamidomethylation | C2:Carbamidomethylation:1000.00;C4:Carbamidomethylation:1000.00 | PEAKS DB |
| N.YPPSQC(+57.02)TGTEQC(+57.02) | N | 60.57 | 1426.5493 | 12 | 0.7 | 714.2824 | 2 | 11.09 | 7 | F7:1878 | DaRuMP\_F7B.raw |  |  |  |  |  |  | 2.5518E5 |  |  |  | 1 | 0 | 0 | 0 | 0 | 0 | 0 | 1 | 0 | 0 | 0 | 110 | 121 | Carbamidomethylation | C6:Carbamidomethylation:1000.00;C12:Carbamidomethylation:1000.00 | PEAKS DB |
| R.C(+57.02)C(+57.02)FVHDC(+57.02)C(+57.02)YEK.V | N | 58.26 | 1576.5568 | 11 | 2.8 | 789.2859 | 2 | 11.03 | 6 | F6:1881 | DaRuMP\_F7A.raw |  |  |  |  | 1.9651E7 | 6.2122E5 | 9.491E5 |  |  |  | 6 | 0 | 0 | 0 | 0 | 2 | 2 | 2 | 0 | 0 | 0 | 43 | 53 | Carbamidomethylation | C1:Carbamidomethylation:1000.00;C2:Carbamidomethylation:1000.00;C7:Carbamidomethylation:1000.00;C8:Carbamidomethylation:1000.00 | PEAKS DB |
| D.YGC(+57.02)YC(+57.02)GWGGK.G | N | 58.24 | 1206.4586 | 10 | 0.0 | 604.2366 | 2 | 11.59 | 5 | F5:2425 | DaRuMP\_F6.raw |  |  |  |  | 1.3442E7 |  |  |  |  |  | 1 | 0 | 0 | 0 | 0 | 1 | 0 | 0 | 0 | 0 | 0 | 24 | 33 | Carbamidomethylation | C3:Carbamidomethylation:1000.00;C5:Carbamidomethylation:1000.00 | PEAKS DB |
| S.YSDYGC(+57.02)YC(+57.02)GWGGK.G | N | 56.38 | 1571.5809 | 13 | 1.1 | 786.7986 | 2 | 15.88 | 8 | F8:2644 | DaRuMP\_F8A.raw |  |  |  |  |  |  |  | 1.1806E6 |  |  | 1 | 0 | 0 | 0 | 0 | 0 | 0 | 0 | 1 | 0 | 0 | 21 | 33 | Carbamidomethylation | C6:Carbamidomethylation:1000.00;C8:Carbamidomethylation:1000.00 | PEAKS DB |
| total 8 peptides |
| --- |

B4XSZ0|SLAF\_MACLB

back to list

  

| Protein Coverage
| Supporting Peptides
|

Protein Coverage:

Supporting Peptides:

| Peptide | Uniq | -10lgP | Mass | Length | ppm | m/z | z | RT | Fraction | Scan | Source File | Area F1 | Area F10 | Area F2\_3 | Area F5 | Area F6 | Area F7A | Area F7B | Area F8A | Area F8B | Area F9 | #Feature | #Feature F1 | #Feature F10 | #Feature F2\_3 | #Feature F5 | #Feature F6 | #Feature F7A | #Feature F7B | #Feature F8A | #Feature F8B | #Feature F9 | Start | End | PTM | AScore | Found By |
| --- | --- | --- | --- | --- | --- | --- | --- | --- | --- | --- | --- | --- | --- | --- | --- | --- | --- | --- | --- | --- | --- | --- | --- | --- | --- | --- | --- | --- | --- | --- | --- | --- | --- | --- | --- | --- | --- |
| R.TWFNLSC(+57.02)GDDYPFVC(+57.02)K.S | Y | 98.07 | 2007.8495 | 16 | 0.0 | 1004.9320 | 2 | 36.30 | 8 | F8:16331 | DaRuMP\_F8A.raw |  | 1.2983E6 |  |  |  |  | 1.2734E6 | 1.1805E8 | 9.8789E5 | 2.1342E6 | 6 | 0 | 1 | 0 | 0 | 0 | 0 | 1 | 2 | 1 | 1 | 138 | 153 | Carbamidomethylation | C7:Carbamidomethylation:1000.00;C15:Carbamidomethylation:1000.00 | PEAKS DB |
| F.NLSC(+57.02)GDDYPFVC(+57.02)K.S | Y | 76.02 | 1573.6541 | 13 | 1.3 | 787.8353 | 2 | 16.41 | 8 | F8:3124 | DaRuMP\_F8A.raw |  |  |  |  |  |  |  | 4.737E6 |  |  | 1 | 0 | 0 | 0 | 0 | 0 | 0 | 0 | 1 | 0 | 0 | 141 | 153 | Carbamidomethylation | C4:Carbamidomethylation:1000.00;C12:Carbamidomethylation:1000.00 | PEAKS DB |
| A.EFVAQLISENIK.T | Y | 59.98 | 1389.7504 | 12 | 0.2 | 695.8826 | 2 | 26.60 | 8 | F8:10279 | DaRuMP\_F8A.raw |  |  |  |  |  |  |  | 7.778E4 |  |  | 1 | 0 | 0 | 0 | 0 | 0 | 0 | 0 | 1 | 0 | 0 | 74 | 85 |  |  | PEAKS DB |
| H.LVSIESVEEAEFVAQLISENIK.T | Y | 58.74 | 2446.2842 | 22 | 0.9 | 816.4360 | 3 | 68.75 | 8 | F8:28574 | DaRuMP\_F8A.raw |  |  |  |  |  |  |  | 2.3773E6 |  |  | 1 | 0 | 0 | 0 | 0 | 0 | 0 | 0 | 1 | 0 | 0 | 64 | 85 |  |  | PEAKS DB |
| E.SVEEAEFVAQLISENIK.T | Y | 52.91 | 1904.9730 | 17 | 0.2 | 635.9984 | 3 | 50.06 | 8 | F8:22350 | DaRuMP\_F8A.raw |  |  |  |  |  |  |  | 8.4961E4 |  |  | 1 | 0 | 0 | 0 | 0 | 0 | 0 | 0 | 1 | 0 | 0 | 69 | 85 |  |  | PEAKS DB |
| total 5 peptides |
| --- |

B4XSY9|SLAE\_MACLB

back to list

  

| Protein Coverage
| Supporting Peptides
|

Protein Coverage:

Supporting Peptides:

| Peptide | Uniq | -10lgP | Mass | Length | ppm | m/z | z | RT | Fraction | Scan | Source File | Area F1 | Area F10 | Area F2\_3 | Area F5 | Area F6 | Area F7A | Area F7B | Area F8A | Area F8B | Area F9 | #Feature | #Feature F1 | #Feature F10 | #Feature F2\_3 | #Feature F5 | #Feature F6 | #Feature F7A | #Feature F7B | #Feature F8A | #Feature F8B | #Feature F9 | Start | End | PTM | AScore | Found By |
| --- | --- | --- | --- | --- | --- | --- | --- | --- | --- | --- | --- | --- | --- | --- | --- | --- | --- | --- | --- | --- | --- | --- | --- | --- | --- | --- | --- | --- | --- | --- | --- | --- | --- | --- | --- | --- | --- |
| R.TWFNLSC(+57.02)GDDYPFVC(+57.02)K.S | Y | 98.07 | 2007.8495 | 16 | 0.0 | 1004.9320 | 2 | 36.30 | 8 | F8:16331 | DaRuMP\_F8A.raw |  | 1.2983E6 |  |  |  |  | 1.2734E6 | 1.1805E8 | 9.8789E5 | 2.1342E6 | 6 | 0 | 1 | 0 | 0 | 0 | 0 | 1 | 2 | 1 | 1 | 138 | 153 | Carbamidomethylation | C7:Carbamidomethylation:1000.00;C15:Carbamidomethylation:1000.00 | PEAKS DB |
| F.NLSC(+57.02)GDDYPFVC(+57.02)K.S | Y | 76.02 | 1573.6541 | 13 | 1.3 | 787.8353 | 2 | 16.41 | 8 | F8:3124 | DaRuMP\_F8A.raw |  |  |  |  |  |  |  | 4.737E6 |  |  | 1 | 0 | 0 | 0 | 0 | 0 | 0 | 0 | 1 | 0 | 0 | 141 | 153 | Carbamidomethylation | C4:Carbamidomethylation:1000.00;C12:Carbamidomethylation:1000.00 | PEAKS DB |
| A.EFVAQLISENIK.T | Y | 59.98 | 1389.7504 | 12 | 0.2 | 695.8826 | 2 | 26.60 | 8 | F8:10279 | DaRuMP\_F8A.raw |  |  |  |  |  |  |  | 7.778E4 |  |  | 1 | 0 | 0 | 0 | 0 | 0 | 0 | 0 | 1 | 0 | 0 | 74 | 85 |  |  | PEAKS DB |
| H.LVSIESVEEAEFVAQLISENIK.T | Y | 58.74 | 2446.2842 | 22 | 0.9 | 816.4360 | 3 | 68.75 | 8 | F8:28574 | DaRuMP\_F8A.raw |  |  |  |  |  |  |  | 2.3773E6 |  |  | 1 | 0 | 0 | 0 | 0 | 0 | 0 | 0 | 1 | 0 | 0 | 64 | 85 |  |  | PEAKS DB |
| E.SVEEAEFVAQLISENIK.T | Y | 52.91 | 1904.9730 | 17 | 0.2 | 635.9984 | 3 | 50.06 | 8 | F8:22350 | DaRuMP\_F8A.raw |  |  |  |  |  |  |  | 8.4961E4 |  |  | 1 | 0 | 0 | 0 | 0 | 0 | 0 | 0 | 1 | 0 | 0 | 69 | 85 |  |  | PEAKS DB |
| total 5 peptides |
| --- |

W5XCJ6|SLCIB\_MACLB

back to list

  

| Protein Coverage
| Supporting Peptides
|

Protein Coverage:

Supporting Peptides:

| Peptide | Uniq | -10lgP | Mass | Length | ppm | m/z | z | RT | Fraction | Scan | Source File | Area F1 | Area F10 | Area F2\_3 | Area F5 | Area F6 | Area F7A | Area F7B | Area F8A | Area F8B | Area F9 | #Feature | #Feature F1 | #Feature F10 | #Feature F2\_3 | #Feature F5 | #Feature F6 | #Feature F7A | #Feature F7B | #Feature F8A | #Feature F8B | #Feature F9 | Start | End | PTM | AScore | Found By |
| --- | --- | --- | --- | --- | --- | --- | --- | --- | --- | --- | --- | --- | --- | --- | --- | --- | --- | --- | --- | --- | --- | --- | --- | --- | --- | --- | --- | --- | --- | --- | --- | --- | --- | --- | --- | --- | --- |
| R.TWFNLSC(+57.02)GDDYPFVC(+57.02)K.S | Y | 98.07 | 2007.8495 | 16 | 0.0 | 1004.9320 | 2 | 36.30 | 8 | F8:16331 | DaRuMP\_F8A.raw |  | 1.2983E6 |  |  |  |  | 1.2734E6 | 1.1805E8 | 9.8789E5 | 2.1342E6 | 6 | 0 | 1 | 0 | 0 | 0 | 0 | 1 | 2 | 1 | 1 | 136 | 151 | Carbamidomethylation | C7:Carbamidomethylation:1000.00;C15:Carbamidomethylation:1000.00 | PEAKS DB |
| F.NLSC(+57.02)GDDYPFVC(+57.02)K.S | Y | 76.02 | 1573.6541 | 13 | 1.3 | 787.8353 | 2 | 16.41 | 8 | F8:3124 | DaRuMP\_F8A.raw |  |  |  |  |  |  |  | 4.737E6 |  |  | 1 | 0 | 0 | 0 | 0 | 0 | 0 | 0 | 1 | 0 | 0 | 139 | 151 | Carbamidomethylation | C4:Carbamidomethylation:1000.00;C12:Carbamidomethylation:1000.00 | PEAKS DB |
| A.EFVAQLISENIK.T | Y | 59.98 | 1389.7504 | 12 | 0.2 | 695.8826 | 2 | 26.60 | 8 | F8:10279 | DaRuMP\_F8A.raw |  |  |  |  |  |  |  | 7.778E4 |  |  | 1 | 0 | 0 | 0 | 0 | 0 | 0 | 0 | 1 | 0 | 0 | 72 | 83 |  |  | PEAKS DB |
| H.LVSIESVEEAEFVAQLISENIK.T | Y | 58.74 | 2446.2842 | 22 | 0.9 | 816.4360 | 3 | 68.75 | 8 | F8:28574 | DaRuMP\_F8A.raw |  |  |  |  |  |  |  | 2.3773E6 |  |  | 1 | 0 | 0 | 0 | 0 | 0 | 0 | 0 | 1 | 0 | 0 | 62 | 83 |  |  | PEAKS DB |
| E.SVEEAEFVAQLISENIK.T | Y | 52.91 | 1904.9730 | 17 | 0.2 | 635.9984 | 3 | 50.06 | 8 | F8:22350 | DaRuMP\_F8A.raw |  |  |  |  |  |  |  | 8.4961E4 |  |  | 1 | 0 | 0 | 0 | 0 | 0 | 0 | 0 | 1 | 0 | 0 | 67 | 83 |  |  | PEAKS DB |
| total 5 peptides |
| --- |

Q2ES50|VKT1\_DABRR

back to list

  

| Protein Coverage
| Supporting Peptides
|

Protein Coverage:

Supporting Peptides:

| Peptide | Uniq | -10lgP | Mass | Length | ppm | m/z | z | RT | Fraction | Scan | Source File | Area F1 | Area F10 | Area F2\_3 | Area F5 | Area F6 | Area F7A | Area F7B | Area F8A | Area F8B | Area F9 | #Feature | #Feature F1 | #Feature F10 | #Feature F2\_3 | #Feature F5 | #Feature F6 | #Feature F7A | #Feature F7B | #Feature F8A | #Feature F8B | #Feature F9 | Start | End | PTM | AScore | Found By |
| --- | --- | --- | --- | --- | --- | --- | --- | --- | --- | --- | --- | --- | --- | --- | --- | --- | --- | --- | --- | --- | --- | --- | --- | --- | --- | --- | --- | --- | --- | --- | --- | --- | --- | --- | --- | --- | --- |
| K.VFFYGGC(+57.02)GGNANNFETR.D | Y | 87.60 | 1908.8213 | 17 | -0.6 | 955.4174 | 2 | 13.89 | 3 | F3:3802 | DaRuMP\_F2\_F3.raw |  |  | 6.0872E4 | 2.9768E5 |  |  |  |  |  |  | 2 | 0 | 0 | 1 | 1 | 0 | 0 | 0 | 0 | 0 | 0 | 58 | 74 | Carbamidomethylation | C7:Carbamidomethylation:1000.00 | PEAKS DB |
| G.HDRPTFC(+57.02)NLAPESGR.C | N | 78.00 | 1755.8110 | 15 | -1.2 | 586.2769 | 3 | 11.26 | 1 | F1:2140 | DaRuMP\_F1.raw | 2.2348E6 |  |  |  |  |  |  |  |  |  | 1 | 1 | 0 | 0 | 0 | 0 | 0 | 0 | 0 | 0 | 0 | 25 | 39 | Carbamidomethylation | C7:Carbamidomethylation:1000.00 | PEAKS DB |
| T.FC(+57.02)NLAPESGR.C | N | 68.04 | 1149.5237 | 10 | 0.9 | 575.7697 | 2 | 11.40 | 1 | F1:2252 | DaRuMP\_F1.raw | 2.7845E6 |  |  |  |  |  |  |  |  | 8.9627E4 | 2 | 1 | 0 | 0 | 0 | 0 | 0 | 0 | 0 | 0 | 1 | 30 | 39 | Carbamidomethylation | C2:Carbamidomethylation:1000.00 | PEAKS DB |
| R.IYYNLESNK.C | N | 59.31 | 1142.5608 | 9 | 0.0 | 572.2877 | 2 | 11.61 | 1 | F1:2461 | DaRuMP\_F1.raw | 2.8939E6 |  |  |  |  |  |  |  |  | 1.7171E5 | 2 | 1 | 0 | 0 | 0 | 0 | 0 | 0 | 0 | 0 | 1 | 47 | 55 |  |  | PEAKS DB |
| R.RIYYNLESNK.C | N | 56.80 | 1298.6619 | 10 | 1.0 | 650.3389 | 2 | 11.23 | 1 | F1:2102 | DaRuMP\_F1.raw | 1.7193E5 |  |  |  |  |  |  |  |  |  | 1 | 1 | 0 | 0 | 0 | 0 | 0 | 0 | 0 | 0 | 0 | 46 | 55 |  |  | PEAKS DB |
| Y.GGC(+57.02)GGNANNFETR.D | N | 55.54 | 1352.5527 | 13 | 0.7 | 677.2841 | 2 | 10.77 | 3 | F3:1663 | DaRuMP\_F2\_F3.raw |  |  | 3.6976E4 |  |  |  |  |  |  |  | 1 | 0 | 0 | 1 | 0 | 0 | 0 | 0 | 0 | 0 | 0 | 62 | 74 | Carbamidomethylation | C3:Carbamidomethylation:1000.00 | PEAKS DB |
| total 6 peptides |
| --- |

A8Y7P0|VKTB7\_DABSI

back to list

  

| Protein Coverage
| Supporting Peptides
|

Protein Coverage:

Supporting Peptides:

| Peptide | Uniq | -10lgP | Mass | Length | ppm | m/z | z | RT | Fraction | Scan | Source File | Area F1 | Area F10 | Area F2\_3 | Area F5 | Area F6 | Area F7A | Area F7B | Area F8A | Area F8B | Area F9 | #Feature | #Feature F1 | #Feature F10 | #Feature F2\_3 | #Feature F5 | #Feature F6 | #Feature F7A | #Feature F7B | #Feature F8A | #Feature F8B | #Feature F9 | Start | End | PTM | AScore | Found By |
| --- | --- | --- | --- | --- | --- | --- | --- | --- | --- | --- | --- | --- | --- | --- | --- | --- | --- | --- | --- | --- | --- | --- | --- | --- | --- | --- | --- | --- | --- | --- | --- | --- | --- | --- | --- | --- | --- |
| K.VFFYGGC(+57.02)GGNANNFETR.D | Y | 87.60 | 1908.8213 | 17 | -0.6 | 955.4174 | 2 | 13.89 | 3 | F3:3802 | DaRuMP\_F2\_F3.raw |  |  | 6.0872E4 | 2.9768E5 |  |  |  |  |  |  | 2 | 0 | 0 | 1 | 1 | 0 | 0 | 0 | 0 | 0 | 0 | 58 | 74 | Carbamidomethylation | C7:Carbamidomethylation:1000.00 | PEAKS DB |
| G.HDRPTFC(+57.02)NLAPESGR.C | N | 78.00 | 1755.8110 | 15 | -1.2 | 586.2769 | 3 | 11.26 | 1 | F1:2140 | DaRuMP\_F1.raw | 2.2348E6 |  |  |  |  |  |  |  |  |  | 1 | 1 | 0 | 0 | 0 | 0 | 0 | 0 | 0 | 0 | 0 | 25 | 39 | Carbamidomethylation | C7:Carbamidomethylation:1000.00 | PEAKS DB |
| T.FC(+57.02)NLAPESGR.C | N | 68.04 | 1149.5237 | 10 | 0.9 | 575.7697 | 2 | 11.40 | 1 | F1:2252 | DaRuMP\_F1.raw | 2.7845E6 |  |  |  |  |  |  |  |  | 8.9627E4 | 2 | 1 | 0 | 0 | 0 | 0 | 0 | 0 | 0 | 0 | 1 | 30 | 39 | Carbamidomethylation | C2:Carbamidomethylation:1000.00 | PEAKS DB |
| R.IYYNLESNK.C | N | 59.31 | 1142.5608 | 9 | 0.0 | 572.2877 | 2 | 11.61 | 1 | F1:2461 | DaRuMP\_F1.raw | 2.8939E6 |  |  |  |  |  |  |  |  | 1.7171E5 | 2 | 1 | 0 | 0 | 0 | 0 | 0 | 0 | 0 | 0 | 1 | 47 | 55 |  |  | PEAKS DB |
| R.RIYYNLESNK.C | N | 56.80 | 1298.6619 | 10 | 1.0 | 650.3389 | 2 | 11.23 | 1 | F1:2102 | DaRuMP\_F1.raw | 1.7193E5 |  |  |  |  |  |  |  |  |  | 1 | 1 | 0 | 0 | 0 | 0 | 0 | 0 | 0 | 0 | 0 | 46 | 55 |  |  | PEAKS DB |
| Y.GGC(+57.02)GGNANNFETR.D | N | 55.54 | 1352.5527 | 13 | 0.7 | 677.2841 | 2 | 10.77 | 3 | F3:1663 | DaRuMP\_F2\_F3.raw |  |  | 3.6976E4 |  |  |  |  |  |  |  | 1 | 0 | 0 | 1 | 0 | 0 | 0 | 0 | 0 | 0 | 0 | 62 | 74 | Carbamidomethylation | C3:Carbamidomethylation:1000.00 | PEAKS DB |
| total 6 peptides |
| --- |

P00990|VKT2\_DABSI

back to list

  

| Protein Coverage
| Supporting Peptides
|

Protein Coverage:

Supporting Peptides:

| Peptide | Uniq | -10lgP | Mass | Length | ppm | m/z | z | RT | Fraction | Scan | Source File | Area F1 | Area F10 | Area F2\_3 | Area F5 | Area F6 | Area F7A | Area F7B | Area F8A | Area F8B | Area F9 | #Feature | #Feature F1 | #Feature F10 | #Feature F2\_3 | #Feature F5 | #Feature F6 | #Feature F7A | #Feature F7B | #Feature F8A | #Feature F8B | #Feature F9 | Start | End | PTM | AScore | Found By |
| --- | --- | --- | --- | --- | --- | --- | --- | --- | --- | --- | --- | --- | --- | --- | --- | --- | --- | --- | --- | --- | --- | --- | --- | --- | --- | --- | --- | --- | --- | --- | --- | --- | --- | --- | --- | --- | --- |
| K.VFFYGGC(+57.02)GGNANNFETR.D | Y | 87.60 | 1908.8213 | 17 | -0.6 | 955.4174 | 2 | 13.89 | 3 | F3:3802 | DaRuMP\_F2\_F3.raw |  |  | 6.0872E4 | 2.9768E5 |  |  |  |  |  |  | 2 | 0 | 0 | 1 | 1 | 0 | 0 | 0 | 0 | 0 | 0 | 34 | 50 | Carbamidomethylation | C7:Carbamidomethylation:1000.00 | PEAKS DB |
| HDRPTFC(+57.02)NLAPESGR.C | N | 78.00 | 1755.8110 | 15 | -1.2 | 586.2769 | 3 | 11.26 | 1 | F1:2140 | DaRuMP\_F1.raw | 2.2348E6 |  |  |  |  |  |  |  |  |  | 1 | 1 | 0 | 0 | 0 | 0 | 0 | 0 | 0 | 0 | 0 | 1 | 15 | Carbamidomethylation | C7:Carbamidomethylation:1000.00 | PEAKS DB |
| T.FC(+57.02)NLAPESGR.C | N | 68.04 | 1149.5237 | 10 | 0.9 | 575.7697 | 2 | 11.40 | 1 | F1:2252 | DaRuMP\_F1.raw | 2.7845E6 |  |  |  |  |  |  |  |  | 8.9627E4 | 2 | 1 | 0 | 0 | 0 | 0 | 0 | 0 | 0 | 0 | 1 | 6 | 15 | Carbamidomethylation | C2:Carbamidomethylation:1000.00 | PEAKS DB |
| R.IYYNLESNK.C | N | 59.31 | 1142.5608 | 9 | 0.0 | 572.2877 | 2 | 11.61 | 1 | F1:2461 | DaRuMP\_F1.raw | 2.8939E6 |  |  |  |  |  |  |  |  | 1.7171E5 | 2 | 1 | 0 | 0 | 0 | 0 | 0 | 0 | 0 | 0 | 1 | 23 | 31 |  |  | PEAKS DB |
| R.RIYYNLESNK.C | N | 56.80 | 1298.6619 | 10 | 1.0 | 650.3389 | 2 | 11.23 | 1 | F1:2102 | DaRuMP\_F1.raw | 1.7193E5 |  |  |  |  |  |  |  |  |  | 1 | 1 | 0 | 0 | 0 | 0 | 0 | 0 | 0 | 0 | 0 | 22 | 31 |  |  | PEAKS DB |
| Y.GGC(+57.02)GGNANNFETR.D | N | 55.54 | 1352.5527 | 13 | 0.7 | 677.2841 | 2 | 10.77 | 3 | F3:1663 | DaRuMP\_F2\_F3.raw |  |  | 3.6976E4 |  |  |  |  |  |  |  | 1 | 0 | 0 | 1 | 0 | 0 | 0 | 0 | 0 | 0 | 0 | 38 | 50 | Carbamidomethylation | C3:Carbamidomethylation:1000.00 | PEAKS DB |
| total 6 peptides |
| --- |

P13647|K2C5\_HUMAN

back to list

  

| Protein Coverage
| Supporting Peptides
|

Protein Coverage:

Supporting Peptides:

| Peptide | Uniq | -10lgP | Mass | Length | ppm | m/z | z | RT | Fraction | Scan | Source File | Area F1 | Area F10 | Area F2\_3 | Area F5 | Area F6 | Area F7A | Area F7B | Area F8A | Area F8B | Area F9 | #Feature | #Feature F1 | #Feature F10 | #Feature F2\_3 | #Feature F5 | #Feature F6 | #Feature F7A | #Feature F7B | #Feature F8A | #Feature F8B | #Feature F9 | Start | End | PTM | AScore | Found By |
| --- | --- | --- | --- | --- | --- | --- | --- | --- | --- | --- | --- | --- | --- | --- | --- | --- | --- | --- | --- | --- | --- | --- | --- | --- | --- | --- | --- | --- | --- | --- | --- | --- | --- | --- | --- | --- | --- |
| R.NLDLDSIIAEVK.A | N | 65.38 | 1328.7188 | 12 | 3.0 | 665.3670 | 2 | 37.00 | 6 | F6:19466 | DaRuMP\_F7A.raw |  | 5.6789E5 |  |  | 1.9663E6 | 5.3878E4 | 2.6962E6 | 1.693E6 | 9.4573E5 | 7.5449E5 | 7 | 0 | 1 | 0 | 0 | 1 | 1 | 1 | 1 | 1 | 1 | 332 | 343 |  |  | PEAKS DB |
| K.LALDVEIATYR.K | N | 62.47 | 1262.6870 | 11 | 0.1 | 632.3508 | 2 | 19.53 | 5 | F5:9106 | DaRuMP\_F6.raw |  |  |  | 1.0143E5 | 2.8248E5 |  | 3.5938E5 |  | 1.8812E5 | 2.4586E5 | 5 | 0 | 0 | 0 | 1 | 1 | 0 | 1 | 0 | 1 | 1 | 461 | 471 |  |  | PEAKS DB |
| R.QNLEPLFEQYINNLR.R | N | 62.43 | 1889.9635 | 15 | 0.4 | 630.9954 | 3 | 43.50 | 9 | F9:14713 | DaRuMP\_F8B.raw |  | 7.2503E4 |  |  |  |  | 1.0471E6 |  | 1.9509E5 |  | 5 | 0 | 1 | 0 | 0 | 0 | 0 | 2 | 0 | 2 | 0 | 213 | 227 |  |  | PEAKS DB |
| R.QLDSIVGER.G | N | 60.84 | 1015.5298 | 9 | 1.2 | 508.7728 | 2 | 11.66 | 7 | F7:2483 | DaRuMP\_F7B.raw |  |  |  |  |  |  | 3.5612E4 |  |  |  | 1 | 0 | 0 | 0 | 0 | 0 | 0 | 1 | 0 | 0 | 0 | 229 | 237 |  |  | PEAKS DB |
| K.YEELQQTAGR.H | N | 60.21 | 1193.5676 | 10 | 0.3 | 597.7913 | 2 | 11.02 | 4 | F4:1911 | DaRuMP\_F5.raw |  |  |  | 9.2458E3 |  |  |  |  |  |  | 1 | 0 | 0 | 0 | 1 | 0 | 0 | 0 | 0 | 0 | 0 | 365 | 374 |  |  | PEAKS DB |
| R.VSLAGAC(+57.02)GVGGYGSR.S | Y | 59.29 | 1409.6721 | 15 | -0.9 | 705.8427 | 2 | 11.74 | 7 | F7:2569 | DaRuMP\_F7B.raw |  |  |  |  |  |  | 4.8722E4 |  |  |  | 1 | 0 | 0 | 0 | 0 | 0 | 0 | 1 | 0 | 0 | 0 | 49 | 63 | Carbamidomethylation | C7:Carbamidomethylation:1000.00 | PEAKS DB |
| K.NKYEDEINKR.T | N | 56.45 | 1307.6470 | 10 | 0.1 | 436.8896 | 3 | 10.71 | 10 | F10:1603 | DaRuMP\_F9.raw |  |  |  |  |  |  |  |  | 2.9837E5 | 1.8812E5 | 2 | 0 | 0 | 0 | 0 | 0 | 0 | 0 | 0 | 1 | 1 | 256 | 265 |  |  | PEAKS DB |
| R.TTAENEFVM(+15.99)LK.K | N | 56.02 | 1297.6224 | 11 | 0.5 | 649.8188 | 2 | 11.97 | 10 | F10:2725 | DaRuMP\_F9.raw |  |  |  |  |  |  |  |  |  | 1.4733E4 | 1 | 0 | 0 | 0 | 0 | 0 | 0 | 0 | 0 | 0 | 1 | 266 | 276 | Oxidation (M) | M9:Oxidation (M):1000.00 | PEAKS DB |
| K.LAELEEALQK.A | Y | 54.42 | 1142.6183 | 10 | -1.1 | 572.3158 | 2 | 12.17 | 1 | F1:2998 | DaRuMP\_F1.raw | 1.0191E5 |  |  |  | 9.2326E4 |  |  |  |  |  | 2 | 1 | 0 | 0 | 0 | 1 | 0 | 0 | 0 | 0 | 0 | 432 | 441 |  |  | PEAKS DB |
| R.ISISTSGGSFR.N | Y | 52.13 | 1110.5669 | 11 | 1.0 | 556.2913 | 2 | 11.70 | 4 | F4:2569 | DaRuMP\_F5.raw |  |  |  | 8.911E4 |  |  |  |  |  |  | 1 | 0 | 0 | 0 | 1 | 0 | 0 | 0 | 0 | 0 | 0 | 74 | 84 |  |  | PEAKS DB |
| total 10 peptides |
| --- |

P15445|PA2A2\_NAJNA

back to list

  

| Protein Coverage
| Supporting Peptides
|

Protein Coverage:

Supporting Peptides:

| Peptide | Uniq | -10lgP | Mass | Length | ppm | m/z | z | RT | Fraction | Scan | Source File | Area F1 | Area F10 | Area F2\_3 | Area F5 | Area F6 | Area F7A | Area F7B | Area F8A | Area F8B | Area F9 | #Feature | #Feature F1 | #Feature F10 | #Feature F2\_3 | #Feature F5 | #Feature F6 | #Feature F7A | #Feature F7B | #Feature F8A | #Feature F8B | #Feature F9 | Start | End | PTM | AScore | Found By |
| --- | --- | --- | --- | --- | --- | --- | --- | --- | --- | --- | --- | --- | --- | --- | --- | --- | --- | --- | --- | --- | --- | --- | --- | --- | --- | --- | --- | --- | --- | --- | --- | --- | --- | --- | --- | --- | --- |
| K.TYSYEC(+57.02)SQGTLTC(+57.02)K.G | Y | 86.56 | 1696.7073 | 14 | 0.3 | 849.3612 | 2 | 11.40 | 5 | F5:2250 | DaRuMP\_F6.raw |  |  |  | 4.687E5 | 9.4585E5 |  |  |  |  |  | 2 | 0 | 0 | 0 | 1 | 1 | 0 | 0 | 0 | 0 | 0 | 66 | 79 | Carbamidomethylation | C6:Carbamidomethylation:1000.00;C13:Carbamidomethylation:1000.00 | PEAKS DB |
| F.AGAPYNDNNYNIDLK.A | Y | 74.12 | 1680.7743 | 15 | -2.4 | 841.3924 | 2 | 11.97 | 5 | F5:2815 | DaRuMP\_F6.raw |  |  |  | 3.0112E5 | 5.6499E5 |  |  |  |  |  | 2 | 0 | 0 | 0 | 1 | 1 | 0 | 0 | 0 | 0 | 0 | 101 | 115 |  |  | PEAKS DB |
| R.GGSGTPVDDLDR.C | Y | 68.68 | 1187.5417 | 12 | -0.5 | 594.7778 | 2 | 11.43 | 4 | F4:2292 | DaRuMP\_F5.raw |  |  |  | 4.4243E5 | 4.9437E5 |  |  |  |  |  | 2 | 0 | 0 | 0 | 1 | 1 | 0 | 0 | 0 | 0 | 0 | 31 | 42 |  |  | PEAKS DB |
| K.GDNNAC(+57.02)AASVC(+57.02)DC(+57.02)DR.L | Y | 65.45 | 1683.6035 | 15 | 0.7 | 842.8096 | 2 | 10.83 | 5 | F5:1714 | DaRuMP\_F6.raw |  |  |  |  | 1.4455E5 |  |  |  |  |  | 1 | 0 | 0 | 0 | 0 | 1 | 0 | 0 | 0 | 0 | 0 | 80 | 94 | Carbamidomethylation | C6:Carbamidomethylation:1000.00;C11:Carbamidomethylation:1000.00;C13:Carbamidomethylation:1000.00 | PEAKS DB |
| total 4 peptides |
| --- |

A5A6M5|K1H1\_PANTR

back to list

  

| Protein Coverage
| Supporting Peptides
|

Protein Coverage:

Supporting Peptides:

| Peptide | Uniq | -10lgP | Mass | Length | ppm | m/z | z | RT | Fraction | Scan | Source File | Area F1 | Area F10 | Area F2\_3 | Area F5 | Area F6 | Area F7A | Area F7B | Area F8A | Area F8B | Area F9 | #Feature | #Feature F1 | #Feature F10 | #Feature F2\_3 | #Feature F5 | #Feature F6 | #Feature F7A | #Feature F7B | #Feature F8A | #Feature F8B | #Feature F9 | Start | End | PTM | AScore | Found By |
| --- | --- | --- | --- | --- | --- | --- | --- | --- | --- | --- | --- | --- | --- | --- | --- | --- | --- | --- | --- | --- | --- | --- | --- | --- | --- | --- | --- | --- | --- | --- | --- | --- | --- | --- | --- | --- | --- |
| R.DSLENTLTESEAR.Y | N | 74.90 | 1463.6740 | 13 | 0.2 | 732.8444 | 2 | 12.07 | 3 | F3:2860 | DaRuMP\_F2\_F3.raw |  |  | 4.2814E4 |  |  |  |  |  |  |  | 1 | 0 | 0 | 1 | 0 | 0 | 0 | 0 | 0 | 0 | 0 | 294 | 306 |  |  | PEAKS DB |
| R.SQYEALVETNR.R | N | 72.60 | 1308.6310 | 11 | 0.4 | 655.3231 | 2 | 11.60 | 3 | F3:2410 | DaRuMP\_F2\_F3.raw |  |  | 0 |  |  |  |  |  |  |  | 0 | 0 | 0 | 0 | 0 | 0 | 0 | 0 | 0 | 0 | 0 | 230 | 240 |  |  | PEAKS DB |
| R.LEC(+57.02)EINTYR.S | N | 69.77 | 1196.5496 | 9 | 0.5 | 599.2823 | 2 | 11.41 | 3 | F3:2220 | DaRuMP\_F2\_F3.raw |  |  | 1.783E5 |  |  |  |  |  |  |  | 1 | 0 | 0 | 1 | 0 | 0 | 0 | 0 | 0 | 0 | 0 | 349 | 357 | Carbamidomethylation | C3:Carbamidomethylation:1000.00 | PEAKS DB |
| R.C(+57.02)GPC(+57.02)NSFVR | Y | 59.15 | 1095.4590 | 9 | 0.2 | 548.7369 | 2 | 11.27 | 3 | F3:2097 | DaRuMP\_F2\_F3.raw |  |  | 8.3082E3 |  |  |  |  |  |  |  | 1 | 0 | 0 | 1 | 0 | 0 | 0 | 0 | 0 | 0 | 0 | 408 | 416 | Carbamidomethylation | C1:Carbamidomethylation:1000.00;C4:Carbamidomethylation:1000.00 | PEAKS DB |
| R.DNAELENLIR.E | N | 57.77 | 1185.5989 | 10 | -0.3 | 593.8065 | 2 | 16.94 | 3 | F3:4566 | DaRuMP\_F2\_F3.raw |  |  | 2.6528E5 |  |  |  |  |  |  |  | 1 | 0 | 0 | 1 | 0 | 0 | 0 | 0 | 0 | 0 | 0 | 80 | 89 |  |  | PEAKS DB |
| R.QNQEYQVLLDVR.A | N | 52.92 | 1503.7681 | 12 | 0.7 | 752.8918 | 2 | 16.16 | 3 | F3:4367 | DaRuMP\_F2\_F3.raw |  |  | 2.3256E5 |  |  |  |  |  |  |  | 1 | 0 | 0 | 1 | 0 | 0 | 0 | 0 | 0 | 0 | 0 | 335 | 346 |  |  | PEAKS DB |
| total 6 peptides |
| --- |

Q15323|K1H1\_HUMAN

back to list

  

| Protein Coverage
| Supporting Peptides
|

Protein Coverage:

Supporting Peptides:

| Peptide | Uniq | -10lgP | Mass | Length | ppm | m/z | z | RT | Fraction | Scan | Source File | Area F1 | Area F10 | Area F2\_3 | Area F5 | Area F6 | Area F7A | Area F7B | Area F8A | Area F8B | Area F9 | #Feature | #Feature F1 | #Feature F10 | #Feature F2\_3 | #Feature F5 | #Feature F6 | #Feature F7A | #Feature F7B | #Feature F8A | #Feature F8B | #Feature F9 | Start | End | PTM | AScore | Found By |
| --- | --- | --- | --- | --- | --- | --- | --- | --- | --- | --- | --- | --- | --- | --- | --- | --- | --- | --- | --- | --- | --- | --- | --- | --- | --- | --- | --- | --- | --- | --- | --- | --- | --- | --- | --- | --- | --- |
| R.DSLENTLTESEAR.Y | N | 74.90 | 1463.6740 | 13 | 0.2 | 732.8444 | 2 | 12.07 | 3 | F3:2860 | DaRuMP\_F2\_F3.raw |  |  | 4.2814E4 |  |  |  |  |  |  |  | 1 | 0 | 0 | 1 | 0 | 0 | 0 | 0 | 0 | 0 | 0 | 294 | 306 |  |  | PEAKS DB |
| R.SQYEALVETNR.R | N | 72.60 | 1308.6310 | 11 | 0.4 | 655.3231 | 2 | 11.60 | 3 | F3:2410 | DaRuMP\_F2\_F3.raw |  |  | 0 |  |  |  |  |  |  |  | 0 | 0 | 0 | 0 | 0 | 0 | 0 | 0 | 0 | 0 | 0 | 230 | 240 |  |  | PEAKS DB |
| R.LEC(+57.02)EINTYR.S | N | 69.77 | 1196.5496 | 9 | 0.5 | 599.2823 | 2 | 11.41 | 3 | F3:2220 | DaRuMP\_F2\_F3.raw |  |  | 1.783E5 |  |  |  |  |  |  |  | 1 | 0 | 0 | 1 | 0 | 0 | 0 | 0 | 0 | 0 | 0 | 349 | 357 | Carbamidomethylation | C3:Carbamidomethylation:1000.00 | PEAKS DB |
| R.C(+57.02)GPC(+57.02)NSFVR | Y | 59.15 | 1095.4590 | 9 | 0.2 | 548.7369 | 2 | 11.27 | 3 | F3:2097 | DaRuMP\_F2\_F3.raw |  |  | 8.3082E3 |  |  |  |  |  |  |  | 1 | 0 | 0 | 1 | 0 | 0 | 0 | 0 | 0 | 0 | 0 | 408 | 416 | Carbamidomethylation | C1:Carbamidomethylation:1000.00;C4:Carbamidomethylation:1000.00 | PEAKS DB |
| R.DNAELENLIR.E | N | 57.77 | 1185.5989 | 10 | -0.3 | 593.8065 | 2 | 16.94 | 3 | F3:4566 | DaRuMP\_F2\_F3.raw |  |  | 2.6528E5 |  |  |  |  |  |  |  | 1 | 0 | 0 | 1 | 0 | 0 | 0 | 0 | 0 | 0 | 0 | 80 | 89 |  |  | PEAKS DB |
| R.QNQEYQVLLDVR.A | N | 52.92 | 1503.7681 | 12 | 0.7 | 752.8918 | 2 | 16.16 | 3 | F3:4367 | DaRuMP\_F2\_F3.raw |  |  | 2.3256E5 |  |  |  |  |  |  |  | 1 | 0 | 0 | 1 | 0 | 0 | 0 | 0 | 0 | 0 | 0 | 335 | 346 |  |  | PEAKS DB |
| total 6 peptides |
| --- |

Q8JFG1|PA2H\_VIPAP

back to list

  

| Protein Coverage
| Supporting Peptides
|

Protein Coverage:

Supporting Peptides:

| Peptide | Uniq | -10lgP | Mass | Length | ppm | m/z | z | RT | Fraction | Scan | Source File | Area F1 | Area F10 | Area F2\_3 | Area F5 | Area F6 | Area F7A | Area F7B | Area F8A | Area F8B | Area F9 | #Feature | #Feature F1 | #Feature F10 | #Feature F2\_3 | #Feature F5 | #Feature F6 | #Feature F7A | #Feature F7B | #Feature F8A | #Feature F8B | #Feature F9 | Start | End | PTM | AScore | Found By |
| --- | --- | --- | --- | --- | --- | --- | --- | --- | --- | --- | --- | --- | --- | --- | --- | --- | --- | --- | --- | --- | --- | --- | --- | --- | --- | --- | --- | --- | --- | --- | --- | --- | --- | --- | --- | --- | --- |
| Y.AIYGC(+57.02)YC(+57.02)GWGGQGR.A | N | 86.30 | 1603.6660 | 14 | 1.3 | 802.8413 | 2 | 12.17 | 7 | F7:2932 | DaRuMP\_F7B.raw |  |  |  |  |  |  | 4.3334E6 |  |  |  | 1 | 0 | 0 | 0 | 0 | 0 | 0 | 1 | 0 | 0 | 0 | 38 | 51 | Carbamidomethylation | C5:Carbamidomethylation:1000.00;C7:Carbamidomethylation:1000.00 | PEAKS DB |
| C.YC(+57.02)GWGGQGR.A | N | 64.40 | 1039.4294 | 9 | -0.3 | 520.7219 | 2 | 11.24 | 7 | F7:2062 | DaRuMP\_F7B.raw |  |  |  |  |  |  | 2.1146E5 |  |  |  | 1 | 0 | 0 | 0 | 0 | 0 | 0 | 1 | 0 | 0 | 0 | 43 | 51 | Carbamidomethylation | C2:Carbamidomethylation:1000.00 | PEAKS DB |
| K.NYEYYSISHC(+57.02)TEESEQC(+57.02) | N | 60.58 | 2197.8206 | 17 | 0.4 | 1099.9180 | 2 | 11.80 | 7 | F7:2602 | DaRuMP\_F7B.raw |  |  |  |  |  |  | 9.133E6 |  |  |  | 1 | 0 | 0 | 0 | 0 | 0 | 0 | 1 | 0 | 0 | 0 | 122 | 138 | Carbamidomethylation | C10:Carbamidomethylation:1000.00;C17:Carbamidomethylation:1000.00 | PEAKS DB |
| K.EAVHSYAIYGC(+57.02)Y.C | N | 59.86 | 1431.6129 | 12 | -1.6 | 716.8126 | 2 | 12.47 | 7 | F7:3181 | DaRuMP\_F7B.raw |  |  |  |  |  |  | 9.7658E7 |  |  |  | 1 | 0 | 0 | 0 | 0 | 0 | 0 | 1 | 0 | 0 | 0 | 32 | 43 | Carbamidomethylation | C11:Carbamidomethylation:1000.00 | PEAKS DB |
| R.AAAIC(+57.02)LGENVNTYDK.N | Y | 58.05 | 1637.7719 | 15 | 4.6 | 819.8970 | 2 | 12.08 | 7 | F7:2866 | DaRuMP\_F7B.raw |  |  |  |  |  |  | 0 |  |  |  | 0 | 0 | 0 | 0 | 0 | 0 | 0 | 0 | 0 | 0 | 0 | 107 | 121 | Carbamidomethylation | C5:Carbamidomethylation:1000.00 | PEAKS DB |
| K.EAVHSYAIYGC(+57.02).Y | N | 54.88 | 1268.5496 | 11 | -1.4 | 635.2812 | 2 | 11.86 | 7 | F7:2668 | DaRuMP\_F7B.raw |  |  |  |  |  |  | 2.17E6 |  |  |  | 1 | 0 | 0 | 0 | 0 | 0 | 0 | 1 | 0 | 0 | 0 | 32 | 42 | Carbamidomethylation | C11:Carbamidomethylation:1000.00 | PEAKS DB |
| Y.SFENGDIVC(+57.02)GDNDLC(+57.02)LR.A | N | 52.93 | 1982.8462 | 17 | 0.7 | 992.4310 | 2 | 16.34 | 7 | F7:6484 | DaRuMP\_F7B.raw |  |  |  |  |  |  | 6.9494E6 |  |  |  | 1 | 0 | 0 | 0 | 0 | 0 | 0 | 1 | 0 | 0 | 0 | 83 | 99 | Carbamidomethylation | C9:Carbamidomethylation:1000.00;C15:Carbamidomethylation:1000.00 | PEAKS DB |
| total 7 peptides |
| --- |

Q10754|PA2H\_VIPAZ

back to list

  

| Protein Coverage
| Supporting Peptides
|

Protein Coverage:

Supporting Peptides:

| Peptide | Uniq | -10lgP | Mass | Length | ppm | m/z | z | RT | Fraction | Scan | Source File | Area F1 | Area F10 | Area F2\_3 | Area F5 | Area F6 | Area F7A | Area F7B | Area F8A | Area F8B | Area F9 | #Feature | #Feature F1 | #Feature F10 | #Feature F2\_3 | #Feature F5 | #Feature F6 | #Feature F7A | #Feature F7B | #Feature F8A | #Feature F8B | #Feature F9 | Start | End | PTM | AScore | Found By |
| --- | --- | --- | --- | --- | --- | --- | --- | --- | --- | --- | --- | --- | --- | --- | --- | --- | --- | --- | --- | --- | --- | --- | --- | --- | --- | --- | --- | --- | --- | --- | --- | --- | --- | --- | --- | --- | --- |
| Y.AIYGC(+57.02)YC(+57.02)GWGGQGR.A | N | 86.30 | 1603.6660 | 14 | 1.3 | 802.8413 | 2 | 12.17 | 7 | F7:2932 | DaRuMP\_F7B.raw |  |  |  |  |  |  | 4.3334E6 |  |  |  | 1 | 0 | 0 | 0 | 0 | 0 | 0 | 1 | 0 | 0 | 0 | 38 | 51 | Carbamidomethylation | C5:Carbamidomethylation:1000.00;C7:Carbamidomethylation:1000.00 | PEAKS DB |
| C.YC(+57.02)GWGGQGR.A | N | 64.40 | 1039.4294 | 9 | -0.3 | 520.7219 | 2 | 11.24 | 7 | F7:2062 | DaRuMP\_F7B.raw |  |  |  |  |  |  | 2.1146E5 |  |  |  | 1 | 0 | 0 | 0 | 0 | 0 | 0 | 1 | 0 | 0 | 0 | 43 | 51 | Carbamidomethylation | C2:Carbamidomethylation:1000.00 | PEAKS DB |
| K.NYEYYSISHC(+57.02)TEESEQC(+57.02) | N | 60.58 | 2197.8206 | 17 | 0.4 | 1099.9180 | 2 | 11.80 | 7 | F7:2602 | DaRuMP\_F7B.raw |  |  |  |  |  |  | 9.133E6 |  |  |  | 1 | 0 | 0 | 0 | 0 | 0 | 0 | 1 | 0 | 0 | 0 | 122 | 138 | Carbamidomethylation | C10:Carbamidomethylation:1000.00;C17:Carbamidomethylation:1000.00 | PEAKS DB |
| K.EAVHSYAIYGC(+57.02)Y.C | N | 59.86 | 1431.6129 | 12 | -1.6 | 716.8126 | 2 | 12.47 | 7 | F7:3181 | DaRuMP\_F7B.raw |  |  |  |  |  |  | 9.7658E7 |  |  |  | 1 | 0 | 0 | 0 | 0 | 0 | 0 | 1 | 0 | 0 | 0 | 32 | 43 | Carbamidomethylation | C11:Carbamidomethylation:1000.00 | PEAKS DB |
| R.AAAIC(+57.02)LGENVNTYDK.N | Y | 58.05 | 1637.7719 | 15 | 4.6 | 819.8970 | 2 | 12.08 | 7 | F7:2866 | DaRuMP\_F7B.raw |  |  |  |  |  |  | 0 |  |  |  | 0 | 0 | 0 | 0 | 0 | 0 | 0 | 0 | 0 | 0 | 0 | 107 | 121 | Carbamidomethylation | C5:Carbamidomethylation:1000.00 | PEAKS DB |
| K.EAVHSYAIYGC(+57.02).Y | N | 54.88 | 1268.5496 | 11 | -1.4 | 635.2812 | 2 | 11.86 | 7 | F7:2668 | DaRuMP\_F7B.raw |  |  |  |  |  |  | 2.17E6 |  |  |  | 1 | 0 | 0 | 0 | 0 | 0 | 0 | 1 | 0 | 0 | 0 | 32 | 42 | Carbamidomethylation | C11:Carbamidomethylation:1000.00 | PEAKS DB |
| Y.SFENGDIVC(+57.02)GDNDLC(+57.02)LR.A | N | 52.93 | 1982.8462 | 17 | 0.7 | 992.4310 | 2 | 16.34 | 7 | F7:6484 | DaRuMP\_F7B.raw |  |  |  |  |  |  | 6.9494E6 |  |  |  | 1 | 0 | 0 | 0 | 0 | 0 | 0 | 1 | 0 | 0 | 0 | 83 | 99 | Carbamidomethylation | C9:Carbamidomethylation:1000.00;C15:Carbamidomethylation:1000.00 | PEAKS DB |
| total 7 peptides |
| --- |

A4VBF0|PA2H\_VIPBN

back to list

  

| Protein Coverage
| Supporting Peptides
|

Protein Coverage:

Supporting Peptides:

| Peptide | Uniq | -10lgP | Mass | Length | ppm | m/z | z | RT | Fraction | Scan | Source File | Area F1 | Area F10 | Area F2\_3 | Area F5 | Area F6 | Area F7A | Area F7B | Area F8A | Area F8B | Area F9 | #Feature | #Feature F1 | #Feature F10 | #Feature F2\_3 | #Feature F5 | #Feature F6 | #Feature F7A | #Feature F7B | #Feature F8A | #Feature F8B | #Feature F9 | Start | End | PTM | AScore | Found By |
| --- | --- | --- | --- | --- | --- | --- | --- | --- | --- | --- | --- | --- | --- | --- | --- | --- | --- | --- | --- | --- | --- | --- | --- | --- | --- | --- | --- | --- | --- | --- | --- | --- | --- | --- | --- | --- | --- |
| Y.AIYGC(+57.02)YC(+57.02)GWGGQGR.A | N | 86.30 | 1603.6660 | 14 | 1.3 | 802.8413 | 2 | 12.17 | 7 | F7:2932 | DaRuMP\_F7B.raw |  |  |  |  |  |  | 4.3334E6 |  |  |  | 1 | 0 | 0 | 0 | 0 | 0 | 0 | 1 | 0 | 0 | 0 | 38 | 51 | Carbamidomethylation | C5:Carbamidomethylation:1000.00;C7:Carbamidomethylation:1000.00 | PEAKS DB |
| C.YC(+57.02)GWGGQGR.A | N | 64.40 | 1039.4294 | 9 | -0.3 | 520.7219 | 2 | 11.24 | 7 | F7:2062 | DaRuMP\_F7B.raw |  |  |  |  |  |  | 2.1146E5 |  |  |  | 1 | 0 | 0 | 0 | 0 | 0 | 0 | 1 | 0 | 0 | 0 | 43 | 51 | Carbamidomethylation | C2:Carbamidomethylation:1000.00 | PEAKS DB |
| K.NYEYYSISHC(+57.02)TEESEQC(+57.02) | N | 60.58 | 2197.8206 | 17 | 0.4 | 1099.9180 | 2 | 11.80 | 7 | F7:2602 | DaRuMP\_F7B.raw |  |  |  |  |  |  | 9.133E6 |  |  |  | 1 | 0 | 0 | 0 | 0 | 0 | 0 | 1 | 0 | 0 | 0 | 122 | 138 | Carbamidomethylation | C10:Carbamidomethylation:1000.00;C17:Carbamidomethylation:1000.00 | PEAKS DB |
| K.EAVHSYAIYGC(+57.02)Y.C | N | 59.86 | 1431.6129 | 12 | -1.6 | 716.8126 | 2 | 12.47 | 7 | F7:3181 | DaRuMP\_F7B.raw |  |  |  |  |  |  | 9.7658E7 |  |  |  | 1 | 0 | 0 | 0 | 0 | 0 | 0 | 1 | 0 | 0 | 0 | 32 | 43 | Carbamidomethylation | C11:Carbamidomethylation:1000.00 | PEAKS DB |
| R.AAAIC(+57.02)LGENVNTYDK.N | Y | 58.05 | 1637.7719 | 15 | 4.6 | 819.8970 | 2 | 12.08 | 7 | F7:2866 | DaRuMP\_F7B.raw |  |  |  |  |  |  | 0 |  |  |  | 0 | 0 | 0 | 0 | 0 | 0 | 0 | 0 | 0 | 0 | 0 | 107 | 121 | Carbamidomethylation | C5:Carbamidomethylation:1000.00 | PEAKS DB |
| K.EAVHSYAIYGC(+57.02).Y | N | 54.88 | 1268.5496 | 11 | -1.4 | 635.2812 | 2 | 11.86 | 7 | F7:2668 | DaRuMP\_F7B.raw |  |  |  |  |  |  | 2.17E6 |  |  |  | 1 | 0 | 0 | 0 | 0 | 0 | 0 | 1 | 0 | 0 | 0 | 32 | 42 | Carbamidomethylation | C11:Carbamidomethylation:1000.00 | PEAKS DB |
| Y.SFENGDIVC(+57.02)GDNDLC(+57.02)LR.A | N | 52.93 | 1982.8462 | 17 | 0.7 | 992.4310 | 2 | 16.34 | 7 | F7:6484 | DaRuMP\_F7B.raw |  |  |  |  |  |  | 6.9494E6 |  |  |  | 1 | 0 | 0 | 0 | 0 | 0 | 0 | 1 | 0 | 0 | 0 | 83 | 99 | Carbamidomethylation | C9:Carbamidomethylation:1000.00;C15:Carbamidomethylation:1000.00 | PEAKS DB |
| total 7 peptides |
| --- |

P04084|PA2A\_VIPAE

back to list

  

| Protein Coverage
| Supporting Peptides
|

Protein Coverage:

Supporting Peptides:

| Peptide | Uniq | -10lgP | Mass | Length | ppm | m/z | z | RT | Fraction | Scan | Source File | Area F1 | Area F10 | Area F2\_3 | Area F5 | Area F6 | Area F7A | Area F7B | Area F8A | Area F8B | Area F9 | #Feature | #Feature F1 | #Feature F10 | #Feature F2\_3 | #Feature F5 | #Feature F6 | #Feature F7A | #Feature F7B | #Feature F8A | #Feature F8B | #Feature F9 | Start | End | PTM | AScore | Found By |
| --- | --- | --- | --- | --- | --- | --- | --- | --- | --- | --- | --- | --- | --- | --- | --- | --- | --- | --- | --- | --- | --- | --- | --- | --- | --- | --- | --- | --- | --- | --- | --- | --- | --- | --- | --- | --- | --- |
| Y.AIYGC(+57.02)YC(+57.02)GWGGQGR.A | N | 86.30 | 1603.6660 | 14 | 1.3 | 802.8413 | 2 | 12.17 | 7 | F7:2932 | DaRuMP\_F7B.raw |  |  |  |  |  |  | 4.3334E6 |  |  |  | 1 | 0 | 0 | 0 | 0 | 0 | 0 | 1 | 0 | 0 | 0 | 22 | 35 | Carbamidomethylation | C5:Carbamidomethylation:1000.00;C7:Carbamidomethylation:1000.00 | PEAKS DB |
| C.YC(+57.02)GWGGQGR.A | N | 64.40 | 1039.4294 | 9 | -0.3 | 520.7219 | 2 | 11.24 | 7 | F7:2062 | DaRuMP\_F7B.raw |  |  |  |  |  |  | 2.1146E5 |  |  |  | 1 | 0 | 0 | 0 | 0 | 0 | 0 | 1 | 0 | 0 | 0 | 27 | 35 | Carbamidomethylation | C2:Carbamidomethylation:1000.00 | PEAKS DB |
| K.NYEYYSISHC(+57.02)TEESEQC(+57.02) | N | 60.58 | 2197.8206 | 17 | 0.4 | 1099.9180 | 2 | 11.80 | 7 | F7:2602 | DaRuMP\_F7B.raw |  |  |  |  |  |  | 9.133E6 |  |  |  | 1 | 0 | 0 | 0 | 0 | 0 | 0 | 1 | 0 | 0 | 0 | 106 | 122 | Carbamidomethylation | C10:Carbamidomethylation:1000.00;C17:Carbamidomethylation:1000.00 | PEAKS DB |
| K.EAVHSYAIYGC(+57.02)Y.C | N | 59.86 | 1431.6129 | 12 | -1.6 | 716.8126 | 2 | 12.47 | 7 | F7:3181 | DaRuMP\_F7B.raw |  |  |  |  |  |  | 9.7658E7 |  |  |  | 1 | 0 | 0 | 0 | 0 | 0 | 0 | 1 | 0 | 0 | 0 | 16 | 27 | Carbamidomethylation | C11:Carbamidomethylation:1000.00 | PEAKS DB |
| R.AAAIC(+57.02)LGENVNTYDK.N | Y | 58.05 | 1637.7719 | 15 | 4.6 | 819.8970 | 2 | 12.08 | 7 | F7:2866 | DaRuMP\_F7B.raw |  |  |  |  |  |  | 0 |  |  |  | 0 | 0 | 0 | 0 | 0 | 0 | 0 | 0 | 0 | 0 | 0 | 91 | 105 | Carbamidomethylation | C5:Carbamidomethylation:1000.00 | PEAKS DB |
| K.EAVHSYAIYGC(+57.02).Y | N | 54.88 | 1268.5496 | 11 | -1.4 | 635.2812 | 2 | 11.86 | 7 | F7:2668 | DaRuMP\_F7B.raw |  |  |  |  |  |  | 2.17E6 |  |  |  | 1 | 0 | 0 | 0 | 0 | 0 | 0 | 1 | 0 | 0 | 0 | 16 | 26 | Carbamidomethylation | C11:Carbamidomethylation:1000.00 | PEAKS DB |
| Y.SFENGDIVC(+57.02)GDNDLC(+57.02)LR.A | N | 52.93 | 1982.8462 | 17 | 0.7 | 992.4310 | 2 | 16.34 | 7 | F7:6484 | DaRuMP\_F7B.raw |  |  |  |  |  |  | 6.9494E6 |  |  |  | 1 | 0 | 0 | 0 | 0 | 0 | 0 | 1 | 0 | 0 | 0 | 67 | 83 | Carbamidomethylation | C9:Carbamidomethylation:1000.00;C15:Carbamidomethylation:1000.00 | PEAKS DB |
| total 7 peptides |
| --- |

Q14525|KT33B\_HUMAN

back to list

  

| Protein Coverage
| Supporting Peptides
|

Protein Coverage:

Supporting Peptides:

| Peptide | Uniq | -10lgP | Mass | Length | ppm | m/z | z | RT | Fraction | Scan | Source File | Area F1 | Area F10 | Area F2\_3 | Area F5 | Area F6 | Area F7A | Area F7B | Area F8A | Area F8B | Area F9 | #Feature | #Feature F1 | #Feature F10 | #Feature F2\_3 | #Feature F5 | #Feature F6 | #Feature F7A | #Feature F7B | #Feature F8A | #Feature F8B | #Feature F9 | Start | End | PTM | AScore | Found By |
| --- | --- | --- | --- | --- | --- | --- | --- | --- | --- | --- | --- | --- | --- | --- | --- | --- | --- | --- | --- | --- | --- | --- | --- | --- | --- | --- | --- | --- | --- | --- | --- | --- | --- | --- | --- | --- | --- |
| R.LEC(+57.02)EINTYR.S | N | 69.77 | 1196.5496 | 9 | 0.5 | 599.2823 | 2 | 11.41 | 3 | F3:2220 | DaRuMP\_F2\_F3.raw |  |  | 1.783E5 |  |  |  |  |  |  |  | 1 | 0 | 0 | 1 | 0 | 0 | 0 | 0 | 0 | 0 | 0 | 349 | 357 | Carbamidomethylation | C3:Carbamidomethylation:1000.00 | PEAKS DB |
| R.NQYEALVETNR.R | Y | 65.87 | 1335.6418 | 11 | 0.0 | 668.8282 | 2 | 11.65 | 3 | F3:2443 | DaRuMP\_F2\_F3.raw |  |  | 4.2338E4 |  |  |  |  |  |  |  | 1 | 0 | 0 | 1 | 0 | 0 | 0 | 0 | 0 | 0 | 0 | 230 | 240 |  |  | PEAKS DB |
| R.LNVEVDAAPAVDLNQVLNETR.N | N | 61.07 | 2279.1758 | 21 | 0.0 | 1140.5952 | 2 | 35.69 | 9 | F9:12666 | DaRuMP\_F8B.raw |  |  |  |  |  |  |  |  | 4.2626E4 |  | 1 | 0 | 0 | 0 | 0 | 0 | 0 | 0 | 0 | 1 | 0 | 209 | 229 |  |  | PEAKS DB |
| R.DNAELENLIR.E | N | 57.77 | 1185.5989 | 10 | -0.3 | 593.8065 | 2 | 16.94 | 3 | F3:4566 | DaRuMP\_F2\_F3.raw |  |  | 2.6528E5 |  |  |  |  |  |  |  | 1 | 0 | 0 | 1 | 0 | 0 | 0 | 0 | 0 | 0 | 0 | 80 | 89 |  |  | PEAKS DB |
| R.EVEQWFATQTEELNK.Q | N | 54.95 | 1850.8687 | 15 | 1.1 | 926.4426 | 2 | 18.46 | 3 | F3:4960 | DaRuMP\_F2\_F3.raw |  |  | 2.1823E4 |  |  |  |  |  |  |  | 1 | 0 | 0 | 1 | 0 | 0 | 0 | 0 | 0 | 0 | 0 | 242 | 256 |  |  | PEAKS DB |
| R.QNQEYQVLLDVR.A | N | 52.92 | 1503.7681 | 12 | 0.7 | 752.8918 | 2 | 16.16 | 3 | F3:4367 | DaRuMP\_F2\_F3.raw |  |  | 2.3256E5 |  |  |  |  |  |  |  | 1 | 0 | 0 | 1 | 0 | 0 | 0 | 0 | 0 | 0 | 0 | 335 | 346 |  |  | PEAKS DB |
| total 6 peptides |
| --- |

B7FDI0|CRVP\_VIPBN

back to list

  

| Protein Coverage
| Supporting Peptides
|

Protein Coverage:

Supporting Peptides:

| Peptide | Uniq | -10lgP | Mass | Length | ppm | m/z | z | RT | Fraction | Scan | Source File | Area F1 | Area F10 | Area F2\_3 | Area F5 | Area F6 | Area F7A | Area F7B | Area F8A | Area F8B | Area F9 | #Feature | #Feature F1 | #Feature F10 | #Feature F2\_3 | #Feature F5 | #Feature F6 | #Feature F7A | #Feature F7B | #Feature F8A | #Feature F8B | #Feature F9 | Start | End | PTM | AScore | Found By |
| --- | --- | --- | --- | --- | --- | --- | --- | --- | --- | --- | --- | --- | --- | --- | --- | --- | --- | --- | --- | --- | --- | --- | --- | --- | --- | --- | --- | --- | --- | --- | --- | --- | --- | --- | --- | --- | --- |
| K.M(+15.99)EWYPEAAANAER.W | N | 77.45 | 1552.6616 | 13 | -1.4 | 777.3370 | 2 | 11.92 | 5 | F5:2764 | DaRuMP\_F6.raw |  |  |  |  | 3.1648E6 |  |  |  |  |  | 1 | 0 | 0 | 0 | 0 | 1 | 0 | 0 | 0 | 0 | 0 | 40 | 52 | Oxidation (M) | M1:Oxidation (M):1000.00 | PEAKS DB |
| K.MEWYPEAAANAER.W | N | 66.12 | 1536.6667 | 13 | 0.5 | 769.3410 | 2 | 12.79 | 5 | F5:3505 | DaRuMP\_F6.raw |  |  |  |  | 2.686E6 | 3.3797E5 |  |  |  |  | 2 | 0 | 0 | 0 | 0 | 1 | 1 | 0 | 0 | 0 | 0 | 40 | 52 |  |  | PEAKS DB |
| H.YTQIVWYK.S | N | 65.78 | 1099.5702 | 8 | 0.4 | 550.7926 | 2 | 12.88 | 5 | F5:3569 | DaRuMP\_F6.raw |  |  |  |  | 4.5275E6 | 5.093E5 | 9.6273E4 |  |  |  | 3 | 0 | 0 | 0 | 0 | 1 | 1 | 1 | 0 | 0 | 0 | 117 | 124 |  |  | PEAKS DB |
| K.DFVYGQGASPANAVVGH.Y | Y | 63.81 | 1687.7954 | 17 | 3.6 | 844.9060 | 2 | 14.55 | 6 | F6:4810 | DaRuMP\_F7A.raw |  |  |  |  |  | 8.5913E5 |  |  |  |  | 1 | 0 | 0 | 0 | 0 | 0 | 1 | 0 | 0 | 0 | 0 | 100 | 116 |  |  | PEAKS DB |
| M.EWYPEAAANAER.W | N | 59.50 | 1405.6262 | 12 | -1.3 | 703.8195 | 2 | 11.87 | 5 | F5:2732 | DaRuMP\_F6.raw |  |  |  |  | 1.2495E5 |  |  |  |  |  | 1 | 0 | 0 | 0 | 0 | 1 | 0 | 0 | 0 | 0 | 0 | 41 | 52 |  |  | PEAKS DB |
| A.NAVVGHYTQIVWYK.S | Y | 53.15 | 1676.8674 | 14 | 0.1 | 559.9631 | 3 | 12.31 | 5 | F5:3152 | DaRuMP\_F6.raw |  |  |  |  | 2.4806E5 |  |  |  |  |  | 1 | 0 | 0 | 0 | 0 | 1 | 0 | 0 | 0 | 0 | 0 | 111 | 124 |  |  | PEAKS DB |
| total 6 peptides |
| --- |

B7FDI1|CRVP\_VIPBE

back to list

  

| Protein Coverage
| Supporting Peptides
|

Protein Coverage:

Supporting Peptides:

| Peptide | Uniq | -10lgP | Mass | Length | ppm | m/z | z | RT | Fraction | Scan | Source File | Area F1 | Area F10 | Area F2\_3 | Area F5 | Area F6 | Area F7A | Area F7B | Area F8A | Area F8B | Area F9 | #Feature | #Feature F1 | #Feature F10 | #Feature F2\_3 | #Feature F5 | #Feature F6 | #Feature F7A | #Feature F7B | #Feature F8A | #Feature F8B | #Feature F9 | Start | End | PTM | AScore | Found By |
| --- | --- | --- | --- | --- | --- | --- | --- | --- | --- | --- | --- | --- | --- | --- | --- | --- | --- | --- | --- | --- | --- | --- | --- | --- | --- | --- | --- | --- | --- | --- | --- | --- | --- | --- | --- | --- | --- |
| K.M(+15.99)EWYPEAAANAER.W | N | 77.45 | 1552.6616 | 13 | -1.4 | 777.3370 | 2 | 11.92 | 5 | F5:2764 | DaRuMP\_F6.raw |  |  |  |  | 3.1648E6 |  |  |  |  |  | 1 | 0 | 0 | 0 | 0 | 1 | 0 | 0 | 0 | 0 | 0 | 58 | 70 | Oxidation (M) | M1:Oxidation (M):1000.00 | PEAKS DB |
| K.MEWYPEAAANAER.W | N | 66.12 | 1536.6667 | 13 | 0.5 | 769.3410 | 2 | 12.79 | 5 | F5:3505 | DaRuMP\_F6.raw |  |  |  |  | 2.686E6 | 3.3797E5 |  |  |  |  | 2 | 0 | 0 | 0 | 0 | 1 | 1 | 0 | 0 | 0 | 0 | 58 | 70 |  |  | PEAKS DB |
| H.YTQIVWYK.S | N | 65.78 | 1099.5702 | 8 | 0.4 | 550.7926 | 2 | 12.88 | 5 | F5:3569 | DaRuMP\_F6.raw |  |  |  |  | 4.5275E6 | 5.093E5 | 9.6273E4 |  |  |  | 3 | 0 | 0 | 0 | 0 | 1 | 1 | 1 | 0 | 0 | 0 | 135 | 142 |  |  | PEAKS DB |
| K.DFVYGQGASPANAVVGH.Y | Y | 63.81 | 1687.7954 | 17 | 3.6 | 844.9060 | 2 | 14.55 | 6 | F6:4810 | DaRuMP\_F7A.raw |  |  |  |  |  | 8.5913E5 |  |  |  |  | 1 | 0 | 0 | 0 | 0 | 0 | 1 | 0 | 0 | 0 | 0 | 118 | 134 |  |  | PEAKS DB |
| M.EWYPEAAANAER.W | N | 59.50 | 1405.6262 | 12 | -1.3 | 703.8195 | 2 | 11.87 | 5 | F5:2732 | DaRuMP\_F6.raw |  |  |  |  | 1.2495E5 |  |  |  |  |  | 1 | 0 | 0 | 0 | 0 | 1 | 0 | 0 | 0 | 0 | 0 | 59 | 70 |  |  | PEAKS DB |
| A.NAVVGHYTQIVWYK.S | Y | 53.15 | 1676.8674 | 14 | 0.1 | 559.9631 | 3 | 12.31 | 5 | F5:3152 | DaRuMP\_F6.raw |  |  |  |  | 2.4806E5 |  |  |  |  |  | 1 | 0 | 0 | 0 | 0 | 1 | 0 | 0 | 0 | 0 | 0 | 129 | 142 |  |  | PEAKS DB |
| total 6 peptides |
| --- |

P78386|KRT85\_HUMAN

back to list

  

| Protein Coverage
| Supporting Peptides
|

Protein Coverage:

Supporting Peptides:

| Peptide | Uniq | -10lgP | Mass | Length | ppm | m/z | z | RT | Fraction | Scan | Source File | Area F1 | Area F10 | Area F2\_3 | Area F5 | Area F6 | Area F7A | Area F7B | Area F8A | Area F8B | Area F9 | #Feature | #Feature F1 | #Feature F10 | #Feature F2\_3 | #Feature F5 | #Feature F6 | #Feature F7A | #Feature F7B | #Feature F8A | #Feature F8B | #Feature F9 | Start | End | PTM | AScore | Found By |
| --- | --- | --- | --- | --- | --- | --- | --- | --- | --- | --- | --- | --- | --- | --- | --- | --- | --- | --- | --- | --- | --- | --- | --- | --- | --- | --- | --- | --- | --- | --- | --- | --- | --- | --- | --- | --- | --- |
| R.GGVSC(+57.02)GGLSYSTTPGR.Q | Y | 65.95 | 1554.7096 | 16 | 1.3 | 778.3631 | 2 | 11.49 | 3 | F3:2311 | DaRuMP\_F2\_F3.raw |  |  | 1.9779E4 |  |  |  |  |  |  |  | 1 | 0 | 0 | 1 | 0 | 0 | 0 | 0 | 0 | 0 | 0 | 450 | 465 | Carbamidomethylation | C5:Carbamidomethylation:1000.00 | PEAKS DB |
| K.LAELEGALQK.A | N | 60.61 | 1070.5972 | 10 | 0.8 | 536.3063 | 2 | 11.80 | 3 | F3:2606 | DaRuMP\_F2\_F3.raw |  |  | 2.276E5 |  |  |  |  |  |  |  | 1 | 0 | 0 | 1 | 0 | 0 | 0 | 0 | 0 | 0 | 0 | 385 | 394 |  |  | PEAKS DB |
| R.SLC(+57.02)NLGSC(+57.02)GPR.I | Y | 60.10 | 1219.5438 | 11 | -0.2 | 610.7791 | 2 | 11.37 | 3 | F3:2186 | DaRuMP\_F2\_F3.raw |  |  | 2.6441E4 |  |  |  |  |  |  |  | 1 | 0 | 0 | 1 | 0 | 0 | 0 | 0 | 0 | 0 | 0 | 55 | 65 | Carbamidomethylation | C3:Carbamidomethylation:1000.00;C8:Carbamidomethylation:1000.00 | PEAKS DB |
| K.LGLDIEIATYR.R | N | 58.31 | 1262.6870 | 11 | 0.9 | 632.3513 | 2 | 24.34 | 9 | F9:9182 | DaRuMP\_F8B.raw |  |  |  |  |  |  |  |  | 3.0158E4 |  | 1 | 0 | 0 | 0 | 0 | 0 | 0 | 0 | 0 | 1 | 0 | 414 | 424 |  |  | PEAKS DB |
| R.TKEEINELNR.M | N | 53.74 | 1244.6360 | 10 | 0.0 | 415.8860 | 3 | 10.81 | 3 | F3:1680 | DaRuMP\_F2\_F3.raw |  |  | 1.0146E4 |  |  |  |  |  |  |  | 1 | 0 | 0 | 1 | 0 | 0 | 0 | 0 | 0 | 0 | 0 | 335 | 344 |  |  | PEAKS DB |
| R.AEAESWYR.S | N | 52.72 | 1010.4457 | 8 | 0.5 | 506.2304 | 2 | 11.49 | 3 | F3:2305 | DaRuMP\_F2\_F3.raw |  |  | 5.3586E4 |  |  |  |  |  |  |  | 1 | 0 | 0 | 1 | 0 | 0 | 0 | 0 | 0 | 0 | 0 | 308 | 315 |  |  | PEAKS DB |
| K.AQYDDVASR.S | N | 52.60 | 1023.4621 | 9 | -0.1 | 512.7383 | 2 | 10.86 | 3 | F3:1722 | DaRuMP\_F2\_F3.raw |  |  | 1.0247E4 |  |  |  |  |  |  |  | 1 | 0 | 0 | 1 | 0 | 0 | 0 | 0 | 0 | 0 | 0 | 297 | 305 |  |  | PEAKS DB |
| total 7 peptides |
| --- |

E5L0E4|VSPB\_DABSI

back to list

  

| Protein Coverage
| Supporting Peptides
|

Protein Coverage:

Supporting Peptides:

| Peptide | Uniq | -10lgP | Mass | Length | ppm | m/z | z | RT | Fraction | Scan | Source File | Area F1 | Area F10 | Area F2\_3 | Area F5 | Area F6 | Area F7A | Area F7B | Area F8A | Area F8B | Area F9 | #Feature | #Feature F1 | #Feature F10 | #Feature F2\_3 | #Feature F5 | #Feature F6 | #Feature F7A | #Feature F7B | #Feature F8A | #Feature F8B | #Feature F9 | Start | End | PTM | AScore | Found By |
| --- | --- | --- | --- | --- | --- | --- | --- | --- | --- | --- | --- | --- | --- | --- | --- | --- | --- | --- | --- | --- | --- | --- | --- | --- | --- | --- | --- | --- | --- | --- | --- | --- | --- | --- | --- | --- | --- |
| L.VVGGDEC(+57.02)NINEHR.S | N | 78.68 | 1497.6630 | 13 | -0.4 | 500.2281 | 3 | 10.71 | 9 | F9:1632 | DaRuMP\_F8B.raw |  |  |  |  | 5.6148E6 | 2.0726E5 | 4.1914E5 |  | 1.3141E6 |  | 6 | 0 | 0 | 0 | 0 | 2 | 1 | 1 | 0 | 2 | 0 | 25 | 37 | Carbamidomethylation | C7:Carbamidomethylation:1000.00 | PEAKS DB |
| K.TSTYIAPLSLPSSPPR.V | Y | 76.36 | 1685.8988 | 16 | 2.0 | 843.9563 | 2 | 18.08 | 6 | F6:7702 | DaRuMP\_F7A.raw |  |  |  |  |  | 2.0483E7 | 1.6682E7 |  |  |  | 2 | 0 | 0 | 0 | 0 | 0 | 1 | 1 | 0 | 0 | 0 | 120 | 135 |  |  | PEAKS DB |
| Q.SIIAGNTAATC(+57.02)PP | N | 62.46 | 1271.6179 | 13 | 0.4 | 636.8165 | 2 | 12.31 | 7 | F7:3064 | DaRuMP\_F7B.raw |  |  |  |  |  |  | 9.889E5 |  | 2.4084E5 |  | 2 | 0 | 0 | 0 | 0 | 0 | 0 | 1 | 0 | 1 | 0 | 244 | 256 | Carbamidomethylation | C11:Carbamidomethylation:1000.00 | PEAKS DB |
| Y.IAPLSLPSSPPR.V | Y | 56.67 | 1233.7080 | 12 | 0.7 | 617.8602 | 2 | 13.24 | 6 | F6:3782 | DaRuMP\_F7A.raw |  |  |  |  | 4.684E5 | 6.3289E5 | 7.1662E5 |  |  |  | 3 | 0 | 0 | 0 | 0 | 1 | 1 | 1 | 0 | 0 | 0 | 124 | 135 |  |  | PEAKS DB |
| R.FFC(+57.02)LSNK.N | N | 56.09 | 914.4320 | 7 | 2.5 | 458.2233 | 2 | 12.01 | 6 | F6:2752 | DaRuMP\_F7A.raw |  |  |  |  | 2.015E6 | 1.6117E6 | 2.3973E6 |  |  |  | 3 | 0 | 0 | 0 | 0 | 1 | 1 | 1 | 0 | 0 | 0 | 94 | 100 | Carbamidomethylation | C3:Carbamidomethylation:1000.00 | PEAKS DB |
| total 5 peptides |
| --- |

H6VC06|VKTK2\_DABRR

back to list

  

| Protein Coverage
| Supporting Peptides
|

Protein Coverage:

Supporting Peptides:

| Peptide | Uniq | -10lgP | Mass | Length | ppm | m/z | z | RT | Fraction | Scan | Source File | Area F1 | Area F10 | Area F2\_3 | Area F5 | Area F6 | Area F7A | Area F7B | Area F8A | Area F8B | Area F9 | #Feature | #Feature F1 | #Feature F10 | #Feature F2\_3 | #Feature F5 | #Feature F6 | #Feature F7A | #Feature F7B | #Feature F8A | #Feature F8B | #Feature F9 | Start | End | PTM | AScore | Found By |
| --- | --- | --- | --- | --- | --- | --- | --- | --- | --- | --- | --- | --- | --- | --- | --- | --- | --- | --- | --- | --- | --- | --- | --- | --- | --- | --- | --- | --- | --- | --- | --- | --- | --- | --- | --- | --- | --- |
| G.HDRPTFC(+57.02)NLAPESGR.C | N | 78.00 | 1755.8110 | 15 | -1.2 | 586.2769 | 3 | 11.26 | 1 | F1:2140 | DaRuMP\_F1.raw | 2.2348E6 |  |  |  |  |  |  |  |  |  | 1 | 1 | 0 | 0 | 0 | 0 | 0 | 0 | 0 | 0 | 0 | 25 | 39 | Carbamidomethylation | C7:Carbamidomethylation:1000.00 | PEAKS DB |
| T.FC(+57.02)NLAPESGR.C | N | 68.04 | 1149.5237 | 10 | 0.9 | 575.7697 | 2 | 11.40 | 1 | F1:2252 | DaRuMP\_F1.raw | 2.7845E6 |  |  |  |  |  |  |  |  | 8.9627E4 | 2 | 1 | 0 | 0 | 0 | 0 | 0 | 0 | 0 | 0 | 1 | 30 | 39 | Carbamidomethylation | C2:Carbamidomethylation:1000.00 | PEAKS DB |
| Y.GGC(+57.02)GGNDNNFSTWDEC(+57.02)R.H | Y | 60.92 | 1944.7115 | 17 | 0.4 | 973.3634 | 2 | 11.82 | 1 | F1:2668 | DaRuMP\_F1.raw | 4.0457E5 |  |  |  |  |  |  |  |  |  | 1 | 1 | 0 | 0 | 0 | 0 | 0 | 0 | 0 | 0 | 0 | 62 | 78 | Carbamidomethylation | C3:Carbamidomethylation:1000.00;C16:Carbamidomethylation:1000.00 | PEAKS DB |
| R.IYYNLESNK.C | N | 59.31 | 1142.5608 | 9 | 0.0 | 572.2877 | 2 | 11.61 | 1 | F1:2461 | DaRuMP\_F1.raw | 2.8939E6 |  |  |  |  |  |  |  |  | 1.7171E5 | 2 | 1 | 0 | 0 | 0 | 0 | 0 | 0 | 0 | 0 | 1 | 47 | 55 |  |  | PEAKS DB |
| R.RIYYNLESNK.C | N | 56.80 | 1298.6619 | 10 | 1.0 | 650.3389 | 2 | 11.23 | 1 | F1:2102 | DaRuMP\_F1.raw | 1.7193E5 |  |  |  |  |  |  |  |  |  | 1 | 1 | 0 | 0 | 0 | 0 | 0 | 0 | 0 | 0 | 0 | 46 | 55 |  |  | PEAKS DB |
| total 5 peptides |
| --- |

A8Y7P4|VKTB4\_DABSI

back to list

  

| Protein Coverage
| Supporting Peptides
|

Protein Coverage:

Supporting Peptides:

| Peptide | Uniq | -10lgP | Mass | Length | ppm | m/z | z | RT | Fraction | Scan | Source File | Area F1 | Area F10 | Area F2\_3 | Area F5 | Area F6 | Area F7A | Area F7B | Area F8A | Area F8B | Area F9 | #Feature | #Feature F1 | #Feature F10 | #Feature F2\_3 | #Feature F5 | #Feature F6 | #Feature F7A | #Feature F7B | #Feature F8A | #Feature F8B | #Feature F9 | Start | End | PTM | AScore | Found By |
| --- | --- | --- | --- | --- | --- | --- | --- | --- | --- | --- | --- | --- | --- | --- | --- | --- | --- | --- | --- | --- | --- | --- | --- | --- | --- | --- | --- | --- | --- | --- | --- | --- | --- | --- | --- | --- | --- |
| G.HDRPTFC(+57.02)NLAPESGR.C | N | 78.00 | 1755.8110 | 15 | -1.2 | 586.2769 | 3 | 11.26 | 1 | F1:2140 | DaRuMP\_F1.raw | 2.2348E6 |  |  |  |  |  |  |  |  |  | 1 | 1 | 0 | 0 | 0 | 0 | 0 | 0 | 0 | 0 | 0 | 25 | 39 | Carbamidomethylation | C7:Carbamidomethylation:1000.00 | PEAKS DB |
| T.FC(+57.02)NLAPESGR.C | N | 68.04 | 1149.5237 | 10 | 0.9 | 575.7697 | 2 | 11.40 | 1 | F1:2252 | DaRuMP\_F1.raw | 2.7845E6 |  |  |  |  |  |  |  |  | 8.9627E4 | 2 | 1 | 0 | 0 | 0 | 0 | 0 | 0 | 0 | 0 | 1 | 30 | 39 | Carbamidomethylation | C2:Carbamidomethylation:1000.00 | PEAKS DB |
| Y.GGC(+57.02)GGNDNNFSTWDEC(+57.02)R.H | Y | 60.92 | 1944.7115 | 17 | 0.4 | 973.3634 | 2 | 11.82 | 1 | F1:2668 | DaRuMP\_F1.raw | 4.0457E5 |  |  |  |  |  |  |  |  |  | 1 | 1 | 0 | 0 | 0 | 0 | 0 | 0 | 0 | 0 | 0 | 62 | 78 | Carbamidomethylation | C3:Carbamidomethylation:1000.00;C16:Carbamidomethylation:1000.00 | PEAKS DB |
| R.IYYNLESNK.C | N | 59.31 | 1142.5608 | 9 | 0.0 | 572.2877 | 2 | 11.61 | 1 | F1:2461 | DaRuMP\_F1.raw | 2.8939E6 |  |  |  |  |  |  |  |  | 1.7171E5 | 2 | 1 | 0 | 0 | 0 | 0 | 0 | 0 | 0 | 0 | 1 | 47 | 55 |  |  | PEAKS DB |
| R.RIYYNLESNK.C | N | 56.80 | 1298.6619 | 10 | 1.0 | 650.3389 | 2 | 11.23 | 1 | F1:2102 | DaRuMP\_F1.raw | 1.7193E5 |  |  |  |  |  |  |  |  |  | 1 | 1 | 0 | 0 | 0 | 0 | 0 | 0 | 0 | 0 | 0 | 46 | 55 |  |  | PEAKS DB |
| total 5 peptides |
| --- |

A8Y7N8|VKTC5\_DABSI

back to list

  

| Protein Coverage
| Supporting Peptides
|

Protein Coverage:

Supporting Peptides:

| Peptide | Uniq | -10lgP | Mass | Length | ppm | m/z | z | RT | Fraction | Scan | Source File | Area F1 | Area F10 | Area F2\_3 | Area F5 | Area F6 | Area F7A | Area F7B | Area F8A | Area F8B | Area F9 | #Feature | #Feature F1 | #Feature F10 | #Feature F2\_3 | #Feature F5 | #Feature F6 | #Feature F7A | #Feature F7B | #Feature F8A | #Feature F8B | #Feature F9 | Start | End | PTM | AScore | Found By |
| --- | --- | --- | --- | --- | --- | --- | --- | --- | --- | --- | --- | --- | --- | --- | --- | --- | --- | --- | --- | --- | --- | --- | --- | --- | --- | --- | --- | --- | --- | --- | --- | --- | --- | --- | --- | --- | --- |
| G.HDRPTFC(+57.02)NLAPESGR.C | N | 78.00 | 1755.8110 | 15 | -1.2 | 586.2769 | 3 | 11.26 | 1 | F1:2140 | DaRuMP\_F1.raw | 2.2348E6 |  |  |  |  |  |  |  |  |  | 1 | 1 | 0 | 0 | 0 | 0 | 0 | 0 | 0 | 0 | 0 | 25 | 39 | Carbamidomethylation | C7:Carbamidomethylation:1000.00 | PEAKS DB |
| T.FC(+57.02)NLAPESGR.C | N | 68.04 | 1149.5237 | 10 | 0.9 | 575.7697 | 2 | 11.40 | 1 | F1:2252 | DaRuMP\_F1.raw | 2.7845E6 |  |  |  |  |  |  |  |  | 8.9627E4 | 2 | 1 | 0 | 0 | 0 | 0 | 0 | 0 | 0 | 0 | 1 | 30 | 39 | Carbamidomethylation | C2:Carbamidomethylation:1000.00 | PEAKS DB |
| F.FYGGC(+57.02)GGNDNNFETR.K | N | 61.65 | 1706.6743 | 15 | 0.4 | 854.3448 | 2 | 11.52 | 1 | F1:2377 | DaRuMP\_F1.raw | 2.1145E5 |  |  |  |  |  |  |  |  |  | 1 | 1 | 0 | 0 | 0 | 0 | 0 | 0 | 0 | 0 | 0 | 60 | 74 | Carbamidomethylation | C5:Carbamidomethylation:1000.00 | PEAKS DB |
| R.IYYNPDSNK.C | Y | 52.46 | 1112.5138 | 9 | 0.5 | 557.2645 | 2 | 11.15 | 1 | F1:2030 | DaRuMP\_F1.raw | 9.291E5 |  |  |  |  |  |  |  |  |  | 1 | 1 | 0 | 0 | 0 | 0 | 0 | 0 | 0 | 0 | 0 | 47 | 55 |  |  | PEAKS DB |
| total 4 peptides |
| --- |

Q4PRD2|SLLC2\_DABSI

back to list

  

| Protein Coverage
| Supporting Peptides
|

Protein Coverage:

Supporting Peptides:

| Peptide | Uniq | -10lgP | Mass | Length | ppm | m/z | z | RT | Fraction | Scan | Source File | Area F1 | Area F10 | Area F2\_3 | Area F5 | Area F6 | Area F7A | Area F7B | Area F8A | Area F8B | Area F9 | #Feature | #Feature F1 | #Feature F10 | #Feature F2\_3 | #Feature F5 | #Feature F6 | #Feature F7A | #Feature F7B | #Feature F8A | #Feature F8B | #Feature F9 | Start | End | PTM | AScore | Found By |
| --- | --- | --- | --- | --- | --- | --- | --- | --- | --- | --- | --- | --- | --- | --- | --- | --- | --- | --- | --- | --- | --- | --- | --- | --- | --- | --- | --- | --- | --- | --- | --- | --- | --- | --- | --- | --- | --- |
| K.EQEC(+57.02)SSEWSDGSSVSYDK.L | Y | 86.32 | 2078.8010 | 18 | 0.4 | 1040.4082 | 2 | 11.57 | 9 | F9:2421 | DaRuMP\_F8B.raw |  |  |  |  |  |  | 5.6986E5 |  | 2.9076E5 |  | 2 | 0 | 0 | 0 | 0 | 0 | 0 | 1 | 0 | 1 | 0 | 101 | 118 | Carbamidomethylation | C4:Carbamidomethylation:1000.00 | PEAKS DB |
| K.DKEQEC(+57.02)SSEWSDGSSVSYDK.L | Y | 74.21 | 2321.9229 | 20 | 1.0 | 774.9824 | 3 | 11.39 | 9 | F9:2244 | DaRuMP\_F8B.raw |  |  |  |  |  |  |  |  | 2.7975E5 |  | 1 | 0 | 0 | 0 | 0 | 0 | 0 | 0 | 0 | 1 | 0 | 99 | 118 | Carbamidomethylation | C6:Carbamidomethylation:1000.00 | PEAKS DB |
| G.LDC(+57.02)PPDSSLYR.Y | Y | 62.37 | 1321.5972 | 11 | -0.5 | 661.8055 | 2 | 11.68 | 9 | F9:2514 | DaRuMP\_F8B.raw |  |  |  |  |  |  |  |  | 1.9717E5 |  | 1 | 0 | 0 | 0 | 0 | 0 | 0 | 0 | 0 | 1 | 0 | 25 | 35 | Carbamidomethylation | C3:Carbamidomethylation:1000.00 | PEAKS DB |
| total 3 peptides |
| --- |

Q4VM07|VM3VB\_MACLB

back to list

  

| Protein Coverage
| Supporting Peptides
|

Protein Coverage:

Supporting Peptides:

| Peptide | Uniq | -10lgP | Mass | Length | ppm | m/z | z | RT | Fraction | Scan | Source File | Area F1 | Area F10 | Area F2\_3 | Area F5 | Area F6 | Area F7A | Area F7B | Area F8A | Area F8B | Area F9 | #Feature | #Feature F1 | #Feature F10 | #Feature F2\_3 | #Feature F5 | #Feature F6 | #Feature F7A | #Feature F7B | #Feature F8A | #Feature F8B | #Feature F9 | Start | End | PTM | AScore | Found By |
| --- | --- | --- | --- | --- | --- | --- | --- | --- | --- | --- | --- | --- | --- | --- | --- | --- | --- | --- | --- | --- | --- | --- | --- | --- | --- | --- | --- | --- | --- | --- | --- | --- | --- | --- | --- | --- | --- |
| L.ESGNVNDYEVVYPQK.I | N | 83.54 | 1739.8002 | 15 | 0.4 | 870.9077 | 2 | 11.80 | 3 | F3:2602 | DaRuMP\_F2\_F3.raw |  |  | 1.005E6 |  |  |  |  |  |  |  | 1 | 0 | 0 | 1 | 0 | 0 | 0 | 0 | 0 | 0 | 0 | 24 | 38 |  |  | PEAKS DB |
| K.YSVGIVQDHSK.I | Y | 82.99 | 1231.6196 | 11 | -0.3 | 616.8169 | 2 | 11.22 | 2 | F2:2069 | DaRuMP\_F10.raw |  | 8.1974E6 |  |  |  |  |  |  |  |  | 2 | 0 | 2 | 0 | 0 | 0 | 0 | 0 | 0 | 0 | 0 | 317 | 327 |  |  | PEAKS DB |
| E.SGNVNDYEVVYPQK.I | N | 72.78 | 1610.7576 | 14 | 0.7 | 806.3866 | 2 | 11.74 | 3 | F3:2548 | DaRuMP\_F2\_F3.raw |  |  | 4.3606E4 |  |  |  |  |  |  |  | 1 | 0 | 0 | 1 | 0 | 0 | 0 | 0 | 0 | 0 | 0 | 25 | 38 |  |  | PEAKS DB |
| total 3 peptides |
| --- |

O76011|KRT34\_HUMAN

back to list

  

| Protein Coverage
| Supporting Peptides
|

Protein Coverage:

Supporting Peptides:

| Peptide | Uniq | -10lgP | Mass | Length | ppm | m/z | z | RT | Fraction | Scan | Source File | Area F1 | Area F10 | Area F2\_3 | Area F5 | Area F6 | Area F7A | Area F7B | Area F8A | Area F8B | Area F9 | #Feature | #Feature F1 | #Feature F10 | #Feature F2\_3 | #Feature F5 | #Feature F6 | #Feature F7A | #Feature F7B | #Feature F8A | #Feature F8B | #Feature F9 | Start | End | PTM | AScore | Found By |
| --- | --- | --- | --- | --- | --- | --- | --- | --- | --- | --- | --- | --- | --- | --- | --- | --- | --- | --- | --- | --- | --- | --- | --- | --- | --- | --- | --- | --- | --- | --- | --- | --- | --- | --- | --- | --- | --- |
| R.LEC(+57.02)EINTYR.S | N | 69.77 | 1196.5496 | 9 | 0.5 | 599.2823 | 2 | 11.41 | 3 | F3:2220 | DaRuMP\_F2\_F3.raw |  |  | 1.783E5 |  |  |  |  |  |  |  | 1 | 0 | 0 | 1 | 0 | 0 | 0 | 0 | 0 | 0 | 0 | 391 | 399 | Carbamidomethylation | C3:Carbamidomethylation:1000.00 | PEAKS DB |
| R.EVEQWFATQTEELNK.Q | N | 54.95 | 1850.8687 | 15 | 1.1 | 926.4426 | 2 | 18.46 | 3 | F3:4960 | DaRuMP\_F2\_F3.raw |  |  | 2.1823E4 |  |  |  |  |  |  |  | 1 | 0 | 0 | 1 | 0 | 0 | 0 | 0 | 0 | 0 | 0 | 284 | 298 |  |  | PEAKS DB |
| R.LLVESDINSIR.R | Y | 54.35 | 1257.6929 | 11 | -0.1 | 629.8536 | 2 | 12.99 | 3 | F3:3482 | DaRuMP\_F2\_F3.raw |  |  | 5.7452E4 |  |  |  |  |  |  |  | 1 | 0 | 0 | 1 | 0 | 0 | 0 | 0 | 0 | 0 | 0 | 195 | 205 |  |  | PEAKS DB |
| R.QNQEYQVLLDVR.A | N | 52.92 | 1503.7681 | 12 | 0.7 | 752.8918 | 2 | 16.16 | 3 | F3:4367 | DaRuMP\_F2\_F3.raw |  |  | 2.3256E5 |  |  |  |  |  |  |  | 1 | 0 | 0 | 1 | 0 | 0 | 0 | 0 | 0 | 0 | 0 | 377 | 388 |  |  | PEAKS DB |
| total 4 peptides |
| --- |

P24541|VKT\_ERIMA

back to list

  

| Protein Coverage
| Supporting Peptides
|

Protein Coverage:

Supporting Peptides:

| Peptide | Uniq | -10lgP | Mass | Length | ppm | m/z | z | RT | Fraction | Scan | Source File | Area F1 | Area F10 | Area F2\_3 | Area F5 | Area F6 | Area F7A | Area F7B | Area F8A | Area F8B | Area F9 | #Feature | #Feature F1 | #Feature F10 | #Feature F2\_3 | #Feature F5 | #Feature F6 | #Feature F7A | #Feature F7B | #Feature F8A | #Feature F8B | #Feature F9 | Start | End | PTM | AScore | Found By |
| --- | --- | --- | --- | --- | --- | --- | --- | --- | --- | --- | --- | --- | --- | --- | --- | --- | --- | --- | --- | --- | --- | --- | --- | --- | --- | --- | --- | --- | --- | --- | --- | --- | --- | --- | --- | --- | --- |
| F.IYGGC(+57.02)GGNANNFETR.A | N | 84.62 | 1628.7001 | 15 | -2.6 | 815.3552 | 2 | 11.33 | 3 | F3:2146 | DaRuMP\_F2\_F3.raw | 9.5628E6 |  | 1.7411E7 | 9.596E5 |  |  |  |  |  |  | 3 | 1 | 0 | 1 | 1 | 0 | 0 | 0 | 0 | 0 | 0 | 31 | 45 | Carbamidomethylation | C5:Carbamidomethylation:1000.00 | PEAKS DB |
| K.NFIYGGC(+57.02)GGNANNFETR.A | Y | 78.71 | 1889.8115 | 17 | 0.4 | 945.9135 | 2 | 12.07 | 3 | F3:2859 | DaRuMP\_F2\_F3.raw | 1.1365E5 |  | 3.3823E5 |  |  |  |  |  |  |  | 2 | 1 | 0 | 1 | 0 | 0 | 0 | 0 | 0 | 0 | 0 | 29 | 45 | Carbamidomethylation | C7:Carbamidomethylation:1000.00 | PEAKS DB |
| Y.GGC(+57.02)GGNANNFETR.A | N | 55.54 | 1352.5527 | 13 | 0.7 | 677.2841 | 2 | 10.77 | 3 | F3:1663 | DaRuMP\_F2\_F3.raw |  |  | 3.6976E4 |  |  |  |  |  |  |  | 1 | 0 | 0 | 1 | 0 | 0 | 0 | 0 | 0 | 0 | 0 | 33 | 45 | Carbamidomethylation | C3:Carbamidomethylation:1000.00 | PEAKS DB |
| total 3 peptides |
| --- |

A8Y7N9|VKTC6\_DABSI

back to list

  

| Protein Coverage
| Supporting Peptides
|

Protein Coverage:

Supporting Peptides:

| Peptide | Uniq | -10lgP | Mass | Length | ppm | m/z | z | RT | Fraction | Scan | Source File | Area F1 | Area F10 | Area F2\_3 | Area F5 | Area F6 | Area F7A | Area F7B | Area F8A | Area F8B | Area F9 | #Feature | #Feature F1 | #Feature F10 | #Feature F2\_3 | #Feature F5 | #Feature F6 | #Feature F7A | #Feature F7B | #Feature F8A | #Feature F8B | #Feature F9 | Start | End | PTM | AScore | Found By |
| --- | --- | --- | --- | --- | --- | --- | --- | --- | --- | --- | --- | --- | --- | --- | --- | --- | --- | --- | --- | --- | --- | --- | --- | --- | --- | --- | --- | --- | --- | --- | --- | --- | --- | --- | --- | --- | --- |
| F.TYGGC(+57.02)GGNANNFETR.D | Y | 68.84 | 1616.6637 | 15 | 1.0 | 809.3399 | 2 | 11.11 | 3 | F3:1936 | DaRuMP\_F2\_F3.raw |  |  | 1.2178E4 |  |  |  |  |  |  |  | 1 | 0 | 0 | 1 | 0 | 0 | 0 | 0 | 0 | 0 | 0 | 60 | 74 | Carbamidomethylation | C5:Carbamidomethylation:1000.00 | PEAKS DB |
| R.FYYNPASNQC(+57.02)QGF.T | N | 66.89 | 1594.6510 | 13 | 1.1 | 798.3337 | 2 | 19.30 | 3 | F3:5295 | DaRuMP\_F2\_F3.raw | 1.2086E7 |  | 1.8478E7 |  |  |  |  |  |  |  | 2 | 1 | 0 | 1 | 0 | 0 | 0 | 0 | 0 | 0 | 0 | 47 | 59 | Carbamidomethylation | C10:Carbamidomethylation:1000.00 | PEAKS DB |
| Y.GGC(+57.02)GGNANNFETR.D | N | 55.54 | 1352.5527 | 13 | 0.7 | 677.2841 | 2 | 10.77 | 3 | F3:1663 | DaRuMP\_F2\_F3.raw |  |  | 3.6976E4 |  |  |  |  |  |  |  | 1 | 0 | 0 | 1 | 0 | 0 | 0 | 0 | 0 | 0 | 0 | 62 | 74 | Carbamidomethylation | C3:Carbamidomethylation:1000.00 | PEAKS DB |
| R.FYYNPASNQC(+57.02).Q | N | 53.29 | 1262.5026 | 10 | 0.2 | 632.2587 | 2 | 12.80 | 3 | F3:3327 | DaRuMP\_F2\_F3.raw |  |  | 3.4592E5 |  |  |  |  |  |  |  | 1 | 0 | 0 | 1 | 0 | 0 | 0 | 0 | 0 | 0 | 0 | 47 | 56 | Carbamidomethylation | C10:Carbamidomethylation:1000.00 | PEAKS DB |
| R.FYYNPASNQC(+57.02)Q.G | N | 52.69 | 1390.5612 | 11 | 0.7 | 696.2883 | 2 | 12.27 | 1 | F1:3065 | DaRuMP\_F1.raw | 1.5225E5 |  |  |  |  |  |  |  |  |  | 1 | 1 | 0 | 0 | 0 | 0 | 0 | 0 | 0 | 0 | 0 | 47 | 57 | Carbamidomethylation | C10:Carbamidomethylation:1000.00 | PEAKS DB |
| total 5 peptides |
| --- |

E0Y419|VSPBF\_MACLB

back to list

  

| Protein Coverage
| Supporting Peptides
|

Protein Coverage:

Supporting Peptides:

| Peptide | Uniq | -10lgP | Mass | Length | ppm | m/z | z | RT | Fraction | Scan | Source File | Area F1 | Area F10 | Area F2\_3 | Area F5 | Area F6 | Area F7A | Area F7B | Area F8A | Area F8B | Area F9 | #Feature | #Feature F1 | #Feature F10 | #Feature F2\_3 | #Feature F5 | #Feature F6 | #Feature F7A | #Feature F7B | #Feature F8A | #Feature F8B | #Feature F9 | Start | End | PTM | AScore | Found By |
| --- | --- | --- | --- | --- | --- | --- | --- | --- | --- | --- | --- | --- | --- | --- | --- | --- | --- | --- | --- | --- | --- | --- | --- | --- | --- | --- | --- | --- | --- | --- | --- | --- | --- | --- | --- | --- | --- |
| L.VVGGDEC(+57.02)NINEHR.S | N | 78.68 | 1497.6630 | 13 | -0.4 | 500.2281 | 3 | 10.71 | 9 | F9:1632 | DaRuMP\_F8B.raw |  |  |  |  | 5.6148E6 | 2.0726E5 | 4.1914E5 |  | 1.3141E6 |  | 6 | 0 | 0 | 0 | 0 | 2 | 1 | 1 | 0 | 2 | 0 | 25 | 37 | Carbamidomethylation | C7:Carbamidomethylation:1000.00 | PEAKS DB |
| R.TLC(+57.02)AGILQGGIDTC(+57.02)K.G | Y | 67.45 | 1605.7854 | 15 | 1.4 | 803.9011 | 2 | 13.87 | 7 | F7:4457 | DaRuMP\_F7B.raw |  |  |  |  | 1.3902E6 |  | 5.9583E5 |  |  |  | 2 | 0 | 0 | 0 | 0 | 1 | 0 | 1 | 0 | 0 | 0 | 186 | 200 | Carbamidomethylation | C3:Carbamidomethylation:1000.00;C14:Carbamidomethylation:1000.00 | PEAKS DB |
| K.FFC(+57.02)LSSK.N | N | 65.32 | 887.4211 | 7 | 0.1 | 444.7168 | 2 | 12.11 | 6 | F6:2844 | DaRuMP\_F7A.raw |  |  |  |  | 2.8418E6 | 8.5101E5 | 1.0094E7 |  | 1.4913E5 |  | 4 | 0 | 0 | 0 | 0 | 1 | 1 | 1 | 0 | 1 | 0 | 95 | 101 | Carbamidomethylation | C3:Carbamidomethylation:1000.00 | PEAKS DB |
| L.SLPSSPPSVGSVC(+57.02)R.I | N | 62.46 | 1428.7031 | 14 | -0.8 | 715.3583 | 2 | 11.69 | 7 | F7:2521 | DaRuMP\_F7B.raw |  |  |  |  | 1.8242E6 |  | 1.0733E6 |  | 1.7309E5 |  | 3 | 0 | 0 | 0 | 0 | 1 | 0 | 1 | 0 | 1 | 0 | 129 | 142 | Carbamidomethylation | C13:Carbamidomethylation:1000.00 | PEAKS DB |
| total 4 peptides |
| --- |

B4XSY8|SLAD\_MACLB

back to list

  

| Protein Coverage
| Supporting Peptides
|

Protein Coverage:

Supporting Peptides:

| Peptide | Uniq | -10lgP | Mass | Length | ppm | m/z | z | RT | Fraction | Scan | Source File | Area F1 | Area F10 | Area F2\_3 | Area F5 | Area F6 | Area F7A | Area F7B | Area F8A | Area F8B | Area F9 | #Feature | #Feature F1 | #Feature F10 | #Feature F2\_3 | #Feature F5 | #Feature F6 | #Feature F7A | #Feature F7B | #Feature F8A | #Feature F8B | #Feature F9 | Start | End | PTM | AScore | Found By |
| --- | --- | --- | --- | --- | --- | --- | --- | --- | --- | --- | --- | --- | --- | --- | --- | --- | --- | --- | --- | --- | --- | --- | --- | --- | --- | --- | --- | --- | --- | --- | --- | --- | --- | --- | --- | --- | --- |
| K.HLATIEWLGK.A | Y | 79.31 | 1166.6448 | 10 | 0.2 | 584.3298 | 2 | 16.31 | 8 | F8:3025 | DaRuMP\_F8A.raw |  | 9.7708E4 |  |  |  |  | 5.8456E6 | 2.0386E6 | 4.102E6 | 2.1545E4 | 5 | 0 | 1 | 0 | 0 | 0 | 0 | 1 | 1 | 1 | 1 | 40 | 49 |  |  | PEAKS DB |
| K.ANFVAELVTLMK.L | Y | 78.02 | 1334.7268 | 12 | 0.5 | 668.3710 | 2 | 52.35 | 8 | F8:23233 | DaRuMP\_F8A.raw |  | 7.8797E4 |  |  |  |  | 5.0704E5 | 8.1724E5 | 1.9097E6 |  | 4 | 0 | 1 | 0 | 0 | 0 | 0 | 1 | 1 | 1 | 0 | 50 | 61 |  |  | PEAKS DB |
| L.PGWSFYEGHC(+57.02)YK.V | Y | 60.94 | 1529.6398 | 12 | -0.3 | 510.8871 | 3 | 11.81 | 9 | F9:2658 | DaRuMP\_F8B.raw |  |  |  |  |  |  |  |  | 4.1806E5 |  | 1 | 0 | 0 | 0 | 0 | 0 | 0 | 0 | 0 | 1 | 0 | 6 | 17 | Carbamidomethylation | C10:Carbamidomethylation:1000.00 | PEAKS DB |
| total 3 peptides |
| --- |

B4XSY7|SLAC\_MACLB

back to list

  

| Protein Coverage
| Supporting Peptides
|

Protein Coverage:

Supporting Peptides:

| Peptide | Uniq | -10lgP | Mass | Length | ppm | m/z | z | RT | Fraction | Scan | Source File | Area F1 | Area F10 | Area F2\_3 | Area F5 | Area F6 | Area F7A | Area F7B | Area F8A | Area F8B | Area F9 | #Feature | #Feature F1 | #Feature F10 | #Feature F2\_3 | #Feature F5 | #Feature F6 | #Feature F7A | #Feature F7B | #Feature F8A | #Feature F8B | #Feature F9 | Start | End | PTM | AScore | Found By |
| --- | --- | --- | --- | --- | --- | --- | --- | --- | --- | --- | --- | --- | --- | --- | --- | --- | --- | --- | --- | --- | --- | --- | --- | --- | --- | --- | --- | --- | --- | --- | --- | --- | --- | --- | --- | --- | --- |
| K.HLATIEWLGK.A | Y | 79.31 | 1166.6448 | 10 | 0.2 | 584.3298 | 2 | 16.31 | 8 | F8:3025 | DaRuMP\_F8A.raw |  | 9.7708E4 |  |  |  |  | 5.8456E6 | 2.0386E6 | 4.102E6 | 2.1545E4 | 5 | 0 | 1 | 0 | 0 | 0 | 0 | 1 | 1 | 1 | 1 | 63 | 72 |  |  | PEAKS DB |
| K.ANFVAELVTLMK.L | Y | 78.02 | 1334.7268 | 12 | 0.5 | 668.3710 | 2 | 52.35 | 8 | F8:23233 | DaRuMP\_F8A.raw |  | 7.8797E4 |  |  |  |  | 5.0704E5 | 8.1724E5 | 1.9097E6 |  | 4 | 0 | 1 | 0 | 0 | 0 | 0 | 1 | 1 | 1 | 0 | 73 | 84 |  |  | PEAKS DB |
| L.PGWSFYEGHC(+57.02)YK.V | Y | 60.94 | 1529.6398 | 12 | -0.3 | 510.8871 | 3 | 11.81 | 9 | F9:2658 | DaRuMP\_F8B.raw |  |  |  |  |  |  |  |  | 4.1806E5 |  | 1 | 0 | 0 | 0 | 0 | 0 | 0 | 0 | 0 | 1 | 0 | 29 | 40 | Carbamidomethylation | C10:Carbamidomethylation:1000.00 | PEAKS DB |
| total 3 peptides |
| --- |

Q6IFZ6|K2C1B\_MOUSE

back to list

  

| Protein Coverage
| Supporting Peptides
|

Protein Coverage:

Supporting Peptides:

| Peptide | Uniq | -10lgP | Mass | Length | ppm | m/z | z | RT | Fraction | Scan | Source File | Area F1 | Area F10 | Area F2\_3 | Area F5 | Area F6 | Area F7A | Area F7B | Area F8A | Area F8B | Area F9 | #Feature | #Feature F1 | #Feature F10 | #Feature F2\_3 | #Feature F5 | #Feature F6 | #Feature F7A | #Feature F7B | #Feature F8A | #Feature F8B | #Feature F9 | Start | End | PTM | AScore | Found By |
| --- | --- | --- | --- | --- | --- | --- | --- | --- | --- | --- | --- | --- | --- | --- | --- | --- | --- | --- | --- | --- | --- | --- | --- | --- | --- | --- | --- | --- | --- | --- | --- | --- | --- | --- | --- | --- | --- |
| R.FLEQQNQVLQTK.W | N | 81.01 | 1474.7780 | 12 | 0.8 | 738.3969 | 2 | 11.70 | 3 | F3:2504 | DaRuMP\_F2\_F3.raw | 2.5845E6 | 6.3219E5 | 7.403E5 | 2.7387E6 | 2.4581E6 |  | 6.9952E6 | 1.1298E6 | 1.6497E6 | 4.0389E5 | 9 | 1 | 1 | 1 | 1 | 1 | 0 | 1 | 1 | 1 | 1 | 187 | 198 |  |  | PEAKS DB |
| R.SLDLDSIIDAVR.A | Y | 59.98 | 1315.6982 | 12 | 0.7 | 658.8569 | 2 | 41.70 | 8 | F8:19108 | DaRuMP\_F8A.raw |  |  |  |  |  |  |  | 6.0324E3 |  |  | 1 | 0 | 0 | 0 | 0 | 0 | 0 | 0 | 1 | 0 | 0 | 331 | 342 |  |  | PEAKS DB |
| K.NKYEDEINKR.T | N | 56.45 | 1307.6470 | 10 | 0.1 | 436.8896 | 3 | 10.71 | 10 | F10:1603 | DaRuMP\_F9.raw |  |  |  |  |  |  |  |  | 2.9837E5 | 1.8812E5 | 2 | 0 | 0 | 0 | 0 | 0 | 0 | 0 | 0 | 1 | 1 | 255 | 264 |  |  | PEAKS DB |
| total 3 peptides |
| --- |

P25669|3L22\_NAJNA

back to list

  

| Protein Coverage
| Supporting Peptides
|

Protein Coverage:

Supporting Peptides:

| Peptide | Uniq | -10lgP | Mass | Length | ppm | m/z | z | RT | Fraction | Scan | Source File | Area F1 | Area F10 | Area F2\_3 | Area F5 | Area F6 | Area F7A | Area F7B | Area F8A | Area F8B | Area F9 | #Feature | #Feature F1 | #Feature F10 | #Feature F2\_3 | #Feature F5 | #Feature F6 | #Feature F7A | #Feature F7B | #Feature F8A | #Feature F8B | #Feature F9 | Start | End | PTM | AScore | Found By |
| --- | --- | --- | --- | --- | --- | --- | --- | --- | --- | --- | --- | --- | --- | --- | --- | --- | --- | --- | --- | --- | --- | --- | --- | --- | --- | --- | --- | --- | --- | --- | --- | --- | --- | --- | --- | --- | --- |
| R.VDLGC(+57.02)AATC(+57.02)PTVR.T | Y | 84.25 | 1418.6646 | 13 | 0.5 | 710.3399 | 2 | 11.63 | 3 | F3:2432 | DaRuMP\_F2\_F3.raw | 8.6735E5 |  | 1.2352E5 | 4.235E4 |  |  |  |  |  |  | 3 | 1 | 0 | 1 | 1 | 0 | 0 | 0 | 0 | 0 | 0 | 37 | 49 | Carbamidomethylation | C5:Carbamidomethylation:1000.00;C9:Carbamidomethylation:1000.00 | PEAKS DB |
| R.C(+57.02)FITPDITSK.D | N | 69.93 | 1180.5798 | 10 | 0.3 | 591.2974 | 2 | 12.54 | 7 | F7:3299 | DaRuMP\_F7B.raw | 4.4604E5 |  |  | 1.5063E5 | 8.3527E4 |  | 1.1354E5 | 2.086E4 |  | 2.0013E4 | 6 | 1 | 0 | 0 | 1 | 1 | 0 | 1 | 1 | 0 | 1 | 3 | 12 | Carbamidomethylation | C1:Carbamidomethylation:1000.00 | PEAKS DB |
| K.TWC(+57.02)DGFC(+57.02)SSR.G | Y | 57.08 | 1274.4808 | 10 | 0.2 | 638.2478 | 2 | 11.78 | 3 | F3:2587 | DaRuMP\_F2\_F3.raw | 3.2974E5 |  | 4.6921E4 |  |  |  |  |  |  |  | 2 | 1 | 0 | 1 | 0 | 0 | 0 | 0 | 0 | 0 | 0 | 24 | 33 | Carbamidomethylation | C3:Carbamidomethylation:1000.00;C7:Carbamidomethylation:1000.00 | PEAKS DB |
| total 3 peptides |
| --- |

Q696W1|SLLC2\_MACLB

back to list

  

| Protein Coverage
| Supporting Peptides
|

Protein Coverage:

Supporting Peptides:

| Peptide | Uniq | -10lgP | Mass | Length | ppm | m/z | z | RT | Fraction | Scan | Source File | Area F1 | Area F10 | Area F2\_3 | Area F5 | Area F6 | Area F7A | Area F7B | Area F8A | Area F8B | Area F9 | #Feature | #Feature F1 | #Feature F10 | #Feature F2\_3 | #Feature F5 | #Feature F6 | #Feature F7A | #Feature F7B | #Feature F8A | #Feature F8B | #Feature F9 | Start | End | PTM | AScore | Found By |
| --- | --- | --- | --- | --- | --- | --- | --- | --- | --- | --- | --- | --- | --- | --- | --- | --- | --- | --- | --- | --- | --- | --- | --- | --- | --- | --- | --- | --- | --- | --- | --- | --- | --- | --- | --- | --- | --- |
| G.LDC(+57.02)PPDSSPYR.Y | Y | 73.95 | 1305.5659 | 11 | 0.4 | 653.7905 | 2 | 11.31 | 9 | F9:2132 | DaRuMP\_F8B.raw |  |  |  |  |  |  | 1.0045E6 |  | 2.8053E6 |  | 2 | 0 | 0 | 0 | 0 | 0 | 0 | 1 | 0 | 1 | 0 | 25 | 35 | Carbamidomethylation | C3:Carbamidomethylation:1000.00 | PEAKS DB |
| K.FITHFWIGLR.I | Y | 66.53 | 1288.7080 | 10 | -0.5 | 430.5764 | 3 | 21.65 | 9 | F9:8131 | DaRuMP\_F8B.raw |  |  |  |  |  |  | 2.3713E6 |  | 6.9793E5 |  | 3 | 0 | 0 | 0 | 0 | 0 | 0 | 1 | 0 | 2 | 0 | 87 | 96 |  |  | PEAKS DB |
| N.C(+57.02)EEPYPFVC(+57.02)K.V | Y | 64.10 | 1327.5576 | 10 | 0.8 | 664.7866 | 2 | 12.12 | 9 | F9:2913 | DaRuMP\_F8B.raw |  |  |  |  |  |  | 2.2837E7 | 1.0407E6 | 3.6404E6 | 7.033E4 | 4 | 0 | 0 | 0 | 0 | 0 | 0 | 1 | 1 | 1 | 1 | 144 | 153 | Carbamidomethylation | C1:Carbamidomethylation:1000.00;C9:Carbamidomethylation:1000.00 | PEAKS DB |
| total 3 peptides |
| --- |

P02845|VIT2\_CHICK

back to list

  

| Protein Coverage
| Supporting Peptides
|

Protein Coverage:

Supporting Peptides:

| Peptide | Uniq | -10lgP | Mass | Length | ppm | m/z | z | RT | Fraction | Scan | Source File | Area F1 | Area F10 | Area F2\_3 | Area F5 | Area F6 | Area F7A | Area F7B | Area F8A | Area F8B | Area F9 | #Feature | #Feature F1 | #Feature F10 | #Feature F2\_3 | #Feature F5 | #Feature F6 | #Feature F7A | #Feature F7B | #Feature F8A | #Feature F8B | #Feature F9 | Start | End | PTM | AScore | Found By |
| --- | --- | --- | --- | --- | --- | --- | --- | --- | --- | --- | --- | --- | --- | --- | --- | --- | --- | --- | --- | --- | --- | --- | --- | --- | --- | --- | --- | --- | --- | --- | --- | --- | --- | --- | --- | --- | --- |
| R.LSQLLESTM(+15.99)QIR.S | Y | 58.19 | 1433.7548 | 12 | -1.9 | 717.8833 | 2 | 12.20 | 5 | F5:3038 | DaRuMP\_F6.raw |  |  |  |  | 3.144E4 |  |  |  |  |  | 1 | 0 | 0 | 0 | 0 | 1 | 0 | 0 | 0 | 0 | 0 | 831 | 842 | Oxidation (M) | M9:Oxidation (M):1000.00 | PEAKS DB |
| R.VGATGEIFVVNSPR.T | Y | 52.90 | 1444.7673 | 14 | 0.6 | 723.3914 | 2 | 13.44 | 5 | F5:4145 | DaRuMP\_F6.raw |  |  |  |  | 3.9105E4 |  |  |  |  |  | 1 | 0 | 0 | 0 | 0 | 1 | 0 | 0 | 0 | 0 | 0 | 642 | 655 |  |  | PEAKS DB |
| total 2 peptides |
| --- |

P01391|3L21\_NAJKA

back to list

  

| Protein Coverage
| Supporting Peptides
|

Protein Coverage:

Supporting Peptides:

| Peptide | Uniq | -10lgP | Mass | Length | ppm | m/z | z | RT | Fraction | Scan | Source File | Area F1 | Area F10 | Area F2\_3 | Area F5 | Area F6 | Area F7A | Area F7B | Area F8A | Area F8B | Area F9 | #Feature | #Feature F1 | #Feature F10 | #Feature F2\_3 | #Feature F5 | #Feature F6 | #Feature F7A | #Feature F7B | #Feature F8A | #Feature F8B | #Feature F9 | Start | End | PTM | AScore | Found By |
| --- | --- | --- | --- | --- | --- | --- | --- | --- | --- | --- | --- | --- | --- | --- | --- | --- | --- | --- | --- | --- | --- | --- | --- | --- | --- | --- | --- | --- | --- | --- | --- | --- | --- | --- | --- | --- | --- |
| K.TGVDIQC(+57.02)C(+57.02)STDNC(+57.02)NPFPTR.K | Y | 71.90 | 2240.9248 | 19 | -0.1 | 1121.4696 | 2 | 12.07 | 1 | F1:2881 | DaRuMP\_F1.raw | 1.3021E6 |  | 1.8401E5 | 1.2306E5 | 1.0165E5 |  |  |  |  |  | 4 | 1 | 0 | 1 | 1 | 1 | 0 | 0 | 0 | 0 | 0 | 50 | 68 | Carbamidomethylation | C7:Carbamidomethylation:1000.00;C8:Carbamidomethylation:1000.00;C13:Carbamidomethylation:1000.00 | PEAKS DB |
| R.C(+57.02)FITPDITSK.D | N | 69.93 | 1180.5798 | 10 | 0.3 | 591.2974 | 2 | 12.54 | 7 | F7:3299 | DaRuMP\_F7B.raw | 4.4604E5 |  |  | 1.5063E5 | 8.3527E4 |  | 1.1354E5 | 2.086E4 |  | 2.0013E4 | 6 | 1 | 0 | 0 | 1 | 1 | 0 | 1 | 1 | 0 | 1 | 3 | 12 | Carbamidomethylation | C1:Carbamidomethylation:1000.00 | PEAKS DB |
| total 2 peptides |
| --- |

A8Y7P1|VKTB1\_DABSI

back to list

  

| Protein Coverage
| Supporting Peptides
|

Protein Coverage:

Supporting Peptides:

| Peptide | Uniq | -10lgP | Mass | Length | ppm | m/z | z | RT | Fraction | Scan | Source File | Area F1 | Area F10 | Area F2\_3 | Area F5 | Area F6 | Area F7A | Area F7B | Area F8A | Area F8B | Area F9 | #Feature | #Feature F1 | #Feature F10 | #Feature F2\_3 | #Feature F5 | #Feature F6 | #Feature F7A | #Feature F7B | #Feature F8A | #Feature F8B | #Feature F9 | Start | End | PTM | AScore | Found By |
| --- | --- | --- | --- | --- | --- | --- | --- | --- | --- | --- | --- | --- | --- | --- | --- | --- | --- | --- | --- | --- | --- | --- | --- | --- | --- | --- | --- | --- | --- | --- | --- | --- | --- | --- | --- | --- | --- |
| R.SFYYDSESKK.C | N | 58.75 | 1252.5612 | 10 | 0.0 | 418.5276 | 3 | 11.02 | 1 | F1:1914 | DaRuMP\_F1.raw | 3.2206E4 |  |  |  |  |  |  |  |  |  | 1 | 1 | 0 | 0 | 0 | 0 | 0 | 0 | 0 | 0 | 0 | 46 | 55 |  |  | PEAKS DB |
| R.SFYYDSESK.K | N | 56.16 | 1124.4662 | 9 | 0.4 | 563.2406 | 2 | 11.48 | 1 | F1:2335 | DaRuMP\_F1.raw | 9.2083E5 |  |  |  |  |  |  |  |  |  | 1 | 1 | 0 | 0 | 0 | 0 | 0 | 0 | 0 | 0 | 0 | 46 | 54 |  |  | PEAKS DB |
| K.FC(+57.02)YLPADPGEC(+57.02)LAHM(+15.99)R.S | Y | 53.02 | 1951.8379 | 16 | -0.1 | 651.6199 | 3 | 12.35 | 1 | F1:3142 | DaRuMP\_F1.raw | 4.1392E5 |  |  |  |  |  |  |  |  |  | 1 | 1 | 0 | 0 | 0 | 0 | 0 | 0 | 0 | 0 | 0 | 30 | 45 | Carbamidomethylation; Oxidation (M) | C2:Carbamidomethylation:1000.00;C11:Carbamidomethylation:1000.00;M15:Oxidation (M):1000.00 | PEAKS DB |
| total 3 peptides |
| --- |

A8Y7P5|VKTB5\_DABSI

back to list

  

| Protein Coverage
| Supporting Peptides
|

Protein Coverage:

Supporting Peptides:

| Peptide | Uniq | -10lgP | Mass | Length | ppm | m/z | z | RT | Fraction | Scan | Source File | Area F1 | Area F10 | Area F2\_3 | Area F5 | Area F6 | Area F7A | Area F7B | Area F8A | Area F8B | Area F9 | #Feature | #Feature F1 | #Feature F10 | #Feature F2\_3 | #Feature F5 | #Feature F6 | #Feature F7A | #Feature F7B | #Feature F8A | #Feature F8B | #Feature F9 | Start | End | PTM | AScore | Found By |
| --- | --- | --- | --- | --- | --- | --- | --- | --- | --- | --- | --- | --- | --- | --- | --- | --- | --- | --- | --- | --- | --- | --- | --- | --- | --- | --- | --- | --- | --- | --- | --- | --- | --- | --- | --- | --- | --- |
| R.SFYYDSESKK.C | N | 58.75 | 1252.5612 | 10 | 0.0 | 418.5276 | 3 | 11.02 | 1 | F1:1914 | DaRuMP\_F1.raw | 3.2206E4 |  |  |  |  |  |  |  |  |  | 1 | 1 | 0 | 0 | 0 | 0 | 0 | 0 | 0 | 0 | 0 | 46 | 55 |  |  | PEAKS DB |
| R.SFYYDSESK.K | N | 56.16 | 1124.4662 | 9 | 0.4 | 563.2406 | 2 | 11.48 | 1 | F1:2335 | DaRuMP\_F1.raw | 9.2083E5 |  |  |  |  |  |  |  |  |  | 1 | 1 | 0 | 0 | 0 | 0 | 0 | 0 | 0 | 0 | 0 | 46 | 54 |  |  | PEAKS DB |
| K.FC(+57.02)YLPADPGEC(+57.02)LAHM(+15.99)R.S | Y | 53.02 | 1951.8379 | 16 | -0.1 | 651.6199 | 3 | 12.35 | 1 | F1:3142 | DaRuMP\_F1.raw | 4.1392E5 |  |  |  |  |  |  |  |  |  | 1 | 1 | 0 | 0 | 0 | 0 | 0 | 0 | 0 | 0 | 0 | 30 | 45 | Carbamidomethylation; Oxidation (M) | C2:Carbamidomethylation:1000.00;C11:Carbamidomethylation:1000.00;M15:Oxidation (M):1000.00 | PEAKS DB |
| total 3 peptides |
| --- |

P86530|VSP1\_DABRR

back to list

  

| Protein Coverage
| Supporting Peptides
|

Protein Coverage:

Supporting Peptides:

| Peptide | Uniq | -10lgP | Mass | Length | ppm | m/z | z | RT | Fraction | Scan | Source File | Area F1 | Area F10 | Area F2\_3 | Area F5 | Area F6 | Area F7A | Area F7B | Area F8A | Area F8B | Area F9 | #Feature | #Feature F1 | #Feature F10 | #Feature F2\_3 | #Feature F5 | #Feature F6 | #Feature F7A | #Feature F7B | #Feature F8A | #Feature F8B | #Feature F9 | Start | End | PTM | AScore | Found By |
| --- | --- | --- | --- | --- | --- | --- | --- | --- | --- | --- | --- | --- | --- | --- | --- | --- | --- | --- | --- | --- | --- | --- | --- | --- | --- | --- | --- | --- | --- | --- | --- | --- | --- | --- | --- | --- | --- |
| VIGGDEC(+57.02)NINEHPF.L | N | 78.07 | 1599.6987 | 14 | -1.0 | 800.8558 | 2 | 12.19 | 7 | F7:2994 | DaRuMP\_F7B.raw |  |  |  |  |  |  | 1.6317E6 |  |  |  | 1 | 0 | 0 | 0 | 0 | 0 | 0 | 1 | 0 | 0 | 0 | 1 | 14 | Carbamidomethylation | C7:Carbamidomethylation:1000.00 | PEAKS DB |
| VIGGDEC(+57.02)NINEHPFL.A | N | 59.49 | 1712.7828 | 15 | 0.3 | 857.3989 | 2 | 16.34 | 7 | F7:6526 | DaRuMP\_F7B.raw |  |  |  |  |  |  | 6.2556E5 |  |  |  | 1 | 0 | 0 | 0 | 0 | 0 | 0 | 1 | 0 | 0 | 0 | 1 | 15 | Carbamidomethylation | C7:Carbamidomethylation:1000.00 | PEAKS DB |
| VIGGDEC(+57.02)NINEHPFLA | Y | 57.43 | 1783.8199 | 16 | 0.3 | 892.9175 | 2 | 15.41 | 7 | F7:5746 | DaRuMP\_F7B.raw |  |  |  |  |  |  | 2.2404E5 |  |  |  | 1 | 0 | 0 | 0 | 0 | 0 | 0 | 1 | 0 | 0 | 0 | 1 | 16 | Carbamidomethylation | C7:Carbamidomethylation:1000.00 | PEAKS DB |
| total 3 peptides |
| --- |

A8Y7N6|VKTC3\_DABSI

back to list

  

| Protein Coverage
| Supporting Peptides
|

Protein Coverage:

Supporting Peptides:

| Peptide | Uniq | -10lgP | Mass | Length | ppm | m/z | z | RT | Fraction | Scan | Source File | Area F1 | Area F10 | Area F2\_3 | Area F5 | Area F6 | Area F7A | Area F7B | Area F8A | Area F8B | Area F9 | #Feature | #Feature F1 | #Feature F10 | #Feature F2\_3 | #Feature F5 | #Feature F6 | #Feature F7A | #Feature F7B | #Feature F8A | #Feature F8B | #Feature F9 | Start | End | PTM | AScore | Found By |
| --- | --- | --- | --- | --- | --- | --- | --- | --- | --- | --- | --- | --- | --- | --- | --- | --- | --- | --- | --- | --- | --- | --- | --- | --- | --- | --- | --- | --- | --- | --- | --- | --- | --- | --- | --- | --- | --- |
| K.EFIYGGC(+57.02)HGNANNFPTR.D | Y | 62.57 | 1952.8588 | 17 | 0.4 | 651.9604 | 3 | 11.63 | 1 | F1:2470 | DaRuMP\_F1.raw | 1.8749E6 |  | 6.7275E4 |  |  |  |  |  |  |  | 3 | 2 | 0 | 1 | 0 | 0 | 0 | 0 | 0 | 0 | 0 | 58 | 74 | Carbamidomethylation | C7:Carbamidomethylation:1000.00 | PEAKS DB |
| R.SFYYDSESKK.C | N | 58.75 | 1252.5612 | 10 | 0.0 | 418.5276 | 3 | 11.02 | 1 | F1:1914 | DaRuMP\_F1.raw | 3.2206E4 |  |  |  |  |  |  |  |  |  | 1 | 1 | 0 | 0 | 0 | 0 | 0 | 0 | 0 | 0 | 0 | 46 | 55 |  |  | PEAKS DB |
| R.SFYYDSESK.K | N | 56.16 | 1124.4662 | 9 | 0.4 | 563.2406 | 2 | 11.48 | 1 | F1:2335 | DaRuMP\_F1.raw | 9.2083E5 |  |  |  |  |  |  |  |  |  | 1 | 1 | 0 | 0 | 0 | 0 | 0 | 0 | 0 | 0 | 0 | 46 | 54 |  |  | PEAKS DB |
| total 3 peptides |
| --- |

A8Y7N7|VKTC4\_DABSI

back to list

  

| Protein Coverage
| Supporting Peptides
|

Protein Coverage:

Supporting Peptides:

| Peptide | Uniq | -10lgP | Mass | Length | ppm | m/z | z | RT | Fraction | Scan | Source File | Area F1 | Area F10 | Area F2\_3 | Area F5 | Area F6 | Area F7A | Area F7B | Area F8A | Area F8B | Area F9 | #Feature | #Feature F1 | #Feature F10 | #Feature F2\_3 | #Feature F5 | #Feature F6 | #Feature F7A | #Feature F7B | #Feature F8A | #Feature F8B | #Feature F9 | Start | End | PTM | AScore | Found By |
| --- | --- | --- | --- | --- | --- | --- | --- | --- | --- | --- | --- | --- | --- | --- | --- | --- | --- | --- | --- | --- | --- | --- | --- | --- | --- | --- | --- | --- | --- | --- | --- | --- | --- | --- | --- | --- | --- |
| K.EFIYGGC(+57.02)HGNANNFPTR.D | Y | 62.57 | 1952.8588 | 17 | 0.4 | 651.9604 | 3 | 11.63 | 1 | F1:2470 | DaRuMP\_F1.raw | 1.8749E6 |  | 6.7275E4 |  |  |  |  |  |  |  | 3 | 2 | 0 | 1 | 0 | 0 | 0 | 0 | 0 | 0 | 0 | 58 | 74 | Carbamidomethylation | C7:Carbamidomethylation:1000.00 | PEAKS DB |
| R.SFYYDSESKK.C | N | 58.75 | 1252.5612 | 10 | 0.0 | 418.5276 | 3 | 11.02 | 1 | F1:1914 | DaRuMP\_F1.raw | 3.2206E4 |  |  |  |  |  |  |  |  |  | 1 | 1 | 0 | 0 | 0 | 0 | 0 | 0 | 0 | 0 | 0 | 46 | 55 |  |  | PEAKS DB |
| R.SFYYDSESK.K | N | 56.16 | 1124.4662 | 9 | 0.4 | 563.2406 | 2 | 11.48 | 1 | F1:2335 | DaRuMP\_F1.raw | 9.2083E5 |  |  |  |  |  |  |  |  |  | 1 | 1 | 0 | 0 | 0 | 0 | 0 | 0 | 0 | 0 | 0 | 46 | 54 |  |  | PEAKS DB |
| total 3 peptides |
| --- |

P00761|TRYP\_PIG

back to list

  

| Protein Coverage
| Supporting Peptides
|

Protein Coverage:

Supporting Peptides:

| Peptide | Uniq | -10lgP | Mass | Length | ppm | m/z | z | RT | Fraction | Scan | Source File | Area F1 | Area F10 | Area F2\_3 | Area F5 | Area F6 | Area F7A | Area F7B | Area F8A | Area F8B | Area F9 | #Feature | #Feature F1 | #Feature F10 | #Feature F2\_3 | #Feature F5 | #Feature F6 | #Feature F7A | #Feature F7B | #Feature F8A | #Feature F8B | #Feature F9 | Start | End | PTM | AScore | Found By |
| --- | --- | --- | --- | --- | --- | --- | --- | --- | --- | --- | --- | --- | --- | --- | --- | --- | --- | --- | --- | --- | --- | --- | --- | --- | --- | --- | --- | --- | --- | --- | --- | --- | --- | --- | --- | --- | --- |
| K.LSSPATLNSR.V | Y | 53.78 | 1044.5564 | 10 | 0.6 | 523.2858 | 2 | 11.22 | 9 | F9:2092 | DaRuMP\_F8B.raw |  |  |  |  |  |  |  |  | 1.0145E5 | 1.5066E5 | 2 | 0 | 0 | 0 | 0 | 0 | 0 | 0 | 0 | 1 | 1 | 98 | 107 |  |  | PEAKS DB |
| total 1 peptides |
| --- |

E0Y420|VSP3\_MACLB

back to list

  

| Protein Coverage
| Supporting Peptides
|

Protein Coverage:

Supporting Peptides:

| Peptide | Uniq | -10lgP | Mass | Length | ppm | m/z | z | RT | Fraction | Scan | Source File | Area F1 | Area F10 | Area F2\_3 | Area F5 | Area F6 | Area F7A | Area F7B | Area F8A | Area F8B | Area F9 | #Feature | #Feature F1 | #Feature F10 | #Feature F2\_3 | #Feature F5 | #Feature F6 | #Feature F7A | #Feature F7B | #Feature F8A | #Feature F8B | #Feature F9 | Start | End | PTM | AScore | Found By |
| --- | --- | --- | --- | --- | --- | --- | --- | --- | --- | --- | --- | --- | --- | --- | --- | --- | --- | --- | --- | --- | --- | --- | --- | --- | --- | --- | --- | --- | --- | --- | --- | --- | --- | --- | --- | --- | --- |
| K.FFC(+57.02)LSSK.N | N | 65.32 | 887.4211 | 7 | 0.1 | 444.7168 | 2 | 12.11 | 6 | F6:2844 | DaRuMP\_F7A.raw |  |  |  |  | 2.8418E6 | 8.5101E5 | 1.0094E7 |  | 1.4913E5 |  | 4 | 0 | 0 | 0 | 0 | 1 | 1 | 1 | 0 | 1 | 0 | 96 | 102 | Carbamidomethylation | C3:Carbamidomethylation:1000.00 | PEAKS DB |
| L.SLPSSPPSVGSVC(+57.02)R.I | N | 62.46 | 1428.7031 | 14 | -0.8 | 715.3583 | 2 | 11.69 | 7 | F7:2521 | DaRuMP\_F7B.raw |  |  |  |  | 1.8242E6 |  | 1.0733E6 |  | 1.7309E5 |  | 3 | 0 | 0 | 0 | 0 | 1 | 0 | 1 | 0 | 1 | 0 | 130 | 143 | Carbamidomethylation | C13:Carbamidomethylation:1000.00 | PEAKS DB |
| K.TSTHIAPLSLPSSPPSVGSVC(+57.02)R.I | Y | 62.01 | 2249.1475 | 22 | -0.2 | 750.7230 | 3 | 13.11 | 7 | F7:3783 | DaRuMP\_F7B.raw |  |  |  |  | 1.0232E6 |  | 5.6989E5 |  |  |  | 2 | 0 | 0 | 0 | 0 | 1 | 0 | 1 | 0 | 0 | 0 | 122 | 143 | Carbamidomethylation | C21:Carbamidomethylation:1000.00 | PEAKS DB |
| total 3 peptides |
| --- |

A7ISW1|QPCT\_BOIIR

back to list

  

| Protein Coverage
| Supporting Peptides
|

Protein Coverage:

Supporting Peptides:

| Peptide | Uniq | -10lgP | Mass | Length | ppm | m/z | z | RT | Fraction | Scan | Source File | Area F1 | Area F10 | Area F2\_3 | Area F5 | Area F6 | Area F7A | Area F7B | Area F8A | Area F8B | Area F9 | #Feature | #Feature F1 | #Feature F10 | #Feature F2\_3 | #Feature F5 | #Feature F6 | #Feature F7A | #Feature F7B | #Feature F8A | #Feature F8B | #Feature F9 | Start | End | PTM | AScore | Found By |
| --- | --- | --- | --- | --- | --- | --- | --- | --- | --- | --- | --- | --- | --- | --- | --- | --- | --- | --- | --- | --- | --- | --- | --- | --- | --- | --- | --- | --- | --- | --- | --- | --- | --- | --- | --- | --- | --- |
| R.TFSNIISTLNPLAK.R | Y | 68.95 | 1517.8453 | 14 | 0.5 | 759.9304 | 2 | 35.00 | 2 | F2:10264 | DaRuMP\_F10.raw |  | 2.9141E5 |  |  |  |  |  |  |  |  | 1 | 0 | 1 | 0 | 0 | 0 | 0 | 0 | 0 | 0 | 0 | 123 | 136 |  |  | PEAKS DB |
| total 1 peptides |
| --- |

Q90YA8|QPCT\_GLOBL

back to list

  

| Protein Coverage
| Supporting Peptides
|

Protein Coverage:

Supporting Peptides:

| Peptide | Uniq | -10lgP | Mass | Length | ppm | m/z | z | RT | Fraction | Scan | Source File | Area F1 | Area F10 | Area F2\_3 | Area F5 | Area F6 | Area F7A | Area F7B | Area F8A | Area F8B | Area F9 | #Feature | #Feature F1 | #Feature F10 | #Feature F2\_3 | #Feature F5 | #Feature F6 | #Feature F7A | #Feature F7B | #Feature F8A | #Feature F8B | #Feature F9 | Start | End | PTM | AScore | Found By |
| --- | --- | --- | --- | --- | --- | --- | --- | --- | --- | --- | --- | --- | --- | --- | --- | --- | --- | --- | --- | --- | --- | --- | --- | --- | --- | --- | --- | --- | --- | --- | --- | --- | --- | --- | --- | --- | --- |
| R.TFSNIISTLNPLAK.R | Y | 68.95 | 1517.8453 | 14 | 0.5 | 759.9304 | 2 | 35.00 | 2 | F2:10264 | DaRuMP\_F10.raw |  | 2.9141E5 |  |  |  |  |  |  |  |  | 1 | 0 | 1 | 0 | 0 | 0 | 0 | 0 | 0 | 0 | 0 | 123 | 136 |  |  | PEAKS DB |
| total 1 peptides |
| --- |

A7ISW2|QPCT\_BOIDE

back to list

  

| Protein Coverage
| Supporting Peptides
|

Protein Coverage:

Supporting Peptides:

| Peptide | Uniq | -10lgP | Mass | Length | ppm | m/z | z | RT | Fraction | Scan | Source File | Area F1 | Area F10 | Area F2\_3 | Area F5 | Area F6 | Area F7A | Area F7B | Area F8A | Area F8B | Area F9 | #Feature | #Feature F1 | #Feature F10 | #Feature F2\_3 | #Feature F5 | #Feature F6 | #Feature F7A | #Feature F7B | #Feature F8A | #Feature F8B | #Feature F9 | Start | End | PTM | AScore | Found By |
| --- | --- | --- | --- | --- | --- | --- | --- | --- | --- | --- | --- | --- | --- | --- | --- | --- | --- | --- | --- | --- | --- | --- | --- | --- | --- | --- | --- | --- | --- | --- | --- | --- | --- | --- | --- | --- | --- |
| R.TFSNIISTLNPLAK.R | Y | 68.95 | 1517.8453 | 14 | 0.5 | 759.9304 | 2 | 35.00 | 2 | F2:10264 | DaRuMP\_F10.raw |  | 2.9141E5 |  |  |  |  |  |  |  |  | 1 | 0 | 1 | 0 | 0 | 0 | 0 | 0 | 0 | 0 | 0 | 123 | 136 |  |  | PEAKS DB |
| total 1 peptides |
| --- |

Q9YIB5|QPCT\_BOTJA

back to list

  

| Protein Coverage
| Supporting Peptides
|

Protein Coverage:

Supporting Peptides:

| Peptide | Uniq | -10lgP | Mass | Length | ppm | m/z | z | RT | Fraction | Scan | Source File | Area F1 | Area F10 | Area F2\_3 | Area F5 | Area F6 | Area F7A | Area F7B | Area F8A | Area F8B | Area F9 | #Feature | #Feature F1 | #Feature F10 | #Feature F2\_3 | #Feature F5 | #Feature F6 | #Feature F7A | #Feature F7B | #Feature F8A | #Feature F8B | #Feature F9 | Start | End | PTM | AScore | Found By |
| --- | --- | --- | --- | --- | --- | --- | --- | --- | --- | --- | --- | --- | --- | --- | --- | --- | --- | --- | --- | --- | --- | --- | --- | --- | --- | --- | --- | --- | --- | --- | --- | --- | --- | --- | --- | --- | --- |
| R.TFSNIISTLNPLAK.R | Y | 68.95 | 1517.8453 | 14 | 0.5 | 759.9304 | 2 | 35.00 | 2 | F2:10264 | DaRuMP\_F10.raw |  | 2.9141E5 |  |  |  |  |  |  |  |  | 1 | 0 | 1 | 0 | 0 | 0 | 0 | 0 | 0 | 0 | 0 | 123 | 136 |  |  | PEAKS DB |
| total 1 peptides |
| --- |

B4XSY5|SLAA\_MACLB

back to list

  

| Protein Coverage
| Supporting Peptides
|

Protein Coverage:

Supporting Peptides:

| Peptide | Uniq | -10lgP | Mass | Length | ppm | m/z | z | RT | Fraction | Scan | Source File | Area F1 | Area F10 | Area F2\_3 | Area F5 | Area F6 | Area F7A | Area F7B | Area F8A | Area F8B | Area F9 | #Feature | #Feature F1 | #Feature F10 | #Feature F2\_3 | #Feature F5 | #Feature F6 | #Feature F7A | #Feature F7B | #Feature F8A | #Feature F8B | #Feature F9 | Start | End | PTM | AScore | Found By |
| --- | --- | --- | --- | --- | --- | --- | --- | --- | --- | --- | --- | --- | --- | --- | --- | --- | --- | --- | --- | --- | --- | --- | --- | --- | --- | --- | --- | --- | --- | --- | --- | --- | --- | --- | --- | --- | --- |
| W.SAYDQHC(+57.02)YQAVDEPK.S | Y | 71.94 | 1809.7628 | 15 | -0.1 | 604.2615 | 3 | 11.10 | 9 | F9:1968 | DaRuMP\_F8B.raw |  |  |  |  |  |  |  |  | 9.9733E4 |  | 1 | 0 | 0 | 0 | 0 | 0 | 0 | 0 | 0 | 1 | 0 | 32 | 46 | Carbamidomethylation | C7:Carbamidomethylation:1000.00 | PEAKS DB |
| H.C(+57.02)YQAVDEPK.S | Y | 65.57 | 1108.4858 | 9 | -0.6 | 555.2499 | 2 | 10.92 | 9 | F9:1802 | DaRuMP\_F8B.raw |  |  |  |  |  |  |  |  | 1.3002E5 |  | 1 | 0 | 0 | 0 | 0 | 0 | 0 | 0 | 0 | 1 | 0 | 38 | 46 | Carbamidomethylation | C1:Carbamidomethylation:1000.00 | PEAKS DB |
| total 2 peptides |
| --- |

B4XSY6|SLAB\_MACLB

back to list

  

| Protein Coverage
| Supporting Peptides
|

Protein Coverage:

Supporting Peptides:

| Peptide | Uniq | -10lgP | Mass | Length | ppm | m/z | z | RT | Fraction | Scan | Source File | Area F1 | Area F10 | Area F2\_3 | Area F5 | Area F6 | Area F7A | Area F7B | Area F8A | Area F8B | Area F9 | #Feature | #Feature F1 | #Feature F10 | #Feature F2\_3 | #Feature F5 | #Feature F6 | #Feature F7A | #Feature F7B | #Feature F8A | #Feature F8B | #Feature F9 | Start | End | PTM | AScore | Found By |
| --- | --- | --- | --- | --- | --- | --- | --- | --- | --- | --- | --- | --- | --- | --- | --- | --- | --- | --- | --- | --- | --- | --- | --- | --- | --- | --- | --- | --- | --- | --- | --- | --- | --- | --- | --- | --- | --- |
| W.SAYDQHC(+57.02)YQAVDEPK.S | Y | 71.94 | 1809.7628 | 15 | -0.1 | 604.2615 | 3 | 11.10 | 9 | F9:1968 | DaRuMP\_F8B.raw |  |  |  |  |  |  |  |  | 9.9733E4 |  | 1 | 0 | 0 | 0 | 0 | 0 | 0 | 0 | 0 | 1 | 0 | 32 | 46 | Carbamidomethylation | C7:Carbamidomethylation:1000.00 | PEAKS DB |
| H.C(+57.02)YQAVDEPK.S | Y | 65.57 | 1108.4858 | 9 | -0.6 | 555.2499 | 2 | 10.92 | 9 | F9:1802 | DaRuMP\_F8B.raw |  |  |  |  |  |  |  |  | 1.3002E5 |  | 1 | 0 | 0 | 0 | 0 | 0 | 0 | 0 | 0 | 1 | 0 | 38 | 46 | Carbamidomethylation | C1:Carbamidomethylation:1000.00 | PEAKS DB |
| total 2 peptides |
| --- |

P81605|DCD\_HUMAN

back to list

  

| Protein Coverage
| Supporting Peptides
|

Protein Coverage:

Supporting Peptides:

| Peptide | Uniq | -10lgP | Mass | Length | ppm | m/z | z | RT | Fraction | Scan | Source File | Area F1 | Area F10 | Area F2\_3 | Area F5 | Area F6 | Area F7A | Area F7B | Area F8A | Area F8B | Area F9 | #Feature | #Feature F1 | #Feature F10 | #Feature F2\_3 | #Feature F5 | #Feature F6 | #Feature F7A | #Feature F7B | #Feature F8A | #Feature F8B | #Feature F9 | Start | End | PTM | AScore | Found By |
| --- | --- | --- | --- | --- | --- | --- | --- | --- | --- | --- | --- | --- | --- | --- | --- | --- | --- | --- | --- | --- | --- | --- | --- | --- | --- | --- | --- | --- | --- | --- | --- | --- | --- | --- | --- | --- | --- |
| K.ENAGEDPGLAR.Q | Y | 59.41 | 1127.5206 | 11 | 0.7 | 564.7680 | 2 | 10.92 | 3 | F3:1748 | DaRuMP\_F2\_F3.raw |  | 1.0373E4 | 2.099E4 |  |  |  |  |  |  |  | 2 | 0 | 1 | 1 | 0 | 0 | 0 | 0 | 0 | 0 | 0 | 43 | 53 |  |  | PEAKS DB |
| total 1 peptides |
| --- |

P10981|ACT5\_DROME

back to list

  

| Protein Coverage
| Supporting Peptides
|

Protein Coverage:

Supporting Peptides:

| Peptide | Uniq | -10lgP | Mass | Length | ppm | m/z | z | RT | Fraction | Scan | Source File | Area F1 | Area F10 | Area F2\_3 | Area F5 | Area F6 | Area F7A | Area F7B | Area F8A | Area F8B | Area F9 | #Feature | #Feature F1 | #Feature F10 | #Feature F2\_3 | #Feature F5 | #Feature F6 | #Feature F7A | #Feature F7B | #Feature F8A | #Feature F8B | #Feature F9 | Start | End | PTM | AScore | Found By |
| --- | --- | --- | --- | --- | --- | --- | --- | --- | --- | --- | --- | --- | --- | --- | --- | --- | --- | --- | --- | --- | --- | --- | --- | --- | --- | --- | --- | --- | --- | --- | --- | --- | --- | --- | --- | --- | --- |
| R.VAPEEHPVLLTEAPLNPK.A | Y | 58.65 | 1953.0570 | 18 | 0.5 | 652.0266 | 3 | 12.27 | 9 | F9:3064 | DaRuMP\_F8B.raw |  |  |  |  |  |  |  |  | 1.7091E5 |  | 1 | 0 | 0 | 0 | 0 | 0 | 0 | 0 | 0 | 1 | 0 | 97 | 114 |  |  | PEAKS DB |
| K.EITALAPSTIK.I | Y | 53.96 | 1142.6547 | 11 | -0.4 | 572.3344 | 2 | 12.23 | 9 | F9:3034 | DaRuMP\_F8B.raw |  |  |  |  |  |  |  |  | 7.5662E4 |  | 1 | 0 | 0 | 0 | 0 | 0 | 0 | 0 | 0 | 1 | 0 | 317 | 327 |  |  | PEAKS DB |
| total 2 peptides |
| --- |

P45886|ACT3\_BACDO

back to list

  

| Protein Coverage
| Supporting Peptides
|

Protein Coverage:

Supporting Peptides:

| Peptide | Uniq | -10lgP | Mass | Length | ppm | m/z | z | RT | Fraction | Scan | Source File | Area F1 | Area F10 | Area F2\_3 | Area F5 | Area F6 | Area F7A | Area F7B | Area F8A | Area F8B | Area F9 | #Feature | #Feature F1 | #Feature F10 | #Feature F2\_3 | #Feature F5 | #Feature F6 | #Feature F7A | #Feature F7B | #Feature F8A | #Feature F8B | #Feature F9 | Start | End | PTM | AScore | Found By |
| --- | --- | --- | --- | --- | --- | --- | --- | --- | --- | --- | --- | --- | --- | --- | --- | --- | --- | --- | --- | --- | --- | --- | --- | --- | --- | --- | --- | --- | --- | --- | --- | --- | --- | --- | --- | --- | --- |
| R.VAPEEHPVLLTEAPLNPK.A | Y | 58.65 | 1953.0570 | 18 | 0.5 | 652.0266 | 3 | 12.27 | 9 | F9:3064 | DaRuMP\_F8B.raw |  |  |  |  |  |  |  |  | 1.7091E5 |  | 1 | 0 | 0 | 0 | 0 | 0 | 0 | 0 | 0 | 1 | 0 | 97 | 114 |  |  | PEAKS DB |
| K.EITALAPSTIK.I | Y | 53.96 | 1142.6547 | 11 | -0.4 | 572.3344 | 2 | 12.23 | 9 | F9:3034 | DaRuMP\_F8B.raw |  |  |  |  |  |  |  |  | 7.5662E4 |  | 1 | 0 | 0 | 0 | 0 | 0 | 0 | 0 | 0 | 1 | 0 | 317 | 327 |  |  | PEAKS DB |
| total 2 peptides |
| --- |

P45885|ACT2\_BACDO

back to list

  

| Protein Coverage
| Supporting Peptides
|

Protein Coverage:

Supporting Peptides:

| Peptide | Uniq | -10lgP | Mass | Length | ppm | m/z | z | RT | Fraction | Scan | Source File | Area F1 | Area F10 | Area F2\_3 | Area F5 | Area F6 | Area F7A | Area F7B | Area F8A | Area F8B | Area F9 | #Feature | #Feature F1 | #Feature F10 | #Feature F2\_3 | #Feature F5 | #Feature F6 | #Feature F7A | #Feature F7B | #Feature F8A | #Feature F8B | #Feature F9 | Start | End | PTM | AScore | Found By |
| --- | --- | --- | --- | --- | --- | --- | --- | --- | --- | --- | --- | --- | --- | --- | --- | --- | --- | --- | --- | --- | --- | --- | --- | --- | --- | --- | --- | --- | --- | --- | --- | --- | --- | --- | --- | --- | --- |
| R.VAPEEHPVLLTEAPLNPK.A | Y | 58.65 | 1953.0570 | 18 | 0.5 | 652.0266 | 3 | 12.27 | 9 | F9:3064 | DaRuMP\_F8B.raw |  |  |  |  |  |  |  |  | 1.7091E5 |  | 1 | 0 | 0 | 0 | 0 | 0 | 0 | 0 | 0 | 1 | 0 | 97 | 114 |  |  | PEAKS DB |
| K.EITALAPSTIK.I | Y | 53.96 | 1142.6547 | 11 | -0.4 | 572.3344 | 2 | 12.23 | 9 | F9:3034 | DaRuMP\_F8B.raw |  |  |  |  |  |  |  |  | 7.5662E4 |  | 1 | 0 | 0 | 0 | 0 | 0 | 0 | 0 | 0 | 1 | 0 | 317 | 327 |  |  | PEAKS DB |
| total 2 peptides |
| --- |

P07836|ACT1\_BOMMO

back to list

  

| Protein Coverage
| Supporting Peptides
|

Protein Coverage:

Supporting Peptides:

| Peptide | Uniq | -10lgP | Mass | Length | ppm | m/z | z | RT | Fraction | Scan | Source File | Area F1 | Area F10 | Area F2\_3 | Area F5 | Area F6 | Area F7A | Area F7B | Area F8A | Area F8B | Area F9 | #Feature | #Feature F1 | #Feature F10 | #Feature F2\_3 | #Feature F5 | #Feature F6 | #Feature F7A | #Feature F7B | #Feature F8A | #Feature F8B | #Feature F9 | Start | End | PTM | AScore | Found By |
| --- | --- | --- | --- | --- | --- | --- | --- | --- | --- | --- | --- | --- | --- | --- | --- | --- | --- | --- | --- | --- | --- | --- | --- | --- | --- | --- | --- | --- | --- | --- | --- | --- | --- | --- | --- | --- | --- |
| R.VAPEEHPVLLTEAPLNPK.A | Y | 58.65 | 1953.0570 | 18 | 0.5 | 652.0266 | 3 | 12.27 | 9 | F9:3064 | DaRuMP\_F8B.raw |  |  |  |  |  |  |  |  | 1.7091E5 |  | 1 | 0 | 0 | 0 | 0 | 0 | 0 | 0 | 0 | 1 | 0 | 97 | 114 |  |  | PEAKS DB |
| K.EITALAPSTIK.I | Y | 53.96 | 1142.6547 | 11 | -0.4 | 572.3344 | 2 | 12.23 | 9 | F9:3034 | DaRuMP\_F8B.raw |  |  |  |  |  |  |  |  | 7.5662E4 |  | 1 | 0 | 0 | 0 | 0 | 0 | 0 | 0 | 0 | 1 | 0 | 317 | 327 |  |  | PEAKS DB |
| total 2 peptides |
| --- |

P02574|ACT4\_DROME

back to list

  

| Protein Coverage
| Supporting Peptides
|

Protein Coverage:

Supporting Peptides:

| Peptide | Uniq | -10lgP | Mass | Length | ppm | m/z | z | RT | Fraction | Scan | Source File | Area F1 | Area F10 | Area F2\_3 | Area F5 | Area F6 | Area F7A | Area F7B | Area F8A | Area F8B | Area F9 | #Feature | #Feature F1 | #Feature F10 | #Feature F2\_3 | #Feature F5 | #Feature F6 | #Feature F7A | #Feature F7B | #Feature F8A | #Feature F8B | #Feature F9 | Start | End | PTM | AScore | Found By |
| --- | --- | --- | --- | --- | --- | --- | --- | --- | --- | --- | --- | --- | --- | --- | --- | --- | --- | --- | --- | --- | --- | --- | --- | --- | --- | --- | --- | --- | --- | --- | --- | --- | --- | --- | --- | --- | --- |
| R.VAPEEHPVLLTEAPLNPK.A | Y | 58.65 | 1953.0570 | 18 | 0.5 | 652.0266 | 3 | 12.27 | 9 | F9:3064 | DaRuMP\_F8B.raw |  |  |  |  |  |  |  |  | 1.7091E5 |  | 1 | 0 | 0 | 0 | 0 | 0 | 0 | 0 | 0 | 1 | 0 | 97 | 114 |  |  | PEAKS DB |
| K.EITALAPSTIK.I | Y | 53.96 | 1142.6547 | 11 | -0.4 | 572.3344 | 2 | 12.23 | 9 | F9:3034 | DaRuMP\_F8B.raw |  |  |  |  |  |  |  |  | 7.5662E4 |  | 1 | 0 | 0 | 0 | 0 | 0 | 0 | 0 | 0 | 1 | 0 | 317 | 327 |  |  | PEAKS DB |
| total 2 peptides |
| --- |

P45887|ACT5\_BACDO

back to list

  

| Protein Coverage
| Supporting Peptides
|

Protein Coverage:

Supporting Peptides:

| Peptide | Uniq | -10lgP | Mass | Length | ppm | m/z | z | RT | Fraction | Scan | Source File | Area F1 | Area F10 | Area F2\_3 | Area F5 | Area F6 | Area F7A | Area F7B | Area F8A | Area F8B | Area F9 | #Feature | #Feature F1 | #Feature F10 | #Feature F2\_3 | #Feature F5 | #Feature F6 | #Feature F7A | #Feature F7B | #Feature F8A | #Feature F8B | #Feature F9 | Start | End | PTM | AScore | Found By |
| --- | --- | --- | --- | --- | --- | --- | --- | --- | --- | --- | --- | --- | --- | --- | --- | --- | --- | --- | --- | --- | --- | --- | --- | --- | --- | --- | --- | --- | --- | --- | --- | --- | --- | --- | --- | --- | --- |
| R.VAPEEHPVLLTEAPLNPK.A | Y | 58.65 | 1953.0570 | 18 | 0.5 | 652.0266 | 3 | 12.27 | 9 | F9:3064 | DaRuMP\_F8B.raw |  |  |  |  |  |  |  |  | 1.7091E5 |  | 1 | 0 | 0 | 0 | 0 | 0 | 0 | 0 | 0 | 1 | 0 | 97 | 114 |  |  | PEAKS DB |
| K.EITALAPSTIK.I | Y | 53.96 | 1142.6547 | 11 | -0.4 | 572.3344 | 2 | 12.23 | 9 | F9:3034 | DaRuMP\_F8B.raw |  |  |  |  |  |  |  |  | 7.5662E4 |  | 1 | 0 | 0 | 0 | 0 | 0 | 0 | 0 | 0 | 1 | 0 | 317 | 327 |  |  | PEAKS DB |
| total 2 peptides |
| --- |

P49871|ACT\_MANSE

back to list

  

| Protein Coverage
| Supporting Peptides
|

Protein Coverage:

Supporting Peptides:

| Peptide | Uniq | -10lgP | Mass | Length | ppm | m/z | z | RT | Fraction | Scan | Source File | Area F1 | Area F10 | Area F2\_3 | Area F5 | Area F6 | Area F7A | Area F7B | Area F8A | Area F8B | Area F9 | #Feature | #Feature F1 | #Feature F10 | #Feature F2\_3 | #Feature F5 | #Feature F6 | #Feature F7A | #Feature F7B | #Feature F8A | #Feature F8B | #Feature F9 | Start | End | PTM | AScore | Found By |
| --- | --- | --- | --- | --- | --- | --- | --- | --- | --- | --- | --- | --- | --- | --- | --- | --- | --- | --- | --- | --- | --- | --- | --- | --- | --- | --- | --- | --- | --- | --- | --- | --- | --- | --- | --- | --- | --- |
| R.VAPEEHPVLLTEAPLNPK.A | Y | 58.65 | 1953.0570 | 18 | 0.5 | 652.0266 | 3 | 12.27 | 9 | F9:3064 | DaRuMP\_F8B.raw |  |  |  |  |  |  |  |  | 1.7091E5 |  | 1 | 0 | 0 | 0 | 0 | 0 | 0 | 0 | 0 | 1 | 0 | 97 | 114 |  |  | PEAKS DB |
| K.EITALAPSTIK.I | Y | 53.96 | 1142.6547 | 11 | -0.4 | 572.3344 | 2 | 12.23 | 9 | F9:3034 | DaRuMP\_F8B.raw |  |  |  |  |  |  |  |  | 7.5662E4 |  | 1 | 0 | 0 | 0 | 0 | 0 | 0 | 0 | 0 | 1 | 0 | 317 | 327 |  |  | PEAKS DB |
| total 2 peptides |
| --- |

Q56EB1|SLAA\_BOTJA

back to list

  

| Protein Coverage
| Supporting Peptides
|

Protein Coverage:

Supporting Peptides:

| Peptide | Uniq | -10lgP | Mass | Length | ppm | m/z | z | RT | Fraction | Scan | Source File | Area F1 | Area F10 | Area F2\_3 | Area F5 | Area F6 | Area F7A | Area F7B | Area F8A | Area F8B | Area F9 | #Feature | #Feature F1 | #Feature F10 | #Feature F2\_3 | #Feature F5 | #Feature F6 | #Feature F7A | #Feature F7B | #Feature F8A | #Feature F8B | #Feature F9 | Start | End | PTM | AScore | Found By |
| --- | --- | --- | --- | --- | --- | --- | --- | --- | --- | --- | --- | --- | --- | --- | --- | --- | --- | --- | --- | --- | --- | --- | --- | --- | --- | --- | --- | --- | --- | --- | --- | --- | --- | --- | --- | --- | --- |
| A.DC(+57.02)PSDWSSHEGHC(+57.02)YK.F | Y | 66.22 | 1863.6941 | 15 | 0.0 | 932.8543 | 2 | 10.90 | 7 | F7:1733 | DaRuMP\_F7B.raw |  |  |  |  | 3.2841E5 |  | 6.5889E6 |  | 7.0657E6 |  | 7 | 0 | 0 | 0 | 0 | 1 | 0 | 3 | 0 | 3 | 0 | 24 | 38 | Carbamidomethylation | C2:Carbamidomethylation:1000.00;C13:Carbamidomethylation:1000.00 | PEAKS DB |
| C.PSDWSSHEGHC(+57.02)YK.F | Y | 59.00 | 1588.6365 | 13 | 0.1 | 530.5528 | 3 | 10.90 | 7 | F7:1753 | DaRuMP\_F7B.raw |  |  |  |  |  |  | 1.55E5 |  | 2.3075E5 |  | 2 | 0 | 0 | 0 | 0 | 0 | 0 | 1 | 0 | 1 | 0 | 26 | 38 | Carbamidomethylation | C11:Carbamidomethylation:1000.00 | PEAKS DB |
| total 2 peptides |
| --- |

Q6T7B7|LEC1\_BITGA

back to list

  

| Protein Coverage
| Supporting Peptides
|

Protein Coverage:

Supporting Peptides:

| Peptide | Uniq | -10lgP | Mass | Length | ppm | m/z | z | RT | Fraction | Scan | Source File | Area F1 | Area F10 | Area F2\_3 | Area F5 | Area F6 | Area F7A | Area F7B | Area F8A | Area F8B | Area F9 | #Feature | #Feature F1 | #Feature F10 | #Feature F2\_3 | #Feature F5 | #Feature F6 | #Feature F7A | #Feature F7B | #Feature F8A | #Feature F8B | #Feature F9 | Start | End | PTM | AScore | Found By |
| --- | --- | --- | --- | --- | --- | --- | --- | --- | --- | --- | --- | --- | --- | --- | --- | --- | --- | --- | --- | --- | --- | --- | --- | --- | --- | --- | --- | --- | --- | --- | --- | --- | --- | --- | --- | --- | --- |
| K.YKPGC(+57.02)HLASLHSK.R | Y | 70.63 | 1496.7557 | 13 | -5.0 | 375.1943 | 4 | 10.73 | 7 | F7:1592 | DaRuMP\_F7B.raw |  |  |  |  |  |  | 1.2984E6 |  |  |  | 1 | 0 | 0 | 0 | 0 | 0 | 0 | 1 | 0 | 0 | 0 | 57 | 69 | Carbamidomethylation | C5:Carbamidomethylation:1000.00 | PEAKS DB |
| total 1 peptides |
| --- |

Q6LAF3|H4\_FLATR

back to list

  

| Protein Coverage
| Supporting Peptides
|

Protein Coverage:

Supporting Peptides:

| Peptide | Uniq | -10lgP | Mass | Length | ppm | m/z | z | RT | Fraction | Scan | Source File | Area F1 | Area F10 | Area F2\_3 | Area F5 | Area F6 | Area F7A | Area F7B | Area F8A | Area F8B | Area F9 | #Feature | #Feature F1 | #Feature F10 | #Feature F2\_3 | #Feature F5 | #Feature F6 | #Feature F7A | #Feature F7B | #Feature F8A | #Feature F8B | #Feature F9 | Start | End | PTM | AScore | Found By |
| --- | --- | --- | --- | --- | --- | --- | --- | --- | --- | --- | --- | --- | --- | --- | --- | --- | --- | --- | --- | --- | --- | --- | --- | --- | --- | --- | --- | --- | --- | --- | --- | --- | --- | --- | --- | --- | --- |
| R.ISGLIYEETR.G | Y | 66.51 | 1179.6135 | 10 | 0.8 | 590.8145 | 2 | 12.05 | 9 | F9:2894 | DaRuMP\_F8B.raw |  |  |  |  |  |  |  |  | 6.5839E4 |  | 1 | 0 | 0 | 0 | 0 | 0 | 0 | 0 | 0 | 1 | 0 | 47 | 56 |  |  | PEAKS DB |
| total 1 peptides |
| --- |

P62887|H4\_LOLTE

back to list

  

| Protein Coverage
| Supporting Peptides
|

Protein Coverage:

Supporting Peptides:

| Peptide | Uniq | -10lgP | Mass | Length | ppm | m/z | z | RT | Fraction | Scan | Source File | Area F1 | Area F10 | Area F2\_3 | Area F5 | Area F6 | Area F7A | Area F7B | Area F8A | Area F8B | Area F9 | #Feature | #Feature F1 | #Feature F10 | #Feature F2\_3 | #Feature F5 | #Feature F6 | #Feature F7A | #Feature F7B | #Feature F8A | #Feature F8B | #Feature F9 | Start | End | PTM | AScore | Found By |
| --- | --- | --- | --- | --- | --- | --- | --- | --- | --- | --- | --- | --- | --- | --- | --- | --- | --- | --- | --- | --- | --- | --- | --- | --- | --- | --- | --- | --- | --- | --- | --- | --- | --- | --- | --- | --- | --- |
| R.ISGLIYEETR.G | Y | 66.51 | 1179.6135 | 10 | 0.8 | 590.8145 | 2 | 12.05 | 9 | F9:2894 | DaRuMP\_F8B.raw |  |  |  |  |  |  |  |  | 6.5839E4 |  | 1 | 0 | 0 | 0 | 0 | 0 | 0 | 0 | 0 | 1 | 0 | 47 | 56 |  |  | PEAKS DB |
| total 1 peptides |
| --- |

P59259|H4\_ARATH

back to list

  

| Protein Coverage
| Supporting Peptides
|

Protein Coverage:

Supporting Peptides:

| Peptide | Uniq | -10lgP | Mass | Length | ppm | m/z | z | RT | Fraction | Scan | Source File | Area F1 | Area F10 | Area F2\_3 | Area F5 | Area F6 | Area F7A | Area F7B | Area F8A | Area F8B | Area F9 | #Feature | #Feature F1 | #Feature F10 | #Feature F2\_3 | #Feature F5 | #Feature F6 | #Feature F7A | #Feature F7B | #Feature F8A | #Feature F8B | #Feature F9 | Start | End | PTM | AScore | Found By |
| --- | --- | --- | --- | --- | --- | --- | --- | --- | --- | --- | --- | --- | --- | --- | --- | --- | --- | --- | --- | --- | --- | --- | --- | --- | --- | --- | --- | --- | --- | --- | --- | --- | --- | --- | --- | --- | --- |
| R.ISGLIYEETR.G | Y | 66.51 | 1179.6135 | 10 | 0.8 | 590.8145 | 2 | 12.05 | 9 | F9:2894 | DaRuMP\_F8B.raw |  |  |  |  |  |  |  |  | 6.5839E4 |  | 1 | 0 | 0 | 0 | 0 | 0 | 0 | 0 | 0 | 1 | 0 | 47 | 56 |  |  | PEAKS DB |
| total 1 peptides |
| --- |

Q6PMI5|H4\_CHEMJ

back to list

  

| Protein Coverage
| Supporting Peptides
|

Protein Coverage:

Supporting Peptides:

| Peptide | Uniq | -10lgP | Mass | Length | ppm | m/z | z | RT | Fraction | Scan | Source File | Area F1 | Area F10 | Area F2\_3 | Area F5 | Area F6 | Area F7A | Area F7B | Area F8A | Area F8B | Area F9 | #Feature | #Feature F1 | #Feature F10 | #Feature F2\_3 | #Feature F5 | #Feature F6 | #Feature F7A | #Feature F7B | #Feature F8A | #Feature F8B | #Feature F9 | Start | End | PTM | AScore | Found By |
| --- | --- | --- | --- | --- | --- | --- | --- | --- | --- | --- | --- | --- | --- | --- | --- | --- | --- | --- | --- | --- | --- | --- | --- | --- | --- | --- | --- | --- | --- | --- | --- | --- | --- | --- | --- | --- | --- |
| R.ISGLIYEETR.G | Y | 66.51 | 1179.6135 | 10 | 0.8 | 590.8145 | 2 | 12.05 | 9 | F9:2894 | DaRuMP\_F8B.raw |  |  |  |  |  |  |  |  | 6.5839E4 |  | 1 | 0 | 0 | 0 | 0 | 0 | 0 | 0 | 0 | 1 | 0 | 47 | 56 |  |  | PEAKS DB |
| total 1 peptides |
| --- |

Q43083|H4\_PYRSA

back to list

  

| Protein Coverage
| Supporting Peptides
|

Protein Coverage:

Supporting Peptides:

| Peptide | Uniq | -10lgP | Mass | Length | ppm | m/z | z | RT | Fraction | Scan | Source File | Area F1 | Area F10 | Area F2\_3 | Area F5 | Area F6 | Area F7A | Area F7B | Area F8A | Area F8B | Area F9 | #Feature | #Feature F1 | #Feature F10 | #Feature F2\_3 | #Feature F5 | #Feature F6 | #Feature F7A | #Feature F7B | #Feature F8A | #Feature F8B | #Feature F9 | Start | End | PTM | AScore | Found By |
| --- | --- | --- | --- | --- | --- | --- | --- | --- | --- | --- | --- | --- | --- | --- | --- | --- | --- | --- | --- | --- | --- | --- | --- | --- | --- | --- | --- | --- | --- | --- | --- | --- | --- | --- | --- | --- | --- |
| R.ISGLIYEETR.S | Y | 66.51 | 1179.6135 | 10 | 0.8 | 590.8145 | 2 | 12.05 | 9 | F9:2894 | DaRuMP\_F8B.raw |  |  |  |  |  |  |  |  | 6.5839E4 |  | 1 | 0 | 0 | 0 | 0 | 0 | 0 | 0 | 0 | 1 | 0 | 47 | 56 |  |  | PEAKS DB |
| total 1 peptides |
| --- |

Q6WZ83|H4\_EUCGL

back to list

  

| Protein Coverage
| Supporting Peptides
|

Protein Coverage:

Supporting Peptides:

| Peptide | Uniq | -10lgP | Mass | Length | ppm | m/z | z | RT | Fraction | Scan | Source File | Area F1 | Area F10 | Area F2\_3 | Area F5 | Area F6 | Area F7A | Area F7B | Area F8A | Area F8B | Area F9 | #Feature | #Feature F1 | #Feature F10 | #Feature F2\_3 | #Feature F5 | #Feature F6 | #Feature F7A | #Feature F7B | #Feature F8A | #Feature F8B | #Feature F9 | Start | End | PTM | AScore | Found By |
| --- | --- | --- | --- | --- | --- | --- | --- | --- | --- | --- | --- | --- | --- | --- | --- | --- | --- | --- | --- | --- | --- | --- | --- | --- | --- | --- | --- | --- | --- | --- | --- | --- | --- | --- | --- | --- | --- |
| R.ISGLIYEETR.G | Y | 66.51 | 1179.6135 | 10 | 0.8 | 590.8145 | 2 | 12.05 | 9 | F9:2894 | DaRuMP\_F8B.raw |  |  |  |  |  |  |  |  | 6.5839E4 |  | 1 | 0 | 0 | 0 | 0 | 0 | 0 | 0 | 0 | 1 | 0 | 47 | 56 |  |  | PEAKS DB |
| total 1 peptides |
| --- |

P70081|H48\_CHICK

back to list

  

| Protein Coverage
| Supporting Peptides
|

Protein Coverage:

Supporting Peptides:

| Peptide | Uniq | -10lgP | Mass | Length | ppm | m/z | z | RT | Fraction | Scan | Source File | Area F1 | Area F10 | Area F2\_3 | Area F5 | Area F6 | Area F7A | Area F7B | Area F8A | Area F8B | Area F9 | #Feature | #Feature F1 | #Feature F10 | #Feature F2\_3 | #Feature F5 | #Feature F6 | #Feature F7A | #Feature F7B | #Feature F8A | #Feature F8B | #Feature F9 | Start | End | PTM | AScore | Found By |
| --- | --- | --- | --- | --- | --- | --- | --- | --- | --- | --- | --- | --- | --- | --- | --- | --- | --- | --- | --- | --- | --- | --- | --- | --- | --- | --- | --- | --- | --- | --- | --- | --- | --- | --- | --- | --- | --- |
| R.ISGLIYEETR.G | Y | 66.51 | 1179.6135 | 10 | 0.8 | 590.8145 | 2 | 12.05 | 9 | F9:2894 | DaRuMP\_F8B.raw |  |  |  |  |  |  |  |  | 6.5839E4 |  | 1 | 0 | 0 | 0 | 0 | 0 | 0 | 0 | 0 | 1 | 0 | 47 | 56 |  |  | PEAKS DB |
| total 1 peptides |
| --- |

P84049|H4\_MYRRU

back to list

  

| Protein Coverage
| Supporting Peptides
|

Protein Coverage:

Supporting Peptides:

| Peptide | Uniq | -10lgP | Mass | Length | ppm | m/z | z | RT | Fraction | Scan | Source File | Area F1 | Area F10 | Area F2\_3 | Area F5 | Area F6 | Area F7A | Area F7B | Area F8A | Area F8B | Area F9 | #Feature | #Feature F1 | #Feature F10 | #Feature F2\_3 | #Feature F5 | #Feature F6 | #Feature F7A | #Feature F7B | #Feature F8A | #Feature F8B | #Feature F9 | Start | End | PTM | AScore | Found By |
| --- | --- | --- | --- | --- | --- | --- | --- | --- | --- | --- | --- | --- | --- | --- | --- | --- | --- | --- | --- | --- | --- | --- | --- | --- | --- | --- | --- | --- | --- | --- | --- | --- | --- | --- | --- | --- | --- |
| R.ISGLIYEETR.G | Y | 66.51 | 1179.6135 | 10 | 0.8 | 590.8145 | 2 | 12.05 | 9 | F9:2894 | DaRuMP\_F8B.raw |  |  |  |  |  |  |  |  | 6.5839E4 |  | 1 | 0 | 0 | 0 | 0 | 0 | 0 | 0 | 0 | 1 | 0 | 47 | 56 |  |  | PEAKS DB |
| total 1 peptides |
| --- |

Q76FF1|H4\_DROOR

back to list

  

| Protein Coverage
| Supporting Peptides
|

Protein Coverage:

Supporting Peptides:

| Peptide | Uniq | -10lgP | Mass | Length | ppm | m/z | z | RT | Fraction | Scan | Source File | Area F1 | Area F10 | Area F2\_3 | Area F5 | Area F6 | Area F7A | Area F7B | Area F8A | Area F8B | Area F9 | #Feature | #Feature F1 | #Feature F10 | #Feature F2\_3 | #Feature F5 | #Feature F6 | #Feature F7A | #Feature F7B | #Feature F8A | #Feature F8B | #Feature F9 | Start | End | PTM | AScore | Found By |
| --- | --- | --- | --- | --- | --- | --- | --- | --- | --- | --- | --- | --- | --- | --- | --- | --- | --- | --- | --- | --- | --- | --- | --- | --- | --- | --- | --- | --- | --- | --- | --- | --- | --- | --- | --- | --- | --- |
| R.ISGLIYEETR.G | Y | 66.51 | 1179.6135 | 10 | 0.8 | 590.8145 | 2 | 12.05 | 9 | F9:2894 | DaRuMP\_F8B.raw |  |  |  |  |  |  |  |  | 6.5839E4 |  | 1 | 0 | 0 | 0 | 0 | 0 | 0 | 0 | 0 | 1 | 0 | 47 | 56 |  |  | PEAKS DB |
| total 1 peptides |
| --- |

P62800|H4\_CAIMO

back to list

  

| Protein Coverage
| Supporting Peptides
|

Protein Coverage:

Supporting Peptides:

| Peptide | Uniq | -10lgP | Mass | Length | ppm | m/z | z | RT | Fraction | Scan | Source File | Area F1 | Area F10 | Area F2\_3 | Area F5 | Area F6 | Area F7A | Area F7B | Area F8A | Area F8B | Area F9 | #Feature | #Feature F1 | #Feature F10 | #Feature F2\_3 | #Feature F5 | #Feature F6 | #Feature F7A | #Feature F7B | #Feature F8A | #Feature F8B | #Feature F9 | Start | End | PTM | AScore | Found By |
| --- | --- | --- | --- | --- | --- | --- | --- | --- | --- | --- | --- | --- | --- | --- | --- | --- | --- | --- | --- | --- | --- | --- | --- | --- | --- | --- | --- | --- | --- | --- | --- | --- | --- | --- | --- | --- | --- |
| R.ISGLIYEETR.G | Y | 66.51 | 1179.6135 | 10 | 0.8 | 590.8145 | 2 | 12.05 | 9 | F9:2894 | DaRuMP\_F8B.raw |  |  |  |  |  |  |  |  | 6.5839E4 |  | 1 | 0 | 0 | 0 | 0 | 0 | 0 | 0 | 0 | 1 | 0 | 47 | 56 |  |  | PEAKS DB |
| total 1 peptides |
| --- |

Q6WV73|H4\_MYTCA

back to list

  

| Protein Coverage
| Supporting Peptides
|

Protein Coverage:

Supporting Peptides:

| Peptide | Uniq | -10lgP | Mass | Length | ppm | m/z | z | RT | Fraction | Scan | Source File | Area F1 | Area F10 | Area F2\_3 | Area F5 | Area F6 | Area F7A | Area F7B | Area F8A | Area F8B | Area F9 | #Feature | #Feature F1 | #Feature F10 | #Feature F2\_3 | #Feature F5 | #Feature F6 | #Feature F7A | #Feature F7B | #Feature F8A | #Feature F8B | #Feature F9 | Start | End | PTM | AScore | Found By |
| --- | --- | --- | --- | --- | --- | --- | --- | --- | --- | --- | --- | --- | --- | --- | --- | --- | --- | --- | --- | --- | --- | --- | --- | --- | --- | --- | --- | --- | --- | --- | --- | --- | --- | --- | --- | --- | --- |
| R.ISGLIYEETR.G | Y | 66.51 | 1179.6135 | 10 | 0.8 | 590.8145 | 2 | 12.05 | 9 | F9:2894 | DaRuMP\_F8B.raw |  |  |  |  |  |  |  |  | 6.5839E4 |  | 1 | 0 | 0 | 0 | 0 | 0 | 0 | 0 | 0 | 1 | 0 | 47 | 56 |  |  | PEAKS DB |
| total 1 peptides |
| --- |

Q6WV72|H4\_MYTTR

back to list

  

| Protein Coverage
| Supporting Peptides
|

Protein Coverage:

Supporting Peptides:

| Peptide | Uniq | -10lgP | Mass | Length | ppm | m/z | z | RT | Fraction | Scan | Source File | Area F1 | Area F10 | Area F2\_3 | Area F5 | Area F6 | Area F7A | Area F7B | Area F8A | Area F8B | Area F9 | #Feature | #Feature F1 | #Feature F10 | #Feature F2\_3 | #Feature F5 | #Feature F6 | #Feature F7A | #Feature F7B | #Feature F8A | #Feature F8B | #Feature F9 | Start | End | PTM | AScore | Found By |
| --- | --- | --- | --- | --- | --- | --- | --- | --- | --- | --- | --- | --- | --- | --- | --- | --- | --- | --- | --- | --- | --- | --- | --- | --- | --- | --- | --- | --- | --- | --- | --- | --- | --- | --- | --- | --- | --- |
| R.ISGLIYEETR.G | Y | 66.51 | 1179.6135 | 10 | 0.8 | 590.8145 | 2 | 12.05 | 9 | F9:2894 | DaRuMP\_F8B.raw |  |  |  |  |  |  |  |  | 6.5839E4 |  | 1 | 0 | 0 | 0 | 0 | 0 | 0 | 0 | 0 | 1 | 0 | 47 | 56 |  |  | PEAKS DB |
| total 1 peptides |
| --- |

P62779|H4\_PYCHE

back to list

  

| Protein Coverage
| Supporting Peptides
|

Protein Coverage:

Supporting Peptides:

| Peptide | Uniq | -10lgP | Mass | Length | ppm | m/z | z | RT | Fraction | Scan | Source File | Area F1 | Area F10 | Area F2\_3 | Area F5 | Area F6 | Area F7A | Area F7B | Area F8A | Area F8B | Area F9 | #Feature | #Feature F1 | #Feature F10 | #Feature F2\_3 | #Feature F5 | #Feature F6 | #Feature F7A | #Feature F7B | #Feature F8A | #Feature F8B | #Feature F9 | Start | End | PTM | AScore | Found By |
| --- | --- | --- | --- | --- | --- | --- | --- | --- | --- | --- | --- | --- | --- | --- | --- | --- | --- | --- | --- | --- | --- | --- | --- | --- | --- | --- | --- | --- | --- | --- | --- | --- | --- | --- | --- | --- | --- |
| R.ISGLIYEETR.G | Y | 66.51 | 1179.6135 | 10 | 0.8 | 590.8145 | 2 | 12.05 | 9 | F9:2894 | DaRuMP\_F8B.raw |  |  |  |  |  |  |  |  | 6.5839E4 |  | 1 | 0 | 0 | 0 | 0 | 0 | 0 | 0 | 0 | 1 | 0 | 47 | 56 |  |  | PEAKS DB |
| total 1 peptides |
| --- |

Q28DR4|H4\_XENTR

back to list

  

| Protein Coverage
| Supporting Peptides
|

Protein Coverage:

Supporting Peptides:

| Peptide | Uniq | -10lgP | Mass | Length | ppm | m/z | z | RT | Fraction | Scan | Source File | Area F1 | Area F10 | Area F2\_3 | Area F5 | Area F6 | Area F7A | Area F7B | Area F8A | Area F8B | Area F9 | #Feature | #Feature F1 | #Feature F10 | #Feature F2\_3 | #Feature F5 | #Feature F6 | #Feature F7A | #Feature F7B | #Feature F8A | #Feature F8B | #Feature F9 | Start | End | PTM | AScore | Found By |
| --- | --- | --- | --- | --- | --- | --- | --- | --- | --- | --- | --- | --- | --- | --- | --- | --- | --- | --- | --- | --- | --- | --- | --- | --- | --- | --- | --- | --- | --- | --- | --- | --- | --- | --- | --- | --- | --- |
| R.ISGLIYEETR.G | Y | 66.51 | 1179.6135 | 10 | 0.8 | 590.8145 | 2 | 12.05 | 9 | F9:2894 | DaRuMP\_F8B.raw |  |  |  |  |  |  |  |  | 6.5839E4 |  | 1 | 0 | 0 | 0 | 0 | 0 | 0 | 0 | 0 | 1 | 0 | 47 | 56 |  |  | PEAKS DB |
| total 1 peptides |
| --- |

P91882|H4\_DIAPU

back to list

  

| Protein Coverage
| Supporting Peptides
|

Protein Coverage:

Supporting Peptides:

| Peptide | Uniq | -10lgP | Mass | Length | ppm | m/z | z | RT | Fraction | Scan | Source File | Area F1 | Area F10 | Area F2\_3 | Area F5 | Area F6 | Area F7A | Area F7B | Area F8A | Area F8B | Area F9 | #Feature | #Feature F1 | #Feature F10 | #Feature F2\_3 | #Feature F5 | #Feature F6 | #Feature F7A | #Feature F7B | #Feature F8A | #Feature F8B | #Feature F9 | Start | End | PTM | AScore | Found By |
| --- | --- | --- | --- | --- | --- | --- | --- | --- | --- | --- | --- | --- | --- | --- | --- | --- | --- | --- | --- | --- | --- | --- | --- | --- | --- | --- | --- | --- | --- | --- | --- | --- | --- | --- | --- | --- | --- |
| R.ISGLIYEETR.G | Y | 66.51 | 1179.6135 | 10 | 0.8 | 590.8145 | 2 | 12.05 | 9 | F9:2894 | DaRuMP\_F8B.raw |  |  |  |  |  |  |  |  | 6.5839E4 |  | 1 | 0 | 0 | 0 | 0 | 0 | 0 | 0 | 0 | 1 | 0 | 47 | 56 |  |  | PEAKS DB |
| total 1 peptides |
| --- |

Q6WV74|H4\_MYTCH

back to list

  

| Protein Coverage
| Supporting Peptides
|

Protein Coverage:

Supporting Peptides:

| Peptide | Uniq | -10lgP | Mass | Length | ppm | m/z | z | RT | Fraction | Scan | Source File | Area F1 | Area F10 | Area F2\_3 | Area F5 | Area F6 | Area F7A | Area F7B | Area F8A | Area F8B | Area F9 | #Feature | #Feature F1 | #Feature F10 | #Feature F2\_3 | #Feature F5 | #Feature F6 | #Feature F7A | #Feature F7B | #Feature F8A | #Feature F8B | #Feature F9 | Start | End | PTM | AScore | Found By |
| --- | --- | --- | --- | --- | --- | --- | --- | --- | --- | --- | --- | --- | --- | --- | --- | --- | --- | --- | --- | --- | --- | --- | --- | --- | --- | --- | --- | --- | --- | --- | --- | --- | --- | --- | --- | --- | --- |
| R.ISGLIYEETR.G | Y | 66.51 | 1179.6135 | 10 | 0.8 | 590.8145 | 2 | 12.05 | 9 | F9:2894 | DaRuMP\_F8B.raw |  |  |  |  |  |  |  |  | 6.5839E4 |  | 1 | 0 | 0 | 0 | 0 | 0 | 0 | 0 | 0 | 1 | 0 | 47 | 56 |  |  | PEAKS DB |
| total 1 peptides |
| --- |

Q6LAF1|H4\_DENKL

back to list

  

| Protein Coverage
| Supporting Peptides
|

Protein Coverage:

Supporting Peptides:

| Peptide | Uniq | -10lgP | Mass | Length | ppm | m/z | z | RT | Fraction | Scan | Source File | Area F1 | Area F10 | Area F2\_3 | Area F5 | Area F6 | Area F7A | Area F7B | Area F8A | Area F8B | Area F9 | #Feature | #Feature F1 | #Feature F10 | #Feature F2\_3 | #Feature F5 | #Feature F6 | #Feature F7A | #Feature F7B | #Feature F8A | #Feature F8B | #Feature F9 | Start | End | PTM | AScore | Found By |
| --- | --- | --- | --- | --- | --- | --- | --- | --- | --- | --- | --- | --- | --- | --- | --- | --- | --- | --- | --- | --- | --- | --- | --- | --- | --- | --- | --- | --- | --- | --- | --- | --- | --- | --- | --- | --- | --- |
| R.ISGLIYEETR.G | Y | 66.51 | 1179.6135 | 10 | 0.8 | 590.8145 | 2 | 12.05 | 9 | F9:2894 | DaRuMP\_F8B.raw |  |  |  |  |  |  |  |  | 6.5839E4 |  | 1 | 0 | 0 | 0 | 0 | 0 | 0 | 0 | 0 | 1 | 0 | 47 | 56 |  |  | PEAKS DB |
| total 1 peptides |
| --- |

P62784|H4\_CAEEL

back to list

  

| Protein Coverage
| Supporting Peptides
|

Protein Coverage:

Supporting Peptides:

| Peptide | Uniq | -10lgP | Mass | Length | ppm | m/z | z | RT | Fraction | Scan | Source File | Area F1 | Area F10 | Area F2\_3 | Area F5 | Area F6 | Area F7A | Area F7B | Area F8A | Area F8B | Area F9 | #Feature | #Feature F1 | #Feature F10 | #Feature F2\_3 | #Feature F5 | #Feature F6 | #Feature F7A | #Feature F7B | #Feature F8A | #Feature F8B | #Feature F9 | Start | End | PTM | AScore | Found By |
| --- | --- | --- | --- | --- | --- | --- | --- | --- | --- | --- | --- | --- | --- | --- | --- | --- | --- | --- | --- | --- | --- | --- | --- | --- | --- | --- | --- | --- | --- | --- | --- | --- | --- | --- | --- | --- | --- |
| R.ISGLIYEETR.G | Y | 66.51 | 1179.6135 | 10 | 0.8 | 590.8145 | 2 | 12.05 | 9 | F9:2894 | DaRuMP\_F8B.raw |  |  |  |  |  |  |  |  | 6.5839E4 |  | 1 | 0 | 0 | 0 | 0 | 0 | 0 | 0 | 0 | 1 | 0 | 47 | 56 |  |  | PEAKS DB |
| total 1 peptides |
| --- |

Q6WV90|H4\_MYTGA

back to list

  

| Protein Coverage
| Supporting Peptides
|

Protein Coverage:

Supporting Peptides:

| Peptide | Uniq | -10lgP | Mass | Length | ppm | m/z | z | RT | Fraction | Scan | Source File | Area F1 | Area F10 | Area F2\_3 | Area F5 | Area F6 | Area F7A | Area F7B | Area F8A | Area F8B | Area F9 | #Feature | #Feature F1 | #Feature F10 | #Feature F2\_3 | #Feature F5 | #Feature F6 | #Feature F7A | #Feature F7B | #Feature F8A | #Feature F8B | #Feature F9 | Start | End | PTM | AScore | Found By |
| --- | --- | --- | --- | --- | --- | --- | --- | --- | --- | --- | --- | --- | --- | --- | --- | --- | --- | --- | --- | --- | --- | --- | --- | --- | --- | --- | --- | --- | --- | --- | --- | --- | --- | --- | --- | --- | --- |
| R.ISGLIYEETR.G | Y | 66.51 | 1179.6135 | 10 | 0.8 | 590.8145 | 2 | 12.05 | 9 | F9:2894 | DaRuMP\_F8B.raw |  |  |  |  |  |  |  |  | 6.5839E4 |  | 1 | 0 | 0 | 0 | 0 | 0 | 0 | 0 | 0 | 1 | 0 | 47 | 56 |  |  | PEAKS DB |
| total 1 peptides |
| --- |

P62782|H4\_LYTPI

back to list

  

| Protein Coverage
| Supporting Peptides
|

Protein Coverage:

Supporting Peptides:

| Peptide | Uniq | -10lgP | Mass | Length | ppm | m/z | z | RT | Fraction | Scan | Source File | Area F1 | Area F10 | Area F2\_3 | Area F5 | Area F6 | Area F7A | Area F7B | Area F8A | Area F8B | Area F9 | #Feature | #Feature F1 | #Feature F10 | #Feature F2\_3 | #Feature F5 | #Feature F6 | #Feature F7A | #Feature F7B | #Feature F8A | #Feature F8B | #Feature F9 | Start | End | PTM | AScore | Found By |
| --- | --- | --- | --- | --- | --- | --- | --- | --- | --- | --- | --- | --- | --- | --- | --- | --- | --- | --- | --- | --- | --- | --- | --- | --- | --- | --- | --- | --- | --- | --- | --- | --- | --- | --- | --- | --- | --- |
| R.ISGLIYEETR.G | Y | 66.51 | 1179.6135 | 10 | 0.8 | 590.8145 | 2 | 12.05 | 9 | F9:2894 | DaRuMP\_F8B.raw |  |  |  |  |  |  |  |  | 6.5839E4 |  | 1 | 0 | 0 | 0 | 0 | 0 | 0 | 0 | 0 | 1 | 0 | 47 | 56 |  |  | PEAKS DB |
| total 1 peptides |
| --- |

P84046|H4\_CHITH

back to list

  

| Protein Coverage
| Supporting Peptides
|

Protein Coverage:

Supporting Peptides:

| Peptide | Uniq | -10lgP | Mass | Length | ppm | m/z | z | RT | Fraction | Scan | Source File | Area F1 | Area F10 | Area F2\_3 | Area F5 | Area F6 | Area F7A | Area F7B | Area F8A | Area F8B | Area F9 | #Feature | #Feature F1 | #Feature F10 | #Feature F2\_3 | #Feature F5 | #Feature F6 | #Feature F7A | #Feature F7B | #Feature F8A | #Feature F8B | #Feature F9 | Start | End | PTM | AScore | Found By |
| --- | --- | --- | --- | --- | --- | --- | --- | --- | --- | --- | --- | --- | --- | --- | --- | --- | --- | --- | --- | --- | --- | --- | --- | --- | --- | --- | --- | --- | --- | --- | --- | --- | --- | --- | --- | --- | --- |
| R.ISGLIYEETR.G | Y | 66.51 | 1179.6135 | 10 | 0.8 | 590.8145 | 2 | 12.05 | 9 | F9:2894 | DaRuMP\_F8B.raw |  |  |  |  |  |  |  |  | 6.5839E4 |  | 1 | 0 | 0 | 0 | 0 | 0 | 0 | 0 | 0 | 1 | 0 | 47 | 56 |  |  | PEAKS DB |
| total 1 peptides |
| --- |

P62803|H4\_BOVIN

back to list

  

| Protein Coverage
| Supporting Peptides
|

Protein Coverage:

Supporting Peptides:

| Peptide | Uniq | -10lgP | Mass | Length | ppm | m/z | z | RT | Fraction | Scan | Source File | Area F1 | Area F10 | Area F2\_3 | Area F5 | Area F6 | Area F7A | Area F7B | Area F8A | Area F8B | Area F9 | #Feature | #Feature F1 | #Feature F10 | #Feature F2\_3 | #Feature F5 | #Feature F6 | #Feature F7A | #Feature F7B | #Feature F8A | #Feature F8B | #Feature F9 | Start | End | PTM | AScore | Found By |
| --- | --- | --- | --- | --- | --- | --- | --- | --- | --- | --- | --- | --- | --- | --- | --- | --- | --- | --- | --- | --- | --- | --- | --- | --- | --- | --- | --- | --- | --- | --- | --- | --- | --- | --- | --- | --- | --- |
| R.ISGLIYEETR.G | Y | 66.51 | 1179.6135 | 10 | 0.8 | 590.8145 | 2 | 12.05 | 9 | F9:2894 | DaRuMP\_F8B.raw |  |  |  |  |  |  |  |  | 6.5839E4 |  | 1 | 0 | 0 | 0 | 0 | 0 | 0 | 0 | 0 | 1 | 0 | 47 | 56 |  |  | PEAKS DB |
| total 1 peptides |
| --- |

P62776|H4\_HOLTU

back to list

  

| Protein Coverage
| Supporting Peptides
|

Protein Coverage:

Supporting Peptides:

| Peptide | Uniq | -10lgP | Mass | Length | ppm | m/z | z | RT | Fraction | Scan | Source File | Area F1 | Area F10 | Area F2\_3 | Area F5 | Area F6 | Area F7A | Area F7B | Area F8A | Area F8B | Area F9 | #Feature | #Feature F1 | #Feature F10 | #Feature F2\_3 | #Feature F5 | #Feature F6 | #Feature F7A | #Feature F7B | #Feature F8A | #Feature F8B | #Feature F9 | Start | End | PTM | AScore | Found By |
| --- | --- | --- | --- | --- | --- | --- | --- | --- | --- | --- | --- | --- | --- | --- | --- | --- | --- | --- | --- | --- | --- | --- | --- | --- | --- | --- | --- | --- | --- | --- | --- | --- | --- | --- | --- | --- | --- |
| R.ISGLIYEETR.G | Y | 66.51 | 1179.6135 | 10 | 0.8 | 590.8145 | 2 | 12.05 | 9 | F9:2894 | DaRuMP\_F8B.raw |  |  |  |  |  |  |  |  | 6.5839E4 |  | 1 | 0 | 0 | 0 | 0 | 0 | 0 | 0 | 0 | 1 | 0 | 47 | 56 |  |  | PEAKS DB |
| total 1 peptides |
| --- |

Q71V09|H4\_CAPAN

back to list

  

| Protein Coverage
| Supporting Peptides
|

Protein Coverage:

Supporting Peptides:

| Peptide | Uniq | -10lgP | Mass | Length | ppm | m/z | z | RT | Fraction | Scan | Source File | Area F1 | Area F10 | Area F2\_3 | Area F5 | Area F6 | Area F7A | Area F7B | Area F8A | Area F8B | Area F9 | #Feature | #Feature F1 | #Feature F10 | #Feature F2\_3 | #Feature F5 | #Feature F6 | #Feature F7A | #Feature F7B | #Feature F8A | #Feature F8B | #Feature F9 | Start | End | PTM | AScore | Found By |
| --- | --- | --- | --- | --- | --- | --- | --- | --- | --- | --- | --- | --- | --- | --- | --- | --- | --- | --- | --- | --- | --- | --- | --- | --- | --- | --- | --- | --- | --- | --- | --- | --- | --- | --- | --- | --- | --- |
| R.ISGLIYEETR.G | Y | 66.51 | 1179.6135 | 10 | 0.8 | 590.8145 | 2 | 12.05 | 9 | F9:2894 | DaRuMP\_F8B.raw |  |  |  |  |  |  |  |  | 6.5839E4 |  | 1 | 0 | 0 | 0 | 0 | 0 | 0 | 0 | 0 | 1 | 0 | 47 | 56 |  |  | PEAKS DB |
| total 1 peptides |
| --- |

Q7KQD1|H4\_CHAVR

back to list

  

| Protein Coverage
| Supporting Peptides
|

Protein Coverage:

Supporting Peptides:

| Peptide | Uniq | -10lgP | Mass | Length | ppm | m/z | z | RT | Fraction | Scan | Source File | Area F1 | Area F10 | Area F2\_3 | Area F5 | Area F6 | Area F7A | Area F7B | Area F8A | Area F8B | Area F9 | #Feature | #Feature F1 | #Feature F10 | #Feature F2\_3 | #Feature F5 | #Feature F6 | #Feature F7A | #Feature F7B | #Feature F8A | #Feature F8B | #Feature F9 | Start | End | PTM | AScore | Found By |
| --- | --- | --- | --- | --- | --- | --- | --- | --- | --- | --- | --- | --- | --- | --- | --- | --- | --- | --- | --- | --- | --- | --- | --- | --- | --- | --- | --- | --- | --- | --- | --- | --- | --- | --- | --- | --- | --- |
| R.ISGLIYEETR.G | Y | 66.51 | 1179.6135 | 10 | 0.8 | 590.8145 | 2 | 12.05 | 9 | F9:2894 | DaRuMP\_F8B.raw |  |  |  |  |  |  |  |  | 6.5839E4 |  | 1 | 0 | 0 | 0 | 0 | 0 | 0 | 0 | 0 | 1 | 0 | 47 | 56 |  |  | PEAKS DB |
| total 1 peptides |
| --- |

P50566|H4\_CHLRE

back to list

  

| Protein Coverage
| Supporting Peptides
|

Protein Coverage:

Supporting Peptides:

| Peptide | Uniq | -10lgP | Mass | Length | ppm | m/z | z | RT | Fraction | Scan | Source File | Area F1 | Area F10 | Area F2\_3 | Area F5 | Area F6 | Area F7A | Area F7B | Area F8A | Area F8B | Area F9 | #Feature | #Feature F1 | #Feature F10 | #Feature F2\_3 | #Feature F5 | #Feature F6 | #Feature F7A | #Feature F7B | #Feature F8A | #Feature F8B | #Feature F9 | Start | End | PTM | AScore | Found By |
| --- | --- | --- | --- | --- | --- | --- | --- | --- | --- | --- | --- | --- | --- | --- | --- | --- | --- | --- | --- | --- | --- | --- | --- | --- | --- | --- | --- | --- | --- | --- | --- | --- | --- | --- | --- | --- | --- |
| R.ISGLIYEETR.T | Y | 66.51 | 1179.6135 | 10 | 0.8 | 590.8145 | 2 | 12.05 | 9 | F9:2894 | DaRuMP\_F8B.raw |  |  |  |  |  |  |  |  | 6.5839E4 |  | 1 | 0 | 0 | 0 | 0 | 0 | 0 | 0 | 0 | 1 | 0 | 47 | 56 |  |  | PEAKS DB |
| total 1 peptides |
| --- |

Q76FD9|H4\_DROSE

back to list

  

| Protein Coverage
| Supporting Peptides
|

Protein Coverage:

Supporting Peptides:

| Peptide | Uniq | -10lgP | Mass | Length | ppm | m/z | z | RT | Fraction | Scan | Source File | Area F1 | Area F10 | Area F2\_3 | Area F5 | Area F6 | Area F7A | Area F7B | Area F8A | Area F8B | Area F9 | #Feature | #Feature F1 | #Feature F10 | #Feature F2\_3 | #Feature F5 | #Feature F6 | #Feature F7A | #Feature F7B | #Feature F8A | #Feature F8B | #Feature F9 | Start | End | PTM | AScore | Found By |
| --- | --- | --- | --- | --- | --- | --- | --- | --- | --- | --- | --- | --- | --- | --- | --- | --- | --- | --- | --- | --- | --- | --- | --- | --- | --- | --- | --- | --- | --- | --- | --- | --- | --- | --- | --- | --- | --- |
| R.ISGLIYEETR.G | Y | 66.51 | 1179.6135 | 10 | 0.8 | 590.8145 | 2 | 12.05 | 9 | F9:2894 | DaRuMP\_F8B.raw |  |  |  |  |  |  |  |  | 6.5839E4 |  | 1 | 0 | 0 | 0 | 0 | 0 | 0 | 0 | 0 | 1 | 0 | 47 | 56 |  |  | PEAKS DB |
| total 1 peptides |
| --- |

P62805|H4\_HUMAN

back to list

  

| Protein Coverage
| Supporting Peptides
|

Protein Coverage:

Supporting Peptides:

| Peptide | Uniq | -10lgP | Mass | Length | ppm | m/z | z | RT | Fraction | Scan | Source File | Area F1 | Area F10 | Area F2\_3 | Area F5 | Area F6 | Area F7A | Area F7B | Area F8A | Area F8B | Area F9 | #Feature | #Feature F1 | #Feature F10 | #Feature F2\_3 | #Feature F5 | #Feature F6 | #Feature F7A | #Feature F7B | #Feature F8A | #Feature F8B | #Feature F9 | Start | End | PTM | AScore | Found By |
| --- | --- | --- | --- | --- | --- | --- | --- | --- | --- | --- | --- | --- | --- | --- | --- | --- | --- | --- | --- | --- | --- | --- | --- | --- | --- | --- | --- | --- | --- | --- | --- | --- | --- | --- | --- | --- | --- |
| R.ISGLIYEETR.G | Y | 66.51 | 1179.6135 | 10 | 0.8 | 590.8145 | 2 | 12.05 | 9 | F9:2894 | DaRuMP\_F8B.raw |  |  |  |  |  |  |  |  | 6.5839E4 |  | 1 | 0 | 0 | 0 | 0 | 0 | 0 | 0 | 0 | 1 | 0 | 47 | 56 |  |  | PEAKS DB |
| total 1 peptides |
| --- |

P84044|H4\_DROYA

back to list

  

| Protein Coverage
| Supporting Peptides
|

Protein Coverage:

Supporting Peptides:

| Peptide | Uniq | -10lgP | Mass | Length | ppm | m/z | z | RT | Fraction | Scan | Source File | Area F1 | Area F10 | Area F2\_3 | Area F5 | Area F6 | Area F7A | Area F7B | Area F8A | Area F8B | Area F9 | #Feature | #Feature F1 | #Feature F10 | #Feature F2\_3 | #Feature F5 | #Feature F6 | #Feature F7A | #Feature F7B | #Feature F8A | #Feature F8B | #Feature F9 | Start | End | PTM | AScore | Found By |
| --- | --- | --- | --- | --- | --- | --- | --- | --- | --- | --- | --- | --- | --- | --- | --- | --- | --- | --- | --- | --- | --- | --- | --- | --- | --- | --- | --- | --- | --- | --- | --- | --- | --- | --- | --- | --- | --- |
| R.ISGLIYEETR.G | Y | 66.51 | 1179.6135 | 10 | 0.8 | 590.8145 | 2 | 12.05 | 9 | F9:2894 | DaRuMP\_F8B.raw |  |  |  |  |  |  |  |  | 6.5839E4 |  | 1 | 0 | 0 | 0 | 0 | 0 | 0 | 0 | 0 | 1 | 0 | 47 | 56 |  |  | PEAKS DB |
| total 1 peptides |
| --- |

Q7K8C0|H4\_MYTED

back to list

  

| Protein Coverage
| Supporting Peptides
|

Protein Coverage:

Supporting Peptides:

| Peptide | Uniq | -10lgP | Mass | Length | ppm | m/z | z | RT | Fraction | Scan | Source File | Area F1 | Area F10 | Area F2\_3 | Area F5 | Area F6 | Area F7A | Area F7B | Area F8A | Area F8B | Area F9 | #Feature | #Feature F1 | #Feature F10 | #Feature F2\_3 | #Feature F5 | #Feature F6 | #Feature F7A | #Feature F7B | #Feature F8A | #Feature F8B | #Feature F9 | Start | End | PTM | AScore | Found By |
| --- | --- | --- | --- | --- | --- | --- | --- | --- | --- | --- | --- | --- | --- | --- | --- | --- | --- | --- | --- | --- | --- | --- | --- | --- | --- | --- | --- | --- | --- | --- | --- | --- | --- | --- | --- | --- | --- |
| R.ISGLIYEETR.G | Y | 66.51 | 1179.6135 | 10 | 0.8 | 590.8145 | 2 | 12.05 | 9 | F9:2894 | DaRuMP\_F8B.raw |  |  |  |  |  |  |  |  | 6.5839E4 |  | 1 | 0 | 0 | 0 | 0 | 0 | 0 | 0 | 0 | 1 | 0 | 47 | 56 |  |  | PEAKS DB |
| total 1 peptides |
| --- |

P0DJL4|SLA\_DABPA

back to list

  

| Protein Coverage
| Supporting Peptides
|

Protein Coverage:

Supporting Peptides:

| Peptide | Uniq | -10lgP | Mass | Length | ppm | m/z | z | RT | Fraction | Scan | Source File | Area F1 | Area F10 | Area F2\_3 | Area F5 | Area F6 | Area F7A | Area F7B | Area F8A | Area F8B | Area F9 | #Feature | #Feature F1 | #Feature F10 | #Feature F2\_3 | #Feature F5 | #Feature F6 | #Feature F7A | #Feature F7B | #Feature F8A | #Feature F8B | #Feature F9 | Start | End | PTM | AScore | Found By |
| --- | --- | --- | --- | --- | --- | --- | --- | --- | --- | --- | --- | --- | --- | --- | --- | --- | --- | --- | --- | --- | --- | --- | --- | --- | --- | --- | --- | --- | --- | --- | --- | --- | --- | --- | --- | --- | --- |
| L.PGWSFYEGNC(+57.02)YK.A | Y | 63.79 | 1506.6238 | 12 | 0.4 | 754.3195 | 2 | 14.20 | 7 | F7:4732 | DaRuMP\_F7B.raw |  |  |  |  |  |  | 2.3103E5 |  |  |  | 1 | 0 | 0 | 0 | 0 | 0 | 0 | 1 | 0 | 0 | 0 | 6 | 17 | Carbamidomethylation | C10:Carbamidomethylation:1000.00 | PEAKS DB |
| total 1 peptides |
| --- |

P82993|AMYB\_HORVS

back to list

  

| Protein Coverage
| Supporting Peptides
|

Protein Coverage:

Supporting Peptides:

| Peptide | Uniq | -10lgP | Mass | Length | ppm | m/z | z | RT | Fraction | Scan | Source File | Area F1 | Area F10 | Area F2\_3 | Area F5 | Area F6 | Area F7A | Area F7B | Area F8A | Area F8B | Area F9 | #Feature | #Feature F1 | #Feature F10 | #Feature F2\_3 | #Feature F5 | #Feature F6 | #Feature F7A | #Feature F7B | #Feature F8A | #Feature F8B | #Feature F9 | Start | End | PTM | AScore | Found By |
| --- | --- | --- | --- | --- | --- | --- | --- | --- | --- | --- | --- | --- | --- | --- | --- | --- | --- | --- | --- | --- | --- | --- | --- | --- | --- | --- | --- | --- | --- | --- | --- | --- | --- | --- | --- | --- | --- |
| R.YDPTAYNTILR.N | Y | 62.40 | 1325.6615 | 11 | 0.2 | 663.8381 | 2 | 13.92 | 7 | F7:4501 | DaRuMP\_F7B.raw |  |  |  |  |  |  | 1.7334E5 |  |  |  | 1 | 0 | 0 | 0 | 0 | 0 | 0 | 1 | 0 | 0 | 0 | 384 | 394 |  |  | PEAKS DB |
| total 1 peptides |
| --- |

P16098|AMYB\_HORVU

back to list

  

| Protein Coverage
| Supporting Peptides
|

Protein Coverage:

Supporting Peptides:

| Peptide | Uniq | -10lgP | Mass | Length | ppm | m/z | z | RT | Fraction | Scan | Source File | Area F1 | Area F10 | Area F2\_3 | Area F5 | Area F6 | Area F7A | Area F7B | Area F8A | Area F8B | Area F9 | #Feature | #Feature F1 | #Feature F10 | #Feature F2\_3 | #Feature F5 | #Feature F6 | #Feature F7A | #Feature F7B | #Feature F8A | #Feature F8B | #Feature F9 | Start | End | PTM | AScore | Found By |
| --- | --- | --- | --- | --- | --- | --- | --- | --- | --- | --- | --- | --- | --- | --- | --- | --- | --- | --- | --- | --- | --- | --- | --- | --- | --- | --- | --- | --- | --- | --- | --- | --- | --- | --- | --- | --- | --- |
| R.YDPTAYNTILR.N | Y | 62.40 | 1325.6615 | 11 | 0.2 | 663.8381 | 2 | 13.92 | 7 | F7:4501 | DaRuMP\_F7B.raw |  |  |  |  |  |  | 1.7334E5 |  |  |  | 1 | 0 | 0 | 0 | 0 | 0 | 0 | 1 | 0 | 0 | 0 | 384 | 394 |  |  | PEAKS DB |
| total 1 peptides |
| --- |

P30271|AMYB\_SECCE

back to list

  

| Protein Coverage
| Supporting Peptides
|

Protein Coverage:

Supporting Peptides:

| Peptide | Uniq | -10lgP | Mass | Length | ppm | m/z | z | RT | Fraction | Scan | Source File | Area F1 | Area F10 | Area F2\_3 | Area F5 | Area F6 | Area F7A | Area F7B | Area F8A | Area F8B | Area F9 | #Feature | #Feature F1 | #Feature F10 | #Feature F2\_3 | #Feature F5 | #Feature F6 | #Feature F7A | #Feature F7B | #Feature F8A | #Feature F8B | #Feature F9 | Start | End | PTM | AScore | Found By |
| --- | --- | --- | --- | --- | --- | --- | --- | --- | --- | --- | --- | --- | --- | --- | --- | --- | --- | --- | --- | --- | --- | --- | --- | --- | --- | --- | --- | --- | --- | --- | --- | --- | --- | --- | --- | --- | --- |
| R.YDPTAYNTILR.N | Y | 62.40 | 1325.6615 | 11 | 0.2 | 663.8381 | 2 | 13.92 | 7 | F7:4501 | DaRuMP\_F7B.raw |  |  |  |  |  |  | 1.7334E5 |  |  |  | 1 | 0 | 0 | 0 | 0 | 0 | 0 | 1 | 0 | 0 | 0 | 80 | 90 |  |  | PEAKS DB |
| total 1 peptides |
| --- |

P17314|IAAC3\_WHEAT

back to list

  

| Protein Coverage
| Supporting Peptides
|

Protein Coverage:

Supporting Peptides:

| Peptide | Uniq | -10lgP | Mass | Length | ppm | m/z | z | RT | Fraction | Scan | Source File | Area F1 | Area F10 | Area F2\_3 | Area F5 | Area F6 | Area F7A | Area F7B | Area F8A | Area F8B | Area F9 | #Feature | #Feature F1 | #Feature F10 | #Feature F2\_3 | #Feature F5 | #Feature F6 | #Feature F7A | #Feature F7B | #Feature F8A | #Feature F8B | #Feature F9 | Start | End | PTM | AScore | Found By |
| --- | --- | --- | --- | --- | --- | --- | --- | --- | --- | --- | --- | --- | --- | --- | --- | --- | --- | --- | --- | --- | --- | --- | --- | --- | --- | --- | --- | --- | --- | --- | --- | --- | --- | --- | --- | --- | --- |
| R.SGNVGESGLIDLPGC(+57.02)PR.E | Y | 62.37 | 1726.8308 | 17 | 1.2 | 864.4237 | 2 | 15.03 | 7 | F7:5444 | DaRuMP\_F7B.raw |  |  |  |  |  |  | 1.8949E5 |  |  |  | 1 | 0 | 0 | 0 | 0 | 0 | 0 | 1 | 0 | 0 | 0 | 116 | 132 | Carbamidomethylation | C15:Carbamidomethylation:1000.00 | PEAKS DB |
| total 1 peptides |
| --- |

P05661|MYSA\_DROME

back to list

  

| Protein Coverage
| Supporting Peptides
|

Protein Coverage:

Supporting Peptides:

| Peptide | Uniq | -10lgP | Mass | Length | ppm | m/z | z | RT | Fraction | Scan | Source File | Area F1 | Area F10 | Area F2\_3 | Area F5 | Area F6 | Area F7A | Area F7B | Area F8A | Area F8B | Area F9 | #Feature | #Feature F1 | #Feature F10 | #Feature F2\_3 | #Feature F5 | #Feature F6 | #Feature F7A | #Feature F7B | #Feature F8A | #Feature F8B | #Feature F9 | Start | End | PTM | AScore | Found By |
| --- | --- | --- | --- | --- | --- | --- | --- | --- | --- | --- | --- | --- | --- | --- | --- | --- | --- | --- | --- | --- | --- | --- | --- | --- | --- | --- | --- | --- | --- | --- | --- | --- | --- | --- | --- | --- | --- |
| R.QIEEAEEIAALNLAK.F | Y | 60.97 | 1640.8621 | 15 | 1.3 | 821.4394 | 2 | 20.19 | 9 | F9:7651 | DaRuMP\_F8B.raw |  |  |  |  |  |  |  |  | 4.0995E4 |  | 1 | 0 | 0 | 0 | 0 | 0 | 0 | 0 | 0 | 1 | 0 | 1880 | 1894 |  |  | PEAKS DB |
| total 1 peptides |
| --- |

P05109|S10A8\_HUMAN

back to list

  

| Protein Coverage
| Supporting Peptides
|

Protein Coverage:

Supporting Peptides:
[truncated: 5,466 more chars]
