# Supplementary figures and images for: Biogeographic venom variation in Russell’s viper (Daboia russelii) and the preclinical inefficacy of antivenom therapy in snakebite hotspots (part 2 of 2)
Source: PLoS Negl Trop Dis. 2021 Mar 25;15(3):e0009247. doi: 10.1371/journal.pntd.0009247 (PMC7993602; doi:10.1371/journal.pntd.0009247)

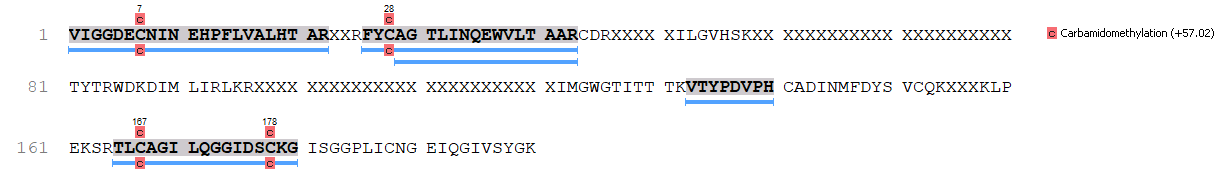

Supplement: S1 Data — (ZIP) [file pntd.0009247.s013.zip › D. russelii_West Bengal/img/cov_12.png]

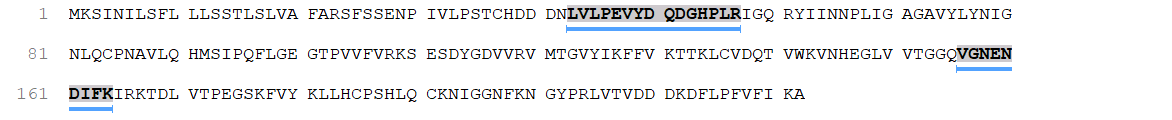

Supplement: S1 Data — (ZIP) [file pntd.0009247.s013.zip › D. russelii_West Bengal/img/cov_120.png]

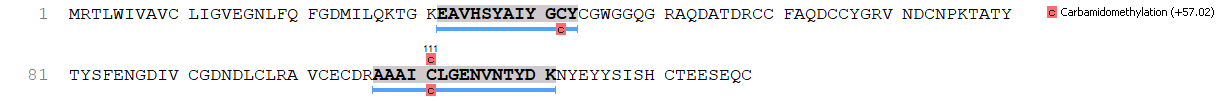

Supplement: S1 Data — (ZIP) [file pntd.0009247.s013.zip › D. russelii_West Bengal/img/cov_127.png]

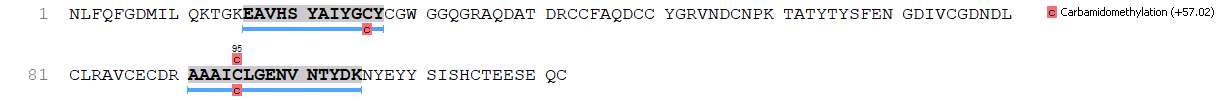

Supplement: S1 Data — (ZIP) [file pntd.0009247.s013.zip › D. russelii_West Bengal/img/cov_128.png]

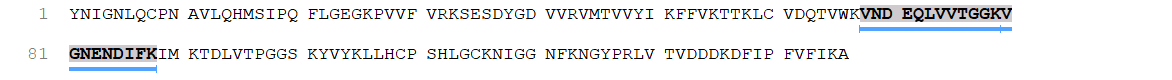

Supplement: S1 Data — (ZIP) [file pntd.0009247.s013.zip › D. russelii_West Bengal/img/cov_130.png]

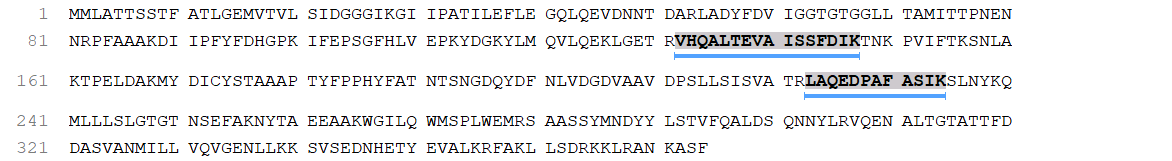

Supplement: S1 Data — (ZIP) [file pntd.0009247.s013.zip › D. russelii_West Bengal/img/cov_137.png]

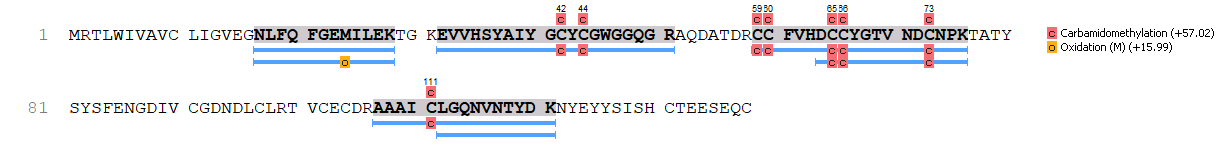

Supplement: S1 Data — (ZIP) [file pntd.0009247.s013.zip › D. russelii_West Bengal/img/cov_15.png]

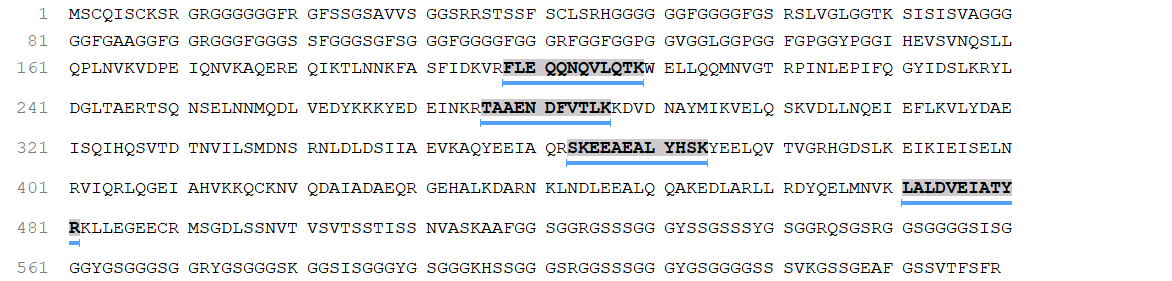

Supplement: S1 Data — (ZIP) [file pntd.0009247.s013.zip › D. russelii_West Bengal/img/cov_16.png]

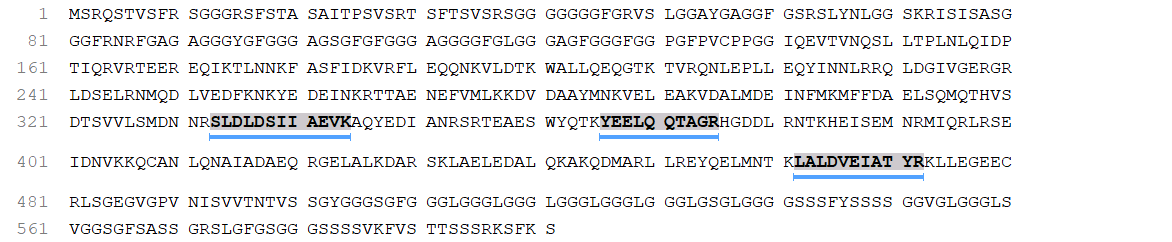

Supplement: S1 Data — (ZIP) [file pntd.0009247.s013.zip › D. russelii_West Bengal/img/cov_19.png]

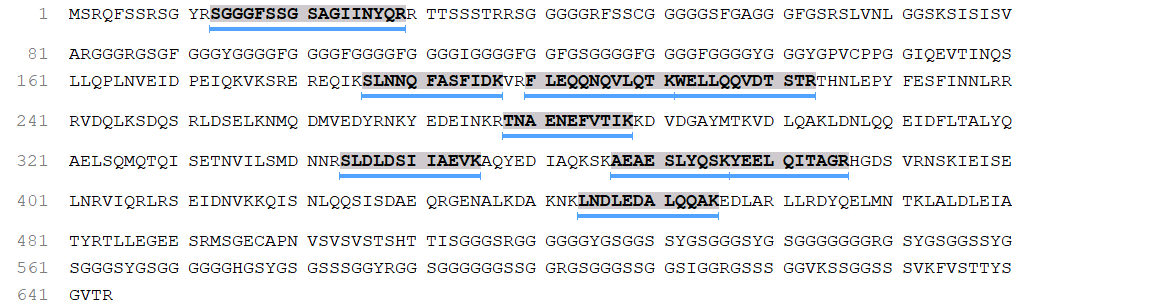

Supplement: S1 Data — (ZIP) [file pntd.0009247.s013.zip › D. russelii_West Bengal/img/cov_2.png]

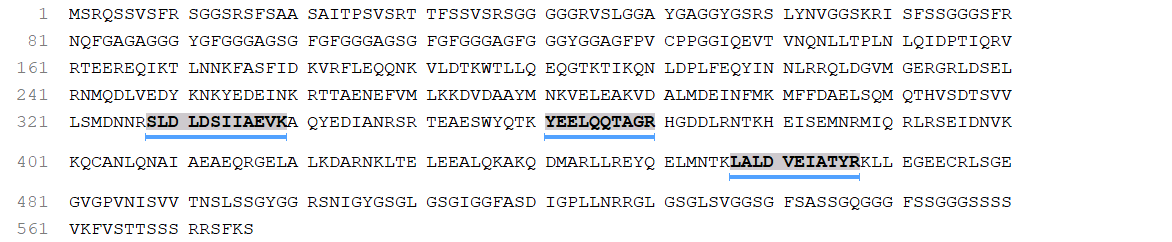

Supplement: S1 Data — (ZIP) [file pntd.0009247.s013.zip › D. russelii_West Bengal/img/cov_20.png]

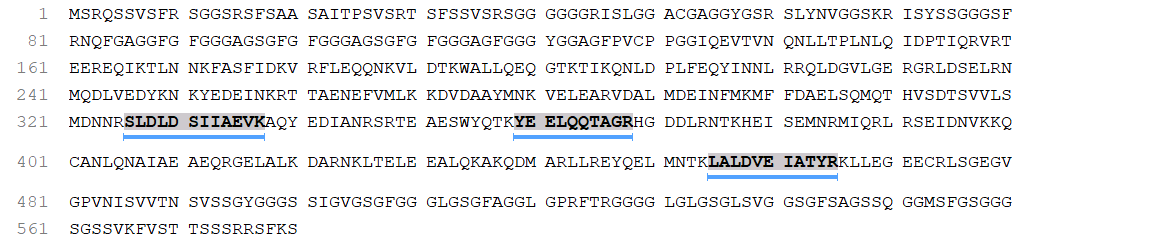

Supplement: S1 Data — (ZIP) [file pntd.0009247.s013.zip › D. russelii_West Bengal/img/cov_21.png]

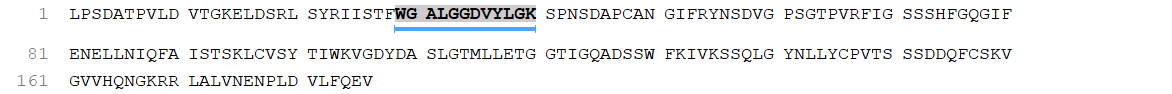

Supplement: S1 Data — (ZIP) [file pntd.0009247.s013.zip › D. russelii_West Bengal/img/cov_214.png]

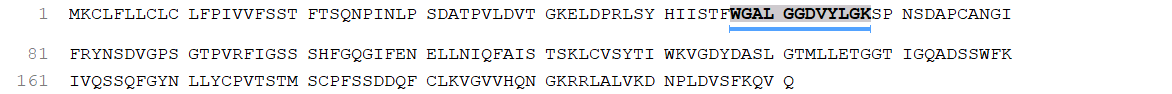

Supplement: S1 Data — (ZIP) [file pntd.0009247.s013.zip › D. russelii_West Bengal/img/cov_215.png]

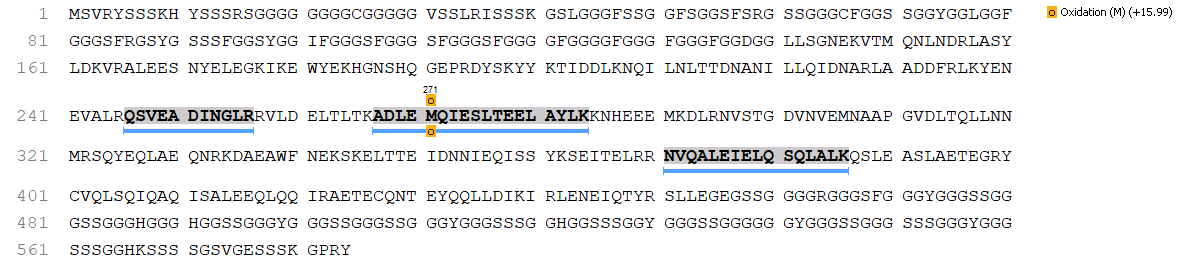

Supplement: S1 Data — (ZIP) [file pntd.0009247.s013.zip › D. russelii_West Bengal/img/cov_23.png]

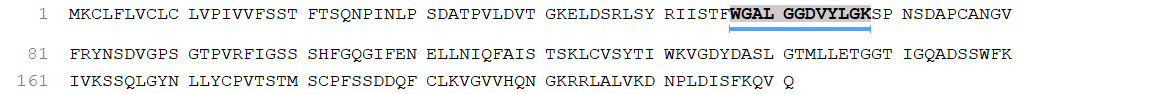

Supplement: S1 Data — (ZIP) [file pntd.0009247.s013.zip › D. russelii_West Bengal/img/cov_230.png]

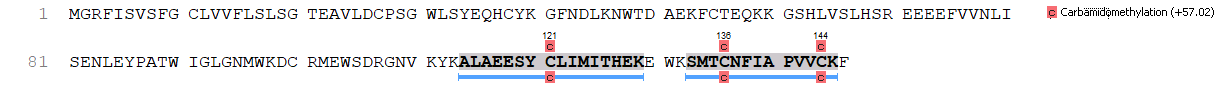

Supplement: S1 Data — (ZIP) [file pntd.0009247.s013.zip › D. russelii_West Bengal/img/cov_233.png]

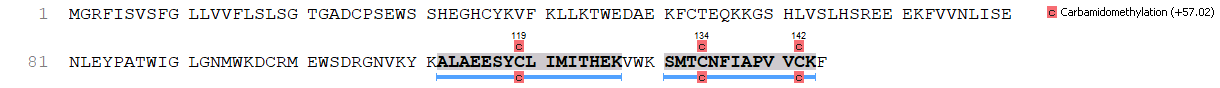

Supplement: S1 Data — (ZIP) [file pntd.0009247.s013.zip › D. russelii_West Bengal/img/cov_234.png]

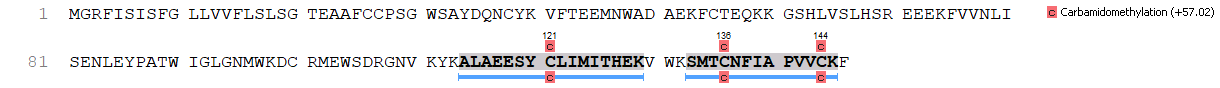

Supplement: S1 Data — (ZIP) [file pntd.0009247.s013.zip › D. russelii_West Bengal/img/cov_235.png]

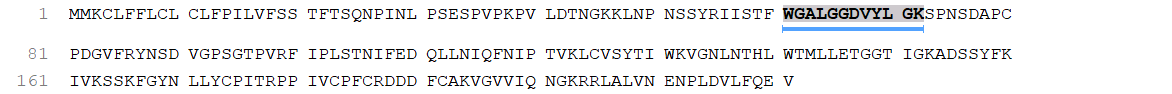

Supplement: S1 Data — (ZIP) [file pntd.0009247.s013.zip › D. russelii_West Bengal/img/cov_236.png]

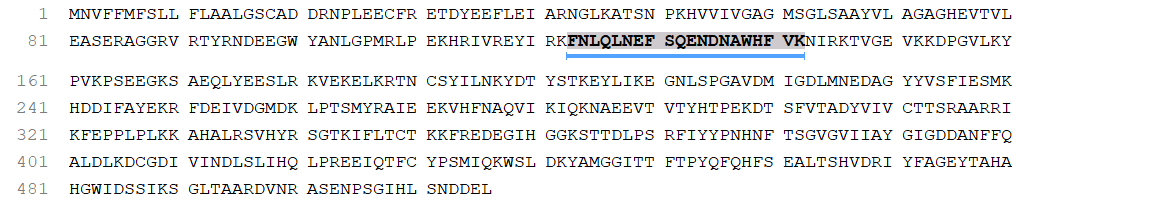

Supplement: S1 Data — (ZIP) [file pntd.0009247.s013.zip › D. russelii_West Bengal/img/cov_237.png]

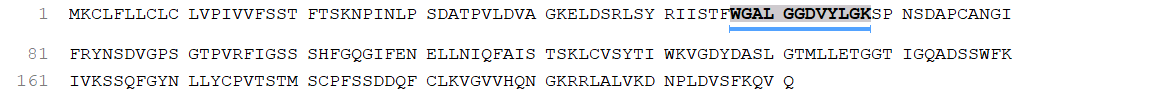

Supplement: S1 Data — (ZIP) [file pntd.0009247.s013.zip › D. russelii_West Bengal/img/cov_249.png]

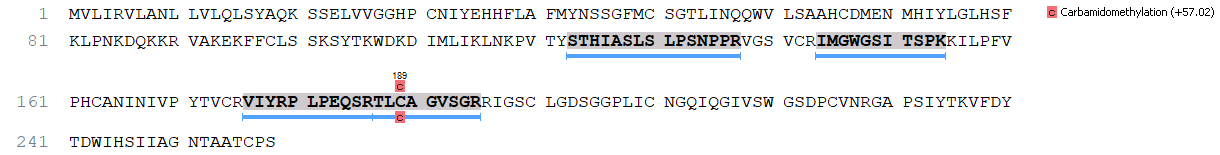

Supplement: S1 Data — (ZIP) [file pntd.0009247.s013.zip › D. russelii_West Bengal/img/cov_25.png]

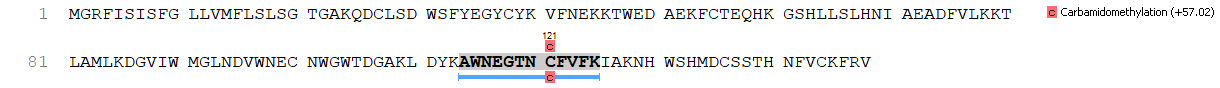

Supplement: S1 Data — (ZIP) [file pntd.0009247.s013.zip › D. russelii_West Bengal/img/cov_261.png]

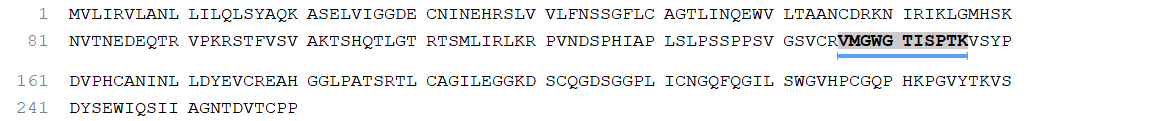

Supplement: S1 Data — (ZIP) [file pntd.0009247.s013.zip › D. russelii_West Bengal/img/cov_266.png]

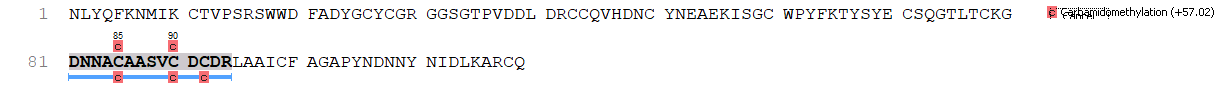

Supplement: S1 Data — (ZIP) [file pntd.0009247.s013.zip › D. russelii_West Bengal/img/cov_270.png]

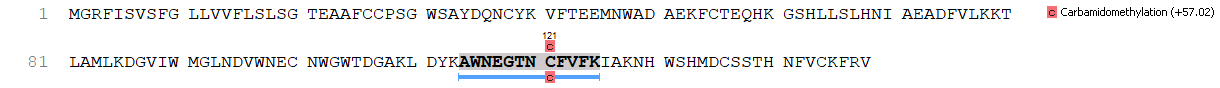

Supplement: S1 Data — (ZIP) [file pntd.0009247.s013.zip › D. russelii_West Bengal/img/cov_272.png]

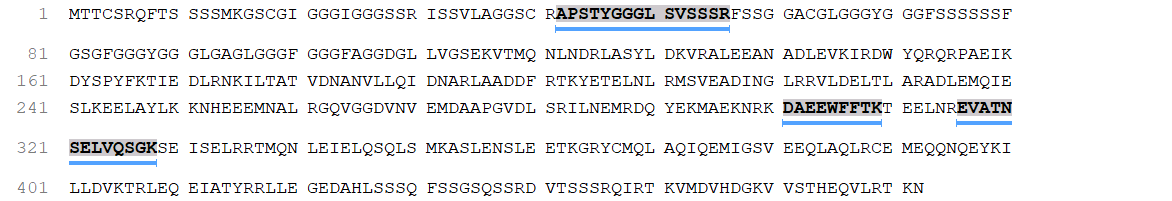

Supplement: S1 Data — (ZIP) [file pntd.0009247.s013.zip › D. russelii_West Bengal/img/cov_28.png]

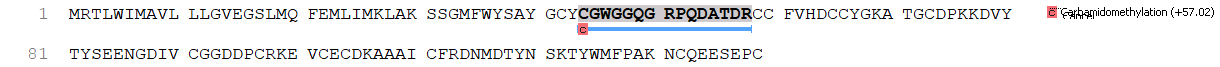

Supplement: S1 Data — (ZIP) [file pntd.0009247.s013.zip › D. russelii_West Bengal/img/cov_287.png]

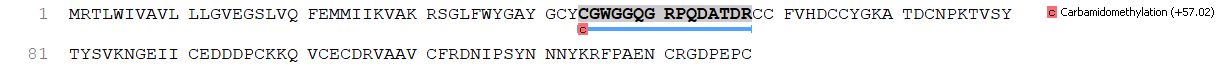

Supplement: S1 Data — (ZIP) [file pntd.0009247.s013.zip › D. russelii_West Bengal/img/cov_293.png]

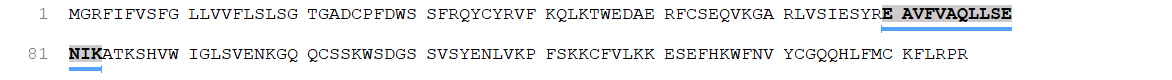

Supplement: S1 Data — (ZIP) [file pntd.0009247.s013.zip › D. russelii_West Bengal/img/cov_294.png]

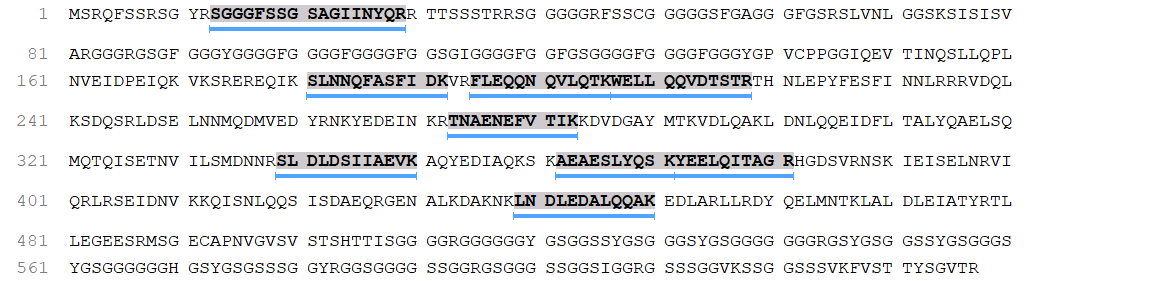

Supplement: S1 Data — (ZIP) [file pntd.0009247.s013.zip › D. russelii_West Bengal/img/cov_3.png]

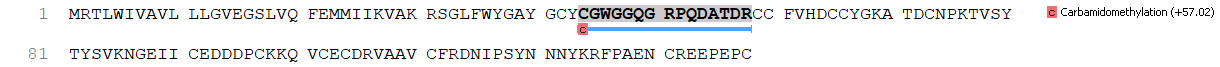

Supplement: S1 Data — (ZIP) [file pntd.0009247.s013.zip › D. russelii_West Bengal/img/cov_308.png]

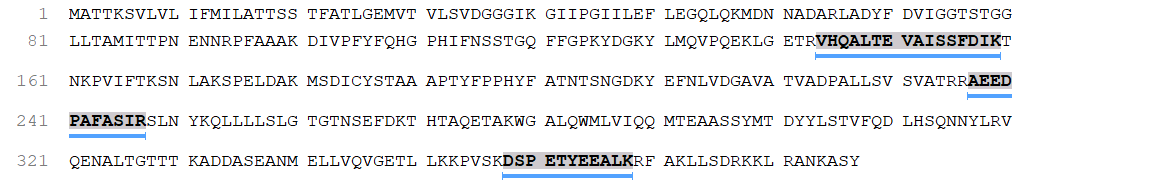

Supplement: S1 Data — (ZIP) [file pntd.0009247.s013.zip › D. russelii_West Bengal/img/cov_31.png]

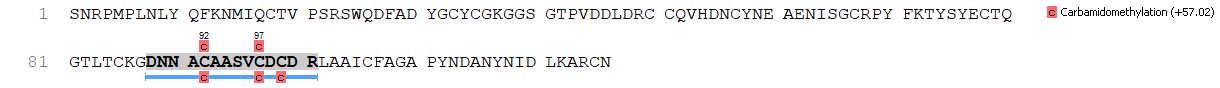

Supplement: S1 Data — (ZIP) [file pntd.0009247.s013.zip › D. russelii_West Bengal/img/cov_315.png]

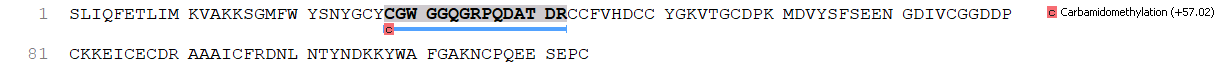

Supplement: S1 Data — (ZIP) [file pntd.0009247.s013.zip › D. russelii_West Bengal/img/cov_318.png]

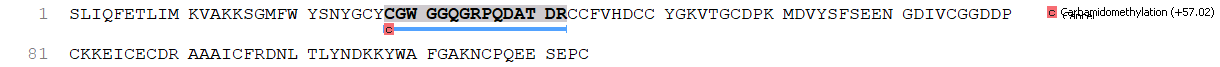

Supplement: S1 Data — (ZIP) [file pntd.0009247.s013.zip › D. russelii_West Bengal/img/cov_319.png]

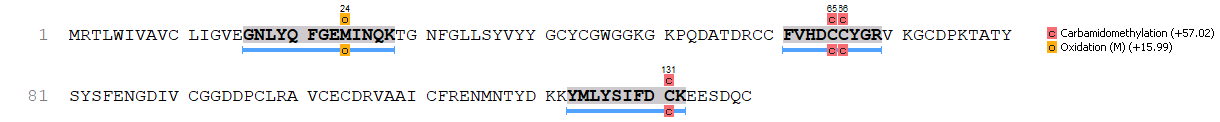

Supplement: S1 Data — (ZIP) [file pntd.0009247.s013.zip › D. russelii_West Bengal/img/cov_32.png]

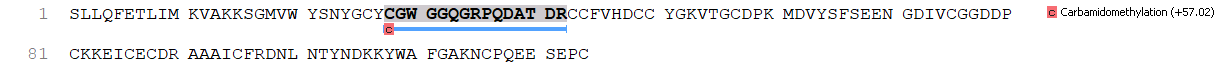

Supplement: S1 Data — (ZIP) [file pntd.0009247.s013.zip › D. russelii_West Bengal/img/cov_320.png]

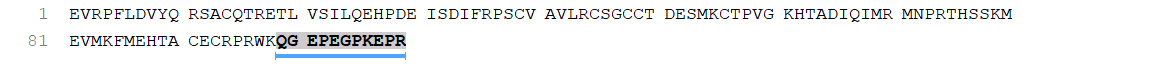

Supplement: S1 Data — (ZIP) [file pntd.0009247.s013.zip › D. russelii_West Bengal/img/cov_325.png]

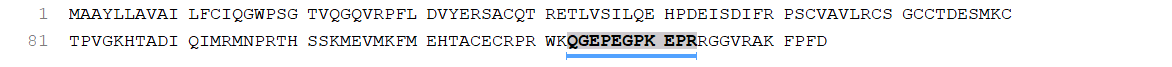

Supplement: S1 Data — (ZIP) [file pntd.0009247.s013.zip › D. russelii_West Bengal/img/cov_326.png]

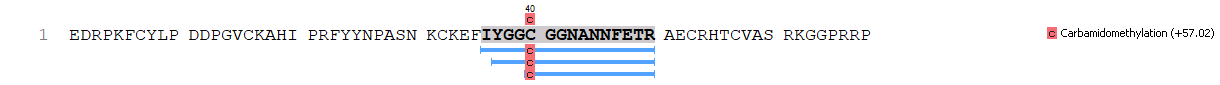

Supplement: S1 Data — (ZIP) [file pntd.0009247.s013.zip › D. russelii_West Bengal/img/cov_330.png]

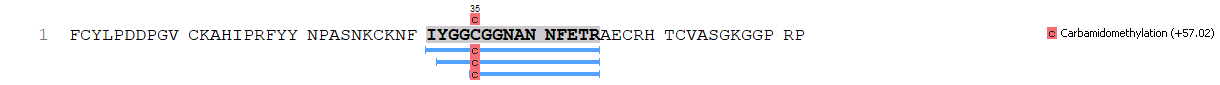

Supplement: S1 Data — (ZIP) [file pntd.0009247.s013.zip › D. russelii_West Bengal/img/cov_333.png]

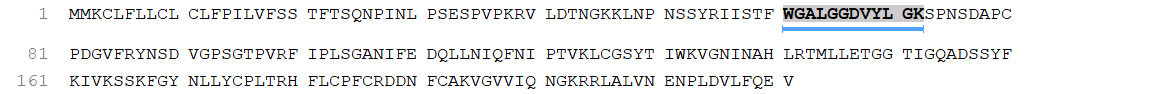

Supplement: S1 Data — (ZIP) [file pntd.0009247.s013.zip › D. russelii_West Bengal/img/cov_336.png]

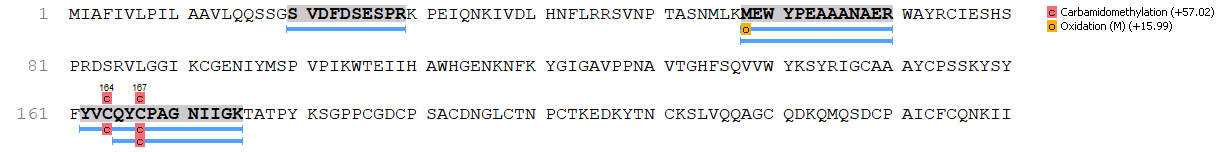

Supplement: S1 Data — (ZIP) [file pntd.0009247.s013.zip › D. russelii_West Bengal/img/cov_34.png]

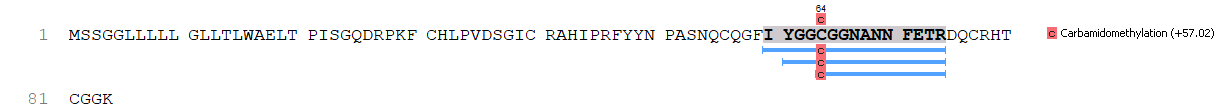

Supplement: S1 Data — (ZIP) [file pntd.0009247.s013.zip › D. russelii_West Bengal/img/cov_341.png]

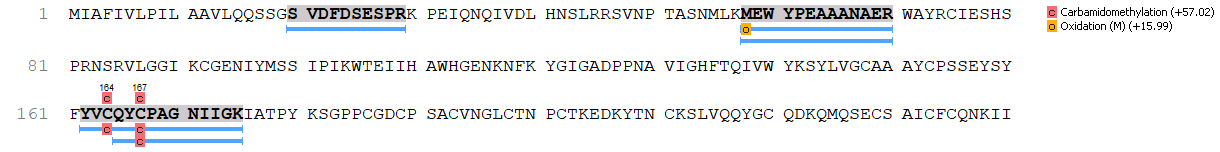

Supplement: S1 Data — (ZIP) [file pntd.0009247.s013.zip › D. russelii_West Bengal/img/cov_35.png]

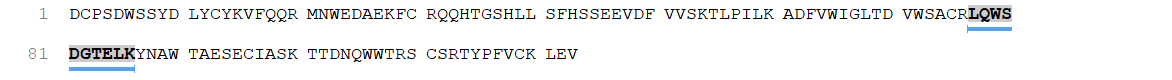

Supplement: S1 Data — (ZIP) [file pntd.0009247.s013.zip › D. russelii_West Bengal/img/cov_357.png]

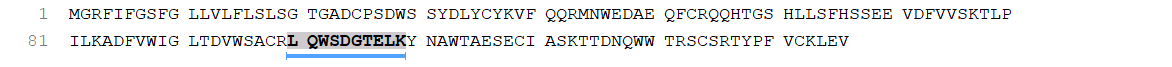

Supplement: S1 Data — (ZIP) [file pntd.0009247.s013.zip › D. russelii_West Bengal/img/cov_358.png]

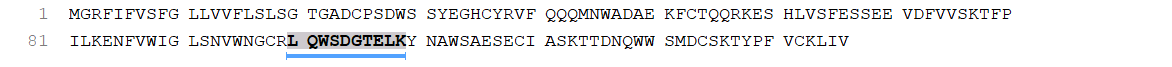

Supplement: S1 Data — (ZIP) [file pntd.0009247.s013.zip › D. russelii_West Bengal/img/cov_369.png]

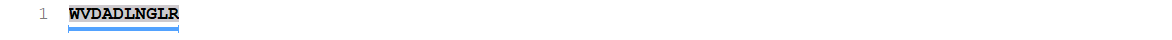

Supplement: S1 Data — (ZIP) [file pntd.0009247.s013.zip › D. russelii_West Bengal/img/cov_370.png]

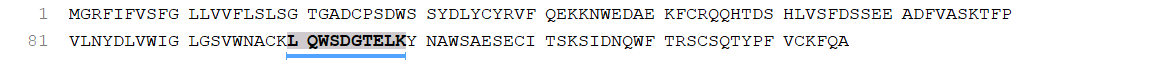

Supplement: S1 Data — (ZIP) [file pntd.0009247.s013.zip › D. russelii_West Bengal/img/cov_380.png]

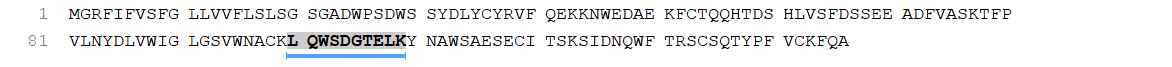

Supplement: S1 Data — (ZIP) [file pntd.0009247.s013.zip › D. russelii_West Bengal/img/cov_381.png]

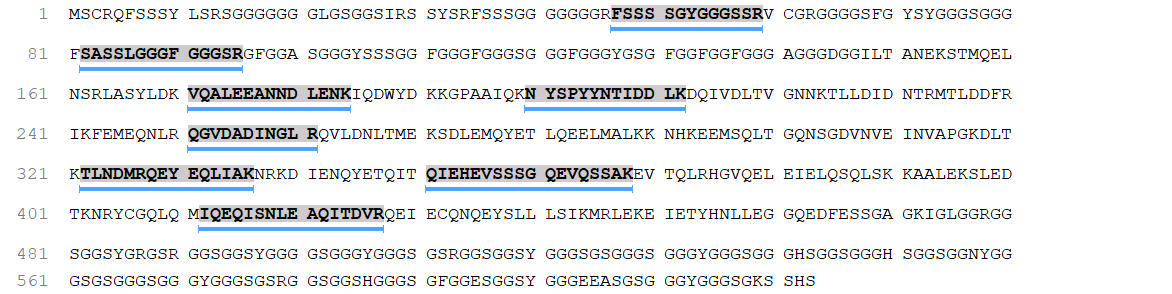

Supplement: S1 Data — (ZIP) [file pntd.0009247.s013.zip › D. russelii_West Bengal/img/cov_4.png]

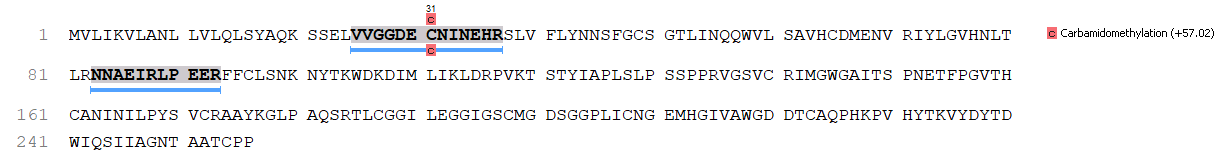

Supplement: S1 Data — (ZIP) [file pntd.0009247.s013.zip › D. russelii_West Bengal/img/cov_44.png]

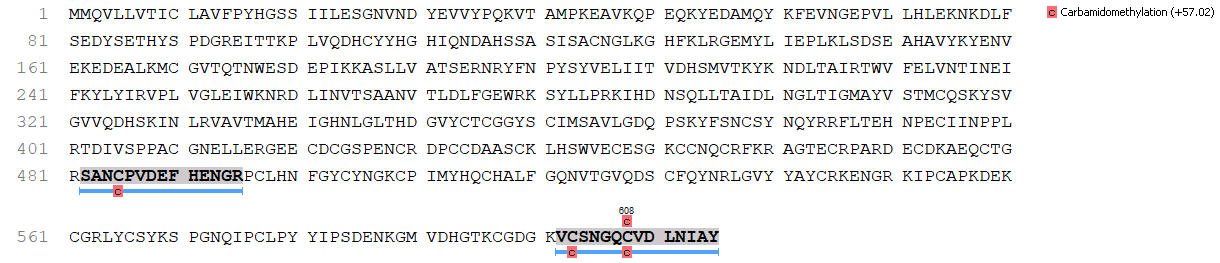

Supplement: S1 Data — (ZIP) [file pntd.0009247.s013.zip › D. russelii_West Bengal/img/cov_46.png]

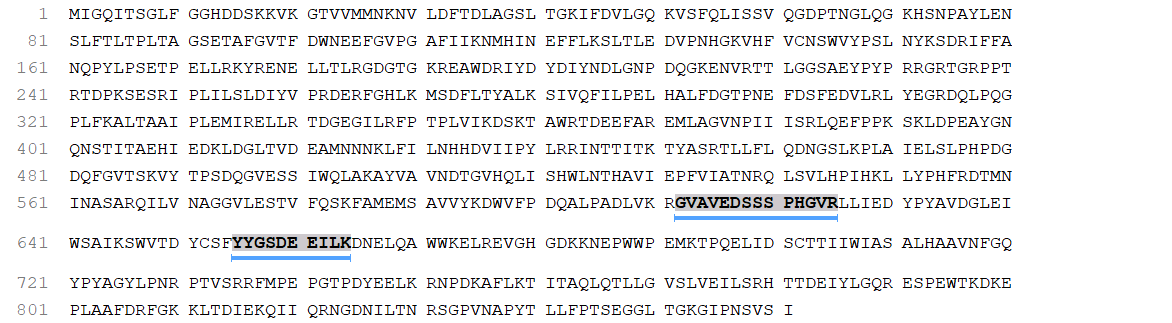

Supplement: S1 Data — (ZIP) [file pntd.0009247.s013.zip › D. russelii_West Bengal/img/cov_48.png]

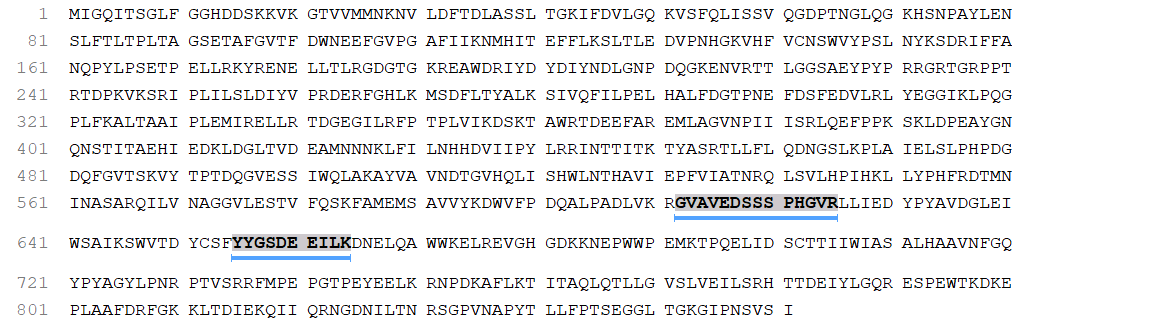

Supplement: S1 Data — (ZIP) [file pntd.0009247.s013.zip › D. russelii_West Bengal/img/cov_50.png]

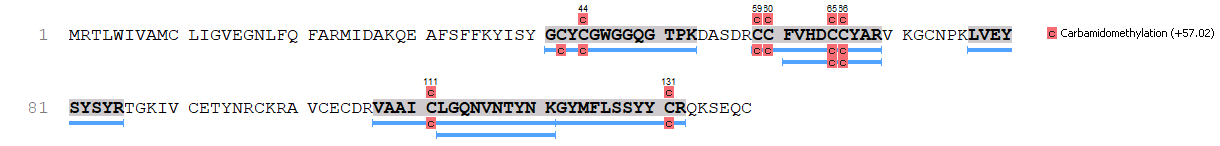

Supplement: S1 Data — (ZIP) [file pntd.0009247.s013.zip › D. russelii_West Bengal/img/cov_6.png]

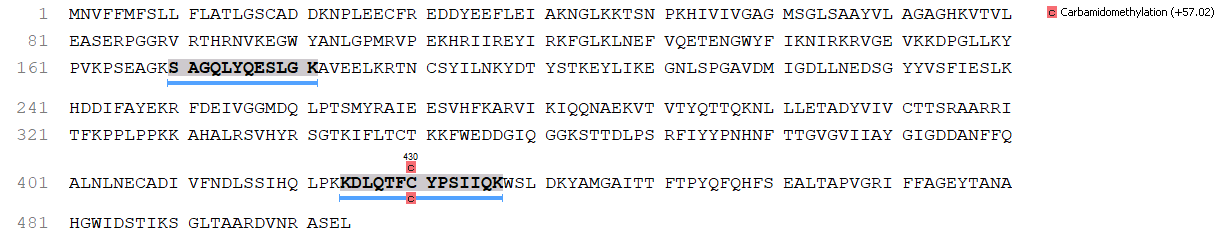

Supplement: S1 Data — (ZIP) [file pntd.0009247.s013.zip › D. russelii_West Bengal/img/cov_61.png]

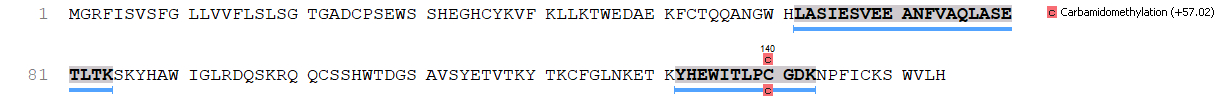

Supplement: S1 Data — (ZIP) [file pntd.0009247.s013.zip › D. russelii_West Bengal/img/cov_66.png]

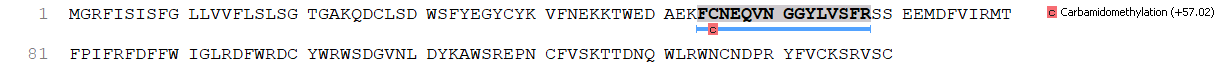

Supplement: S1 Data — (ZIP) [file pntd.0009247.s013.zip › D. russelii_West Bengal/img/cov_67.png]

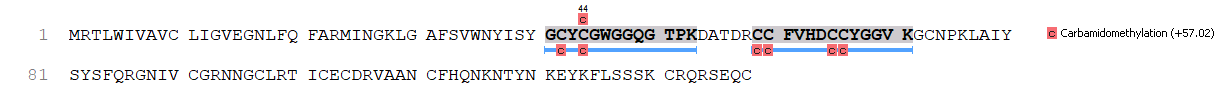

Supplement: S1 Data — (ZIP) [file pntd.0009247.s013.zip › D. russelii_West Bengal/img/cov_68.png]

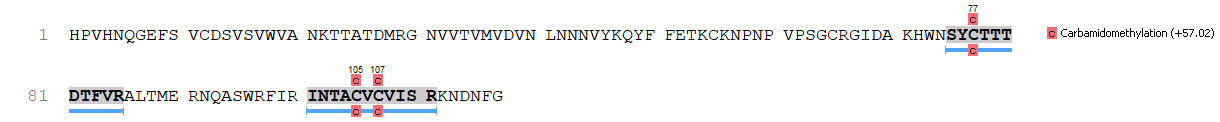

Supplement: S1 Data — (ZIP) [file pntd.0009247.s013.zip › D. russelii_West Bengal/img/cov_69.png]

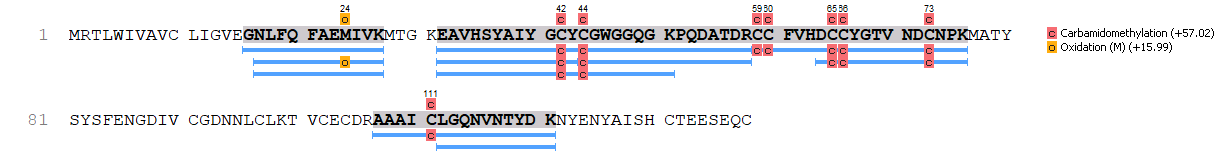

Supplement: S1 Data — (ZIP) [file pntd.0009247.s013.zip › D. russelii_West Bengal/img/cov_7.png]

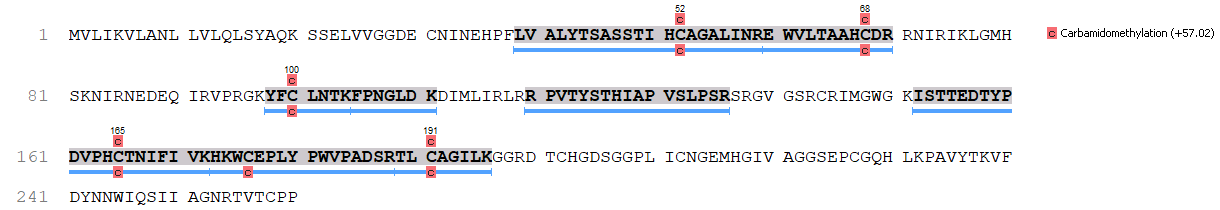

Supplement: S1 Data — (ZIP) [file pntd.0009247.s013.zip › D. russelii_West Bengal/img/cov_8.png]

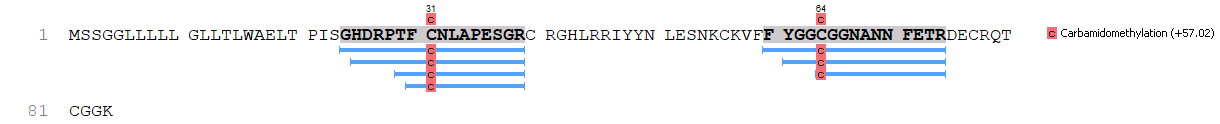

Supplement: S1 Data — (ZIP) [file pntd.0009247.s013.zip › D. russelii_West Bengal/img/cov_81.png]

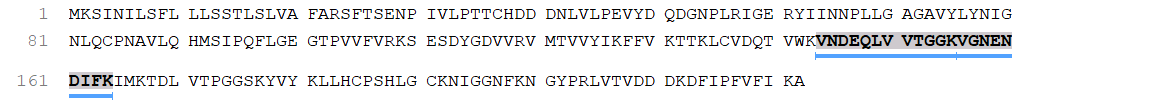

Supplement: S1 Data — (ZIP) [file pntd.0009247.s013.zip › D. russelii_West Bengal/img/cov_88.png]

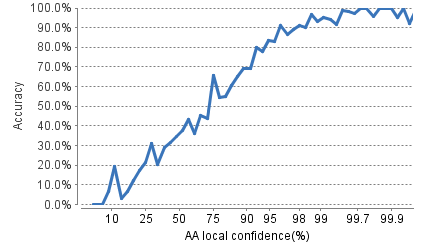

Supplement: S1 Data — (ZIP) [file pntd.0009247.s013.zip › D. russelii_West Bengal/img/DenovoFDRCurveFigure3380592265228111404.png]

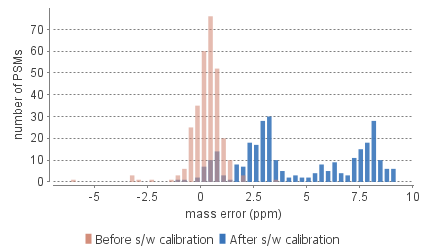

Supplement: S1 Data — (ZIP) [file pntd.0009247.s013.zip › D. russelii_West Bengal/img/ErrorCalibratedHistogram4177947474901191017.png]

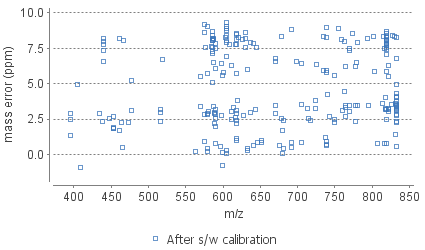

Supplement: S1 Data — (ZIP) [file pntd.0009247.s013.zip › D. russelii_West Bengal/img/ErrorPlotFigure2654755660432654142.png]

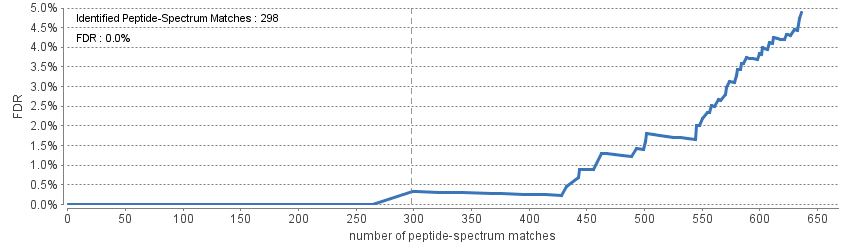

Supplement: S1 Data — (ZIP) [file pntd.0009247.s013.zip › D. russelii_West Bengal/img/FDRFigure2379377012917912895.png]

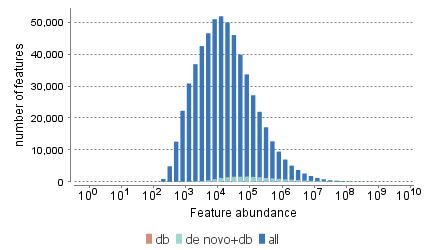

Supplement: S1 Data — (ZIP) [file pntd.0009247.s013.zip › D. russelii_West Bengal/img/FeatureIntensityDistributionHistogram8084489395598381403.png]

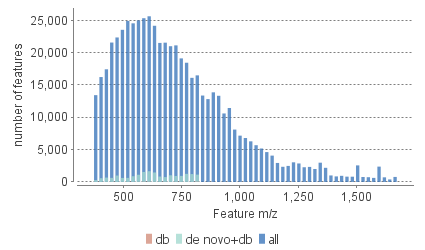

Supplement: S1 Data — (ZIP) [file pntd.0009247.s013.zip › D. russelii_West Bengal/img/FeatureMzHistogram5368355355086653766.png]

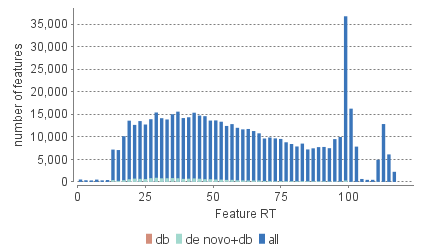

Supplement: S1 Data — (ZIP) [file pntd.0009247.s013.zip › D. russelii_West Bengal/img/FeatureRtHistogram1106136461435507072.png]

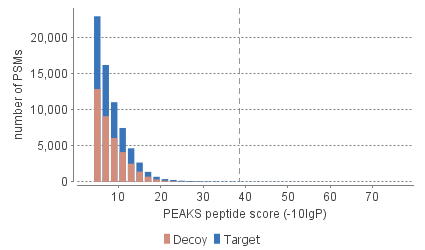

Supplement: S1 Data — (ZIP) [file pntd.0009247.s013.zip › D. russelii_West Bengal/img/ScoreHistogram3985976929289802520.png]

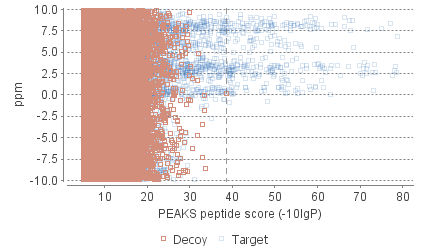

Supplement: S1 Data — (ZIP) [file pntd.0009247.s013.zip › D. russelii_West Bengal/img/ScorePlotFigure8367473231727960011.png]

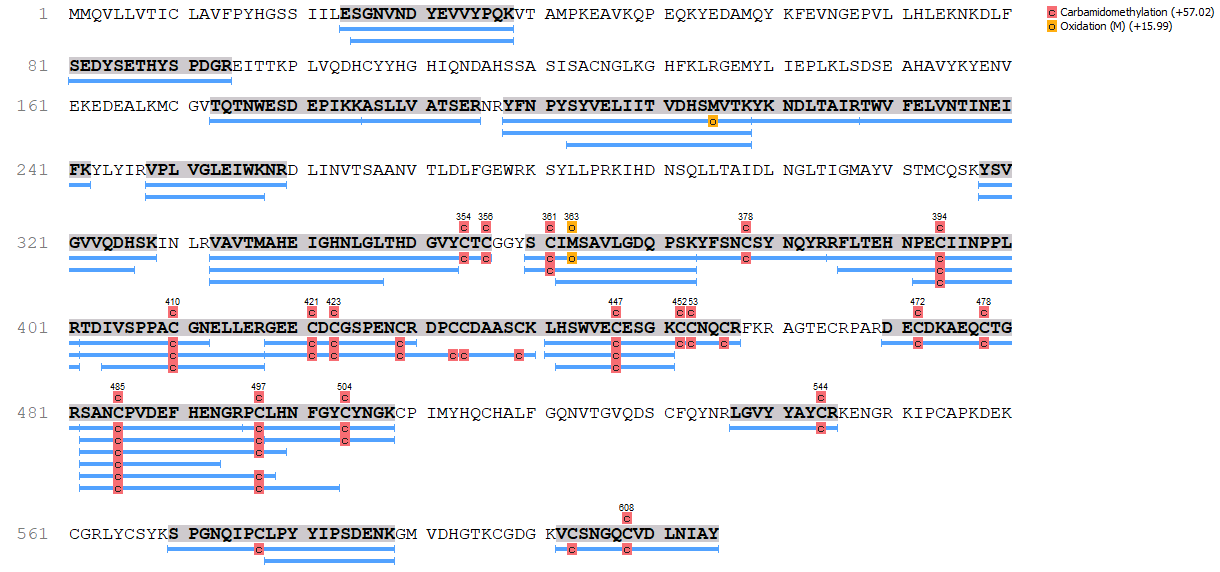

Supplement: S1 Data — (ZIP) [file pntd.0009247.s013.zip › D. russelii_Madhya Pradesh/img/cov_1.png]

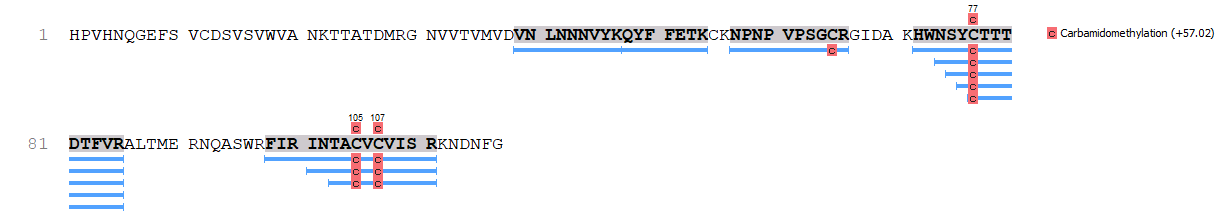

Supplement: S1 Data — (ZIP) [file pntd.0009247.s013.zip › D. russelii_Madhya Pradesh/img/cov_100.png]

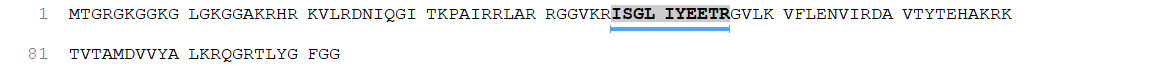

Supplement: S1 Data — (ZIP) [file pntd.0009247.s013.zip › D. russelii_Madhya Pradesh/img/cov_1000.png]

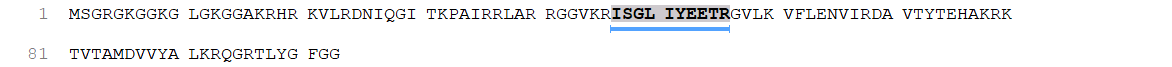

Supplement: S1 Data — (ZIP) [file pntd.0009247.s013.zip › D. russelii_Madhya Pradesh/img/cov_1002.png]

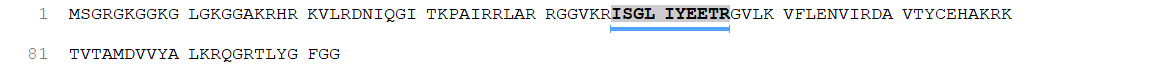

Supplement: S1 Data — (ZIP) [file pntd.0009247.s013.zip › D. russelii_Madhya Pradesh/img/cov_1005.png]

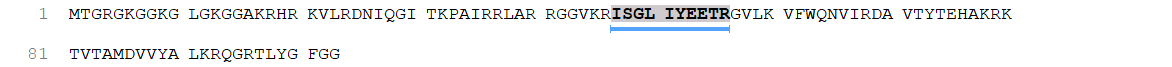

Supplement: S1 Data — (ZIP) [file pntd.0009247.s013.zip › D. russelii_Madhya Pradesh/img/cov_1007.png]

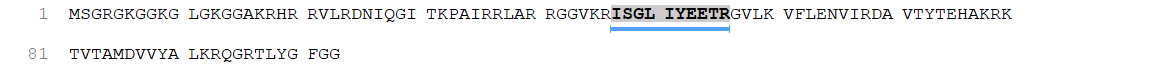

Supplement: S1 Data — (ZIP) [file pntd.0009247.s013.zip › D. russelii_Madhya Pradesh/img/cov_1008.png]

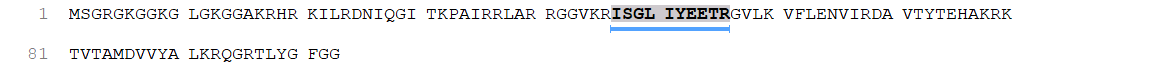

Supplement: S1 Data — (ZIP) [file pntd.0009247.s013.zip › D. russelii_Madhya Pradesh/img/cov_1009.png]
