# Supplementary figures and images for: Biogeographic venom variation in Russell’s viper (Daboia russelii) and the preclinical inefficacy of antivenom therapy in snakebite hotspots (part 1 of 2)
Source: PLoS Negl Trop Dis. 2021 Mar 25;15(3):e0009247. doi: 10.1371/journal.pntd.0009247 (PMC7993602; doi:10.1371/journal.pntd.0009247)

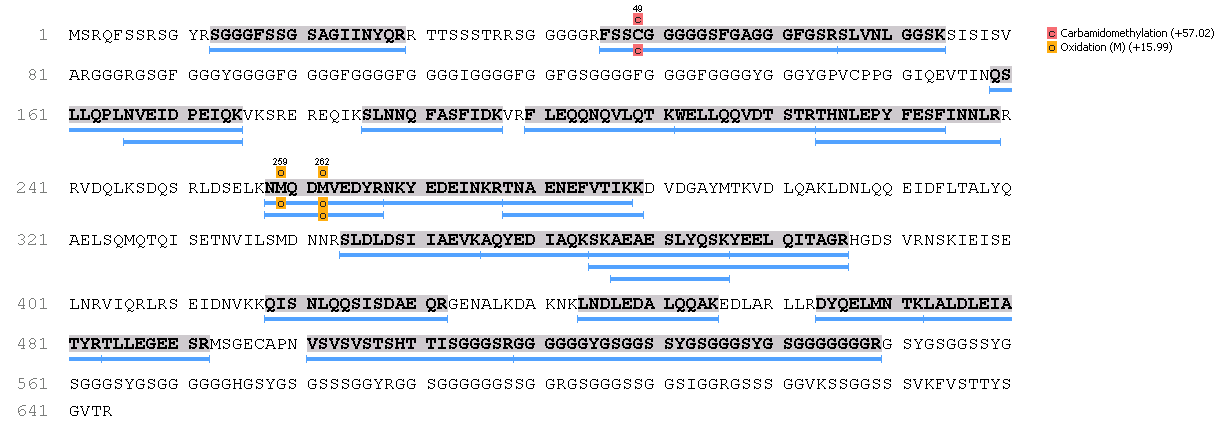

Supplement: S1 Data — (ZIP) [file pntd.0009247.s013.zip › D. russelii_Maharashtra/img/cov_1.png]

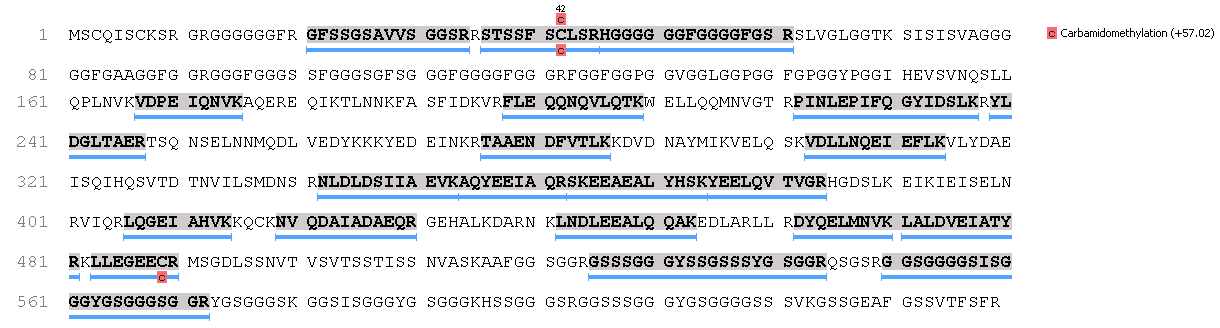

Supplement: S1 Data — (ZIP) [file pntd.0009247.s013.zip › D. russelii_Maharashtra/img/cov_10.png]

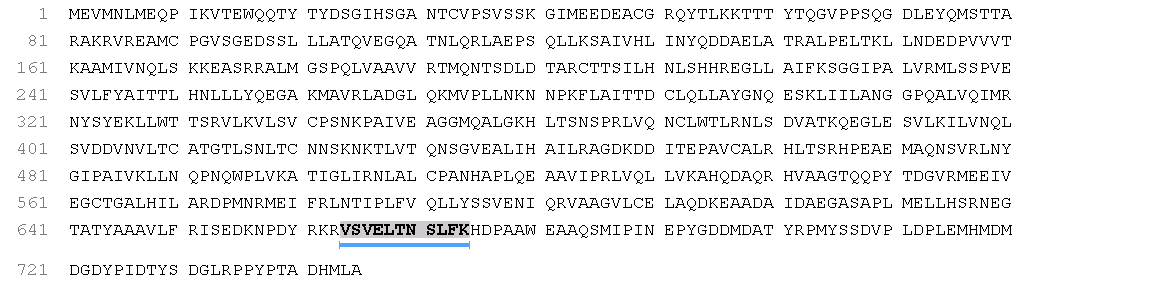

Supplement: S1 Data — (ZIP) [file pntd.0009247.s013.zip › D. russelii_Maharashtra/img/cov_100.png]

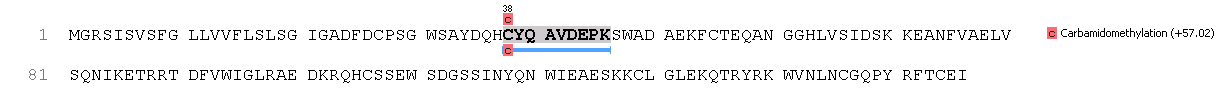

Supplement: S1 Data — (ZIP) [file pntd.0009247.s013.zip › D. russelii_Maharashtra/img/cov_1029.png]

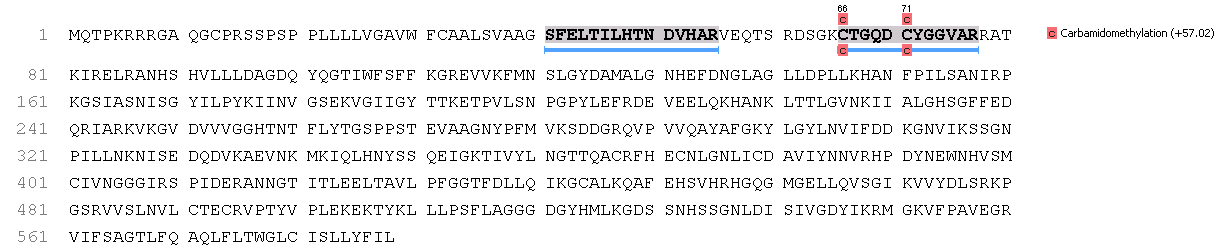

Supplement: S1 Data — (ZIP) [file pntd.0009247.s013.zip › D. russelii_Maharashtra/img/cov_103.png]

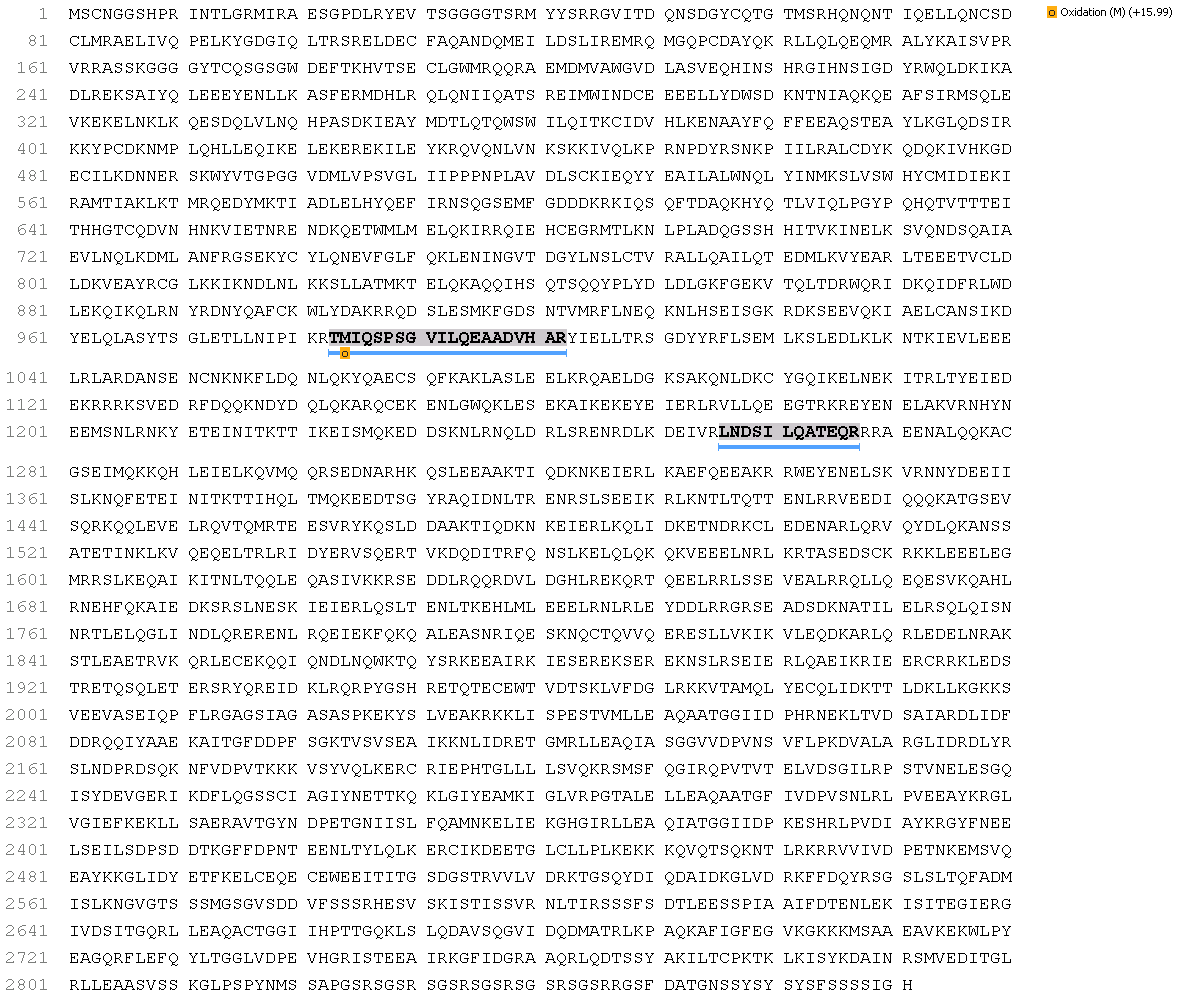

Supplement: S1 Data — (ZIP) [file pntd.0009247.s013.zip › D. russelii_Maharashtra/img/cov_105.png]

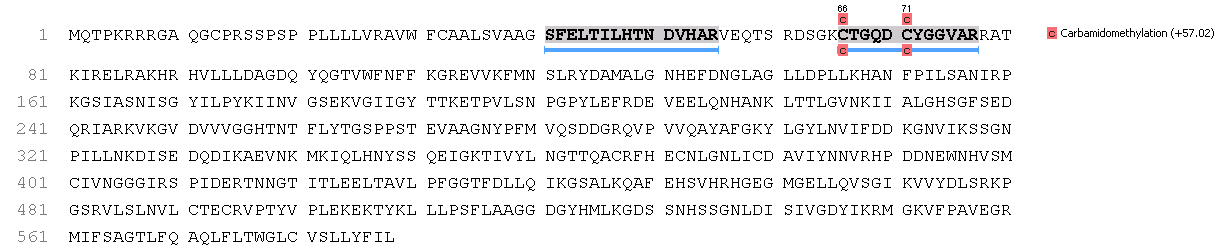

Supplement: S1 Data — (ZIP) [file pntd.0009247.s013.zip › D. russelii_Maharashtra/img/cov_106.png]

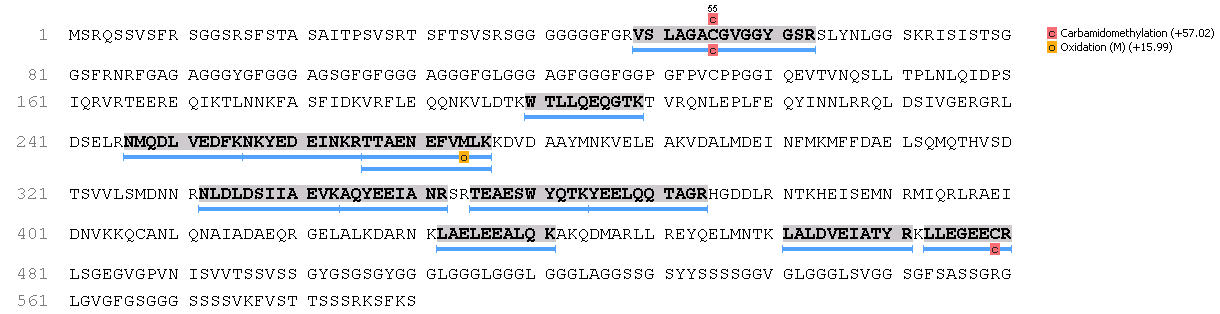

Supplement: S1 Data — (ZIP) [file pntd.0009247.s013.zip › D. russelii_Maharashtra/img/cov_11.png]

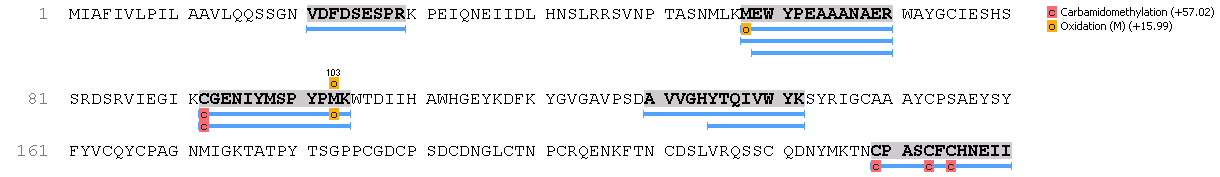

Supplement: S1 Data — (ZIP) [file pntd.0009247.s013.zip › D. russelii_Maharashtra/img/cov_114.png]

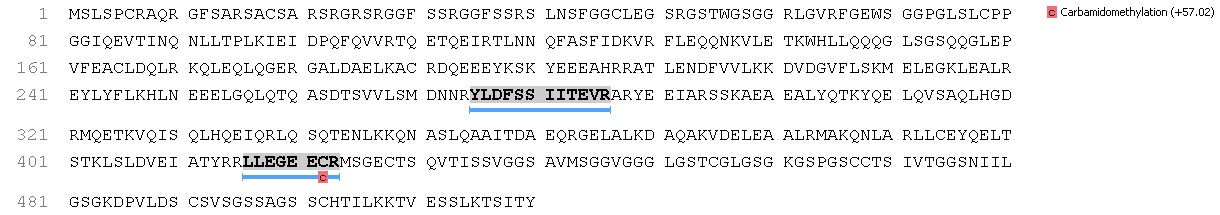

Supplement: S1 Data — (ZIP) [file pntd.0009247.s013.zip › D. russelii_Maharashtra/img/cov_116.png]

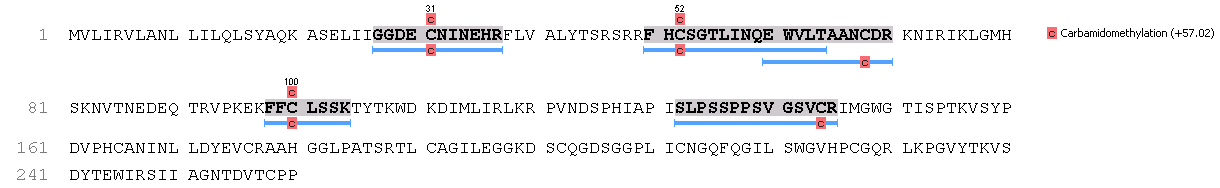

Supplement: S1 Data — (ZIP) [file pntd.0009247.s013.zip › D. russelii_Maharashtra/img/cov_123.png]

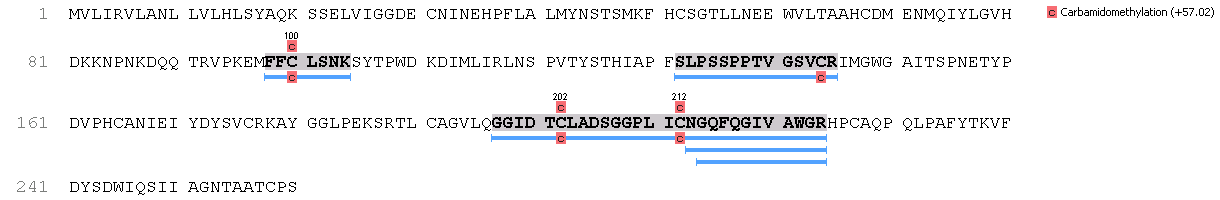

Supplement: S1 Data — (ZIP) [file pntd.0009247.s013.zip › D. russelii_Maharashtra/img/cov_127.png]

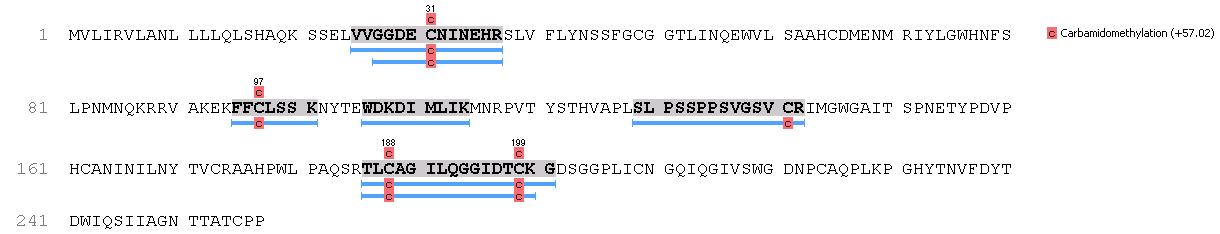

Supplement: S1 Data — (ZIP) [file pntd.0009247.s013.zip › D. russelii_Maharashtra/img/cov_129.png]

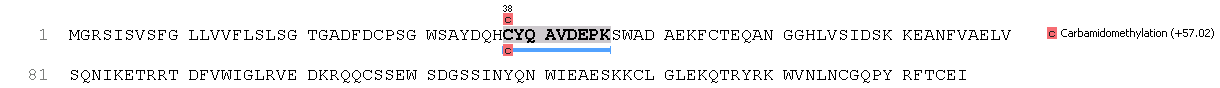

Supplement: S1 Data — (ZIP) [file pntd.0009247.s013.zip › D. russelii_Maharashtra/img/cov_1315.png]

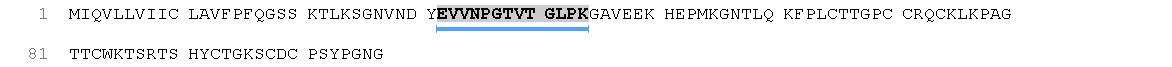

Supplement: S1 Data — (ZIP) [file pntd.0009247.s013.zip › D. russelii_Maharashtra/img/cov_1320.png]

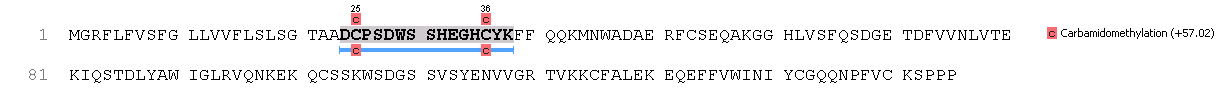

Supplement: S1 Data — (ZIP) [file pntd.0009247.s013.zip › D. russelii_Maharashtra/img/cov_1322.png]

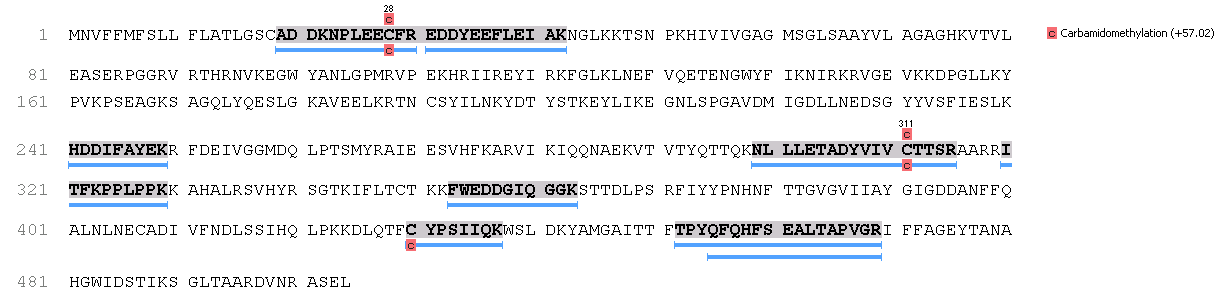

Supplement: S1 Data — (ZIP) [file pntd.0009247.s013.zip › D. russelii_Maharashtra/img/cov_14.png]

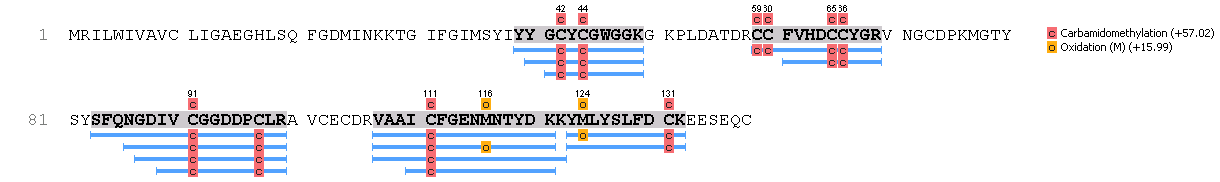

Supplement: S1 Data — (ZIP) [file pntd.0009247.s013.zip › D. russelii_Maharashtra/img/cov_145.png]

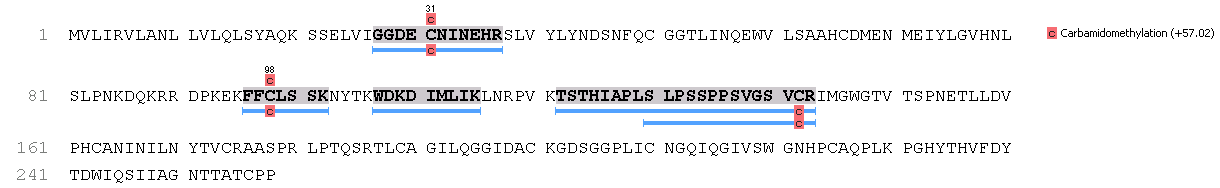

Supplement: S1 Data — (ZIP) [file pntd.0009247.s013.zip › D. russelii_Maharashtra/img/cov_148.png]

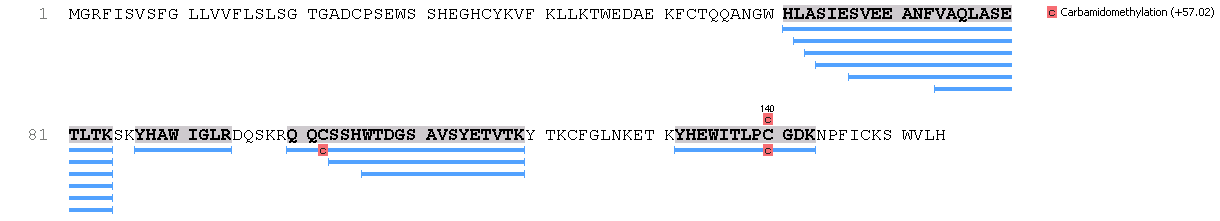

Supplement: S1 Data — (ZIP) [file pntd.0009247.s013.zip › D. russelii_Maharashtra/img/cov_149.png]

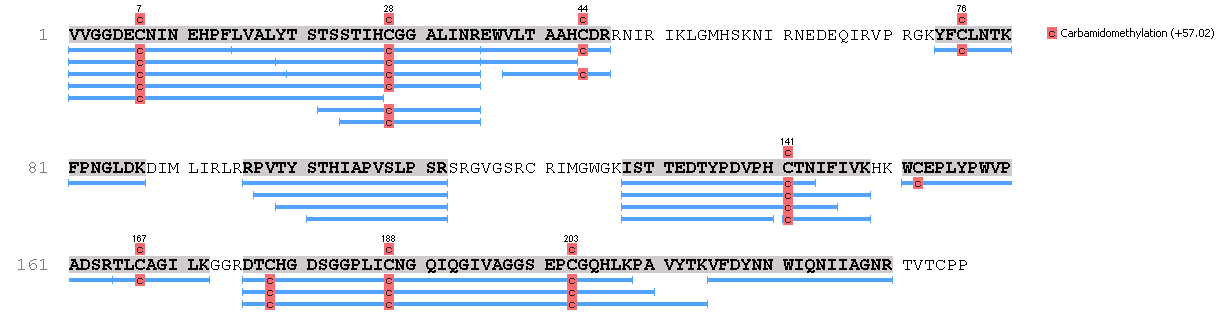

Supplement: S1 Data — (ZIP) [file pntd.0009247.s013.zip › D. russelii_Maharashtra/img/cov_15.png]

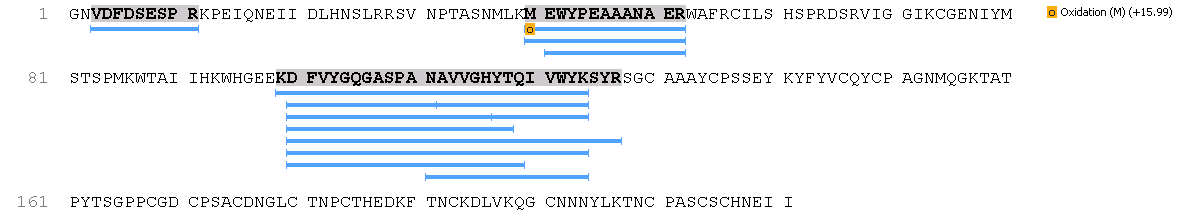

Supplement: S1 Data — (ZIP) [file pntd.0009247.s013.zip › D. russelii_Maharashtra/img/cov_154.png]

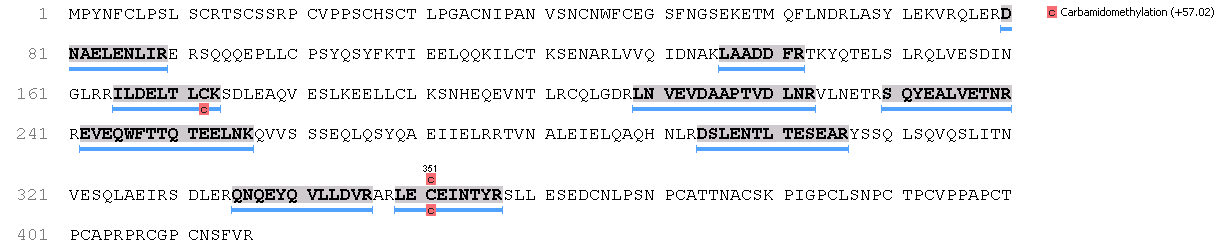

Supplement: S1 Data — (ZIP) [file pntd.0009247.s013.zip › D. russelii_Maharashtra/img/cov_16.png]

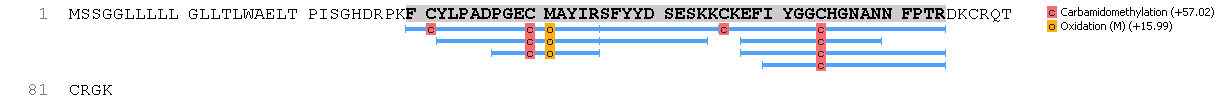

Supplement: S1 Data — (ZIP) [file pntd.0009247.s013.zip › D. russelii_Maharashtra/img/cov_167.png]

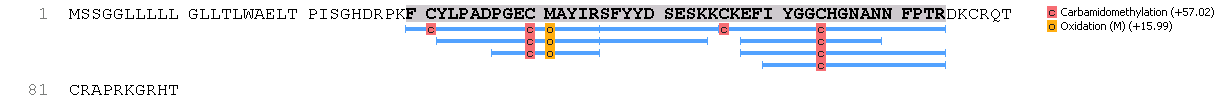

Supplement: S1 Data — (ZIP) [file pntd.0009247.s013.zip › D. russelii_Maharashtra/img/cov_168.png]

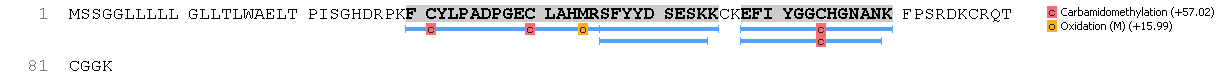

Supplement: S1 Data — (ZIP) [file pntd.0009247.s013.zip › D. russelii_Maharashtra/img/cov_169.png]

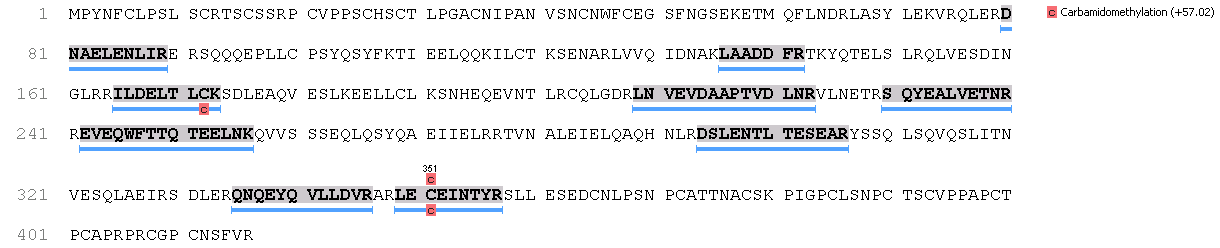

Supplement: S1 Data — (ZIP) [file pntd.0009247.s013.zip › D. russelii_Maharashtra/img/cov_17.png]

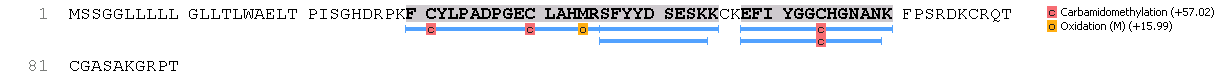

Supplement: S1 Data — (ZIP) [file pntd.0009247.s013.zip › D. russelii_Maharashtra/img/cov_170.png]

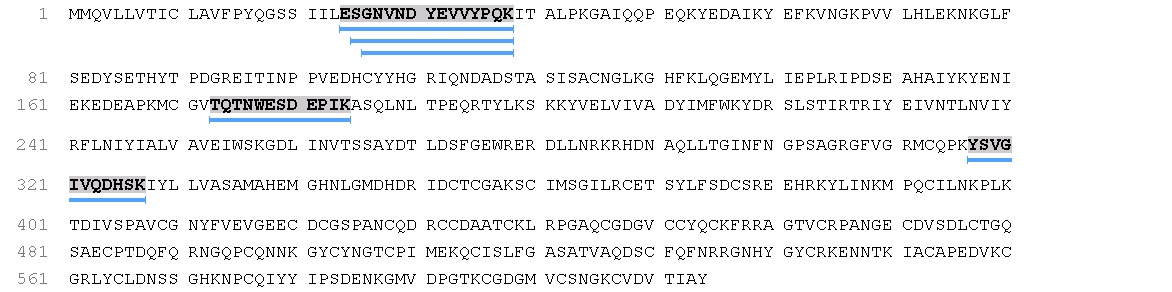

Supplement: S1 Data — (ZIP) [file pntd.0009247.s013.zip › D. russelii_Maharashtra/img/cov_191.png]

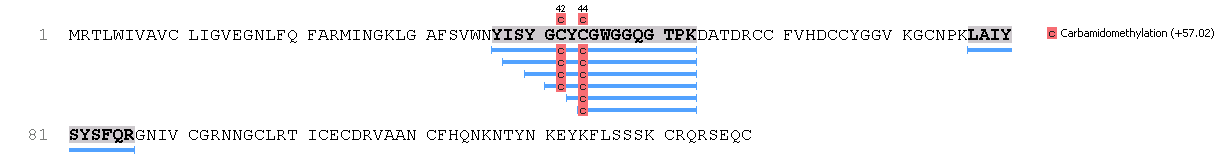

Supplement: S1 Data — (ZIP) [file pntd.0009247.s013.zip › D. russelii_Maharashtra/img/cov_208.png]

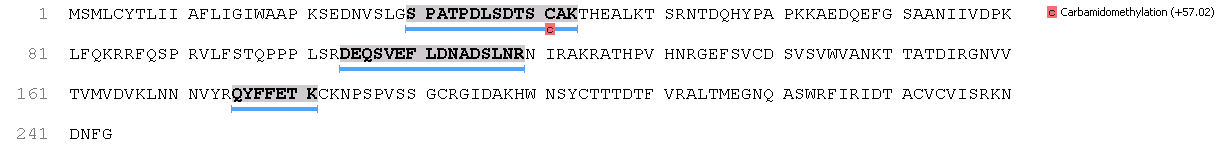

Supplement: S1 Data — (ZIP) [file pntd.0009247.s013.zip › D. russelii_Maharashtra/img/cov_210.png]

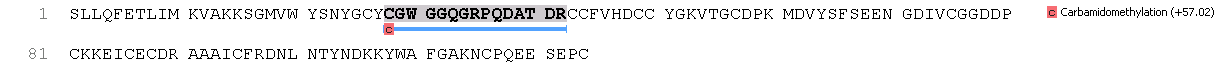

Supplement: S1 Data — (ZIP) [file pntd.0009247.s013.zip › D. russelii_Maharashtra/img/cov_212.png]

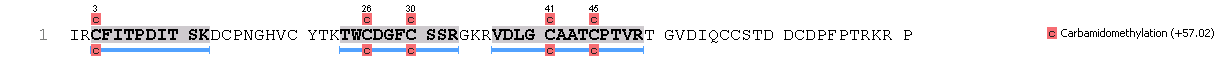

Supplement: S1 Data — (ZIP) [file pntd.0009247.s013.zip › D. russelii_Maharashtra/img/cov_217.png]

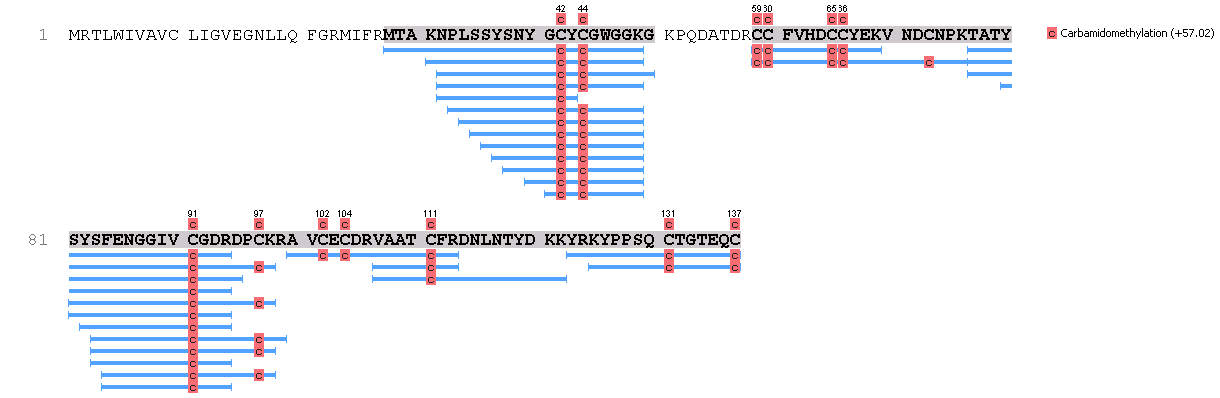

Supplement: S1 Data — (ZIP) [file pntd.0009247.s013.zip › D. russelii_Maharashtra/img/cov_22.png]

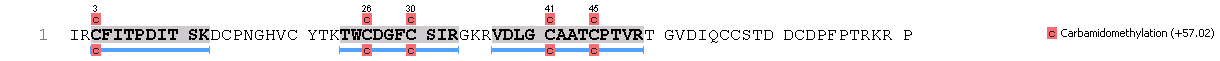

Supplement: S1 Data — (ZIP) [file pntd.0009247.s013.zip › D. russelii_Maharashtra/img/cov_220.png]

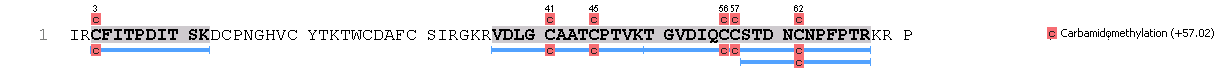

Supplement: S1 Data — (ZIP) [file pntd.0009247.s013.zip › D. russelii_Maharashtra/img/cov_240.png]

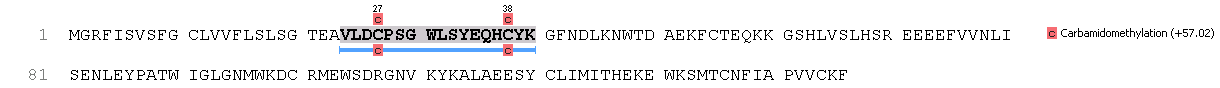

Supplement: S1 Data — (ZIP) [file pntd.0009247.s013.zip › D. russelii_Maharashtra/img/cov_248.png]

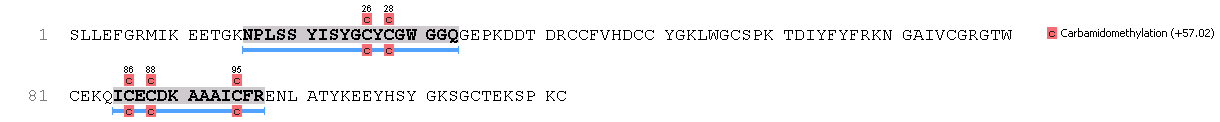

Supplement: S1 Data — (ZIP) [file pntd.0009247.s013.zip › D. russelii_Maharashtra/img/cov_272.png]

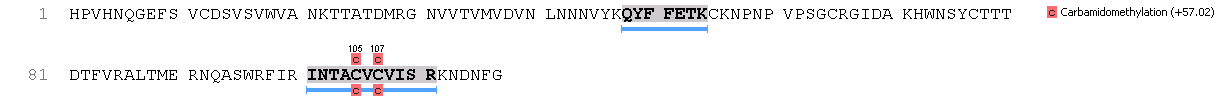

Supplement: S1 Data — (ZIP) [file pntd.0009247.s013.zip › D. russelii_Maharashtra/img/cov_275.png]

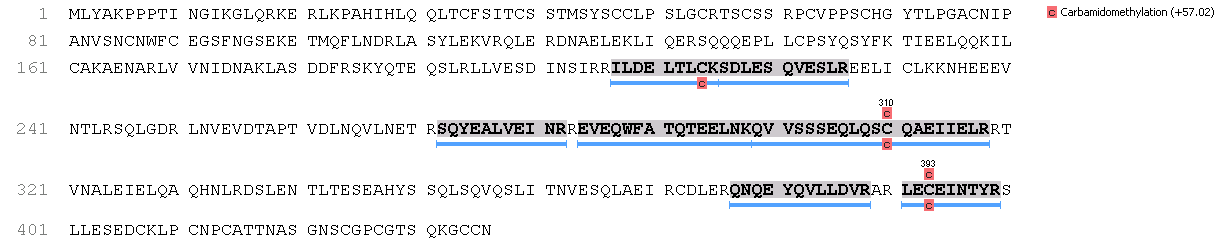

Supplement: S1 Data — (ZIP) [file pntd.0009247.s013.zip › D. russelii_Maharashtra/img/cov_28.png]

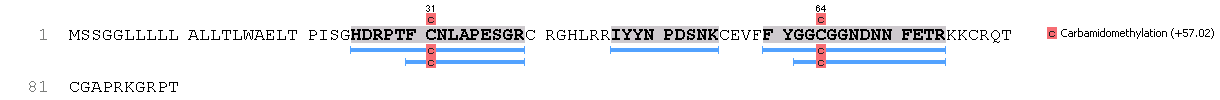

Supplement: S1 Data — (ZIP) [file pntd.0009247.s013.zip › D. russelii_Maharashtra/img/cov_304.png]

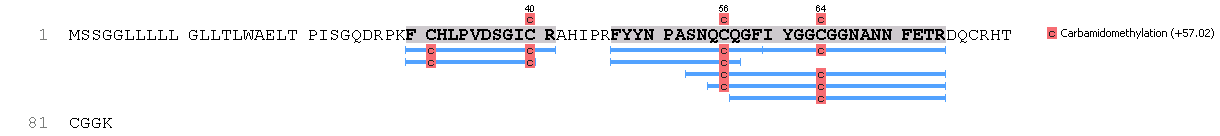

Supplement: S1 Data — (ZIP) [file pntd.0009247.s013.zip › D. russelii_Maharashtra/img/cov_307.png]

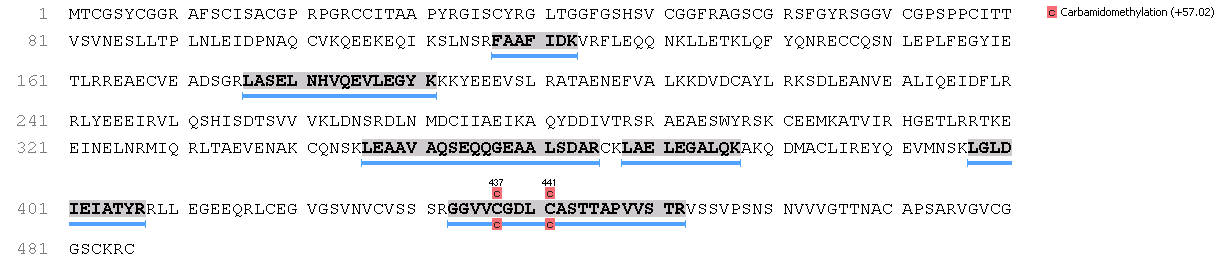

Supplement: S1 Data — (ZIP) [file pntd.0009247.s013.zip › D. russelii_Maharashtra/img/cov_31.png]

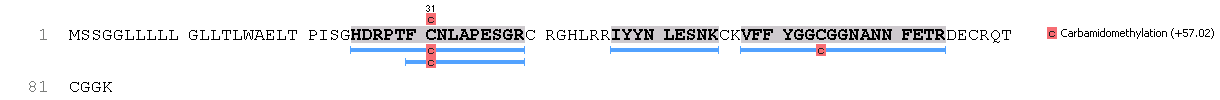

Supplement: S1 Data — (ZIP) [file pntd.0009247.s013.zip › D. russelii_Maharashtra/img/cov_310.png]

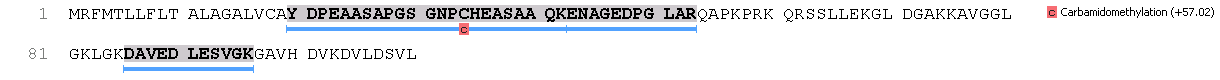

Supplement: S1 Data — (ZIP) [file pntd.0009247.s013.zip › D. russelii_Maharashtra/img/cov_316.png]

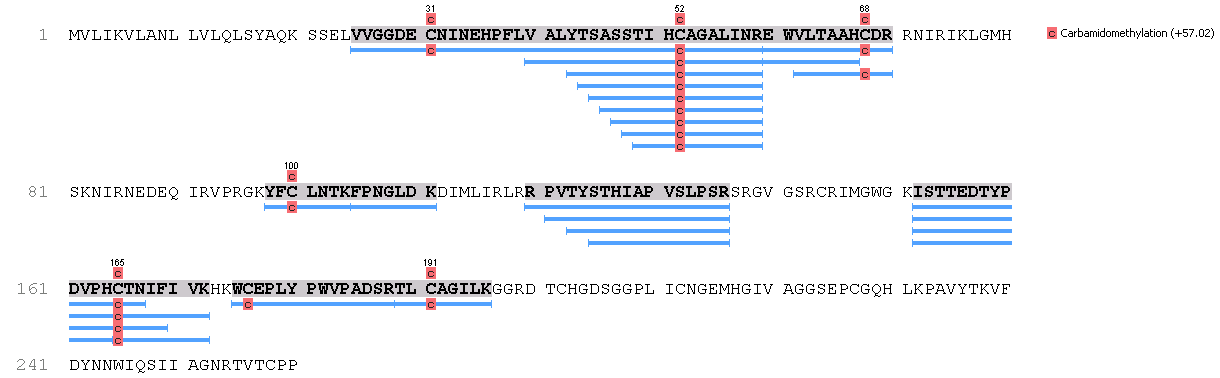

Supplement: S1 Data — (ZIP) [file pntd.0009247.s013.zip › D. russelii_Maharashtra/img/cov_33.png]

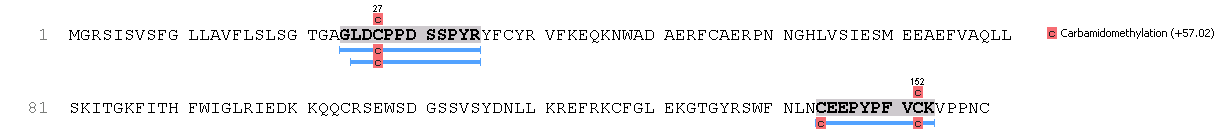

Supplement: S1 Data — (ZIP) [file pntd.0009247.s013.zip › D. russelii_Maharashtra/img/cov_331.png]

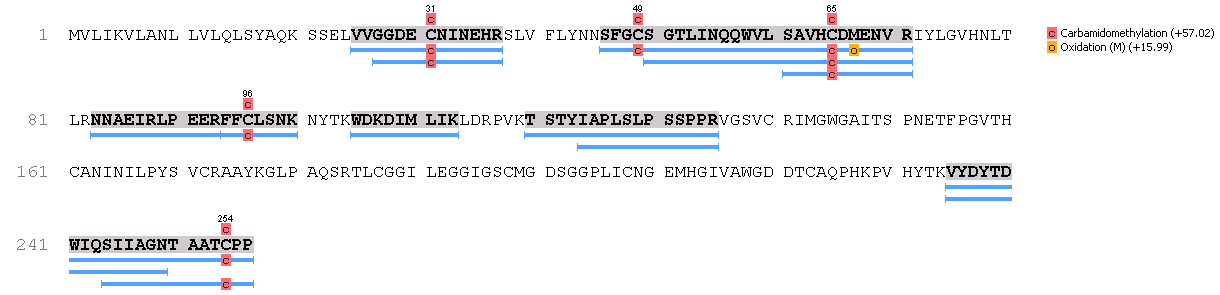

Supplement: S1 Data — (ZIP) [file pntd.0009247.s013.zip › D. russelii_Maharashtra/img/cov_34.png]

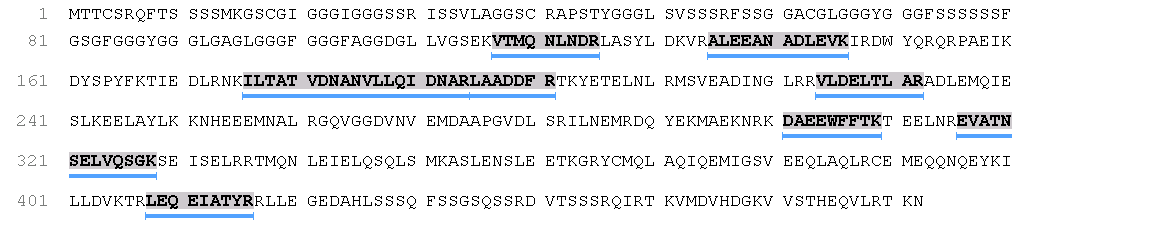

Supplement: S1 Data — (ZIP) [file pntd.0009247.s013.zip › D. russelii_Maharashtra/img/cov_35.png]

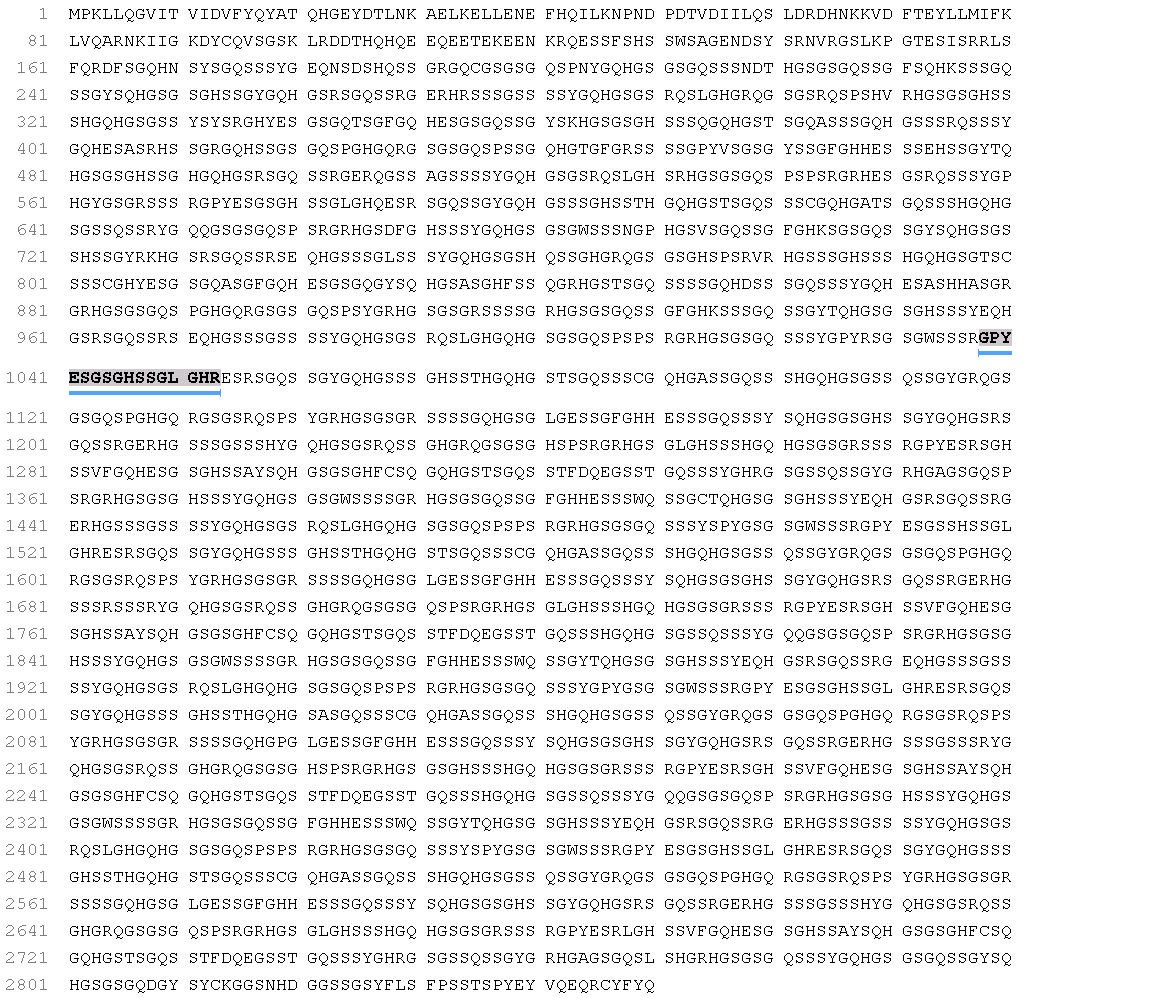

Supplement: S1 Data — (ZIP) [file pntd.0009247.s013.zip › D. russelii_Maharashtra/img/cov_398.png]

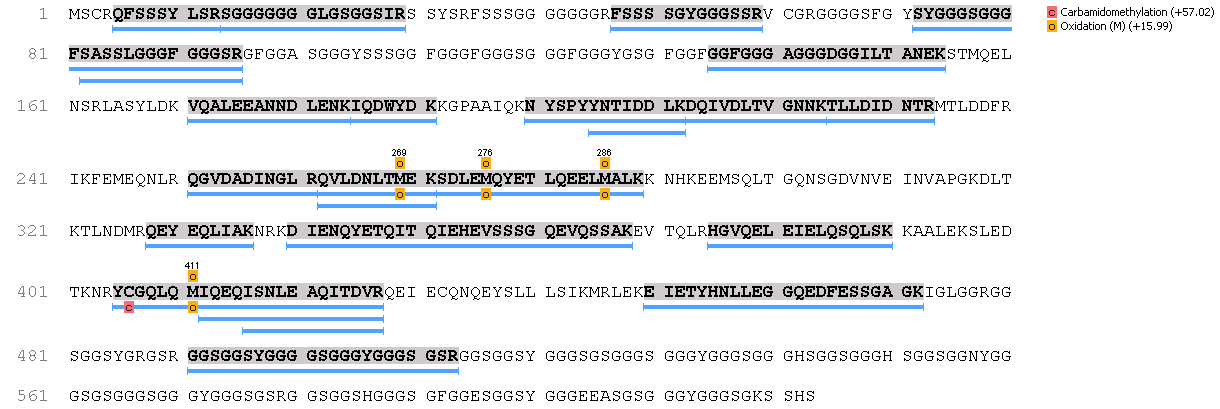

Supplement: S1 Data — (ZIP) [file pntd.0009247.s013.zip › D. russelii_Maharashtra/img/cov_4.png]

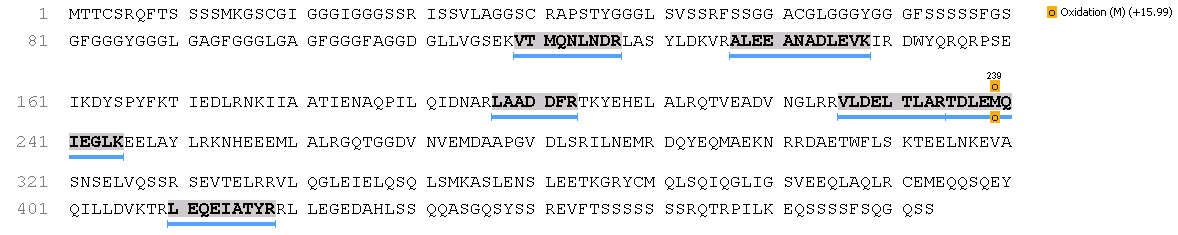

Supplement: S1 Data — (ZIP) [file pntd.0009247.s013.zip › D. russelii_Maharashtra/img/cov_41.png]

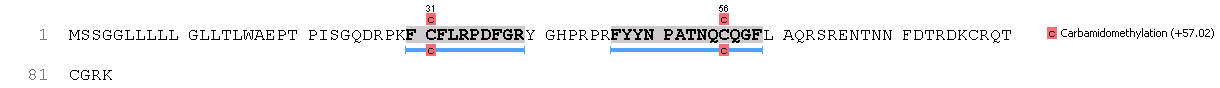

Supplement: S1 Data — (ZIP) [file pntd.0009247.s013.zip › D. russelii_Maharashtra/img/cov_419.png]

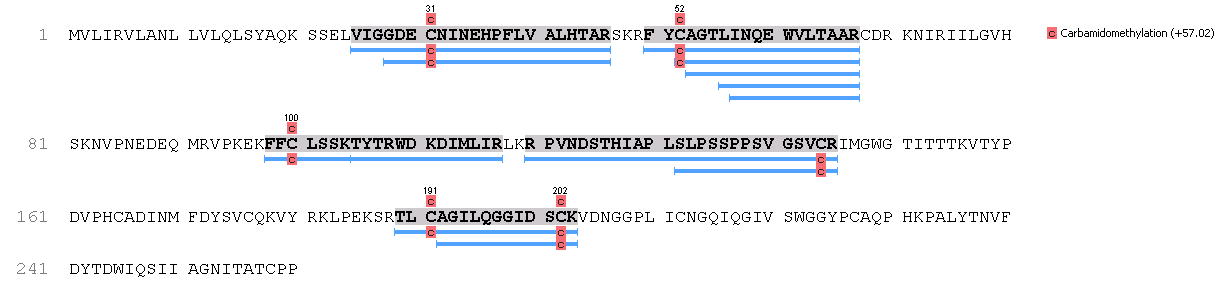

Supplement: S1 Data — (ZIP) [file pntd.0009247.s013.zip › D. russelii_Maharashtra/img/cov_43.png]

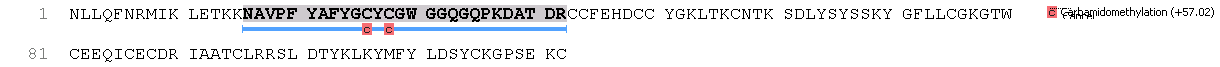

Supplement: S1 Data — (ZIP) [file pntd.0009247.s013.zip › D. russelii_Maharashtra/img/cov_458.png]

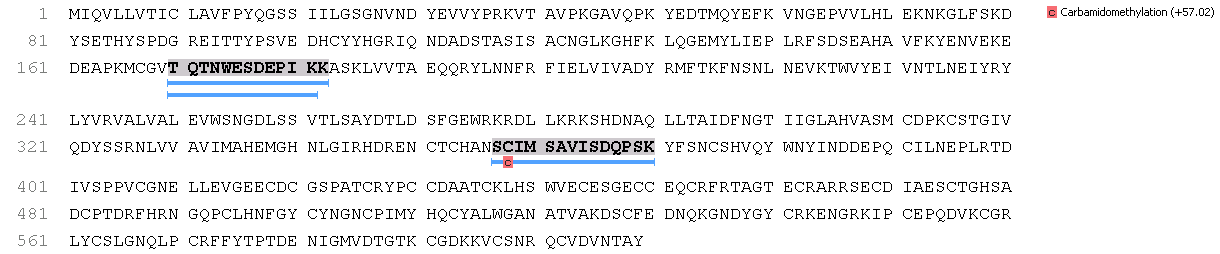

Supplement: S1 Data — (ZIP) [file pntd.0009247.s013.zip › D. russelii_Maharashtra/img/cov_463.png]

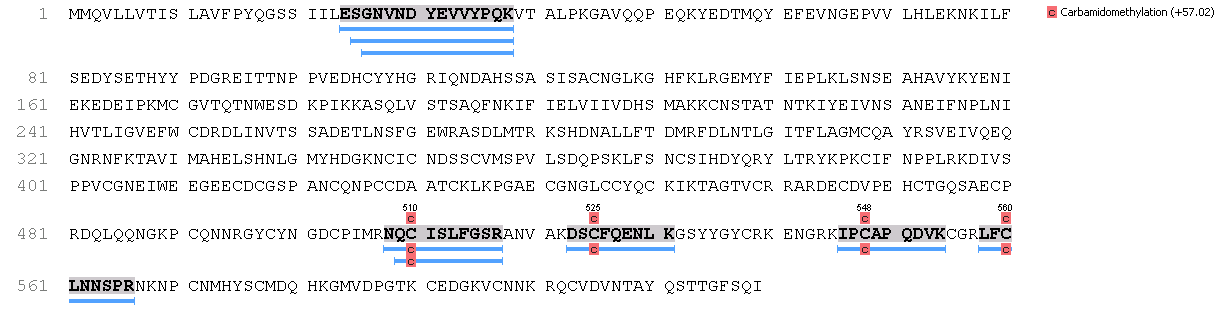

Supplement: S1 Data — (ZIP) [file pntd.0009247.s013.zip › D. russelii_Maharashtra/img/cov_47.png]

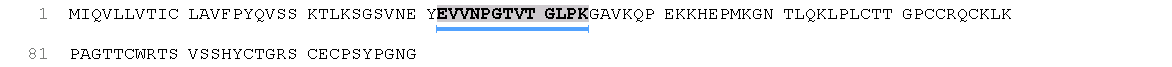

Supplement: S1 Data — (ZIP) [file pntd.0009247.s013.zip › D. russelii_Maharashtra/img/cov_479.png]

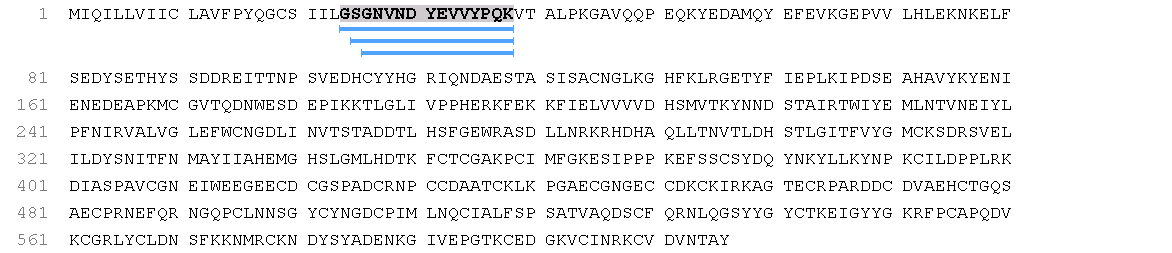

Supplement: S1 Data — (ZIP) [file pntd.0009247.s013.zip › D. russelii_Maharashtra/img/cov_507.png]

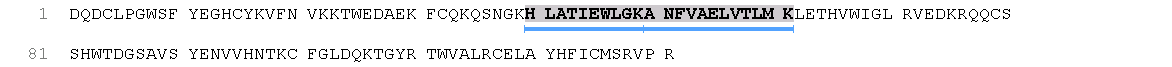

Supplement: S1 Data — (ZIP) [file pntd.0009247.s013.zip › D. russelii_Maharashtra/img/cov_549.png]

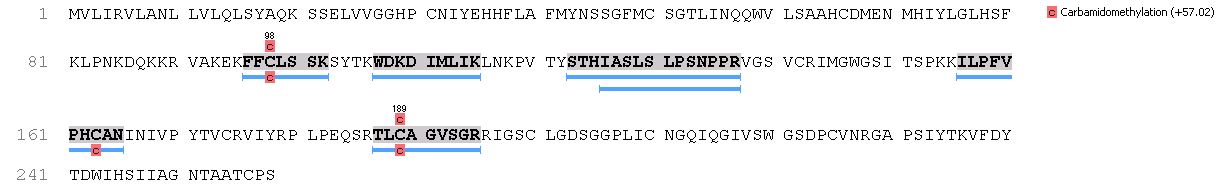

Supplement: S1 Data — (ZIP) [file pntd.0009247.s013.zip › D. russelii_Maharashtra/img/cov_55.png]

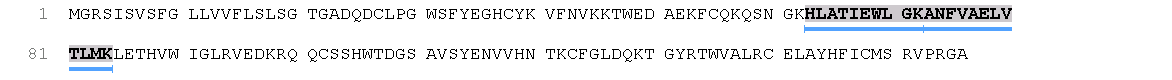

Supplement: S1 Data — (ZIP) [file pntd.0009247.s013.zip › D. russelii_Maharashtra/img/cov_550.png]

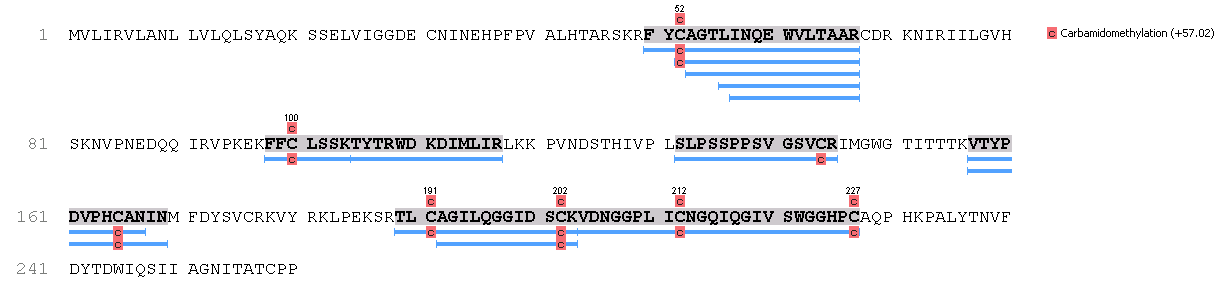

Supplement: S1 Data — (ZIP) [file pntd.0009247.s013.zip › D. russelii_Maharashtra/img/cov_57.png]

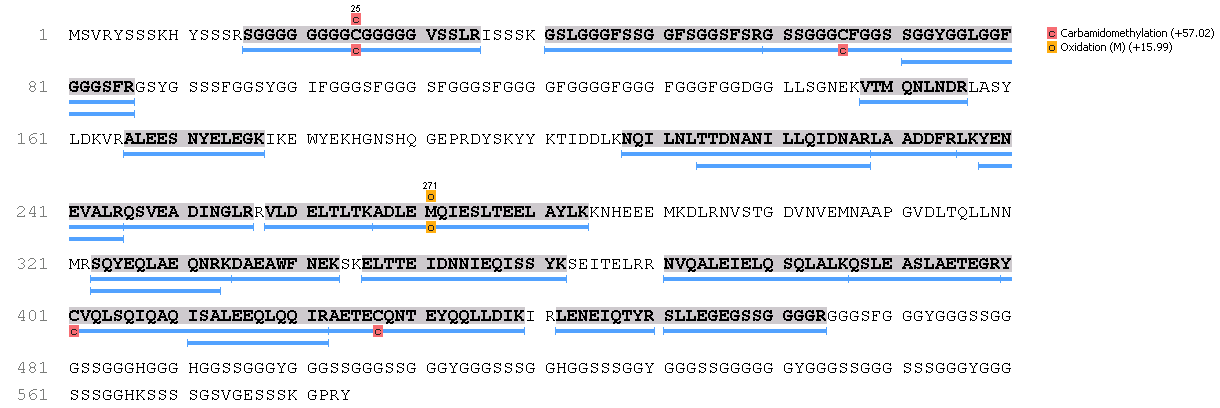

Supplement: S1 Data — (ZIP) [file pntd.0009247.s013.zip › D. russelii_Maharashtra/img/cov_6.png]

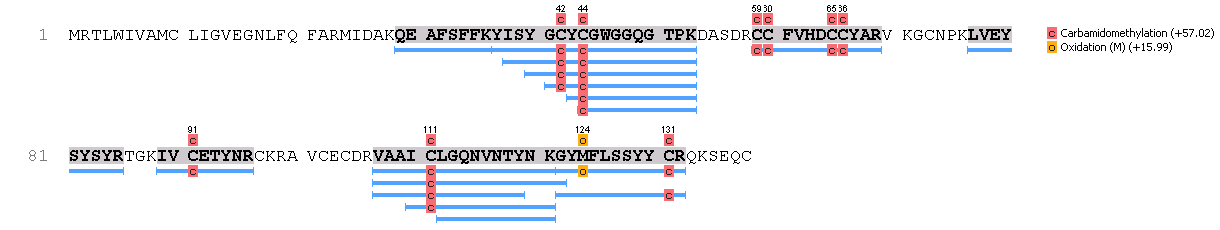

Supplement: S1 Data — (ZIP) [file pntd.0009247.s013.zip › D. russelii_Maharashtra/img/cov_60.png]

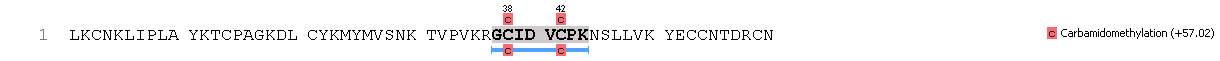

Supplement: S1 Data — (ZIP) [file pntd.0009247.s013.zip › D. russelii_Maharashtra/img/cov_605.png]

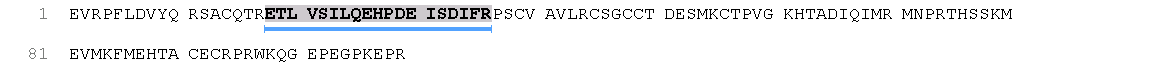

Supplement: S1 Data — (ZIP) [file pntd.0009247.s013.zip › D. russelii_Maharashtra/img/cov_634.png]

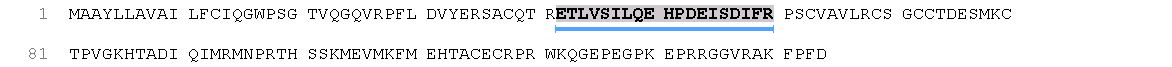

Supplement: S1 Data — (ZIP) [file pntd.0009247.s013.zip › D. russelii_Maharashtra/img/cov_635.png]

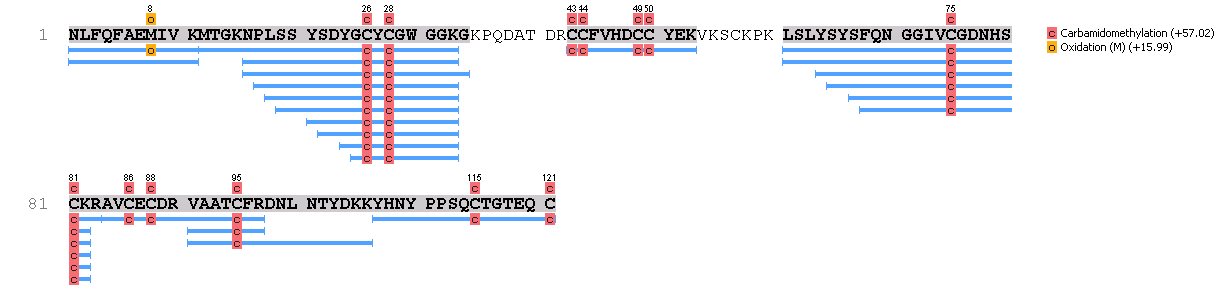

Supplement: S1 Data — (ZIP) [file pntd.0009247.s013.zip › D. russelii_Maharashtra/img/cov_69.png]

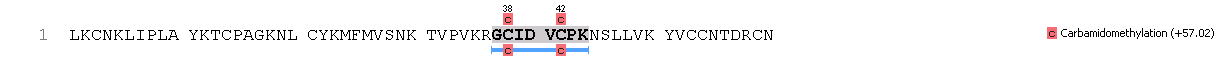

Supplement: S1 Data — (ZIP) [file pntd.0009247.s013.zip › D. russelii_Maharashtra/img/cov_697.png]

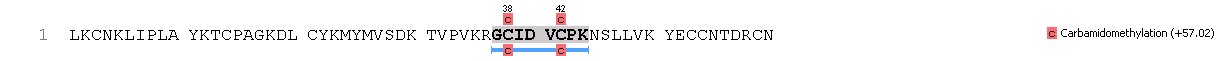

Supplement: S1 Data — (ZIP) [file pntd.0009247.s013.zip › D. russelii_Maharashtra/img/cov_698.png]

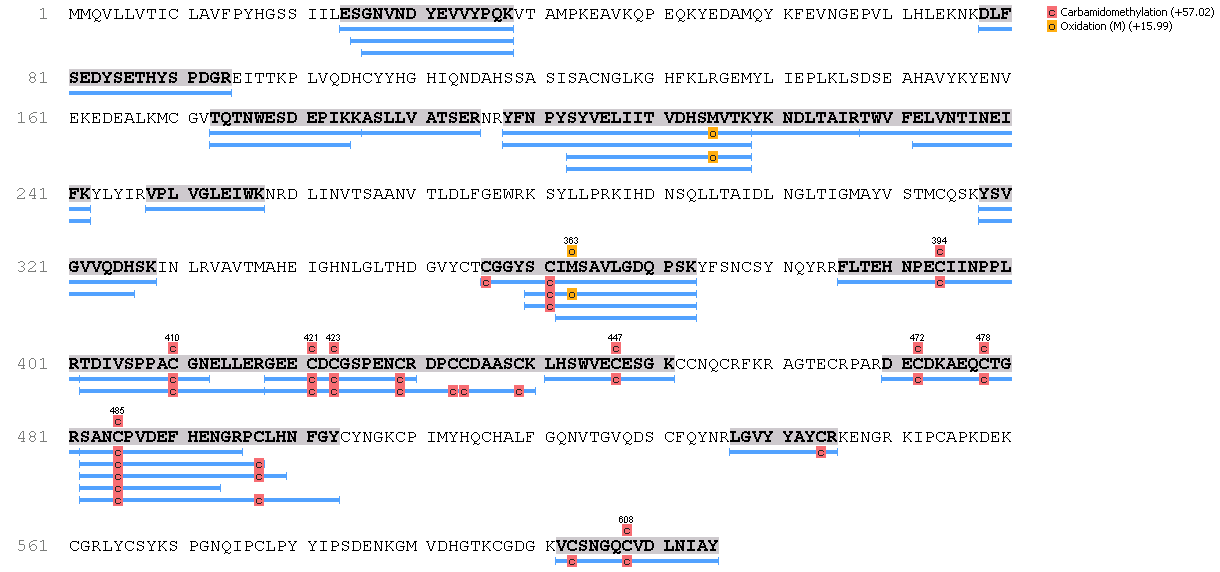

Supplement: S1 Data — (ZIP) [file pntd.0009247.s013.zip › D. russelii_Maharashtra/img/cov_7.png]

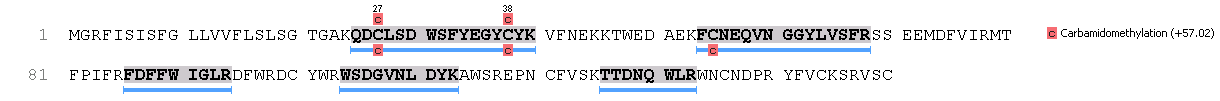

Supplement: S1 Data — (ZIP) [file pntd.0009247.s013.zip › D. russelii_Maharashtra/img/cov_71.png]

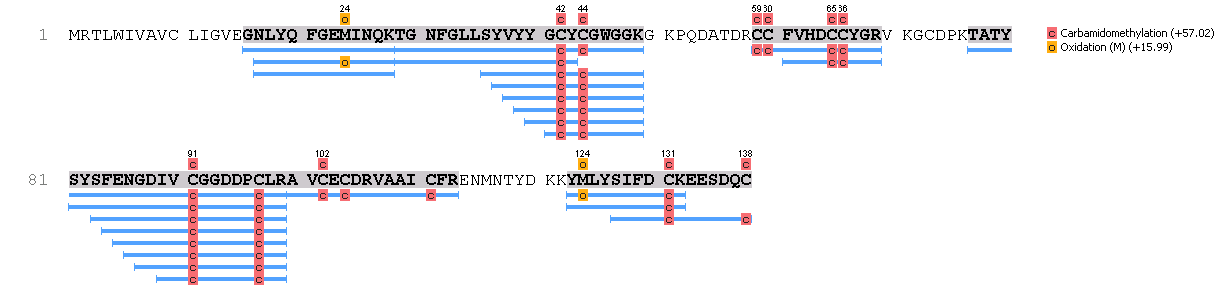

Supplement: S1 Data — (ZIP) [file pntd.0009247.s013.zip › D. russelii_Maharashtra/img/cov_81.png]

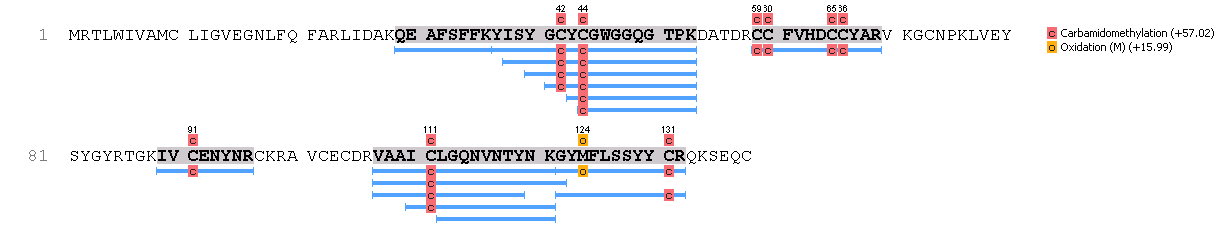

Supplement: S1 Data — (ZIP) [file pntd.0009247.s013.zip › D. russelii_Maharashtra/img/cov_84.png]

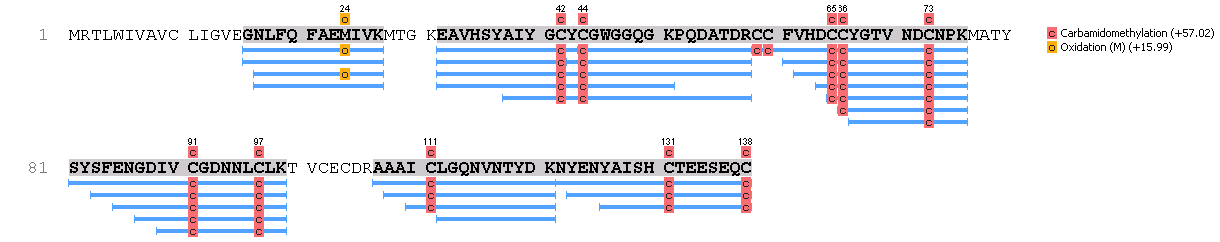

Supplement: S1 Data — (ZIP) [file pntd.0009247.s013.zip › D. russelii_Maharashtra/img/cov_88.png]

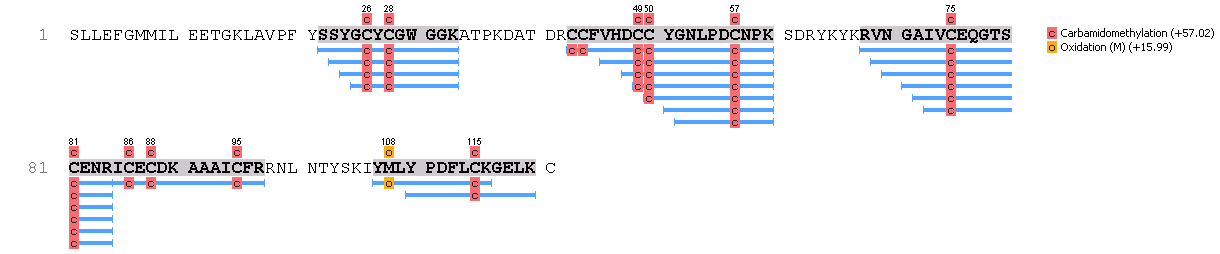

Supplement: S1 Data — (ZIP) [file pntd.0009247.s013.zip › D. russelii_Maharashtra/img/cov_90.png]

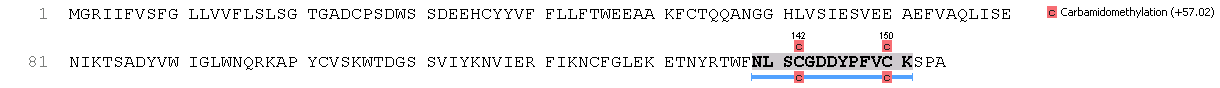

Supplement: S1 Data — (ZIP) [file pntd.0009247.s013.zip › D. russelii_Maharashtra/img/cov_919.png]

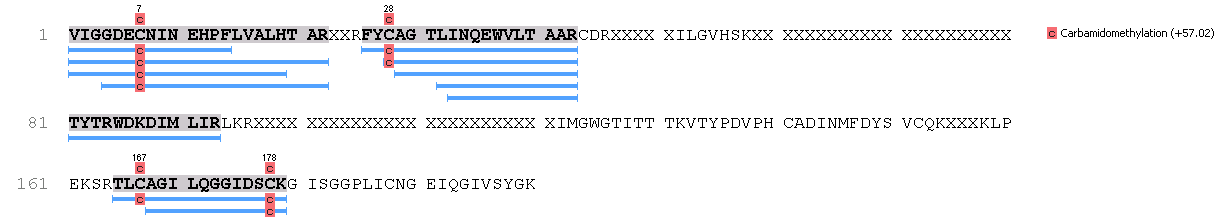

Supplement: S1 Data — (ZIP) [file pntd.0009247.s013.zip › D. russelii_Maharashtra/img/cov_92.png]

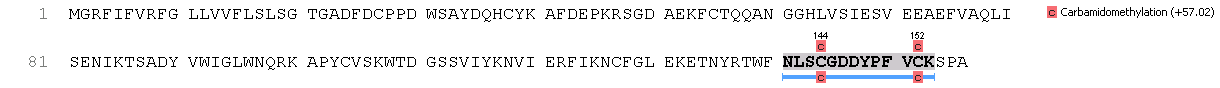

Supplement: S1 Data — (ZIP) [file pntd.0009247.s013.zip › D. russelii_Maharashtra/img/cov_920.png]

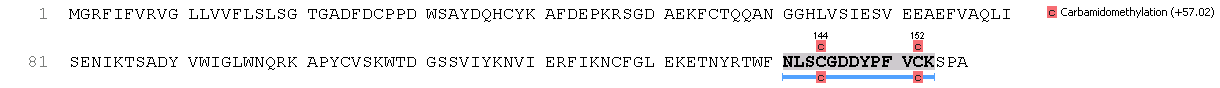

Supplement: S1 Data — (ZIP) [file pntd.0009247.s013.zip › D. russelii_Maharashtra/img/cov_921.png]

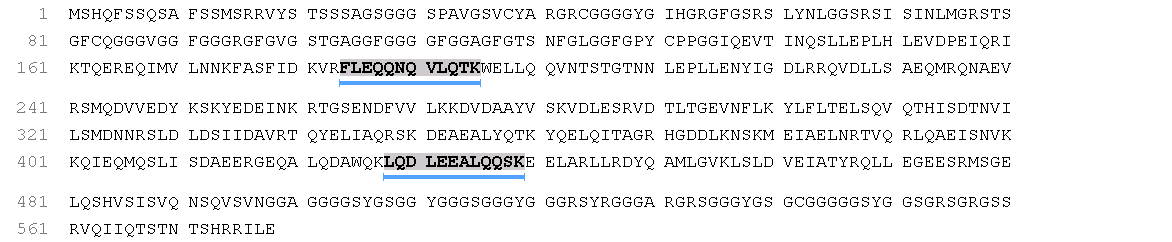

Supplement: S1 Data — (ZIP) [file pntd.0009247.s013.zip › D. russelii_Maharashtra/img/cov_96.png]

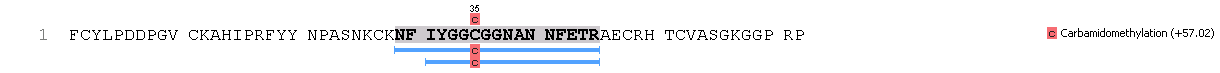

Supplement: S1 Data — (ZIP) [file pntd.0009247.s013.zip › D. russelii_Maharashtra/img/cov_979.png]

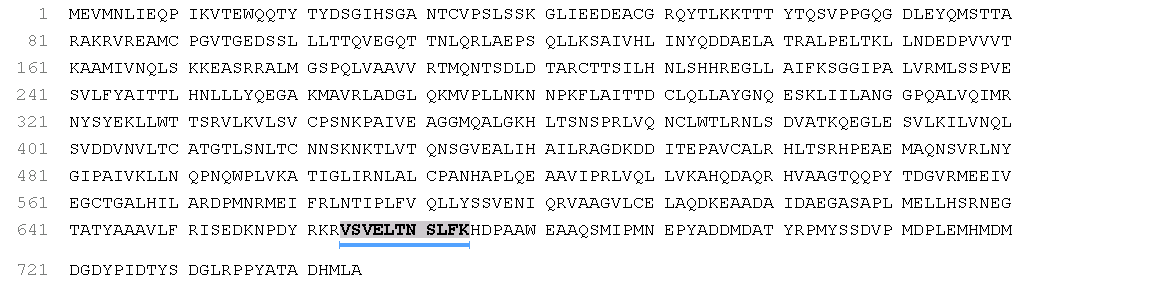

Supplement: S1 Data — (ZIP) [file pntd.0009247.s013.zip › D. russelii_Maharashtra/img/cov_98.png]

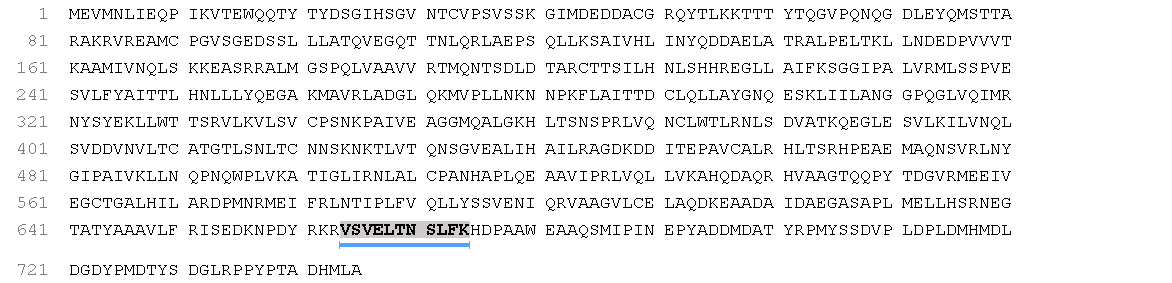

Supplement: S1 Data — (ZIP) [file pntd.0009247.s013.zip › D. russelii_Maharashtra/img/cov_99.png]

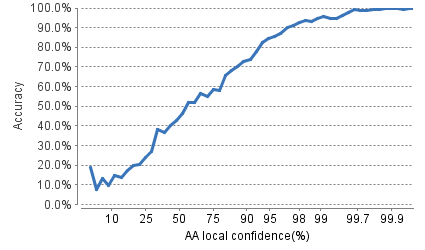

Supplement: S1 Data — (ZIP) [file pntd.0009247.s013.zip › D. russelii_Maharashtra/img/DenovoFDRCurveFigure4256561071334303456.png]

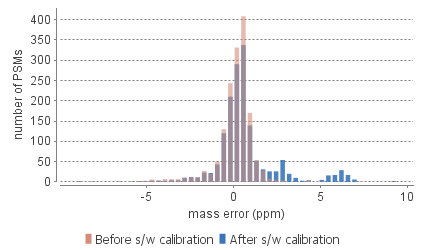

Supplement: S1 Data — (ZIP) [file pntd.0009247.s013.zip › D. russelii_Maharashtra/img/ErrorCalibratedHistogram4953504326050086661.png]

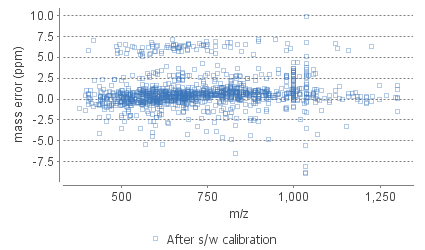

Supplement: S1 Data — (ZIP) [file pntd.0009247.s013.zip › D. russelii_Maharashtra/img/ErrorPlotFigure5639279665293543154.png]

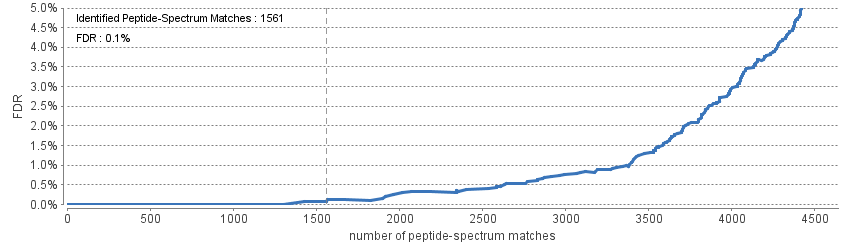

Supplement: S1 Data — (ZIP) [file pntd.0009247.s013.zip › D. russelii_Maharashtra/img/FDRFigure8575872097641631434.png]

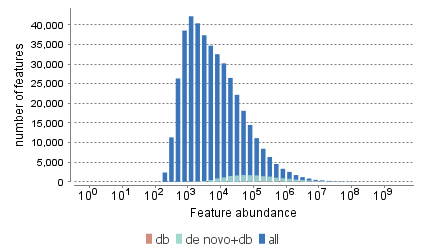

Supplement: S1 Data — (ZIP) [file pntd.0009247.s013.zip › D. russelii_Maharashtra/img/FeatureIntensityDistributionHistogram5073109149366834568.png]

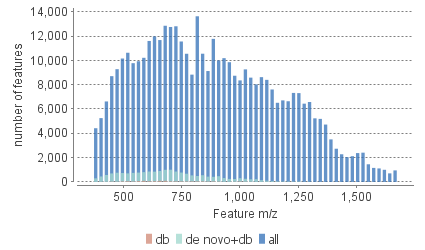

Supplement: S1 Data — (ZIP) [file pntd.0009247.s013.zip › D. russelii_Maharashtra/img/FeatureMzHistogram311694466730548972.png]

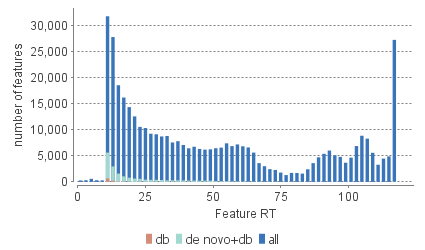

Supplement: S1 Data — (ZIP) [file pntd.0009247.s013.zip › D. russelii_Maharashtra/img/FeatureRtHistogram1710025804596367051.png]

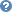

Supplement: S1 Data — (ZIP) [file pntd.0009247.s013.zip › D. russelii_Maharashtra/img/q.png]

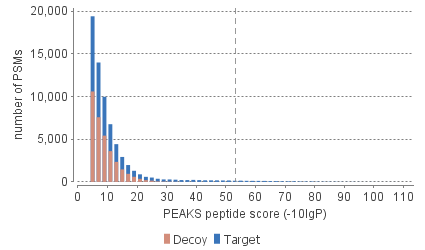

Supplement: S1 Data — (ZIP) [file pntd.0009247.s013.zip › D. russelii_Maharashtra/img/ScoreHistogram3392122293967942834.png]

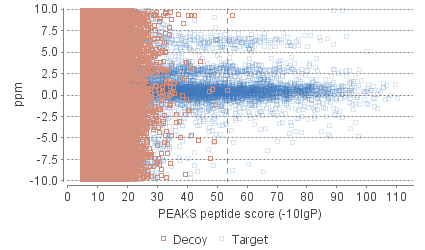

Supplement: S1 Data — (ZIP) [file pntd.0009247.s013.zip › D. russelii_Maharashtra/img/ScorePlotFigure5835143719402878653.png]

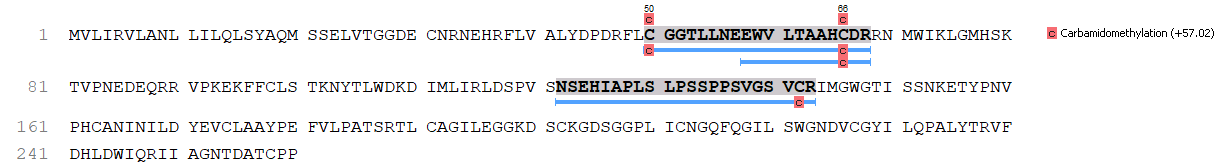

Supplement: S1 Data — (ZIP) [file pntd.0009247.s013.zip › D. russelii_West Bengal/img/cov_108.png]

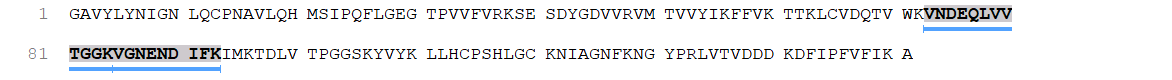

Supplement: S1 Data — (ZIP) [file pntd.0009247.s013.zip › D. russelii_West Bengal/img/cov_109.png]

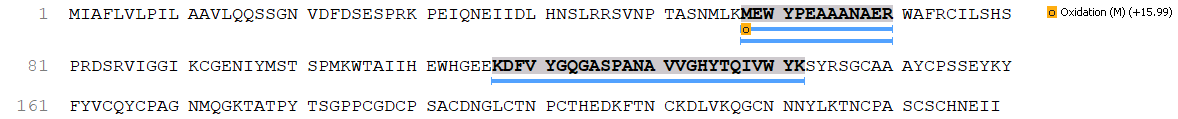

Supplement: S1 Data — (ZIP) [file pntd.0009247.s013.zip › D. russelii_West Bengal/img/cov_113.png]

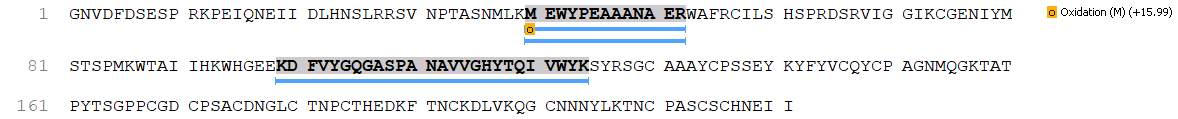

Supplement: S1 Data — (ZIP) [file pntd.0009247.s013.zip › D. russelii_West Bengal/img/cov_114.png]

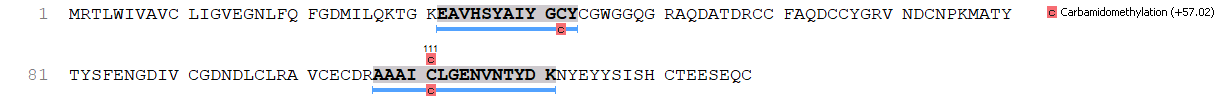

Supplement: S1 Data — (ZIP) [file pntd.0009247.s013.zip › D. russelii_West Bengal/img/cov_118.png]
